# Discovery of TYR inhibitors from de novo molecular generation to dual-track lead optimization: “Competition” between AI and chemists

**DOI:** 10.1126/sciadv.aeg0376

**Published:** 2026-06-19

**Authors:** Yinyan Sun, Jiahui Wang, Wenchao Chen, Xiaoying Jiang, Shan Wang, Jia Zhi, Feifan Li, Meiling Feng, Xiaotian Niu, Bin Ju, Jianan Guo, Renren Bai

**Affiliations:** ^1^ School of Pharmacy, Hangzhou Normal University, Hangzhou 311121, PR China.; ^2^ Key Laboratory of Elemene Class Anti-Cancer Chinese Medicines; Engineering Laboratory of Development and Application of Traditional Chinese Medicines; Collaborative Innovation Center of Traditional Chinese Medicines of Zhejiang Province, Hangzhou Normal University, Hangzhou 311121, PR China.; ^3^ Zhejiang Angel Medical AI Technology Co., Ltd., Hangzhou 311103, PR China.; ^4^ Department of Pharmacy, Jinhua Municipal Central Hospital, Jinhua 321000, PR China.

## Abstract

This study introduces a unified framework combining artificial intelligence (AI)–directed de novo molecular generation with dual-track lead optimization—comprising expert-guided strategies and AI-driven pathways—to discover tyrosinase (TYR) inhibitors for hyperpigmentation disorders. Using a reinforcement learning (RL)–based generative model, the lead compound **AI10** was identified. Subsequent optimization followed two parallel routes. The expert-guided approach yielded **AI10-m15** as the most potent TYR inhibitor, with notable antipigmentation activity and excellent cellular safety profiles. In contrast, the AI-driven pathway explored broader chemical spaces, generating unconventional chemotypes, exemplified by the potent TYR inhibitor **AI10-a2**, highlighting AI’s capacity to uncover nonintuitive activity cliffs despite greater output variability. Systematic comparison revealed that the AI model offers exploratory diversity, whereas expert-guided optimization provides predictable improvements in activity and developability. In summary, starting from an AI-generated lead and subsequently integrating both expert-guided and AI-driven structural optimization strategies, these findings further underscore that combining AI technologies with experts’ medicinal chemistry insights can substantially accelerate the discovery of viable candidate compounds.

## Teaser

TYR inhibitors were identified by artificial intelligence molecular generation and lead optimization.

## INTRODUCTION

Drug development remains a complex and resource-intensive process, typically requiring over a decade of effort and an estimated $2.5 billion to bring a new drug to market (*1*). Historically, this process has relied heavily on the expertise of drug discovery experts and an iterative trial-and-error methodology. However, in recent years, artificial intelligence (AI) has emerged as a transformative force in drug discovery, offering unprecedented opportunities to expedite the overall pipeline and optimize its constituent stages of drug development (*2*–*4*). AI-driven approaches, including target identification (*5*, *6*), virtual screening (*7*), de novo molecular generation (*8*), and ADMET (absorption, distribution, metabolism, excretion, and toxicity) predictions (*9*), have notably reduced both the time and cost associated with traditional drug discovery pipelines. Despite these advancements, traditional AI molecular generation models still face notable limitations, including a reliance on predefined chemical libraries, a lack of synthetic feasibility for some generated molecules, and insufficient exploration of previously unknown chemical spaces (*4*, *10*, *11*). These constraints inevitably impede the structural innovation and the discovery of bioactive compounds. To overcome these challenges, there is a pressing need for more sophisticated AI algorithms that can effectively integrate chemical knowledge, ensure synthetic feasibility, and enable target-specific optimization.

Deep reinforcement learning (RL) models, a rapidly advancing branch of AI, have demonstrated substantial potential in drug discovery and development due to their unique capacity to iteratively optimize molecular structures through dynamic interactions with the environment (*3*, *12*, *13*). Unlike supervised and unsupervised learning, RL’s core advantage lies in its ability to make continuous, data-driven decisions, optimizing complex processes based on feedback signals, rather than relying on static, labeled datasets. In the context of drug development, RL emulates the “trial-error-feedback-optimization” cycle, enabling autonomous exploration of vast chemical spaces. This is particularly valuable in multiobjective optimization tasks, where the goal is to simultaneously refine multiple parameters, including activity, selectivity, and ADMET properties. Despite the promise, traditional RL approaches, particularly those based on atom/bond editing, often face limitations due to insufficient constraints for chemical validity (*14*), leading to the generation of chemically invalid structures or compounds with poor synthetic feasibility (*15*). To overcome these challenges, recent advancements in RL for drug discovery have pivoted toward fragment-based growth strategies (*16*). These methods incorporate established drug-like chemical priors (e.g., functional group compatibility and scaffold stability) into the design of action spaces, thereby enhancing both the drug-likeness and synthetic accessibility of the generated molecules. In practice, this approach involves decomposing molecules into validated bioactive fragment libraries, serving as modular building blocks for further optimization. During the decision-making process, the RL agent links and modifies these fragments, ensuring structural integrity while simultaneously optimizing target properties through reward functions. These functions may include predictions of binding free energy or synthetic accessibility scores. This paradigm shift underscores the distinct advantages of RL in key phases of drug discovery, including virtual screening, scaffold hopping, and lead compound optimization, where the ability to navigate complex chemical spaces and balance multiple optimization objectives is critical (*17*–*19*).

In summary, the RL framework offers multiple advantages over other traditional AI generative models, such as FRAME (*20*) and ResGen (*21*). First, by embedding real reaction templates and building blocks, the model inherently ensures synthetic feasibility, thereby avoiding the pitfall of prioritizing activity over synthesizability. Second, the reward function can simultaneously integrate multiple objectives, including docking scores, drug-likeness, and synthetic feasibility, to achieve synergistic multiobjective optimization. Third, RL models are particularly adept at addressing complex dynamic decision-making problems, which provides a robust foundation for developing optimal synthetic routes.

Skin hyperpigmentation disorders, including melasma, age spots, and postinflammatory hyperpigmentation, are prevalent conditions affecting millions of individuals worldwide. These disorders arise from the overproduction of melanin, leading to aesthetic concerns and psychological distress for patients. Current clinical treatments primarily center on tyrosinase (TYR) inhibitors, including kojic acid, arbutin, and hydroquinone (*22*–*24*). However, these agents often exhibit limited efficacy, poor stability, and adverse side effects, underscoring the urgent need for more potent and safer alternatives. Consequently, the development of next-generation TYR inhibitors constitutes a pressing need and a major research focus in dermatology (*25*, *26*). TYR [enzyme commission (EC) 1.14.18.1] is a type 3 copper-containing enzyme that plays a central role in melanin biosynthesis. It catalyzes the oxidation of l-tyrosine to l-dopa and subsequently to dopaquinone, the key precursor of both eumelanin and pheomelanin (*27*, *28*). The active site of TYR features a binuclear copper center coordinated by six histidine residues, making it a challenging target for inhibitor design. Effective TYR inhibitors must not only exhibit high binding affinity but also navigate the complex stereoelectronic environment of the copper center. Despite considerable research efforts, few inhibitors have successfully achieved both high potency and clinical applicability, underscoring the necessity for innovative approaches in TYR inhibitor development (*29*).

**Fig. 1.**
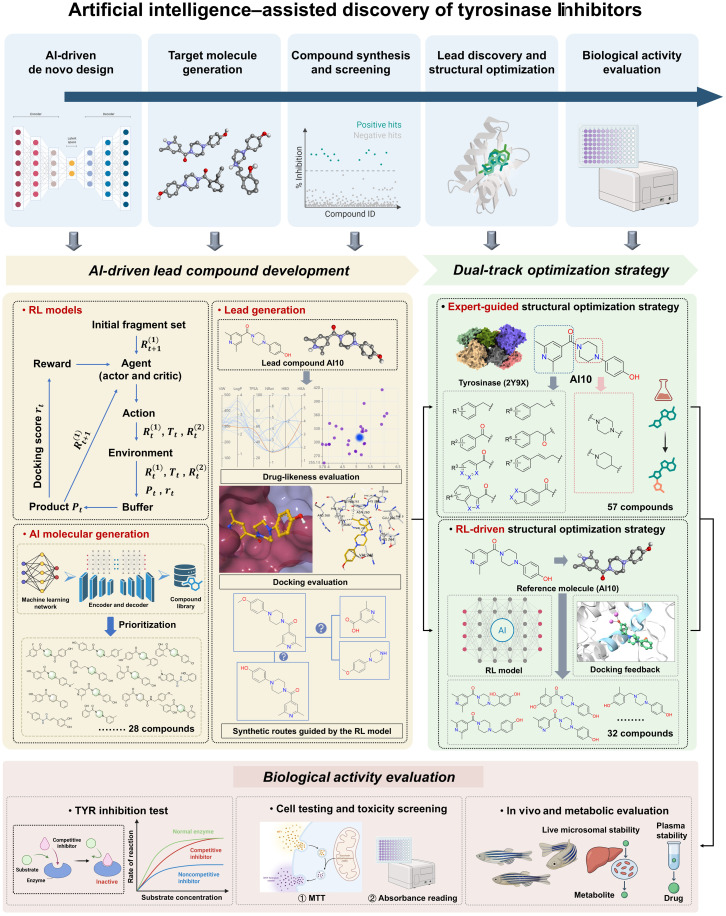
Integrated workflow for TYR inhibitor development combining AI models and conventional drug discovery strategies. The core research approach of this study begins with RL-based de novo molecular generation, followed by the implementation of a dual-track optimization strategy for the resulting lead compound, **AI10**. This strategy encompasses rational design based on medicinal chemistry expertise and AI-driven structural generation, ultimately converging on biological validation of activity and safety. MTT, methylthiazolyldiphenyl-tetrazolium bromide.

In this study, we used an RL-based de novo molecular generation approach to develop previously unreported TYR inhibitors (Fig. 1). Specifically, starting from the three-dimensional structure of TYR and using a fragment-based generation strategy, we constructed a virtual molecular library comprising a large number of generated compounds through the RL algorithm. By comprehensively considering synthetic accessibility and drug-likeness criteria, 28 potential molecules were selected for laboratory synthesis and biological activity evaluation. Among these, compound **AI10** emerged as a promising lead candidate; however, it exhibited moderate activity alongside marked cytotoxicity. To address these limitations, we implemented a dual-track optimization strategy that integrates expert-guided structural optimization with AI-driven structural modification. This approach yielded separate compound libraries from each track, which were then subjected to biological screening.

Using this integrated drug discovery framework, we identified a series of compounds featuring previously unidentified scaffolds and high TYR affinity. Among them, **AI10-m15** exhibited nanomolar TYR inhibitory activity and demonstrated potent antimelanogenic effects both in vitro and in vivo. This study delineates the similarities and distinctions between expert-guided and AI-driven structural optimization strategies, as well as their respective advantages and limitations, providing valuable theoretical insights and methodological guidance for AI-assisted drug discovery.

## RESULTS

### RL-driven de novo molecular generation

**Fig. 2.**
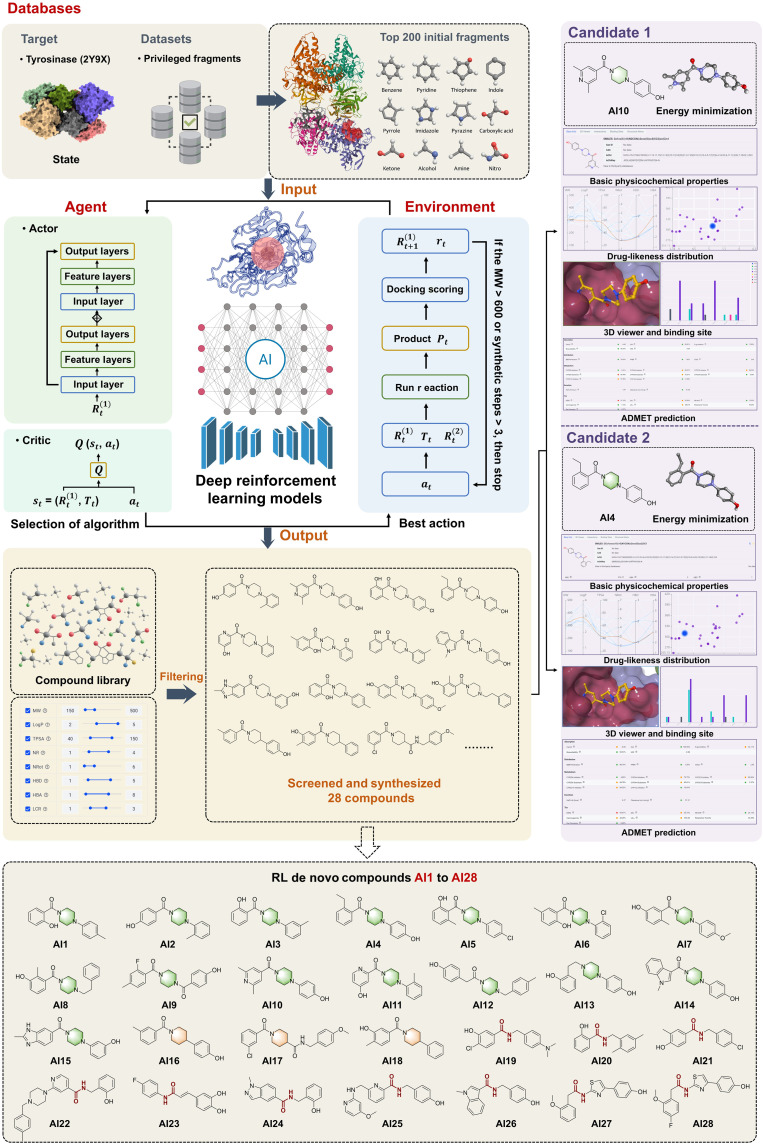
General workflow for RL-assisted de novo generation of TYR inhibitors. The RL-driven molecular generation process was formalized as an MDP, clearly outlining the following steps: Starting from a reaction template library and a fragment library, proceeding through virtual synthesis and docking scoring, incorporating synthesizability and drug-likeness as reward signals, and ultimately obtaining potential active molecules **AI1-AI28** through multistage screening. 3D, three-dimensional; MW, molecular weight.

Here, we establish a unified molecular generation strategy in which RL-driven forward synthesis planning is intrinsically coupled with synthetic feasibility assessment, enabling efficient molecule generation while substantially increasing the likelihood of successful chemical synthesis (Fig. 2). At the core of this method, the molecular generation process is formulated as a Markov decision process (MDP): (i) The state space is defined as the current molecular structure; (ii) the action space corresponds to the set of available chemical reactions or building blocks; and (iii) the reward function jointly evaluates molecular properties such as binding affinity (e.g., docking scores), drug-likeness properties, and synthetic accessibility. To enhance exploration efficiency and prevent the generation of redundant structures, the soft actor-critic (SAC) algorithm was used in combination with a dynamic fragment library strategy. For each design task, an initial fragment pool comprising 200 docking-enriched building blocks was constructed. As the generation proceeded, fragment selection was regulated by a diversity-aware scheme that combined fingerprint-based clustering with synthesis-complexity weighting, thereby maintaining structural novelty while expanding chemical space coverage. Molecule assembly and reaction pathway expansion were further directed by Monte Carlo tree search during the generation process. At each step, less-frequently-used fragments were prioritized from the dynamically updated pool, and the feasibility of the resulting product was predicted via a state-transition probability model. This strategy is distinguished by its concurrent treatment of biological performance and chemical realizability, in which docking-derived activity feedback and retrosynthetic traceability jointly inform the optimization process. In parallel, an adaptive exploration mechanism is implemented through reward modulation that penalizes overused fragments, thereby mitigating redundant structure generation and promoting exploration of underrepresented chemical motifs.

Based on the aforementioned RL-driven iterative molecular generation strategy, a diversity control mechanism was implemented to effectively reduce the redundancy rate of generated molecules. In each iteration, the agent selects a reaction template *T_t_* from a de-redundantized reaction template library according to the current molecular state $R_{t}^{\left(1\right)}$ and performs a virtual synthesis by combining it with a second reactant $R_{t}^{\left(2\right)}$, which is filtered based on molecular fingerprint similarity. This reaction yields molecule *P_t_*. Subsequently, molecular docking is performed between *P_t_* and the target protein TYR [Protein Data Bank (PDB) ID: 2Y9X], and the normalized docking score *r_t_* serves as the reward signal. In parallel, the Tanimoto similarity between the generated molecule and previously generated molecules is calculated and incorporated as a diversity penalty term. The resulting transition tuple ($R_{t}^{\left(1\right)}$, *T_t_*, $R_{t}^{\left(2\right)}$, *P_t_*, and *r_t_*) is stored in a replay buffer using a prioritized experience replay strategy, where low-similarity molecular samples are preferentially sampled to promote structural diversity. If the generated molecule *P_t_* satisfies predefined constraints (molecular weight ≤ 600 Da and synthetic route length ≤ 3 steps), it is assigned as the next state $R_{t+1}^{\left(1\right)}$ for subsequent iterations.

Based on this framework, we generated a set of virtual molecules targeting TYR and constructed a corresponding compound library. The virtual library was subsequently subjected to a multistage screening process to identify candidate molecules with potential bioactivity. Specifically, the screening strategy consisted of two major components: (i) Computational prescreening: This step involved molecular docking and drug-likeness filtering. Particular attention was paid to key interactions within the TYR active site, such as copper ion coordination and hydrophobic pocket binding. Molecules ranking in the top 20% in terms of docking scores were retained. (2) Multidimensional refined screening: This phase incorporated both structural activity analysis and synthetic feasibility evaluation. Compounds containing privileged scaffolds known for TYR inhibition, including phenolic or piperazine motifs, were prioritized. Following this two-tiered selection, 28 potential AI-generated compounds were selected. These candidate compounds were subsequently subjected to actual synthesis, followed by in vitro and in vivo pharmacological evaluations. The results of these pharmacological assays confirmed the effectiveness of the proposed algorithm.

### Synthesis of RL-generated de novo compounds

The 28 target compounds were efficiently synthesized via routes proposed or slightly modified from those generated by the AI model, using only commercially available reagents. Based on differences in the core scaffolds, these compounds were categorized into three series: piperazine derivatives (**AI1-AI15**), piperidine derivatives (**AI16-AI18**), and amide derivatives (**AI19-AI28**) (Fig. 2).

**Fig. 3.**
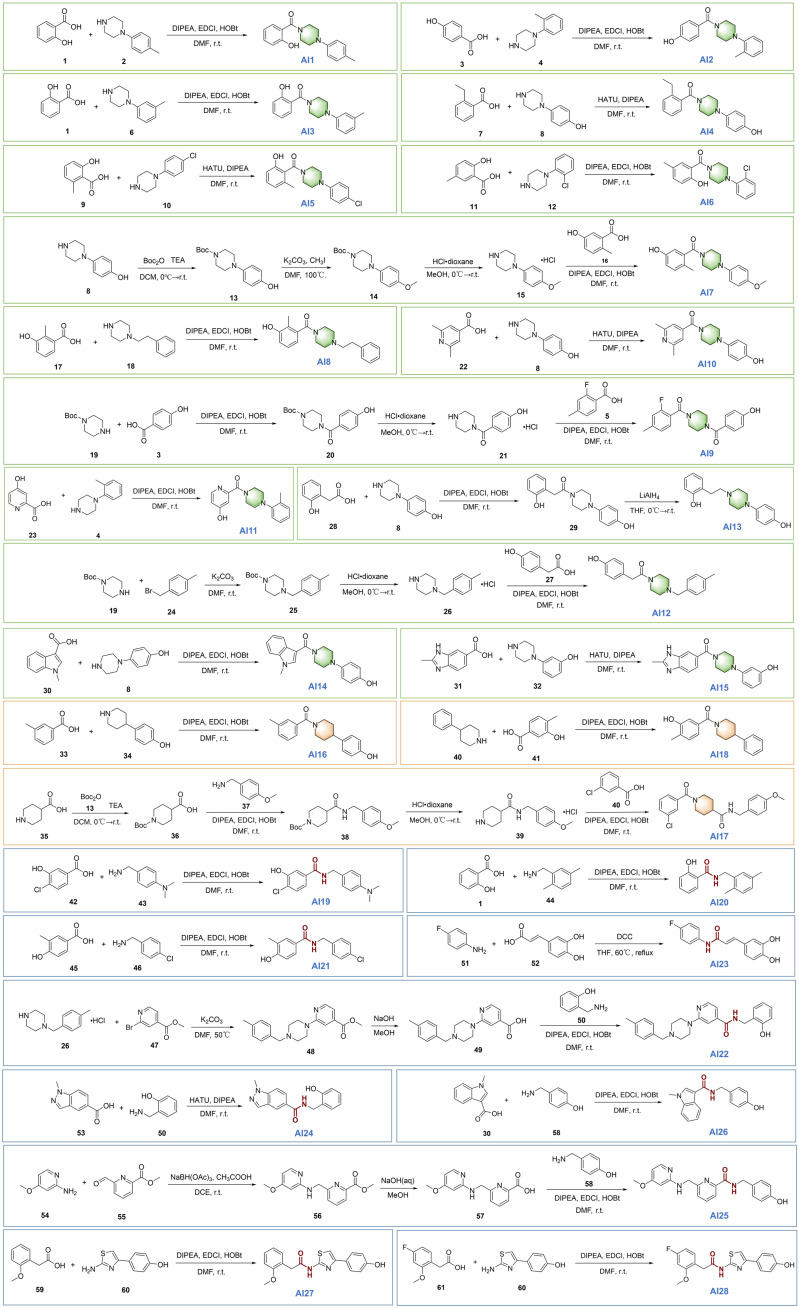
Synthetic strategy for RL de novo generated compounds AI1-AI28. Following the synthetic routes designed (or fine-tuned) by the RL model, 28 target compounds featuring three core scaffolds—piperazines, piperidines, and amides—were successfully synthesized. This achievement provides solid experimental evidence for the chemical feasibility of the AI-generated results and intuitively demonstrates the AI’s preference for specific scaffolds during its exploration of chemical space. r.t., room temperature.

The piperazine series was obtained through amide coupling between various substituted benzoic acid or phenylacetic acid derivatives and piperazine derivatives (Fig. 3). The piperidine derivatives **AI16** and **AI18** were synthesized by coupling 4-phenylpiperidine derivatives with differently substituted benzoic acids. Compound **AI17** was prepared from 4-piperidinecarboxylic acid (**35**) as the starting material, which was first protected with Boc_2_O and then subjected to successive condensations with *p*-methoxybenzylamine and *m*-chlorobenzoic acid to yield the target compound. Most members of the amide series were synthesized by amide condensation of substituted benzoic acids with aromatic amine derivatives. Two compounds in this series, **AI22** and **AI25**, were synthesized via different routes. Compound **AI22** was prepared by reacting a 1-(4-methylbenzyl)piperazine (**26**) intermediate with a 2-bromopyridine derivative (**47**) to form intermediate **48**, followed by hydrolysis and condensation to yield the final product. Derivative **AI25** was synthesized from 4-methoxy-2-aminopyridine (**54**) and methyl 6-formylpyridine-2-carboxylate (**55**) via a sequence of aldol condensation, hydrolysis, and amide coupling reactions.

### Activity validation and toxicity evaluation

The TYR inhibitory activity of 28 AI-generated compounds was assessed using mushroom TYR, with l-dopa and l-tyrosine serving as substrates. Kojic acid and β-arbutin, clinically used TYR inhibitors, were used as positive controls. Overall, 10 out of the 28 AI-designed compounds exhibited notable inhibitory activity (Table 1). For most compounds, the inhibitory activity against monophenolase was superior to that against diphenolase. Among the different structural classes, piperazine derivatives demonstrated superior inhibitory activity compared to piperidine and amide analogs. Notably, compound **AI10** exhibited the most potent activity [l-dopa median inhibitory concentration (IC_50_) = 11.14 ± 1.81 μM and l-tyrosine IC_50_ = 5.93 ± 0.64 μM], being approximately three to four times more potent than kojic acid (l-dopa IC_50_ = 48.17 ± 2.88 μM and l-tyrosine IC_50_ = 19.98 ± 1.36 μM). Detailed comparison of piperazine derivatives revealed that substitution at the terminal aryl group with a *p*-hydroxyphenyl group resulted in enhanced activity. Compounds such as **AI4**, **AI10**, and **AI13** all outperformed kojic acid. In contrast, replacement of the piperazine ring with a piperidine moiety led to a reduction in both drug-likeness (e.g., solubility) and enzymatic activity. Among these, **AI17** demonstrated weak but relatively better inhibition (l-dopa IC_50_ = 346.80 ± 38.89 μM) within the piperidine class. In terms of the amide-based compounds, **AI19** and **AI21** showed moderate TYR inhibitory effects.

**Table 1.** TYR inhibitory activity of AI de novo generated compounds and reference drugs.

Compound	IC_50_ value ± SD (μM, *n* = 3)	
	l-dopa	l-tyrosine
AI1	>100^*^	>100
AI2	>40	>40
AI3	>200	>200
AI4	26.46 ± 4.81	8.91 ± 1.32
AI5	>40	>40
AI6	>40	>40
AI7	>200	>200
AI8	>100	>100
AI9	>800	323.40 ± 43.21
AI10	11.14 ± 1.81	5.93 ± 0.64
AI11	>800	>800
AI12	>800	52.92 ± 2.56
AI13	21.32 ± 3.20	11.45 ± 1.53
AI14	32.76 ± 1.60	24.30 ± 0.49
AI15	500.90 ± 9.12	58.47 ± 2.64
AI16	>100	>100
AI17	346.80 ± 38.89	>800
AI18	>40	>40
AI19	>100	138.60 ± 4.51
AI20	>100	>100
AI21	180.50 ± 69.83	70.66 ± 6.05
AI22	>40	>40
AI23	>800	>800
AI24	>200	>200
AI25	>100	>100
AI26	>100	>100
AI27	>40	>40
AI28	>40	>40
Kojic acid	48.17 ± 2.88	19.98 ± 1.36
β-Arbutin	>800	307.10 ± 65.52

* The upper limits of the specific TYR inhibitory activity testing concentrations (e.g., 40, 100, 200, and 800 μM) were determined by the solubility of the compounds. If a compound exhibits good solubility, further testing of its activity at higher concentrations was conducted. For example, IC_50_ > 100 μM indicates that the maximum soluble concentration of the compound in the solvent is 100 μM.

To further evaluate the pharmacological potential of this promising AI-designed compound, a cellular safety assessment of the most active candidate, **AI10**, was conducted on tumor and normal skin cell lines (A375, B16F10, HEM, and HaCaT) (table S1). The results revealed that **AI10** exhibited a maximum nontoxic concentration (MNC; cell viability ≥ 85%) of 50 μM across multiple cell lines, indicating pronounced cytotoxicity that was markedly higher than that of the reference agents kojic acid (200 μM) and β-arbutin (200 μM).

Therefore, based on comprehensive enzymatic activity assays, **AI10** was selected as the lead compound for subsequent experimental investigations. The rationale for this selection is threefold: (i) It exhibited the most potent TYR inhibitory activity at the enzymatic level (l-tyrosine IC_50_ = 5.93 μM), significantly outperforming the positive control kojic acid; (ii) its structure contains a piperazine ring and a phenolic hydroxyl group, providing well-defined modification sites for subsequent structural optimization; (iii) although it demonstrates moderate cytotoxicity (MNC = 50 μM), this toxicity profile can potentially be improved through structural modifications, such as replacing the piperazine ring with a piperidine ring. In summary, using an AI-driven drug design approach, we successfully identified **AI10** as a potent TYR inhibitor in vitro. Although showing notable cytotoxicity in cellular assays, **AI10** remains a promising lead compound and provides a solid foundation for subsequent structural optimization.

### Structure optimization based on RL-generated compound AI10

To optimize the lead compound **AI10**, we systematically integrated conventional rational design methodologies with AI-driven generative approaches to enhance its TYR inhibitory activity and drug-like properties. Specifically, this study used a dual strategy combining expert-guided drug design with RL-driven molecular generation, thereby advancing structural optimization of the lead compound from two complementary dimensions: chemical space exploration and target-binding mechanism elucidation. The following sections delineate the detailed account of the design principles underlying these strategies and the rationale for the construction of the resulting derivative libraries.

#### Lead optimization strategy I: Expert-guided structural optimization

**Fig. 4.**
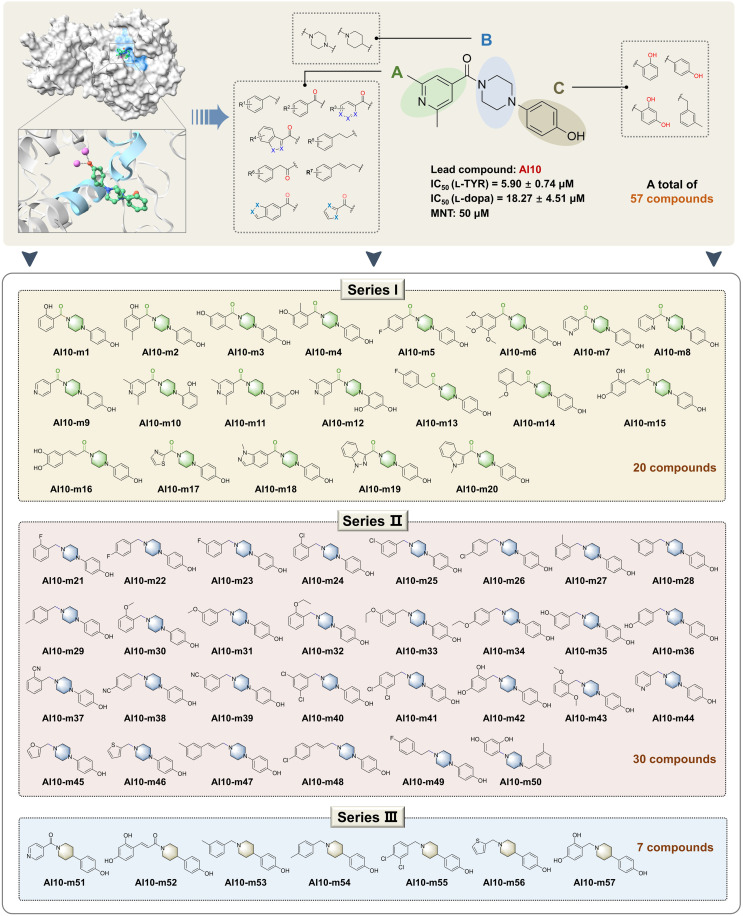
Structural optimization of the lead compound AI10 based on the conventional medicinal chemistry strategy. The systematic structural modification strategy targeting the “A, B, and C regions,” guided by expert experience, was used. Through the design of three compound series—piperazinone, piperazine-benzyl, and piperidine-benzyl—and notably, the introduction of the resorcinol-vinyl moiety as a key pharmacophore, this work illustrates how traditional medicinal chemistry, grounded in a deep understanding of established structure-activity relationships, can efficiently enhance compound potency and drug-likeness. This strategy ultimately yielded ultrapotent inhibitors such as **AI10-m15** and **AI10-m52**.

To further improve its pharmacological profile and the inhibitory potency, we used an expert-guided structural optimization strategy using **AI10** as the lead scaffold (Fig. 4). Systematic modifications were conducted on the A and B regions, while the C-ring was diversified with various substituted aryl groups. Three series of derivatives were designed: piperazinone-based, piperazine-benzyl-based, and piperidine-benzyl-based compounds, resulting in a total of 57 derivatives (**AI10-m1** to **AI10-m57**). Molecular docking analysis of **AI10** revealed that the A region fits directly within the active pocket of TYR and plays a critical role in mediating binding affinity. Therefore, subsequent detailed structural modifications were prioritized in this region. A variety of substituted aromatic and heteroaromatic groups were introduced to investigate the influence of electronic properties (including electron-donating and electron-withdrawing substituents), bioisosteric heterocycles (e.g., pyridine and pyrimidine), and linker types on TYR inhibition. In terms of improving physicochemical properties, we observed that among the 28 compounds generated in the first round, several failed to yield measurable IC_50_ values due to poor solubility. Therefore, solubility-enhancing strategies were deliberately incorporated during subsequent structural modifications, including the introduction of hydrophilic groups (e.g., phenolic hydroxyl groups and piperazine rings), reduction in the number of aromatic rings, and avoidance of excessively hydrophobic substituents.

#### Lead optimization strategy II: RL-driven structural optimization

**Fig. 5.**
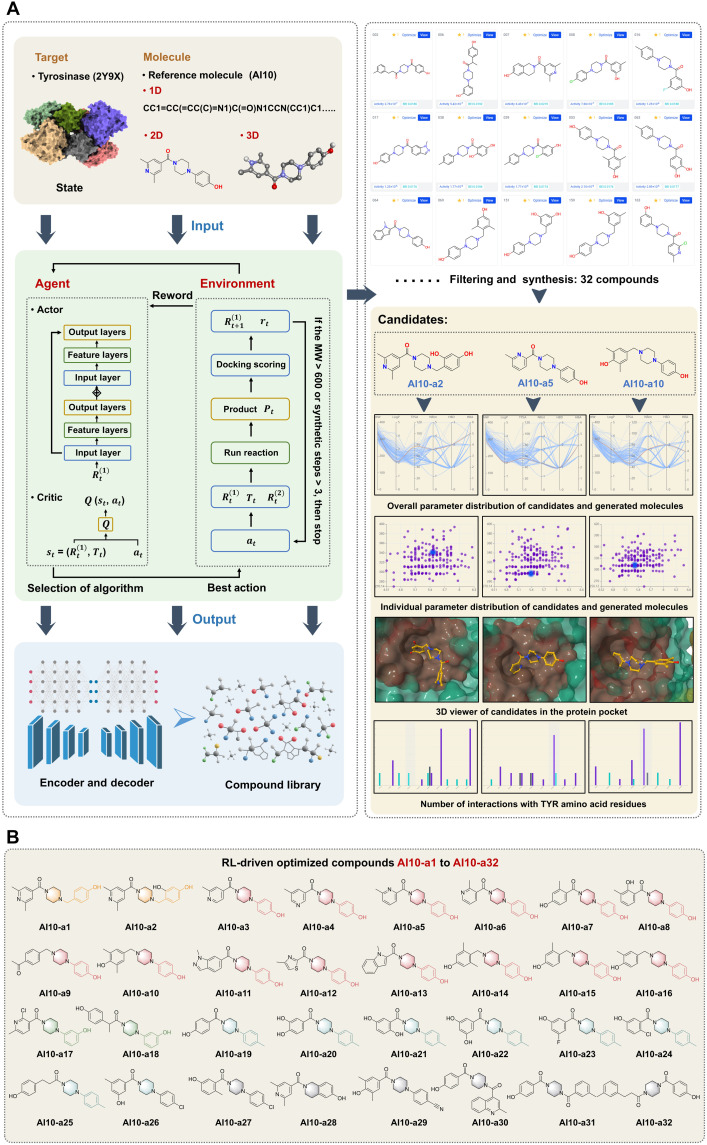
RL-driven structural optimization of lead compound AI10. (**A**) RL-optimized screening workflow. (**B**) Compound structures generated by the RL model and refined through expert evaluation.

Compound **AI10** was selected as the reference scaffold, from which RL-assisted molecular design was carried out to explore structurally related candidates (Fig. 5A). The design workflow followed a fragment-based growth strategy, in which **AI10** was first dissected into a set of representative query fragments to guide subsequent expansion. These query fragments were then recombined with structurally compatible building blocks using predefined reaction templates, enabling stepwise molecular growth. The resulting molecules were assessed by their docking performance and structural resemblance to the AI10 scaffold, with preference given to candidates maintaining comparable architectures and physicochemical characteristics.

RL training and optimization were performed using the ZINC database, a chemically diverse library of molecular fragments. Focusing on compound **AI10** and TYR as the target, multiple iterative molecular generation tasks were conducted. The generated molecules underwent further predictive analyses encompassing activity, ADMET profiles, target engagement, synthetic accessibility, structural alerts, and distributions of both global and individual molecular attributes. Taking into account predicted bioactivity, drug-likeness, synthetic accessibility, and scaffold similarity to **AI10**, 32 candidate molecules (**AI10-a1** to **AI10-a32**) were selected for chemical synthesis (Fig. 5B).

### Synthesis of structurally optimized compounds

For the expert-guided optimized compounds, a total of 57 target compounds were designed based on conventional structural modification strategies and synthesized via the following routes. The piperazine ketone series (**AI10-m1** to **AI10-m20**) was prepared using 4-piperazinophenol (**8**) as the key starting material, which underwent one-step amide condensation with various substituted benzoic acids, phenylacetic acid, or heteroarylcarboxylic acid derivatives to afford the desired structures (fig. S1A). Compounds **AI10-m10** to **AI10-m12** were prepared starting from picolinic acid derivatives (**22**), which were coupled with differently substituted piperidine derivatives via amidation. The piperazine benzyl series (**AI10-m21** to **AI10-m50**) was likewise constructed from 4-piperazinophenol (**8**) via efficient aldol condensation with substituted benzaldehyde derivatives (fig. S2). Notably, compounds **AI10-m47** were synthesized via a distinct route, wherein structural modification was achieved through a nucleophilic substitution reaction between intermediates **104** and **105**. Meanwhile, the piperidine series (**AI10-m51** to **AI10-m56**) was obtained from 4-piperidinophenol (**34**) as the starting material, using either amide condensation or aldol condensation in a single step (fig. S1B). Collectively, using 4-piperazinophenol and 4-piperidinophenol as central building blocks, a modular synthetic strategy was adopted to efficiently access three structurally diverse series, yielding a total of 57 target compounds.

For the RL-driven optimized compounds, a total of 32 target compounds were synthesized according to the RL-driven molecular generation strategy. For the preparation of compounds **AI10-a1** and **AI10-a2**, intermediate **107** was first obtained and subsequently subjected to aldol condensation with *p*-hydroxybenzaldehyde (**90**) and 1,3-dihydroxybenzaldehyde (**96**), respectively (fig. S3). Compounds **AI10-a3** to **AI10-a16** were synthesized from 4-piperazinophenol as the starting material, which underwent single-step condensation with substituted benzoic acid or benzaldehyde derivatives. Compounds **AI10-a17** and **AI10-a18** were prepared from 3-piperazinophenol (**32**) as the precursor, which was coupled with compounds **121** and **122** via amide condensation. Compounds **AI10-a19** to **AI10-a25** were obtained from 4-piperazinetoluene (**2**), which underwent a single-step reaction with a series of substituted benzoic acid derivatives. Last, compounds **AI10-a26** to **AI10-a32** were similarly prepared by condensation of various piperazine derivatives with benzoic acid derivatives.

### TYR inhibitory activity test of structurally modified compounds

The same procedure was applied to evaluate the in vitro TYR inhibitory activities of the compounds obtained from different optimization strategies (expert-guided: 57 compounds; RL-driven: 32 compounds). The results are summarized in Table 2.

**Table 2.** TYR inhibitory activity of the structurally modified compounds and reference drugs.

Compound	IC_50_ value ± SD (μM, *n* = 3)		Compound	IC_50_ value ± SD (μM, *n* = 3)	
	l-dopa	l-tyrosine		l-dopa	l-tyrosine
AI10	17.01 ± 2.39	10.83 ± 1.73	**AI10-m29**	2.46 ± 0.44	2.31 ± 0.03
AI10-m1	14.26 ± 0.67	7.01 ± 0.57	**AI10-m30**	2.56 ± 0.18	2.92 ± 0.06
AI10-m2	11.74 ± 0.84	8.02 ± 0.89	**AI10-m31**	13.57 ± 0.77	3.59 ± 0.30
AI10-m3	17.14 ± 2.91	9.80 ± 0.66	**AI10-m32**	6.20 ± 0.41	2.11 ± 0.07
AI10-m4	7.61 ± 0.40	2.50 ± 0.22	**AI10-m33**	6.44 ± 0.79	4.42 ± 0.38
AI10-m5	16.64 ± 1.93	6.98 ± 0.84	**AI10-m34**	4.18 ± 0.25	4.31 ± 0.13
AI10-m6	4.07 ± 0.08	1.87 ± 0.27	**AI10-m35**	7.04 ± 0.40	1.63 ± 0.08
AI10-m7	5.33 ± 1.01	1.13 ± 0.36	**AI10-m36**	5.55 ± 0.38	4.55 ± 0.67
AI10-m8	4.38 ± 0.51	12.39 ± 1.83	**AI10-m37**	10.38 ± 1.09	6.07 ± 0.15
AI10-m9	2.57 ± 0.98	2.25 ± 0.09	**AI10-m38**	2.75 ± 0.43	1.31 ± 0.11
AI10-m10	12.59 ± 0.40	14.31 ± 1.14	**AI10-m39**	2.40 ± 0.50	1.48 ± 0.09
AI10-m11	>200	120.90 ± 5.08	**AI10-m40**	6.20 ± 0.10	4.44 ± 0.63
AI10-m12	>200	30.61 ± 2.71	**AI10-m41**	0.45 ± 0.09	0.10 ± 0.01
AI10-m13	28.66 ± 2.48	13.66 ± 0.51	**AI10-m42**	0.88 ± 0.33	0.48 ± 0.01
AI10-m14	17.60 ± 1.79	7.63 ± 0.46	**AI10-m43**	6.03 ± 0.52	3.79 ± 0.31
AI10-m15	0.019 ± 0.002	0.020 ± 0.0004	**AI10-m44**	6.52 ± 0.69	4.07 ± 0.26
AI10-m16	7.54 ± 0.44	15.94 ± 0.98	**AI10-m45**	7.39 ± 0.36	1.26 ± 0.26
AI10-m17	9.98 ± 1.95	4.64 ± 0.82	**AI10-m46**	3.95 ± 0.40	1.17 ± 0.22
AI10-m18	9.84 ± 0.21	7.96 ± 0.40	**AI10-m47**	>200	>200
AI10-m19	19.23 ± 2.64	15.47 ± 1.58	**AI10-m48**	4.31 ± 0.62	3.30 ± 0.14
AI10-m20	8.19 ± 1.15	15.48 ± 4.57	**AI10-m49**	27.67 ± 0.09	3.28 ± 0.47
AI10-m21	2.36 ± 0.18	3.00 ± 0.13	**AI10-m50**	6.20 ± 0.69	2.27 ± 0.16
AI10-m22	4.73 ± 0.49	1.73 ± 0.09	**AI10-m51**	39.15 ± 0.55	17.68 ± 1.16
AI10-m23	18.85 ± 1.42	2.95 ± 0.34	**AI10-m52**	0.0063 ± 0.0004	0.0057 ± 0.0002
AI10-m24	20.25 ± 1.62	4.51 ± 0.71	**AI10-m53**	>100	62.56 ± 7.89
AI10-m25	7.08 ± 0.07	1.42 ± 0.10	**AI10-m54**	>100	>100
AI10-m26	4.00 ± 0.47	1.40 ± 0.14	**AI10-m55**	>100	>100
AI10-m27	6.94 ± 0.31	2.11 ± 0.18	**AI10-m56**	>100	65.06 ± 4.39
AI10-m28	2.87 ± 0.68	2.75 ± 0.12	**AI10-m57**	34.24 ± 5.97	10.25 ± 0.83
AI10-a1	>200	48.47 ± 3.59	**AI10-a17**	>200	>200
AI10-a2	0.92 ± 0.21	0.68 ± 0.06	**AI10-a18**	224.60 ± 35.98	29.53 ± 0.88
AI10-a3	17.84 ± 1.03	3.09 ± 0.14	**AI10-a19**	>80	>80
AI10-a4	8.88 ± 0.81	2.31 ± 0.30	**AI10-a20**	53.53 ± 3.58	32.07 ± 2.99
AI10-a5	10.11 ± 0.68	2.21 ± 0.46	**AI10-a21**	43.39 ± 2.52	50.83 ± 1.60
AI10-a6	12.2 ± 1.66	3.72 ± 0.36	**AI10-a22**	>200	>200
AI10-a7	9.84 ± 0.46	4.36 ± 0.27	**AI10-a23**	>80	>80
AI10-a8	13.7 ± 0.57	3.74 ± 0.53	**AI10-a24**	>100	>100
AI10-a9	14.38 ± 0.88	2.18 ± 0.12	**AI10-a25**	31.37 ± 1.56	19.22 ± 0.43
AI10-a10	7.00 ± 0.55	1.55 ± 0.08	**AI10-a26**	73.61 ± 2.71	33.00 ± 1.64
AI10-a11	2.45 ± 0.10	2.33 ± 0.29	**AI10-a27**	>100	97.28 ± 4.09
AI10-a12	17.69 ± 1.08	3.25 ± 0.30	**AI10-a28**	>200	>200
AI10-a13	14.92 ± 1.32	3.89 ± 0.11	**AI10-a29**	>100	>100
AI10-a14	23.43 ± 1.34	3.11 ± 0.10	**AI10-a30**	>100	>100
AI10-a15	9.91 ± 0.53	3.17 ± 0.25	**AI10-a31**	>100	>100
AI10-a16	11.28 ± 1.67	2.63 ± 0.14	**AI10-a32**	>100	>100
β-Arbutin	>800	307.10 ± 65.52	Kojic acid	47.16 ± 2.19	17.55 ± 0.37

**Fig. 6.**
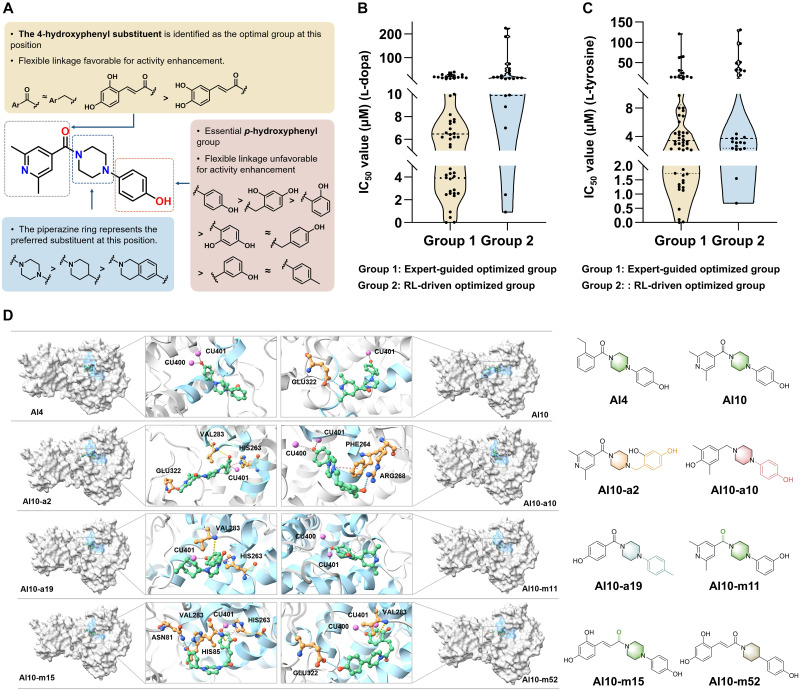
Activity analysis and molecular docking simulation of the modified compounds. (**A**) Structure activity relationship analysis of the modified compounds. (**B**) Activity distribution of optimized compounds under different strategies (l-dopa as the enzymatic substrate). (**C**) Activity distribution of optimized compounds under different strategies (l-tryrosine as the enzymatic substrate). (**D**) Binding mode of compounds **AI4**, **AI10**, **AI10-a2**, **AI10-a10**, **AI10-a19**, **AI10-m11**, **AI10-m15**, and **AI10-m52** with TYR (PDB: 2Y9X) (H-bonds, blue; π-π stack, pink; π-Sigma, yellow; metal acceptor, gray).

On the one hand, among the 57 compounds designed and synthesized through expert-guided modification, the majority exhibited moderate to potent TYR inhibitory activity. Notably, 34 compounds showed superior inhibition to the lead compound **AI10** and the reference drugs. Specifically, compounds **AI10-m52** and **AI10-m15** demonstrated nanomolar-level potency. The piperazinone-based derivatives (**AI10-m1** to **AI10-m20**) obtained through modifications at the A region generally exhibited moderate to potent inhibitory activity. Most maintained activity levels comparable to **AI10**, suggesting that simple electronic modifications in this region had a minimal impact on potency. However, alterations to the hydroxyl substitution pattern on the C-ring resulted in a notable loss of activity, further confirming that the *p*-hydroxyphenyl group represents the optimal conformation for this position (Fig. 6A). Substitution with a resorcinol-vinyl moiety led to a substantial activity enhancement, achieving nanomolar-level potency (**AI10-m15**: l-dopa IC_50_ = 0.019 ± 0.002 μM and l-tyrosine IC_50_ = 0.020 ± 0.0004 μM). Analysis of the piperazine-benzyl series (**AI10-m21** to **AI10-m50**) revealed that the presence of a carbonyl group was not essential for maintaining activity; even in its absence, compounds retained potent TYR inhibitory effects. Most piperazine-benzyl derivatives displayed stronger activity than the lead compound **AI10**, with **AI10-m41** emerging as one of the most promising candidates in this series (l-dopa 0.45 ± 0.09 μM and l-tyrosine IC_50_ = 0.10 ± 0.01 μM). In contrast, most of the piperidine-benzyl derivatives, synthesized by replacing the piperazine ring with a piperidine ring, exhibited negligible activity. However, compound **AI10-m52** was a notable exception, demonstrating exceptionally potent TYR inhibition (l-dopa IC_50_ = 0.0063 ± 0.0004 μM and l-tyrosine IC_50_ = 0.0057 ± 0.0002 μM). Collectively, these findings highlight that the resorcinol-vinyl moiety constitutes a privileged structural motif for optimizing TYR inhibition in the A region. In summary, biological evaluation of these 57 structurally modified derivatives identified **AI10-m15** and **AI10-m52** as highly potent inhibitors, exhibiting 1000-fold and 300-fold activity enhancements, respectively, compared to the parent compound **AI10**.

On the other hand, among the 32 compounds optimized through the RL-driven optimization, the majority exhibited higher TYR inhibitory activity than the positive control kojic acid. Notably, 15 of these compounds showed more potent activity than the lead compound **AI10**, with **AI10-a2** displaying the highest potency, achieving the nanomolar-level inhibition (l-dopa IC_50_ = 0.92 ± 0.21 μM and l-tyrosine IC_50_ = 0.68 ± 0.06 μM). Comparative analysis further indicated that the presence of a 4-piperazinophenol fragment within the structure correlated with enhanced activity (**AI10-a3** to **AI10-a16**), aligning with the trends previously identified. Conversely, substitution at the C-ring with groups such as methyl or halogens led to a marked decrease in activity. In summary, a comparative analysis of the compounds derived from two optimization strategies revealed distinct activity profiles. The majority of the expert-guided modified compounds exhibited superior activity compared to the lead compound **AI10**, with only six not evaluable due to solubility constraints. Notably, compounds **AI10-m52** and **AI10-m15** demonstrated activity enhancements of 1000-fold and 300-fold, respectively. Among the RL-driven generated compounds, approximately half demonstrated higher activity than **AI10**, with **AI10-a2** achieving a 20-fold improvement, while 11 compounds could not be evaluated due to solubility limitations.

Statistical analysis further demonstrated that, irrespective of whether l-dopa or l-tyrosine was used as the enzymatic substrate, the majority of expert-guided compounds exhibited inhibitory activity below 10 μM. In contrast, only six RL-driven compounds displayed activity below 10 μM, and merely two below 5 μM (Fig. 6B). The median activity, represented by the thick line within the violin plot, of the conventional modified compounds was consistently higher than that of the RL-driven group, indicating that the conventional modification strategy yielded compounds with overall more robust activity (Fig. 6, B and C). Collectively, these results suggest that expert-guided modification offers a more stable and predictable route to potent inhibitors. Meanwhile, the RL-driven generation strategy, although more exploratory and inherently less certain, can occasionally produce highly active molecules, but at the expense of a higher proportion of poorly active candidates. This highlights the dual nature of generative models; they offer the potential to explore previously unknown chemical space, yet carry inherent risks in reliability and a higher rate of inactive candidates.

### Molecular docking

Molecular docking studies were performed using Discovery Studio 2023 to investigate the binding interactions between the AI-designed compounds, their optimized derivatives, and TYR. Representative docking poses are illustrated in Fig. 6D. To validate docking accuracy, two molecules with low activity (**AI10-a19** and **AI10-m11**) were analyzed. Both exhibited negligible binding affinity within the catalytic pocket, failing to form hydrogen bond interactions.

In contrast, the active, RL-generated compounds **AI4** and **AI10** exhibited favorable binding modes within the active site. Specifically, **AI10** interacted with copper ion^401^ and formed a hydrogen bond with Glu^322^. The RL-optimized derivative **AI10-a2** displayed enhanced interactions, including coordination with copper ion^401^, hydrogen bonding with Glu^322^, π-π stacking with His^263^, and π-σ interactions with Val^283^. Another derivative, **AI10-a10**, engaged in dual interactions with copper ion^400^ and copper ion^401^, as well as forming a hydrogen bond with Arg^268^ and π-π stacking with Phe^264^.

Among manually modified derivatives, **AI10-m15** formed two hydrogen bonds with Asn^81^ and His^85^, whereas **AI10-m52** engaged in dual copper coordination (with both copper ion^401^ and copper ion^400^), hydrogen bonding with Glu^322^, and π-σ interactions with Val^283^. Notably, **AI10-m15** and **AI10-m52** formed more extensive and complex interaction networks compared to AI-optimized molecules, which may underlie their substantially enhanced inhibitory activity against TYR.

### Cytotoxicity evaluation

**Fig. 7.**
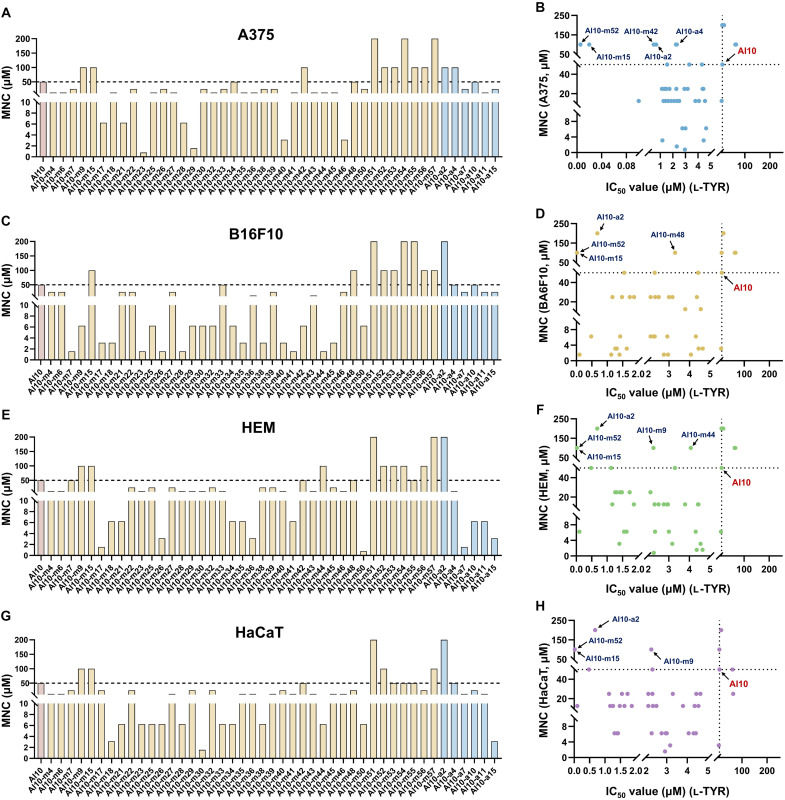
Comprehensive evaluation of cellular safety and multiparameter analysis of the compounds. MNC (cell viability ≥ 85%). (**A**) Cytotoxicity of compounds in the A375 cell line. (**B**) Integrated assessment of compounds on their respective intracellular MNC values (A375) and L-tyrosine inhibitory activity. (**C**) Cytotoxicity of compounds in the B16F10 cell line. (**D**) Integrated assessment of compounds on their respective intracellular MNC values (B16F10) and L-tyrosine inhibitory activity. (**E**) Cytotoxicity of compounds in the HEM cell line. (**F**) Integrated assessment of compounds on their respective intracellular MNC values (HEM) and L-tyrosine inhibitory activity. (**G**) Cytotoxicity of compounds in the HaCaT cell line. (**H**) Integrated assessment of compounds on their respective intracellular MNC values (HaCaT) and L-tyrosine inhibitory activity.

Based on preliminary TYR inhibition screening, candidate compounds exhibiting superior activity were selected for further assessment of cellular safety. Evaluations were performed using two melanoma cell lines: the human-derived A354 (Fig. 7A) and murine-derived B16F10 cell lines (Fig. 7C). Initial experiments revealed that the lead compound **AI10** exhibited marked cytotoxicity in vitro, with an MNC of only 50 μM. Considering that the piperazine ring in **AI10** may represent a potential source of toxicity, structural modifications were implemented by replacing the piperazine moiety with a safer piperidine scaffold in several enzyme-inhibitory analogs (**AI10-m51** to **AI10-m57**). The results demonstrated that most of these derivatives exhibited improved safety profiles (e.g., MNC: 3.125 μM for **AI10-m29** versus 200 μM for **AI10-m54**), highlighting the advantage of expert-guided structural modification.

Overall, several derivatives (e.g., **AI10-m15**, **AI10-m42**, **AI10-m51**, **AI10-m52**, and **AI10-a2**) displayed substantially improved cellular safety, with MNC values ≥100 μM. Notably, **AI10-m15**, **AI10-m52**, and **AI10-a2** not only maintained but markedly enhanced TYR inhibitory activity, while doubling the MNC (to 100 μM) compared to **AI10** (50 μM), thereby effectively broadening the therapeutic window (Fig. 7, B and D).

To assess potential off-target toxicity, the MNCs of the target compounds were further evaluated in normal human epidermal melanocytes (HEMs) (Fig. 7E) and immortalized human keratinocytes (HaCaT) (Fig. 7G). The results revealed that, relative to **AI10**, compounds **AI10-m9**, **AI10-m15**, **AI10-m51**, **AI10-m52**, **AI10-m57**, and **AI10-a2** exhibited improved cellular safety (MNC ≥ 100 μM). Considering their corresponding TYR inhibitory activities, compounds **AI10-m9**, **AI10-m15**, **AI10-m52**, and **AI10-a2** demonstrated simultaneous improvements in both safety and enzymatic inhibition potency (Fig. 7, F and H). In summary, through the synergistic integration of expert-guided and RL-driven optimization strategies, three promising drug-like candidates—**AI10-m15**, **AI10-m52**, and **AI10-a2**—were identified. These compounds exhibited both potent TYR inhibitory activity coupled with superior cellular safety. Their overall performance markedly surpassed that of the lead compound **AI10**, highlighting strong potential for further pharmacological development.

### Skin permeability prediction

Good skin permeability represents a critical determinant of a molecule’s druggability, particularly for topical applications. The logarithm of the skin permeation coefficient (log *K*_p_) serves as a well-established parameter for evaluating compound penetration through the skin barrier. According to the classical model proposed by Potts and Guy in 1992 (*30*), log *K*_p_ values ranging from −3 to −1 cm/hour typically indicate favorable skin permeability. This range characterizes moderately lipophilic compounds that have sufficient hydrophobicity to traverse the lipid-rich stratum corneum while maintaining adequate aqueous solubility for subsequent absorption into the dermal layer. Values below −3 cm/hour suggest poor permeability, often resulting from excessive hydrophilicity, whereas values above −1 cm/hour may indicate excessive lipophilicity that could lead to compound sequestration within the stratum corneum. This threshold is widely recognized in studies of both transdermal drug delivery and cosmetic absorption.

In this study, we systematically evaluated candidate molecules using the pkCSM pharmacokinetic prediction platform (*31*). The results are summarized in table S2. Preliminary analysis revealed that the lead compound **AI10** exhibited a relatively low log *K*_p_ (−3.371 cm/hour), indicating limited skin permeability. In contrast, derivatives obtained through rational structural modifications (**AI10-m15** and **AI10-m52**), along with an AI-optimized analog (**AI10-a2**), showed markedly improved log *K*_p_ values, suggesting enhanced skin penetration. To further elucidate the impact of physicochemical properties on transdermal behavior, we used the SwissADME platform to predict the lipophilicity parameter (iLog *P*) of the aforementioned compounds, with the results presented in table S2. A combined analysis of iLog *P* and log *K*_p_ indicated that future optimization of candidate molecules should focus on moderately modulating lipophilicity, reducing polar surface area, or optimizing the number of hydrogen bond donors and acceptors, while maintaining activity, to achieve enhanced transdermal performance. Collectively, these findings demonstrate that strategic structural optimization notably improved the skin permeability of the lead compound, thereby establishing a robust foundation for subsequent transdermal drug development initiatives.

### Intracellular melanogenesis inhibition assay

**Fig. 8.**
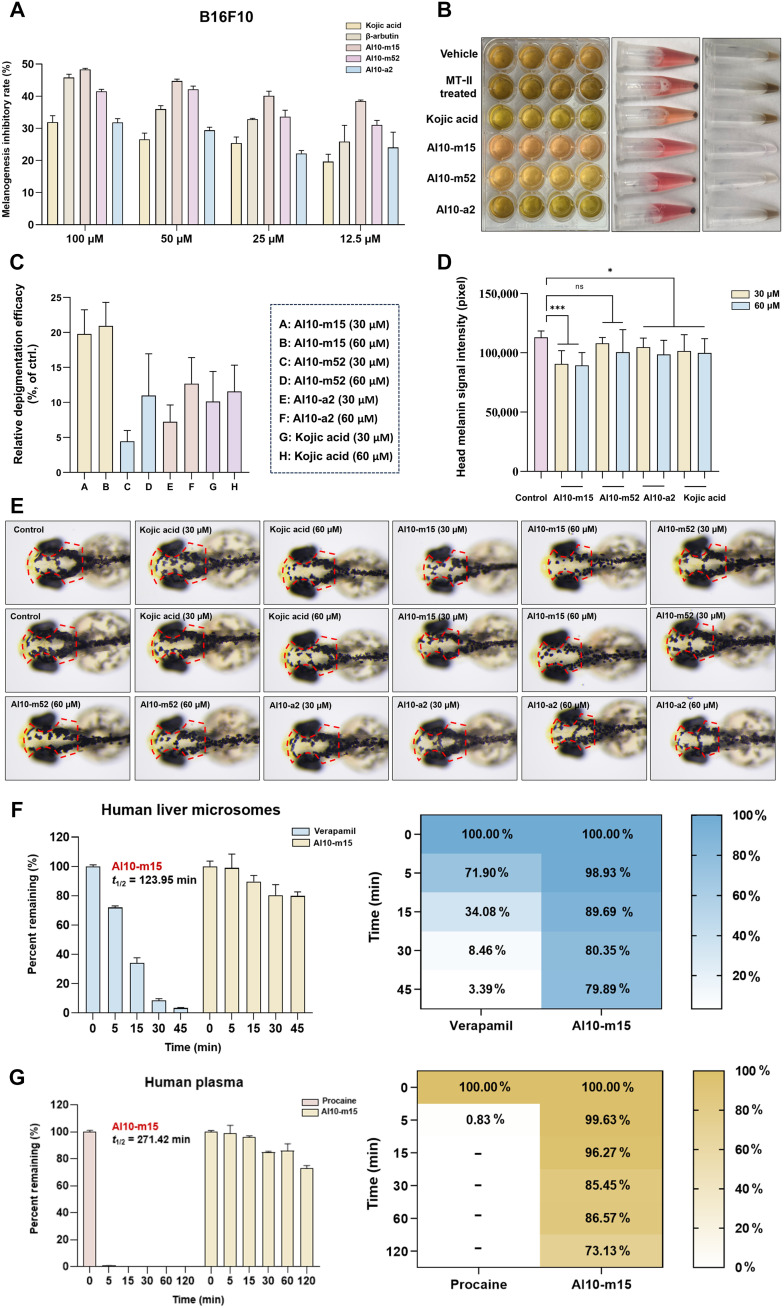
Evaluation of melanogenesis inhibition of active compounds and the positive control. (**A**) Dose-dependent intracellular melanogenesis inhibitory rate of candidate compounds and positive control on B16F10 cells at various concentrations. (**B**) Melanin deposition in B16F10 cells after 72 hours coincubation with candidate compounds and MT-II. (**C**) Comparative depigmentation efficacy among different compounds. ctrl., control. (**D**) Quantification of head melanophore signal intensity in zebrafish embryos. (**E**) Representative images of zebrafish embryos under a dissecting microscope. Data are presented as means ± SD (^*^P < 0.05; ^**^P < 0.01; ^***^P < 0.001, compared to the control group, *n* = 8). (**F**) Metabolic profile of compound **AI10-m15** and verapamil in HLMs. (**G**) Metabolic profile of compound **AI10-m15** and procaine in human plasma.

The ability of selected candidates (**AI10-m15**, **AI10-m52**, and **AI10-a2**) to suppress intracellular melanin production was examined in B16F10 cells, and their melanogenesis inhibition rates were directly compared with those of the reference inhibitors kojic acid and β-arbutin under optimized experimental conditions (*22*). Melanogenesis was stimulated with melanotan II (MT-II; 100 nM), and then, the cells were treated with a range of concentrations of the test compounds (Fig. 8A). The results demonstrated a clear dose-dependent inhibition of melanin synthesis, with intracellular melanin levels decreasing progressively as compound concentrations increased. Among all tested molecules, compound **AI10-m15** exhibited the most potent inhibitory activity, surpassing both kojic acid and β-arbutin. Compound **AI10-m52** showed efficacy comparable to β-arbutin and superior potency to kojic acid, whereas **AI10-a2** displayed the weakest inhibitory effect, similar to kojic acid.

To visualize and corroborate the inhibition of intracellular melanin synthesis at the cellular level, B16F10 cells stimulated with MT-II (100 nM) were coincubated with each compound at a concentration of 100 μM. Macroscopic examination of 24-well plates, pelleted cell masses, and dissolved cell lysates revealed that MT-II stimulation led to pronounced darkening of the cells, reflecting elevated melanin production. In contrast, treatment with the test compounds resulted in visibly lighter cell pellets, confirming their melanogenesis-suppressing effects (Fig. 8B). Consistent with the quantitative data, **AI10-m15** demonstrated the strongest melanin inhibition, followed by **AI10-m52**, while **AI10-a2** showed the weakest effect.

In summary, these findings indicate that both expert-guided and RL-driven molecular optimization strategies successfully yielded structurally modified derivatives (**AI10-m15**, **AI10-m52**, and **AI10-a2**) with varying degrees of antimelanogenic efficacy. Notably, **AI10-m15** emerged as the most potent inhibitor, underscoring the superior effectiveness of the expert-guided optimization strategy in improving antimelanogenic efficacy.

### Melanin inhibition assay in zebrafish embryos

The in vivo antimelanogenic efficacy of the lead compound **AI10** and its structurally optimized derivatives (**AI10-m15**, **AI10-m52**, and **AI10-a2**) was systematically evaluated using zebrafish embryos at 6 hours postfertilization (hpf), with head melanophore signal intensity serving as a quantitative end point. Zebrafish embryos are a well-established model for pigmentation studies due to their early-stage transparency, readily observable melanophore formation, and the neural crest origin of melanocytes, which allows modulation of their proliferation, differentiation, and migration to suppress melanin biosynthesis (*32*).

Based on cytotoxicity profiling, kojic acid (positive control) and the derivatives **AI10-m15**, **AI10-m52**, and **AI10-a2** were tested at concentrations of 30 and 60 μM. However, due to its inherent toxicity, **AI10** was restricted to 30 μM. Embryos treated with **AI10** exhibited notable toxicity and were consequently excluded from the final statistical analysis. In contrast, all structurally optimized derivatives displayed minimal or negligible toxicity, indicating that the implemented modification strategies successfully enhanced compound biocompatibility. Regarding antimelanogenic efficacy, **AI10-m15** treatment resulted in a pronounced reduction of head pigmentation in zebrafish embryos, highlighting its marked antimelanogenic activity (depigmentation efficacy: 20.93 ± 3.37%, 60 μM). **AI10-a2** also demonstrated measurable but weaker inhibitory effects (depigmentation efficacy: 12.68 ± 3.72%, 60 μM), whereas **AI10-m52** showed no appreciable antimelanogenic activity (Fig. 8, C and D). These findings were visually corroborated by microscopic examination and subsequent quantitative analysis (Fig. 8E). In summary, comparative analysis revealed that compound **AI10-m15** exhibited the most potent antimelanogenic activity in vivo. When integrated with its previously demonstrated robust in vitro enzymatic inhibition (l-dopa IC_50_ = 0.019 ± 0.002 μM and l-tyrosine IC_50_ = 0.020 ± 0.0004 μM) and favorable cellular safety profile (MNC = 100 μM), **AI10-m15** emerges as a highly promising candidate warranting further in-depth investigation.

### Metabolic stability study

An in vitro metabolic stability assessment was systematically performed to characterize the metabolic profile of the optimized compound **AI10-m15** in both human liver microsomes (HLMs) and human plasma, with established reference drugs included for comparison. As shown in Fig. 8F, **AI10-m15** exhibited excellent metabolic stability in the HLM system. Its half-life (*t*_1/2_) was determined to be 123.95 min, substantially exceeding that of the reference drug verapamil. After 45 min of incubation, ~79.89% of the parent compound remained intact, further confirming its high enzymatic stability. Key pharmacokinetic parameters were calculated: intrinsic clearance (*CL*_int_) = 12.93 ml/min/kg, hepatic extraction ratio (*E*_h_) = 38.45%, and predicted hepatic clearance (*CL*_h_) = 7.96 ml/min/kg. Collectively, these parameters indicate that **AI10-m15** undergoes slow clearance through CYP450-mediated phase 1 metabolism, suggesting the potential for prolonged systemic circulation. In plasma stability assays (Fig. 8G), **AI10-m15** similarly displayed remarkable stability, with a plasma half-life of 271.72 min. Even after 120 min of incubation, 73.13% of the parent compound remained, in sharp contrast to the rapid hydrolysis observed for the positive control procaine (*t*_1/2_ < 5 min). These results demonstrate that **AI10-m15** is resistant to degradation by hydrolytic enzymes such as esterases and proteases in plasma, thereby avoiding a common pathway of metabolic inactivation. In summary, **AI10-m15** demonstrated outstanding metabolic stability in both HLM and plasma, suggesting that it may have a long in vivo half-life and favorable pharmacokinetic properties. These findings provide important in vitro metabolic evidence supporting its further development as a promising drug candidate.

### The collision between AI and medicinal chemists: Competition or collaboration?

This study implemented two fundamentally distinct lead compound optimization strategies in parallel: an AI-driven and an expert-guided structural optimization strategy. These paradigms differed markedly in their underlying philosophy, execution workflows, and outcomes—prompting a critical examination of whether AI and medicinal chemists are best viewed as competitors or collaborators in modern drug discovery. The AI-driven structural optimization strategy demonstrated remarkable exploratory capacity and creativity. Leveraging reaction template-based fragment assembly and RL-guided molecular generation, the model autonomously explored chemical space without human bias, producing previously unexplored structurally distinct and highly diverse compounds. For instance, beginning with the lead compound **AI10**, the RL model generated a range of structural variants, among which **AI10-a2** exhibited nanomolar-level TYR inhibition—representing a 20-fold improvement over **AI10**. This validated AI’s ability to achieve “leapfrog” structural innovation that may elude conventional human design intuition. However, the approach also revealed inherent limitations: A notable proportion of generated molecules were ultimately discarded due to low synthetic accessibility or suboptimal physicochemical properties. Moreover, the overall activity distribution of AI-optimized compounds was broad, with lower structural stability compared to expert-designed analogs. These observations underscore that while AI can transcend human cognitive boundaries, its practical performance remains heavily dependent on the quality of training data, the design of reward functions, and the implementation of rigorous synthetic feasibility constraints.

In contrast, the expert-guided structural optimization strategy highlighted the enduring strengths of human reasoning and medicinal chemistry expertise. Through systematic modification of the A and B rings of **AI10**, guided by molecular docking results and structure-activity relationship (SAR) analyses, the researchers introduced carefully considered electronic substitutions, bioisosteric replacements, and linker optimizations. This strategy yielded compounds with markedly improved potency and safety profiles. For example, derivatives **AI10-m15** and **AI10-m52** achieved nanomolar inhibitory activity while exhibiting reduced cytotoxicity and enhanced skin permeability. The advantages of this human-centric approach lie in its interpretability, mechanistic clarity, and synthetic tractability, although its exploration breadth is naturally constrained by the scope of existing medicinal chemists’ knowledge and time-intensive iteration.

Therefore, should the dynamic between AI and medicinal chemists be characterized as competitive or collaborative? Our dual-track optimization framework provides compelling evidence that the two function not as adversaries but as synergistic partners. AI serves as a powerful exploratory engine, expanding the frontiers of drug discovery and generating unconventional molecular hypotheses beyond the immediate reach of human intuition. Medicinal chemists, in turn, constitute the indispensable intelligent decision-making core, ensuring the chemical plausibility, developmental quality, and translational potential of the designed compounds. By effectively transforming potential competition into productive collaboration, this study establishes a hybrid optimization paradigm that effectively balances radical innovation with practical applicability. Ultimately, the future of drug discovery will be defined not by the replacement of human expertise with computational systems, but by their deep integration—a model of human-AI symbiosis that amplifies collective creativity, precision, and overall discovery efficiency.

## DISCUSSION

In this study, we established an innovative drug discovery paradigm—“AI-driven de novo lead generation followed by dual-track AI and medicinal chemists–led optimization”—that integrates the compound modification experience of medicinal chemists with AI to systematically advance the development of TYR inhibitors. Beginning with the AI-generated lead compound **AI10**, we conducted two parallel optimization pathways: expert-guided and RL-driven structural optimization. This dual-track strategy not only notably expanded the breadth of chemical space exploration but also leveraged human expertise to ensure the structural soundness and synthetic feasibility of the resulting candidate molecules.

Specifically, the expert-guided optimization yielded **AI10-m15**, a potential candidate that demonstrated superior antimelanogenic activity, metabolic stability, and cellular safety in vitro and in vivo compared to both the lead compound **AI10** and positive controls. Concurrently, the AI-driven strategy successfully generated a series of structurally distinct compounds, with **AI10-a2** achieving nanomolar-level activity, validating the distinct capability of generative models to discover nontraditional chemotypes. These outcomes collectively illustrate that the strengths of AI, and medicinal chemists are complementary and synergistic, not competitive. AI excels in exploring uncharted chemical space and identifying “leapfrog” active molecules, whereas expert-guided modification provides a robust framework for predictably optimizing activity and safety profiles.

Looking forward, this expert-AI collaborative paradigm presents both opportunities and challenges for future development, such as the following: For opportunities, (i) advances in multimodal learning will empower AI to more comprehensively integrate chemical, biological, and pharmacological data, enabling more precise prediction and generation of optimized molecular properties; (ii) progress in automated synthesis and high-throughput screening technologies provides crucial infrastructure for rapid experimental validation and iterative optimization of AI-generated compounds; and (iii) AI-driven exploration can effectively broaden the conceptual horizons of medicinal chemists, providing groundbreaking design hypotheses. Thus, integrating AI with medicinal chemistry expertise represents a more efficient, innovative, and viable strategic direction for future drug discovery. For challenges, (i) the interpretability of AI models requires improvement to achieve “white-box” generation that better incorporates domain knowledge; (ii) accurate prediction of synthetic feasibility for AI-generated molecules remains limited and demands more sophisticated reaction prediction models; (iii) the complexity of biological systems continues to challenge accurate prediction of target interaction networks and off-target effects; and (iv) medicinal chemists remain indispensable in the final selection of candidate compounds; exclusive reliance on AI predictions remains risky and may lead to costly experimental failures.

While this study focuses on TYR inhibition as a proof-of-concept application, the proposed RL framework is not restricted to this specific target. Because the molecular generation process is formulated as a general MDP and optimized using a policy-learning algorithm, the framework can, in principle, be applied to other protein targets or molecular design tasks by modifying the reward evaluation module. For example, docking-based scoring could be replaced or complemented with machine learning–based binding affinity predictors, pharmacophore matching, or experimentally derived activity models. Furthermore, the reward function can naturally accommodate additional objectives, such as ADMET properties, toxicity prediction, or scaffold constraints, enabling more complex multiobjective optimization tasks in drug discovery. Nevertheless, several limitations should be noted. In the present implementation, docking scores are used as a proxy for binding affinity, which may introduce biases due to the inherent limitations of empirical scoring functions. In addition, the current experiments were conducted using a single target and a specific scaffold class, and further validation across multiple targets would be valuable to systematically evaluate the transferability of the approach. Future work will therefore focus on extending the framework to diverse biological targets and integrating more accurate activity prediction models to further improve generalization capability.

In summary, this study not only identified **AI10-m15** as a highly promising TYR inhibitor but also provided crucial methodological insights for modern drug discovery. We anticipate that future drug discovery and development will increasingly rely on intelligent expert-AI collaboration. Through the deep integration of generative models with automated synthesis and high-throughput validation platforms, the efficient translation from in silico designs to preclinical candidates becomes achievable, ultimately driving the intellectual transformation of drug discovery paradigms. This integrated model is expected to provide a solution for complex disease treatment, paving the way for collaborative pharmaceutical innovation.

## MATERIALS AND METHODS

### De novo molecular generation

#### Overview of the RL model

An RL framework was formulated for de novo molecular generation, conceptualized as an MDP. The objective of the agent was to sequentially construct molecules with high affinity for the target protein TYR (PDB ID: 2Y9X) by selecting synthetic steps from a defined chemical space. The SAC algorithm was adopted, owing to its strong capability in handling continuous action spaces and its inherent maximum-entropy framework, which facilitates a principled balance between exploration and exploitation.

#### Construction of chemical libraries

A reaction template library was derived from the USPTO 1K TPL dataset. The 300 most frequently occurring reaction templates were selected to ensure broad coverage of synthetically accessible chemical transformations. This final set comprised 68 unimolecular and 232 bimolecular reaction templates. A large collection of ~100,000 commercially available small molecules (obtained from TOPSCIENCE Company) served as the initial pool for the building block library (*33*). This pool was subjected to a filtering process to retain fragments with favorable drug-like properties. The filtering criteria were as follows: molecular weight < 250, clog *P* < 2, number of hydrogen bond donors ≤ 2, and number of hydrogen bond acceptors ≤ 4. This process yielded a refined library of 13,000 building blocks. To initialize the molecular generation process, a set of initial fragments was required. A library of 1500 fragment molecules was generated by applying the BRICS (Breaking of Retrosynthetically Interesting Chemical Substructures) algorithm to a set of Food and Drug Administration–approved drugs (*34*). The resulting fragments were deduplicated and manually verified for chemical validity. For each fragment, the most structurally similar molecule from the molecular building block library was identified using Morgan fingerprints (radius = 2, 1024 bits), thereby creating a starting fragment library with high synthetic feasibility.

#### Molecular generation and training process

The molecular generation task was modeled as an MDP M=(S,A,R,T), where each episode corresponds to the stepwise construction of a single molecule.

1) State (s∈S): The state s was defined as the current partial molecular structure, represented as a SMILES string, the first reactant $R_{t}^{\left(1\right)}$, the second reactant $R_{t}^{\left(2\right)}$, and the generated product molecule *P_t_*.

2) Action (a∈A): The action space consisted of selecting a reaction template (*T_a_*) and, for bimolecular templates, a second reactant $\left[R_{t}^{\left(2\right)}\right]$ from the respective libraries.

3) State transition (T): Upon action selection, the environment (a chemical reaction simulator) applied the selected template to the current molecule and the chosen reactant $\left[R_{t}^{\left(2\right)}\right]$, if applicable to generate a product molecule (*P_t_*), which became the previously unidentified state.

4) Reward (*r*): The reward signal was computed immediately after product generation. The product *P_t_* was docked into the binding pocket of the target protein TYR (PDB ID: 2Y9X) using AutoDock Vina. The resulting docking score was negated and used as the reward *r_t_*, thus encouraging the generation of high-affinity binders. The reward at step *t* is now defined as the following

$$r_{t}=W_{d}S_{\text{dock}}\left(P_{t}\right)+W_{l}S_{\text{lip}}\left(P_{t}\right)+W_{s}S_{\text{syn}}\left(P_{t}\right)-\lambda S_{\text{div}}\left(P_{t}\right)$$

where *P_t_* is the generated product molecule at step *t*; *S*_dock_ is the normalized docking score obtained from AutoDock Vina; *S*_lip_ is the normalized drug-likeness score computed from Lipinski’s Rule-of-Five descriptors using RDKit (*35*); *S*_syn_ is the normalized synthetic accessibility score, which combines fragment contributions and molecular complexity penalties to approximate the ease of chemical synthesis (*36*); *S*_div_ is a diversity penalty term calculated as the Tanimoto similarity between Morgan fingerprints (*37*); *W*_d_, *W*_l_, and *W*_s_ are weighting coefficients, which are set to 0.6, 0.2, and 0.2, respectively; and λ controls diversity regularization. The components are combined through a weighted linear formulation.

Unlike the hard filtering applied during the postscreening stage, these scores were incorporated into the reward function as soft constraints to guide the RL process. To ensure numerical comparability and stable optimization, all components are minimum-maximum normalized, as illustrated below with the example of $S\left(P_{t}\right)$

$$S\left(P_{t}\right)=\frac{S\left(P_{t}\right)-S_{\text{min}}}{S_{\text{max}}-S_{\text{min}}}$$

Docking scores (negative binding energies) are first negated before normalization. Synthetic accessibility scores are inversely scaled such that higher synthetic feasibility corresponds to higher reward values. The final reward is clipped to the interval [−5,5] to prevent gradient instability.

It should be noted that docking scores provide only an approximate estimation of binding affinity. The empirical scoring function implemented in AutoDock Vina simplifies intermolecular energetics and does not explicitly account for solvent effects, entropic contributions, or full protein flexibility. Consequently, docking scores may exhibit ranking inaccuracies and limited quantitative correlation with experimental binding affinities. In this framework, docking scores are therefore treated as heuristic optimization signals rather than precise thermodynamic predictors. To mitigate potential bias arising from scoring inaccuracies, docking evaluation is integrated with drug-likeness constraints, synthetic accessibility assessment, diversity regularization, and subsequent experimental validation. This multiobjective reward formulation reduces the risk of reward exploitation and promotes chemically realistic molecular generation.

1) Experience replay and training: Each transition tuple (*s_t_*, *a_t_*, *r_t_*, and *s*_*t*+1_) was stored in an experience replay buffer. The SAC neural networks (an actor and two critic networks) were updated every 32 episodes. A mini-batch of experiences was randomly sampled from the replay buffer to train the networks, minimizing the policy and value function losses as defined by the SAC algorithm. The temperature parameter (α) was automatically tuned to manage the trade-off between reward maximization and policy entropy.

2) Termination conditions: An episode terminated if any of the following conditions were met: (i) the number of synthetic steps reached a predefined maximum of 3; (ii) the molecular weight of the product exceeded 600 Da; (iii) no valid reaction template applied to the current molecule; or (iv) the reaction execution failed.

#### Compound selection and postprocessing

The model was trained for 20,000 episodes. Upon completion, all successfully generated molecules were collected into a virtual library. This library was first filtered for drug-likeness using Lipinski’s Rule of Five. The top 200 molecules with the most favorable docking scores against TYR were retained. From this shortlist, a final set of 28 compounds (designated **AI1-AI28**) was selected for synthesis. This selection integrated computational data with medicinal chemistry expertise, prioritizing molecules that contained key pharmacophores for TYR binding and for which the AI-proposed synthetic routes were deemed straightforward and cost-effective.

Although the present study focuses on TYR as the target protein, the RL framework is designed to be modular and target-agnostic. The MDP formulation, reaction template–based molecular construction strategy, and SAC optimization algorithm are independent of the specific biological target. In practice, adapting the framework to other targets primarily requires replacing or extending the reward evaluation module. For example, the docking-based activity score used in this work can be substituted with alternative predictors such as machine learning–based binding affinity models, pharmacophore matching scores, or quantitative structure-activity relationship (QSAR) models. Additional objectives, including ADMET properties or physicochemical constraints, can also be incorporated into the reward function to enable multiobjective optimization in diverse drug discovery scenarios.

### RL-driven molecular generation from template AI10

We used an RL-driven fragment growth strategy to generate a series of previously unreported, synthetically accessible TYR inhibitors (**AI10-a1** to **AI10-a32**) starting from the lead compound **AI10**. The detailed procedure is as follows.

#### Step 1: Reference molecule fragmentation

The reference molecule **AI10** was iteratively fragmented using the BRICS algorithm via single-bond cleavage to produce multiple query fragments.

#### Step 2: Similar fragment retrieval

Molecular Morgan fingerprints were computed for the query fragments and compared against the ZINC building block library using Dice similarity. Fragments with a similarity score ≥ 0.5 were selected to form a customized fragment library for subsequent molecular generation.

#### Step 3: AI-driven molecular generation

For fragment-based molecular assembly, a Twin Delayed Deep Deterministic Policy Gradient (TD3) model was used. The model integrates three core modules:

1) Actor module: Comprising the f1 network (predicts the most probable reaction template) and the f2 network (generates the embedding of the second reactant).

2) Critic module (Q-network): Estimates the expected cumulative reward for state-action pairs.

3) Environment module: Performs fragment coupling based on the selected template, conducts molecular docking (AutoDock Vina), and returns the docking score as a reward.

The generation process terminated if either the molecular weight exceeded 500 Da or the number of synthesis steps surpassed three.

#### Step 4: Verification and filtering

Generated molecules were subjected to physicochemical property checks, including compliance with Lipinski’s Rule of Five and a limit of no more than five rings, to ensure drug-likeness.

#### Step 5: Evaluation and selection

Candidate molecules were evaluated based on docking scores, key protein-ligand interactions, structural diversity, and synthetic feasibility. Based on the integrated screening criteria, 32 representative candidates (**AI10-a1** to **AI10-a32**) were taken forward for chemical synthesis and subsequent biological evaluation.

### Chemical synthesis

The general synthetic procedures A to D involved in the compound synthesis carried out in this study are described below; detailed synthetic routes are provided in the Supplementary Materials.

#### General synthetic procedure A

Amide coupling was carried out by combining the corresponding amine and carboxylic acid precursors in *N*,*N*′-dimethylformamide (DMF) in the presence of a carbodiimide-based activation system and an organic base. The reaction was allowed to proceed at ambient temperature and monitored by thin-layer chromatography. Upon completion, the mixture was worked up by aqueous dilution and organic extraction, and the resulting material was purified by thin-layer chromatography to afford the target compound.

#### General synthetic procedure B

Reductive amination was performed by allowing aldehyde and amine substrates to undergo condensation in the presence of acetic acid, followed by stepwise reduction with sodium triacetoxyborohydride. After removal of the solvent, the reaction mixture was neutralized and processed by standard extraction. The desired products were purified by column chromatography to afford the corresponding solids.

#### General synthetic procedure C

Amide formation was carried out by treating carboxylic acid derivatives with the corresponding amines in the presence of a uronium-type coupling reagent and an organic base. The reactions were conducted at ambient temperature and monitored by thin-layer chromatography. Following aqueous quenching and extraction, purification by preparative chromatography afforded the target compounds.

#### General synthetic procedure D

Acid-mediated deprotection of t-Butyloxycarbonyl (Boc)-protected intermediates was conducted under low-temperature conditions, followed by gradual warming to ambient temperature. After removal of volatile components, the resulting materials were washed and isolated to provide the corresponding deprotected products.

### TYR inhibitory activity assay

The inhibitory activity of the compounds against TYR was examined using an in vitro enzymatic assay adapted and optimized from previously reported procedures (*38*). Mushroom TYR (EC 1.14.18.1) was obtained from Sigma-Aldrich (T3824, USA), with kojic acid and β-arbutin included as reference inhibitors. l-dopa and l-tyrosine were used as substrates to evaluate diphenolase and monophenolase activities, respectively. Test compounds and positive controls were initially prepared as 100 mM stock solutions in dimethyl sulfoxide and subsequently diluted with phosphate-buffered saline (PBS; pH 6.8) to afford a series of 8 to 10 working concentrations, each 1.25-fold higher than the corresponding final assay concentrations. For the enzymatic assay, 160 μl of compound solution was combined with 20 μl of TYR solution (100 U/ml for the l-dopa assay or 300 U/ml for the l-tyrosine assay) in a 96-well plate and preincubated at 25°C for 10 min. The reaction was initiated by the addition of 20 μl of substrate solution (0.85 mM l-dopa or 1 mM l-tyrosine) and allowed to proceed at 25°C for 20 min. Absorbance was then recorded immediately at 475 nm using a microplate reader. Control wells contained enzyme and substrate in the absence of inhibitors, while blank wells contained PBS instead of the enzyme solution. Inhibition percentages were calculated accordingly, and IC_50_ values were obtained by fitting the concentration-response curves using GraphPad Prism

$$\text{Inhibition}\left(\%\right)=\frac{OD_{\text{Control}}-OD_{\text{Sample}}}{OD_{\text{Control}}-OD_{\text{Blank}}}\times 100\%$$

### Molecular docking simulation

To provide more detailed interaction information, molecular docking was performed using Discovery Studio 2023 to evaluate the interactions between TYR (PDB ID: 2Y9X) and the potent TYR inhibitors. The crystal structure was retrieved from the PDB and preprocessed by removing water molecules, cocrystallized ligands, and nonessential ions, followed by the addition of hydrogens, optimization of protonation states, and energy minimization. The binding pocket was defined based on the coordinates of the endogenous ligand within the crystal structure. Ligands were prepared by structural conversion, force field assignment, conformational sampling, and geometry optimization. Docking was carried out with the CDOCKER module, which uses the CHARMm force field combined with grid-based docking and molecular dynamics simulated annealing to predict binding modes and affinities.

### Cell culture

Human melanoma A375 cells, mouse melanoma B16F10 cells, and human keratinocyte HaCaT cells were cultured in high-glucose Dulbecco’s modified Eagle’s medium supplemented with 10% fetal bovine serum and 1% penicillin-streptomycin (Gibco, New York, USA). HEMs were maintained in a commercially available melanocyte growth medium with the corresponding supplements, following the manufacturer’s instructions. All cell lines were incubated at 37°C in a humidified atmosphere containing 5% CO_2_ and were routinely confirmed to be free of mycoplasma contamination.

### Cytotoxicity assay

Cellular viability following compound treatment was evaluated in human melanoma A375 cells (RRID:CVCL 0132), mouse melanoma B16F10 cells (RRID:CVCL 0159), HEM (cell from the beauty industry, not registered in RRID), and human keratinocyte HaCaT cells (RRID:CVCL 0038). A375 and B16F10 cell lines were sourced from Hangzhou Jesimo Biotechnology Co., Ltd., while HEM and HaCaT cells were obtained from Qingqi (Shanghai) Biotechnology Development Co., Ltd. Kojic acid and β-arbutin were used as reference controls. Cells were plated in 96-well formats and allowed to establish prior to exposure to a concentration gradient of the test compounds. Vehicle-treated cells served as controls. After prolonged incubation, cell viability was quantified using a cell counting kit-8 (CCK-8) assay according to the manufacturer’s instructions, and absorbance was measured at 450 nm using a microplate reader.

### Prediction of skin permeability

Skin permeability is a fundamental parameter for evaluating the efficacy of topical drugs, cosmetic formulations, and transdermal delivery systems, as it determines whether active compounds can efficiently penetrate the skin barrier to exert their intended effects. The key descriptor, log *K*_p_ (logarithm of the skin permeability coefficient, in centimeter per hour), quantitatively reflects transdermal absorption capacity. A log *K*_p_ value between −3 and −1 cm/hour is generally considered optimal, with higher values indicating stronger permeability; thus, it serves as a decisive metric for formulation optimization and bioavailability prediction. In this study, skin permeability indices were estimated using the pkCSM pharmacokinetic prediction platform, developed by the bioinformatics group at the University of Queensland. This platform leverages graph-based signatures to enable rapid and reliable prediction of diverse ADMET-related parameters, including skin permeability.

### Intracellular melanogenesis inhibition assay

The effects of selected compounds (**AI10-m15**, **AI10-m52**, and **AI10-a2**) on cellular melanin production were examined in B16F10 and A375 cells, with kojic acid and β-arbutin included as reference inhibitors. Cells were seeded in multiwell plates and allowed to stabilize prior to stimulation with MT-II, followed by treatment with a concentration range of the test compounds. After extended incubation, intracellular melanin levels were determined by spectrophotometric analysis at 405 nm following cell lysis. Inhibition rates were calculated relative to stimulation-only controls. For visual assessment of melanin suppression, B16F10 cells were cultured under identical stimulation and treatment conditions in larger well formats. After treatment, representative images of intact cultures were recorded. Cells were subsequently collected and processed under alkaline conditions to solubilize melanin, and additional images were acquired to document changes in pigmentation.

### In vivo melanogenesis inhibition assay in zebrafish

Zebrafish embryos (wild-type AB strain, 6 hpf) (*n* = 8) were used to evaluate the in vivo antimelanogenic effects of candidate compounds **AI10-m15**, **AI10-m52**, and **AI10-a2**. The breeding and maintenance of adult zebrafish complied with international Association for Assessment and Accreditation of Laboratory Animal Care (AAALAC) accreditation standards (no. 001458). The ethical approval number for this study is SHZ24110398. Kojic acid was included as a positive control to validate the sensitivity and reliability of the experimental system, while untreated embryos served as the baseline control. Embryos were randomly allocated to treatment groups and placed in six-well plates and then exposed to two concentrations (30 and 60 μM) of each candidate compound or kojic acid under dark conditions for 45 hours. After treatment, eight embryos per group were randomly selected for imaging of the head region. Melanin signal intensity was quantified using specialized image analysis software, and the melanin inhibition rate was calculated according to the following formula

$$\text{Antipigmentation effect}\left(\%\right)=\frac{S_{\text{Control}}-S_{\text{Sample}}}{S_{\text{Control}}}\times 100\%$$

Data were analyzed using Student’s *t* test, with *P* < 0.05 considered statistically significant. This approach allowed a comprehensive assessment of the relative efficacy of each candidate compound in suppressing melanin synthesis at the organismal level.
