# Supplementary material for: Discovery of TYR inhibitors from de novo molecular generation to dual-track lead optimization: “Competition” between AI and chemists
Source: Sci Adv. 2026 Jun 19;12(25):eaeg0376. doi: 10.1126/sciadv.aeg0376 (PMC13281790; doi:10.1126/sciadv.aeg0376)
Supplement: Supplementary file 1 — Figs. S1 to S3 Tables S1 and S2 Supplementary Text [file sciadv.aeg0376_sm.pdf]

Science Advances
AAAS

Supplementary Materials for

Discovery of TYR inhibitors from de novo molecular generation to dual-track lead optimization: "Competition" between AI and chemists

Yinyan Sun *et al.*

Corresponding author: Bin Ju, jubin_hz@163.com; Jianan Guo, jiananguo_@outlook.com; Renren Bai, renrenbai@hznu.edu.cn, renrenbai@126.com

Sci. Adv. **12**, eaeg0376 (2026)
DOI: 10.1126/sciadv.aeg0376

This PDF file includes:

Figs. S1 to S3
Tables S1 and S2
Supplementary Text

A

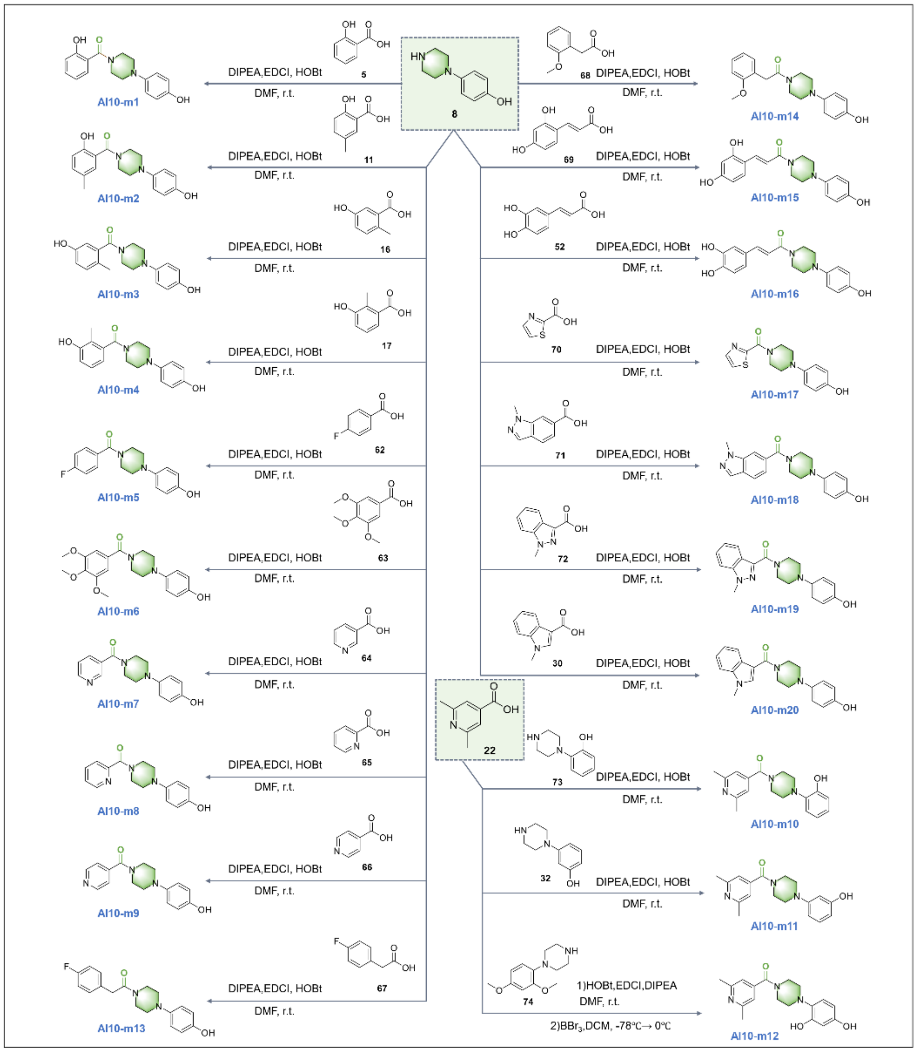

B

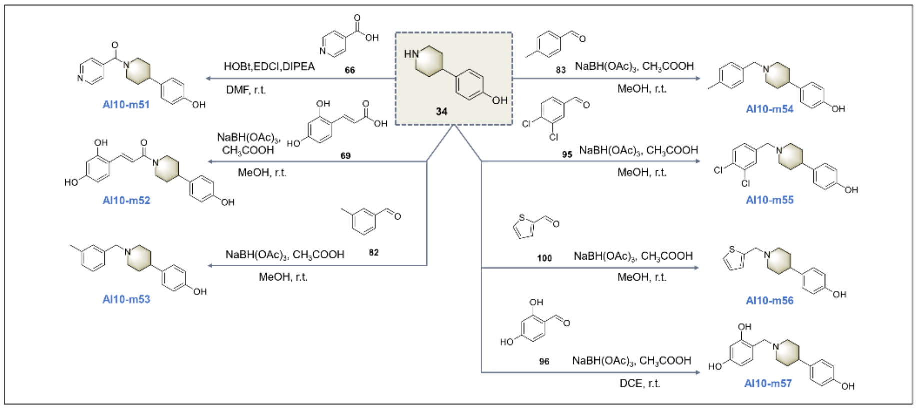

Fig. S1. Representative synthetic routes of conventionally structure-modified compounds. A) Synthesis of compounds AI10-m1 to AI10-m20; B) Synthesis of compounds AI10-m51 to AI10-m56.

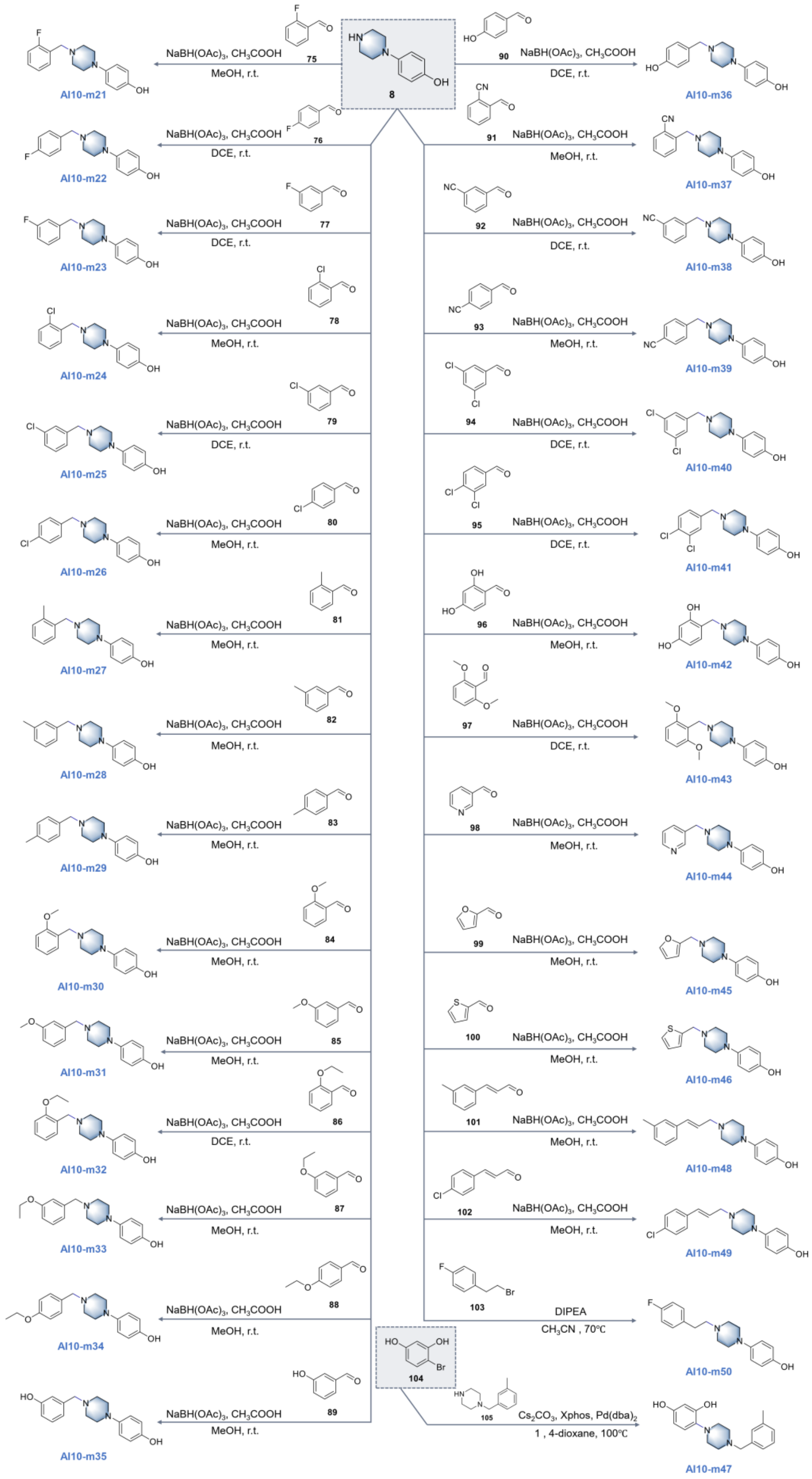

Fig. S2. Synthetic routes of compounds **AI10-m21** to **AI10-m50**.

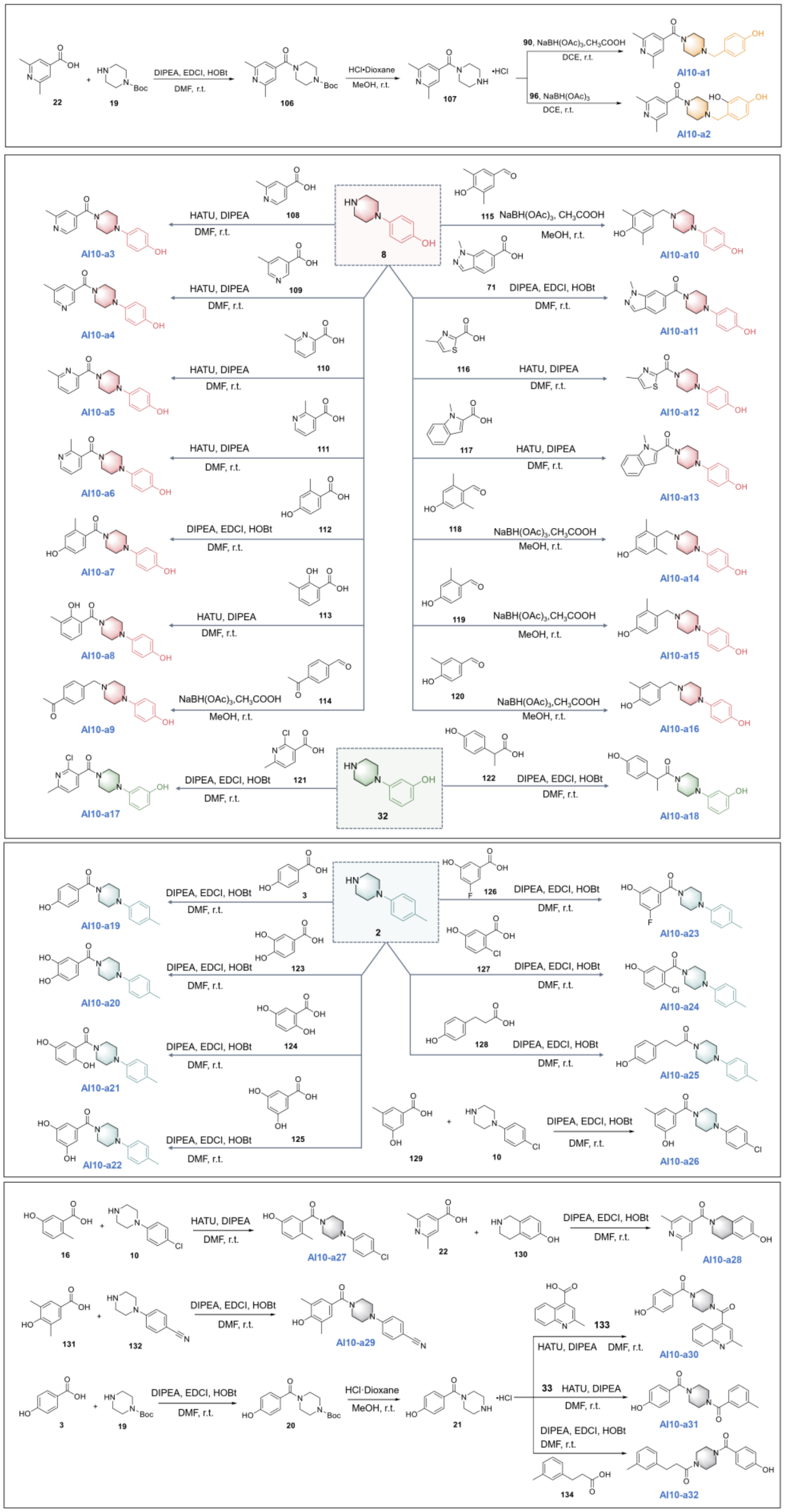

Fig. S3. Synthetic routes of the RL-generated compounds **AI10-a1** to **AI10-a32.**

Table S1. Cytotoxicity evaluation of compound AI10 and reference drugs

Compound	The maximum non-toxic concentration (MNC, μM) [a]			
	A375	B16F10	HEM	HaCaT
**AI10**	50	50	50	50
**kojic acid**	200	200	> 200	> 200
**β-arbutin**	200	200	200	200

[a] Cell viability ≥ 85%.

Table S2. Prediction of skin permeability of the representative compounds

Compound	Structure	MW	iLog P [a]	Skin permeability (Log Kp, cm/h) [b]
**AI10**	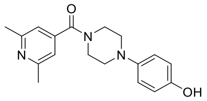	311	2.44	-3.371
**AI10-m15**	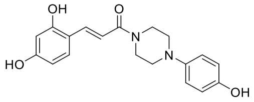	340	2.14	-2.622
**AI10-m52**	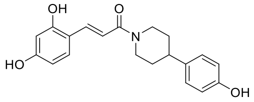	339	2.54	-2.775
**AI10-a2**	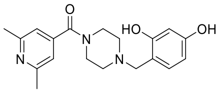	341	2.40	-2.846

[a] iLog P: https://www.swissadme.ch/; [a] pkCSM: https://biosig.lab.uq.edu.au/pkcsm/; good skin permeability (cm/h): $-3 < \text{Log } K_p < -1$; poor skin permeability: Log $K_p \geqslant -1$ or Log $K_p \leqslant -3$.

Supplementary Text

Reagents and Instrumentation

All chemicals were procured from Bidepharm, Aladdin or Sigma Aldrich and were utilized without any subsequent purification. For both reaction and column chromatography (CC), solvents of analytical grade (Shanghai Chemical Reagents Co., Ltd., China) were employed, while solvents for HPLC were of HPLC grade (J & K Scientific Ltd.). Silica gel (100-200, 200-300 mesh, Qingdao Haiyang Chemical Co., Ltd., China) was utilized for CC. For thin-layer chromatography (TLC), precoated silica gel GF254 plates (Qingdao Haiyang Chemical Co., Ltd., China) were employed. The progress of the reaction was monitored using TLC, and visualization was achieved with UV light or iodine staining. Melting points were determined with an X-4BII+ microscope melting point apparatus. ^{1}H NMR and ^{13}C NMR spectra were recorded on Bruker instruments (500 MHz and 125 MHz, respectively), with tetramethylsilane used as the internal standard. High-resolution mass spectra were obtained using an Agilent 1290LC-6530QTOF instrument, employing electrospray ionization as the ion source. The purity of all compounds was evaluated through NMR spectroscopy and HPLC analysis. All compounds showed ≥ 98% purity by HPLC (SHIMADZU Labsolutions, UV detection at λ = 254 nm), utilizing an Agilent C18 column (4.6 × 250 mm, 5 μm) and eluting at a flow rate of 0.5 mL/min with methanol as mobile phase.

Synthetic strategy for the traget compounds

General synthetic procedure for compounds **AI1-3**, **AI6**, **AI8**, **AI11**, **AI14**, **AI16**, **AI18-21**, **AI26-28**, **AI10-m1** to **AI10-m11**, **AI10-m12** to **AI10-m20**, **AI10-m51**, **AI10-a11**, **AI10-a17** to **AI10-a26**, **AI10-a28**, **AI10-a29**: Amine derivatives **2**, **4**, **6**, **8**, **10**, **12**, **18**, **22, 32**, **34**, **40**, **43**, **44**, **46**, **58**, **60**, **130**, **132** were reacted with carboxylic acid derivatives **1**, **3**, **5**, **11**, **16**, **17**, **22**, **23**, **30**, **32**, **33**, **41**, **42**, **45**, **52**, **59**, **61-74**, **121-129**, **131** under reaction ***procedure A***, affording the corresponding final products. Compound **AI10-m12** was further subjected to reaction BBr_3 to afford the final product.

General synthetic procedure for compounds **AI10-m21** to **AI10-m46**, **AI10-m48**, **AI10-m49**, **AI10-m52** to **AI10-m57**, **AI10-a9**, **AI10-a10**, **AI10-a14** to **AI10-a16**: Using amine compounds **8** and **34** together with aldehyde compounds **69**, **75-102**, **114**, **115**, and **118-120** as starting materials, the corresponding final products were obtained through reaction ***procedure B***.

General synthetic procedure for compounds **AI4**, **AI5**, **AI10**, **AI15**, **AI24**, **AI10-a3** to **AI10-a8**, **AI10-a12**, **AI10-a13**, **AI10-a27**: Using amine compounds **8**, **10**, **32**, and **50** together with carboxylic acid compounds **7**, **9**, **16**, **22**, **31**, **53**, **108-113**, **116**, and **117** as starting materials, the corresponding final products were obtained through reaction ***procedure C***.

Synthetic procedure for compounds **AI7**: In a 50 mL round-bottom flask, **8** (3.0 mmol), triethylamine (6.0 mmol), and DCM (5 mL) were added sequentially. Under ice-bath conditions, Boc_2O (3.9 mmol) was added dropwise, and the reaction mixture was stirred at room temperature. The progress was monitored by TLC. Upon completion, the crude mixture was purified by column chromatography (DCM/MeOH = 100:1-60:1, v/v) to yield intermediate **13**. Intermediate **13** (0.72 mmol), KI (0.86 mmol), K_2CO_3 (1.44 mmol), and DMF (5 mL) were combined and reacted at room temperature. The progress was monitored by TLC. After completion, the reaction mixture was diluted with H_2O and extracted with ethyl acetate (3 × 20 mL). The combined organic phases were washed with saturated brine, dried over anhydrous Na_2SO_4, and concentrated under reduced pressure. The residue was purified by column chromatography (DCM/MeOH = 100:1-80:1, v/v) to afford intermediate **14**. The intermediate was subjected to reaction ***procedure D*** to yield the deprotected product **15**. Finally, intermediate **15** (0.67 mmol) and compound **16** (0.56 mmol) were subjected to reaction ***procedure A*** to afford the target compound **AI7**.

Synthetic procedure for compounds **AI9**: Compound **19** and **3** were used as starting materials and subjected sequentially to reaction **procedures A** and **D** to afford intermediate **21**. Finally, intermediate **21** was coupled with 2-fluoro-4-methylbenzoic acid under reaction ***procedure A*** to furnish the final product **AI9**.

Synthetic procedure for compounds **AI12**: Compound **19** (2.4 mmol), **24** (2.0 mmol), K_2CO_3 (4.0 mmol), and DMF (6 mL) were combined in a 50 mL round-bottom flask and stirred at room temperature. Reaction progress was monitored by TLC. Upon completion, the mixture was diluted with H_2O and extracted with ethyl acetate (3 ×). The combined organic layers were washed with saturated brine, dried over anhydrous Na_2SO_4, and concentrated under reduced pressure. The crude product was purified by column chromatography (DCM/MeOH = 100:1-80:1, v/v) to afford intermediate **25**. Subsequently, intermediate **25** and compound **27** were subjected to reaction ***procedures D*** and ***A***, respectively, to furnish the final product **AI12**.

Synthetic procedure for compounds **AI13**: Compound **28** and **8** were used as starting materials and subjected to reaction ***procedure A*** to afford intermediate **29**. Subsequently, intermediate **29**

(0.5 mmol) was dissolved in a 50 mL round-bottom flask under ice-bath cooling. A solution of $LiAlH_4$ (1.5 mmol) in THF (5 mL) was added dropwise, and the reaction mixture was then heated to 70 °C. Progress was monitored by TLC. Upon completion, the reaction was quenched under ice-bath conditions with NaOH solution, and the pH was adjusted to 6-7 using 1 M HCl. The precipitate was collected by filtration, dissolved, and purified by column chromatography (DCM/MeOH = 80:1-40:1, v/v) to afford the target compound **AI13** as a yellow solid.

Synthetic procedure for compounds **AI17**: Compound **35** (3.0 mmol), triethylamine (6.0 mmol), and DCM (5 mL) were combined in a 50 mL round-bottom flask. Boc_2O (4.5 mmol) was added dropwise under ice-bath conditions, and the reaction mixture was then allowed to warm to room temperature. Progress was monitored by TLC. Upon completion, the organic layer was washed with water (3 ×), followed by saturated brine, dried over anhydrous Na_2SO_4, and concentrated under reduced pressure. The crude product was purified by column chromatography (DCM/MeOH = 80:1-60:1, v/v) to yield intermediate **36**. Subsequently, intermediates **36** and **37** were subjected to reaction ***procedures A*** and ***D***, respectively, to afford intermediate **39**. Finally, intermediate **39** was coupled with compound **40** under reaction ***procedure A*** to furnish the final product **AI17**.

Synthetic procedure for compounds **AI22**: Intermediate **26** (1.0 mmol), compound **47** (1.2 mmol), K_2CO_3 (2.0 mmol), and DMF (5 mL) were sequentially added to a 50 mL round-bottom flask and stirred at 60 °C. The reaction progress was monitored by TLC. Upon completion, the mixture was diluted with H_2O and extracted with EA (3 ×). The combined organic layers were washed with saturated brine, dried over anhydrous Na_2SO_4, and concentrated under reduced pressure. The crude residue was purified by column chromatography (DCM/MeOH = 100:1-60:1, v/v) to afford intermediate **48**.

Subsequently, intermediate **48** (0.14 mmol) was dissolved in MeOH (3 mL) in a 50 mL round-bottom flask. NaOH was added under ice-bath conditions, and the reaction mixture was then warmed to room temperature. Reaction progress was monitored by TLC. After completion, the reaction was quenched with 1 M HCl to adjust the pH to 6-7, followed by extraction with DCM (3 ×). The combined organic extracts were washed with saturated brine, dried over anhydrous Na_2SO_4, and concentrated under reduced pressure. The product was purified by TLC (DCM/MeOH = 50:1, v/v) to give compound **57** as a white solid. Finally, compound **57** was reacted with compound **58** under reaction ***procedure A*** to furnish the target product **AI22**.

Synthetic procedure for compounds **AI23**: A 50 mL round-bottom flask was charged sequentially with 4-fluoroaniline (**51**, 0.5 mmol), 3,4-dihydroxycinnamic acid (**52**, 0.6 mmol), *N,N'*-dicyclohexylcarbodiimide (DCC, 0.65 mmol), and THF (5 mL). The mixture was refluxed at 60 °C, and the progress of the reaction was monitored by TLC. Upon completion, the reaction mixture was diluted with H_2O and extracted with EA (3 ×). The combined organic phases were washed with saturated brine, dried over anhydrous Na_2SO_4, and concentrated under reduced pressure to remove the solvent. The crude product was purified by column chromatography (DCM/MeOH = 80:1-20:1, v/v), followed by TLC purification (DCM/MeOH = 60:1, v/v), to afford the target compound **AI23** as a yellow solid.

Synthetic procedure for compounds **AI25**: Compounds **54** and **55** were first subjected to reaction ***procedure B*** to afford the intermediate **56**. Subsequently, intermediate **56** (0.55 mmol) was dissolved in methanol (5 mL), followed by the addition of 10% aqueous NaOH solution (3.75 mmol). The mixture was refluxed at 65 °C, and the progress of the reaction was monitored by TLC. Upon completion, the solvent was removed under reduced pressure, and the residue was adjusted to pH 5-6 with 20% aqueous HCl under an ice bath. The resulting precipitate was collected by filtration to give the target compound **57** as a white solid. Finally, compound **57** and compound **58** were subjected to reaction ***procedure A*** to yield the final product **AI25**.

Synthetic procedure for compounds **AI10-m47**: A 100 mL round-bottom flask was charged sequentially with compound **104** (0.5 mmol), **105** (0.6 mmol), $CsCO_3$ (0.75 mmol), XPhos (0.1 mmol), $Pd(dba)_2$ (0.05 mmol), and 1,4-dioxane (4 mL). The reaction mixture was stirred at 100 °C and monitored by TLC. Upon completion, the mixture was cooled, diluted with water, and filtered under reduced pressure. The residue was washed with EA, while the filtrate was extracted with EA (3 × 20 mL). The combined organic extracts were washed with saturated brine (2-3 times), dried over anhydrous Na_2SO_4, and concentrated under reduced pressure. The crude product was purified by column chromatography (DCM/MeOH = 120:1-50:1, v/v) to afford a brown oily intermediate. The crude product was purified by thin-layer chromatography (DCM/MeOH = 30:1, v/v) to yield **AI10-m47** as a colorless oil.

Synthetic procedure for compounds **AI10-m50**: A 50 mL round-bottom flask equipped with a magnetic stir bar was charged sequentially with compound **103** (0.5 mmol), compound **8** (0.55 mmol), *N,N*-diisopropylethylamine (DIPEA, 0.75 mmol), and acetonitrile (5 mL). The reaction mixture was stirred at room temperature, and its progress was monitored by TLC. Upon

completion, the solvent was removed under reduced pressure, and the residue was diluted with water and extracted with dichloromethane (3 × 20 mL). The combined organic layers were washed with saturated brine (2-3 times), dried over anhydrous Na_2SO_4, and concentrated under reduced pressure. The crude product was purified by column chromatography (DCM/MeOH = 70:1, v/v) to afford the target compound **AI10-m50** as a pale-yellow solid.

Synthetic procedure for compounds **AI10-a1, AI10-a2**: Starting from compounds **22** and **19**, the reaction was performed according to general ***procedure A***, affording the key intermediate **106**. Subsequent deprotection of **106** *via* general *procedure D* furnished compound **107**. Finally, compound **107** was subjected to general *procedure B* with compounds **90** and **96**, respectively, to yield the target products **AI10-a1** and **AI10-a2**.

Synthetic procedure for compounds **AI10-a30 to AI10-a32**: Starting from compounds **3** and **19**, the reaction was carried out according to general ***procedure A*** to afford the key intermediate **20**. Subsequent deprotection of **20** *via* general ***procedure D*** furnished compound **21**. Intermediate **21** was then subjected to general ***procedure C*** with compounds **133** and **33**, respectively, to yield the final products **AI10-a30** and **AI10-a31**. In addition, treatment of **21** with compound **134** under general ***procedure A*** afforded the final product **AI10-a32**.

NMR, HRMS and HPLC data for target compounds

(2-hydroxyphenyl)(4-(p-tolyl)piperazin-1-yl)methanone (**AI1**). White solid, yield 14%, m.p. 217.7-218.3 °C. ^{1}H NMR (500 MHz, DMSO-d_6) δ 9.80 (s, 1H), 7.23 (ddd, J = 8.5, 7.4, 1.8 Hz, 1H), 7.14 (dd, J = 7.5, 1.8 Hz, 1H), 7.03 (d, J = 8.2 Hz, 2H), 6.92 – 6.82 (m, 4H), 3.73 (s, 4H), 3.07 (s, 4H), 2.20 (s, 3H). ^{13}C NMR (125 MHz, DMSO-d_6) δ 167.0, 153.3, 148.8, 130.2, 129.4, 128.2, 128.1, 123.8, 119.1, 116.2, 115.6, 49.1, 20.1. ESI-HRMS: m/z calcd for $C_{18}H_{21}N_2O_2$ $[M+H]^+$: 297.1598; found: 297.1602. HPLC analysis: retention time = 6.795 min; peak area, 98.78%.

(4-hydroxyphenyl)(4-(o-tolyl)piperazin-1-yl)methanone (**AI2**). White solid, yield 72%, m.p. 198.0-198.6 °C. ^{1}H NMR (500 MHz, DMSO-d_6) δ 9.85 (s, 1H), 7.34 – 7.28 (m, 2H), 7.19 – 7.12 (m, 2H), 7.02 (dd, J = 8.1, 1.3 Hz, 1H), 6.97 (td, J = 7.4, 1.3 Hz, 1H), 6.83 – 6.79 (m, 2H), 3.64 (s, 4H), 2.83 (d, J = 4.9 Hz, 4H), 2.27 (s, 3H). ^{13}C NMR (125 MHz, DMSO-d_6) δ 169.3, 158.7, 150.9, 131.9, 130.8, 129.3, 126.6, 126.1, 123.2, 119.1, 114.9, 51.6, 17.5. ESI-HRMS: m/z calcd for

$C_{18}H_{21}N_2O_2$ [M+H]$^+$: 297.1598; found: 297.1602. HPLC analysis: retention time = 6.816 min; peak area, 97.96%.

(2-hydroxyphenyl)(4-(m-tolyl)piperazin-1-yl)methanone (**AI3**). Light yellow solid, yield 19%, m.p. 167.1-167.6 °C. ^{1}H NMR (500 MHz, DMSO-d_6) δ 9.81 (s, 1H), 7.26 – 7.20 (m, 1H), 7.15 (qd, *J* = 8.3, 7.5, 3.8 Hz, 3H), 7.02 – 6.94 (m, 2H), 6.90 – 6.82 (m, 2H), 3.33 (s, 4H), 2.81 (s, 4H), 2.26 (s, 3H). ^{13}C NMR (125 MHz, DMSO-d_6) δ 167.2, 153.2, 150.9, 131.9, 130.8, 130.1, 128.1, 126.6, 123.9, 123.2, 119.1, 119.0, 115.6, 51.5, 17.6. ESI-HRMS: m/z calcd for $C_{18}H_{21}N_2O_2$ [M+H]$^+$: 297.1598; found: 297.1600. HPLC analysis: retention time = 7.840 min; peak area, 99.77%.

(2-ethylphenyl)(4-(4-hydroxyphenyl)piperazin-1-yl)methanone (**AI4**). White solid, yield 96%, m.p. 109.5-110.3 °C. ^{1}H NMR (500 MHz, DMSO-d_6) δ 8.89 (s, 1H), 7.38 – 7.30 (m, 2H), 7.25 (td, *J* = 7.3, 1.7 Hz, 1H), 7.17 (dd, *J* = 7.5, 1.4 Hz, 1H), 6.83 – 6.78 (m, 2H), 6.69 – 6.63 (m, 2H), 3.78 (q, *J* = 5.0 Hz, 2H), 3.24 (dt, *J* = 14.4, 5.5 Hz, 2H), 3.01 (dt, *J* = 10.9, 5.2 Hz, 2H), 2.93 – 2.76 (m, 2H), 2.66 – 2.51 (m, 2H), 1.15 (t, *J* = 7.6 Hz, 3H). ^{13}C NMR (125 MHz, DMSO-d_6) δ 168.5, 151.5, 143.8, 140.1, 135.7, 128.9, 128.8, 125.8, 125.8, 118.6, 115.5, 50.6, 50.4, 46.6, 41.0, 25.5, 15.4. ESI-HRMS: m/z calcd for $C_{19}H_{23}N_2O_2$ [M+H]$^+$: 311.1754; found: 311.1753. HPLC analysis: retention time = 6.492 min; peak area, 99.11%.

(4-(4-chlorophenyl)piperazin-1-yl)(2-hydroxy-6-methylphenyl)methanone (**AI5**). Light yellow solid, yield 26%, m.p. 240.2-240.8 °C. ^{1}H NMR (500 MHz, DMSO-d_6) δ 9.60 (s, 1H), 7.24 (d, *J* = 9.0 Hz, 2H), 7.08 (t, *J* = 7.8 Hz, 1H), 6.96 (d, *J* = 9.0 Hz, 2H), 6.69 (dd, *J* = 8.0, 2.5 Hz, 2H), 3.85 (ddd, *J* = 13.0, 6.5, 3.5 Hz, 1H), 3.68 (ddd, *J* = 12.9, 7.5, 3.4 Hz, 1H), 3.26 (t, *J* = 5.2 Hz, 3H), 3.15 (ddt, *J* = 20.2, 12.2, 4.4 Hz, 2H), 2.98 (dd, *J* = 11.7, 5.7 Hz, 1H), 2.13 (s, 3H). ^{13}C NMR (125 MHz, DMSO-d_6) δ 166.4, 153.1, 149.6, 135.2, 129.1, 128.7, 123.9, 122.9, 120.6, 117.4, 112.7, 48.7, 48.3, 45.3, 18.7. ESI-HRMS: m/z calcd for $C_{18}H_{20}ClN_2O_2$ [M+H]$^+$: 331.1208; found: 331.1206. HPLC analysis: retention time = 7.841 min; peak area, 99.81%.

(4-(2-chlorophenyl)piperazin-1-yl)(2-hydroxy-5-methylphenyl)methanone (**AI6**). White solid, yield 75%, m.p. 180.4-181.1 °C. ^{1}H NMR (500 MHz, DMSO-d_6) δ 9.56 (s, 1H), 7.42 (dd, *J* = 7.9, 1.5 Hz, 1H), 7.30 (td, *J* = 7.7, 1.5 Hz, 1H), 7.15 (dd, *J* = 8.1, 1.5 Hz, 1H), 7.08 – 7.01 (m, 2H), 6.95 (d, *J* = 2.2 Hz, 1H), 6.77 (d, *J* = 8.2 Hz, 1H), 3.75 (s, 2H), 3.37 (s, 2H), 2.96 (s, 4H), 2.21 (s, 3H). ^{13}C NMR (125 MHz, DMSO-d_6) δ 167.4, 151.0, 148.7, 130.7, 130.4, 128.4, 128.2,

127.8, 124.4, 123.6, 121.2, 115.7, 51.1, 20.0. ESI-HRMS: m/z calcd for $C_{18}H_{20}ClN_2O_2$ [M+H]$^+$: 331.1208; found: 331.1206. HPLC analysis: retention time = 8.187 min; peak area, 98.96%.

(5-hydroxy-2-methylphenyl)(4-(4-methoxyphenyl)piperazin-1-yl)methanone (**AI7**). White solid, yield 70%, m.p. 162.5-162.9 °C. ^{1}H NMR (500 MHz, DMSO-d_6) δ 9.43 (s, 1H), 7.05 (d, J = 8.3 Hz, 1H), 6.93 – 6.88 (m, 2H), 6.85 – 6.80 (m, 2H), 6.71 (dd, J = 8.3, 2.6 Hz, 1H), 6.55 (d, J = 2.6 Hz, 1H), 3.76 (t, J = 5.2 Hz, 2H), 3.68 (s, 3H), 3.27 (t, J = 5.1 Hz, 2H), 3.05 (t, J = 5.2 Hz, 2H), 2.90 (d, J = 5.2 Hz, 2H), 2.10 (s, 3H). ^{13}C NMR (125 MHz, DMSO-d_6) δ 168.4, 155.2, 153.4, 145.1, 137.0, 131.2, 123.4, 118.2, 115.7, 114.3, 112.3, 55.2, 50.3, 50.1, 46.2, 40.9, 17.6. ESI-HRMS: m/z calcd for $C_{19}H_{23}N_2O_3$ [M+H]$^+$: 331.1208; found: 331.1206. HPLC analysis: retention time = 7.484 min; peak area, 99.70%.

(3-hydroxy-2-methylphenyl)(4-phenethylpiperazin-1-yl)methanone (**AI8**). White solid, yield 49%, m.p. 130.6-131.2 °C. ^{1}H NMR (500 MHz, DMSO-d_6) δ 9.55 (s, 1H), 7.29 – 7.24 (m, 2H), 7.23 – 7.20 (m, 2H), 7.17 (td, J = 7.0, 1.5 Hz, 1H), 7.03 (t, J = 7.8 Hz, 1H), 6.80 (dd, J = 8.1, 1.2 Hz, 1H), 6.56 (dd, J = 7.5, 1.2 Hz, 1H), 3.63 (d, J = 18.1 Hz, 2H), 3.11 (t, J = 5.1 Hz, 2H), 2.72 (dd, J = 9.3, 6.3 Hz, 2H), 2.54 (d, J = 8.6 Hz, 2H), 2.48 – 2.28 (m, 4H), 1.99 (s, 3H). ^{13}C NMR (125 MHz, DMSO-d_6) δ 168.4, 155.6, 140.3, 137.8, 128.6, 128.2, 126.6, 125.8, 120.0, 116.1, 114.6, 59.5, 52.9, 52.4, 46.2, 40.8, 32.6, 12.4. ESI-HRMS: m/z calcd for $C_{20}H_{25}N_2O_2$ [M+H]$^+$: 325.1911; found: 325.1910. HPLC analysis: retention time = 10.284 min; peak area, 99.52%.

(4-(2-fluoro-4-methylbenzoyl)piperazin-1-yl)(4-hydroxyphenyl)methanone (**AI9**). White solid, yield 53%, m.p. 191.0-191.5 °C. ^{1}H NMR (500 MHz, DMSO-d_6) δ 9.87 (s, 1H), 7.30 (t, J = 8.2 Hz, 3H), 7.16 – 7.07 (m, 2H), 6.79 (d, J = 8.6 Hz, 2H), 3.68 (s, 2H), 3.51 (d, J = 44.2 Hz, 4H), 3.28 (s, 2H), 2.34 (s, 3H). ^{13}C NMR (125 MHz, DMSO- d_6) δ 169.5, 164.4, 158.9, 158.5 (d, ^1J = 243.75 Hz), 142.1 (d, ^3J = 7.5 Hz), 129.4, 128.7 (d, ^3J = 5 Hz), 125.7, 125.5 (d, ^4J = 3.75 Hz), 120.9 (d, ^2J = 17.5 Hz), 116.2 (d, ^2J = 21.25 Hz), 114.9, 46.5, 41.5, 20.7. ESI-HRMS: m/z calcd for $C_{19}H_{20}FN_2O_3$ [M+H]$^+$: 343.1452; found: 343.1444. HPLC analysis: retention time = 10.281 min; peak area, 100.0%.

(2,6-dimethylpyridin-4-yl)(4-(4-hydroxyphenyl)piperazin-1-yl)methanone (**AI10**). White solid, yield 24%, m.p. 240.1-240.5 °C. ^{1}H NMR (500 MHz, DMSO-d_6) δ 8.89 (s, 1H), 7.05 (s, 2H), 6.80 (d, J = 8.9 Hz, 2H), 6.66 (d, J = 8.9 Hz, 2H), 3.73 (s, 2H), 3.38 (s, 2H), 3.01 (s, 2H), 2.90 (s, 2H), 2.45 (s, 6H). ^{13}C NMR (125 MHz, DMSO-d_6) δ 167.2, 157.9, 151.5, 144.2, 143.8, 118.6,

117.4, 115.5, 54.9, 50.6, 50.3, 46.9, 41.5, 24.0. ESI-HRMS: m/z calcd for $C_{18}H_{22}N_3O_2$ $[M+H]^+$: 312.1707; found: 312.1708. HPLC analysis: retention time = 6.289 min; peak area, 99.24%.

(4-hydroxypyridin-2-yl)(4-(o-tolyl)piperazin-1-yl)methanone (**AI11**). Light yellow solid, yield 61%, m.p. 199.6-200.2 °C. ^{1}H NMR (500 MHz, DMSO-d_6) δ 10.95 (s, 1H), 8.25 (s, 1H), 7.15 (ddd, *J* = 17.4, 7.5, 1.6 Hz, 2H), 7.04 – 7.00 (m, 1H), 6.98 (dd, *J* = 7.3, 1.2 Hz, 1H), 6.96 – 6.63 (m, 2H), 3.77 (s, 2H), 3.54 (s, 2H), 2.84 (dt, *J* = 40.3, 4.8 Hz, 4H), 2.27 (s, 3H). ^{13}C NMR (125 MHz, DMSO-d_6) δ 150.8, 132.0, 130.8, 126.6, 123.2, 119.1, 51.7, 51.3, 47.1, 42.0, 17.5. ESI-HRMS: m/z calcd for $C_{17}H_{20}N_3O_2$ $[M+H]^+$: 298.1550; found: 298.1554. HPLC analysis: retention time = 7.699 min; peak area, 99.50%.

2-(4-hydroxyphenyl)-1-(4-(4-methylbenzyl)piperazin-1-yl)ethan-1-one (**AI12**). White solid, yield 62%, m.p. 182.6-183.5 ℃. ^{1}H NMR (400 MHz, DMSO-d_6) δ 9.26 (s, 1H), 7.13 (q, *J* = 7.9 Hz, 4H), 7.01 – 6.96 (m, 2H), 6.70 – 6.64 (m, 2H), 3.54 (s, 2H), 3.42 (dt, *J* = 6.8, 3.3 Hz, 4H), 3.38 (s, 2H), 2.27 (s, 3H), 2.22 (dt, *J* = 14.7, 4.9 Hz, 4H). ^{13}C NMR (100 MHz, DMSO-d_6) δ 169.2, 155.8, 136.1, 134.7, 129.8, 128.9, 128.8, 125.8, 115.1, 61.6, 52.7, 52.2, 45.5, 41.2, 38.8, 20.8. ESI-HRMS: m/z calcd for $C_{17}H_{20}N_3O_2$ $[M+H]^+$: 325.1911; found: 325.1909. HPLC analysis: retention time = 10.282 min; peak area, 98.98%.

2-(2-(4-(4-hydroxyphenyl)piperazin-1-yl)ethyl)phenol (**AI13**). Yellowish brown, yield 94%, m.p. 171.5-172.2 °C. ^{1}H NMR (500 MHz, DMSO-d_6) δ 10.54 (s, 1H), 8.83 (s, 1H), 7.08 – 6.97 (m, 2H), 6.81 – 6.76 (m, 2H), 6.75 (dd, *J* = 8.1, 1.2 Hz, 1H), 6.69 (dd, *J* = 7.3, 1.3 Hz, 1H), 6.67 – 6.64 (m, 2H), 2.98 (t, *J* = 4.9 Hz, 4H), 2.74 (t, *J* = 6.8 Hz, 2H), 2.63 (t, *J* = 4.9 Hz, 4H), 2.56 (t, *J* = 6.8 Hz, 2H). ^{13}C NMR (125 MHz, DMSO-d_6) δ 155.9, 151.0, 144.1, 130.5, 127.2, 126.9, 118.7, 117.8, 115.7, 115.5, 58.2, 52.8, 49.9, 28.5. ESI-HRMS: m/z calcd for $C_{17}H_{20}N_3O_2$ $[M+H]^+$: 299.1754; found: 299.1751. HPLC analysis: retention time = 9.866 min; peak area, 98.34%.

(4-(4-hydroxyphenyl)piperazin-1-yl)(1-methyl-1H-indol-3-yl)methanone (**AI14**). White solid, yield 88%, m.p. 240.0-240.6 °C. ^{1}H NMR (500 MHz, DMSO-d_6) δ 8.88 (s, 1H), 7.76 (s, 1H), 7.72 (d, *J* = 8.0 Hz, 1H), 7.50 (d, *J* = 8.2 Hz, 1H), 7.23 (ddd, *J* = 8.2, 7.0, 1.2 Hz, 1H), 7.15 (ddd, *J* = 8.0, 6.9, 1.1 Hz, 1H), 6.82 (d, *J* = 9.0 Hz, 2H), 6.67 (d, *J* = 8.9 Hz, 2H), 3.83 (s, 3H), 3.79 – 3.72 (m, 4H), 2.99 (t, *J* = 5.0 Hz, 4H). ^{13}C NMR (125 MHz, DMSO-d_6) δ 165.1, 151.3, 144.0, 136.2, 132.1, 126.3, 121.9, 120.5, 120.4, 118.4, 115.5, 110.3, 108.7, 50.7, 32.7. ESI-HRMS: m/z calcd for $C_{20}H_{22}N_3O_2$ $[M+H]^+$: 336.1707; found: 336.1708. HPLC analysis: retention time = 6.400 min; peak area, 99.00%.

(4-(3-hydroxyphenyl)piperazin-1-yl)(2-methyl-1H-benzo[d]imidazol-6-yl)methanone (**AI15**). Light yellow solid, yield 27%, m.p. 157.2-158.1 ℃. ^{1}H NMR (500 MHz, DMSO-d_6) δ 12.38 (s, 1H), 9.16 (s, 1H), 7.55 – 7.46 (m, 2H), 7.19 (dd, *J* = 8.1, 1.6 Hz, 1H), 6.99 (t, *J* = 8.1 Hz, 1H), 6.39 (dd, *J* = 8.2, 2.3 Hz, 1H), 6.32 (t, *J* = 2.3 Hz, 1H), 6.23 (dd, *J* = 7.8, 2.1 Hz, 1H), 3.62 (s, 4H), 3.11 (s, 4H), 2.50 (s, 3H). ^{13}C NMR (125 MHz, DMSO-d_6) δ 169.9, 158.1, 153.0, 152.2, 129.6, 128.6, 120.7, 107.0, 106.6, 102.9, 48.6, 14.7. ESI-HRMS: m/z calcd for $C_{19}H_{21}N_4O_2$ $[M+H]^+$: 337.1659; found: 337.1653. HPLC analysis: retention time = 10.245 min; peak area, 98.80%.

(4-(4-hydroxyphenyl)piperidin-1-yl)(m-tolyl)methanone (**AI16**). White solid, yield 34%, m.p. 199.6-200.2 ℃. ^{1}H NMR (500 MHz, DMSO-d_6) δ 9.17 (s, 1H), 7.32 (t, *J* = 7.5 Hz, 1H), 7.27 – 7.17 (m, 3H), 7.05 (d, *J* = 8.5 Hz, 2H), 6.71 – 6.66 (m, 2H), 4.60 (s, 1H), 3.65 (s, 1H), 3.09 (s, 1H), 2.80 (s, 1H), 2.67 (tt, *J* = 12.1, 3.6 Hz, 1H), 2.34 (s, 3H), 1.73 (d, *J* = 70.2 Hz, 2H), 1.52 (s, 2H). ^{13}C NMR (125 MHz, DMSO-d_6) δ 169.0, 155.7, 137.8, 136.5, 135.8, 129.8, 128.2, 127.5, 127.1, 123.7, 115.1, 47.7, 42.0, 41.0, 33.6, 33.1, 20.9. ESI-HRMS: m/z calcd for $C_{19}H_{22}NO_2$ $[M+H]^+$: 296.1645; found: 296.1645. HPLC analysis: retention time = 6.029 min; peak area, 98.80%.

1-(3-chlorobenzoyl)-N-(4-methoxybenzyl)piperidine-4-carboxamide (**AI17**). White solid, yield 69%, m.p. 115.4-116.3 °C. ^{1}H NMR (500 MHz, DMSO-d_6) δ 8.28 (t, *J* = 5.9 Hz, 1H), 7.52 (ddd, *J* = 8.1, 2.2, 1.2 Hz, 1H), 7.47 (t, *J* = 7.7 Hz, 1H), 7.43 (t, *J* = 1.8 Hz, 1H), 7.34 (dt, *J* = 7.4, 1.4 Hz, 1H), 7.15 (d, *J* = 8.6 Hz, 2H), 6.89 – 6.85 (m, 2H), 4.43 (s, 1H), 4.19 (d, *J* = 6.1 Hz, 2H), 3.72 (s, 3H), 3.53 (s, 1H), 3.06 (s, 1H), 2.82 (s, 1H), 2.48 – 2.41 (m, 1H), 1.82 – 1.47 (m, 4H). ^{13}C NMR (125 MHz, DMSO-d_6) δ 173.5, 167.3, 158.1, 138.4, 133.2, 131.5, 130.5, 129.3, 128.4, 126.5, 125.2, 113.7, 55.0, 46.6, 41.7, 41.3. ESI-HRMS: m/z calcd for $C_{21}H_{24}ClN_2O_3$ $[M+H]^+$: 387.1470; found: 387.1470. HPLC analysis: retention time = 7.504 min; peak area, 97.88%.

(3-hydroxy-4-methylphenyl)(4-phenylpiperidin-1-yl)methanone (**AI18**). White solid, yield 46%, m.p. 208.1-209.1 °C. ^{1}H NMR (500 MHz, DMSO-d_6) δ 9.54 (s, 1H), 7.33 – 7.25 (m, 4H), 7.20 (t, *J* = 7.0 Hz, 1H), 7.11 (d, *J* = 7.6 Hz, 1H), 6.82 (d, *J* = 1.6 Hz, 1H), 6.75 (dd, *J* = 7.5, 1.7 Hz, 1H), 4.58 (s, 1H), 3.77 (s, 1H), 3.08 (s, 1H), 2.80 (ddt, *J* = 12.1, 8.5, 3.7 Hz, 2H), 2.14 (s, 3H), 1.78 (s, 2H), 1.57 (d, *J* = 10.6 Hz, 2H). ^{13}C NMR (125 MHz, DMSO-d_6) δ 169.0, 155.2, 145.6, 134.8, 130.4, 128.4, 126.7, 126.2, 125.4, 117.2, 113.0, 41.8, 15.8. ESI-HRMS: m/z calcd for $C_{19}H_{22}NO_2$ $[M+H]^+$: 296.1645; found: 296.1641. HPLC analysis: retention time = 7.994 min; peak area, 99.40%.

4-chloro-N-(4-(dimethylamino)benzyl)-3-hydroxybenzamide (**AI19**). White solid, yield 38%, m.p. 165.3-166.0 °C. ^{1}H NMR (500 MHz, DMSO-d_6) δ 10.41 (s, 1H), 8.86 (t, *J* = 6.0 Hz, 1H), 7.48 – 7.38 (m, 2H), 7.29 (dt, *J* = 8.4, 1.4 Hz, 1H), 7.13 (d, *J* = 8.7 Hz, 2H), 6.72 – 6.65 (m, 2H), 4.31 (d, *J* = 5.9 Hz, 2H), 2.85 (s, 6H). ^{13}C NMR (125 MHz, DMSO-d_6) δ 165.2, 152.9, 149.6, 134.7, 129.6, 128.3, 127.1, 122.6, 118.4, 115.8, 112.4, 42.2, 40.3. ESI-HRMS: m/z calcd for $C_{16}H_{18}ClN_2O_2$ [M+H]$^+$: 305.1051; found: 305.1051. HPLC analysis: retention time = 6.626 min; peak area, 98.19%.

N-(2,5-dimethylbenzyl)-2-hydroxybenzamide (**AI20**). Light yellow solid, yield 37%, m.p. 88.8-89.3 °C. ^{1}H NMR (500 MHz, DMSO-d_6) δ 12.50 (s, 1H), 9.16 (t, *J* = 5.8 Hz, 1H), 7.93 (dd, *J* = 7.9, 1.7 Hz, 1H), 7.41 (ddd, *J* = 8.5, 7.2, 1.6 Hz, 1H), 7.10 – 7.05 (m, 2H), 6.98 (dd, *J* = 7.7, 1.8 Hz, 1H), 6.94 – 6.87 (m, 2H), 4.46 (d, *J* = 5.7 Hz, 2H), 2.25 (d, *J* = 17.7 Hz, 6H). ^{13}C NMR (125 MHz, DMSO-d_6) δ 168.8, 160.1, 136.4, 134.7, 133.8, 132.5, 130.1, 128.2, 128.1, 127.6, 118.8, 117.5, 115.4, 40.6, 20.8, 18.4. ESI-HRMS: m/z calcd for $C_{16}H_{18}NO_2$ [M+H]$^+$: 256.1332; found: 256.1334. HPLC analysis: retention time = 8.301 min; peak area, 99.43%.

N-(4-chlorobenzyl)-4-hydroxy-3-methylbenzamide (**AI21**). Light yellow solid, yield 75%, m.p. 171.9-172.6 °C. ^{1}H NMR (500 MHz, DMSO-d_6) δ 9.88 (s, 1H), 8.75 (t, *J* = 6.1 Hz, 1H), 7.66 (d, *J* = 2.3 Hz, 1H), 7.58 (dd, *J* = 8.4, 2.3 Hz, 1H), 7.41 – 7.34 (m, 2H), 7.31 (d, *J* = 8.4 Hz, 2H), 6.80 (d, *J* = 8.3 Hz, 1H), 4.41 (d, *J* = 6.0 Hz, 2H), 2.14 (s, 3H). ^{13}C NMR (125 MHz, DMSO-d_6) δ 166.1, 158.3, 139.1, 131.1, 130.0, 129.0, 128.1, 126.5, 124.7, 123.5, 114.0, 41.8, 16.0. ESI-HRMS: m/z calcd for $C_{15}H_{15}ClNO_2$ [M+H]$^+$: 276.0786; found: 276.0783. HPLC analysis: retention time = 6.480 min; peak area, 99.30%.

N-(2-hydroxybenzyl)-2-(4-(4-methylbenzyl)piperazin-1-yl)isonicotinamide (**AI22**). Light yellow solid, yield 15%, m.p. 184.5-185.3 °C. ^{1}H NMR (500 MHz, DMSO-d_6) δ 9.01 (t, *J* = 5.9 Hz, 1H), 8.20 (d, *J* = 5.2 Hz, 1H), 7.24 – 7.18 (m, 3H), 7.14 (d, *J* = 7.8 Hz, 2H), 7.11 (dd, *J* = 7.5, 1.7 Hz, 1H), 7.07 (td, *J* = 7.7, 1.8 Hz, 1H), 7.03 (dd, *J* = 5.1, 1.3 Hz, 1H), 6.81 (dd, *J* = 8.1, 1.2 Hz, 1H), 6.75 (td, *J* = 7.4, 1.2 Hz, 1H), 4.41 (d, *J* = 5.8 Hz, 2H), 3.56 – 3.50 (m, 4H), 3.46 (s, 2H), 2.44 (t, *J* = 5.0 Hz, 4H), 2.28 (s, 3H). ^{13}C NMR (125 MHz, DMSO-d_6) δ 165.5, 159.4, 154.8, 148.2, 142.9, 136.0, 134.9, 128.9, 128.8, 128.2, 127.9, 124.8, 118.8, 115.0, 110.4, 104.6, 61.9, 52.3, 44.7, 38.0, 20.7. ESI-HRMS: m/z calcd for $C_{25}H_{29}N_4O_2$ [M+H]$^+$: 417.2285; found: 417.2287. HPLC analysis: retention time = 11.123 min; peak area, 98.00%.

(E)-3-(3,4-dihydroxyphenyl)-N-(4-fluorophenyl)acrylamide (**AI23**). Light yellow solid, yield 65%, m.p. 65.8-66.9 °C. ^{1}H NMR (500 MHz, DMSO-d_6) δ 10.12 (s, 1H), 9.46 (s, 1H), 9.19 (s, 1H), 7.74 – 7.68 (m, 2H), 7.41 (d, J = 15.6 Hz, 1H), 7.19 – 7.13 (m, 2H), 7.02 (d, J = 2.1 Hz, 1H), 6.91 (dd, J = 8.2, 2.1 Hz, 1H), 6.78 (d, J = 8.1 Hz, 1H), 6.52 (d, J = 15.5 Hz, 1H). ^{13}C NMR (125 MHz, DMSO-d_6) δ 164.0, 158.9 (d, ^1J = 238.75 Hz), 147.8, 145.6, 140.9, 135.9 (d, ^4J = 2.5 Hz), 126.2, 120.9, 120.8 (d, ^3J = 7.5 Hz), 118.2, 115.8, 115.4 (d, ^2J = 21.25 Hz), 114.0, 48.6. ESI-HRMS: m/z calcd for $C_{15}H_{13}FNO_3$ $[M+H]^+$: 274.0874; found: 274.0868. HPLC analysis: retention time = 7.078 min; peak area, 99.44%.

N-(2-hydroxybenzyl)-1-methyl-1H-indazole-5-carboxamide (**AI24**). White solid, yield 29%, m.p. 185.0-185.5 °C. ^{1}H NMR (500 MHz, DMSO-d_6) δ 9.66 (s, 1H), 9.02 – 8.95 (m, 1H), 8.43 – 8.38 (m, 1H), 8.19 (d, J = 1.0 Hz, 1H), 7.95 (dd, J = 8.8, 1.6 Hz, 1H), 7.70 (dd, J = 8.8, 1.0 Hz, 1H), 7.16 (dd, J = 7.6, 1.7 Hz, 1H), 7.07 (td, J = 7.6, 1.7 Hz, 1H), 6.82 (dt, J = 8.0, 1.1 Hz, 1H), 6.76 (td, J = 7.5, 1.2 Hz, 1H), 4.45 (d, J = 5.8 Hz, 2H), 4.07 (s, 3H). ^{13}C NMR (125 MHz, DMSO-d_6) δ 166.9, 154.9, 140.6, 133.8, 128.3, 127.8, 126.6, 125.4, 125.2, 123.0, 121.0, 118.8, 115.2, 109.4, 38.2, 35.5. ESI-HRMS: m/z calcd for $C_{16}H_{16}N_3O_2$ $[M+H]^+$: 282.1237; found: 282.1231. HPLC analysis: retention time = 6.373 min; peak area, 98.04%.

N-(4-hydroxybenzyl)-6-(((4-methoxypyridin-2-yl)amino)methyl)picolinamide (**AI25**). White solid, yield 53%, m.p. 187.7-188.4 °C. ^{1}H NMR (500 MHz, DMSO-d_6) δ 9.28 (s, 1H), 9.14 (t, J = 6.4 Hz, 1H), 7.91 (q, J = 7.1, 6.5 Hz, 2H), 7.76 (d, J = 5.8 Hz, 1H), 7.49 (dd, J = 6.7, 2.2 Hz, 1H), 7.14 (d, J = 8.4 Hz, 3H), 6.71 (d, J = 8.4 Hz, 2H), 6.17 (dd, J = 5.8, 2.3 Hz, 1H), 6.12 (d, J = 2.3 Hz, 1H), 4.62 (d, J = 5.9 Hz, 2H), 4.41 (d, J = 6.4 Hz, 2H), 3.71 (s, 3H). ^{13}C NMR (125 MHz, DMSO-d_6) δ 166.2, 163.8, 160.4, 159.1, 156.4, 149.2, 148.6, 138.2, 129.7, 128.8, 123.8, 120.0, 115.1, 101.6, 91.5, 54.8, 46.1, 41.9. ESI-HRMS: m/z calcd for $C_{20}H_{20}N_4O_3$ $[M+H]^+$: 365.1608; found: 365.1612. HPLC analysis: retention time = 10.267 min; peak area, 100.00%.

N-(4-hydroxybenzyl)-1-methyl-1H-indole-3-carboxamide (**AI26**). Light yellow solid, yield 48%, m.p. 206.1-206.5 °C. ^{1}H NMR (500 MHz, DMSO-d_6) δ 9.23 (dd, J = 2.8, 1.5 Hz, 1H), 8.32 – 8.25 (m, 1H), 8.19 – 8.14 (m, 1H), 8.01 (s, 1H), 7.48 (dd, J = 8.3, 1.0 Hz, 1H), 7.21 (ddd, J = 8.2, 7.0, 1.3 Hz, 1H), 7.17 – 7.10 (m, 3H), 6.73 – 6.67 (m, 2H), 4.35 (d, J = 5.9 Hz, 2H), 3.81 (s, 3H). ^{13}C NMR (125 MHz, DMSO-d_6) δ 164.0, 156.1, 136.7, 131.7, 130.5, 128.5, 126.5, 121.9, 121.2, 120.5, 114.9, 110.2, 109.6, 41.4, 32.9. ESI-HRMS: m/z calcd for $C_{17}H_{17}N_2O_2$ $[M+H]^+$: 281.1285; found: 281.1285. HPLC analysis: retention time = 6.250 min; peak area, 97.91%.

N-(4-(4-hydroxyphenyl)thiazol-2-yl)-2-(2-methoxyphenyl)acetamide (**AI27**). White solid, yield 53%, m.p. 134.3-135.1 °C. ^{1}H NMR (500 MHz, DMSO-d_6) δ 7.83 – 7.81 (m, 1H), 7.81 (d, J = 1.0 Hz, 1H), 7.30 (ddt, J = 6.5, 4.7, 2.0 Hz, 2H), 7.09 – 7.05 (m, 4H), 7.03 (dd, J = 8.7, 1.0 Hz, 1H), 6.99 (s, 1H), 6.94 (td, J = 7.4, 1.1 Hz, 1H), 3.86 (s, 2H), 3.83 (s, 3H). ^{13}C NMR (125 MHz, DMSO-d_6) δ 170.1, 168.3, 157.3, 149.6, 149.0, 132.6, 131.0, 128.7, 126.6, 122.6, 121.7, 120.3, 110.8, 101.5, 55.5, 35.6. ESI-HRMS: m/z calcd for $C_{18}H_{17}N_2O_3S$ [M+H]$^+$: 341.0954; found: 341.0956. HPLC analysis: retention time = 7.352 min; peak area, 99.17%.

2-(4-fluoro-2-methoxyphenyl)-N-(4-(4-hydroxyphenyl)thiazol-2-yl)acetamide (**AI28**). White solid, yield 66%, m.p. 145.6-146.0 ℃. ^{1}H NMR (500 MHz, DMSO-d_6) δ 7.85 – 7.79 (m, 2H), 7.21 (dd, J = 9.0, 3.2 Hz, 1H), 7.12 (td, J = 8.7, 3.2 Hz, 1H), 7.10 – 7.07 (m, 2H), 7.07 – 7.01 (m, 3H), 6.99 (s, 1H), 3.89 (s, 2H), 3.82 (s, 3H). ^{13}C NMR (125 MHz, DMSO-d_6) δ 169.7, 168.4, 156.9 (d, ^1J = 233.75 Hz), 153.8, 149.6, 149.1, 132.7, 126.7, 124.5 (d, ^3J = 7.5 Hz), 121.7, 117.9 (d, ^2J = 23.75 Hz), 114.6 (d, ^2J = 22.5 Hz), 112.0 (d, ^3J = 8.75 Hz), 101.6, 56.2, 35.4. ESI-HRMS: m/z calcd for $C_{18}H_{16}FN_2O_3S$ [M+H]$^+$: 359.0860; found: 359.0866. HPLC analysis: retention time = 7.213 min; peak area, 98.20%.

(2-hydroxyphenyl)(4-(4-hydroxyphenyl)piperazin-1-yl)methanone (**AI10-m1**). Yellowish brown solid, yield 65%, m.p. 182.9-183.7 °C. ^{1}H NMR (400 MHz, DMSO-d_6) δ 9.82 (s, 1H), 8.90 (s, 1H), 7.22 (ddd, J = 8.2, 7.3, 1.8 Hz, 1H), 7.13 (dd, J = 7.5, 1.7 Hz, 1H), 6.90 – 6.83 (m, 2H), 6.83 – 6.77 (m, 2H), 6.67 – 6.61 (m, 2H), 3.72 (s, 2H), 3.32 (s, 2H), 2.94 (s, 4H). ^{13}C NMR (100 MHz, DMSO-d_6) δ 167.0, 153.3, 151.4, 144.0, 130.2, 128.2, 123.9, 119.2, 118.5, 115.7, 115.5, 50.5, 48.7. ESI-HRMS: m/z calcd for $C_{17}H_{19}N_2O_3$ [M+H]$^+$: 299.1390; found: 299.1386. HPLC analysis: retention time = 10.238 min; peak area, 100.00%.

(2-hydroxy-5-methylphenyl)(4-(4-hydroxyphenyl)piperazin-1-yl)methanone (**AI10-m2**). White solid, yield 64%, m.p. 193.8-194.1 °C. ^{1}H NMR (500 MHz, DMSO-d_6) δ 9.54 (s, 1H), 8.88 (s, 1H), 7.02 (dd, J = 8.4, 2.3 Hz, 1H), 6.93 (d, J = 2.2 Hz, 1H), 6.83 – 6.73 (m, 3H), 6.69 – 6.62 (m, 2H), 3.56 (d, J = 145.8 Hz, 4H), 2.93 (s, 4H), 2.20 (s, 3H). ^{13}C NMR (125 MHz, DMSO-d_6) δ 167.1, 151.4, 151.0, 144.0, 130.6, 128.3, 127.7, 123.6, 118.5, 115.5, 115.5, 50.5, 19.9. ESI-HRMS: m/z calcd for $C_{18}H_{21}N_2O_3$ [M+H]$^+$: 313.1547; found: 313.1541. HPLC analysis: retention time = 8.990 min; peak area, 98.46%.

(5-hydroxy-2-methylphenyl)(4-(4-hydroxyphenyl)piperazin-1-yl)methanone (**AI10-m3**). Yellowish brown solid, yield 79%, m.p. 108.3-109.1 °C. ^{1}H NMR (500 MHz, DMSO-d_6) δ 9.41

(s, 1H), 8.89 (s, 1H), 7.05 (d, $J = 8.3$ Hz, 1H), 6.83 – 6.77 (m, 2H), 6.70 (dd, $J = 8.3$, 2.6 Hz, 1H), 6.68 – 6.63 (m, 2H), 6.54 (d, $J = 2.7$ Hz, 1H), 3.75 (t, $J = 5.1$ Hz, 2H), 3.26 (t, $J = 5.1$ Hz, 2H), 3.00 (d, $J = 5.8$ Hz, 2H), 2.89 – 2.79 (m, 2H), 2.09 (s, 3H). ^{13}C NMR (125 MHz, DMSO-d_6) δ 168.4, 155.2, 151.5, 143.8, 137.1, 131.2, 123.4, 118.6, 115.6, 115.5, 112.3, 50.7, 50.5, 46.3, 40.9, 17.6. ESI-HRMS: m/z calcd for $C_{18}H_{21}N_2O_3$ [M+H]$^+$: 313.1547; found:313.1548. HPLC analysis: retention time = 10.193 min; peak area, 99.54%.

(3-hydroxy-2-methylphenyl)(4-(4-hydroxyphenyl)piperazin-1-yl)methanone (**AI10-m4**). Yellowish brown solid, yield 28%, m.p. 117.6-118.4 °C. ^{1}H NMR (400 MHz, DMSO-d_6) δ 9.61 (s, 1H), 8.92 (s, 1H), 7.05 (t, $J = 7.8$ Hz, 1H), 6.83 – 6.77 (m, 3H), 6.68 – 6.63 (m, 2H), 6.60 (dd, $J = 7.6$, 1.2 Hz, 1H), 3.76 (dtt, $J = 19.9$, 12.9, 3.7 Hz, 2H), 3.23 (t, $J = 5.1$ Hz, 2H), 2.99 (ddd, $J =$ 11.5, 6.5, 4.2 Hz, 2H), 2.91 – 2.73 (m, 2H), 2.01 (s, 3H). ^{13}C NMR (100 MHz, DMSO-d_6) δ 168.6, 155.6, 151.5, 143.9, 137.8, 126.7, 120.1, 118.6, 116.2, 115.5, 114.7, 50.7, 50.5, 46.4, 40.9, 12.6. ESI-HRMS: m/z calcd for $C_{18}H_{21}N_2O_3$ [M+H]$^+$: 313.1547; found: 313.1541. HPLC analysis: retention time = 10.176 min; peak area, 99.44%.

(4-fluorophenyl)(4-(4-hydroxyphenyl)piperazin-1-yl)methanone (**AI10-m5**). White solid, yield 36%, m.p. 164.7-165.3 °C. ^{1}H NMR (500 MHz, DMSO-d_6) δ 8.88 (s, 1H), 7.52 – 7.48 (m, 2H), 7.31 – 7.26 (m, 2H), 6.82 – 6.78 (m, 2H), 6.68 – 6.64 (m, 2H), 3.72 (s, 4H), 2.97 (s, 4H). ^{13}C NMR (125 MHz, DMSO-d_6) δ 168.1, 163.5 (d, $^1J = 245$ Hz), 151.4, 143.8, 132.3 (d, $^4J = 2.5$ Hz), 129.6 (d, $^3J = 8.75$ Hz), 118.5, 115.5, 115.3, 50.4. ESI-HRMS: m/z calcd for $C_{17}H_{18}FN_2O_3$ [M+H]$^+$: 301.1347; found: 301.1346. HPLC analysis: retention time = 10.289 min; peak area, 100.00%.

(4-(4-hydroxyphenyl)piperazin-1-yl)(3,4,5-trimethoxyphenyl)methanone (**AI10-m6**). White solid, yield 74%, m.p. 212.1-212.5 °C. ^{1}H NMR (400 MHz, DMSO-d_6) δ 8.91 (s, 1H), 6.81 (d, $J = 8.9$ Hz, 2H), 6.70 (s, 2H), 6.66 (d, $J = 8.9$ Hz, 2H), 3.79 (s, 6H), 3.71 (s, 2H), 3.68 (s, 3H), 3.49 (s, 2H), 2.97 (s, 4H). ^{13}C NMR (100 MHz, DMSO-d_6) δ 168.7, 152.8, 151.4, 143.9, 138.2, 131.3, 118.6, 115.5, 104.4, 60.1, 56.1. ESI-HRMS: m/z calcd for $C_{20}H_{25}N_2O_5$ [M+H]$^+$:373.1758; found: 373.1760. HPLC analysis: retention time = 10.166 min; peak area, 100.00%.

(4-(4-hydroxyphenyl)piperazin-1-yl)(pyridin-3-yl)methanone (**AI10-m7**). Brown solid, yield 37%, m.p. 155.8-156.3 °C. ^{1}H NMR (500 MHz, DMSO-d_6) δ 8.90 (s, 1H), 8.70 – 8.62 (m, 2H), 7.87 (dt, $J = 7.8$, 2.0 Hz, 1H), 7.49 (ddd, $J = 7.8$, 4.9, 0.9 Hz, 1H), 6.81 (d, $J = 8.9$ Hz, 2H), 6.66 (d, $J = 8.9$ Hz, 2H), 3.76 (s, 2H), 3.45 (s, 2H), 2.99 (d, $J = 42.1$ Hz, 4H). ^{13}C NMR (125 MHz, DMSO-d_6) δ 166.7, 151.5, 150.5, 147.7, 143.8, 134.9, 131.7, 123.6, 118.6, 115.5, 50.5, 50.2, 47.3,

41.7. ESI-HRMS: m/z calcd for $C_{16}H_{18}N_3O_2$ $[M+H]^+$: 284.1394; found: 284.1392. HPLC analysis: retention time = 6.162 min; peak area, 99.01%.

(4-(4-hydroxyphenyl)piperazin-1-yl)(pyridin-2-yl)methanone (**AI10-m8**). White solid, yield 31%, m.p. 136.7-137.4 °C. ^{1}H NMR (500 MHz, DMSO-d_6) δ 8.89 (s, 1H), 8.60 (ddd, J = 4.9, 1.8, 1.0 Hz, 1H), 7.93 (td, J = 7.7, 1.7 Hz, 1H), 7.60 (dt, J = 7.8, 1.1 Hz, 1H), 7.48 (ddd, J = 7.7, 4.8, 1.2 Hz, 1H), 6.81 (d, J = 8.8 Hz, 2H), 6.66 (d, J = 8.8 Hz, 2H), 3.80 – 3.76 (m, 2H), 3.55 – 3.50 (m, 2H), 3.03 (t, J = 5.2 Hz, 2H), 2.92 (t, J = 5.0 Hz, 2H). ^{13}C NMR (125 MHz, DMSO-d_6) δ 166.6, 154.0, 151.4, 148.4, 143.9, 137.4, 124.6, 123.3, 118.5, 115.5, 50.6, 50.4, 46.6, 41.7. ESI-HRMS: m/z calcd for $C_{16}H_{18}N_3O_2$ $[M+H]^+$: 284.1394; found: 284.1388. HPLC analysis: retention time = 6.185 min; peak area, 99.50%.

(4-(4-hydroxyphenyl)piperazin-1-yl)(pyridin-4-yl)methanone (**AI10-m9**). Yellow solid, yield 30%, m.p. 236.9-237.5 °C. ^{1}H NMR (500 MHz, DMSO-d_6) δ 8.91 (s, 1H), 8.68 (d, J = 5.9 Hz, 2H), 7.45 – 7.38 (m, 2H), 6.84 – 6.77 (m, 2H), 6.69 – 6.63 (m, 2H), 3.75 (t, J = 5.0 Hz, 2H), 3.37 (t, J = 4.8 Hz, 2H), 3.03 (t, J = 5.2 Hz, 2H), 2.92 (t, J = 5.0 Hz, 2H). ^{13}C NMR (125 MHz, DMSO-d_6) δ 166.7, 151.5, 150.1, 143.8, 143.4, 121.3, 118.6, 115.5, 50.5, 50.2, 46.9, 41.5. ESI-HRMS: m/z calcd for $C_{16}H_{18}N_3O_2$ $[M+H]^+$: 284.1394; found: 284.1390. HPLC analysis: retention time = 10.253 min; peak area, 98.28%.

(2,6-dimethylpyridin-4-yl)(4-(2-hydroxyphenyl)piperazin-1-yl)methanone (**AI10-m10**). White solid, yield 62%, m.p. 97.8-98.7 °C. ^{1}H NMR (500 MHz, DMSO-d_6) δ 9.03 (s, 1H), 7.06 (s, 2H), 6.89 (dd, J = 7.8, 1.6 Hz, 1H), 6.85 (td, J = 7.5, 1.5 Hz, 1H), 6.79 (dd, J = 7.9, 1.7 Hz, 1H), 6.74 (td, J = 7.4, 1.6 Hz, 1H), 3.77 (t, J = 5.1 Hz, 2H), 3.41 (t, J = 4.8 Hz, 2H), 2.97 (t, J = 5.0 Hz, 2H), 2.88 (t, J = 5.5 Hz, 2H), 2.46 (s, 6H). ^{13}C NMR (125 MHz, DMSO-d_6) δ 167.2, 157.8, 150.2, 144.2, 139.3, 123.4, 119.4, 119.1, 117.4, 115.6, 50.5, 50.1, 47.1, 41.5, 24.0. ESI-HRMS: m/z calcd for $C_{18}H_{22}N_3O_2$ $[M+H]^+$: 312.1707; found: 312.1704. HPLC analysis: retention time = 7.455 min; peak area, 99.63%.

(2,6-dimethylpyridin-4-yl)(4-(3-hydroxyphenyl)piperazin-1-yl)methanone (**AI10-m11**). Yellow solid, yield 52%, m.p. 111.8-112.5 °C. ^{1}H NMR (500 MHz, DMSO-d_6) δ 9.17 (s, 1H), 7.06 (s, 2H), 6.99 (t, J = 8.1 Hz, 1H), 6.39 (dd, J = 8.2, 2.3 Hz, 1H), 6.32 (t, J = 2.3 Hz, 1H), 6.24 (dd, J = 7.9, 2.1 Hz, 1H), 3.73 (t, J = 5.1 Hz, 2H), 3.41 – 3.35 (m, 2H), 3.15 (t, J = 5.1 Hz, 2H), 3.05 (t, J = 5.2 Hz, 2H), 2.46 (s, 6H). ^{13}C NMR (125 MHz, DMSO-d_6) δ 167.2, 158.1, 157.8, 152.1, 144.1, 129.6, 117.4, 107.1, 106.7, 103.0, 48.6, 48.3, 46.6, 41.2, 24.0. ESI-HRMS: m/z calcd for

$C_{18}H_{22}N_3O_2$ [M+H]$^+$: 312.1707; found: 312.1706. HPLC analysis: retention time = 6.328 min; peak area, 98.37%.

(4-(2,4-dihydroxyphenyl)piperazin-1-yl)(2,6-dimethylpyridin-4-yl)methanone **(AI10-m12)**. Brown solid, yield 60%, m.p. 229.6-230.2 °C. ^{1}H NMR (500 MHz, DMSO-d_6) δ 8.94 (s, 1H), 8.67 (s, 1H), 7.05 (s, 2H), 6.77 (d, J = 8.5 Hz, 1H), 6.27 (d, J = 2.7 Hz, 1H), 6.15 (dd, J = 8.5, 2.7 Hz, 1H), 3.73 (d, J = 16.1 Hz, 2H), 3.38 (s, 2H), 2.85 – 2.71 (m, 4H), 2.45 (s, 6H). ^{13}C NMR (125 MHz, DMSO-d_6) δ 167.1, 157.8, 154.4, 151.6, 144.2, 131.3, 120.6, 117.4, 105.7, 102.9, 51.5, 51.2, 47.2, 41.7, 24.0. ESI-HRMS: m/z calcd for $C_{18}H_{22}N_3O_3$ [M+H]$^+$: 328.1656; found: 328.1656. HPLC analysis: retention time = 10.186 min; peak area, 98.47%.

2-(4-fluorophenyl)-1-(4-(4-hydroxyphenyl)piperazin-1-yl)ethan-1-one **(AI10-m13)**. White solid, yield 57%, m.p. 202.6-203.4 °C. ^{1}H NMR (500 MHz, DMSO-d_6) δ 8.87 (s, 1H), 7.29 – 7.24 (m, 2H), 7.15 – 7.10 (m, 2H), 6.80 – 6.76 (m, 2H), 6.67 – 6.63 (m, 2H), 3.74 (s, 2H), 3.60 (dt, J = 10.6, 5.1 Hz, 4H), 2.87 (dt, J = 9.7, 5.1 Hz, 4H). ^{13}C NMR (125 MHz, DMSO-d_6) δ 168.7, 161.9 (d, ^1J = 241.25 Hz), 151.4, 143.9, 132.1 (d, ^4J = 3.75 Hz), 131.0 (d, ^3J = 7.5 Hz), 118.4, 115.5 (d, ^2J = 21.25 Hz), 114.9, 50.6, 50.1, 45.4, 41.3, 38.4. ESI-HRMS: m/z calcd for $C_{18}H_{20}FN_2O_2$ [M+H]$^+$: 315.1503; found: 315.1499. HPLC analysis: retention time = 10.325 min; peak area, 100.00%.

1-(4-(4-hydroxyphenyl)piperazin-1-yl)-2-(2-methoxyphenyl)ethan-1-one **(AI10-m14)**. White solid, yield 62%, m.p. 193.7-194.1 °C. ^{1}H NMR (500 MHz, DMSO-d_6) δ 8.87 (s, 1H), 7.22 (ddd, J = 8.1, 7.4, 1.8 Hz, 1H), 7.11 (dd, J = 7.4, 1.8 Hz, 1H), 6.97 (dd, J = 8.3, 1.1 Hz, 1H), 6.88 (td, J = 7.4, 1.1 Hz, 1H), 6.81 – 6.77 (m, 2H), 6.68 – 6.63 (m, 2H), 3.76 (s, 3H), 3.63 (s, 2H), 3.61 – 3.58 (m, 4H), 2.88 (q, J = 6.0, 5.4 Hz, 4H). ^{13}C NMR (125 MHz, DMSO-d_6) δ 168.8, 156.8, 151.3, 143.9, 130.2, 127.9, 124.1, 120.2, 118.4, 115.5, 110.7, 55.4, 50.6, 50.2, 45.5, 41.3, 33.8. ESI-HRMS: m/z calcd for $C_{19}H_{23}N_2O_3$ [M+H]$^+$: 327.1703; found: 327.1704. HPLC analysis: retention time = 6.394 min; peak area, 98.56%.

(E)-3-(2,4-dihydroxyphenyl)-1-(4-(4-hydroxyphenyl)piperazin-1-yl)prop-2-en-1-one **(AI10-m15)**. Yellow solid, yield 46%, m.p. 150.2-150.7 °C. ^{1}H NMR (500 MHz, DMSO-d_6) δ 9.88 (s, 1H), 9.70 (s, 1H), 8.88 (s, 1H), 7.72 (d, J = 15.4 Hz, 1H), 7.50 (d, J = 8.6 Hz, 1H), 6.99 (d, J = 15.4 Hz, 1H), 6.85 – 6.79 (m, 2H), 6.69 – 6.63 (m, 2H), 6.34 (d, J = 2.3 Hz, 1H), 6.25 (dd, J = 8.5, 2.4 Hz, 1H), 3.71 (s, 4H), 3.02 – 2.87 (m, 4H). ^{13}C NMR (125 MHz, DMSO-d_6) δ 165.7, 160.4, 158.1, 151.7, 144.3, 137.9, 129.9, 118.8, 115.8, 113.9, 113.1, 107.8, 102.8. ESI-HRMS: m/z calcd

for $C_{19}H_{21}N_2O_4$ $[M+H]^+$: 341.1496; found: 341.1493. HPLC analysis: retention time = 6.680 min; peak area, 99.04%.

(E)-3-(3,4-dihydroxyphenyl)-1-(4-(4-hydroxyphenyl)piperazin-1-yl)prop-2-en-1-one (**AI10-m16**). Yellow solid, yield 36%, m.p. 238.2-239.3 °C. ^{1}H NMR (400 MHz, DMSO-d_6) δ 9.47 (s, 1H), 8.96 (d, J = 40.5 Hz, 2H), 7.34 (d, J = 15.2 Hz, 1H), 7.10 (d, J = 2.1 Hz, 1H), 7.04 – 6.93 (m, 2H), 6.84 – 6.78 (m, 2H), 6.74 (d, J = 8.1 Hz, 1H), 6.69 – 6.60 (m, 2H), 3.73 (d, J = 44.6 Hz, 4H), 2.94 (s, 4H). ^{13}C NMR (100 MHz, DMSO-d_6) δ 164.8, 151.4, 147.4, 145.5, 144.0, 142.4, 126.7, 120.7, 118.5, 115.6, 115.5, 115.0, 114.2. ESI-HRMS: m/z calcd for $C_{19}H_{21}N_2O_4$ $[M+H]^+$: 341.1496; found: 341.1490. HPLC analysis: retention time = 10.136 min; peak area, 99.89%.

(4-(4-hydroxyphenyl)piperazin-1-yl)(thiazol-2-yl)methanone (**AI10-m17**). Yellow solid, yield 43%, m.p. 144.8-145.1 °C. ^{1}H NMR (500 MHz, DMSO-d_6) δ 8.88 (s, 1H), 8.03 (q, J = 3.2 Hz, 2H), 6.85 – 6.81 (m, 2H), 6.68 – 6.65 (m, 2H), 4.42 (t, J = 4.9 Hz, 2H), 3.80 (d, J = 6.3 Hz, 2H), 3.04 (d, J = 5.8 Hz, 4H). ^{13}C NMR (125 MHz, DMSO-d_6) δ 164.5, 158.4, 151.4, 143.8, 143.5, 125.4, 118.5, 115.5, 50.7, 50.3, 45.9, 43.0. ESI-HRMS: m/z calcd for $C_{14}H_{16}N_3O_2S$ $[M+H]^+$: 290.0958; found: 290.0957. HPLC analysis: retention time = 10.438 min; peak area, 99.19%.

(4-(4-hydroxyphenyl)piperazin-1-yl)(1-methyl-1H-indazol-6-yl)methanone (**AI10-m18**). Yellow solid, yield 46%, m.p. 208.6-209.2 °C. ^{1}H NMR (500 MHz, DMSO-d_6) δ 8.89 (s, 1H), 8.13 (d, J = 0.9 Hz, 1H), 7.87 (d, J = 1.2 Hz, 1H), 7.70 (dd, J = 8.7, 0.9 Hz, 1H), 7.46 (dd, J = 8.6, 1.5 Hz, 1H), 6.84 – 6.78 (m, 2H), 6.69 – 6.62 (m, 2H), 4.07 (s, 3H), 3.63 (s, 4H), 2.98 (s, 4H). ^{13}C NMR (125 MHz, DMSO-d_6) δ 169.4, 151.4, 143.9, 139.7, 133.3, 127.9, 125.4, 122.8, 120.3, 118.5, 115.5, 109.7, 50.5, 35.5. ESI-HRMS: m/z calcd for $C_{19}H_{21}N_4O_2$ $[M+H]^+$: 290.0958; found: 290.0957. HPLC analysis: retention time = 6.394 min; peak area, 97.92%.

(4-(4-hydroxyphenyl)piperazin-1-yl)(1-methyl-1H-indazol-3-yl)methanone (**AI10-m19**). White solid, yield 74%, m.p. 201.8-202.2 °C. ^{1}H NMR (500 MHz, DMSO-d_6) δ 8.89 (d, J = 1.9 Hz, 1H), 8.00 (d, J = 8.2 Hz, 1H), 7.71 (d, J = 8.5 Hz, 1H), 7.46 (t, J = 7.7 Hz, 1H), 7.25 (t, J = 7.5 Hz, 1H), 6.83 (d, J = 8.6 Hz, 2H), 6.67 (d, J = 8.6 Hz, 2H), 4.12 (s, 5H), 3.85 (s, 2H), 3.09 – 2.92 (m, 4H). ^{13}C NMR (125 MHz, DMSO-d_6) δ 161.5, 151.4, 143.9, 140.1, 137.2, 126.6, 123.5, 122.0, 121.7, 118.5, 115.5, 110.1, 50.9, 50.5, 46.5, 42.0, 35.9. ESI-HRMS: m/z calcd for $C_{19}H_{21}N_4O_2$ $[M+H]^+$: 337.1659; found: 337.1654. HPLC analysis: retention time = 6.458 min; peak area, 98.37%.

(4-(4-hydroxyphenyl)piperazin-1-yl)(1-methyl-1H-indol-3-yl)methanone (**AI10-m20**). Pink solid, yield 43%, m.p. 239.7-240.3 °C. ^{1}H NMR (500 MHz, DMSO-d_6) δ 8.88 (s, 1H), 7.76 (s, 1H), 7.72 (dt, J = 7.9, 1.0 Hz, 1H), 7.50 (dt, J = 8.2, 0.9 Hz, 1H), 7.23 (ddd, J = 8.1, 7.0, 1.2 Hz, 1H), 7.15 (ddd, J = 8.0, 7.0, 1.0 Hz, 1H), 6.84 – 6.79 (m, 2H), 6.69 – 6.64 (m, 2H), 3.83 (s, 3H), 3.75 (t, J = 5.1 Hz, 4H), 2.99 (t, J = 5.1 Hz, 4H). ^{13}C NMR (125 MHz, DMSO-d_6) δ 165.1, 151.4, 144.0, 136.2, 132.1, 126.3, 121.9, 120.5, 120.4, 118.4, 115.5, 110.3, 108.7, 50.7, 32.7. ESI-HRMS: m/z calcd for $C_{20}H_{22}N_3O_2$ [M+H]$^+$: 336.1707; found: 336.1703. HPLC analysis: retention time = 6.397 min; peak area, 98.58%.

4-(4-(2-fluorobenzyl)piperazin-1-yl)phenol (**AI10-m21**). Pink solid, yield 43%, m.p. 239.7-240.3 °C. Orange solid, yield 79%, m.p. 151.1-151.8 °C. ^{1}H NMR (500 MHz, DMSO-d_6) δ 8.79 (s, 1H), 7.46 – 7.39 (m, 1H), 7.36 – 7.29 (m, 1H), 7.22 – 7.11 (m, 2H), 6.75 (d, J = 9.0 Hz, 2H), 6.63 (d, J = 8.9 Hz, 2H), 3.56 (s, 2H), 2.99 – 2.89 (m, 4H), 2.51 (t, J = 4.4 Hz, 4H). ^{13}C NMR (125 MHz, DMSO-d_6) δ 161.8 (d, ^1J = 242.5 Hz), 150.9, 144.2, 131.7 (d, ^4J = 3.75 Hz), 129.1 (d, ^3J = 7.5 Hz), 124.5 (d, ^2J = 15 Hz), 124.2, (d, ^4J = 2.5 Hz), 117.8, 115.4, 115.2 (d, ^2J = 22.5 Hz), 54.5, 52.6, 50.0. ESI-HRMS: m/z calcd for $C_{17}H_{20}FN_2O$ [M+H]$^+$: 287.1554; found: 287.1554. HPLC analysis: retention time = 10.207 min; peak area, 98.49%.

4-(4-(4-fluorobenzyl)piperazin-1-yl)phenol (**AI10-m22**). Pink solid, yield 58%, m.p. 166.4-167.4 °C. ^{1}H NMR (500 MHz, DMSO-d_6) δ 8.79 (s, 1H), 7.38 – 7.32 (m, 2H), 7.17 – 7.11 (m, 2H), 6.78 – 6.73 (m, 2H), 6.65 – 6.61 (m, 2H), 3.49 (s, 2H), 2.94 (t, J = 4.9 Hz, 4H), 2.48 (t, J = 5.0 Hz, 4H). ^{13}C NMR (125 MHz, DMSO-d_6) δ 162.2 (d, ^1J = 241.25 Hz), 150.9, 144.2, 134.3, 130.7 (d, ^3J = 8.75 Hz) 117.7, 115.4, 115.0 (d, ^2J = 21.25 Hz), 61.1, 52.6, 50.0. ESI-HRMS: m/z calcd for $C_{17}H_{20}FN_2O$ [M+H]$^+$: 287.1554; found: 287.1561. HPLC analysis: retention time = 6.139 min; peak area, 98.24%.

4-(4-(3-fluorobenzyl)piperazin-1-yl)phenol (**AI10-m23**). White solid, yield 34%, m.p. 156.0-157.0 °C. ^{1}H NMR (500 MHz, DMSO-d_6) δ 8.79 (s, 1H), 7.40 – 7.34 (m, 1H), 7.15 (dd, J = 12.5, 7.6 Hz, 2H), 7.11 – 7.05 (m, 1H), 6.76 (d, J = 9.0 Hz, 2H), 6.63 (d, J = 8.9 Hz, 2H), 3.53 (s, 2H), 2.95 (dd, J = 6.2, 3.6 Hz, 4H), 2.50 – 2.47 (m, 4H). ^{13}C NMR (125 MHz, DMSO-d_6) δ 163.2 (d, ^1J = 240 Hz), 150.9, 144.2, 141.4 (d, ^3J = 6.25 Hz), 130.1 (d, ^3J = 7.5 Hz), 124.8 (d, ^4J = 2.5 Hz), 117.7, 115.4, 115.3 (d, ^2J = 21.25 Hz), 113.8 (d, ^2J = 21.25 Hz), 61.3, 52.7, 50.0. ESI-HRMS: m/z calcd for $C_{17}H_{20}FN_2O$ [M+H]$^+$: 287.1554; found: 287.1553. HPLC analysis: retention time = 10.199 min; peak area, 99.75%.

4-(4-(2-chlorobenzyl)piperazin-1-yl)phenol (**AI10-m24**). Gray solid, yield 84%, m.p. 128.3-128.8 °C. ^{1}H NMR (500 MHz, DMSO-d_6) δ 8.82 (s, 1H), 7.51 (dd, *J* = 7.6, 1.9 Hz, 1H), 7.43 (dd, *J* = 7.8, 1.5 Hz, 1H), 7.31 (dtd, *J* = 24.0, 7.4, 1.7 Hz, 2H), 6.80 – 6.73 (m, 2H), 6.68 – 6.61 (m, 2H), 3.61 (s, 2H), 3.02 – 2.91 (m, 4H), 2.61 – 2.52 (m, 4H). ^{13}C NMR (125 MHz, DMSO-d_6) δ 150.9, 144.2, 135.5, 133.3, 130.9, 129.3, 128.6, 127.0, 117.8, 115.4, 58.6, 52.9, 50.0. ESI-HRMS: m/z calcd for $C_{17}H_{20}ClN_2O$ [M+H]$^+$: 303.1259; found: 303.1258. HPLC analysis: retention time = 7.958 min; peak area, 99.84%.

4-(4-(3-chlorobenzyl)piperazin-1-yl)phenol (**AI10-m25**). Brown solid, yield 25%, m.p. 157.1-157.7 °C. ^{1}H NMR (500 MHz, DMSO-d_6) δ 8.82 (s, 1H), 7.41 – 7.25 (m, 4H), 6.76 (d, *J* = 8.9 Hz, 2H), 6.68 – 6.60 (m, 2H), 3.52 (s, 2H), 3.02 – 2.89 (m, 4H), 2.49 (dd, *J* = 5.5, 3.4 Hz, 4H). ^{13}C NMR (125 MHz, DMSO-d_6) δ 150.9, 144.2, 140.9, 132.9, 130.1, 128.5, 127.5, 126.9, 117.8, 115.4, 61.2, 52.7, 50.0. ESI-HRMS: m/z calcd for $C_{17}H_{20}ClN_2O$ [M+H]$^+$: 303.1259; found: 303.1259. HPLC analysis: retention time = 10.211 min; peak area, 100.00%.

4-(4-(4-chlorobenzyl)piperazin-1-yl)phenol (**AI10-m26**). Reddish-brown solid, yield 57%, m.p. 170.2-171.0 °C. ^{1}H NMR (500 MHz, DMSO-d_6) δ 8.81 (s, 1H), 7.41 – 7.32 (m, 4H), 6.79 – 6.72 (m, 2H), 6.63 (d, *J* = 8.9 Hz, 2H), 3.49 (s, 2H), 2.98 – 2.91 (m, 4H), 2.48 (t, *J* = 4.9 Hz, 4H). ^{13}C NMR (125 MHz, DMSO-d_6) δ 150.9, 144.2, 137.3, 131.4, 130.6, 128.1, 117.7, 115.4, 61.1, 52.7, 50.0. ESI-HRMS: m/z calcd for $C_{17}H_{20}ClN_2O$ [M+H]$^+$: 303.1259; found: 303.1260. HPLC analysis: retention time = 10.191 min; peak area, 99.39%.

4-(4-(2-methylbenzyl)piperazin-1-yl)phenol (**AI10-m27**). Pink solid, yield 66%, m.p. 132.1-132.8 °C. ^{1}H NMR (500 MHz, DMSO-d_6) δ 8.82 (s, 1H), 7.23 (s, 1H), 7.16 (d, *J* = 3.5 Hz, 3H), 6.75 (d, *J* = 9.0 Hz, 2H), 6.63 (d, *J* = 8.9 Hz, 2H), 3.46 (s, 2H), 2.93 (t, *J* = 4.9 Hz, 4H), 2.49 (t, *J* = 3.7 Hz, 4H), 2.33 (s, 3H). ^{13}C NMR (125 MHz, DMSO-d_6) δ 150.9, 144.2, 137.1, 136.2, 130.1, 129.6, 127.0, 125.4, 117.7, 115.4, 60.2, 52.9, 50.1, 18.8. ESI-HRMS: m/z calcd for $C_{18}H_{23}N_2O$ [M+H]$^+$: 283.1805; found: 283.1805. HPLC analysis: retention time = 10.177 min; peak area, 99.47%.

4-(4-(3-methylbenzyl)piperazin-1-yl)phenol (**AI10-m28**). Pink solid, yield 52%, m.p. 167.5-168.0 °C. ^{1}H NMR (500 MHz, DMSO-d_6) δ 8.84 (s, 1H), 7.21 (t, *J* = 7.5 Hz, 1H), 7.15 – 7.08 (m, 2H), 7.06 (d, *J* = 7.5 Hz, 1H), 6.79 – 6.72 (m, 2H), 6.69 – 6.61 (m, 2H), 3.45 (s, 2H), 2.94 (t, *J* = 4.9 Hz, 4H), 2.47 (t, *J* = 4.9 Hz, 4H), 2.29 (s, 3H). ^{13}C NMR (125 MHz, DMSO-d_6) δ 150.9, 144.2, 138.0, 137.2, 129.5, 128.1, 127.6, 126.0, 117.7, 115.4, 62.2, 52.8, 50.0, 21.0. ESI-HRMS: m/z

calcd for $C_{18}H_{23}N_2O$ [M+H]$^+$: 283.1805; found: 283.1804. HPLC analysis: retention time = 10.204 min; peak area, 98.49%.

4-(4-(4-methylbenzyl)piperazin-1-yl)phenol (**AI10-m29**). White solid, yield 64%, m.p. 179.4-179.9 °C. ^{1}H NMR (400 MHz, DMSO-d_6) δ 8.82 (s, 1H), 7.19 (d, J = 8.0 Hz, 2H), 7.13 (d, J = 7.8 Hz, 2H), 6.78 – 6.72 (m, 2H), 6.65 – 6.60 (m, 2H), 3.44 (s, 2H), 2.93 (t, J = 4.9 Hz, 4H), 2.46 (t, J = 4.9 Hz, 4H), 2.28 (s, 3H). ^{13}C NMR (100 MHz, DMSO-d_6) δ 150.9, 144.3, 136.0, 135.1, 128.9, 128.8, 117.7, 115.4, 61.9, 52.8, 50.0, 20.8. ESI-HRMS: m/z calcd for $C_{18}H_{23}N_2O$ [M+H]$^+$: 283.1805; found: 283.1805. HPLC analysis: retention time = 10.936 min; peak area, 97.43%.

4-(4-(2-methoxybenzyl)piperazin-1-yl)phenol (**AI10-m30**). Pink solid, yield 64%, m.p. 70.1-70.8 °C. ^{1}H NMR (500 MHz, DMSO-d_6) δ 8.80 (s, 1H), 7.33 (d, J = 7.4 Hz, 1H), 7.26 – 7.21 (m, 1H), 6.98 (d, J = 8.3 Hz, 1H), 6.92 (t, J = 6.8 Hz, 1H), 6.76 (d, J = 9.0 Hz, 2H), 6.63 (d, J = 8.9 Hz, 2H), 3.78 (s, 3H), 3.51 (s, 2H), 2.99 – 2.91 (m, 4H), 2.54 – 2.51 (m, 4H). ^{13}C NMR (125 MHz, DMSO-d_6) δ 157.4, 150.8, 144.3, 129.8, 128.0, 125.7, 120.1, 117.7, 115.4, 110.8, 55.4, 55.3, 52.9, 50.1. ESI-HRMS: m/z calcd for $C_{18}H_{23}N_2O_2$ [M+H]$^+$: 299.1754; found: 299.1754. HPLC analysis: retention time = 10.427 min; peak area, 98.35%.

4-(4-(3-methoxybenzyl)piperazin-1-yl)phenol (**AI10-m31**). White solid, yield 60%, m.p. 158.6-159.5 °C. ^{1}H NMR (500 MHz, DMSO-d_6) δ 8.79 (s, 1H), 7.24 (t, J = 7.8 Hz, 1H), 6.91 – 6.87 (m, 2H), 6.82 (ddd, J = 8.2, 2.5, 1.1 Hz, 1H), 6.78 – 6.73 (m, 2H), 6.66 – 6.61 (m, 2H), 3.74 (s, 3H), 3.48 (s, 2H), 2.95 (t, J = 4.8 Hz, 4H), 2.48 (d, J = 4.9 Hz, 4H). ^{13}C NMR (125 MHz, DMSO-d_6) δ 159.3, 150.8, 144.2, 139.8, 129.2, 121.0, 117.7, 115.4, 114.3, 112.3, 62.0, 54.9, 52.8, 50.0. ESI-HRMS: m/z calcd for $C_{18}H_{23}N_2O_2$ [M+H]$^+$: 299.1754; found: 299.1754. HPLC analysis: retention time = 8.412 min; peak area, 99.24%.

4-(4-(2-ethoxybenzyl)piperazin-1-yl)phenol (**AI10-m32**). Pink solid, yield 55%, m.p. 158.3-158.9 °C. ^{1}H NMR (500 MHz, DMSO-d_6) δ 8.79 (s, 1H), 7.32 (d, J = 9.3 Hz, 1H), 7.20 (t, J = 8.7 Hz, 1H), 6.95 (d, J = 8.2 Hz, 1H), 6.90 (d, J = 7.4 Hz, 1H), 6.76 (d, J = 8.9 Hz, 2H), 6.63 (d, J = 8.9 Hz, 2H), 4.02 (q, J = 6.9 Hz, 2H), 3.52 (s, 2H), 2.98 – 2.91 (m, 4H), 2.56 – 2.51 (m, 4H), 1.34 (t, J = 6.9 Hz, 3H). ^{13}C NMR (125 MHz, DMSO-d_6) δ 156.7, 150.8, 144.3, 129.9, 127.9, 125.9, 120.1, 117.7, 115.4, 111.8, 63.3, 55.3, 52.8, 50.1, 14.8. ESI-HRMS: m/z calcd for $C_{19}H_{25}N_2O_2$ [M+H]$^+$: 313.1911; found: 313.1911. HPLC analysis: retention time = 7.821 min; peak area, 98.82%.

4-(4-(3-ethoxybenzyl)piperazin-1-yl)phenol (**AI10-m33**). Yellow solid, yield 71%, m.p. 161.1-161-9 °C. ^{1}H NMR (500 MHz, DMSO-d_6) δ 8.80 (s, 1H), 7.22 (t, J = 7.9 Hz, 1H), 6.90 – 6.84 (m, 2H), 6.82 – 6.71 (m, 3H), 6.63 (d, J = 8.9 Hz, 2H), 4.00 (q, J = 7.0 Hz, 2H), 3.47 (s, 2H), 2.95 (t, J = 5.0 Hz, 4H), 2.48 (s, 4H), 1.31 (t, J = 7.0 Hz, 3H). ^{13}C NMR (125 MHz, DMSO-d_6) δ 158.5, 150.8, 144.2, 139.8, 129.2, 120.9, 117.7, 115.4, 114.8, 112.8, 62.8, 62.0, 52.8, 50.0, 14.7. ESI-HRMS: m/z calcd for $C_{19}H_{25}N_2O_2$ [M+H]$^+$: 313.1911; found: 313.1911. HPLC analysis: retention time = 10.163 min; peak area, 99.30%.

4-(4-(4-ethoxybenzyl)piperazin-1-yl)phenol (**AI10-m34**). White solid, yield 53%, m.p. 170.3-171.0 °C. ^{1}H NMR (500 MHz, DMSO-d_6) δ 8.83 (s, 1H), 7.25 – 7.14 (m, 2H), 6.86 (d, J = 8.5 Hz, 2H), 6.75 (d, J = 9.0 Hz, 2H), 6.68 – 6.61 (m, 2H), 3.99 (q, J = 6.9 Hz, 2H), 3.42 (s, 2H), 2.93 (t, J = 4.7 Hz, 4H), 2.46 (t, J = 4.8 Hz, 4H), 1.31 (t, J = 7.0 Hz, 3H). ^{13}C NMR (125 MHz, DMSO-d_6) δ 157.6, 150.8, 144.2, 130.1, 129.8, 117.7, 115.4, 114.0, 62.9, 61.5, 52.7, 50.0, 14.7. ESI-HRMS: m/z calcd for $C_{19}H_{25}N_2O_2$ [M+H]$^+$: 313.1911; found: 313.1911. HPLC analysis: retention time = 6.292 min; peak area, 97.52%.

3-((4-(4-hydroxyphenyl)piperazin-1-yl)methyl)phenol (**AI10-m35**). White solid, yield 48%, m.p. 208.4-208.8 °C. ^{1}H NMR (500 MHz, DMSO-d_6) δ 9.29 (s, 1H), 8.79 (s, 1H), 7.10 (t, J = 7.7 Hz, 1H), 6.78 – 6.74 (m, 3H), 6.72 (dt, J = 7.5, 1.3 Hz, 1H), 6.66 – 6.61 (m, 3H), 3.41 (s, 2H), 2.94 (t, J = 4.8 Hz, 4H), 2.47 (t, J = 4.9 Hz, 4H). ^{13}C NMR (125 MHz, DMSO-d_6) δ 157.3, 150.8, 144.2, 139.6, 129.1, 119.5, 117.7, 115.6, 115.4, 113.9, 62.1, 52.8, 50.0. ESI-HRMS: m/z calcd for $C_{17}H_{21}N_2O_2$ [M+H]$^+$: 285.1598; found: 285.1598. HPLC analysis: retention time = 6.498 min; peak area, 99.76%.

4-(4-(4-hydroxybenzyl)piperazin-1-yl)phenol (**AI10-m36**). Brown solid, yield 23%, m.p. 210.2-210.9 °C. ^{1}H NMR (500 MHz, DMSO-d_6) δ 9.27 (s, 1H), 8.78 (s, 1H), 7.09 (d, J = 8.4 Hz, 2H), 6.75 (d, J = 2.3 Hz, 1H), 6.74 (d, J = 2.3 Hz, 1H), 6.71 (d, J = 2.1 Hz, 1H), 6.70 (d, J = 2.0 Hz, 1H), 6.63 (d, J = 2.3 Hz, 1H), 6.62 (d, J = 2.2 Hz, 1H), 3.37 (s, 2H), 2.93 (t, J = 4.8 Hz, 4H), 2.45 (t, J = 4.9 Hz, 4H). ^{13}C NMR (125 MHz, DMSO-d_6) δ 156.3, 150.8, 144.2, 130.1, 117.7, 115.4, 114.9, 61.7, 52.6, 50.0. ESI-HRMS: m/z calcd for $C_{17}H_{21}N_2O_2$ [M+H]$^+$: 285.1598; found: 285.1589. HPLC analysis: retention time = 6.205 min; peak area, 98.70%.

2-((4-(4-hydroxyphenyl)piperazin-1-yl)methyl)benzonitrile (**AI10-m37**). Pink oil, yield 62%. ^{1}H NMR (400 MHz, DMSO-d_6) δ 8.84 (s, 1H), 7.82 (dd, J = 7.8, 1.3 Hz, 1H), 7.69 (td, J = 7.6, 1.4 Hz, 1H), 7.60 (dd, J = 7.9, 1.3 Hz, 1H), 7.47 (td, J = 7.6, 1.3 Hz, 1H), 6.79 – 6.73 (m, 2H),

6.67 – 6.60 (m, 2H), 3.68 (s, 2H), 2.95 (t, *J* = 4.9 Hz, 4H), 2.54 (t, *J* = 4.9 Hz, 4H). ^{13}C NMR (100 MHz, DMSO-d_6) δ 151.0, 144.2, 142.0, 133.1, 130.2, 128.1, 117.9, 117.8, 115.4, 112.2, 59.8, 52.8, 50.0. ESI-HRMS: m/z calcd for $C_{18}H_{20}N_3O$ [M+H]$^+$: 294.1601; found: 294.1601. HPLC analysis: retention time = 10.204 min; peak area, 99.51%.

3-((4-(4-hydroxyphenyl)piperazin-1-yl)methyl)benzonitrile (**AI10-m38**). Dark green solid, yield 37%, m.p. 83.7-84.3 °C. ^{1}H NMR (500 MHz, DMSO-d_6) δ 8.81 (s, 1H), 7.75 (d, *J* = 8.4 Hz, 2H), 7.68 (d, *J* = 9.4 Hz, 1H), 7.55 (t, *J* = 7.7 Hz, 1H), 6.79 – 6.73 (m, 2H), 6.66 – 6.60 (m, 2H), 3.57 (s, 2H), 2.99 – 2.92 (m, 4H), 2.50 – 2.47 (m, 4H). ^{13}C NMR (125 MHz, DMSO-d_6) δ 150.9, 144.2, 140.0, 133.8, 132.2, 130.9, 129.5, 118.9, 117.8, 115.4, 111.2, 60.9, 52.7, 50.0. ESI-HRMS: m/z calcd for $C_{18}H_{20}N_3O$ [M+H]$^+$: 294.1601; found: 294.1601. HPLC analysis: retention time = 10.208 min; peak area, 99.60%.

4-((4-(4-hydroxyphenyl)piperazin-1-yl)methyl)benzonitrile (**AI10-m39**). Pink solid, yield 39%, m.p. 212.7-213.6 °C. ^{1}H NMR (400 MHz, DMSO-d_6) δ 8.84 (s, 1H), 7.82 – 7.78 (m, 2H), 7.56 – 7.51 (m, 2H), 6.78 – 6.73 (m, 2H), 6.66 – 6.60 (m, 2H), 3.60 (s, 2H), 2.95 (t, *J* = 4.9 Hz, 4H), 2.49 (t, *J* = 3.5 Hz, 4H). ^{13}C NMR (100 MHz, DMSO-d_6) δ 150.9, 144.4, 144.2, 132.2, 129.6, 119.0, 117.8, 115.4, 109.7, 61.4, 52.8, 50.0. ESI-HRMS: m/z calcd for $C_{18}H_{20}N_3O$ [M+H]$^+$: 294.1601; found: 294.1601. HPLC analysis: retention time = 10.141 min; peak area, 100.00%.

4-(4-(3,5-dichlorobenzyl)piperazin-1-yl)phenol (**AI10-m40**). Pink solid, yield 56%, m.p. 156.1-152.2 °C. ^{1}H NMR (500 MHz, DMSO-d_6) δ 8.79 (s, 1H), 7.49 (t, *J* = 2.0 Hz, 1H), 7.38 (d, *J* = 2.0 Hz, 2H), 6.77 – 6.75 (m, 2H), 6.65 – 6.62 (m, 2H), 3.53 (s, 2H), 2.96 (t, *J* = 4.9 Hz, 4H), 2.49 (d, *J* = 2.9 Hz, 4H). ^{13}C NMR (125 MHz, DMSO-d_6) δ 150.9, 144.1, 142.9, 133.9, 127.3, 126.6, 117.8, 115.4, 60.5, 52.6, 50.0. ESI-HRMS: m/z calcd for $C_{17}H_{19}Cl_2N_2O$ [M+H]$^+$: 337.0869; found: 337.0869. HPLC analysis: retention time = 10.227 min; peak area, 98.56%.

4-(4-(3,4-dichlorobenzyl)piperazin-1-yl)phenol (**AI10-m41**). White solid, yield 39%, m.p. 101.5-102.3 °C. ^{1}H NMR (500 MHz, DMSO-d_6) δ 8.79 (s, 1H), 7.60 – 7.56 (m, 2H), 7.32 (dd, *J* = 8.2, 2.0 Hz, 1H), 6.78 – 6.74 (m, 2H), 6.65 – 6.61 (m, 2H), 3.51 (s, 2H), 2.95 (dd, *J* = 6.2, 3.7 Hz, 4H), 2.49 (d, *J* = 5.0 Hz, 4H). ^{13}C NMR (125 MHz, DMSO-d_6) δ 150.9, 144.1, 139.7, 130.9, 130.5, 130.4, 129.4, 129.1, 117.8, 115.4, 60.4, 52.6, 50.0. ESI-HRMS: m/z calcd for $C_{17}H_{19}Cl_2N_2O$ [M+H]$^+$: 337.0869; found: 337.0869. HPLC analysis: retention time = 10.230 min; peak area, 99.06%.

4-((4-(4-hydroxyphenyl)piperazin-1-yl)methyl)benzene-1,3-diol (**AI10-m42**). Yellow oil, yield 23%. ^{1}H NMR (500 MHz, DMSO-d_6) δ 6.87 – 6.84 (m, 1H), 6.79 – 6.75 (m, 2H), 6.66 – 6.62 (m, 2H), 6.17 (d, J = 7.2 Hz, 2H), 3.54 (s, 2H), 2.96 (t, J = 4.9 Hz, 4H), 2.55 (t, J = 4.8 Hz, 4H). ^{13}C NMR (125 MHz, DMSO-d_6) δ 157.7, 157.6, 151.0, 144.0, 130.0, 117.9, 115.4, 112.7, 106.0, 102.6, 58.4, 52.2, 50.1. ESI-HRMS: m/z calcd for $C_{17}H_{21}N_2O_3$ $[M+H]^+$: 301.1547; found: 301.1547. HPLC analysis: retention time = 10.248 min; peak area, 99.85%.

4-(4-(2,6-dimethoxybenzyl)piperazin-1-yl)phenol (**AI10-m43**). White solid, yield 48%, m.p. 231.3-231.6 °C. ^{1}H NMR (500 MHz, DMSO-d_6) δ 8.76 (s, 1H), 7.23 (t, J = 8.3 Hz, 1H), 6.74 – 6.71 (m, 2H), 6.66 – 6.60 (m, 4H), 3.76 (s, 6H), 3.55 (s, 2H), 2.87 (t, J = 4.9 Hz, 4H), 2.50 (dd, J = 4.4, 2.5 Hz, 4H). ^{13}C NMR (125 MHz, DMSO-d_6) δ 158.9, 150.7, 144.4, 128.8, 117.7, 115.6, 115.4, 104.1, 55.7, 52.6, 50.1, 48.7. ESI-HRMS: m/z calcd for $C_{19}H_{25}N_2O_3$ $[M+H]^+$: 329.1860; found: 329.1863. HPLC analysis: retention time = 10.452 min; peak area, 98.02%.

4-(4-(pyridin-3-ylmethyl)piperazin-1-yl)phenol (**AI10-m44**). Yellow solid, yield 39%, m.p. 195.4-196.2 °C. ^{1}H NMR (500 MHz, DMSO-d_6) δ 8.81 (s, 1H), 8.51 (s, 1H), 8.47 (d, J = 6.4 Hz, 1H), 7.73 (d, J = 7.8 Hz, 1H), 7.39 – 7.34 (m, 1H), 6.76 (d, J = 8.9 Hz, 2H), 6.63 (d, J = 8.9 Hz, 2H), 3.54 (s, 2H), 2.98 – 2.92 (m, 4H), 2.50 – 2.47 (m, 4H). ^{13}C NMR (125 MHz, DMSO-d_6) δ 150.9, 150.1, 148.3, 144.1, 136.6, 133.5, 123.4, 117.7, 115.4, 59.1, 52.7, 50.0. ESI-HRMS: m/z calcd for $C_{16}H_{20}N_3O$ $[M+H]^+$: 270.1601; found: 270.1597. HPLC analysis: retention time = 10.227 min; peak area, 98.95%.

4-(4-(furan-2-ylmethyl)piperazin-1-yl)phenol (**AI10-m45**). Brown solid, yield 37%, m.p. 191.1-191.3 °C. ^{1}H NMR (500 MHz, DMSO-d_6) δ 8.79 (s, 1H), 7.59 (dd, J = 1.8, 0.9 Hz, 1H), 6.77 – 6.73 (m, 2H), 6.65 – 6.61 (m, 2H), 6.40 (dd, J = 3.2, 1.8 Hz, 1H), 6.30 (d, J = 3.1 Hz, 1H), 3.53 (s, 2H), 2.93 (dd, J = 6.3, 3.6 Hz, 4H), 2.49 (d, J = 1.8 Hz, 4H). ^{13}C NMR (125 MHz, DMSO-d_6) δ 151.7, 150.9, 144.2, 142.4, 117.7, 115.4, 110.3, 108.7, 53.9, 52.4, 49.9. ESI-HRMS: m/z calcd for $C_{15}H_{19}N_2O_2$ $[M+H]^+$: 259.1441; found: 259.1441. HPLC analysis: retention time = 10.174 min; peak area, 99.59%.

4-(4-(thiophen-2-ylmethyl)piperazin-1-yl)phenol (**AI10-m46**). White solid, yield 56%, m.p. 185.7-185.9 °C. ^{1}H NMR (500 MHz, DMSO-d_6) δ 8.79 (s, 1H), 7.43 (dd, J = 4.7, 1.7 Hz, 1H), 7.01 – 6.95 (m, 2H), 6.78 – 6.72 (m, 2H), 6.66 – 6.61 (m, 2H), 3.72 (s, 2H), 2.97 – 2.93 (m, 4H), 2.52 (t, J = 4.9 Hz, 4H). ^{13}C NMR (125 MHz, DMSO-d_6) δ 150.9, 144.2, 141.6, 126.5, 126.1,

125.5, 117.8, 115.4, 56.3, 52.5, 50.0. ESI-HRMS: m/z calcd for $C_{15}H_{19}N_2OS$ $[M+H]^+$: 275.1213; found: 275.1212. HPLC analysis: retention time = 10.228 min; peak area, 99.32%.

4-(4-(3-methylbenzyl)piperazin-1-yl)benzene-1,3-diol (**AI10-m47**). Colorless oil, yield 75%. ^{1}H NMR (400 MHz, DMSO-d_6) δ 8.88 (s, 1H), 8.55 (s, 1H), 7.20 (t, J = 7.5 Hz, 1H), 7.15 – 7.01 (m, 3H), 6.73 (d, J = 8.5 Hz, 1H), 6.25 (d, J = 2.7 Hz, 1H), 6.13 (dd, J = 8.5, 2.7 Hz, 1H), 3.45 (s, 2H), 2.83 (d, J = 41.6 Hz, 4H), 2.48 (s, 4H), 2.29 (s, 3H). ^{13}C NMR (100 MHz, DMSO-d_6) δ 153.9, 151.4, 138.2, 137.2, 132.0, 129.6, 128.1, 127.6, 126.1, 120.0, 105.6, 102.9, 62.3, 53.0, 51.2, 21.1. ESI-HRMS: m/z calcd for $C_{19}H_{23}N_2O_2$ $[M+H]^+$: 299.1754; found: 299.1754. HPLC analysis: retention time = 10.182 min; peak area, 98.69%.

(E)-4-(4-(3-(m-tolyl)allyl)piperazin-1-yl)phenol (**AI10-m48**). White solid, yield 50%, m.p. 187.5-188.0 °C. ^{1}H NMR (500 MHz, DMSO-d_6) δ 8.79 (s, 1H), 7.33 (d, J = 8.2 Hz, 2H), 7.13 (d, J = 7.8 Hz, 2H), 6.78 – 6.74 (m, 2H), 6.65 – 6.62 (m, 2H), 6.51 (d, J = 15.9 Hz, 1H), 6.25 (dt, J = 15.9, 6.7 Hz, 1H), 3.12 (dd, J = 6.7, 1.4 Hz, 2H), 2.96 (t, J = 4.9 Hz, 4H), 2.53 (t, J = 4.9 Hz, 4H), 2.28 (s, 3H). ^{13}C NMR (125 MHz, DMSO-d_6) δ 150.8, 144.2, 136.7, 133.9, 132.1, 129.2, 126.1, 125.9, 117.7, 115.4, 60.3, 52.8, 50.0, 20.8. ESI-HRMS: m/z calcd for $C_{20}H_{25}N_2O$ $[M+H]^+$: 309.1961; found: 309.1961. HPLC analysis: retention time = 6.567 min; peak area, 99.83%.

(E)-4-(4-(3-(4-chlorophenyl)allyl)piperazin-1-yl)phenol (**AI10-m49**). White solid, yield 48%, m.p. 191.0-191.5 °C. ^{1}H NMR (400 MHz, DMSO-d_6) δ 8.83 (s, 1H), 7.52 – 7.45 (m, 2H), 7.41 – 7.34 (m, 2H), 6.81 – 6.73 (m, 2H), 6.67 – 6.61 (m, 2H), 6.55 (d, J = 15.9 Hz, 1H), 6.36 (dt, J = 15.9, 6.5 Hz, 1H), 3.15 – 3.09 (m, 2H), 2.96 (t, J = 4.8 Hz, 4H), 2.56 – 2.51 (m, 4H). ^{13}C NMR (100 MHz, DMSO-d_6) δ 150.9, 144.2, 135.6, 131.8, 130.8, 128.6, 128.2, 128.0, 117.7, 115.4, 60.2, 52.9, 50.0. ESI-HRMS: m/z calcd for $C_{19}H_{22}ClN_2O$ $[M+H]^+$: 329.1415; found: 329.1415. HPLC analysis: retention time = 10.844 min; peak area, 98.07%.

4-(4-(4-fluorophenethyl)piperazin-1-yl)phenol (**AI10-m50**). Brown solid, yield 32%, m.p. 185.0-185.7 °C. ^{1}H NMR (500 MHz, DMSO-d_6) δ 8.79 (s, 1H), 7.27 (dd, J = 8.5, 5.7 Hz, 2H), 7.09 (t, J = 8.9 Hz, 2H), 6.77 (d, J = 9.0 Hz, 2H), 6.64 (d, J = 8.9 Hz, 2H), 2.99 – 2.92 (m, 4H), 2.75 (dd, J = 9.2, 6.4 Hz, 2H), 2.57 – 2.54 (m, 4H), 2.52 (d, J = 7.6 Hz, 2H). ^{13}C NMR (125 MHz, DMSO-d_6) δ 161.6 (d, ^1J = 240 Hz), 150.8, 144.2, 136.6 (d, ^4J = 3.75 Hz), 130.4 (d, ^3J = 7.5 Hz), 117.6, 115.4, 114.9 (d, ^2J = 21.25 Hz), 59.7, 52.8, 50.0, 31.8. ESI-HRMS: m/z calcd for $C_{18}H_{22}FN_2O$ $[M+H]^+$: 301.1711; found: 301.1714. HPLC analysis: retention time = 10.074 min; peak area, 100.00%.

(4-(4-hydroxyphenyl)piperidin-1-yl)(pyridin-4-yl)methanone (**AI10-m51**). White solid, yield 35%, m.p. 190.6-191.3 °C. ^{1}H NMR (500 MHz, DMSO-d_6) δ 9.18 (s, 1H), 8.70 – 8.64 (m, 2H), 7.45 – 7.40 (m, 2H), 7.06 (d, J = 8.5 Hz, 2H), 6.69 (d, J = 8.5 Hz, 2H), 4.60 (d, J = 13.0 Hz, 1H), 3.49 (d, J = 13.5 Hz, 1H), 3.18 – 3.10 (m, 1H), 2.83 (td, J = 13.0, 2.9 Hz, 1H), 2.69 (t, J = 3.6 Hz, 1H), 1.82 (d, J = 13.1 Hz, 1H), 1.65 (d, J = 12.9 Hz, 1H), 1.55 (t, J = 12.5 Hz, 2H). ^{13}C NMR (125 MHz, DMSO-d_6) δ 166.6, 155.7, 150.0, 144.0, 135.7, 127.6, 121.0, 115.1, 47.5, 41.9, 40.9, 33.4, 32.9. ESI-HRMS: m/z calcd for $C_{17}H_{19}N_2O_2$ $[M+H]^+$: 283.1441; found: 283.1442. HPLC analysis: retention time = 6.213 min; peak area, 98.66%.

(E)-3-(2,4-dihydroxyphenyl)-1-(4-(4-hydroxyphenyl)piperidin-1-yl)prop-2-en-1-one (**AI10-m52**). Yellow solid, yield 61%, m.p. 98.6-99.3 °C. ^{1}H NMR (500 MHz, DMSO-d_6) δ 9.86 (s, 1H), 9.65 (s, 1H), 9.15 (s, 1H), 7.69 (d, J = 15.4 Hz, 1H), 7.48 (d, J = 8.6 Hz, 1H), 7.08 – 6.92 (m, 3H), 6.71 – 6.63 (m, 2H), 6.33 (d, J = 2.3 Hz, 1H), 6.24 (dd, J = 8.5, 2.4 Hz, 1H), 4.61 (s, 1H), 4.30 (s, 1H), 3.11 (s, 1H), 2.74 – 2.57 (m, 2H), 1.76 (s, 2H), 1.44 (s, 2H). ^{13}C NMR (125 MHz, DMSO-d_6) δ 165.3, 159.9, 157.7, 155.6, 137.3, 136.0, 129.5, 127.5, 115.1, 113.7, 113.2, 107.4, 102.5, 41.2. ESI-HRMS: m/z calcd for $C_{20}H_{21}NNaO_4$ $[M+Na]^+$: 362.1363; found: 362.1363. HPLC analysis: retention time = 10.246 min; peak area, 100.00%.

4-(1-(3-methylbenzyl)piperidin-4-yl)phenol (**AI10-m53**). White solid, yield 61%, m.p. 143.2-143.9 °C. ^{1}H NMR (500 MHz, DMSO-d_6) δ 8.21 (s, 1H), 7.23 (t, J = 7.5 Hz, 1H), 7.17 – 7.12 (m, 2H), 7.09 (d, J = 7.6 Hz, 1H), 7.01 (dd, J = 9.1, 2.6 Hz, 2H), 6.67 (dd, J = 9.0, 2.5 Hz, 2H), 3.58 (s, 2H), 3.00 – 2.93 (m, 2H), 2.41 (tt, J = 11.8, 4.0 Hz, 1H), 2.30 (s, 3H), 2.17 (td, J = 11.7, 2.6 Hz, 2H), 1.74 – 1.67 (m, 2H), 1.62 (qd, J = 12.3, 3.7 Hz, 2H). ^{13}C NMR (125 MHz, DMSO-d_6) δ 155.6, 137.3, 136.9, 136.1, 129.9, 128.1, 127.9, 127.4, 126.4, 115.1, 61.8, 53.3, 40.5, 32.7, 21.0. ESI-HRMS: m/z calcd for $C_{19}H_{24}NO$ $[M+H]^+$: 282.1852; found: 282.1855. HPLC analysis: retention time = 6.698 min; peak area, 99.33%.

4-(1-(4-methylbenzyl)piperidin-4-yl)phenol (**AI10-m54**). White solid, yield 78%, m.p. 96.9-97.5 °C. ^{1}H NMR (500 MHz, DMSO-d_6) δ 9.12 (s, 1H), 7.19 (d, J = 7.6 Hz, 2H), 7.12 (d, J = 7.7 Hz, 2H), 7.03 – 6.98 (m, 2H), 6.69 – 6.63 (m, 2H), 3.44 (s, 2H), 2.88 (d, J = 11.0 Hz, 2H), 2.41 – 2.32 (m, 1H), 2.28 (s, 3H), 2.00 (s, 2H), 1.66 (d, J = 12.6 Hz, 2H), 1.56 (qd, J = 12.4, 3.7 Hz, 2H). ^{13}C NMR (125 MHz, DMSO-d_6) δ 155.5, 136.3, 136.2, 134.2, 129.2, 128.8, 127.4, 115.0, 61.7, 54.9, 53.3, 40.6, 32.9, 20.7. ESI-HRMS: m/z calcd for $C_{19}H_{24}NO$ $[M+H]^+$: 282.1852; found: 282.1852. HPLC analysis: retention time = 10.197 min; peak area, 98.16%.

4-(1-(3,4-dichlorobenzyl)piperidin-4-yl)phenol (**AI10-m55**). White solid, yield 23%, m.p. 95.2-96.0 °C. ^{1}H NMR (500 MHz, DMSO-d_6) δ 9.12 (s, 1H), 7.57 (d, J = 8.2 Hz, 1H), 7.55 (d, J = 1.9 Hz, 1H), 7.31 (dd, J = 8.2, 2.0 Hz, 1H), 7.03 – 6.99 (m, 2H), 6.69 – 6.65 (m, 2H), 3.47 (s, 2H), 2.85 (dt, J = 8.9, 3.2 Hz, 2H), 2.35 (tt, J = 11.9, 3.8 Hz, 1H), 2.02 (td, J = 11.5, 2.4 Hz, 2H), 1.70 – 1.63 (m, 2H), 1.57 (qd, J = 12.3, 3.7 Hz, 2H). ^{13}C NMR (125 MHz, DMSO-d_6) δ 155.5, 140.1, 136.4, 130.8, 130.4, 130.3, 129.3, 129.0, 127.4, 115.0, 60.9, 53.6, 40.9, 33.4. ESI-HRMS: m/z calcd for $C_{18}H_{20}Cl_2NO$ [M+H]$^+$: 336.0916; found: 336.0916. HPLC analysis: retention time = 10.255 min; peak area, 100.00%.

4-(1-(thiophen-2-ylmethyl)piperidin-4-yl)phenol (**AI10-m56**). White solid, yield 33%, m.p. 160.5-161.1 °C. ^{1}H NMR (500 MHz, DMSO-d_6) δ 9.13 (s, 1H), 7.43 – 7.39 (m, 1H), 7.04 – 6.99 (m, 2H), 6.98 – 6.94 (m, 2H), 6.70 – 6.64 (m, 2H), 3.69 (s, 2H), 2.93 (d, J = 11.7 Hz, 2H), 2.35 (tt, J = 12.0, 3.8 Hz, 1H), 2.08 – 1.97 (m, 2H), 1.68 (d, J = 14.2 Hz, 2H), 1.57 (qd, J = 12.3, 3.7 Hz, 2H). ^{13}C NMR (125 MHz, DMSO-d_6) δ 155.5, 142.2, 136.4, 127.4, 126.5, 125.9, 125.3, 115.0, 56.7, 53.4, 40.9, 33.3. ESI-HRMS: m/z calcd for $C_{16}H_{20}NOS$ [M+H]$^+$: 274.1260; found: 274.1262. HPLC analysis: retention time = 7.853 min; peak area, 98.28%.

4-((4-(4-hydroxyphenyl)piperidin-1-yl)methyl)benzene-1,3-diol (**AI10-m57**). Orange solid, yield 24%, m.p. 190.9-191.7 °C. ^{1}H NMR (500 MHz, DMSO-d_6) δ 7.05 – 6.99 (m, 2H), 6.82 (d, J = 8.8 Hz, 1H), 6.70 – 6.64 (m, 2H), 6.18 – 6.14 (m, 2H), 3.54 (s, 2H), 2.96 (dt, J = 11.7, 3.0 Hz, 2H), 2.42 (tt, J = 12.0, 3.7 Hz, 1H), 2.13 – 2.03 (m, 2H), 1.73 (d, J = 12.4 Hz, 2H), 1.55 (qd, J = 12.6, 3.8 Hz, 2H). ^{13}C NMR (125 MHz, DMSO-d_6) δ 158.1, 157.6, 155.5, 136.1, 129.6, 127.4, 115.1, 112.8, 105.8, 102.6, 59.3, 53.1, 40.7, 33.3. ESI-HRMS: m/z calcd for $C_{18}H_{22}NO_3$ [M+H]$^+$: 300.1594; found: 300.1598. HPLC analysis: retention time = 10.264 min; peak area, 98.98%.

(2,6-dimethylpyridin-4-yl)(4-(4-hydroxybenzyl)piperazin-1-yl)methanone (**AI10-a1**). White solid, yield 26%, m.p. 123.7-123.9 °C. ^{1}H NMR (400 MHz, DMSO-d_6) δ 9.31 (s, 1H), 7.11 – 7.03 (m, 2H), 6.99 (s, 2H), 6.72 – 6.66 (m, 2H), 3.61 – 3.53 (m, 2H), 3.36 (s, 2H), 3.22 (t, J = 4.8 Hz, 2H), 2.43 (s, 6H), 2.36 (d, J = 4.9 Hz, 2H), 2.28 (d, J = 5.3 Hz, 2H). ^{13}C NMR (100 MHz, DMSO-d_6) δ 167.1, 157.8, 156.4, 144.2, 130.2, 127.7, 117.3, 114.9, 61.4, 52.6, 52.0, 46.9, 41.3, 24.0. ESI-HRMS: m/z calcd for $C_{19}H_{24}N_3O_2$ [M+H]$^+$: 326.1863; found: 326.1868. HPLC analysis: retention time = 9.016 min; peak area, 98.62%.

(4-(2,4-dihydroxybenzyl)piperazin-1-yl)(2,6-dimethylpyridin-4-yl)methanone (**AI10-a2**). Yellow solid, yield 59%, m.p. 127.0-127.5 °C. ^{1}H NMR (500 MHz, DMSO-d_6) δ 9.70 (s, 1H),

9.15 (s, 1H), 7.00 (s, 2H), 6.86 (d, *J* = 8.2 Hz, 1H), 6.24 – 6.10 (m, 2H), 3.61 (s, 2H), 3.39 – 3.22 (m, 6H), 2.44 (s, 6H), 2.39 – 2.33 (m, 2H). ^{13}C NMR (125 MHz, DMSO-d_6) δ 167.1, 157.8, 157.6, 157.3, 144.1, 130.5, 117.3, 112.8, 106.1, 102.5, 57.3, 52.2, 51.7, 46.7, 41.2, 24.0. ESI-HRMS: m/z calcd for $C_{19}H_{24}N_3O_3$ [M+H]$^+$: 342.1812; found: 342.1817. HPLC analysis: retention time = 6.806 min; peak area, 98.77%.

(4-(4-hydroxyphenyl)piperazin-1-yl)(2-methylpyridin-4-yl)methanone (**AI10-a3**). Yellow solid, yield 35%, m.p. 212.2-212.4 °C. ^{1}H NMR (400 MHz, DMSO-d_6) δ 8.92 (d, *J* = 4.4 Hz, 1H), 8.53 (d, *J* = 5.0 Hz, 1H), 7.28 (s, 1H), 7.20 (dd, *J* = 5.0, 1.5 Hz, 1H), 6.83 – 6.77 (m, 2H), 6.66 (dd, *J* = 8.8, 1.9 Hz, 2H), 3.74 (t, *J* = 5.1 Hz, 2H), 3.38 (d, *J* = 4.6 Hz, 2H), 3.02 (t, *J* = 5.2 Hz, 2H), 2.91 (t, *J* = 5.1 Hz, 2H), 2.51 (s, 3H). ^{13}C NMR (100 MHz, DMSO-d_6) δ 166.9, 158.6, 151.5, 149.4, 143.8, 143.7, 120.4, 118.6, 118.3, 115.5, 50.5, 50.2, 46.9, 41.4, 24.1. ESI-HRMS: m/z calcd for $C_{17}H_{20}N_3O_2$ [M+H]$^+$: 298.1550; found: 298.1546. HPLC analysis: retention time = 8.996 min; peak area, 100.00%.

(4-(4-hydroxyphenyl)piperazin-1-yl)(5-methylpyridin-3-yl)methanone (**AI10-a4**). White solid, yield 58%, m.p. 196.9-197.8 °C. ^{1}H NMR (500 MHz, DMSO-d_6) δ 8.91 (s, 1H), 8.50 (d, *J* = 2.1 Hz, 1H), 8.43 (d, *J* = 2.1 Hz, 1H), 7.68 (q, *J* = 1.8 Hz, 1H), 6.83 – 6.78 (m, 2H), 6.69 – 6.64 (m, 2H), 3.76 (s, 2H), 3.45 (s, 2H), 2.98 (d, *J* = 41.1 Hz, 4H), 2.34 (s, 3H). ^{13}C NMR (125 MHz, DMSO-d_6) δ 166.9, 151.5, 150.8, 144.8, 143.8, 135.0, 133.1, 131.3, 118.6, 115.5, 17.8. ESI-HRMS: m/z calcd for $C_{17}H_{20}N_3O_2$ [M+H]$^+$: 298.1550; found: 298.1551. HPLC analysis: retention time = 7.048 min; peak area, 99.56%.

(4-(4-hydroxyphenyl)piperazin-1-yl)(6-methylpyridin-2-yl)methanone (**AI10-a5**). Yellow solid, yield 58%, m.p. 86.1-86.9 °C. ^{1}H NMR (500 MHz, DMSO-d_6) δ 8.90 (s, 1H), 7.80 (t, *J* = 7.7 Hz, 1H), 7.35 (dd, *J* = 14.1, 7.7 Hz, 2H), 6.82 – 6.79 (m, 2H), 6.69 – 6.64 (m, 2H), 3.77 (t, *J* = 5.1 Hz, 2H), 3.53 – 3.47 (m, 2H), 3.03 (t, *J* = 5.2 Hz, 2H), 2.92 (t, *J* = 5.0 Hz, 2H), 2.50 (s, 3H). ^{13}C NMR (125 MHz, DMSO-d_6) δ 166.8, 157.1, 153.4, 151.4, 143.9, 137.5, 123.9, 120.0, 118.5, 115.5, 50.7, 50.3, 46.7, 41.6, 24.0. ESI-HRMS: m/z calcd for $C_{17}H_{20}N_3O_2$ [M+H]$^+$: 298.1550; found: 298.1549. HPLC analysis: retention time = 6.241 min; peak area, 99.66%.

(4-(4-hydroxyphenyl)piperazin-1-yl)(2-methylpyridin-3-yl)methanone (**AI10-a6**). Brown solid, yield 58%, m.p. 205.7-206.3 °C. ^{1}H NMR (500 MHz, DMSO-d_6) δ 8.90 (s, 1H), 8.50 (dd, *J* = 4.9, 1.7 Hz, 1H), 7.64 (dd, *J* = 7.6, 1.8 Hz, 1H), 7.29 (dd, *J* = 7.6, 4.9 Hz, 1H), 6.83 – 6.77 (m, 2H), 6.69 – 6.63 (m, 2H), 3.79 (s, 2H), 3.26 (t, *J* = 5.1 Hz, 2H), 3.03 (s, 2H), 2.88 (s, 2H), 2.42 (s,

3H). ^{13}C NMR (125 MHz, DMSO-d_6) δ 167.1, 153.7, 151.5, 149.2, 143.8, 134.0, 131.3, 121.1, 118.6, 115.5, 50.7, 50.3, 46.5, 41.1, 22.0. ESI-HRMS: m/z calcd for $C_{17}H_{20}N_3O_2$ $[M+H]^+$: 298.1550; found: 298.1551. HPLC analysis: retention time = 6.196 min; peak area, 99.68%.

(4-hydroxy-2-methylphenyl)(4-(4-hydroxyphenyl)piperazin-1-yl)methanone (**AI10-a7**). Colorless oil, yield 28%. ^{1}H NMR (500 MHz, DMSO-d_6) δ 9.57 (s, 1H), 8.89 (s, 1H), 6.98 (d, J = 8.2 Hz, 1H), 6.82 – 6.76 (m, 2H), 6.66 (d, J = 2.3 Hz, 1H), 6.64 (d, J = 2.3 Hz, 2H), 6.61 (dd, J = 8.2, 2.5 Hz, 1H), 3.74 (s, 2H), 3.27 (s, 2H), 2.98 (s, 2H), 2.84 (s, 2H), 2.14 (s, 3H). ^{13}C NMR (125 MHz, DMSO-d_6) δ 169.0, 157.5, 151.4, 143.9, 135.7, 127.4, 127.0, 118.5, 116.8, 115.5, 112.5, 50.8, 18.9. ESI-HRMS: m/z calcd for $C_{18}H_{21}N_2O_3$ $[M+H]^+$: 313.1547; found: 313.1545. HPLC analysis: retention time = 10.109 min; peak area, 100.00%.

(2-hydroxy-3-methylphenyl)(4-(4-hydroxyphenyl)piperazin-1-yl)methanone (**AI10-a8**). Brown oil, yield 50%, m.p. 190.5-190.8°C. ^{1}H NMR (400 MHz, DMSO-d_6) δ 9.02 (d, J = 2.0 Hz, 1H), 8.91 (s, 1H), 7.19 – 7.10 (m, 1H), 7.00 (dd, J = 7.6, 1.8 Hz, 1H), 6.85 – 6.75 (m, 3H), 6.70 – 6.62 (m, 2H), 3.55 (s, 4H), 2.95 (t, J = 4.8 Hz, 4H), 2.19 (s, 3H). ^{13}C NMR (100 MHz, DMSO-d_6) δ 167.7, 151.8, 151.4, 143.9, 131.6, 125.6, 123.5, 119.4, 118.5, 115.5, 50.4, 16.4. ESI-HRMS: m/z calcd for $C_{18}H_{21}N_2O_3$ $[M+H]^+$: 313.1547; found: 313.1546. HPLC analysis: retention time = 7.724 min; peak area, 99.70%.

1-(4-((4-(4-hydroxyphenyl)piperazin-1-yl)methyl)phenyl)ethan-1-one (**AI10-a9**). Yellow solid, yield 50%, m.p. 169.4-169.9°C. ^{1}H NMR (500 MHz, DMSO-d_6) δ 8.80 (s, 1H), 7.99 – 7.88 (m, 2H), 7.47 (d, J = 8.1 Hz, 2H), 6.78 – 6.73 (m, 2H), 6.66 – 6.61 (m, 2H), 3.58 (s, 2H), 2.95 (t, J = 4.9 Hz, 4H), 2.57 (s, 3H), 2.49 (d, J = 4.3 Hz, 4H). ^{13}C NMR (125 MHz, DMSO-d_6) δ 197.6, 150.9, 144.2, 143.9, 135.7, 129.0, 128.2, 117.8, 115.4, 61.6, 52.8, 50.0, 26.7. ESI-HRMS: m/z calcd for $C_{19}H_{23}N_2O_2$ $[M+H]^+$: 311.1754; found: 311.1753. ESI-HRMS: m/z calcd for $C_{19}H_{23}N_2O_2$ $[M+H]^+$: 311.1754; found: 311.1753. HPLC analysis: retention time = 9.192 min; peak area, 98.48%.

4-((4-(4-hydroxyphenyl)piperazin-1-yl)methyl)-2,6-dimethylphenol (**AI10-a10**). White solid, yield 14%, m.p. 200.8-201.4°C. ^{1}H NMR (400 MHz, DMSO-d_6) δ 8.81 (s, 1H), 8.10 (s, 1H), 6.83 (s, 2H), 6.77 – 6.71 (m, 2H), 6.66 – 6.59 (m, 2H), 3.31 (s, 2H), 2.92 (t, J = 4.7 Hz, 4H), 2.44 (t, J = 4.9 Hz, 4H), 2.14 (s, 6H). ^{13}C NMR (100 MHz, DMSO-d_6) δ 152.2, 150.8, 144.3, 129.1, 123.8, 117.7, 115.4, 61.9, 52.8, 50.0, 16.7. ESI-HRMS: m/z calcd for $C_{19}H_{25}N_2O_2$ $[M+H]^+$: 313.1911; found: 313.1917. HPLC analysis: retention time = 10.220 min; peak area, 98.74%.

(4-(4-hydroxyphenyl)piperazin-1-yl)(1-methyl-1H-indazol-6-yl)methanone (**AI10-a11**). Orange oil, yield 21%. ^{1}H NMR (500 MHz, DMSO-d_6) δ 8.89 (s, 1H), 8.11 (d, J = 0.9 Hz, 1H), 7.82 (dd, J = 8.2, 0.9 Hz, 1H), 7.74 (q, J = 1.1 Hz, 1H), 7.15 (dd, J = 8.2, 1.3 Hz, 1H), 6.83 – 6.78 (m, 2H), 6.68 – 6.64 (m, 2H), 4.08 (s, 3H), 3.63 (d, J = 157.4 Hz, 4H), 2.99 (d, J = 42.9 Hz, 4H). ^{13}C NMR (125 MHz, DMSO-d_6) δ 169.2, 151.4, 143.9, 139.0, 133.6, 132.5, 123.7, 121.0, 119.2, 118.5, 115.5, 108.6, 50.4, 35.5. ESI-HRMS: m/z calcd for $C_{19}H_{21}N_4O_2$ $[M+H]^+$: 337.1659; found: 337.1659. HPLC analysis: retention time = 10.540 min; peak area, 99.27%.

(4-(4-hydroxyphenyl)piperazin-1-yl)(4-methylthiazol-2-yl)methanone (**AI10-a12**). Yellow solid, yield 80%, m.p. 176.8-177.5℃. ^{1}H NMR (400 MHz, DMSO-d_6) δ 8.92 (s, 1H), 7.59 (d, J = 1.1 Hz, 1H), 6.86 – 6.79 (m, 2H), 6.69 – 6.63 (m, 2H), 4.43 (t, J = 4.9 Hz, 2H), 3.78 (d, J = 5.1 Hz, 2H), 3.03 (t, J = 5.1 Hz, 4H), 2.44 (s, 3H). ^{13}C NMR (100 MHz, DMSO-d_6) δ 163.6, 158.4, 153.1, 151.5, 143.8, 120.0, 118.5, 115.5, 50.7, 50.4, 45.9, 43.0, 17.1. ESI-HRMS: m/z calcd for $C_{15}H_{18}N_3O_2S$ $[M+H]^+$: 304.1114; found: 304.1114. HPLC analysis: retention time = 9.554 min; peak area, 99.16%.

(4-(4-hydroxyphenyl)piperazin-1-yl)(1-methyl-1H-indol-2-yl)methanone (**AI10-a13**). Orange solid, yield 75%, m.p. 192.1-192.8℃. ^{1}H NMR (400 MHz, DMSO-d_6) δ 8.94 (s, 1H), 7.61 (d, J = 7.9 Hz, 1H), 7.52 (d, J = 8.3 Hz, 1H), 7.25 (ddd, J = 8.2, 6.9, 1.2 Hz, 1H), 7.10 (t, J = 7.4 Hz, 1H), 6.85 – 6.79 (m, 2H), 6.70 – 6.64 (m, 3H), 3.77 (s, 7H), 3.01 (s, 4H). ^{13}C NMR (125 MHz, DMSO-d_6) δ 162.0, 151.5, 143.9, 137.3, 132.1, 126.0, 122.9, 121.2, 120.0, 118.6, 115.5, 110.4, 102.7, 55.0, 50.7, 30.9. ESI-HRMS: m/z calcd for $C_{20}H_{22}N_3O_2$ $[M+H]^+$: 336.1707; found: 336.1706. HPLC analysis: retention time = 10.783 min; peak area, 99.19%.

4-((4-(4-hydroxyphenyl)piperazin-1-yl)methyl)-3,5-dimethylphenol (**AI10-a14**). Colorless oil, yield 10%. ^{1}H NMR (500 MHz, DMSO-d_6) δ 9.03 (s, 1H), 8.78 (s, 1H), 6.76 – 6.71 (m, 2H), 6.65 – 6.60 (m, 2H), 6.40 (s, 2H), 3.36 (s, 2H), 2.87 (d, J = 5.6 Hz, 4H), 2.47 (t, J = 4.9 Hz, 4H), 2.25 (s, 6H). ^{13}C NMR (125 MHz, DMSO-d_6) δ 155.7, 150.8, 144.3, 138.8, 125.0, 117.8, 115.4, 114.7, 55.0, 52.5, 50.3, 20.0. ESI-HRMS: m/z calcd for $C_{19}H_{25}N_2O_2$ $[M+H]^+$: 313.1911; found: 313.1912. HPLC analysis: retention time = 7.221 min; peak area, 98.88%.

4-((4-(4-hydroxyphenyl)piperazin-1-yl)methyl)-3-methylphenol (**AI10-a15**). Colorless oil, yield 17%. ^{1}H NMR (500 MHz, DMSO-d_6) δ 9.14 (s, 1H), 8.78 (s, 1H), 6.98 (d, J = 8.1 Hz, 1H), 6.77 – 6.73 (m, 2H), 6.64 – 6.60 (m, 2H), 6.57 (d, J = 2.6 Hz, 1H), 6.51 (dd, J = 8.1, 2.6 Hz, 1H), 3.38 (s, 2H), 2.94 – 2.87 (m, 4H), 2.45 (t, J = 4.8 Hz, 4H), 2.24 (s, 3H). ^{13}C NMR (125 MHz,

DMSO-d_6) δ 156.3, 150.8, 144.3, 138.4, 131.0, 126.5, 117.7, 117.0, 115.4, 111.9, 59.9, 52.8, 50.1, 19.0. ESI-HRMS: m/z calcd for $C_{18}H_{23}N_2O_2$ [M+H]$^+$: 299.1754; found: 299.1756. HPLC analysis: retention time = 8.815 min; peak area, 99.65%.

4-((4-(4-hydroxyphenyl)piperazin-1-yl)methyl)-2-methylphenol (**AI10-a16**). Reddish brown solid, yield 43%, m.p. 119.3-119.9°C. ^{1}H NMR (500 MHz, DMSO-d_6) δ 9.14 (s, 1H), 8.78 (s, 1H), 6.98 (d, J = 2.1 Hz, 1H), 6.90 (dd, J = 8.1, 2.2 Hz, 1H), 6.77 – 6.73 (m, 2H), 6.71 (d, J = 8.1 Hz, 1H), 6.65 – 6.61 (m, 2H), 3.34 (s, 2H), 2.96 – 2.89 (m, 4H), 2.48 – 2.41 (m, 4H), 2.10 (s, 3H). ^{13}C NMR (125 MHz, DMSO-d_6) δ 154.4, 150.8, 144.3, 131.4, 127.9, 127.4, 123.3, 117.7, 115.4, 114.2, 61.8, 52.7, 50.0, 16.0. ESI-HRMS: m/z calcd for $C_{18}H_{23}N_2O_2$ [M+H]$^+$: 299.1754; found: 299.1759. HPLC analysis: retention time = 10.178 min; peak area, 99.55%.

(2-chloro-6-methylpyridin-3-yl)(4-(3-hydroxyphenyl)piperazin-1-yl)methanone (**AI10-a17**). Brown oil, yield 89%. ^{1}H NMR (500 MHz, DMSO-d_6) δ 9.19 (s, 1H), 7.80 (d, J = 7.6 Hz, 1H), 7.38 (d, J = 7.7 Hz, 1H), 6.99 (t, J = 8.1 Hz, 1H), 6.38 (dd, J = 7.9, 2.3 Hz, 1H), 6.32 (t, J = 2.3 Hz, 1H), 6.25 (dd, J = 7.7, 2.2 Hz, 1H), 3.76 (t, J = 5.2 Hz, 2H), 3.31 – 3.26 (m, 2H), 3.17 (q, J = 4.7 Hz, 2H), 3.05 (q, J = 4.5 Hz, 2H), 2.50 (s, 3H). ^{13}C NMR (125 MHz, DMSO-d_6) δ 164.3, 159.7, 158.1, 152.0, 144.8, 137.8, 129.6, 128.9, 122.8, 107.1, 106.8, 103.1, 48.6, 48.2, 46.1, 41.1, 23.5. ESI-HRMS: m/z calcd for $C_{17}H_{19}ClN_3O_2$ [M+H]$^+$: 332.1160; found: 332.1158. HPLC analysis: retention time = 10.728 min; peak area, 99.19%.

2-(4-hydroxyphenyl)-1-(4-(3-hydroxyphenyl)piperazin-1-yl)propan-1-one (**AI10-a18**). Brown oil, yield 61%. ^{1}H NMR (500 MHz, DMSO-d_6) δ 9.26 (s, 1H), 9.13 (s, 1H), 7.05 (d, J = 8.5 Hz, 2H), 6.95 (t, J = 8.1 Hz, 1H), 6.72 – 6.65 (m, 2H), 6.31 (dd, J = 8.0, 2.3 Hz, 1H), 6.25 – 6.17 (m, 2H), 4.00 (q, J = 6.7 Hz, 1H), 3.60 (dt, J = 56.4, 11.9 Hz, 4H), 3.22 – 2.64 (m, 4H), 1.24 (d, J = 6.8 Hz, 3H). ^{13}C NMR (125 MHz, DMSO-d_6) δ 171.7, 158.1, 155.9, 152.1, 132.5, 129.6, 128.1, 115.4, 106.9, 106.5, 102.7, 48.4, 48.2, 44.8, 41.2, 40.5, 20.8. ESI-HRMS: m/z calcd for $C_{19}H_{23}N_2O_3$ [M+H]$^+$: 327.1703; found: 327.1701. HPLC analysis: retention time = 10.499 min; peak area, 99.37%.

(4-hydroxyphenyl)(4-(p-tolyl)piperazin-1-yl)methanone (**AI10-a19**). White solid, yield 68%, m.p. 210.4-211.3°C. ^{1}H NMR (500 MHz, DMSO-d_6) δ 9.85 (s, 1H), 7.34 – 7.26 (m, 2H), 7.08 – 6.99 (m, 2H), 6.89 – 6.83 (m, 2H), 6.83 – 6.78 (m, 2H), 3.61 (s, 4H), 3.08 (t, J = 5.0 Hz, 4H), 2.20 (s, 3H). ^{13}C NMR (125 MHz, DMSO-d_6) δ 169.3, 158.8, 148.7, 129.4, 129.2, 128.2, 126.0, 116.2,

114.9, 49.1, 20.0. ESI-HRMS: m/z calcd for $C_{18}H_{21}N_2O_2$ $[M+H]^+$: 297.1598; found: 297.1599. HPLC analysis: retention time = 6.710 min; peak area, 98.05%.

(3,4-dihydroxyphenyl)(4-(p-tolyl)piperazin-1-yl)methanone (**AI10-a20**). Grayish-green solid, yield 37%, m.p. 143.0-143.4°C. ^{1}H NMR (400 MHz, DMSO-d_6) δ 9.37 (s, 1H), 9.23 (s, 1H), 7.03 (d, J = 8.2 Hz, 2H), 6.90 – 6.81 (m, 3H), 6.78 – 6.71 (m, 2H), 3.61 (s, 4H), 3.06 (t, J = 5.3 Hz, 4H), 2.20 (s, 3H). ^{13}C NMR (100 MHz, DMSO-d_6) δ 169.3, 148.8, 147.1, 145.0, 129.4, 128.3, 126.3, 119.0, 116.2, 115.1, 115.1, 49.2, 20.1. ESI-HRMS: m/z calcd for $C_{18}H_{21}N_2O_3$ $[M+H]^+$: 313.1547; found: 313.1547. HPLC analysis: retention time = 11.018 min; peak area, 99.31%.

(2,5-dihydroxyphenyl)(4-(p-tolyl)piperazin-1-yl)methanone (**AI10-a21**). reddish brown solid, yield 74%, m.p. 113.2-113.8°C. ^{1}H NMR (500 MHz, DMSO-d_6) δ 9.03 (s, 1H), 8.89 (s, 1H), 7.06 – 7.00 (m, 2H), 6.88 – 6.82 (m, 2H), 6.69 (d, J = 8.7 Hz, 1H), 6.64 (dd, J = 8.7, 2.9 Hz, 1H), 6.51 (d, J = 2.9 Hz, 1H), 3.67 (s, 4H), 3.06 (s, 4H), 2.20 (s, 3H). ^{13}C NMR (125 MHz, DMSO-d_6) δ 166.9, 149.8, 148.7, 145.6, 129.4, 128.2, 124.0, 116.9, 116.5, 116.2, 114.0, 49.1, 20.0. ESI-HRMS: m/z calcd for $C_{18}H_{21}N_2O_3$ $[M+H]^+$: 313.1547; found: 313.1546. HPLC analysis: retention time = 10.857 min; peak area, 99.92%.

(3,5-dihydroxyphenyl)(4-(p-tolyl)piperazin-1-yl)methanone (**AI10-a22**). Yellow solid, yield 68%, m.p. 212.5-212.8°C. ^{1}H NMR (500 MHz, DMSO-d_6) δ 9.50 (s, 2H), 7.06 – 6.98 (m, 2H), 6.88 – 6.82 (m, 2H), 6.28 (t, J = 2.2 Hz, 1H), 6.21 (d, J = 2.2 Hz, 2H), 3.57 (d, J = 103.8 Hz, 4H), 3.06 (s, 4H), 2.20 (s, 3H). ^{13}C NMR (125 MHz, DMSO-d_6) δ 169.0, 158.4, 148.7, 137.6, 129.4, 128.3, 116.2, 104.7, 103.4, 54.9, 20.0. ESI-HRMS: m/z calcd for $C_{18}H_{21}N_2O_3$ $[M+H]^+$: 313.1547; found: 313.1545. HPLC analysis: retention time = 6.378 min; peak area, 99.42%.

(3-fluoro-5-hydroxyphenyl)(4-(p-tolyl)piperazin-1-yl)methanone (**AI10-a23**). White solid, yield 88%, m.p. 194.5-195.0°C. ^{1}H NMR (500 MHz, DMSO-d_6) δ 10.23 (s, 1H), 7.04 (d, J = 8.3 Hz, 2H), 6.89 – 6.81 (m, 2H), 6.69 – 6.61 (m, 3H), 3.71 (s, 2H), 3.44 (s, 2H), 3.08 (d, J = 36.1 Hz, 4H), 2.20 (s, 3H). ^{13}C NMR (125 MHz, DMSO-d_6) δ 167.6 (d, ^4J = 2.5 Hz), 163.6 (d, ^1J = 242.5 Hz), 159.1 (d, ^3J = 11.25 Hz), 148.7, 138.5 (d, ^3J = 8.75 Hz), 129.4, 128.3, 116.3, 110.1, 104.3 (d, ^2J = 22.25 Hz), 103.6 (d, ^2J = 22.25 Hz), 49.0, 20.1. ESI-HRMS: m/z calcd for $C_{18}H_{20}FN_2O_2$ $[M+H]^+$: 315.1503; found: 315.1504. HPLC analysis: retention time = 6.795 min; peak area, 98.96%.

(2-chloro-5-hydroxyphenyl)(4-(p-tolyl)piperazin-1-yl)methanone (**AI10-a24**). White solid, yield 32%, m.p. 214.6-215.4°C. ^{1}H NMR (500 MHz, DMSO-d_6) δ 9.97 (s, 1H), 7.30 (d, J = 8.7

Hz, 1H), 7.07 – 7.01 (m, 2H), 6.88 – 6.82 (m, 3H), 6.71 (d, *J* = 2.9 Hz, 1H), 3.75 (t, *J* = 5.3 Hz, 2H), 3.27 (t, *J* = 5.1 Hz, 2H), 3.14 (dt, *J* = 12.5, 6.2 Hz, 2H), 3.02 (tq, *J* = 11.7, 6.0, 5.1 Hz, 2H), 2.20 (s, 3H). ^{13}C NMR (125 MHz, DMSO-d_6) δ 165.4, 156.6, 148.6, 136.4, 130.3, 129.4, 128.4, 118.3, 117.4, 116.3, 114.3, 49.2, 48.9, 46.0, 40.9, 20.0. ESI-HRMS: m/z calcd for $C_{18}H_{20}ClN_2O_2$ $[M+H]^+$: 331.1208; found: 331.1207. HPLC analysis: retention time = 5.350 min; peak area, 98.71%.

3-(4-hydroxyphenyl)-1-(4-(p-tolyl)piperazin-1-yl)propan-1-one (**AI10-a25**). White solid, yield 77%, m.p. 150.8-151.4°C. ^{1}H NMR (500 MHz, DMSO-d_6) δ 9.12 (s, 1H), 7.02 (dd, *J* = 8.6, 2.7 Hz, 4H), 6.85 – 6.81 (m, 2H), 6.68 – 6.63 (m, 2H), 3.55 (dt, *J* = 26.9, 5.1 Hz, 4H), 2.98 (q, *J* = 5.3 Hz, 4H), 2.71 (t, *J* = 7.7 Hz, 2H), 2.61 – 2.56 (m, 2H), 2.20 (s, 3H). ^{13}C NMR (125 MHz, DMSO-d_6) δ 170.1, 155.5, 148.8, 131.3, 129.4, 129.3, 128.2, 116.2, 115.0, 49.2, 48.9, 44.8, 40.9, 34.3, 30.0, 20.0. ESI-HRMS: m/z calcd for $C_{20}H_{25}N_2O_2$ $[M+H]^+$: 325.1911; found: 325.1911. HPLC analysis: retention time = 6.687 min; peak area, 99.32%.

(4-(4-chlorophenyl)piperazin-1-yl)(3-hydroxy-5-methylphenyl)methanone (**AI10-a26**). Yellow solid, yield 83%, m.p. 157.7-158.4°C. ^{1}H NMR (500 MHz, DMSO-d_6) δ 9.58 (s, 1H), 7.24 (d, *J* = 9.0 Hz, 2H), 6.99 – 6.93 (m, 2H), 6.68 – 6.61 (m, 2H), 6.57 (d, *J* = 2.0 Hz, 1H), 3.59 (d, *J* = 118.7 Hz, 4H), 3.15 (s, 4H), 2.25 (s, 3H). ^{13}C NMR (125 MHz, DMSO-d_6) δ 169.5, 157.7, 150.0, 139.7, 137.3, 129.2, 123.3, 118.5, 117.8, 117.5, 111.4, 48.8, 21.4. ESI-HRMS: m/z calcd for $C_{18}H_{20}ClN_2O_2$ $[M+H]^+$: 331.1208; found: 331.1207. HPLC analysis: retention time = 6.735 min; peak area, 98.67%.

(4-(4-chlorophenyl)piperazin-1-yl)(5-hydroxy-2-methylphenyl)methanone (**AI10-a27**). Yellow oil, yield 80%. ^{1}H NMR (400 MHz, DMSO-d_6) δ 9.46 (s, 1H), 7.27 – 7.20 (m, 2H), 7.05 (d, *J* = 8.3 Hz, 1H), 6.98 – 6.93 (m, 2H), 6.71 (dd, *J* = 8.3, 2.6 Hz, 1H), 6.55 (d, *J* = 2.6 Hz, 1H), 3.76 (t, *J* = 5.2 Hz, 2H), 3.27 (t, *J* = 5.2 Hz, 2H), 3.20 (t, *J* = 5.3 Hz, 2H), 3.09 – 2.99 (m, 2H), 2.09 (s, 3H). ^{13}C NMR (100 MHz, DMSO-d_6) δ 168.5, 155.3, 149.6, 137.0, 131.3, 128.7, 123.4, 123.0, 117.5, 115.7, 112.4, 48.7, 48.4, 45.9, 40.6, 17.7. ESI-HRMS: m/z calcd for $C_{18}H_{20}ClN_2O_2$ $[M+H]^+$: 331.1208; found: 331.1208. HPLC analysis: retention time = 9.189 min; peak area, 98.36%.

(2,6-dimethylpyridin-4-yl)(6-hydroxy-3,4-dihydroisoquinolin-2(1H) yl)methanone (**AI10-a28**). Yellow solid, yield 54%, m.p. 183.9-184.4°C. ^{1}H NMR (500 MHz, DMSO-d_6) δ 9.27 (s, 1H), 7.08 – 7.00 (m, 3H), 6.67 – 6.50 (m, 2H), 4.63 (s, 1H), 4.37 – 4.33 (m, 1H), 3.77 (t, *J* = 6.0 Hz,

1H), 3.43 (t, J = 5.8 Hz, 1H), 2.75 (dt, J = 32.0, 5.9 Hz, 2H), 2.45 (d, J = 4.2 Hz, 6H). ESI-HRMS: m/z calcd for $C_{17}H_{19}N_2O_2$ $[M+H]^+$: 283.1441; found: 283.1442. HPLC analysis: retention time = 10.220 min; peak area, 98.12%.

4-(4-(4-hydroxy-3,5-dimethylbenzoyl)piperazin-1-yl)benzonitrile (**AI10-a29**). Yellow solid, yield 26%, m.p. 229.0-229.8°C. ^{1}H NMR (400 MHz, DMSO-d_6) δ 8.71 (s, 1H), 7.63 – 7.57 (m, 2H), 7.05 – 6.99 (m, 4H), 3.61 (s, 4H), 3.39 (s, 4H), 2.18 (s, 6H). ^{13}C NMR (100 MHz, DMSO-d_6) δ 169.6, 154.8, 153.0, 133.4, 127.8, 126.0, 124.0, 120.0, 114.2, 98.6, 46.4, 16.6. ESI-HRMS: m/z calcd for $C_{20}H_{22}N_3O_2$ $[M+H]^+$: 336.1707; found:336.1710. HPLC analysis: retention time = 6.274 min; peak area, 98.15%.

(4-(4-hydroxybenzoyl)piperazin-1-yl)(2-methylquinolin-4-yl)methanone (**AI10-a30**). Brown solid, yield 74%, m.p. 189.8-190.4°C. ^{1}H NMR (400 MHz, DMSO-d_6) δ 9.91 (s, 1H), 7.98 (d, J = 9.2 Hz, 1H), 7.80 – 7.73 (m, 2H), 7.59 (td, J = 7.4, 6.9, 1.3 Hz, 1H), 7.41 (s, 1H), 7.28 (d, J = 8.1 Hz, 2H), 6.78 (d, J = 8.1 Hz, 2H), 3.82 (d, J = 14.5 Hz, 2H), 3.66 (s, 2H), 3.43 (s, 2H), 3.17 (s, 2H), 2.68 (s, 3H). ^{13}C NMR (100 MHz, DMSO-d_6) δ 169.6, 166.1, 159.0, 158.8, 147.4, 142.1, 130.1, 129.5, 128.8, 126.7, 125.6, 124.7, 122.1, 118.9, 115.0, 46.5, 24.9. ESI-HRMS: m/z calcd for $C_{22}H_{22}N_3O_3$ $[M+H]^+$: 376.1656; found: 376.1656. HPLC analysis: retention time = 9.631 min; peak area, 98.24%.

(4-(4-hydroxybenzoyl)piperazin-1-yl)(m-tolyl)methanone (**AI10-a31**). White solid, yield 59%, m.p. 208.0-208.4°C. ^{1}H NMR (400 MHz, DMSO-d_6) δ 9.91 (s, 1H), 7.33 (t, J = 7.5 Hz, 1H), 7.30 – 7.25 (m, 3H), 7.24 – 7.17 (m, 2H), 6.83 – 6.75 (m, 2H), 3.58 (d, J = 43.3 Hz, 8H), 2.33 (s, 3H). ^{13}C NMR (125 MHz, DMSO-d_6) δ 169.5, 169.3, 158.9, 137.9, 135.7, 130.3, 129.5, 128.3, 127.6, 125.7, 124.1, 115.0, 48.7, 47.2, 20.9. ESI-HRMS: m/z calcd for $C_{19}H_{21}N_2O_3$ $[M+H]^+$: 325.1547; found: 325.1546. HPLC analysis: retention time = 10.405 min; peak area, 99.81%.

1-(4-(4-hydroxybenzoyl)piperazin-1-yl)-3-(m-tolyl)propan-1-one (**AI10-a32**). Colorless oil, yield 79%. ^{1}H NMR (500 MHz, DMSO-d_6) δ 9.86 (s, 1H), 7.31 – 7.24 (m, 2H), 7.15 (t, J = 7.5 Hz, 1H), 7.05 (d, J = 2.0 Hz, 1H), 7.02 (d, J = 7.7 Hz, 1H), 6.98 (d, J = 7.5 Hz, 1H), 6.85 – 6.77 (m, 2H), 3.52 – 3.38 (m, 8H), 2.78 (t, J = 7.8 Hz, 2H), 2.61 (t, J = 7.8 Hz, 2H), 2.26 (s, 3H). ^{13}C NMR (125 MHz, DMSO-d_6) δ 170.2, 169.5, 158.8, 141.2, 137.2, 129.3, 129.1, 128.1, 126.5, 125.8, 125.4, 114.9, 44.8, 41.0, 34.0, 30.7, 21.0. ESI-HRMS: m/z calcd for $C_{21}H_{25}N_2O_3$ $[M+H]^+$: 353.1860; found: 353.1859. HPLC analysis: retention time = 11.011 min; peak area, 99.61%.

NMR spectra of target compounds

(2-hydroxyphenyl)(4-(p-tolyl)piperazin-1-yl)methanone **(AI1)**

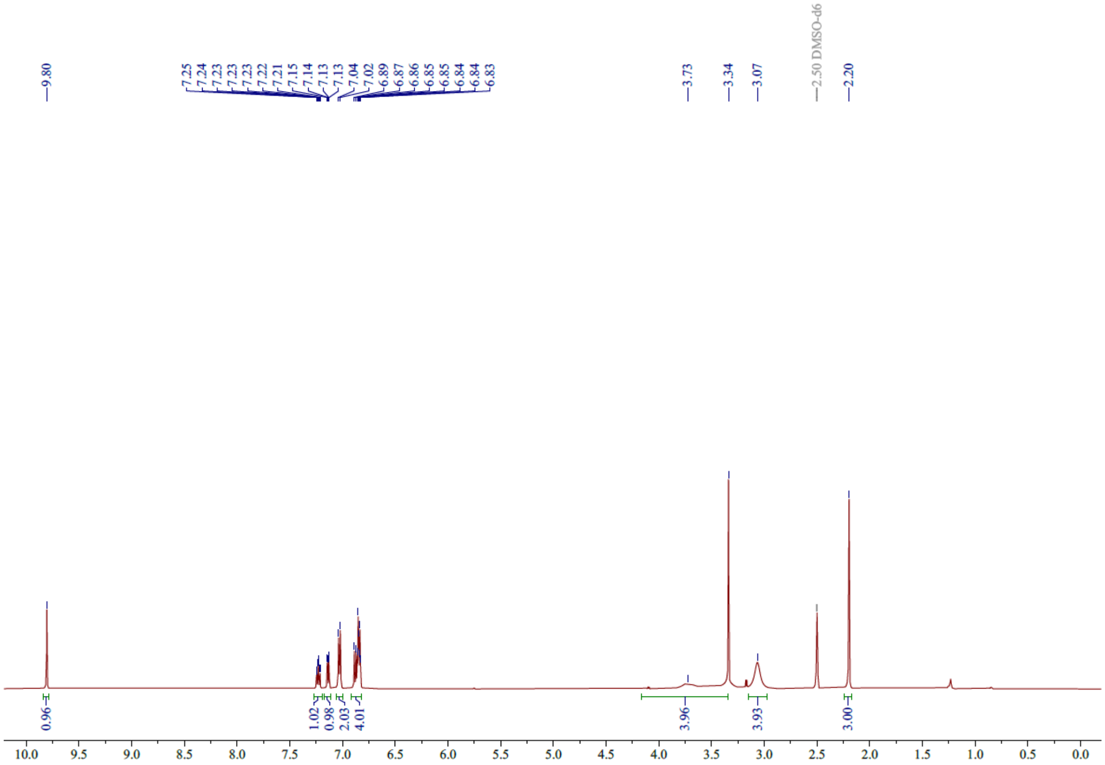

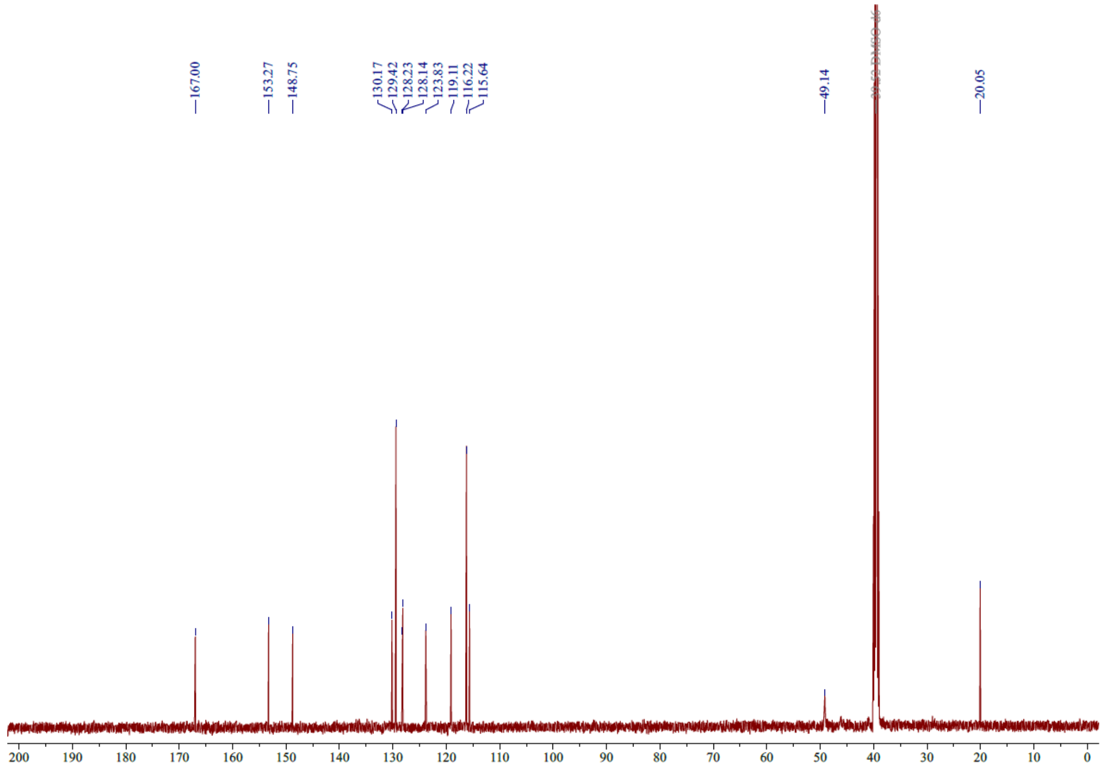

(4-hydroxyphenyl)(4-(o-tolyl)piperazin-1-yl)methanone **(AI2)**

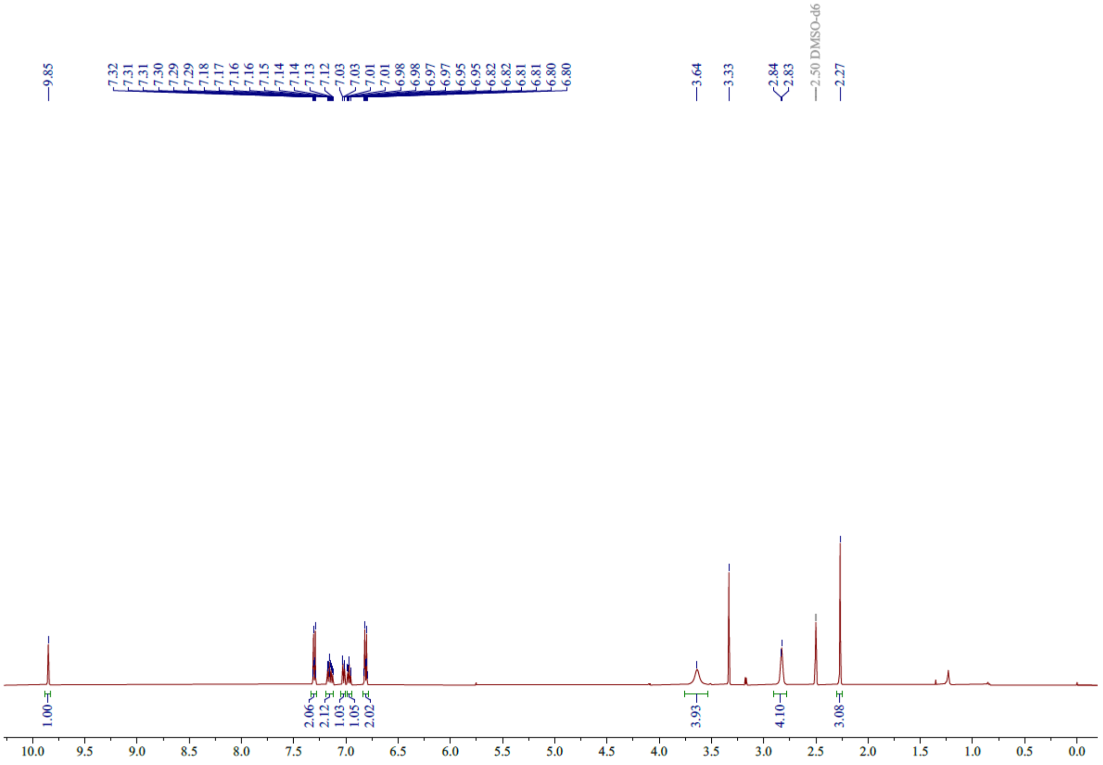

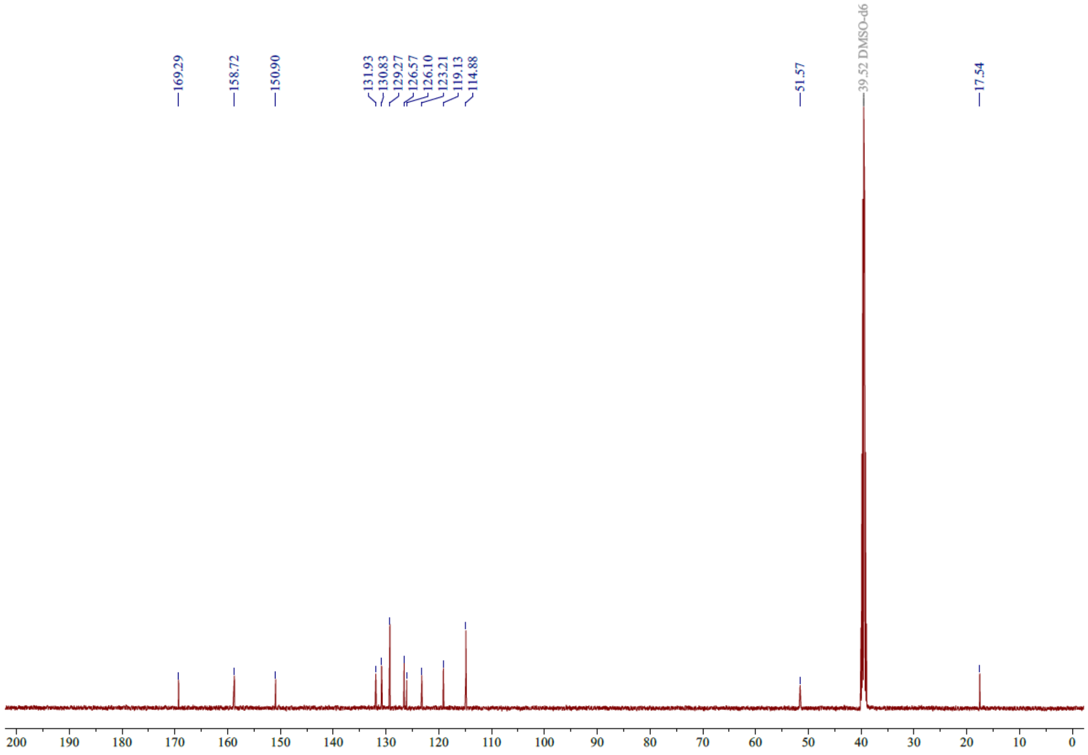

(2-hydroxyphenyl)(4-(m-tolyl)piperazin-1-yl)methanone **(AI3)**

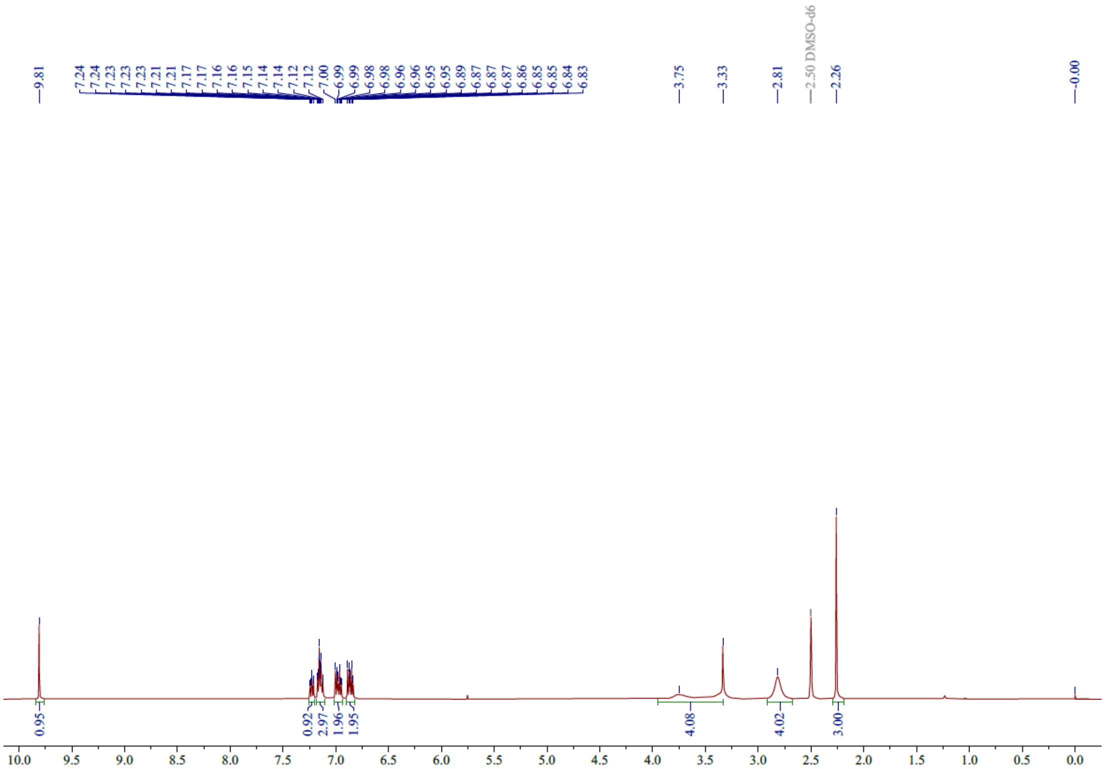

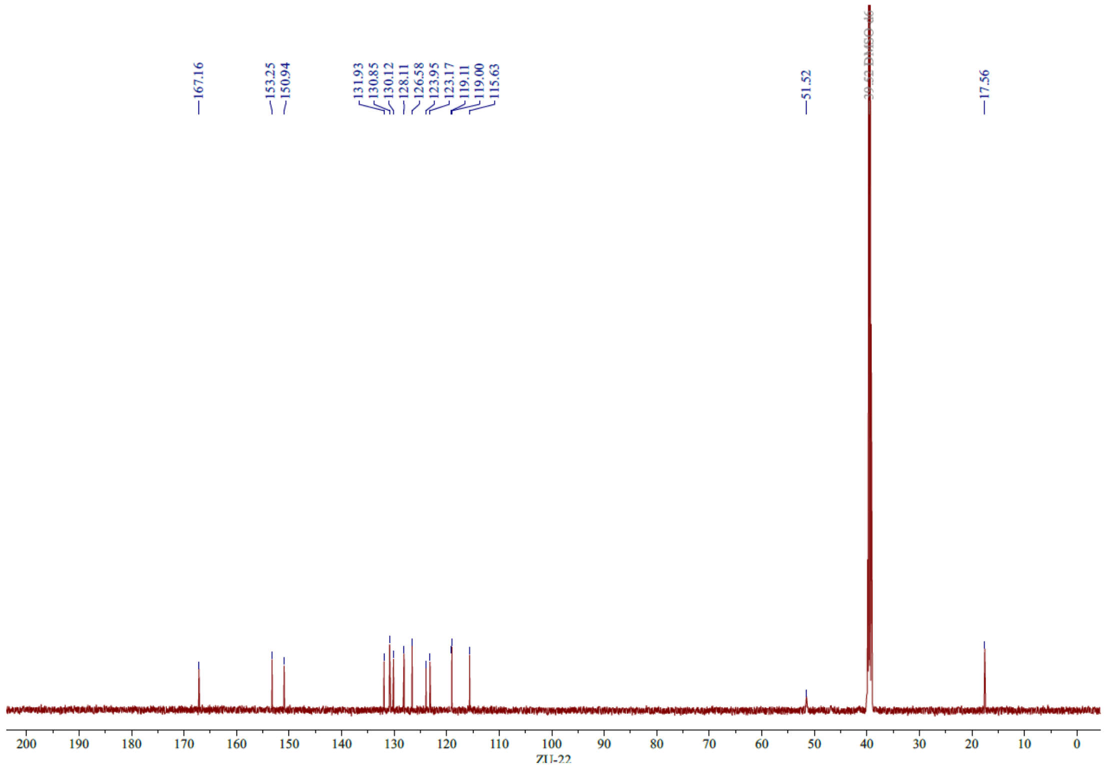

(2-ethylphenyl)(4-(4-hydroxyphenyl)piperazin-1-yl)methanone **(AI4)**

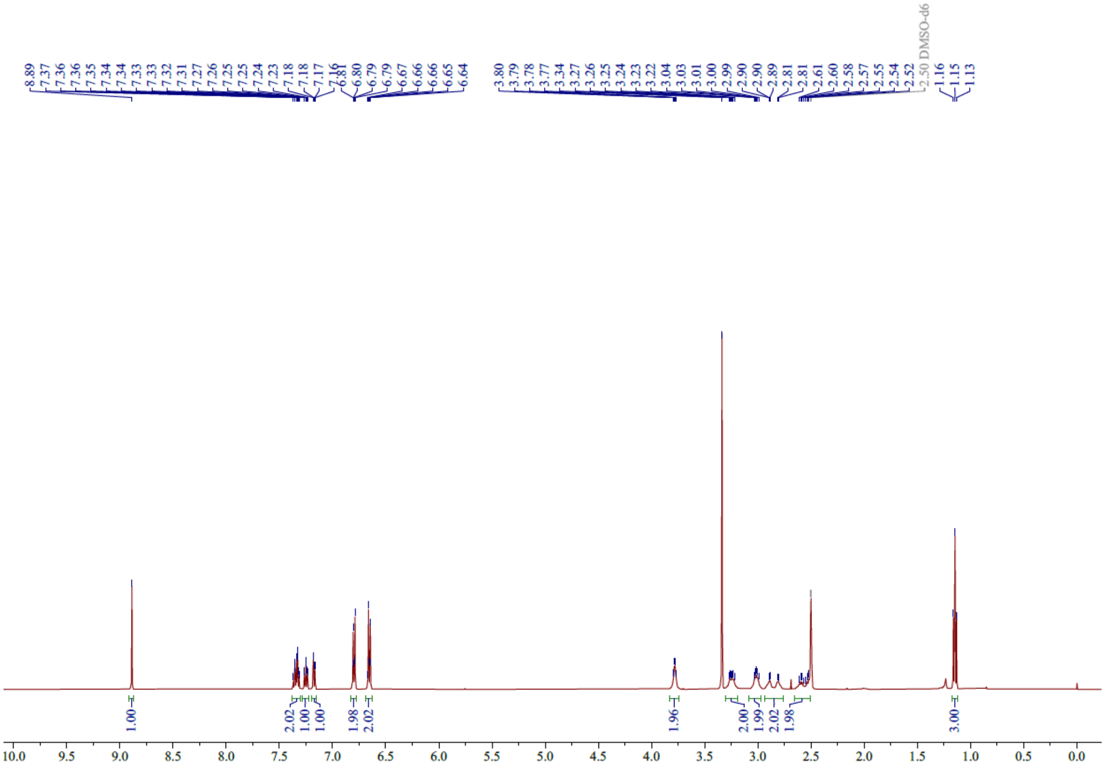

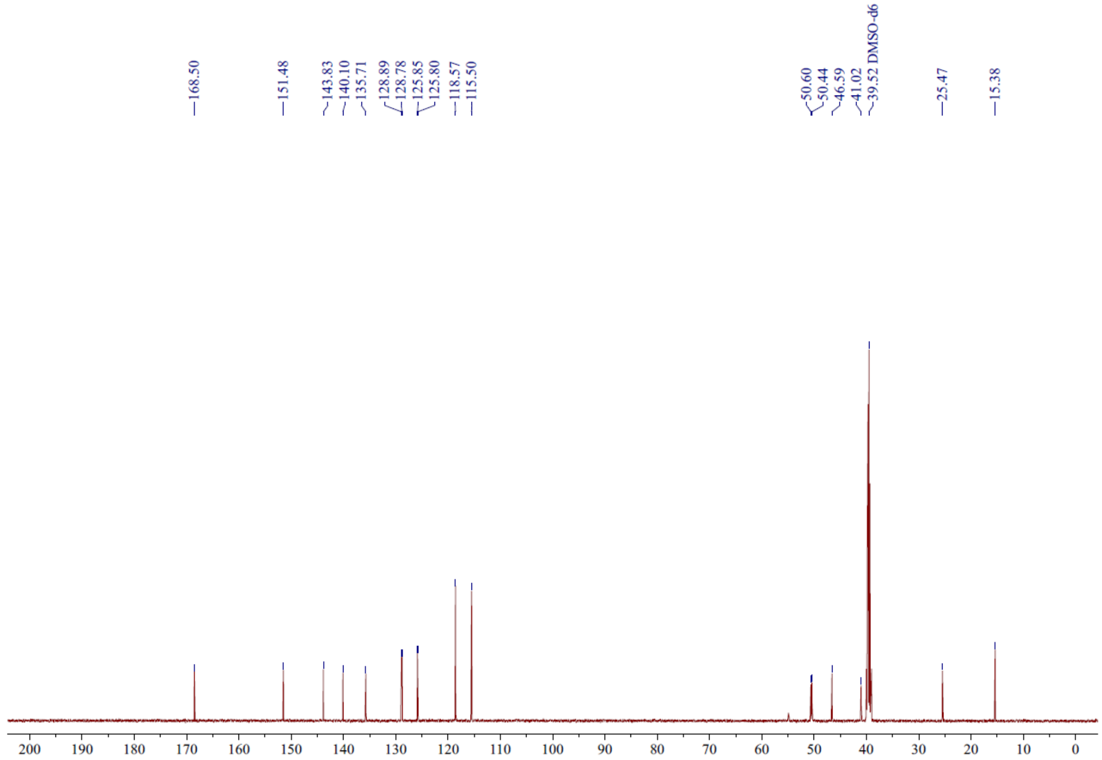

(4-(4-chlorophenyl)piperazin-1-yl)(2-hydroxy-6-methylphenyl)methanone **(AI5)**

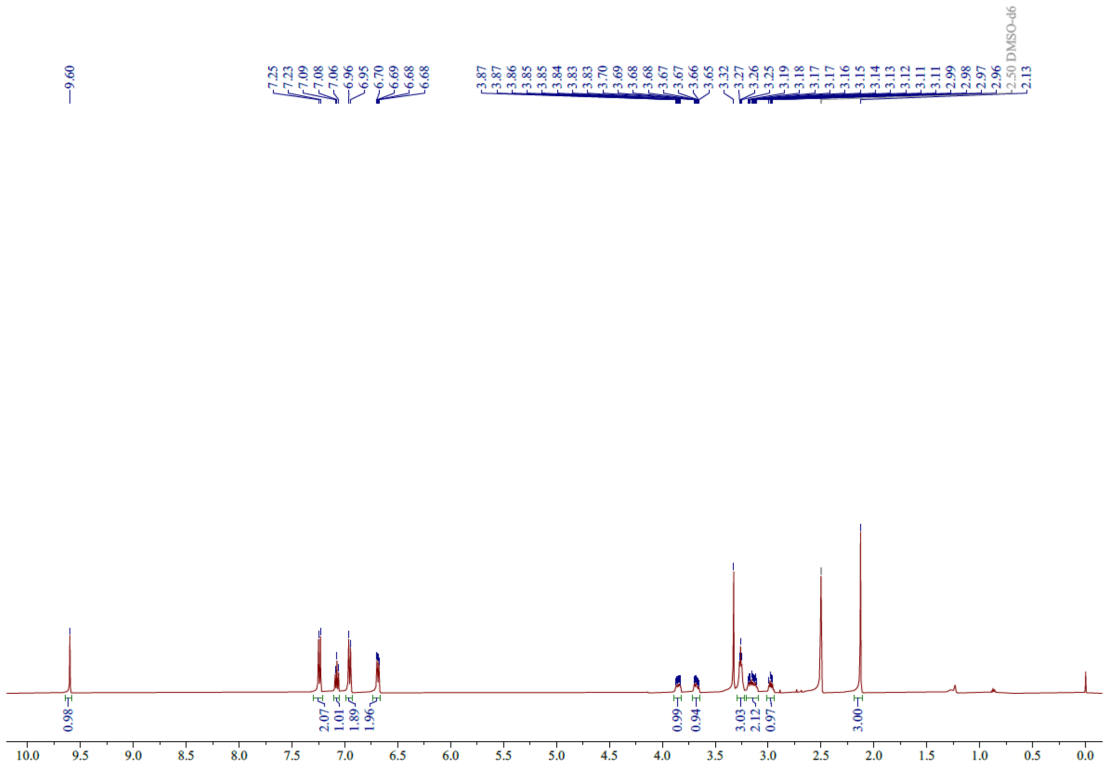

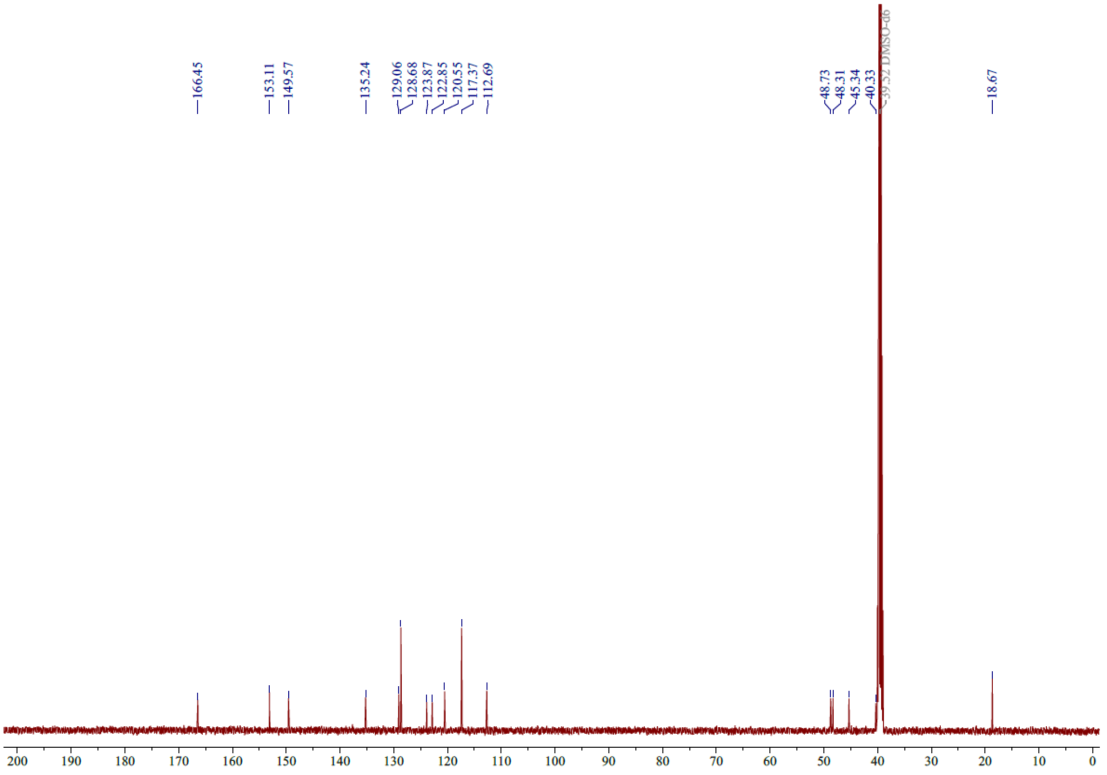

***(4-(2-chlorophenyl)piperazin-1-yl)(2-hydroxy-5-methylphenyl)methanone* (AI6)**

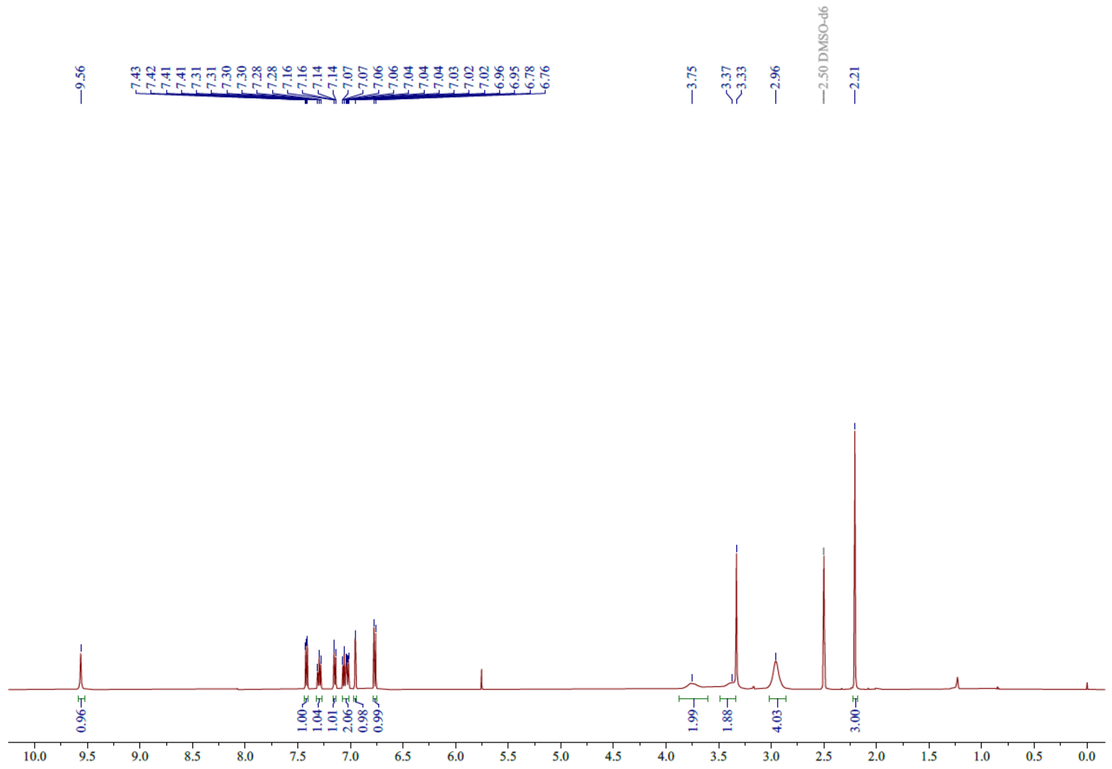

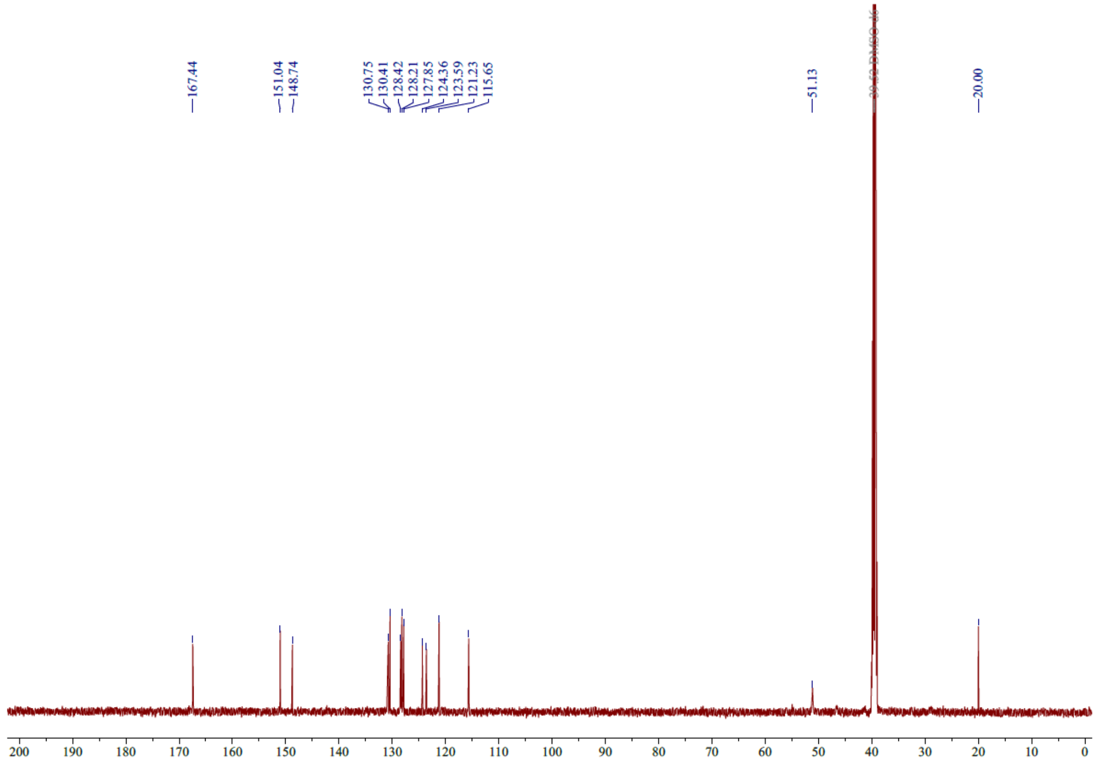

(5-hydroxy-2-methylphenyl)(4-(4-methoxyphenyl)piperazin-1-yl)methanone (**AI7**)

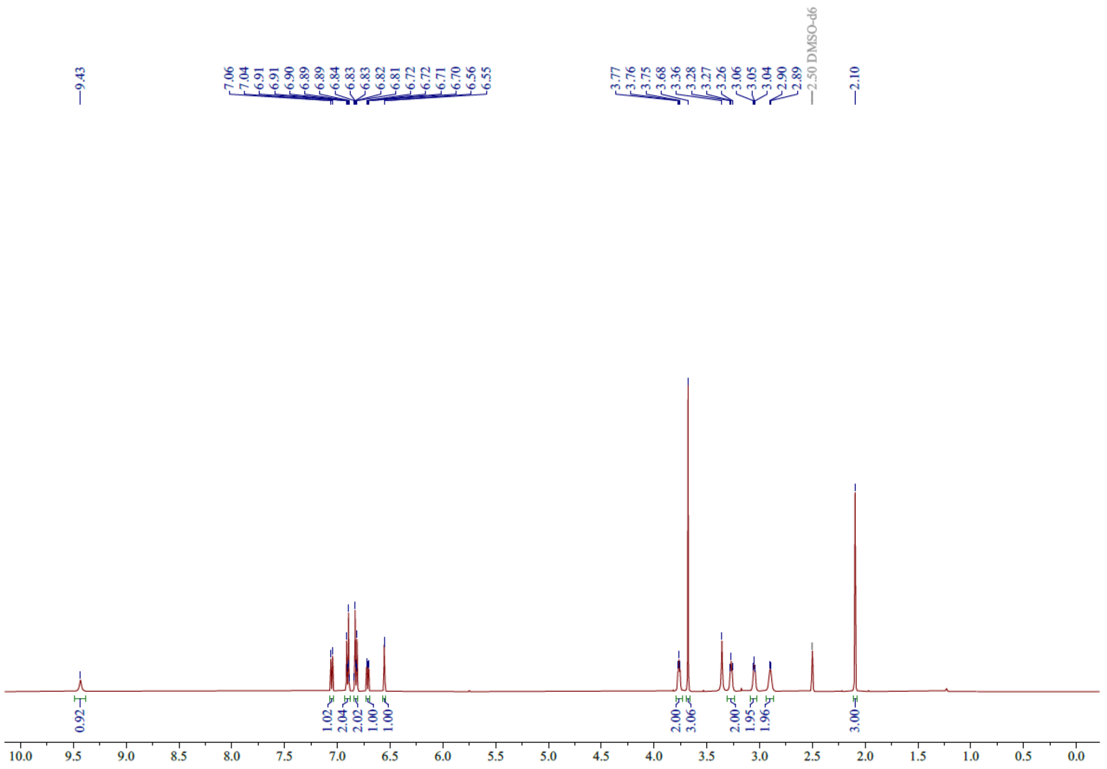

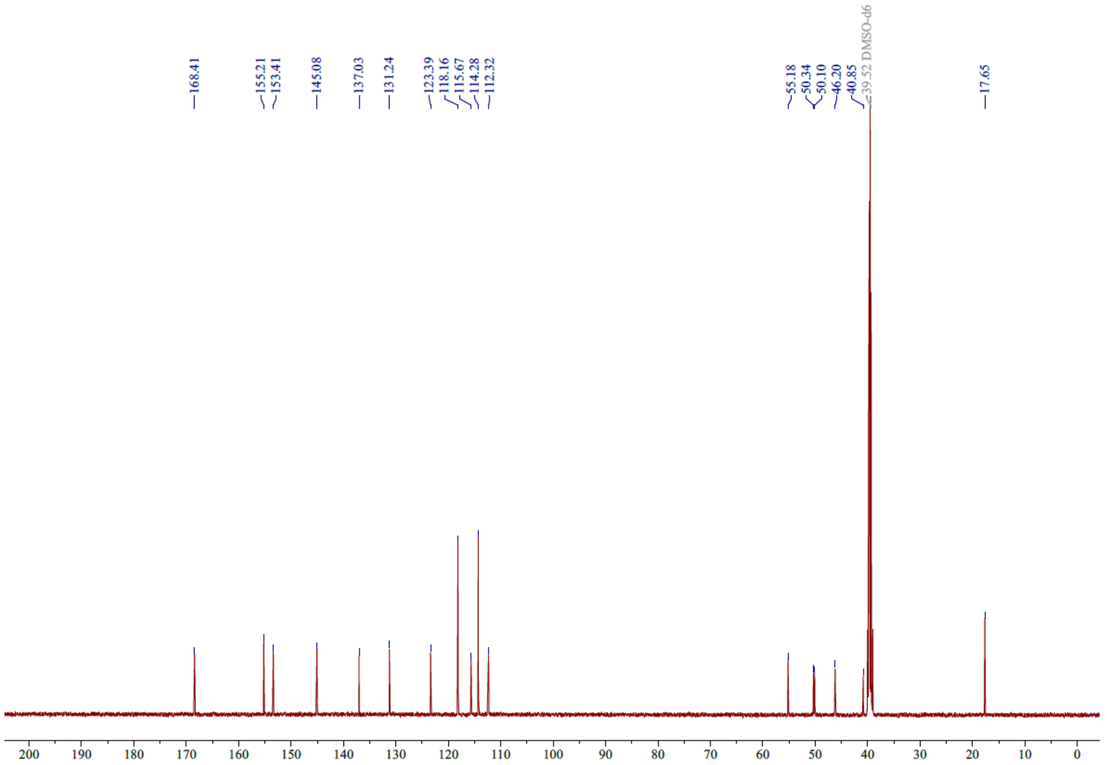

(3-hydroxy-2-methylphenyl)(4-phenethylpiperazin-1-yl)methanone **(AI8)**

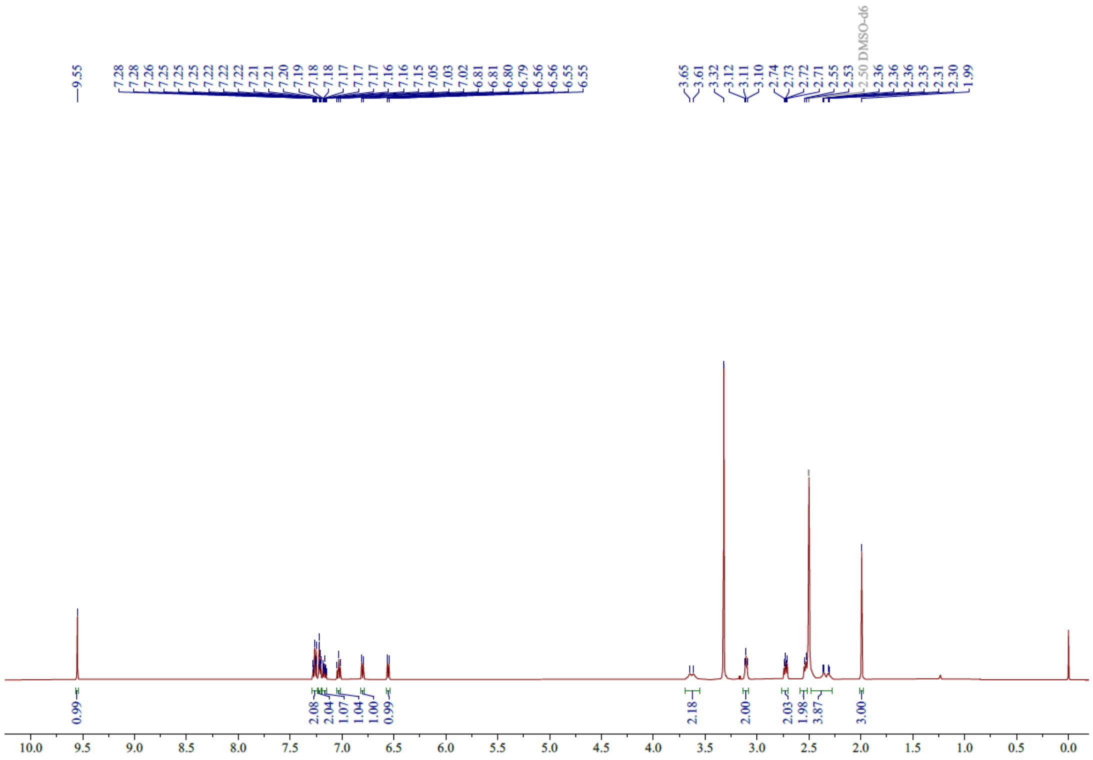

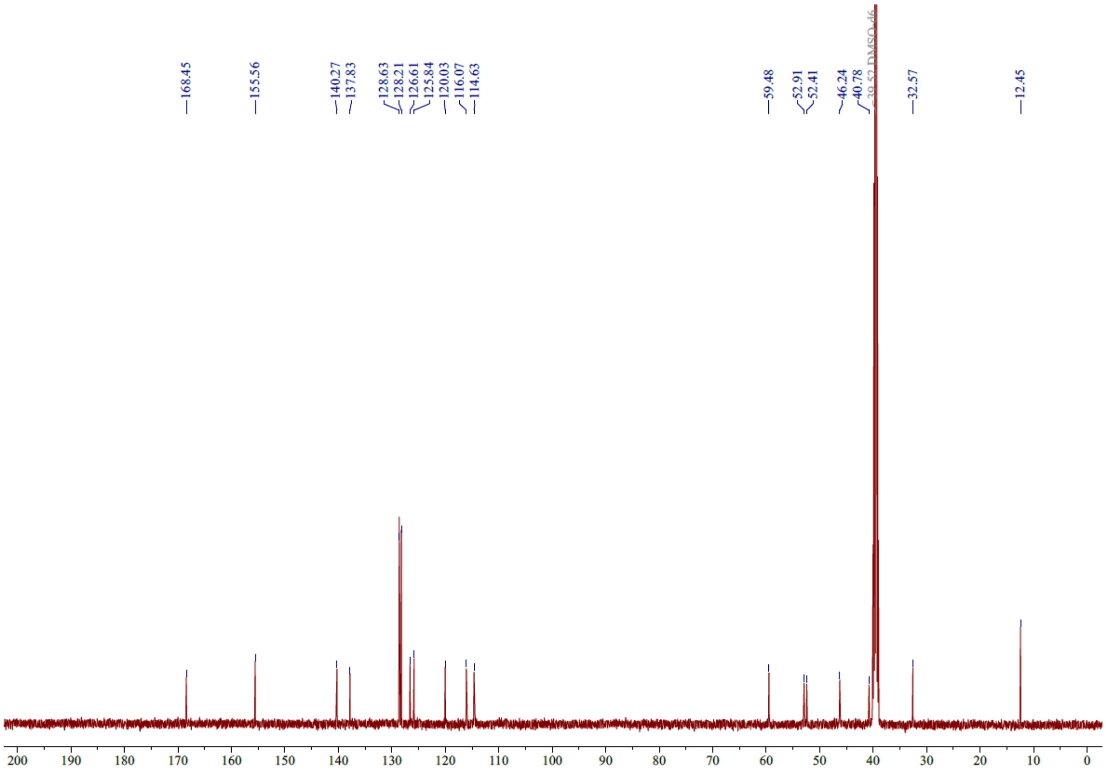

(4-(2-fluoro-4-methylbenzoyl)piperazin-1-yl)(4-hydroxyphenyl)methanone **(AI9)**

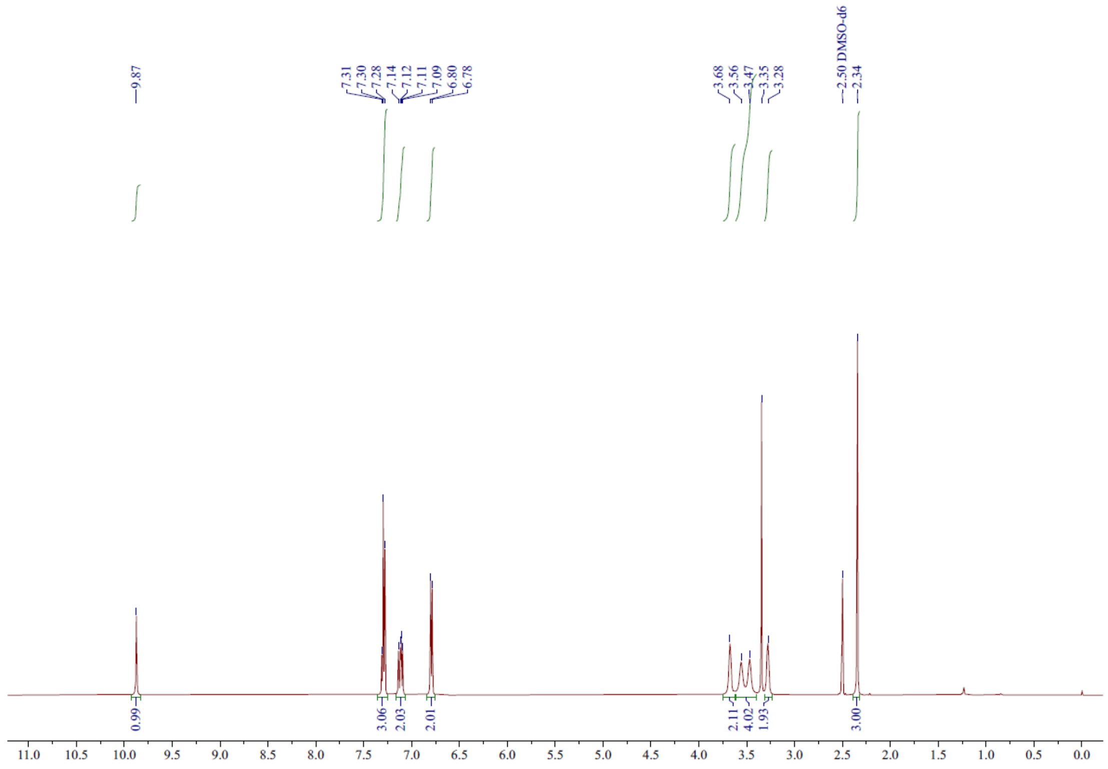

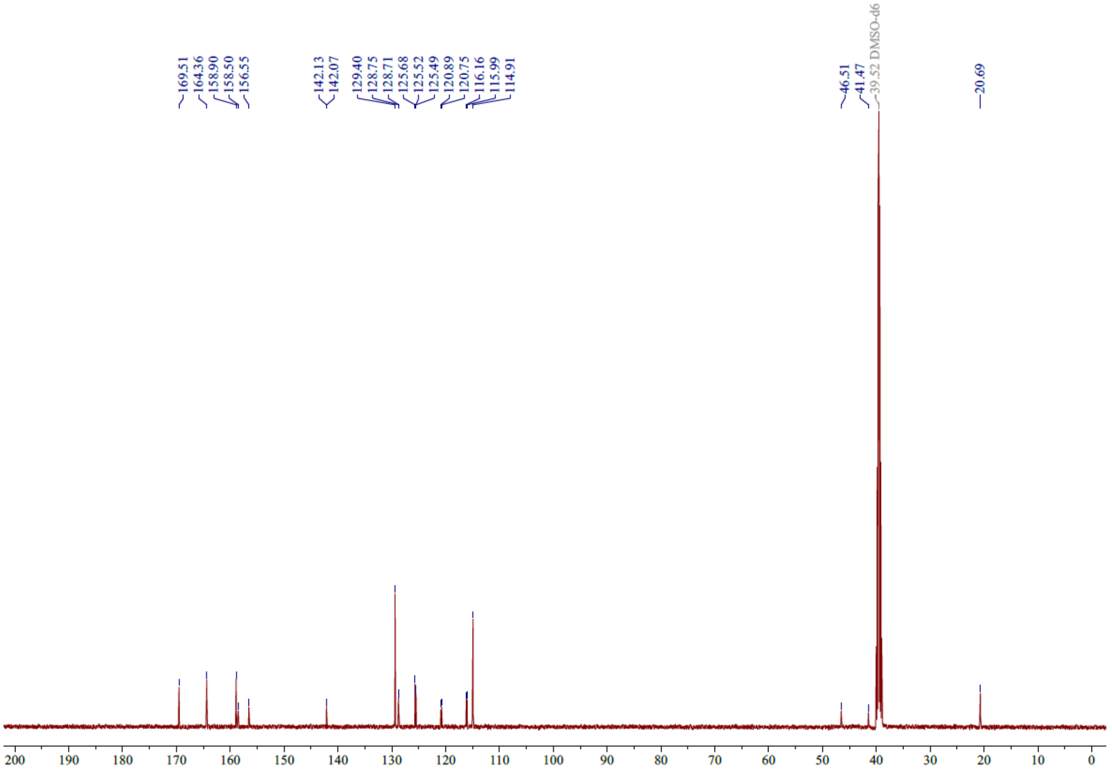

(2,6-dimethylpyridin-4-yl)(4-(4-hydroxyphenyl)piperazin-1-yl)methanone **(AI10)**

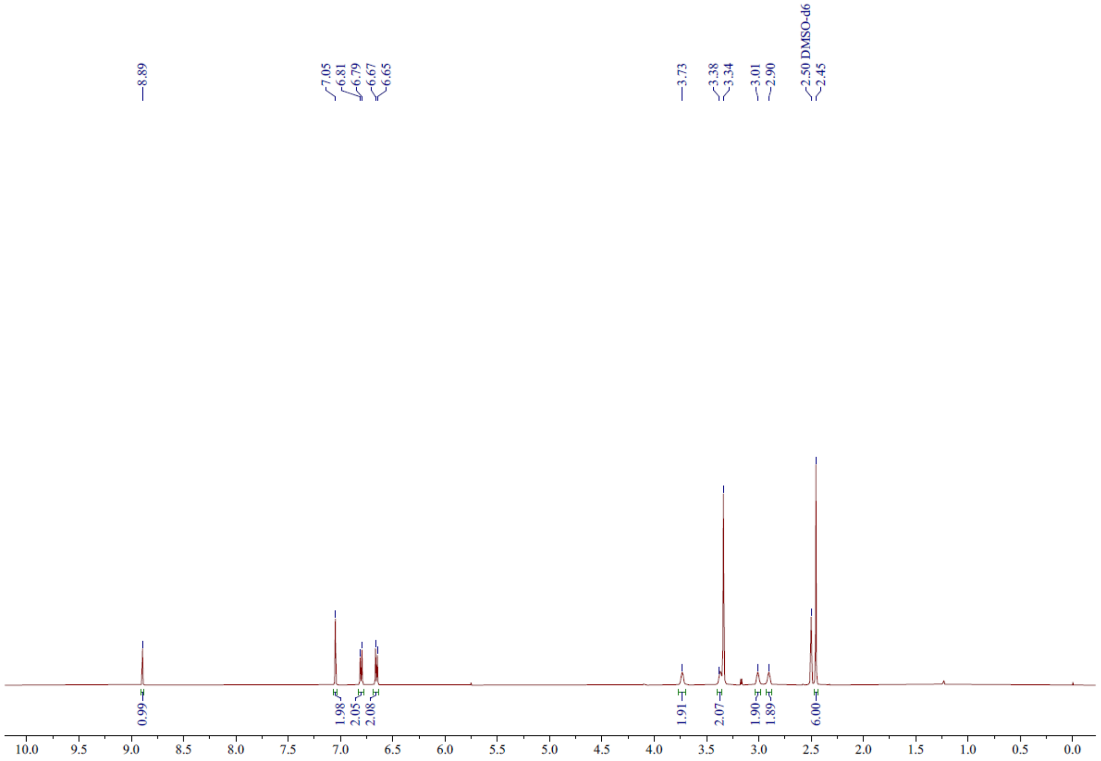

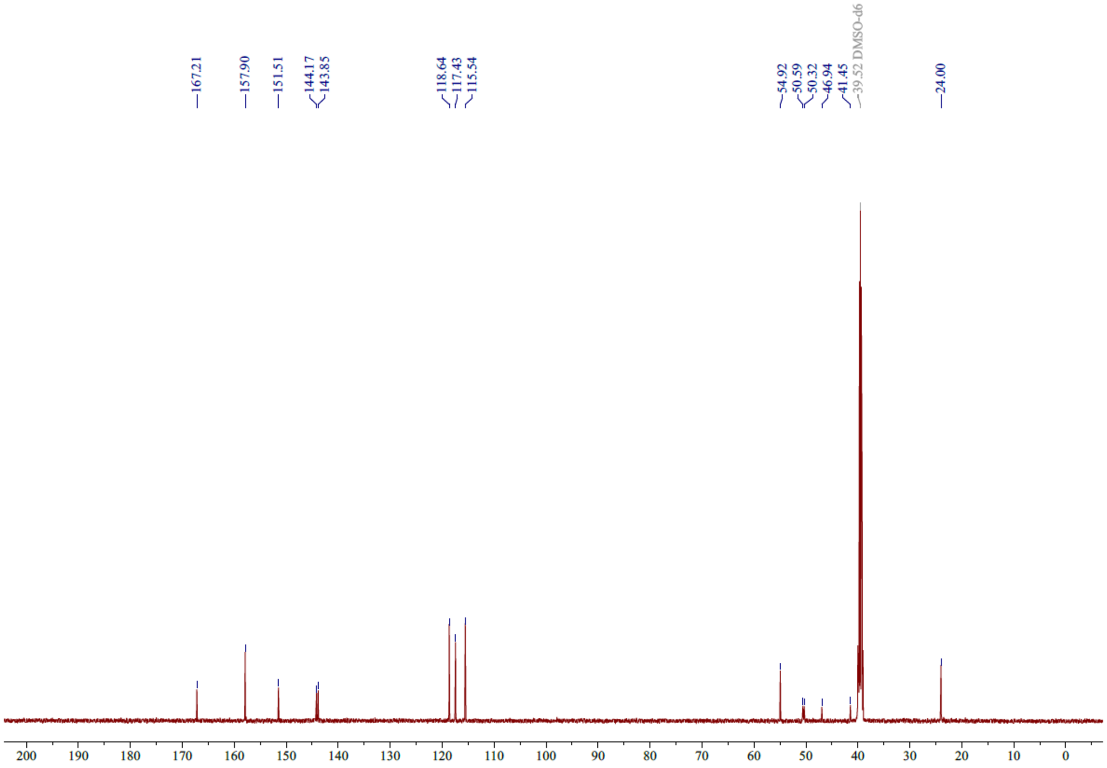

(4-hydroxypyridin-2-yl)(4-(o-tolyl)piperazin-1-yl)methanone **(AI11)**

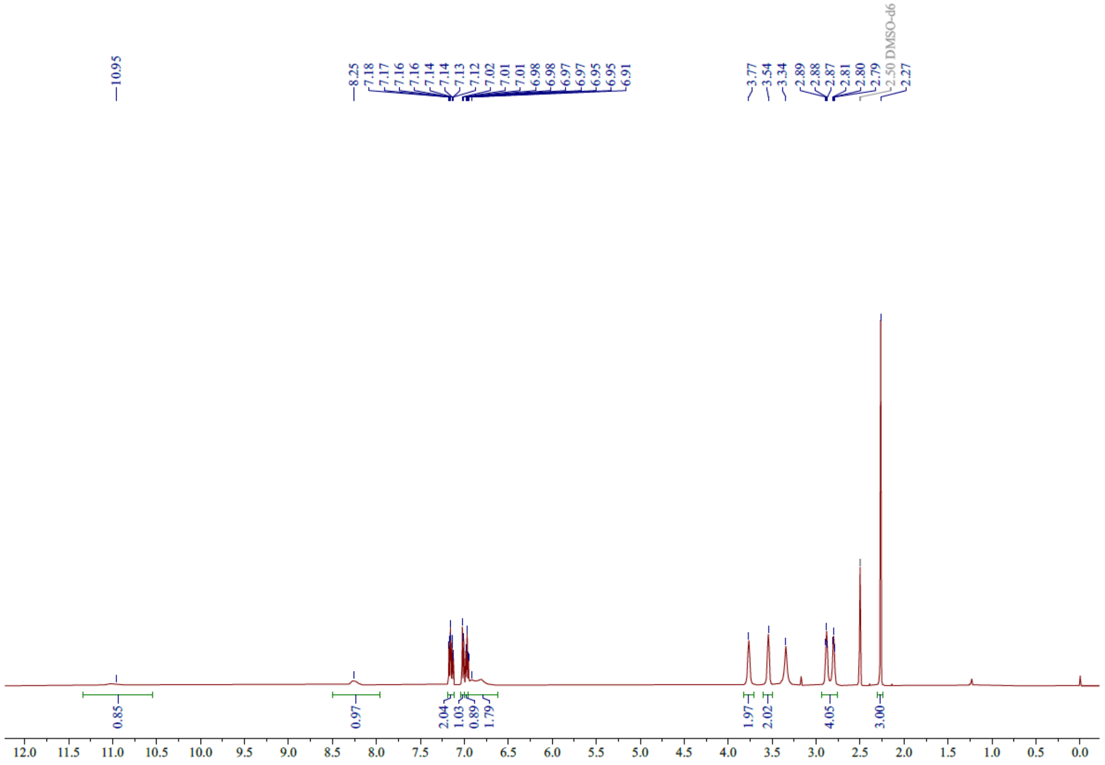

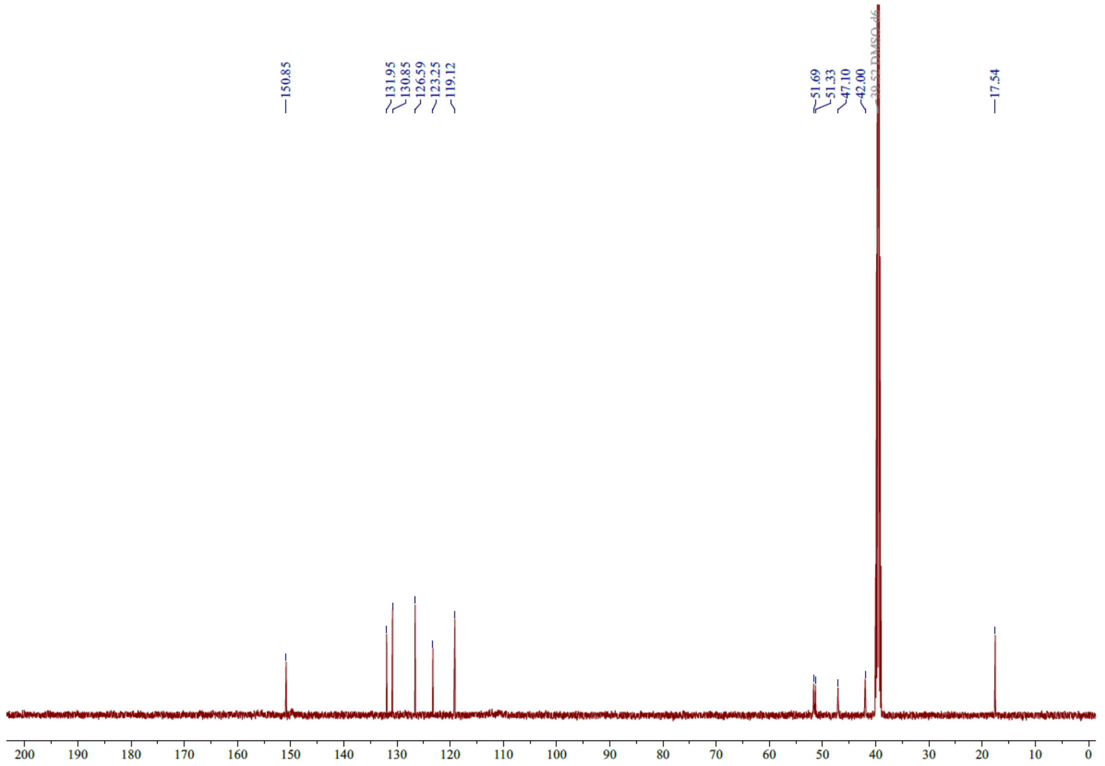

2-(4-hydroxyphenyl)-1-(4-(4-methylbenzyl)piperazin-1-yl)ethan-1-one **(AI12)**

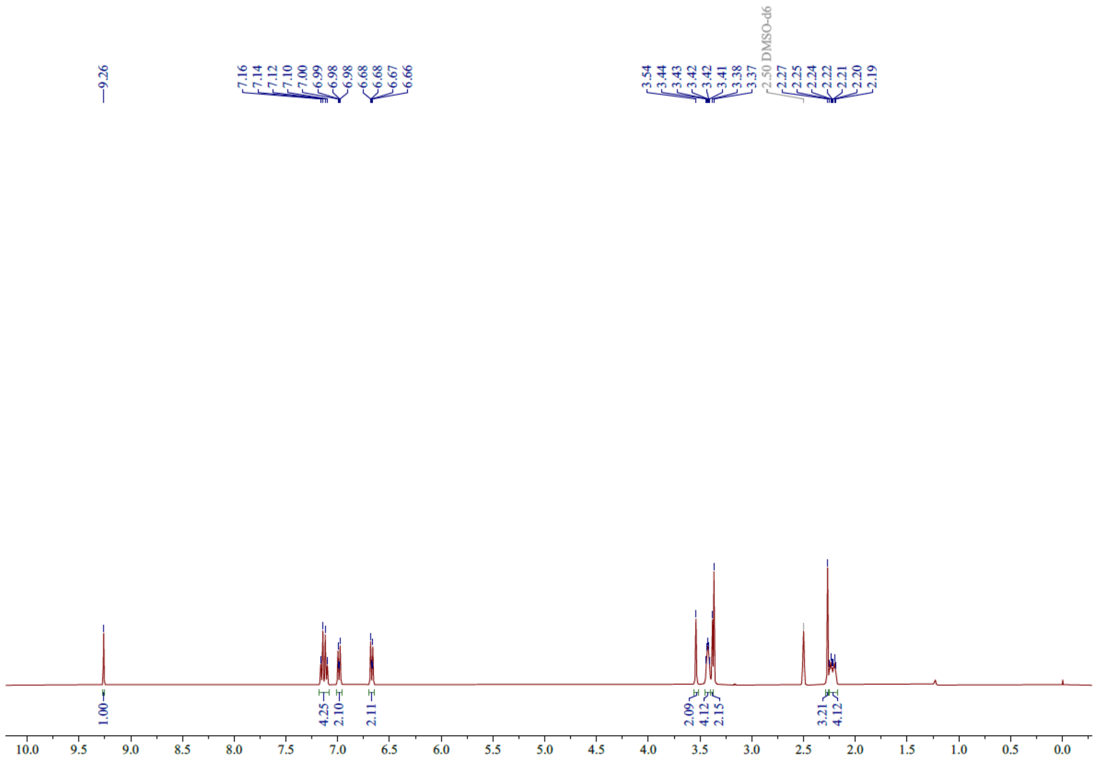

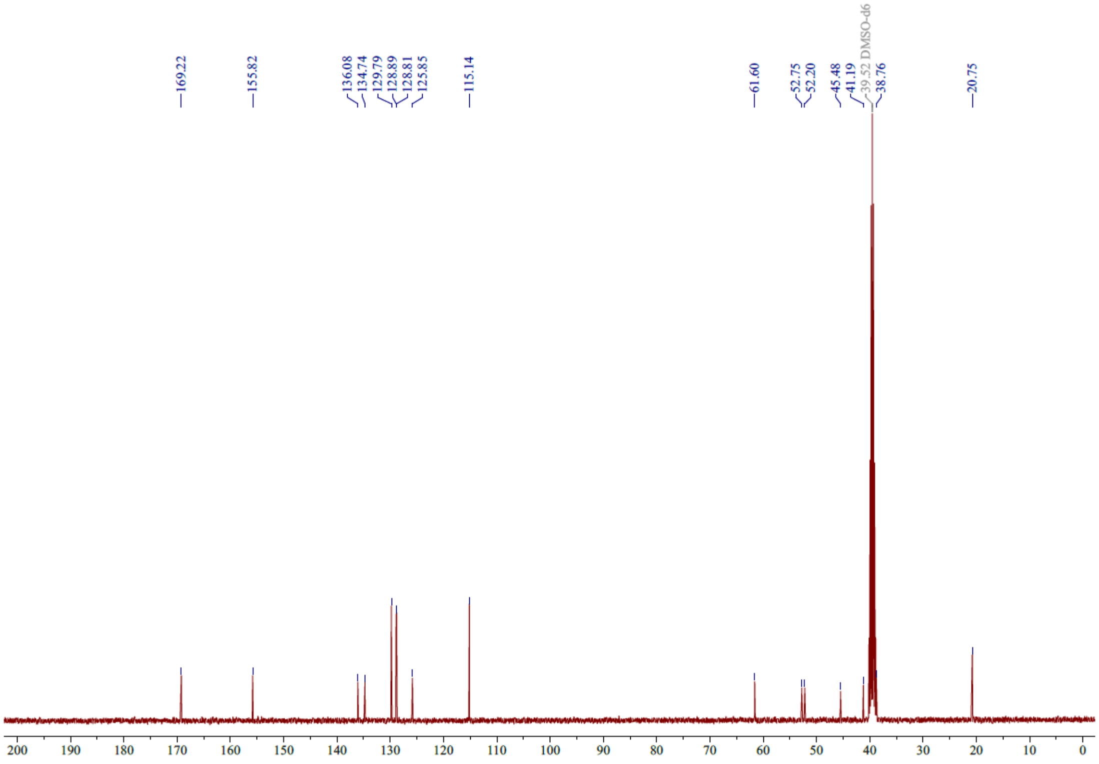

***2-(2-(4-(4-hydroxyphenyl)piperazin-1-yl)ethyl)phenol* (AI13)**

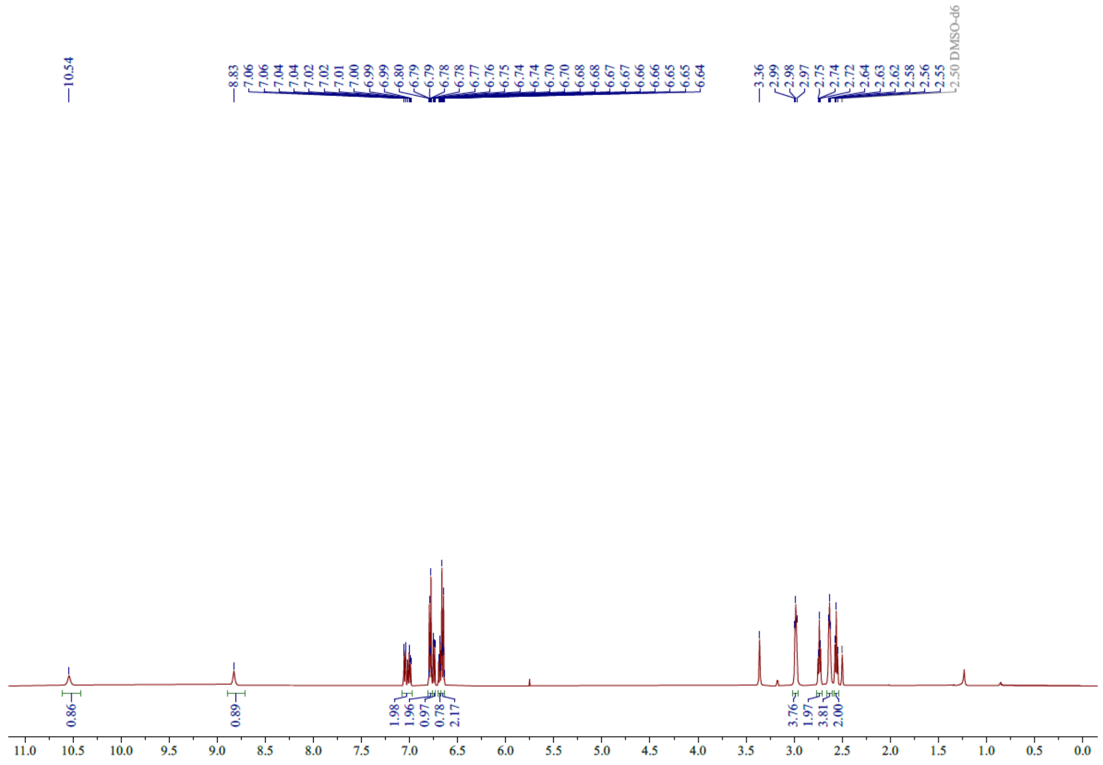

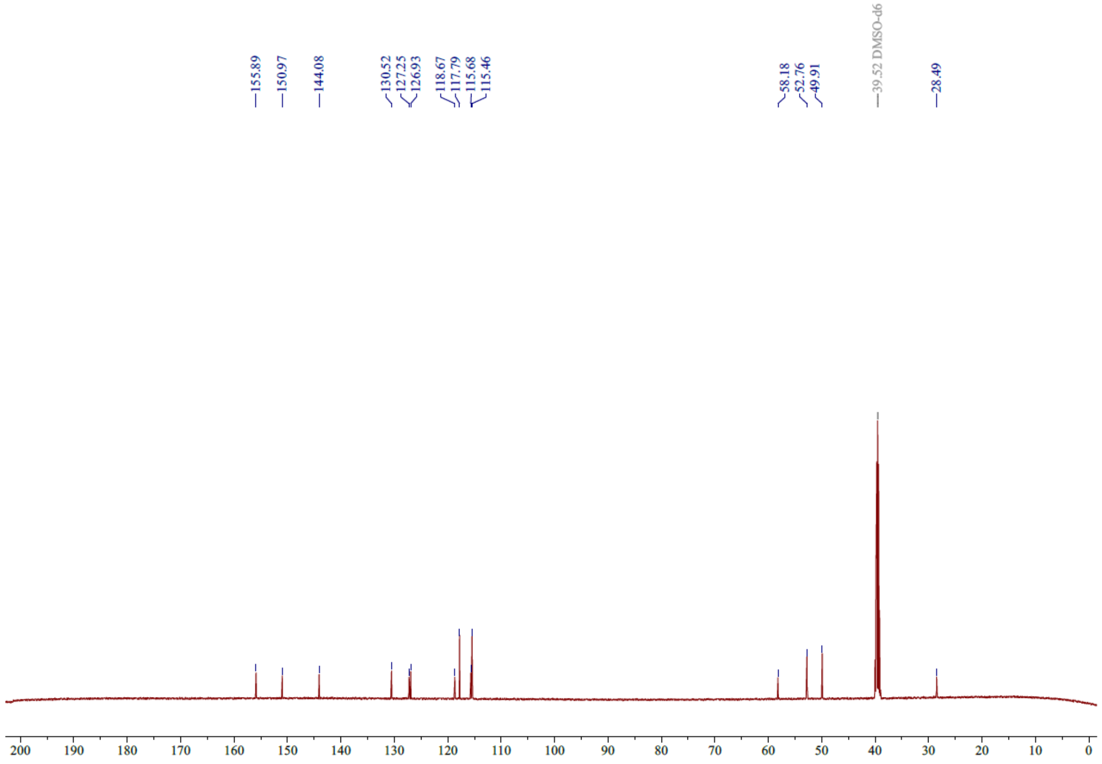

(4-(4-hydroxyphenyl)piperazin-1-yl)(1-methyl-1H-indol-3-yl)methanone **(AI14)**

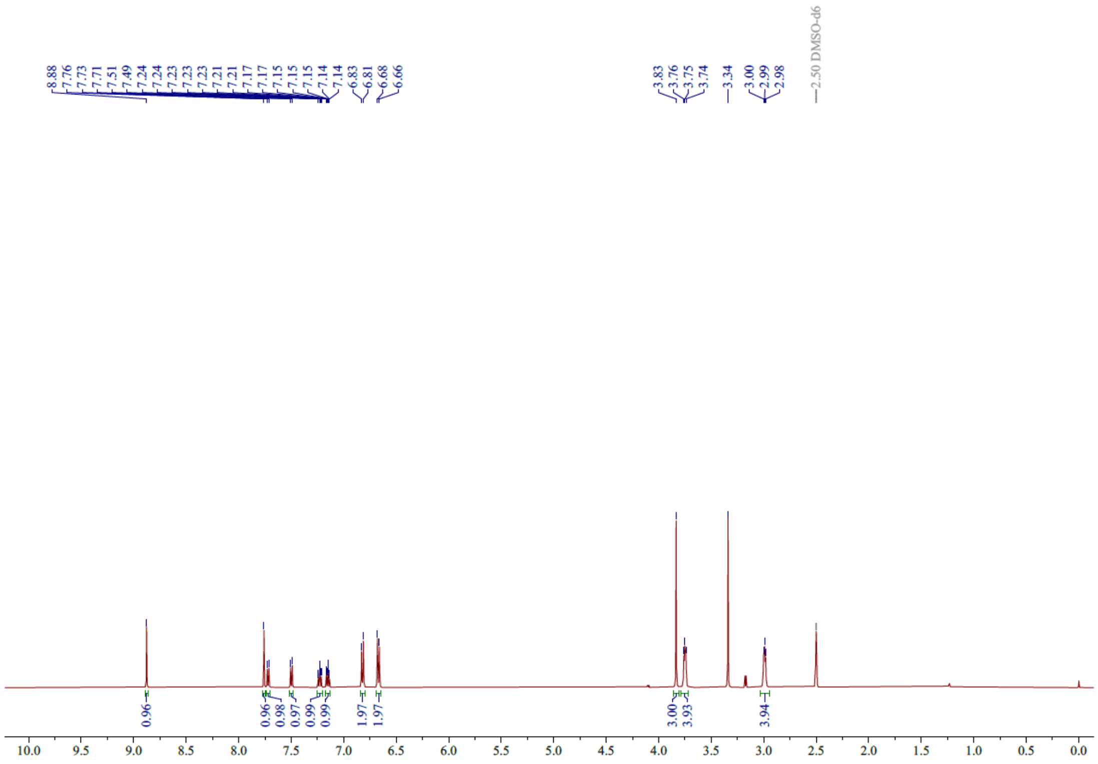

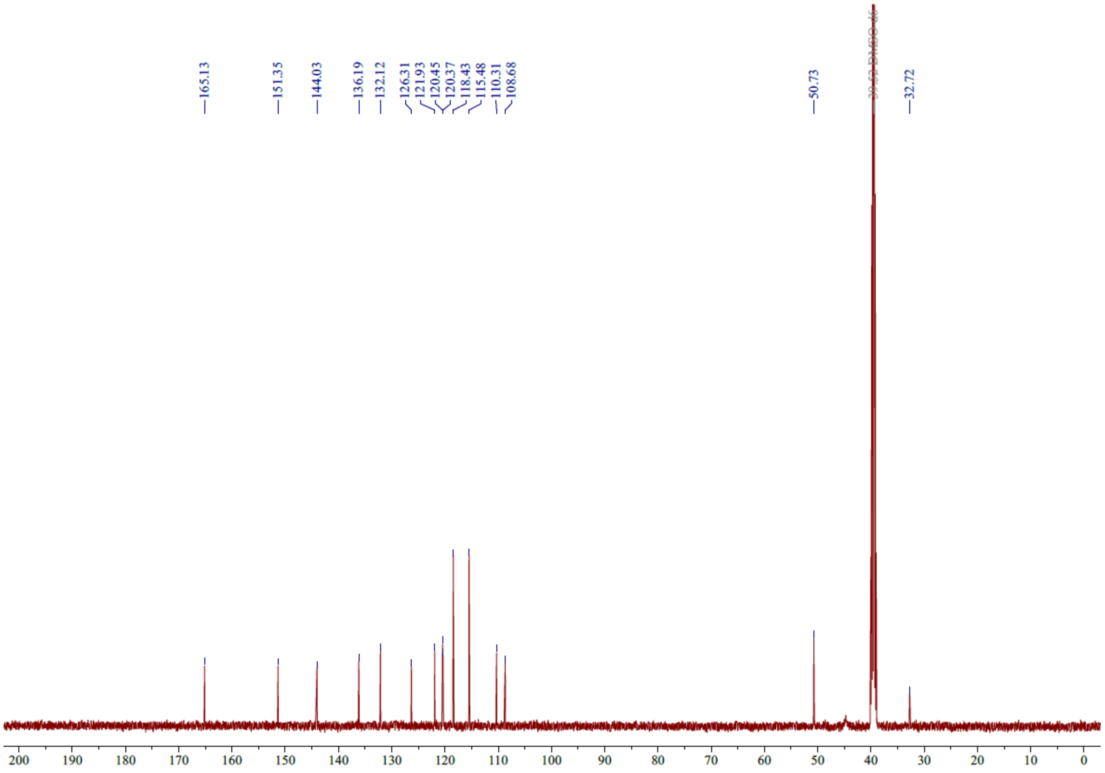

(4-(3-hydroxyphenyl)piperazin-1-yl)(2-methyl-1H-benzo[d]imidazol-6-yl)methanone **(AI15)**

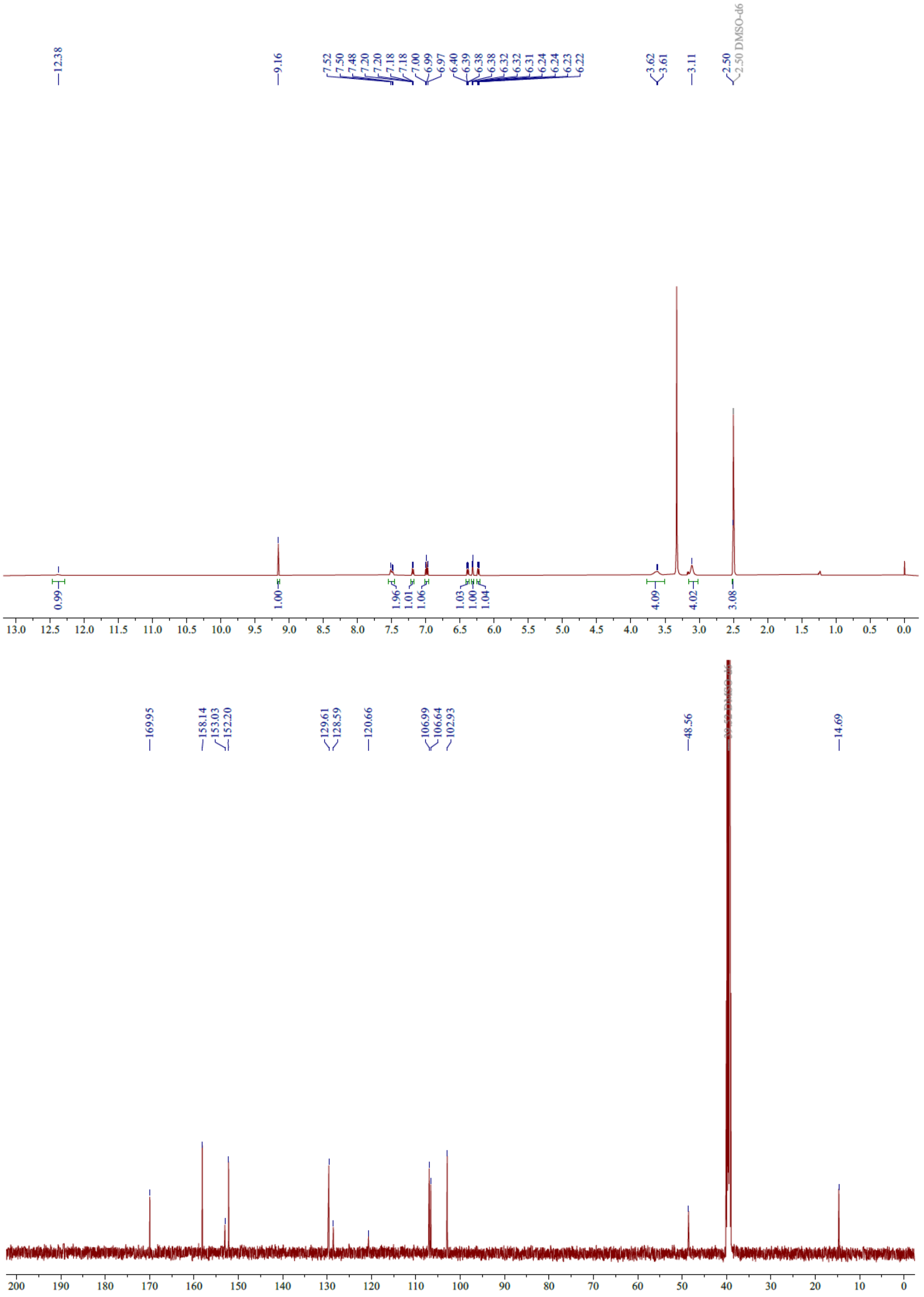

(4-(4-hydroxyphenyl)piperidin-1-yl)(m-tolyl)methanone **(AI16)**

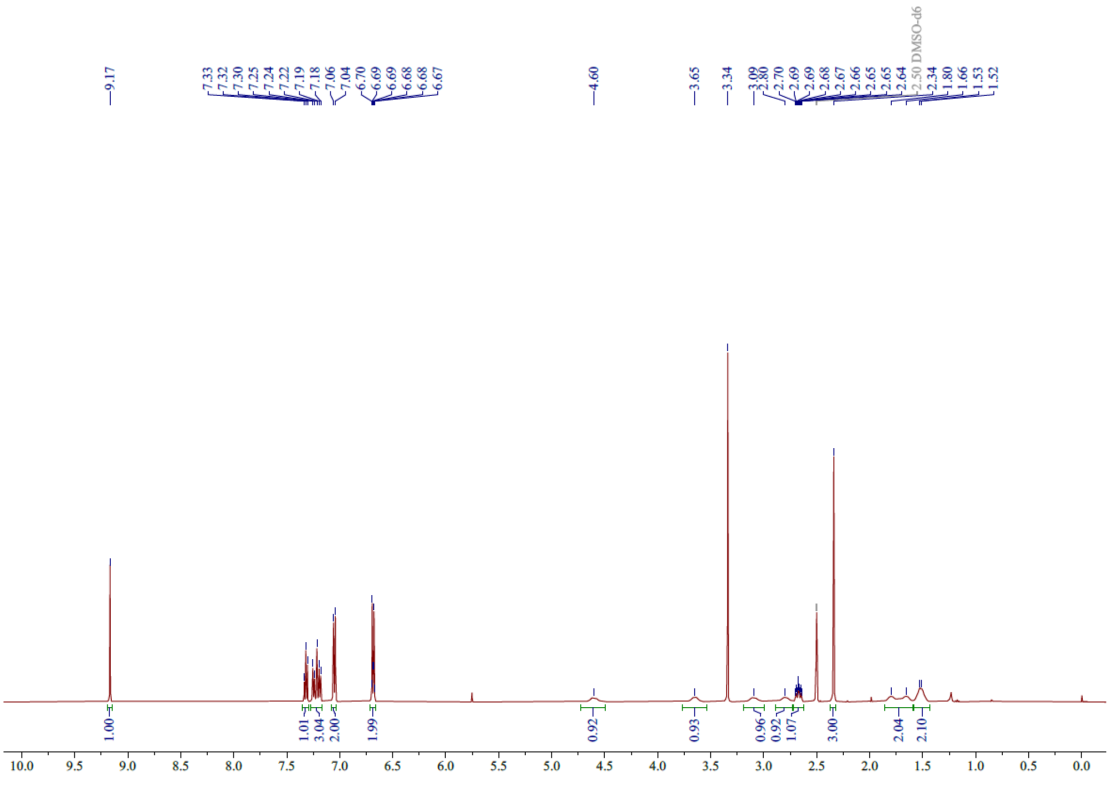

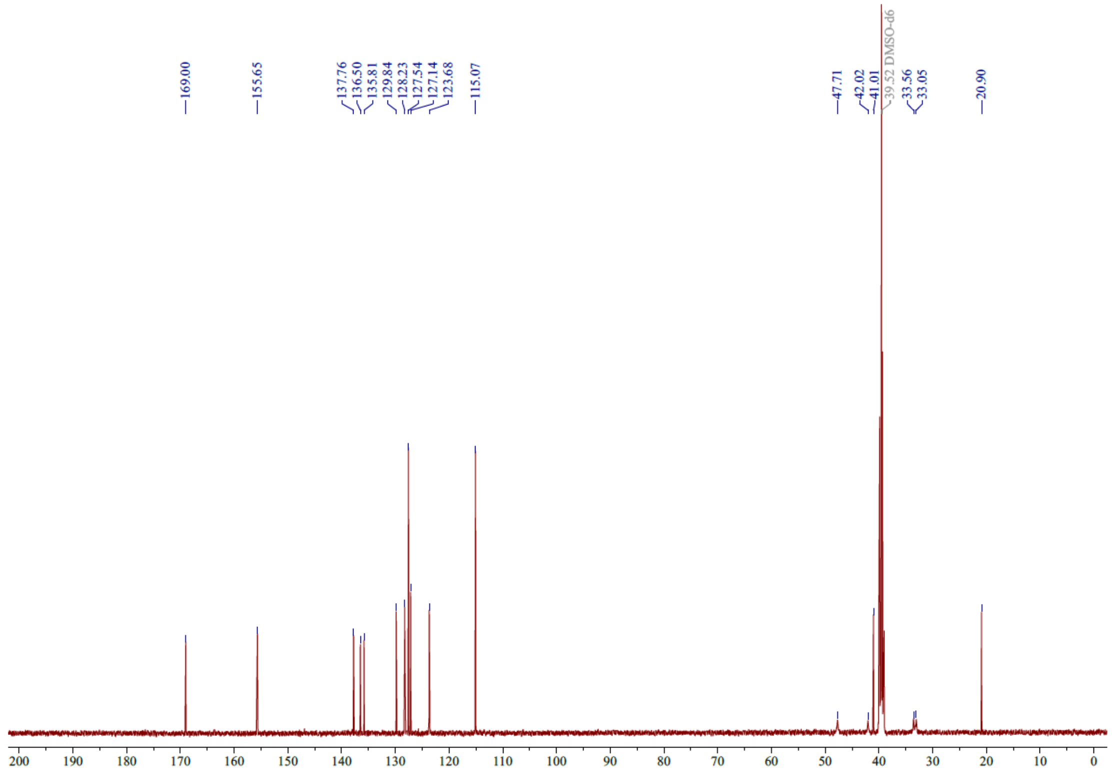

***1-(3-chlorobenzoyl)-N-(4-methoxybenzyl)piperidine-4-carboxamide* (AI17)**

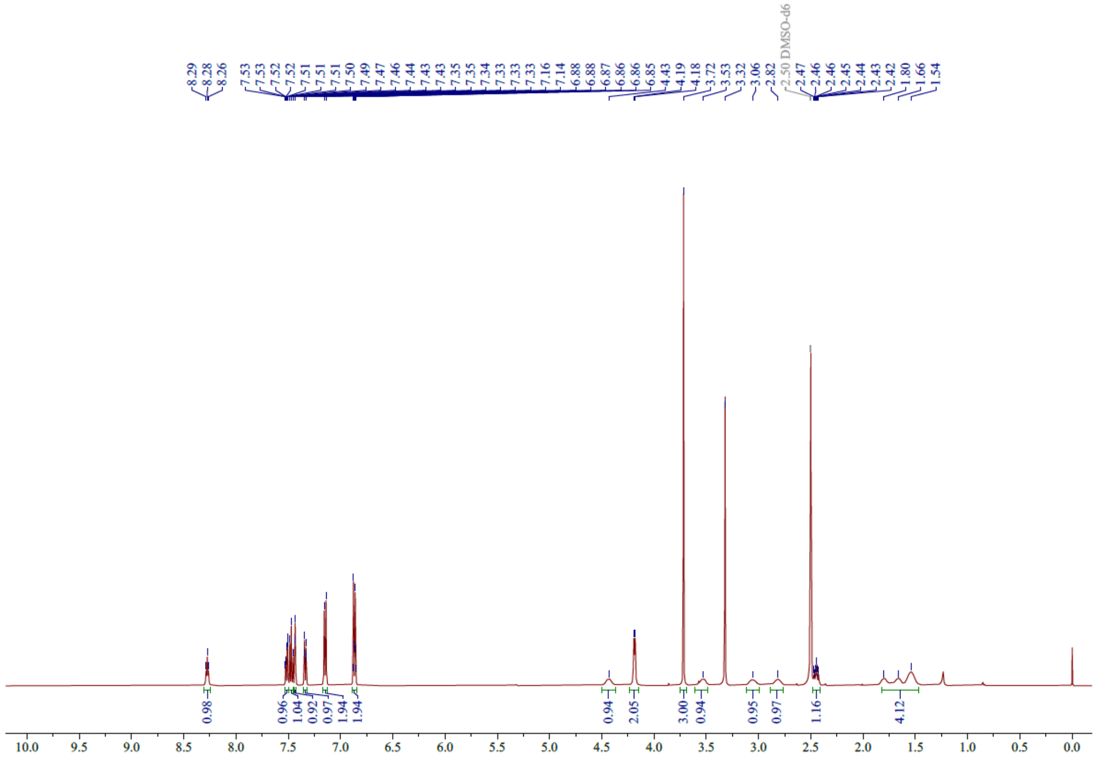

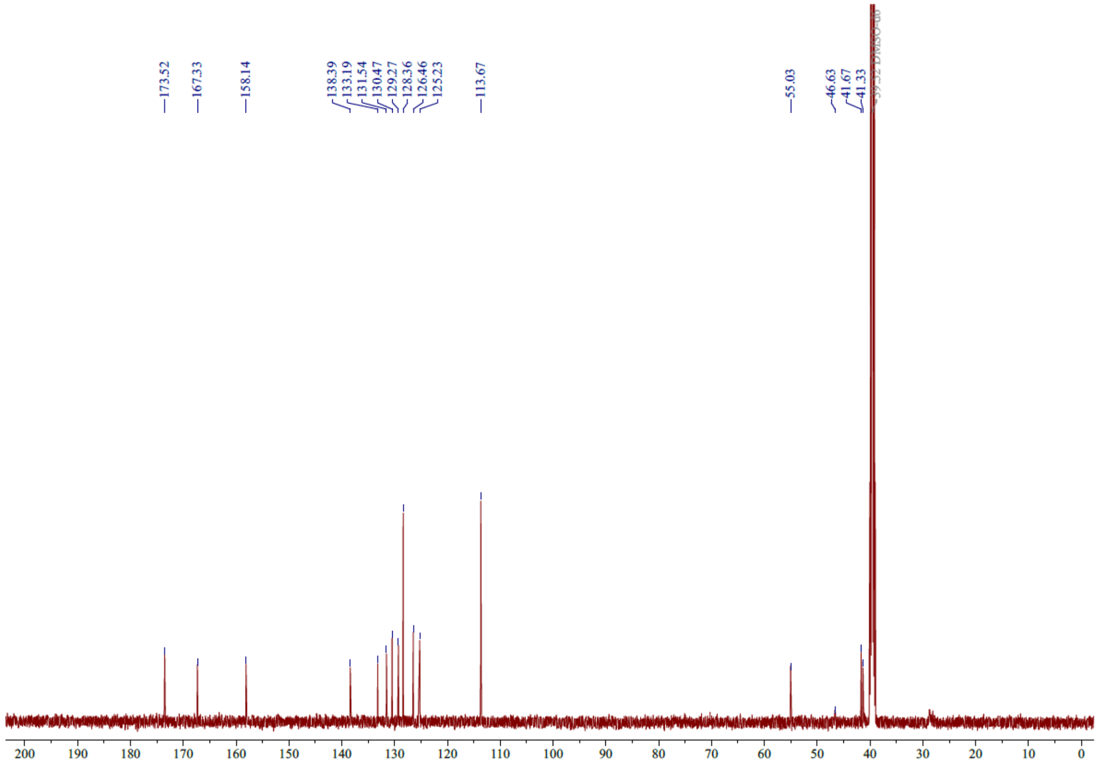

(3-hydroxy-4-methylphenyl)(4-phenylpiperidin-1-yl)methanone **(AI18)**

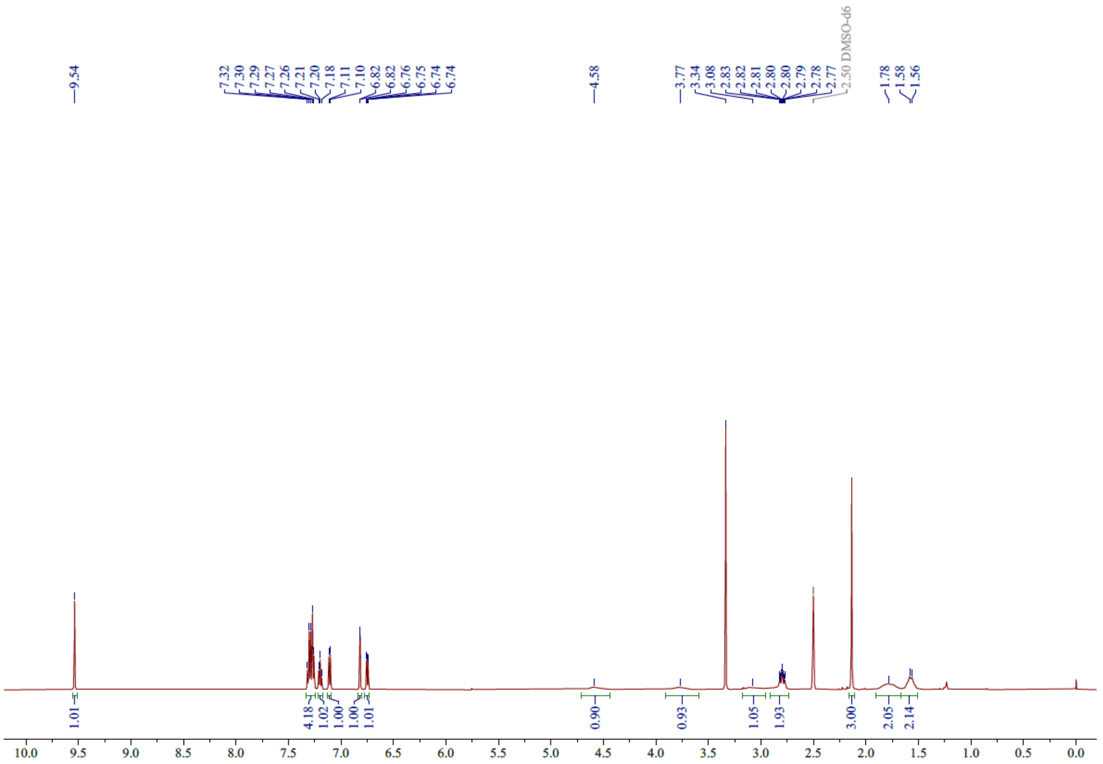

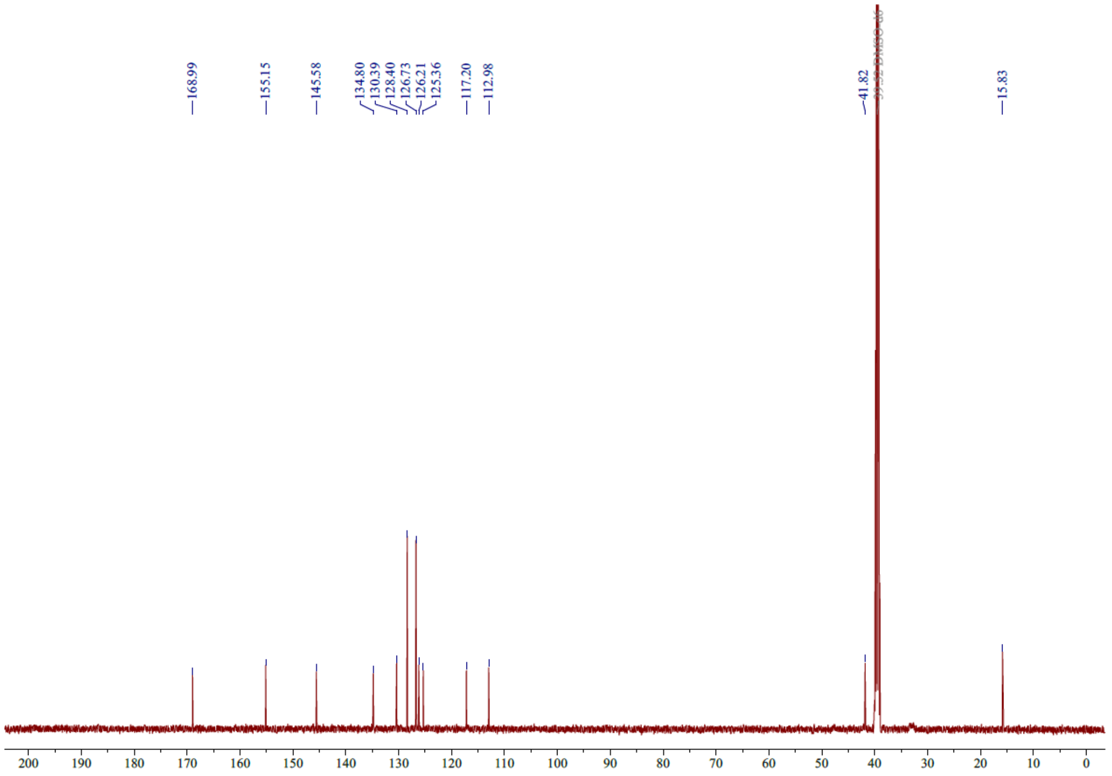

***4-chloro-N-(4-(dimethylamino)benzyl)-3-hydroxybenzamide* (AI19)**

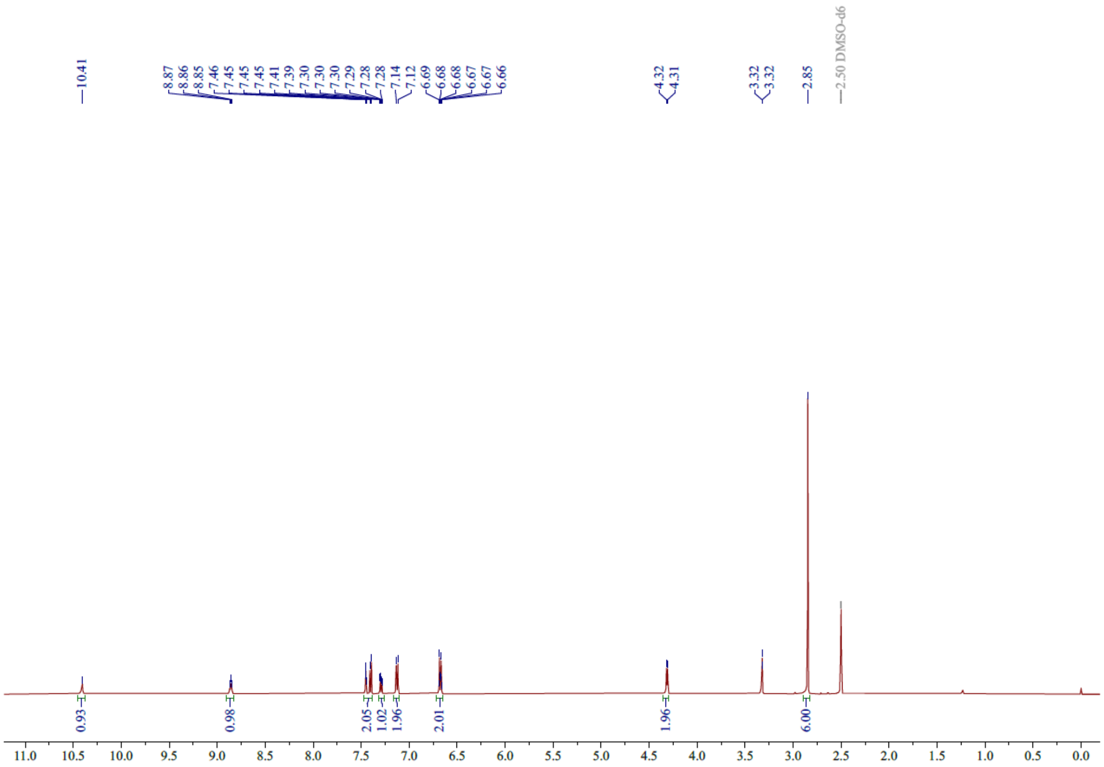

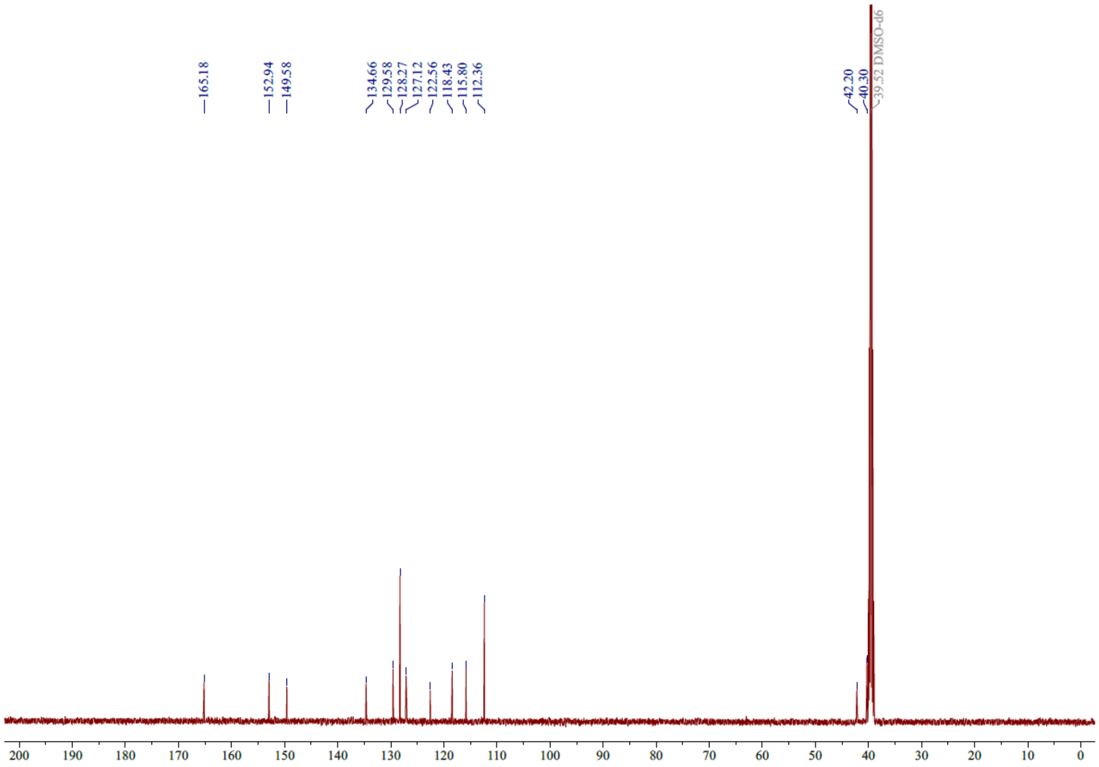

N-(2,5-dimethylbenzyl)-2-hydroxybenzamide **(AI20)**

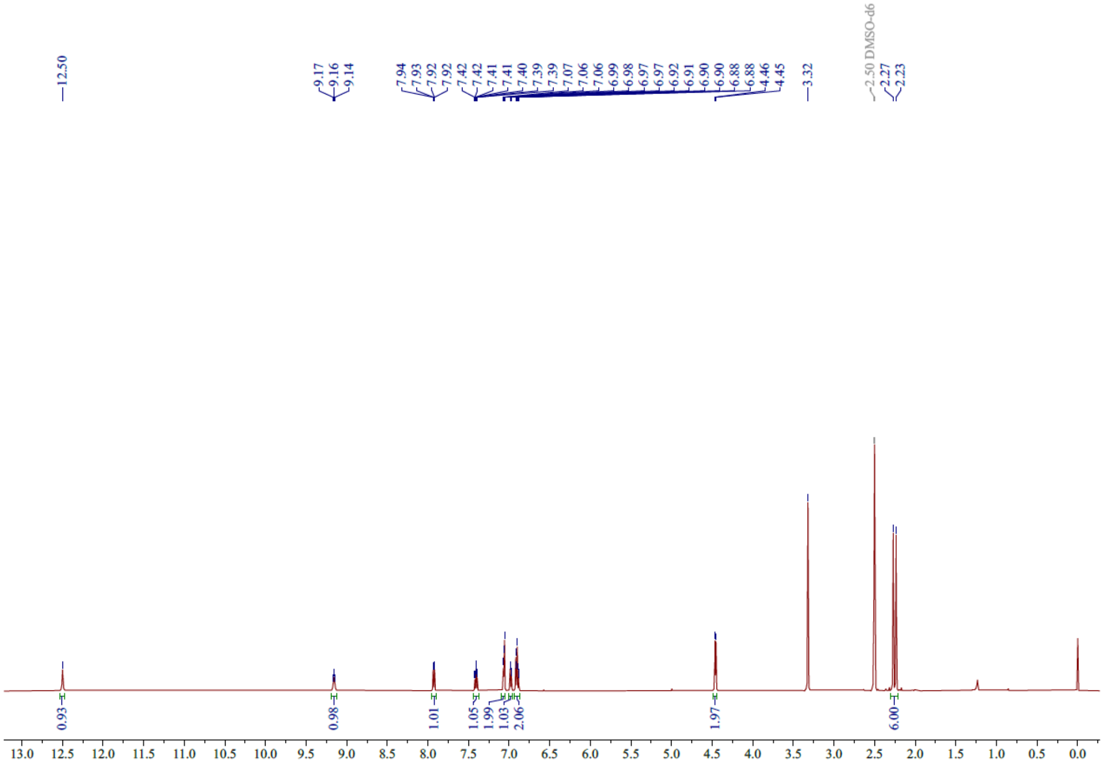

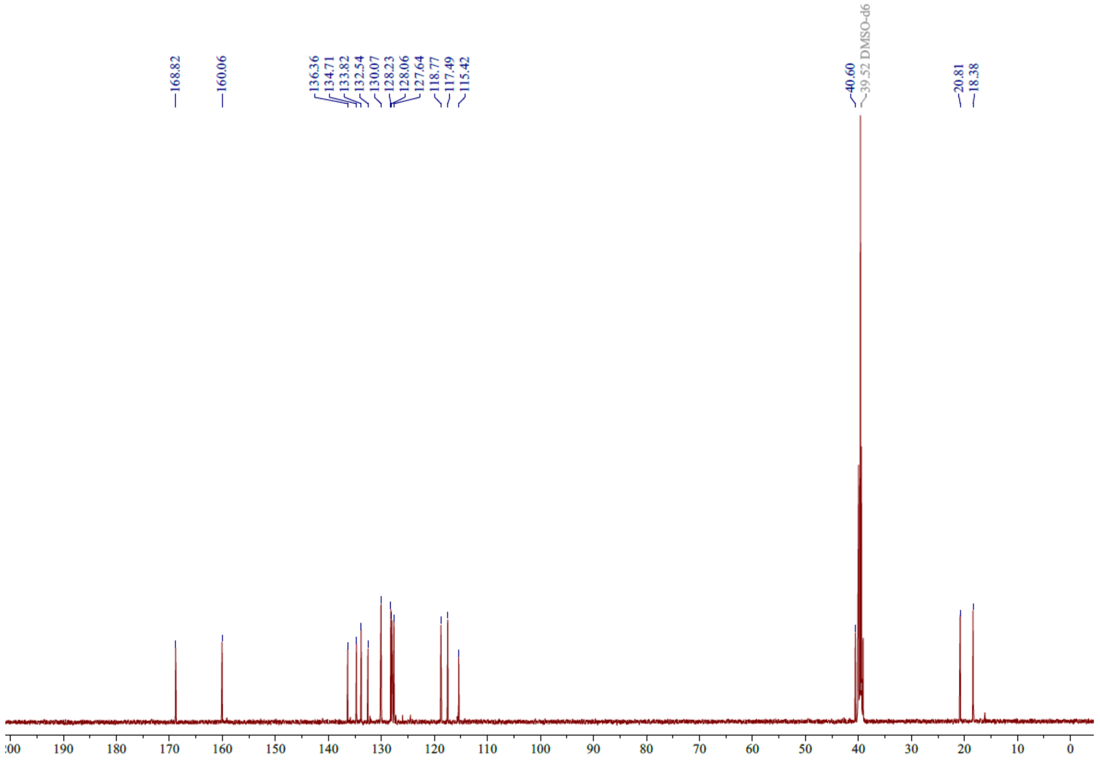

N-(4-chlorobenzyl)-4-hydroxy-3-methylbenzamide (**AI21**)

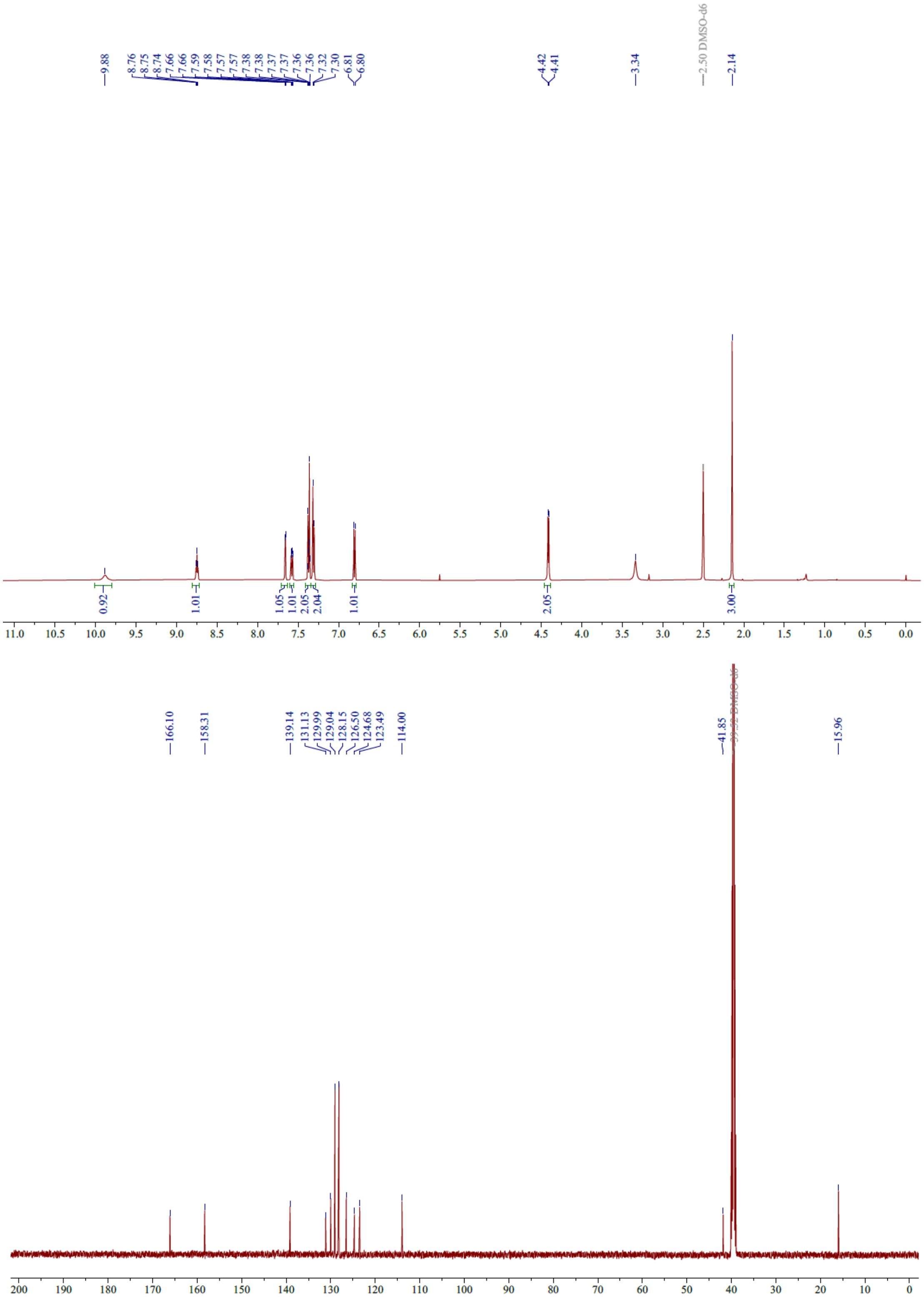

N-(2-hydroxybenzyl)-2-(4-(4-methylbenzyl)piperazin-1-yl)isonicotinamide **(AI22)**

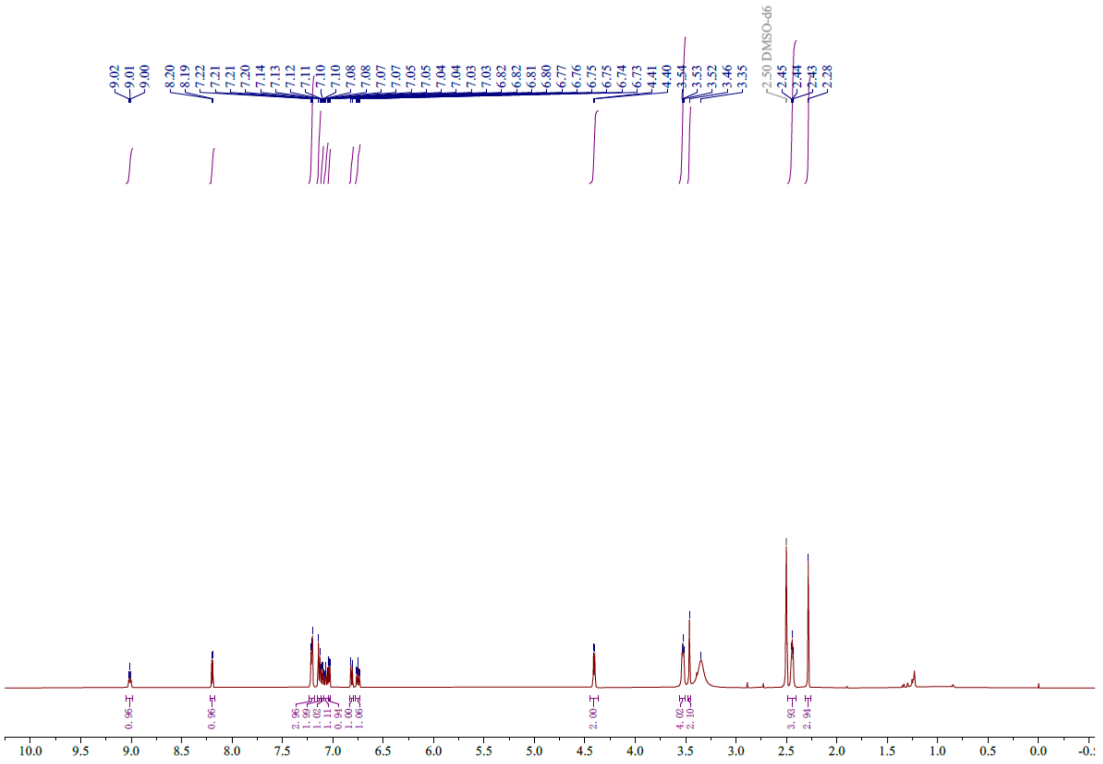

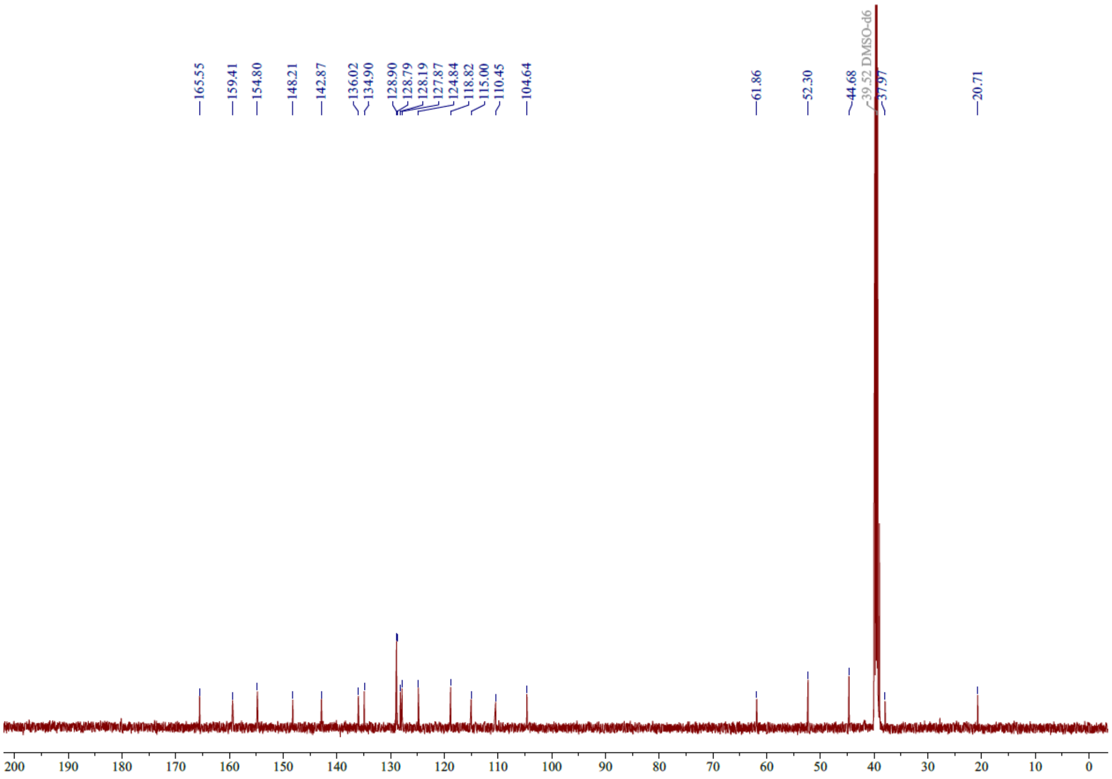

***(E)-3-(3,4-dihydroxyphenyl)-N-(4-fluorophenyl)acrylamide* (AI23)**

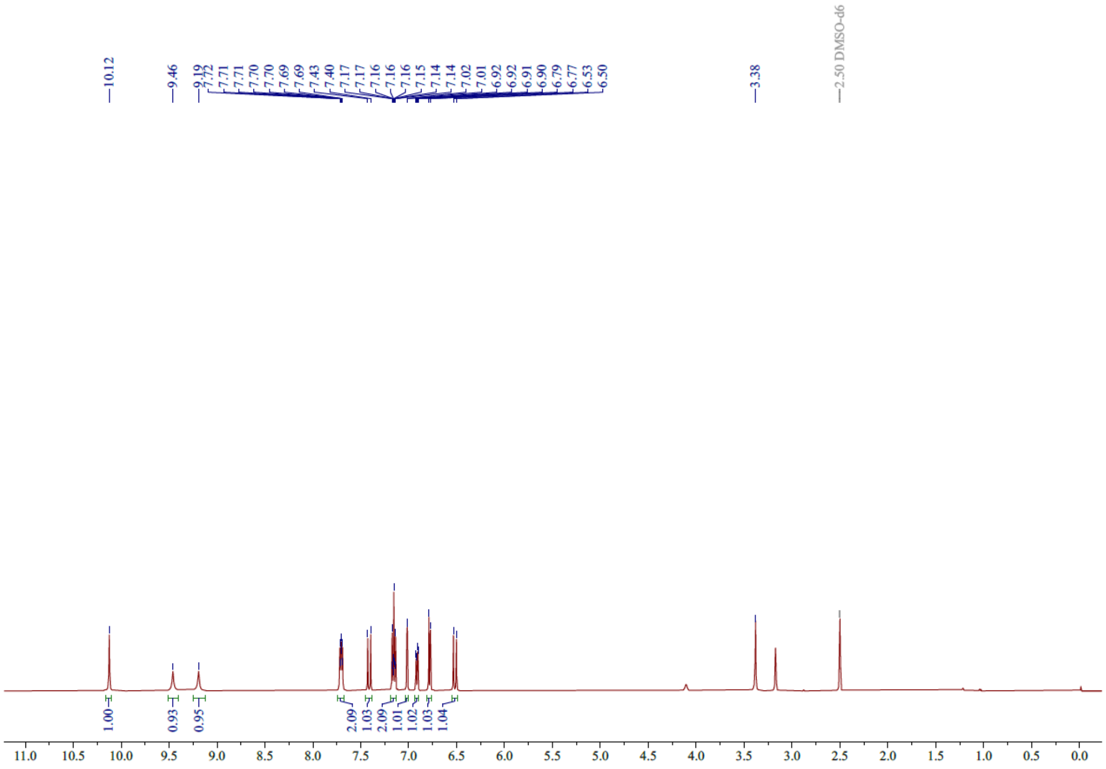

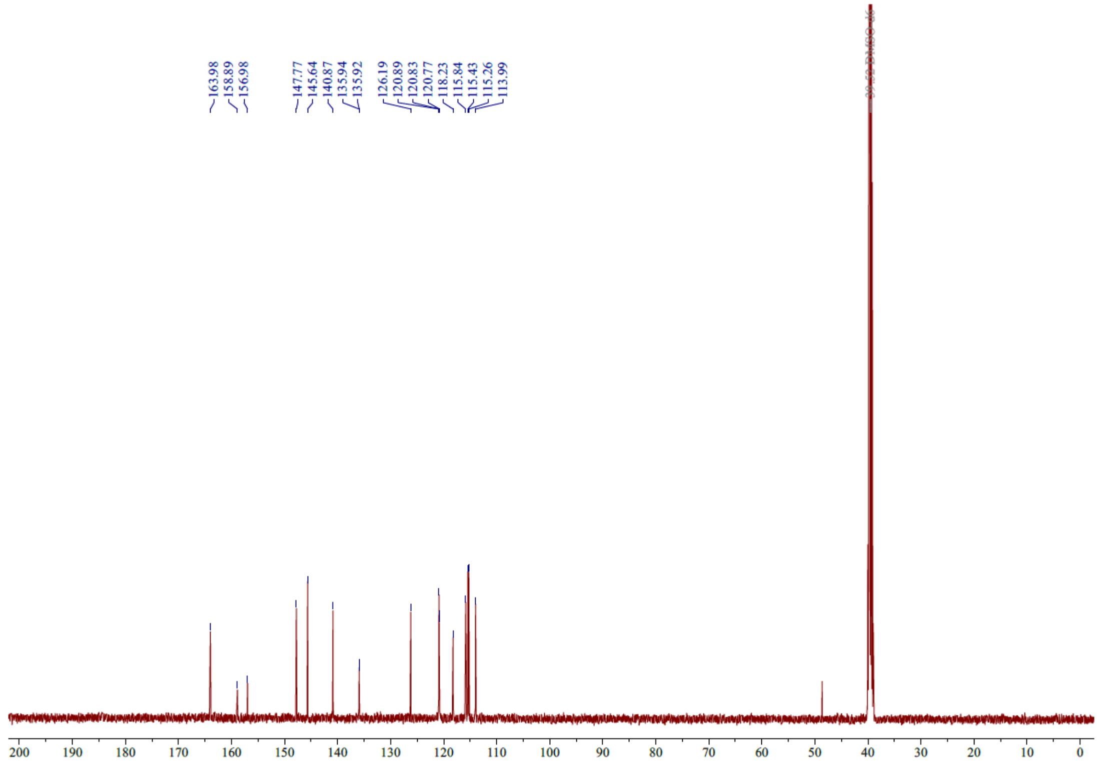

N-(2-hydroxybenzyl)-1-methyl-1H-indazole-5-carboxamide **(AI24)**

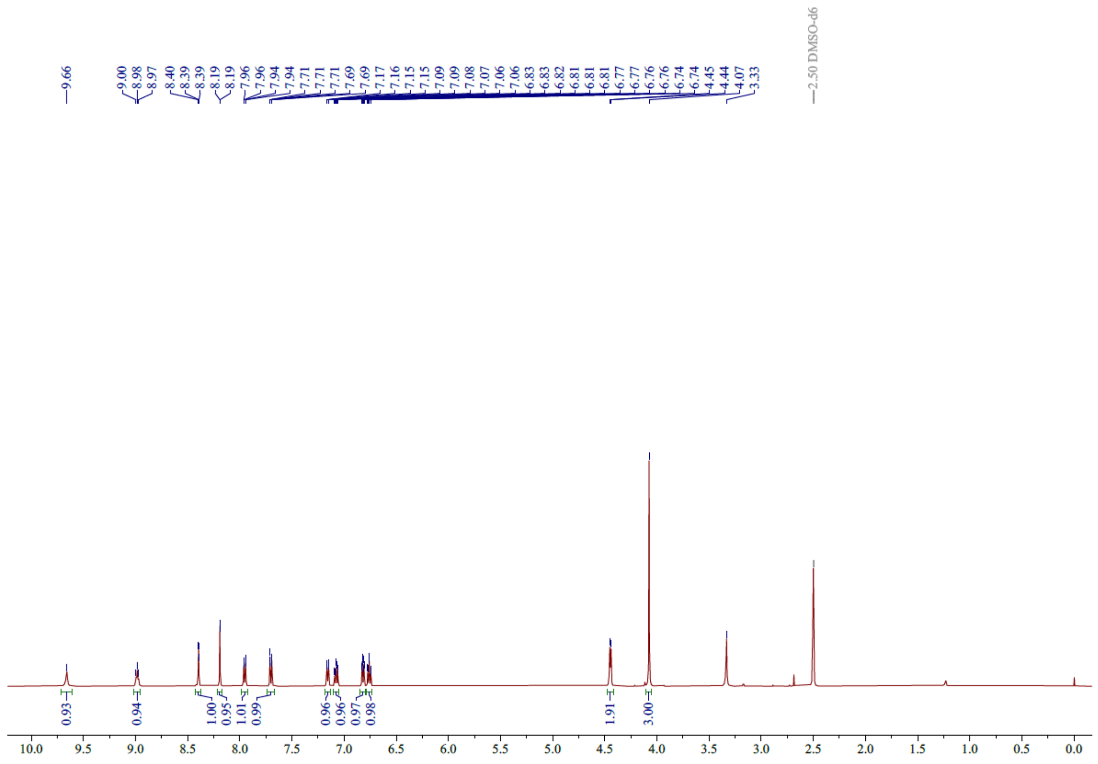

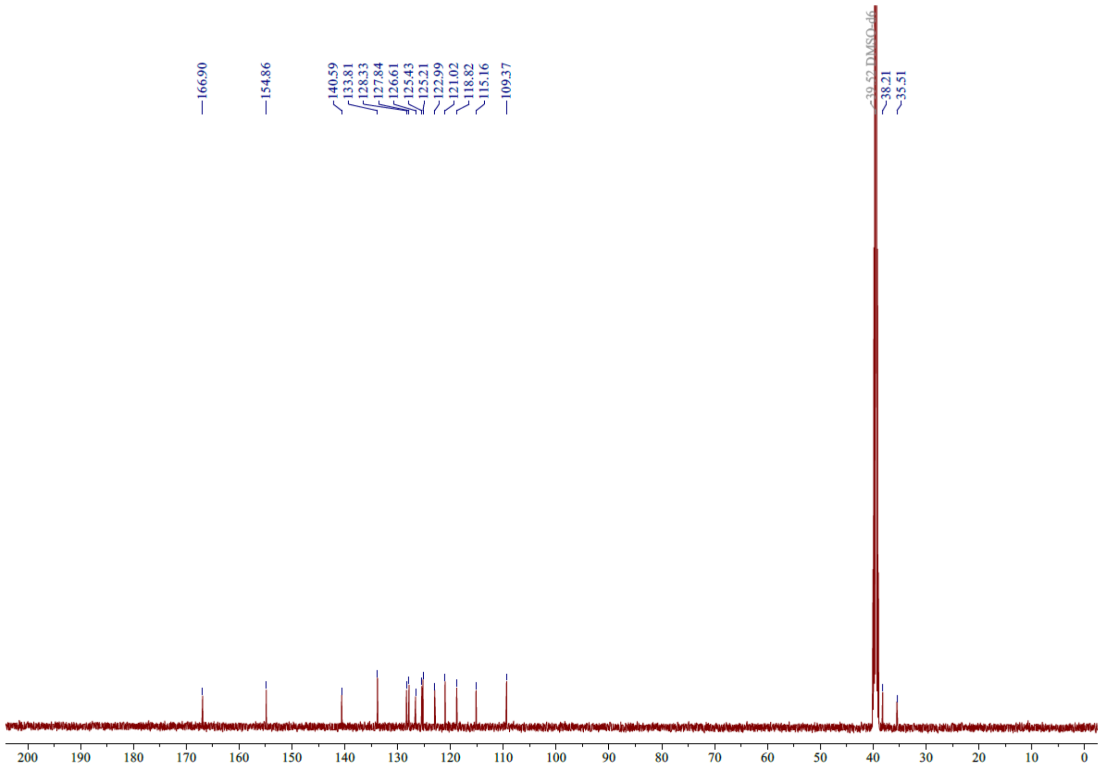

N-(4-hydroxybenzyl)-6-(((4-methoxypyridin-2-yl)amino)methyl)picolinamide **(AI25)**

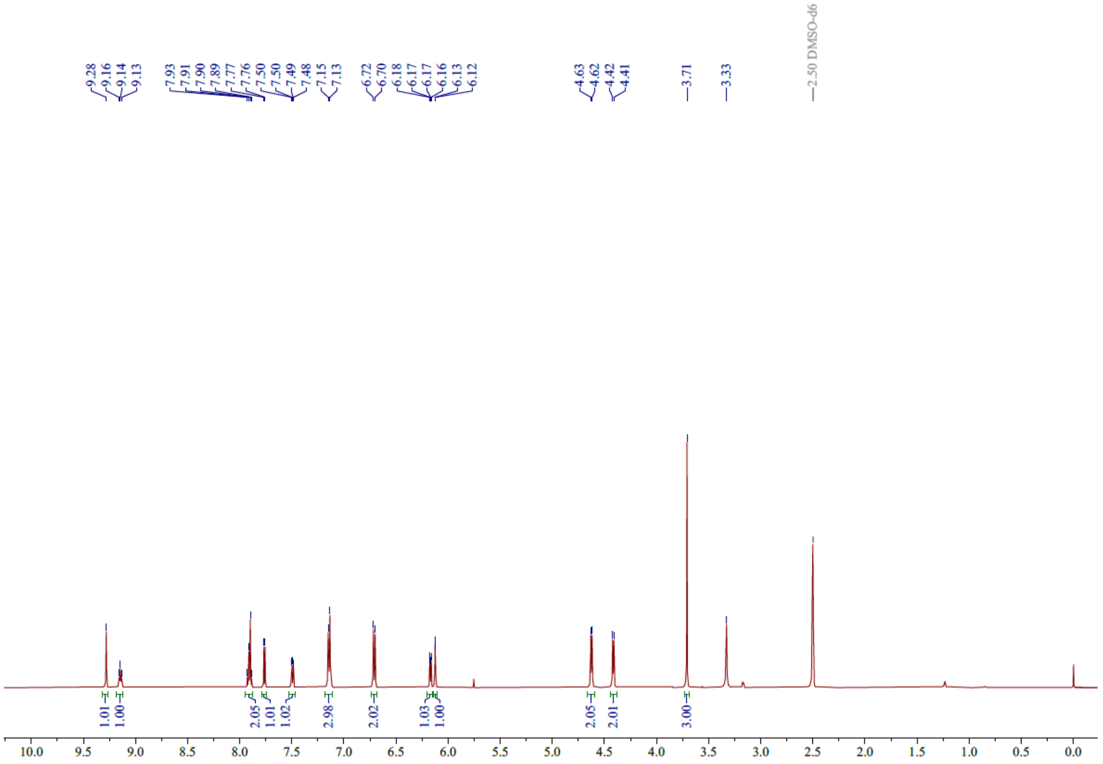

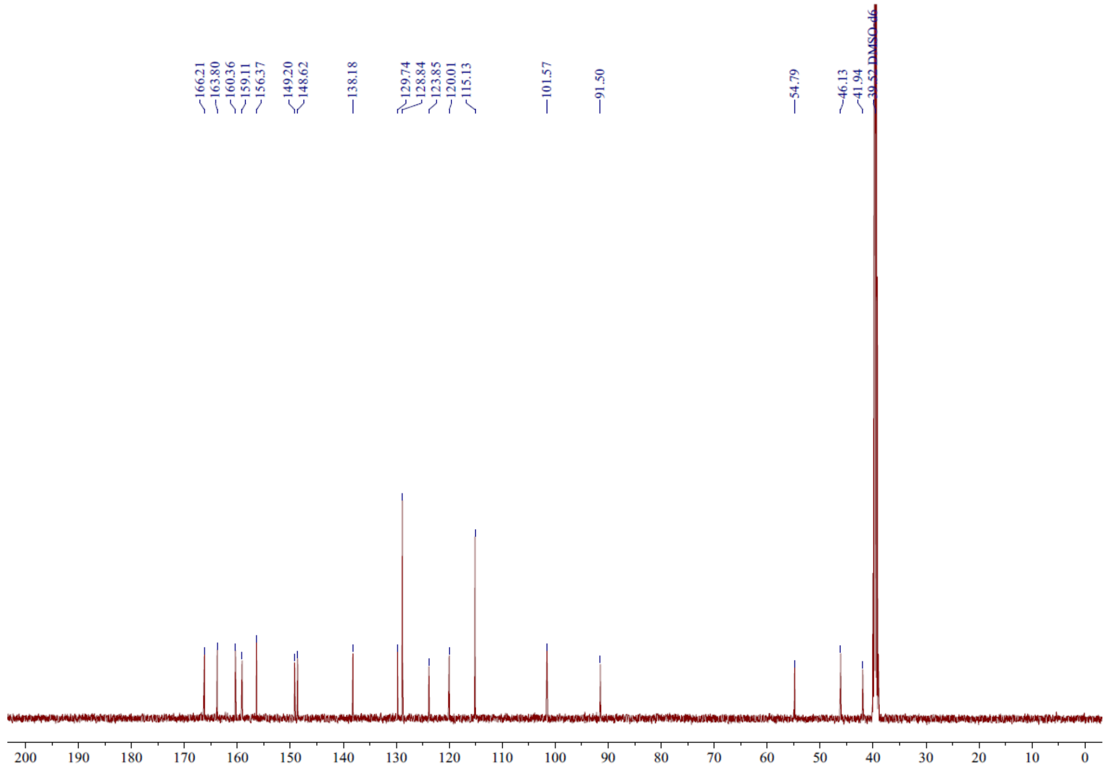

N-(4-hydroxybenzyl)-1-methyl-1H-indole-3-carboxamide **(AI26)**

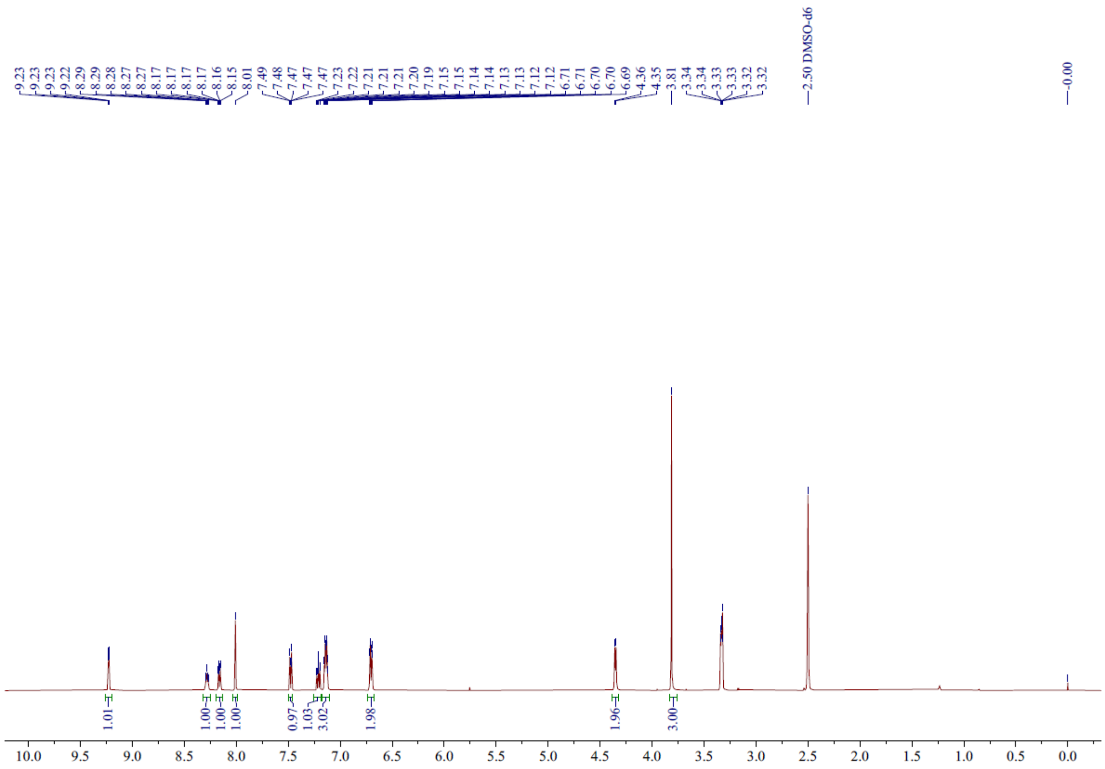

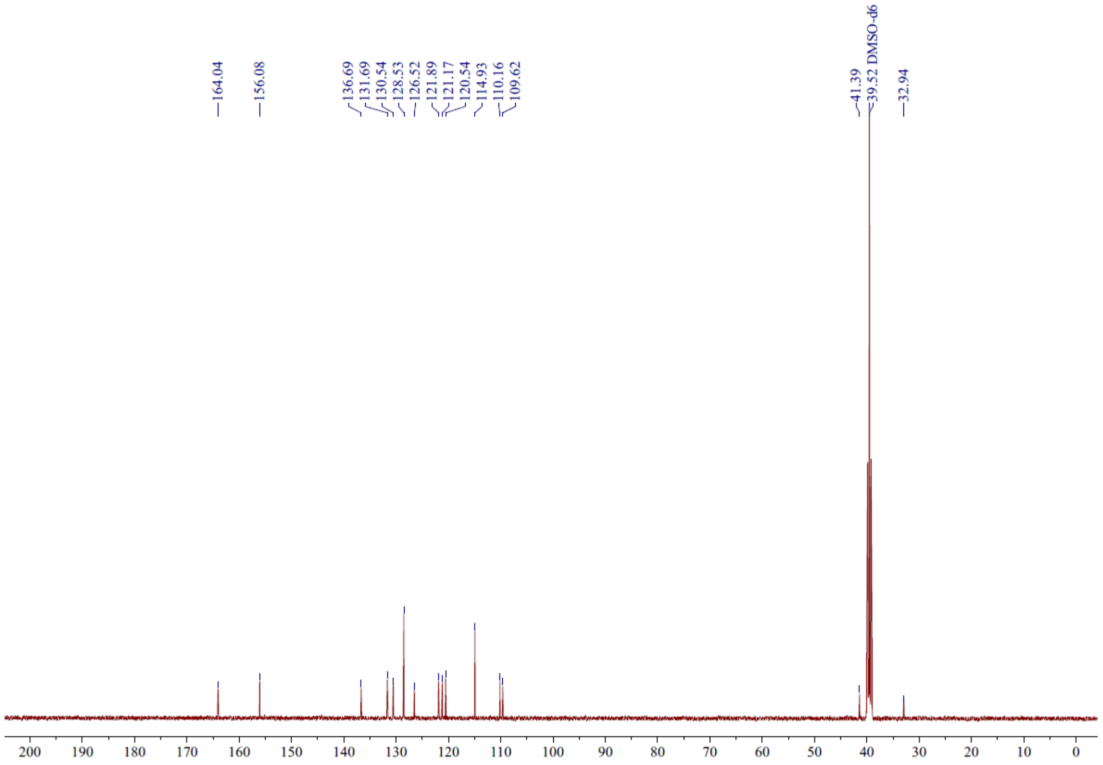

***N-(4-(4-hydroxyphenyl)thiazol-2-yl)-2-(2-methoxyphenyl)acetamide* (AI27)**

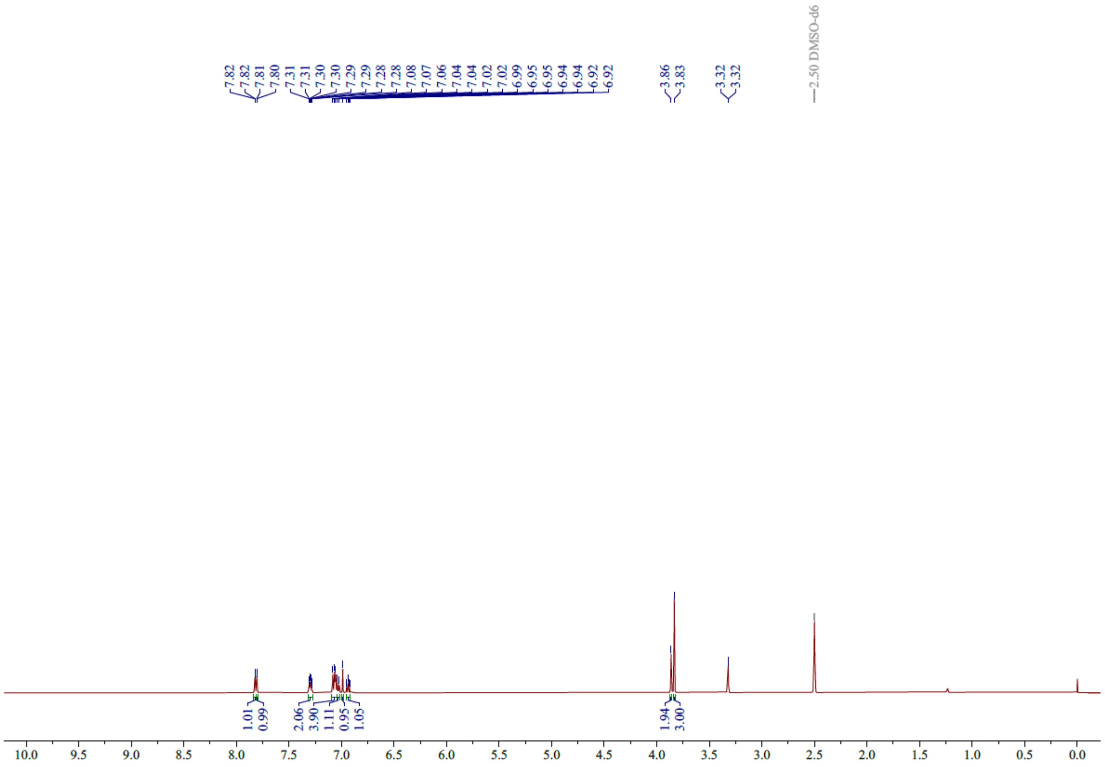

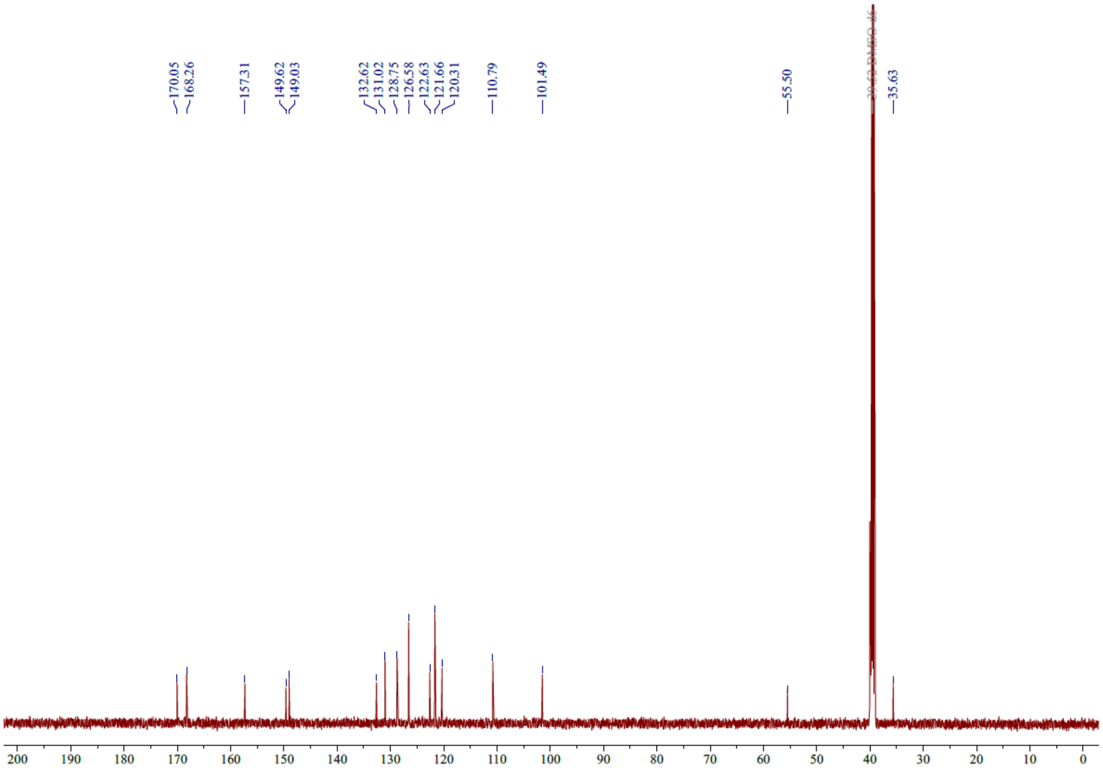

2-(4-fluoro-2-methoxyphenyl)-N-(4-(4-hydroxyphenyl)thiazol-2-yl)acetamide (AI28)

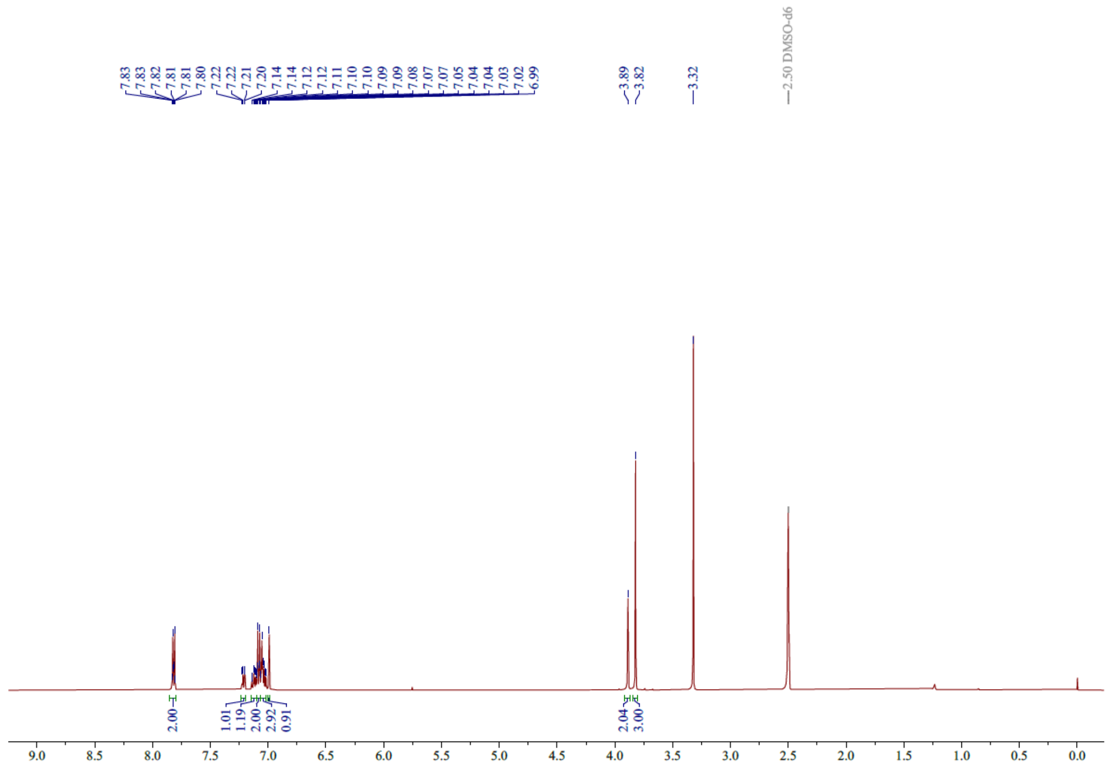

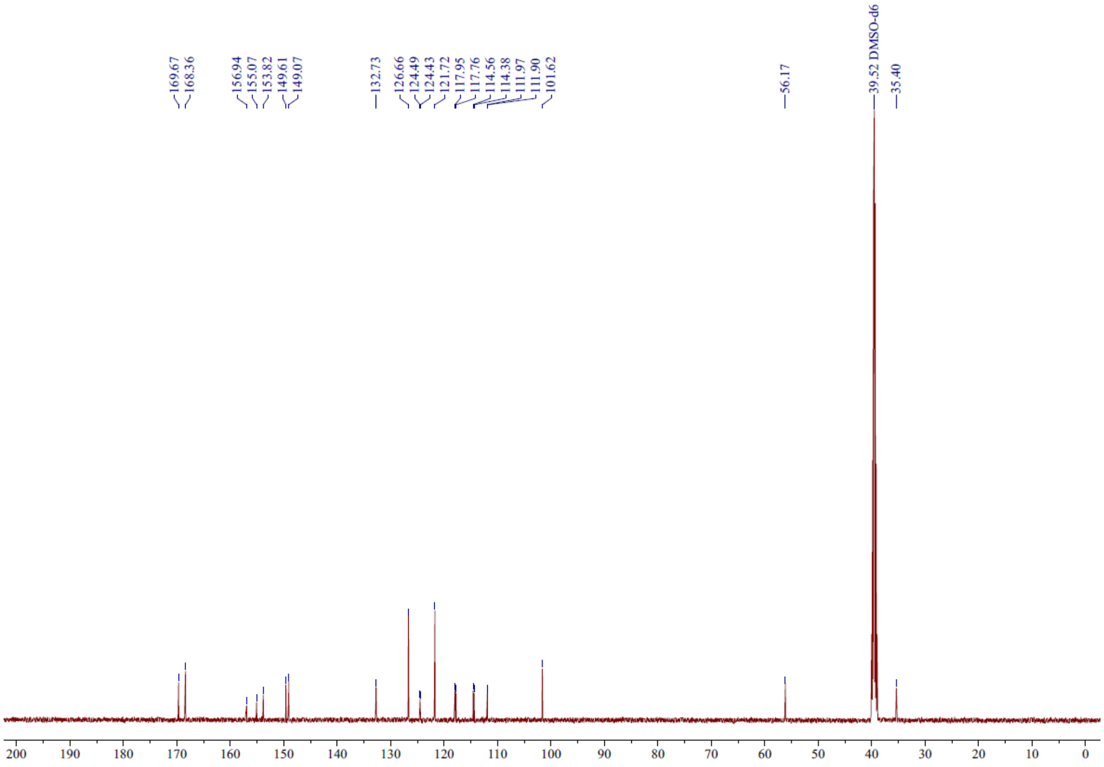

(2-hydroxyphenyl)(4-(4-hydroxyphenyl)piperazin-1-yl)methanone **(AI10-m1)**

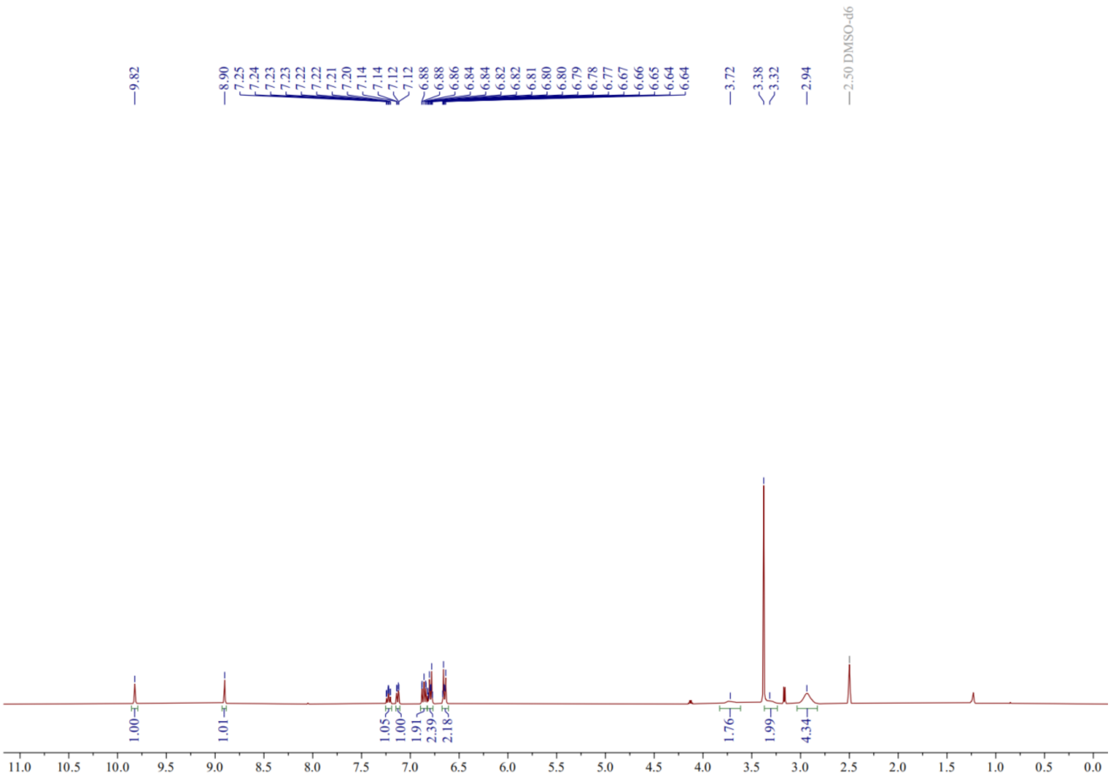

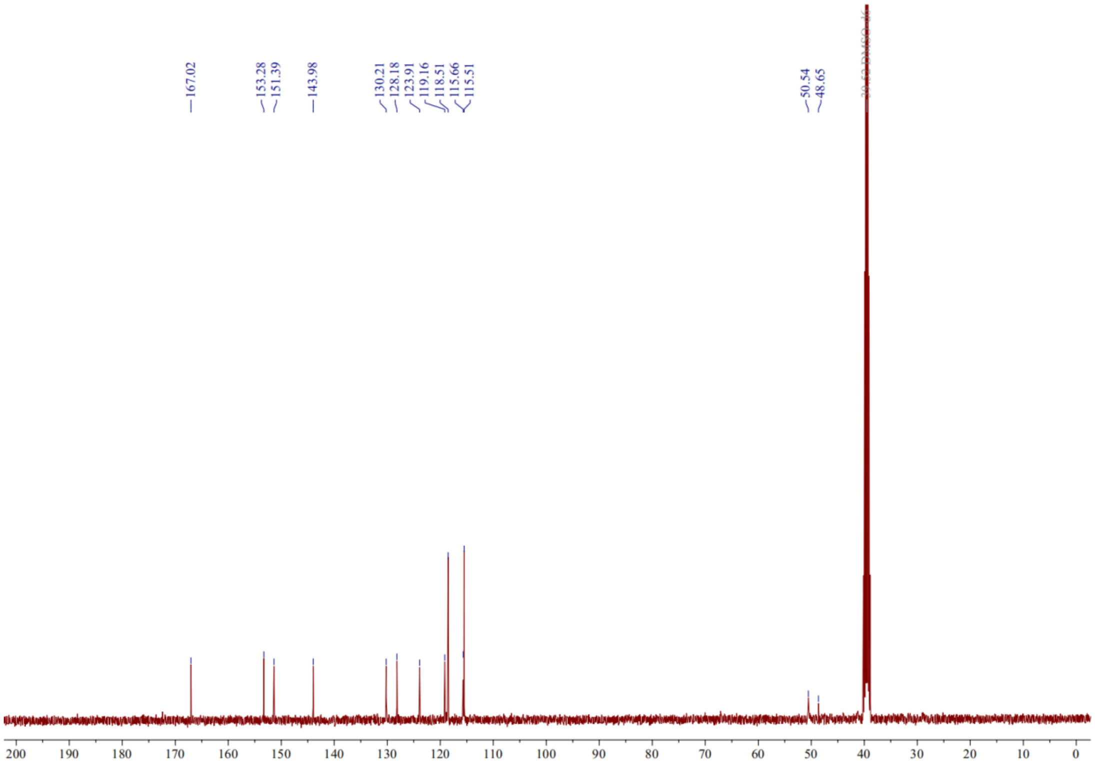

(2-hydroxy-5-methylphenyl)(4-(4-hydroxyphenyl)piperazin-1-yl)methanone **(AI10-m2)**

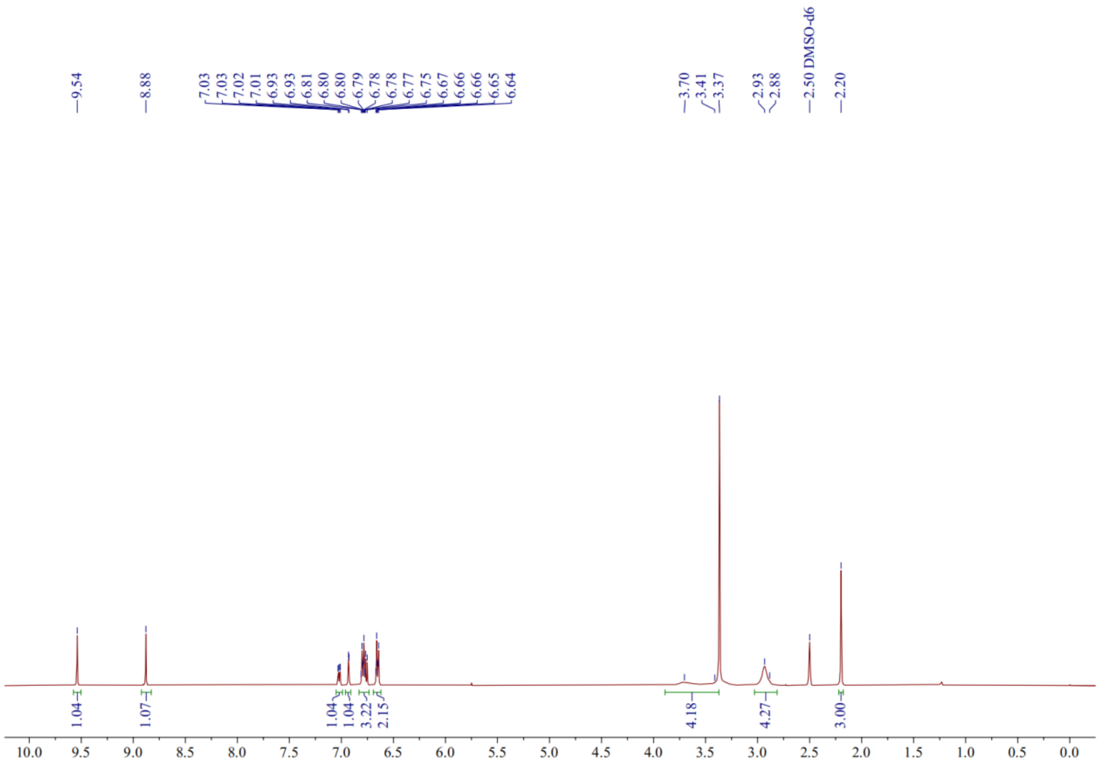

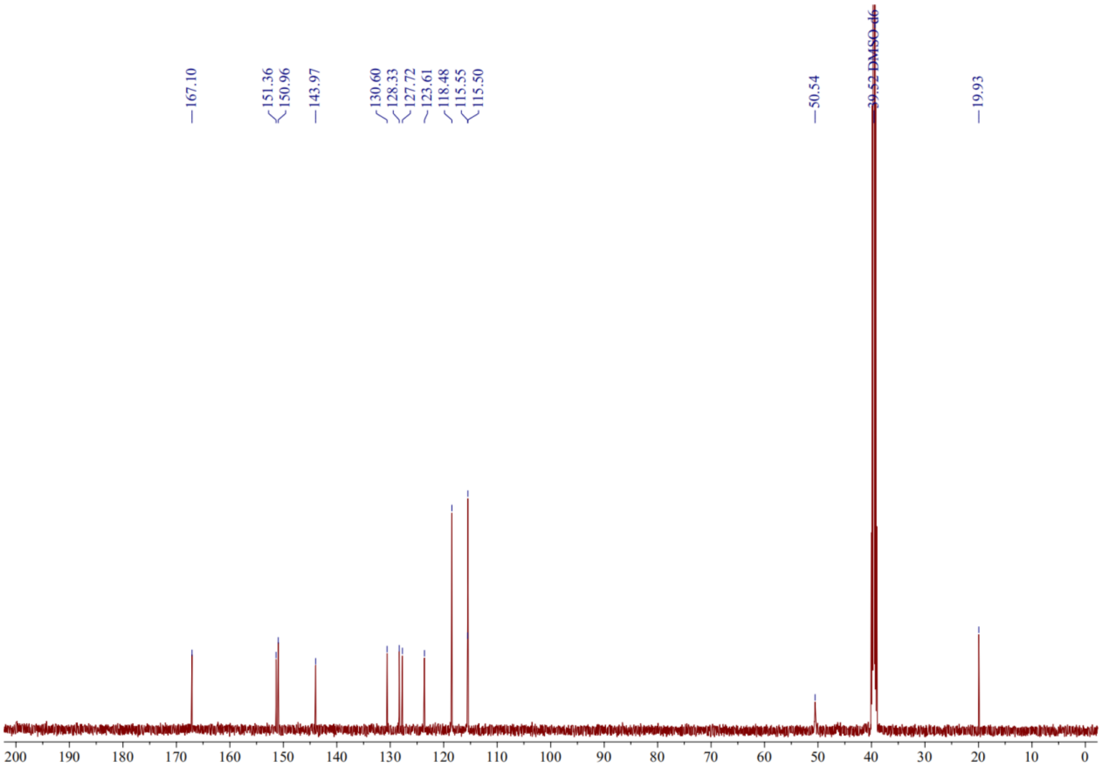

(5-hydroxy-2-methylphenyl)(4-(4-hydroxyphenyl)piperazin-1-yl)methanone **(AI10-m3)**

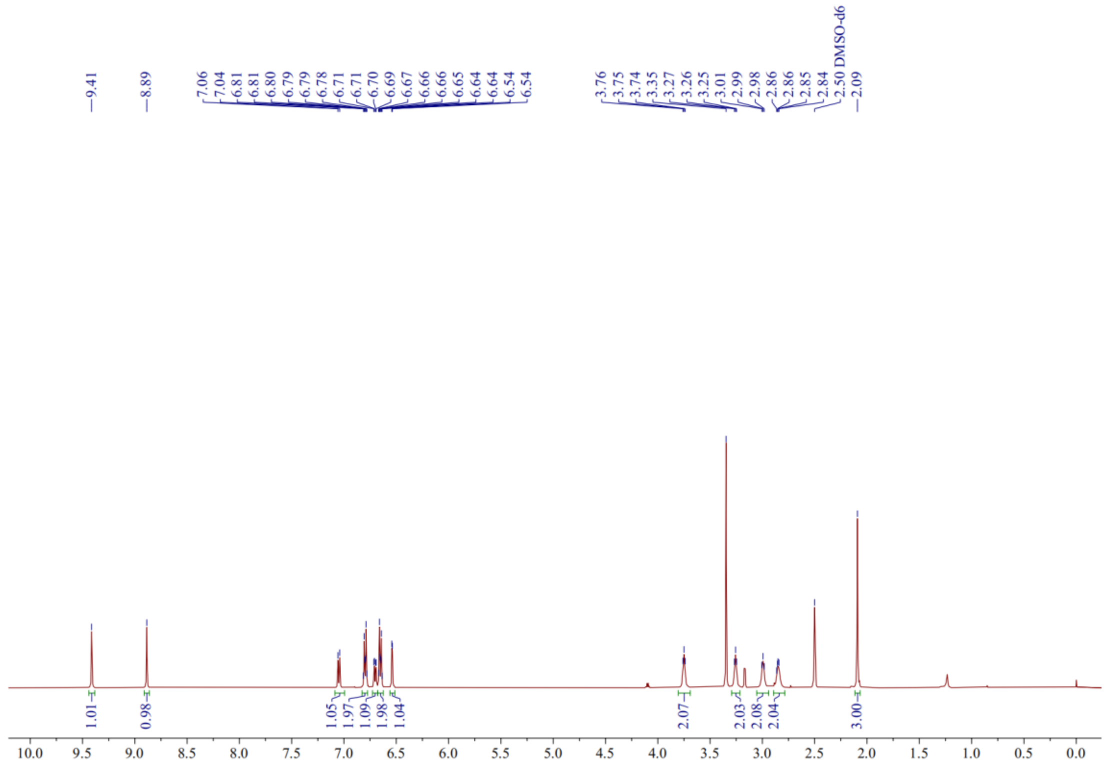

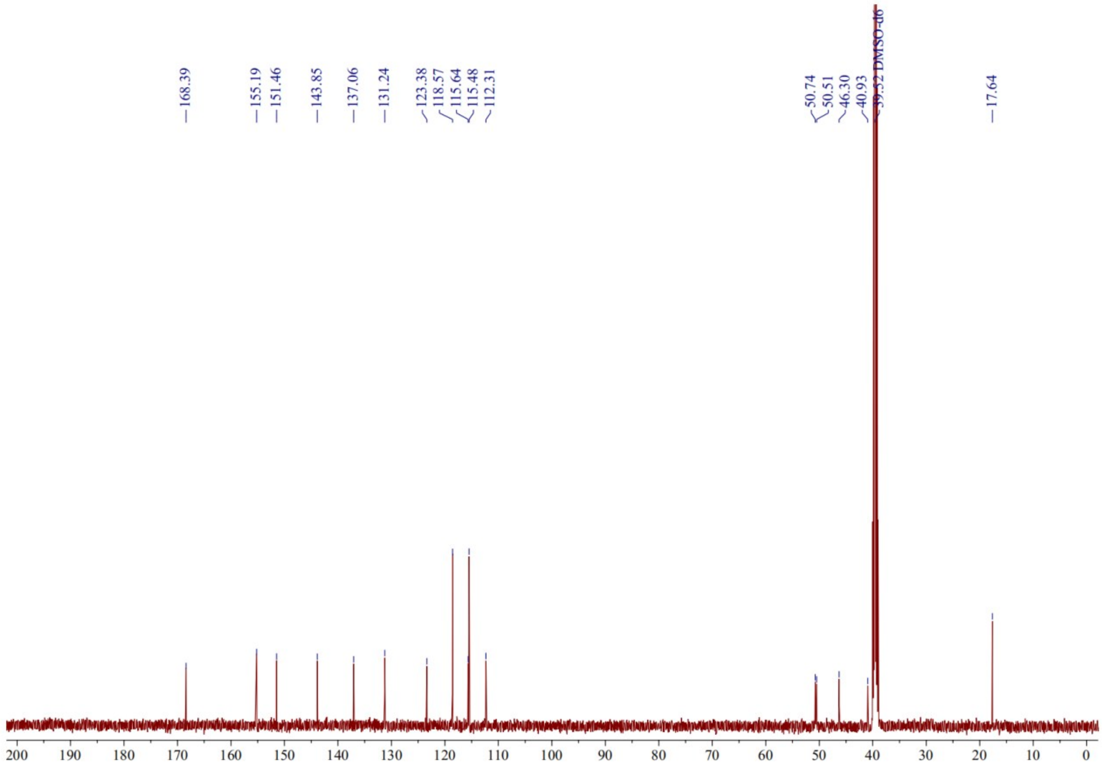

***(3-hydroxy-2-methylphenyl)(4-(4-hydroxyphenyl)piperazin-1-yl)methanone* (AI10-m4)**

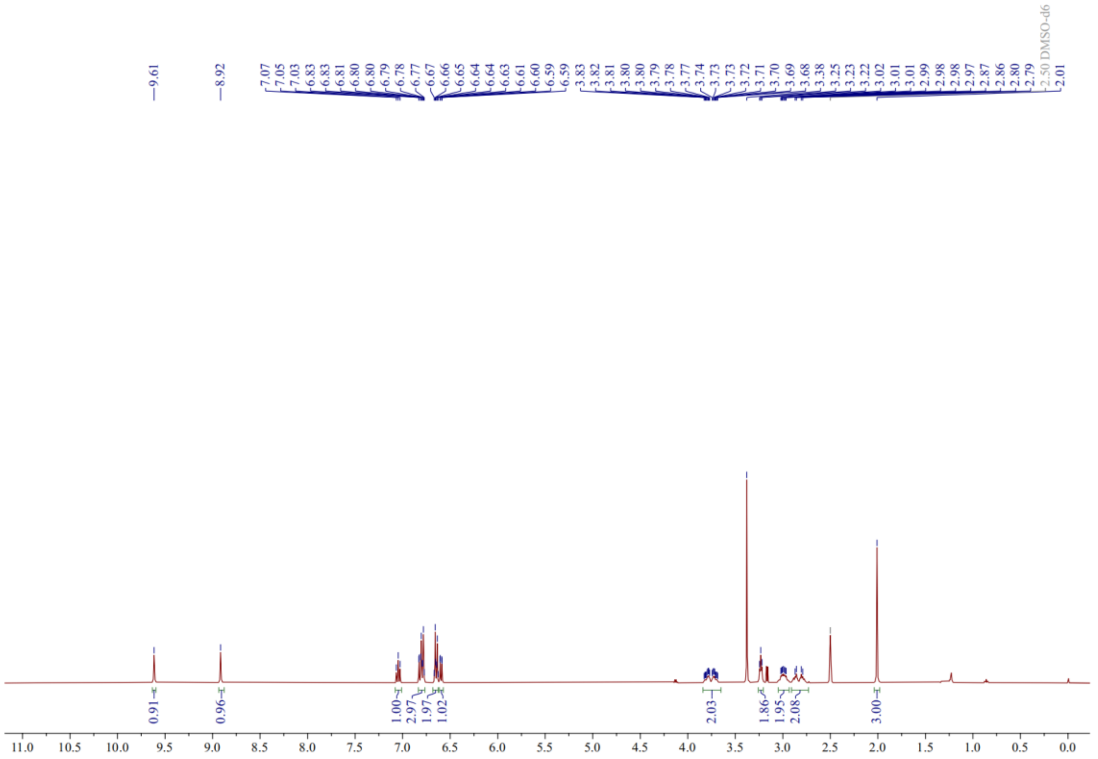

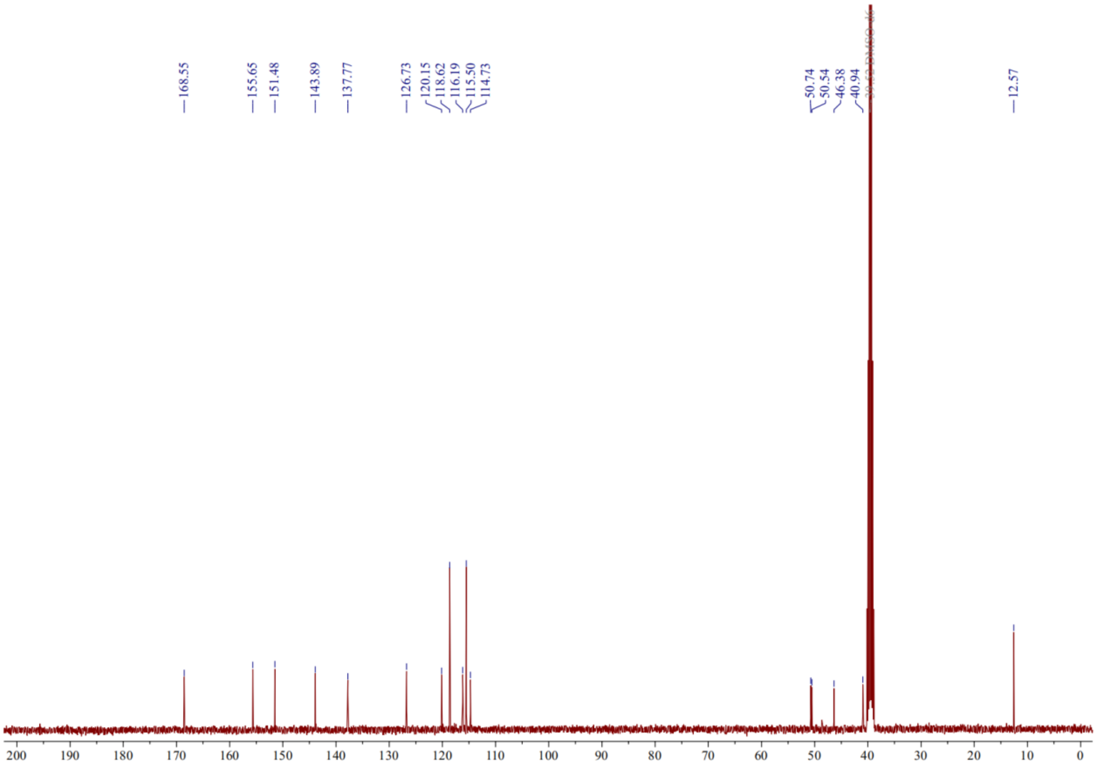

(4-fluorophenyl)(4-(4-hydroxyphenyl)piperazin-1-yl)methanone **(AI10-m5)**

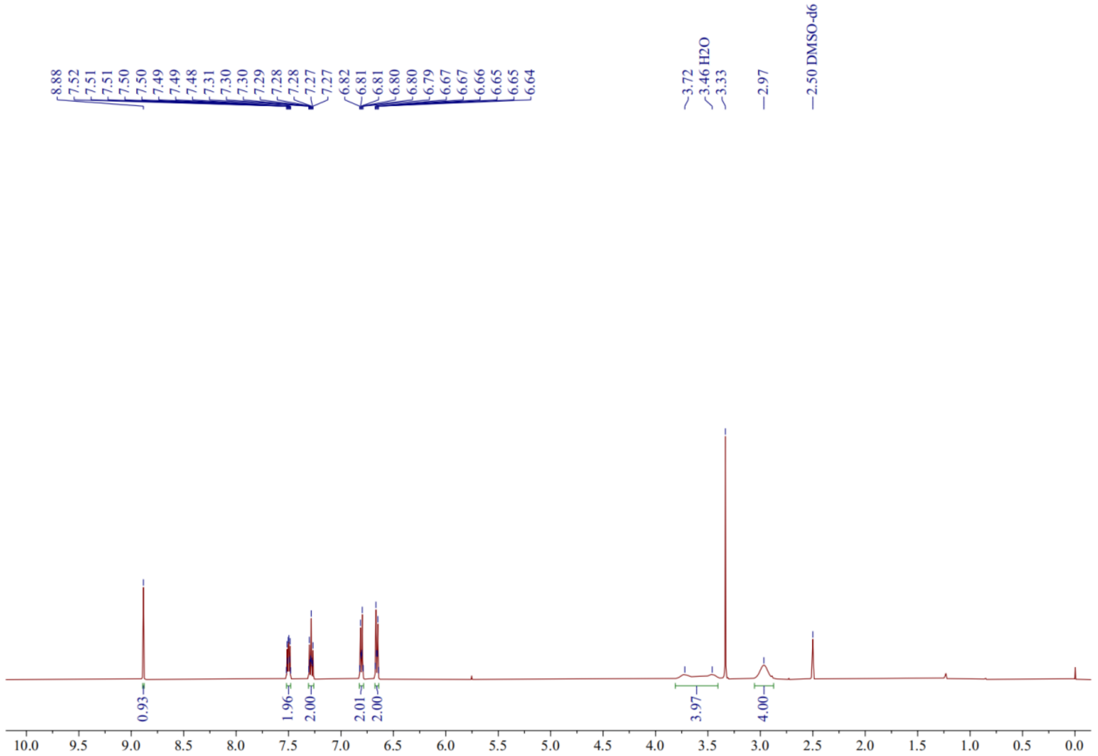

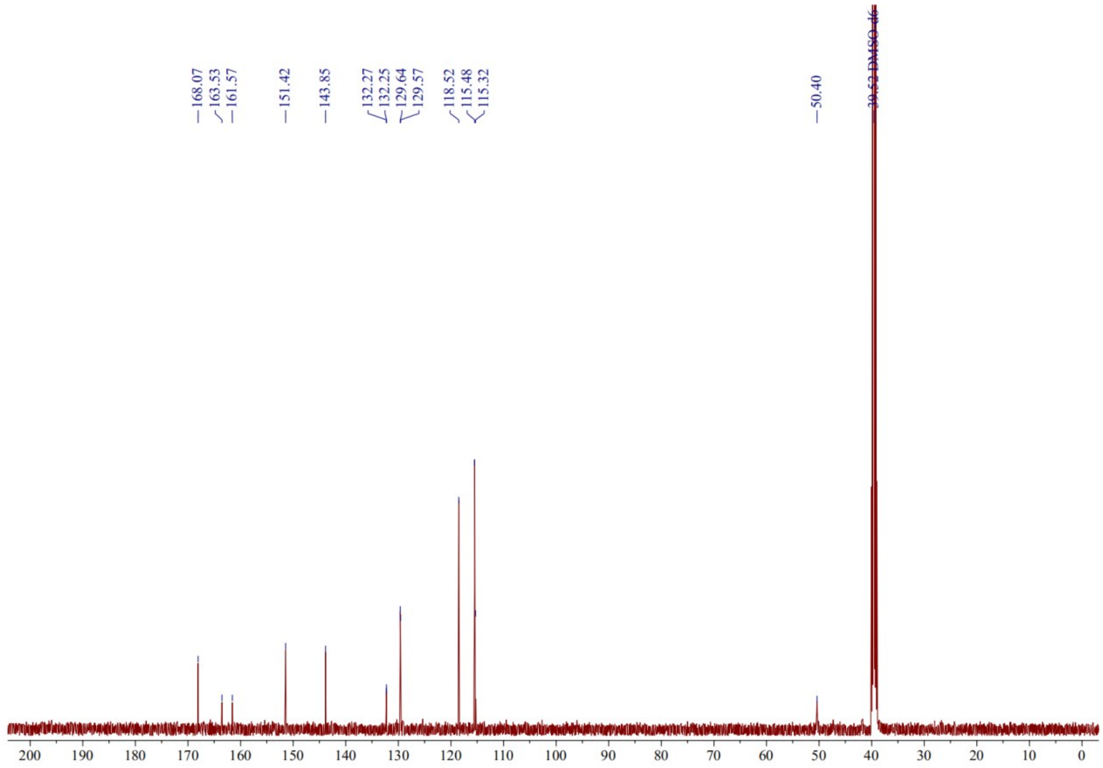

(4-(4-hydroxyphenyl)piperazin-1-yl)(3,4,5-trimethoxyphenyl)methanone **(AI10-m6)**

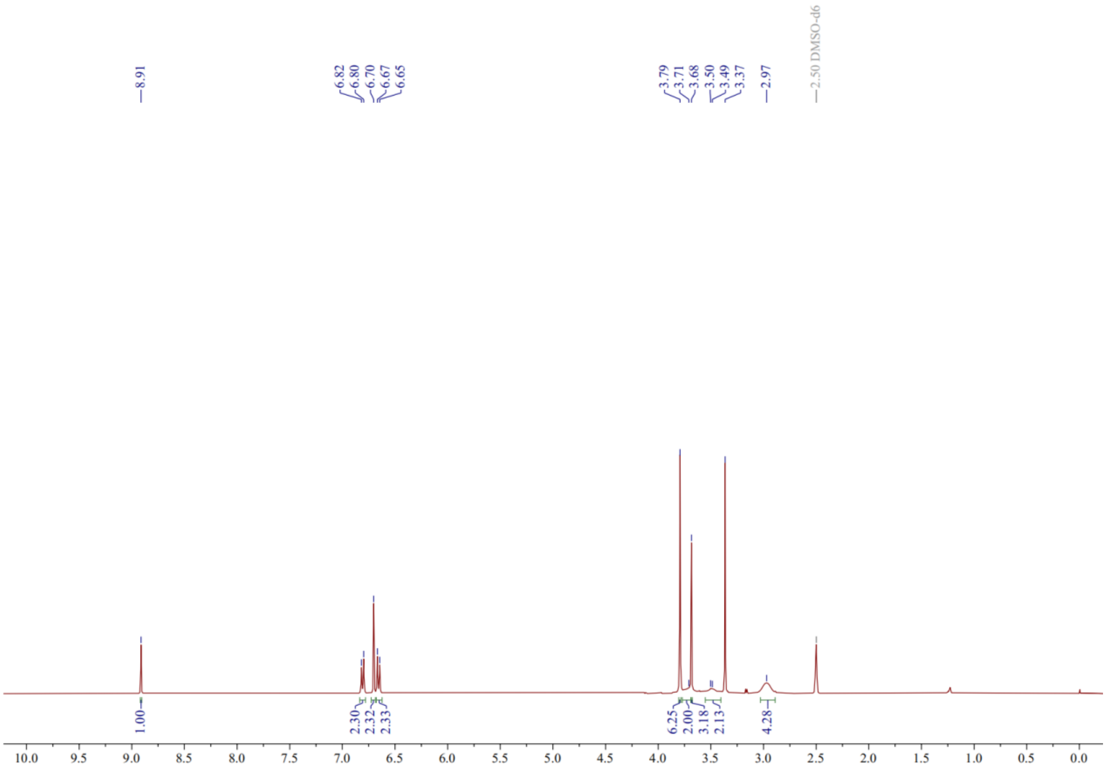

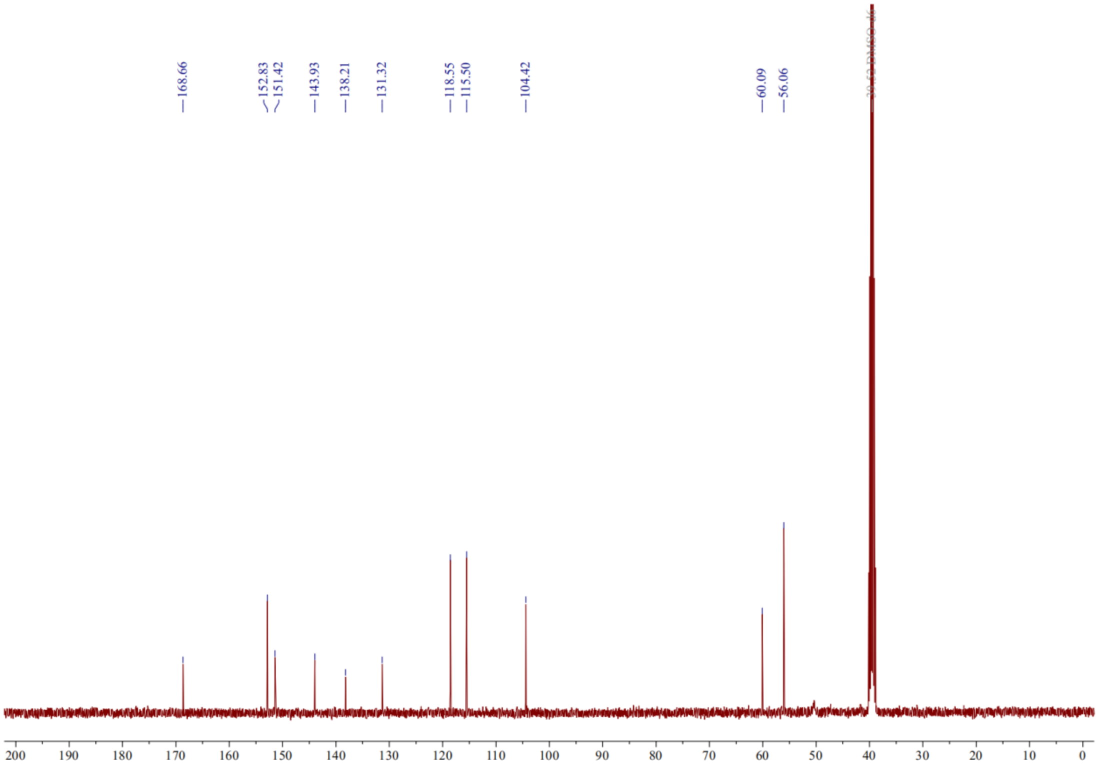

(4-(4-hydroxyphenyl)piperazin-1-yl)(pyridin-3-yl)methanone **(AI10-m7)**

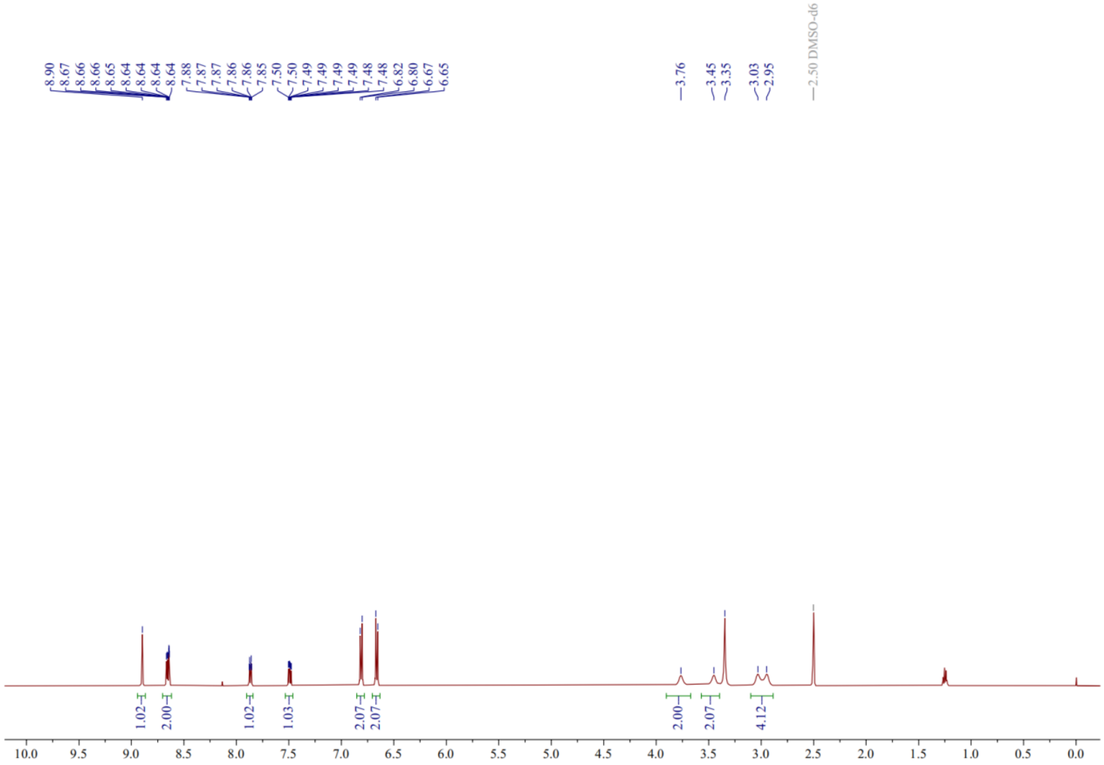

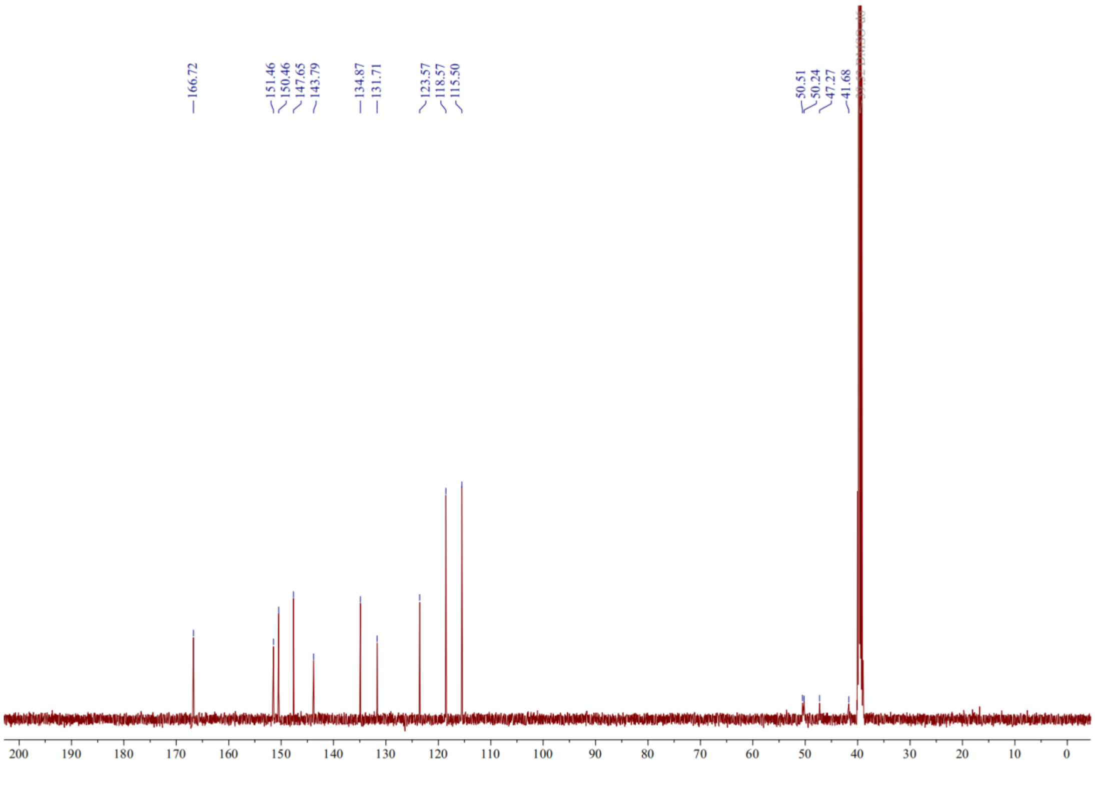

***(4-(4-hydroxyphenyl)piperazin-1-yl)(pyridin-2-yl)methanone* (AI10-m8)**

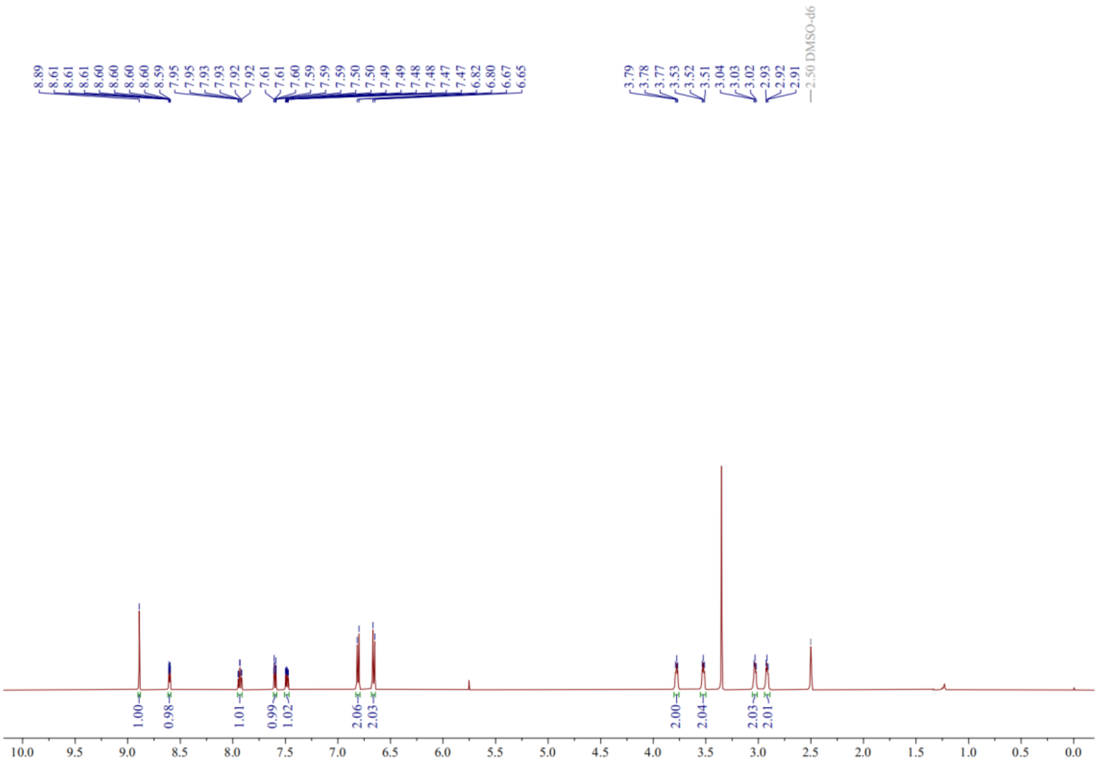

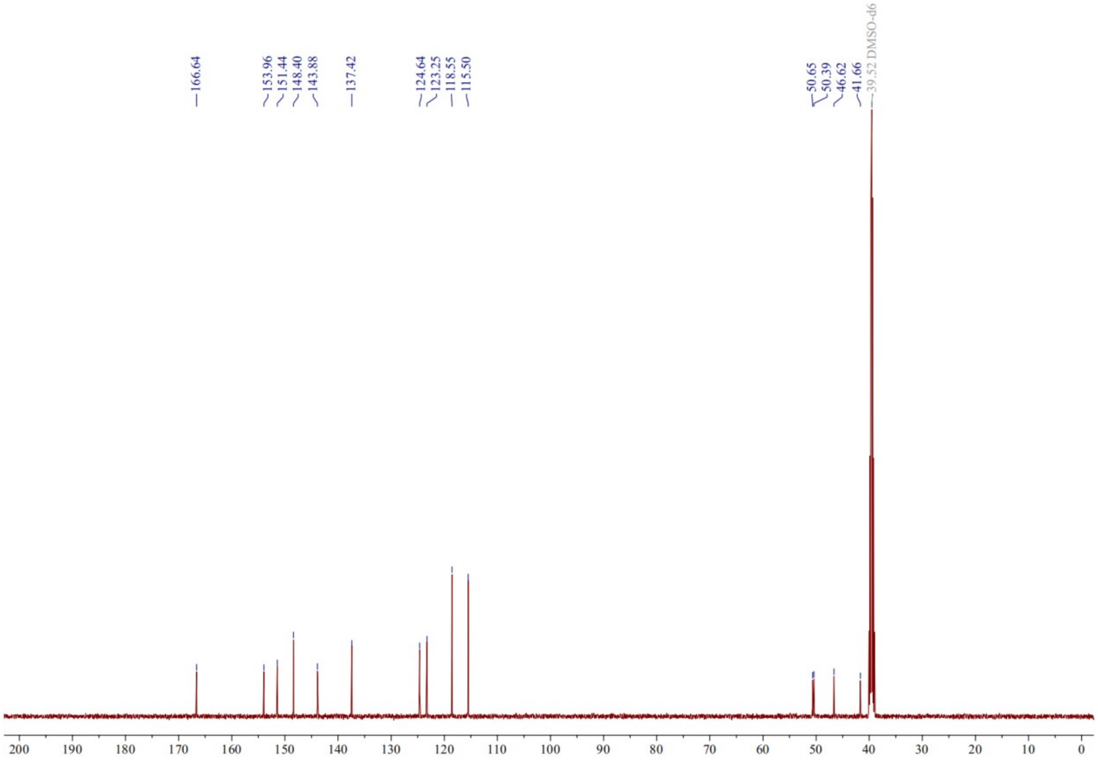

(4-(4-hydroxyphenyl)piperazin-1-yl)(pyridin-4-yl)methanone **(AI10-m9)**

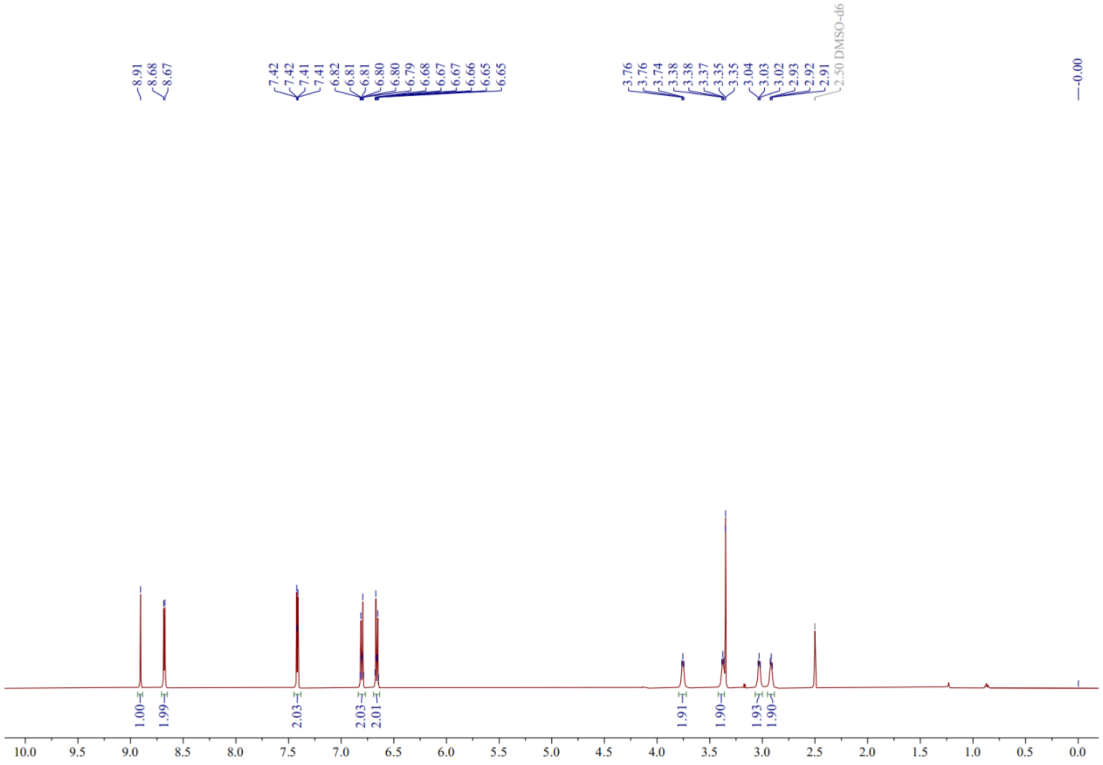

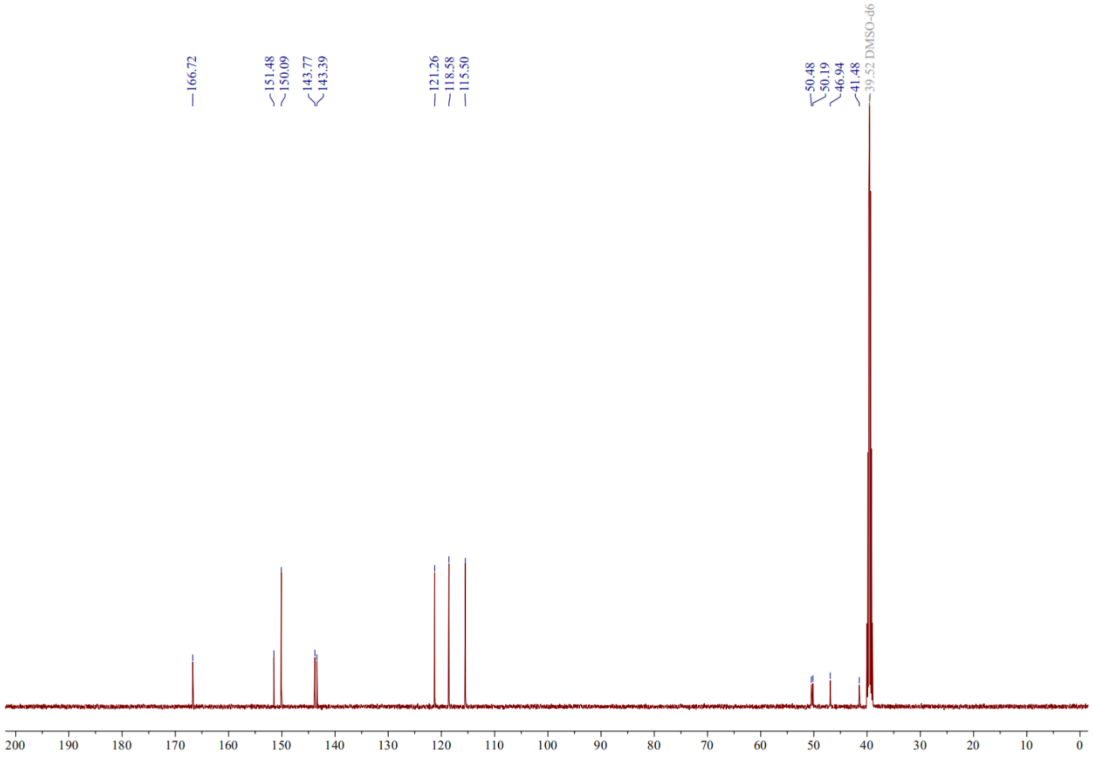

(2,6-dimethylpyridin-4-yl)(4-(2-hydroxyphenyl)piperazin-1-yl)methanone **(AI10-m10)**

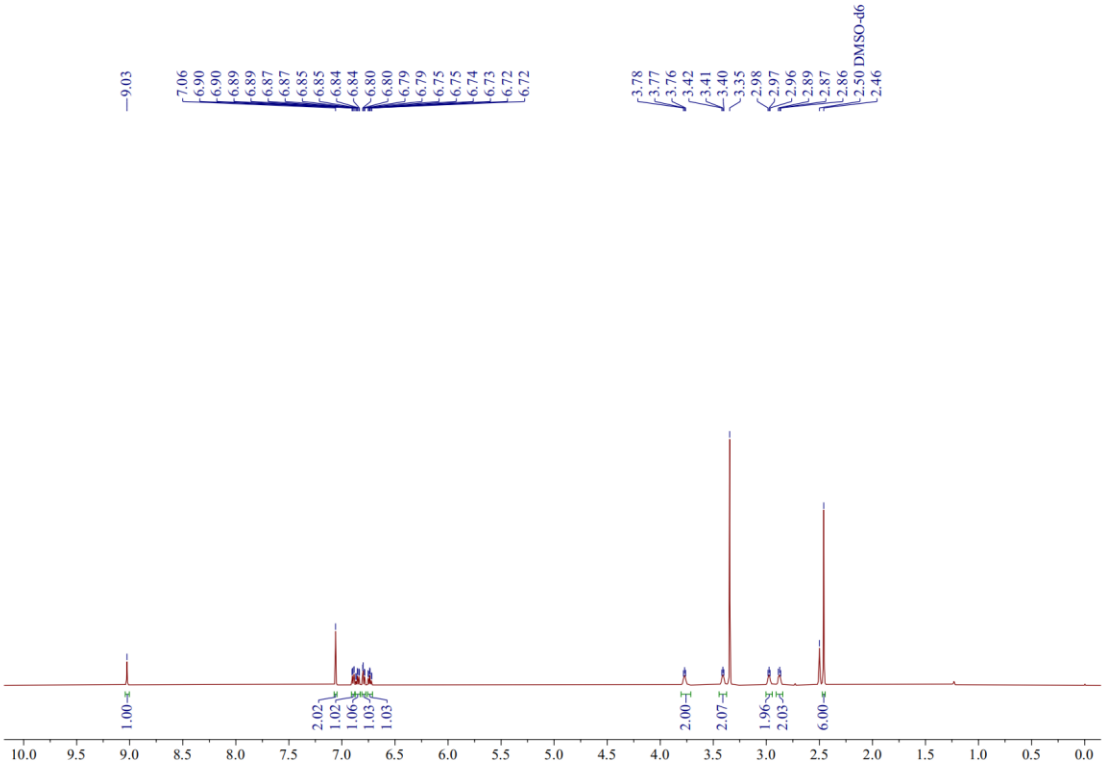

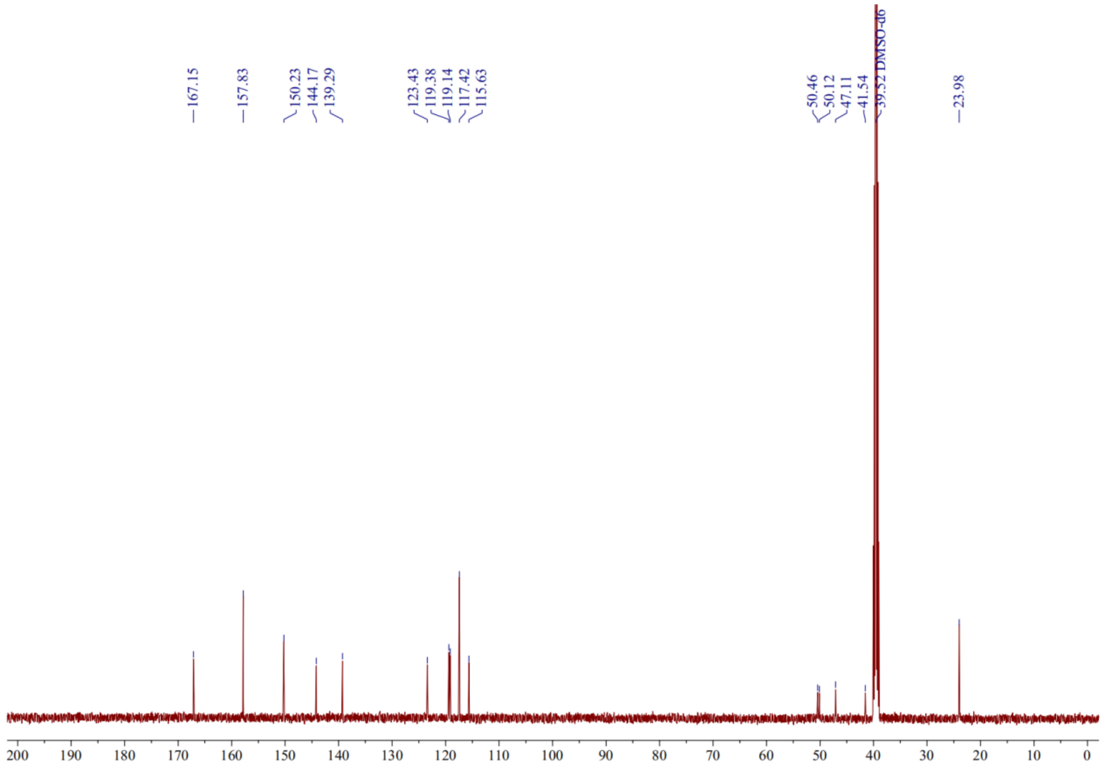

(2,6-dimethylpyridin-4-yl)(4-(3-hydroxyphenyl)piperazin-1-yl)methanone **(AI10-m11)**

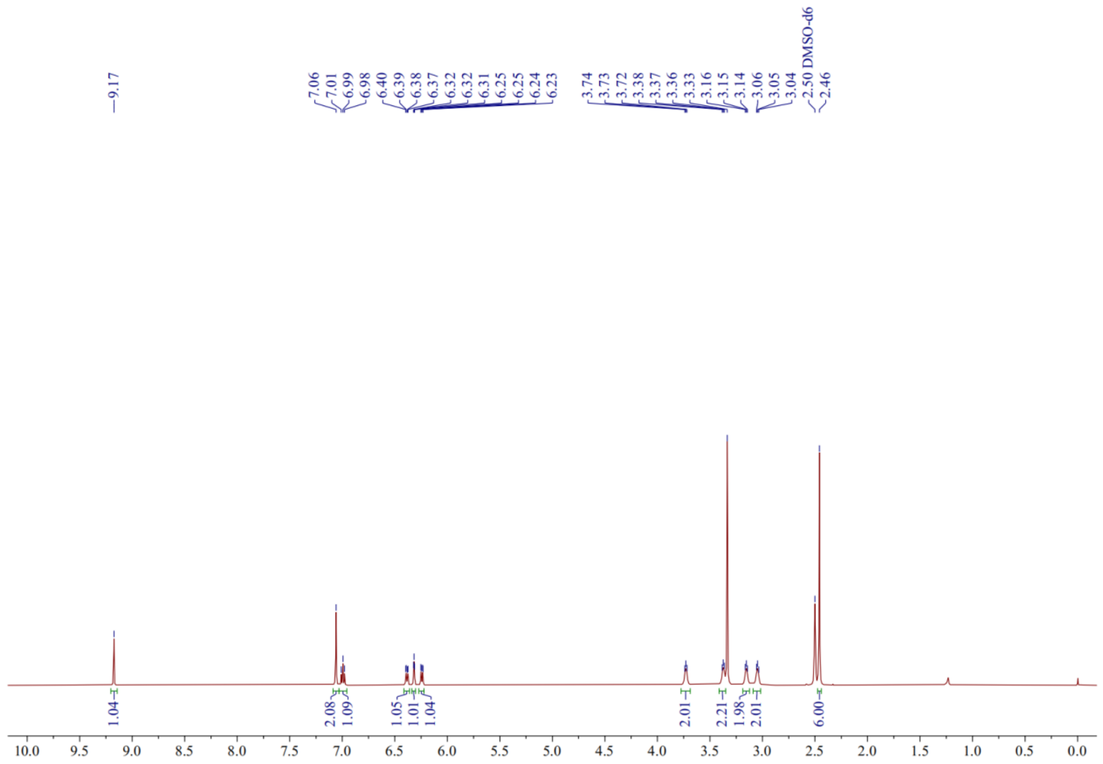

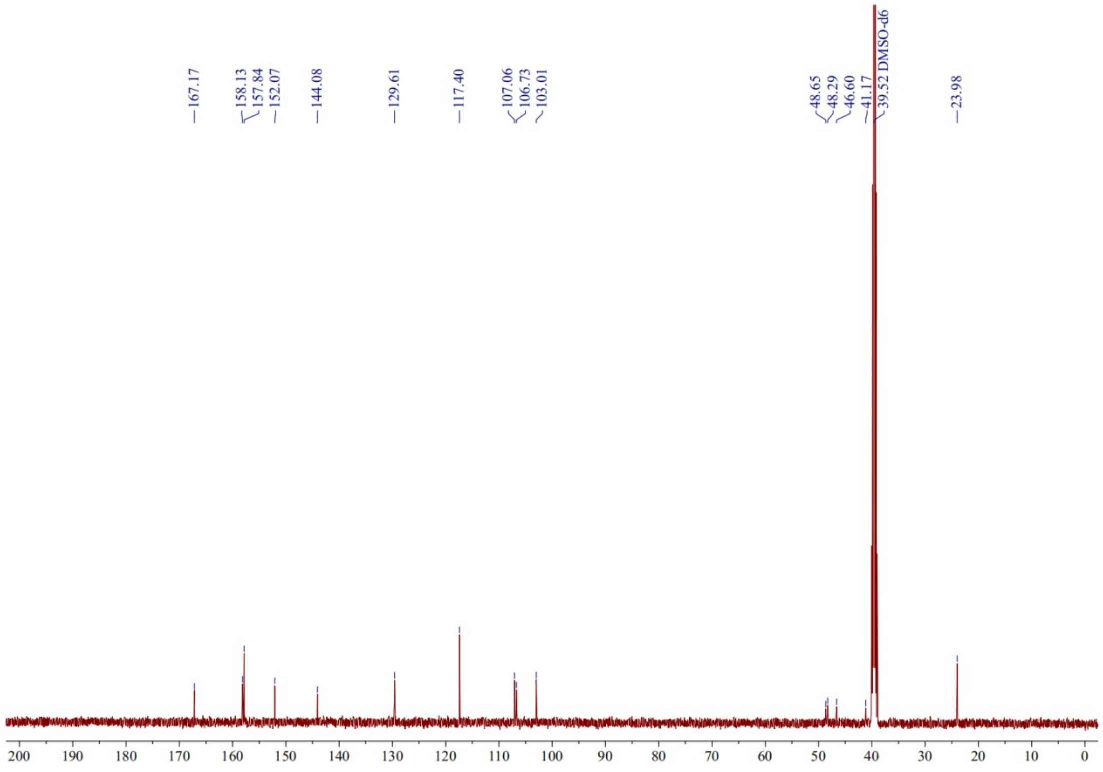

(4-(2,4-dihydroxyphenyl)piperazin-1-yl)(2,6-dimethylpyridin-4-yl)methanone **(AI10-m12)**

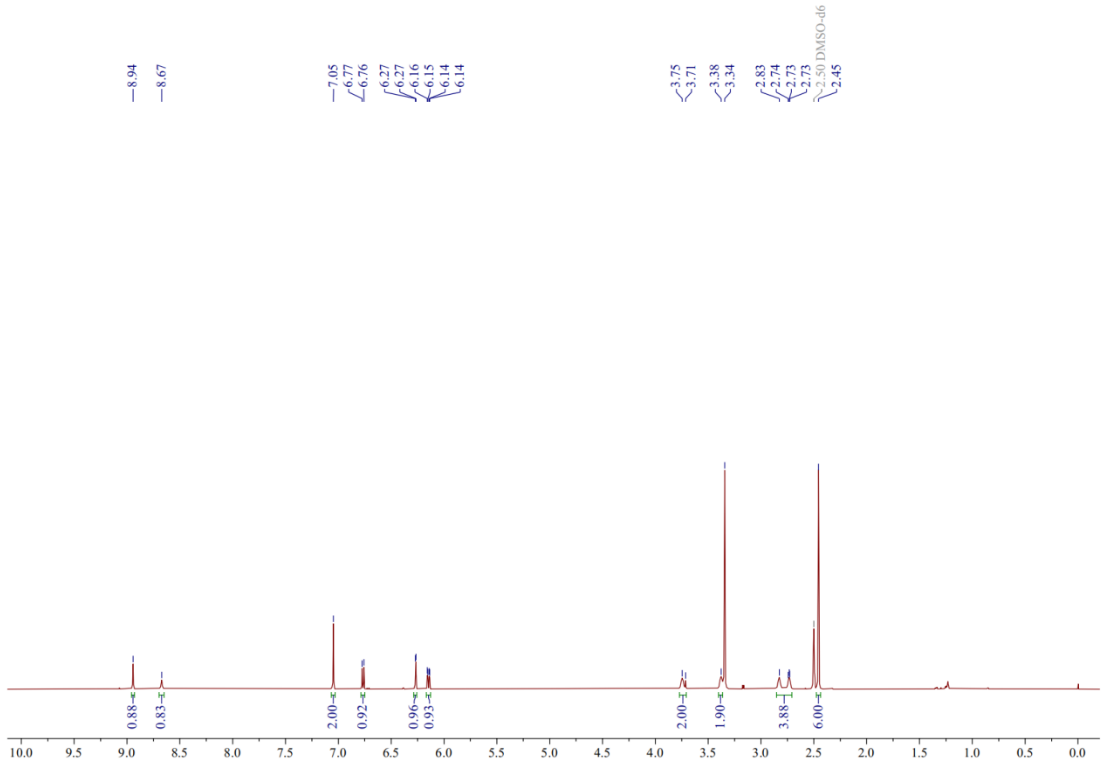

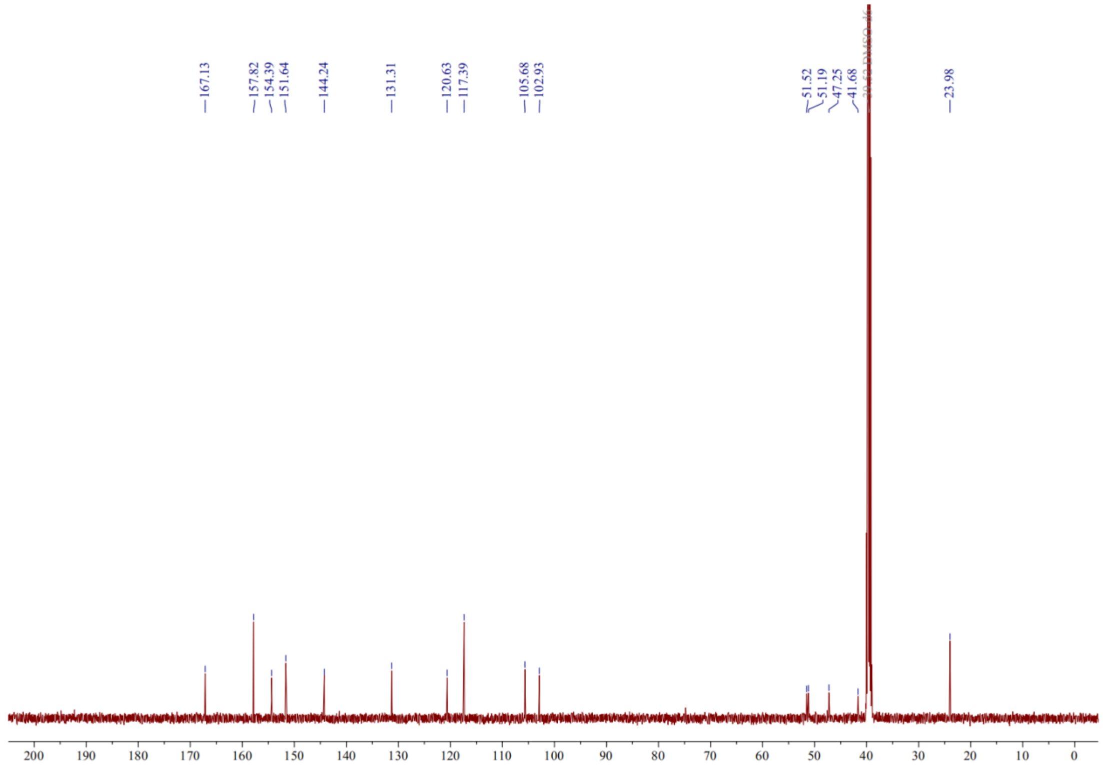

2-(4-fluorophenyl)-1-(4-(4-hydroxyphenyl)piperazin-1-yl)ethan-1-one **(AI10-m13)**

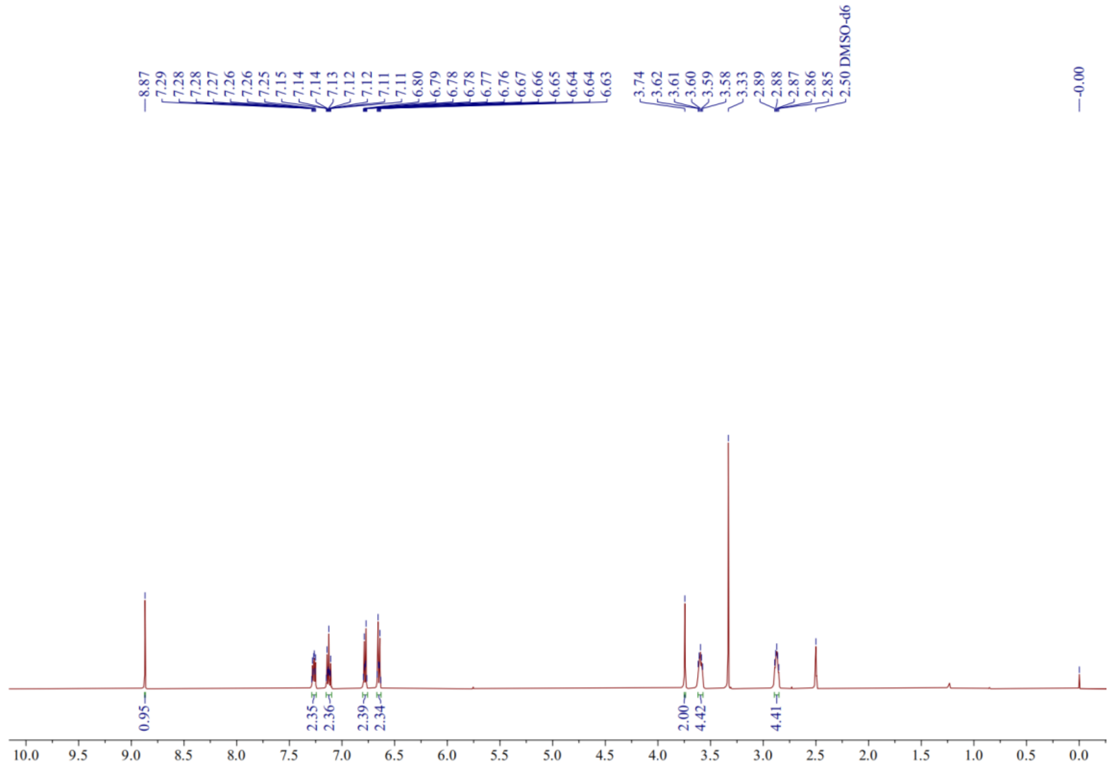

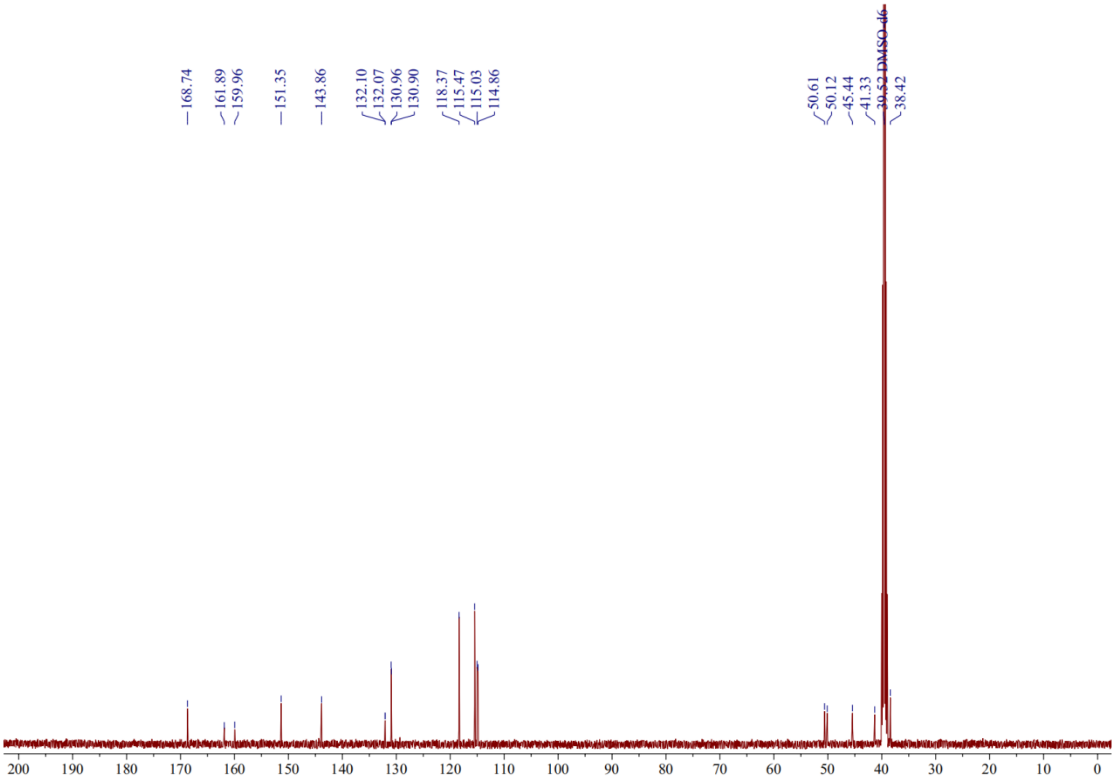

1-(4-(4-hydroxyphenyl)piperazin-1-yl)-2-(2-methoxyphenyl)ethan-1-one **(AI10-m14)**

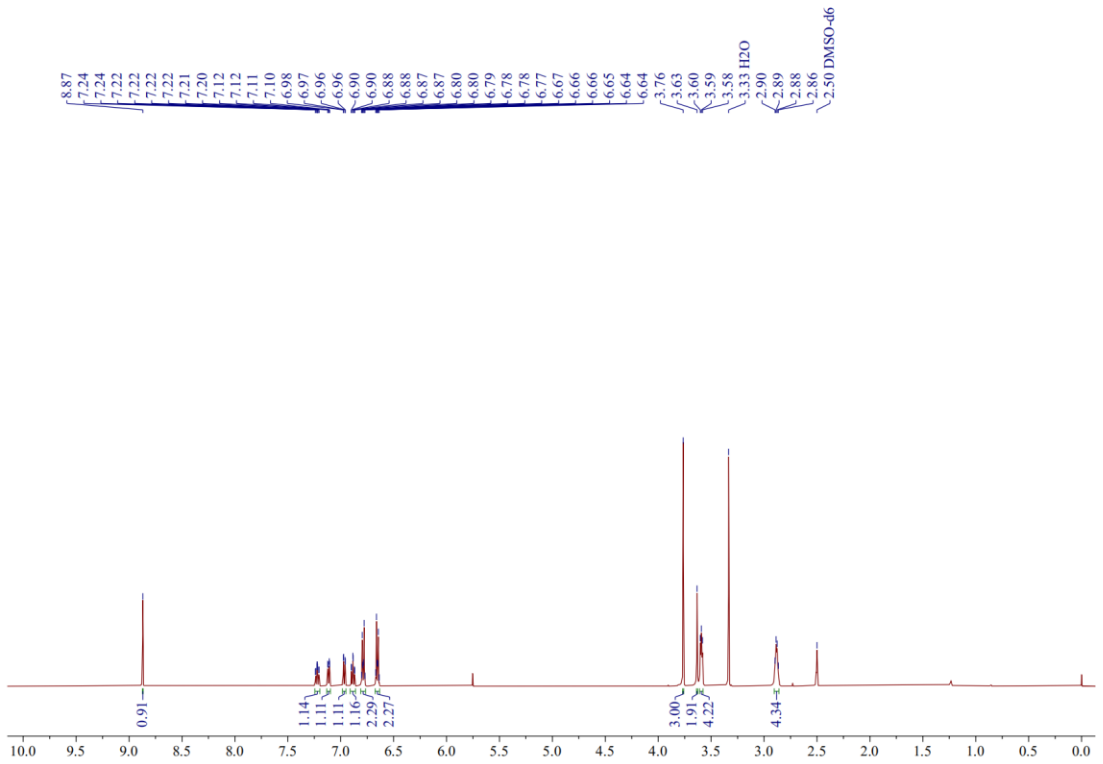

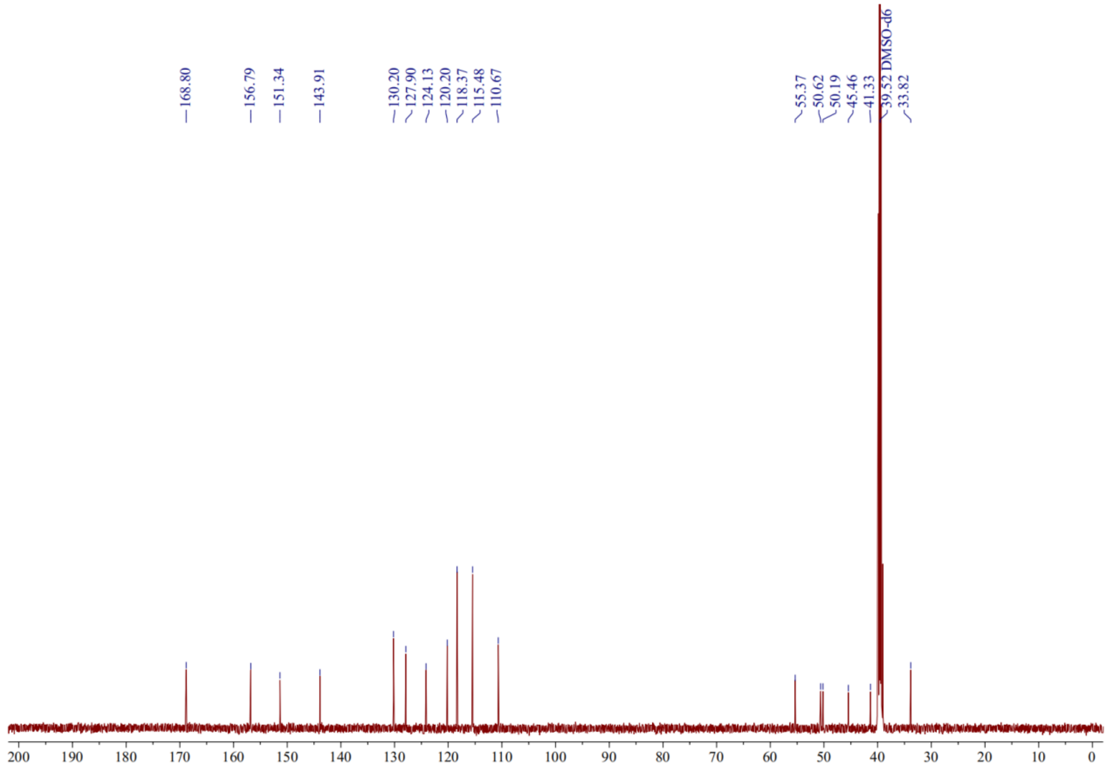

***(E)-3-(2,4-dihydroxyphenyl)-1-(4-(4-hydroxyphenyl)piperazin-1-yl)prop-2-en-1-one* (AI10-m15)**

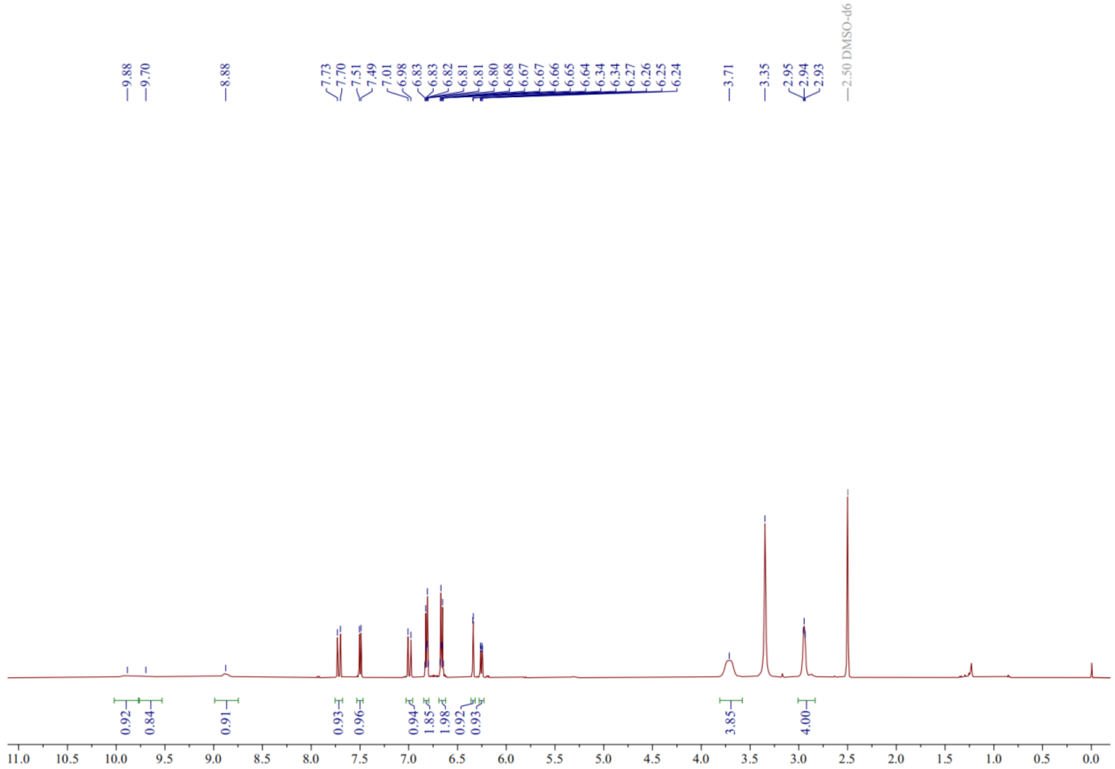

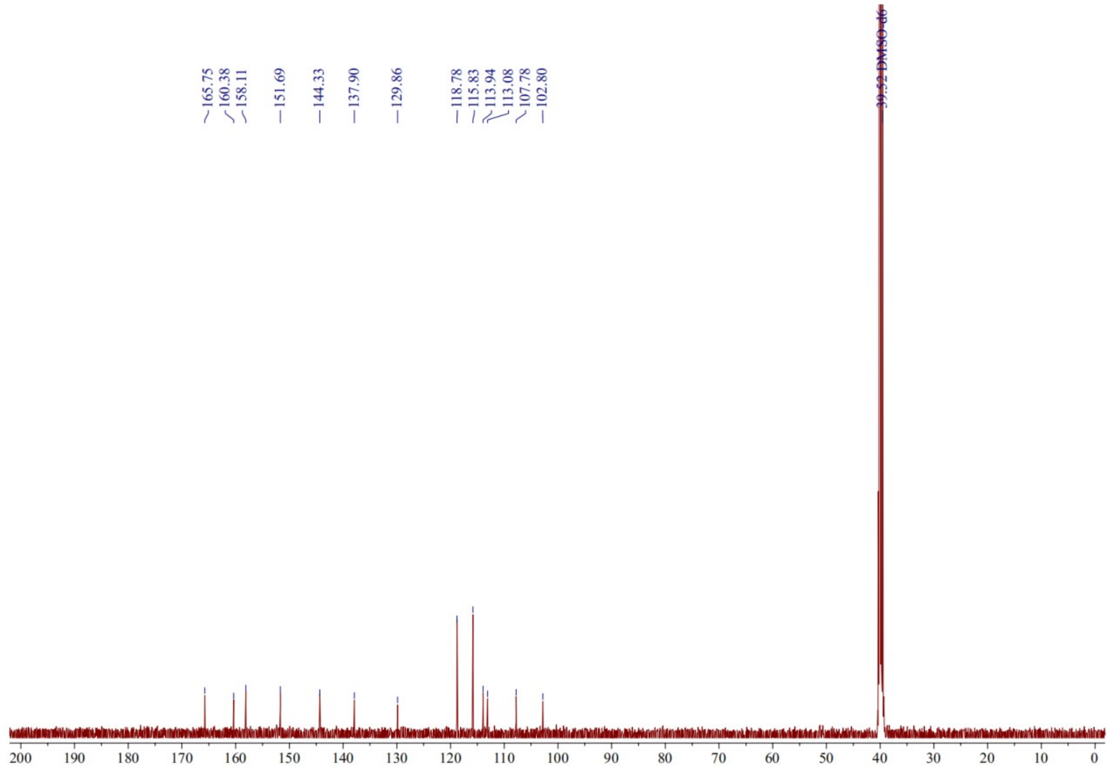

(E)-3-(3,4-dihydroxyphenyl)-1-(4-(4-hydroxyphenyl)piperazin-1-yl)prop-2-en-1-one **(AI10-m16)**

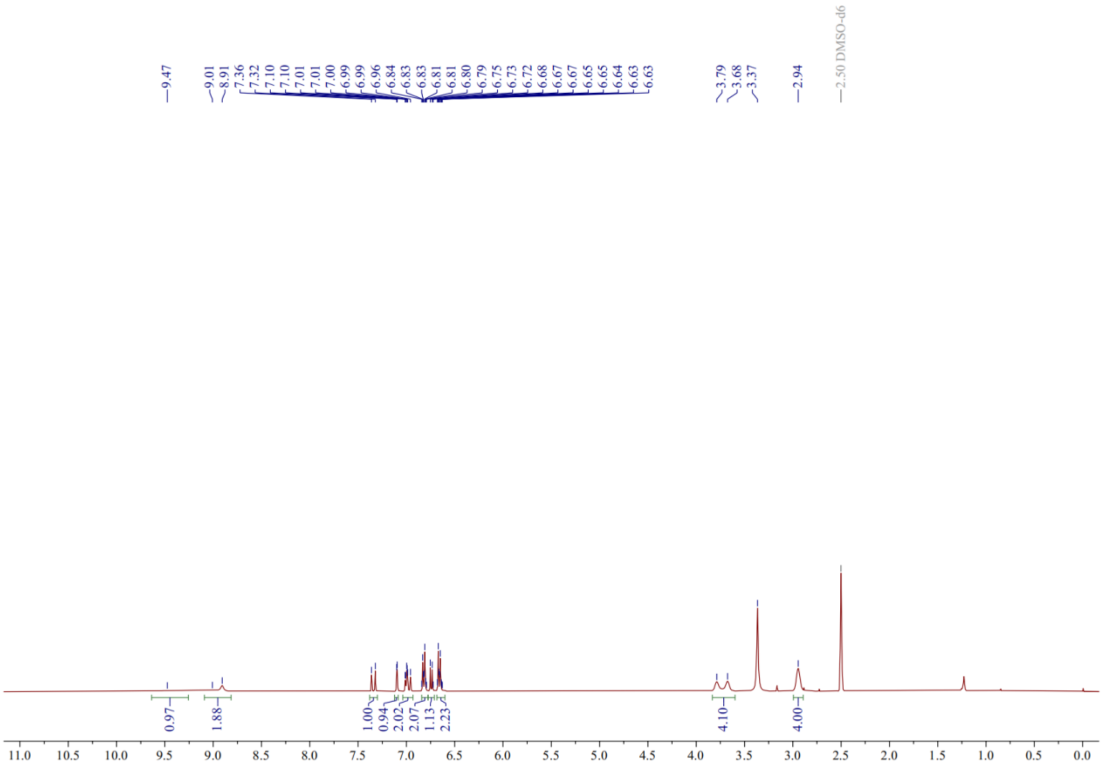

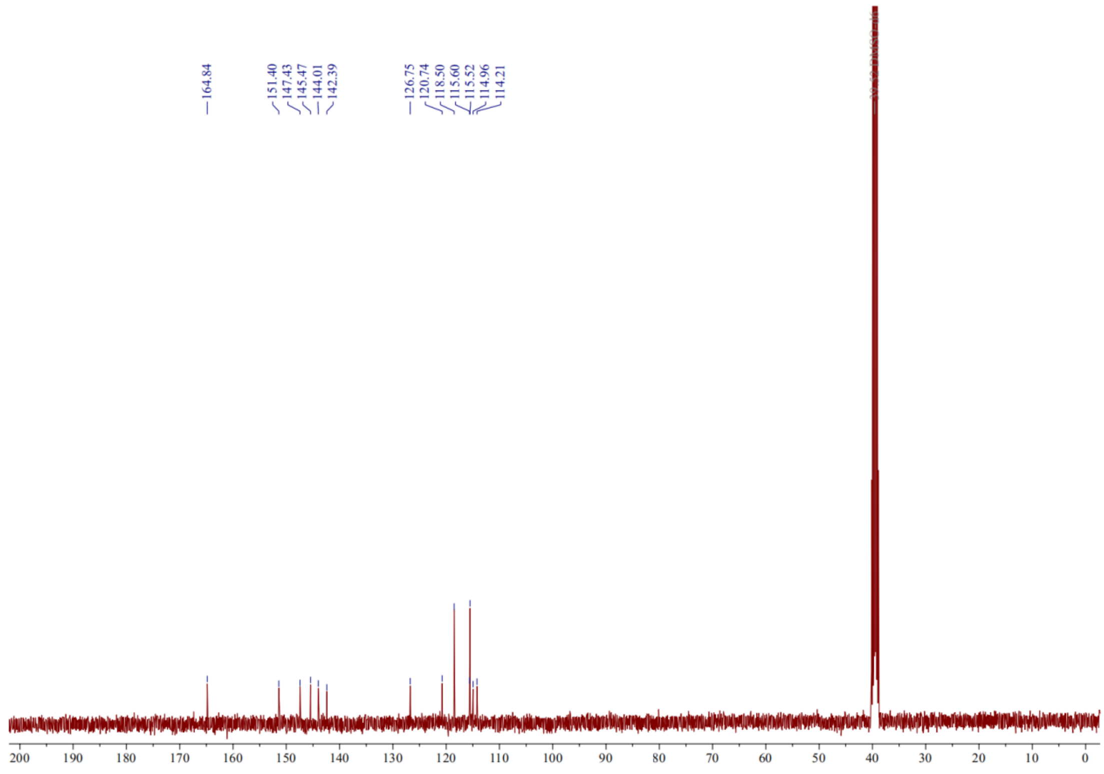

(4-(4-hydroxyphenyl)piperazin-1-yl)(thiazol-2-yl)methanone **(AI10-m17)**

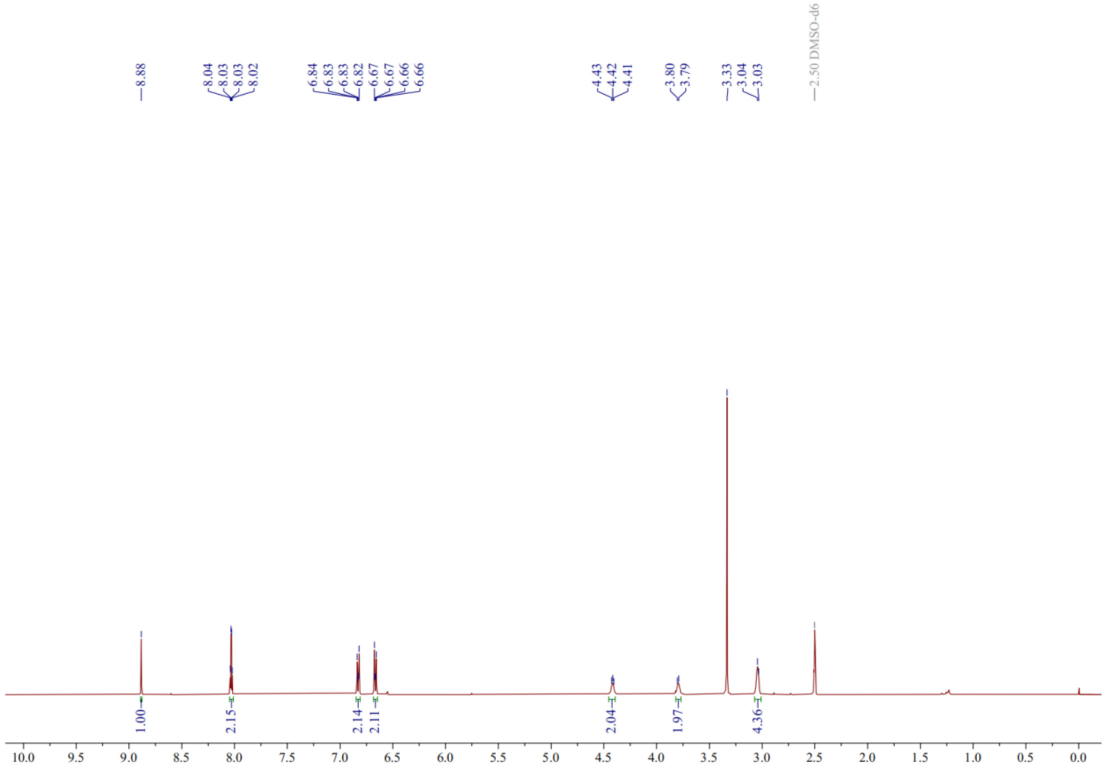

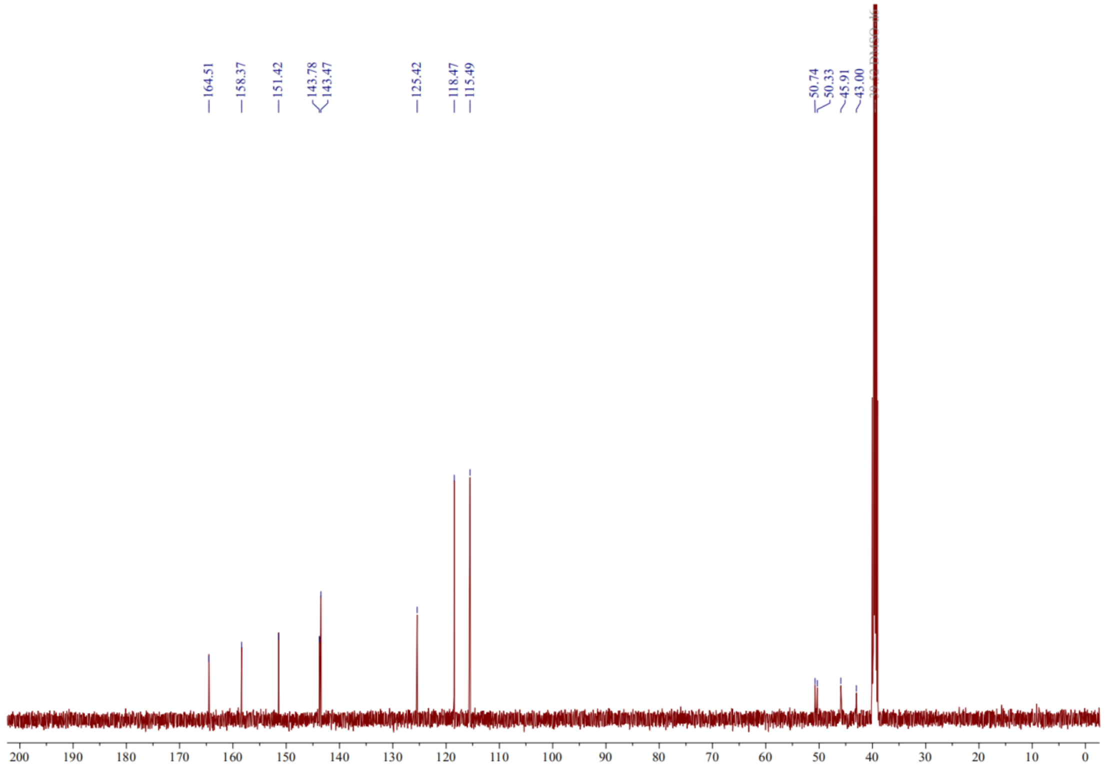

(4-(4-hydroxyphenyl)piperazin-1-yl)(1-methyl-1H-indazol-6-yl)methanone **(AI10-m18)**

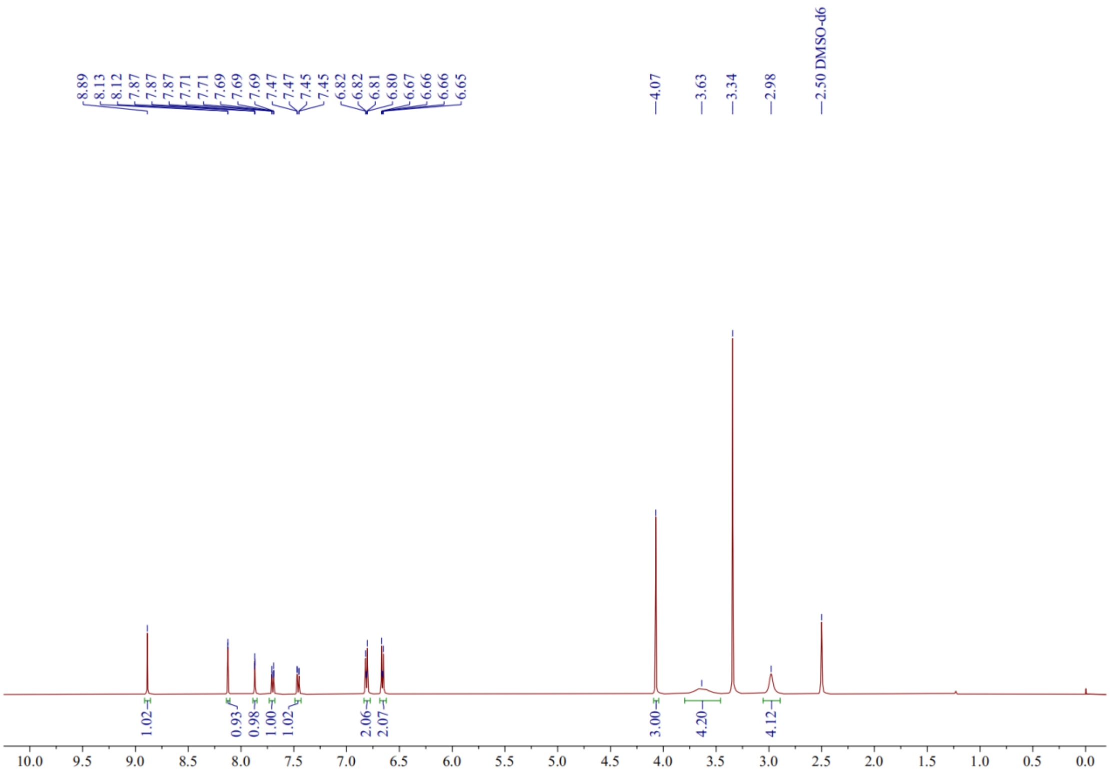

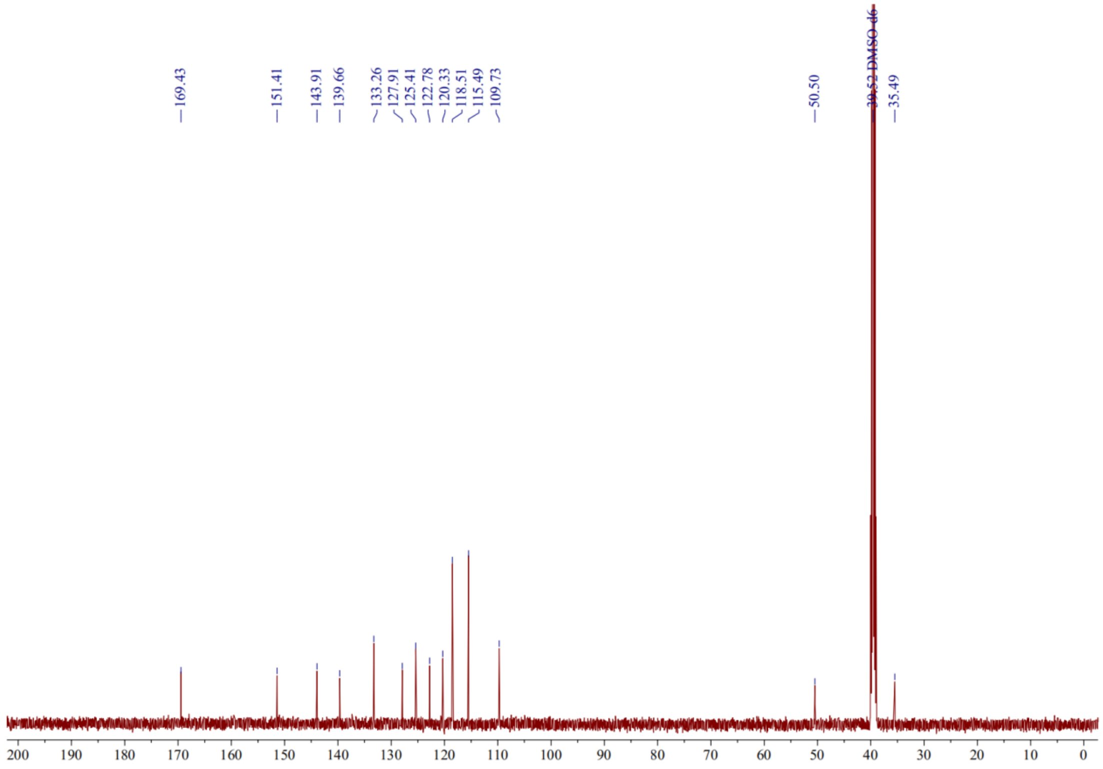

(4-(4-hydroxyphenyl)piperazin-1-yl)(1-methyl-1H-indazol-3-yl)methanone **(AI10-m19)**

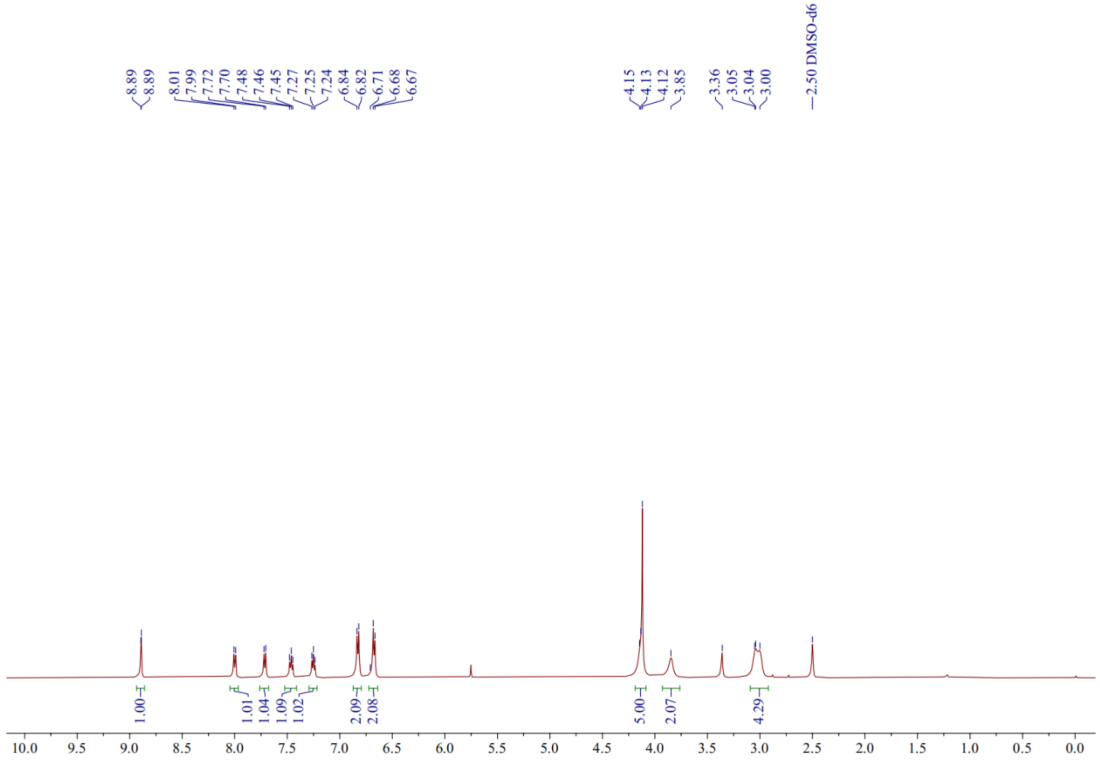

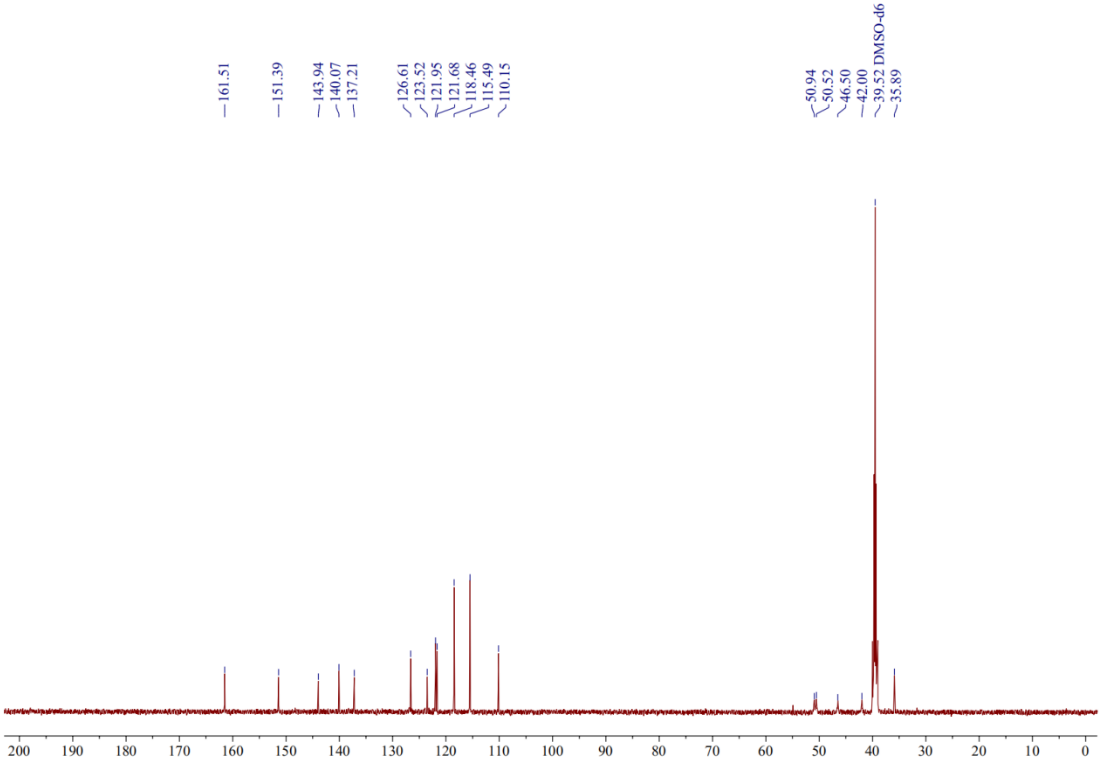

(4-(4-hydroxyphenyl)piperazin-1-yl)(1-methyl-1H-indol-3-yl)methanone **(AI10-m20)**

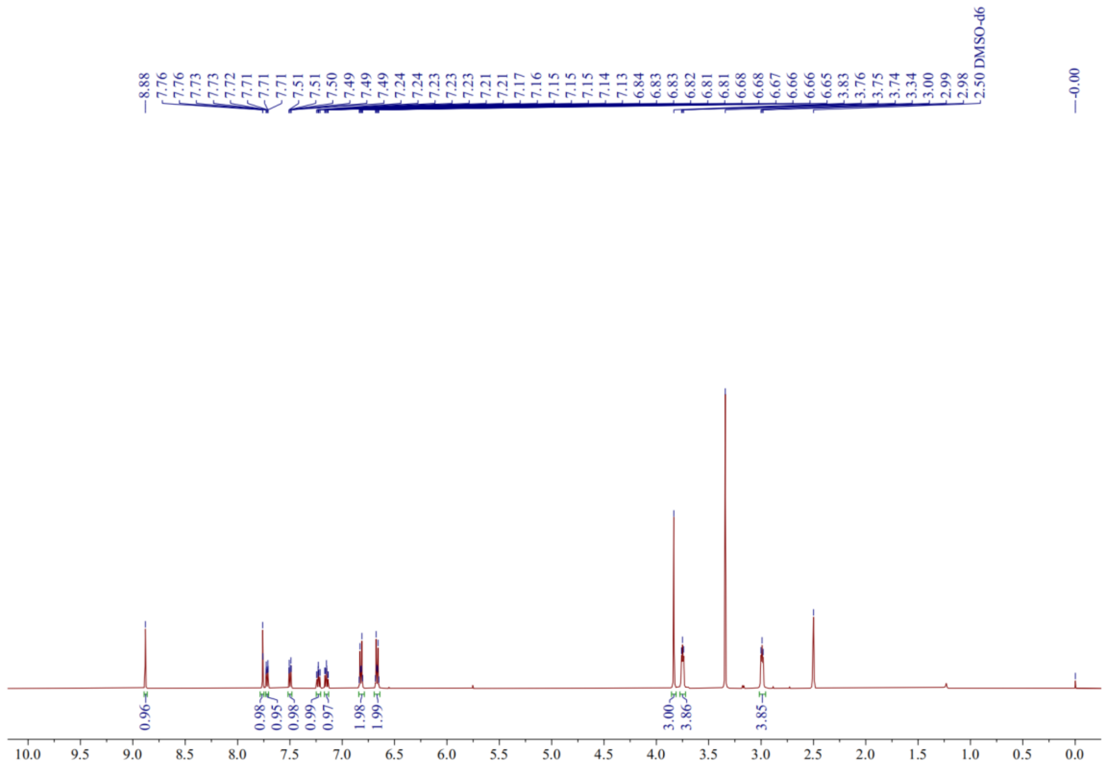

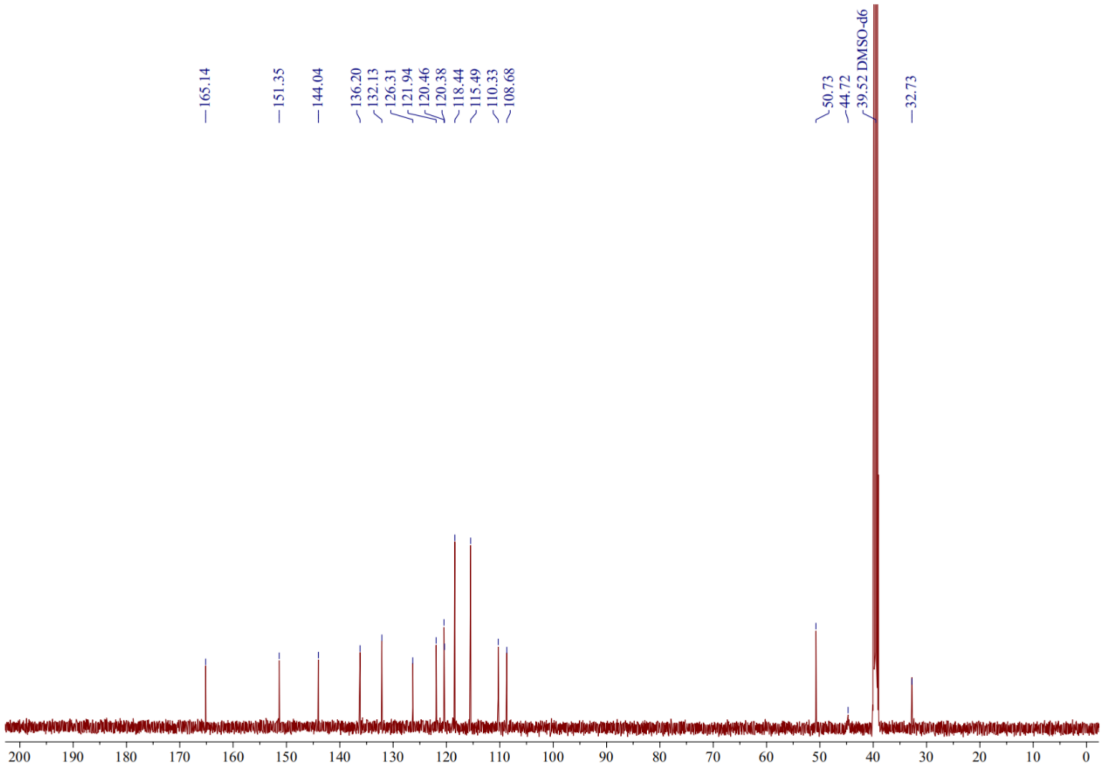

***4-(4-(2-fluorobenzyl)piperazin-1-yl)phenol* (AI10-m21)**

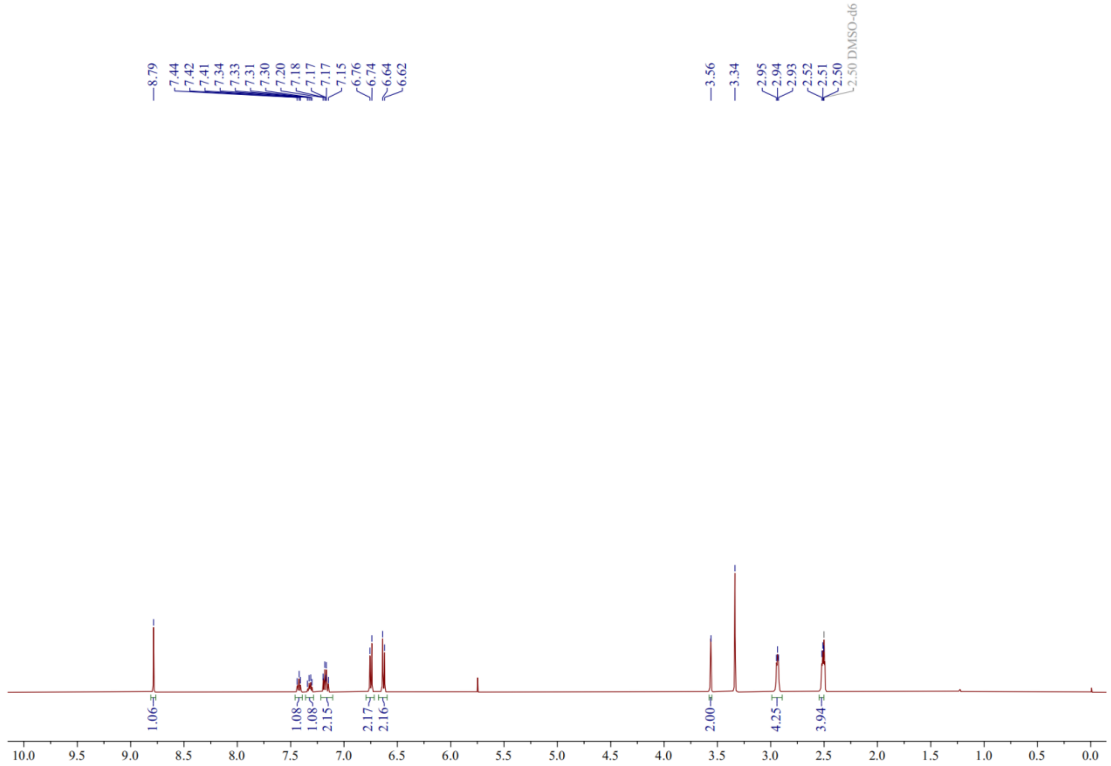

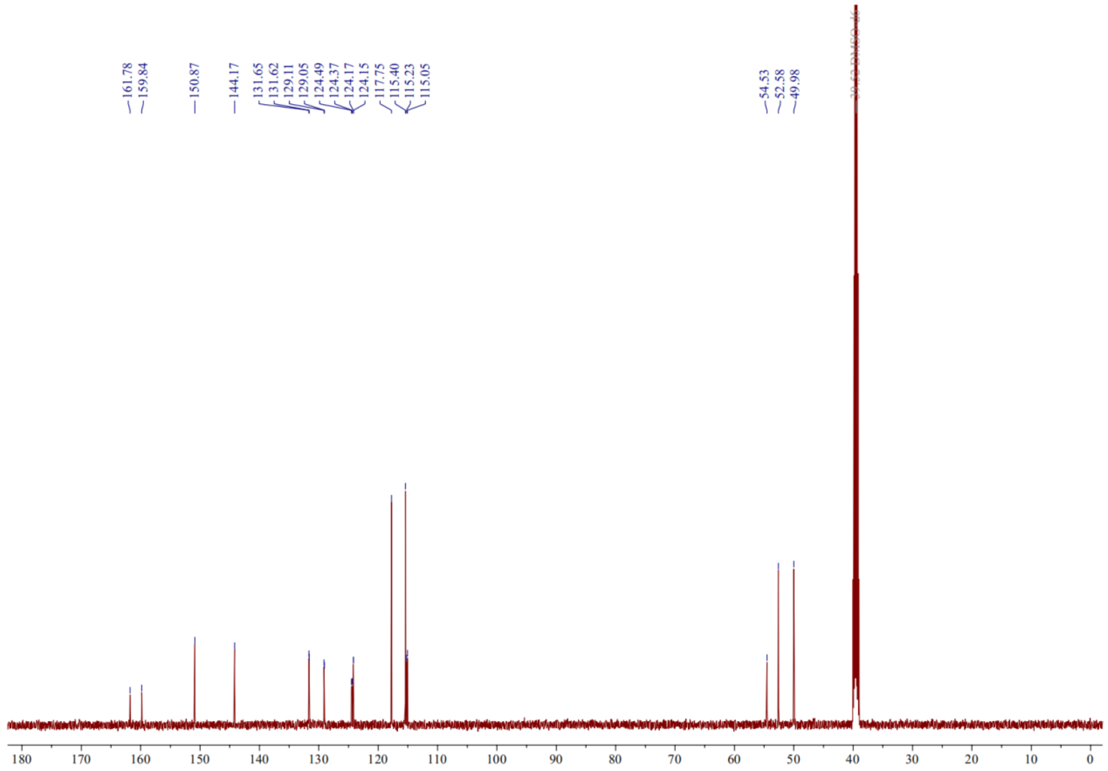

4-(4-(4-fluorobenzyl)piperazin-1-yl)phenol **(AI10-m22)**

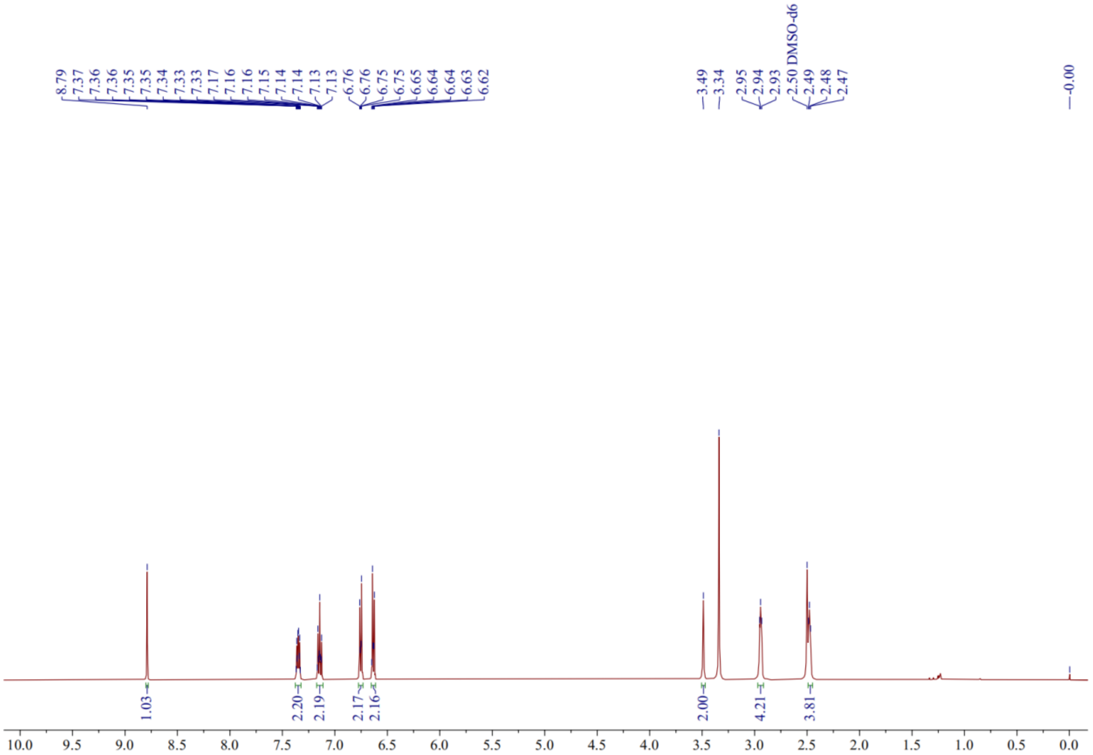

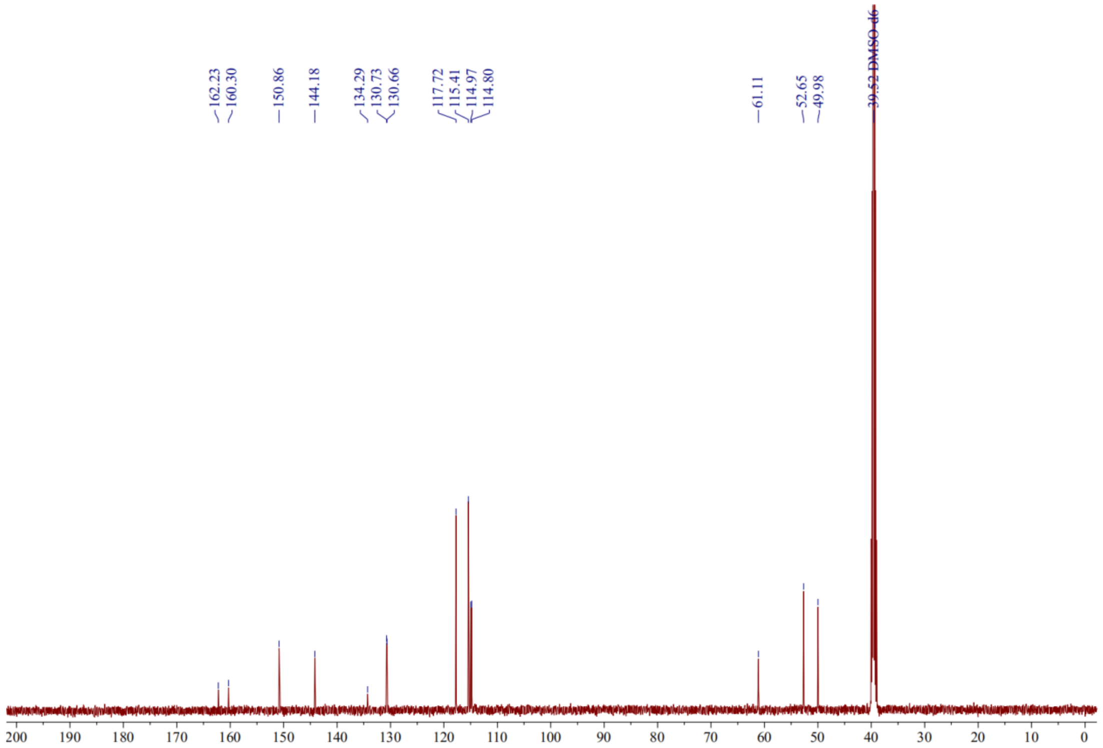

***4-(4-(3-fluorobenzyl)piperazin-1-yl)phenol* (AI10-m23)**

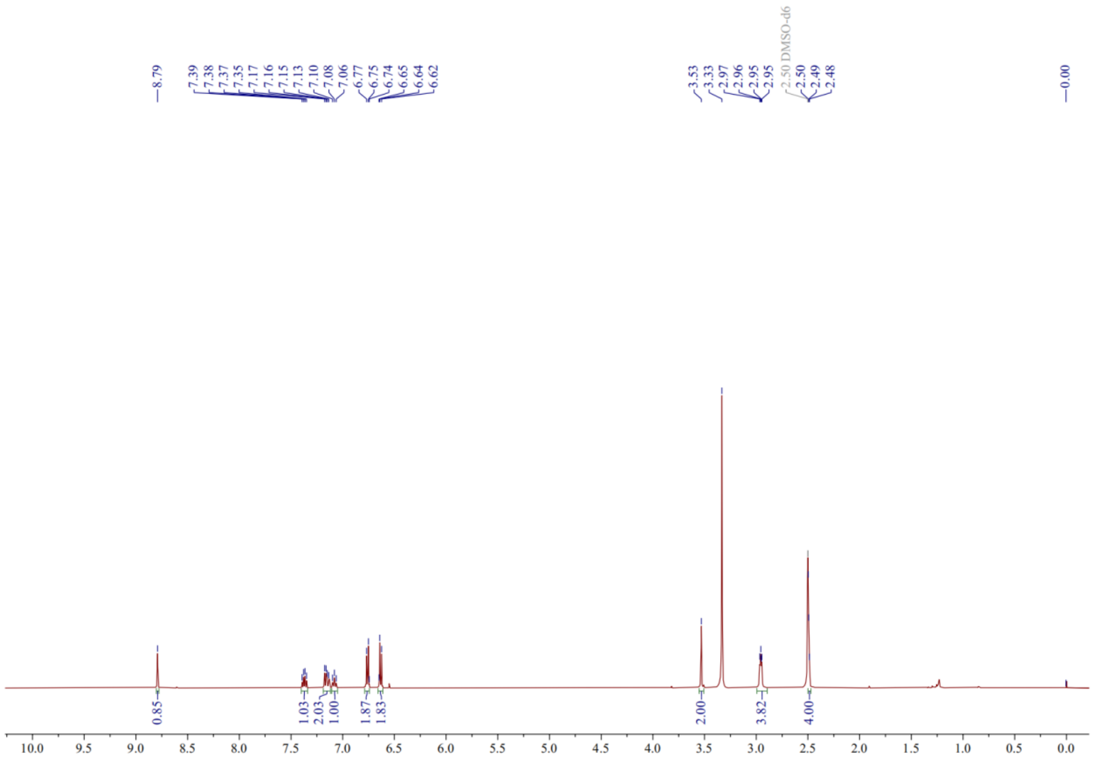

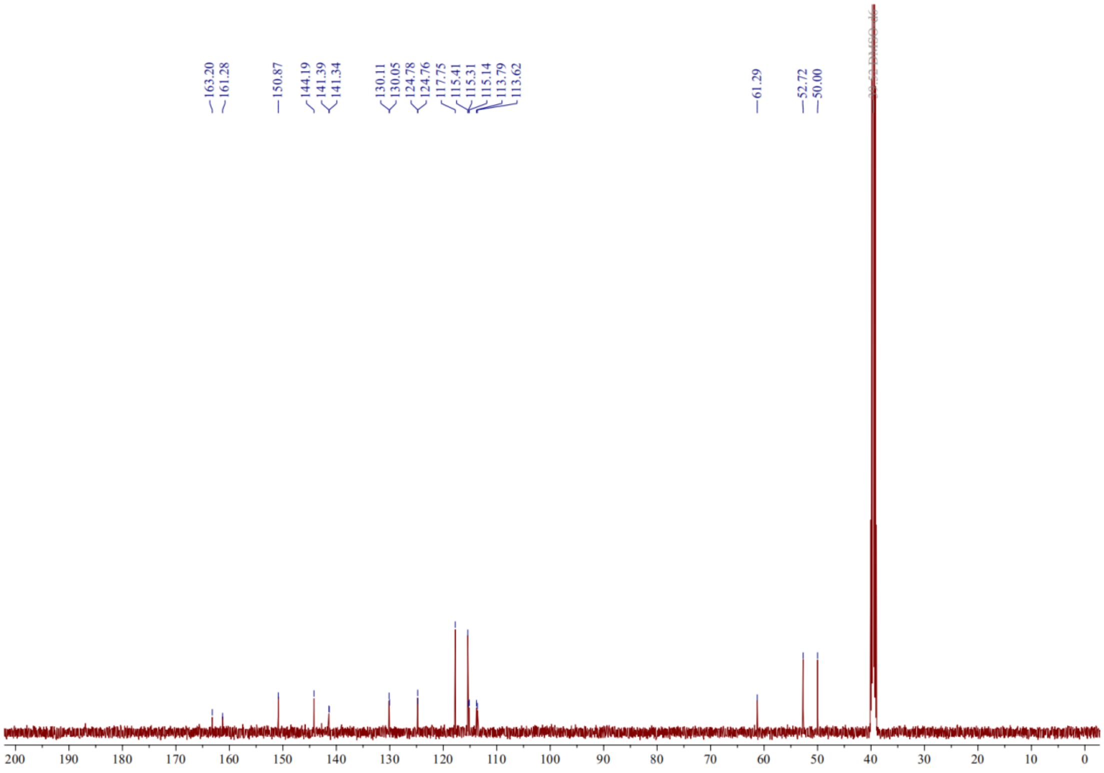

4-(4-(2-chlorobenzyl)piperazin-1-yl)phenol **(AI10-m24)**

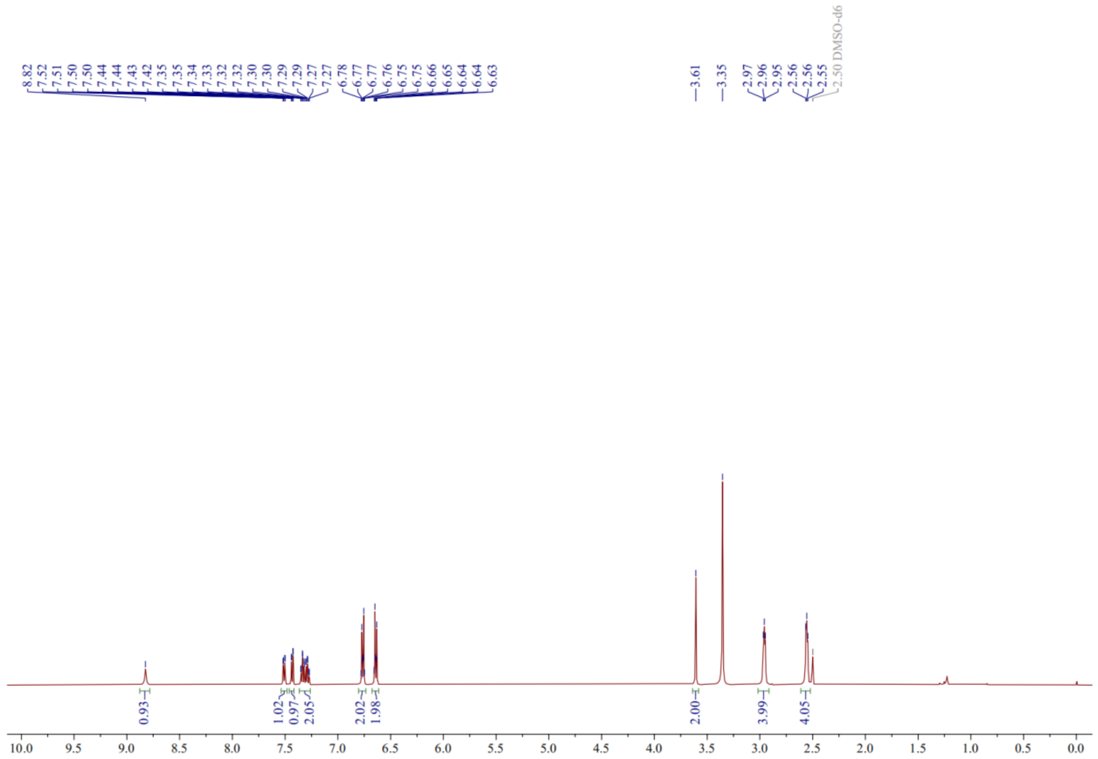

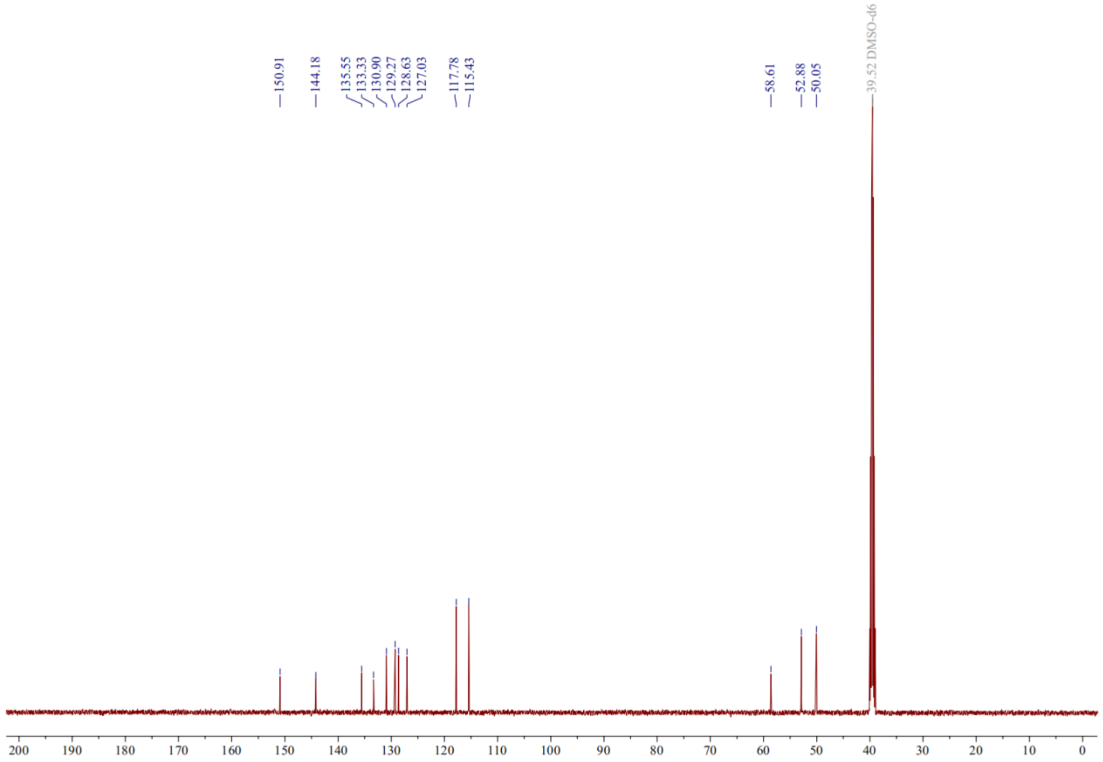

***4-(4-(3-chlorobenzyl)piperazin-1-yl)phenol* (AI10-m25)**

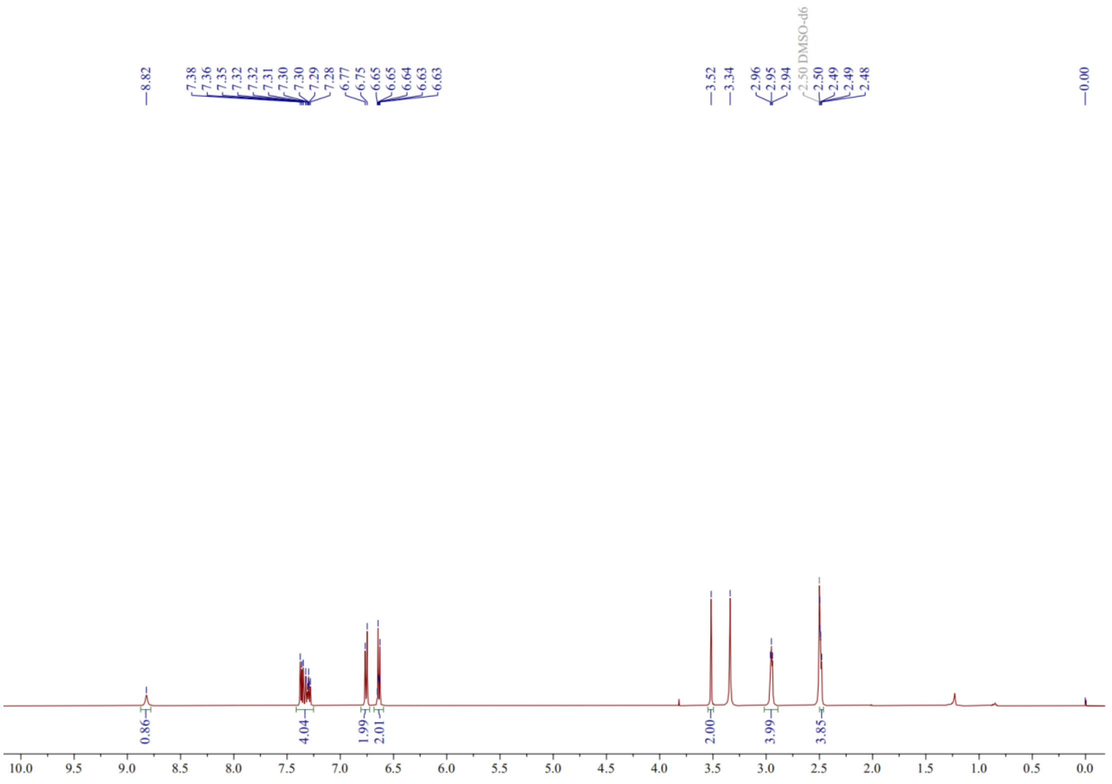

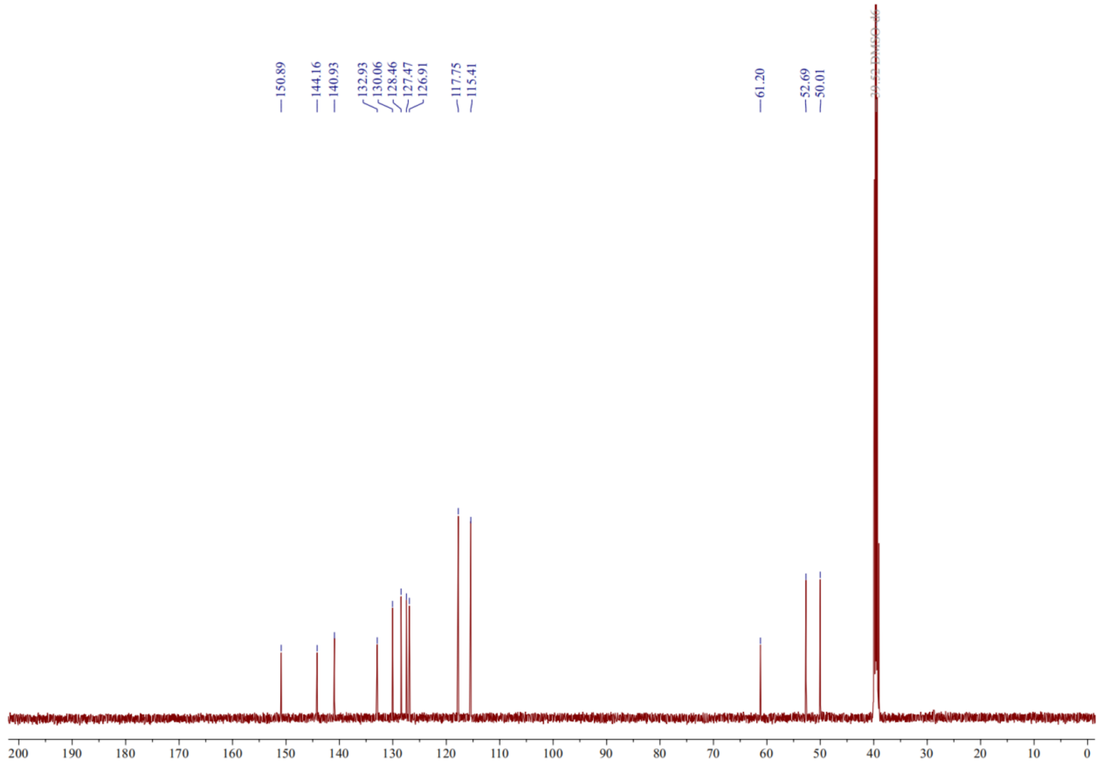

4-(4-(4-chlorobenzyl)piperazin-1-yl)phenol **(AI10-m26)**

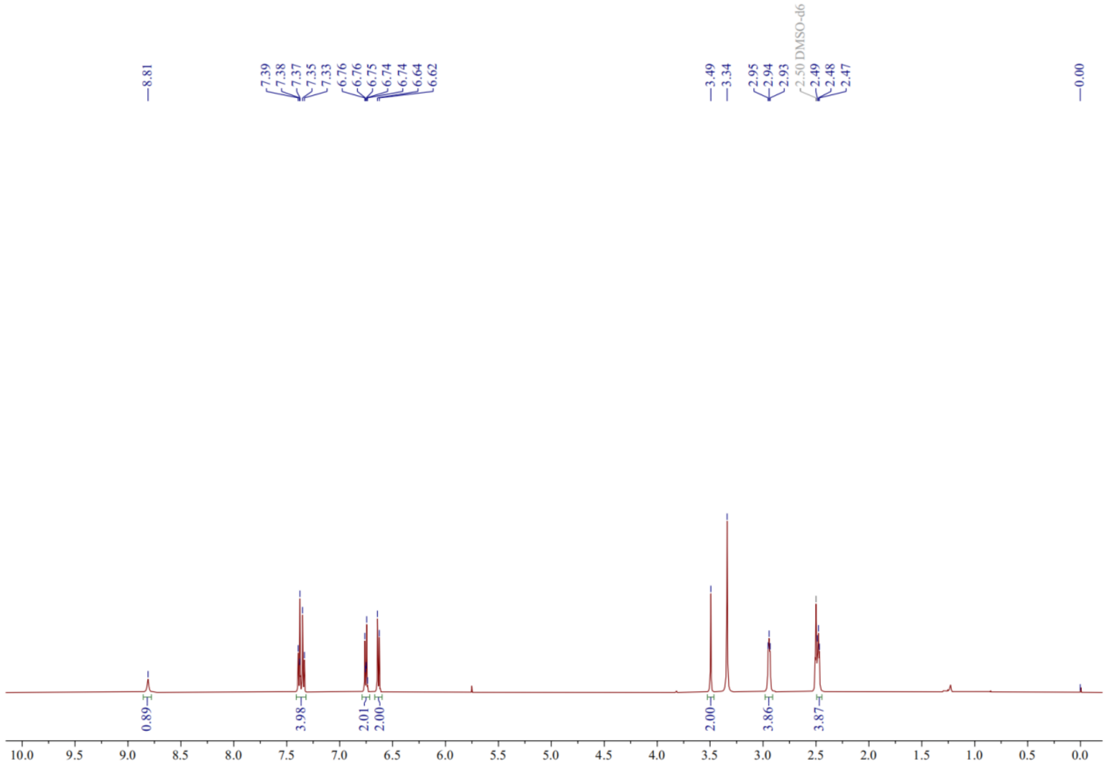

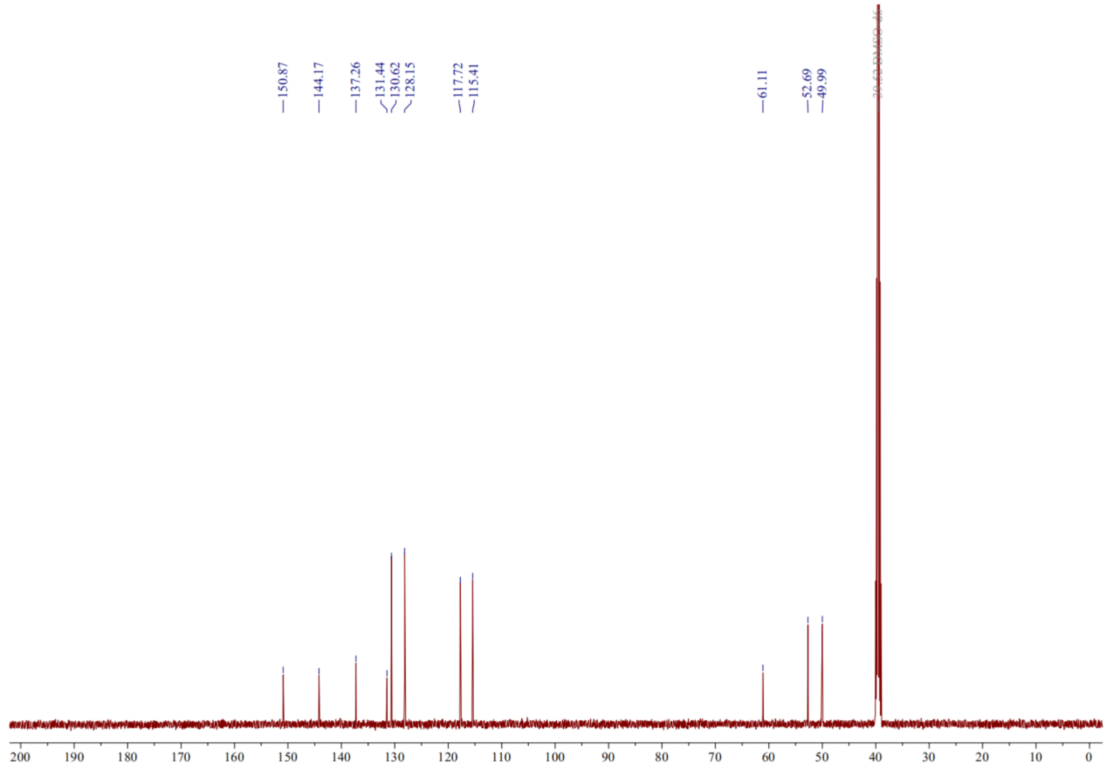

4-(4-(2-methylbenzyl)piperazin-1-yl)phenol **(AI10-m27)**

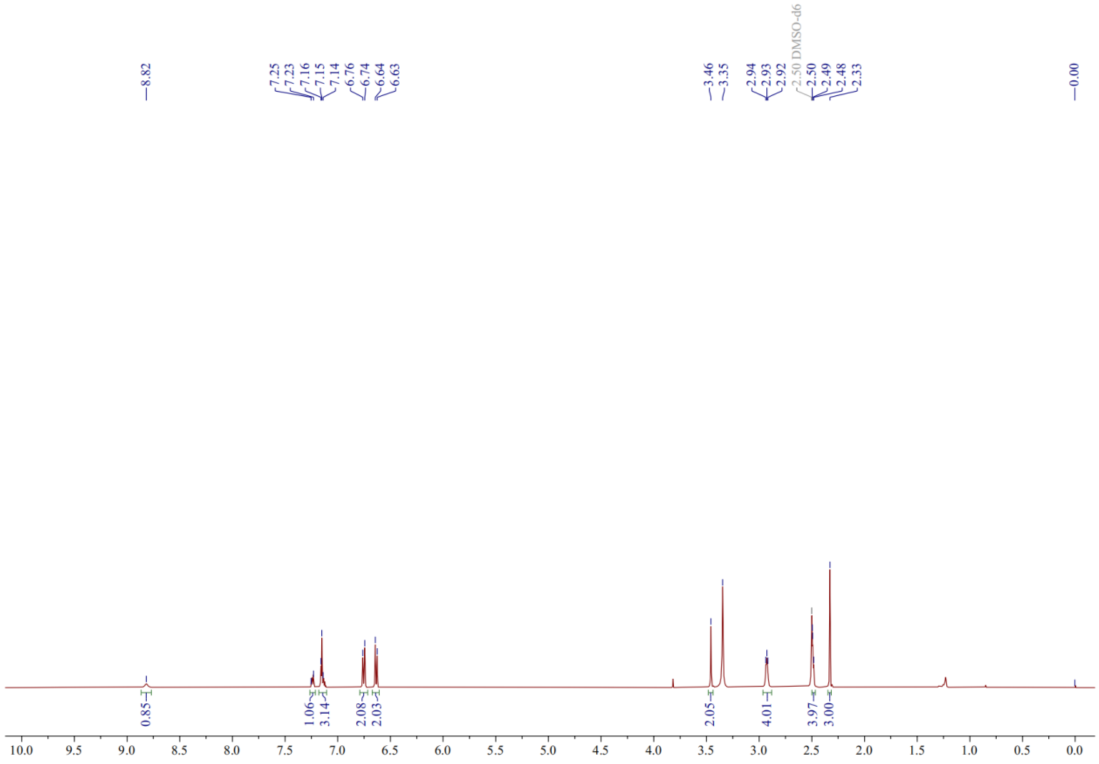

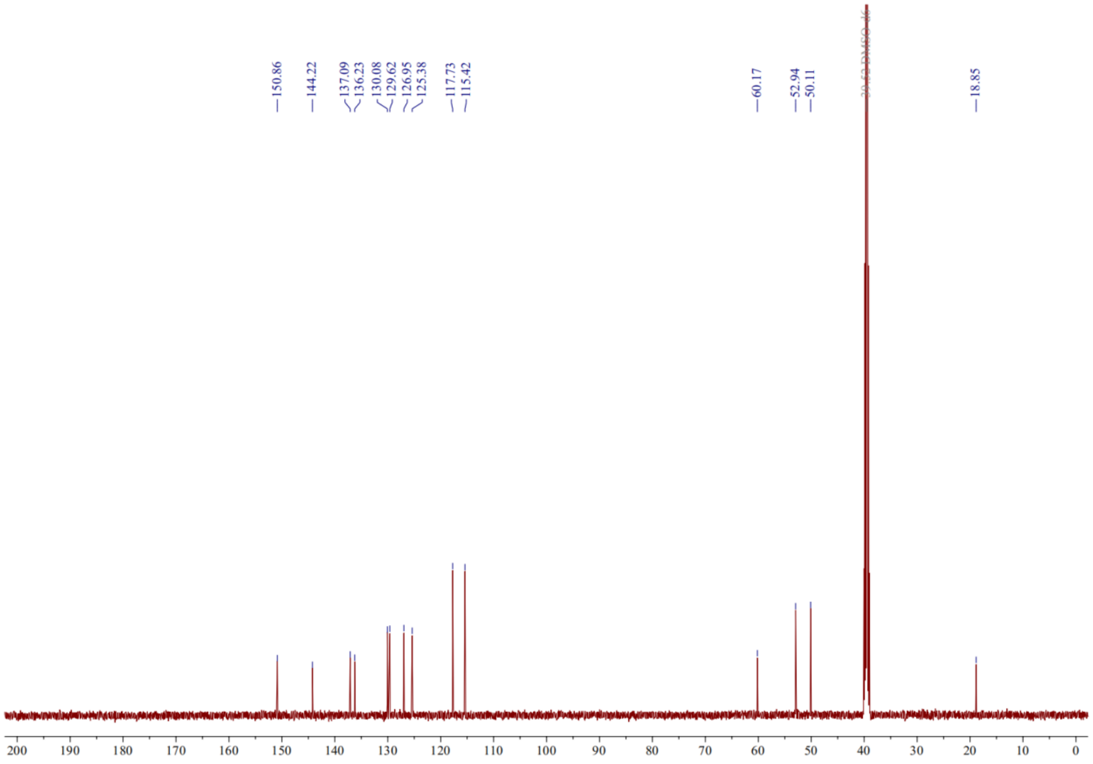

4-(4-(3-methylbenzyl)piperazin-1-yl)phenol **(AI10-m28)**

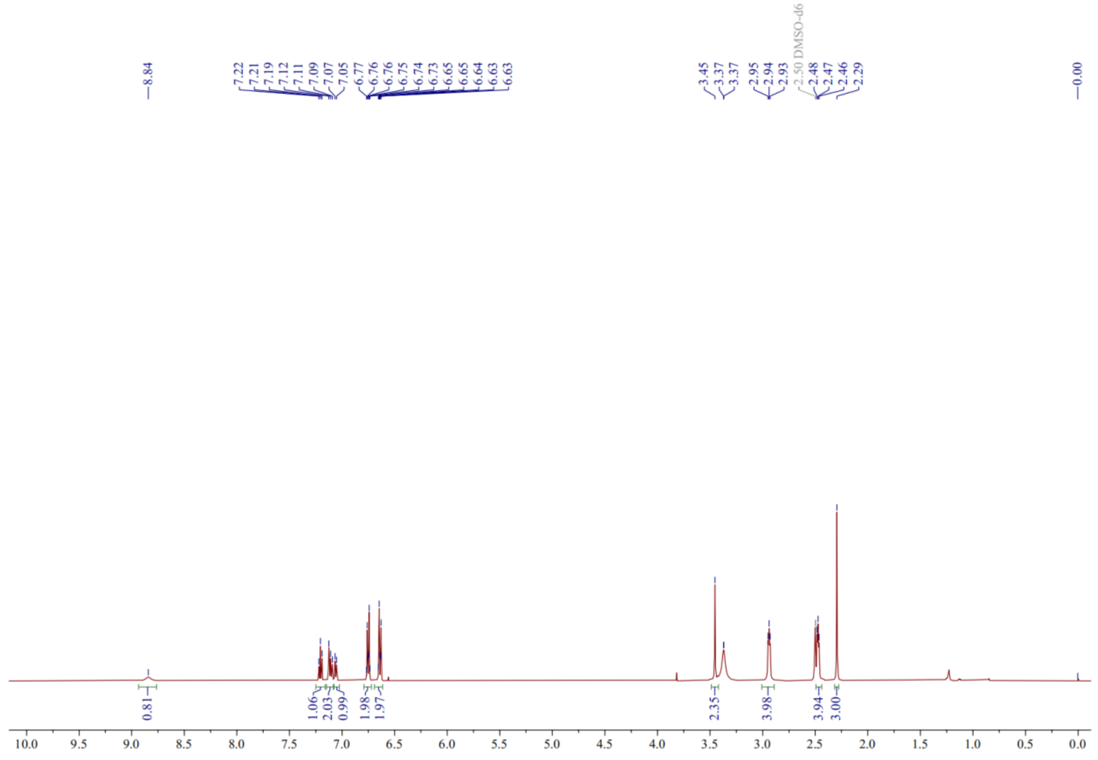

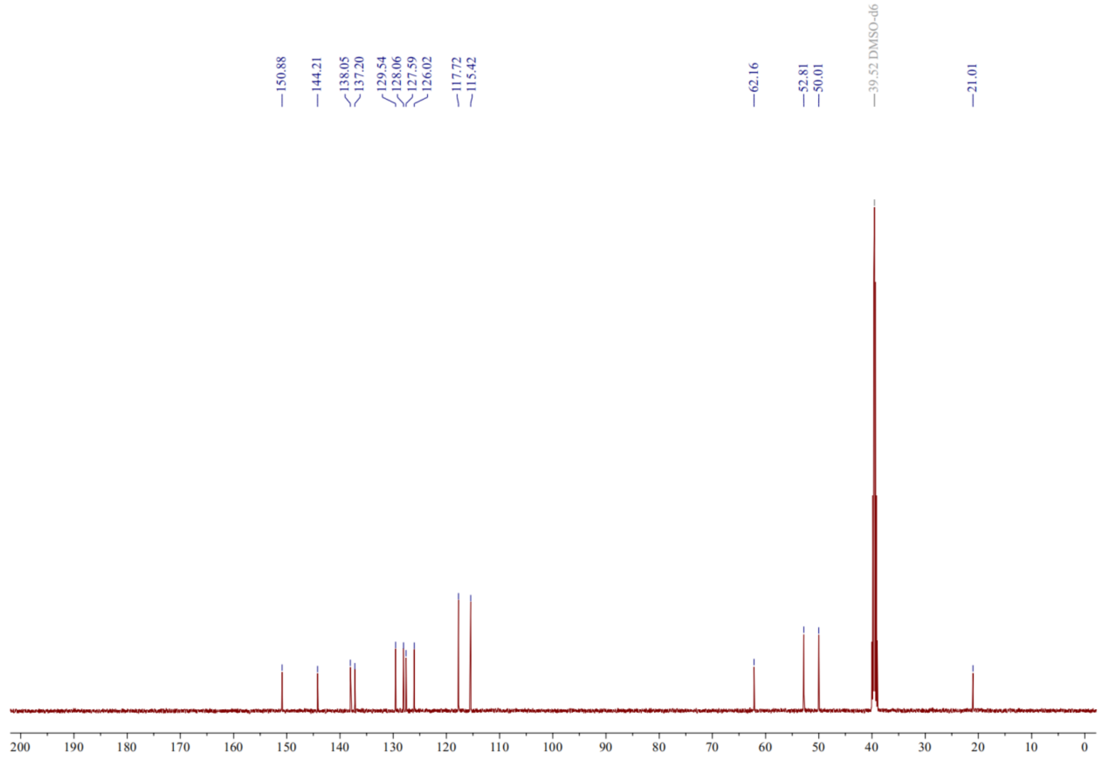

4-(4-(4-methylbenzyl)piperazin-1-yl)phenol **(AI10-m29)**

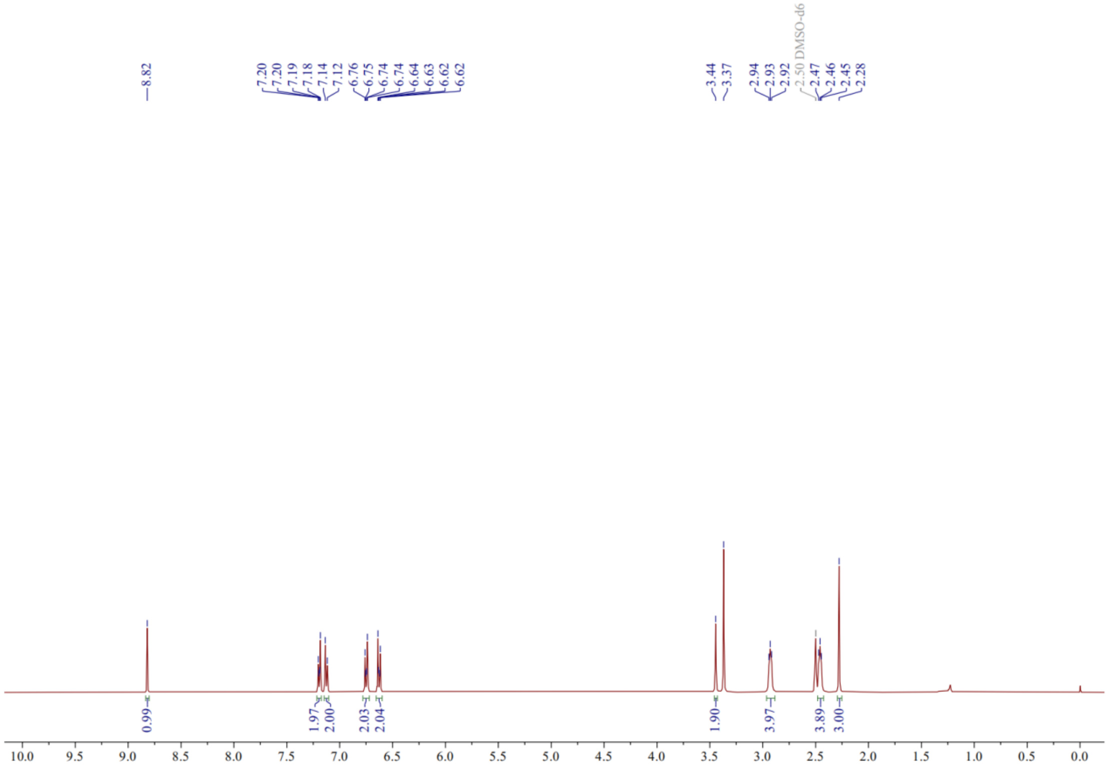

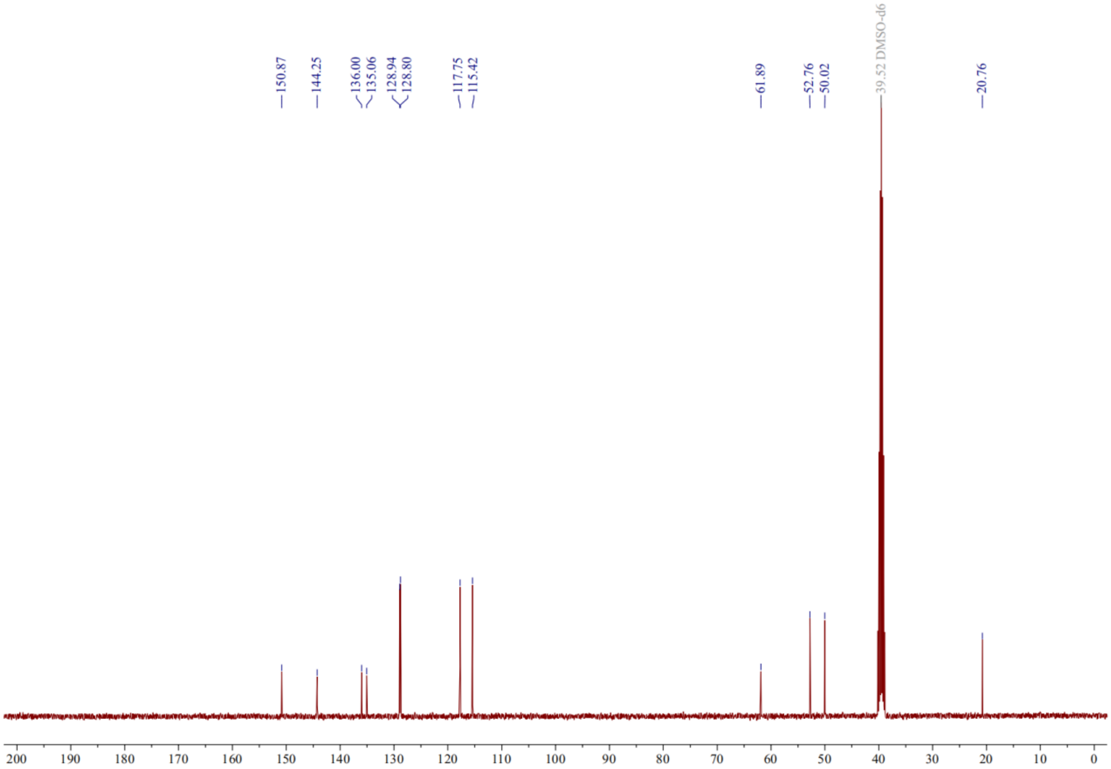

4-(4-(2-methoxybenzyl)piperazin-1-yl)phenol **(AI10-m30)**

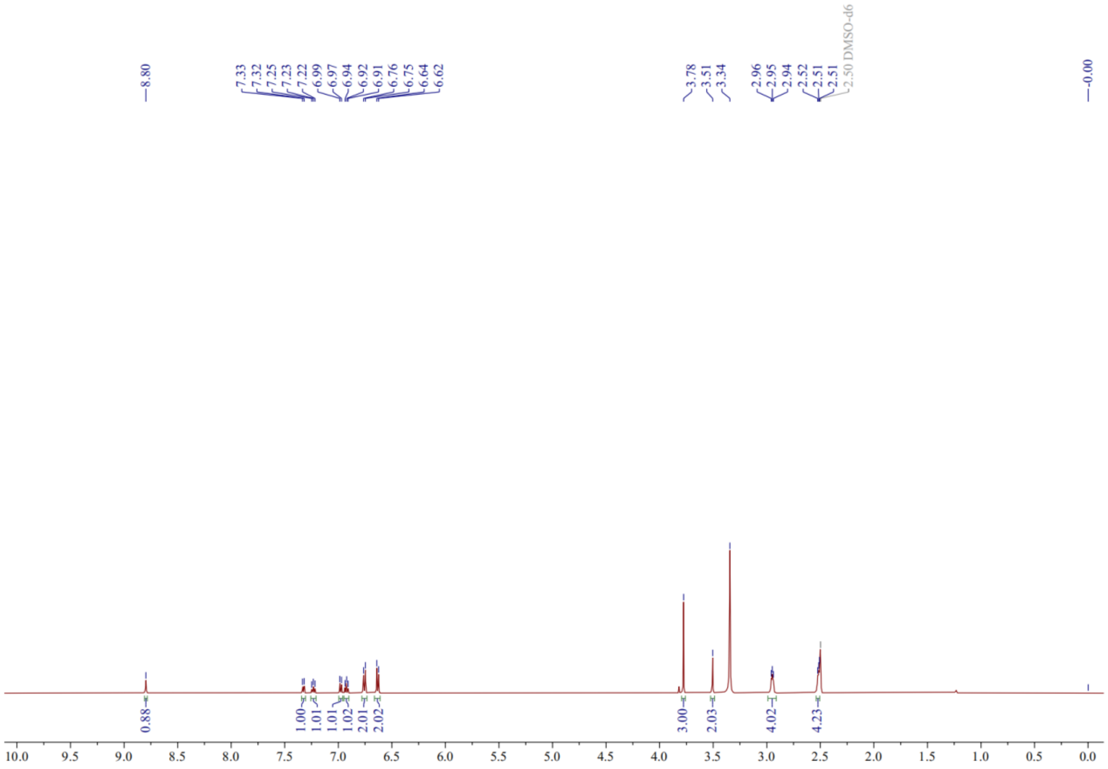

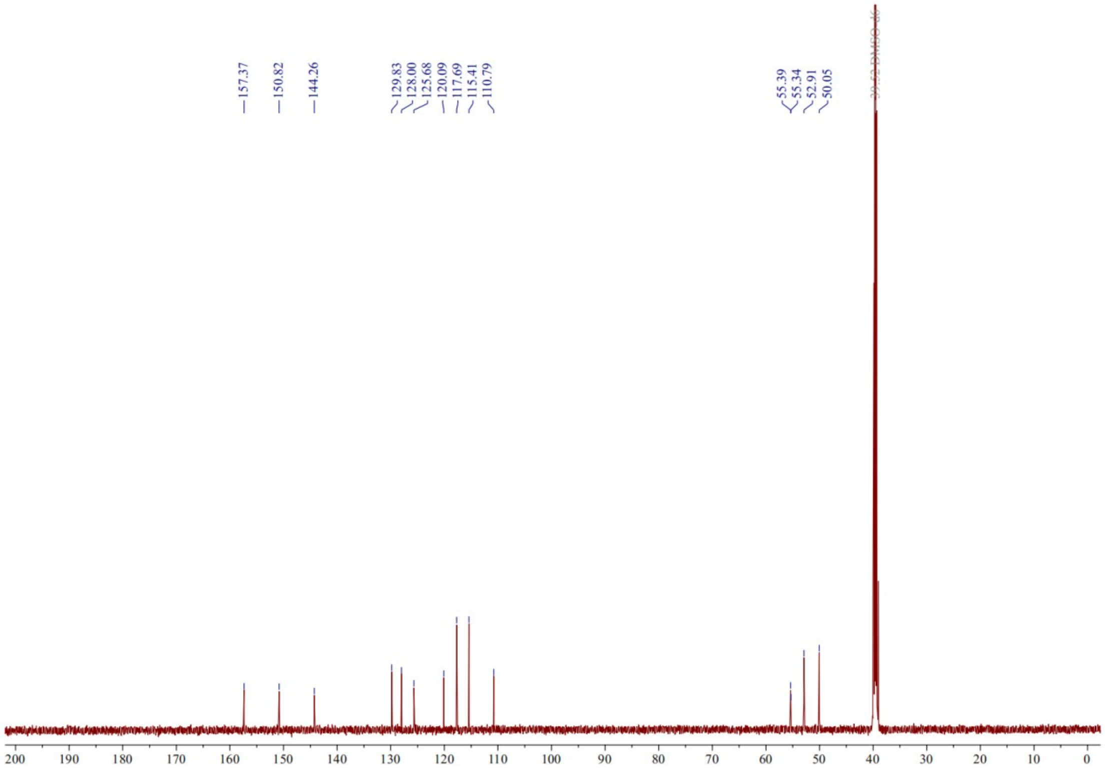

4-(4-(3-methoxybenzyl)piperazin-1-yl)phenol **(AI10-m31)**

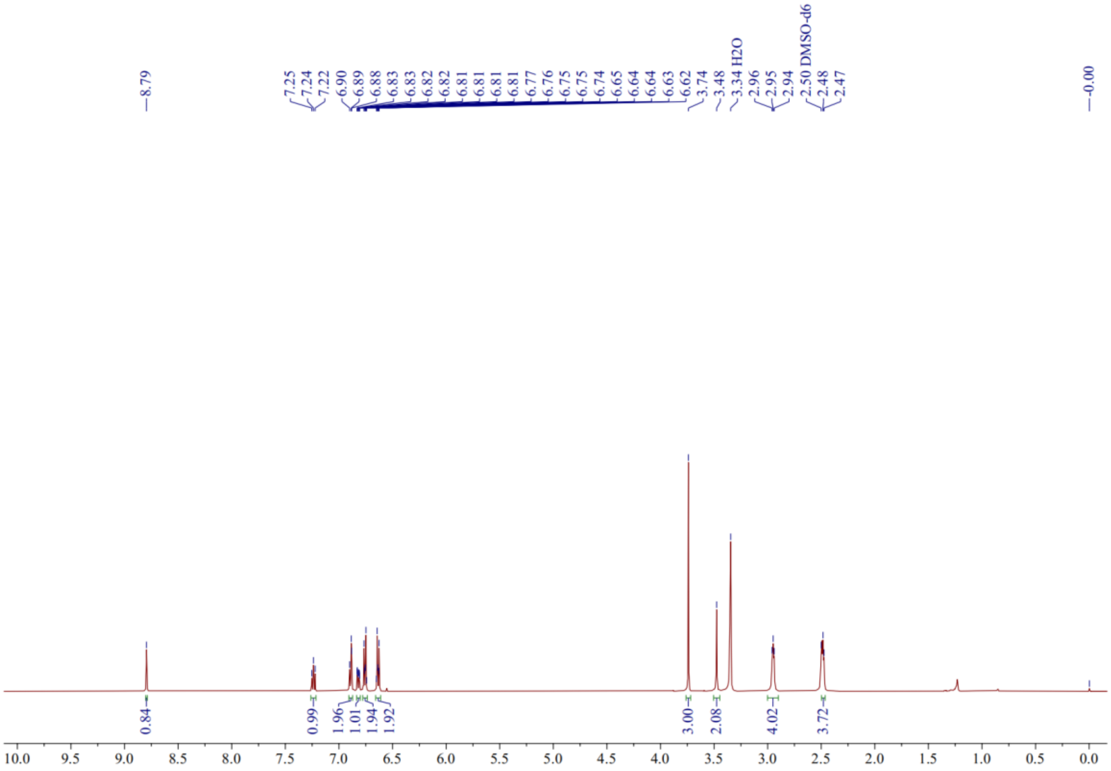

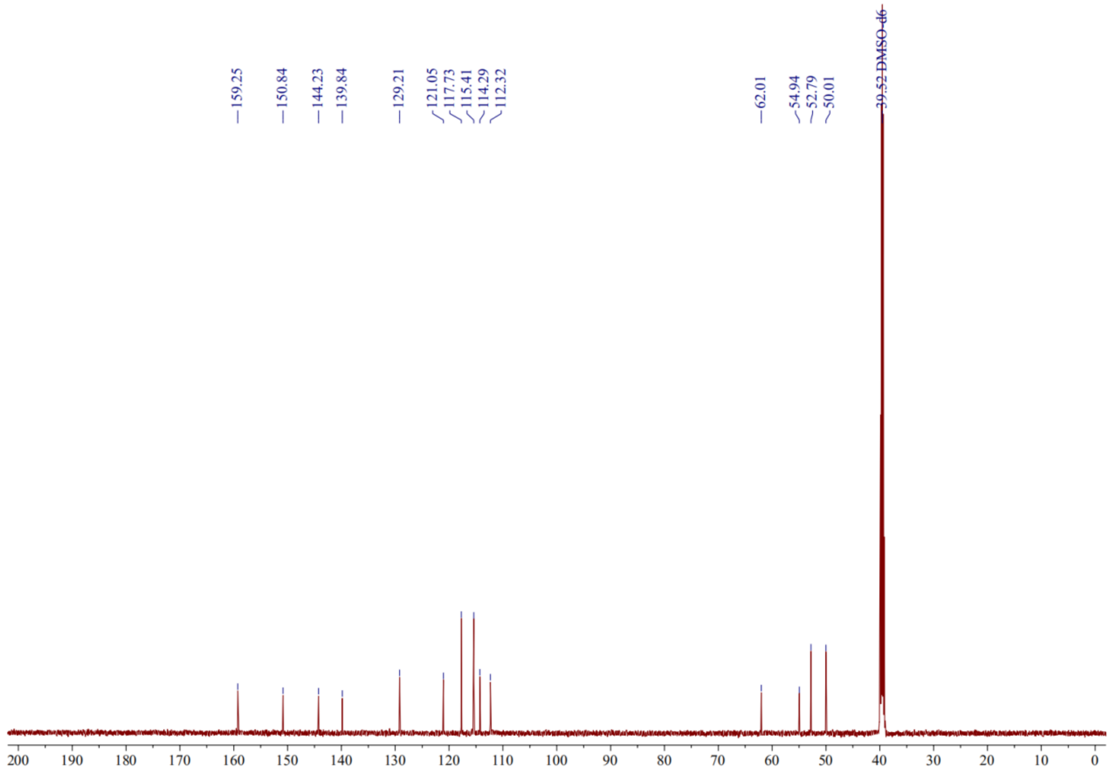

4-(4-(2-ethoxybenzyl)piperazin-1-yl)phenol **(AI10-m32)**

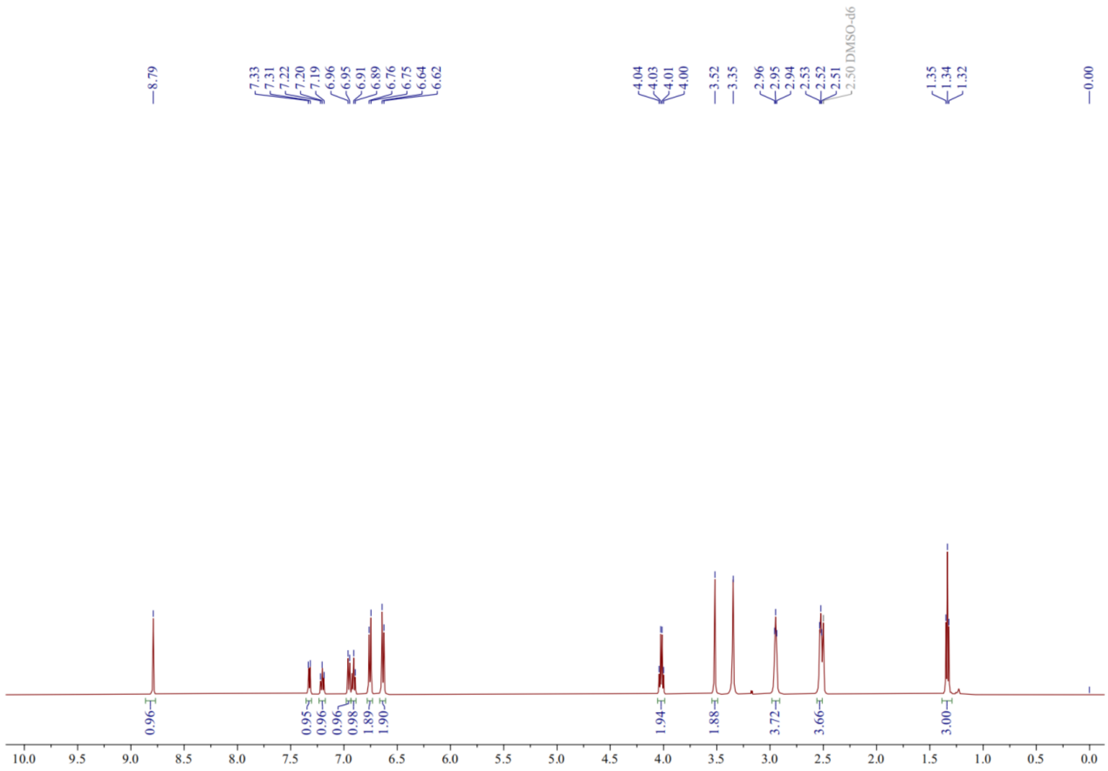

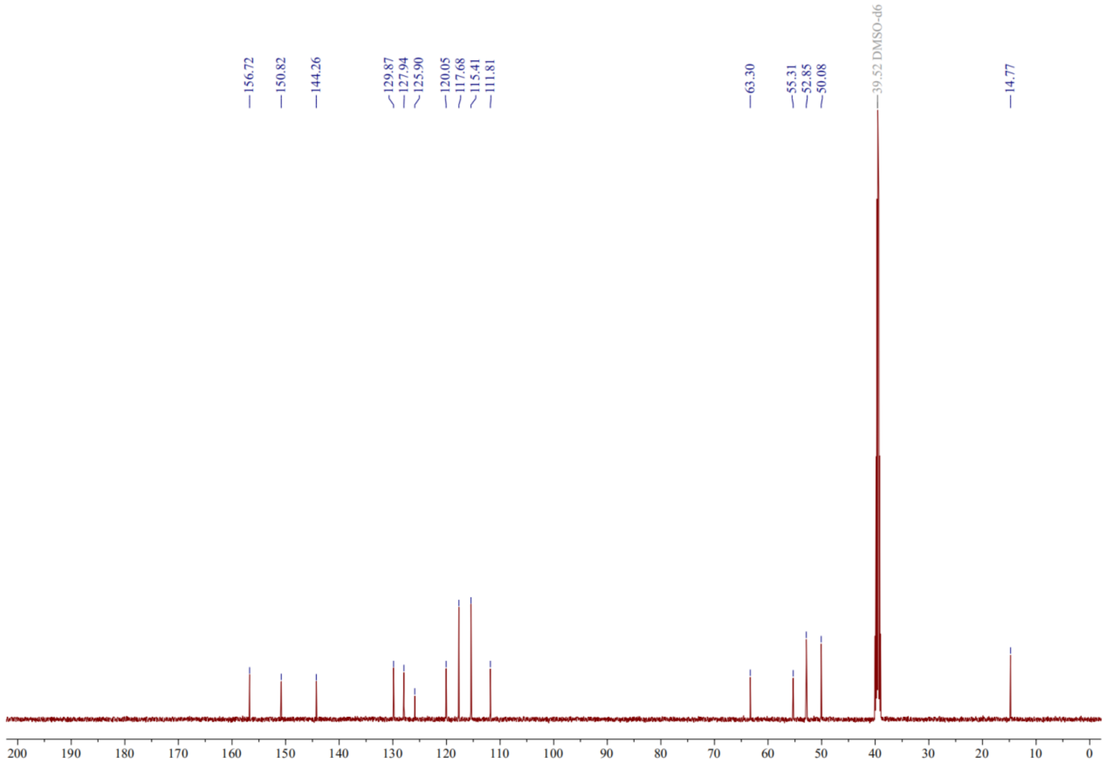

4-(4-(3-ethoxybenzyl)piperazin-1-yl)phenol **(AI10-m33)**

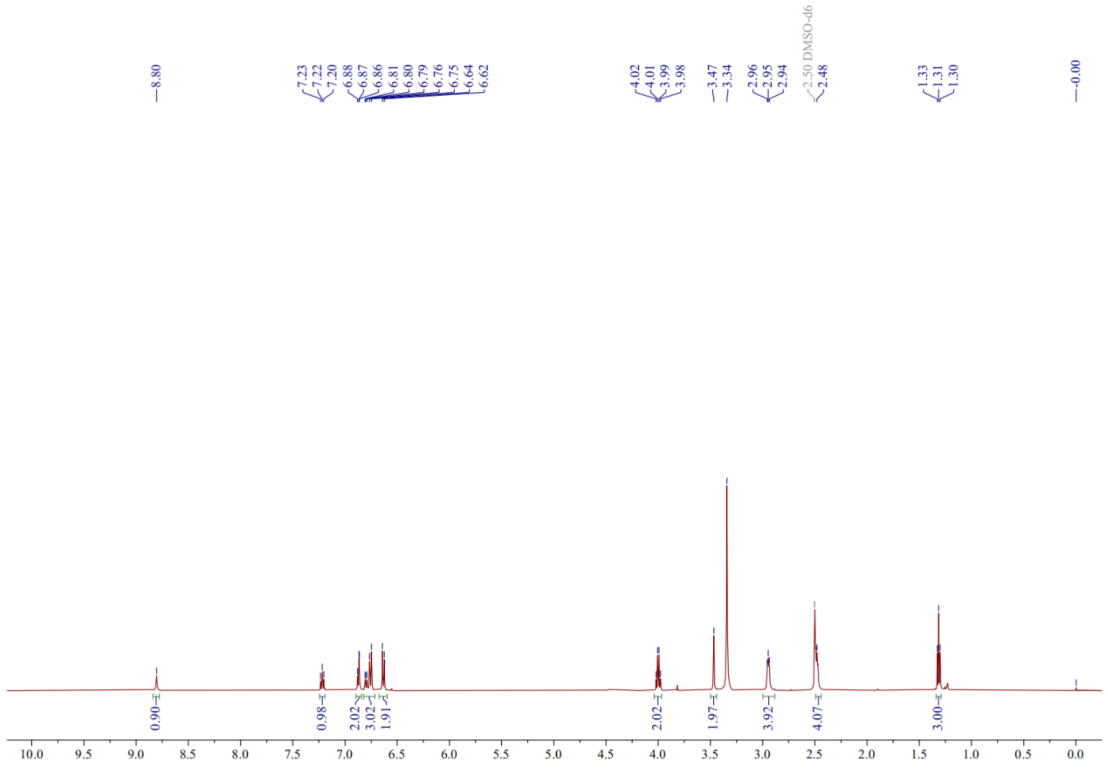

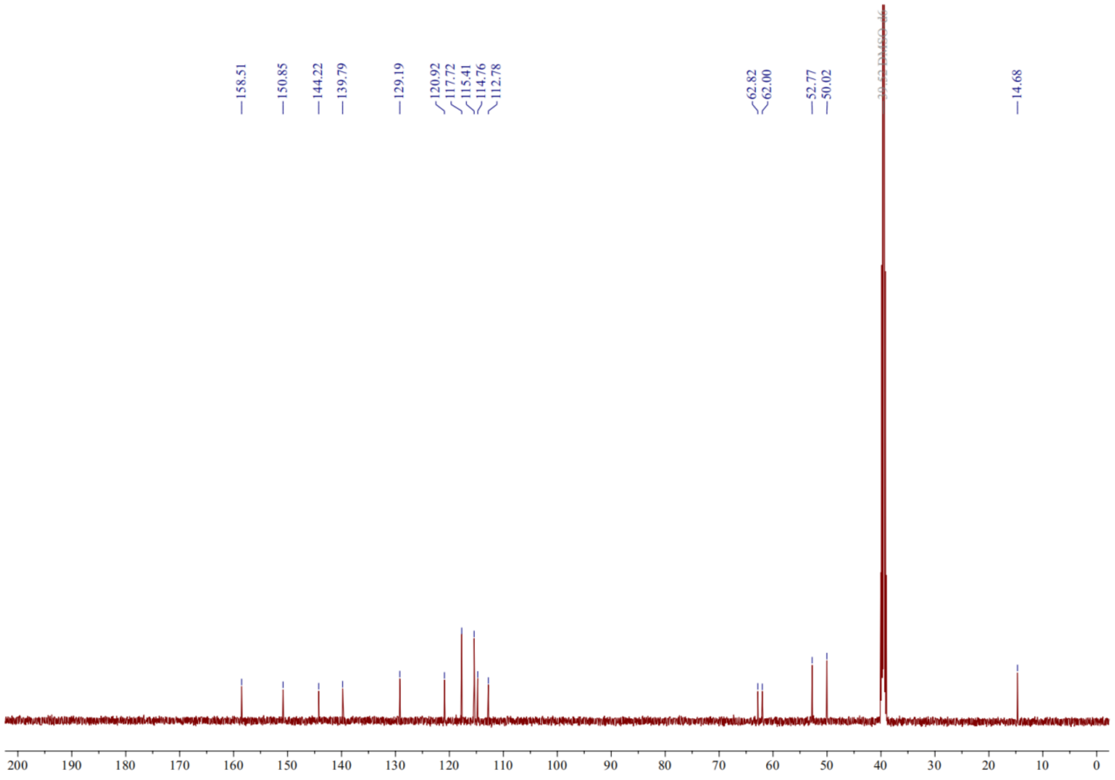

***4-(4-(4-ethoxybenzyl)piperazin-1-yl)phenol* (AI10-m34)**

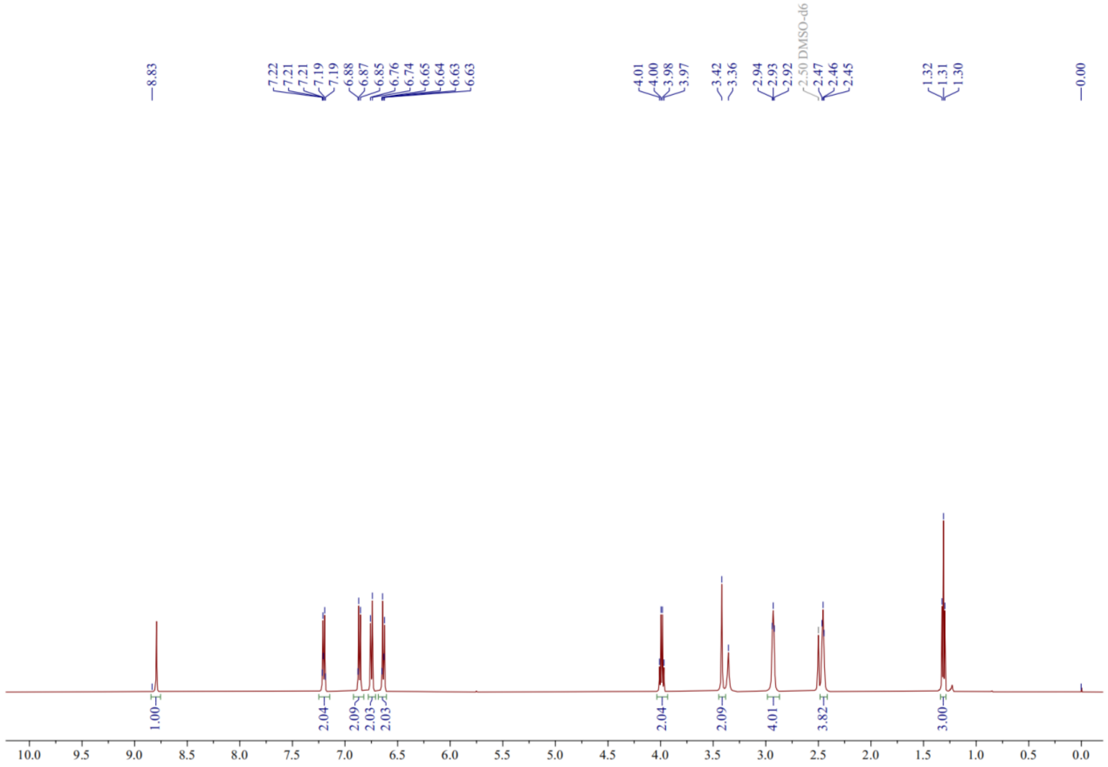

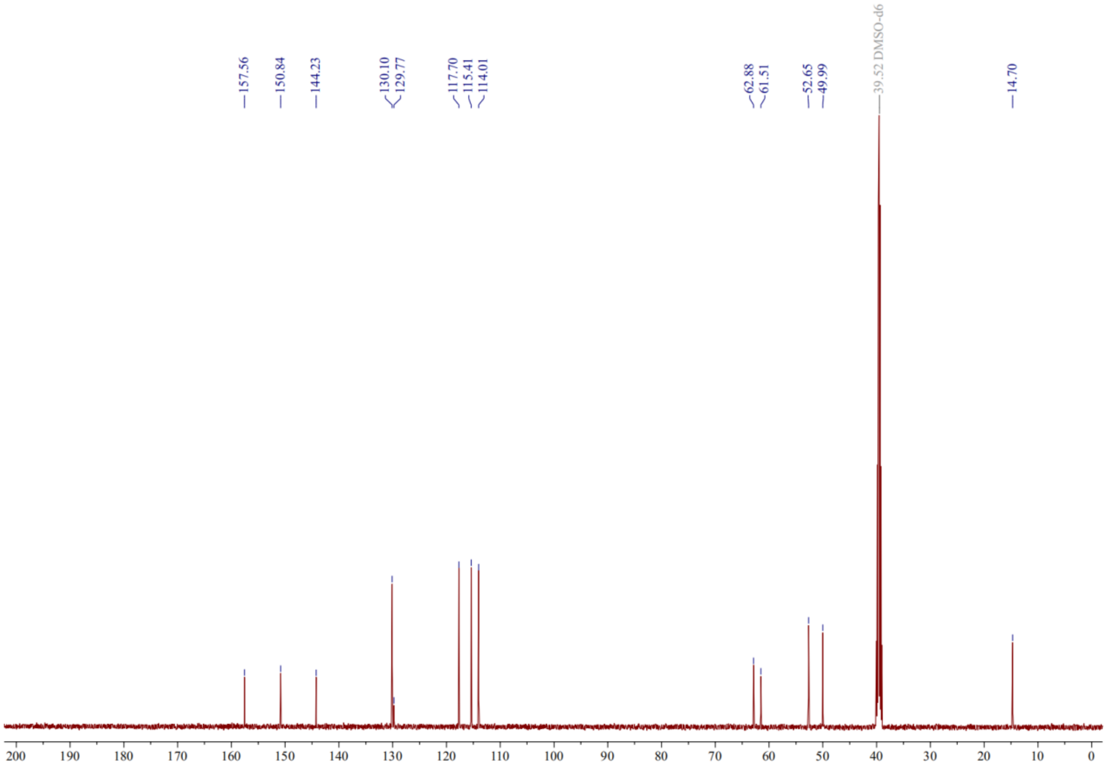

3-((4-(4-hydroxyphenyl)piperazin-1-yl)methyl)phenol **(AI10-m35)**

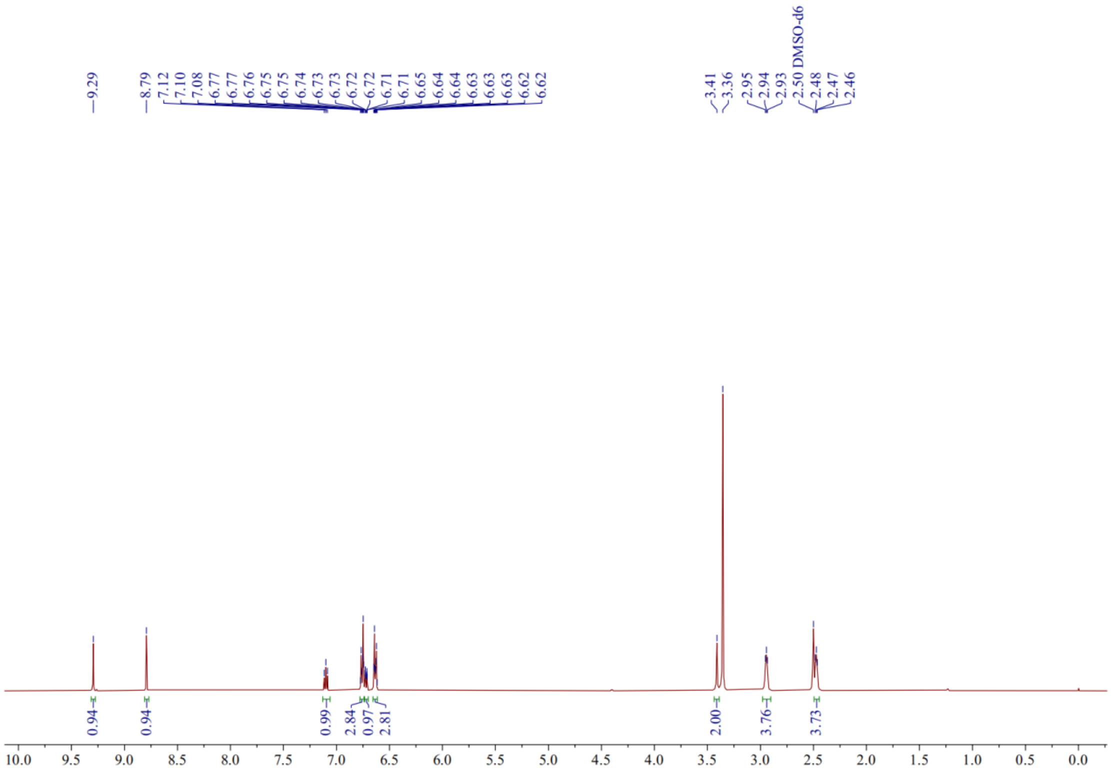

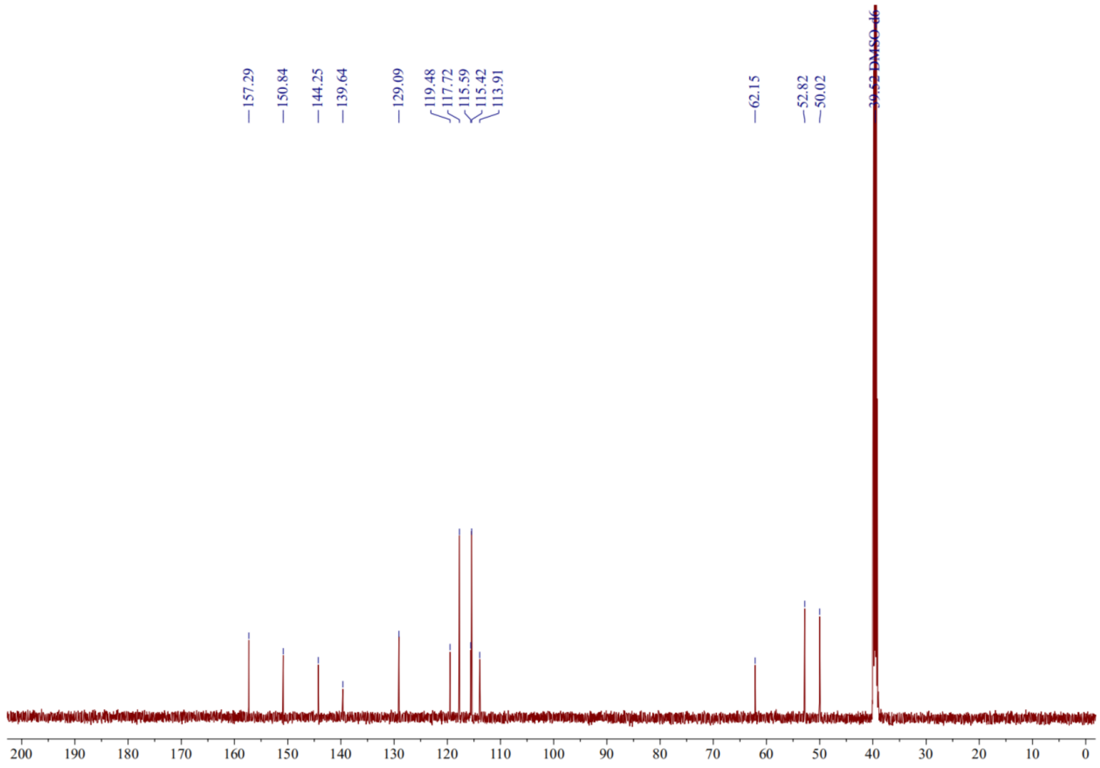

4-(4-(4-hydroxybenzyl)piperazin-1-yl)phenol **(AI10-m36)**

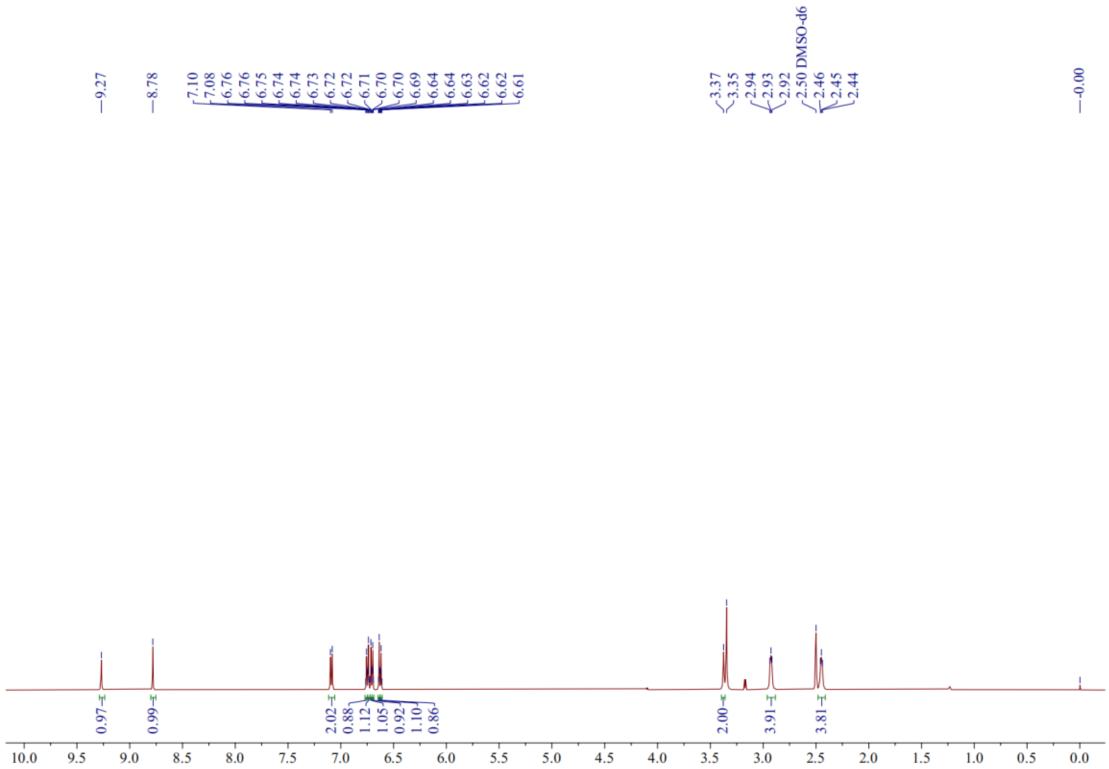

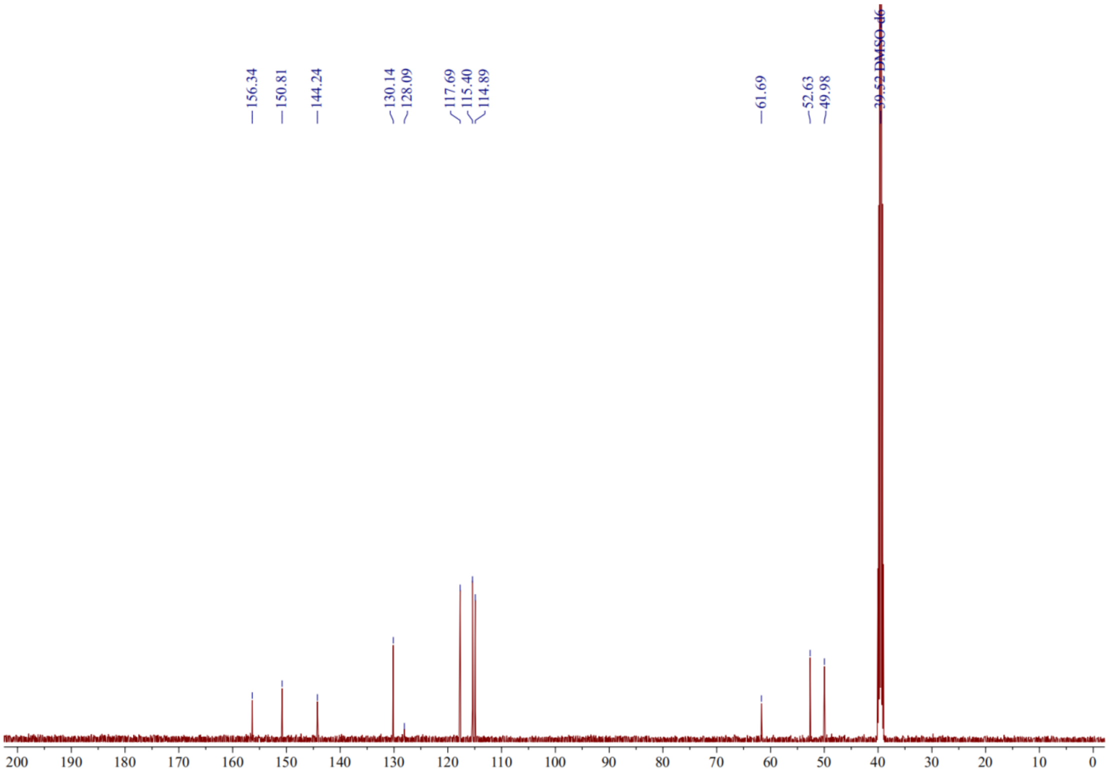

2-((4-(4-hydroxyphenyl)piperazin-1-yl)methyl)benzonitrile **(AI10-m37)**

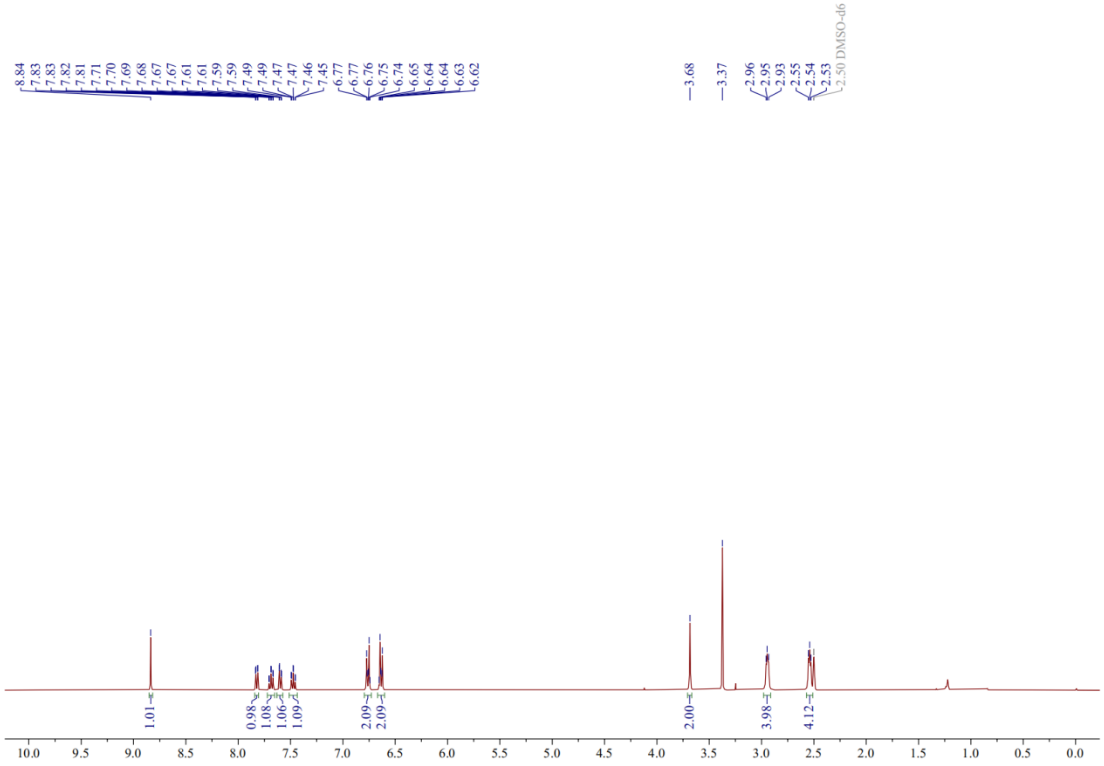

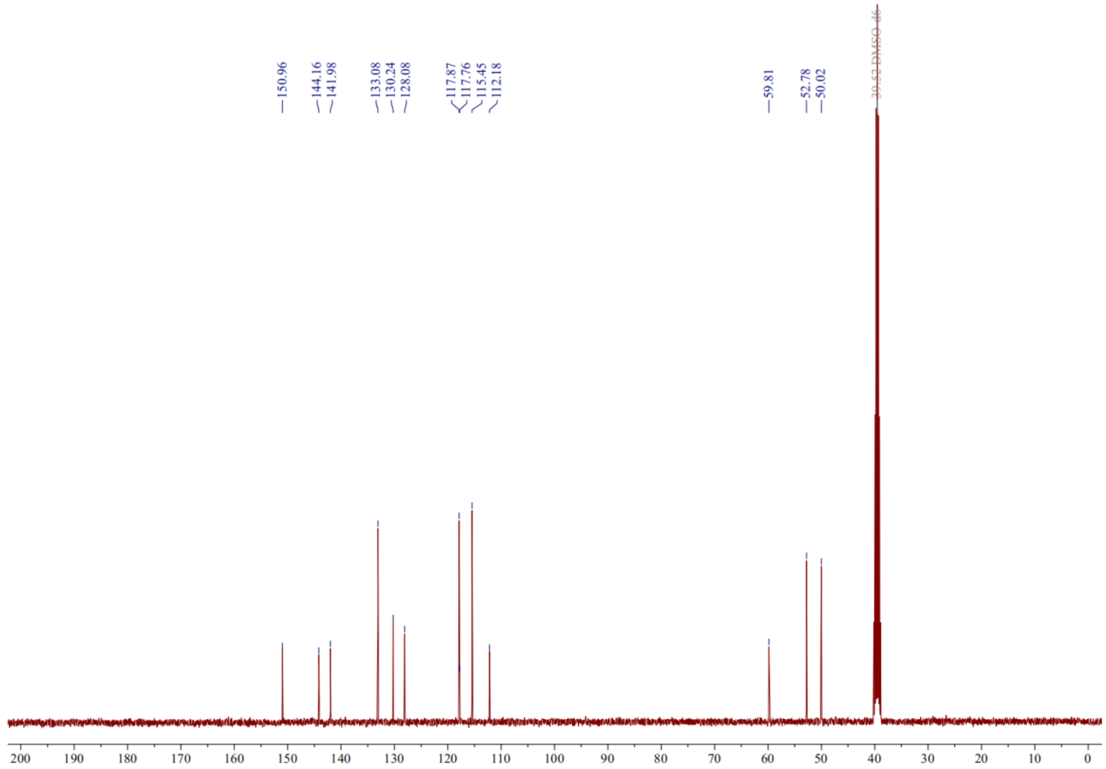

***3-((4-(4-hydroxyphenyl)piperazin-1-yl)methyl)benzonitrile* (AI10-m38)**

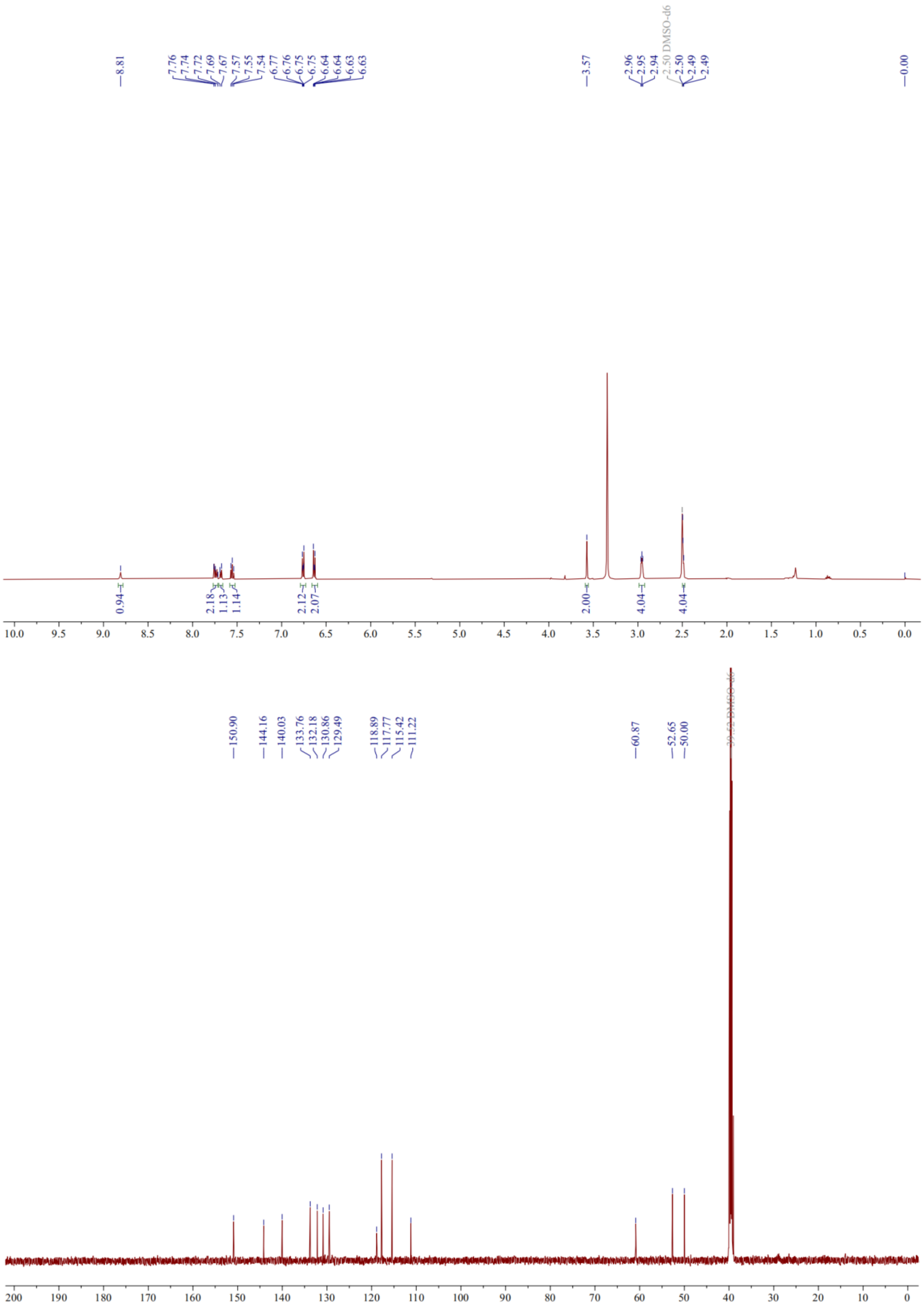

4-((4-(4-hydroxyphenyl)piperazin-1-yl)methyl)benzonitrile **(AI10-m39)**

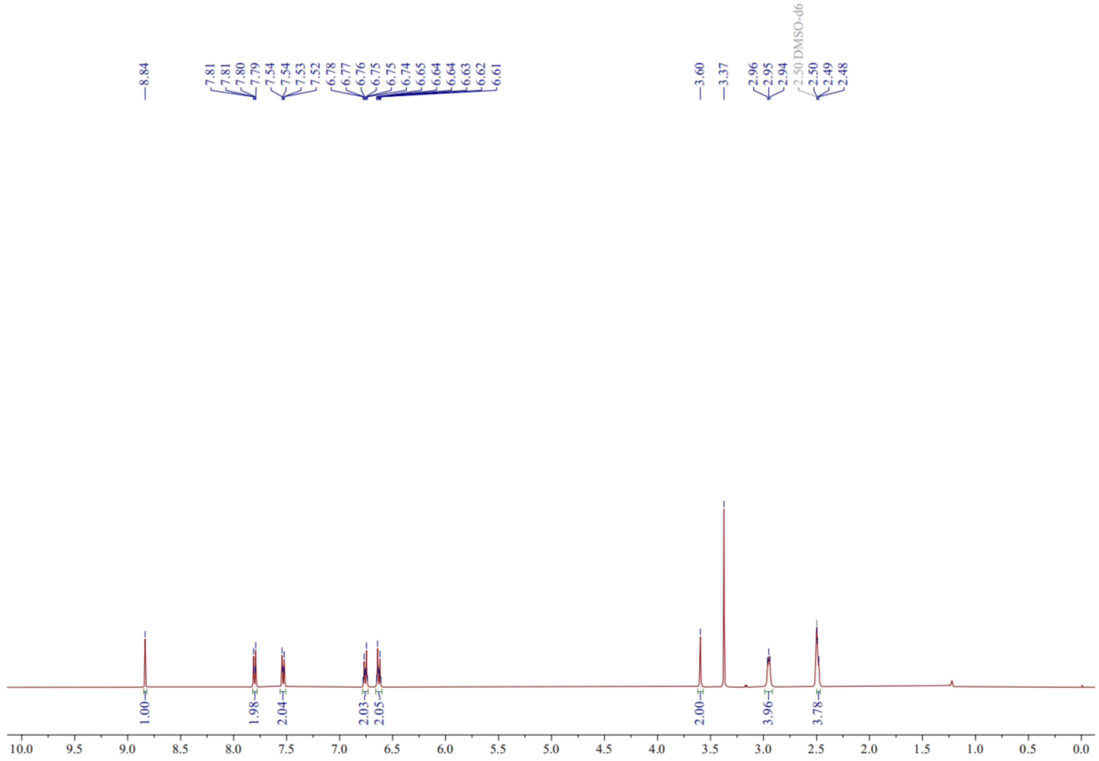

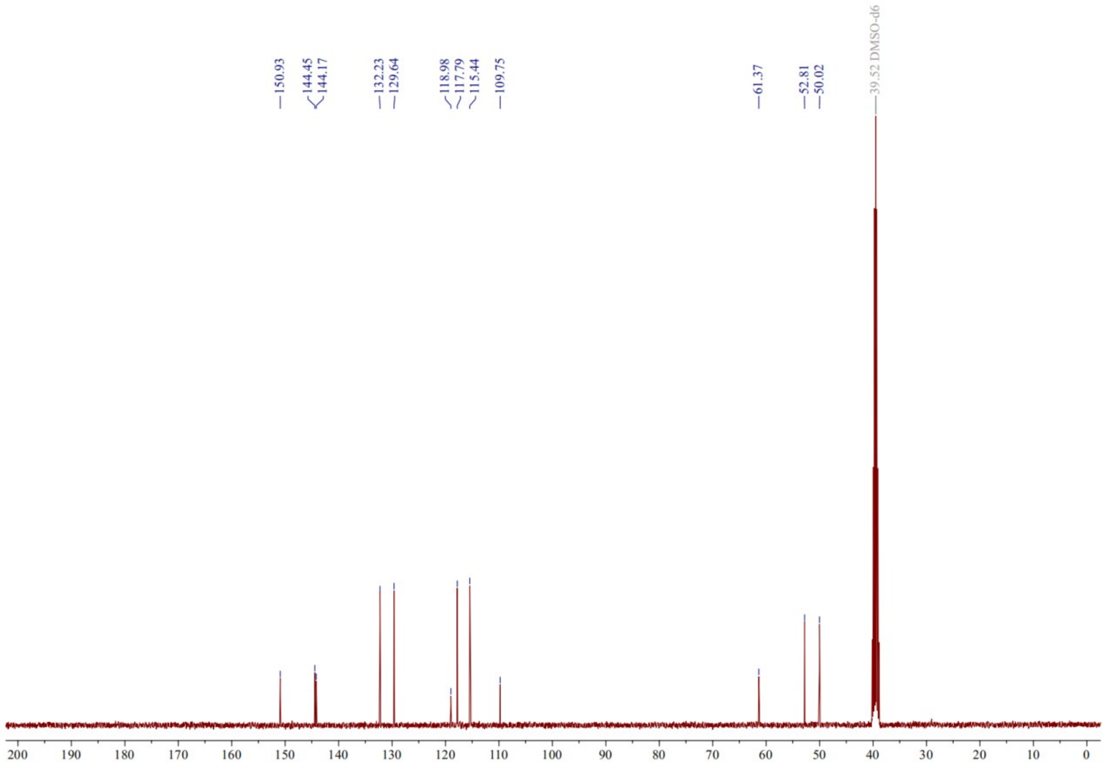

***4-(4-(3,5-dichlorobenzyl)piperazin-1-yl)phenol* (AI10-m40)**

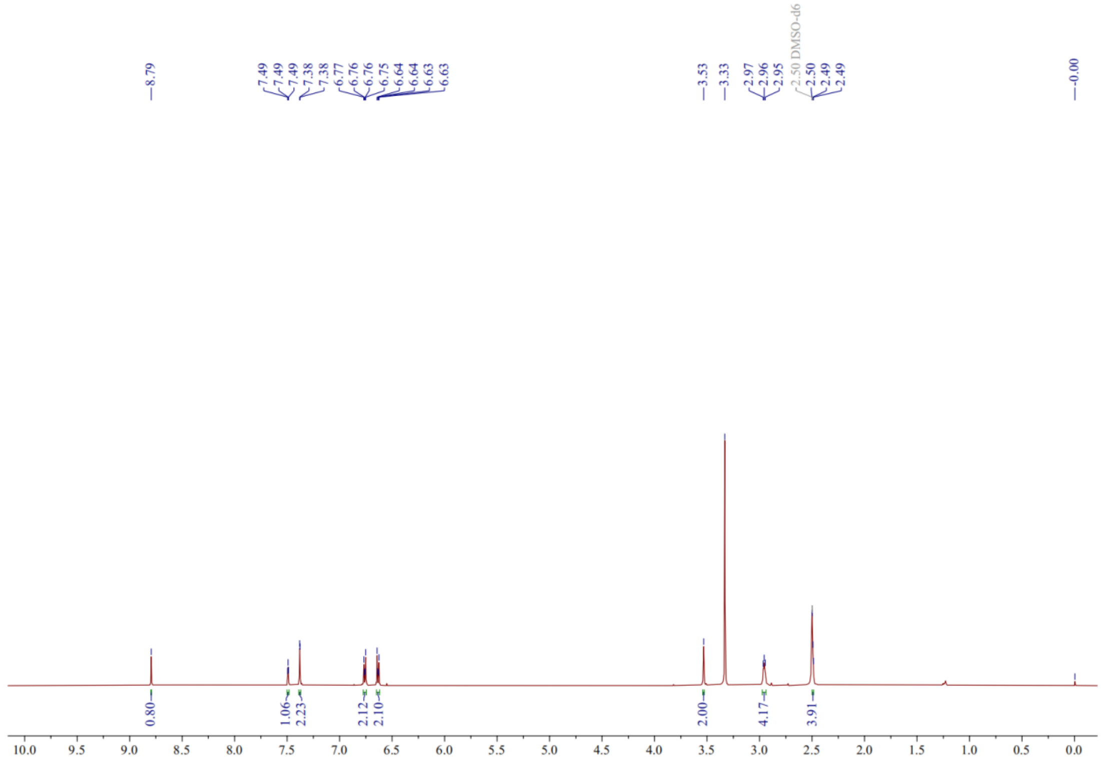

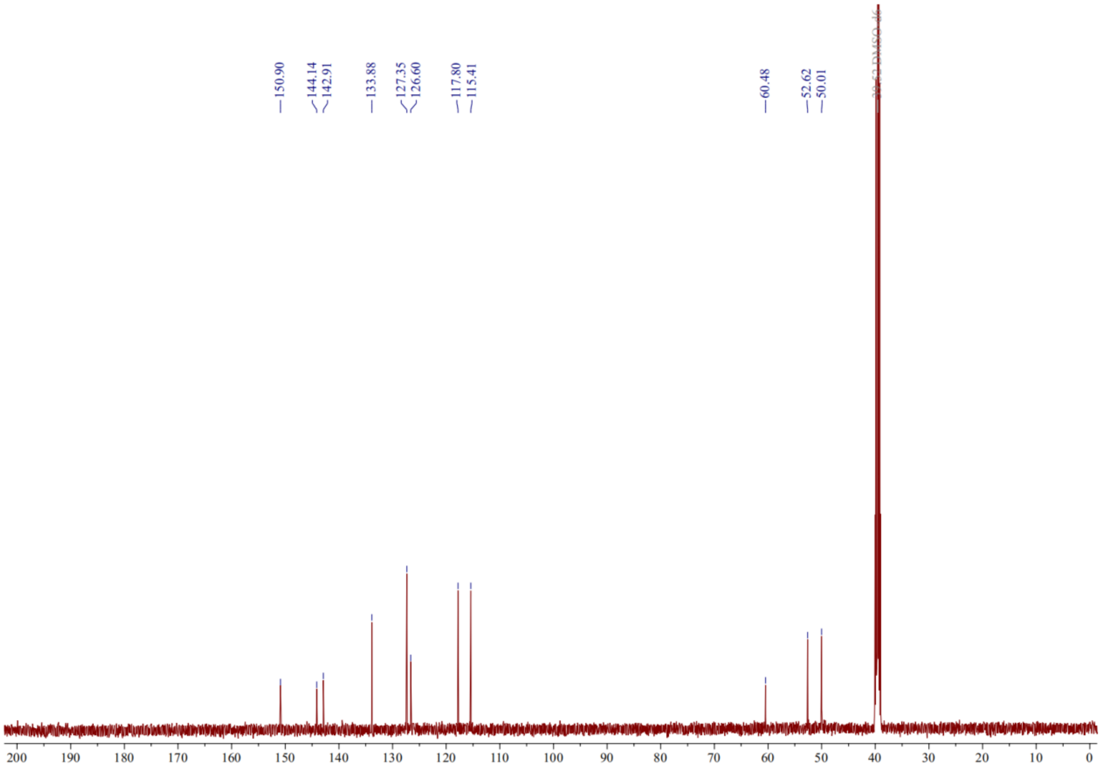

***4-(4-(3,4-dichlorobenzyl)piperazin-1-yl)phenol* (AI10-m41)**

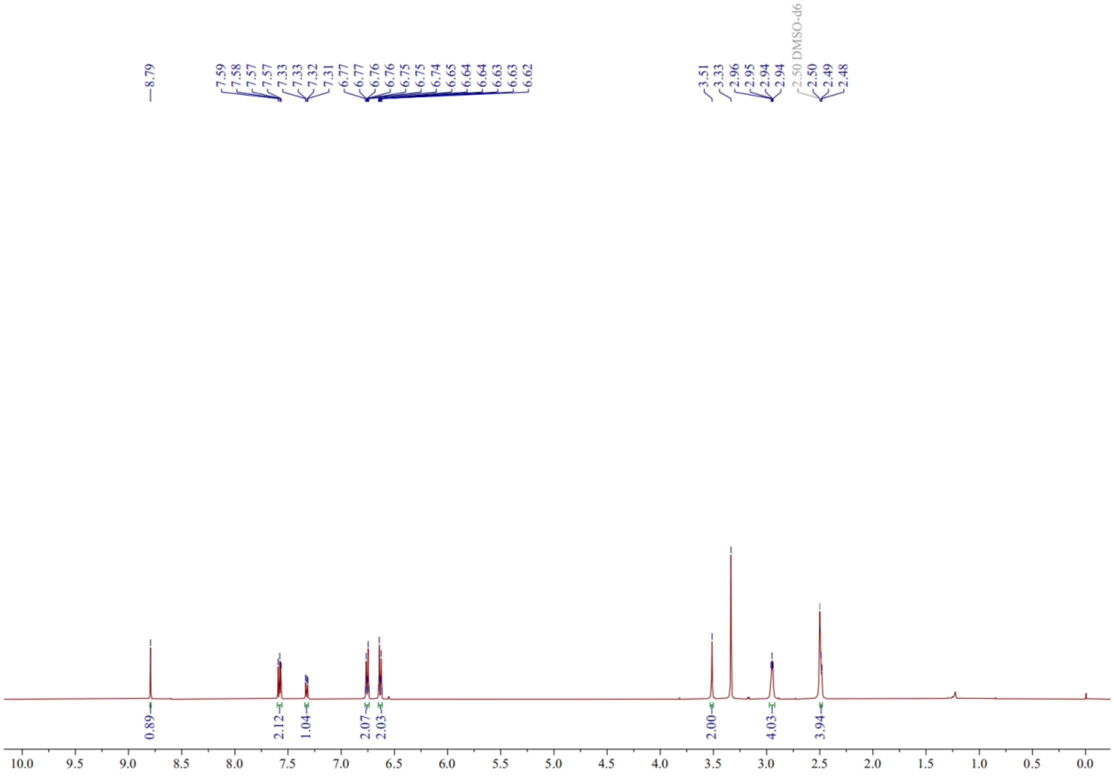

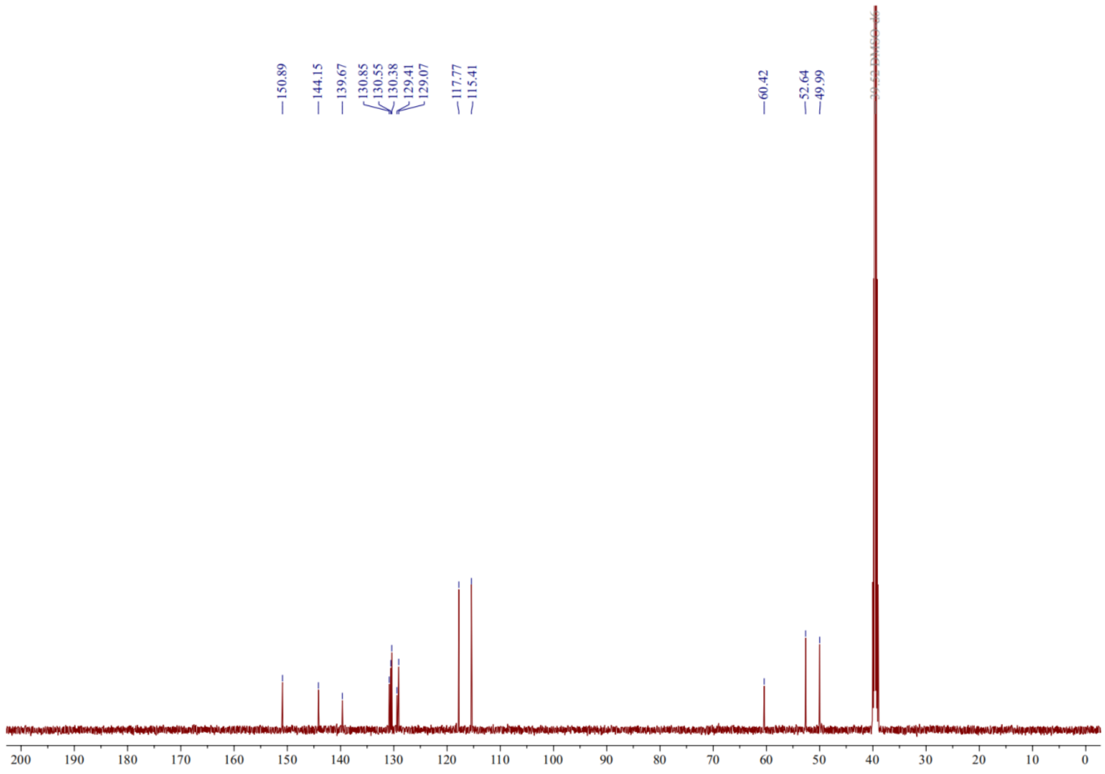

4-((4-(4-hydroxyphenyl)piperazin-1-yl)methyl)benzene-1,3-diol **(AI10-m42)**

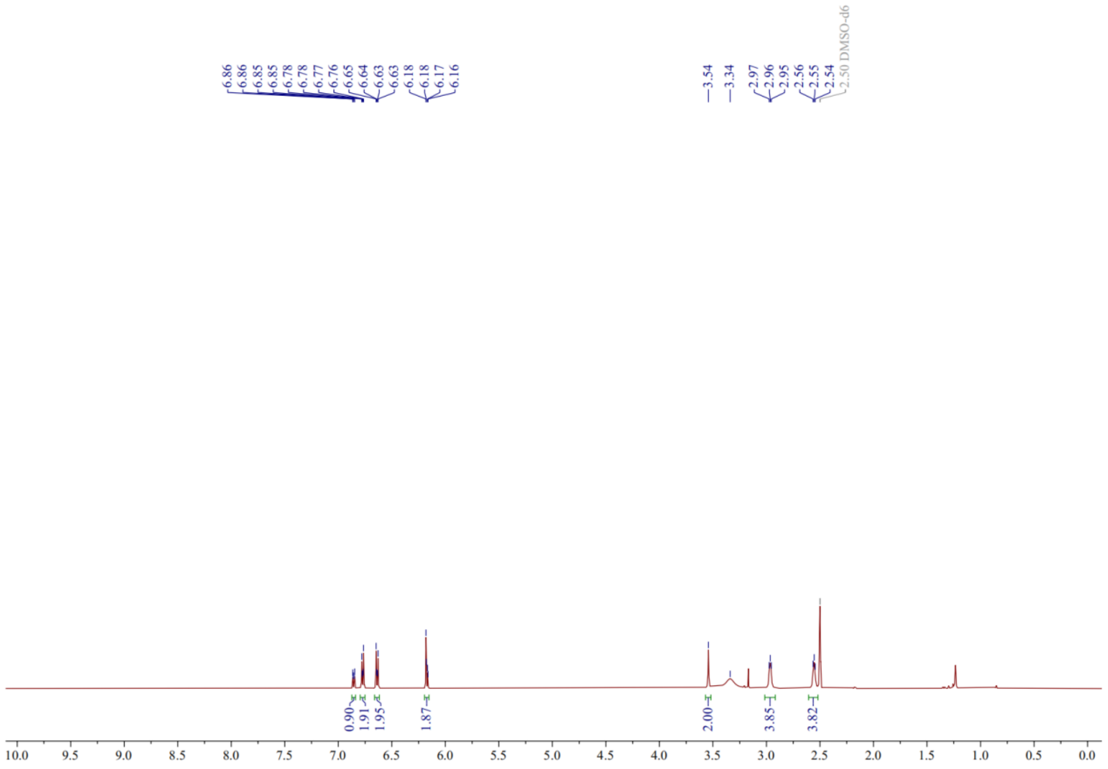

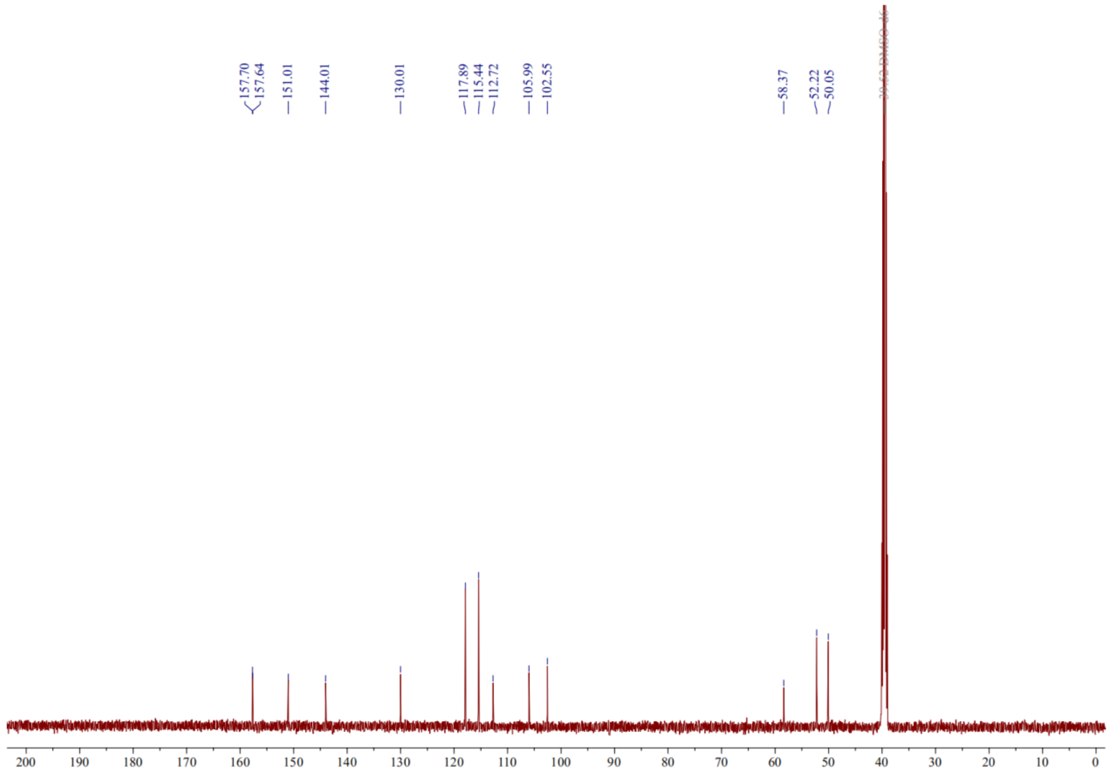

4-(4-(2,6-dimethoxybenzyl)piperazin-1-yl)phenol **(AI10-m43)**

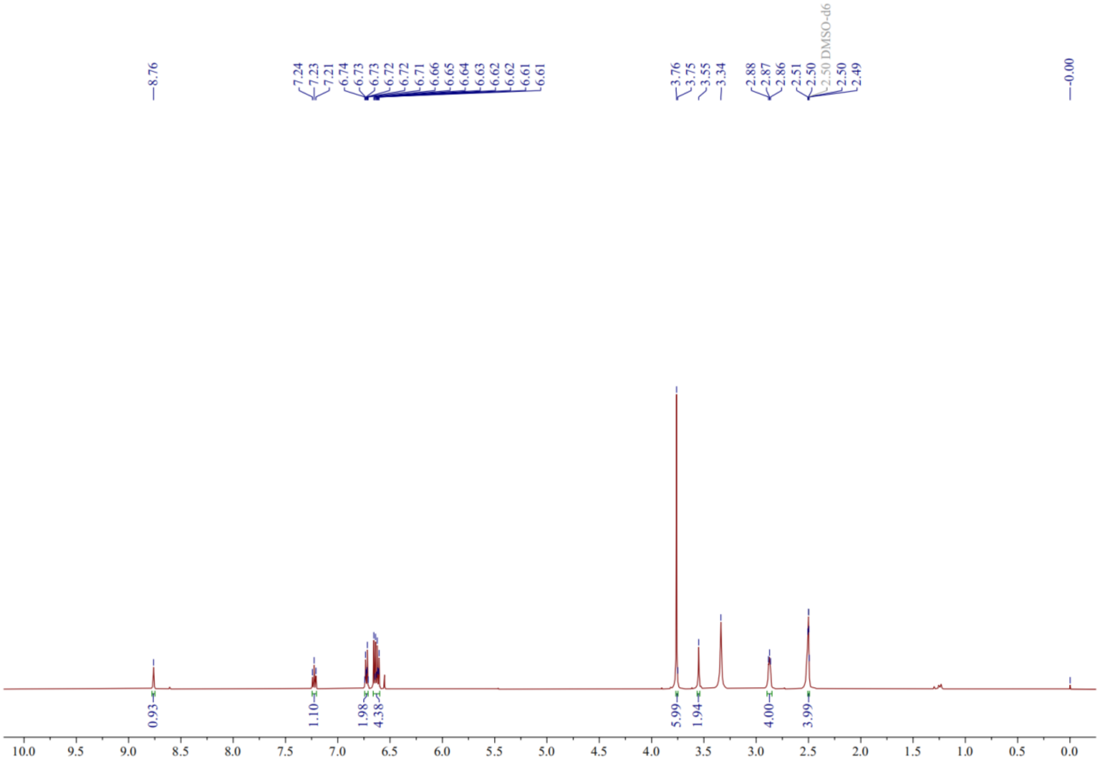

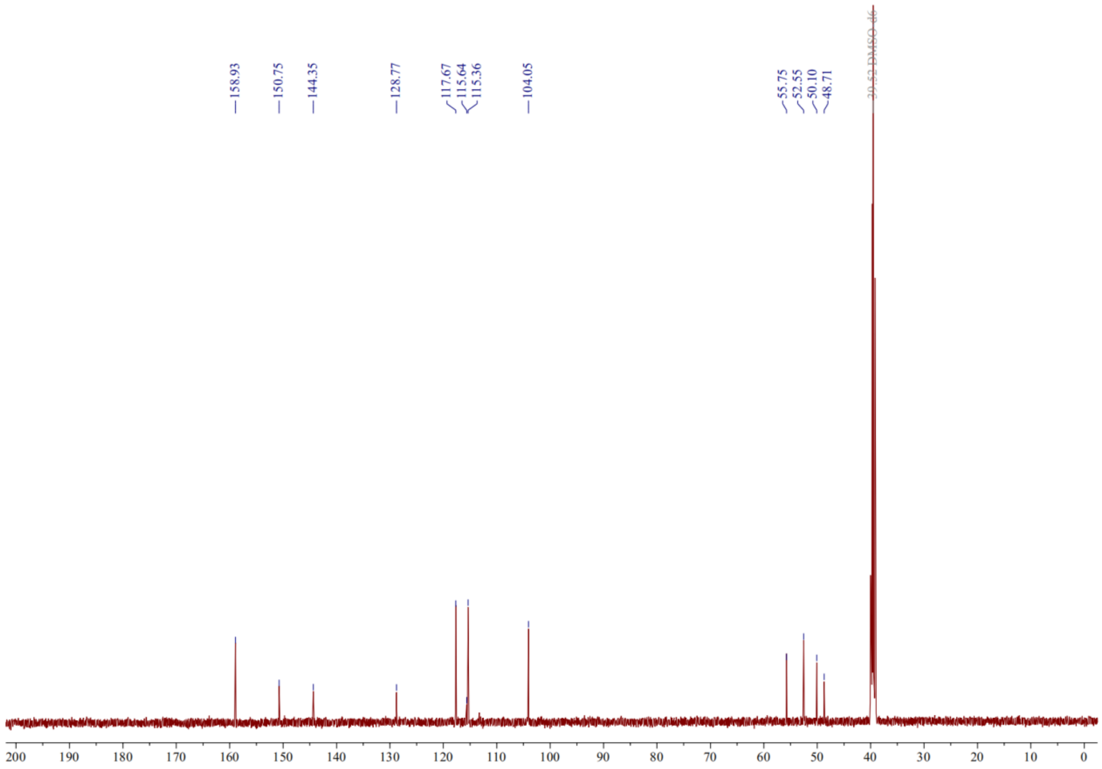

4-(4-(pyridin-3-ylmethyl)piperazin-1-yl)phenol **(AI10-m44)**

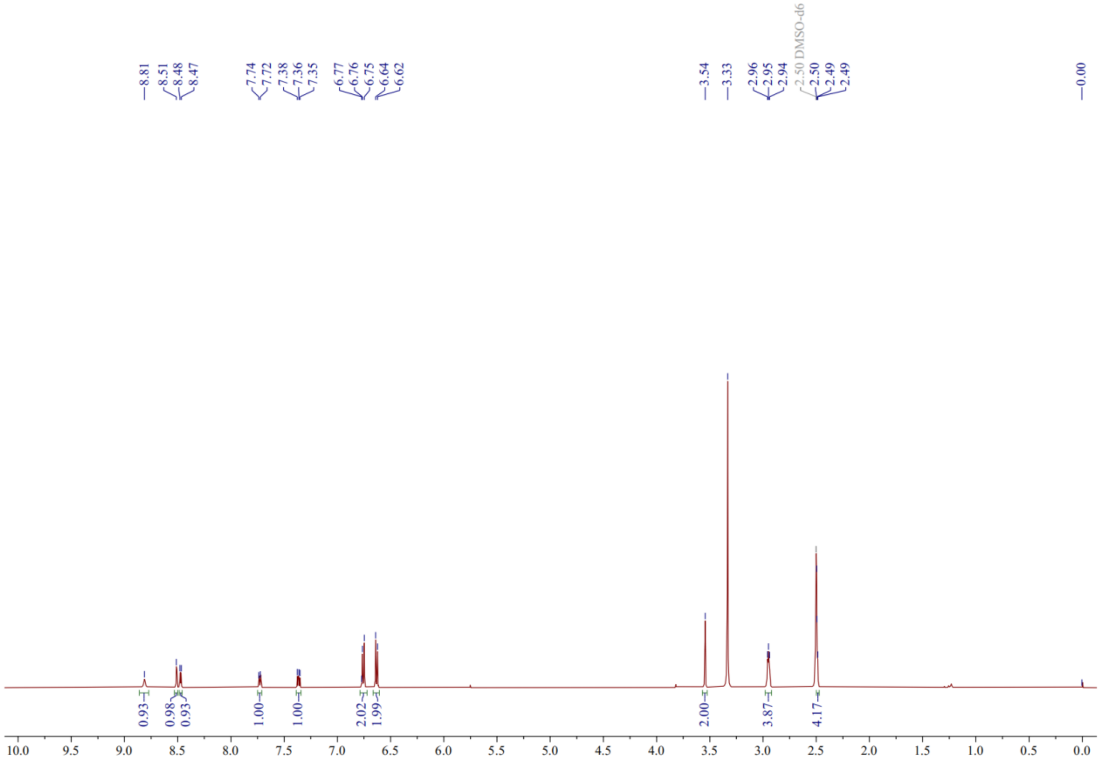

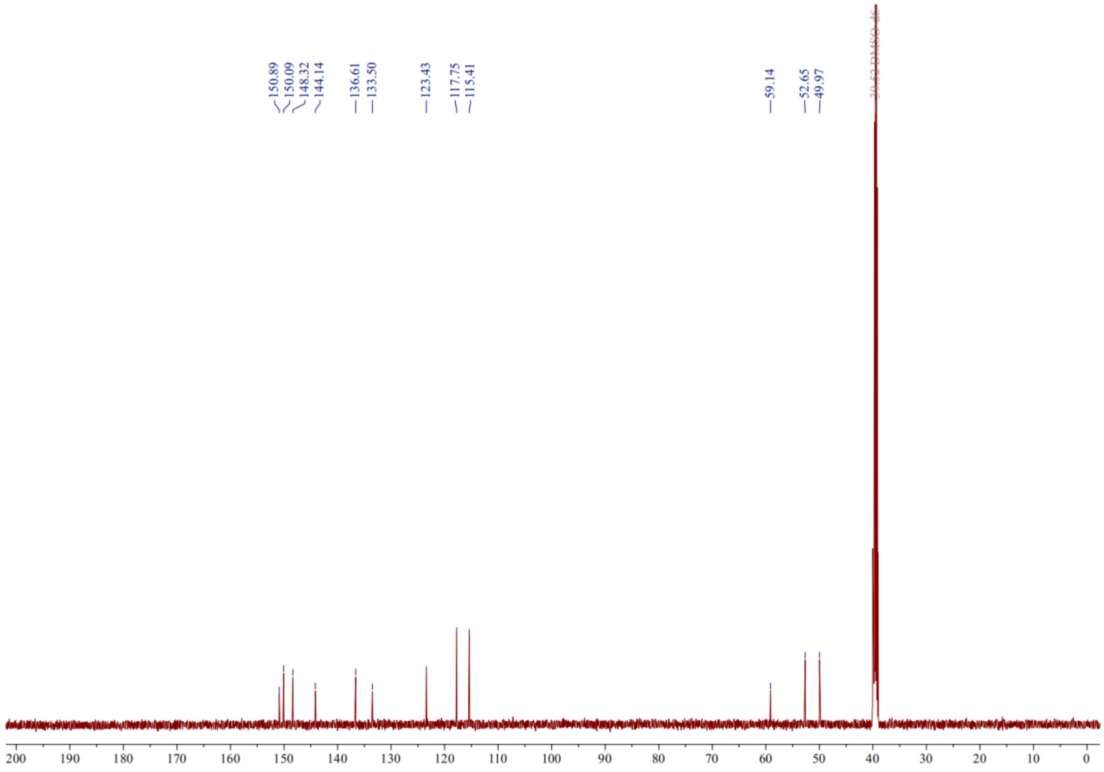

4-(4-(furan-2-ylmethyl)piperazin-1-yl)phenol **(AI10-m45)**

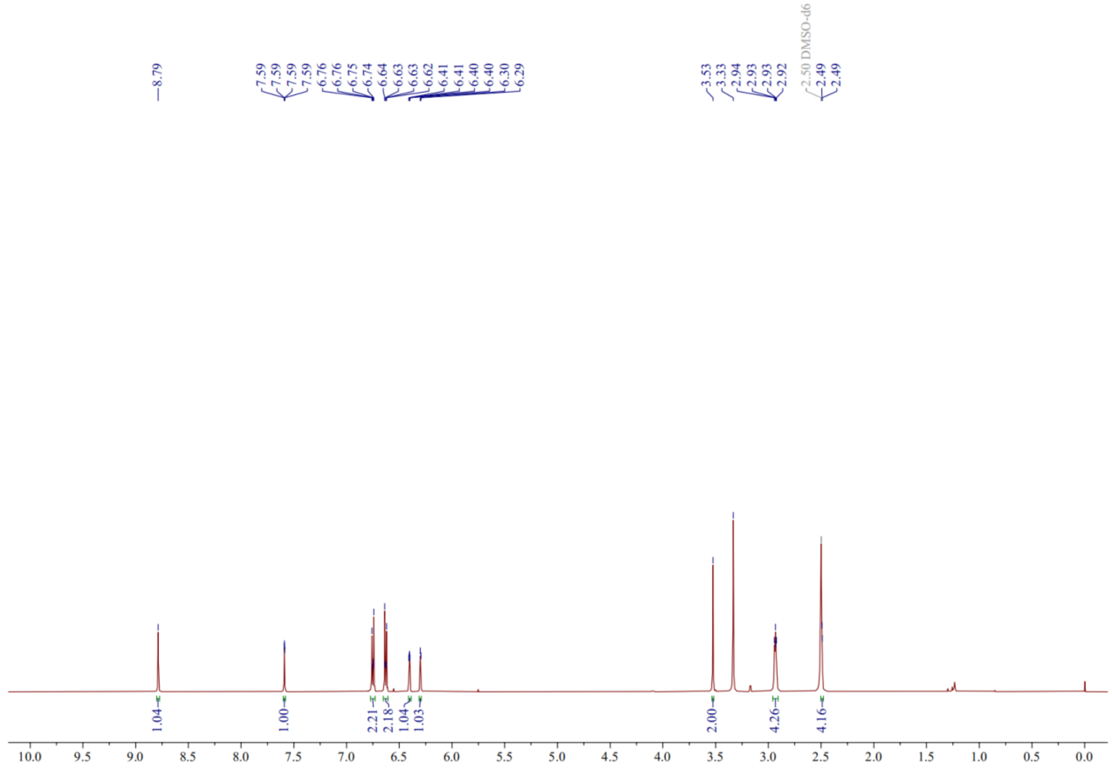

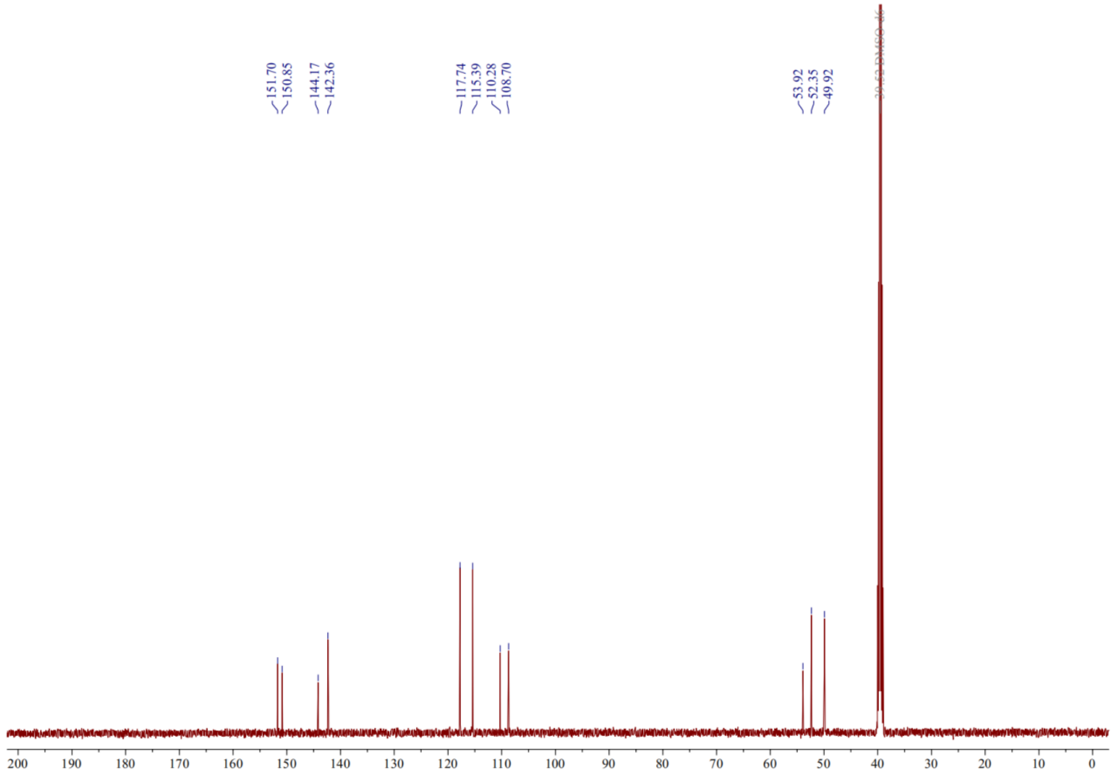

4-(4-(thiophen-2-ylmethyl)piperazin-1-yl)phenol **(AI10-m46)**

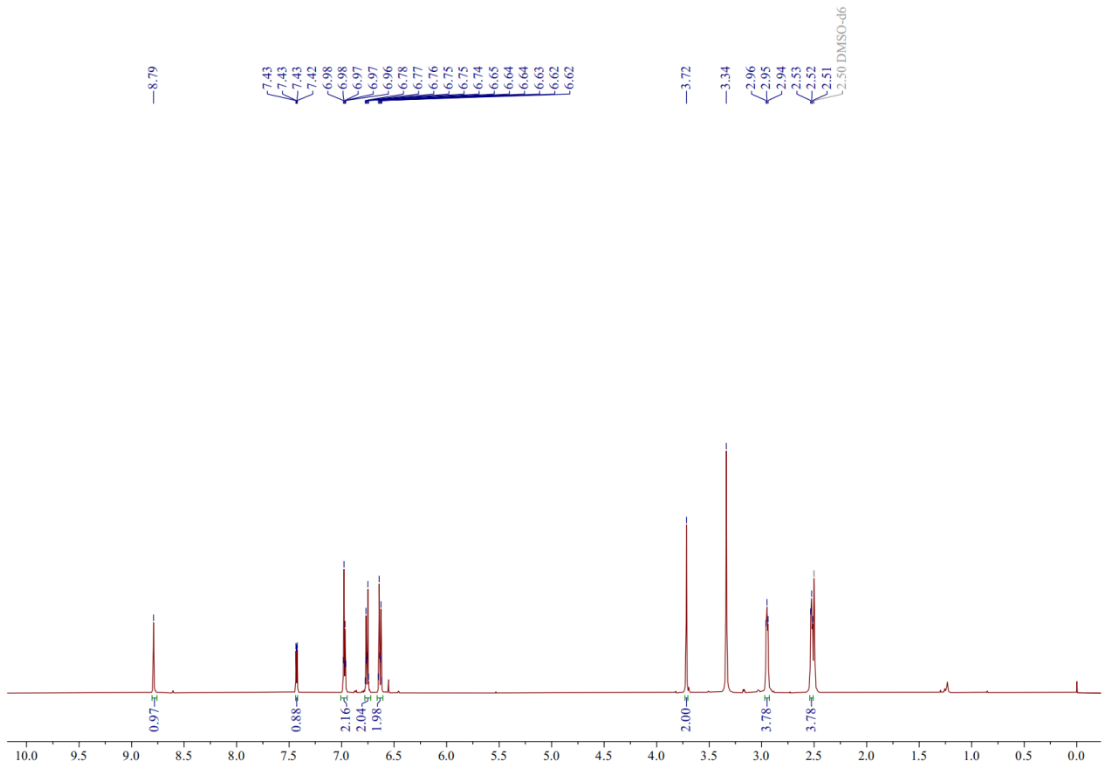

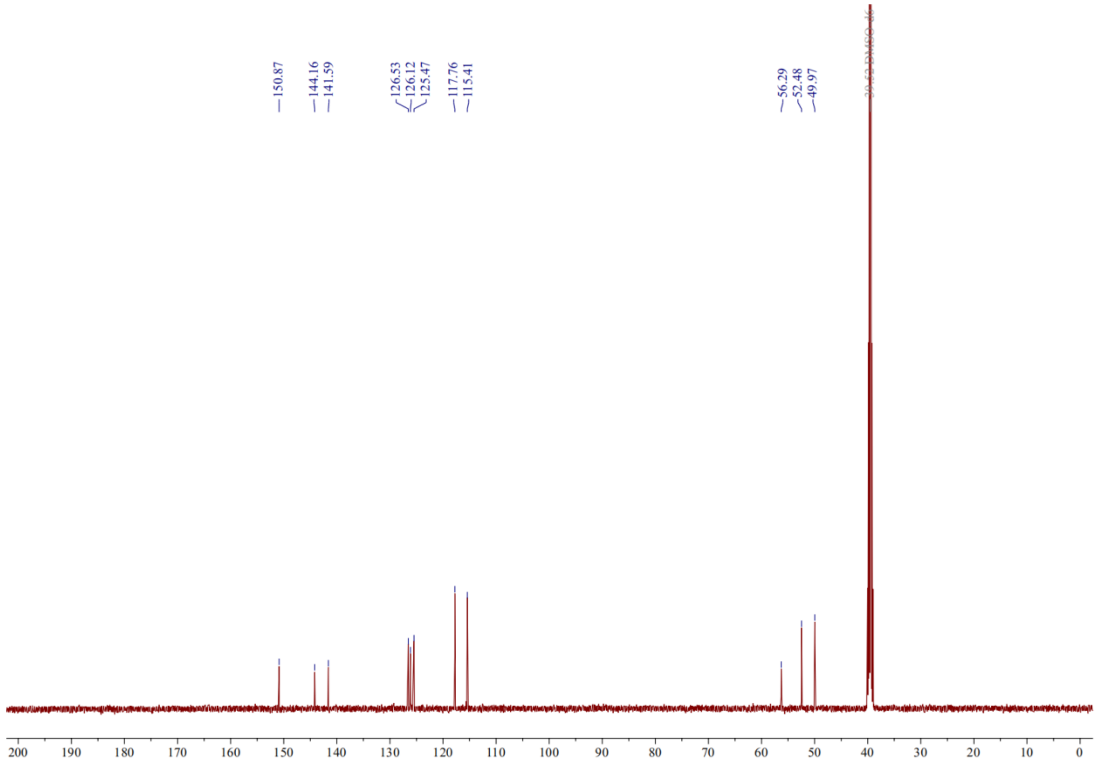

***4-(4-(3-methylbenzyl)piperazin-1-yl)benzene-1,3-diol* (AI10-m47)**

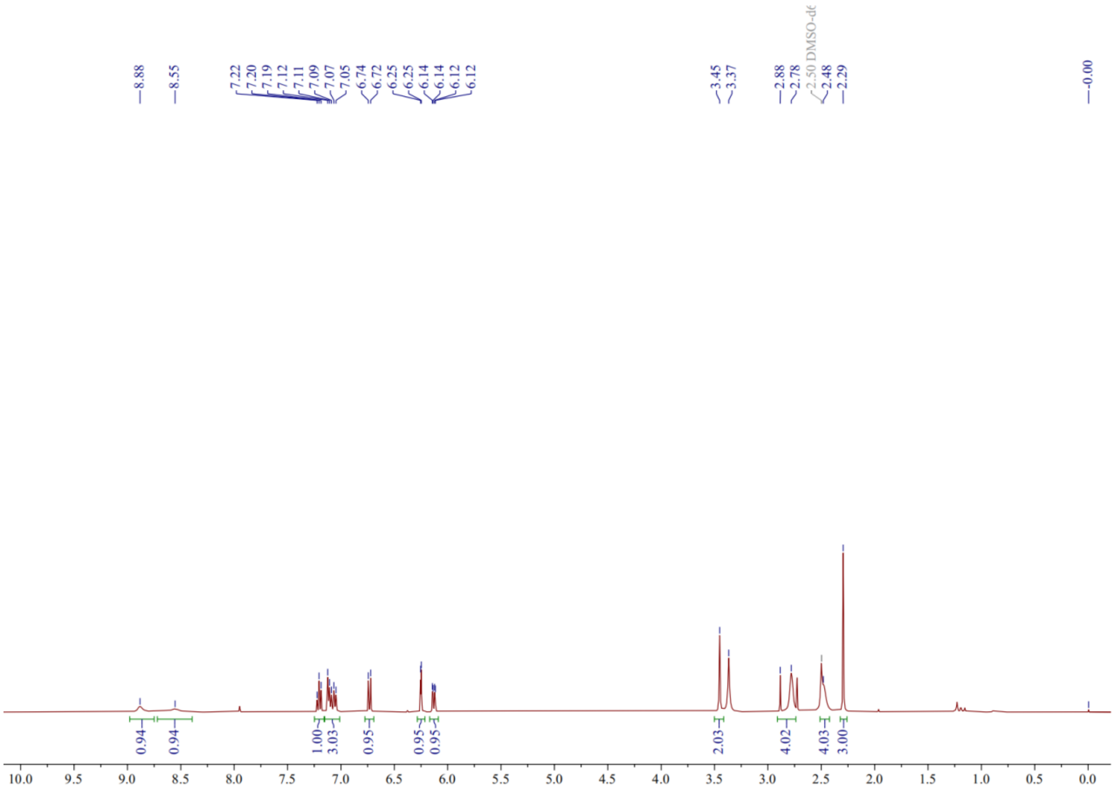

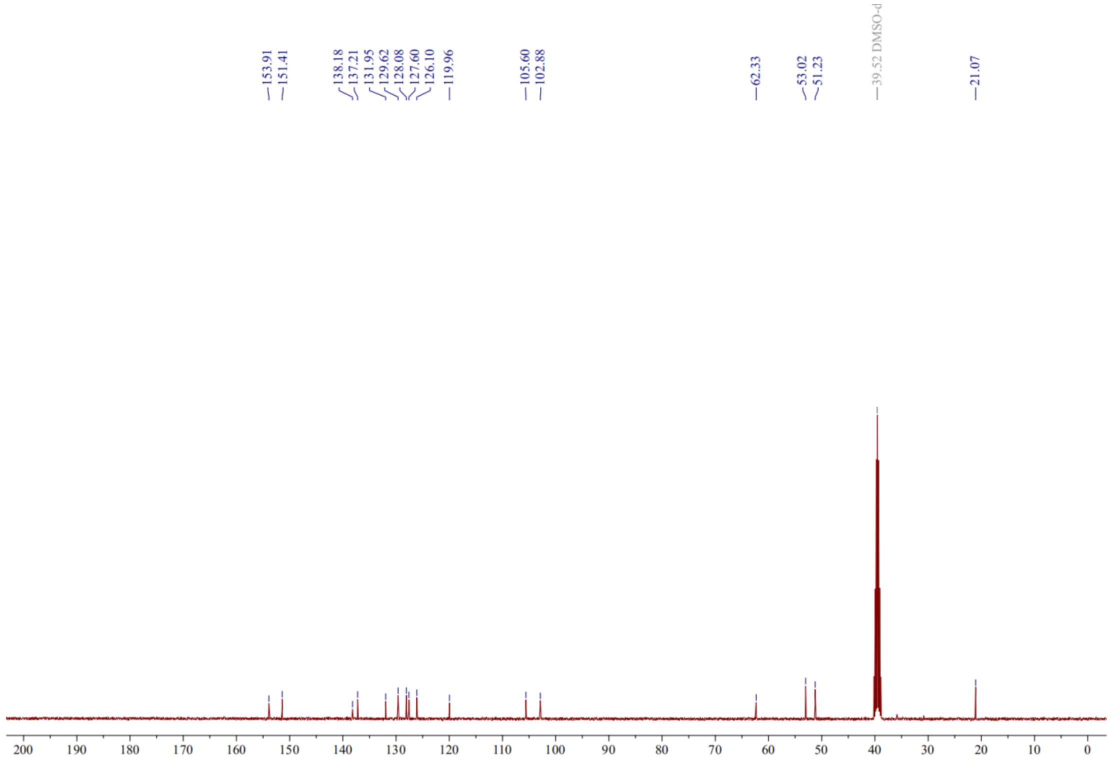

***(E)-4-(4-(3-(m-tolyl)allyl)piperazin-1-yl)phenol* (AI10-m48)**

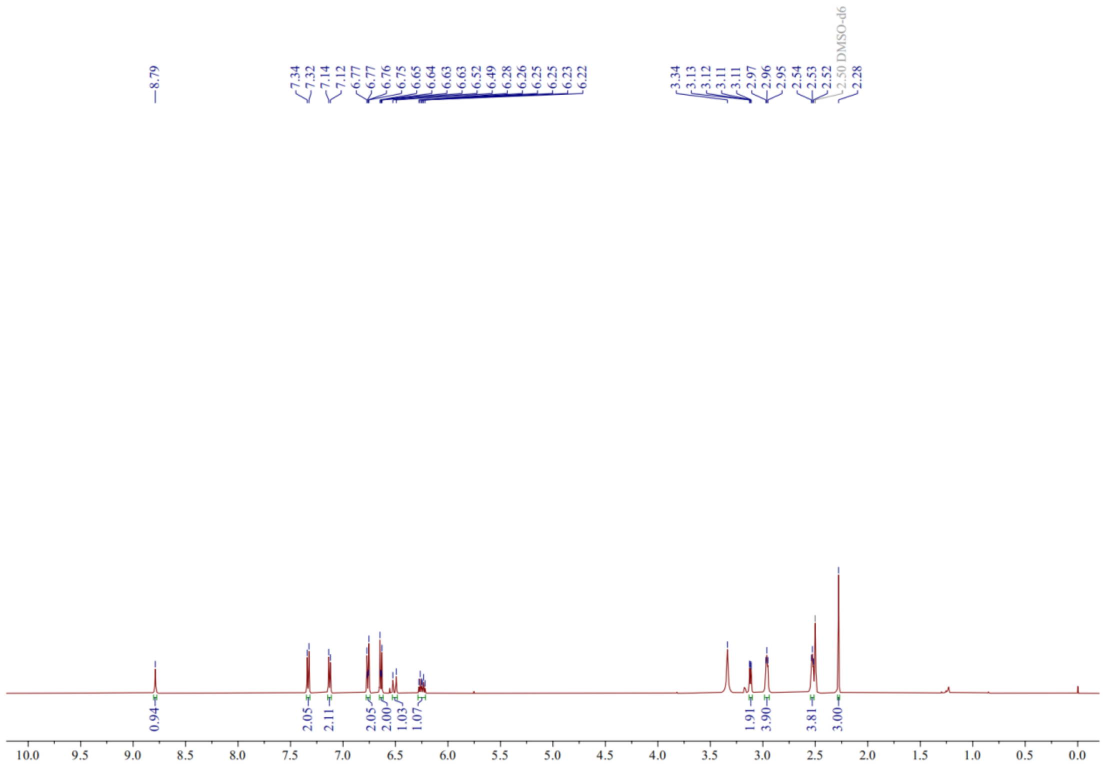

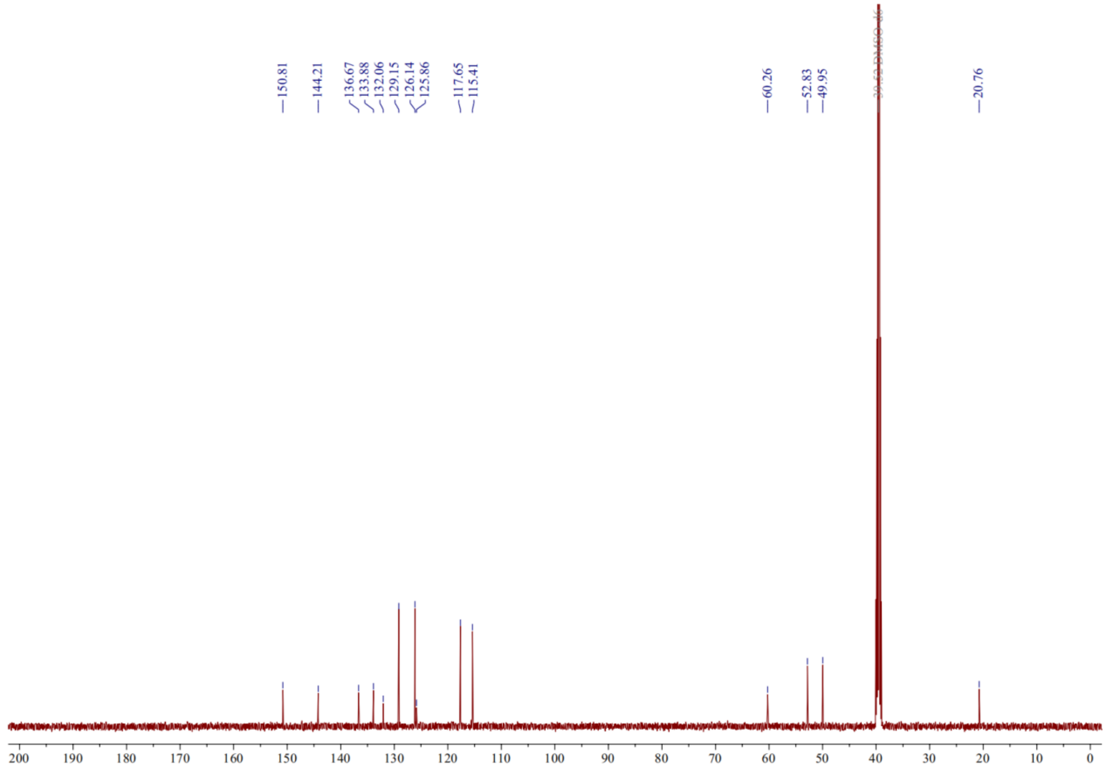

***(E)*-4-(4-(3-(4-chlorophenyl)allyl)piperazin-1-yl)phenol** **(AI10-m49)**

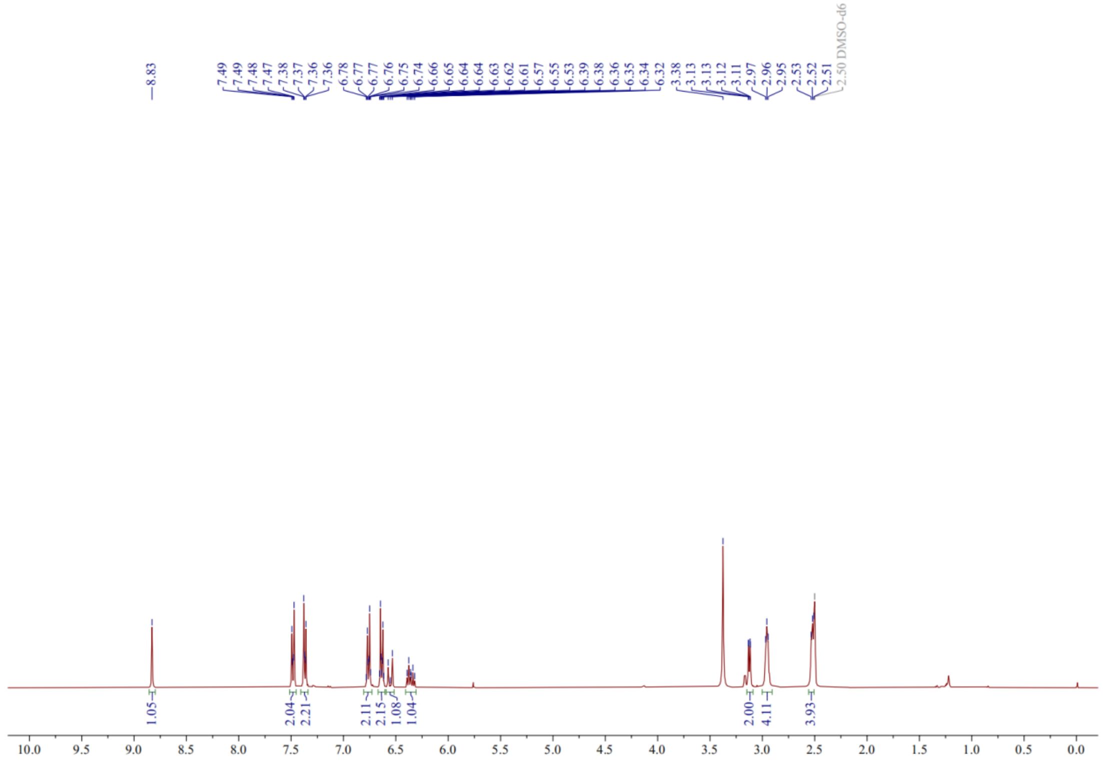

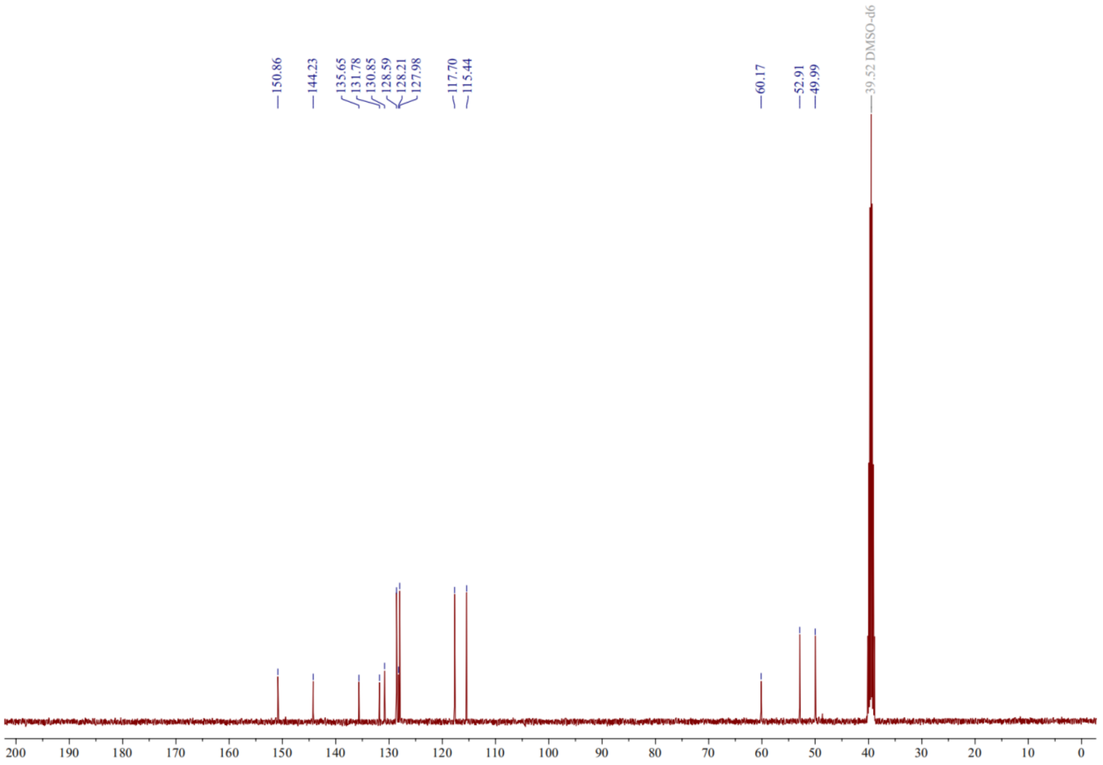

4-(4-(4-fluorophenethyl)piperazin-1-yl)phenol **(AI10-m50)**

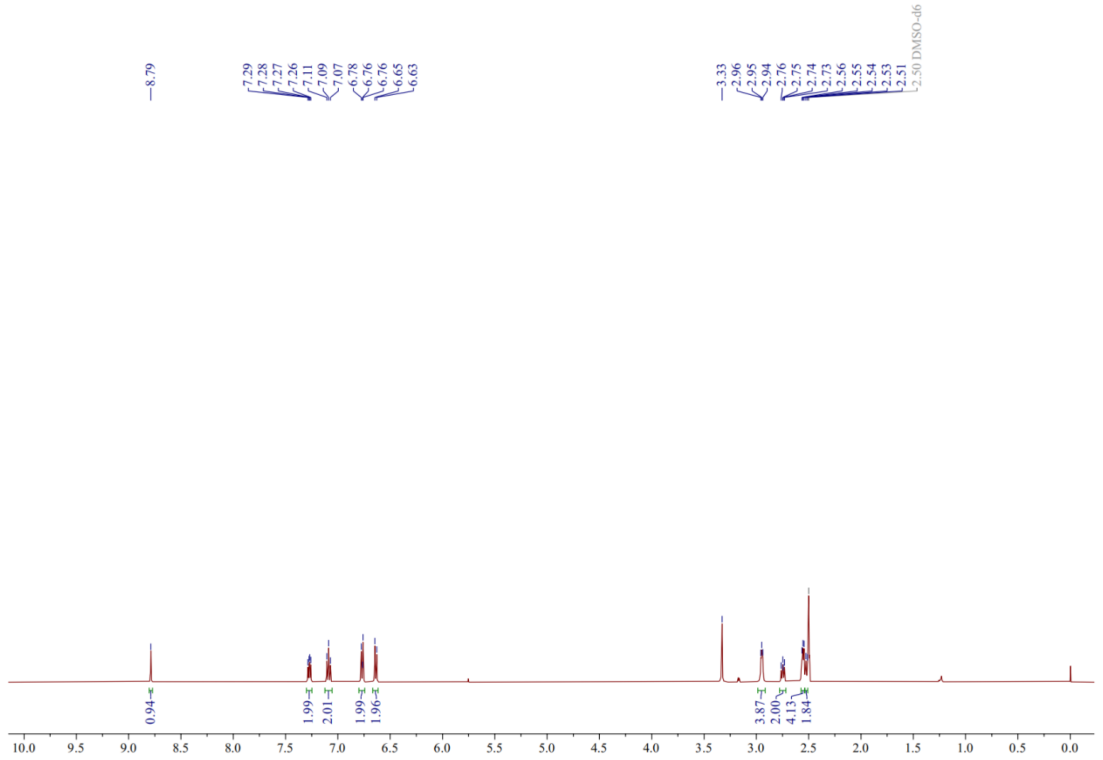

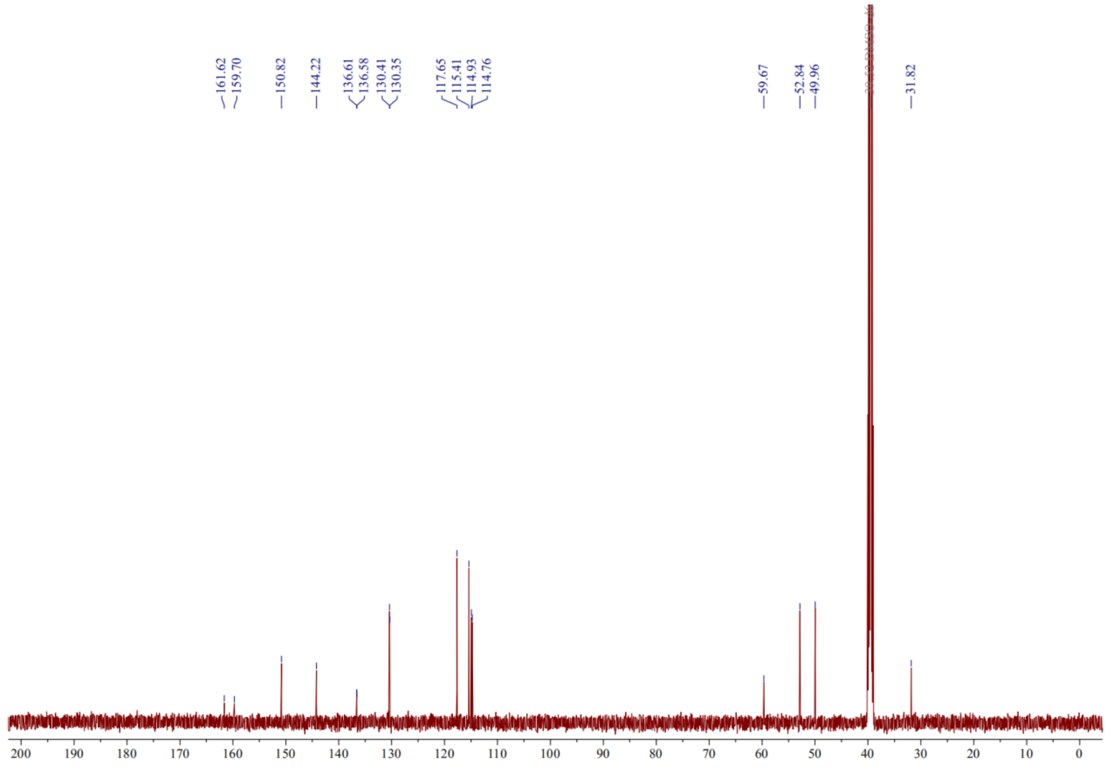

(4-(4-hydroxyphenyl)piperidin-1-yl)(pyridin-4-yl)methanone **(AI10-m51)**

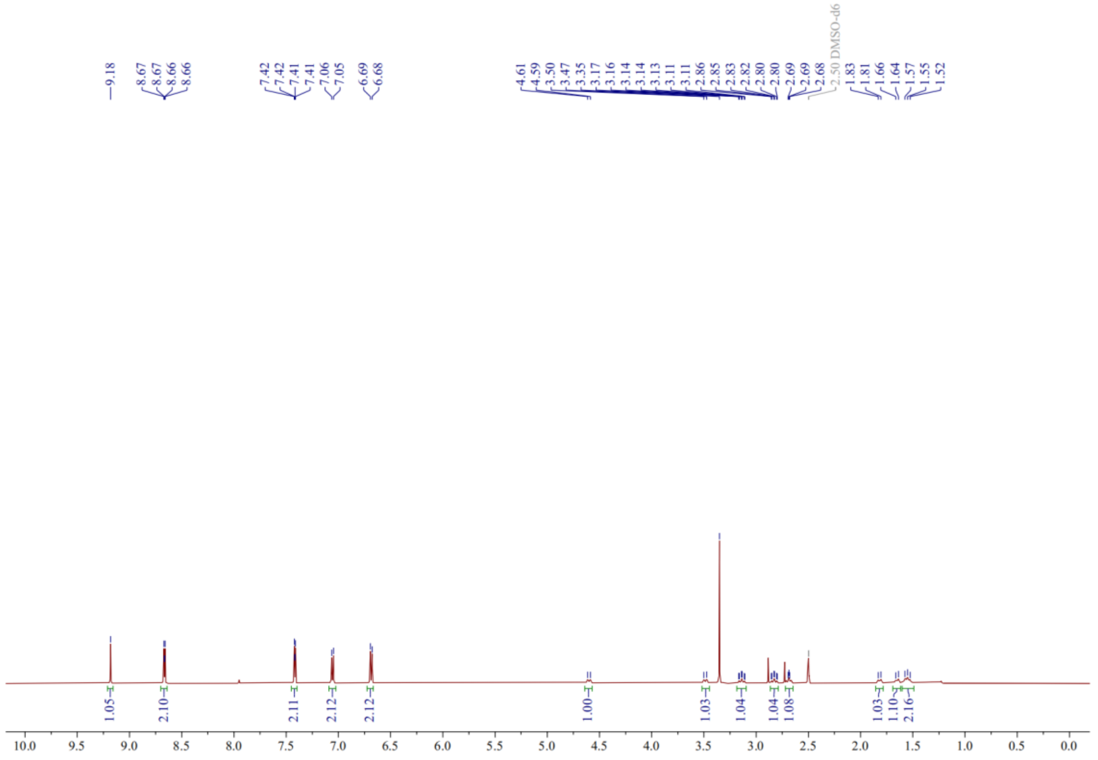

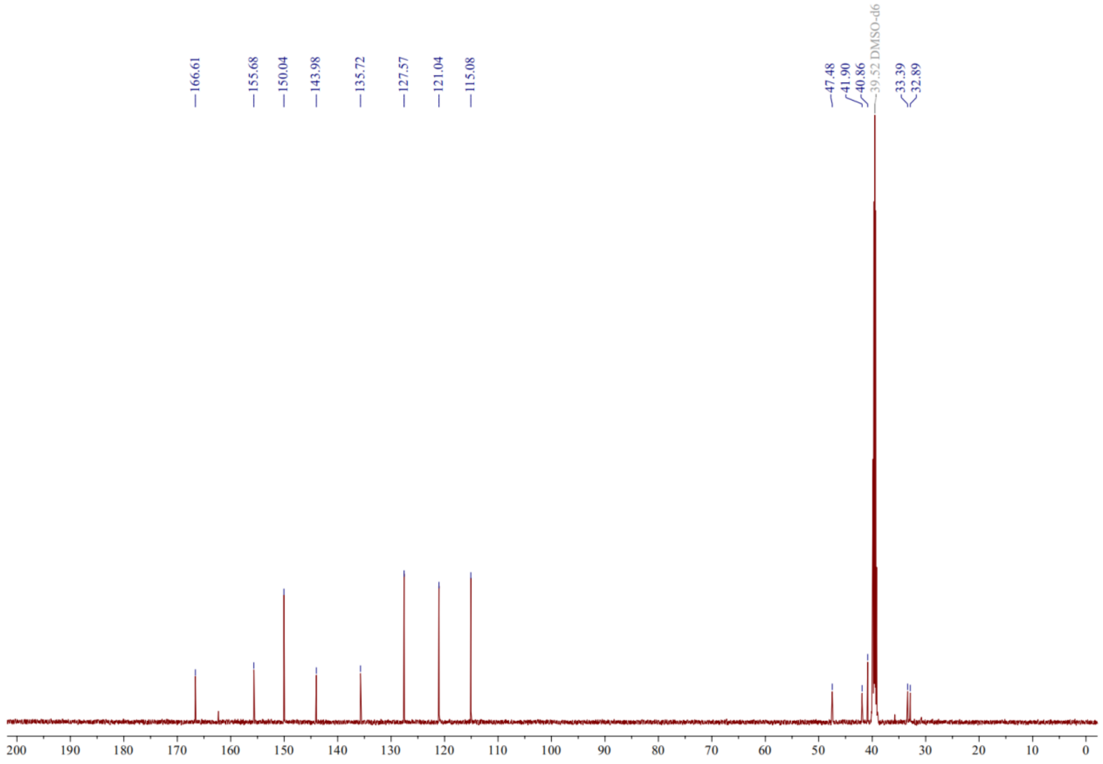

(E)-3-(2,4-dihydroxyphenyl)-1-(4-(4-hydroxyphenyl)piperidin-1-yl)prop-2-en-1-one **(AI10-m52)**

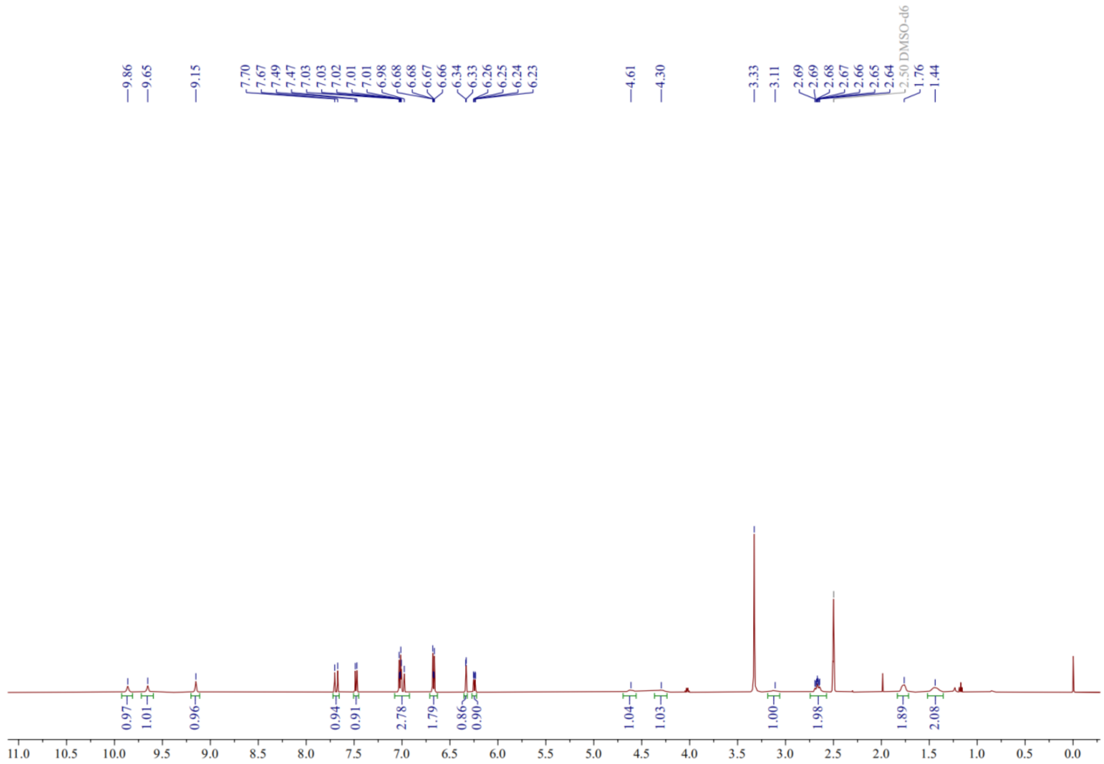

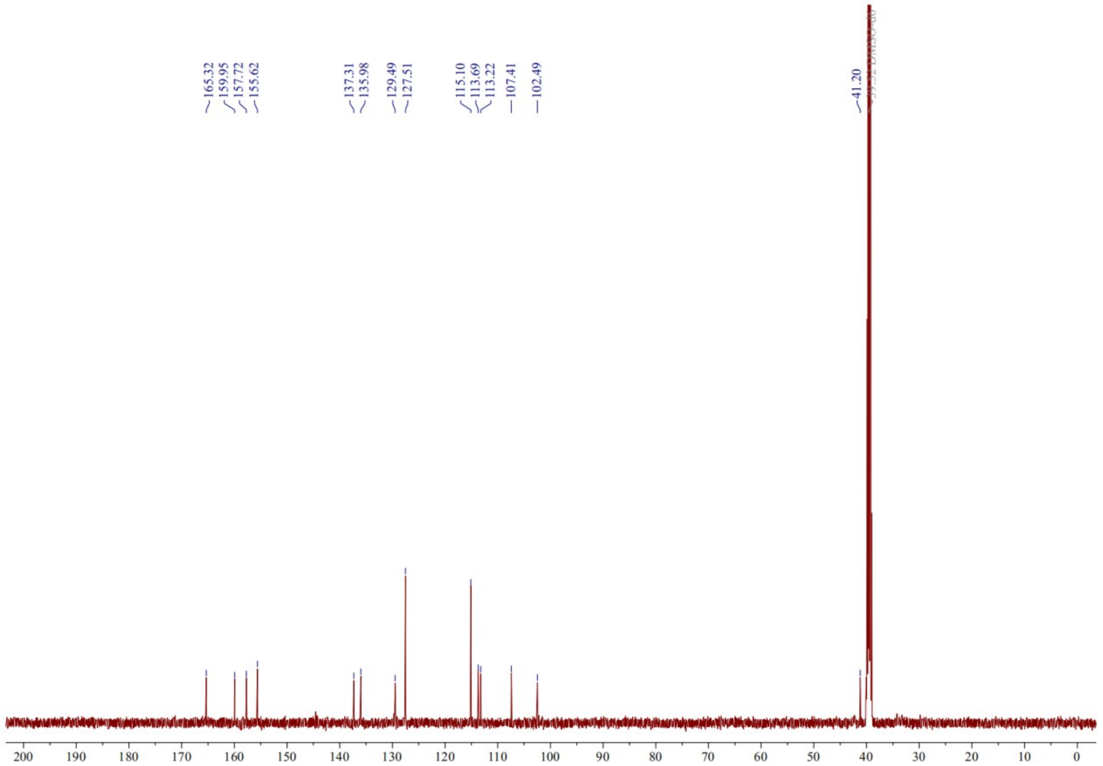

***4-(1-(3-methylbenzyl)piperidin-4-yl)phenol* (AI10-m53)**

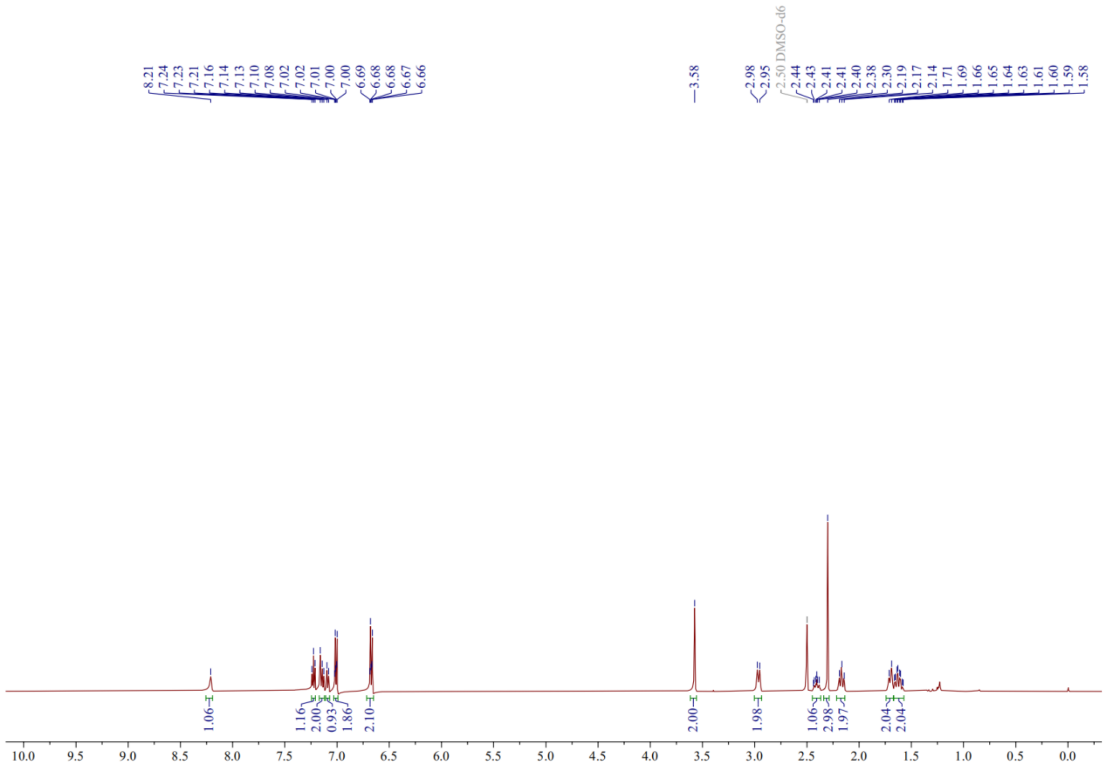

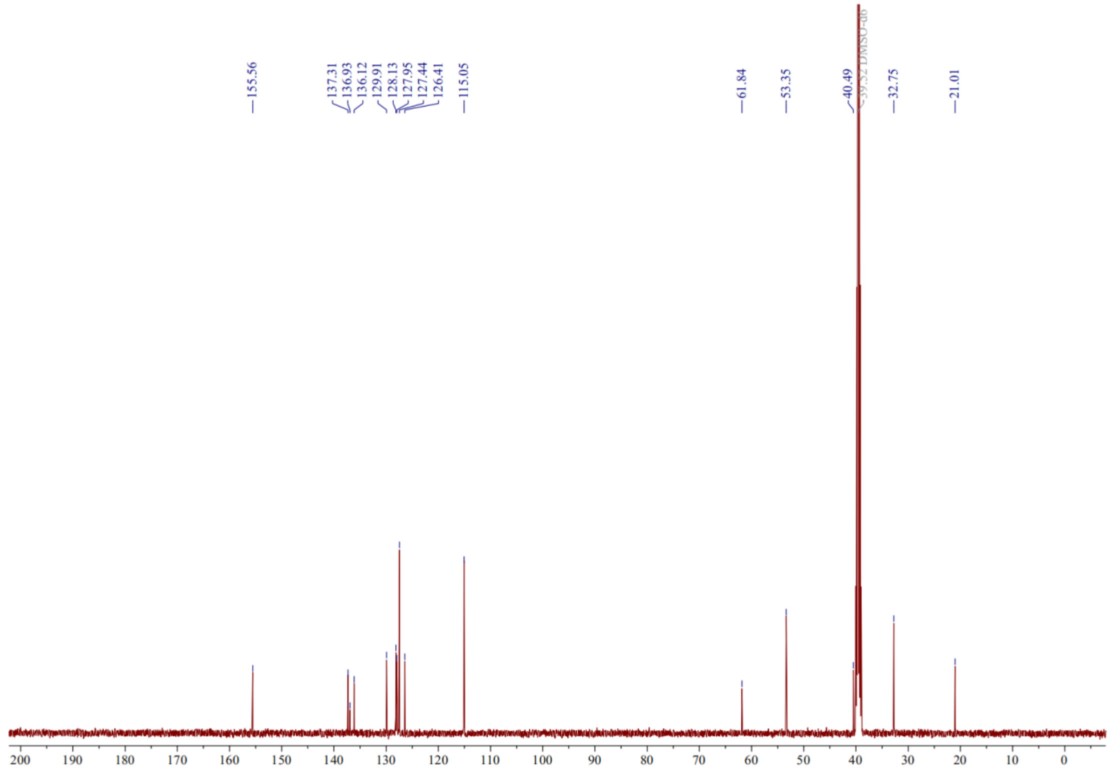

4-(1-(4-methylbenzyl)piperidin-4-yl)phenol **(AI10-m54)**

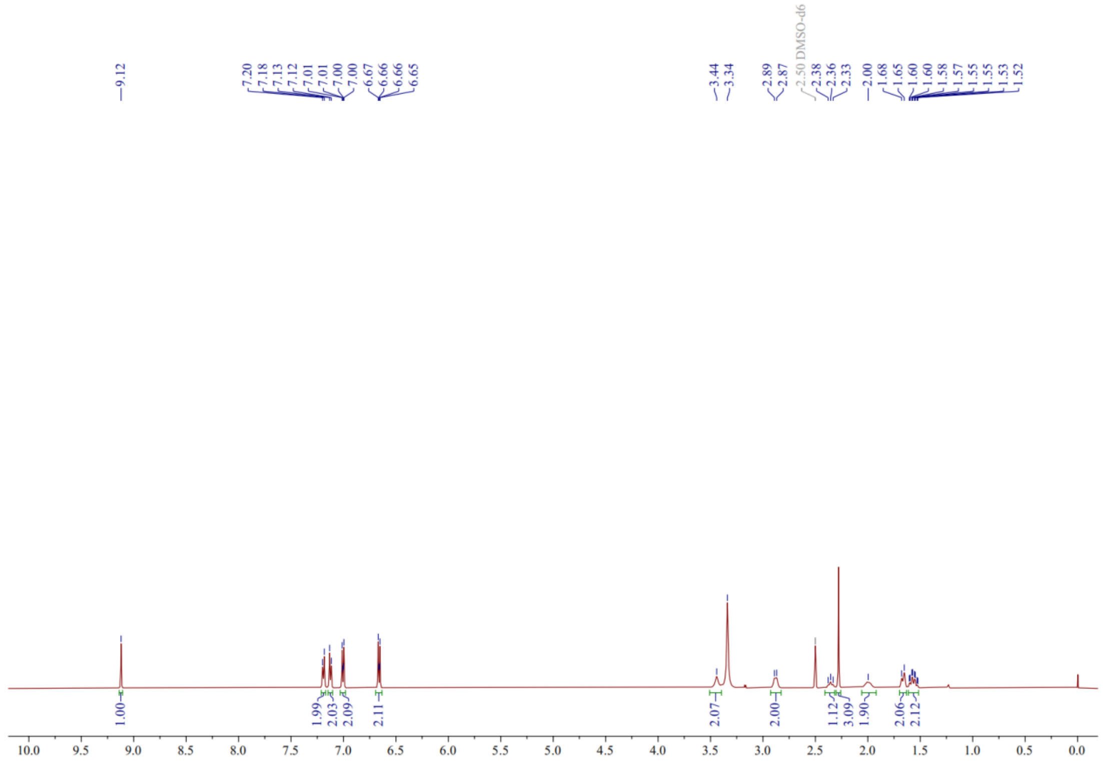

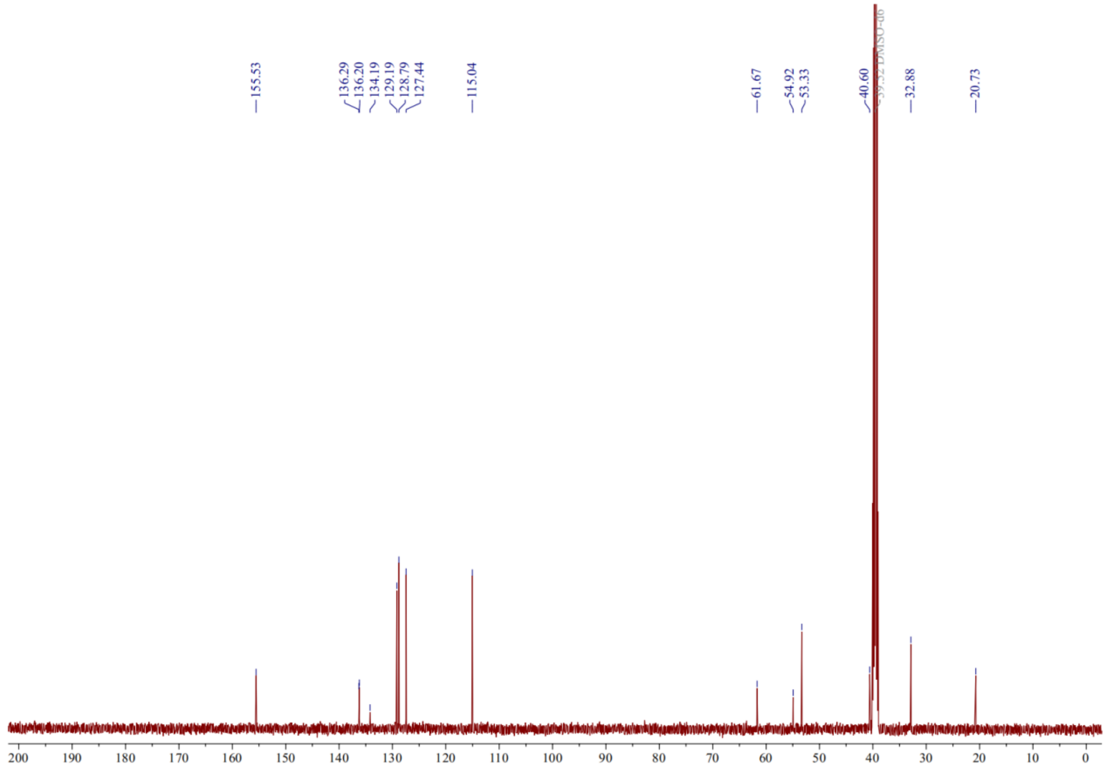

4-(1-(3,4-dichlorobenzyl)piperidin-4-yl)phenol **(AI10-m55)**

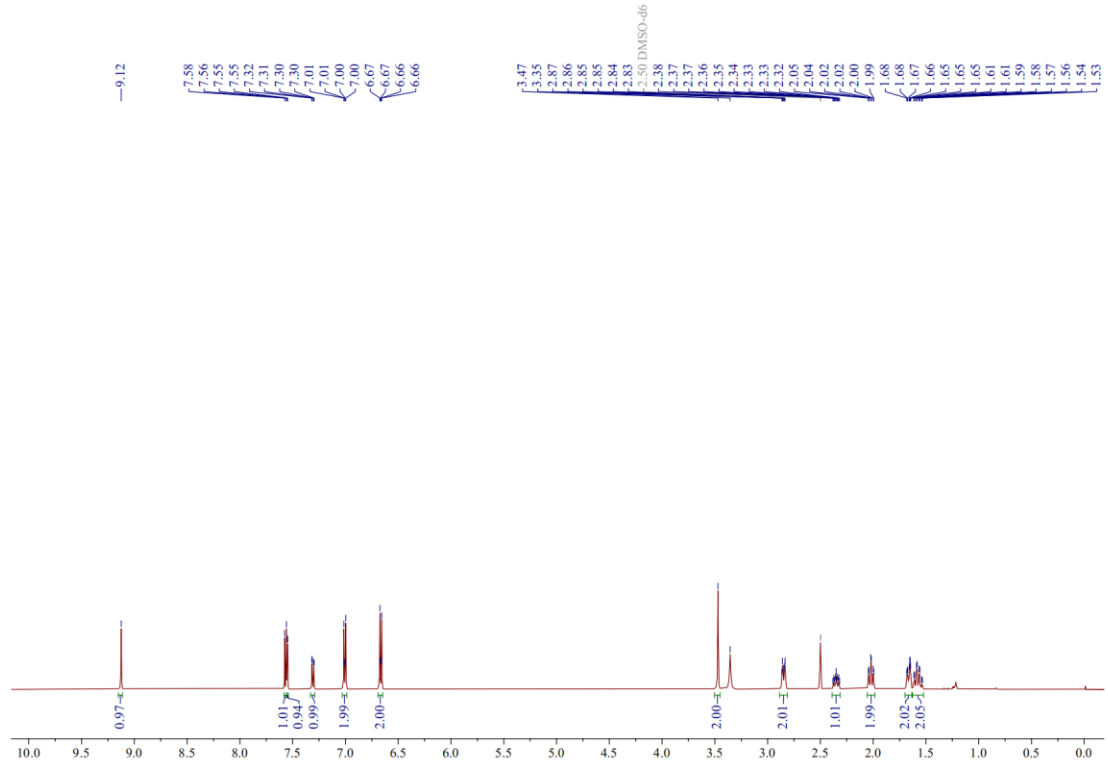

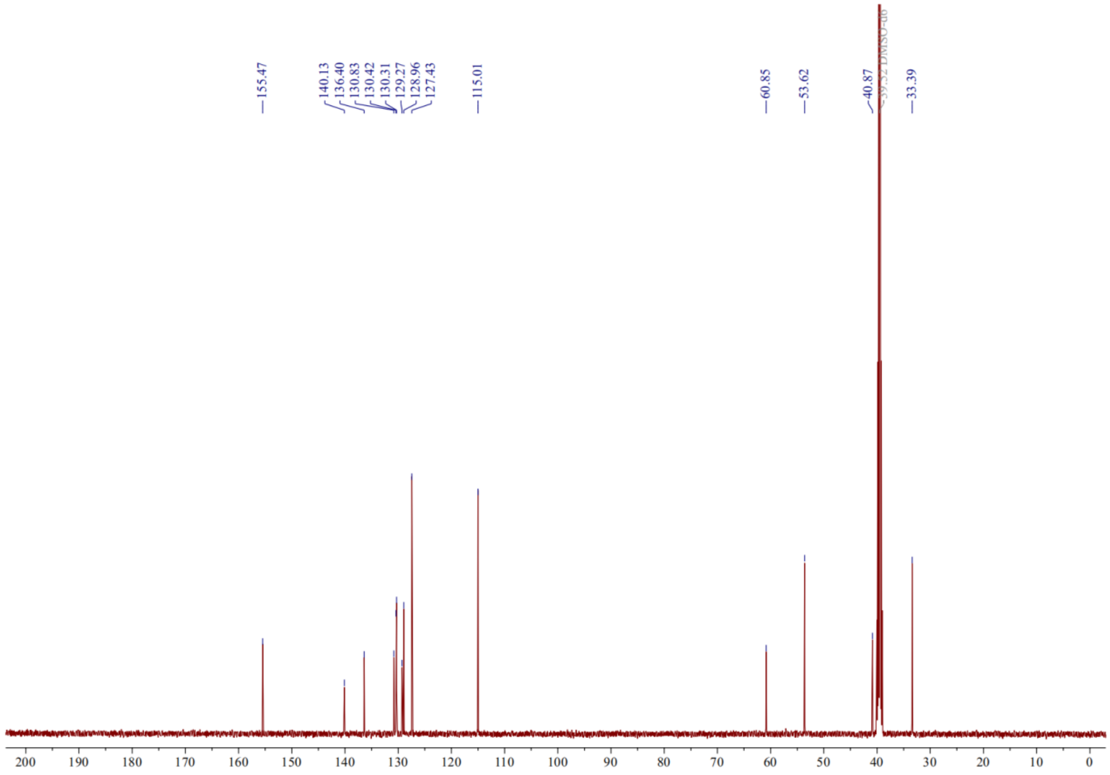

4-(1-(thiophen-2-ylmethyl)piperidin-4-yl)phenol **(AI10-m56)**

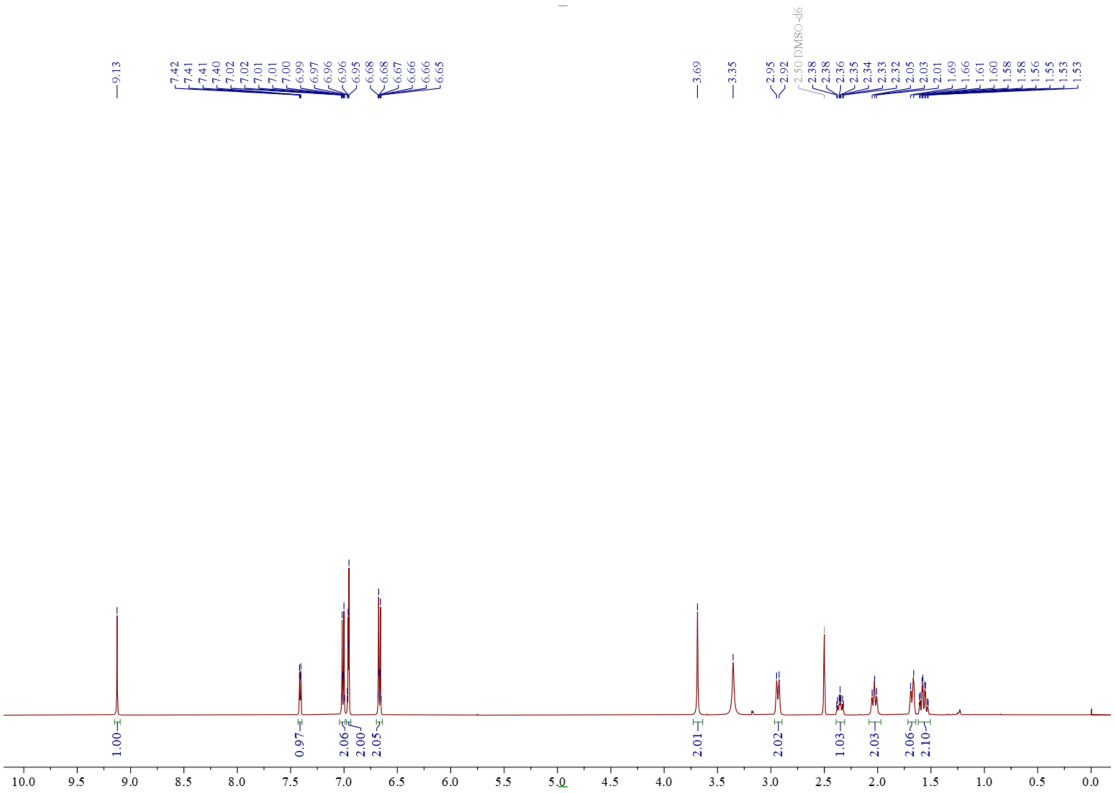

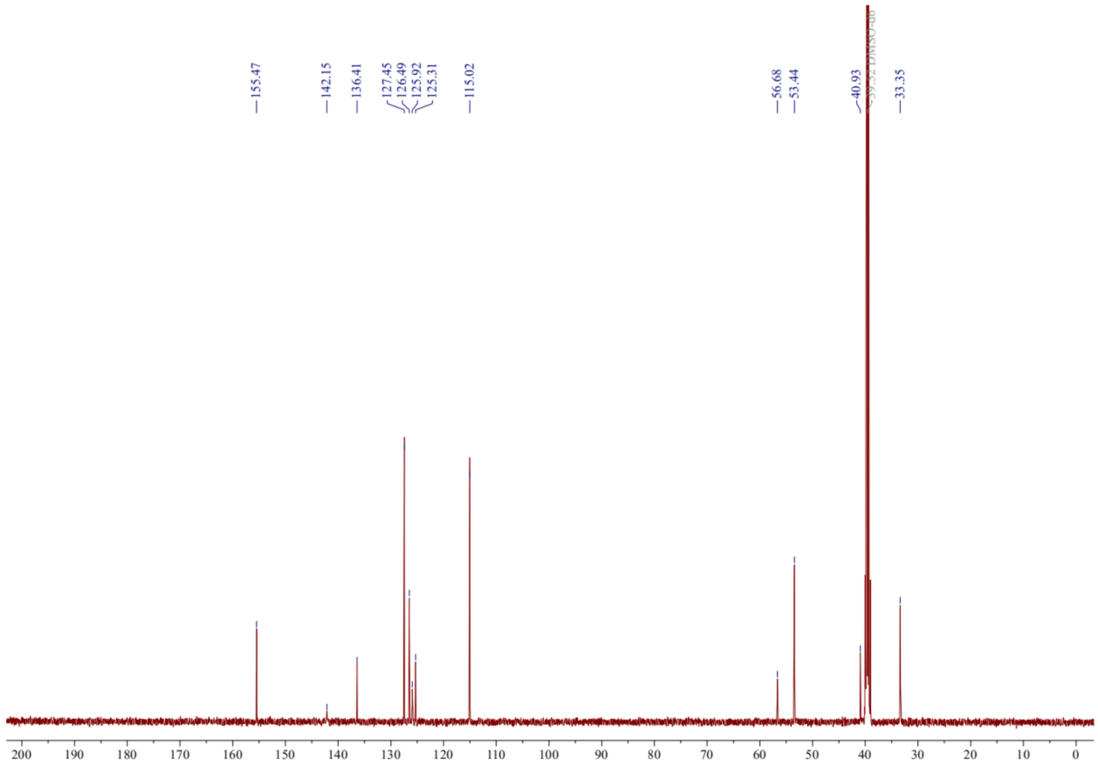

4-((4-(4-hydroxyphenyl)piperidin-1-yl)methyl)benzene-1,3-diol **(AI10-m57)**

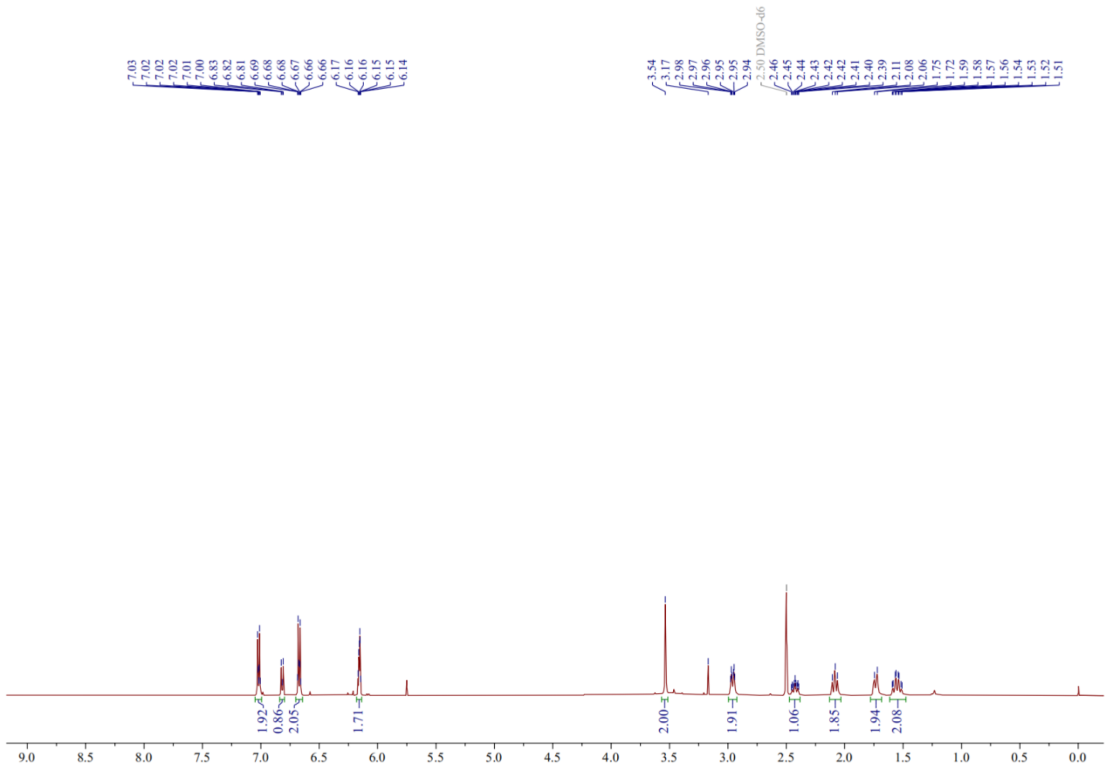

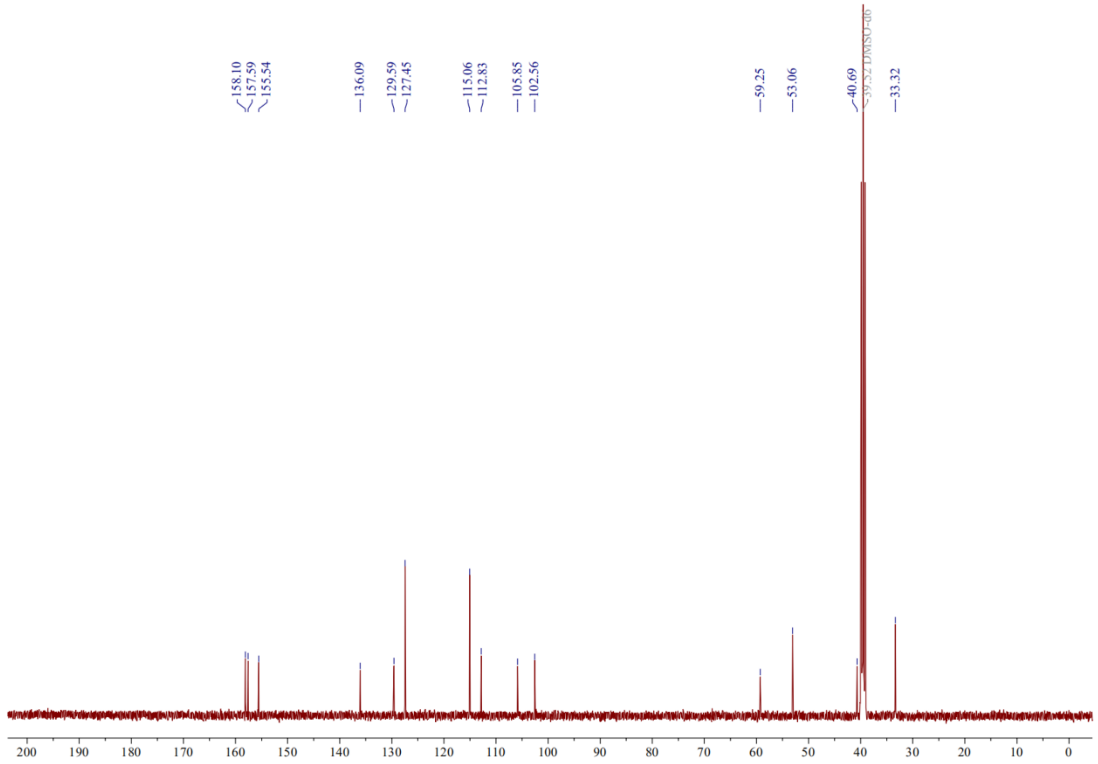

(2,6-dimethylpyridin-4-yl)(4-(4-hydroxybenzyl)piperazin-1-yl)methanone **(AI10-a1)**

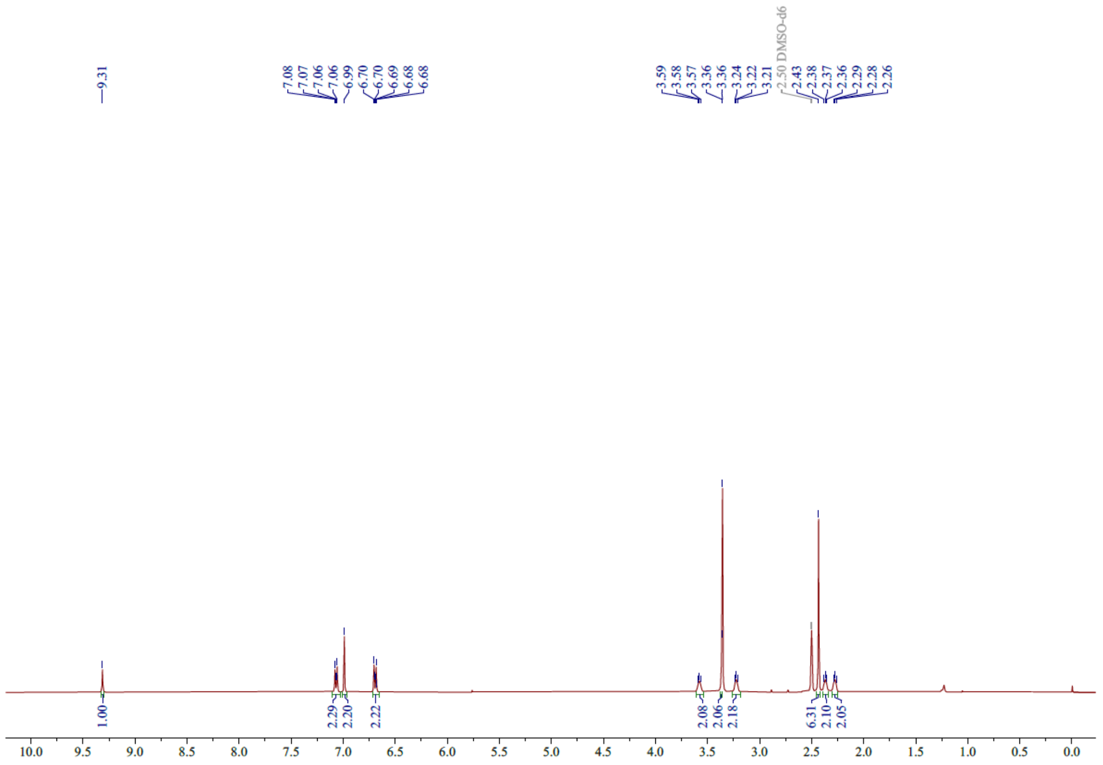

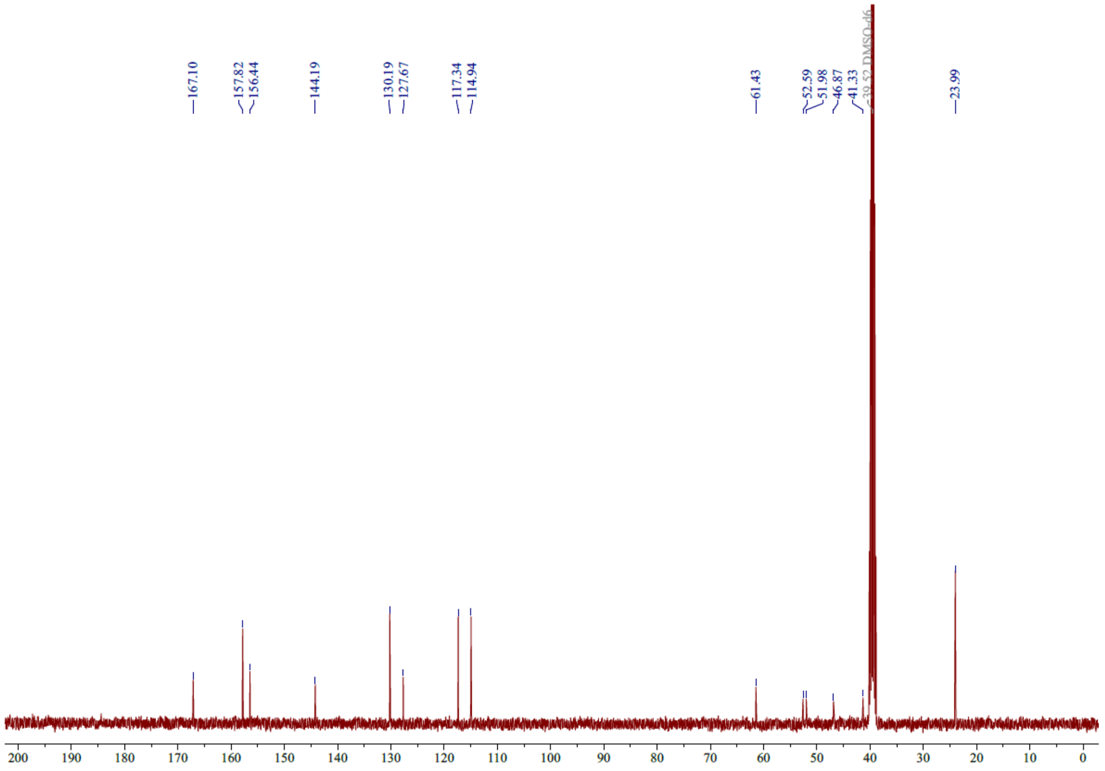

(4-(2,4-dihydroxybenzyl)piperazin-1-yl)(2,6-dimethylpyridin-4-yl)methanone **(AI10-a2)**

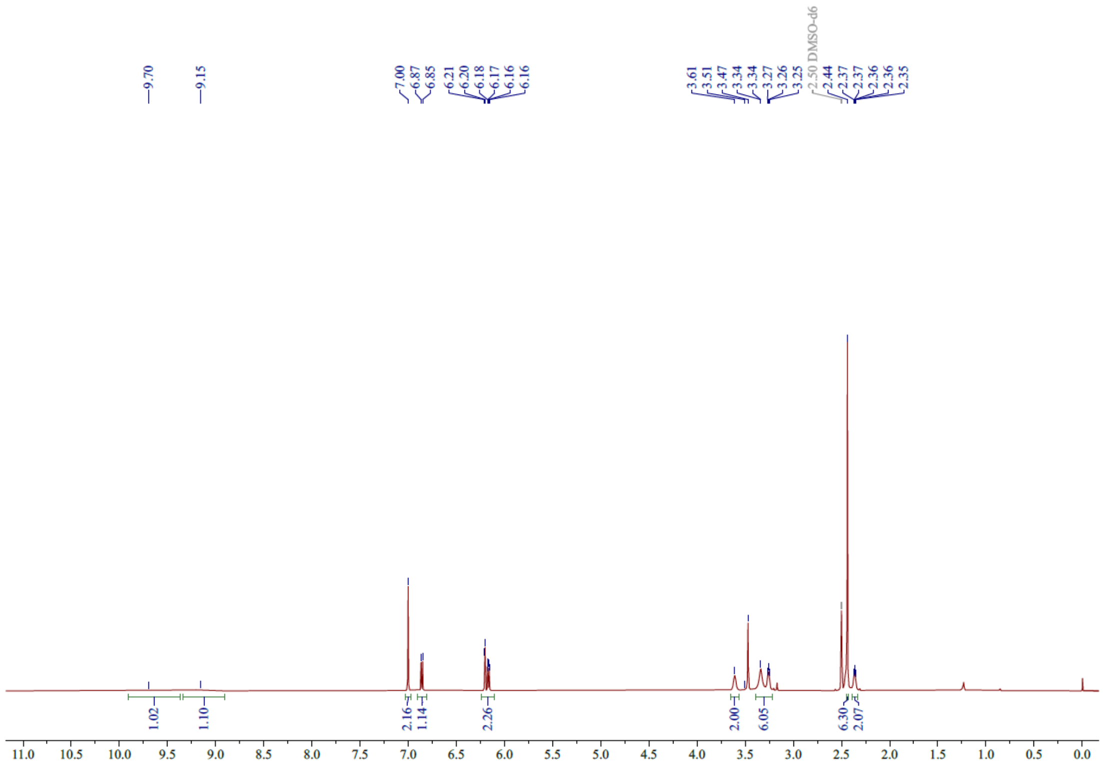

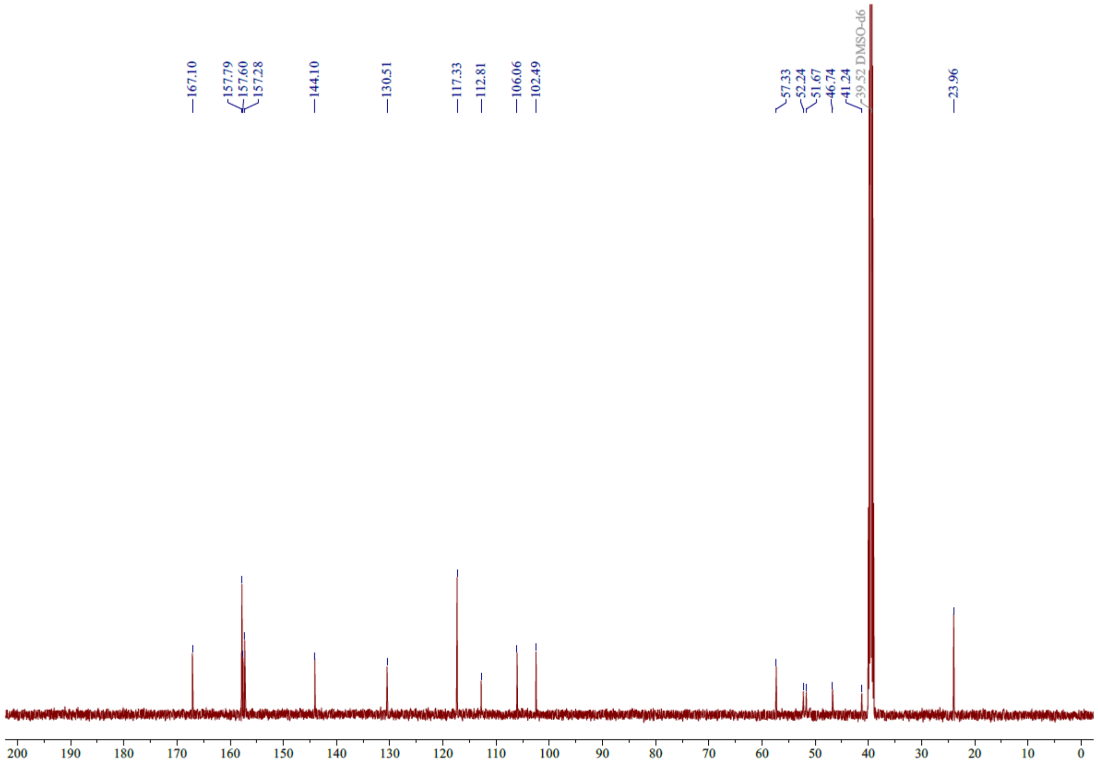

(4-(4-hydroxyphenyl)piperazin-1-yl)(2-methylpyridin-4-yl)methanone **(AI10-a3)**

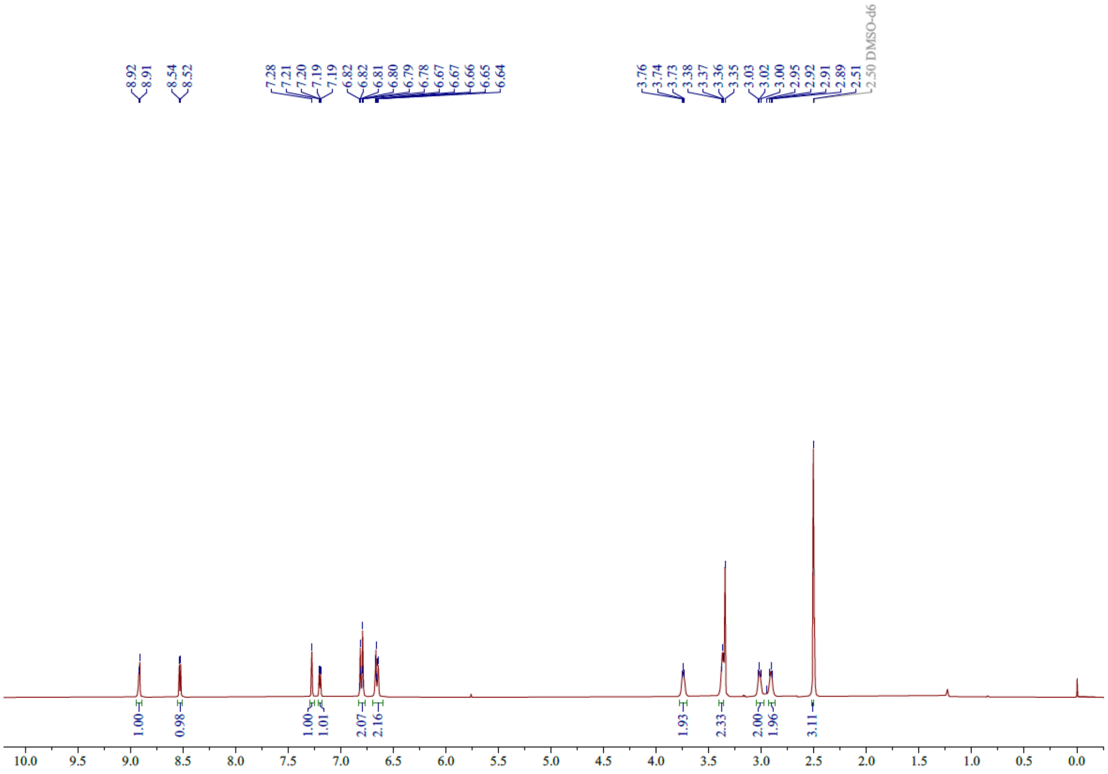

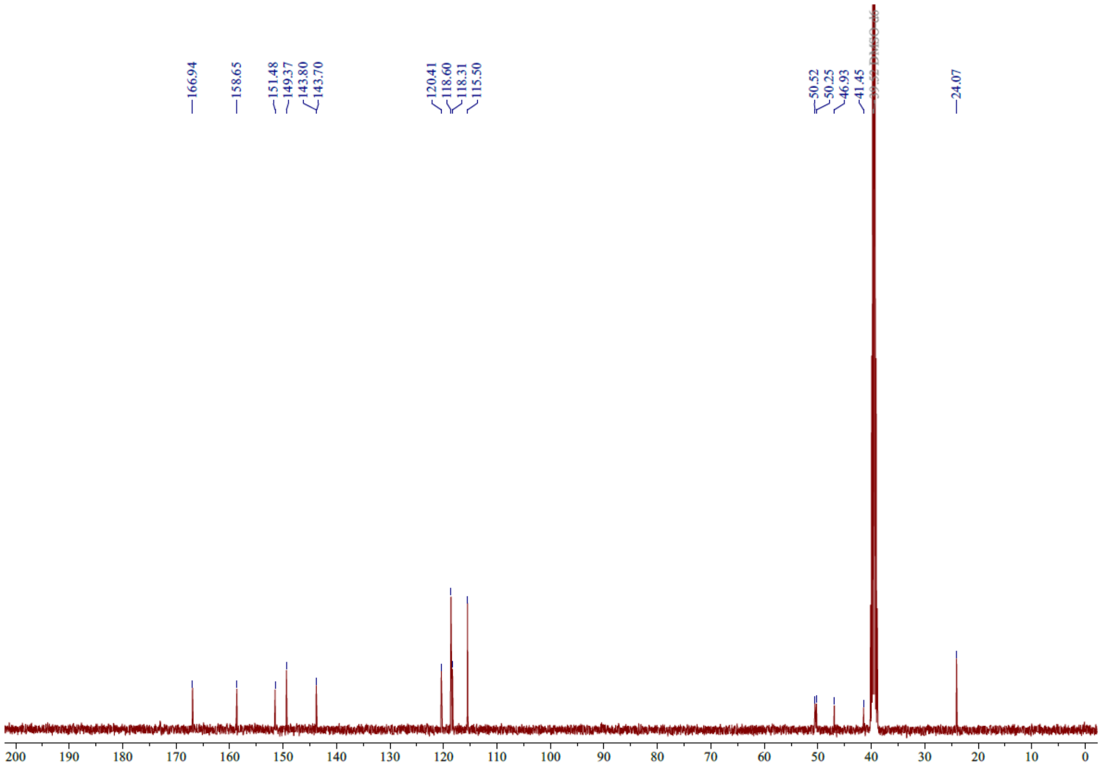

(4-(4-hydroxyphenyl)piperazin-1-yl)(5-methylpyridin-3-yl)methanone **(AI10-a4)**

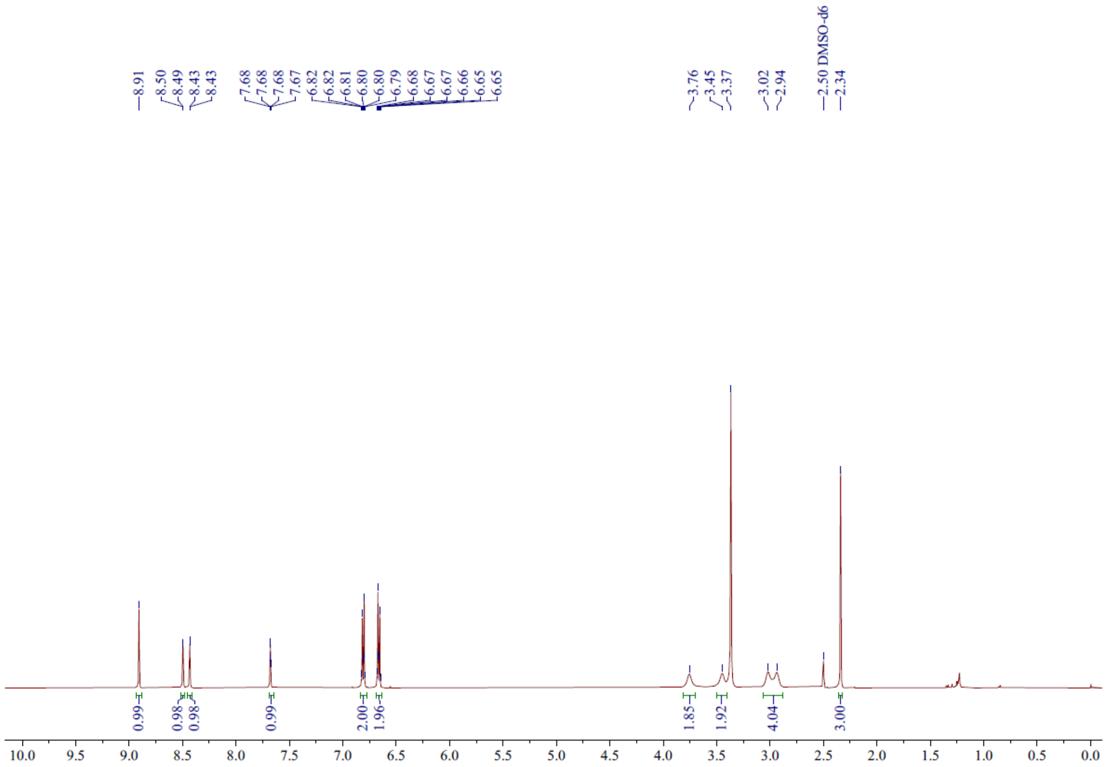

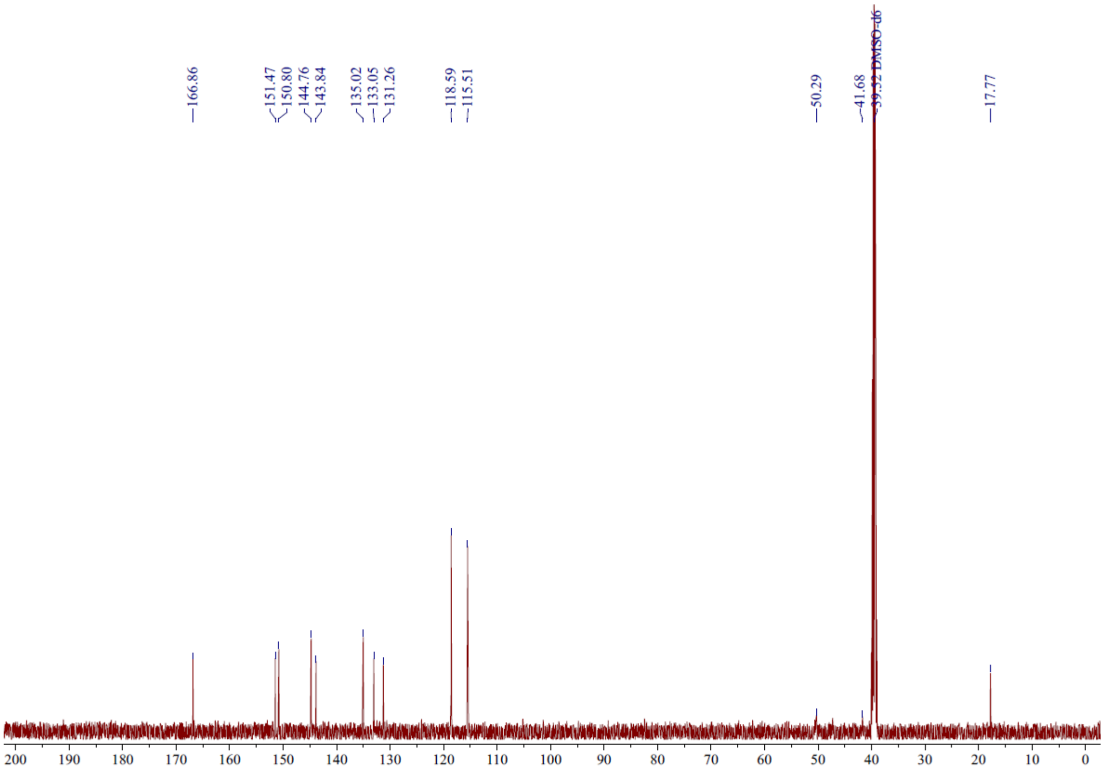

(4-(4-hydroxyphenyl)piperazin-1-yl)(6-methylpyridin-2-yl)methanone **(AI10-a5)**

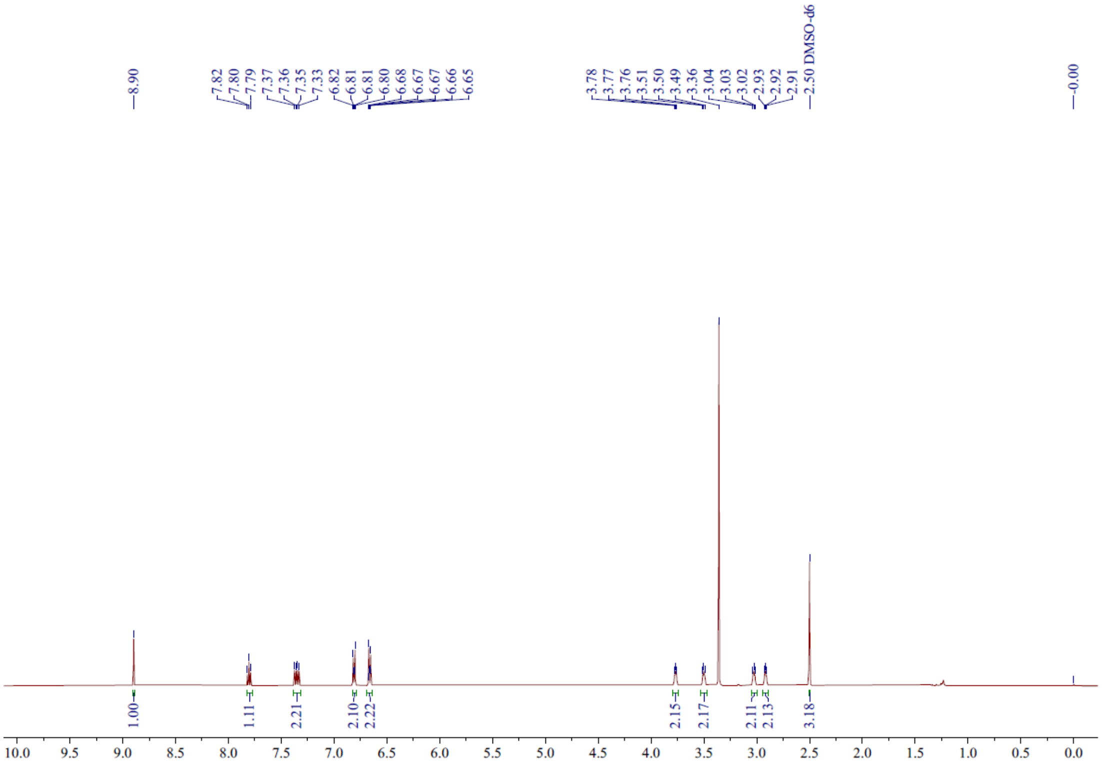

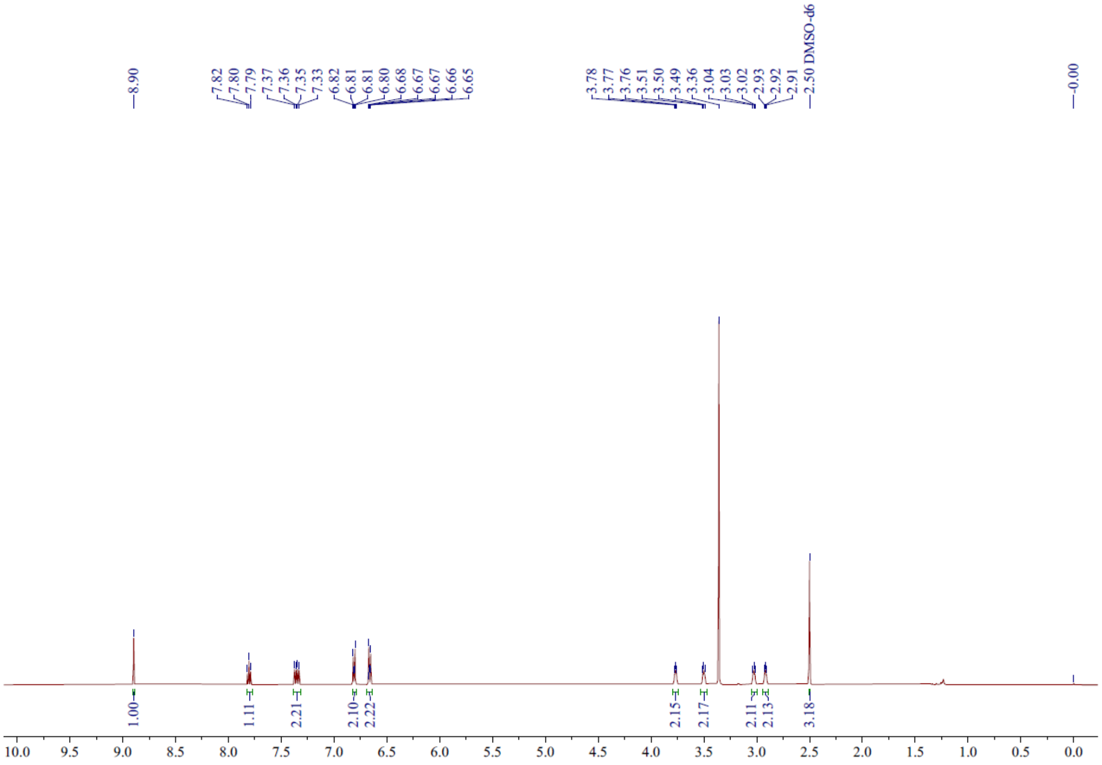

(4-(4-hydroxyphenyl)piperazin-1-yl)(2-methylpyridin-3-yl)methanone **(AI10-a6)**

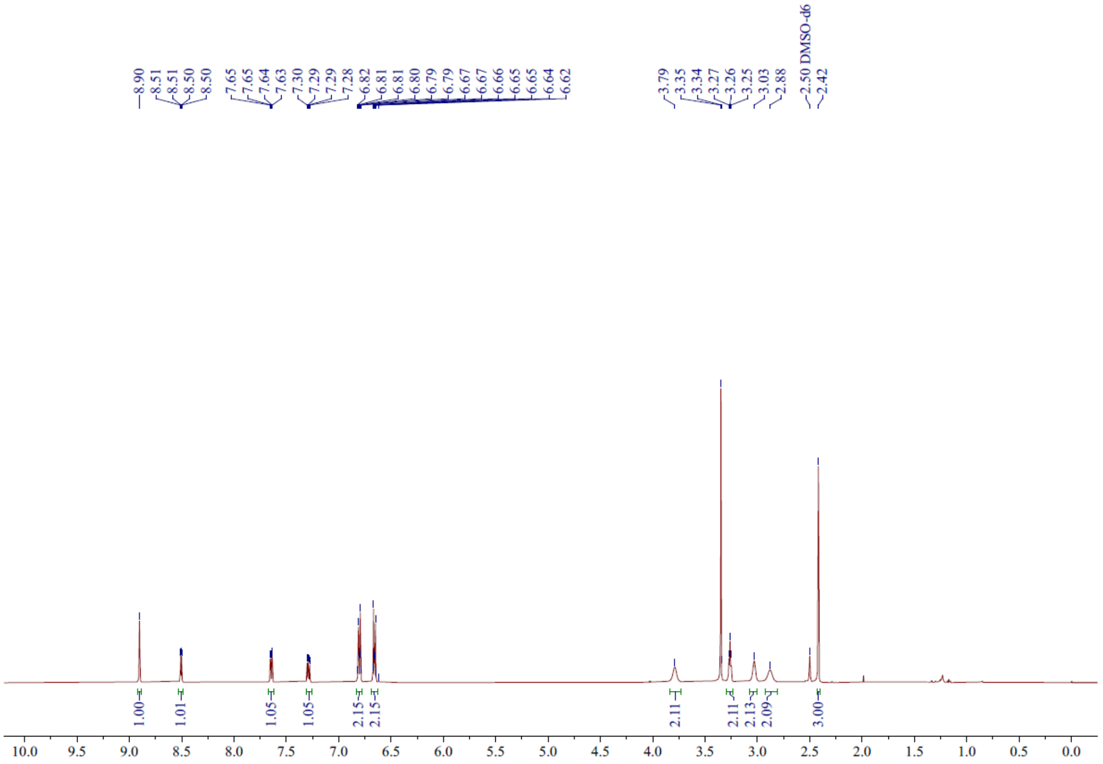

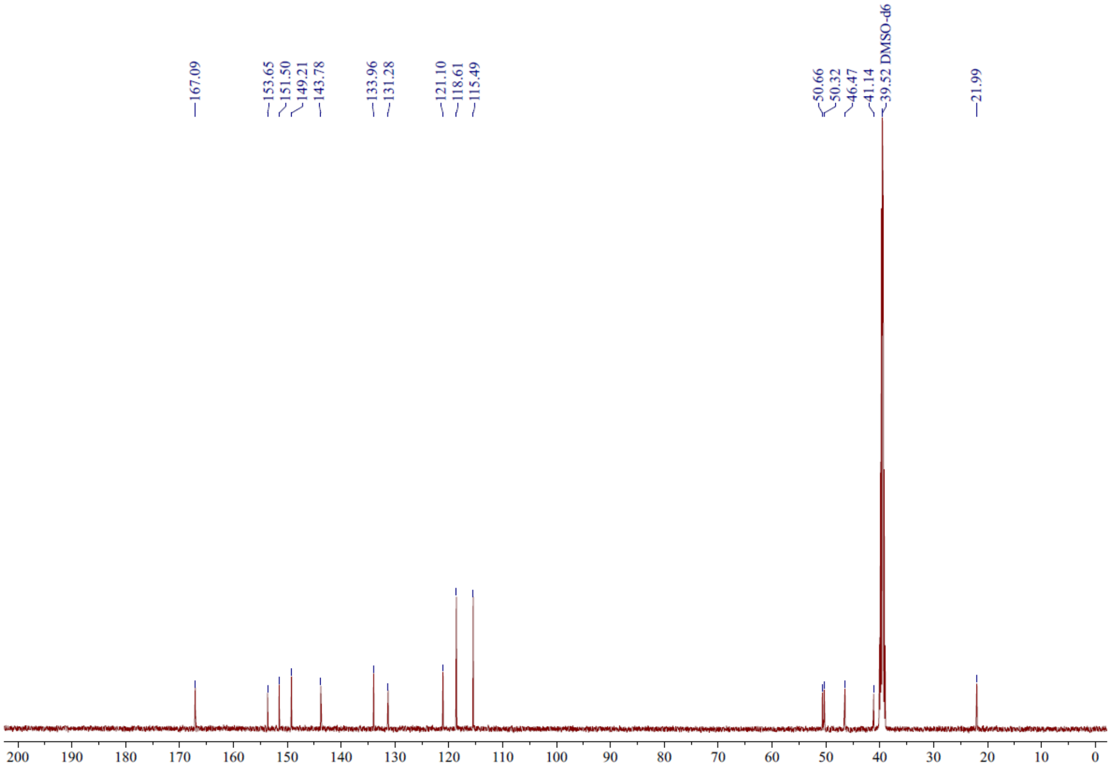

(4-hydroxy-2-methylphenyl)(4-(4-hydroxyphenyl)piperazin-1-yl)methanone **(AI10-a7)**

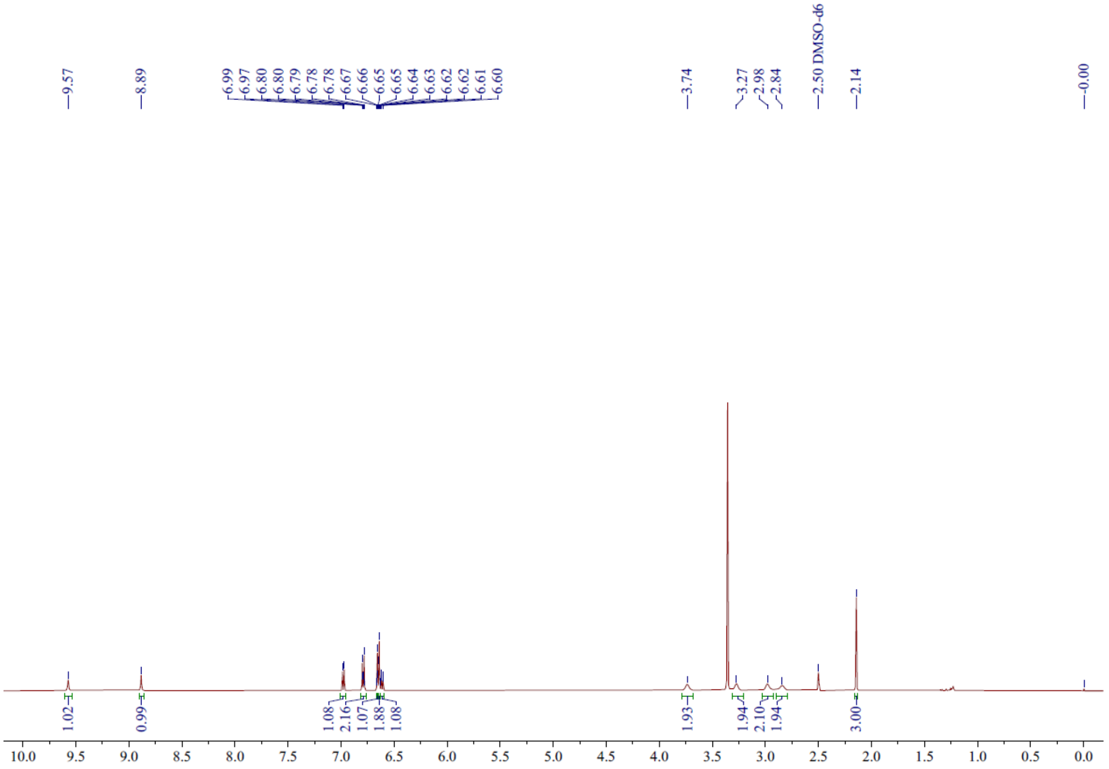

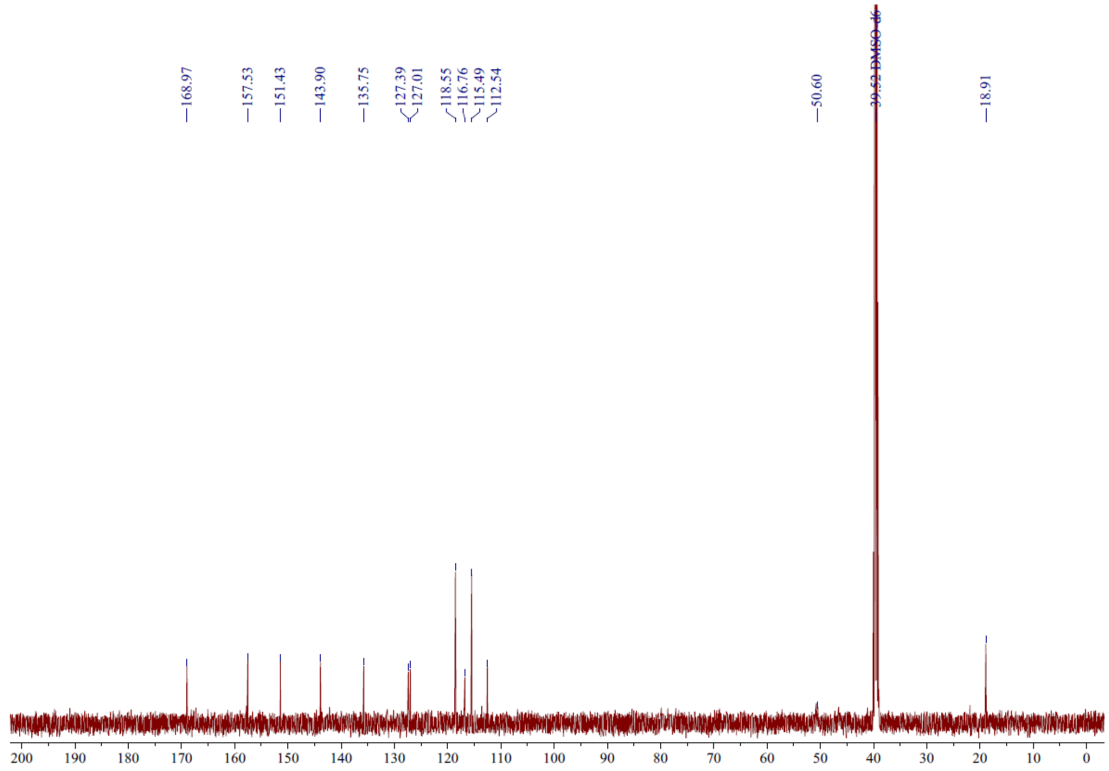

(2-hydroxy-3-methylphenyl)(4-(4-hydroxyphenyl)piperazin-1-yl)methanone **(AI10-a8)**

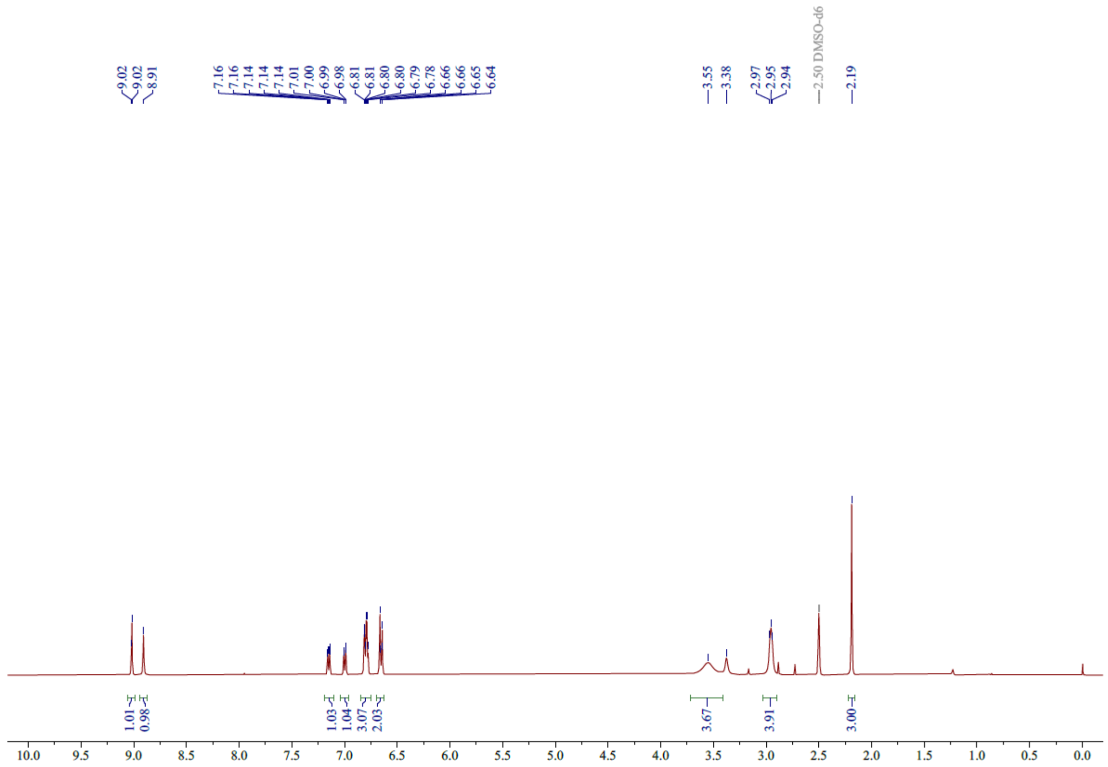

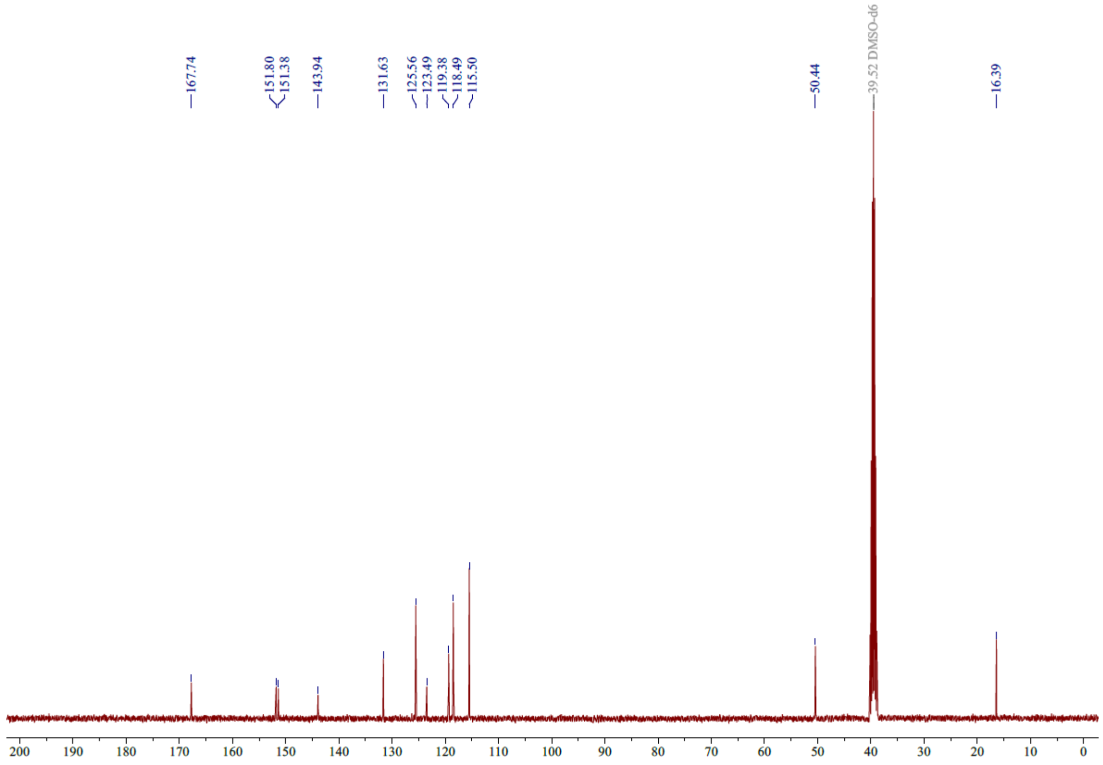

1-(4-((4-(4-hydroxyphenyl)piperazin-1-yl)methyl)phenyl)ethan-1-one **(AI10-a9)**

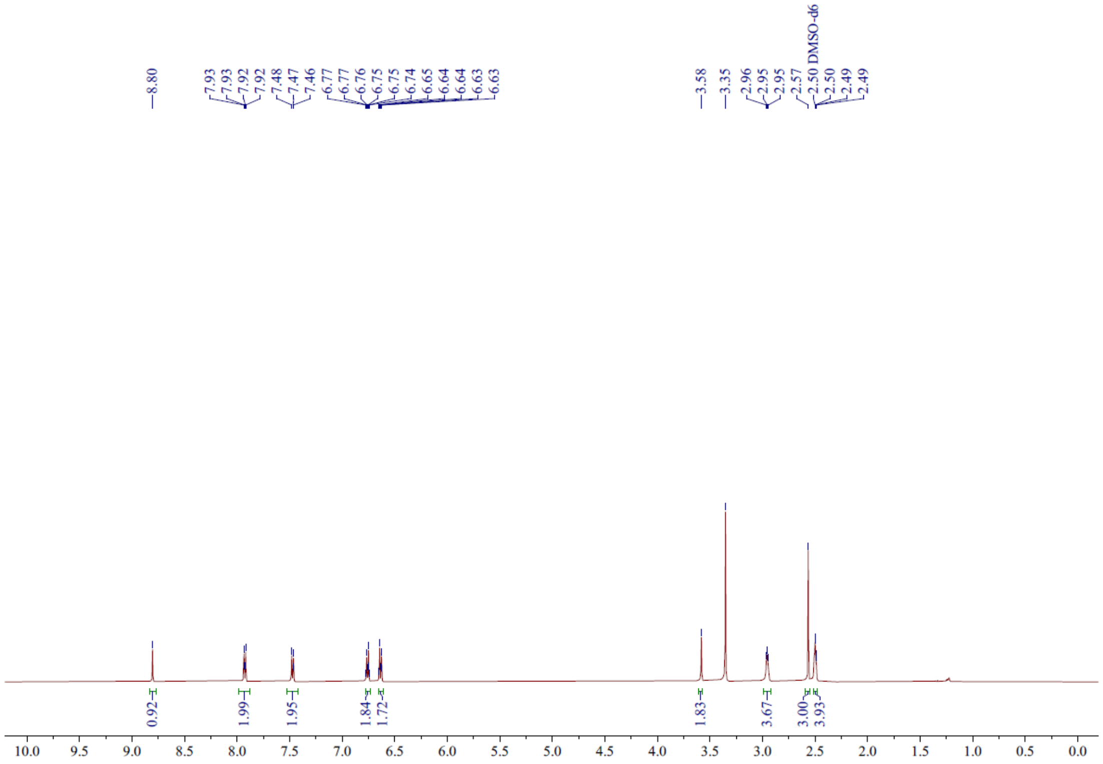

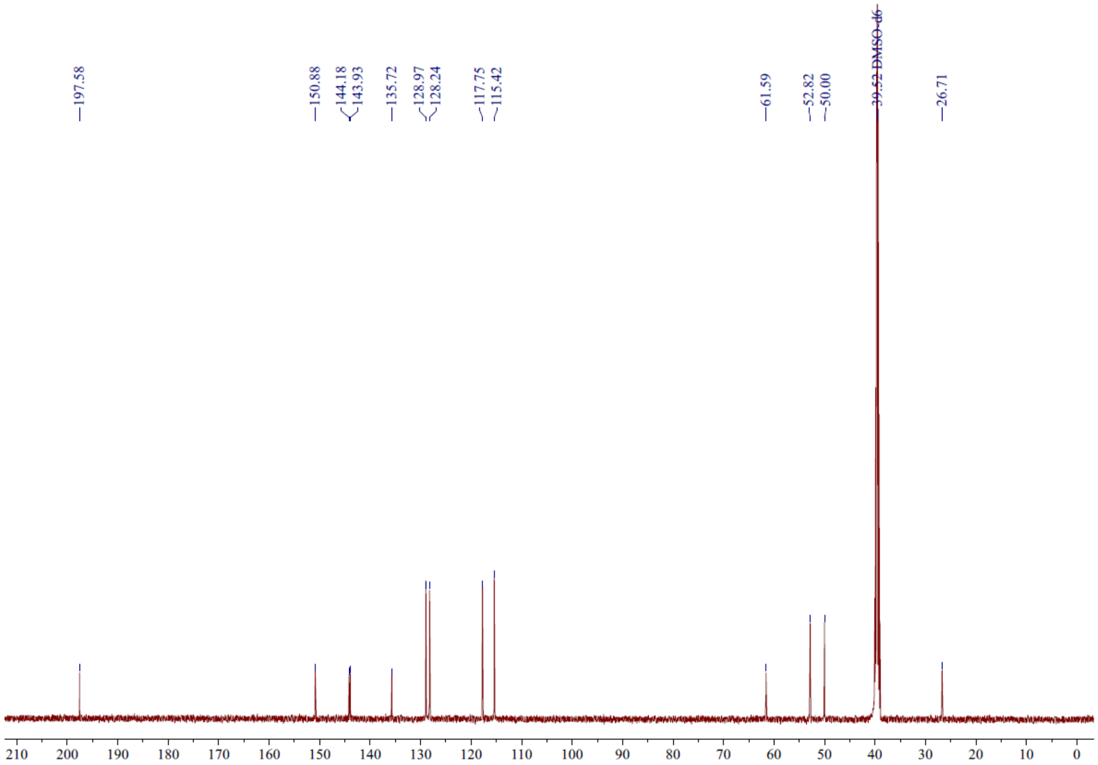

4-((4-(4-hydroxyphenyl)piperazin-1-yl)methyl)-2,6-dimethylphenol **(AI10-a10)**

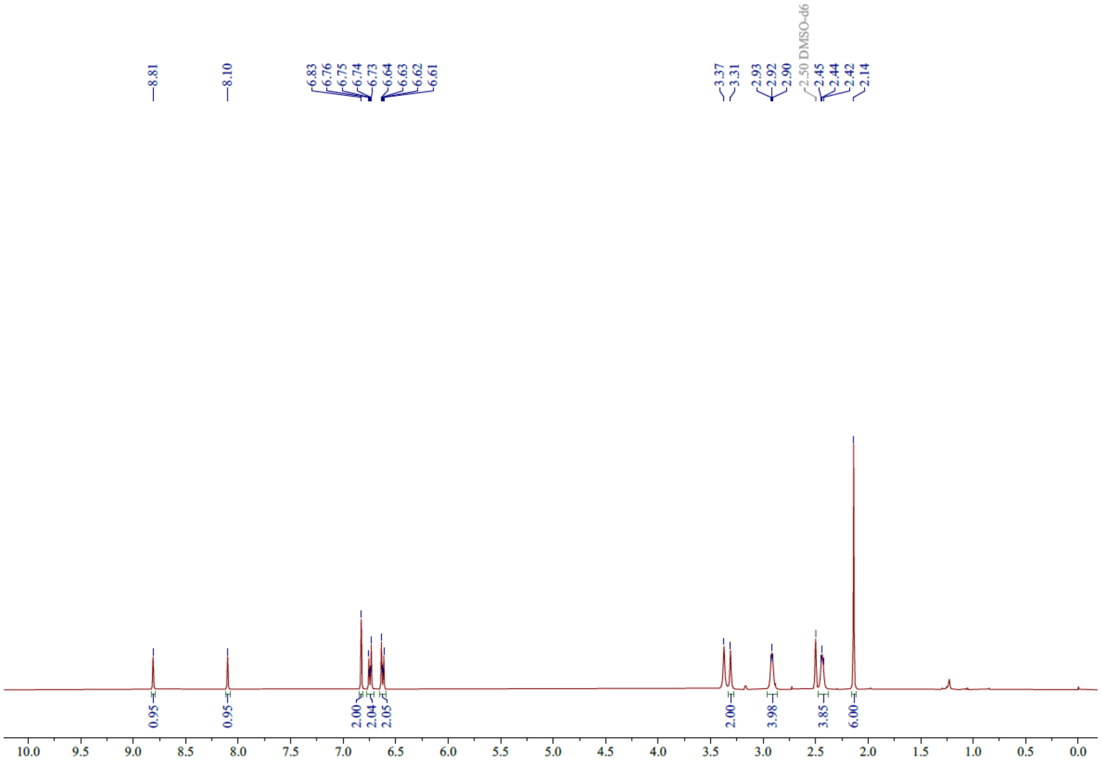

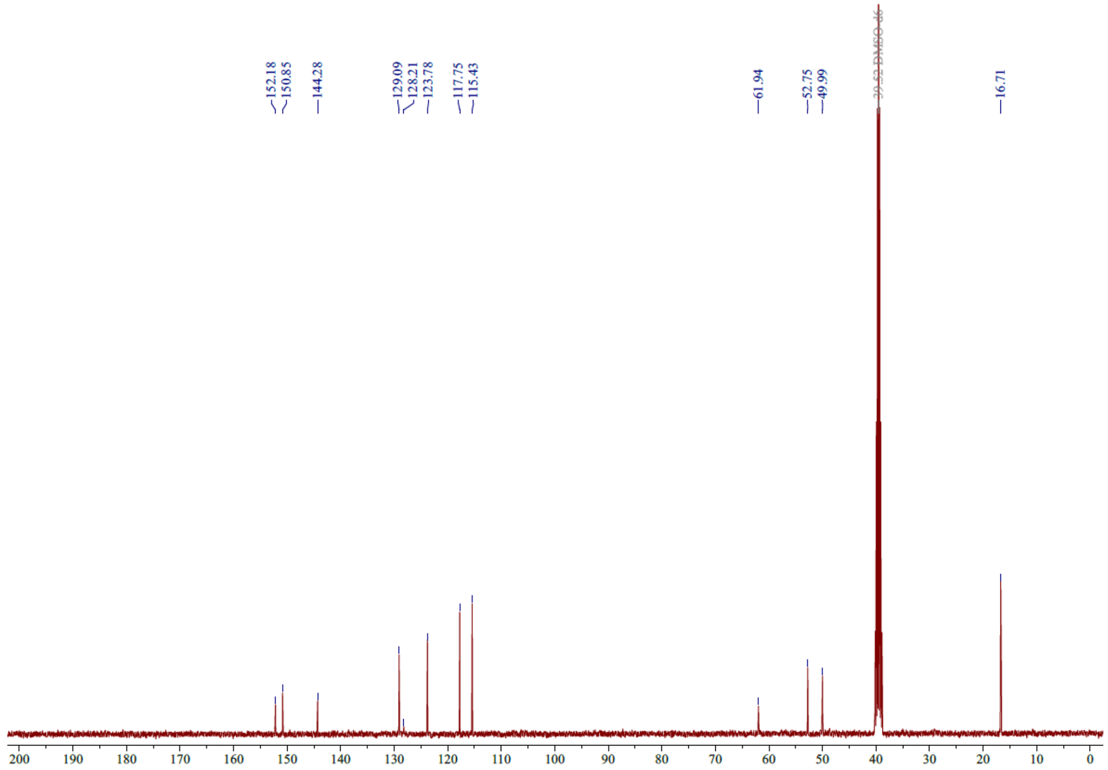

***(4-(4-hydroxyphenyl)piperazin-1-yl)(1-methyl-1H-indazol-6-yl)methanone* (AI10-a11)**

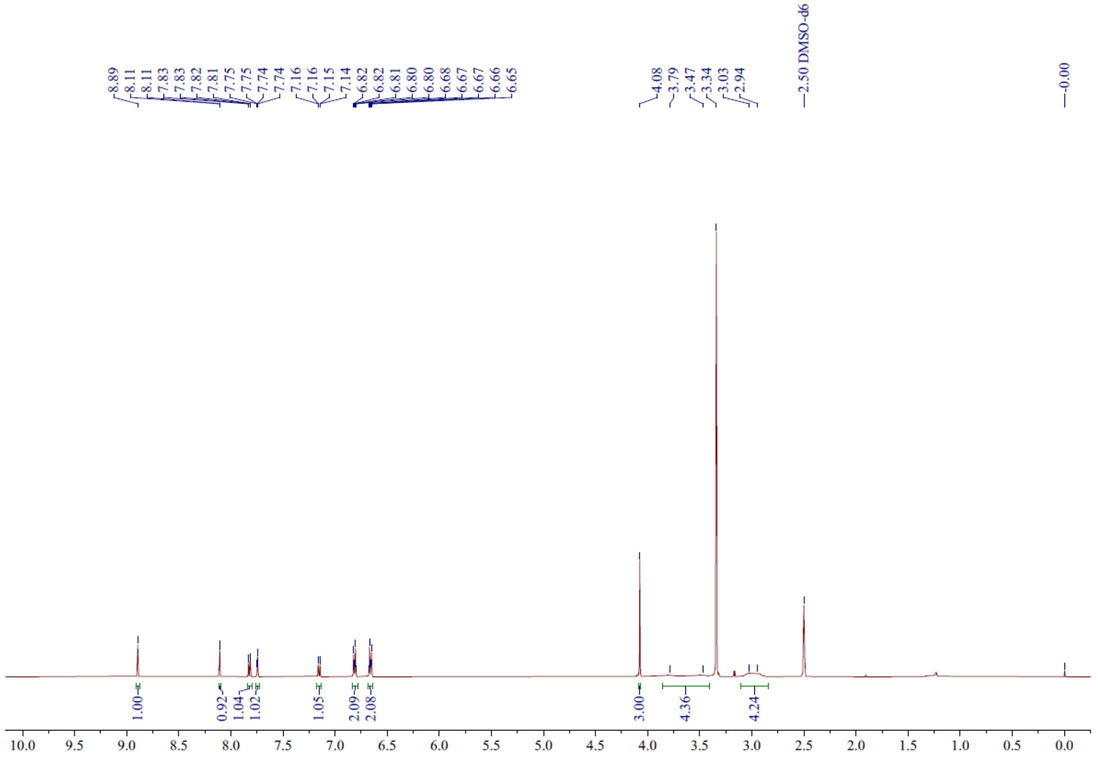

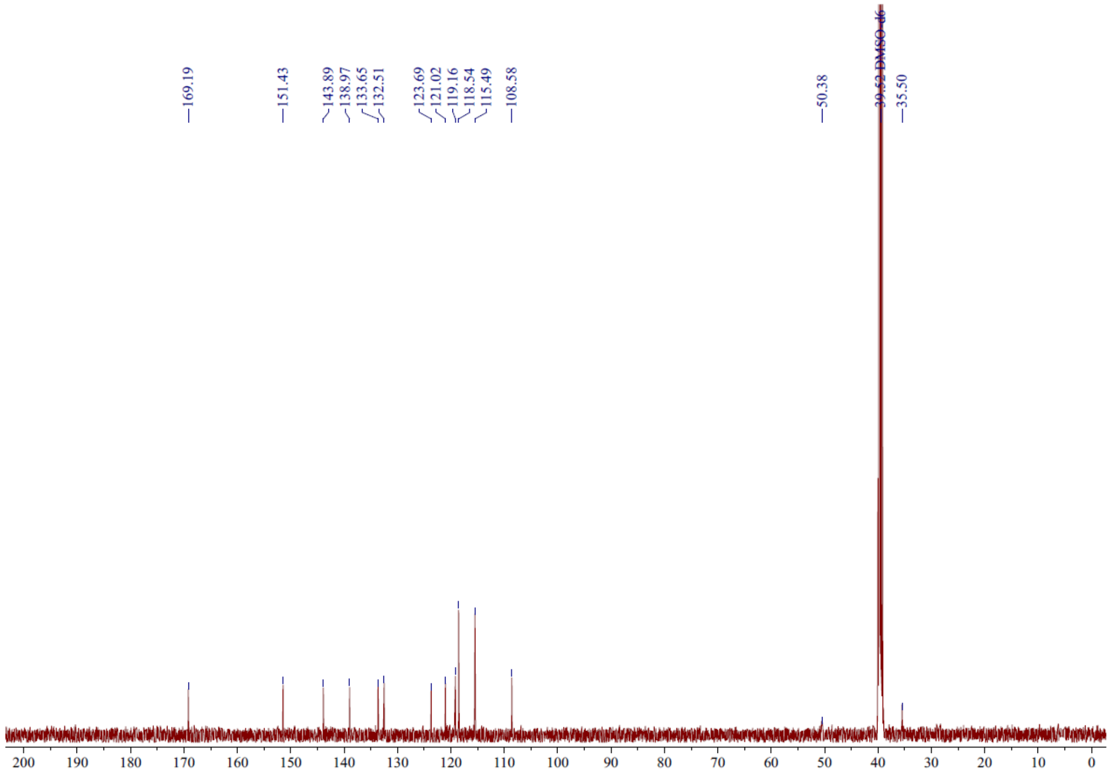

***(4-(4-hydroxyphenyl)piperazin-1-yl)(4-methylthiazol-2-yl)methanone* (AI10-a12)**

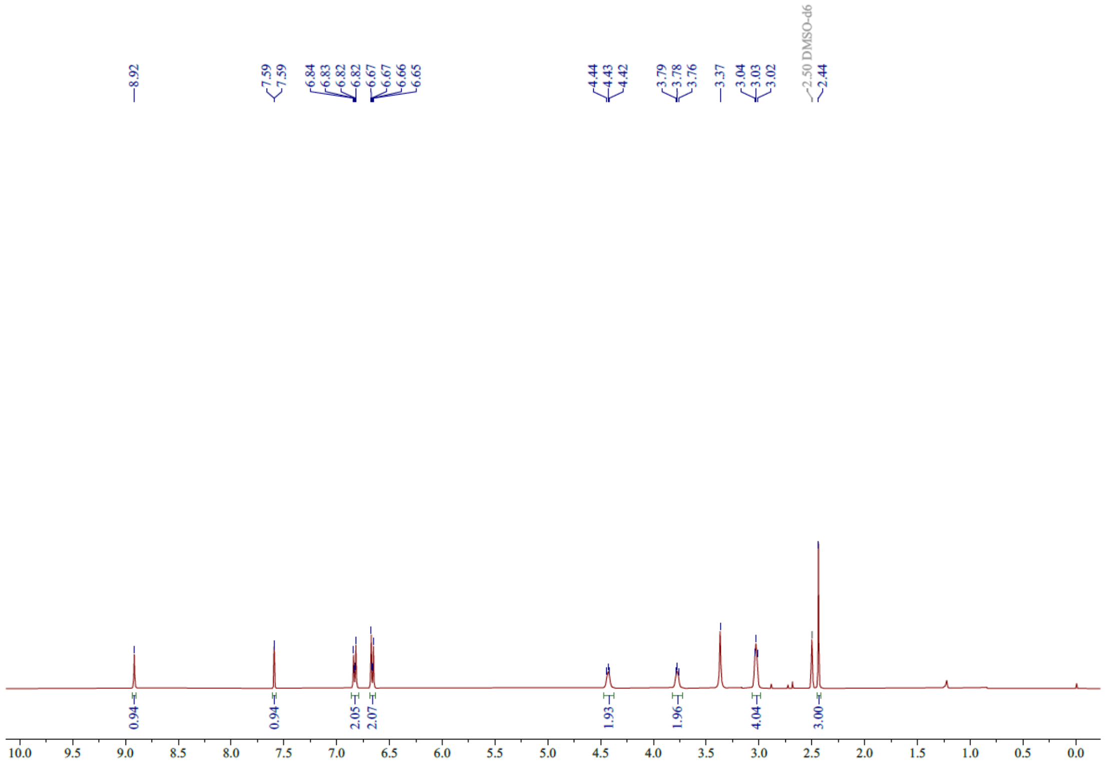

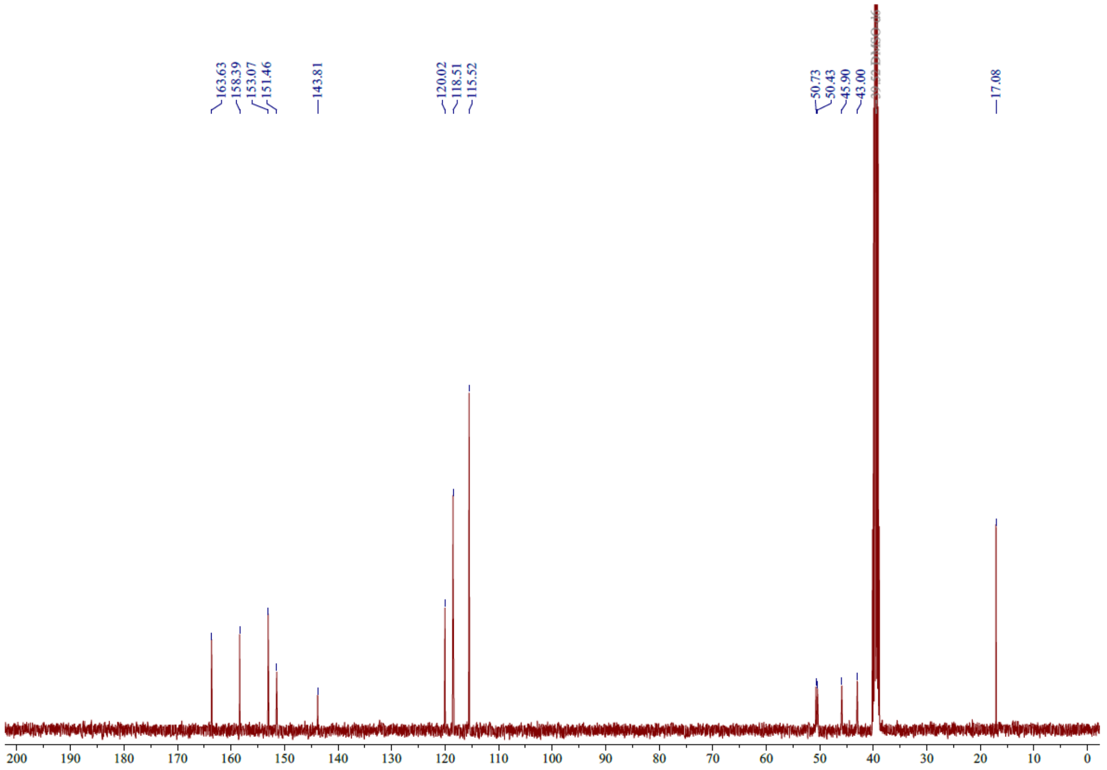

(4-(4-hydroxyphenyl)piperazin-1-yl)(1-methyl-1H-indol-2-yl)methanone (AI10-a13)

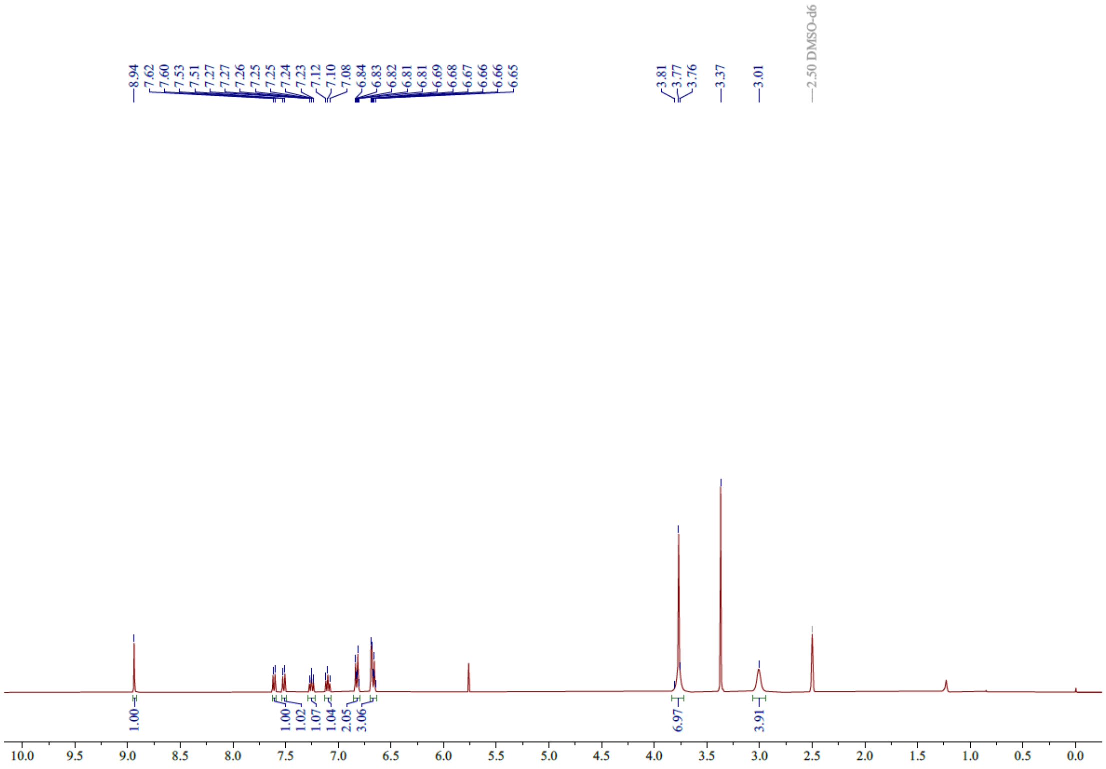

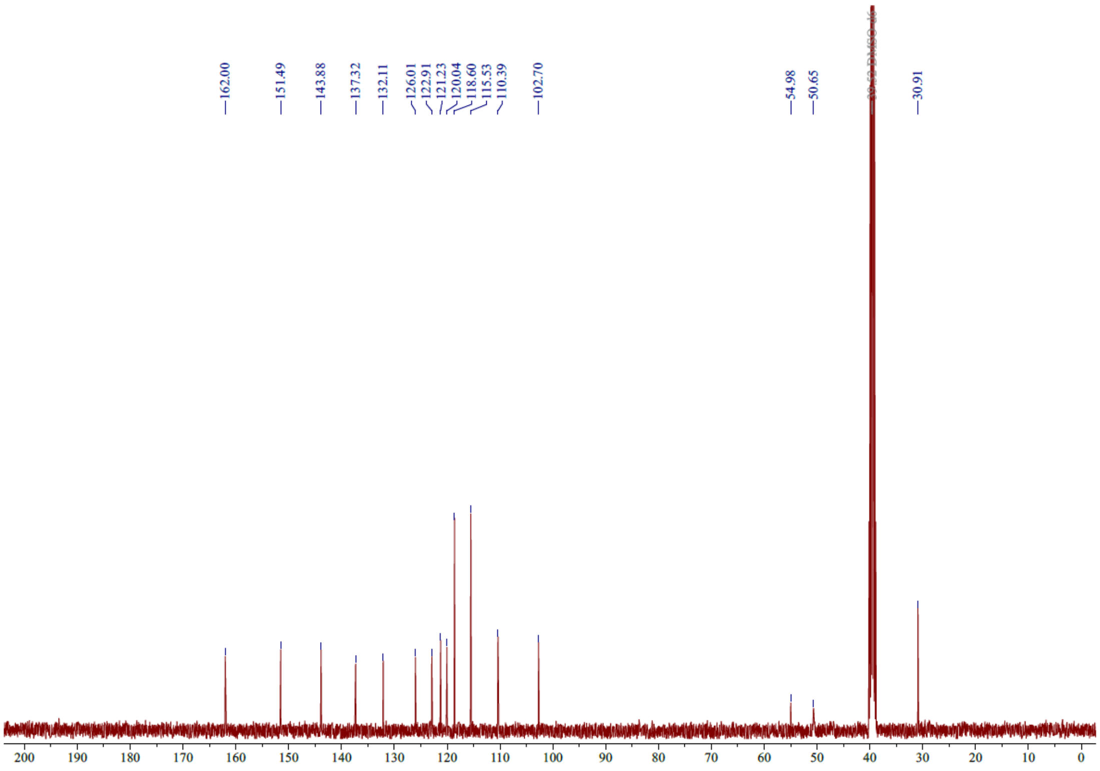

4-((4-(4-hydroxyphenyl)piperazin-1-yl)methyl)-3,5-dimethylphenol **(AI10-a14)**

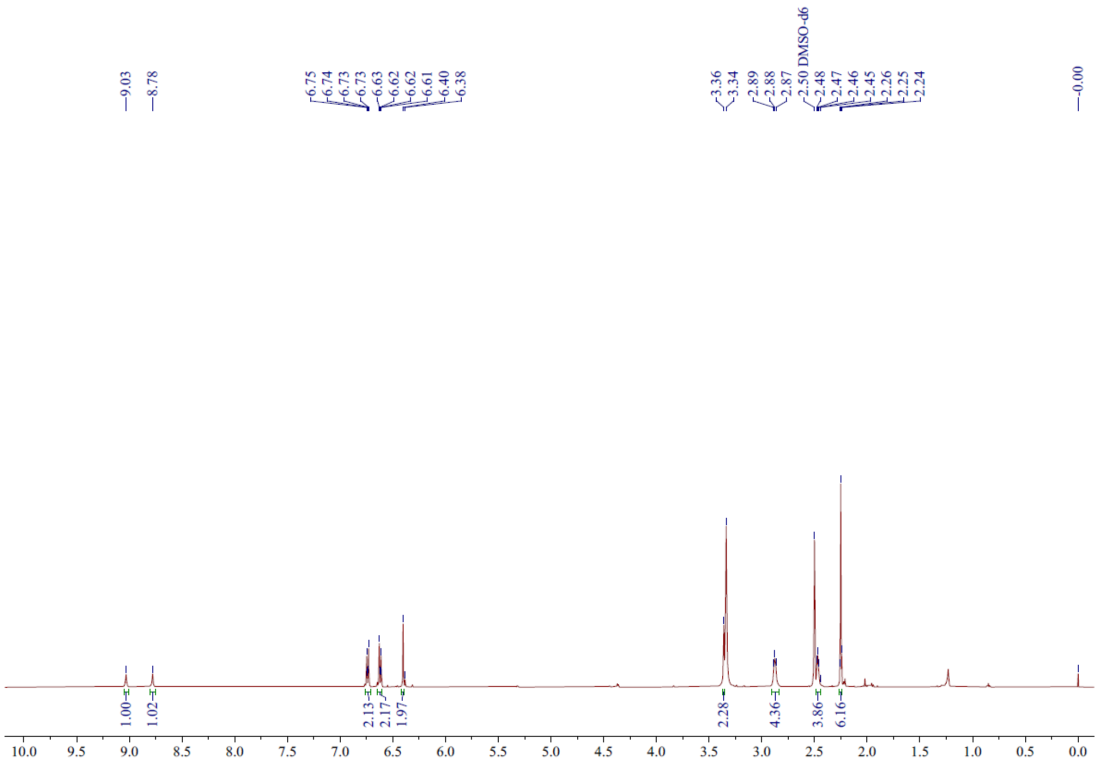

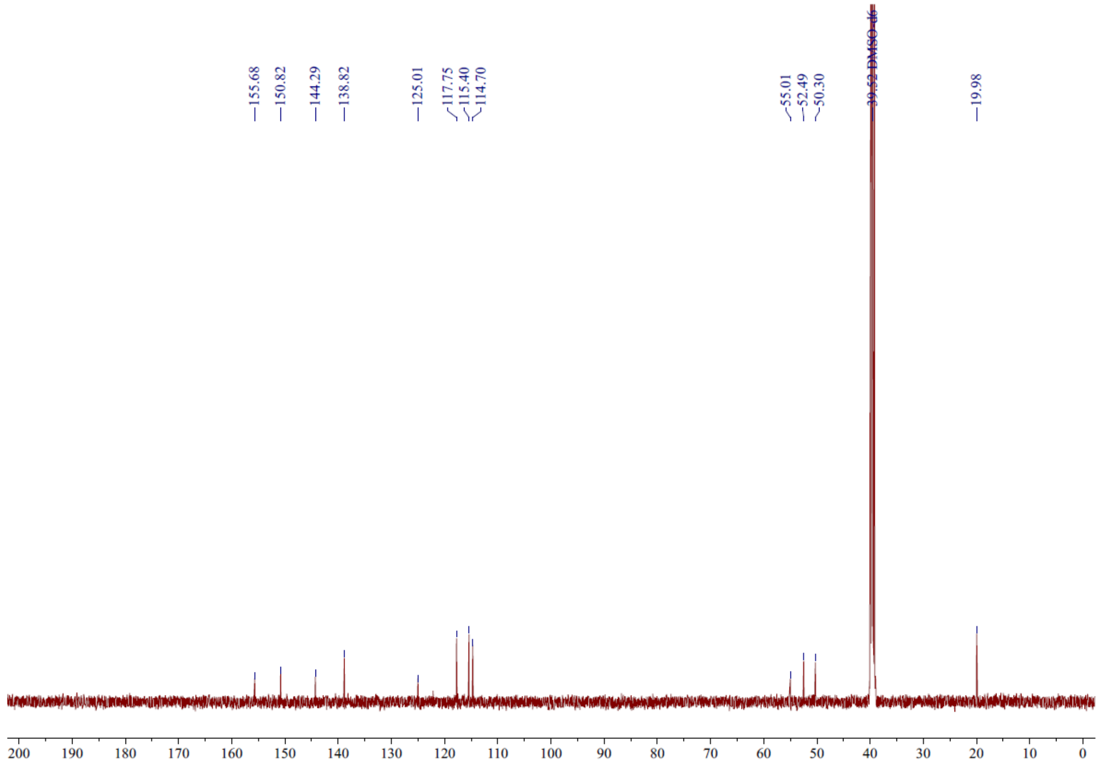

***4-((4-(4-hydroxyphenyl)piperazin-1-yl)methyl)-3-methylphenol* (AI10-a15)**

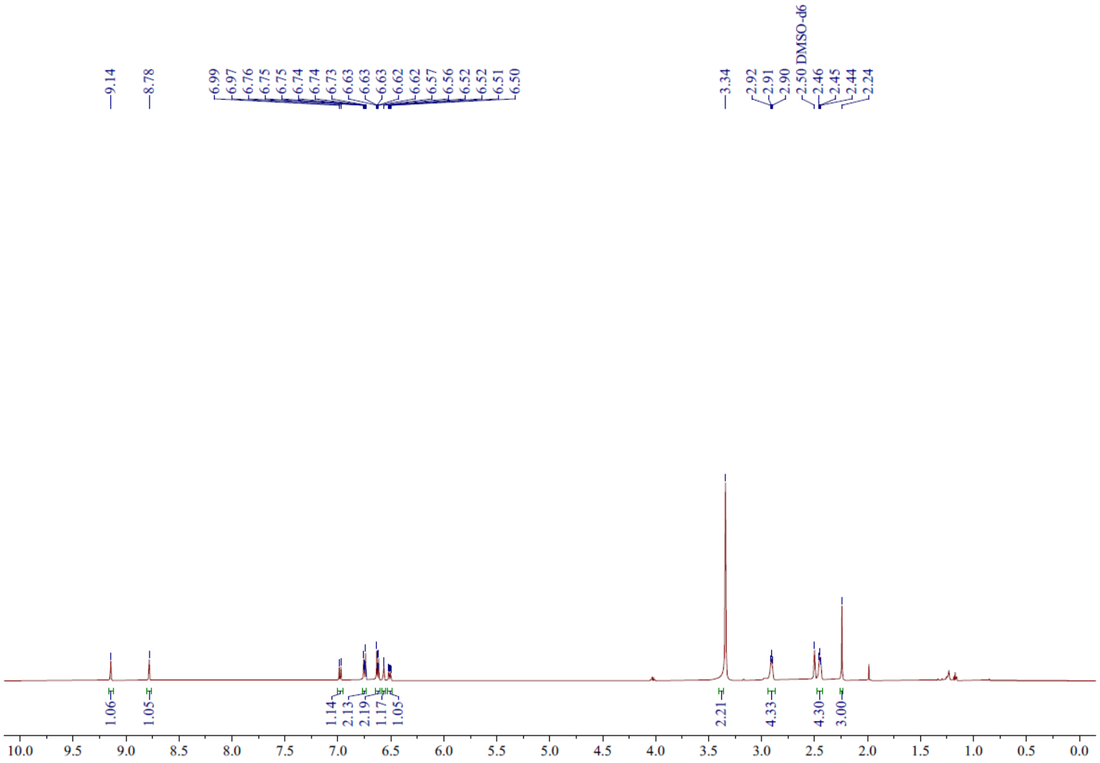

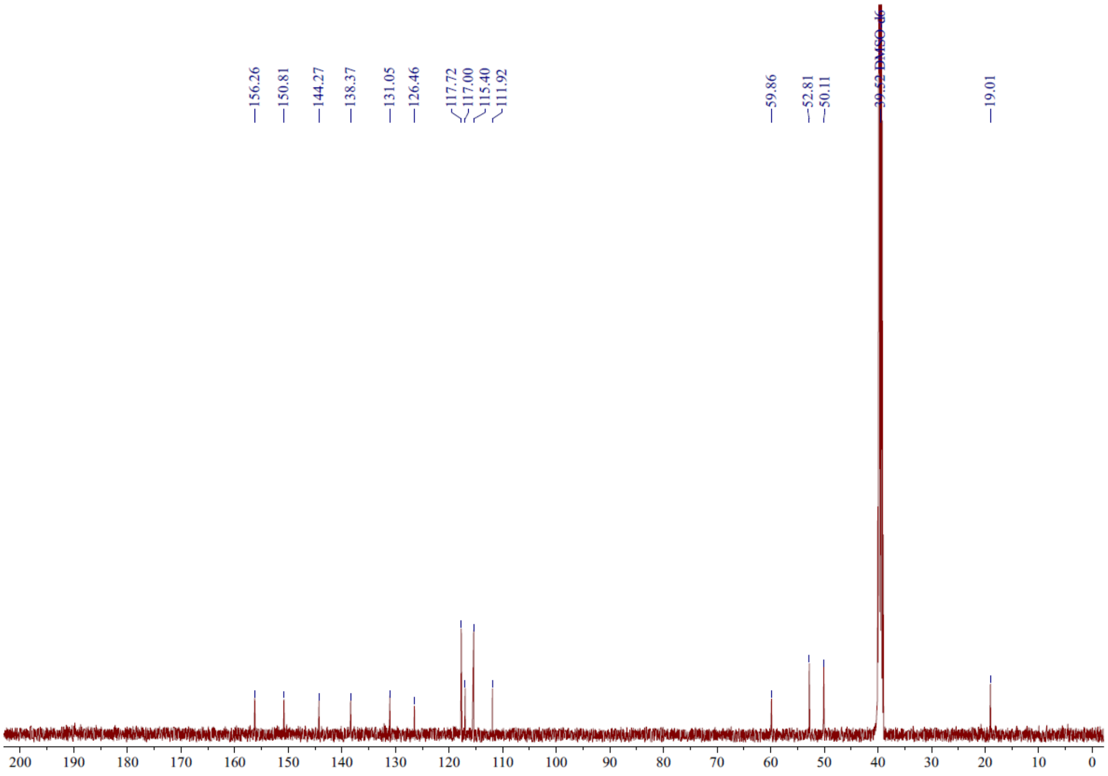

4-((4-(4-hydroxyphenyl)piperazin-1-yl)methyl)-2-methylphenol **(AI10-a16)**

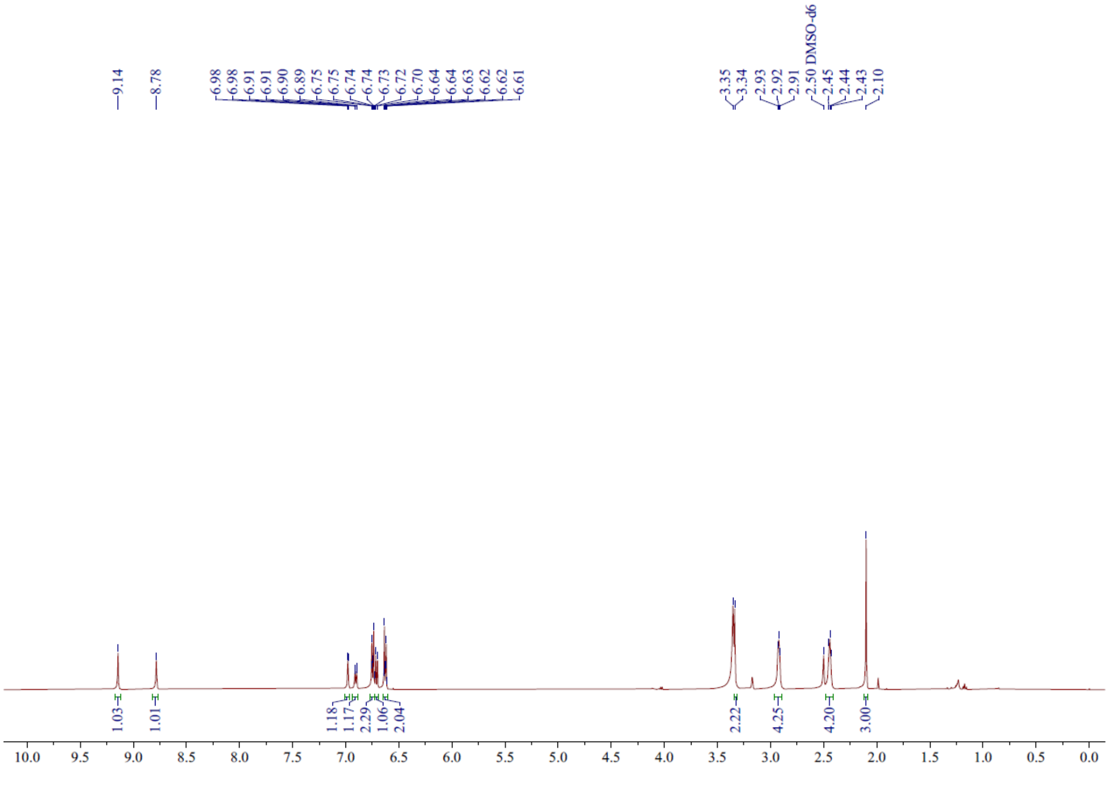

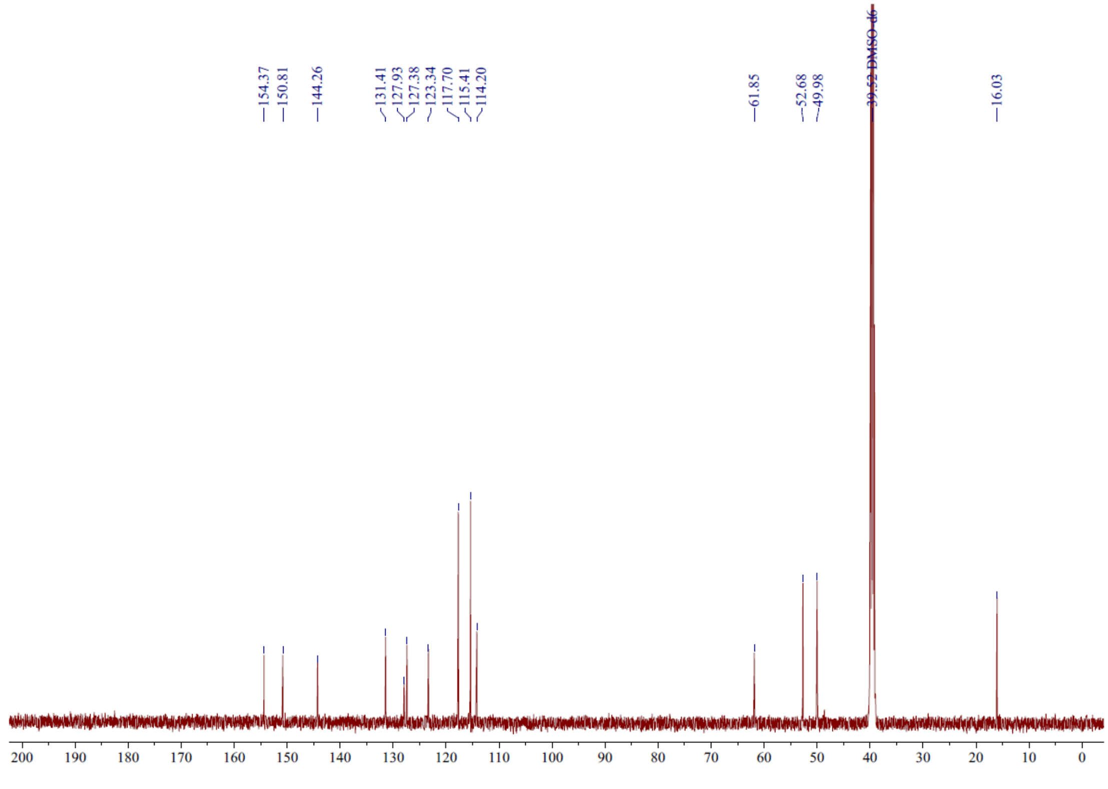

(2-chloro-6-methylpyridin-3-yl)(4-(3-hydroxyphenyl)piperazin-1-yl)methanone **(AI10-a17)**

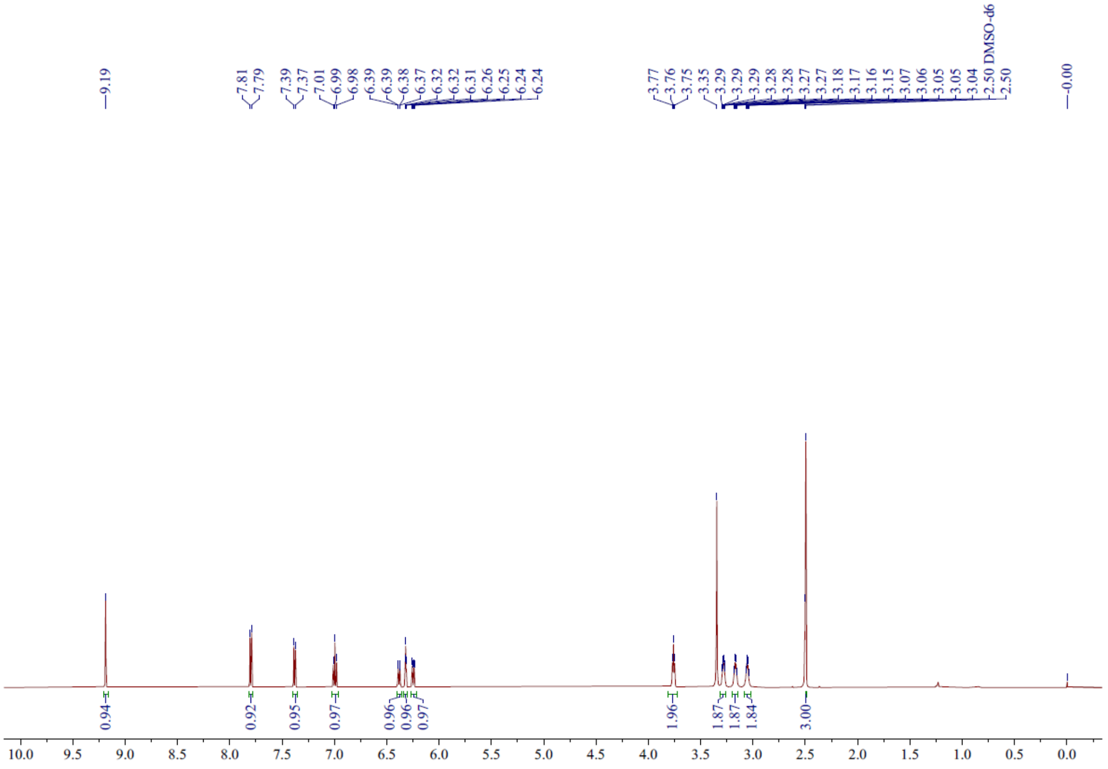

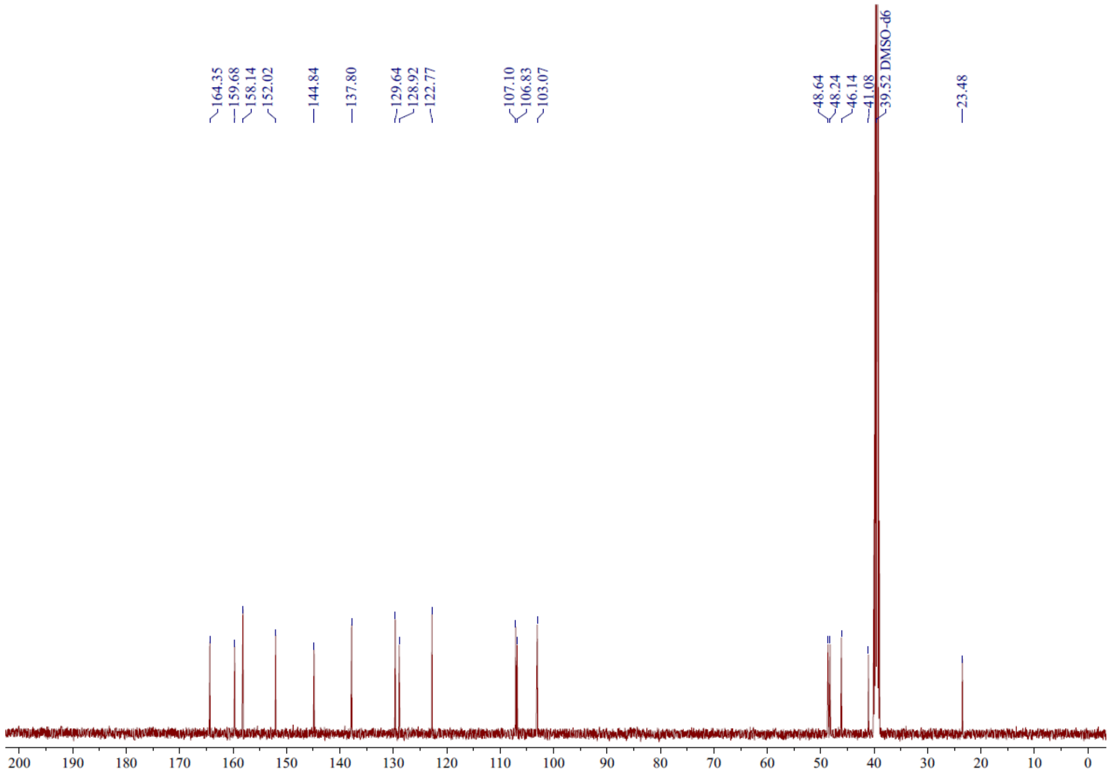

***2-(4-hydroxyphenyl)-1-(4-(3-hydroxyphenyl)piperazin-1-yl)propan-1-one* (AI10-a18)**

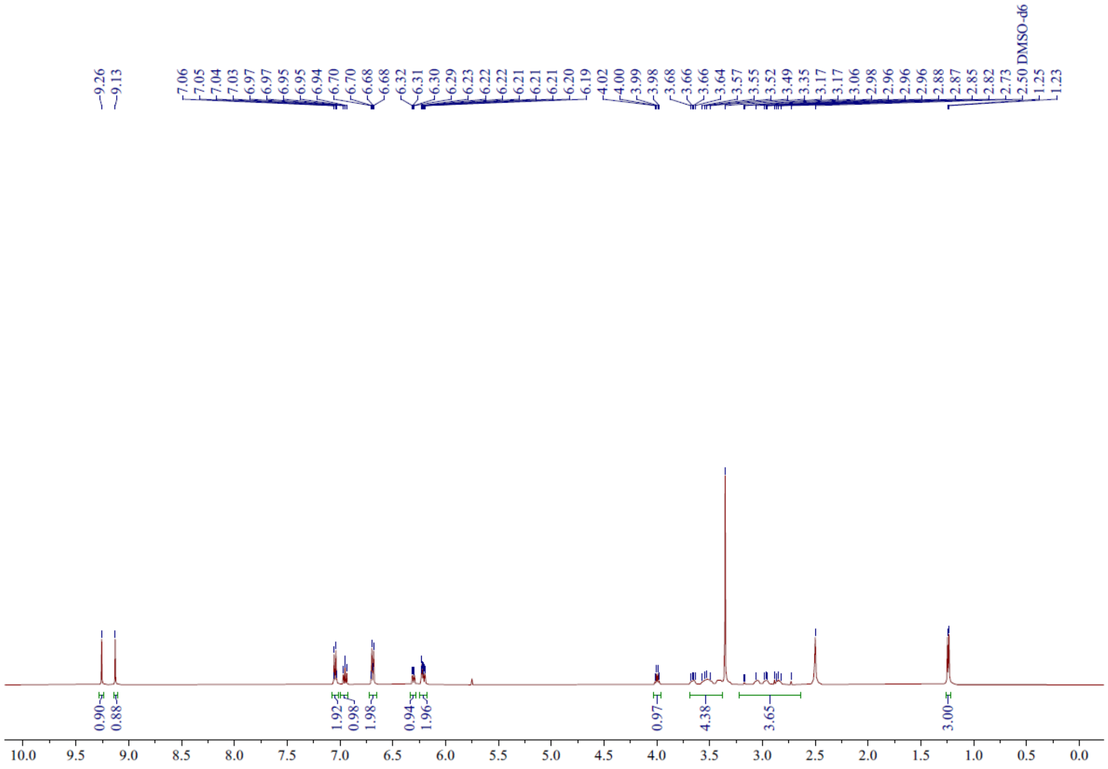

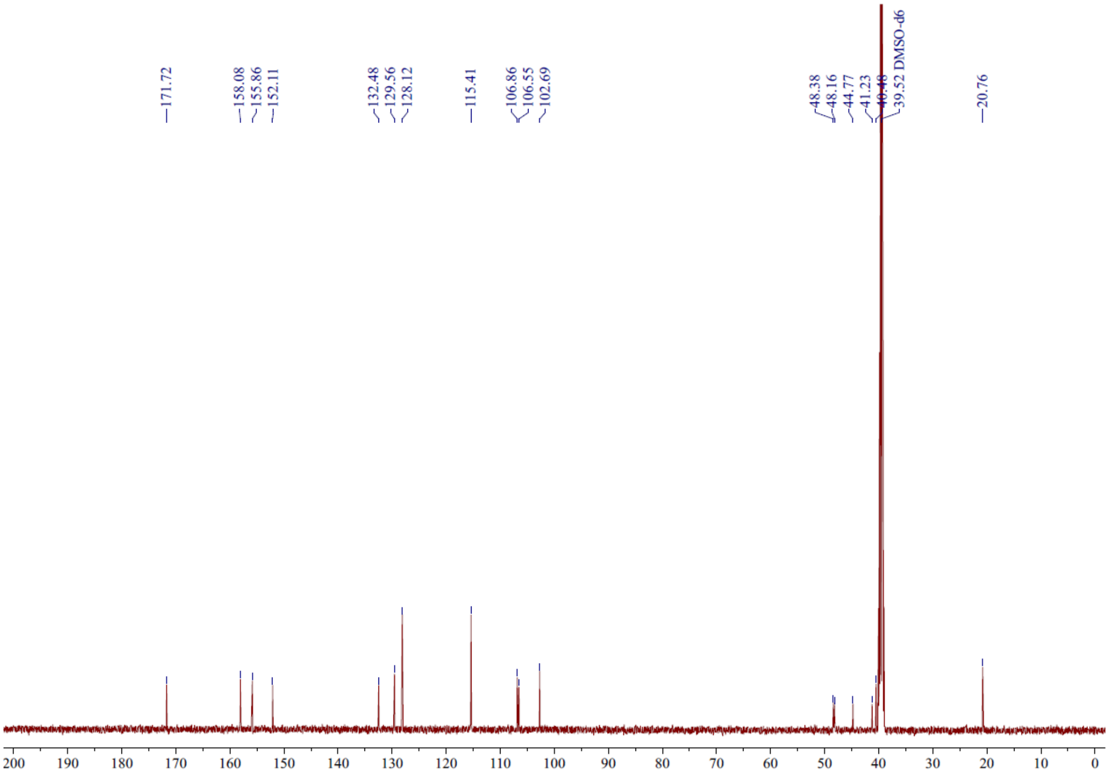

(4-hydroxyphenyl)(4-(p-tolyl)piperazin-1-yl)methanone **(AI10-a19)**

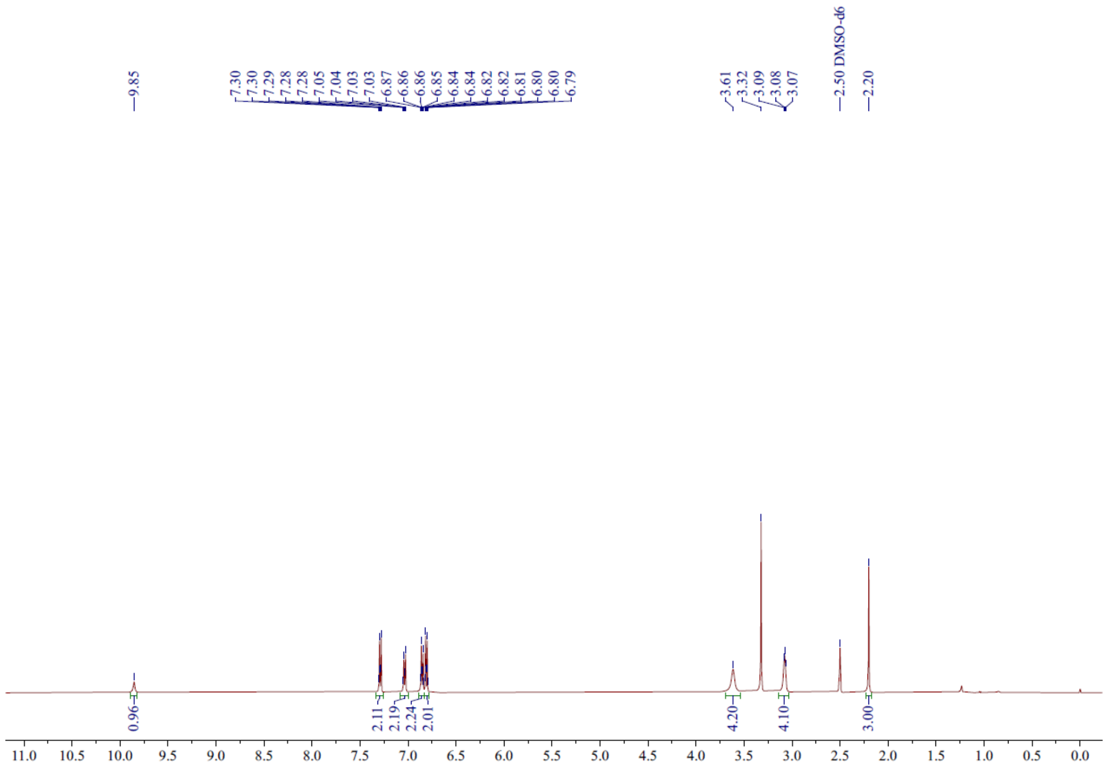

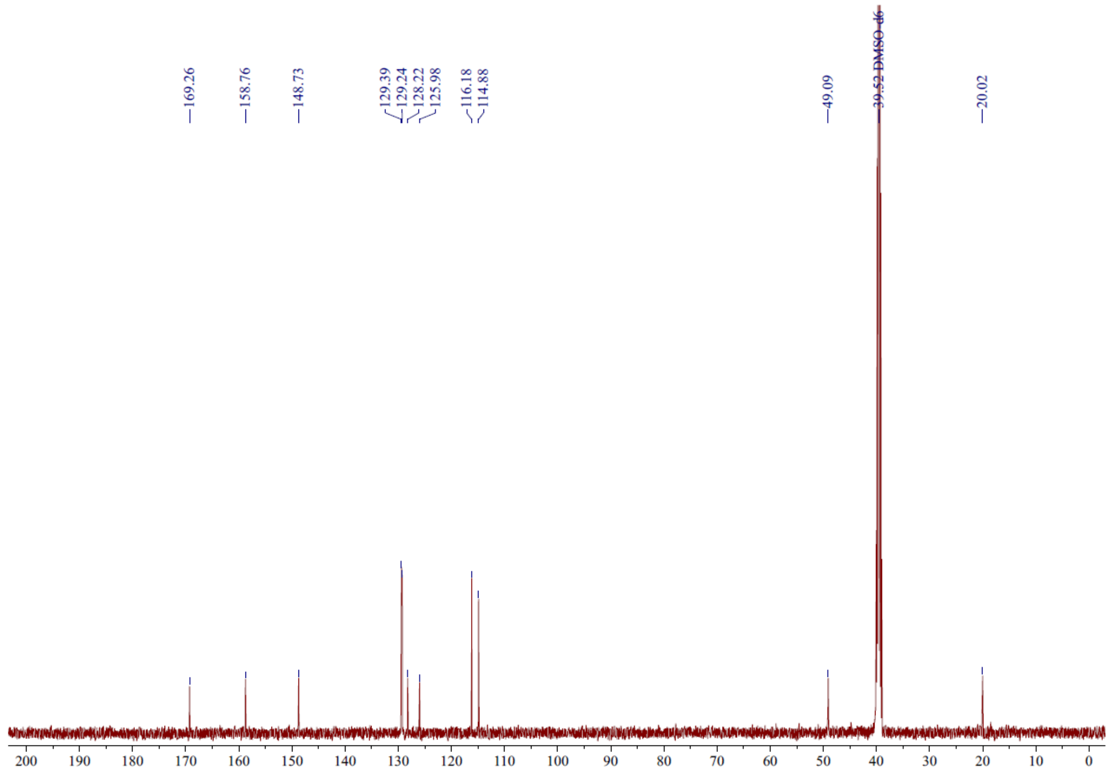

(3,4-dihydroxyphenyl)(4-(p-tolyl)piperazin-1-yl)methanone **(AI10-a20)**

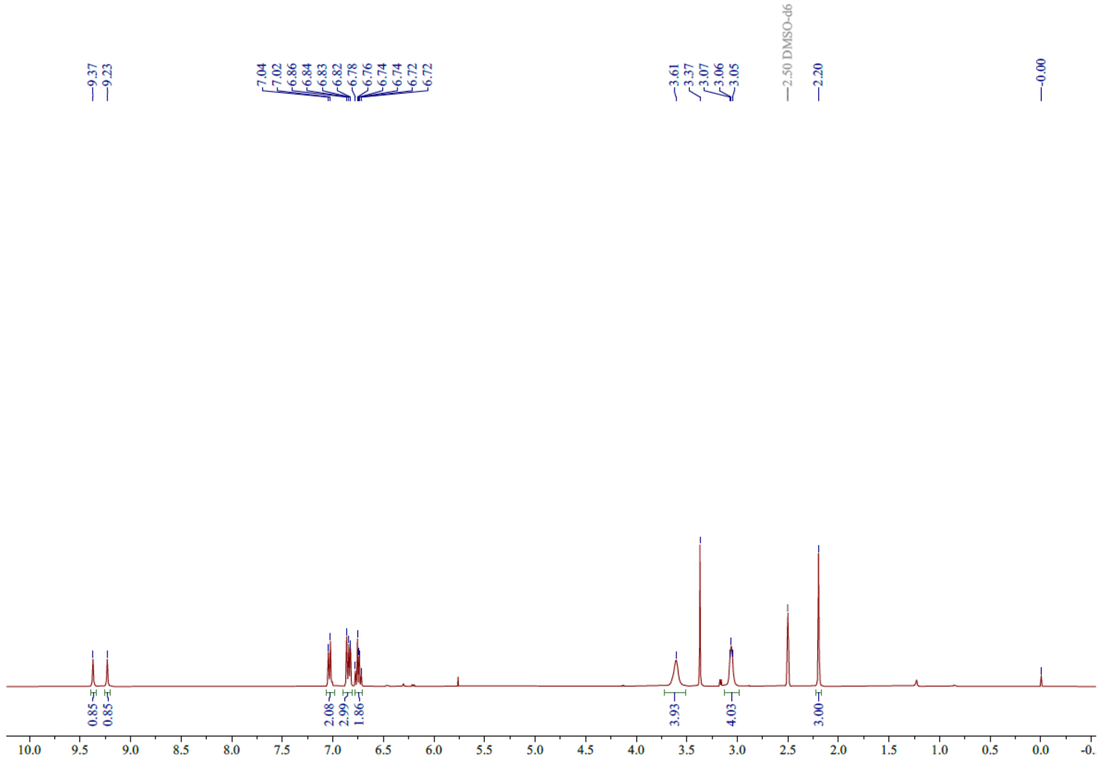

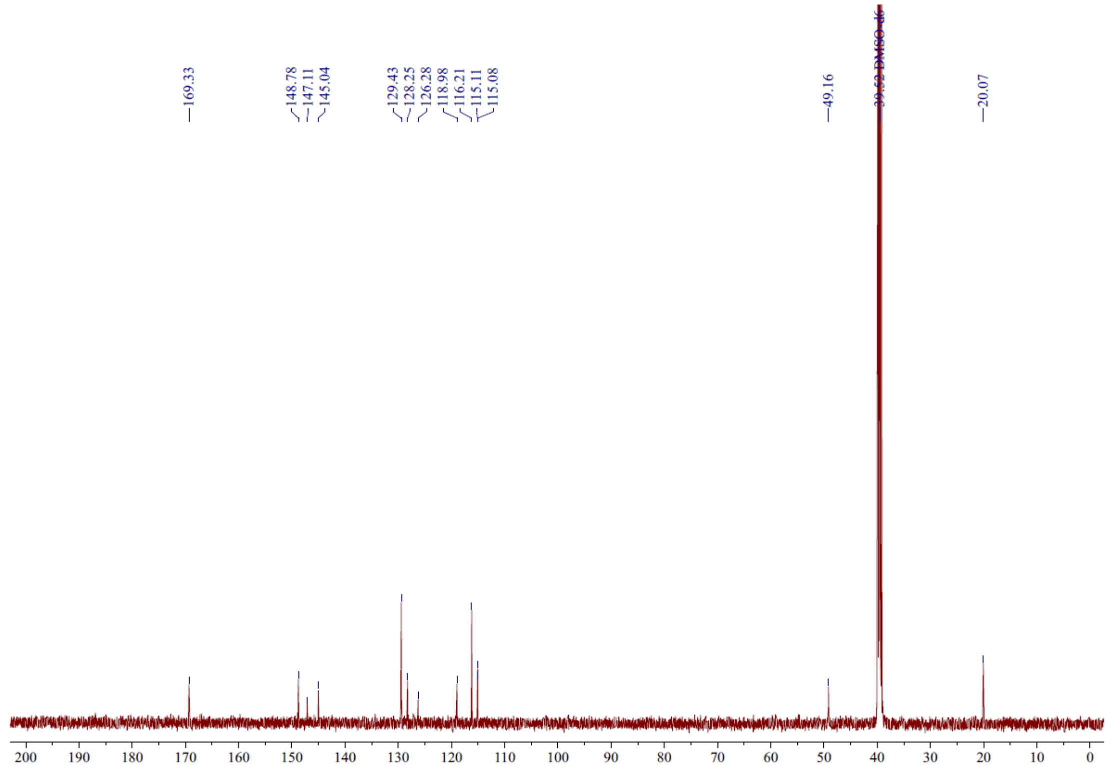

***(2,5-dihydroxyphenyl)(4-(p-tolyl)piperazin-1-yl)methanone* (AI10-a21)**

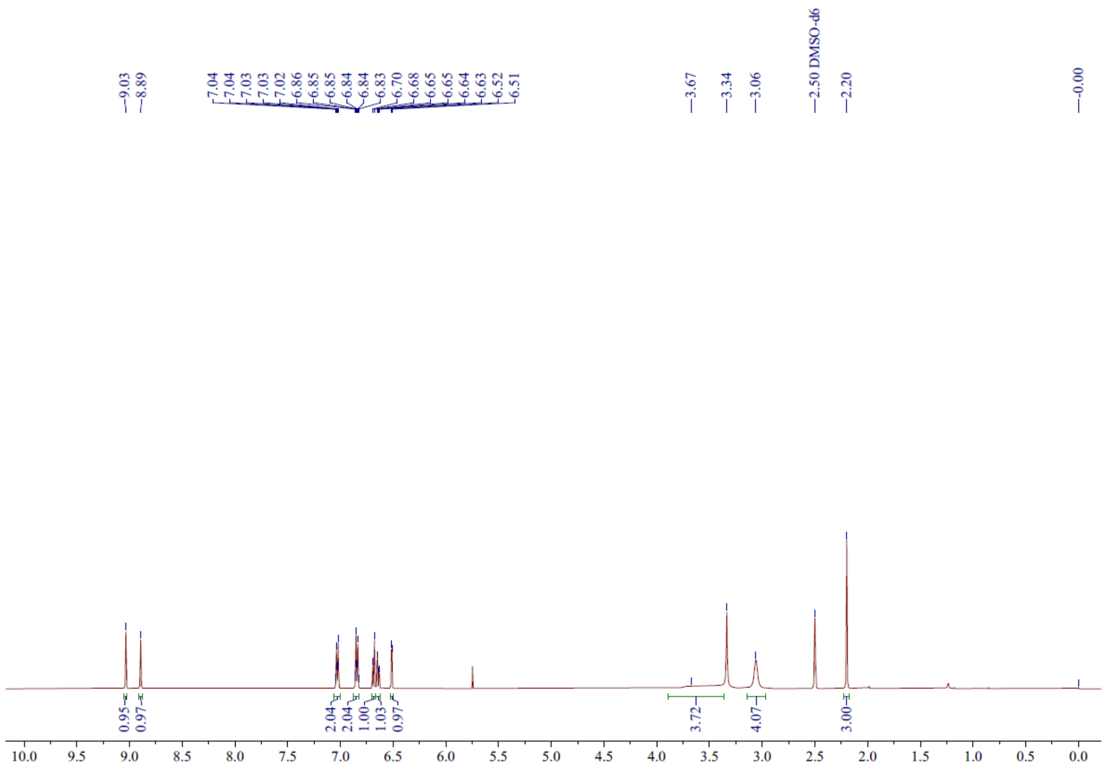

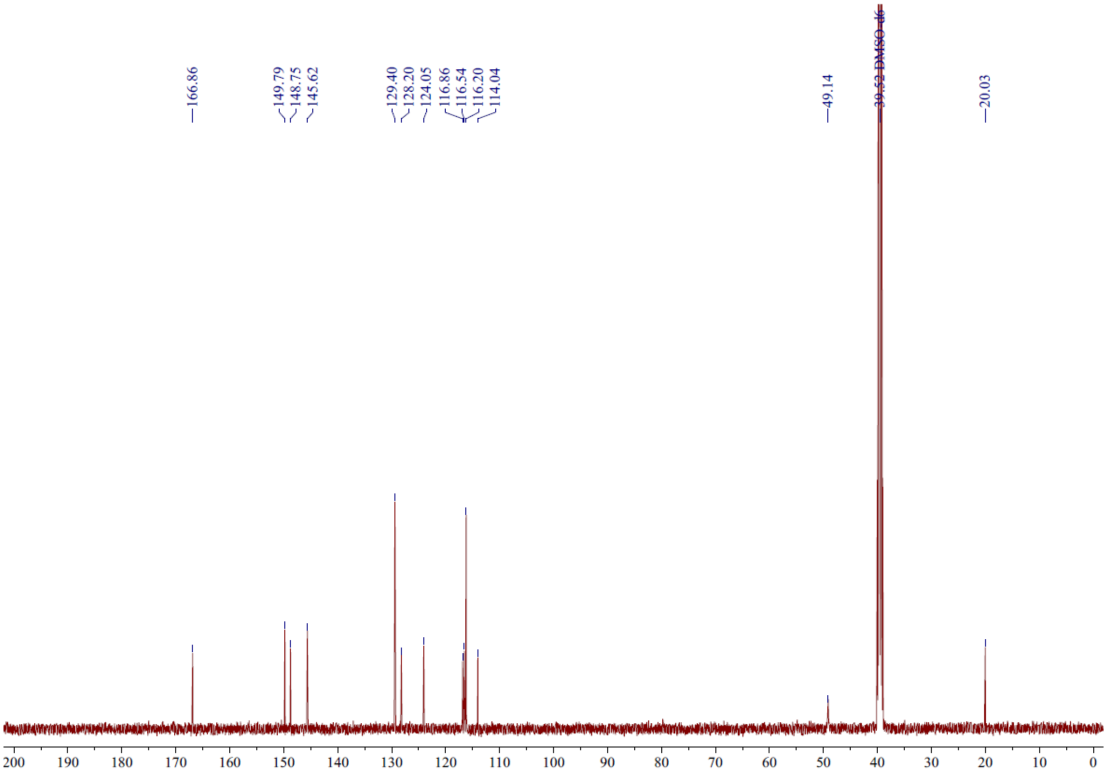

(3,5-dihydroxyphenyl)(4-(p-tolyl)piperazin-1-yl)methanone **(AI10-a22)**

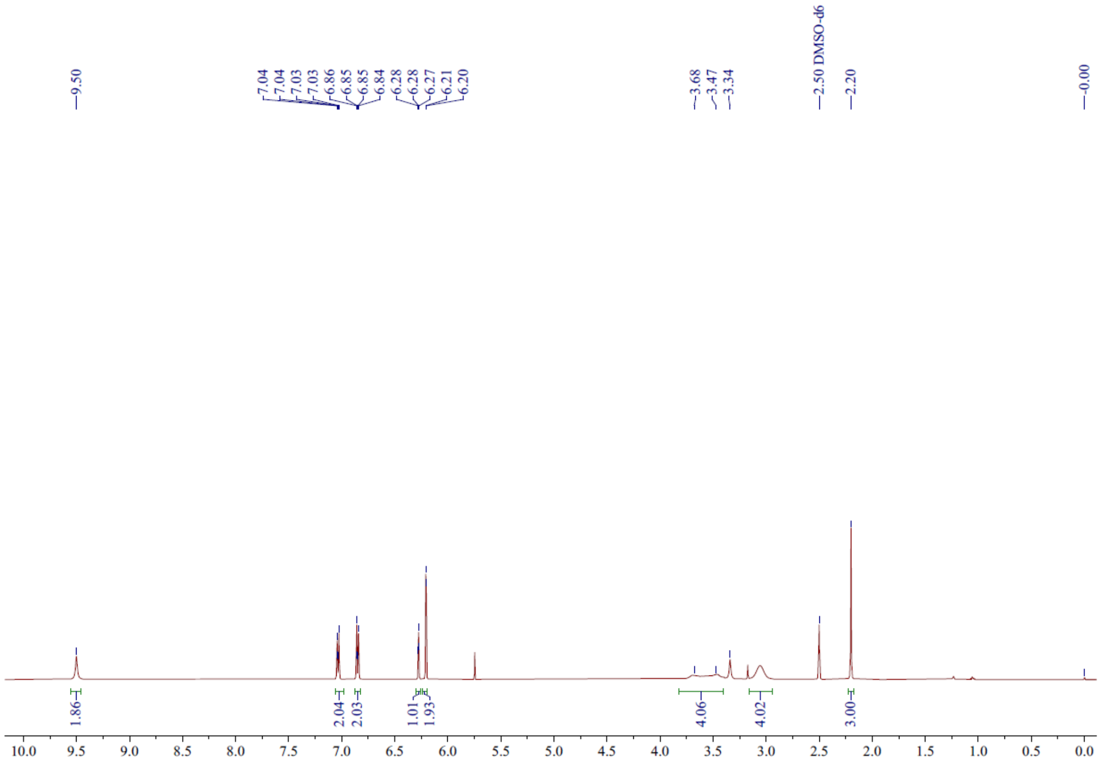

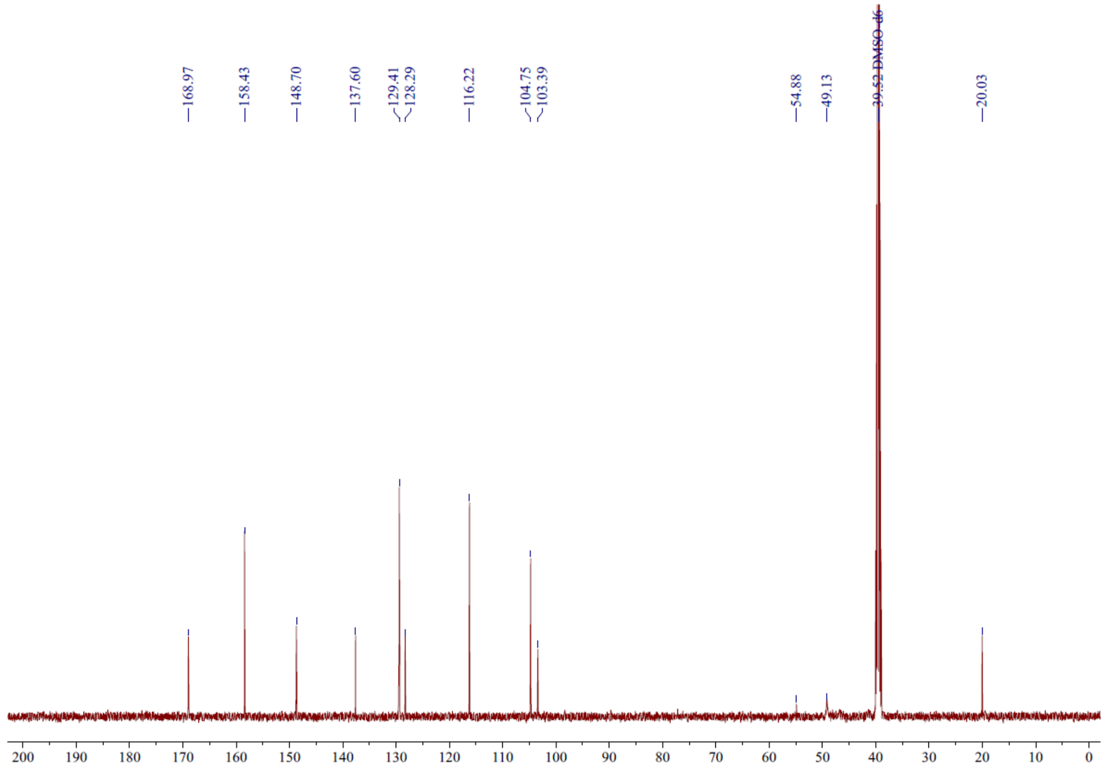

(3-fluoro-5-hydroxyphenyl)(4-(p-tolyl)piperazin-1-yl)methanone **(AI10-a23)**

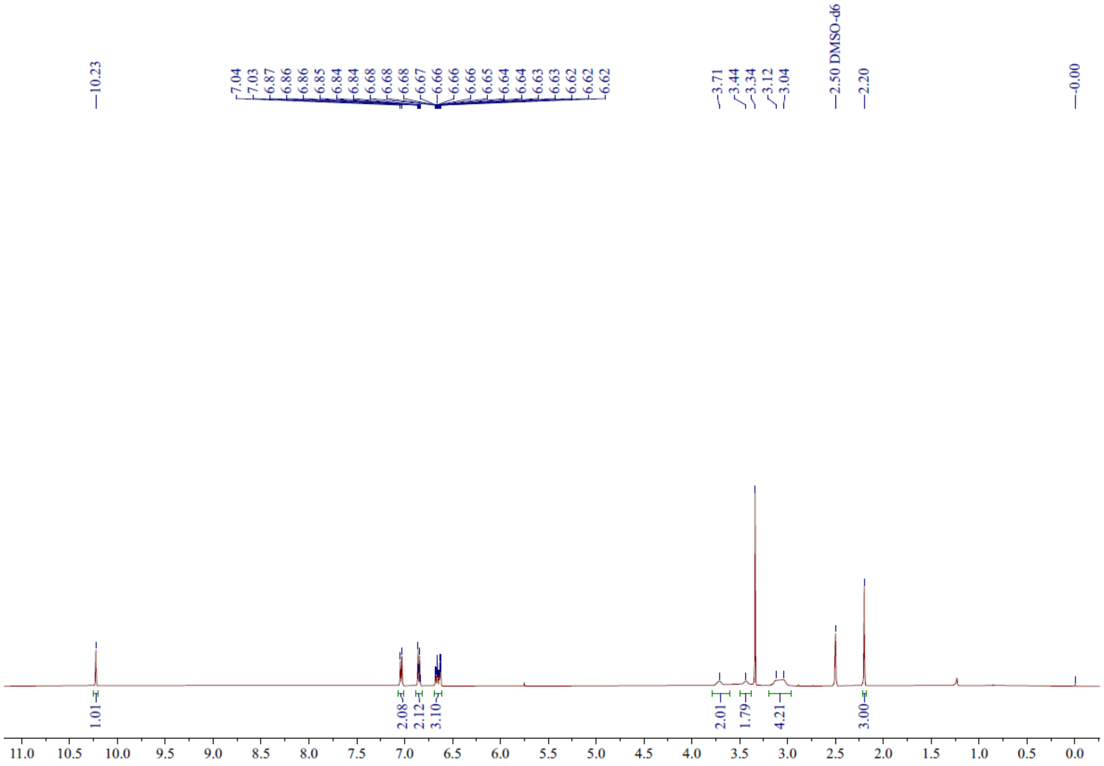

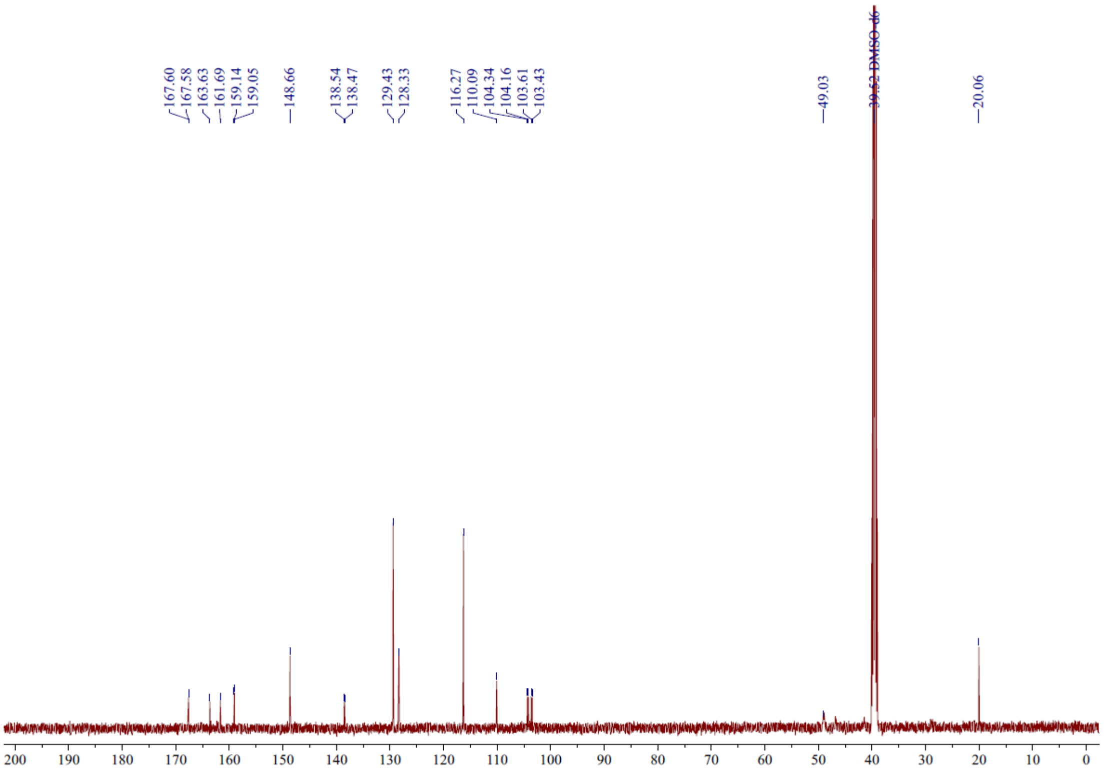

(2-chloro-5-hydroxyphenyl)(4-(p-tolyl)piperazin-1-yl)methanone **(AI10-a24)**

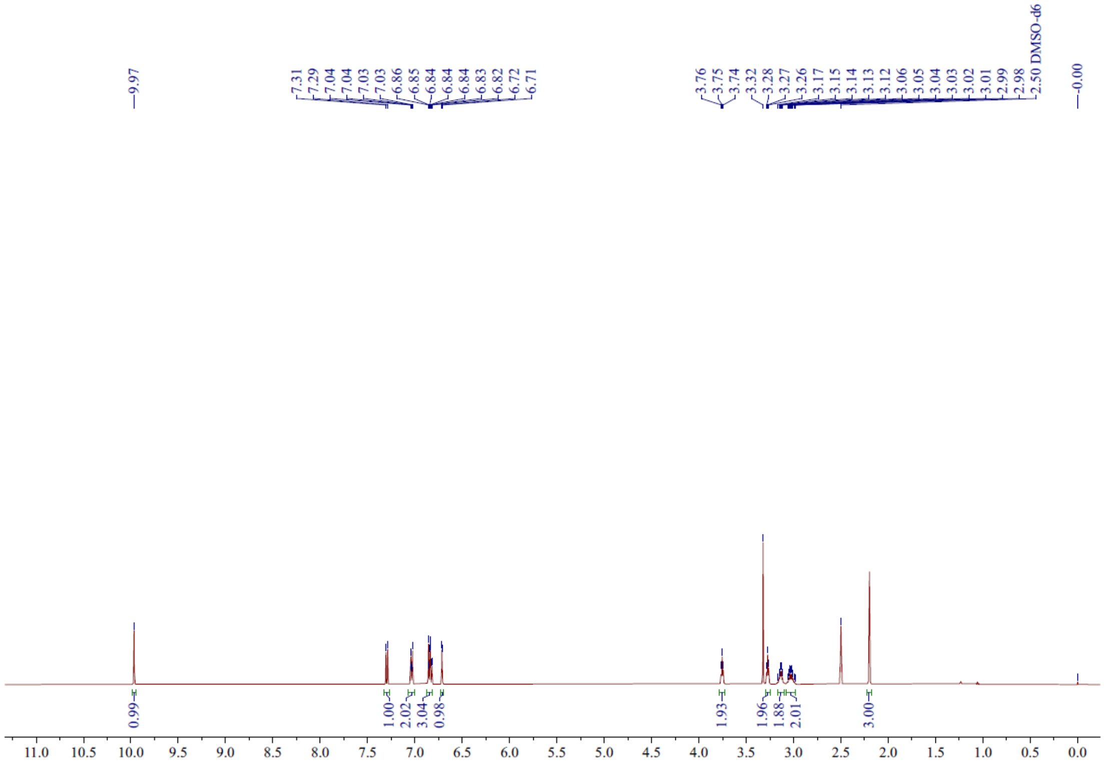

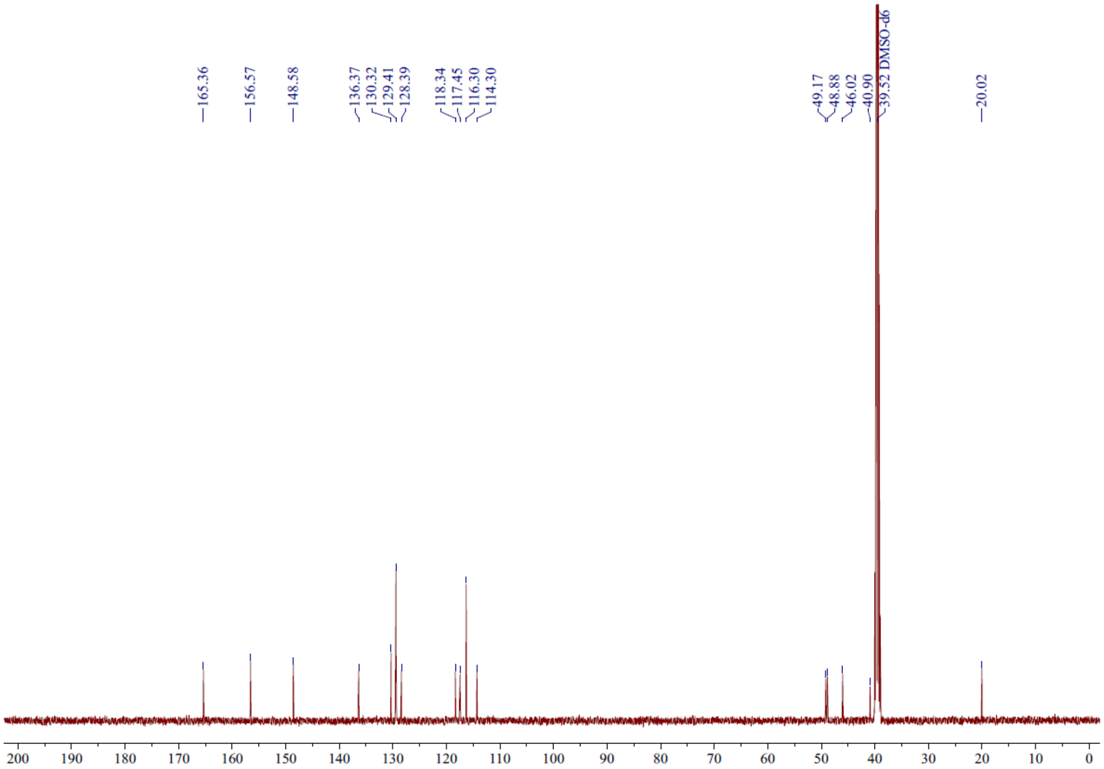

***3-(4-hydroxyphenyl)-1-(4-(p-tolyl)piperazin-1-yl)propan-1-one* (AI10-a25)**

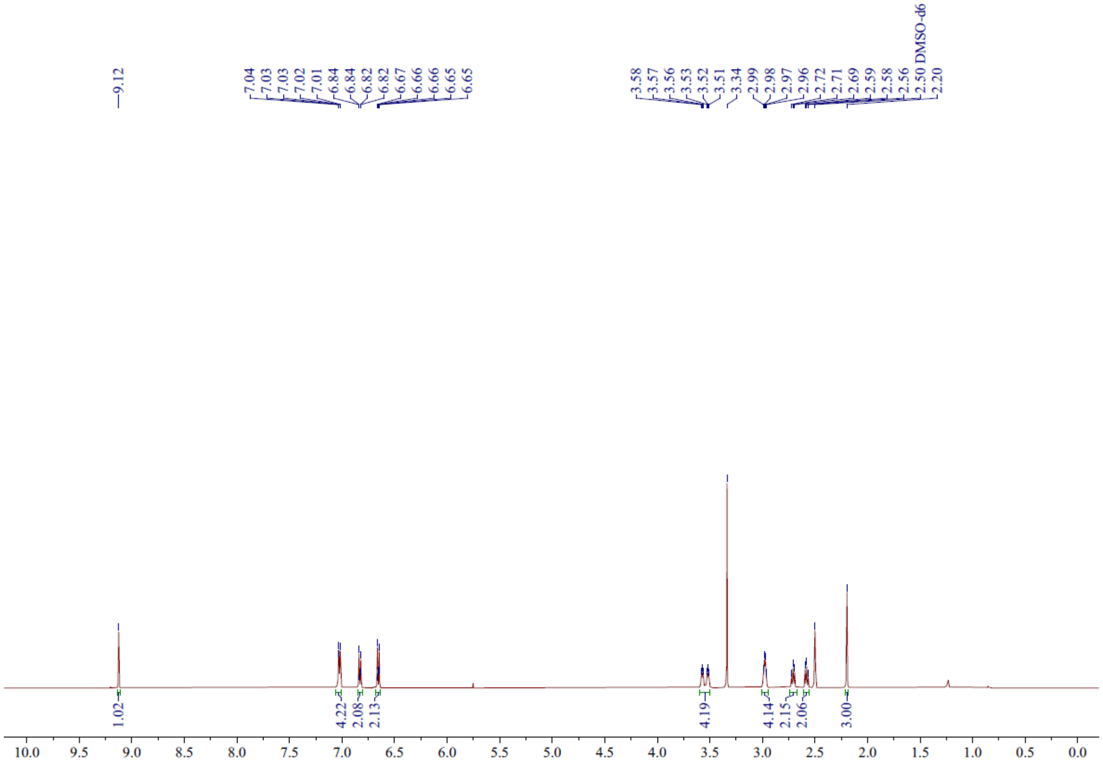

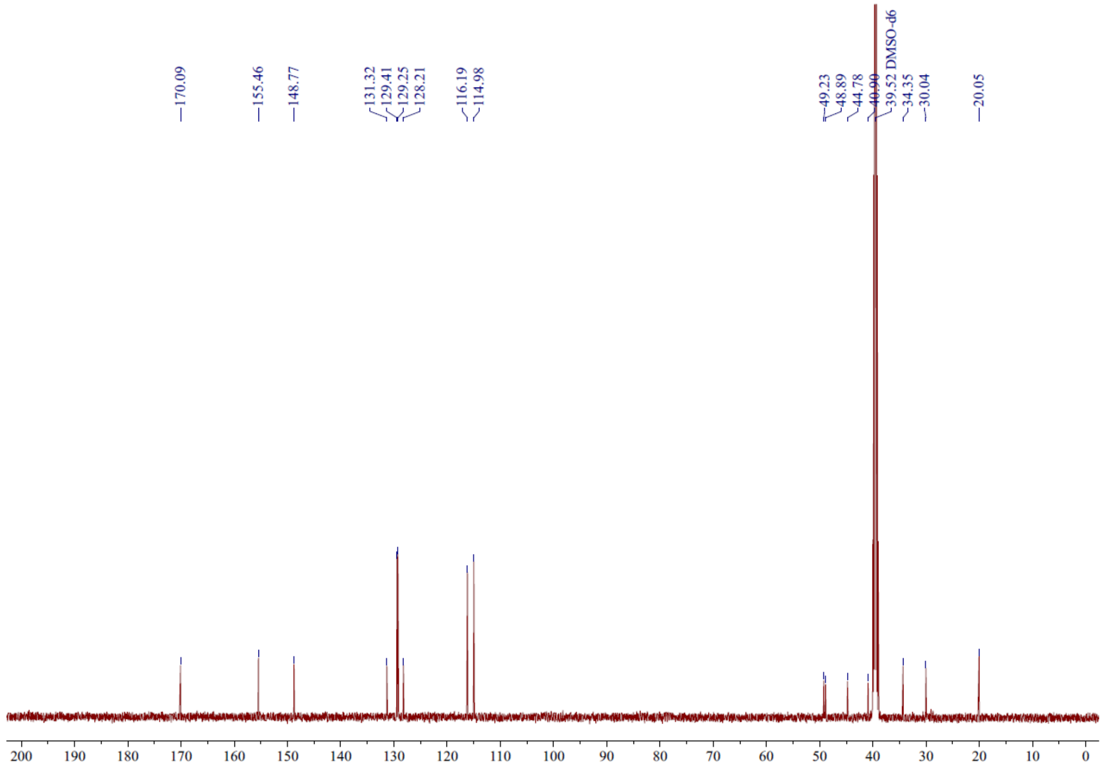

(4-(4-chlorophenyl)piperazin-1-yl)(3-hydroxy-5-methylphenyl)methanone **(AI10-a26)**

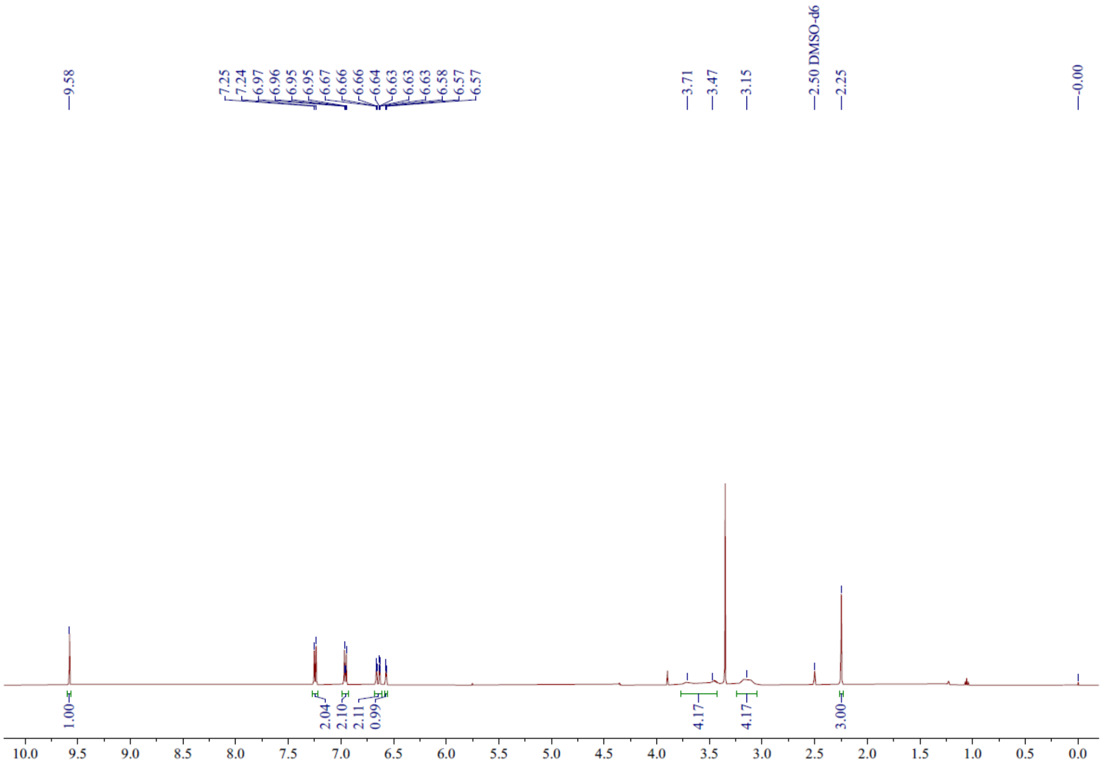

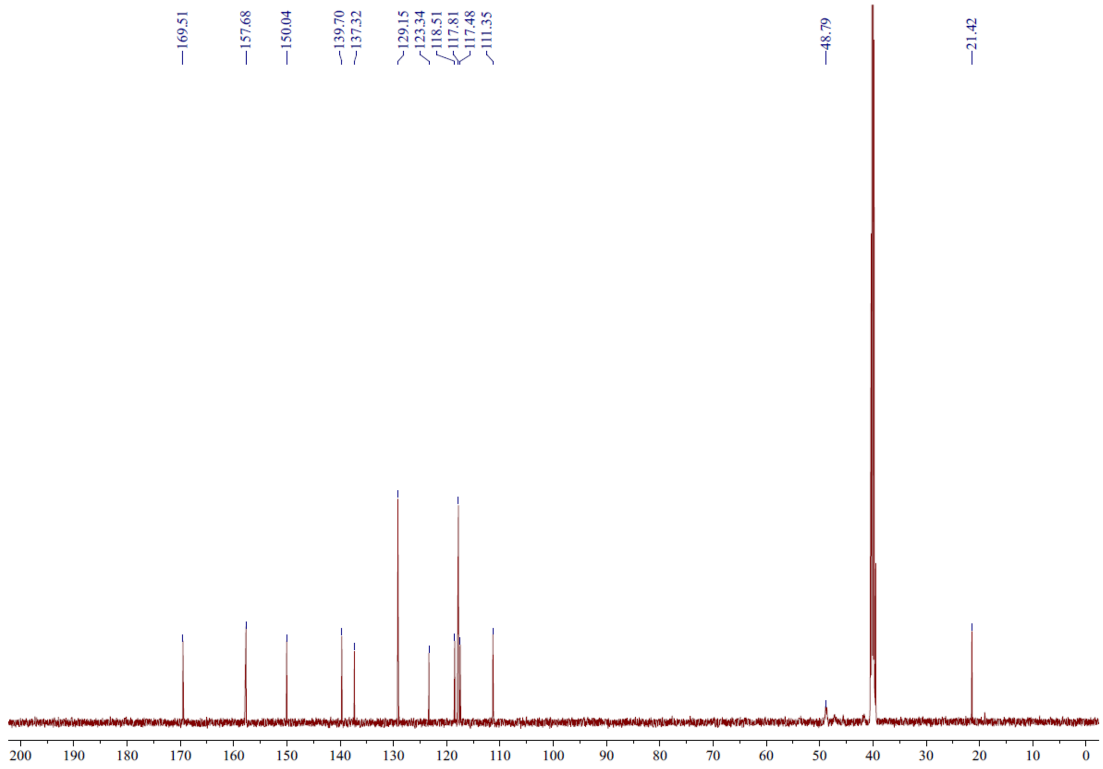

***(4-(4-chlorophenyl)piperazin-1-yl)(5-hydroxy-2-methylphenyl)methanone* (AI10-a27)**

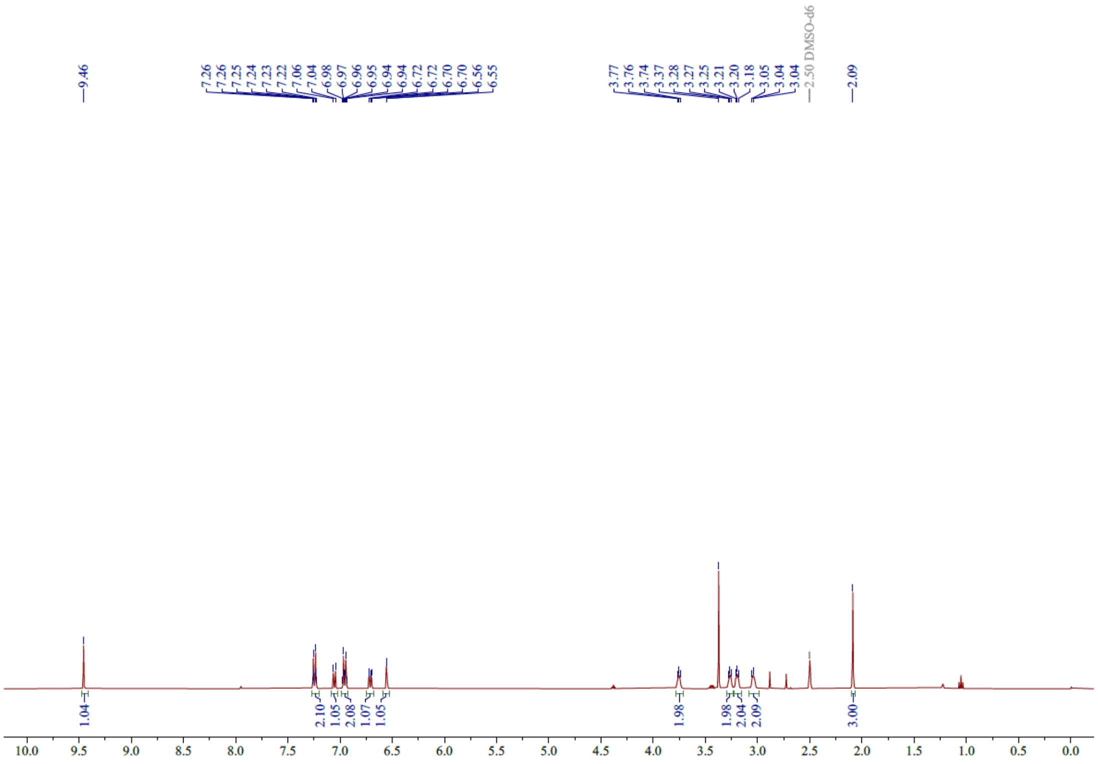

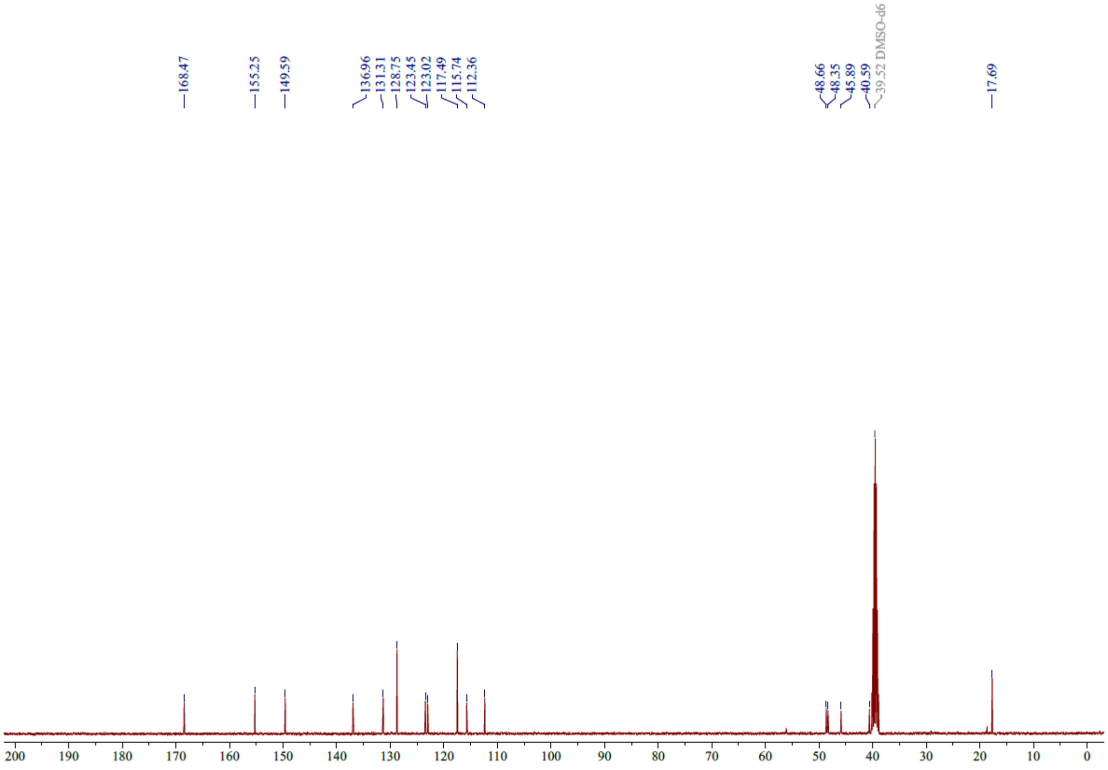

(2,6-dimethylpyridin-4-yl)(6-hydroxy-3,4-dihydroisoquinolin-2(1H) yl)methanone **(AI10-a28)**

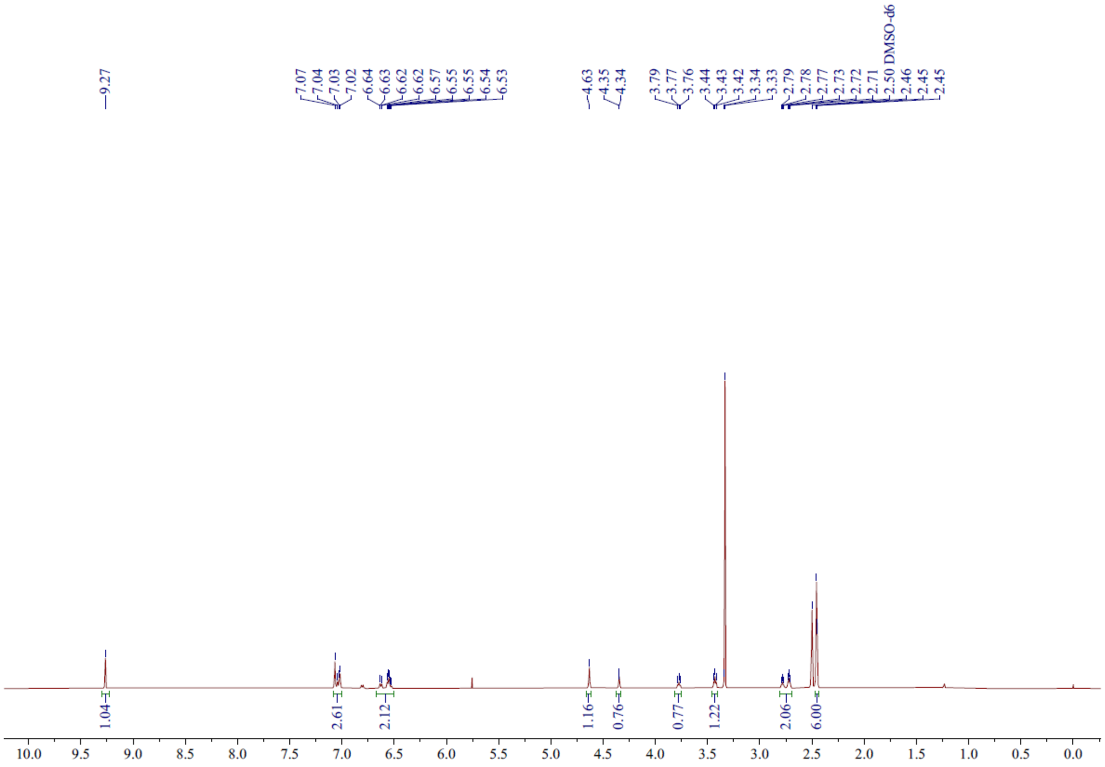

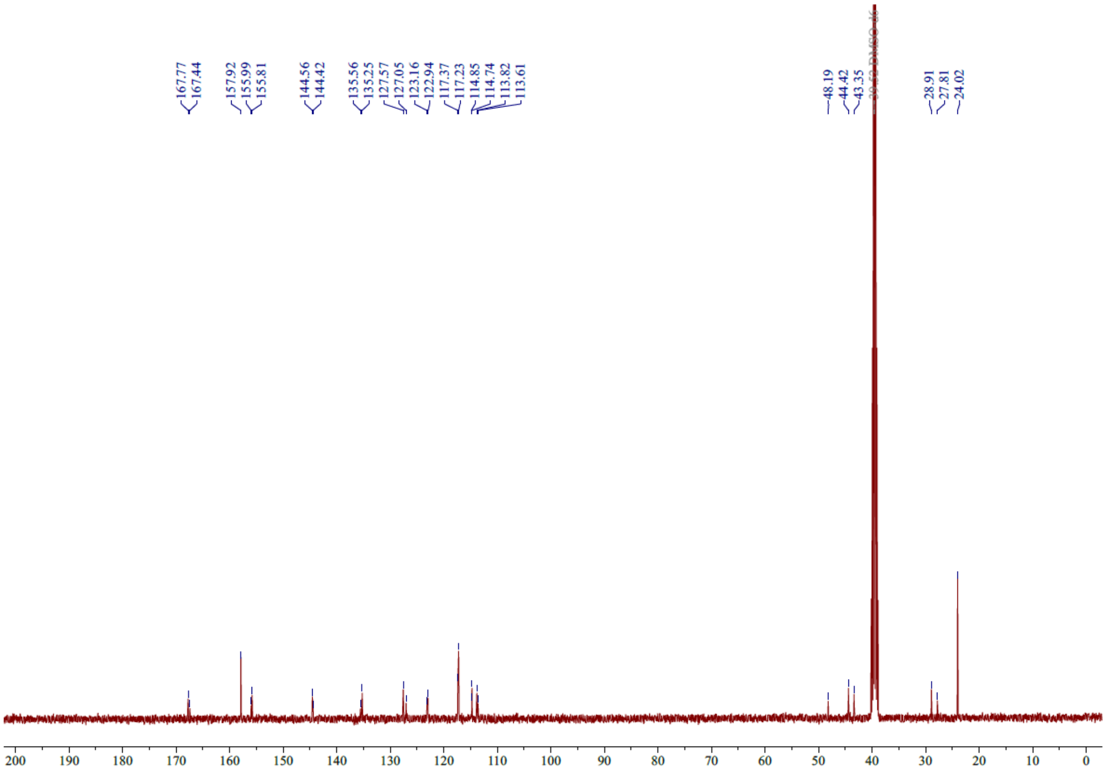

4-(4-(4-hydroxy-3,5-dimethylbenzoyl)piperazin-1-yl)benzonitrile (AI10-a29)

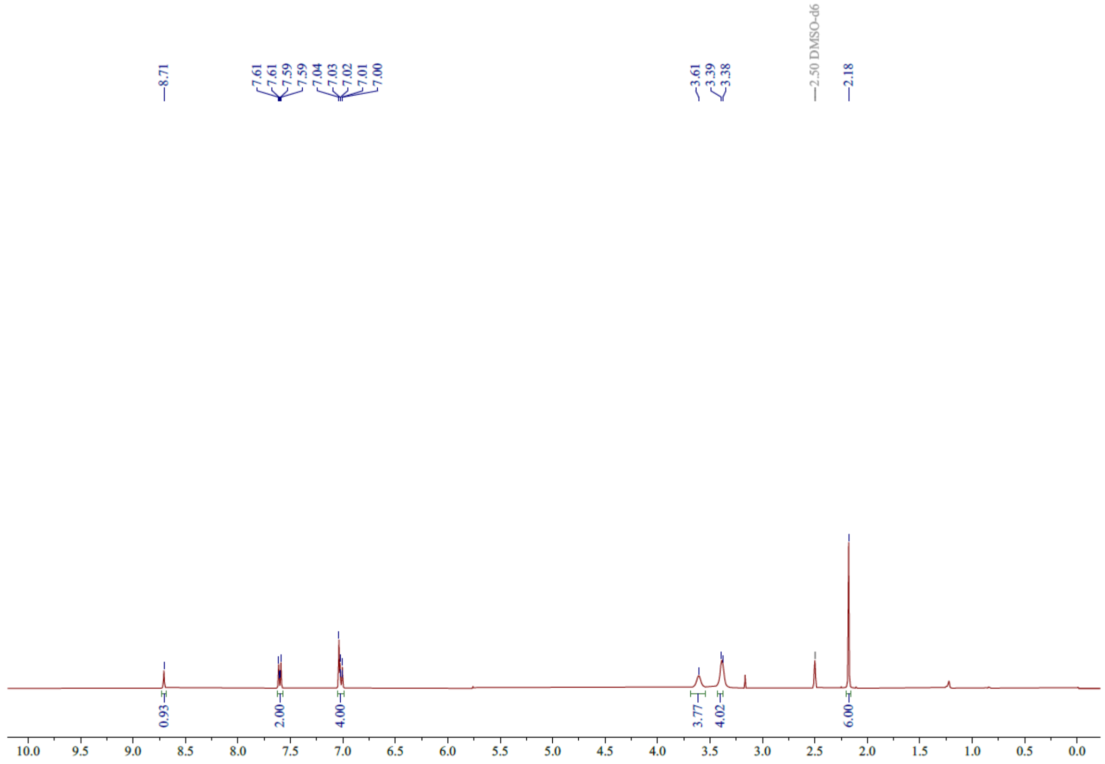

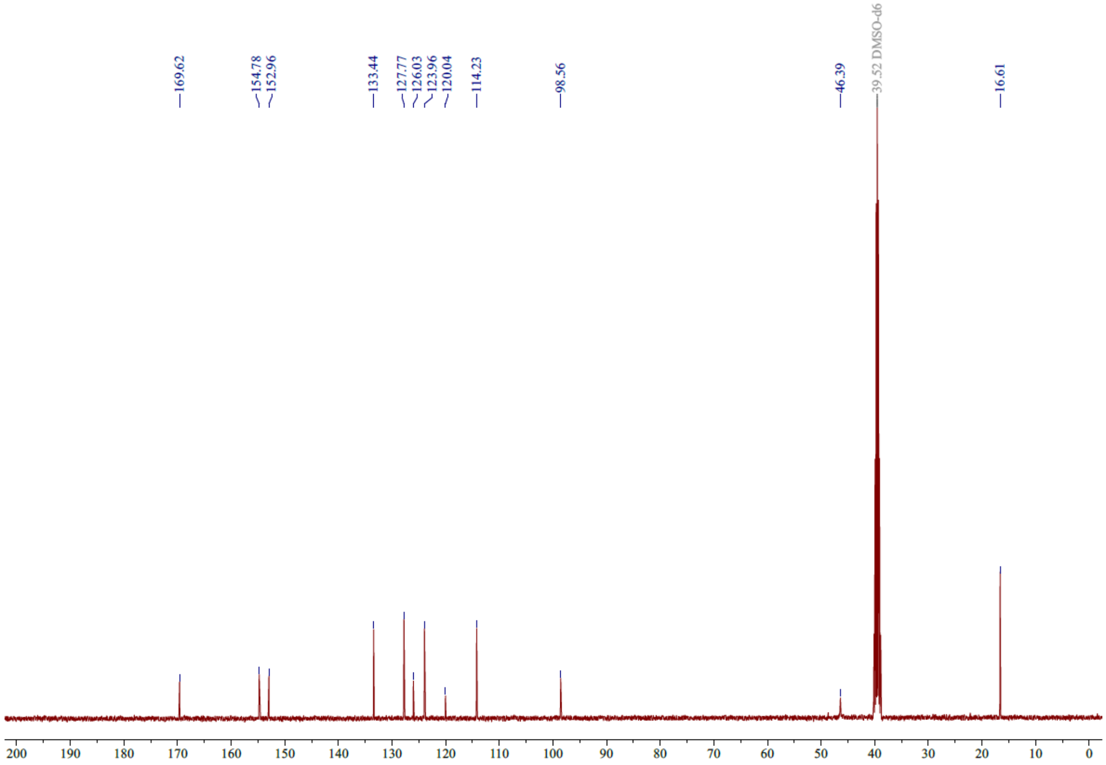

(4-(4-hydroxybenzoyl)piperazin-1-yl)(2-methylquinolin-4-yl)methanone **(AI10-a30)**

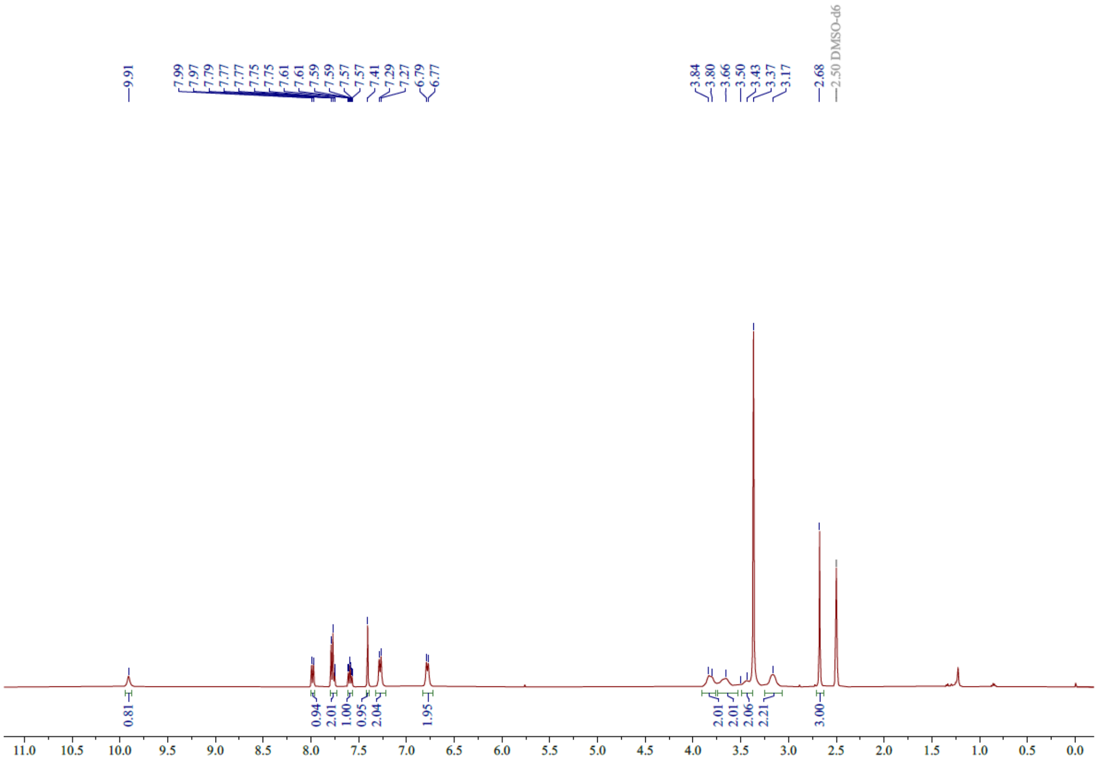

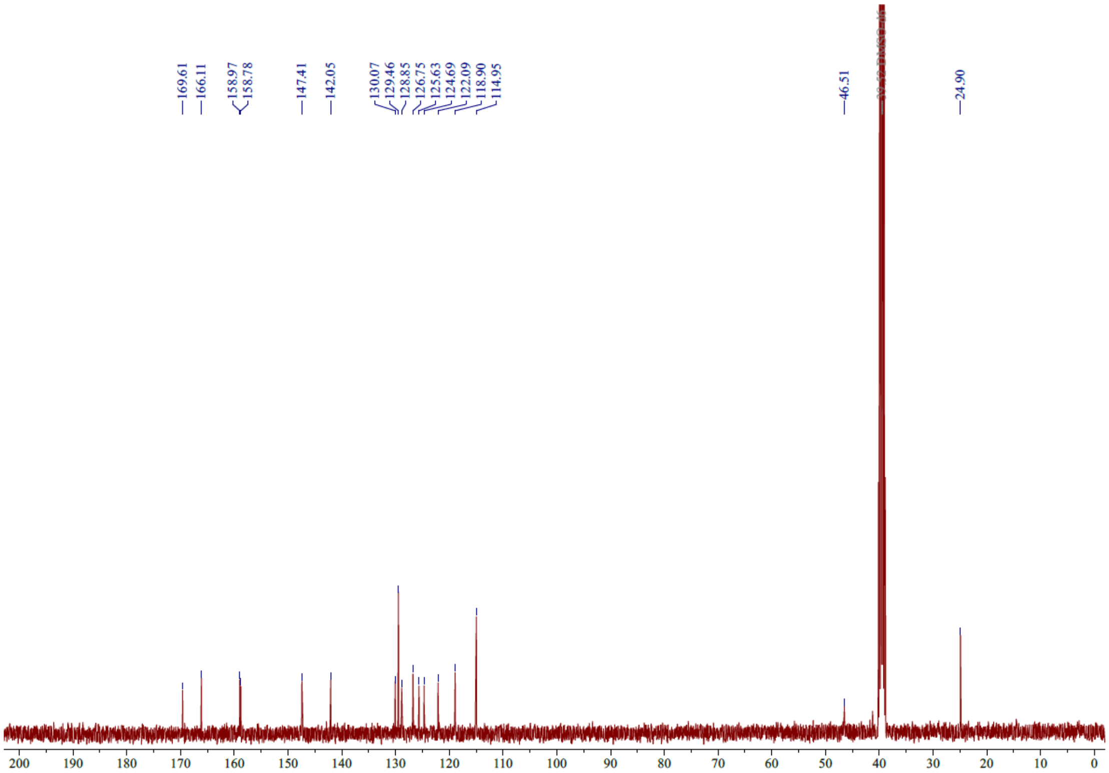

(4-(4-hydroxybenzoyl)piperazin-1-yl)(m-tolyl)methanone **(AI10-a31)**

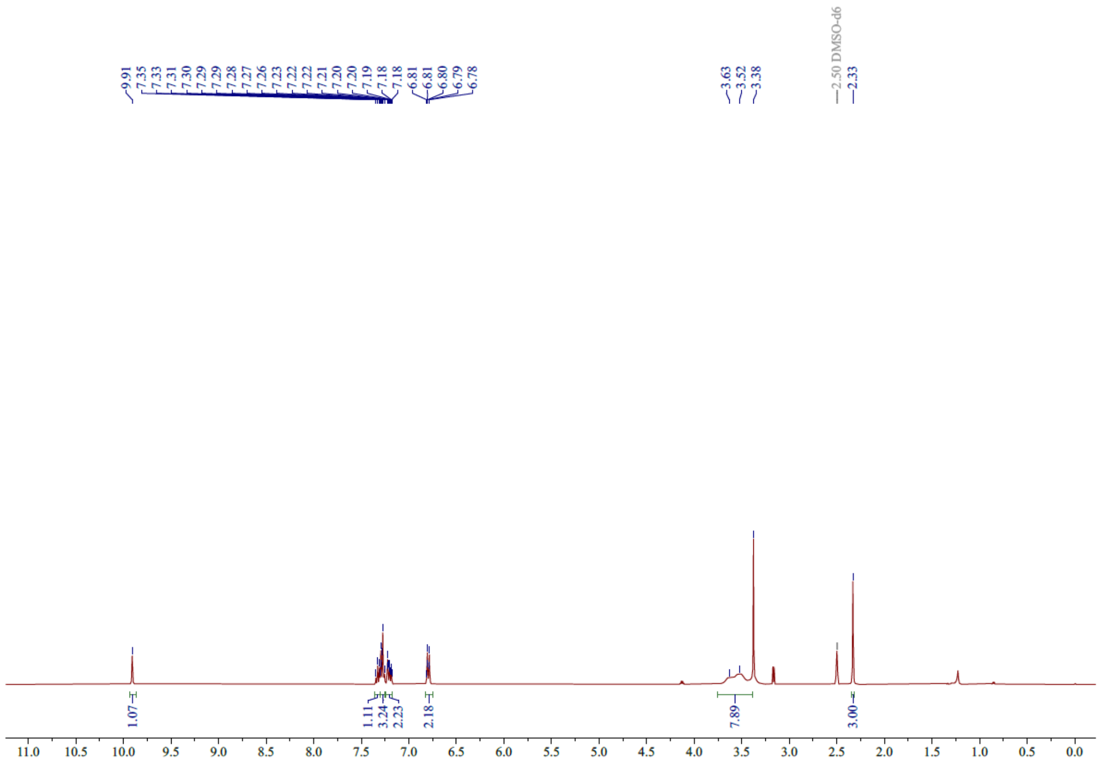

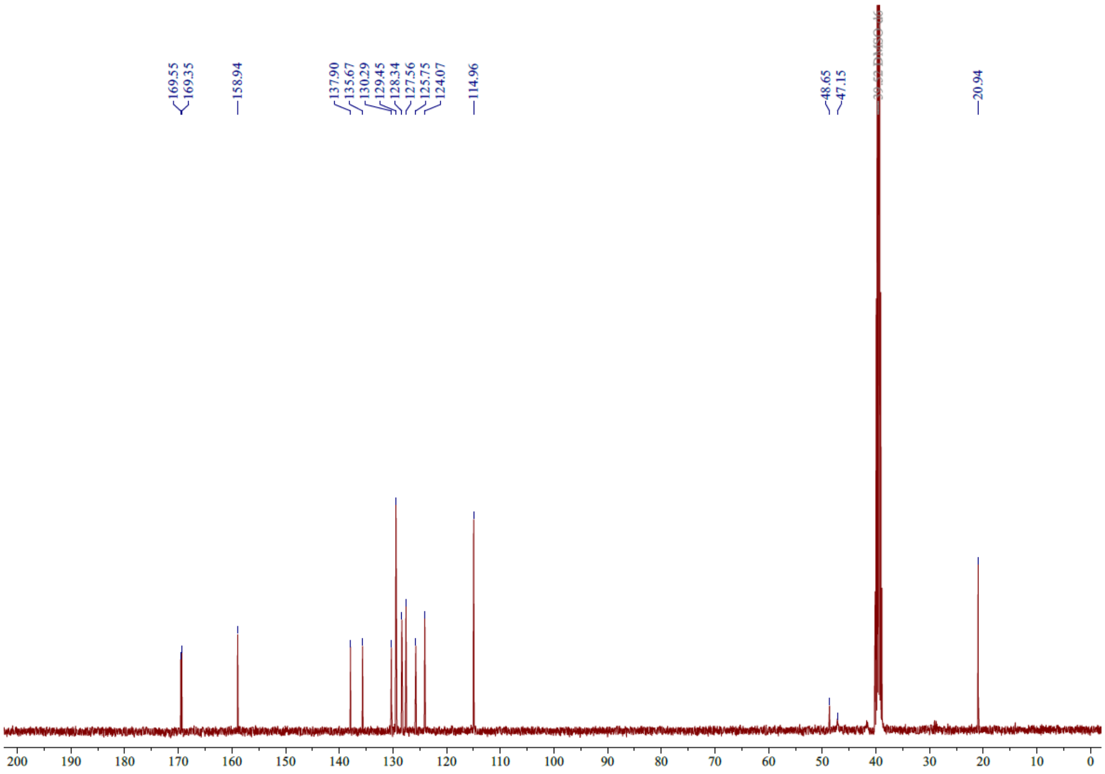

1-(4-(4-hydroxybenzoyl)piperazin-1-yl)-3-(m-tolyl)propan-1-one **(AI10-a32)**

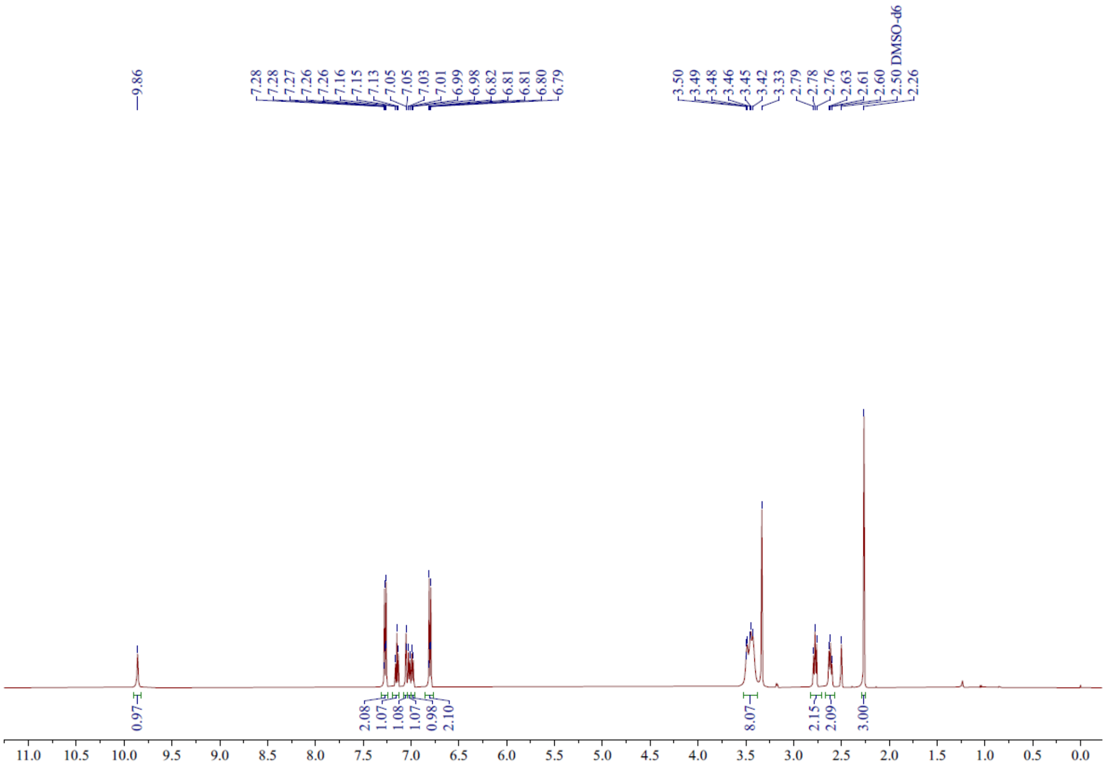

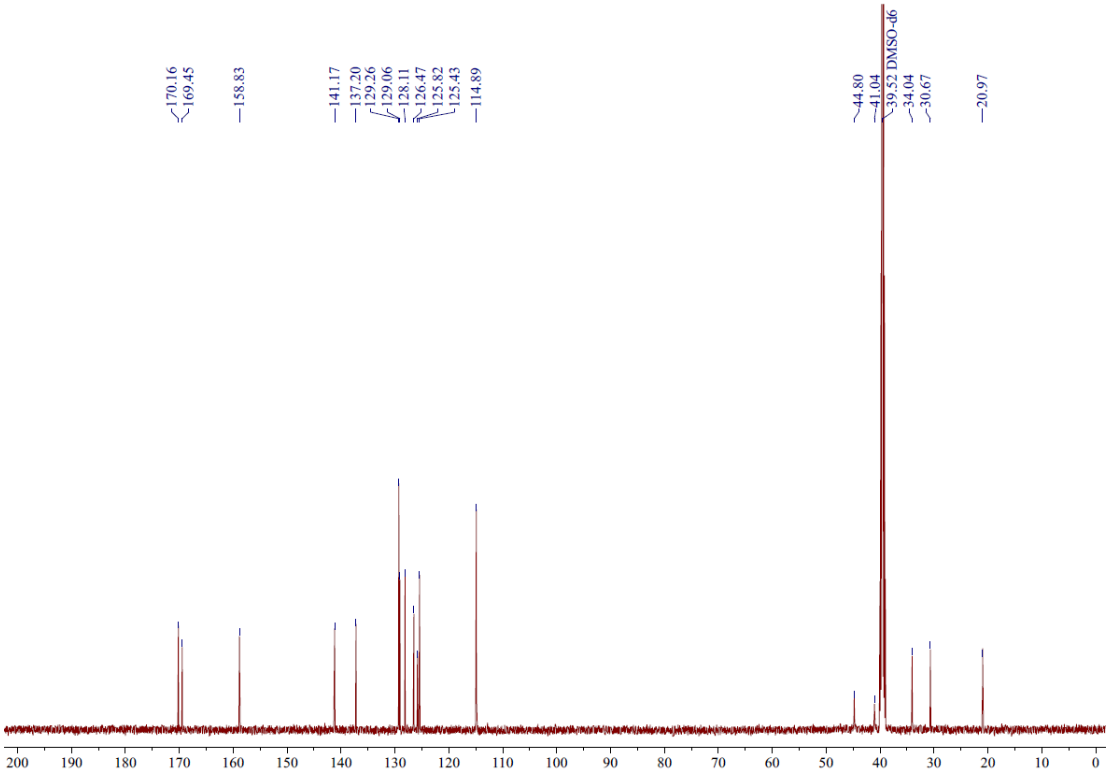

HRMS and HPLC analysis result data of target compounds

(2-hydroxyphenyl)(4-(p-tolyl)piperazin-1-yl)methanone **(AI1)**

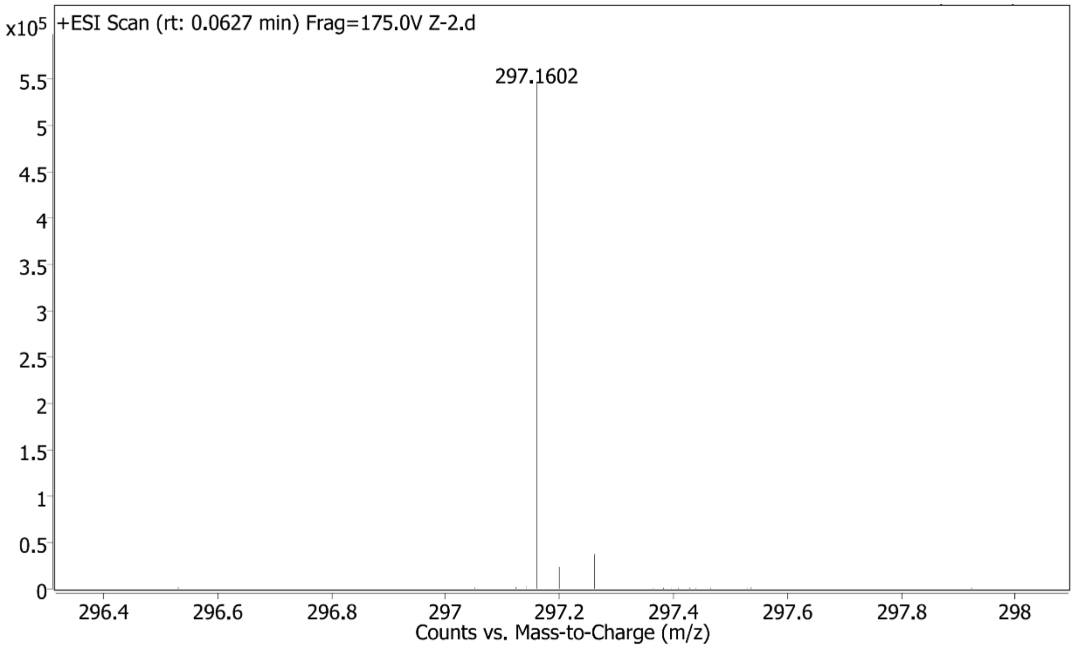

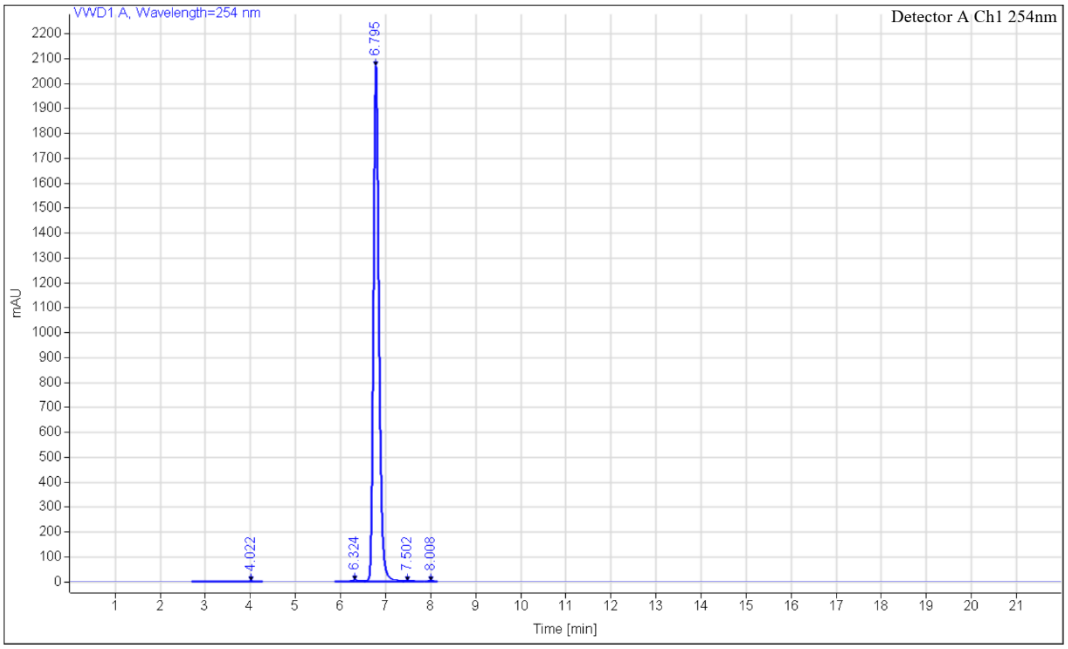

⟨Peak table⟩

Detector A Ch1 254nm

Number	Retention time	Peak area	Peak area%	Separation efficiency
1	4. 022	59. 68397	0. 33	--
2	6. 324	89. 62962	0. 50	5. 47823
3	6. 795	17736. 08789	98. 78	1. 37809
4	7. 502	52. 30208	0. 29	2. 35597
5	8. 008	16. 71774	0. 09	1. 66679
Total		17954. 4213	100. 00	

(USP)

(4-hydroxyphenyl)(4-(o-tolyl)piperazin-1-yl)methanone **(AI2)**

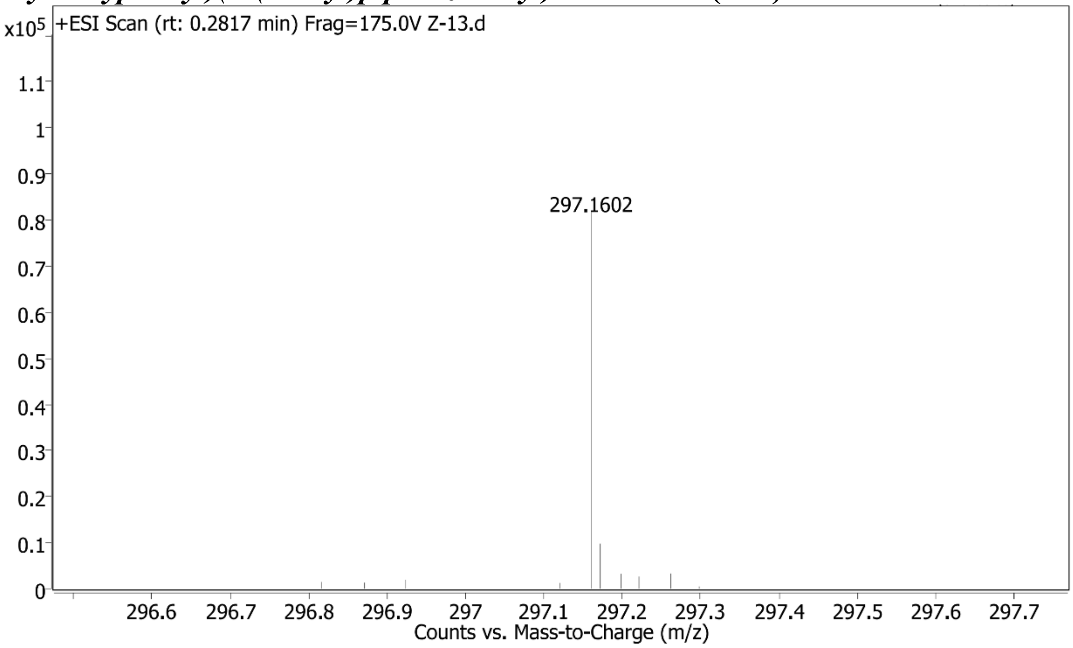

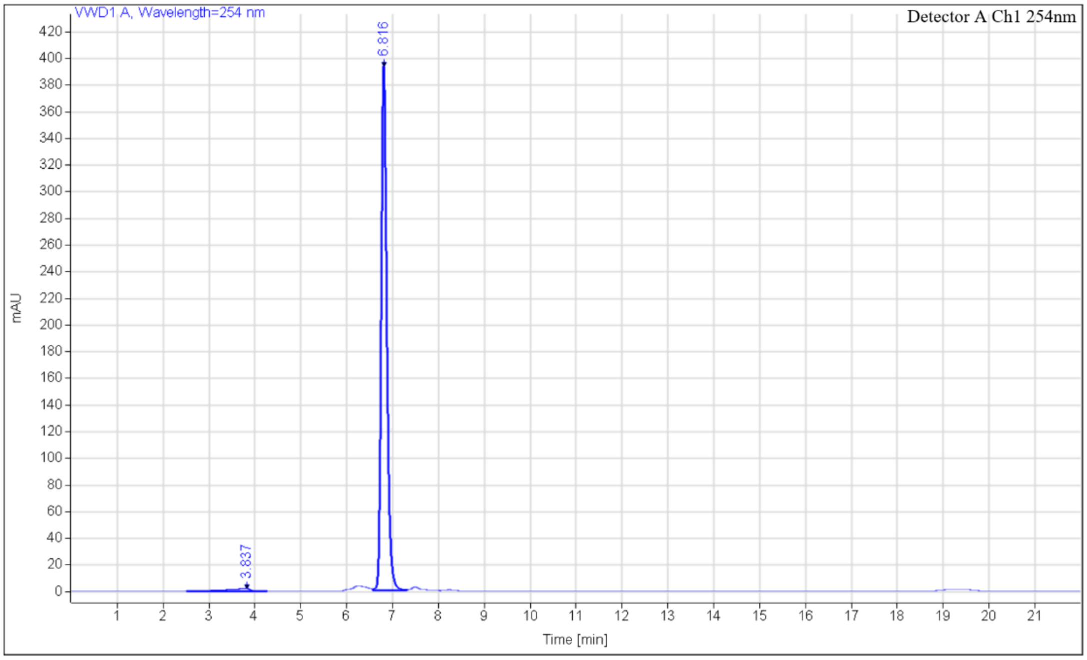

<Peak table>

Detector A Ch1 254nm

Number	Retention time	Peak area	Peak area%	Separation efficiency
1	3. 837	69. 42944	2. 04	--
2	6. 816	3337. 81421	97. 96	7. 98681
Total		3407. 24365	100. 00	

(USP)

(2-hydroxyphenyl)(4-(m-tolyl)piperazin-1-yl)methanone **(AI3)**

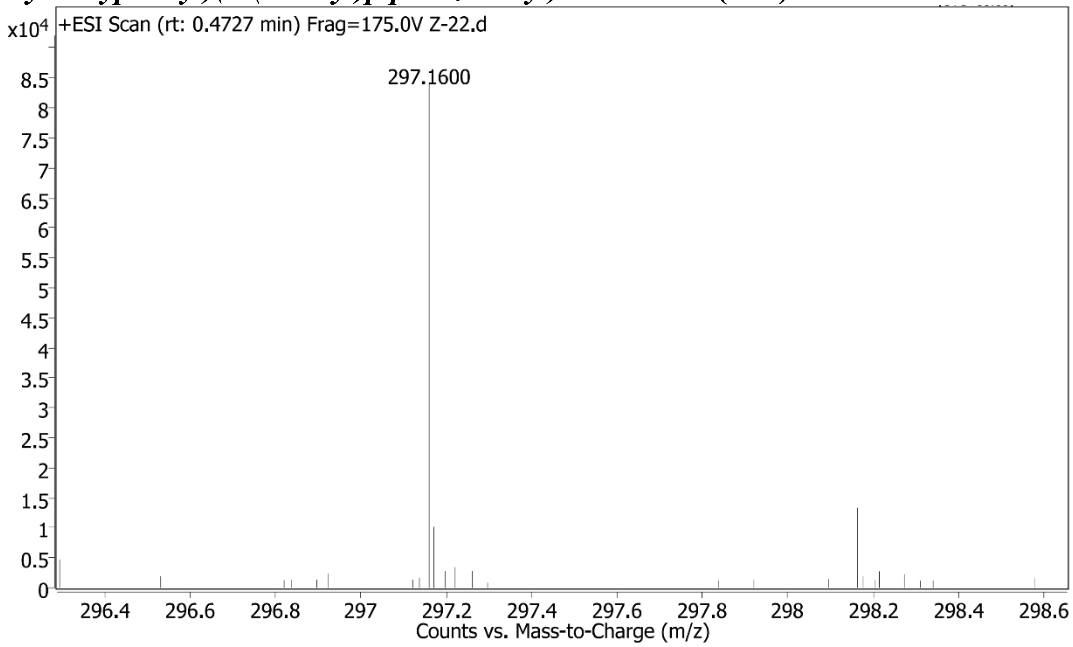

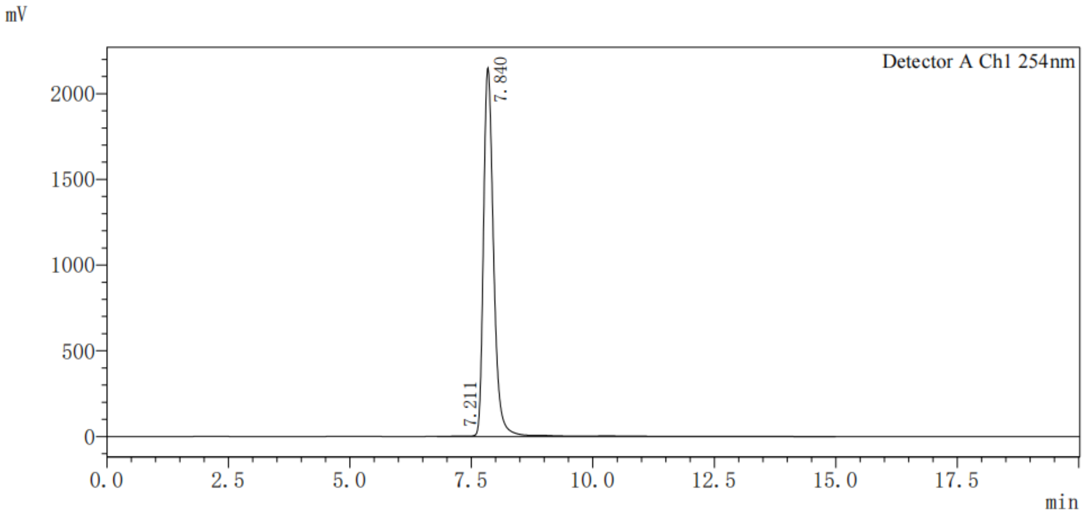

<Peak table>

Detector A Ch1 254nm

Number	Retention time	Peak area	Peak area%	Separation efficiency
1	7.211	71090	0.225	--
2	7.840	31564381	99.775	1.020
Total		31635471	100.000	

(USP)

(2-ethylphenyl)(4-(4-hydroxyphenyl)piperazin-1-yl)methanone **(AI4)**

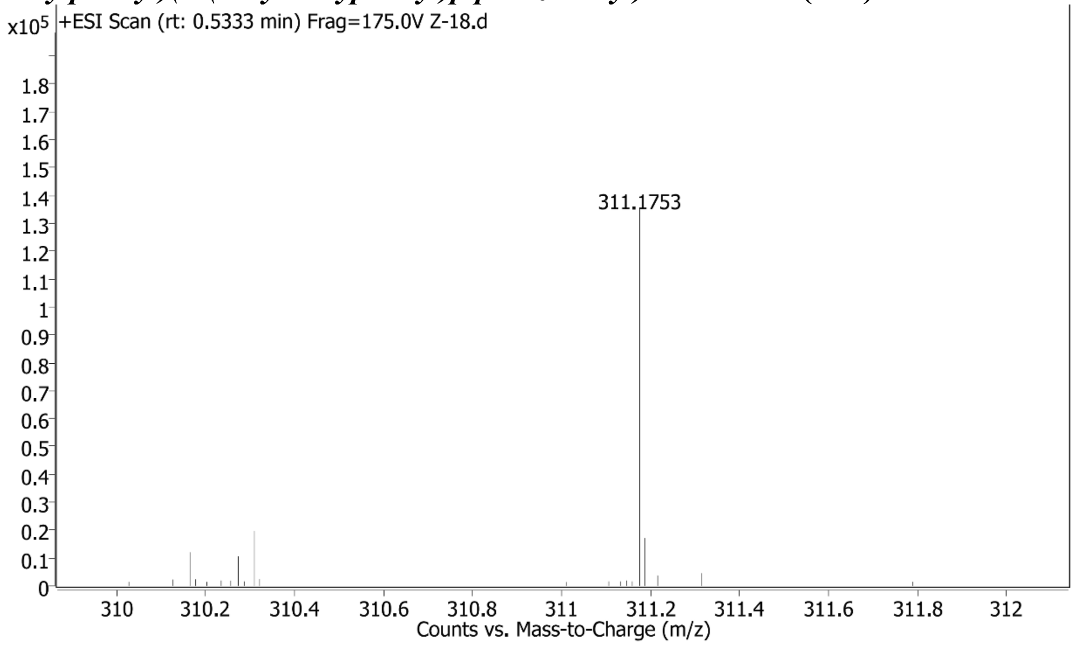

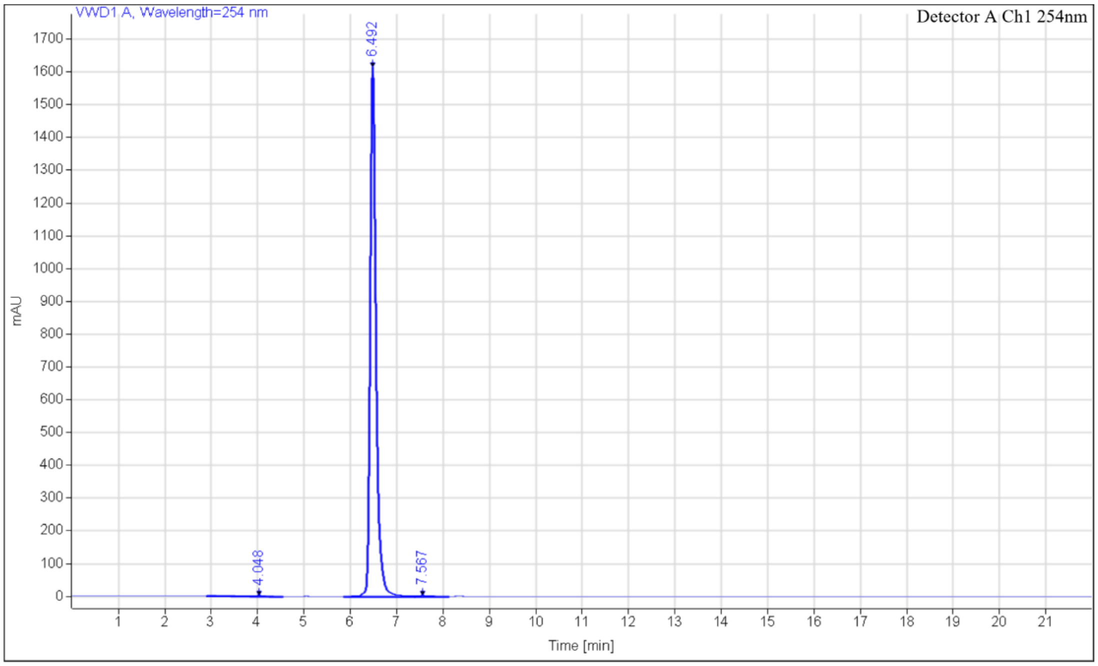

<Peak table>

Detector A Ch1 254nm

Number	Retention time	Peak area	Peak area%	Separation efficiency
1	4. 048	85. 01288	0. 59	--
2	6. 492	14326. 41211	99. 11	7. 08963
3	7. 567	43. 81482	0. 30	4. 26459
Total		14455. 23981	100. 00	

(USP)

(4-(4-chlorophenyl)piperazin-1-yl)(2-hydroxy-6-methylphenyl)methanone **(AI5)**

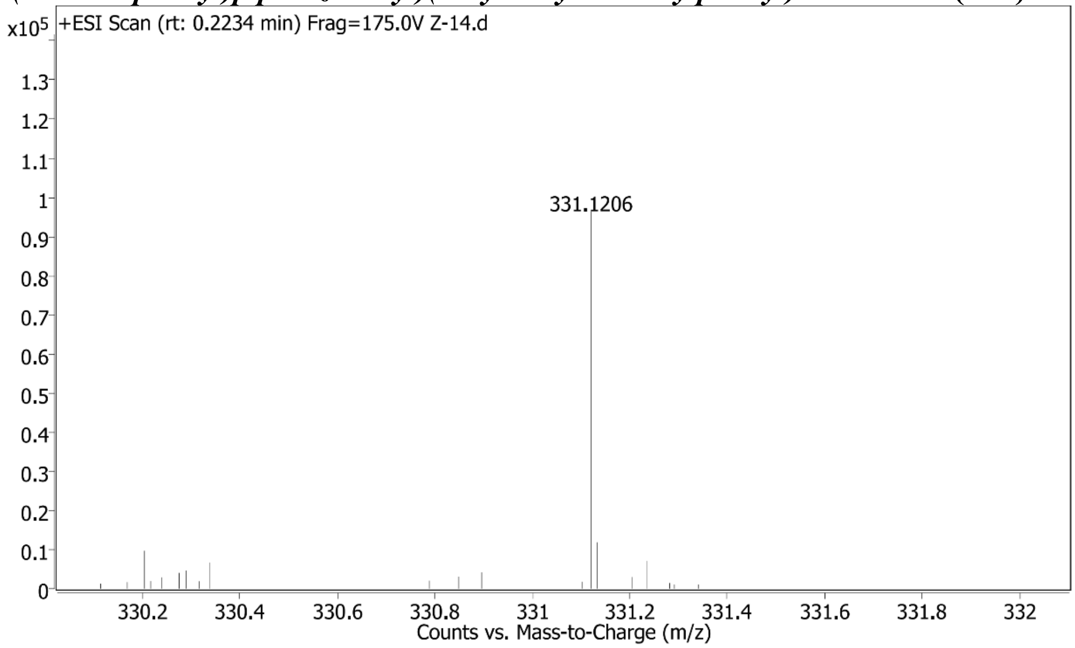

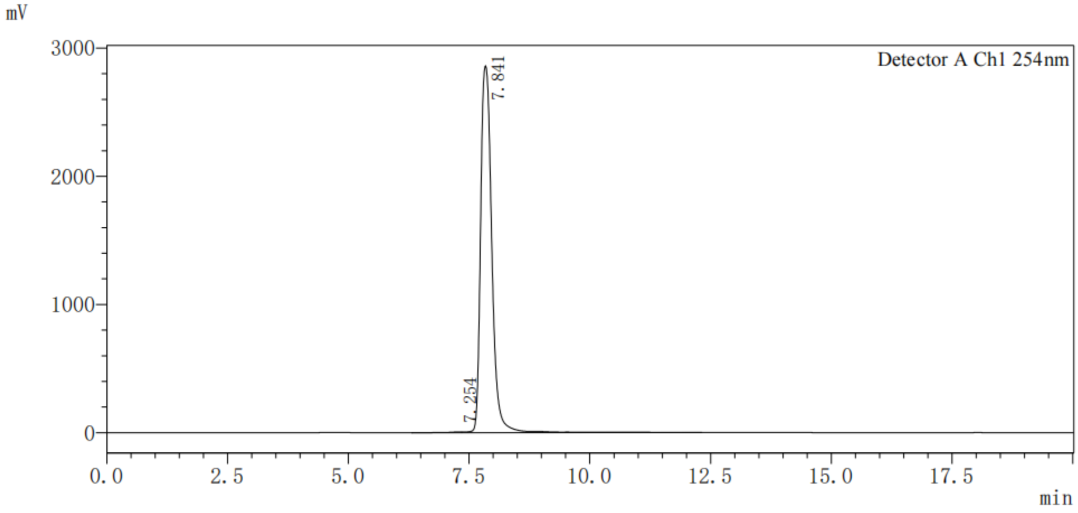

<Peak table>
Detector A Ch1 254nm

Number	Retention time	Peak area	Peak area%	Separation efficiency
1	7.254	89099	0.192	--
2	7.841	46232830	99.808	0.714
Total		46321929	100.000	

(USP)

***(4-(2-chlorophenyl)piperazin-1-yl)(2-hydroxy-5-methylphenyl)methanone* (AI6)**

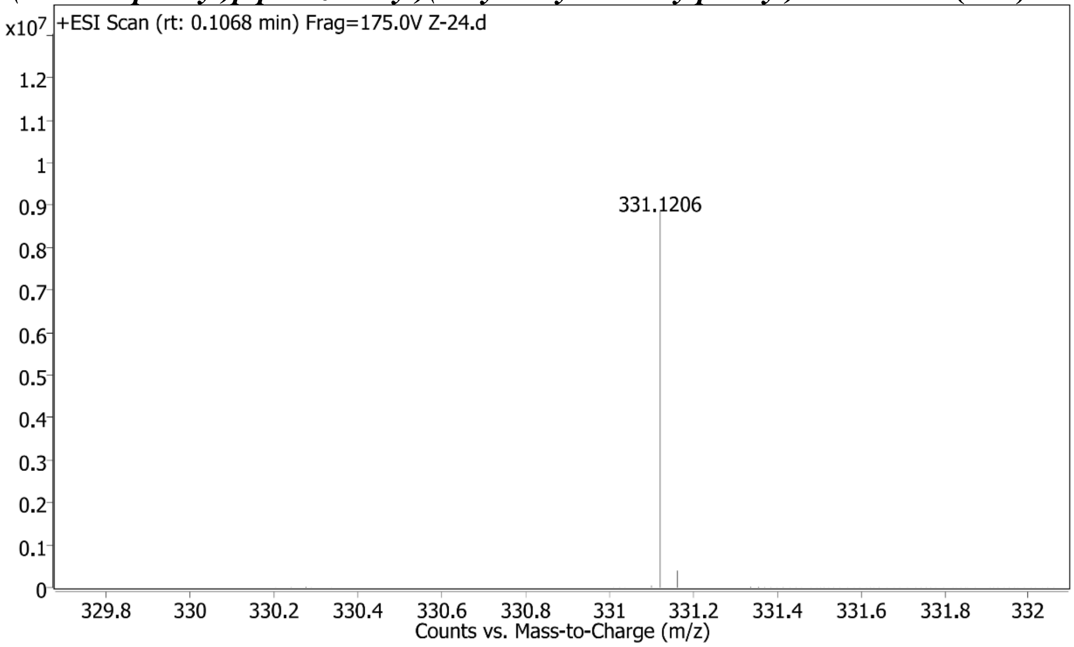

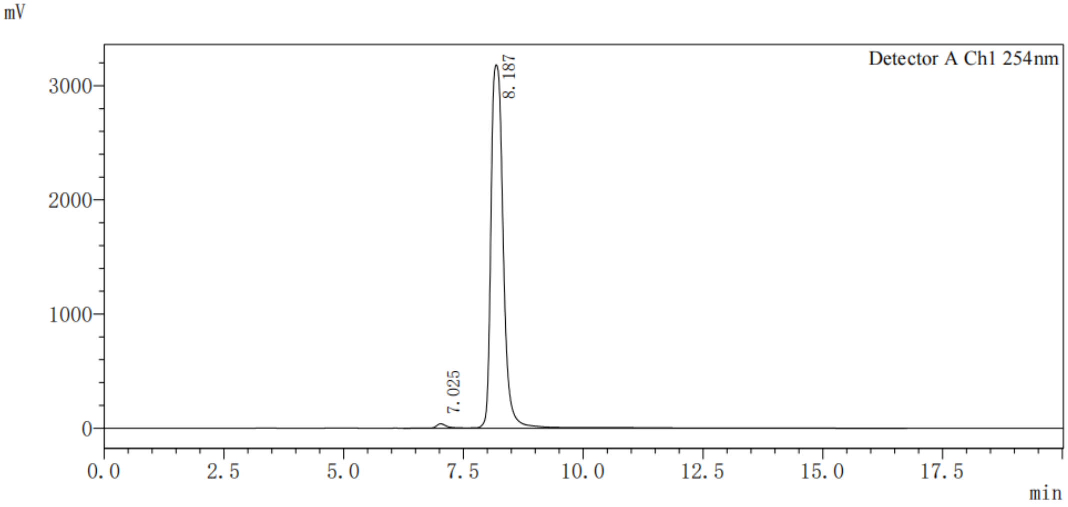

<Peak table>

Detector A Ch1 254nm

Number	Retention time	Peak area	Peak area%	Separation efficiency
1	7.025	603167	1.041	--
2	8.187	57333733	98.959	3.007
Total		57936900	100.000	

(USP)

(5-hydroxy-2-methylphenyl)(4-(4-methoxyphenyl)piperazin-1-yl)methanone **(AI7)**

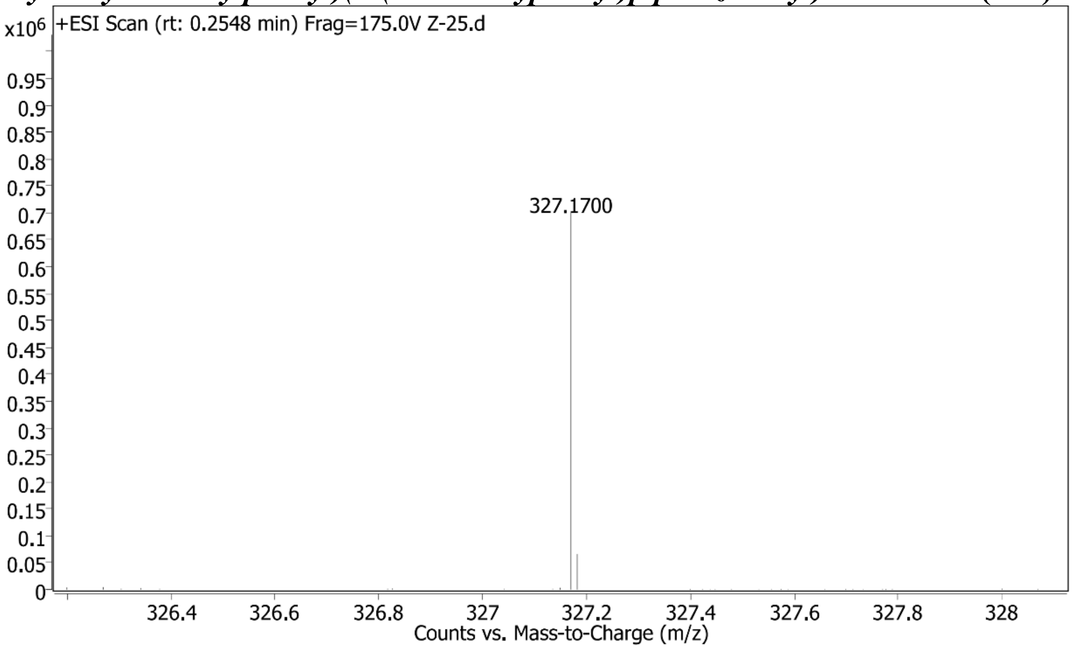

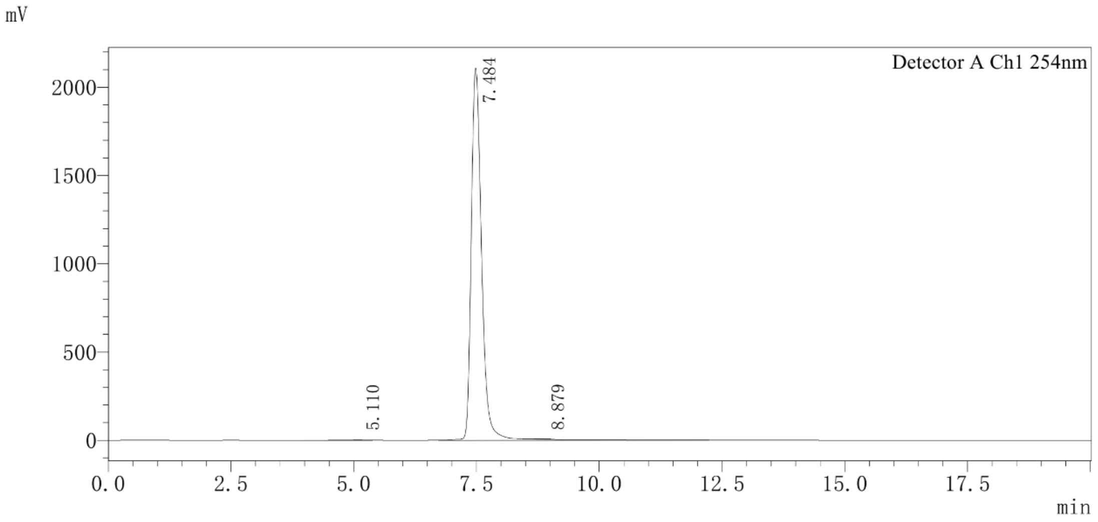

<Peak table>
Detector A Ch1 254nm

Number	Retention time	Peak area	Peak area%	Separation efficiency
1	5.110	57405	0.184	--
2	7.484	31152552	99.696	5.828
3	8.879	37544	0.120	3.322
Total		31247500	100.000	

(USP)

(3-hydroxy-2-methylphenyl)(4-phenethylpiperazin-1-yl)methanone **(AI8)**

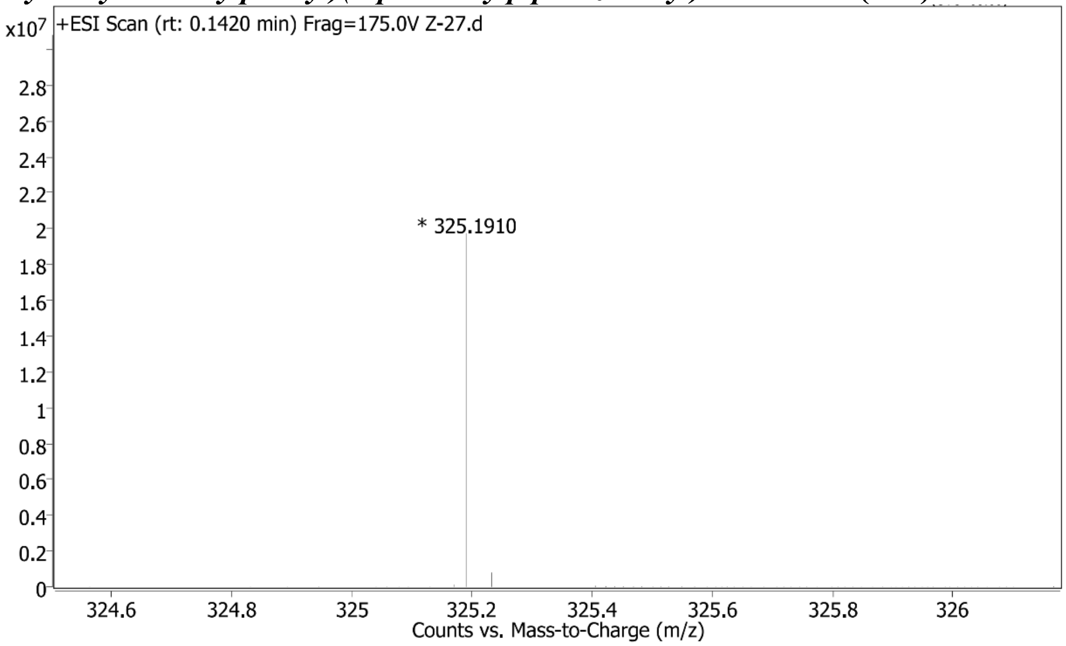

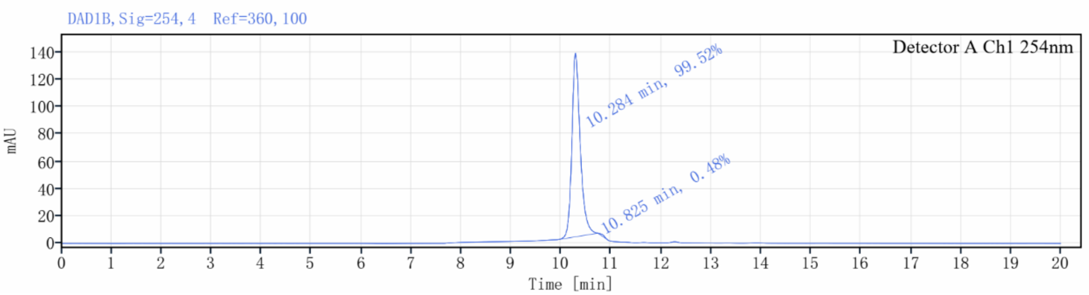

<Peak table>

Detector A Ch1 254nm

Number	Retention time	Peak area	Peak area%	Separation efficiency
1	10. 284	1573. 0	99. 52	--
2	10. 825	7. 6	0. 48	--
Total		1580. 6	100. 00	

(USP)

(4-(2-fluoro-4-methylbenzoyl)piperazin-1-yl)(4-hydroxyphenyl)methanone **(AI9)**

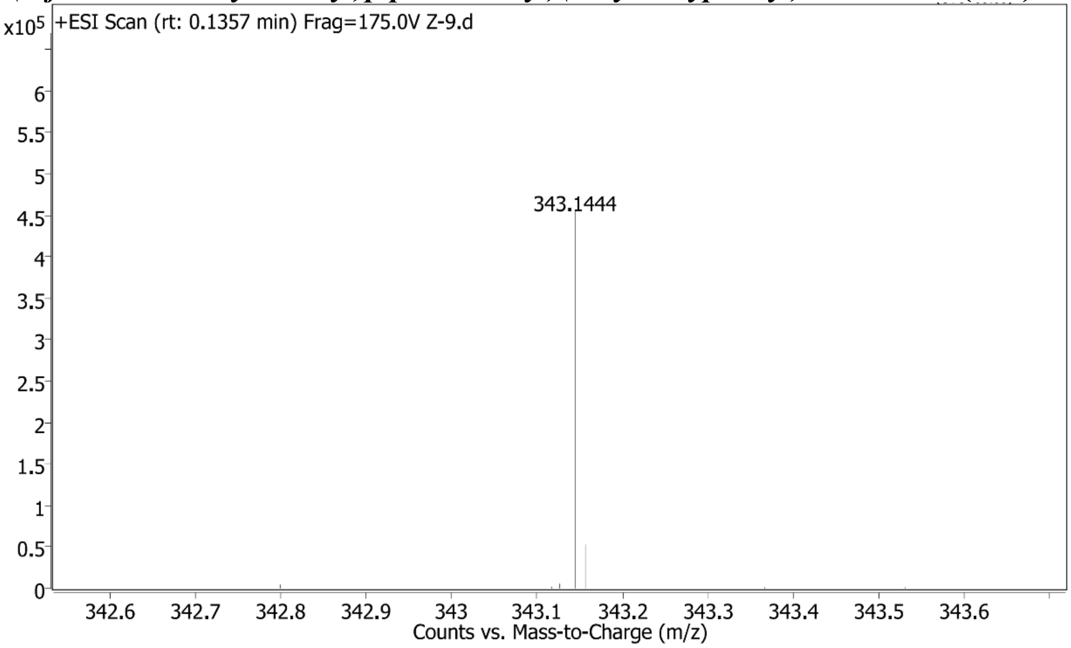

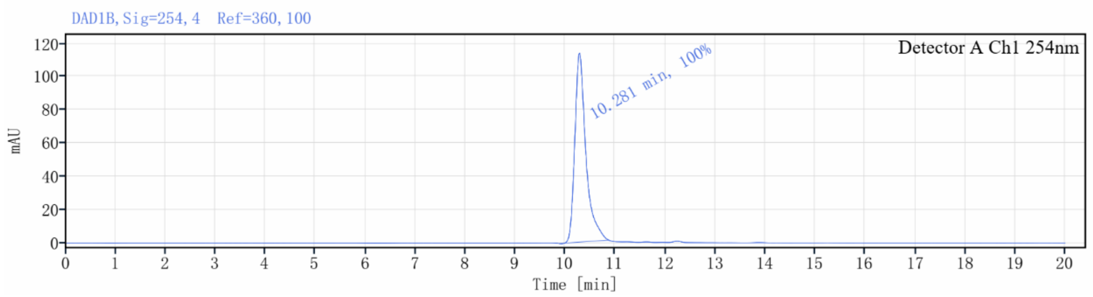

<Peak table>

Detector A Ch1 254nm

Number	Retention time	Peak area	Peak area%	Separation efficiency
1	10. 281	1754. 2	100. 00	--
Total		1754. 2	100. 00	

(USP)

***(2,6-dimethylpyridin-4-yl)(4-(4-hydroxyphenyl)piperazin-1-yl)methanone* (AI10)**

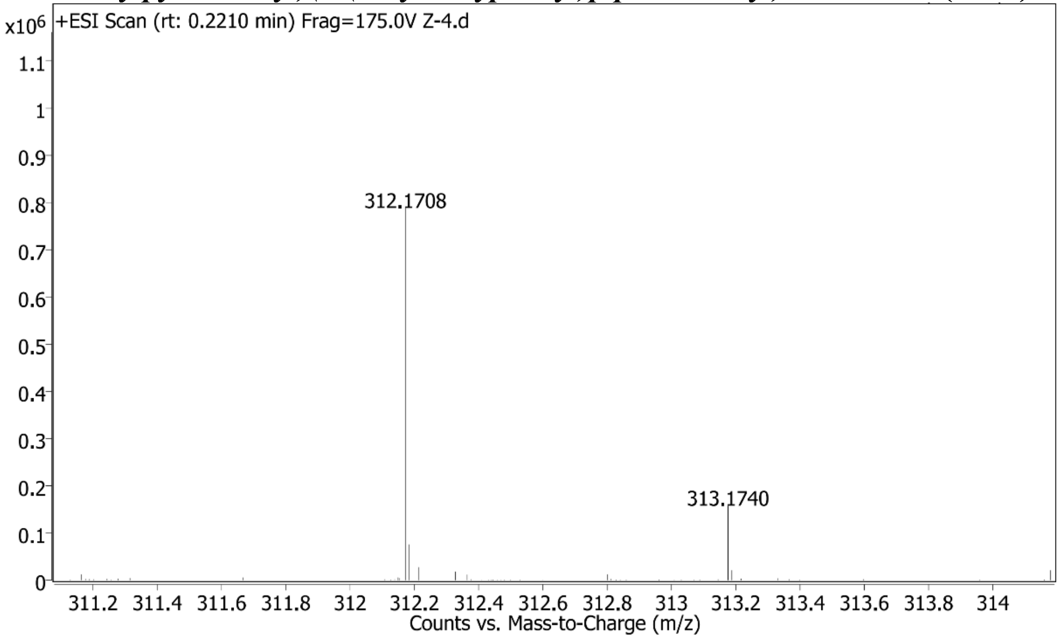

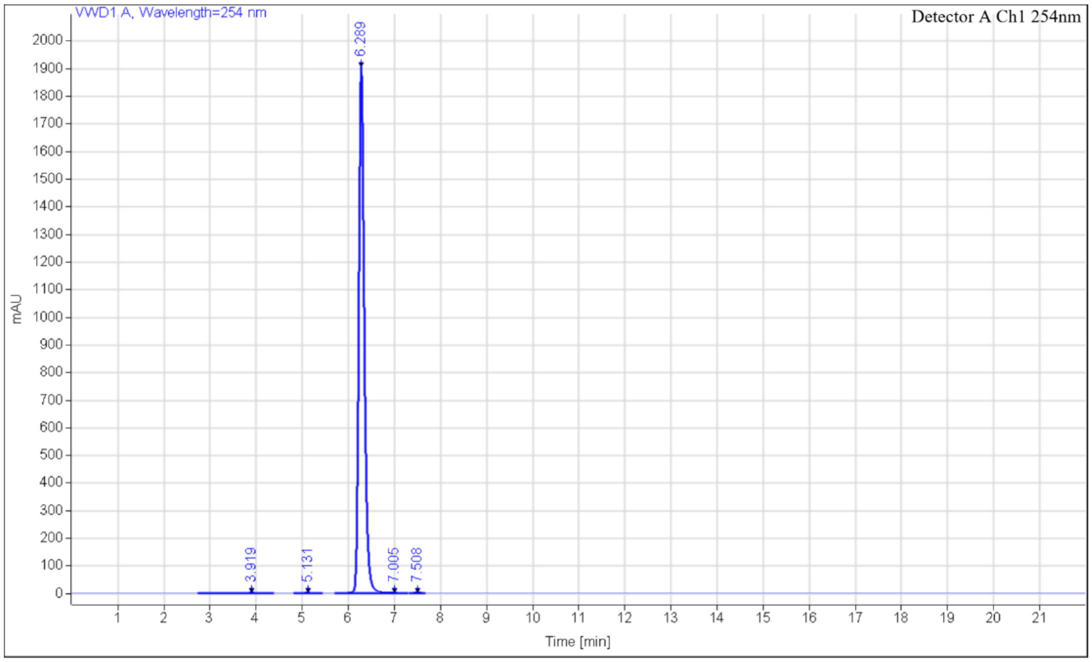

<Peak table>
Detector A Ch1 254nm

Number	Retention time	Peak area	Peak area%	Separation efficiency
1	3. 919	57. 16572	0. 36	--
2	5. 131	24. 94020	0. 16	3. 11913
3	6. 289	15851. 94434	99. 24	4. 77542
4	7. 005	16. 87517	0. 11	1. 49811
5	7. 508	21. 75615	0. 14	1. 03551
Total		15972. 68158	100. 00	

(USP)

(4-hydroxypyridin-2-yl)(4-(o-tolyl)piperazin-1-yl)methanone (AI11)

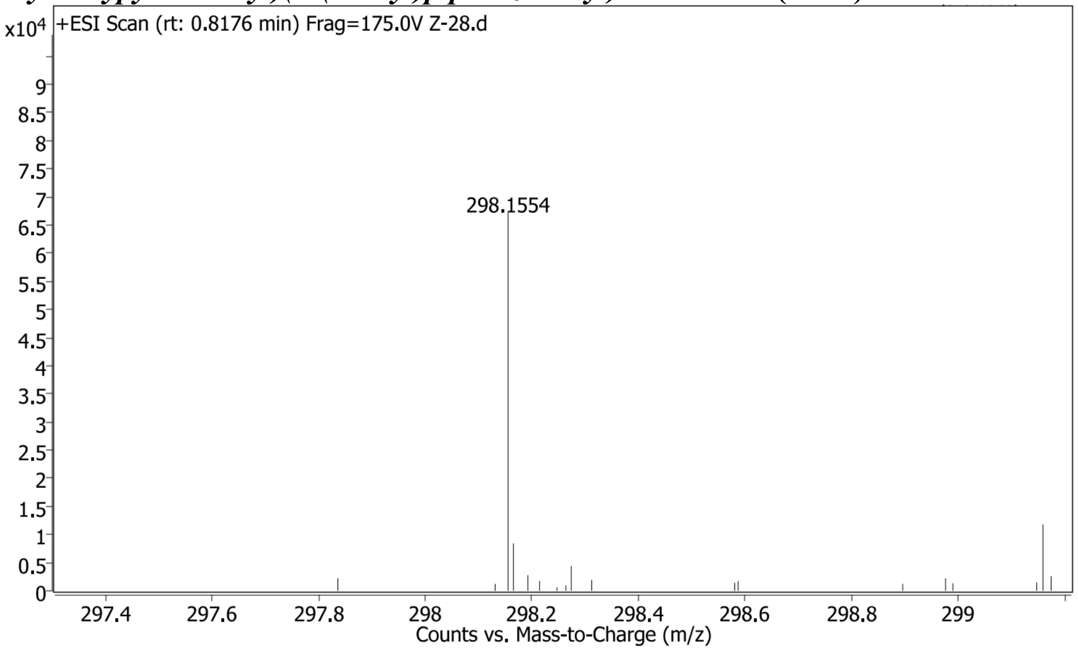

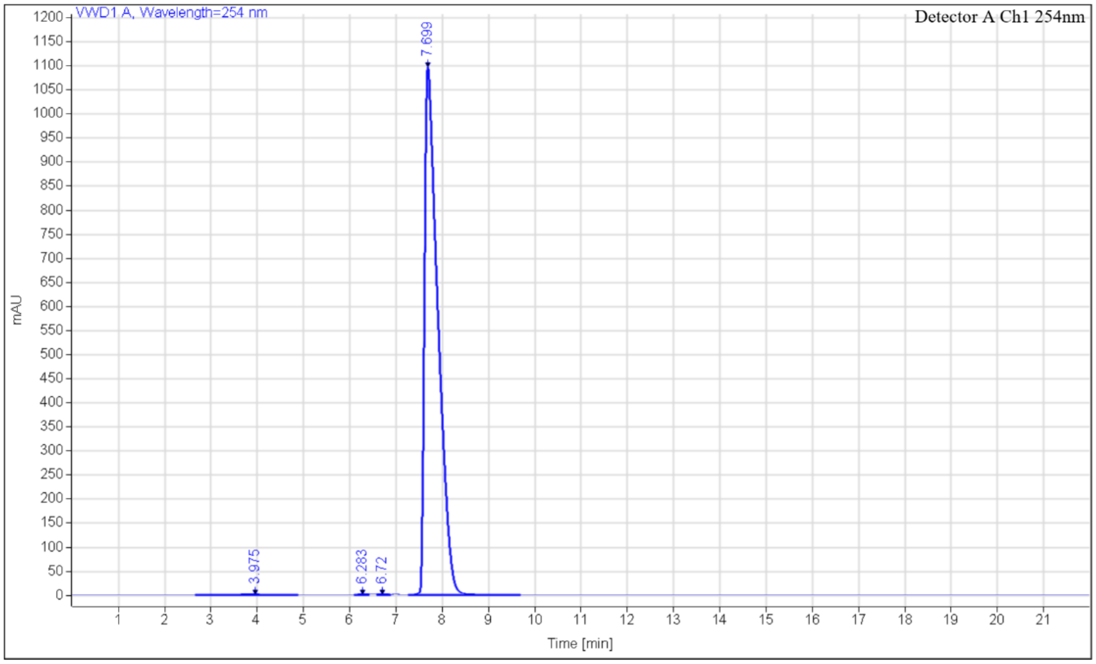

<Peak table>

Detector A Ch1 254nm

Number	Retention time	Peak area	Peak area%	Separation efficiency
1	3. 975	66. 00632	0. 30	--
2	6. 283	18. 60034	0. 08	6. 03863
3	6. 720	24. 94047	0. 11	1. 62732
4	7. 699	21912. 76953	99. 50	2. 30456
Total		22022. 31666	100. 00	

(USP)

2-(4-hydroxyphenyl)-1-(4-(4-methylbenzyl)piperazin-1-yl)ethan-1-one **(AI12)**

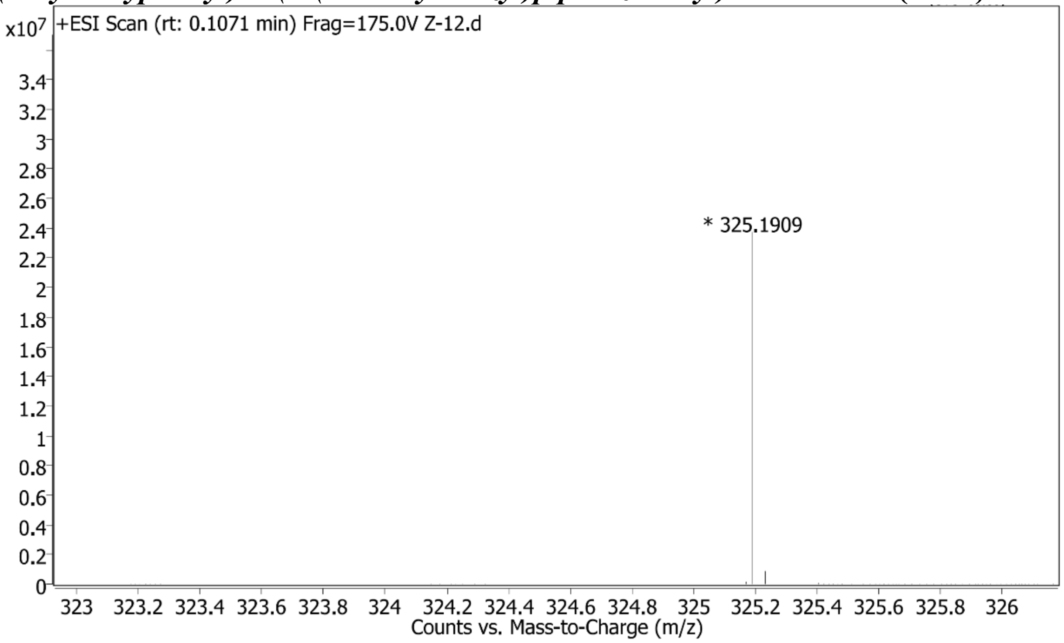

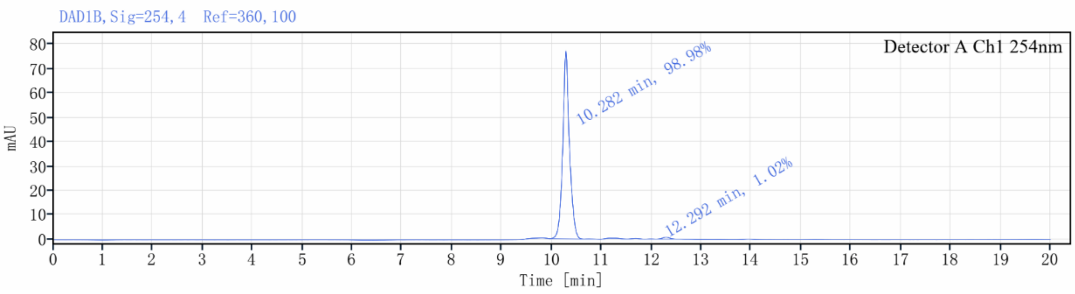

<Peak table>

Detector A Ch1 254nm

Number	Retention time	Peak area	Peak area%	Separation efficiency
1	10.282	726.4	98.98	--
2	12.292	7.5	1.02	--
Total		733.9	100.00	

(USP)

2-(2-(4-(4-hydroxyphenyl)piperazin-1-yl)ethyl)phenol **(AI13)**

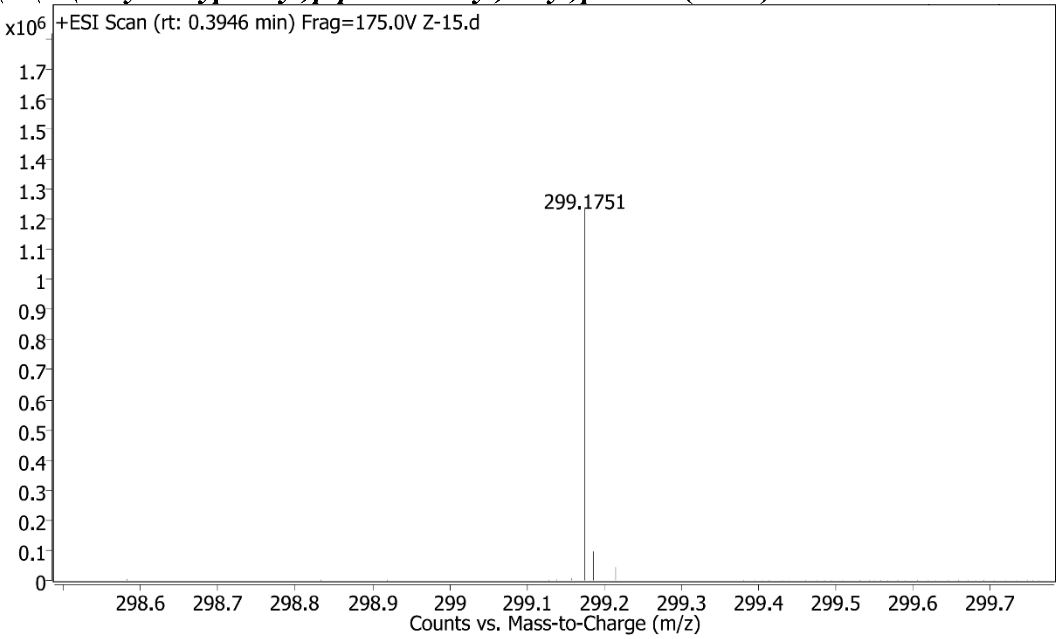

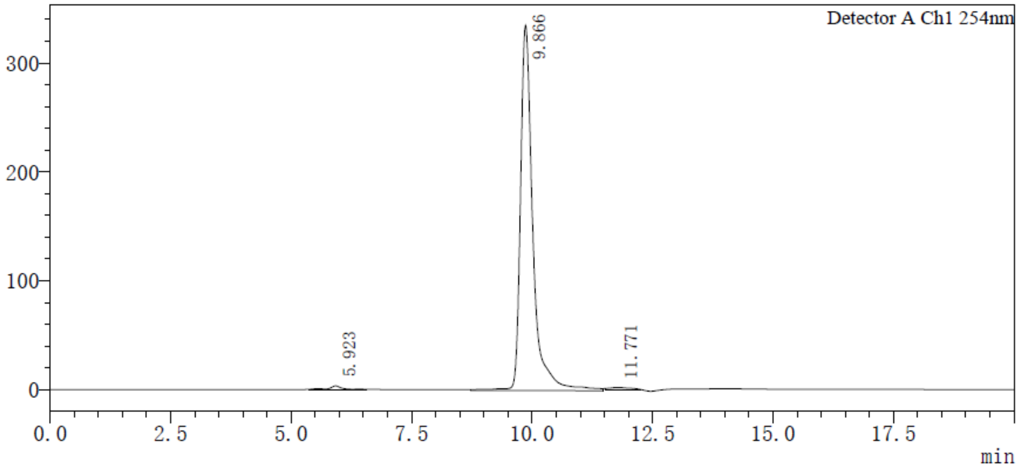

<Peak table>

Detector A Ch1 254nm

Number	Retention time	Peak area	Peakarea%	Separation efficiency
1	5. 923	51862	0. 852	--
2	9. 866	5985198	98. 338	10. 203
3	11. 771	49287	0. 810	3. 040
Total		6086346	100. 000	

(USP)

***(4-(4-hydroxyphenyl)piperazin-1-yl)(1-methyl-1H-indol-3-yl)methanone* (AI14)**

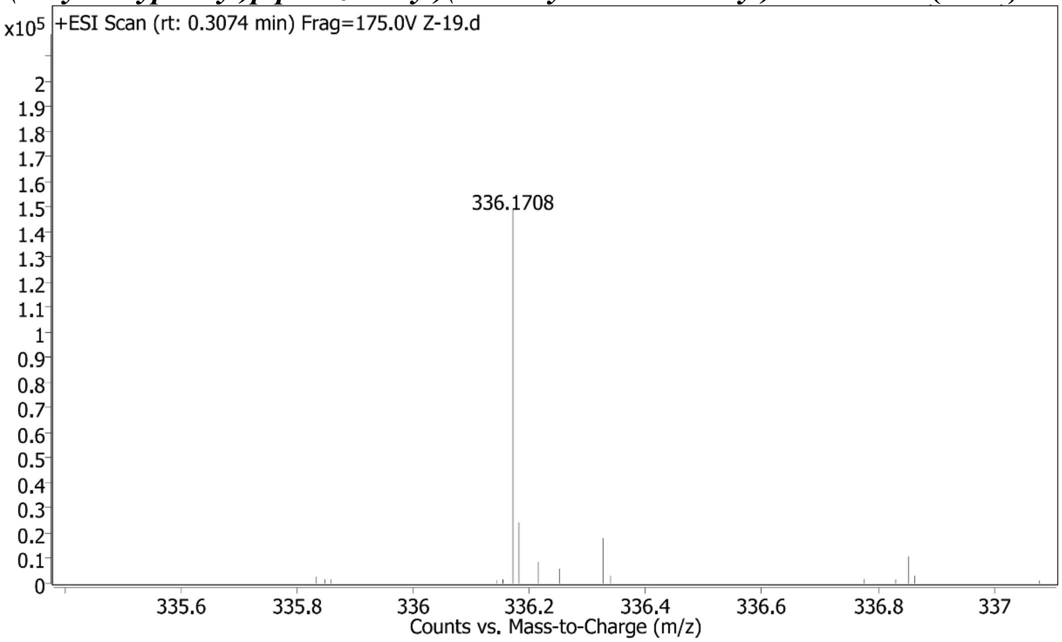

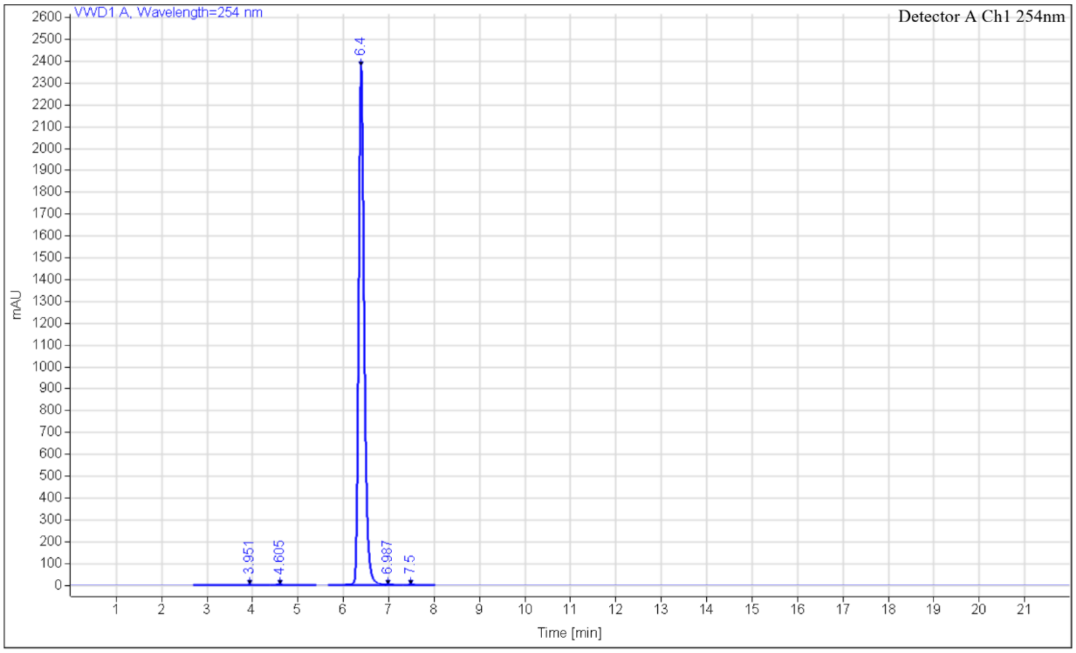

<Peak table>

Detector A Ch1 254nm

Number	Retention time	Peak area	Peak area%	Separation efficiency
1	3.951	72.01163	0.35	--
2	4.605	43.26950	0.21	2.03410
3	6.400	20242.78125	99.00	8.11420
4	6.987	47.04013	0.23	0.68735
5	7.500	42.36061	0.21	0.57609
Total		20447.46312	100.00	

(USP)

***(4-(3-hydroxyphenyl)piperazin-1-yl)(2-methyl-1H-benzo[d]imidazol-6-yl)methanone* (AI15)**

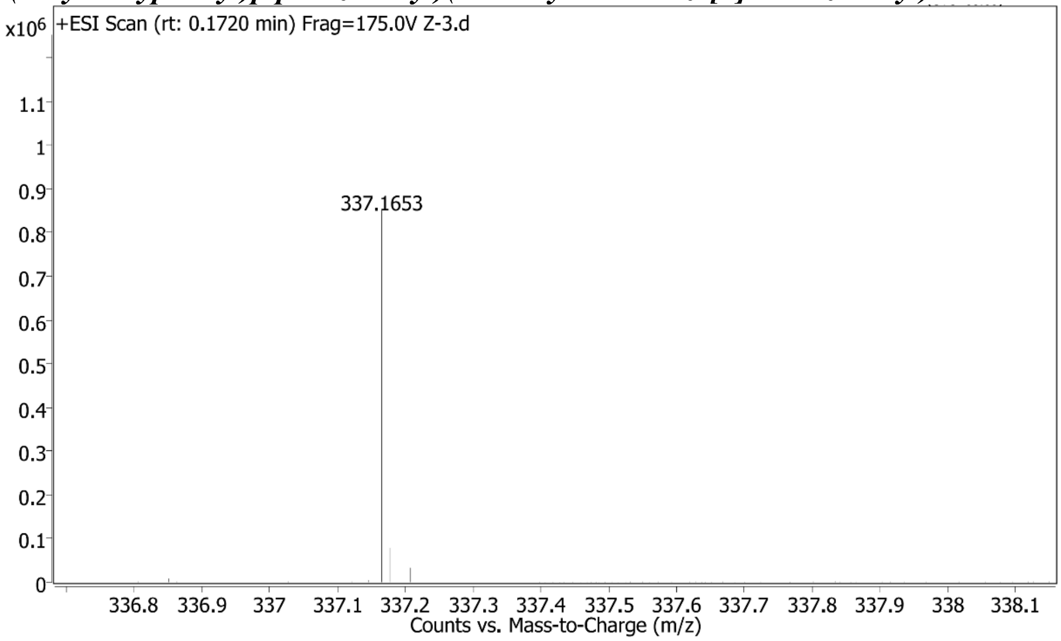

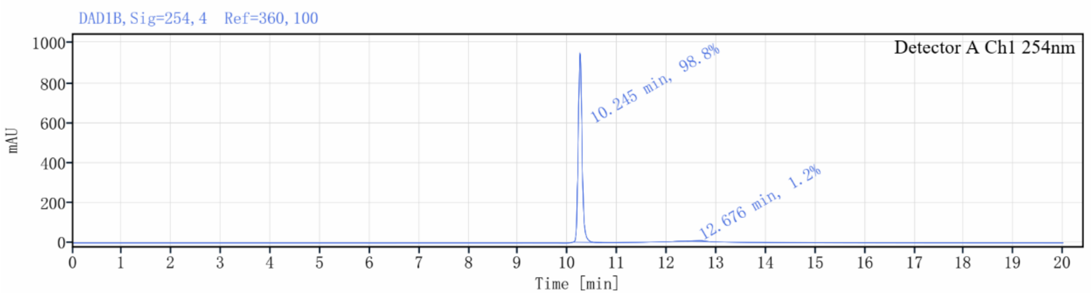

<Peak table>

Detector A Ch1 254nm

Number	Retention time	Peak area	Peak area%	Separation efficiency
1	10. 245	5142. 7	98. 80	--
2	12. 676	62. 7	1. 20	--
Total		5205. 4	100. 00	

(USP)

(4-(4-hydroxyphenyl)piperidin-1-yl)(m-tolyl)methanone **(AI16)**

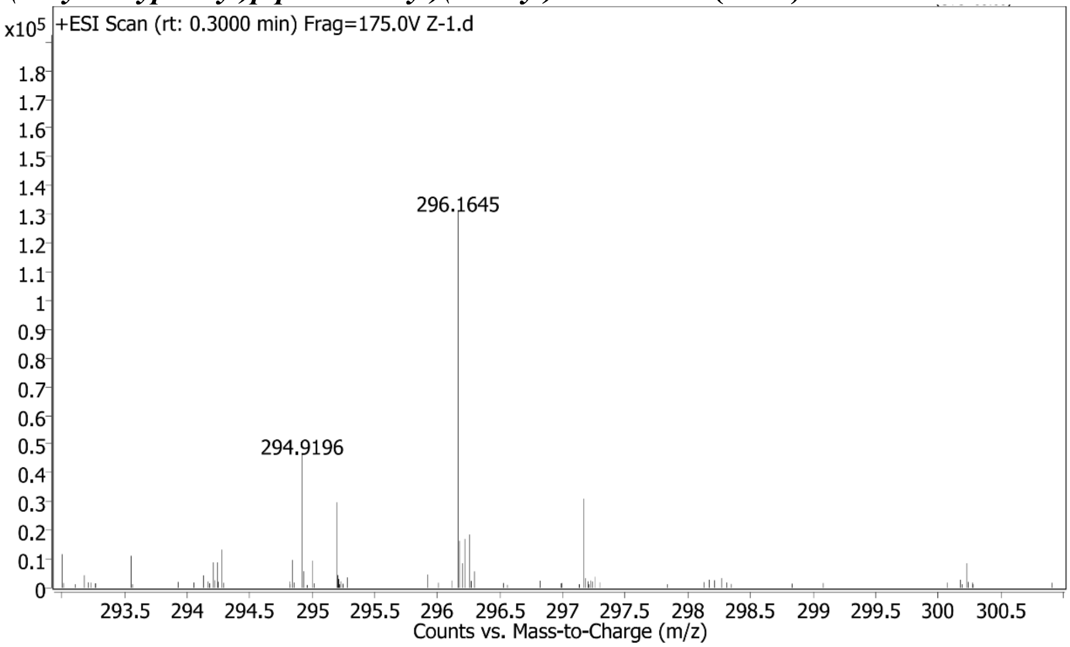

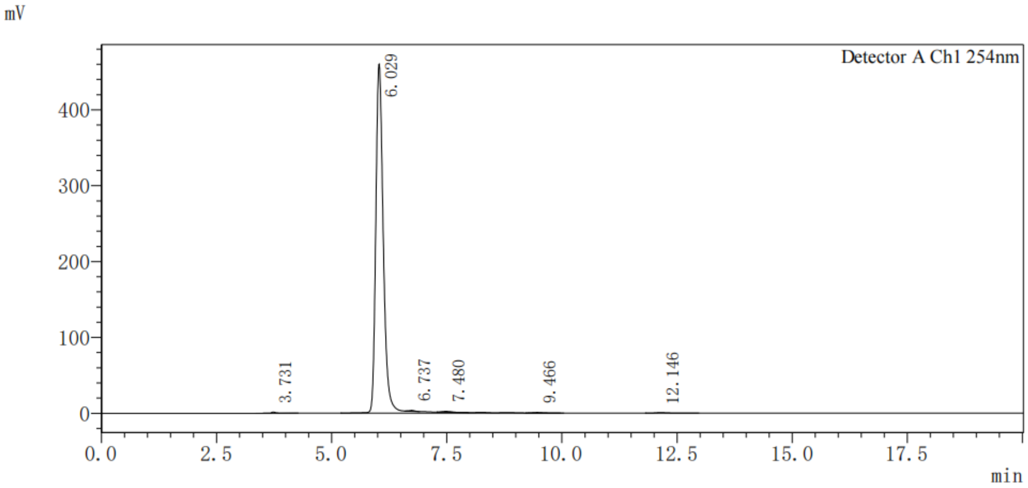

<Peak table>

Detector A Ch1 254nm

Number	Retention time	Peak area	peak area%	Separation efficiency
1	3.731	13939	0.256	--
2	6.029	5382012	98.804	9.772
3	6.737	8621	0.158	2.713
4	7.480	15864	0.291	2.436
5	9.466	11063	0.203	5.136
6	12.146	15636	0.287	5.987
Total		5447135	100.000	

(USP)

***1-(3-chlorobenzoyl)-N-(4-methoxybenzyl)piperidine-4-carboxamide* (AI17)**

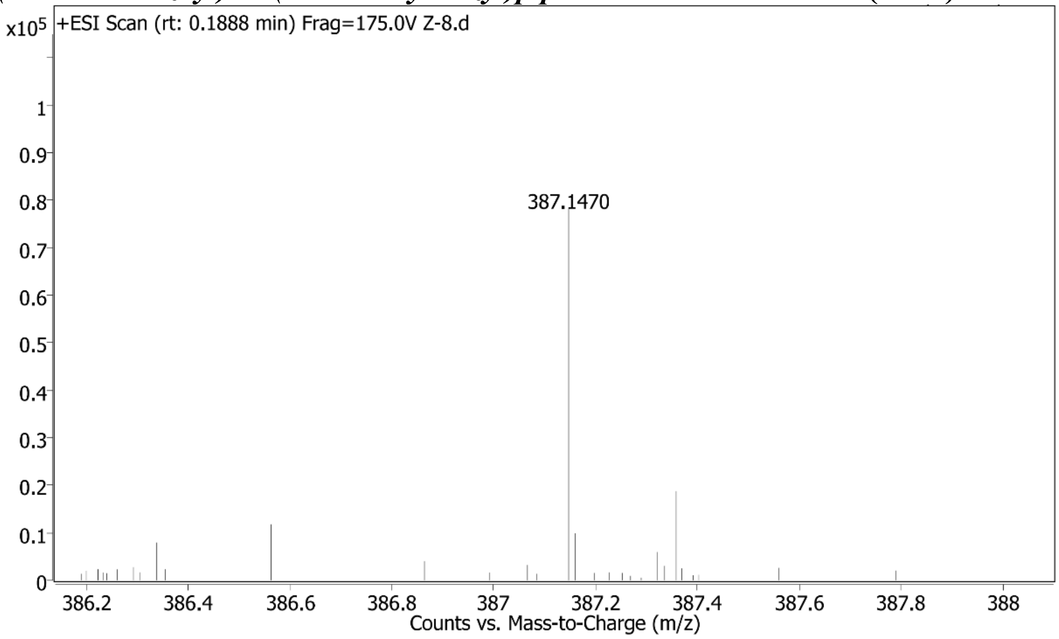

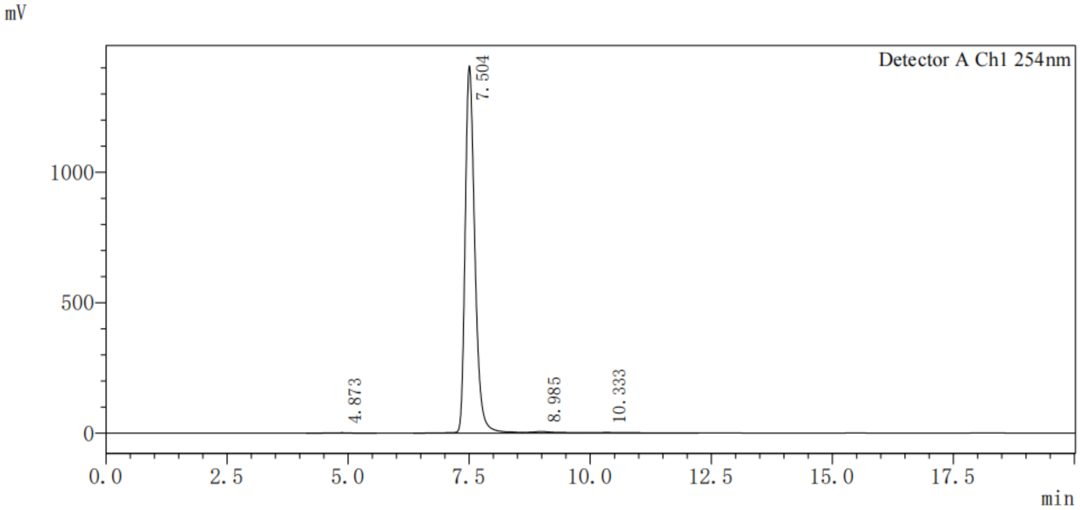

<Peak table>

Detector A Ch1 254nm

Number	Retention time	Peak area	Peak area%	Separation efficiency
1	4. 873	21747	0. 109	--
2	7. 504	19611344	97. 885	8. 563
3	8. 985	261158	1. 303	2. 389
4	10. 333	140935	0. 703	0. 778
Total		20035185	100. 000	

(USP)

***(3-hydroxy-4-methylphenyl)(4-phenylpiperidin-1-yl)methanone* (AI18)**

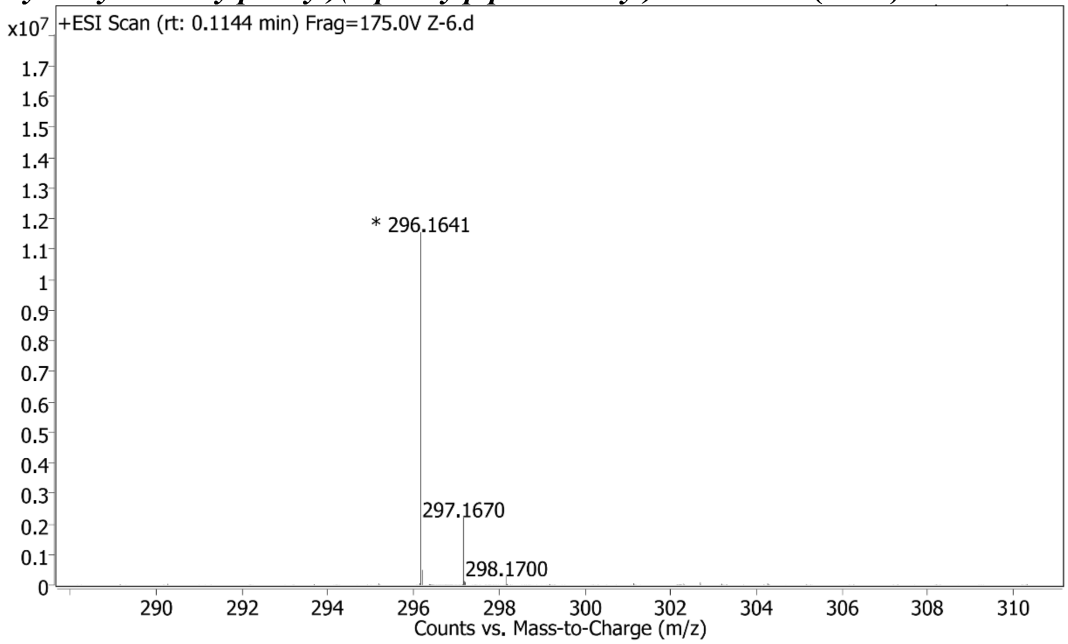

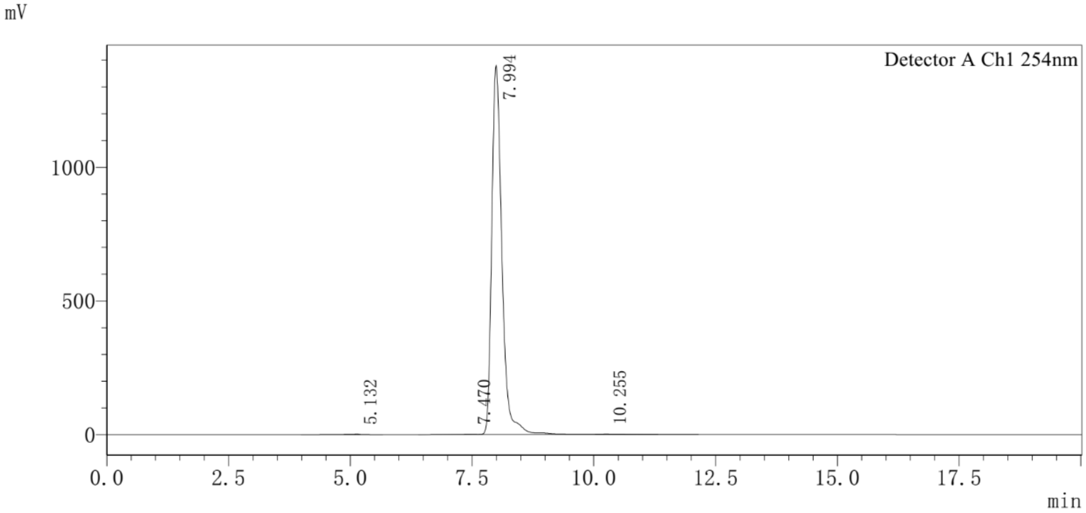

<Peak table>

Detector A Ch1 254nm

Number	Retention time	Peak area	Peak area%	Separation efficiency
1	5.132	55411	0.273	--
2	7.470	40093	0.197	3.826
3	7.994	20212164	99.401	0.819
4	10.255	26322	0.129	4.250
Total		20333991	100.000	

(USP)

4-chloro-N-(4-(dimethylamino)benzyl)-3-hydroxybenzamide **(AI19)**

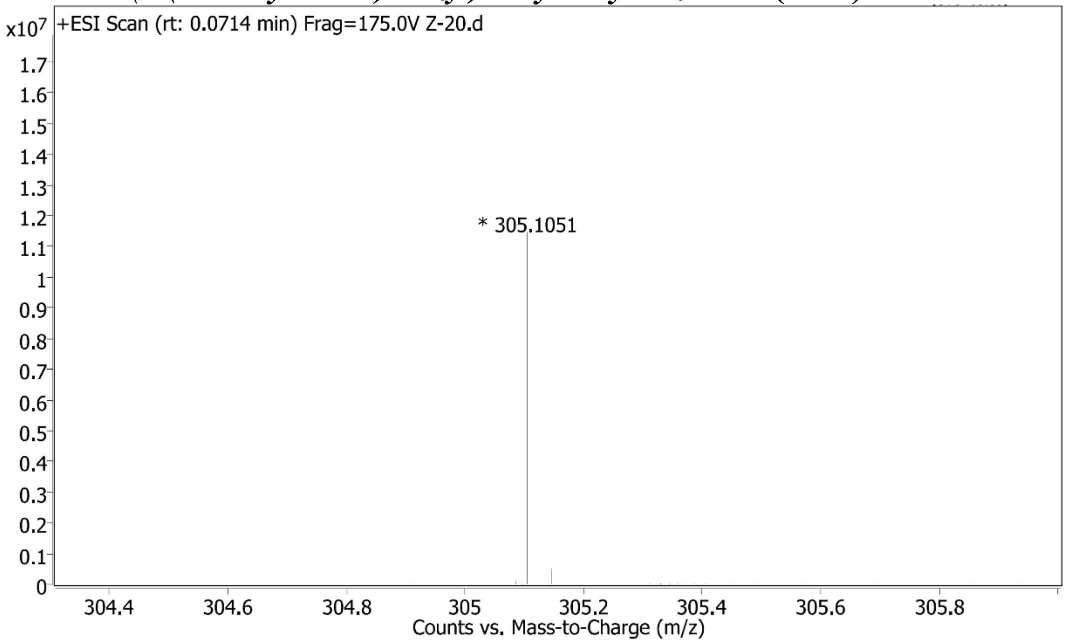

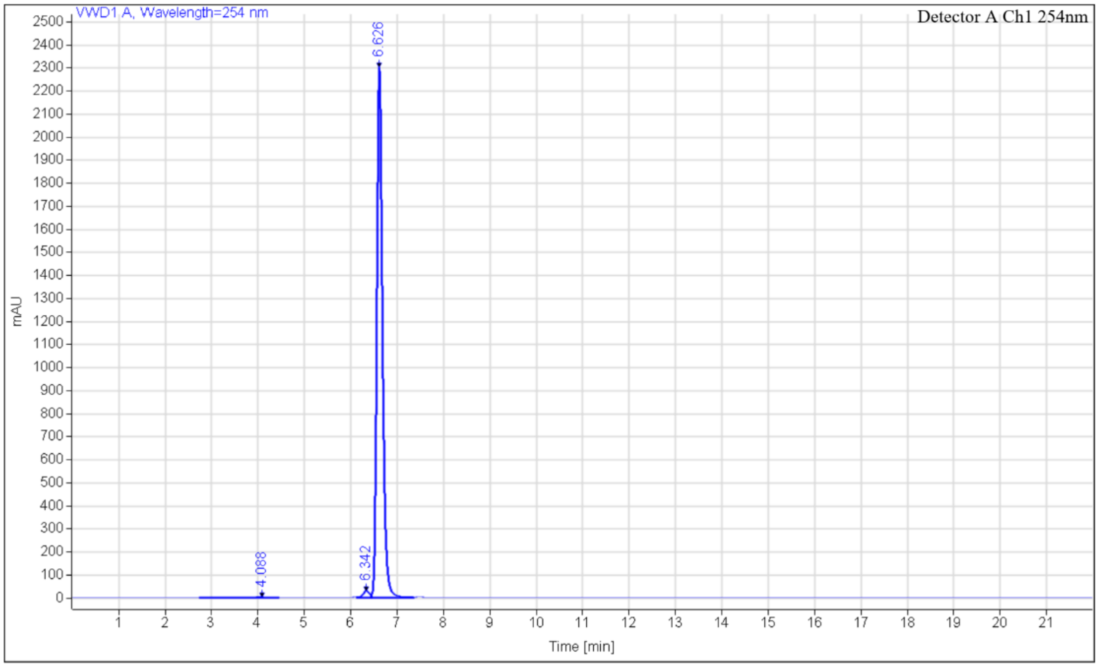

<Peak table>

Detector A Ch1 254nm

Number	Retention time	Peak area	Peak area%	Separation efficiency
1	4. 088	84. 65752	0. 42	--
2	6. 342	275. 83286	1. 38	7. 93806
3	6. 626	19578. 02930	98. 19	1. 20030
Total		19938. 51968	100. 00	

(USP)

N-(2,5-dimethylbenzyl)-2-hydroxybenzamide **(AI20)**

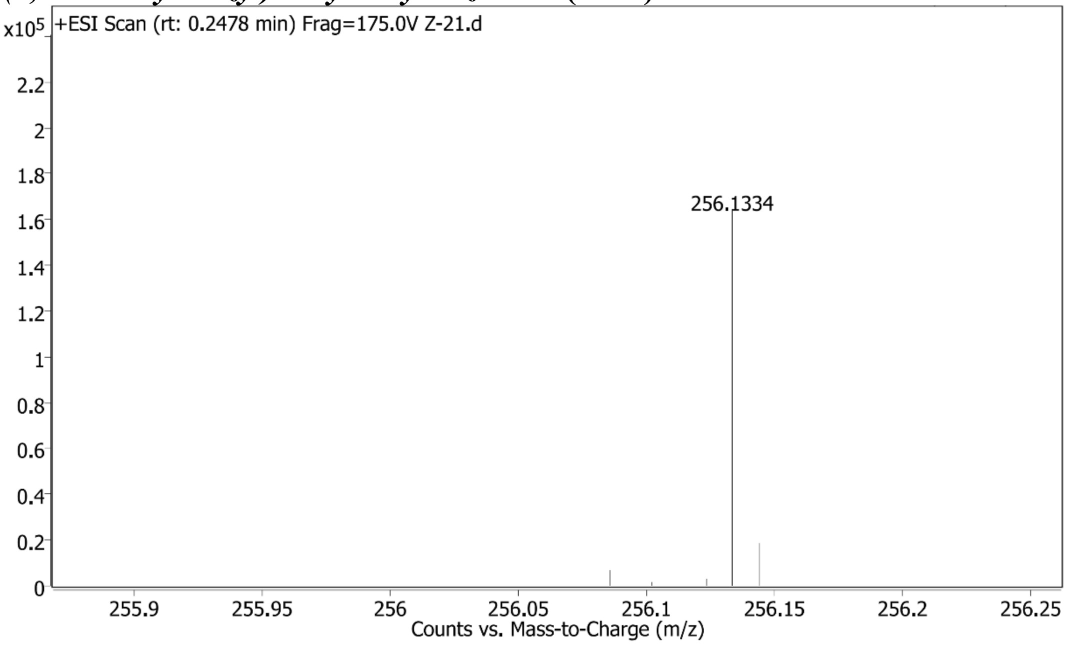

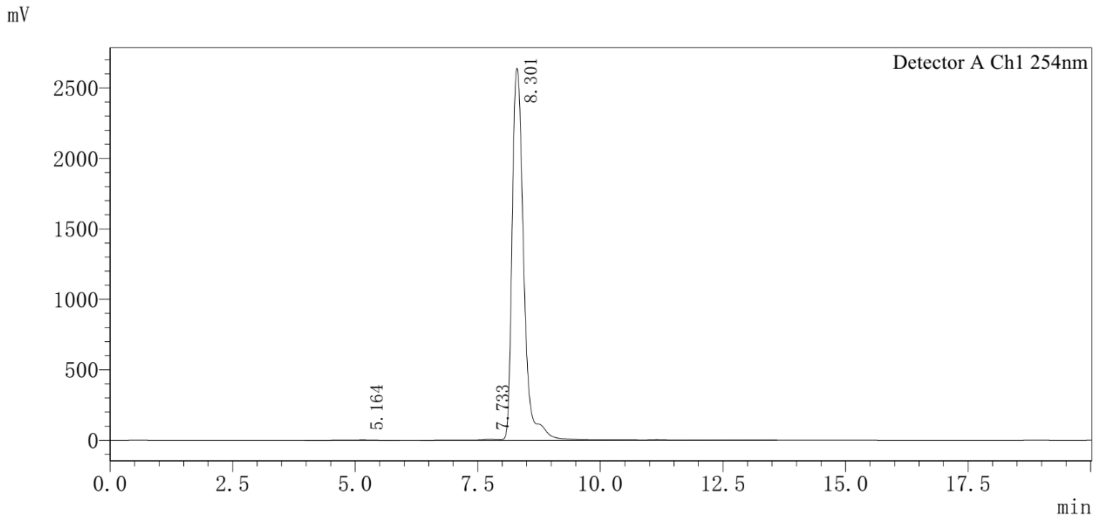

<Peak table>

Detector A Ch1 254nm

Number	Retention time	Peak area	Peak area%	Separation efficiency
1	5.164	68357	0.155	--
2	7.733	181786	0.413	5.013
3	8.301	43753059	99.432	1.087
Total		44003203	100.000	

(USP)

N-(4-chlorobenzyl)-4-hydroxy-3-methylbenzamide **(AI21)**

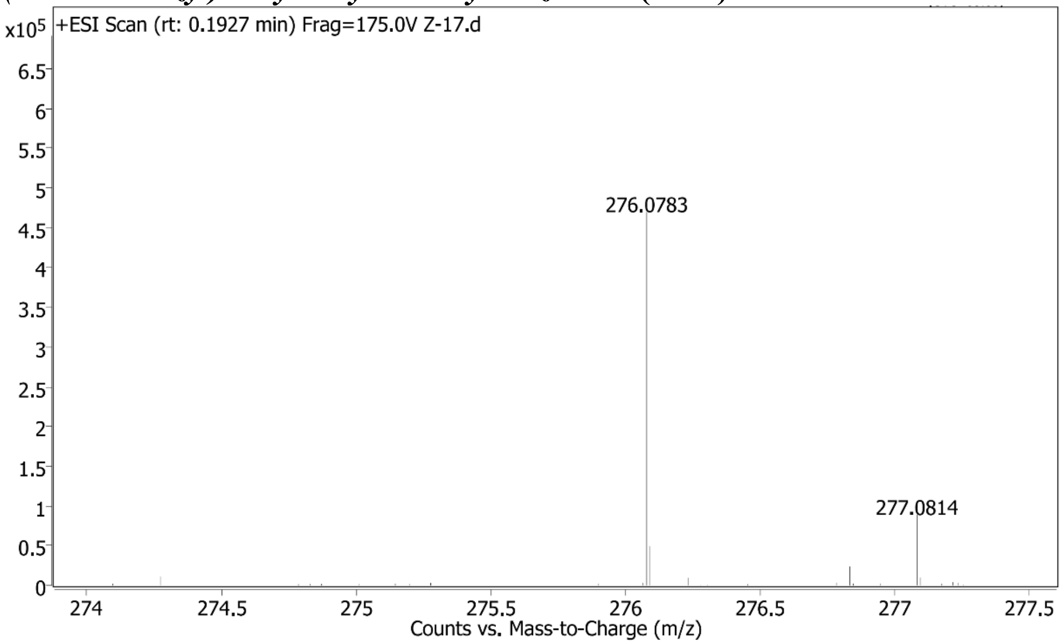

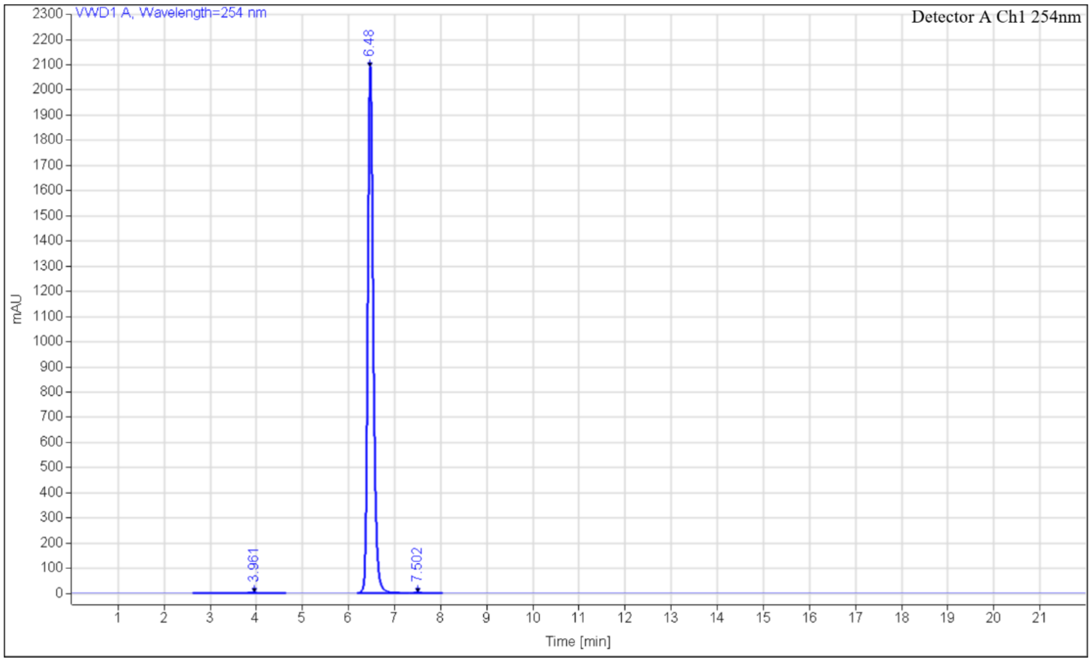

<Peak table>
Detector A Ch1 254nm

Number	Retention time	Peak area	Peak area%	Separation efficiency
1	3. 961	79. 33712	0. 45	--
2	6. 480	17544. 47070	99. 30	6. 98109
3	7. 502	45. 01069	0. 25	4. 10526
Total		17668. 81851	100. 00	

(USP)

N-(2-hydroxybenzyl)-2-(4-(4-methylbenzyl)piperazin-1-yl)isonicotinamide **(AI22)**

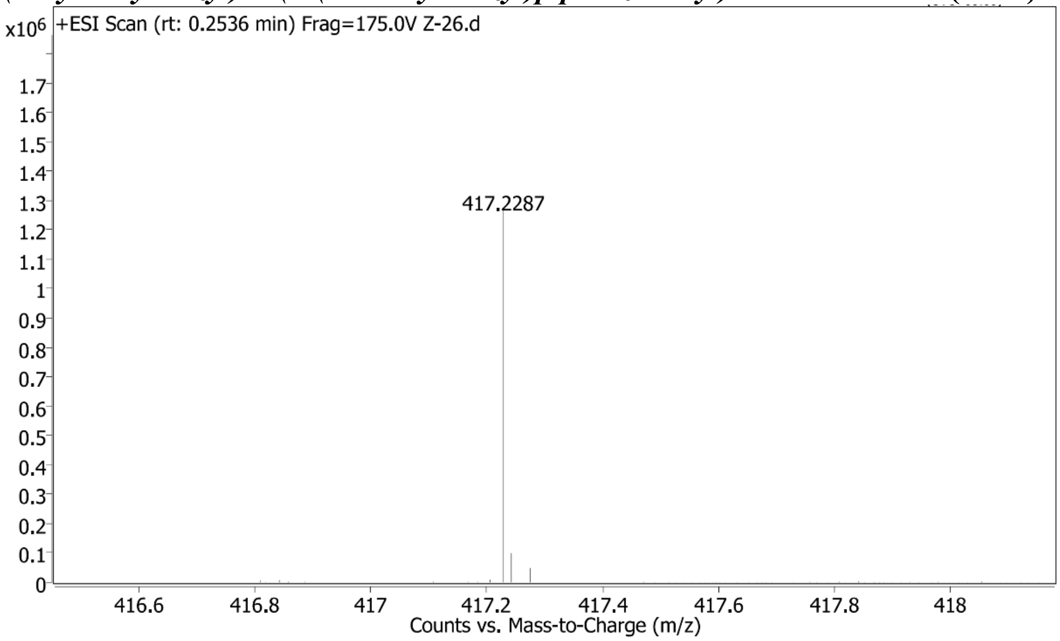

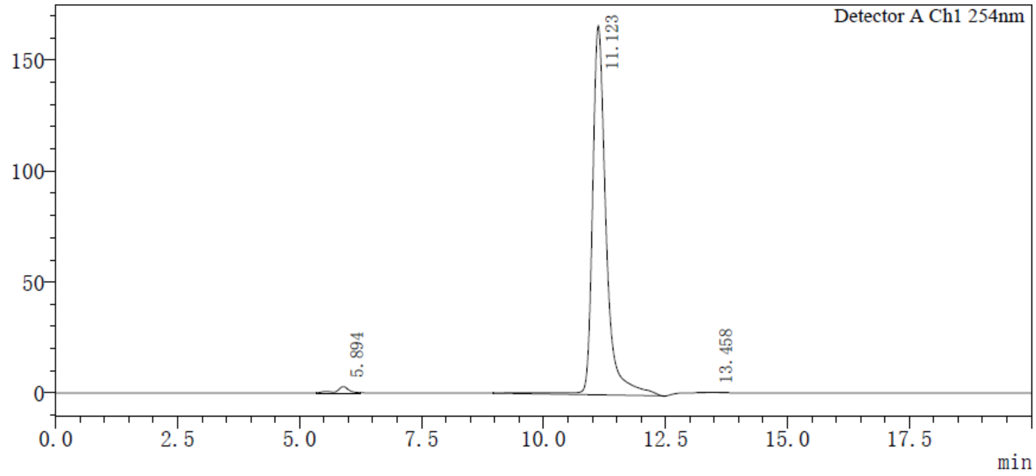

<Peak table>

Detector A Ch1 254nm

Number	Retention time	Peak area	Peakarea%	Separation efficiency
1	5.894	61411	1.861	--
2	11.123	3233803	98.003	12.359
3	13.458	4490	0.136	3.920
Total		3299705	100.000	

(USP)

(E)-3-(3,4-dihydroxyphenyl)-N-(4-fluorophenyl)acrylamide **(AI23)**

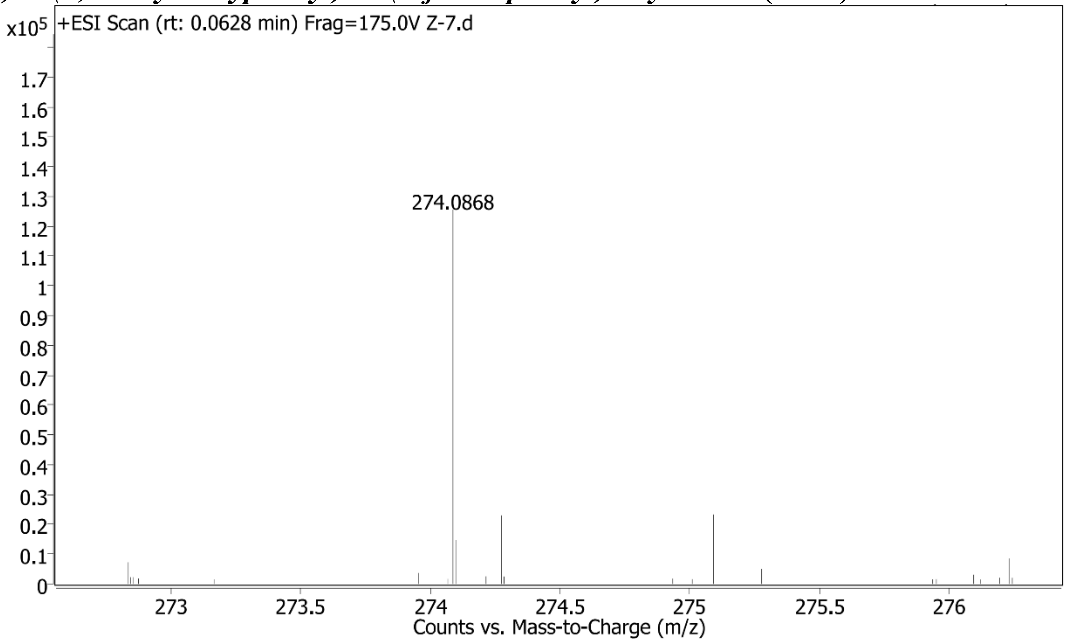

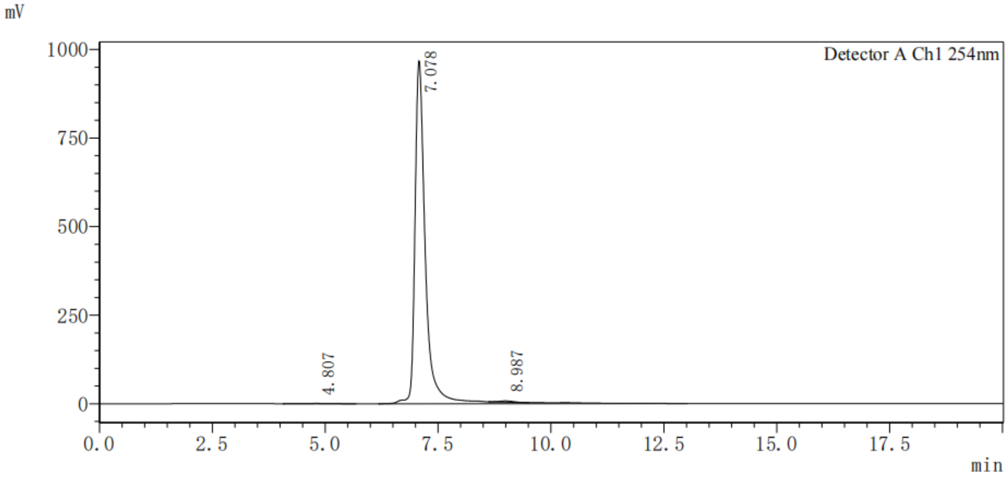

<Peak table>

Detector A Ch1 254nm

Number	Retention time	Peak area	Peak area%	Separation efficiency
1	4.807	23035	0.145	--
2	7.078	15817806	99.437	7.141
3	8.987	66502	0.418	4.238
Total		15907343	100.000	

(USP)

N-(2-hydroxybenzyl)-1-methyl-1H-indazole-5-carboxamide **(AI24)**

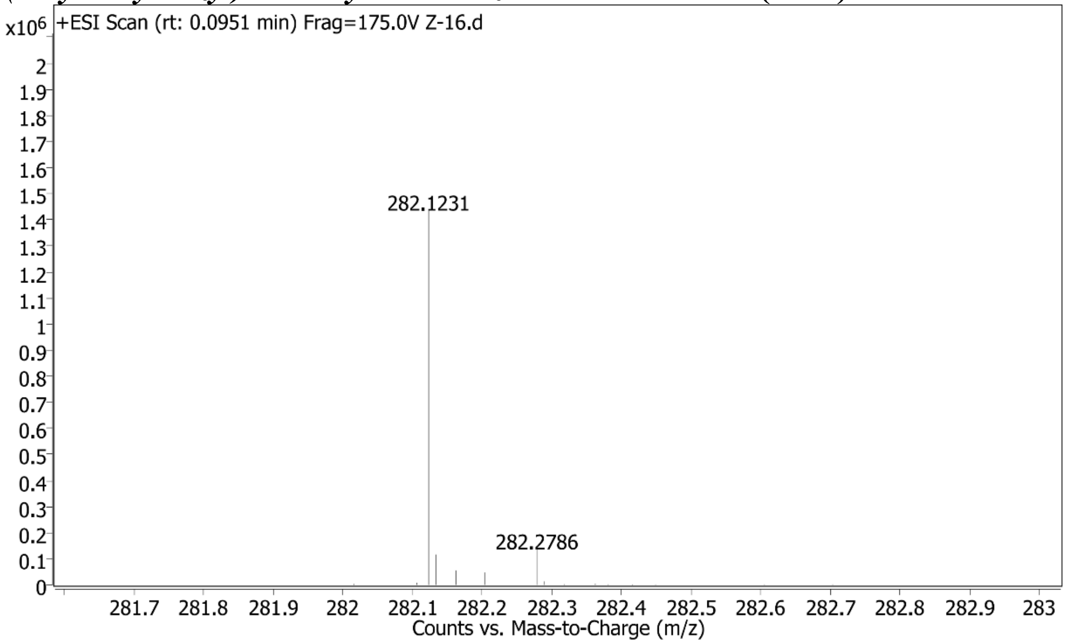

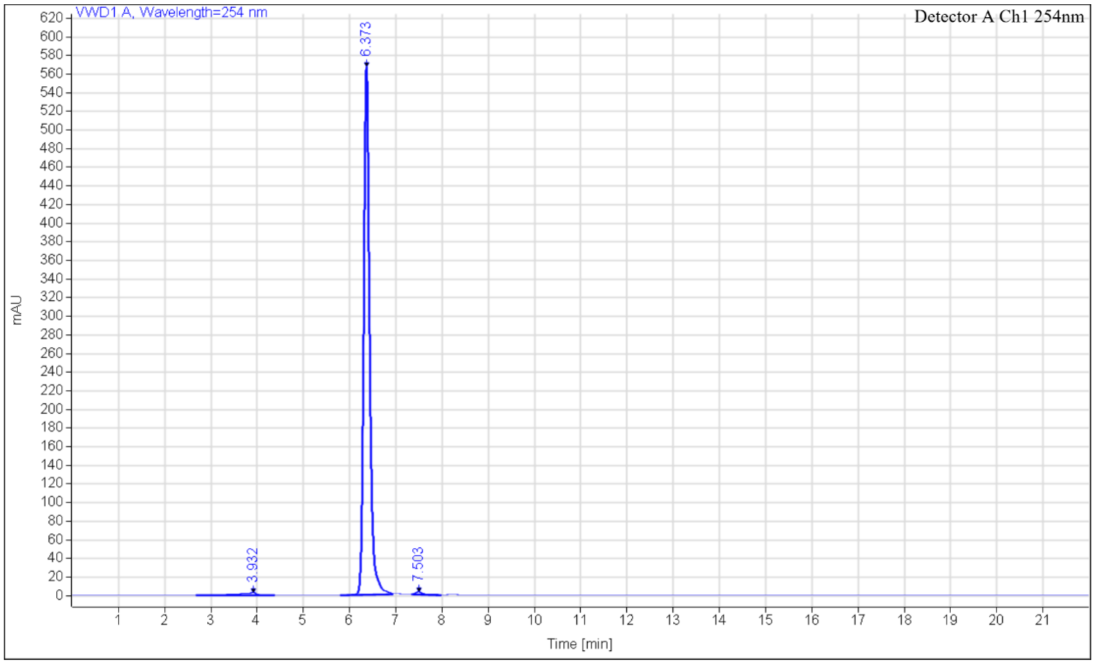

<Peak table>

Detector A Ch1 254nm

Number	Retention time	Peak area	Peak area%	Separation efficiency
1	3. 932	69. 95598	1. 36	--
2	6. 373	5030. 12012	98. 04	6. 52962
3	7. 503	30. 57705	0. 60	4. 84605
Total		5130. 65315	100. 00	

(USP)

N-(4-hydroxybenzyl)-6-(((4-methoxypyridin-2-yl)amino)methyl)picolinamide **(AI25)**

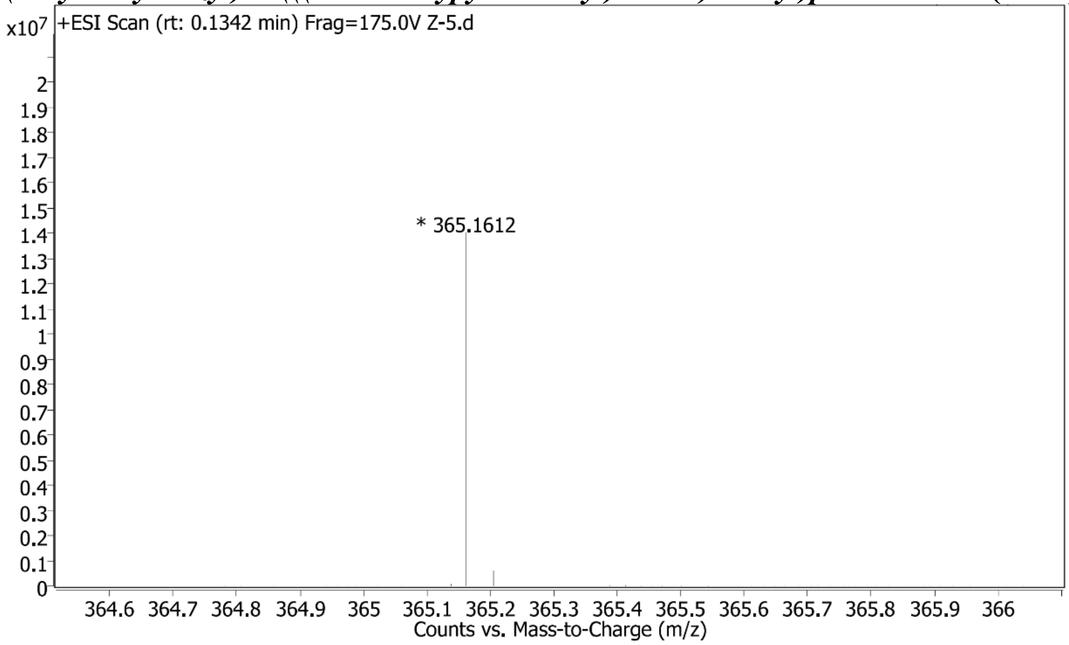

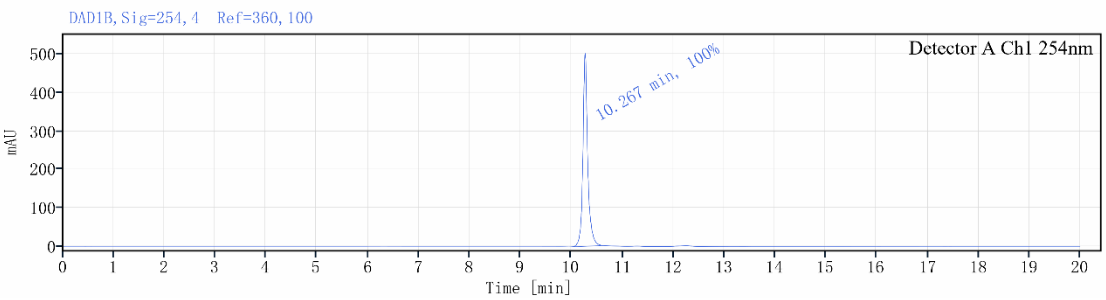

<Peak table>

Detector A Ch1 254nm

Number	Retention time	Peak area	Peak area%	Separation efficiency
1	10. 267	3283. 0	100. 00	--
Total		3283. 0	100. 00	

(USP)

N-(4-hydroxybenzyl)-1-methyl-1H-indole-3-carboxamide **(AI26)**

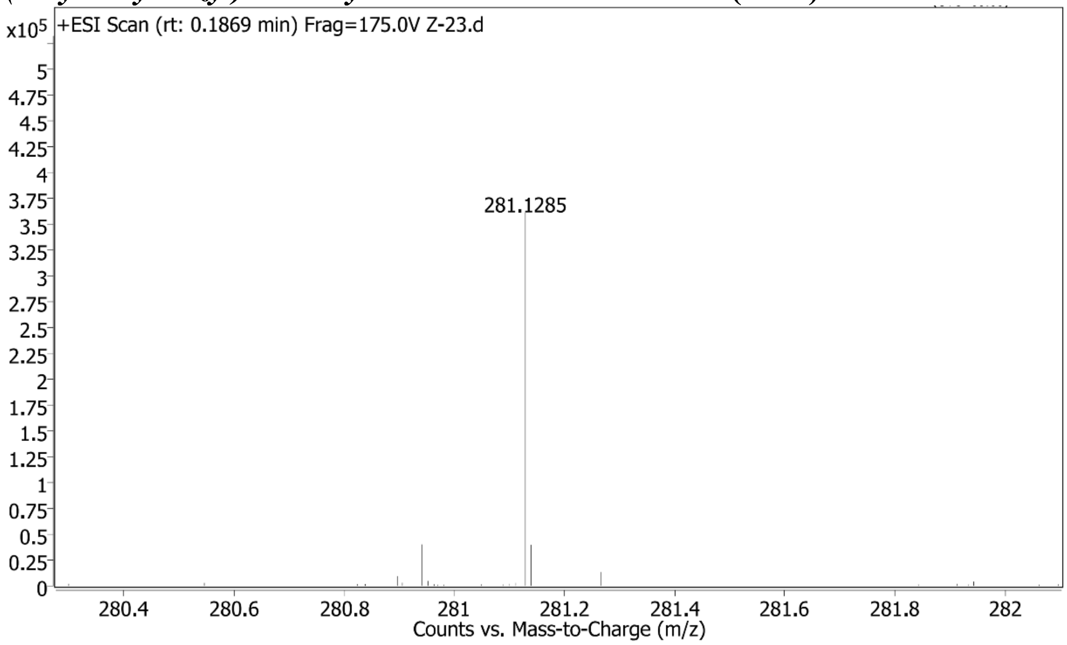

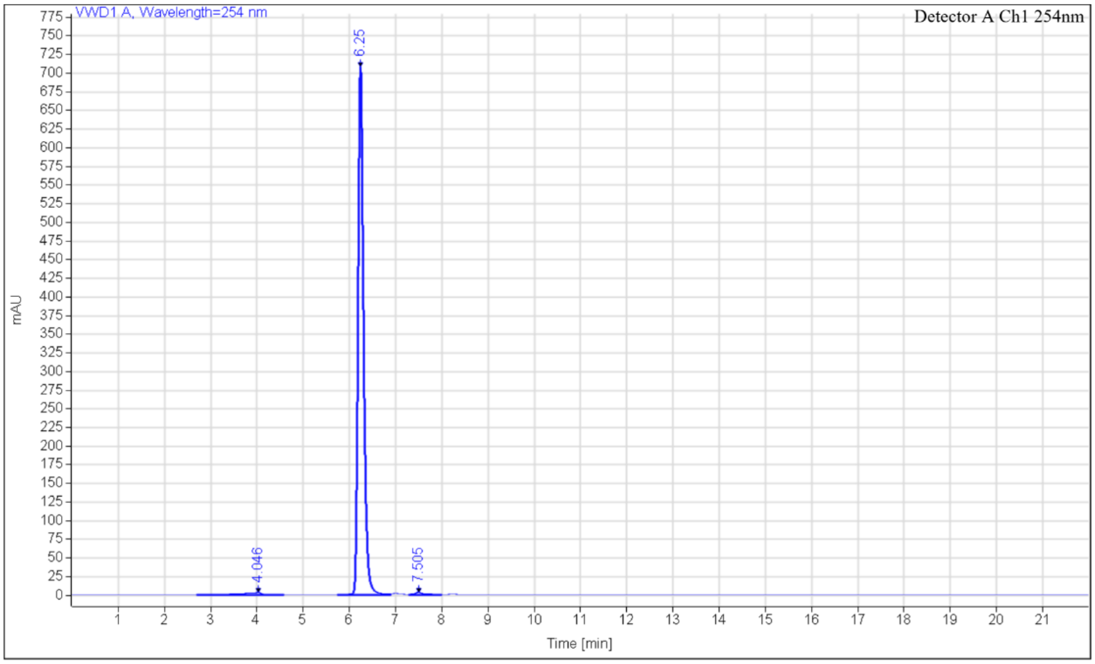

<Peak table>

Detector A Ch1 254nm

Number	Retention time	Peak area	Peak area%	Separation efficiency
1	4. 046	91. 93114	1. 53	--
2	6. 250	5873. 91797	97. 91	4. 95565
3	7. 505	33. 16623	0. 55	5. 36902
Total		5999. 01534	100. 00	

(USP)

N-(4-(4-hydroxyphenyl)thiazol-2-yl)-2-(2-methoxyphenyl)acetamide **(AI27)**

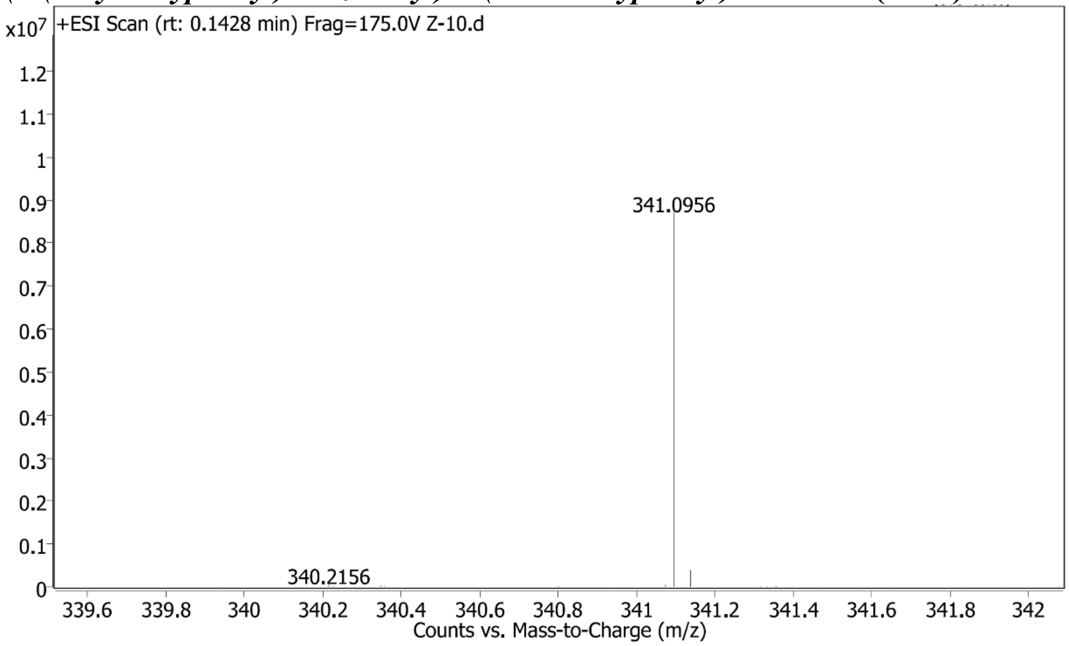

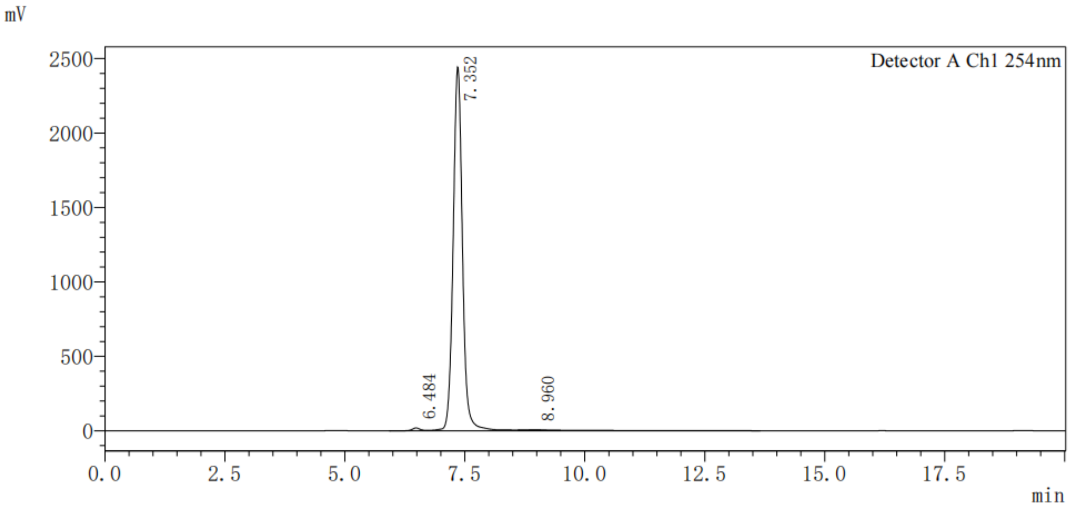

<Peak table>

Detector A Ch1 254nm

Number	Retention time	Peak area	Peak area%	Separation efficiency
1	6.484	224953	0.665	--
2	7.352	33560422	99.166	2.666
3	8.960	57241	0.169	3.740
Total		33842616	100.000	

(USP)

2-(4-fluoro-2-methoxyphenyl)-N-(4-(4-hydroxyphenyl)thiazol-2-yl)acetamide **(AI28)**

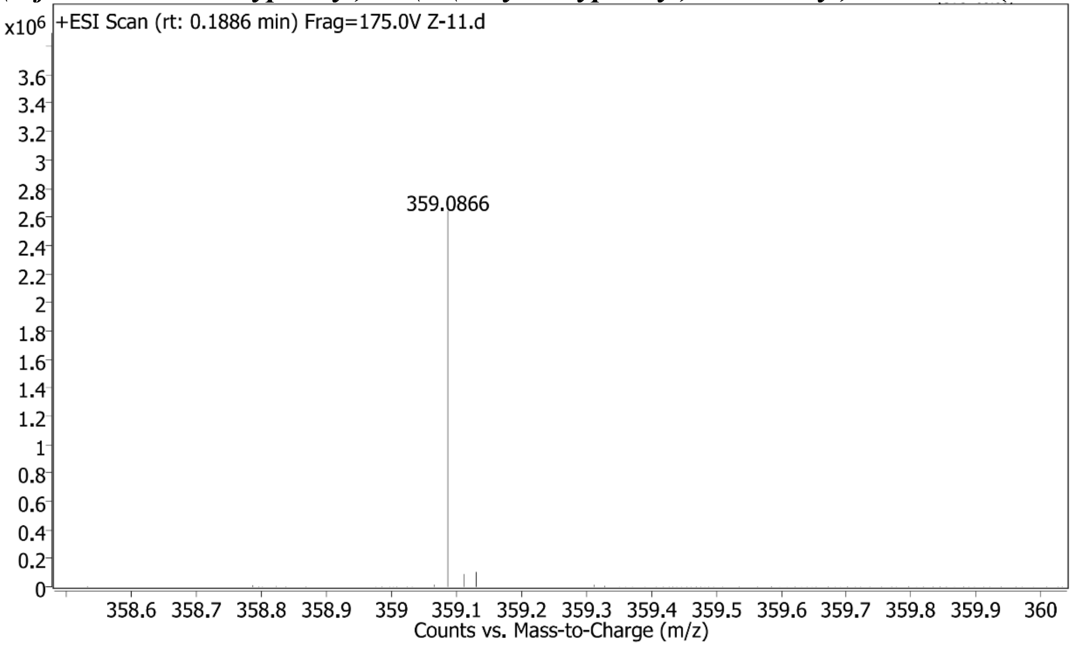

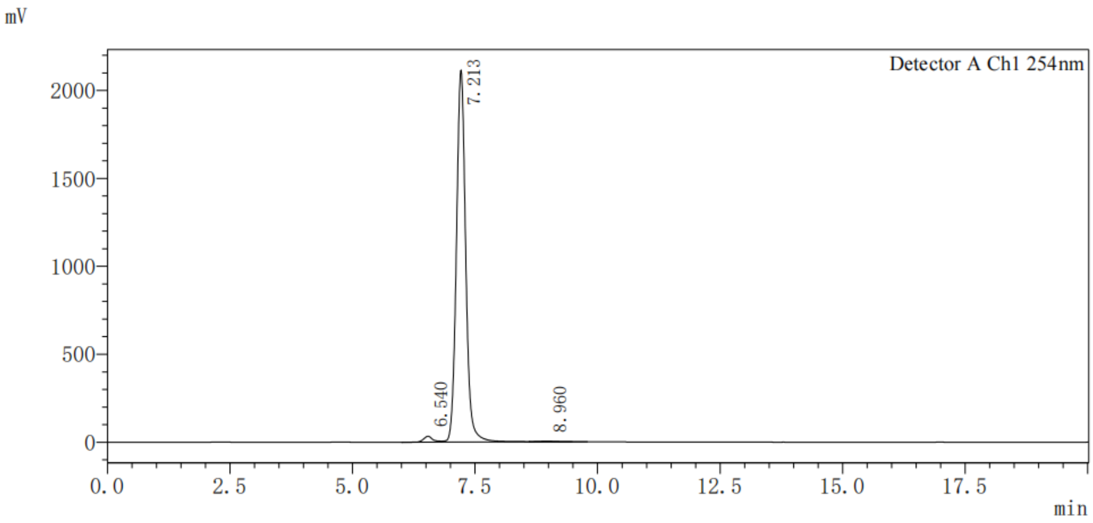

<Peak table>

Detector A Ch1 254nm

Number	Retention time	Peak area	Peak area%	Separation efficiency
1	6.540	446990	1.567	--
2	7.213	28022008	98.205	2.000
3	8.960	65230	0.229	3.798
Total		28534228	100.000	

(USP)

(2-hydroxyphenyl)(4-(4-hydroxyphenyl)piperazin-1-yl)methanone (AI10-m1)

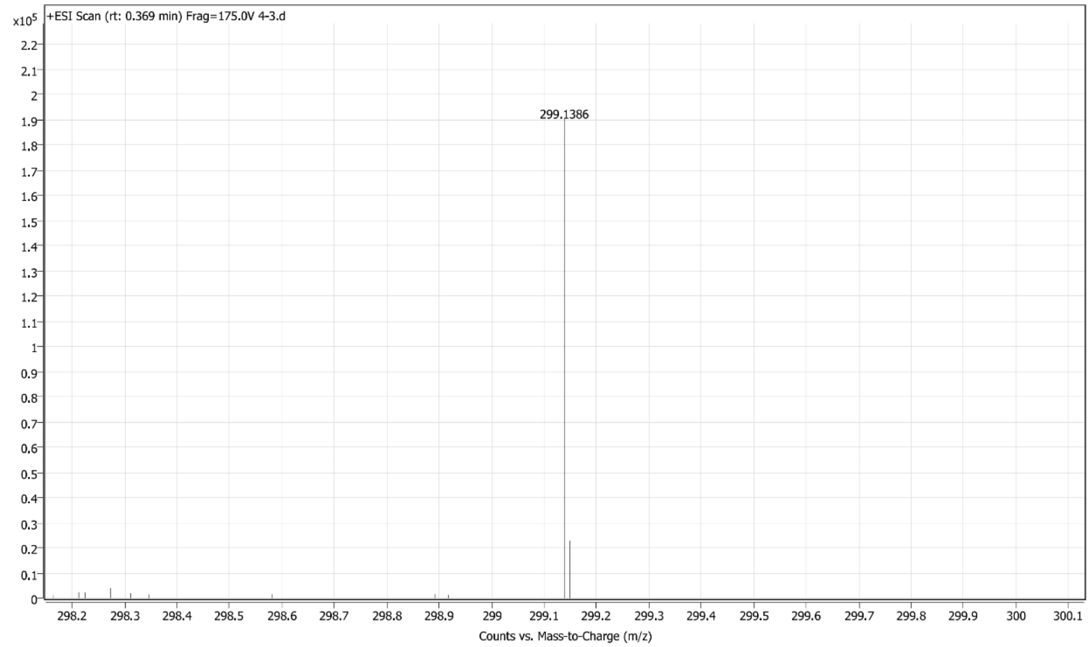

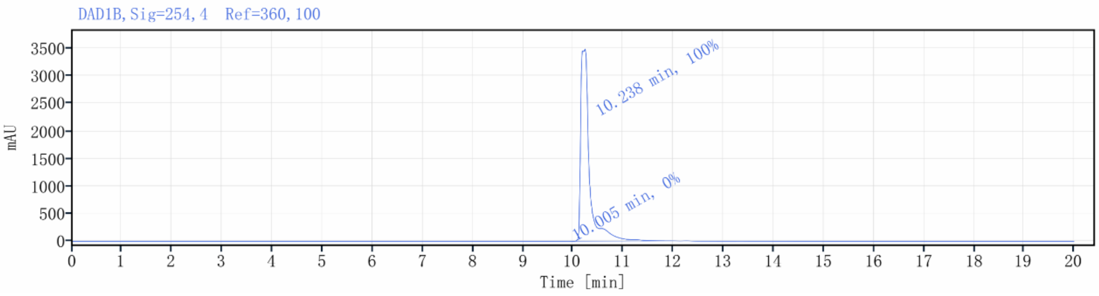

<Peak table>

Detector A Ch1 254nm

Number	Retention time	Peak area	Peak area%	Separation efficiency
1	10.005	0.3	0.00	--
2	10.238	42631.5	100.00	--
Total		42631.8	100.000	

(USP)

(2-hydroxy-5-methylphenyl)(4-(4-hydroxyphenyl)piperazin-1-yl)methanone **(AI10-m2)**

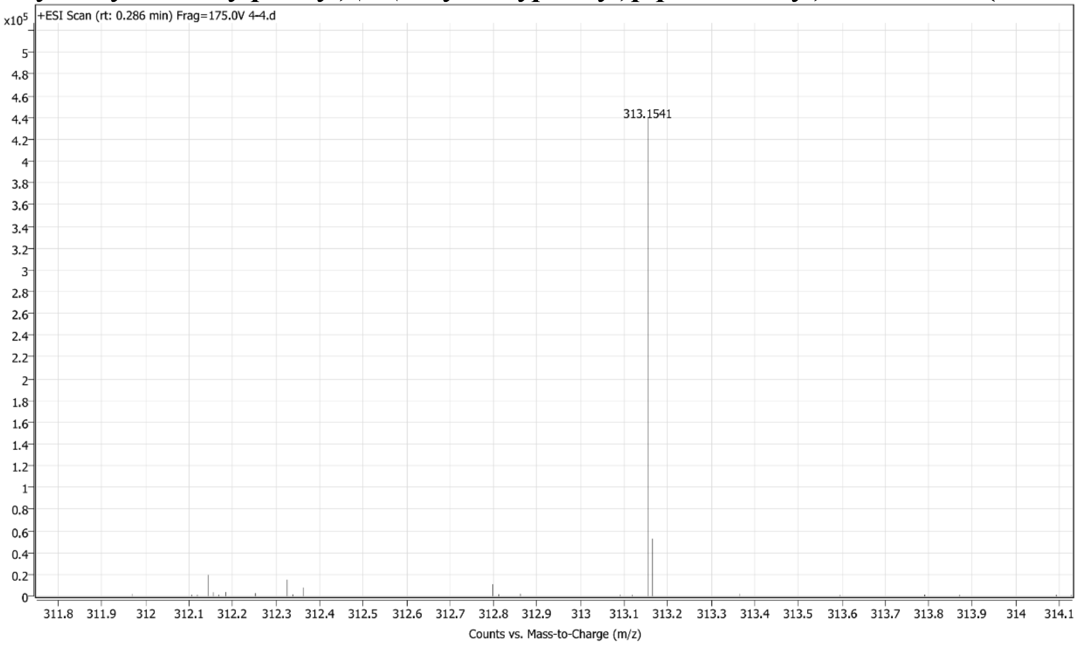

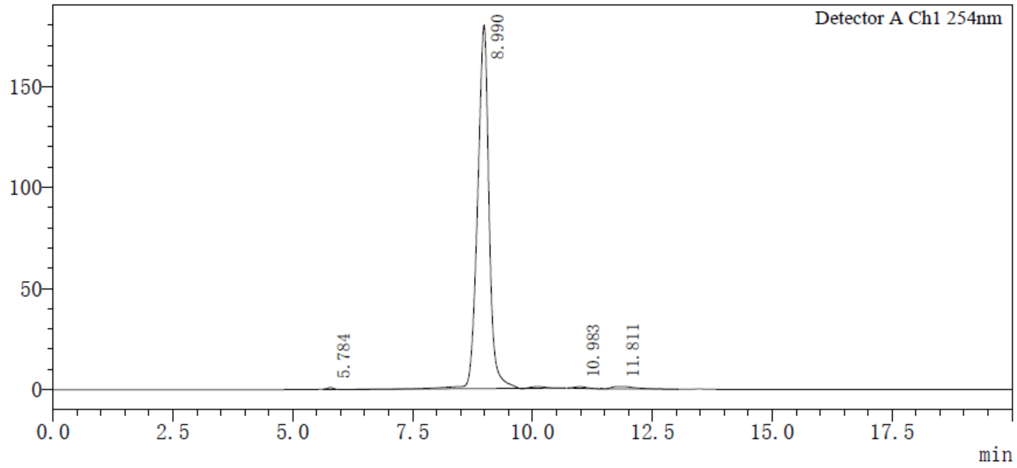

<Peak table>

Detector A Ch1 254nm

Number	Retention time	Peakarea	Peakarea%	Separation efficiency
1	5.784	5715	0.422	--
2	8.990	3014897	98.457	9.988
3	10.983	14017	0.436	4.370
4	11.811	41583	0.686	1.286
Total		3076213	100.000	

(USP)

(5-hydroxy-2-methylphenyl)(4-(4-hydroxyphenyl)piperazin-1-yl)methanone **(AI10-m3)**

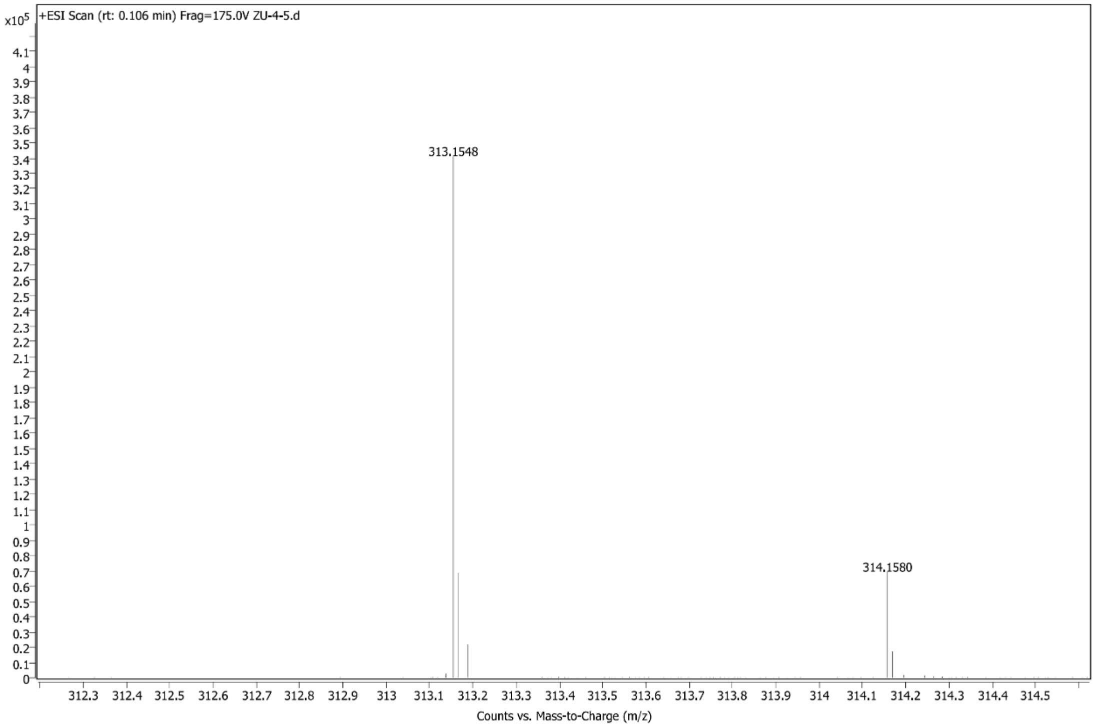

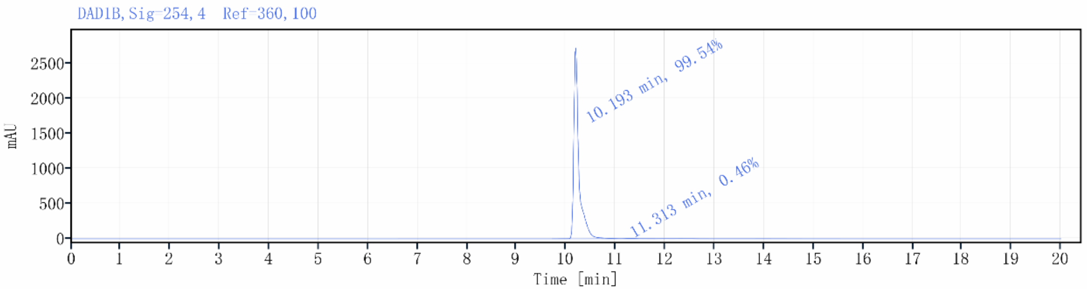

<Peak table>

Detector A Ch1 254nm

Number	Retention time	Peak area	Peak area%	Separation efficiency
1	10. 193	19697. 4	99. 54	--
2	11. 313	91. 8	0. 46	3. 143
Total		19789. 2	100. 00	

(USP)

(3-hydroxy-2-methylphenyl)(4-(4-hydroxyphenyl)piperazin-1-yl)methanone **(AI10-m4)**

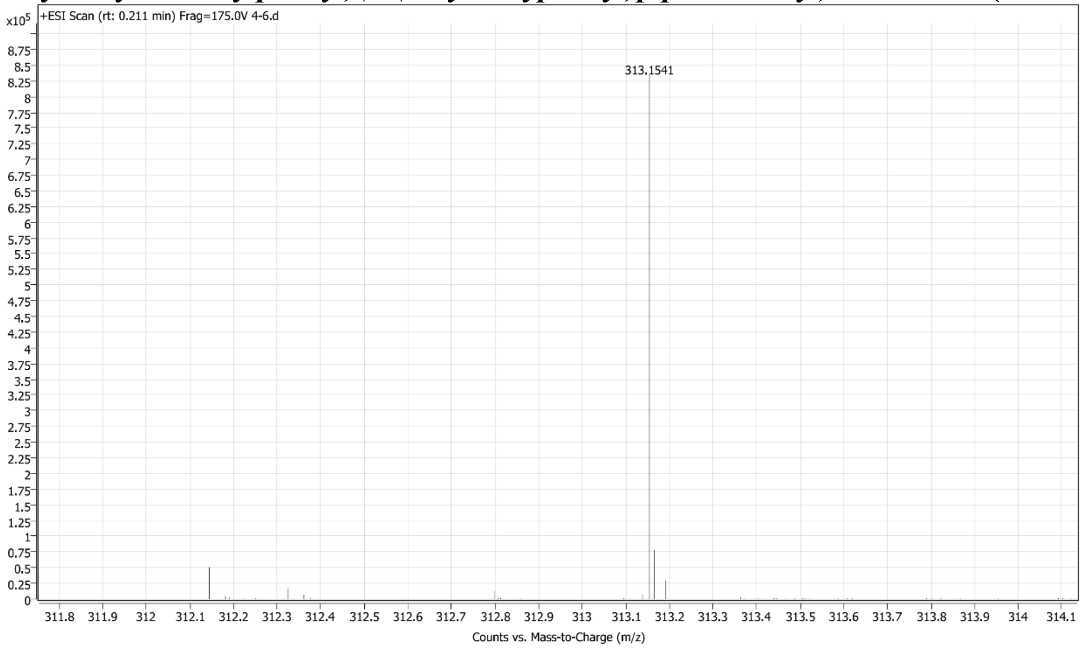

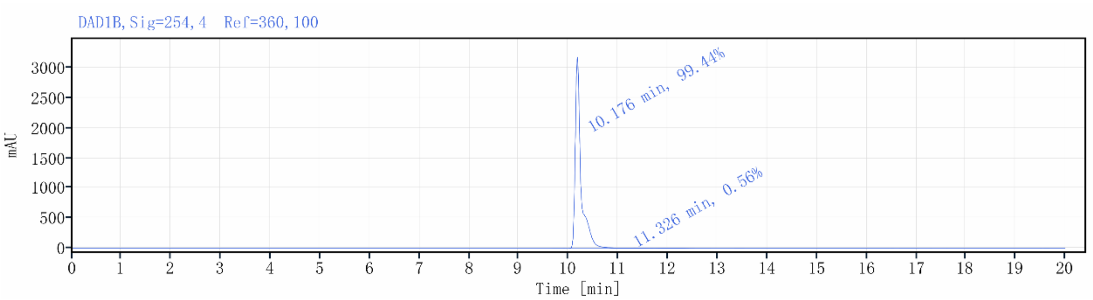

<Peak table>

Detector A Ch1 254nm

Number	Retention time	Peak area	Peak area%	Separation efficiency
1	10.176	25960.3	99.44	--
2	11.326	146.8	0.56	3.143
Total		26107.1	100.00	

(USP)

(4-fluorophenyl)(4-(4-hydroxyphenyl)piperazin-1-yl)methanone **(AI10-m5)**

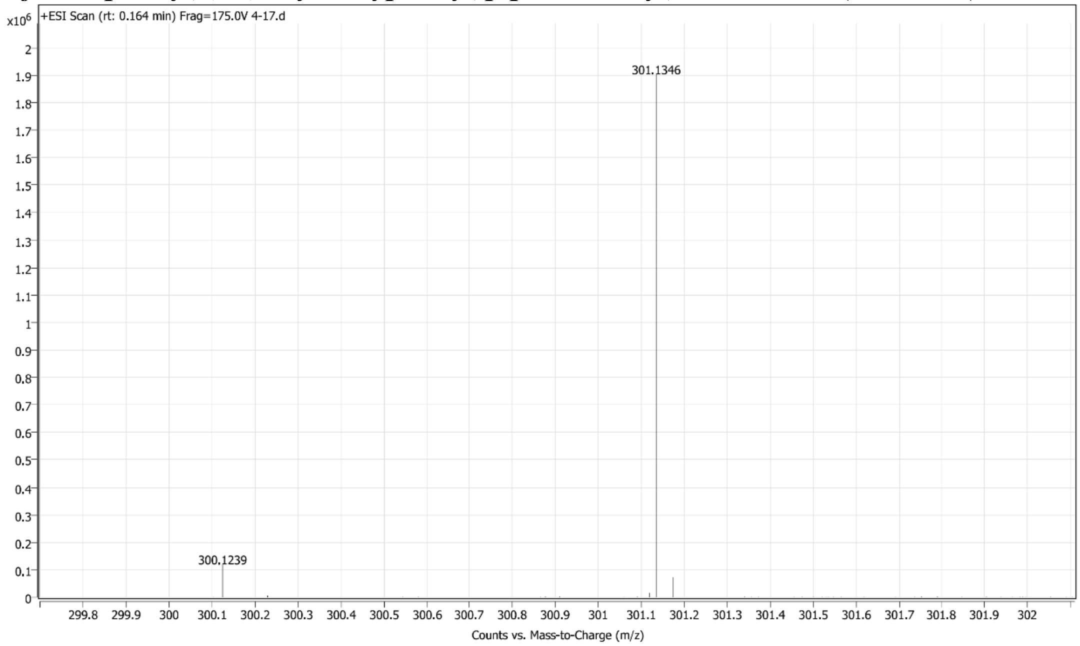

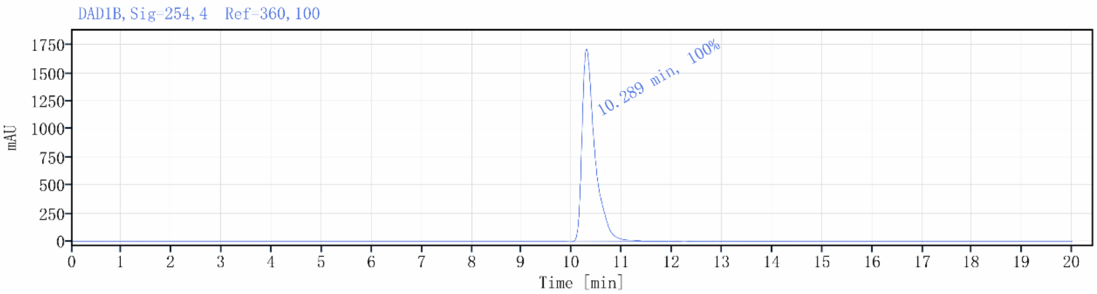

<Peak table>

Detector A Ch1 254nm

Number	Retention time	Peak area	Peak area%	Separation efficiency
1	10.289	31093.4	100.00	--
Total		31093.4	100.00	

(USP)

***(4-(4-hydroxyphenyl)piperazin-1-yl)(3,4,5-trimethoxyphenyl)methanone* (AI10-m6)**

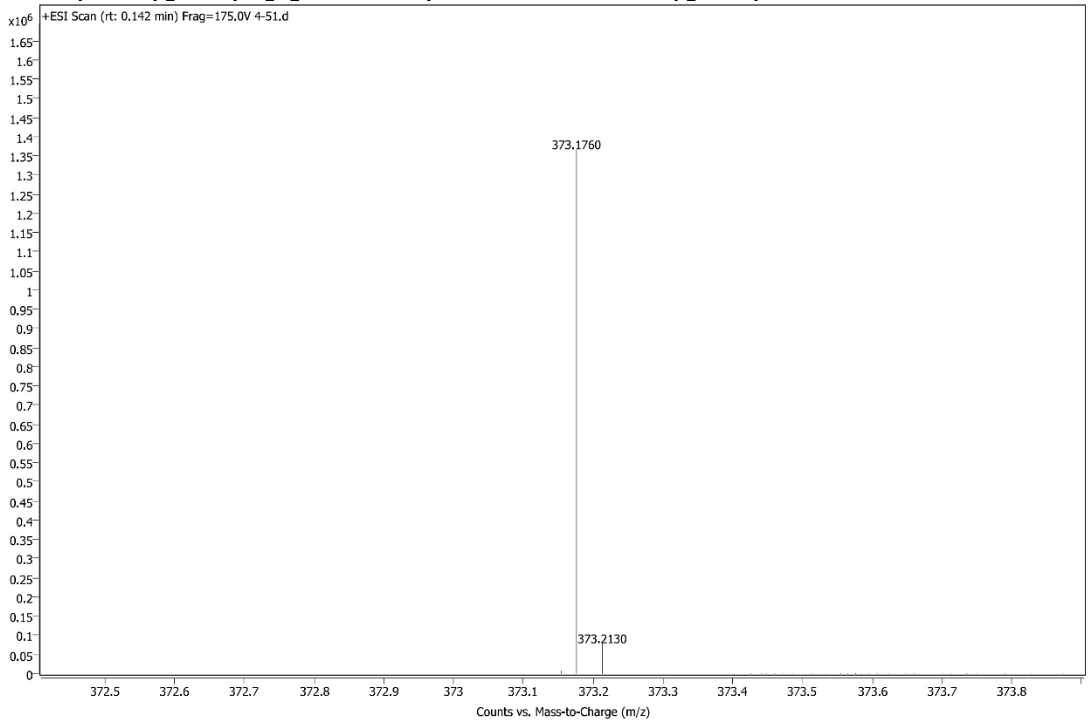

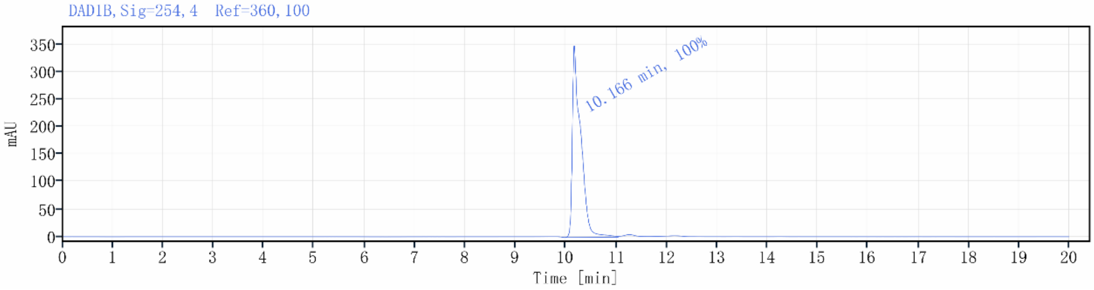

<Peak table>
Detector A Ch1 254nm

Number	Retention time	Peak area	Peak area%	Separation efficiency
1	10. 166	4067. 3	100. 00	--
Total		4067. 3	100. 00	

(USP)

(4-(4-hydroxyphenyl)piperazin-1-yl)(pyridin-3-yl)methanone (AI10-m7)

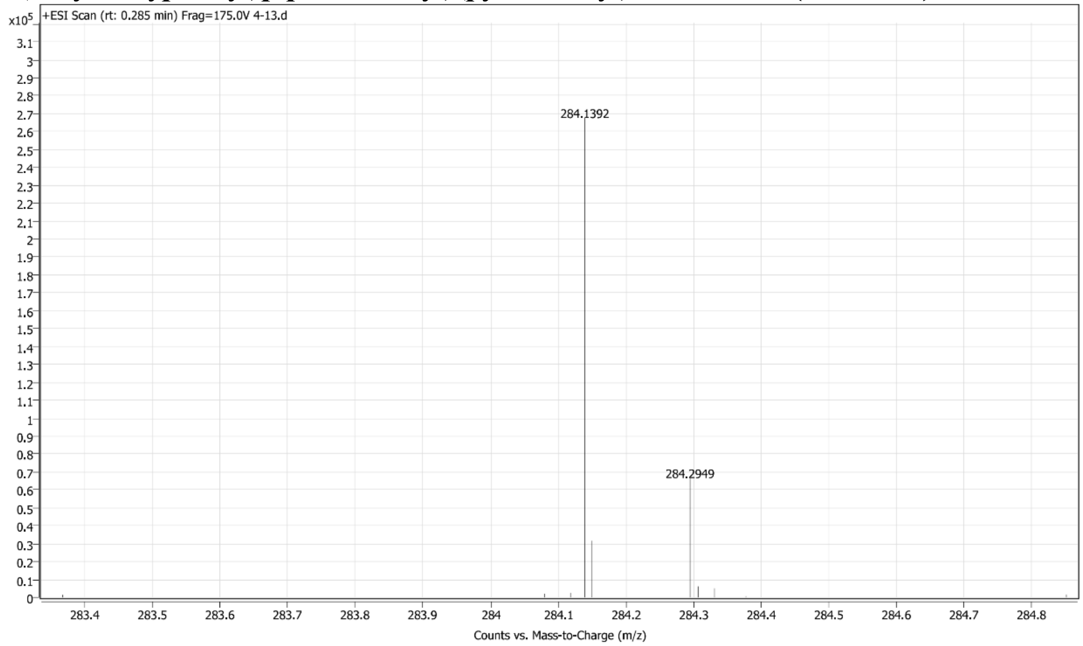

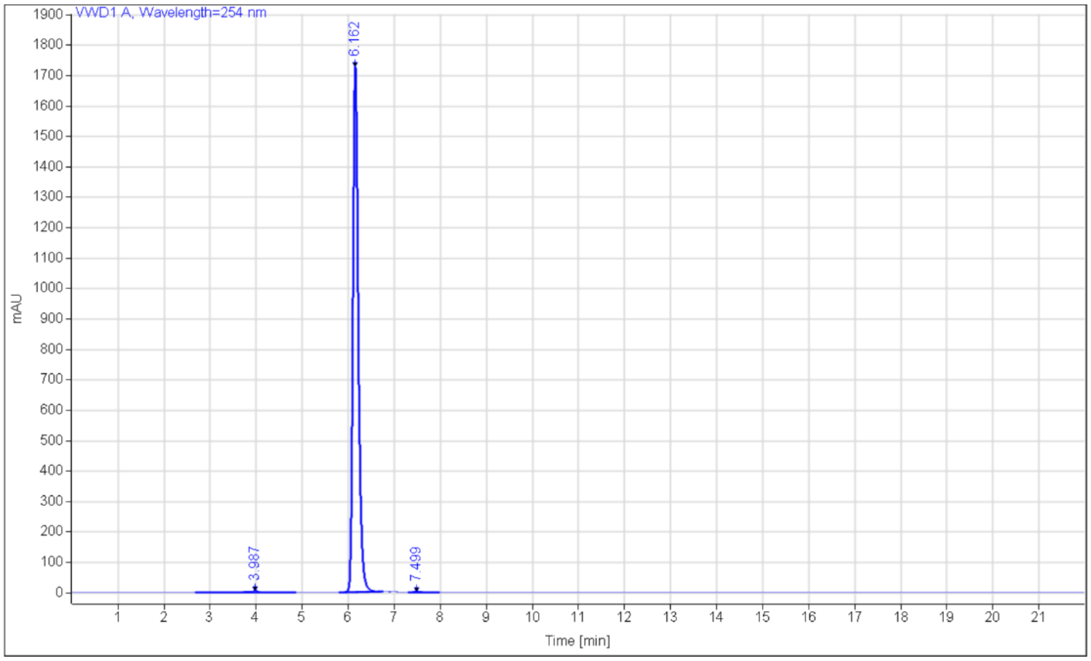

<Peak table>

Detector A Ch1 254nm

Number	Retention time	Peak area	Peak area%	Separation efficiency
1	3. 987	105. 60979	0. 74	--
2	6. 162	14128. 20215	99. 01	11. 28068
3	7. 499	35. 50211	0. 25	5. 73036
Total		14269. 31405	100. 00	

(USP)

(4-(4-hydroxyphenyl)piperazin-1-yl)(pyridin-2-yl)methanone **(AI10-m8)**

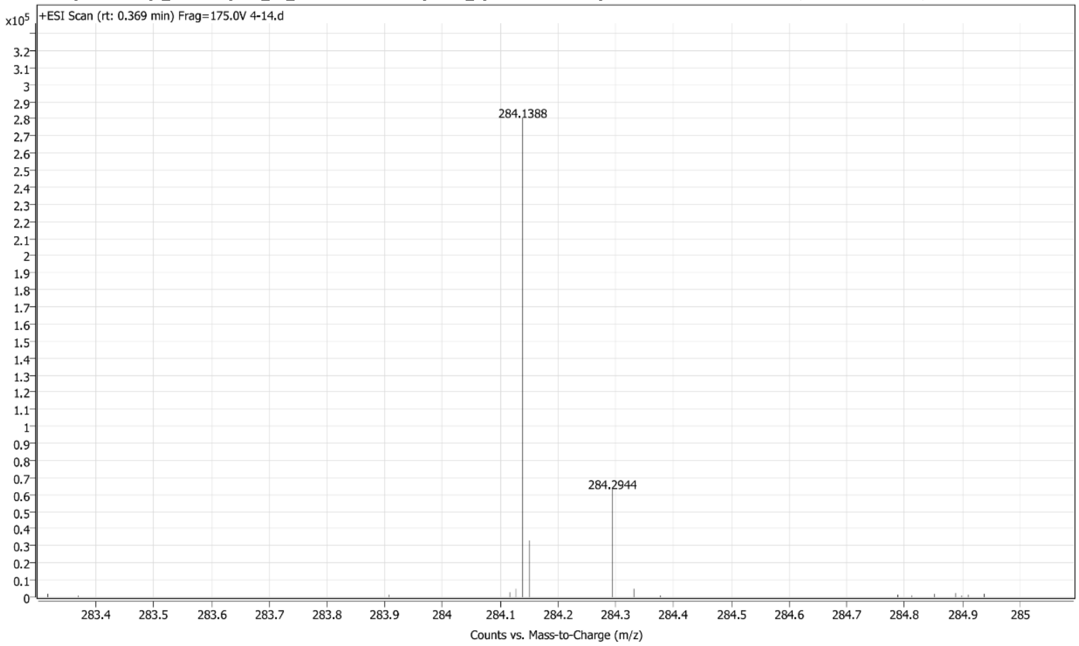

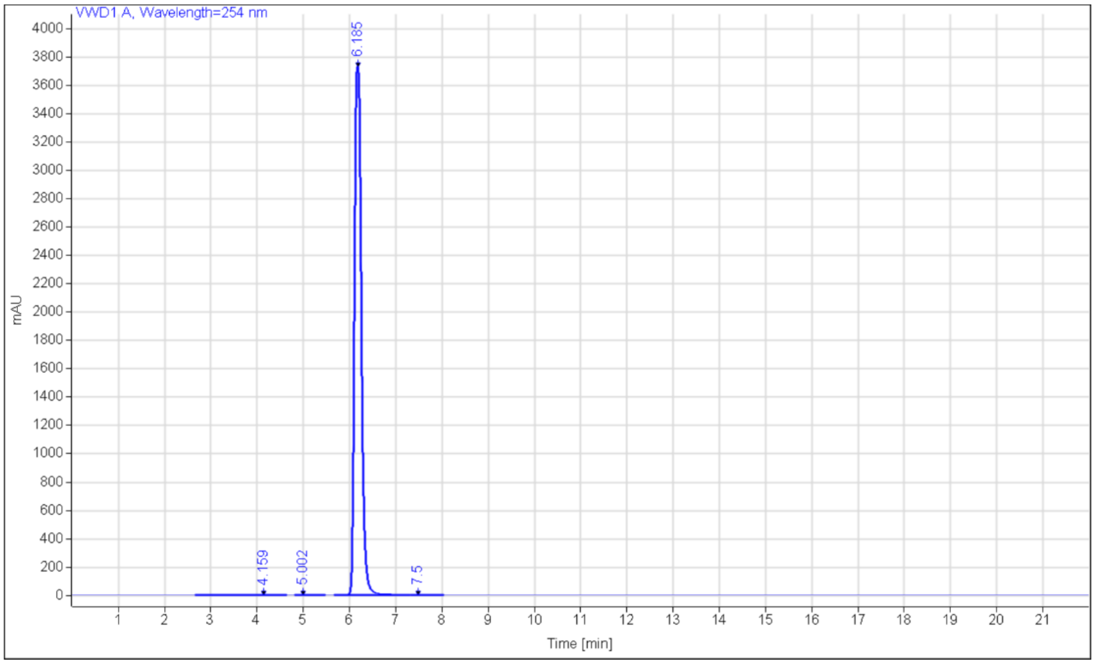

<Peak table>

Detector A Ch1 254nm

Number	Retention time	Peak area	Peak area%	Separation efficiency
1	4. 159	131. 60849	0. 34	--
2	5. 002	16. 16681	0. 04	1. 86489
3	6. 185	38664. 15625	99. 50	5. 12356
4	7. 500	47. 09562	0. 12	4. 92294
Total		38859. 02717	100. 00	

(USP)

***(4-(4-hydroxyphenyl)piperazin-1-yl)(pyridin-4-yl)methanone* (AI10-m9)**

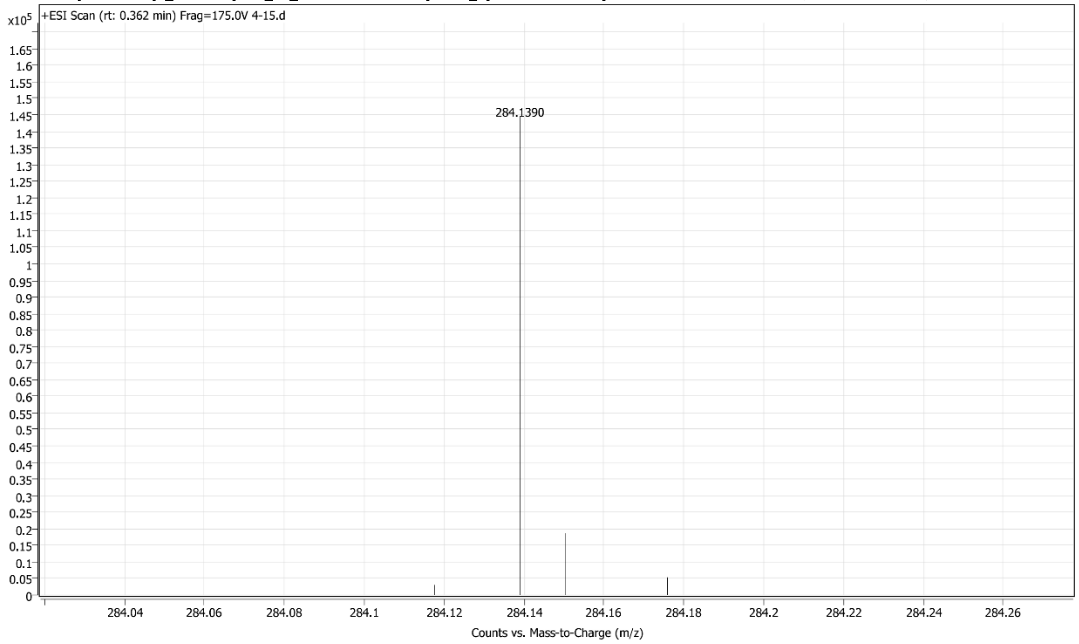

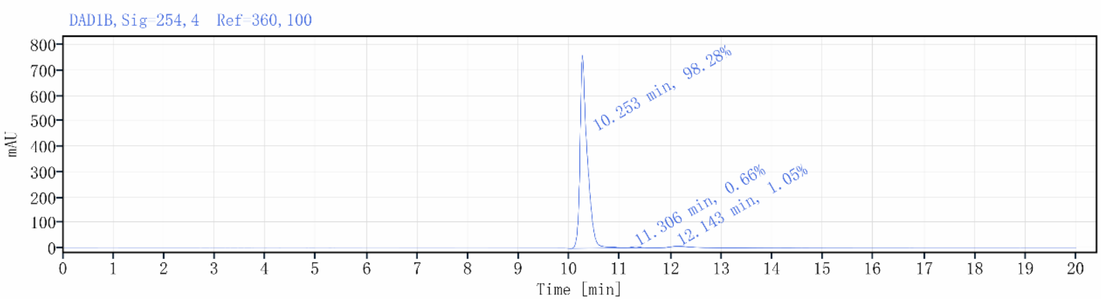

<Peak table>

Detector A Ch1 254nm

Number	Retention time	Peak area	Peak area%	Separation efficiency
1	10.253	7563.2	98.28	--
2	11.306	51.1	0.66	--
3	12.143	81.2	1.05	--
Total		7695.5	100.00	

(USP)

***(2,6-dimethylpyridin-4-yl)(4-(2-hydroxyphenyl)piperazin-1-yl)methanone* (AI10-m10)**

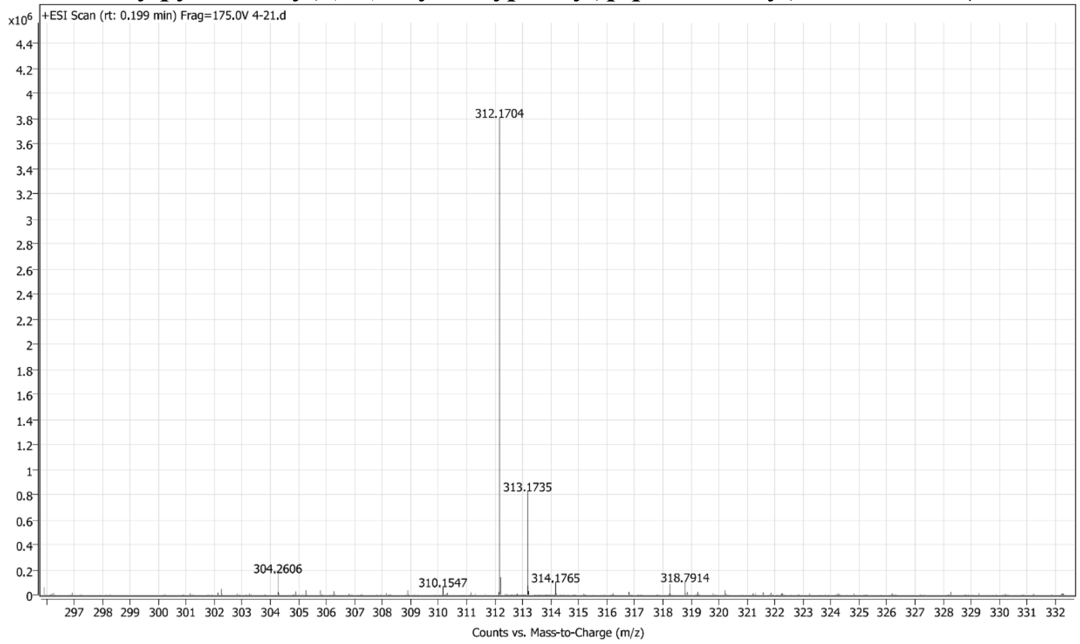

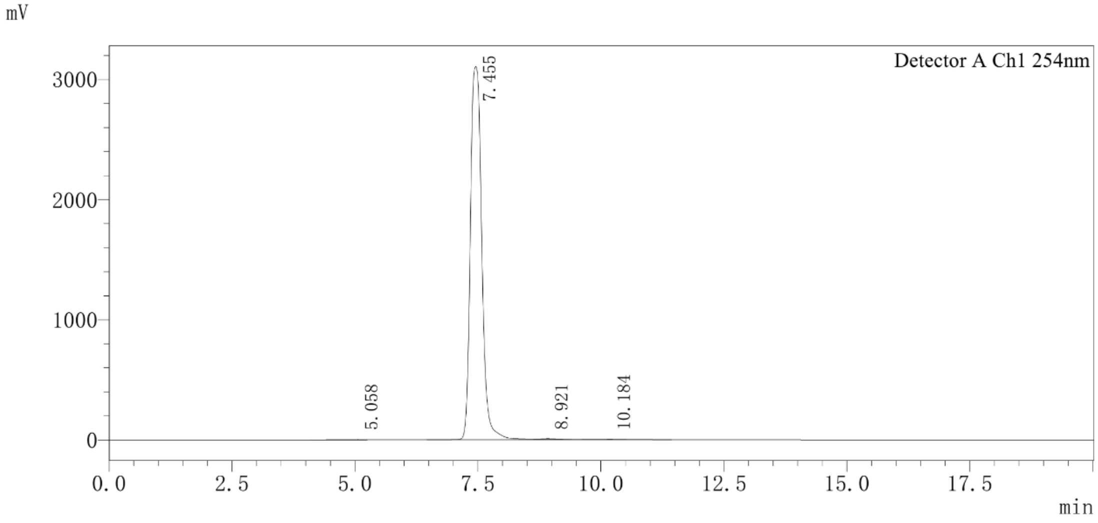

<Peak table>

Detector A Ch1 254nm

Number	Retention time	Peak area	Peak area%	Separation efficiency
1	5.058	53974	0.107	--
2	7.455	50455830	99.626	5.128
3	8.921	79240	0.156	3.344
4	10.184	56318	0.111	1.878
Total		50645361	100.000	

(USP)

(2,6-dimethylpyridin-4-yl)(4-(3-hydroxyphenyl)piperazin-1-yl)methanone **(AI10-m11)**

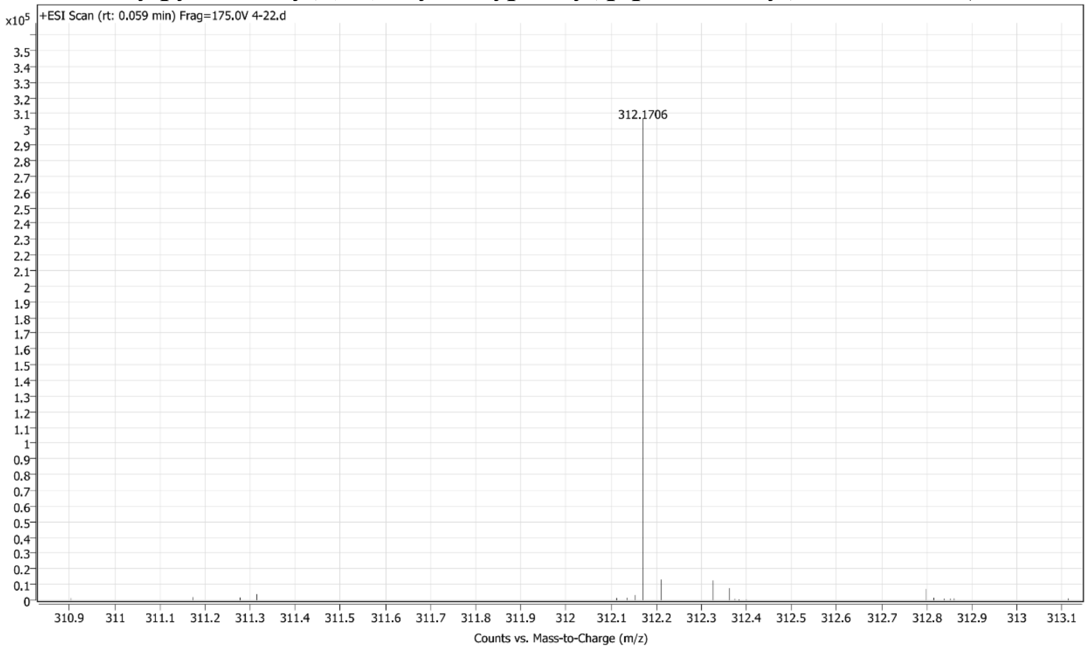

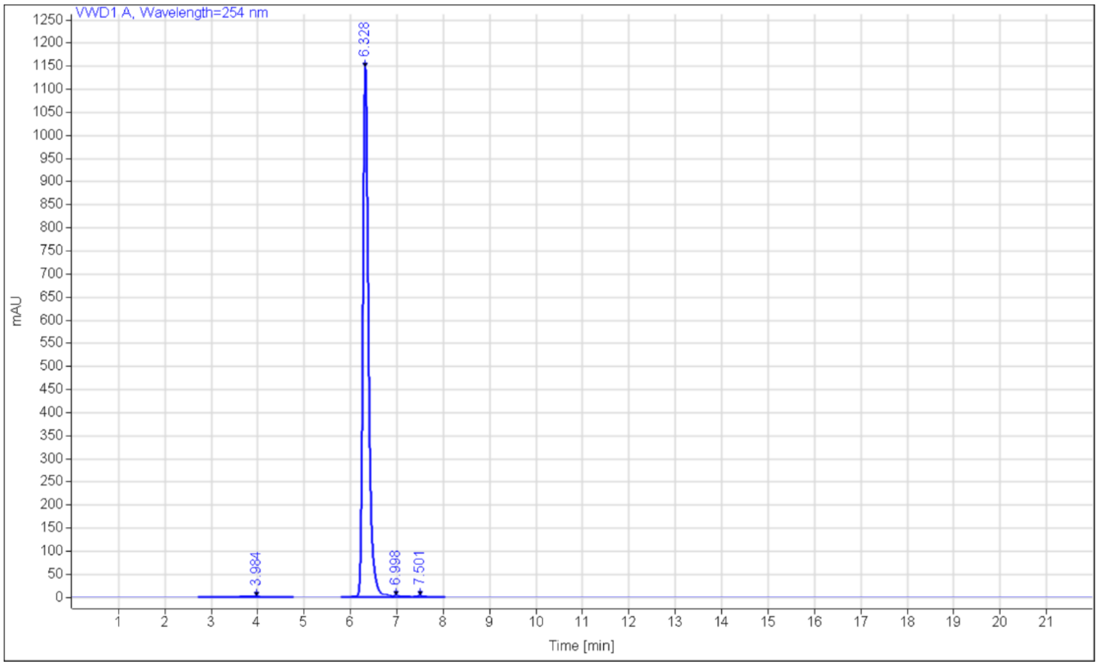

<Peak table>

Detector A Ch1 254nm

Number	Retention time	Peak area	Peak area%	Separation efficiency
1	3. 984	74. 01992	0. 75	--
2	6. 328	9763. 00391	98. 37	6. 34625
3	6. 998	46. 12660	0. 46	2. 10891
4	7. 501	41. 12880	0. 41	1. 41993
Total		9924. 27923	100. 00	

(USP)

(4-(2,4-dihydroxyphenyl)piperazin-1-yl)(2,6-dimethylpyridin-4-yl)methanone **(AI10-m12)**

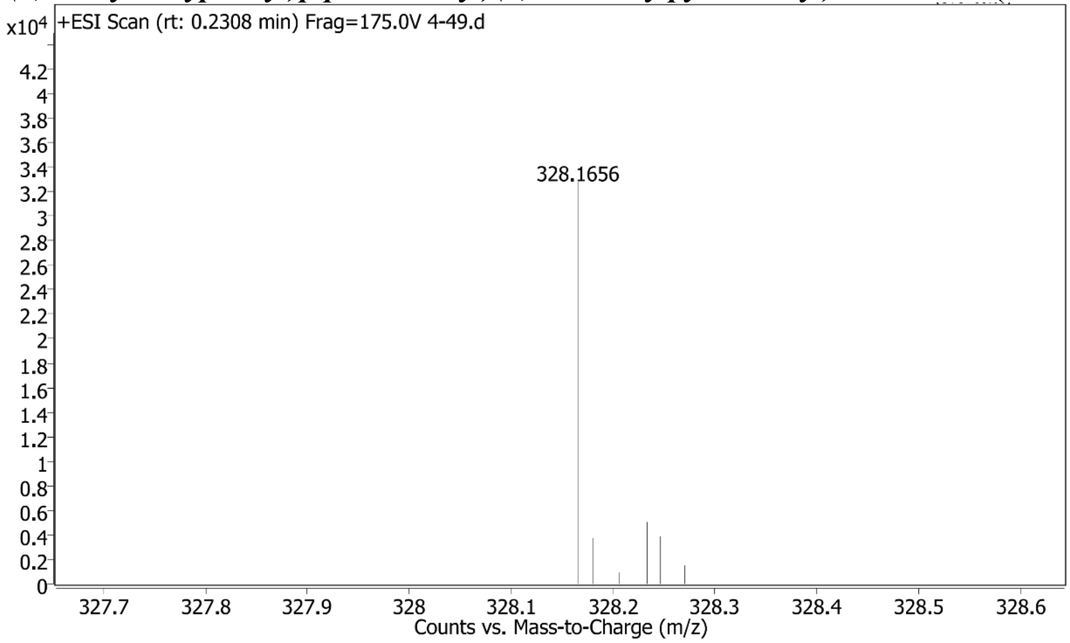

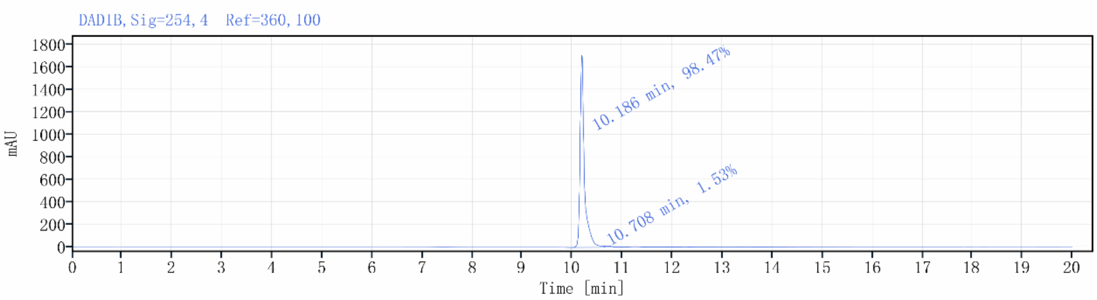

<Peak table>

Detector A Ch1 254nm

Number	Retention time	Peak area	Peak area%	Separation efficiency
1	10.186	10400.8	98.47	--
2	10.708	161.8	1.53	--
Total		10562.6	100.00	

(USP)

2-(4-fluorophenyl)-1-(4-(4-hydroxyphenyl)piperazin-1-yl)ethan-1-one **(AI10-m13)**

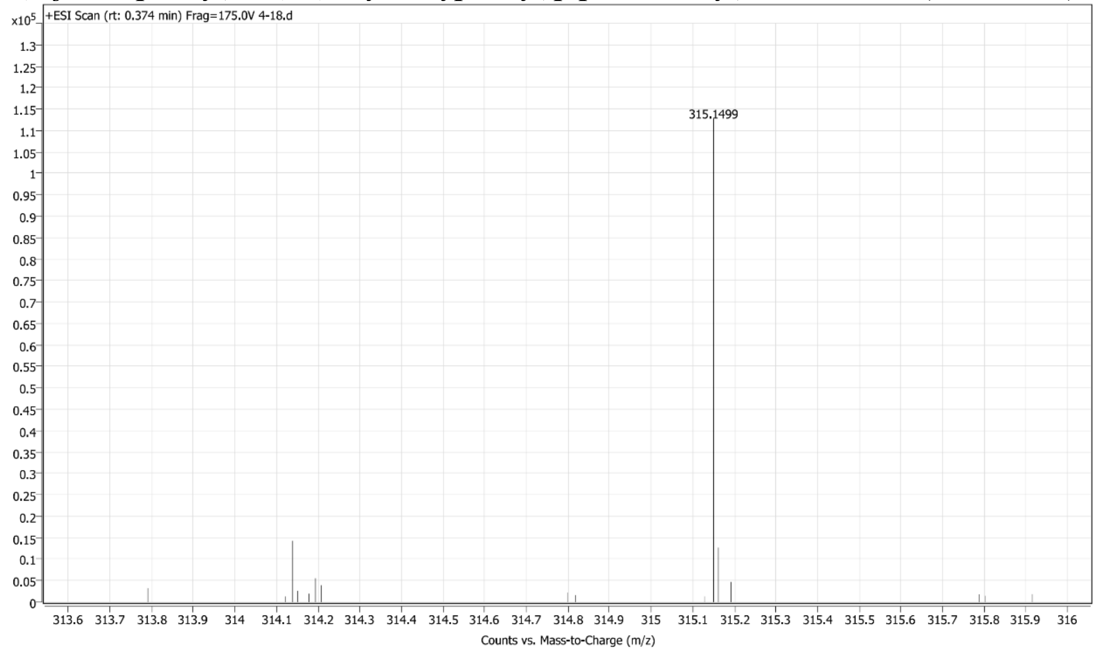

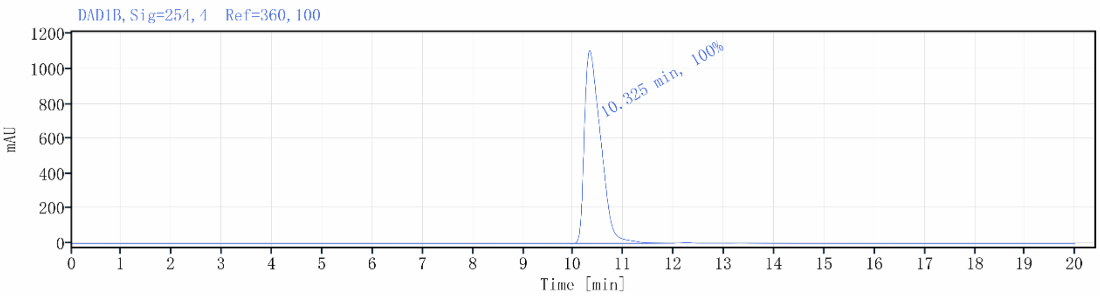

<Peak table>

Detector A Ch1 254nm

Number	Retention time	Peak area	Peak area%	Separation efficiency
1	10. 325	24987. 2	100. 00	--
Total		24987. 2	100. 00	

(USP)

1-(4-(4-hydroxyphenyl)piperazin-1-yl)-2-(2-methoxyphenyl)ethan-1-one **(AI10-m14)**

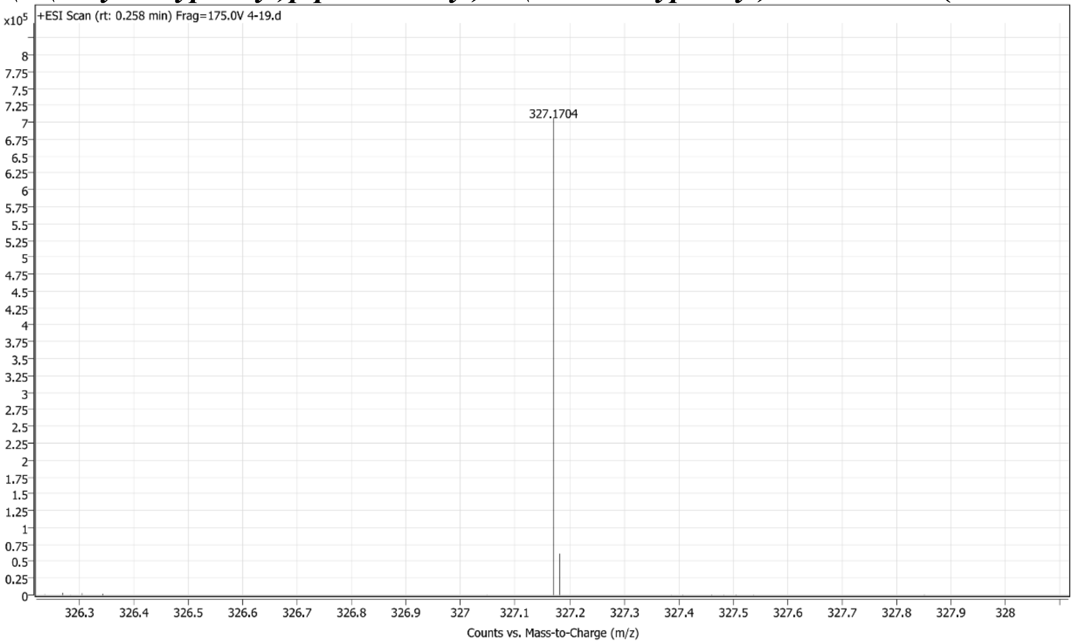

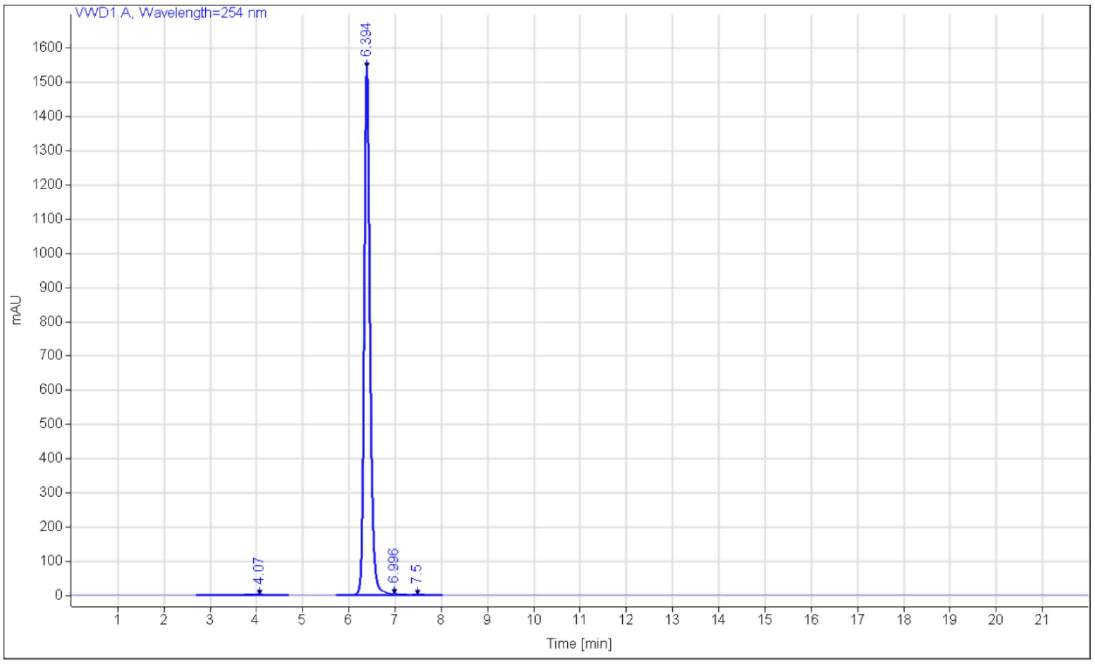

<Peak table>
Detector A Ch1 254nm

Number	Retention time	Peak area	Peak area%	Separation efficiency
1	4.070	92.75680	0.67	--
2	6.394	13547.53809	98.56	5.74577
3	6.996	57.21896	0.42	1.58145
4	7.500	47.56437	0.35	1.20906
Total		13745.07822	100.00	

(USP)

***(E)-3-(2,4-dihydroxyphenyl)-1-(4-(4-hydroxyphenyl)piperazin-1-yl)prop-2-en-1-one* (AI10-m15)**

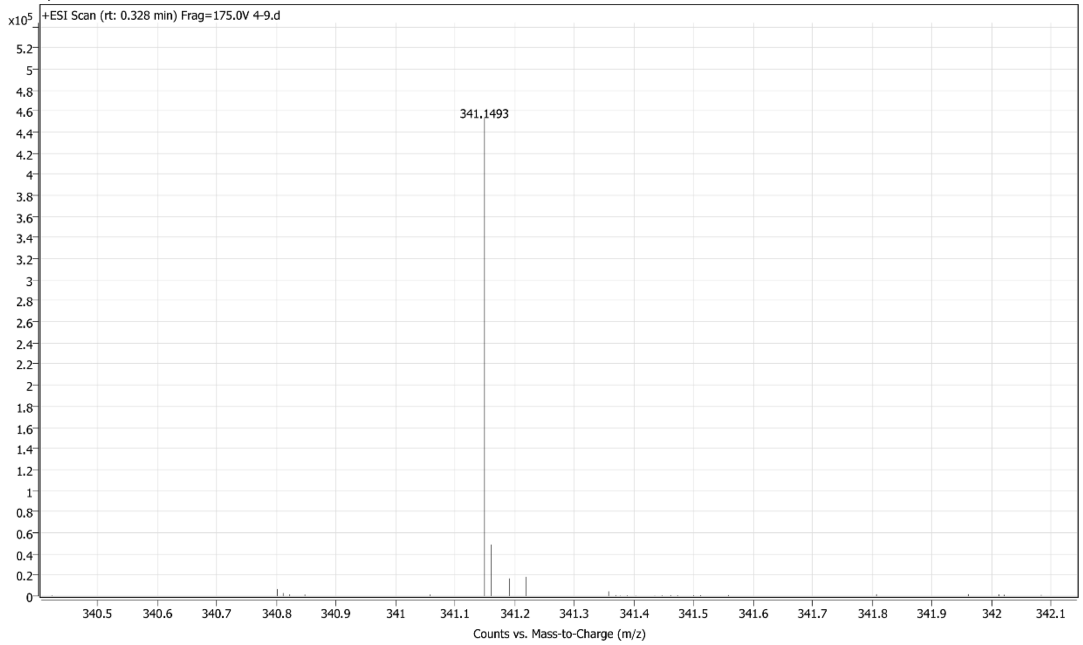

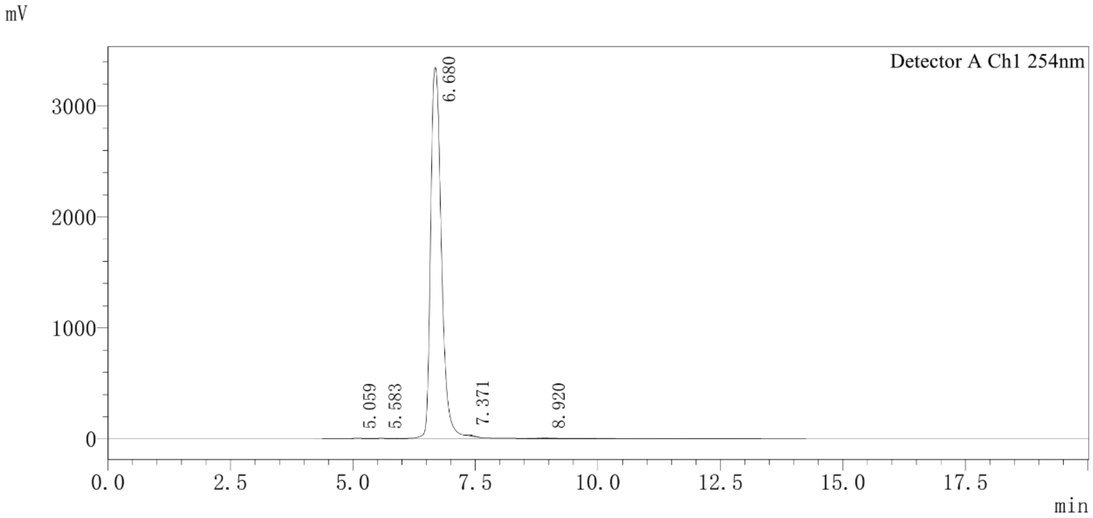

<Peak table>

Detector A Ch1 254nm

Number	Retention time	Peak area	Peak area%	Separation efficiency
1	5.059	163520	0.308	--
2	5.583	146409	0.276	0.335
3	6.680	52532230	99.038	1.625
4	7.371	64218	0.121	2.221
5	8.920	136385	0.257	3.828
Total		53042762	100.000	

(USP)

***(E)-3-(3,4-dihydroxyphenyl)-1-(4-(4-hydroxyphenyl)piperazin-1-yl)prop-2-en-1-one* (AI10-m16)**

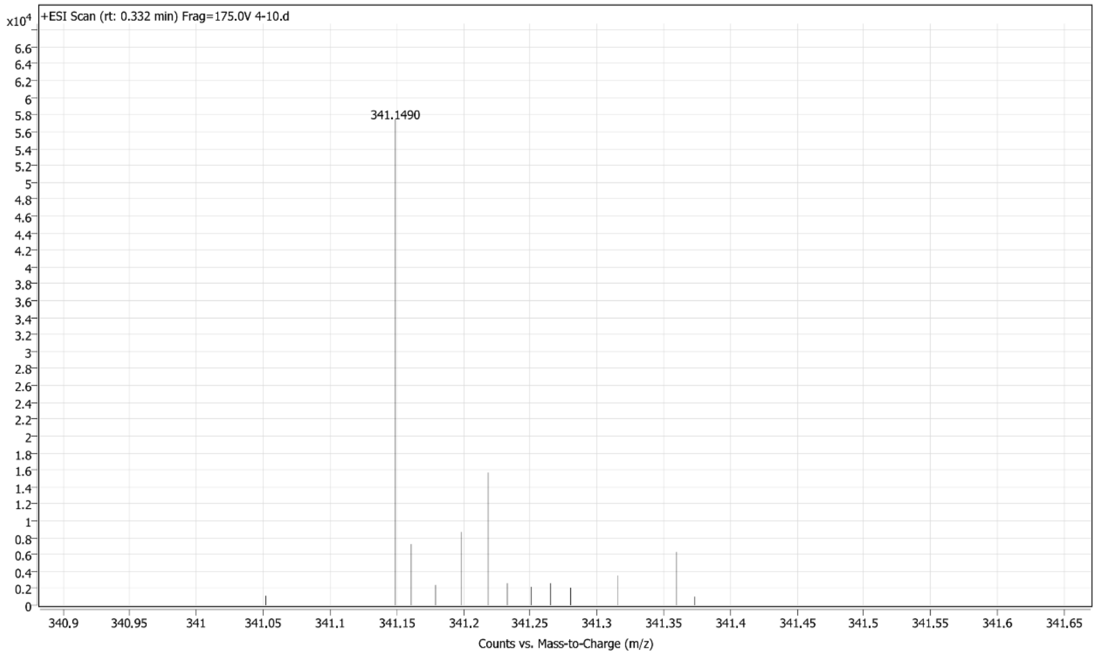

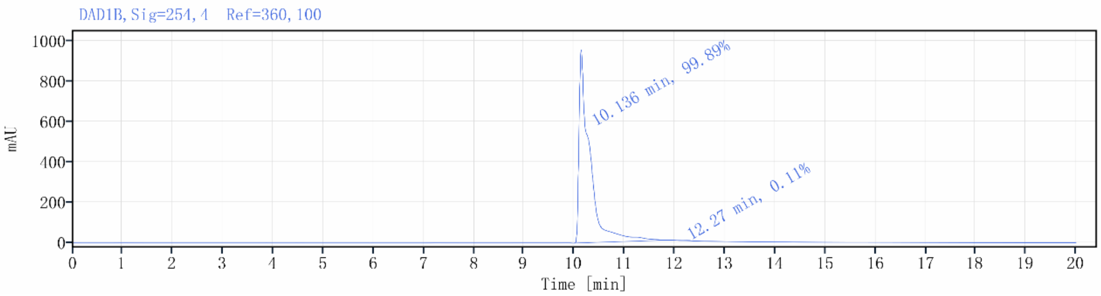

<Peak table>

Detector A Ch1 254nm

Number	Retention time	Peak area	Peak area%	Separation efficiency
1	10. 136	14281. 7	99. 89	--
2	12. 270	15. 6	0. 11	--
Total		14297. 3	100. 00	

(USP)

(4-(4-hydroxyphenyl)piperazin-1-yl)(thiazol-2-yl)methanone **(AI10-m17)**

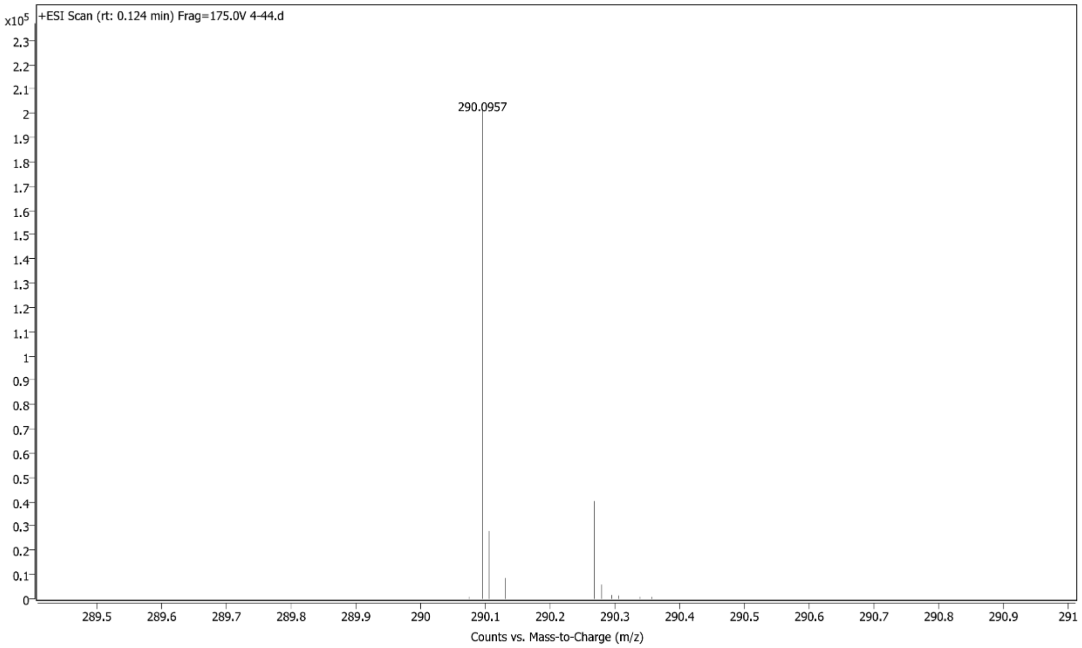

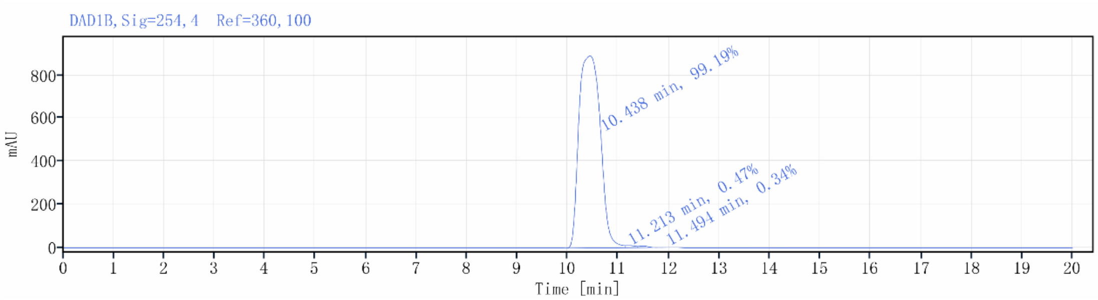

<Peak table>

Detector A Ch1 254nm

Number	Retention time	Peak area	Peak area%	Separation efficiency
1	10. 438	26350. 9	99. 19	--
2	11. 213	124. 9	0. 47	--
3	11. 494	90. 8	0. 34	
Total		26566. 6	100. 00	

(USP)

(4-(4-hydroxyphenyl)piperazin-1-yl)(1-methyl-1H-indazol-6-yl)methanone **(AI10-m18)**

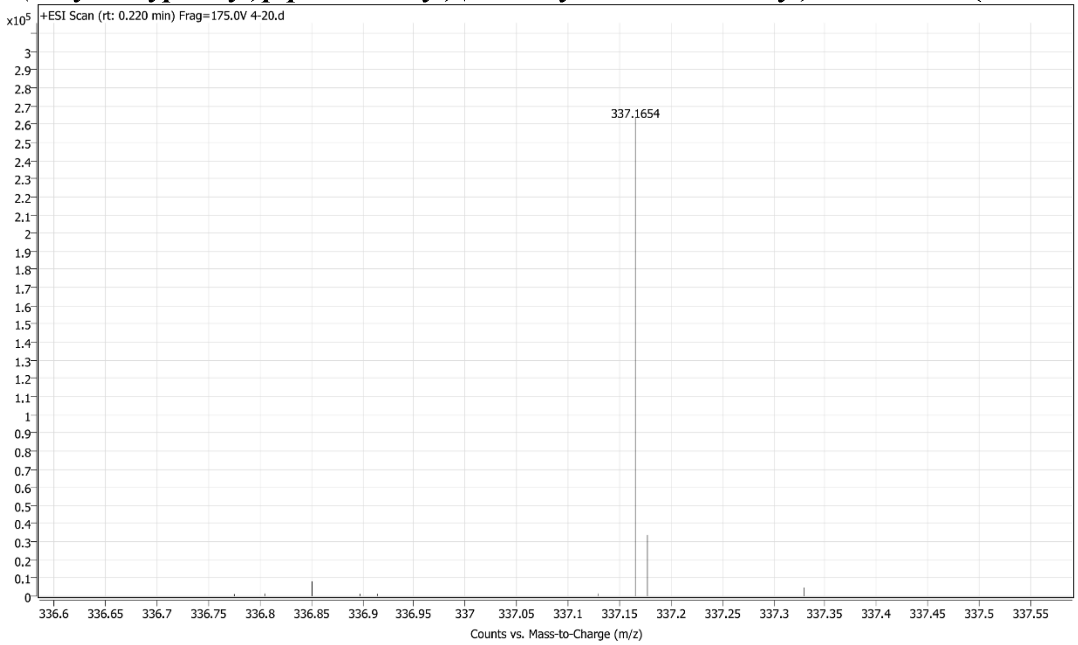

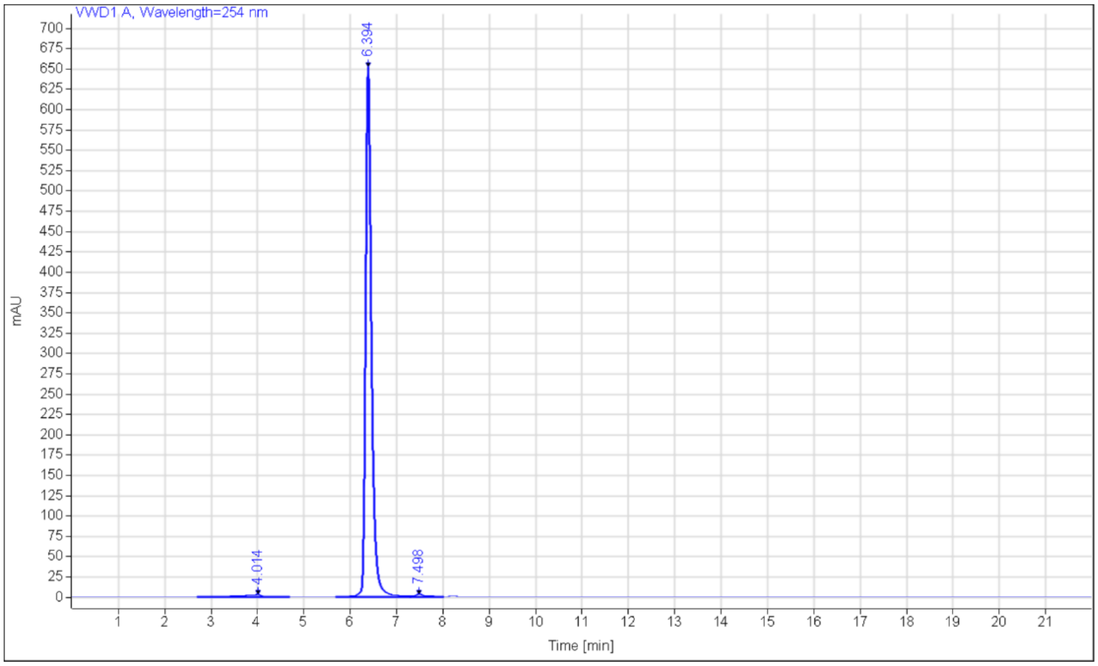

<Peak table>
Detector A Ch1 254nm

Number	Retention time	Peak area	Peak area%	Separation efficiency
1	4.014	82.31475	1.39	--
2	6.394	5790.48145	97.92	6.59130
3	7.498	40.47484	0.68	4.45559
Total		5913.27104	100.00	

(USP)

(4-(4-hydroxyphenyl)piperazin-1-yl)(1-methyl-1H-indazol-3-yl)methanone **(AI10-m19)**

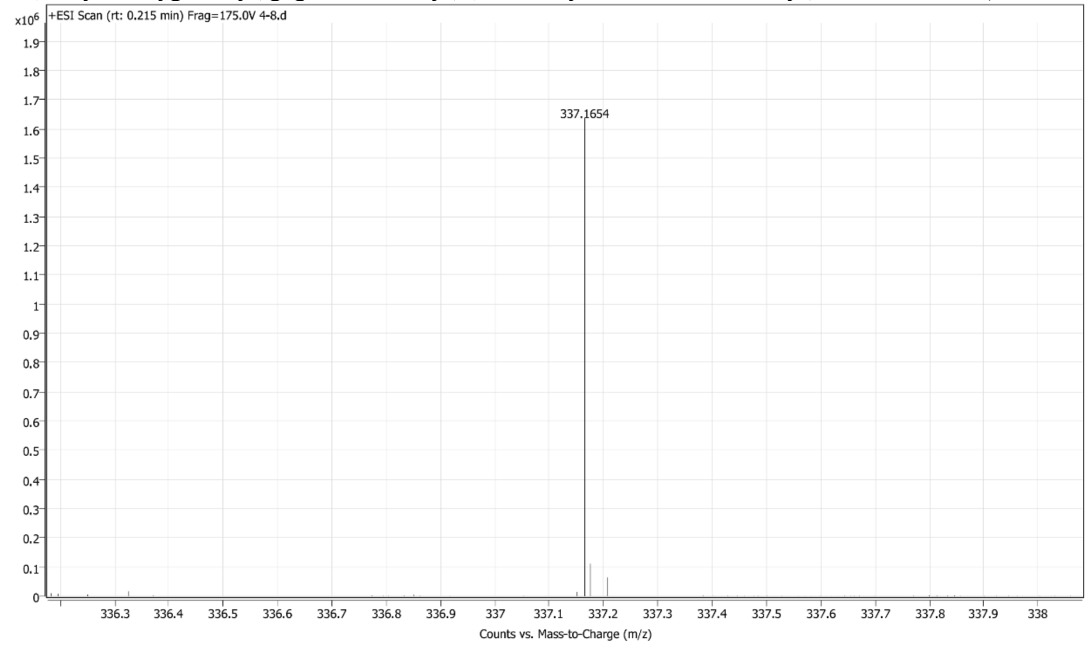

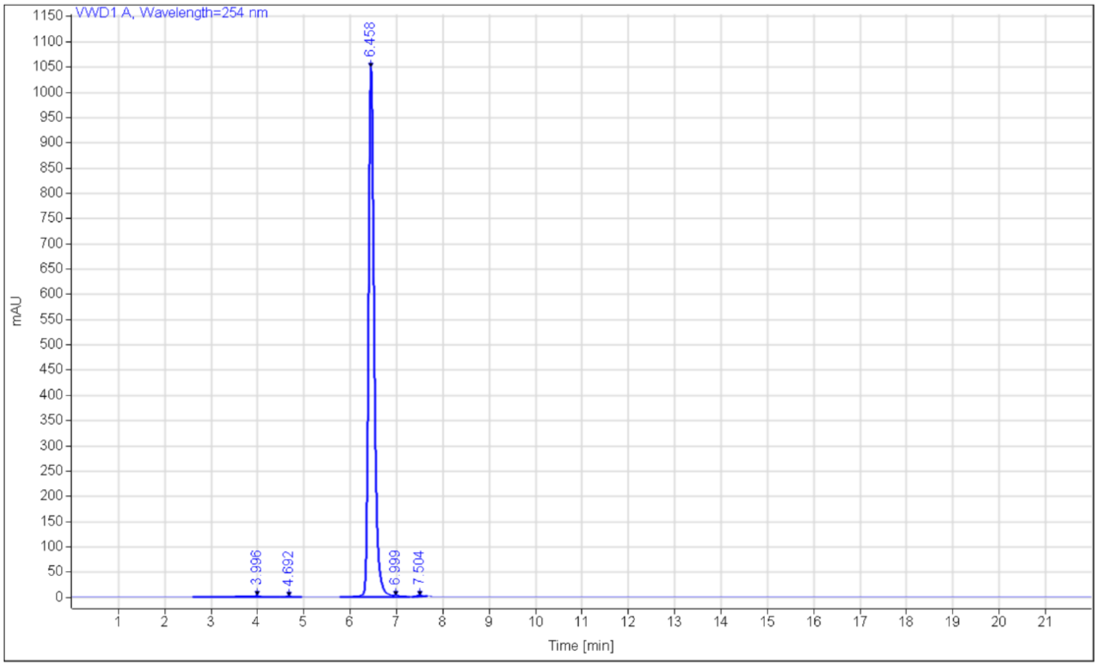

<Peak table>

Detector A Ch1 254nm

Number	Retention time	Peak area	Peak area%	Separation efficiency
1	3. 996	85. 95647	0. 95	--
2	4. 692	14. 41614	0. 16	1. 60062
3	6. 458	8918. 79590	98. 37	8. 13161
4	6. 999	26. 04619	0. 29	1. 51927
5	7. 504	21. 76230	0. 24	1. 39659
Total		9066. 97700	100. 00	

(USP)

(4-(4-hydroxyphenyl)piperazin-1-yl)(1-methyl-1H-indol-3-yl)methanone **(AI10-m20)**

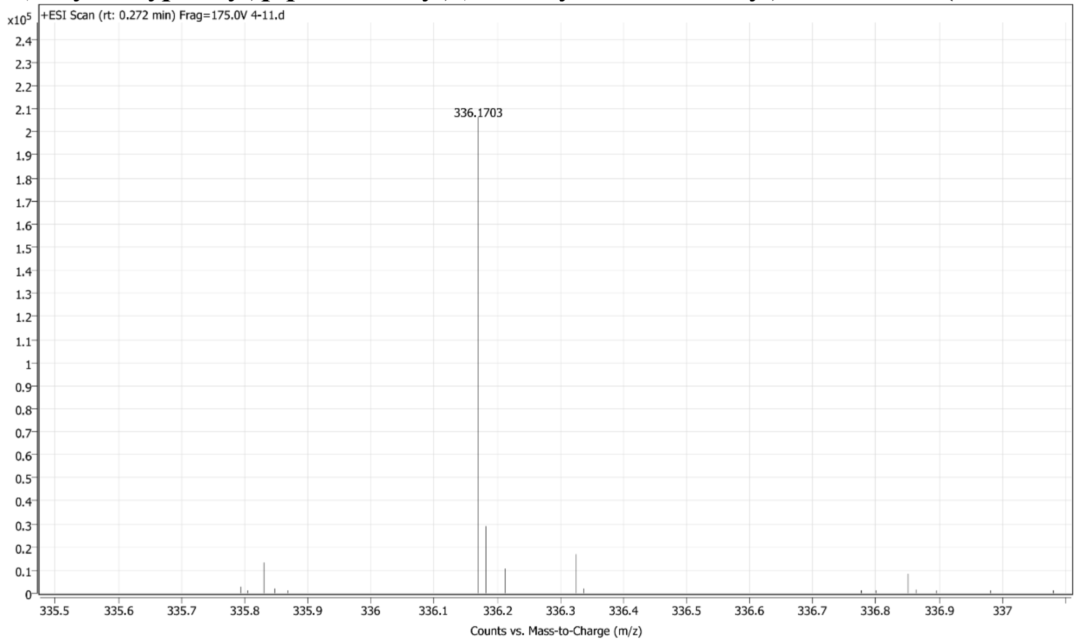

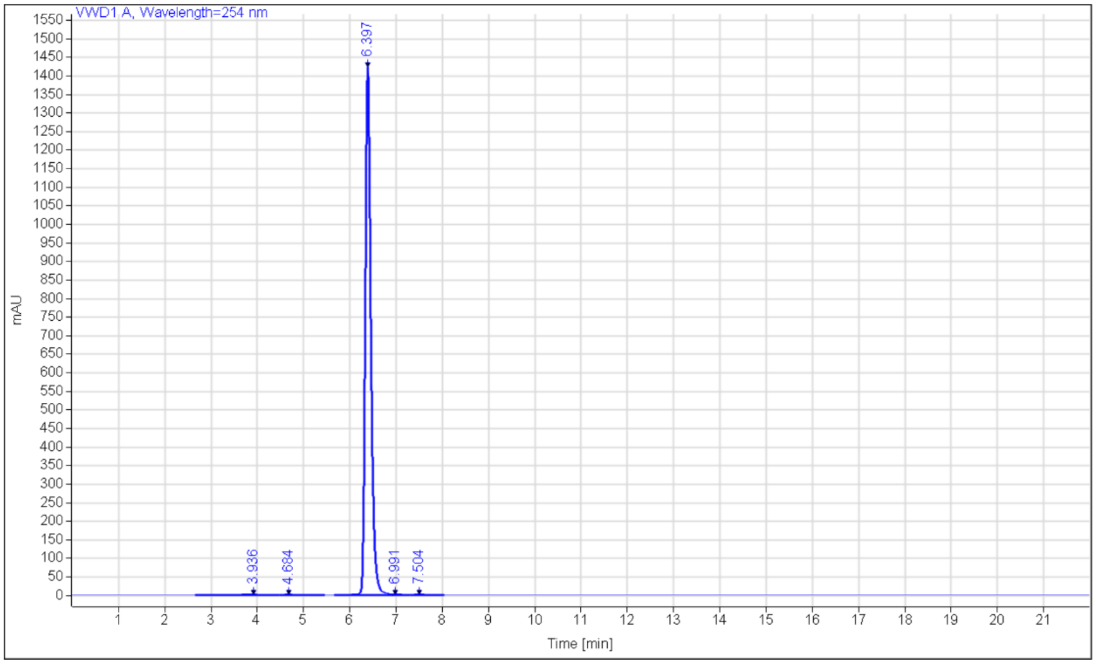

<Peak table>
Detector A Ch1 254nm

Number	Retention time	Peak area	Peak area%	Separation efficiency
1	3. 926	70. 95599	0. 58	--
2	4. 684	28. 04492	0. 23	1. 99121
3	6. 397	12056. 30664	98. 58	7. 81334
4	6. 991	34. 64684	0. 28	1. 15582
5	7. 504	39. 91316	0. 33	0. 93779
Total		12229. 86755	100. 00	

(USP)

4-(4-(2-fluorobenzyl)piperazin-1-yl)phenol **(AI10-m21)**

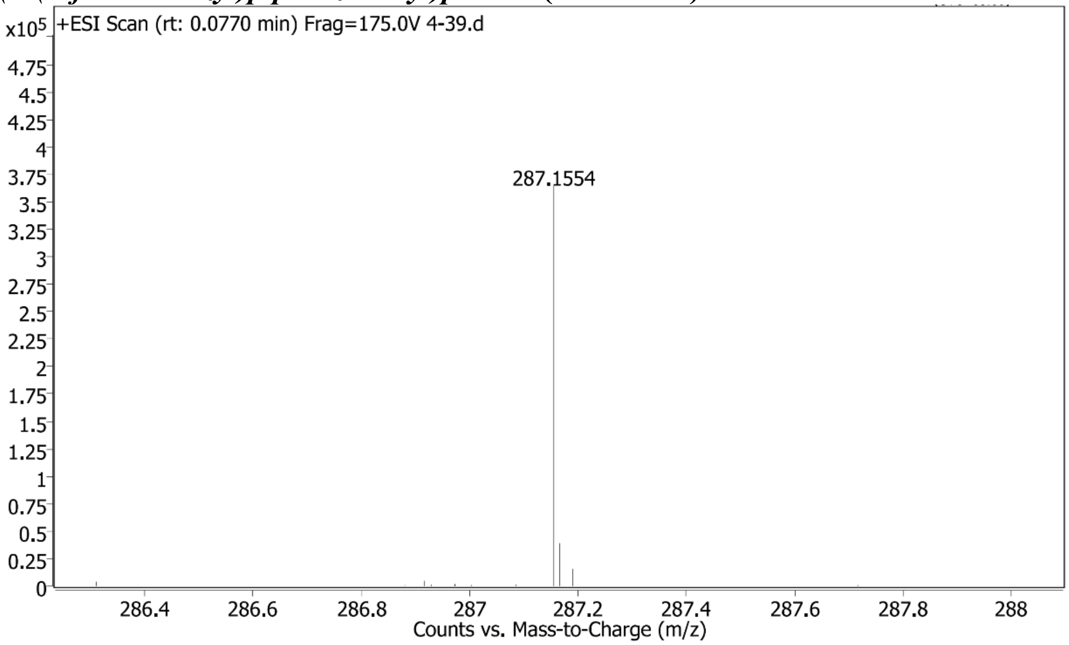

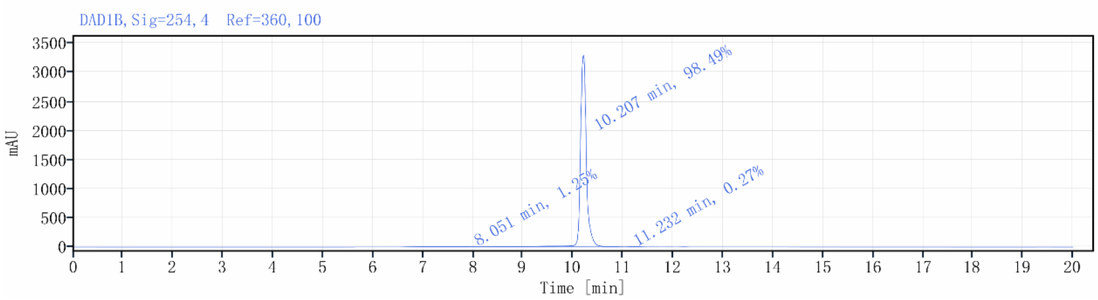

<Peak table>
Detector A Ch1 254nm

Number	Retention time	Peak area	Peak area%	Separation efficiency
1	8. 051	341. 3	1. 25	--
2	10. 207	26978. 3	98. 49	--
3	11. 232	72. 7	0. 27	
Total		27392. 4	100. 00	

(USP)

4-(4-(4-fluorobenzyl)piperazin-1-yl)phenol **(AI10-m22)**

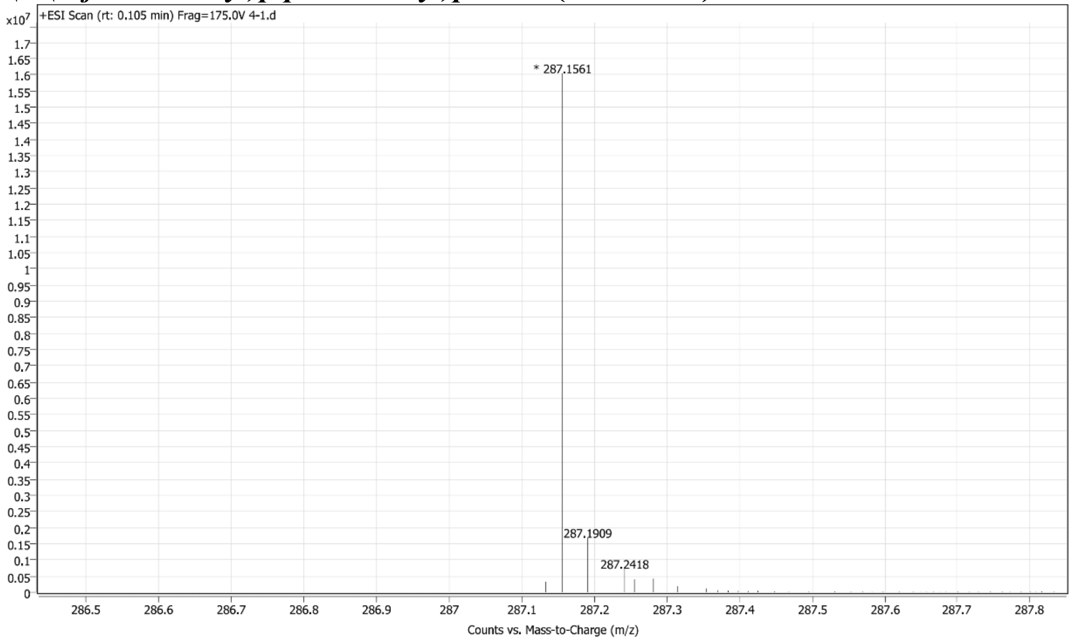

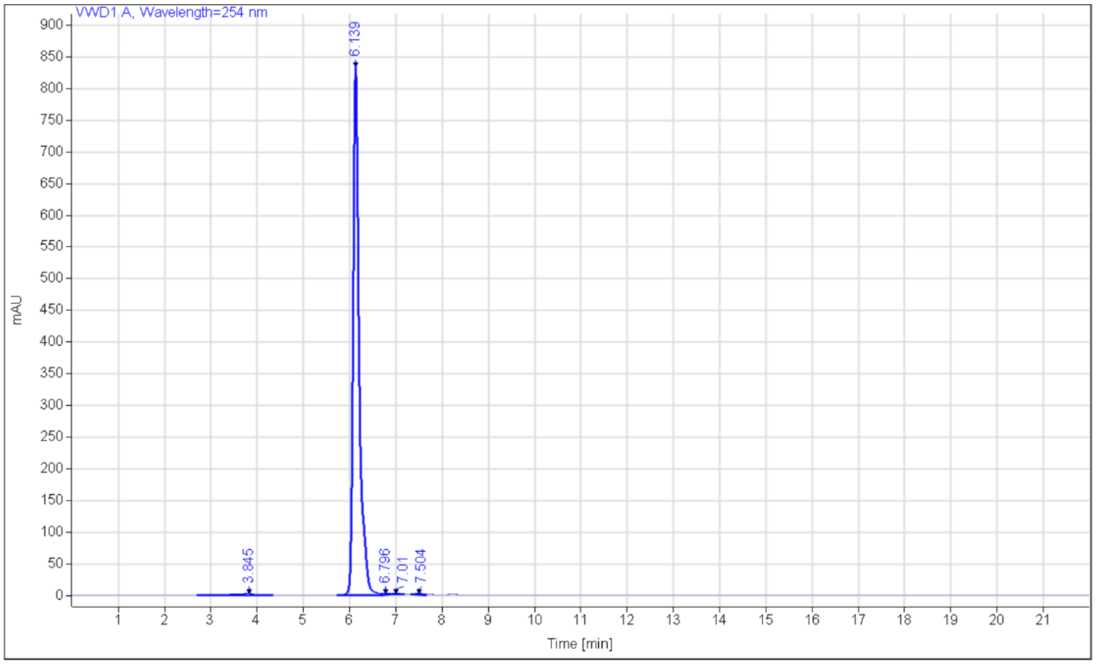

<Peak table>

Detector A Ch1 254nm

Number	Retention time	Peak area	peak area%	Separation efficiency
1	3. 845	66. 71871	0. 87	--
2	6. 139	7553. 68408	98. 24	6. 69659
3	6. 796	24. 62466	0. 32	2. 37484
4	7. 010	20. 25782	0. 26	0. 72906
5	7. 504	23. 55406	0. 31	2. 01149
Total		7688. 83933	100. 000	

(USP)

4-(4-(3-fluorobenzyl)piperazin-1-yl)phenol **(AI10-m23)**

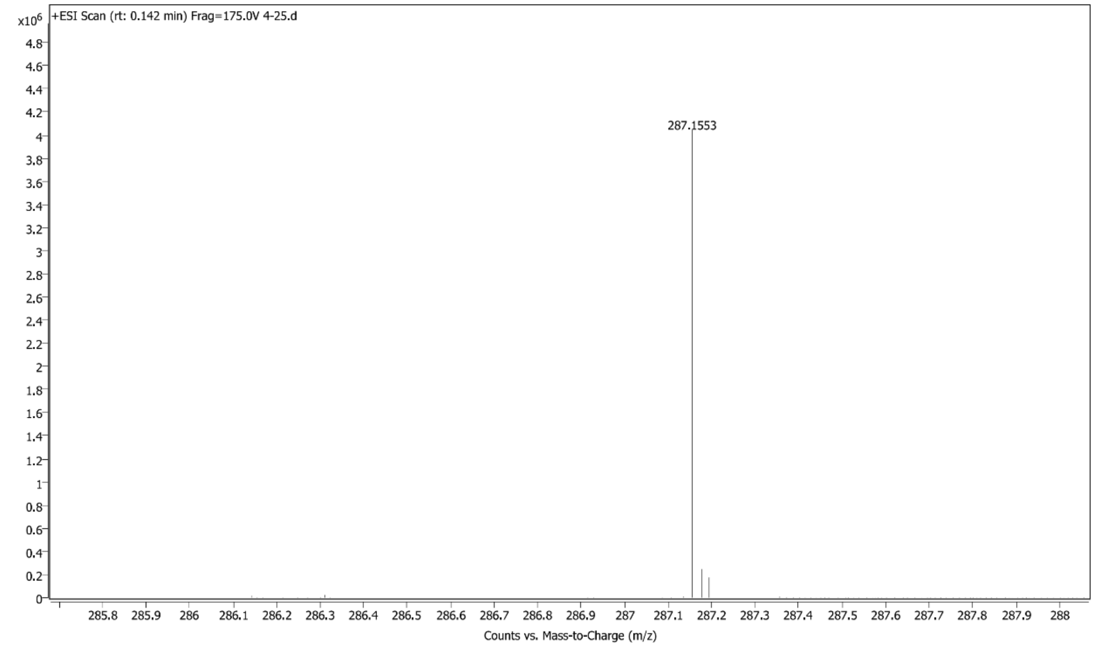

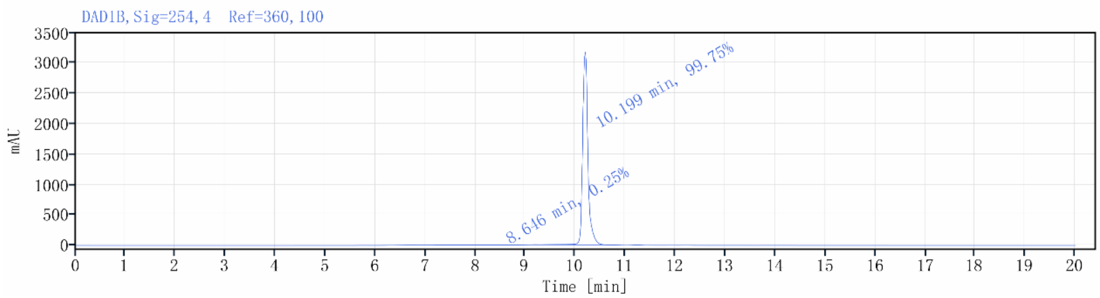

<Peak table>

Detector A Ch1 254nm

Number	Retention time	Peak area	Peak area%	Separation efficiency
1	8. 646	59. 2	0. 25	--
2	10. 199	23661. 0	99. 75	--
Total		23720. 2	100. 00	

(USP)

4-(4-(2-chlorobenzyl)piperazin-1-yl)phenol **(AI10-m24)**

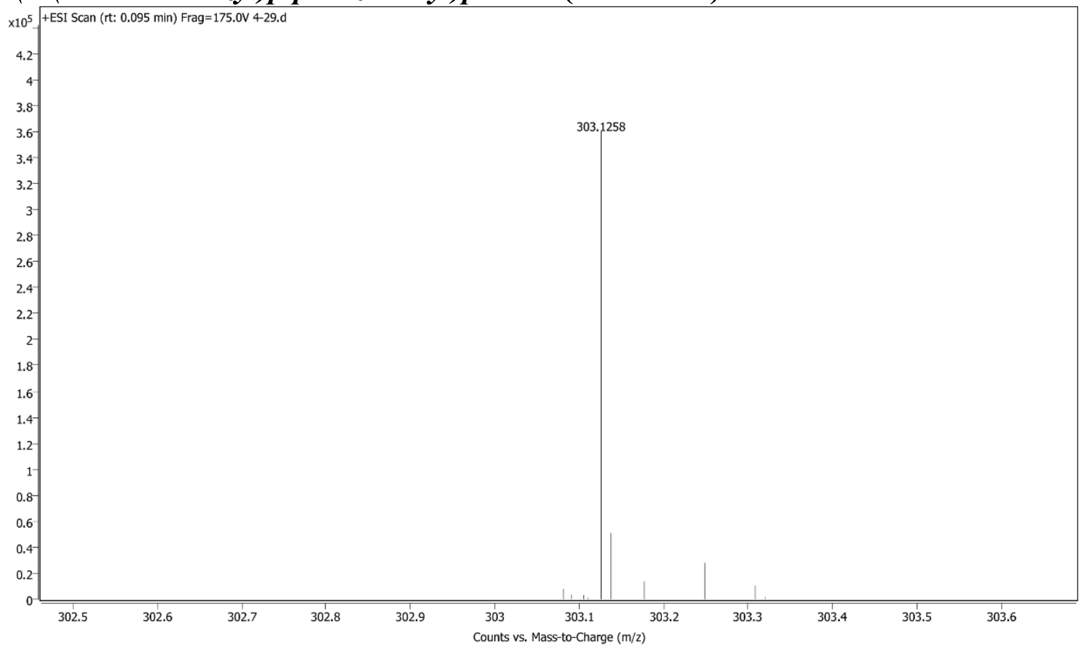

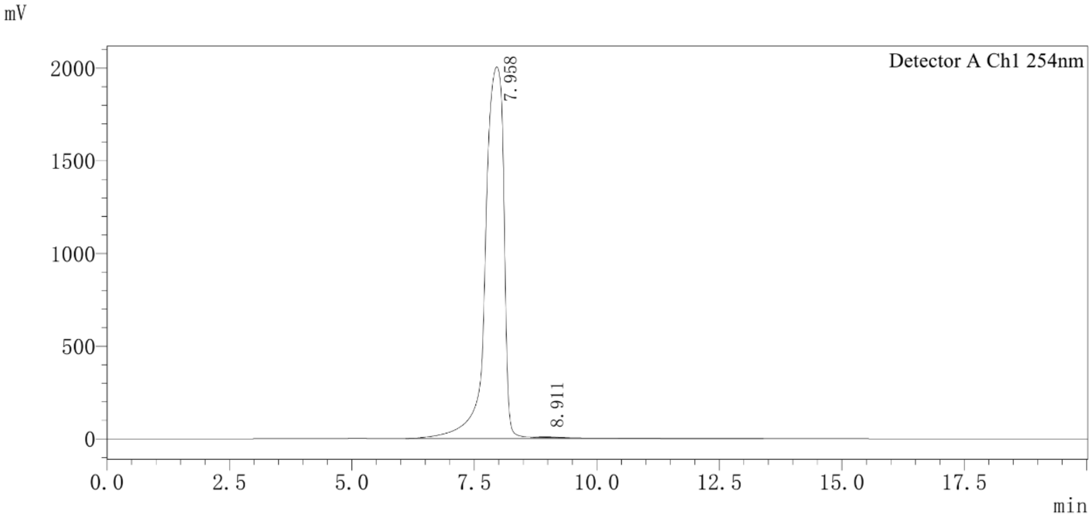

<Peak table>

Detector A Ch1 254nm

Number	Retention time	Peak area	Peak area%	Separation efficiency
1	7.958	53669725	99.843	--
2	8.911	84140	0.157	1.748
Total		53753864	100.000	

(USP)

***4-(4-(3-chlorobenzyl)piperazin-1-yl)phenol* (AI10-m25)**

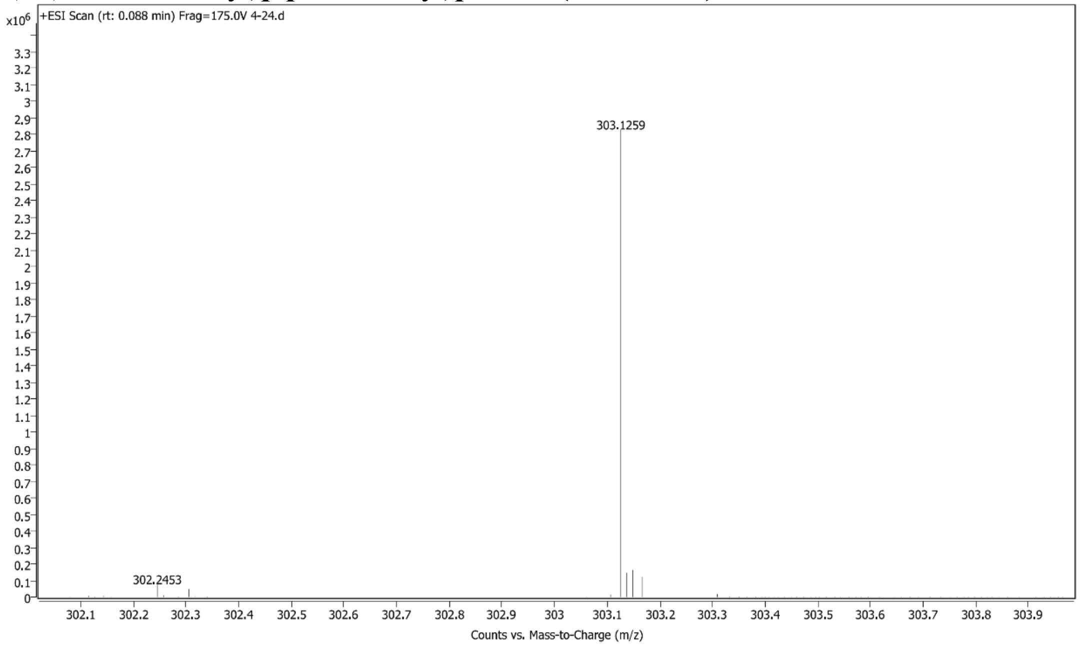

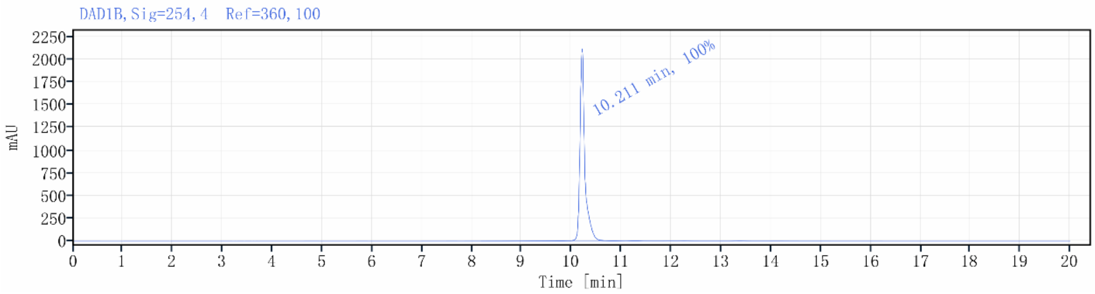

<Peak table>
Detector A Ch1 254nm

Number	Retention time	Peak area	Peak area%	Separation efficiency
1	10. 211	14926. 1	100. 00	--
Total		14926. 1	100. 00	

(USP)

4-(4-(4-chlorobenzyl)piperazin-1-yl)phenol **(AI10-m26)**

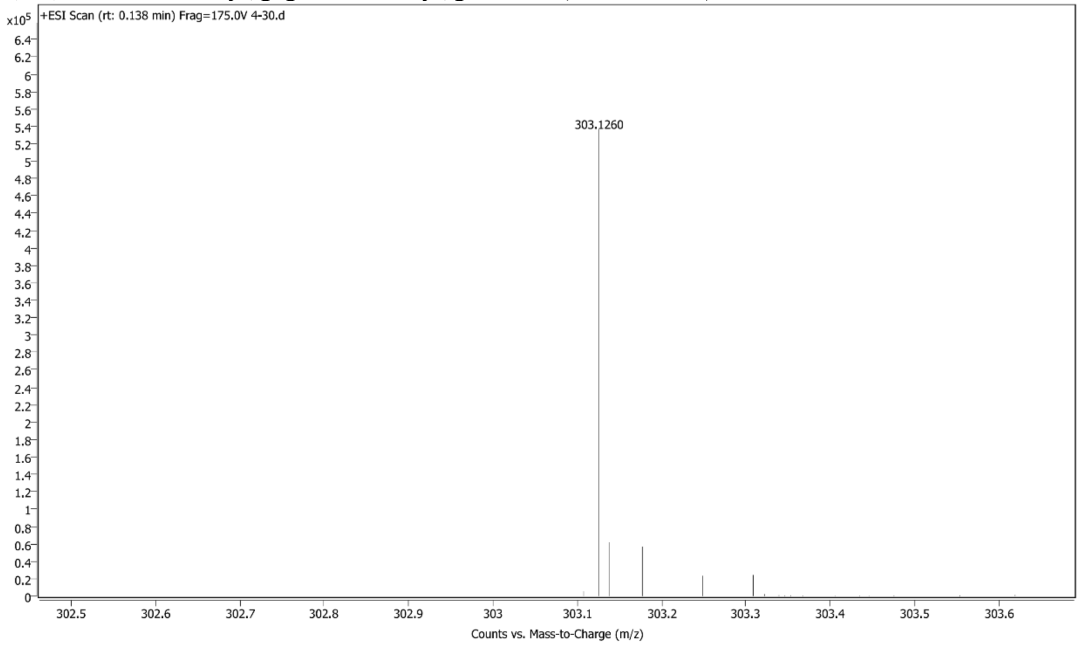

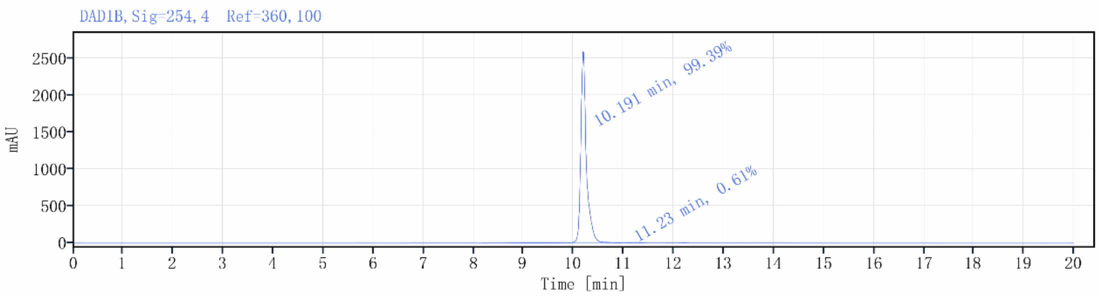

<Peak table>

Detector A Ch1 254nm

Number	Retention time	Peak area	Peak area%	Separation efficiency
1	10. 191	20897. 0	99. 39	--
2	11. 230	128. 4	0. 61	--
Total		21025. 4	100. 00	

(USP)

4-(4-(2-methylbenzyl)piperazin-1-yl)phenol **(AI10-m27)**

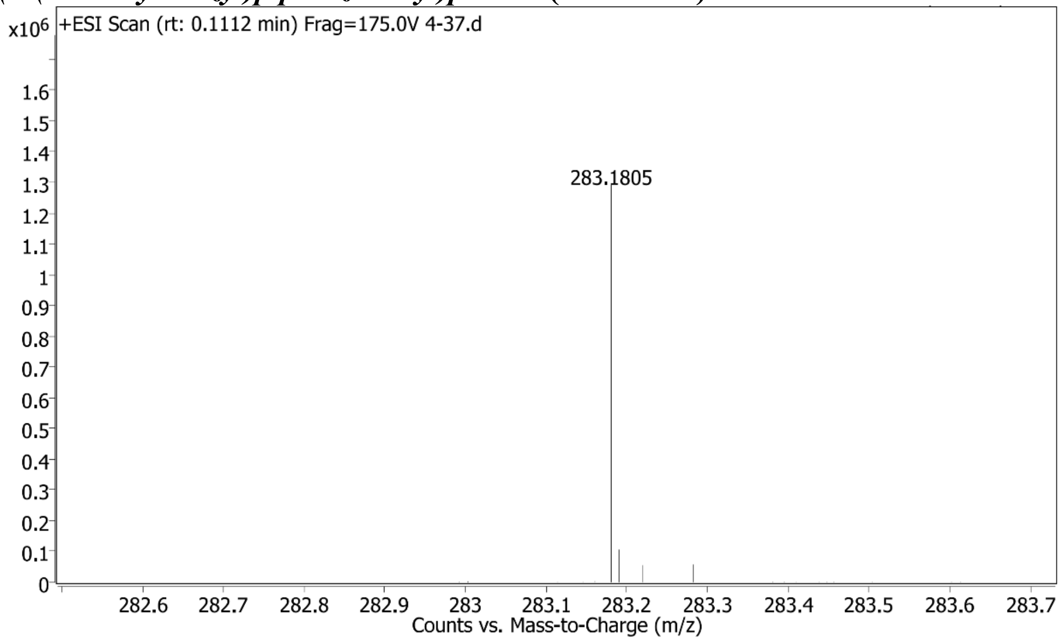

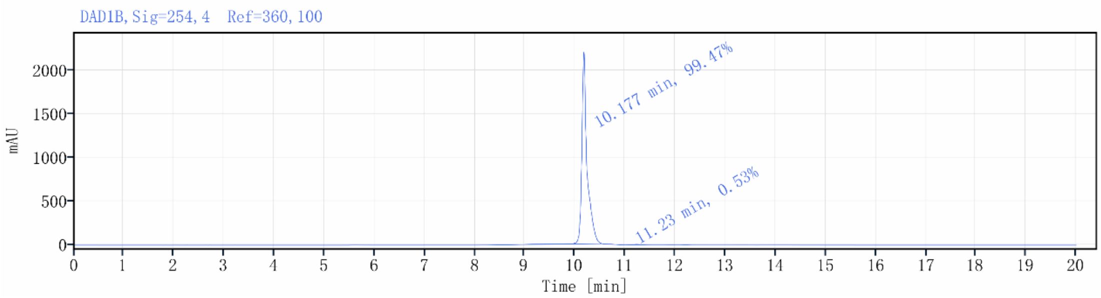

<Peak table>

Detector A Ch1 254nm

Number	Retention time	Peak area	Peak area%	Separation efficiency
1	10. 177	16235. 3	99. 47	--
2	11. 230	87. 3	0. 53	--
Total		16322. 6	100. 00	

(USP)

***4-(4-(3-methylbenzyl)piperazin-1-yl)phenol* (AI10-m28)**

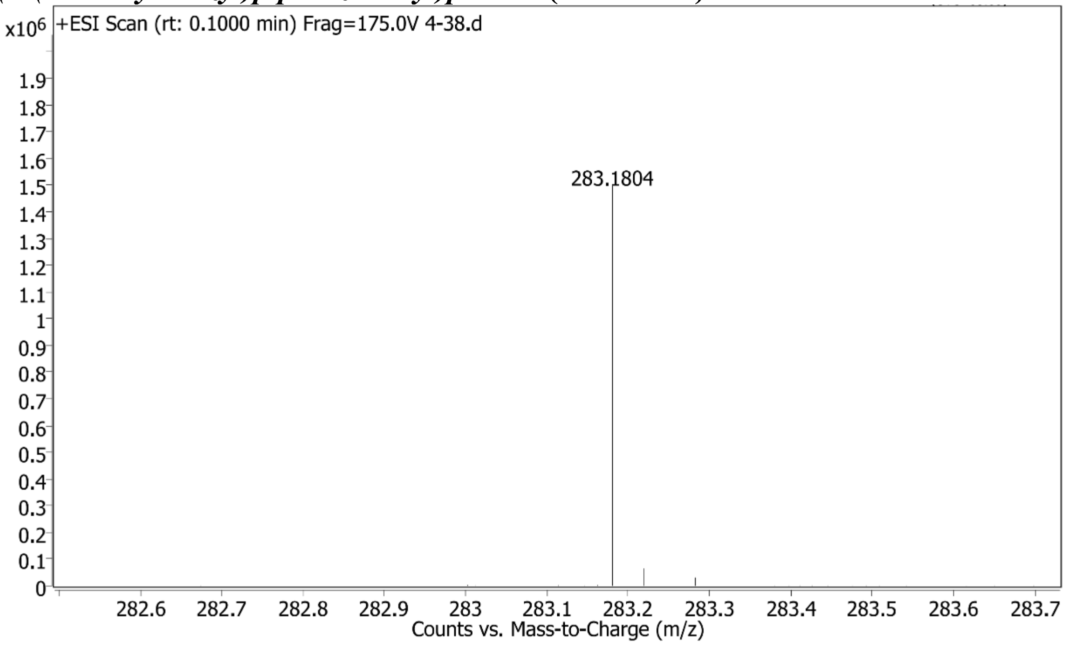

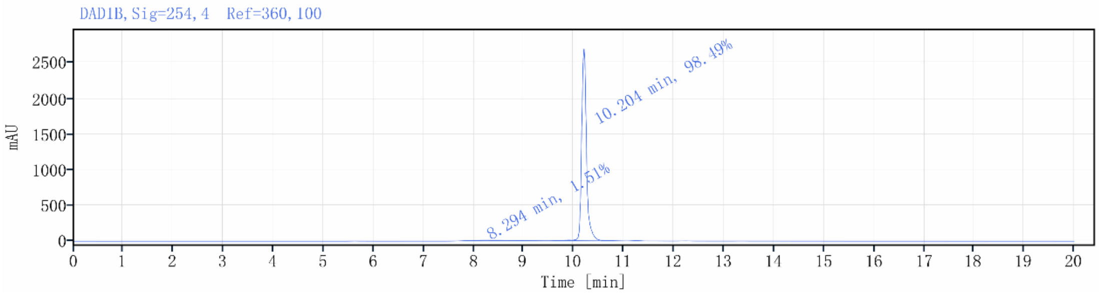

<Peak table>

Detector A Ch1 254nm

Number	Retention time	Peak area	Peak area%	Separation efficiency
1	8. 294	280. 3	1. 51	--
2	10. 204	18303. 1	98. 49	--
Total		18583. 4	100. 00	

(USP)

***4-(4-(4-methylbenzyl)piperazin-1-yl)phenol* (AI10-m29)**

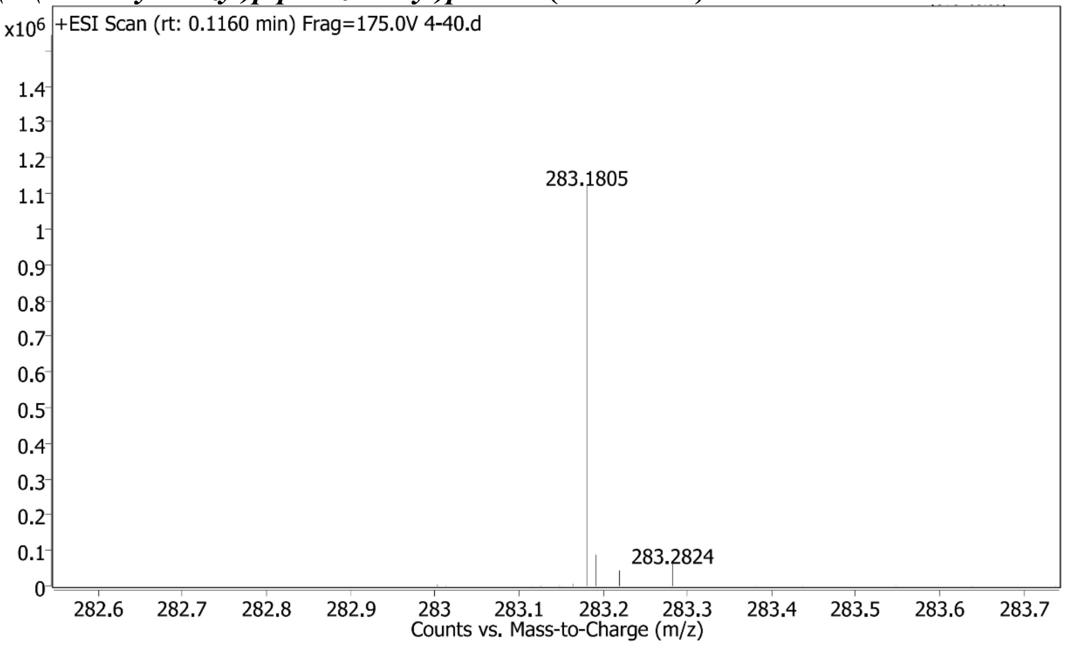

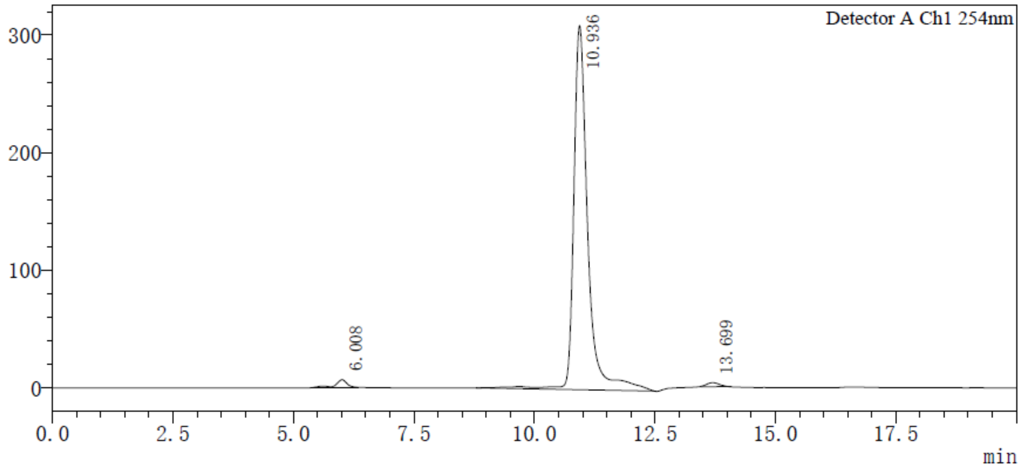

<Peak table>
Detector A Ch1 254nm

Number	Retention time	Peakarea	Peakarea%	Separation efficiency
1	6.008	103504	1.616	--
2	10.936	6239078	97.428	11.750
3	13.699	61180	0.955	5.587
Total		6403762	100.000	

(USP)

4-(4-(2-methoxybenzyl)piperazin-1-yl)phenol **(AI10-m30)**

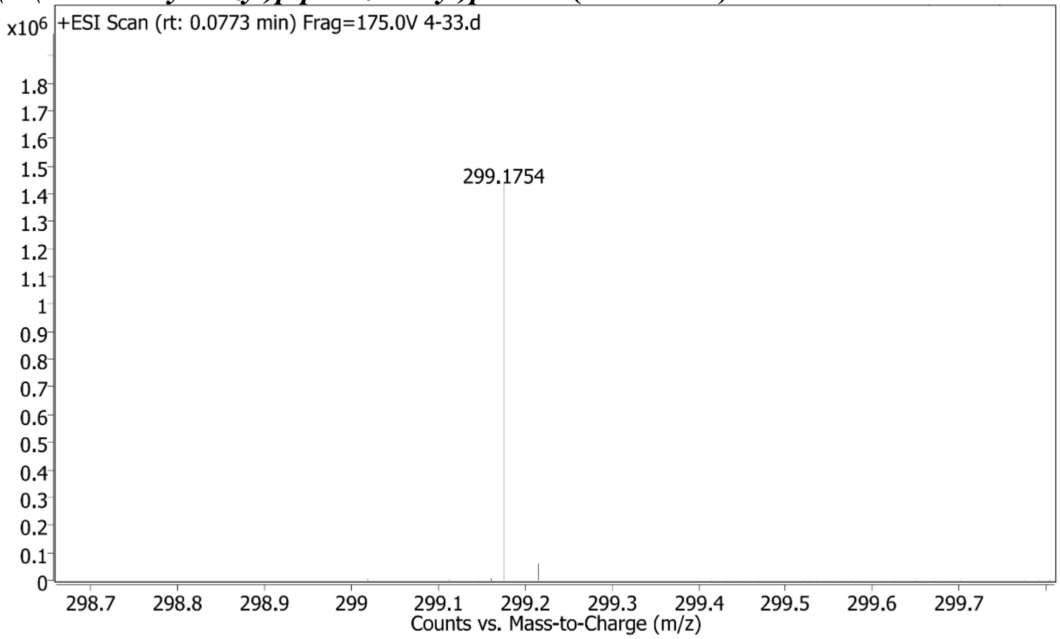

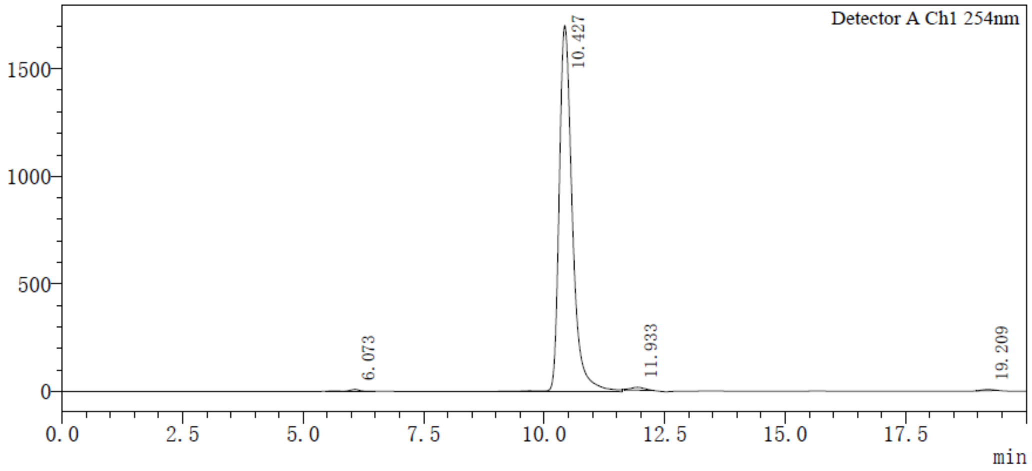

<Peak table>

Detector A Ch1 254nm

Number	Retention time	Peakarea	Peakarea%	Separation efficiency
1	6.073	191565	0.547	--
2	10.427	32270152	98.346	10.070
3	11.933	282119	0.809	2.890
4	19.209	90613	0.298	13.842
Total		32834449	100.000	

(USP)

4-(4-(3-methoxybenzyl)piperazin-1-yl)phenol **(AI10-m31)**

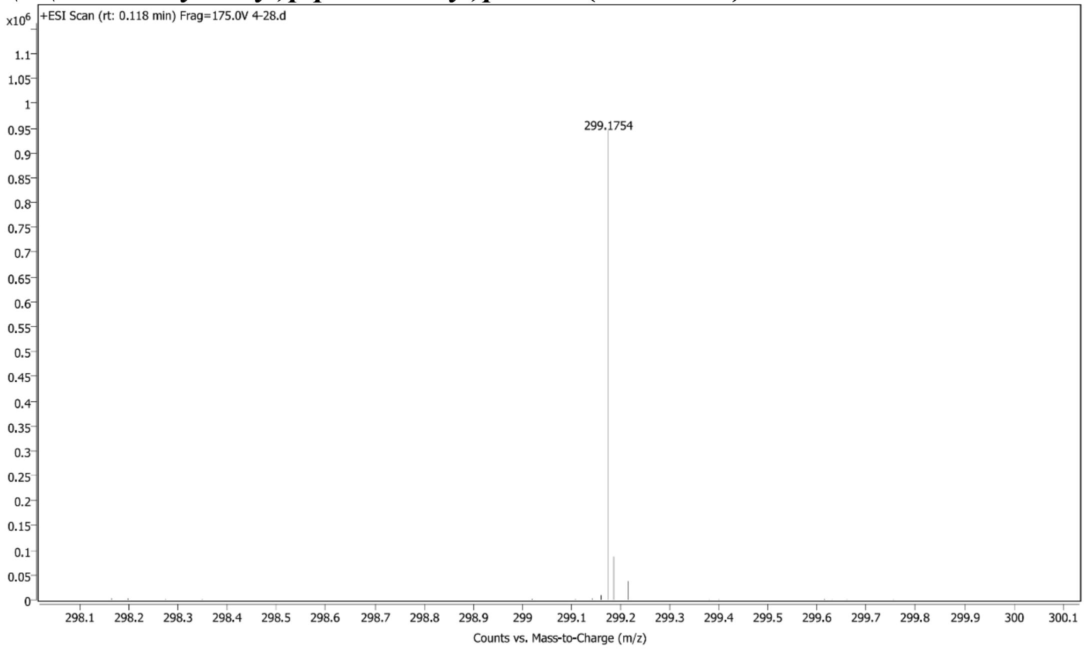

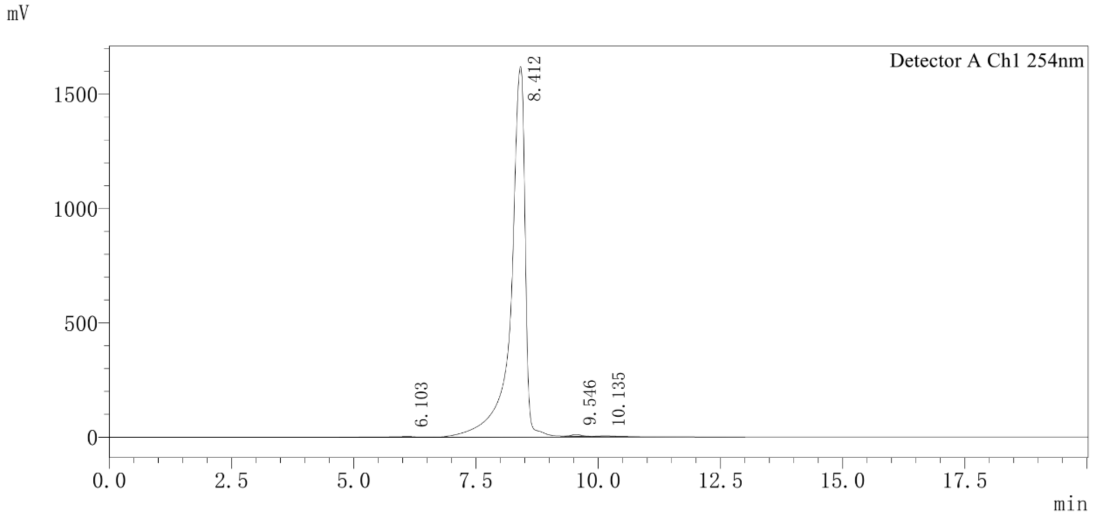

<Peak table>

Detector A Ch1 254nm

Number	Retention time	Peak area	Peak area%	Separation efficiency
1	6.103	101339	0.305	--
2	8.412	32987624	99.241	3.982
3	9.546	102188	0.307	2.854
4	10.135	48876	0.147	1.331
Total		33240027	100.000	

(USP)

4-(4-(2-ethoxybenzyl)piperazin-1-yl)phenol **(AI10-m32)**

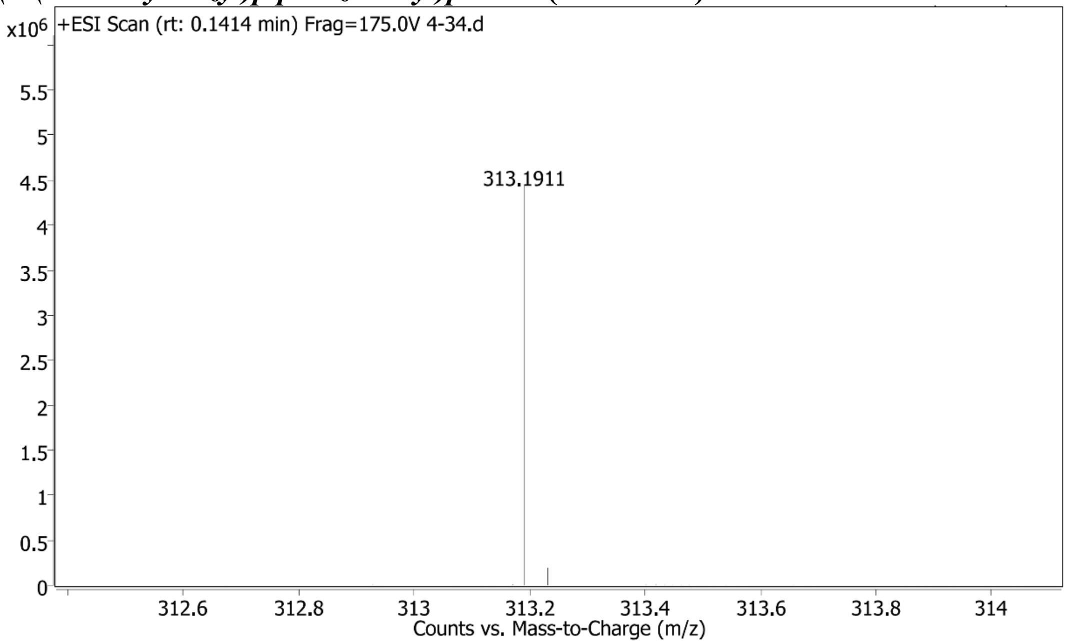

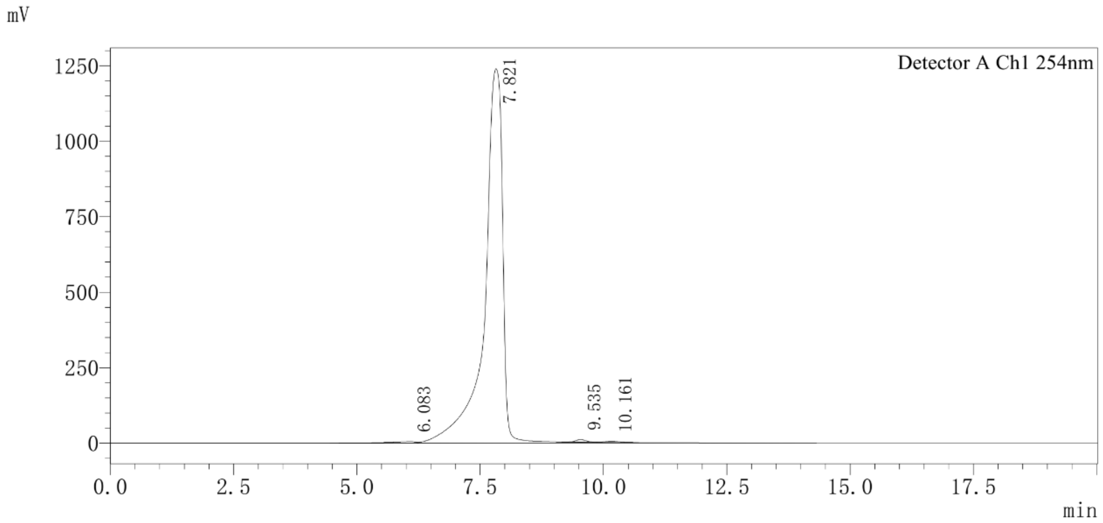

<Peak table>

Detector A Ch1 254nm

Number	Retention time	Peak area	Peak area%	Separation efficiency
1	6.083	183858	0.568	--
2	7.821	31977089	98.818	2.653
3	9.535	124567	0.385	3.865
4	10.161	74046	0.229	1.375
Total		32359560	100.000	

(USP)

***4-(4-(3-ethoxybenzyl)piperazin-1-yl)phenol* (AI10-m33)**

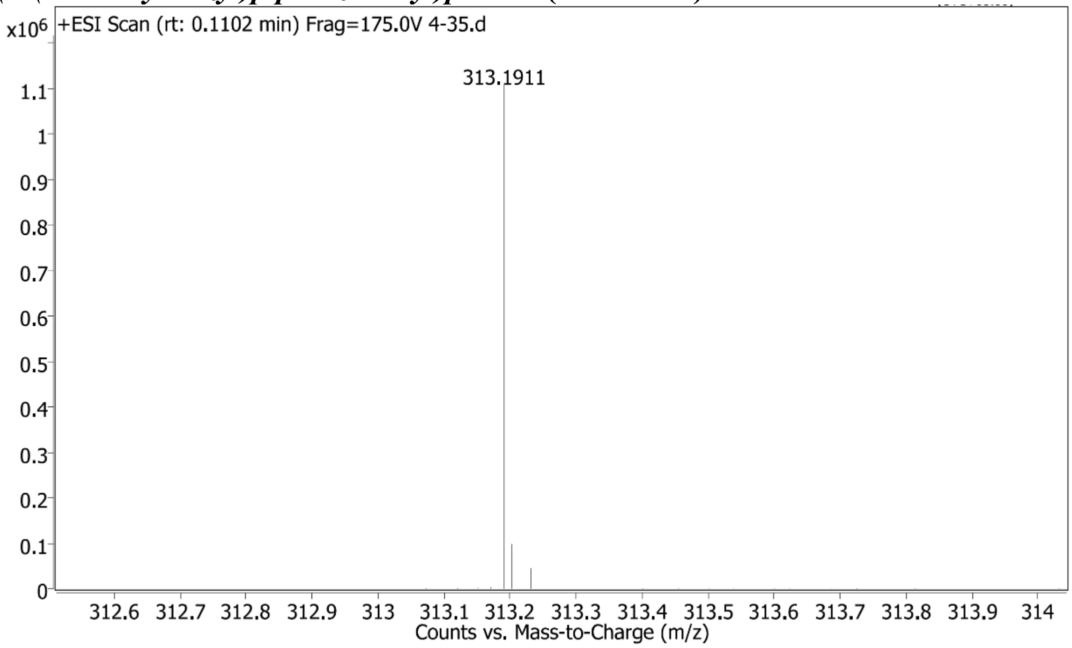

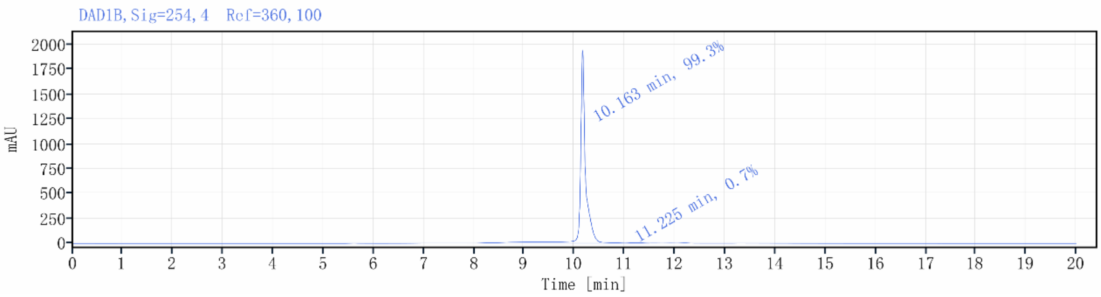

<Peak table>

Detector A Ch1 254nm

Number	Retention time	Peak area	Peak area%	Separation efficiency
1	10. 163	14052. 3	99. 30	--
2	11. 225	99. 3	0. 70	--
Total		14151. 6	100. 00	

(USP)

***4-(4-(4-ethoxybenzyl)piperazin-1-yl)phenol* (AI10-m34)**

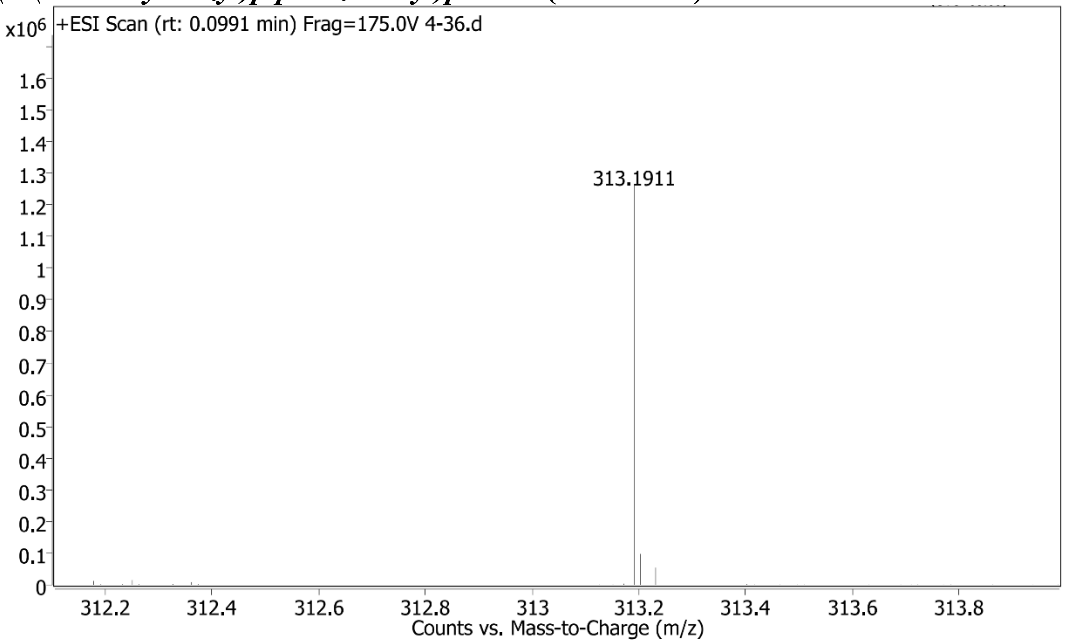

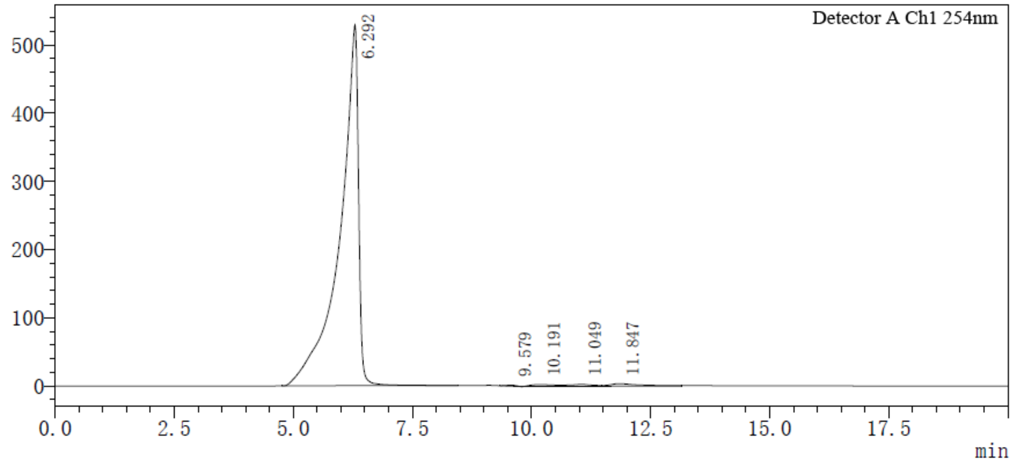

<Peak table>

Detector A Ch1 254nm

Number	Retention time	Peakarea	Peakarea%	Separation efficiency
1	6. 292	14333138	97. 520	--
2	9. 579	18701	0. 127	6. 914
3	10. 191	92777	0. 631	0. 841
4	11. 049	98310	0. 669	0. 819
5	11. 847	154666	1. 052	0. 796
Total		14697592	100. 000	

(USP)

***3-((4-(4-hydroxyphenyl)piperazin-1-yl)methyl)phenol* (AI10-m35)**

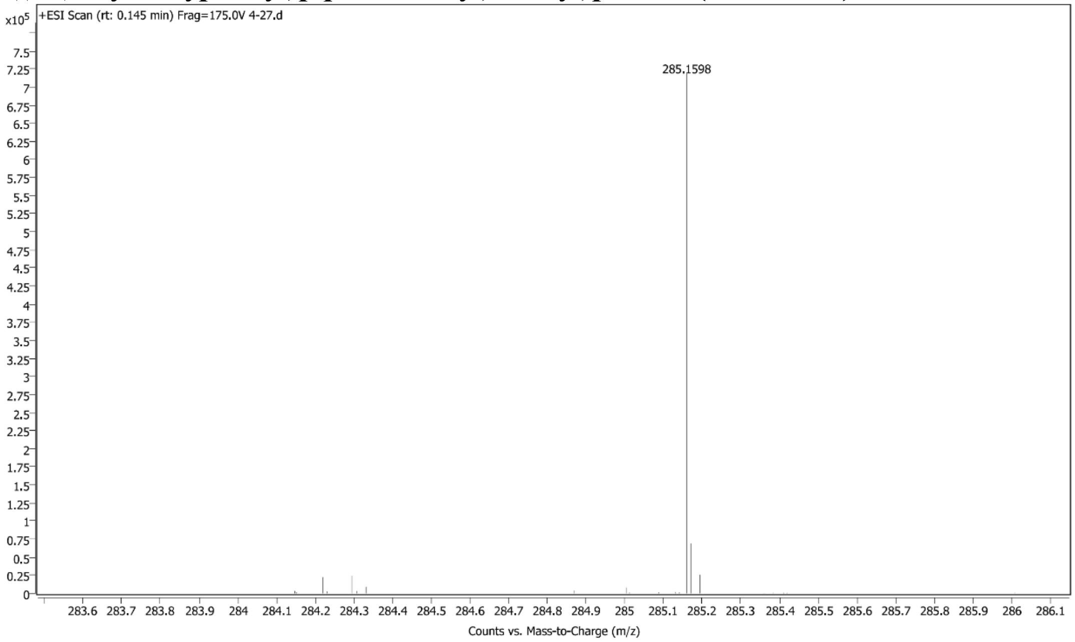

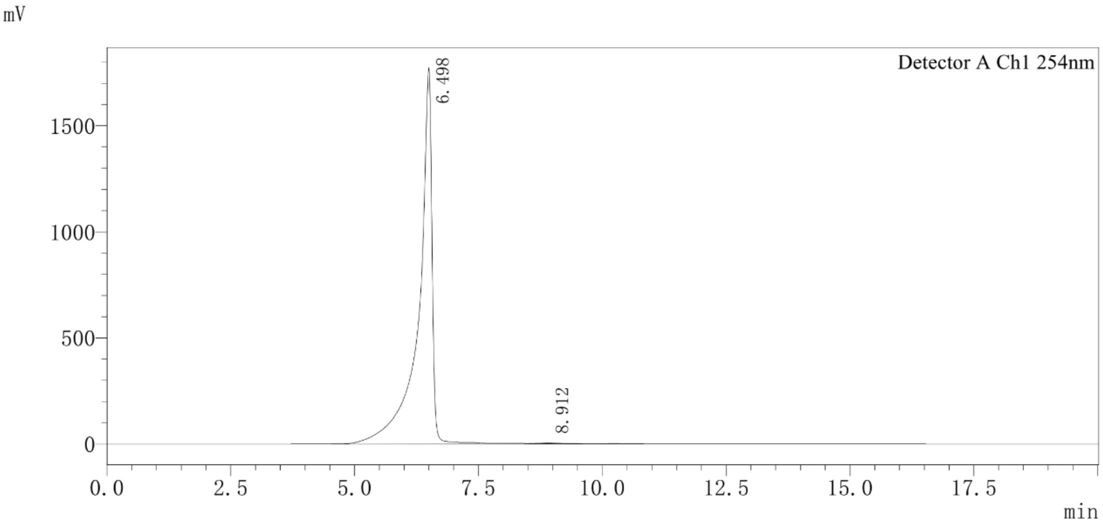

<Peak table>

Detector A Ch1 254nm

Number	Retention time	Peak area	Peak area%	Separation efficiency
1	6.498	33292931	99.757	--
2	8.912	81102	0.243	5.423
Total		33374033	100.000	

(USP)

4-(4-(4-hydroxybenzyl)piperazin-1-yl)phenol (AI10-m36)

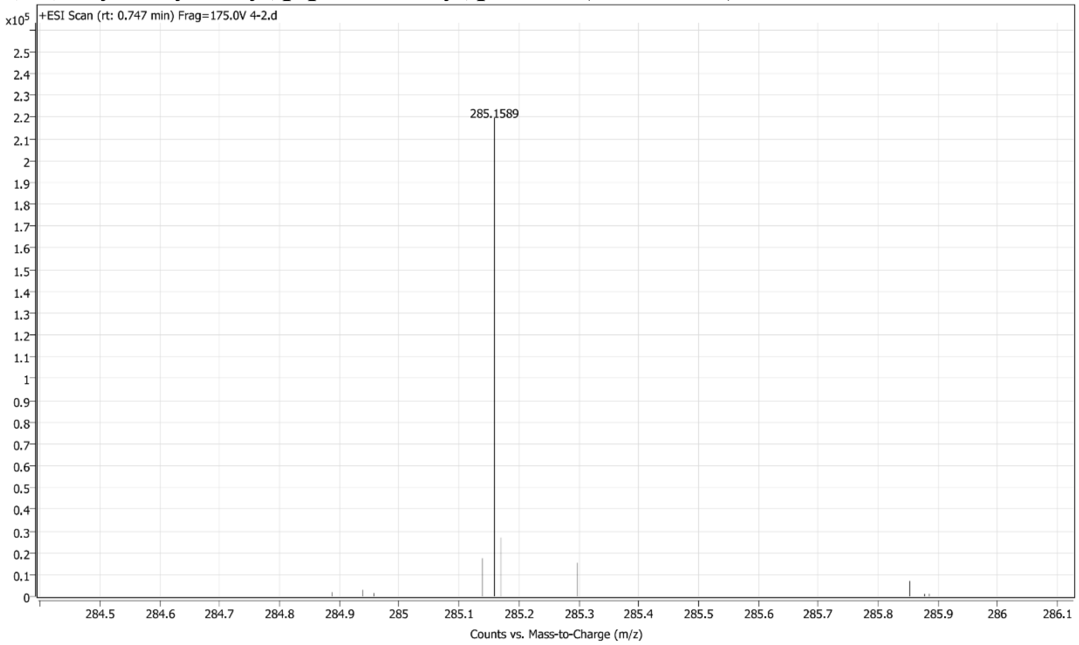

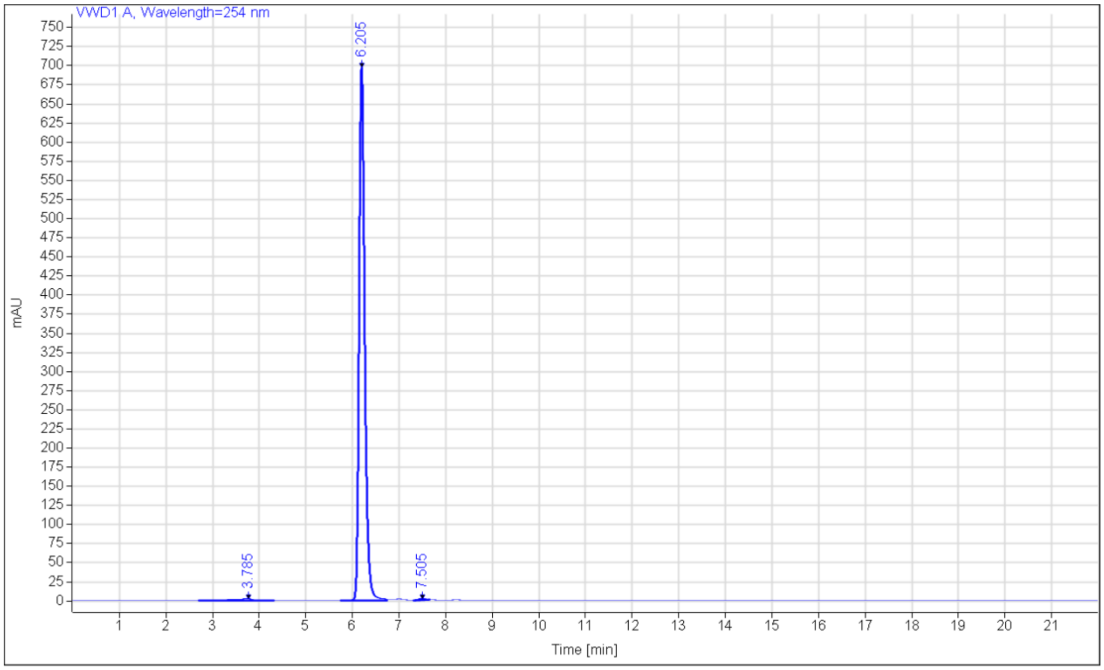

<Peak table>

Detector A Ch1 254nm

Number	Retention time	Peak area	Peak area%	Separation efficiency
1	3. 785	59. 38832	1. 00	--
2	6. 205	5845. 19873	98. 70	6. 68215
3	7. 505	17. 75208	0. 30	5. 91088
Total		45853834	100. 000	

(USP)

2-((4-(4-hydroxyphenyl)piperazin-1-yl)methyl)benzonitrile **(AI10-m37)**

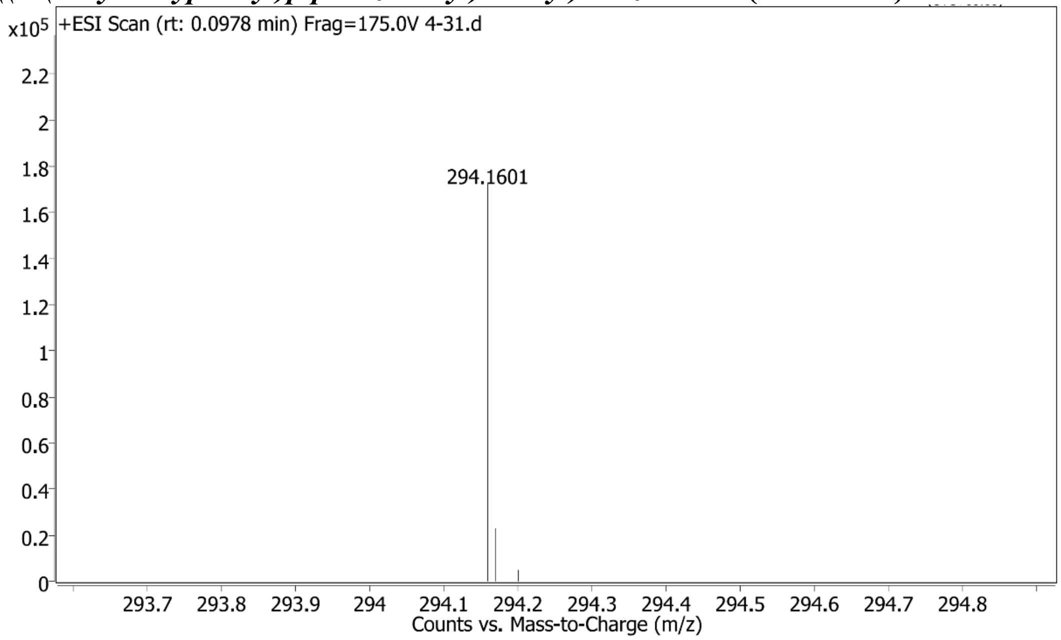

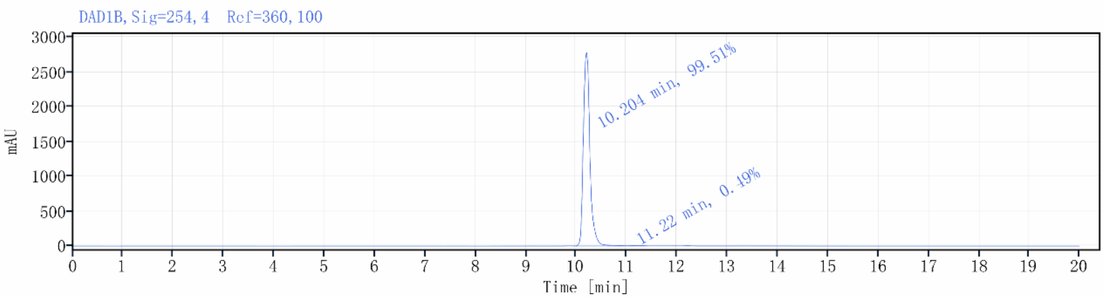

<Peak table>

Detector A Ch1 254nm

Number	Retention time	Peak area	Peak area%	Separation efficiency
1	10. 204	25257. 4	99. 51	--
2	11. 220	123. 2	0. 49	--
Total		25380. 6	100. 00	

(USP)

3-((4-(4-hydroxyphenyl)piperazin-1-yl)methyl)benzonitrile **(AI10-m38)**

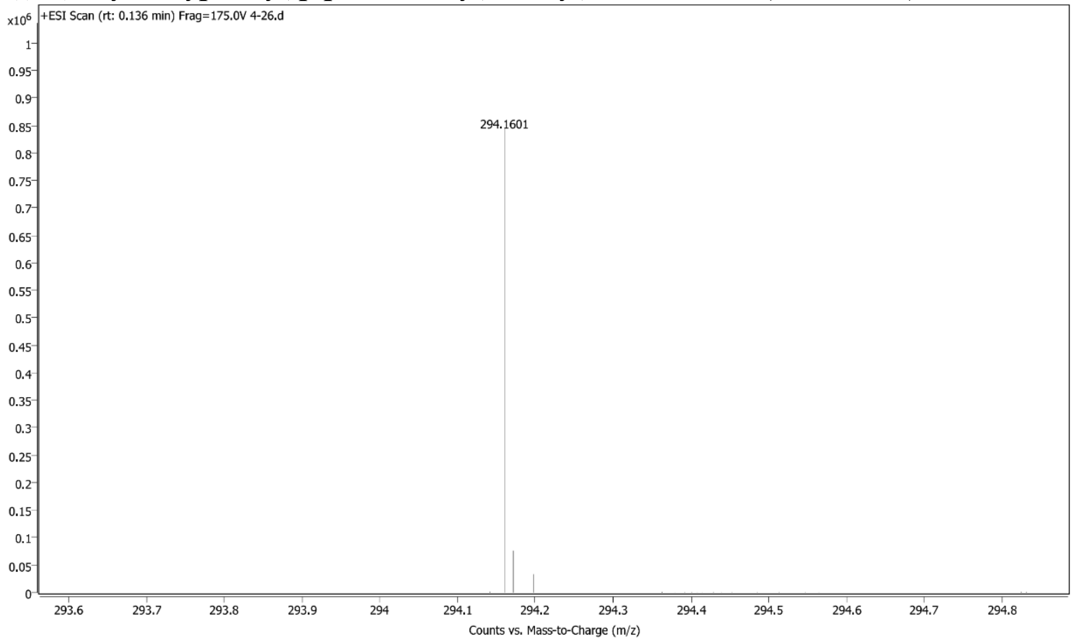

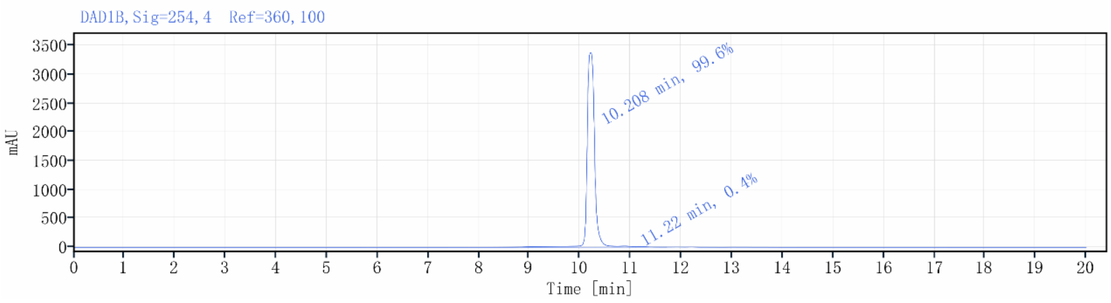

<Peak table>

Detector A Ch1 254nm

Number	Retention time	Peak area	Peak area%	Separation efficiency
1	10. 208	34566. 1	99. 60	--
2	11. 220	138. 5	0. 40	--
Total		34704. 6	100. 00	

(USP)

4-((4-(4-hydroxyphenyl)piperazin-1-yl)methyl)benzonitrile **(AI10-m39)**

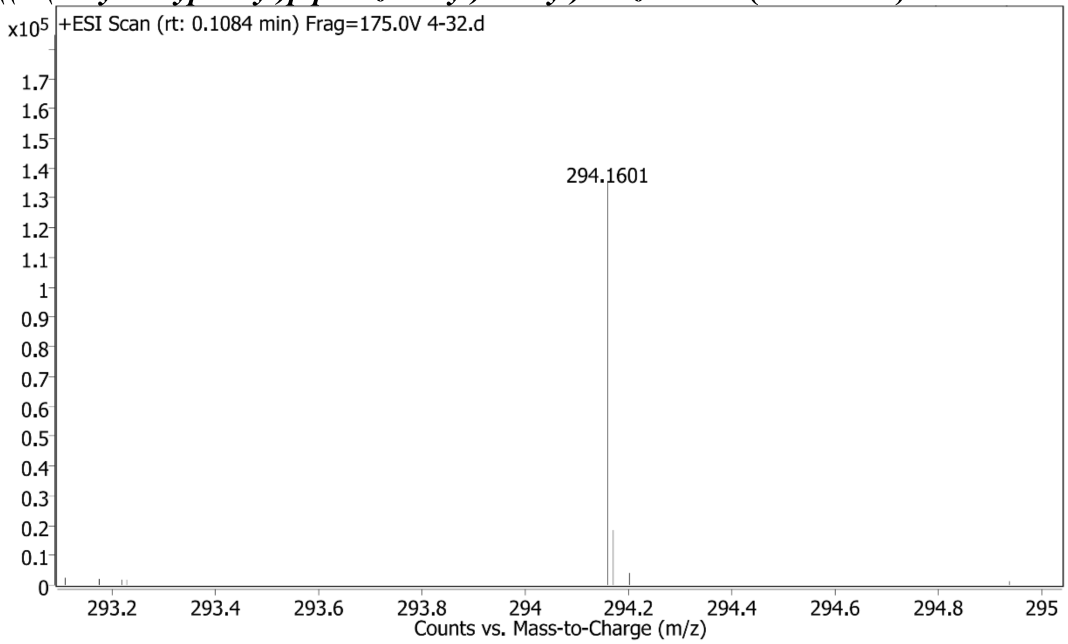

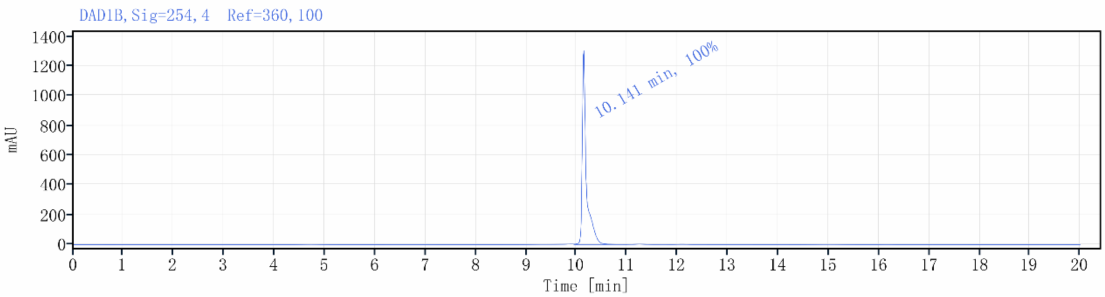

<Peak table>
Detector A Ch1 254nm

Number	Retention time	Peak area	Peak area%	Separation efficiency
1	10. 141	7544. 6	100. 00	--
Total		7544. 6	100. 00	

(USP)

4-(4-(3,5-dichlorobenzyl)piperazin-1-yl)phenol **(AI10-m40)**

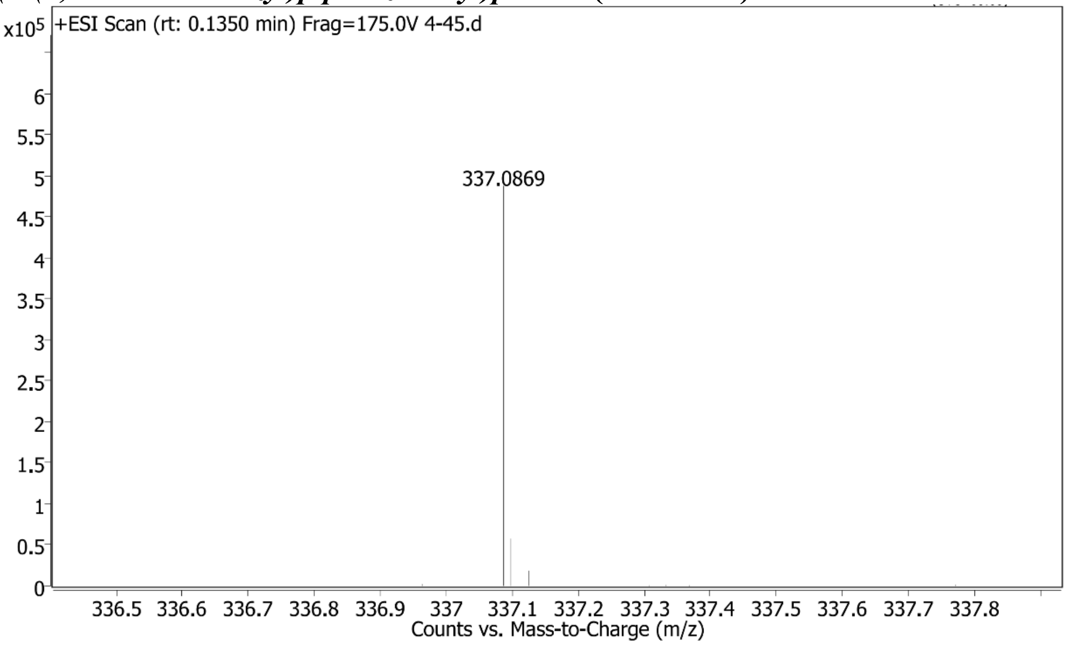

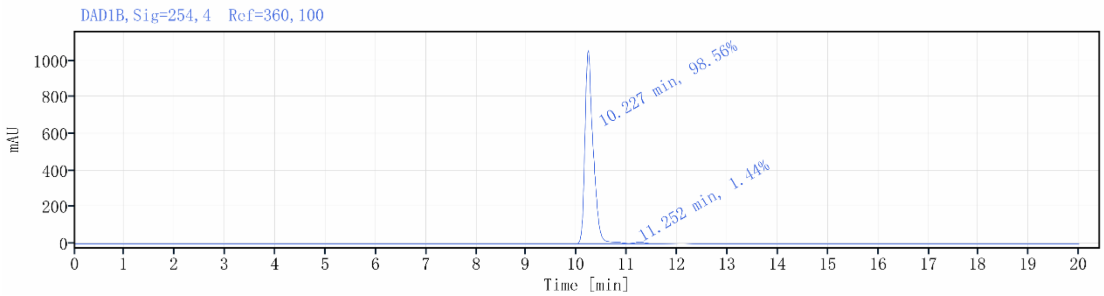

<Peak table>

Detector A Ch1 254nm

Number	Retention time	Peak area	Peak area%	Separation efficiency
1	10.227	12067.8	98.56	--
2	11.252	176.8	1.44	--
Total		12244.6	100.00	

(USP)

4-(4-(3,4-dichlorobenzyl)piperazin-1-yl)phenol **(AI10-m41)**

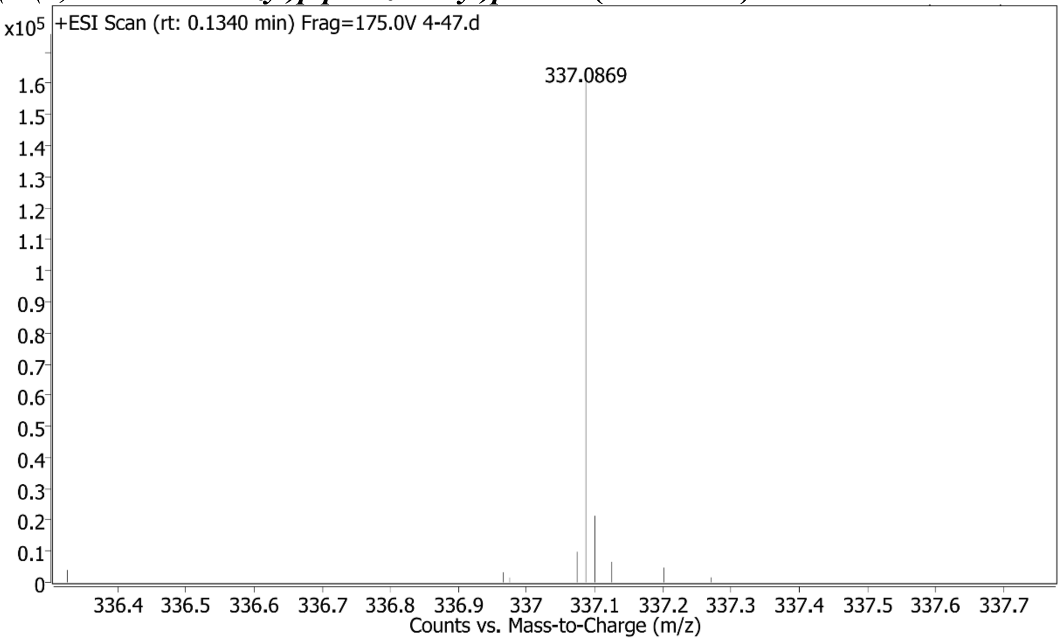

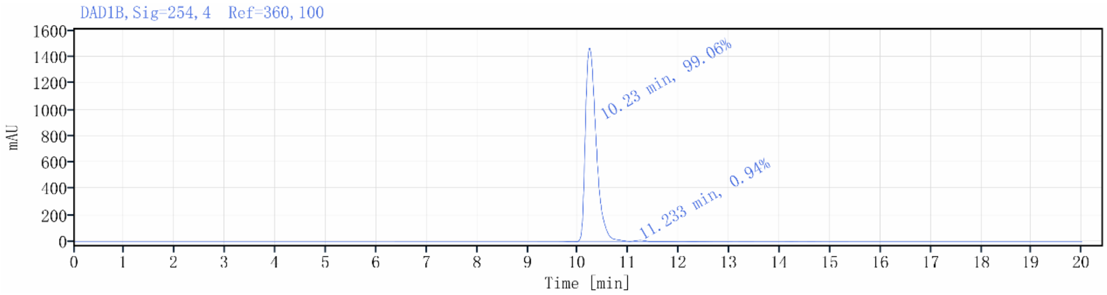

<Peak table>
Detector A Ch1 254nm

Number	Retention time	Peak area	Peak area%	Separation efficiency
1	10.230	22101.0	99.06	--
2	11.233	210.3	0.94	--
Total		22311.3	100.00	

(USP)

4-((4-(4-hydroxyphenyl)piperazin-1-yl)methyl)benzene-1,3-diol **(AI10-m42)**

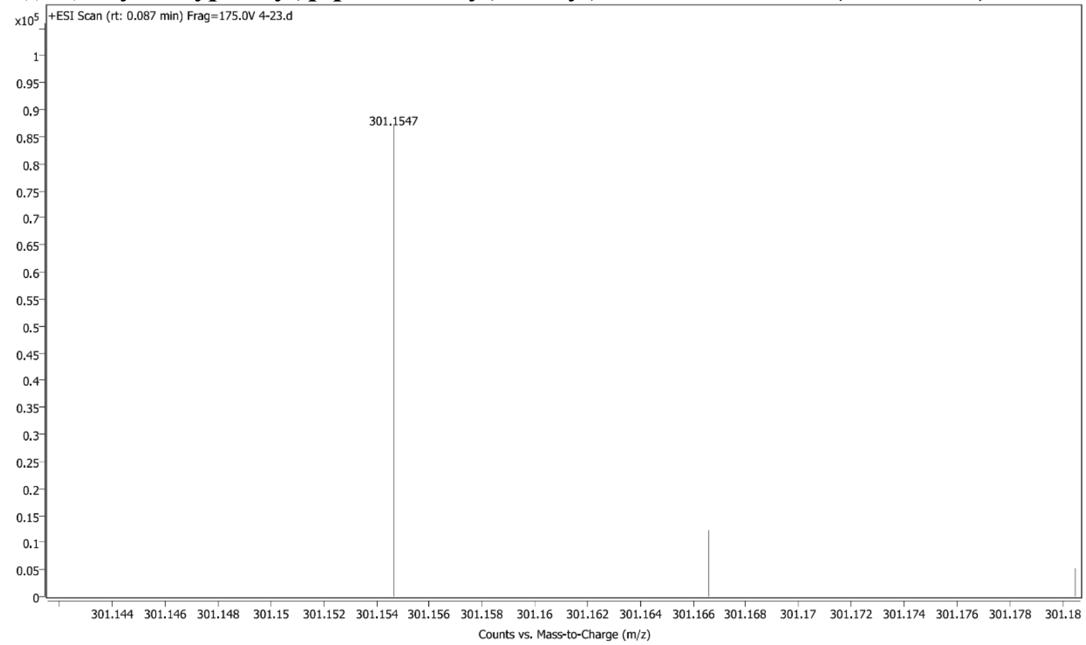

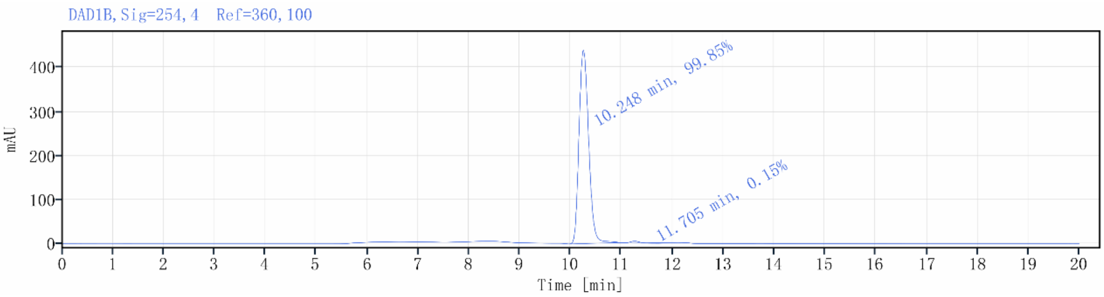

<Peak table>

Detector A Ch1 254nm

Number	Retention time	Peak area	Peak area%	Separation efficiency
1	10.248	5799.4	99.85	--
2	11.705	8.7	0.15	--
Total		5808.1	100.00	

(USP)

4-(4-(2,6-dimethoxybenzyl)piperazin-1-yl)phenol **(AI10-m43)**

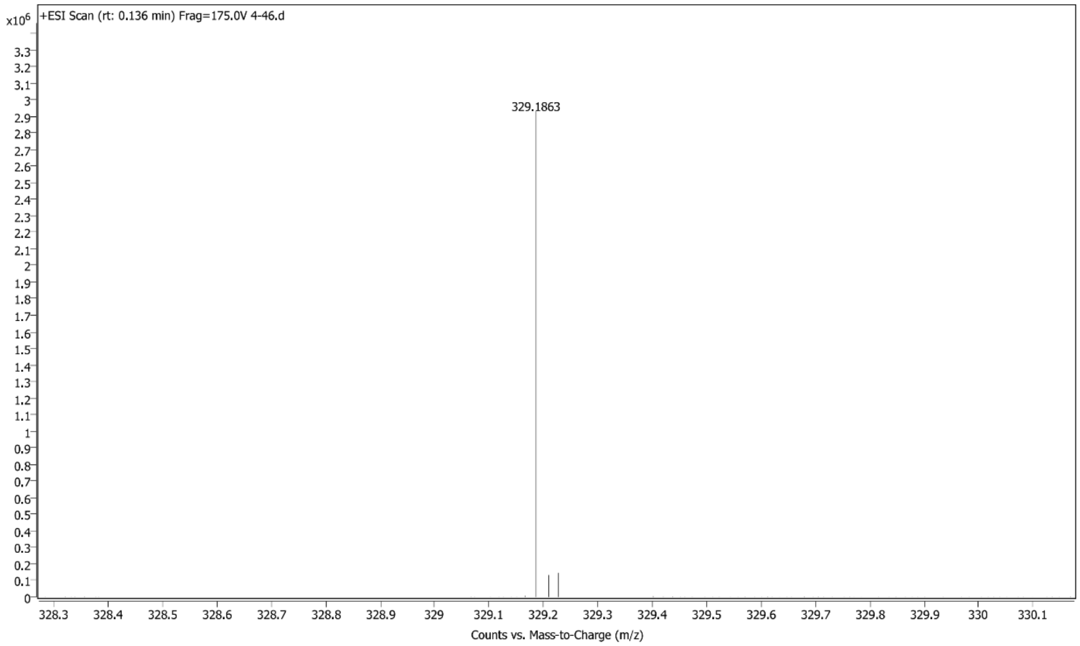

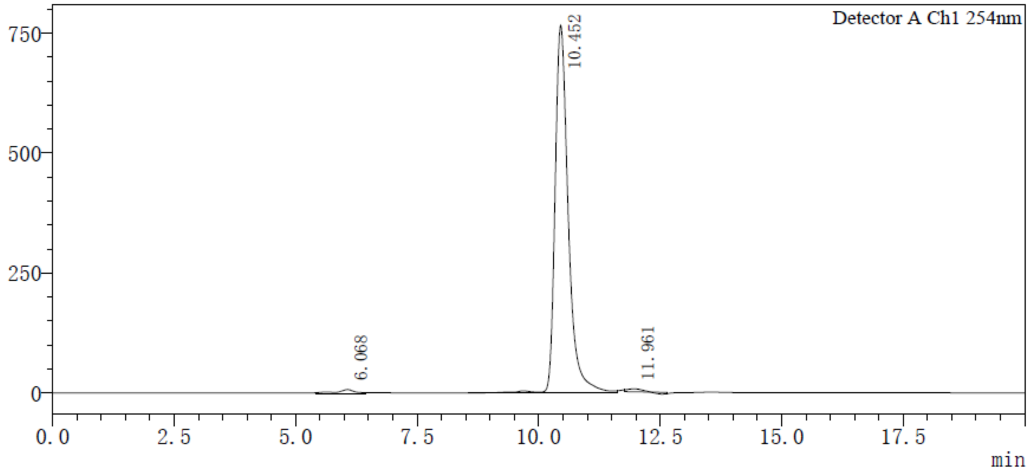

<Peak table>

Detector A Ch1 254nm

Number	Retention time	Peakarea	Peakarea%	Separation efficiency
1	6.068	209400	1.397	--
2	10.452	14689124	98.020	9.118
3	11.961	87296	0.583	2.438
Total		14985820	100.000	

(USP)

4-(4-(pyridin-3-ylmethyl)piperazin-1-yl)phenol **(AI10-m44)**

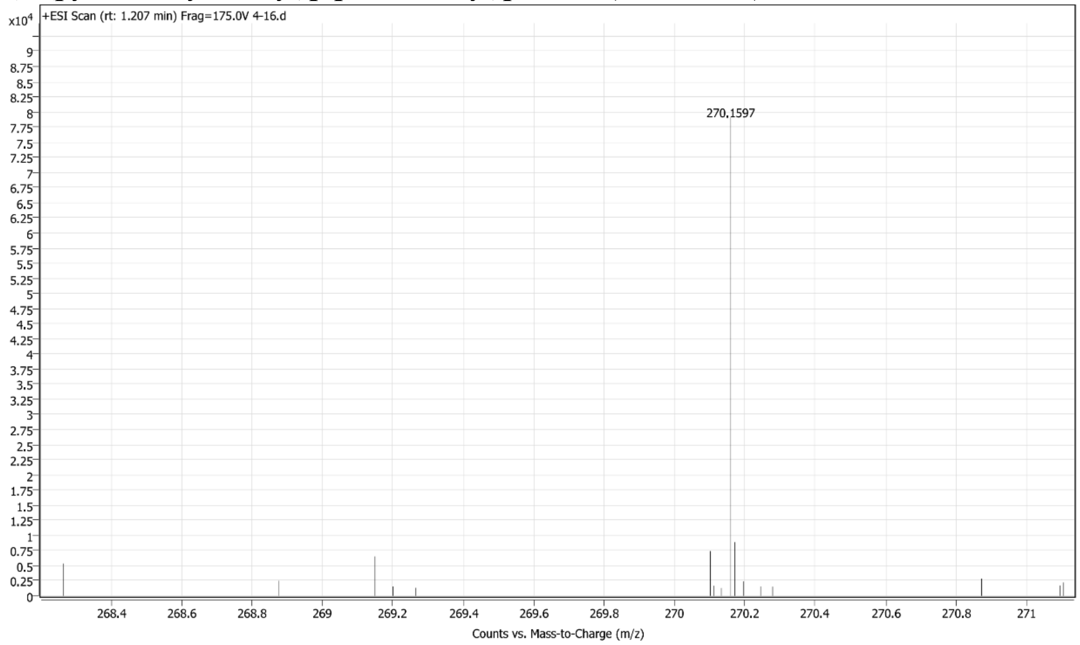

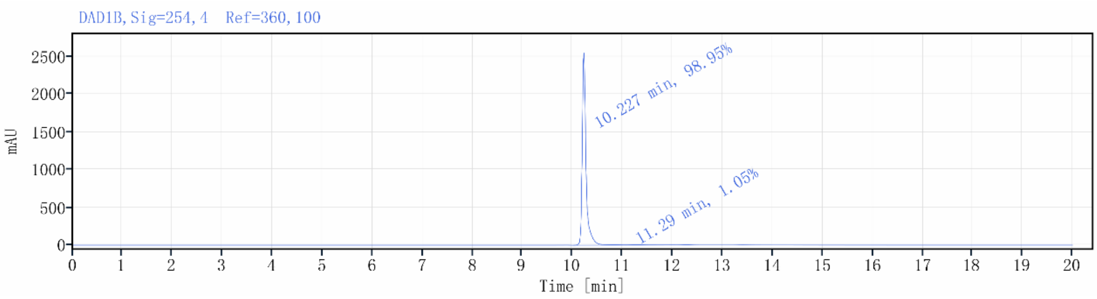

<Peak table>

Detector A Ch1 254nm

Number	Retention time	Peak area	Peak area%	Separation efficiency
1	10. 227	13754. 9	98. 95	--
2	11. 290	145. 5	1. 05	--
Total		13900. 4	100. 000	

(USP)

***4-(4-(furan-2-ylmethyl)piperazin-1-yl)phenol* (AI10-m45)**

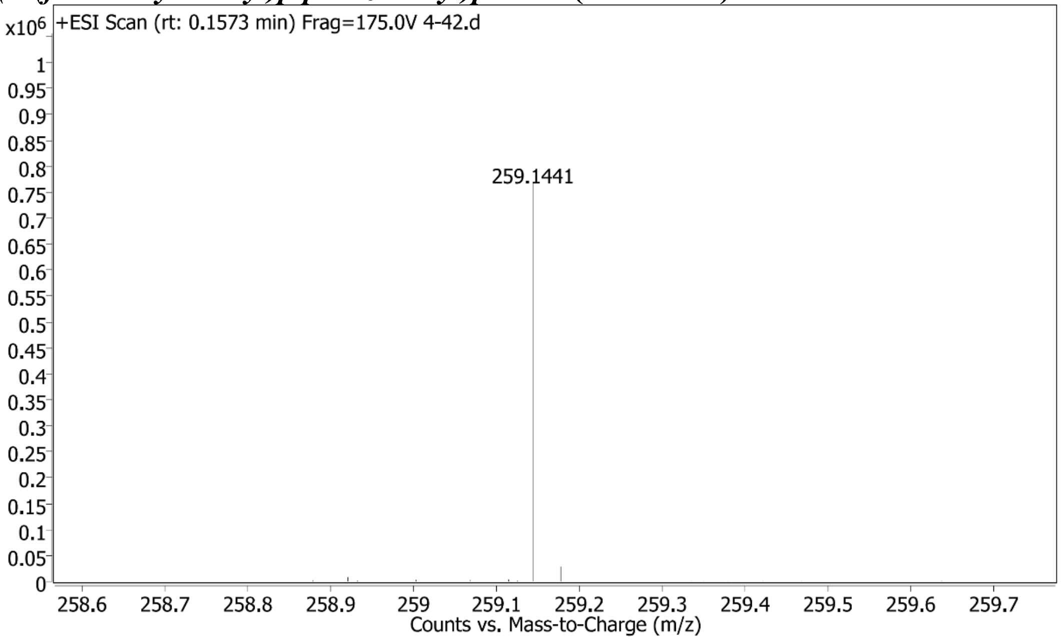

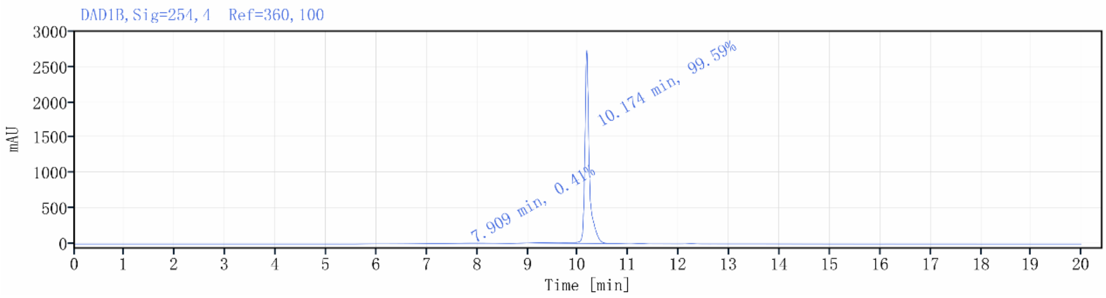

<Peak table>

Detector A Ch1 254nm

Number	Retention time	Peak area	Peak area%	Separation efficiency
1	7. 909	72. 2	0. 41	--
2	10. 174	17492. 0	99. 59	--
Total		17564. 2	100. 00	

(USP)

4-(4-(thiophen-2-ylmethyl)piperazin-1-yl)phenol **(AI10-m46)**

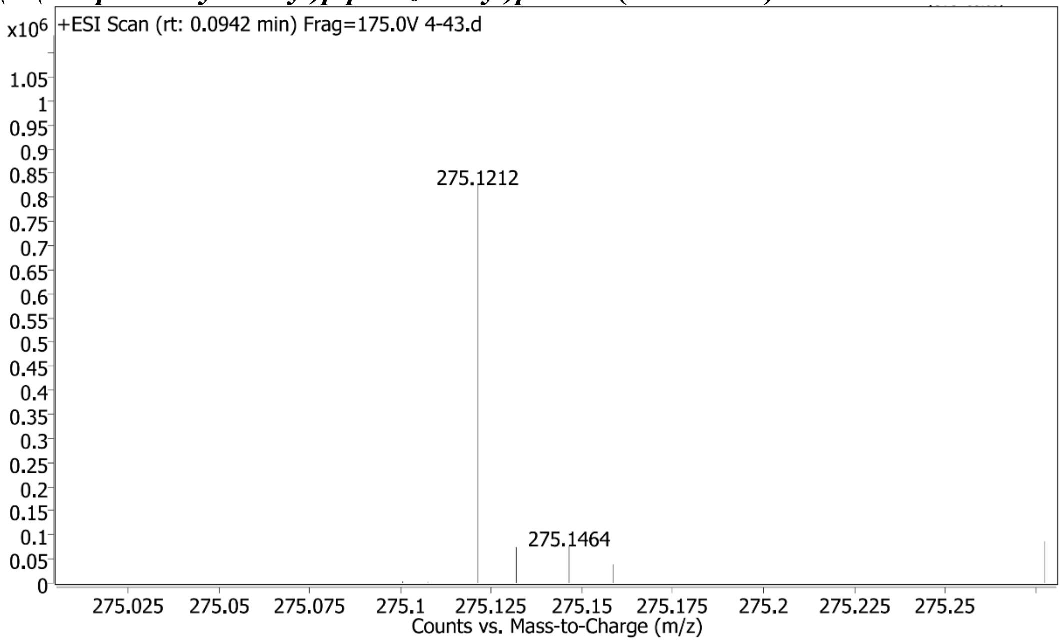

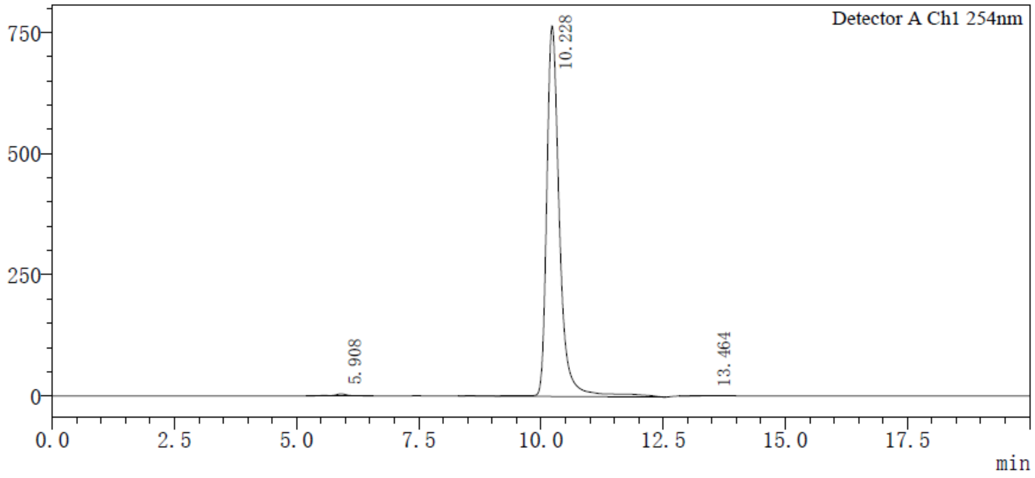

<Peak table>

Detector A Ch1 254nm

Number	Retention time	Peakarea	Peakarea%	Separation efficiency
1	5. 908	75192	0. 535	--
2	10. 228	13957202	99. 320	10. 397
3	13. 464	20298	0. 144	3. 486
Total		14052692	100. 000	

(USP)

4-(4-(3-methylbenzyl)piperazin-1-yl)benzene-1,3-diol **(AI10-m47)**

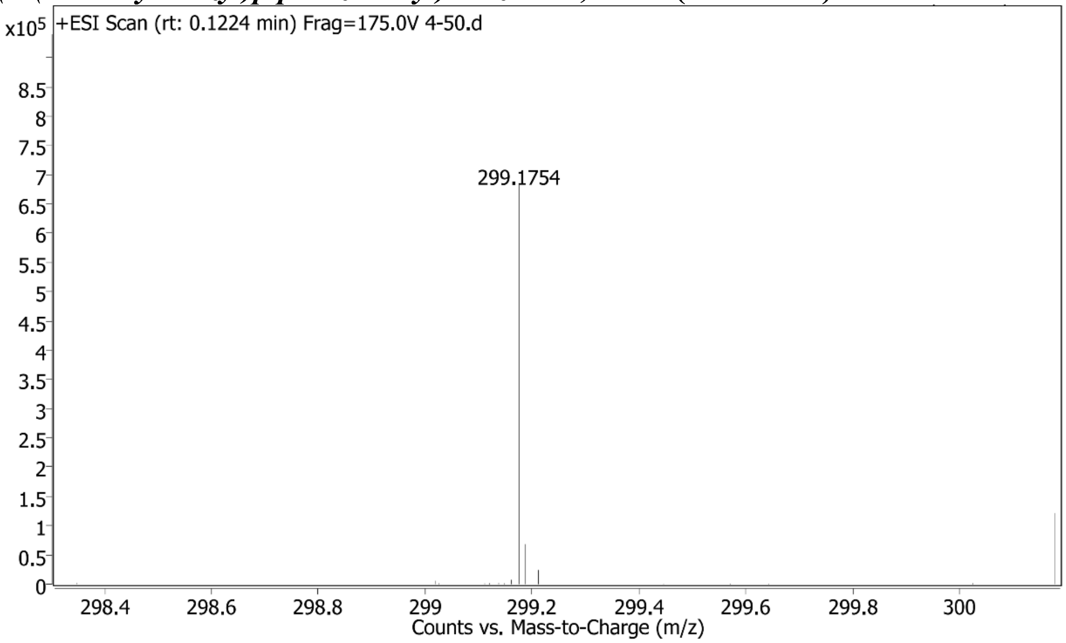

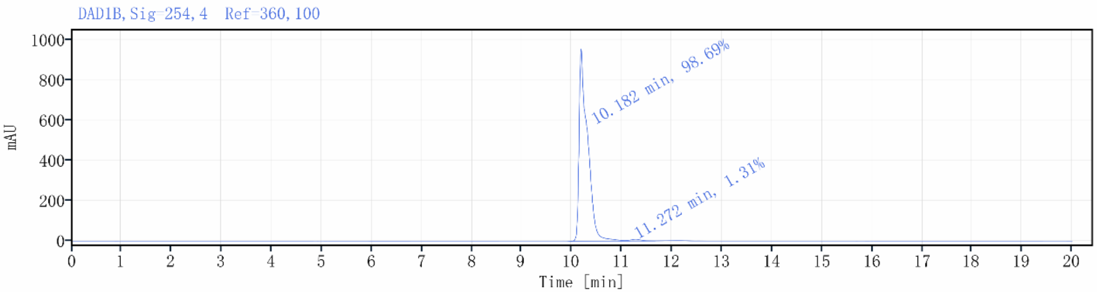

<Peak table>

Detector A Ch1 254nm

Number	Retention time	Peak area	Peak area%	Separation efficiency
1	10. 182	11270. 2	98. 69	--
2	11. 272	150. 0	1. 31	--
Total		11420. 2	100. 00	

(USP)

(E)-4-(4-(3-(m-tolyl)allyl)piperazin-1-yl)phenol **(AI10-m48)**

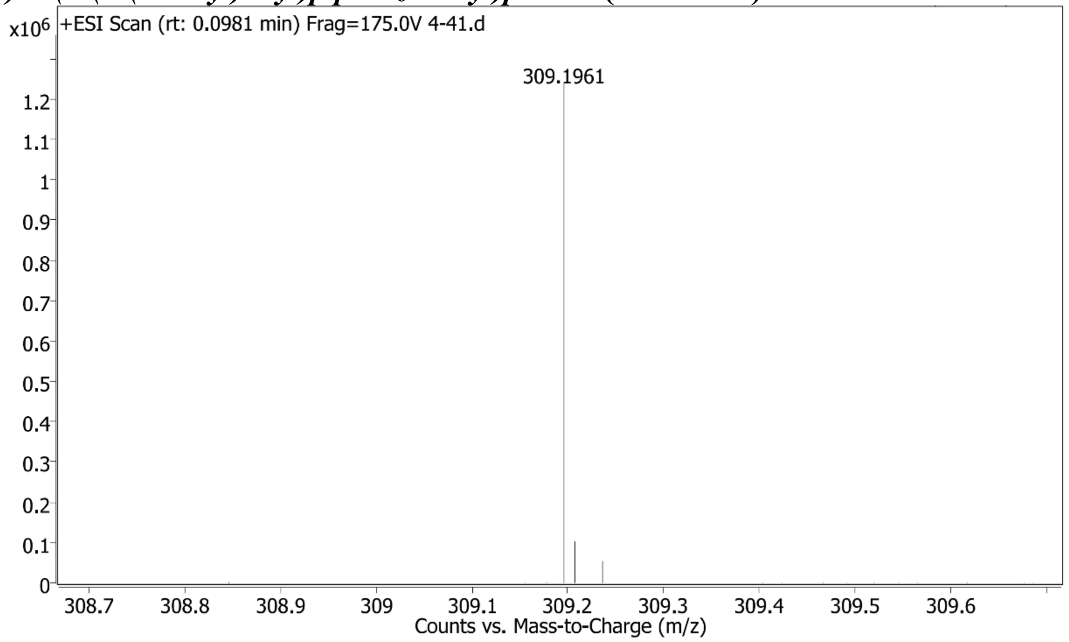

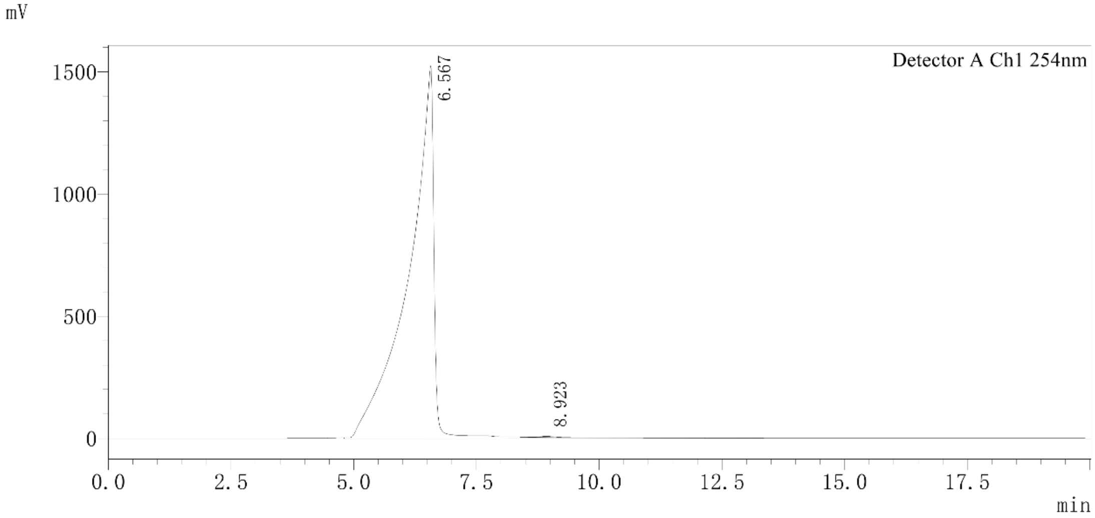

<Peak table>

Detector A Ch1 254nm

Number	Retention time	Peak area	Peak area%	Separation efficiency
1	6.567	54870570	99.828	--
2	8.923	94771	0.172	3.795
Total		54965341	100.000	

(USP)

(E)-4-(4-(3-(4-chlorophenyl)allyl)piperazin-1-yl)phenol **(AI10-m49)**

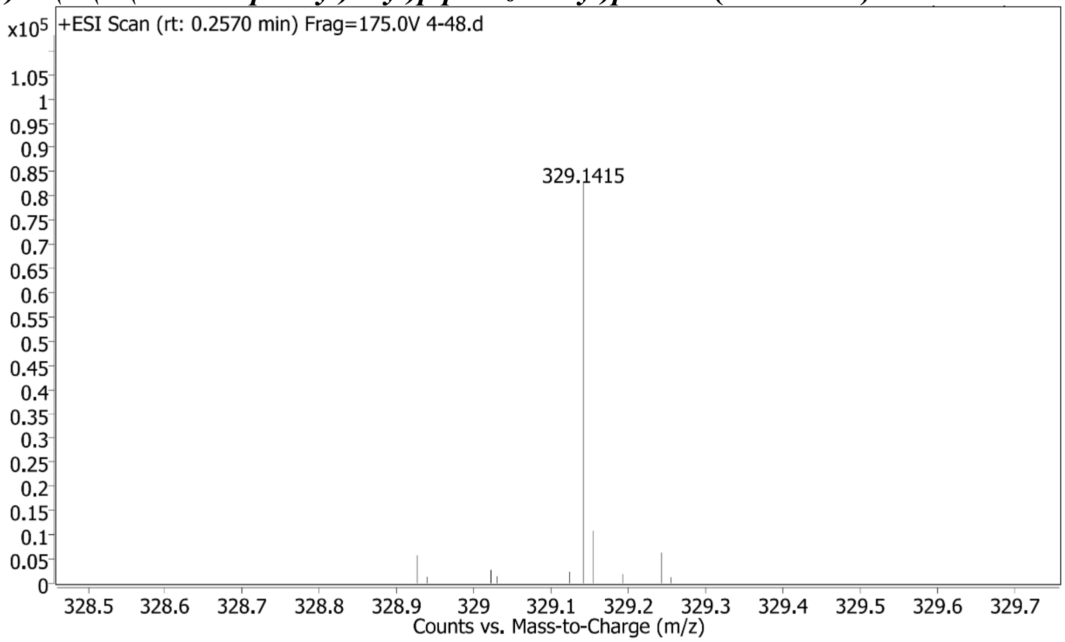

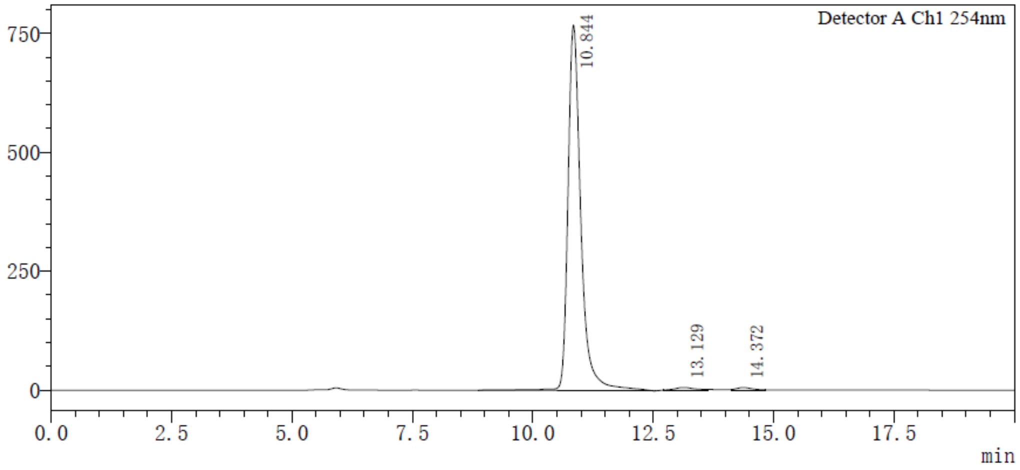

<Peak table>

Detector A Ch1 254nm

Number	Retention time	Peakarea	Peakarea%	Separation efficiency
1	10.844	14314136	98.070	--
2	13.129	150023	1.028	3.994
3	14.372	131734	0.903	1.858
Total		14595893	100.000	

(USP)

4-(4-(4-fluorophenethyl)piperazin-1-yl)phenol **(AI10-m50)**

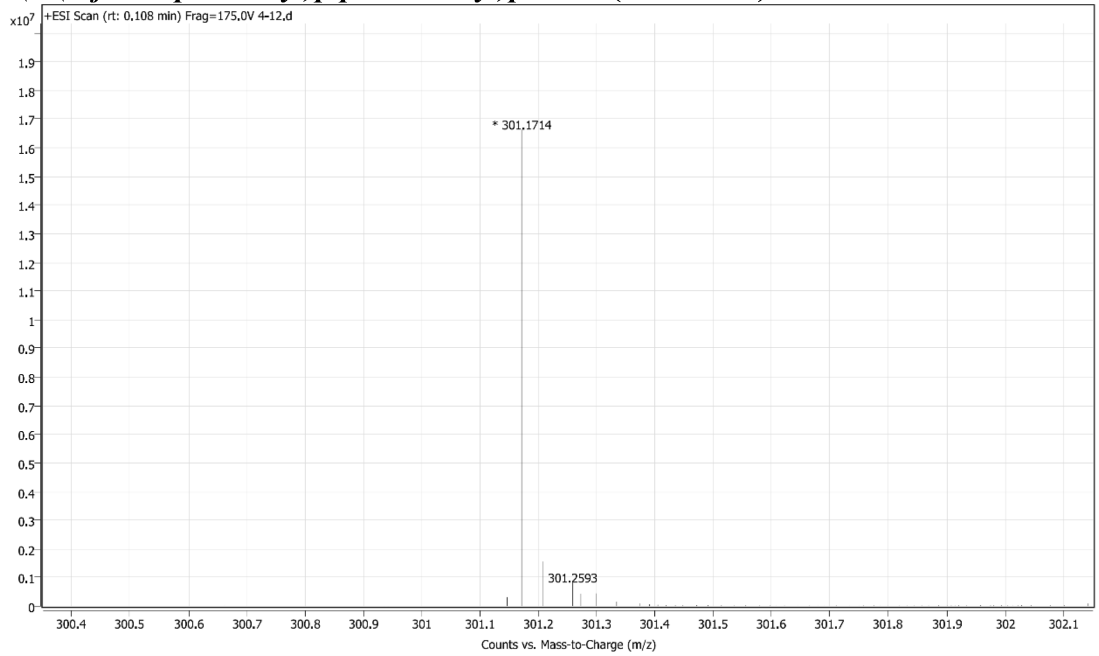

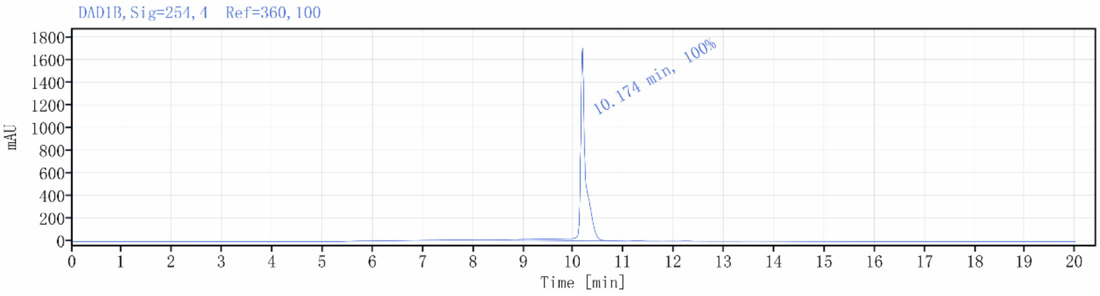

<Peak table>

Detector A Ch1 254nm

Number	Retention time	Peak area	Peak area%	Separation efficiency
1	10.074	12765.5	100.00	--
Total		12765.5	100.00	

(USP)

(4-(4-hydroxyphenyl)piperidin-1-yl)(pyridin-4-yl)methanone **(AI10-m51)**

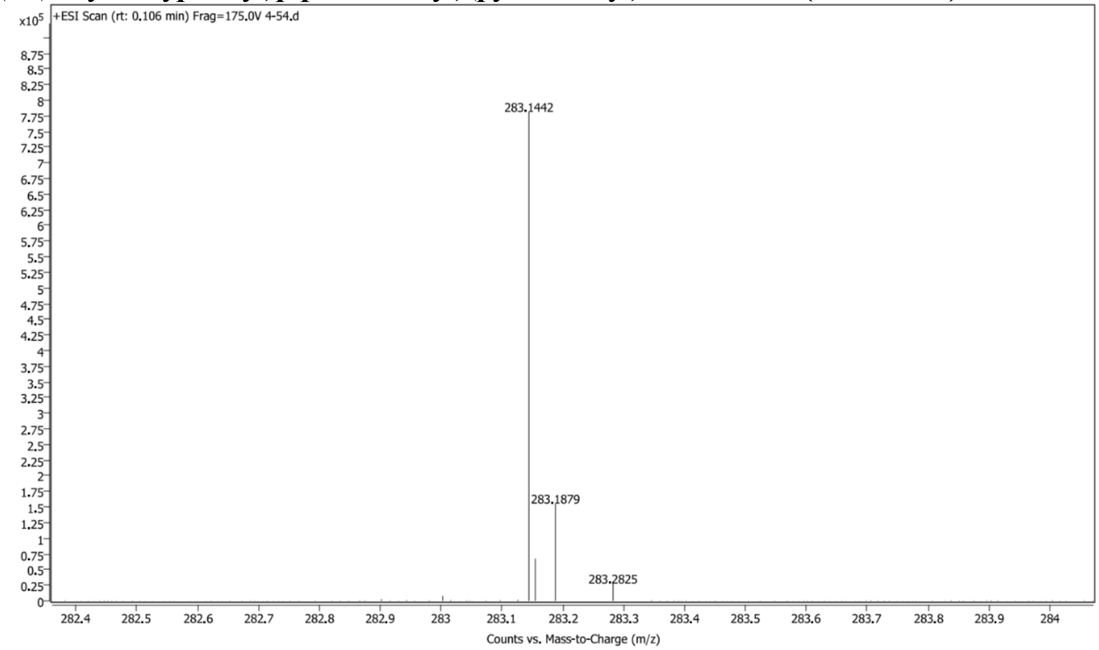

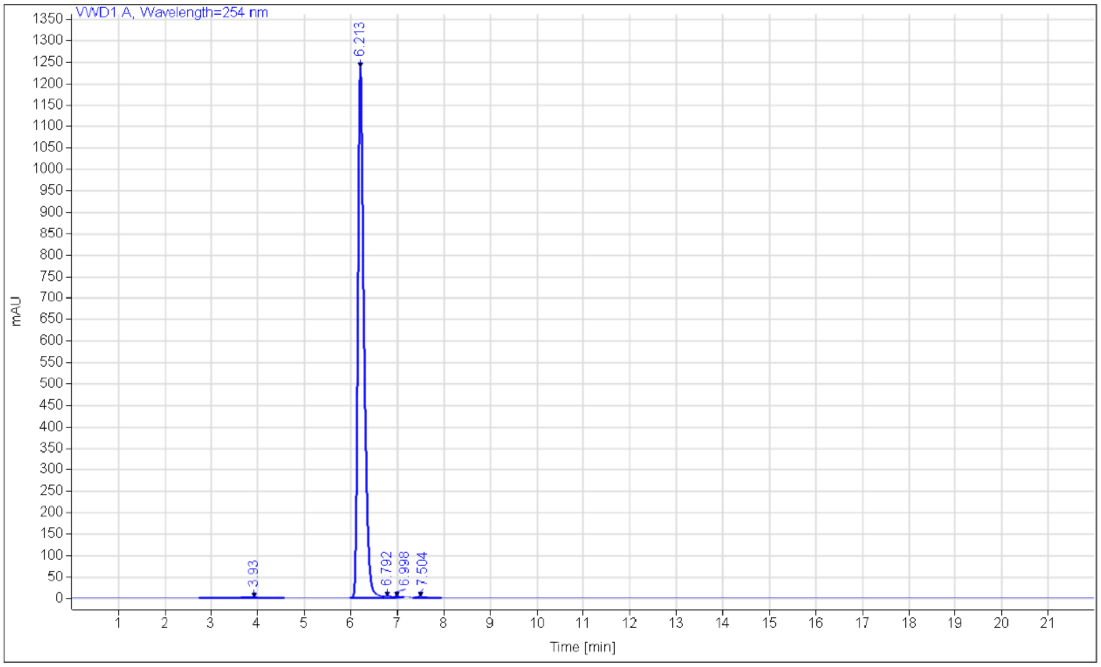

<Peak table>

Detector A Ch1 254nm

Number	Retention time	Peak area	Peak area%	Separation efficiency
1	3. 930	66. 79581	0. 59	--
2	6. 213	11169. 05664	98. 66	5. 93999
3	6. 792	36. 06821	0. 32	2. 12395
4	6. 998	20. 60956	0. 18	0. 72106
5	7. 504	28. 46054	0. 25	2. 03623
Total		11320. 99072	100. 00	

(USP)

(E)-3-(2,4-dihydroxyphenyl)-1-(4-(4-hydroxyphenyl)piperidin-1-yl)prop-2-en-1-one **(AI10-m52)**

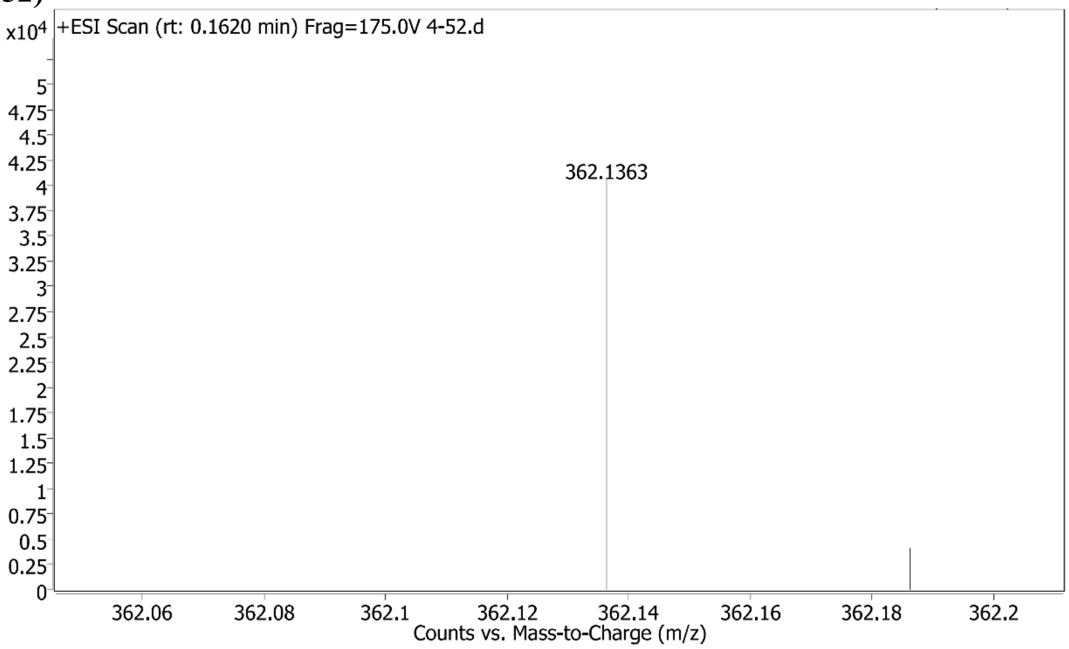

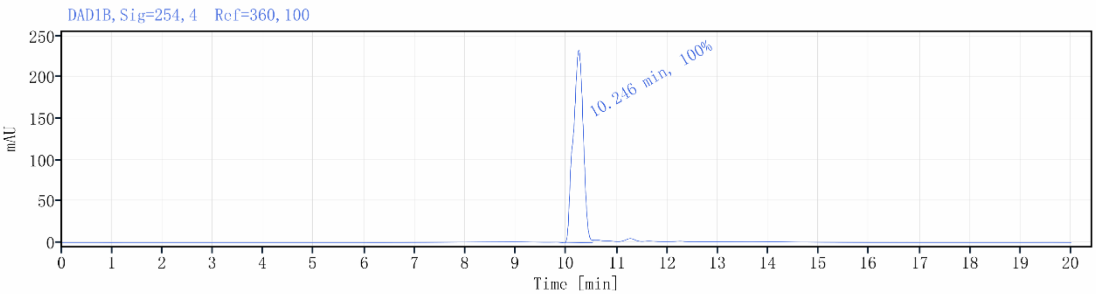

<Peak table>

Detector A Ch1 254nm

Number	Retention time	Peak area	Peak area%	Separation efficiency
1	10. 246	3240. 0	100. 00	--
Total		3240. 0	100. 00	

(USP)

4-(1-(3-methylbenzyl)piperidin-4-yl)phenol **(AI10-m53)**

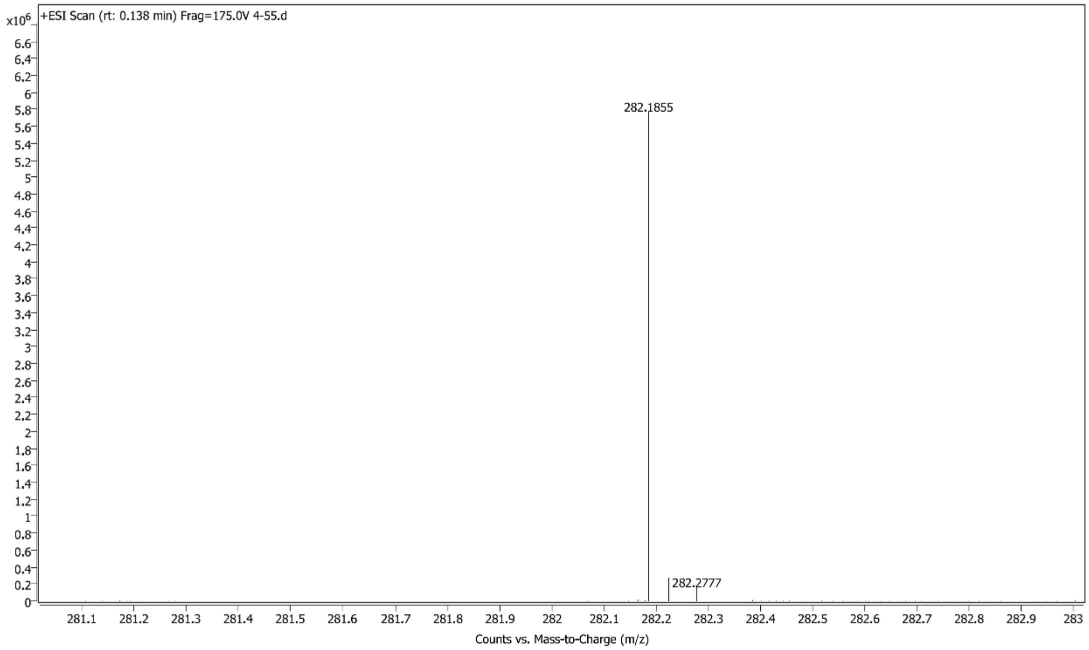

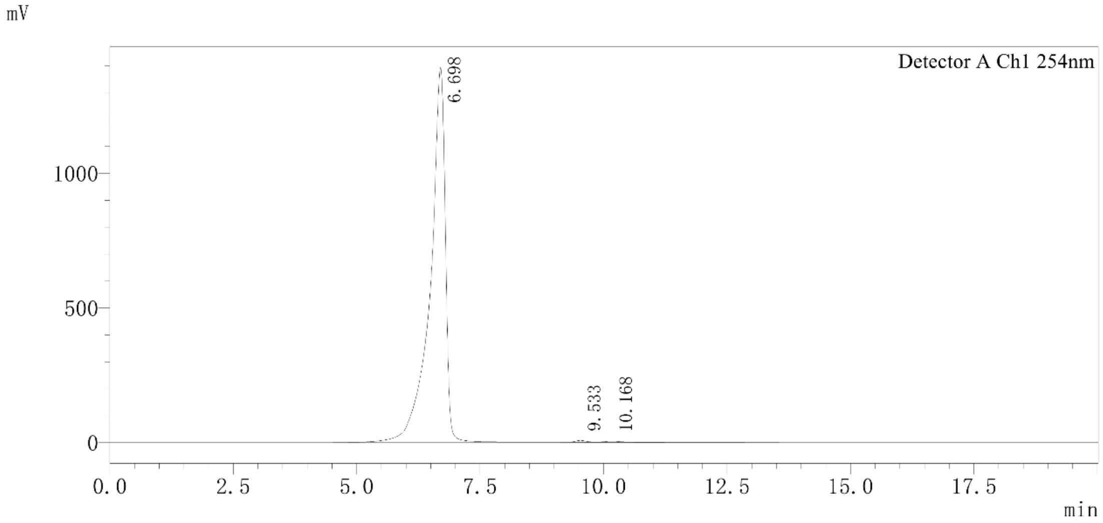

<Peak table>

Detector A Ch1 254nm

Number	Retention time	Peak area	Peak area%	Separation efficiency
1	6.698	30140285	99.334	--
2	9.533	100300	0.331	6.522
3	10.168	101845	0.336	1.278
Total		30342429	100.000	

(USP)

4-(1-(4-methylbenzyl)piperidin-4-yl)phenol (AI10-m54)

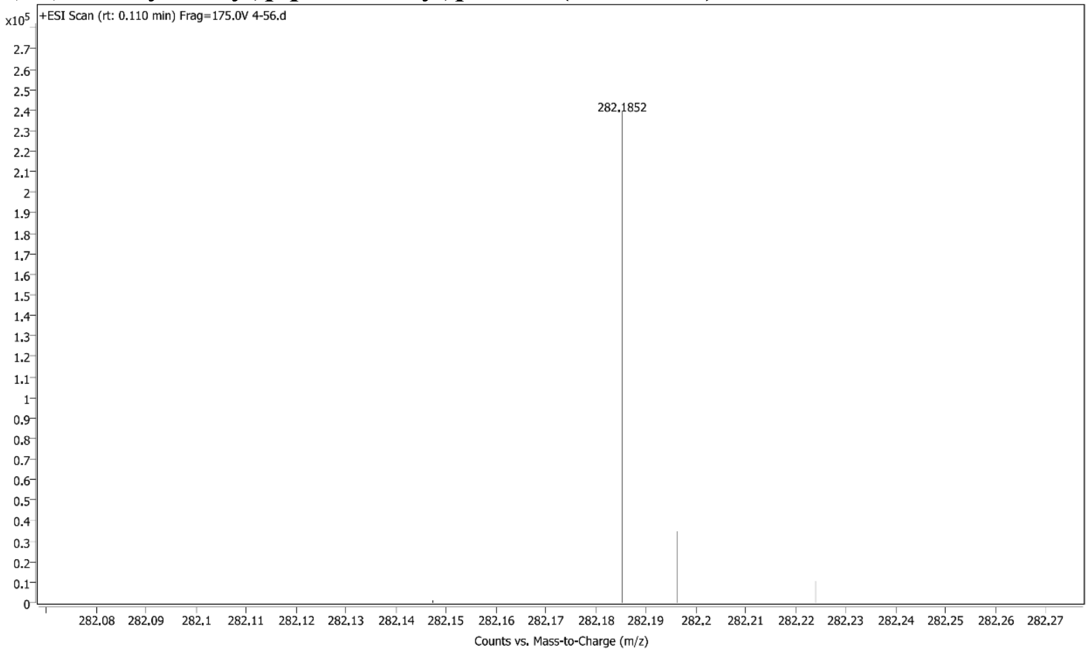

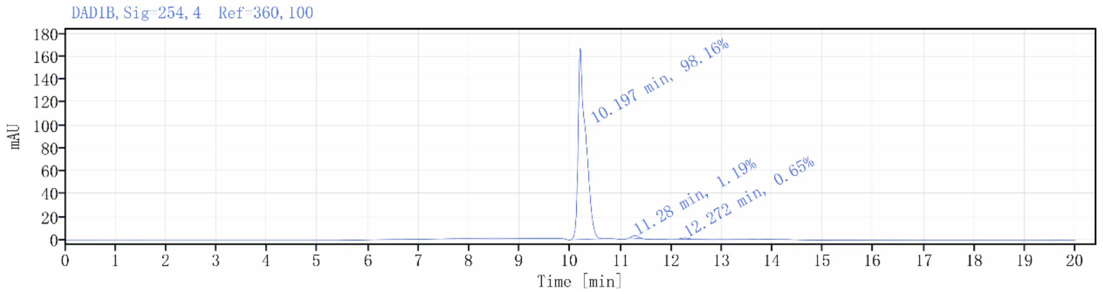

<Peak table>

Detector A Ch1 254nm

Number	Retention time	Peak area	Peak area%	Separation efficiency
1	10.197	1730.6	98.16	--
2	11.280	20.9	1.19	--
3	12.272	11.5	0.65	--
Total		1763.0	100.00	

(USP)

4-(1-(3,4-dichlorobenzyl)piperidin-4-yl)phenol **(AI10-m55)**

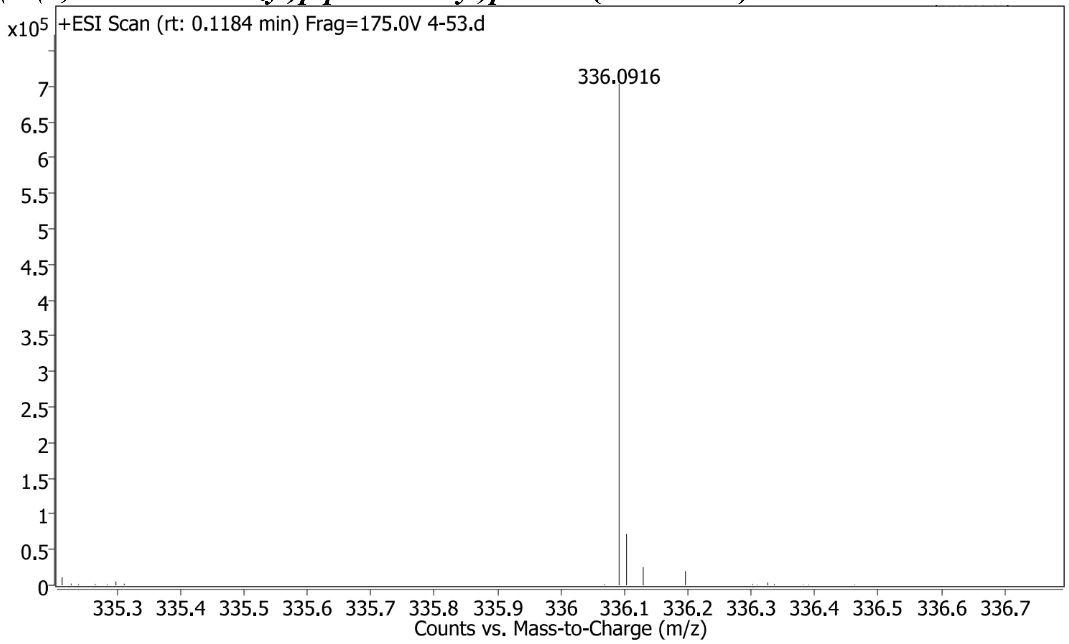

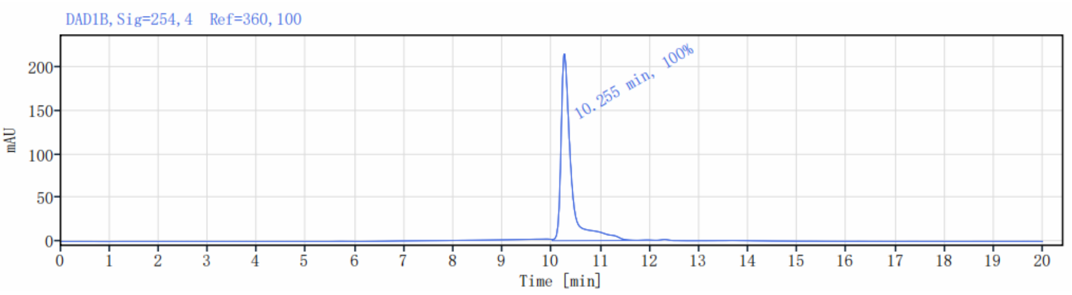

<Peak table>

Detector A Ch1 254nm

Number	Retention time	Peak area	Peak area%	Separation efficiency
1	10. 255	3076. 3	100. 00	--
Total		3076. 3	100. 00	

(USP)

4-(1-(thiophen-2-ylmethyl)piperidin-4-yl)phenol **(AI10-m56)**

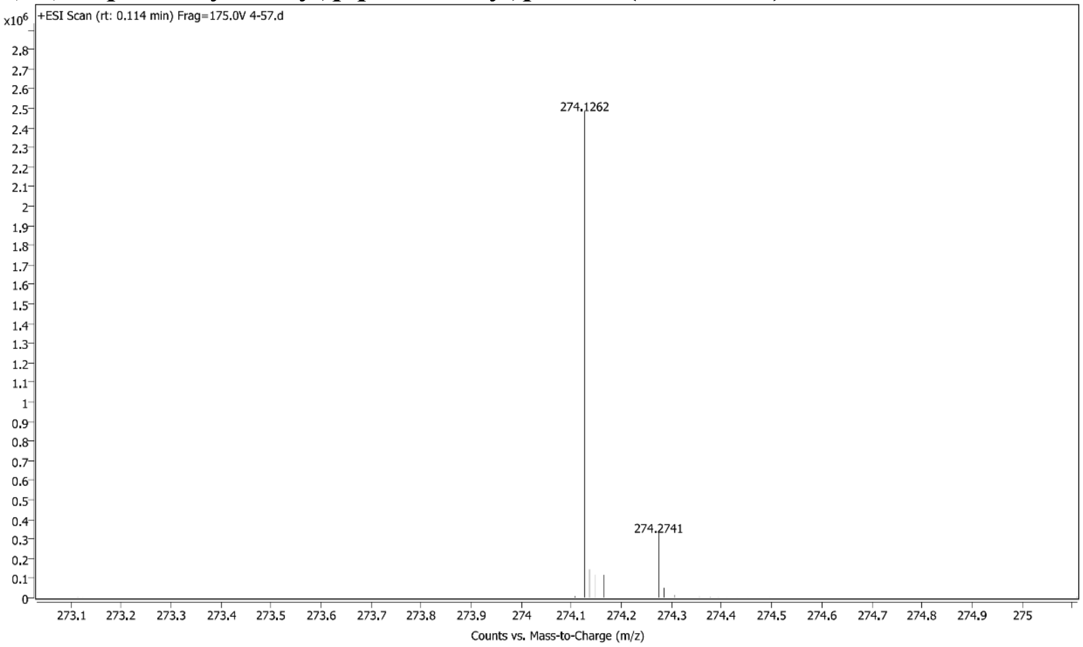

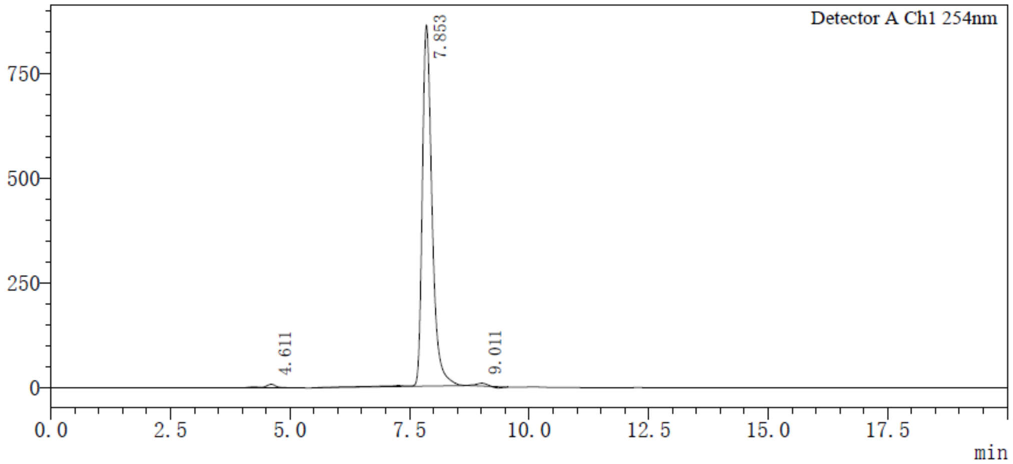

<Peak table>

Detector A Ch1 254nm

Number	Retention time	Peakarea	Peakarea%	Separation efficiency
1	4.611	130088	1.041	--
2	7.853	12297330	98.376	9.409
3	9.011	72880	0.583	2.931
Total		12500298	100.000	

(USP)

4-((4-(4-hydroxyphenyl)piperidin-1-yl)methyl)benzene-1,3-diol **(AI10-m57)**

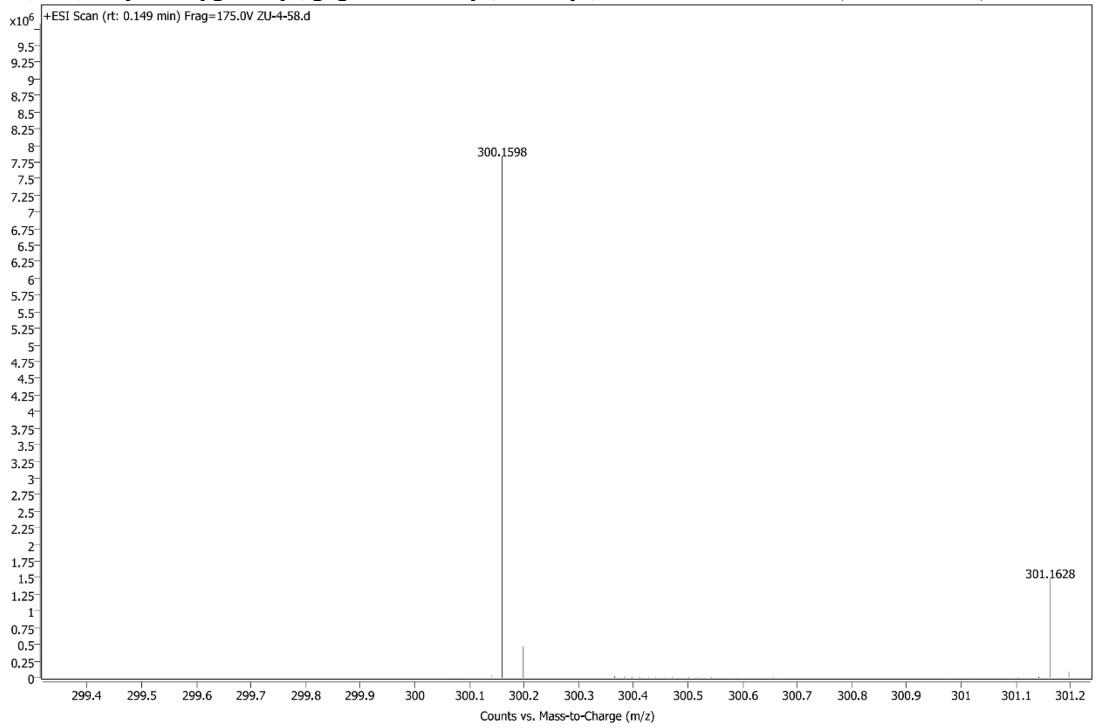

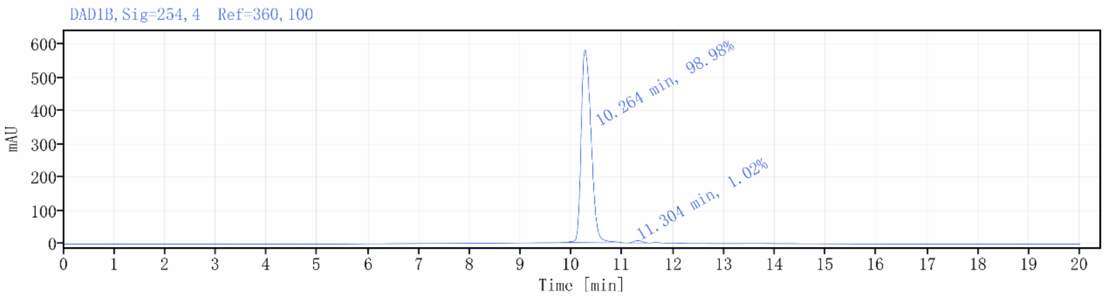

<Peak table>

Detector A Ch1 254nm

Number	Retention time	Peak area	Peak area%	Separation efficiency
1	10. 264	7792. 9	98. 98	--
2	11. 304	80. 6	1. 02	--
Total		7873. 5	100. 00	

(USP)

(2,6-dimethylpyridin-4-yl)(4-(4-hydroxybenzyl)piperazin-1-yl)methanone (**AI10-a1**)

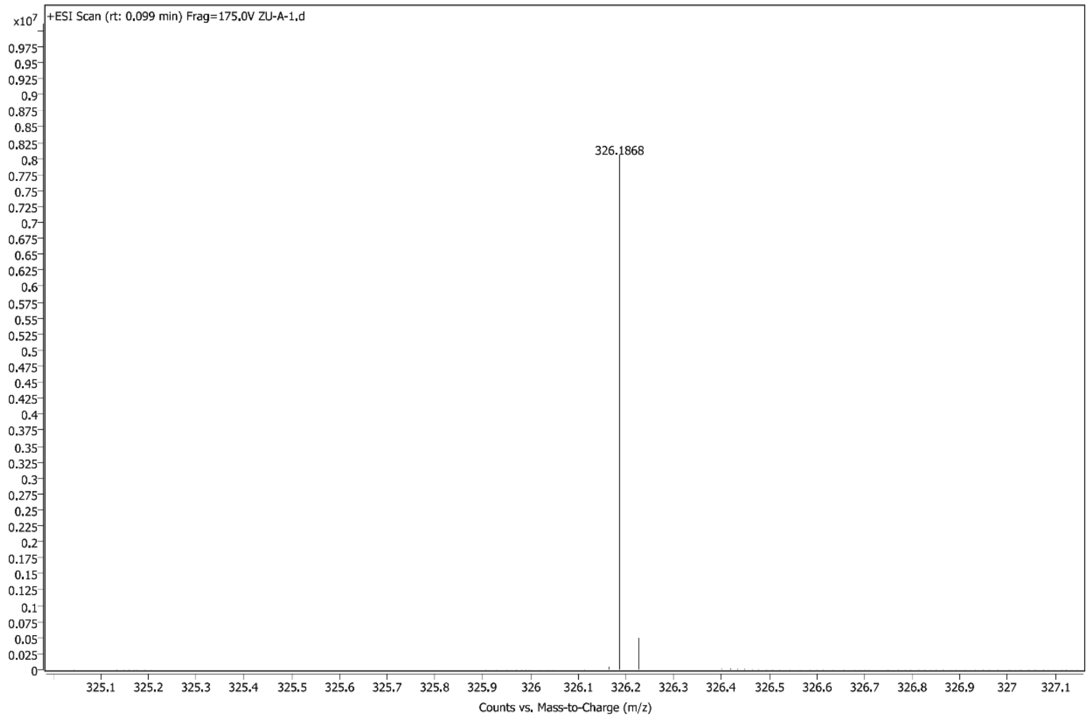

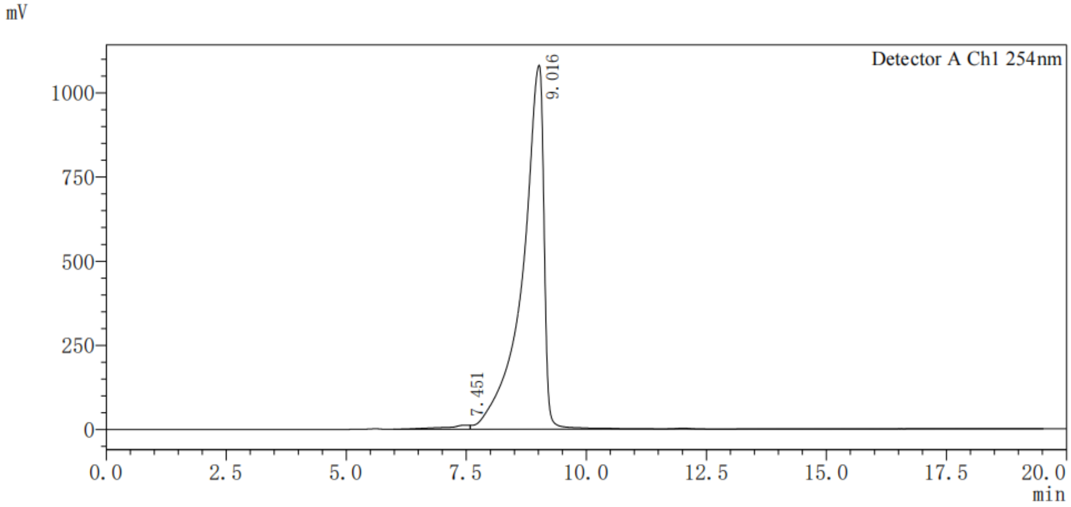

<Peak table>

Detector A Ch1 254nm

Number	Retention time	Peak area	Peak area%	Separation efficiency
1	7.451	475836	1.381	--
2	9.016	33975594	98.619	0.847
Total		34451430	100.000	

(USP)

(4-(2,4-dihydroxybenzyl)piperazin-1-yl)(2,6-dimethylpyridin-4-yl)methanone **(AI10-a2)**

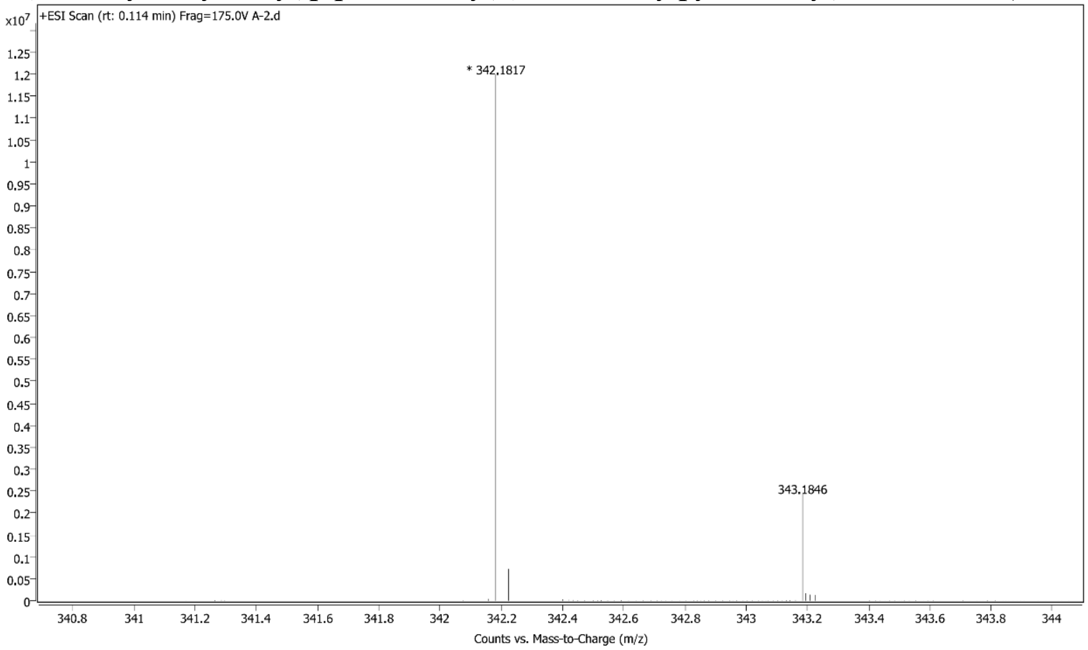

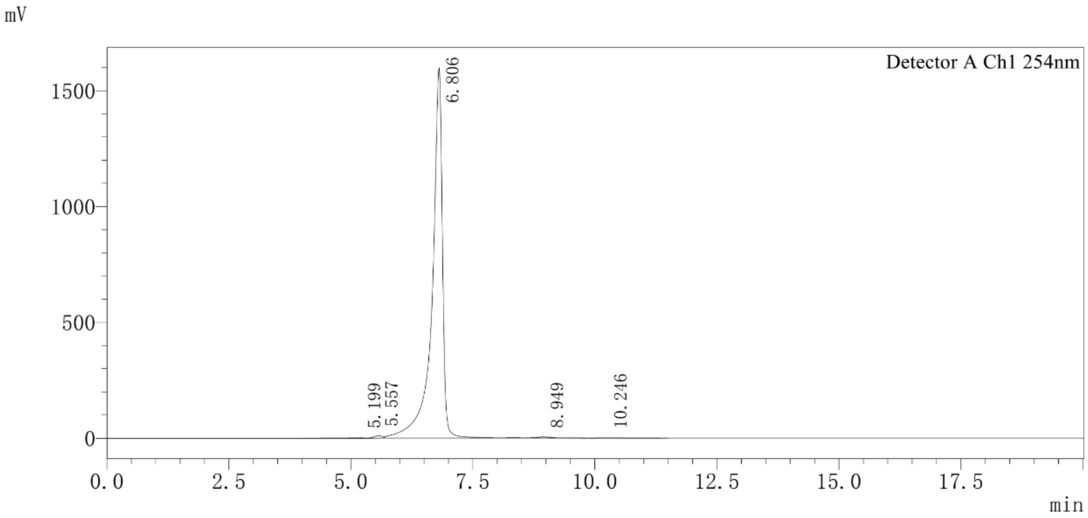

<Peak table>

Detector A Ch1 254nm

Number	Retention time	Peak area	Peak area%	Separation efficiency
1	5.199	58778	0.245	--
2	5.557	132982	0.553	0.724
3	6.806	23743696	98.768	2.952
4	8.949	78658	0.327	4.925
5	10.246	25818	0.107	2.201
Total		24039932	100.000	

(USP)

(4-(4-hydroxyphenyl)piperazin-1-yl)(2-methylpyridin-4-yl)methanone **(AI10-a3)**

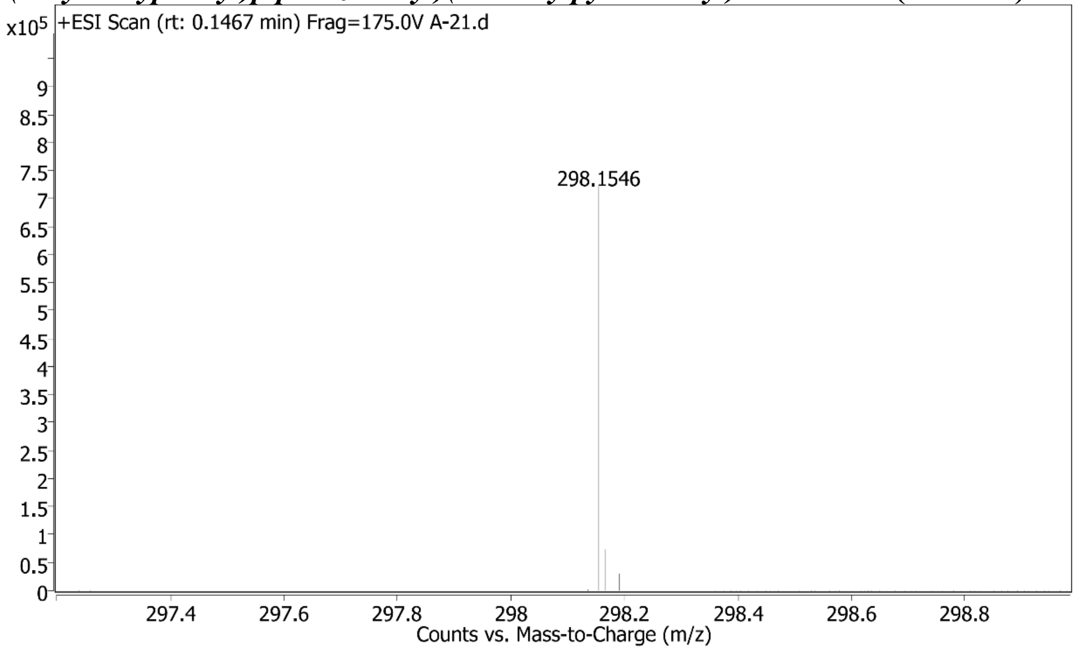

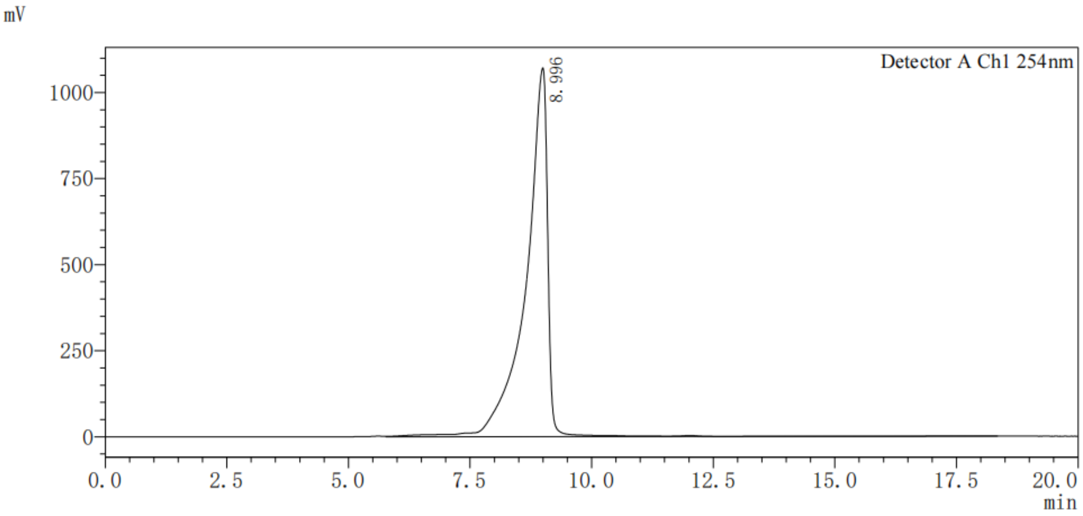

<Peak table>

Detector A Ch1 254nm

Number	Retention time	Peak area	Peak area%	Separation efficiency
1	8.996	33315959	100.000	--
Total		33315959	100.000	

(USP)

(4-(4-hydroxyphenyl)piperazin-1-yl)(5-methylpyridin-3-yl)methanone **(AI10-a4)**

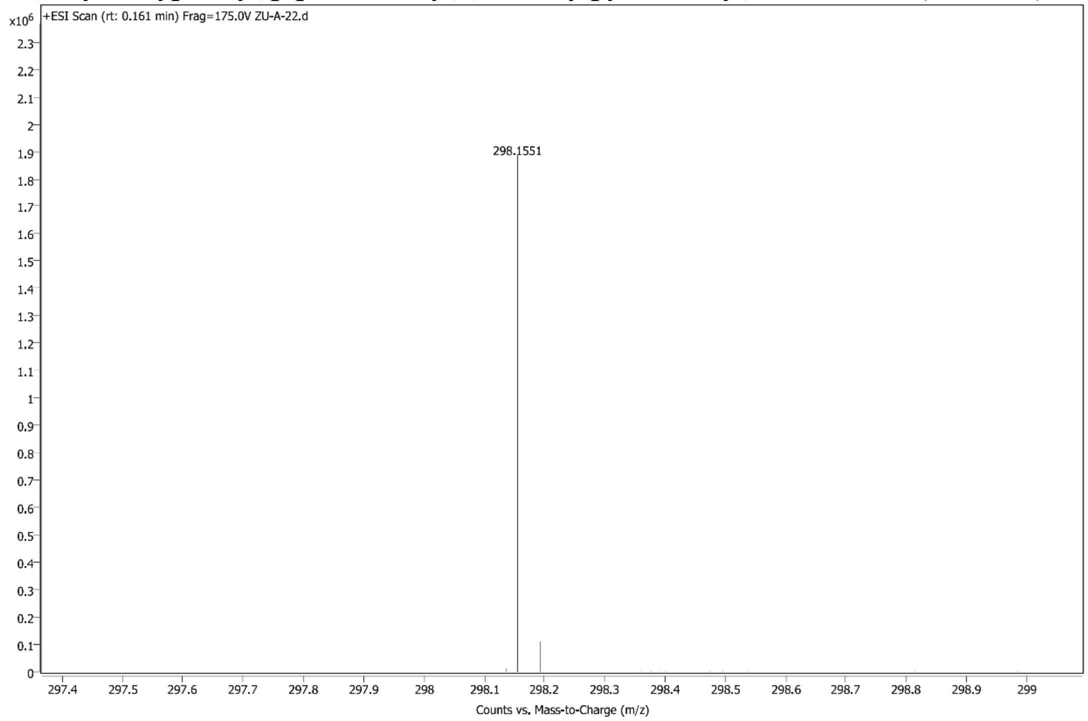

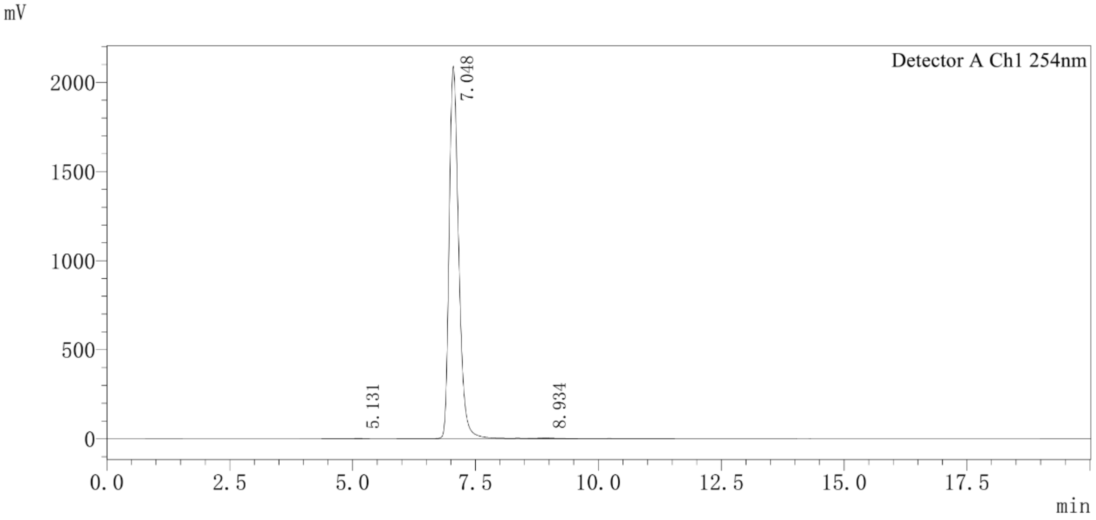

<Peak table>

Detector A Ch1 254nm

Number	Retention time	Peak area	Peak area%	Separation efficiency
1	5.131	54811	0.190	--
2	7.048	28671704	99.565	5.841
3	8.934	70334	0.244	4.238
Total		28796849	100.000	

(USP)

(4-(4-hydroxyphenyl)piperazin-1-yl)(6-methylpyridin-2-yl)methanone **(AI10-a5)**

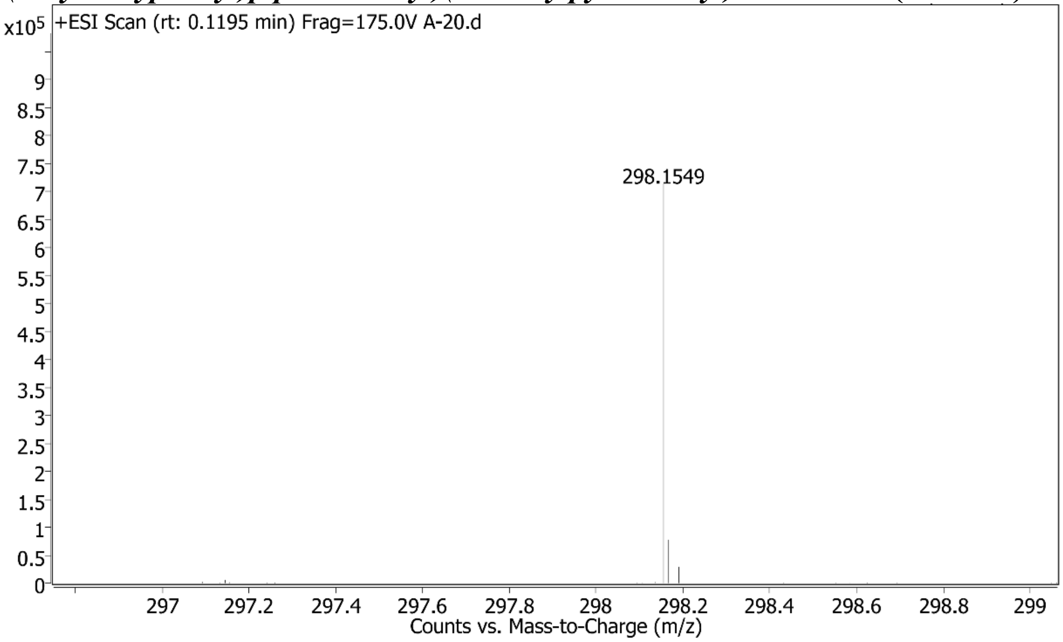

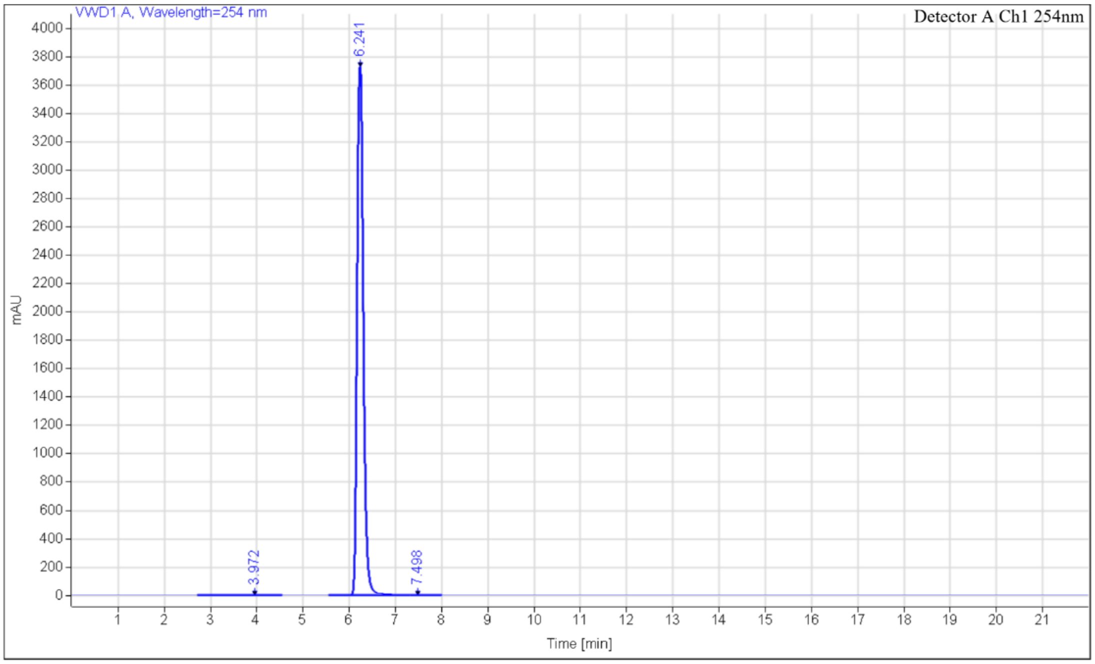

〈Peak table〉

Detector A Ch1 254nm

Number	Retention time	Peak area	Peak area%	Separation efficiency
1	3. 972	76. 83105	0. 22	--
2	6. 241	35180. 66797	99. 66	6. 01157
3	7. 498	41. 60472	0. 12	4. 73842
Total		35299. 10374	100. 00	

(USP)

(4-(4-hydroxyphenyl)piperazin-1-yl)(2-methylpyridin-3-yl)methanone (AI10-a6)

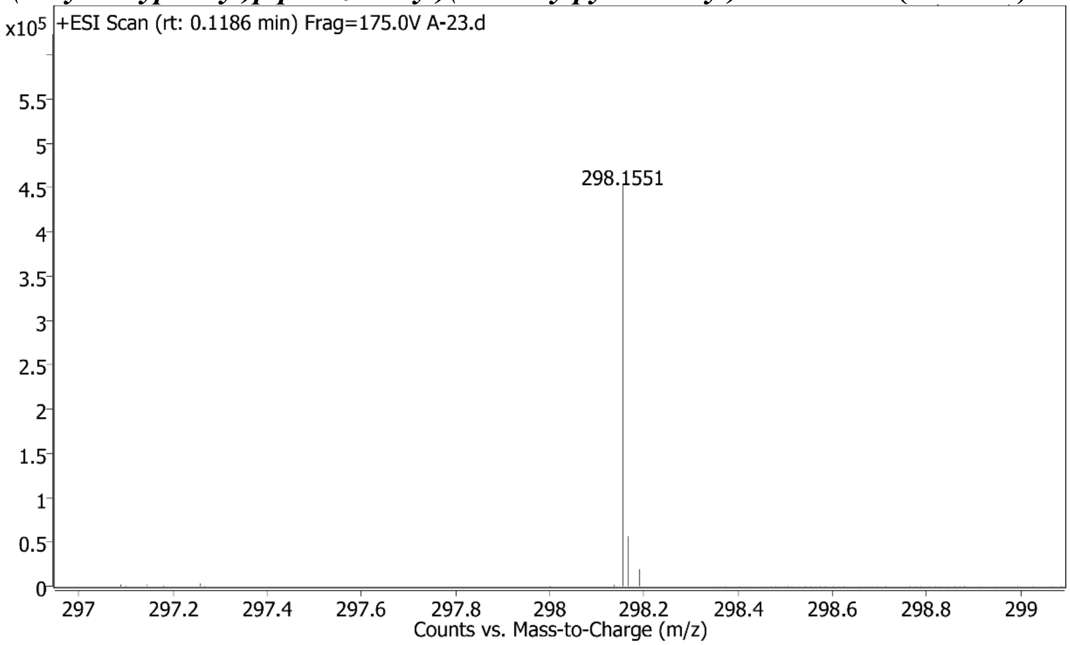

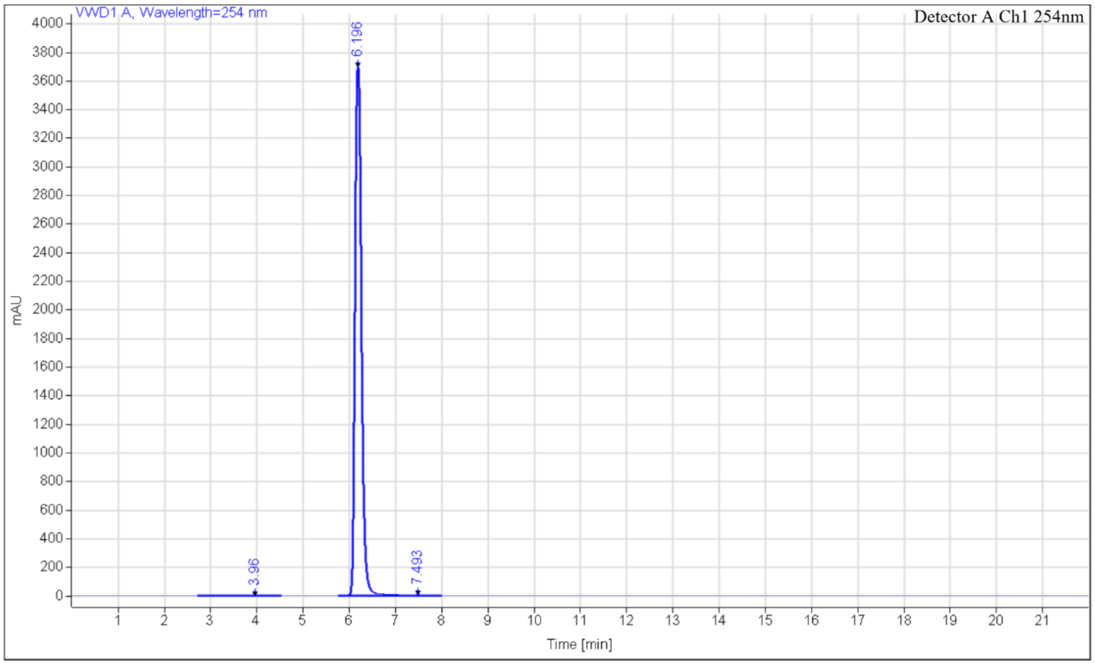

〈Peak table〉

Detector A Ch1 254nm

Number	Retention time	Peak area	Peak area%	Separation efficiency
1	3. 960	70. 54427	0. 19	--
2	6. 196	36083. 89453	99. 68	5. 70764
3	7. 493	46. 83747	0. 13	4. 57047
Total		36201. 27627	100. 000	

(USP)

***(4-hydroxy-2-methylphenyl)(4-(4-hydroxyphenyl)piperazin-1-yl)methanone* (AI10-a7)**

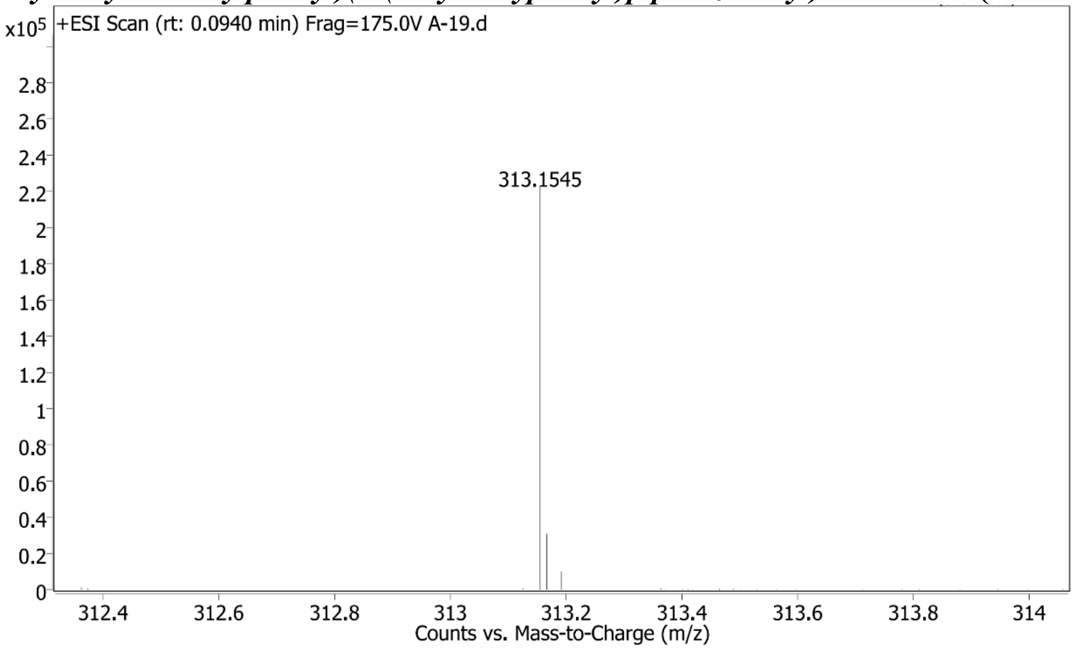

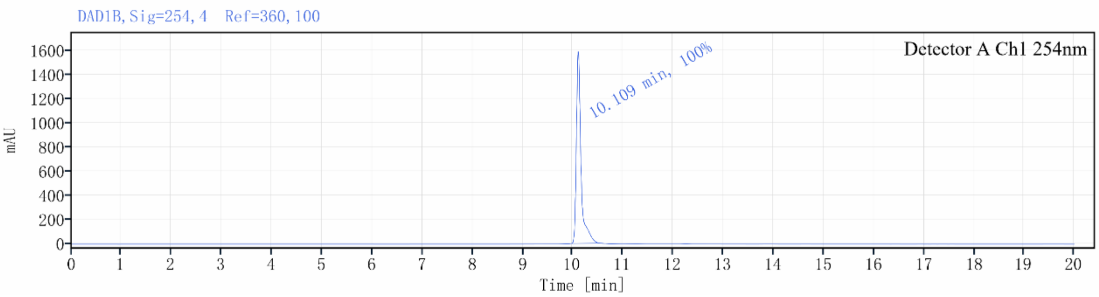

〈Peak table〉

Detector A Ch1 254nm

Number	Retention time	Peak area	Peak area%	Separation efficiency
1	10. 109	10284. 4	100. 00	--
Total		10284. 4	100. 00	

(USP)

(2-hydroxy-3-methylphenyl)(4-(4-hydroxyphenyl)piperazin-1-yl)methanone **(AI10-a8)**

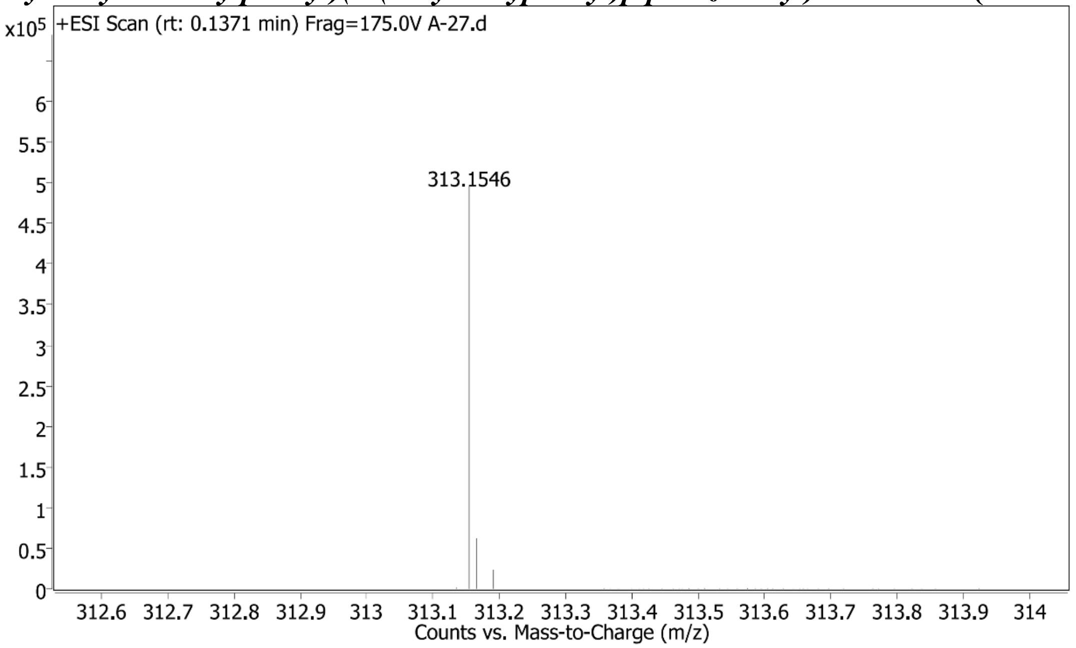

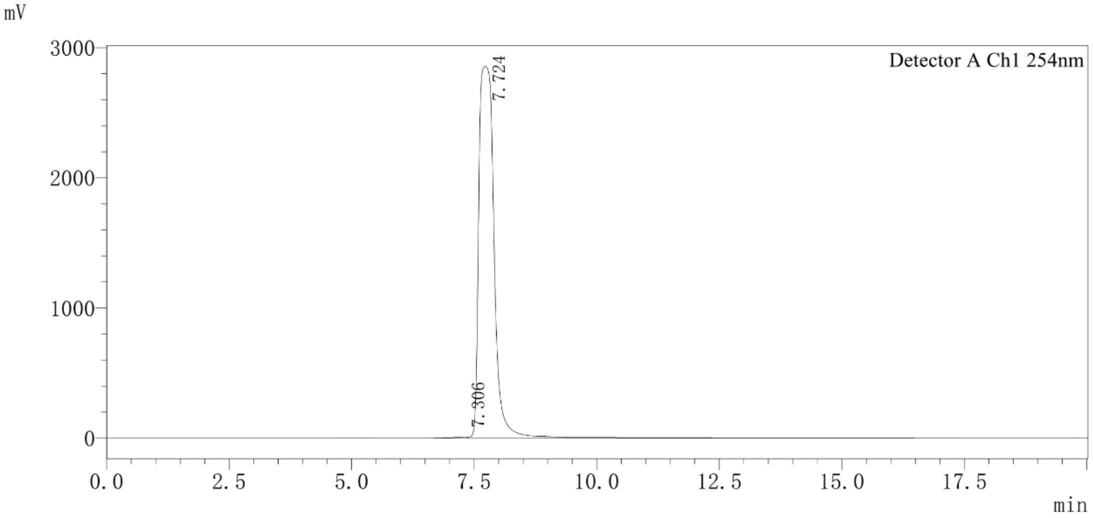

<Peak table>

Detector A Ch1 254nm

Number	Retention time	Peak area	Peak area%	Separation efficiency
1	7.306	188036	0.301	--
2	7.724	62379250	99.699	0.417
Total		62567286	100.000	

(USP)

***1-(4-((4-(4-hydroxyphenyl)piperazin-1-yl)methyl)phenyl)ethan-1-one* (AI10-a9)**

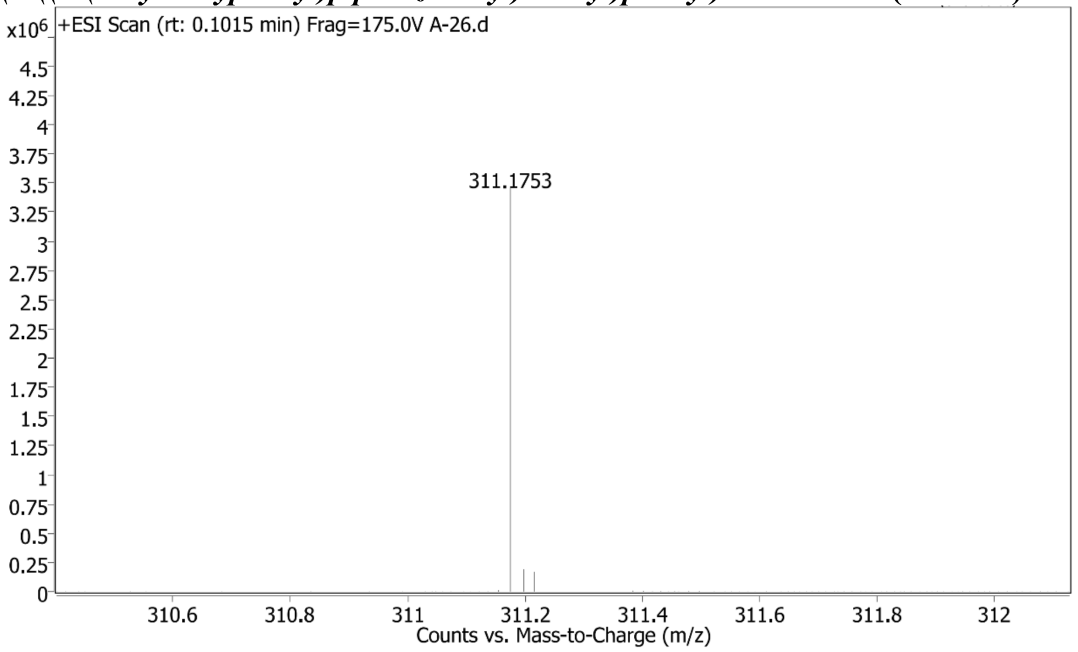

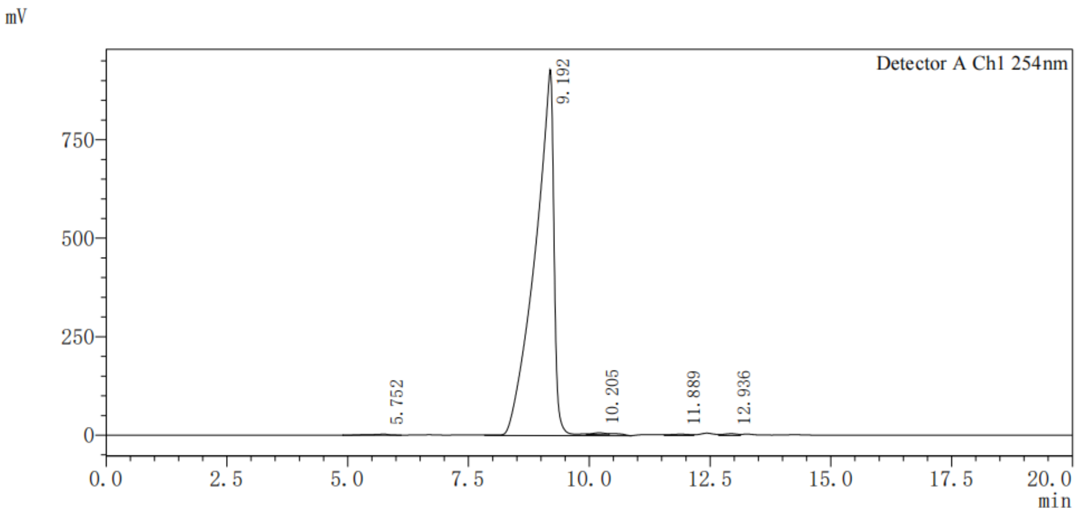

<Peak table>

Detector A Ch1 254nm

Number	Retention time	Peak area	Peak area%	Separation efficiency
1	5.752	91578	0.354	--
2	9.192	25459505	98.480	3.994
3	10.205	101148	0.391	1.432
4	11.889	103354	0.400	1.773
5	12.936	96807	0.374	1.112
Total		25852392	100.000	

(USP)

***4-((4-(4-hydroxyphenyl)piperazin-1-yl)methyl)-2,6-dimethylphenol* (AI10-a10)**

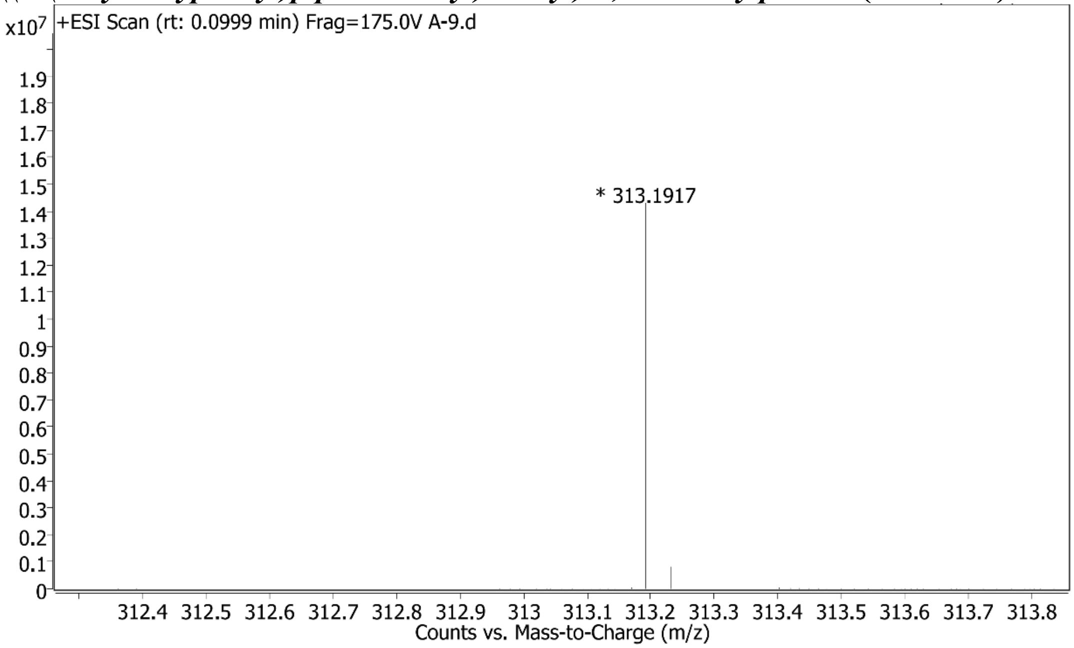

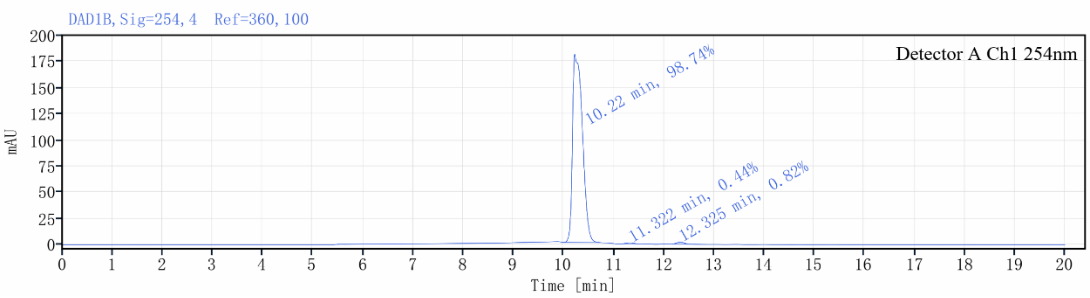

〈Peak table〉

Detector A Ch1 254nm

Number	Retention time	Peak area	Peak area%	Separation efficiency
1	10. 220	2489. 1	98. 74	--
2	11. 322	11. 1	0. 44	--
3	12. 325	20. 7	0. 82	--
Total		2520. 9	100. 00	

(USP)

(4-(4-hydroxyphenyl)piperazin-1-yl)(1-methyl-1H-indazol-6-yl)methanone **(AI10-a11)**

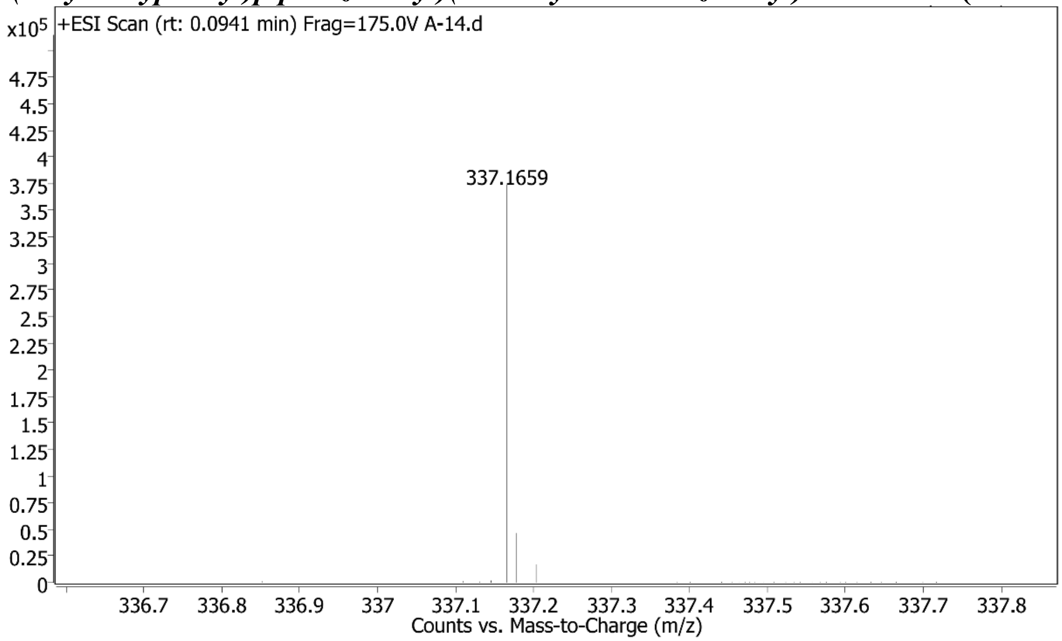

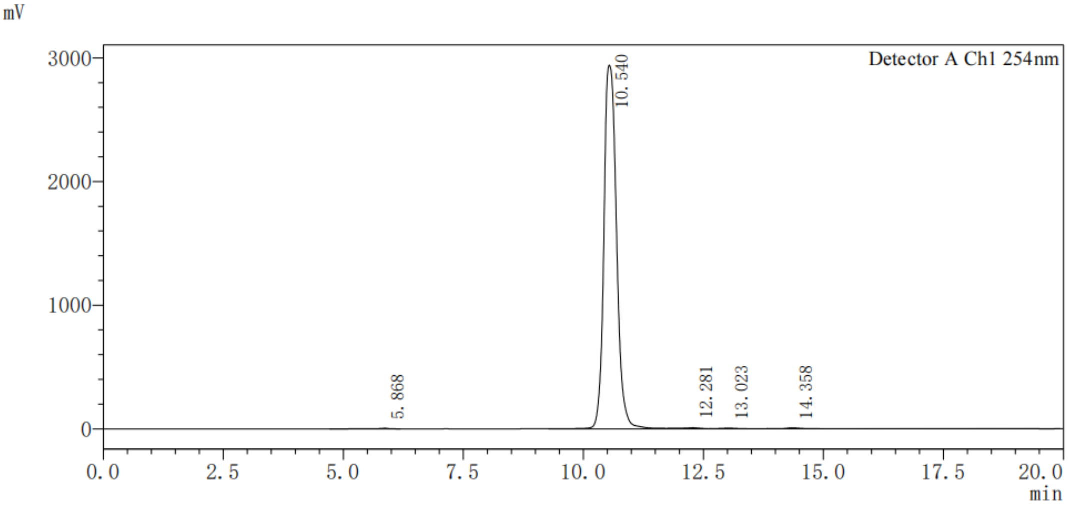

<Peak table>

Detector A Ch1 254nm

Number	Retention time	Peak area	Peak area%	Separation efficiency
1	5.868	93827	0.172	--
2	10.540	54302975	99.268	12.944
3	12.281	84806	0.155	4.303
4	13.023	76094	0.139	1.832
5	14.358	145590	0.266	3.043
Total		54703292	100.000	

(USP)

(4-(4-hydroxyphenyl)piperazin-1-yl)(4-methylthiazol-2-yl)methanone **(AI10-a12)**

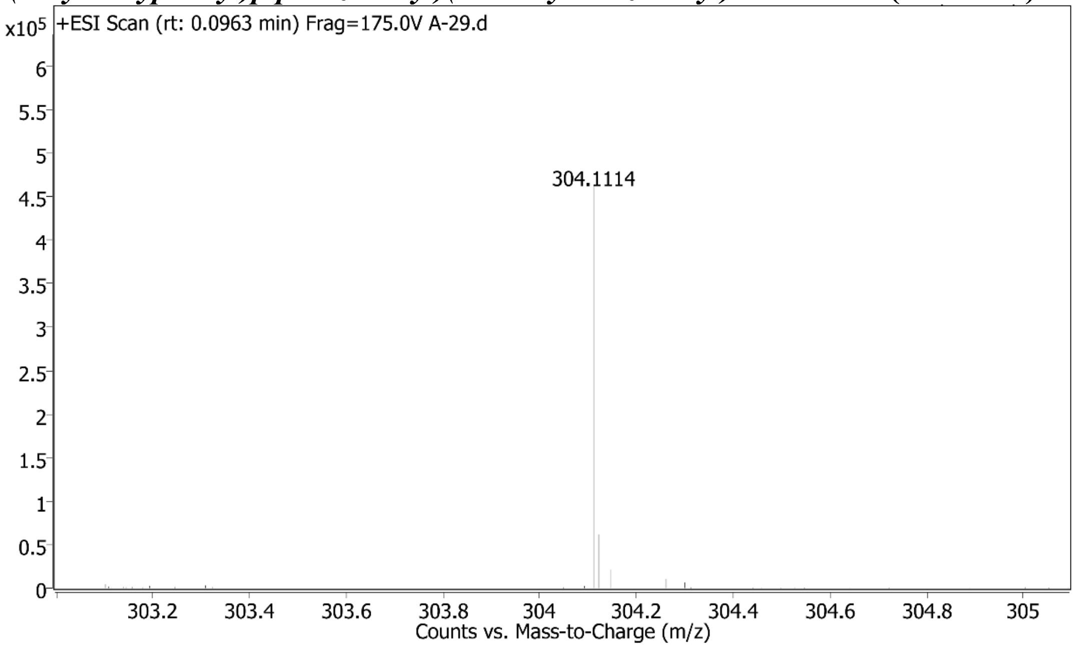

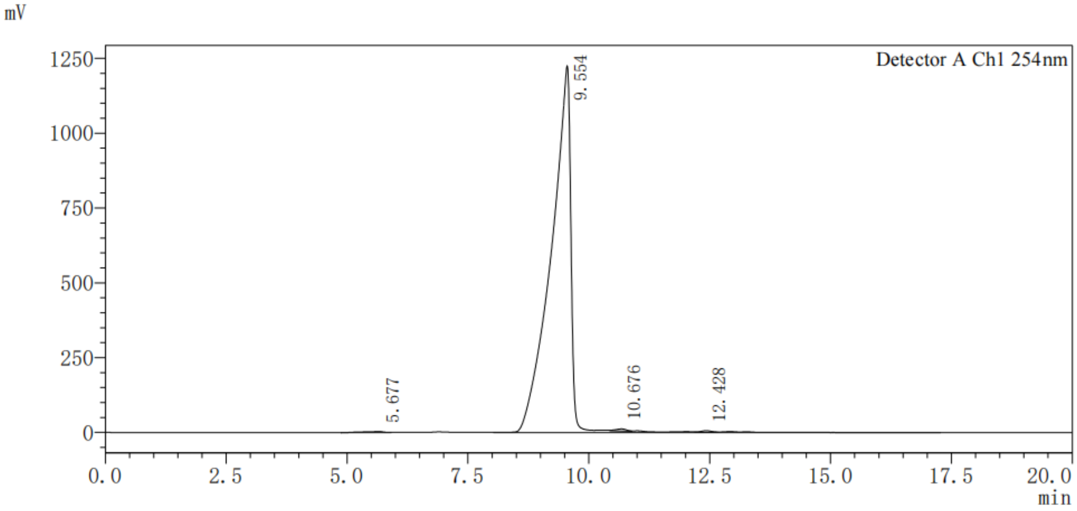

<Peak table>

Detector A Ch1 254nm

Number	Retention time	Peak area	Peak area%	Separation efficiency
1	5.677	94755	0.268	--
2	9.554	35048761	99.163	4.627
3	10.676	114926	0.325	1.977
4	12.428	86097	0.244	3.989
Total		35344539	100.000	

(USP)

(4-(4-hydroxyphenyl)piperazin-1-yl)(1-methyl-1H-indol-2-yl)methanone **(AI10-a13)**

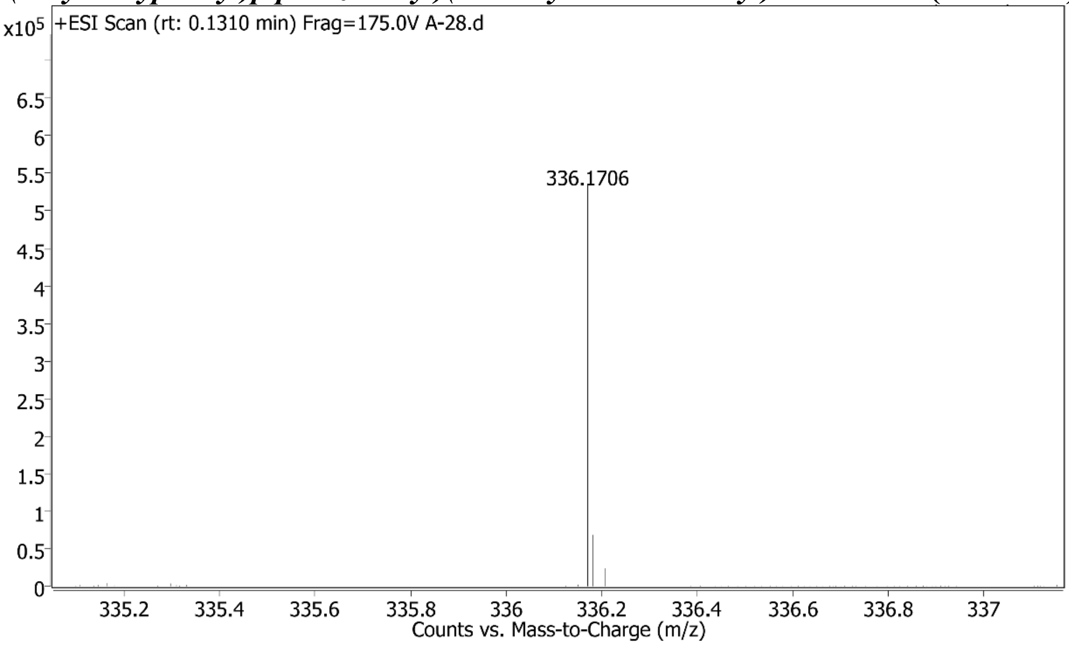

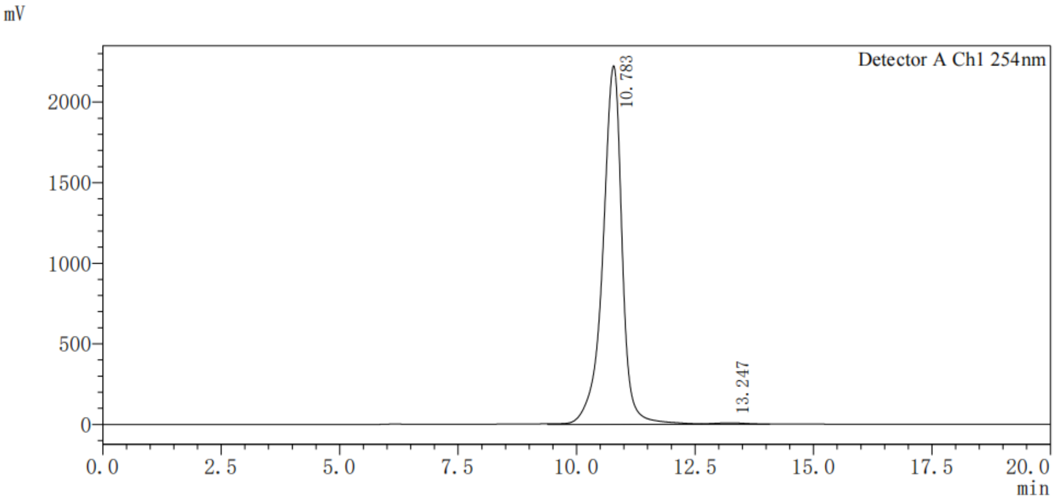

<Peak table>

Detector A Ch1 254nm

Number	Retention time	Peak area	Peak area%	Separation efficiency
1	10. 783	63201930	99. 188	--
2	13. 247	517307	0. 812	2. 302
Total		63719237	100. 000	

(USP)

4-((4-(4-hydroxyphenyl)piperazin-1-yl)methyl)-3,5-dimethylphenol **(AI10-a14)**

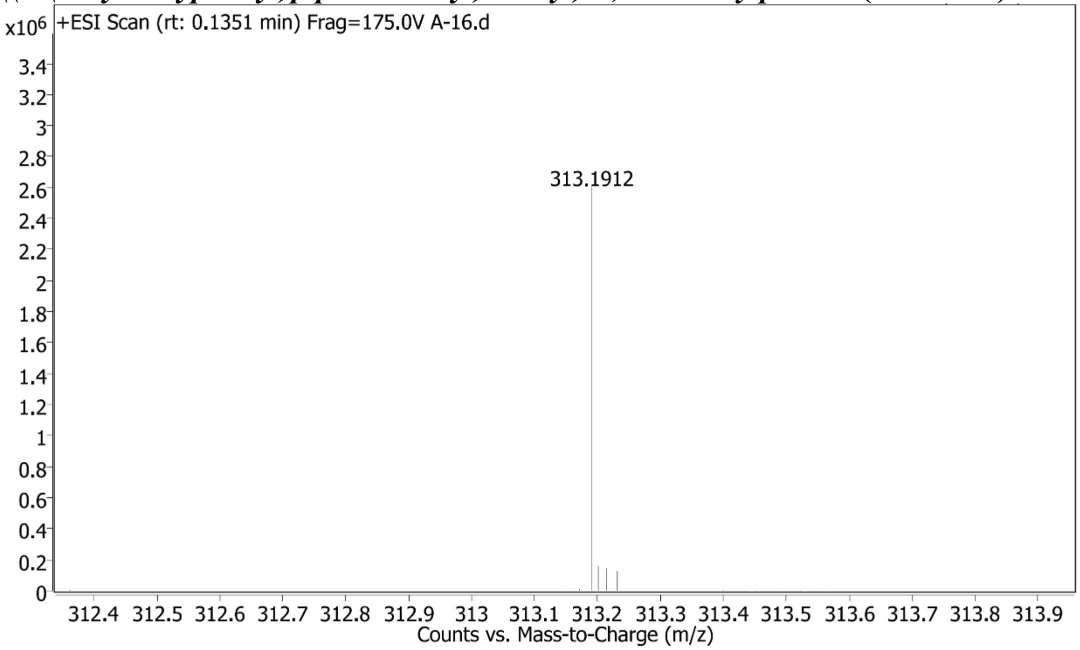

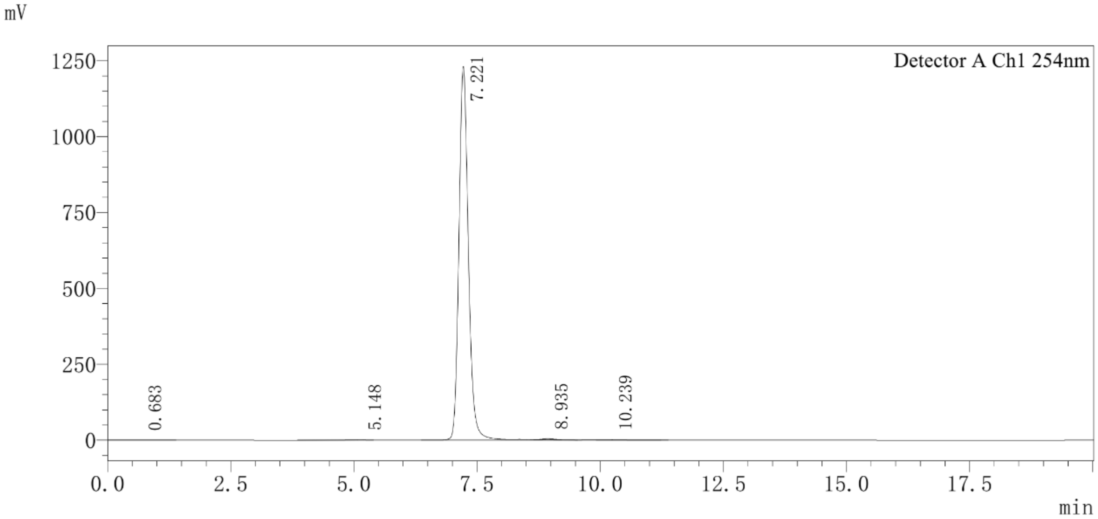

<Peak table>

Detector A Ch1 254nm

Number	Retention time	Peak area	Peak area%	Separation efficiency
1	0.683	30275	0.180	--
2	5.148	55140	0.328	5.484
3	7.221	16625420	98.879	5.318
4	8.935	68946	0.410	3.908
5	10.239	34180	0.203	2.063
Total		16813962	100.000	

(USP)

4-((4-(4-hydroxyphenyl)piperazin-1-yl)methyl)-3-methylphenol **(AI10-a15)**

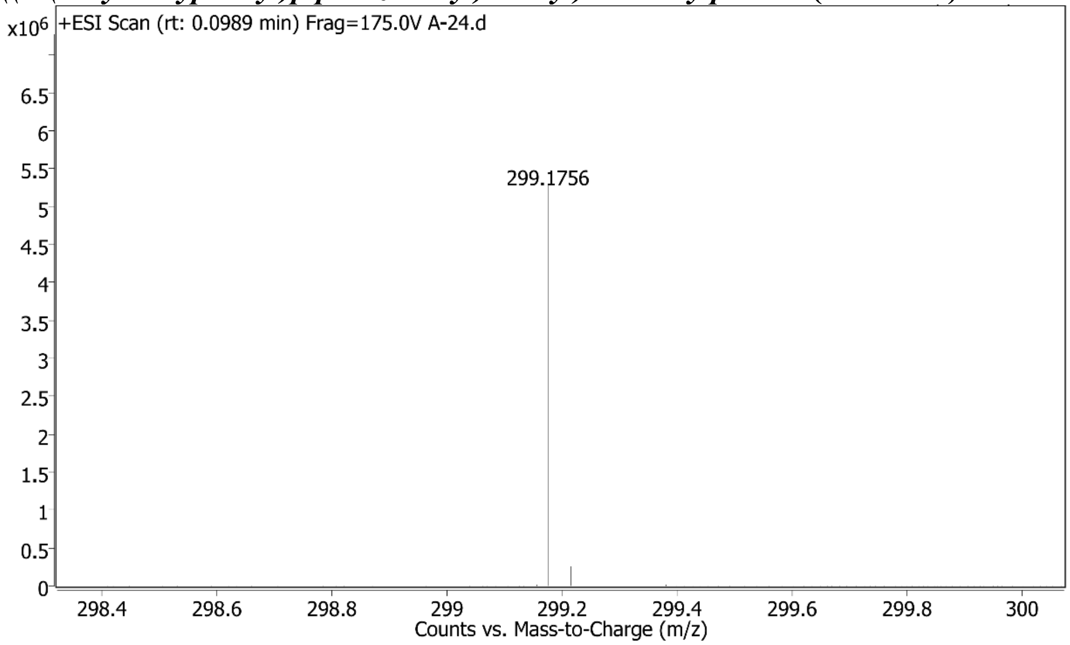

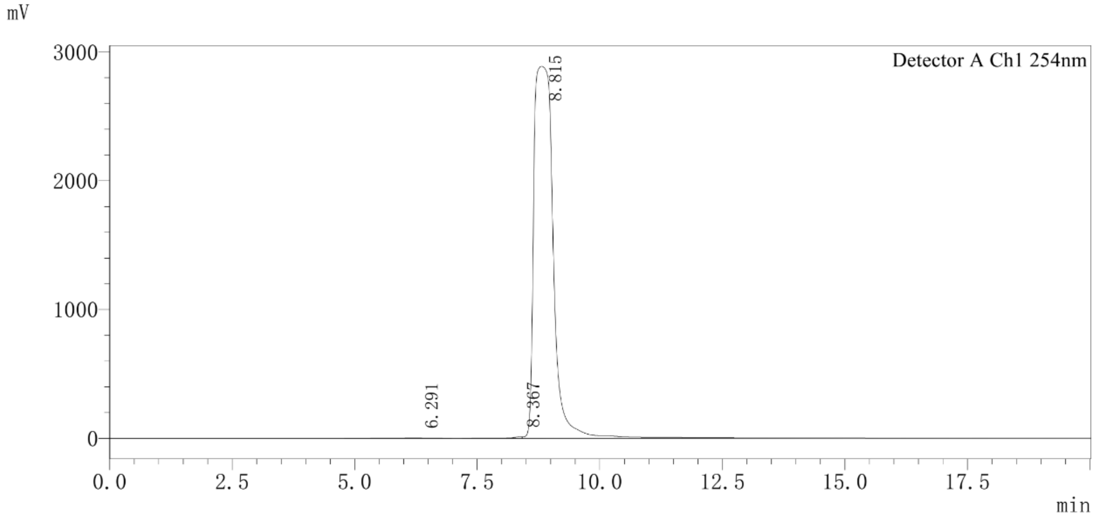

<Peak table>

Detector A Ch1 254nm

Number	Retention time	Peak area	Peak area%	Separation efficiency
1	6.291	123985	0.153	--
2	8.367	159039	0.197	1.996
3	8.815	80561090	99.650	0.458
Total		80844114	100.000	

(USP)

***4-((4-(4-hydroxyphenyl)piperazin-1-yl)methyl)-2-methylphenol* (AI10-a16)**

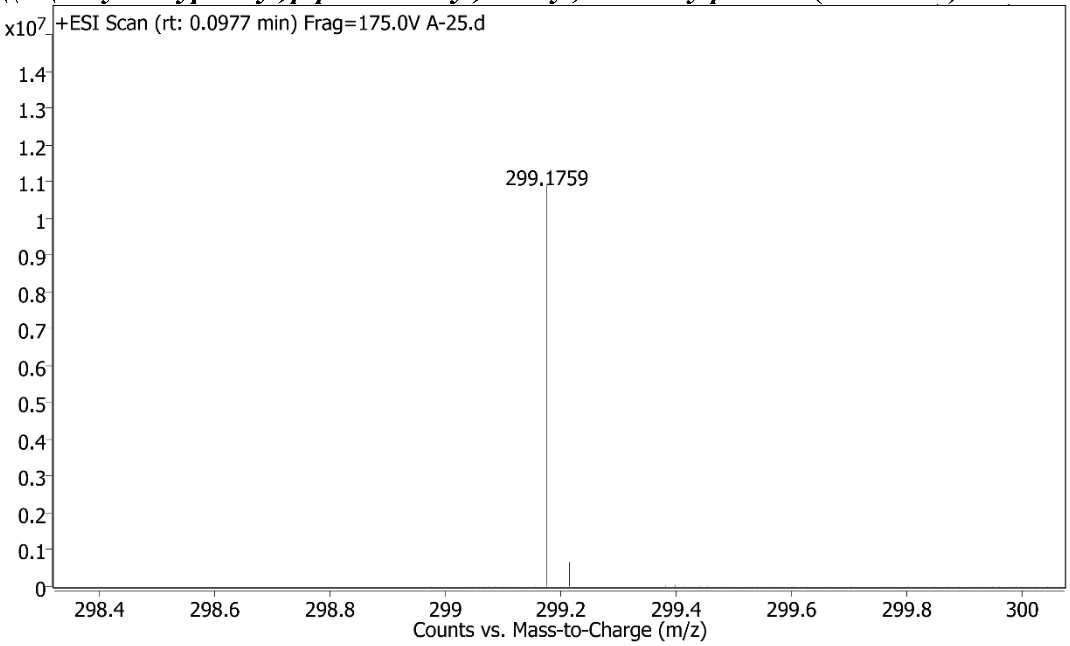

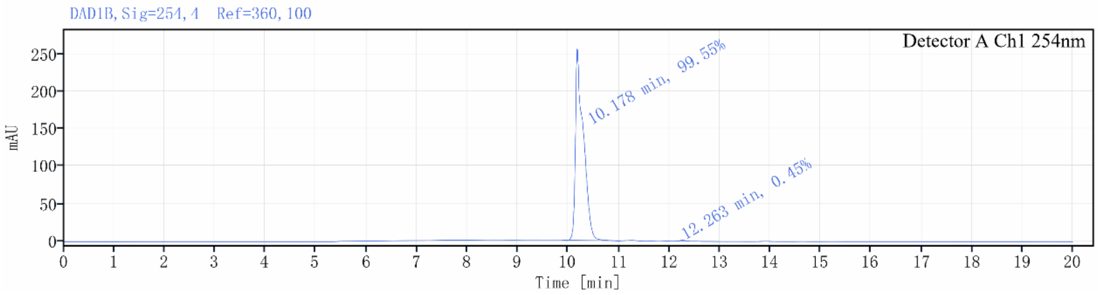

〈Peak table〉

Detector A Ch1 254nm

Number	Retention time	Peak area	Peak area%	Separation efficiency
1	10.178	2784.1	99.55	--
2	12.263	12.5	0.45	--
Total		2796.6	100.00	

(USP)

(2-chloro-6-methylpyridin-3-yl)(4-(3-hydroxyphenyl)piperazin-1-yl)methanone **(AI10-a17)**

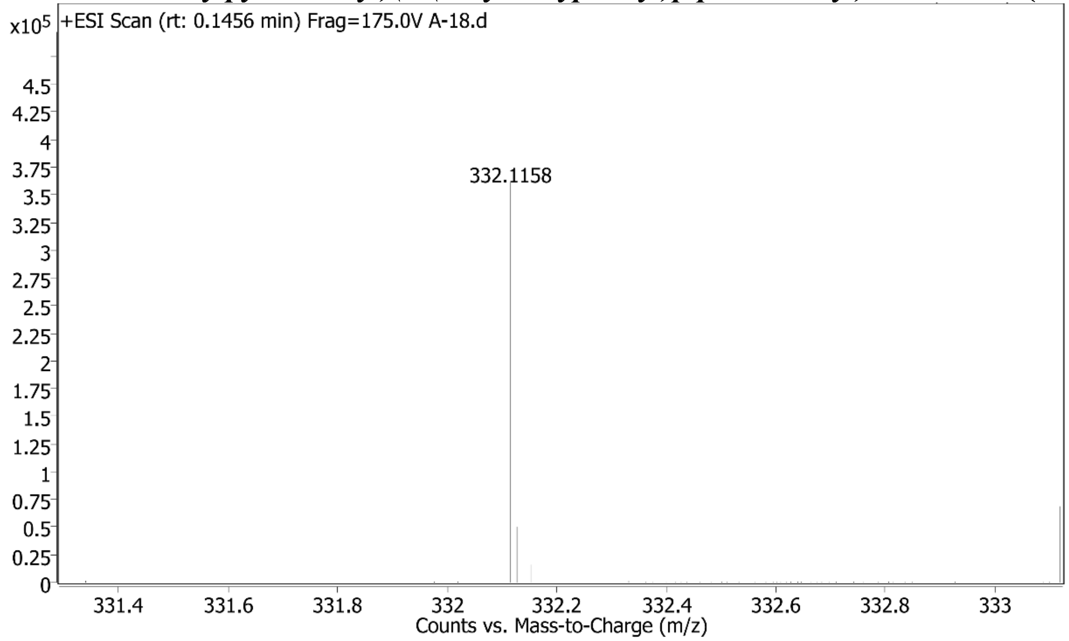

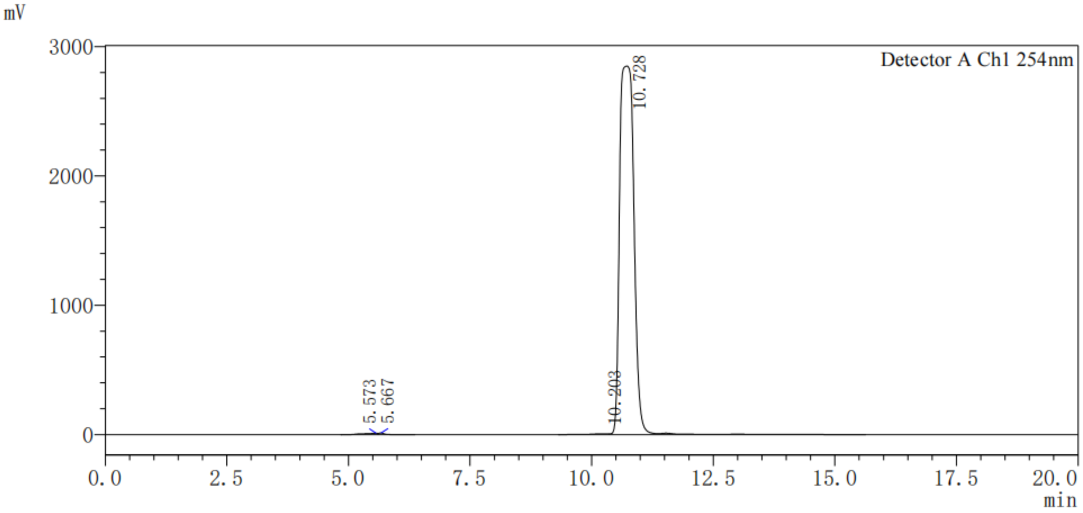

<Peak table>

Detector A Ch1 254nm

Number	Retention time	Peak area	Peak area%	Separation efficiency
1	5. 573	213544	0. 360	--
2	5. 667	90224	0. 152	0. 092
3	10. 203	177172	0. 299	5. 471
4	10. 728	58768625	99. 188	0. 613
Total		59249565	100. 000	

(USP)

2-(4-hydroxyphenyl)-1-(4-(3-hydroxyphenyl)piperazin-1-yl)propan-1-one **(AI10-a18)**

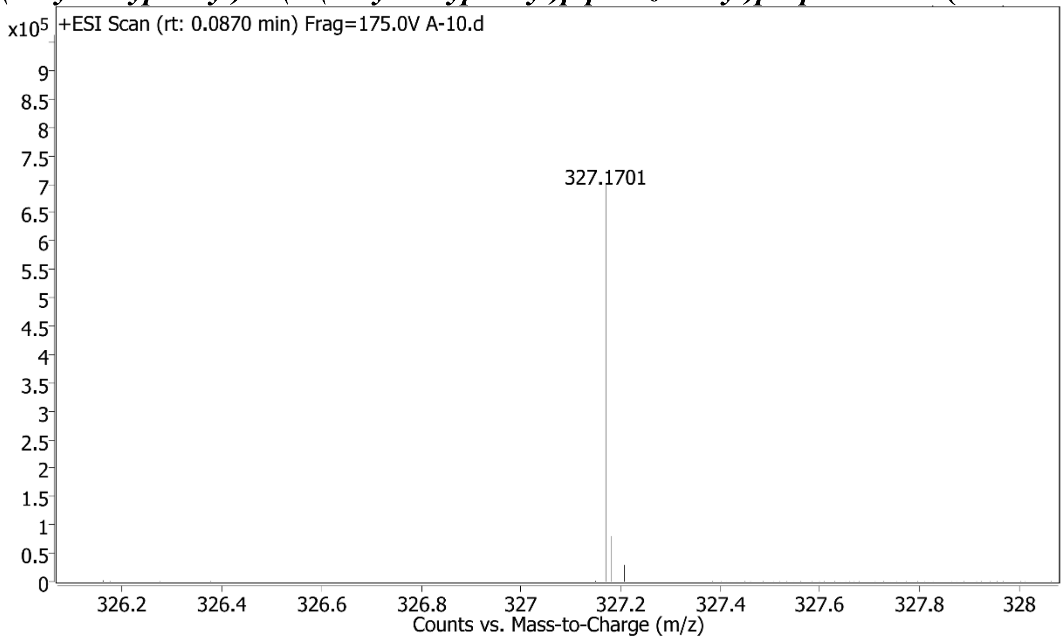

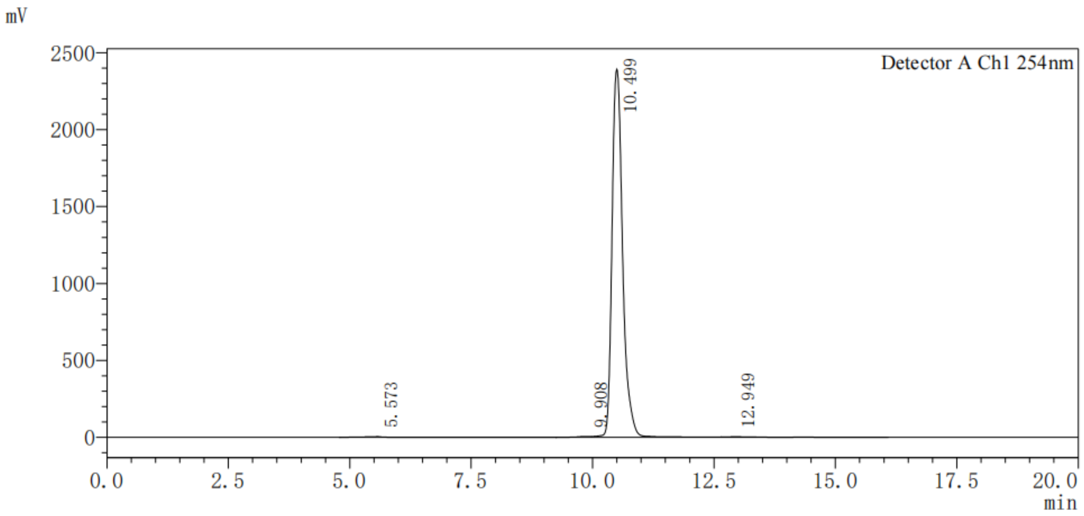

<Peak table>

Detector A Ch1 254nm

Number	Retention time	Peak area	Peak area%	Separation efficiency
1	5.573	94759	0.258	--
2	9.908	95921	0.261	2.853
3	10.499	36487349	99.368	0.386
4	12.949	41509	0.113	6.479
Total		36719538	100.000	

(USP)

(4-hydroxyphenyl)(4-(p-tolyl)piperazin-1-yl)methanone **(AI10-a19)**

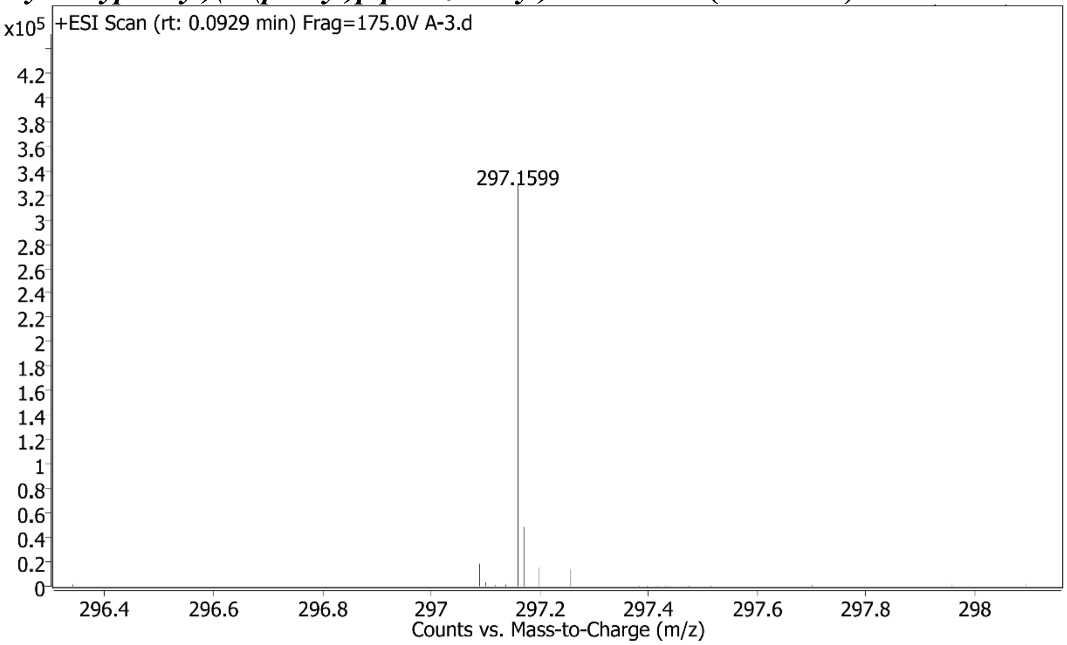

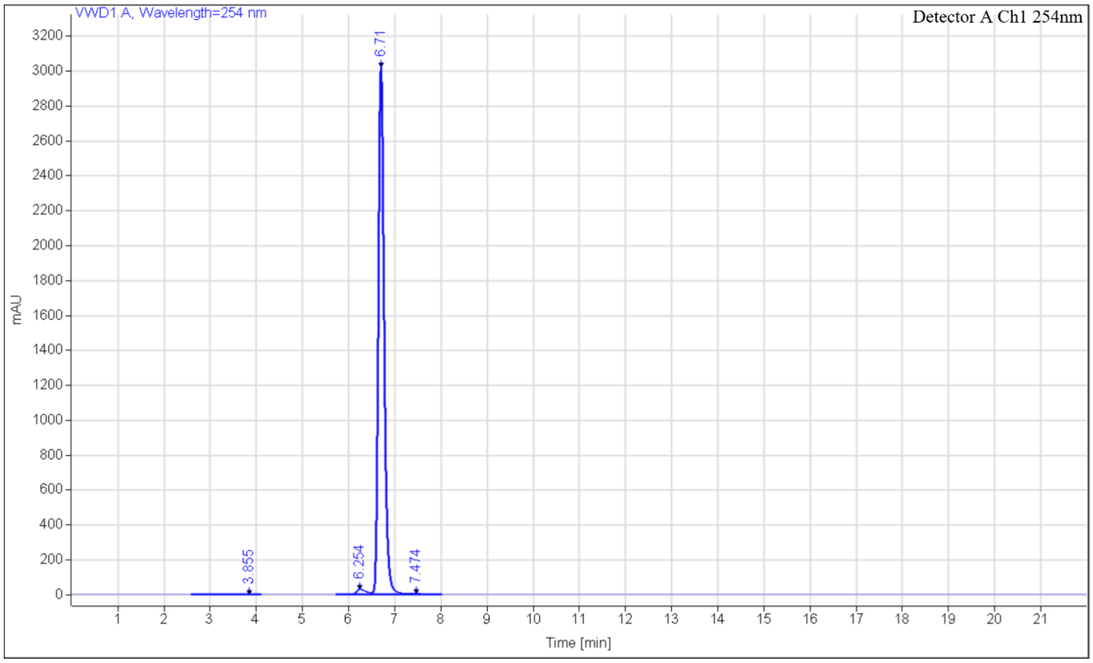

<Peak table>

Detector A Ch1 254nm

Number	Retention time	Peak area	Peak area%	Separation efficiency
1	3. 855	52. 40270	0. 20	--
2	6. 254	414. 47623	1. 54	7. 34211
3	6. 710	26348. 51563	98. 05	1. 53291
4	7. 474	56. 21617	0. 21	2. 05857
Total		26871. 61073	100. 00	

(USP)

(3,4-dihydroxyphenyl)(4-(p-tolyl)piperazin-1-yl)methanone **(AI10-a20)**

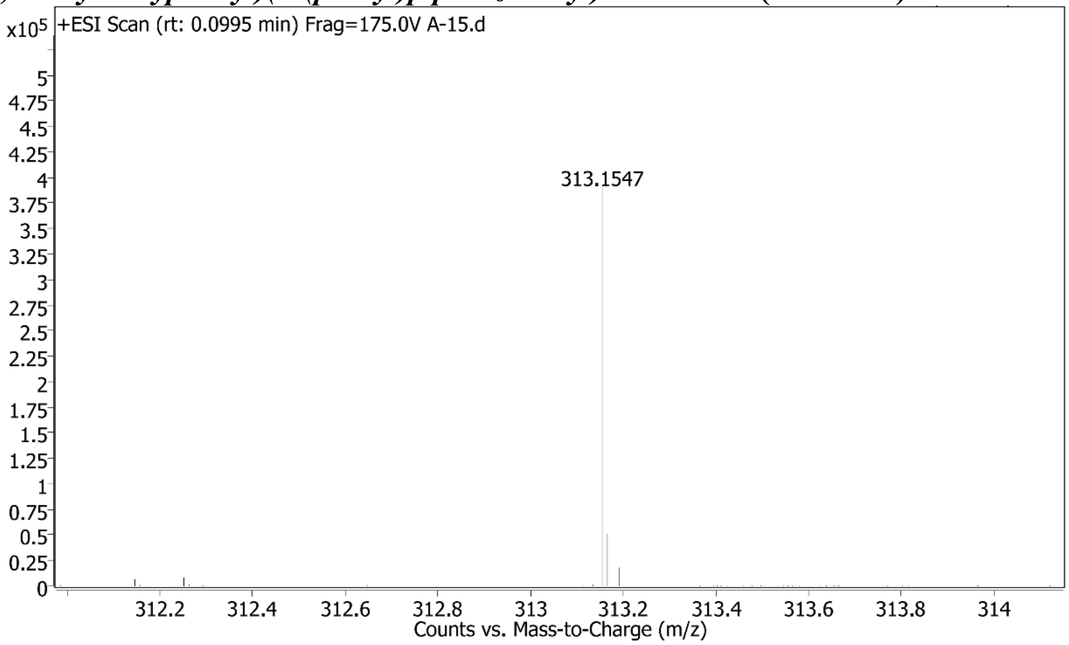

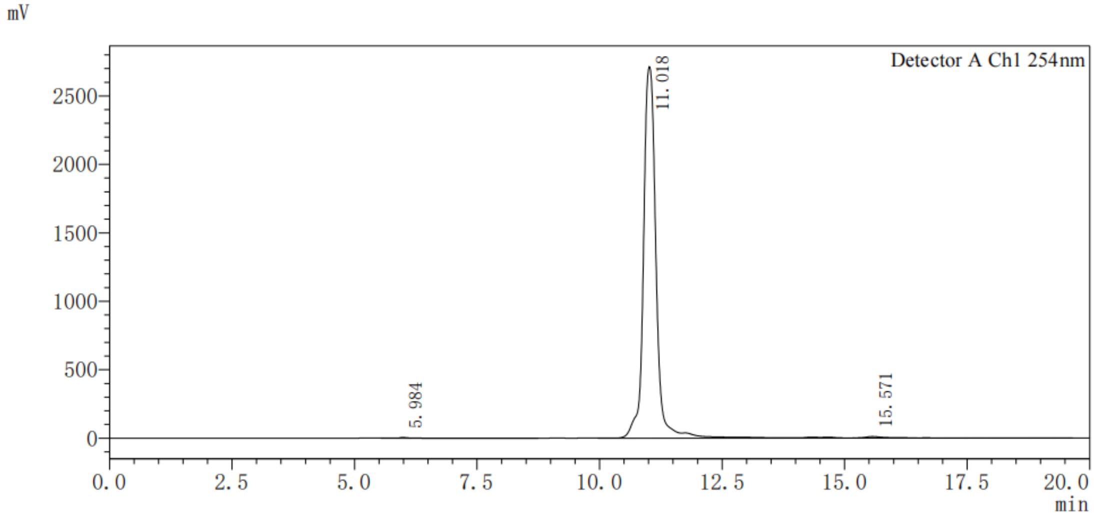

<Peak table>

Detector A Ch1 254nm

Number	Retention time	Peak area	Peak area%	Separation efficiency
1	5.984	85695	0.174	--
2	11.018	48994401	99.314	15.432
3	15.571	252537	0.512	9.421
Total		49332633	100.000	

(USP)

***(2,5-dihydroxyphenyl)(4-(p-tolyl)piperazin-1-yl)methanone* (AI10-a21)**

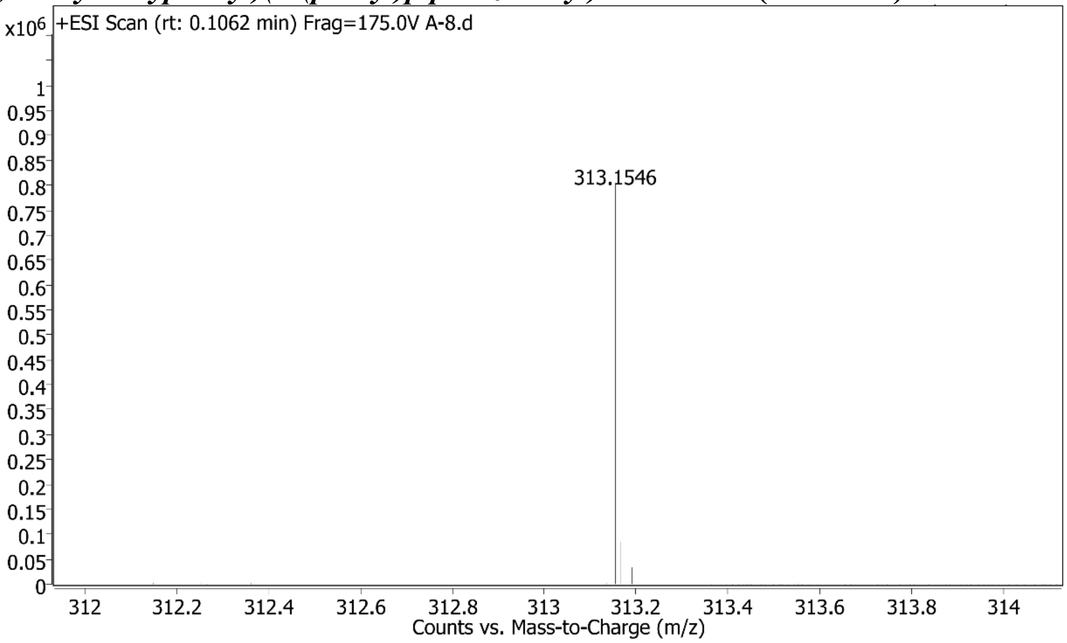

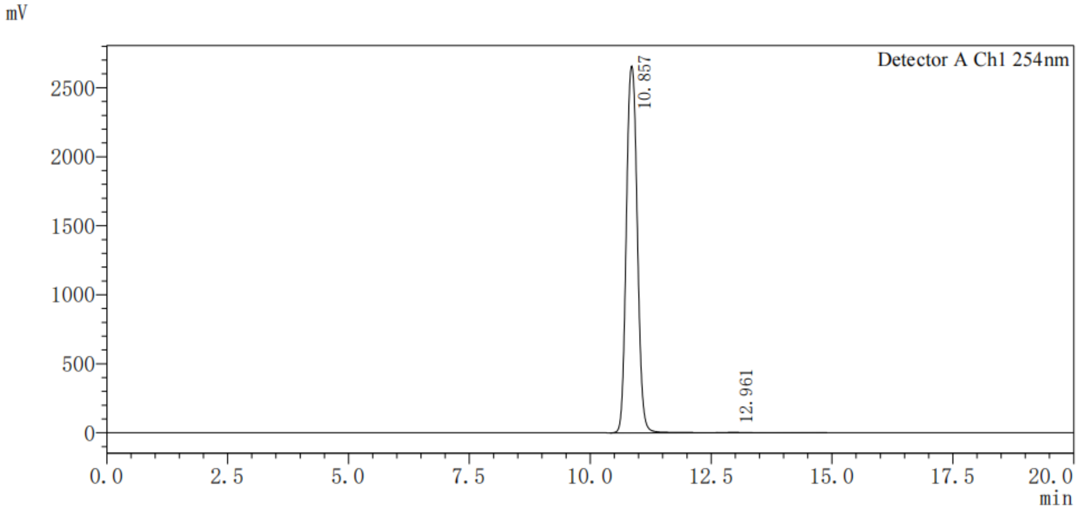

<Peak table>

Detector A Ch1 254nm

Number	Retention time	Peak area	Peak area%	Separation efficiency
1	10.857	42084131	99.924	--
2	12.961	32057	0.076	5.199
Total		42116189	100.000	

(USP)

(3,5-dihydroxyphenyl)(4-(p-tolyl)piperazin-1-yl)methanone **(AI10-a22)**

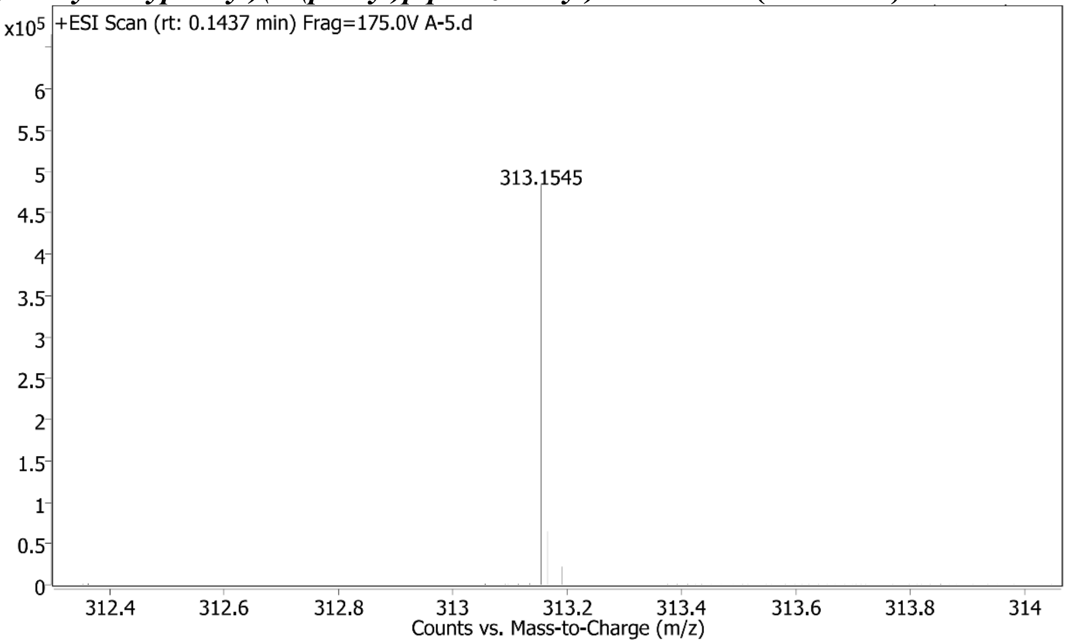

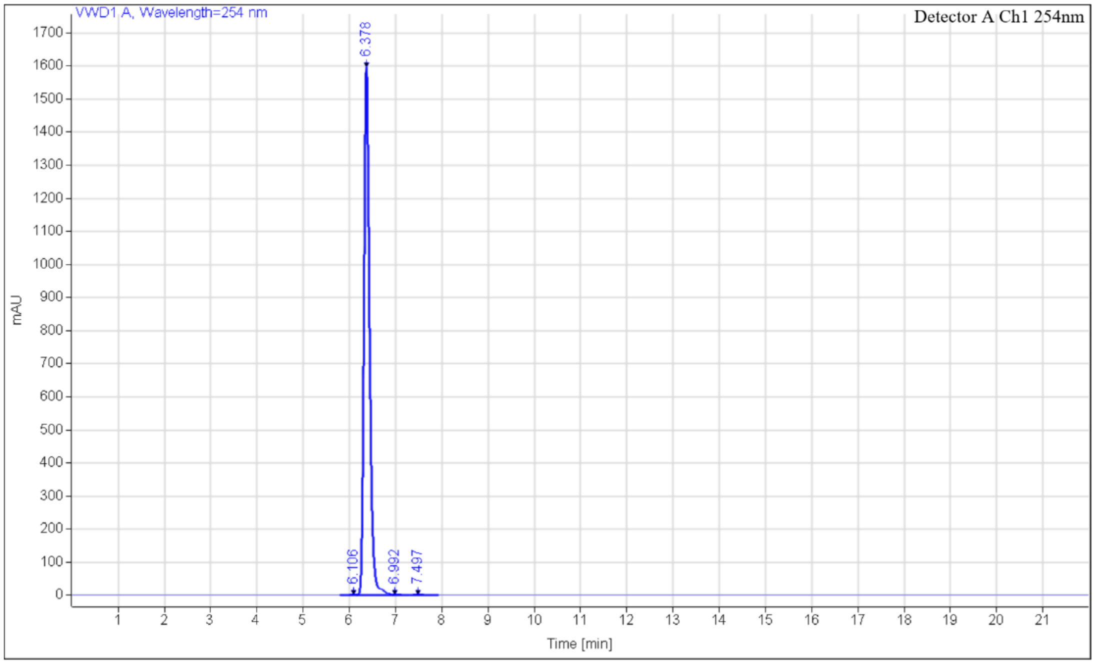

<Peak table>
Detector A Ch1 254nm

Number	Retention time	Peak area	Peak area%	Separation efficiency
1	6. 106	25. 83263	0. 19	--
2	6. 378	13457. 76758	99. 42	0. 88836
3	6. 992	23. 83414	0. 18	0. 66233
4	7. 497	28. 23927	0. 21	0. 53440
Total		13535. 67362	100. 00	

(USP)

(3-fluoro-5-hydroxyphenyl)(4-(p-tolyl)piperazin-1-yl)methanone **(AI10-a23)**

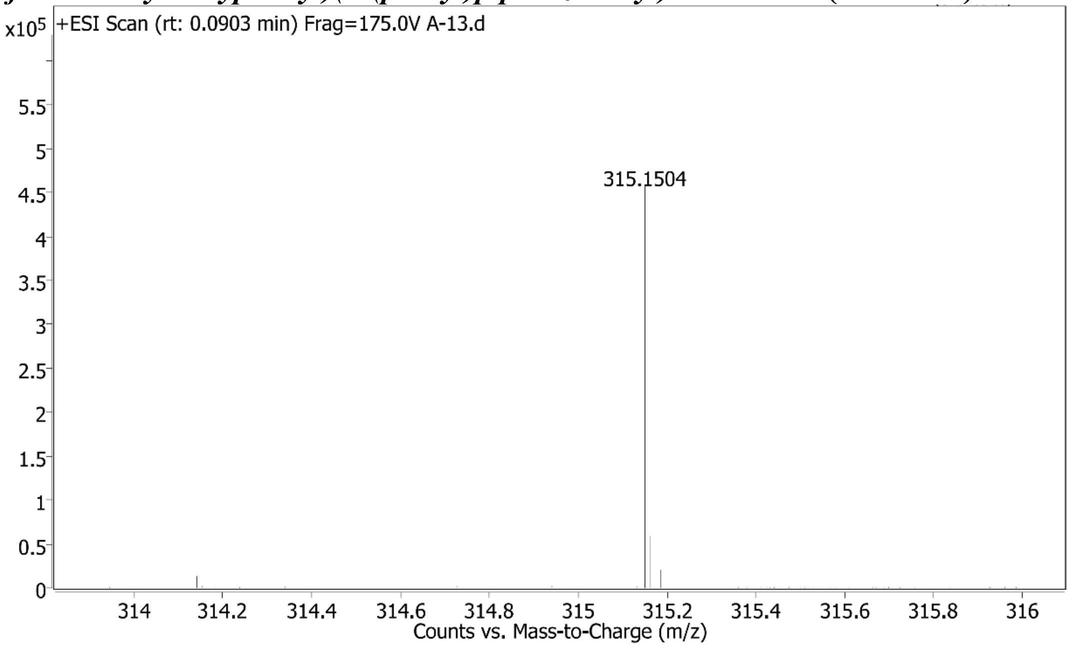

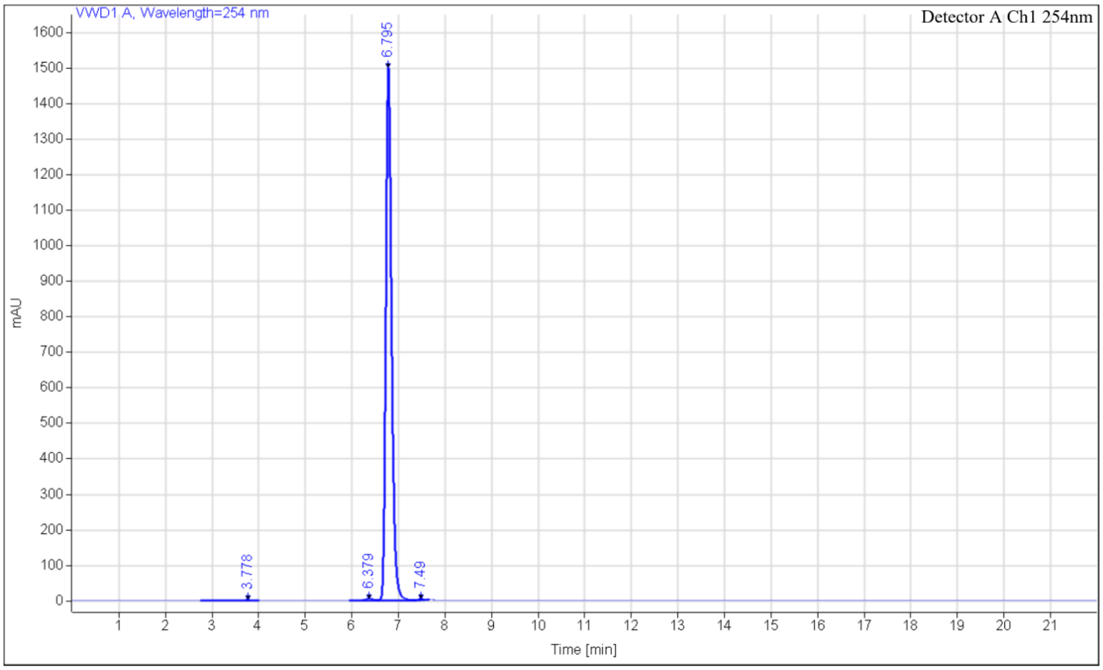

〈Peak table〉

Detector A Ch1 254nm

Number	Retention time	Peak area	Peak area%	Separation efficiency
1	3. 778	39. 92621	0. 31	--
2	6. 379	66. 17012	0. 51	6. 86512
3	6. 795	12873. 26367	98. 96	1. 47401
4	7. 490	28. 66509	0. 22	2. 88968
Total		13008. 02509	100. 00	

(USP)

(2-chloro-5-hydroxyphenyl)(4-(p-tolyl)piperazin-1-yl)methanone **(AI10-a24)**

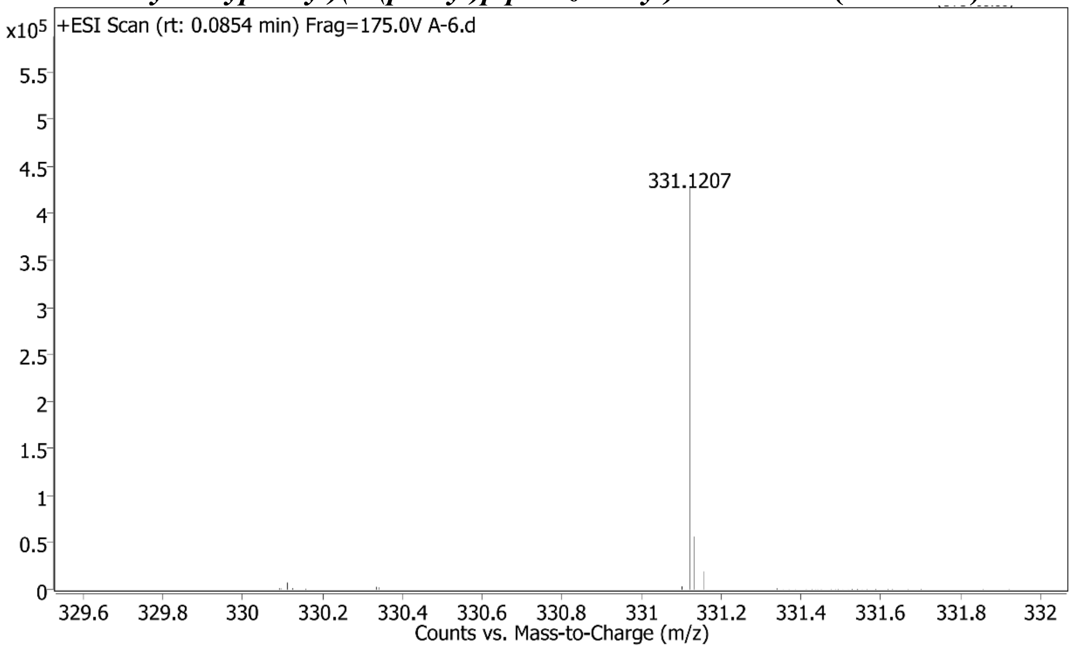

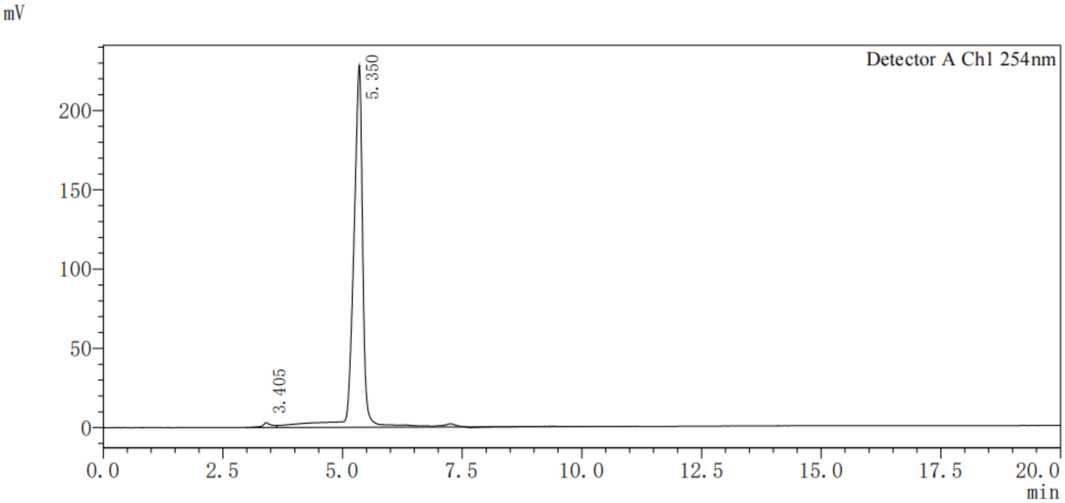

<Peak table>

Detector A Ch1 254nm

Number	Retention time	Peak area	Peak area%	Separation efficiency
1	3.405	41813	1.293	--
2	5.350	3192355	98.707	6.150
Total		3234168	100.000	

(USP)

3-(4-hydroxyphenyl)-1-(4-(p-tolyl)piperazin-1-yl)propan-1-one **(AI10-a25)**

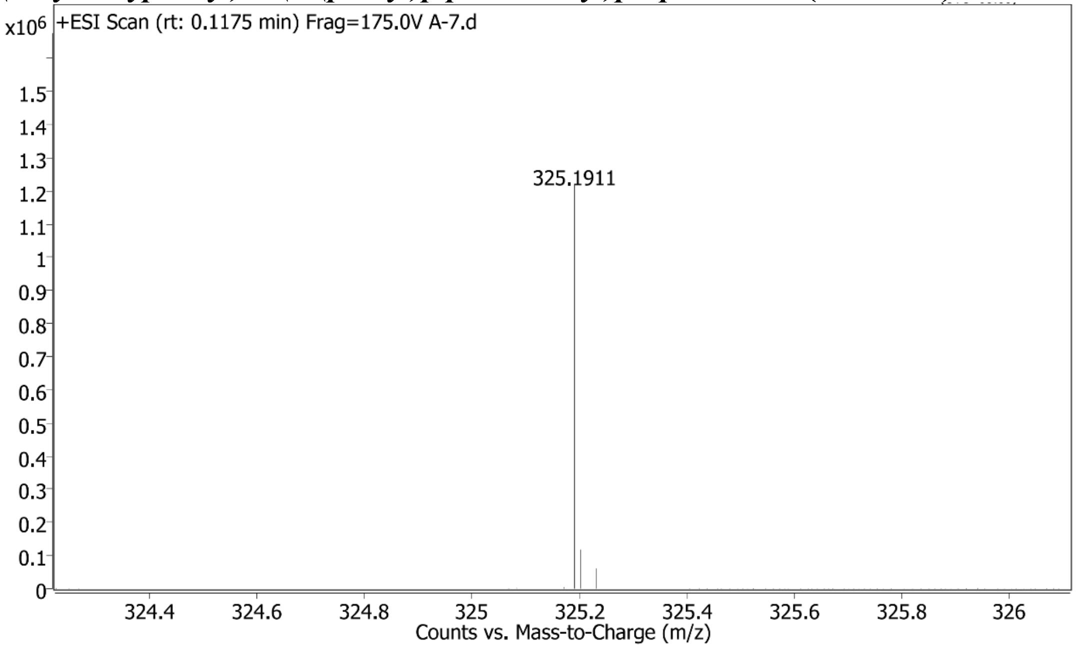

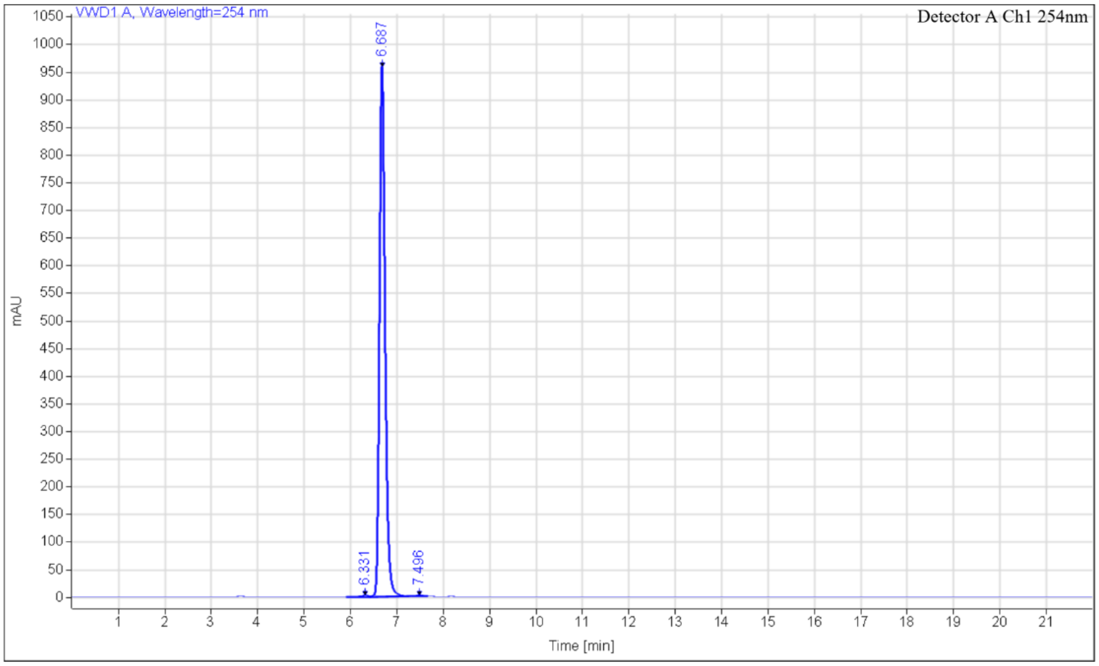

〈Peak table〉

Detector A Ch1 254nm

Number	Retention time	Peak area	Peak area%	Separation efficiency
1	6. 331	32. 00441	0. 39	--
2	6. 687	8126. 08154	99. 32	1. 30330
3	7. 496	23. 63216	0. 29	3. 51651
Total		8181. 71811	100. 00	

(USP)

(4-(4-chlorophenyl)piperazin-1-yl)(3-hydroxy-5-methylphenyl)methanone **(AI10-a26)**

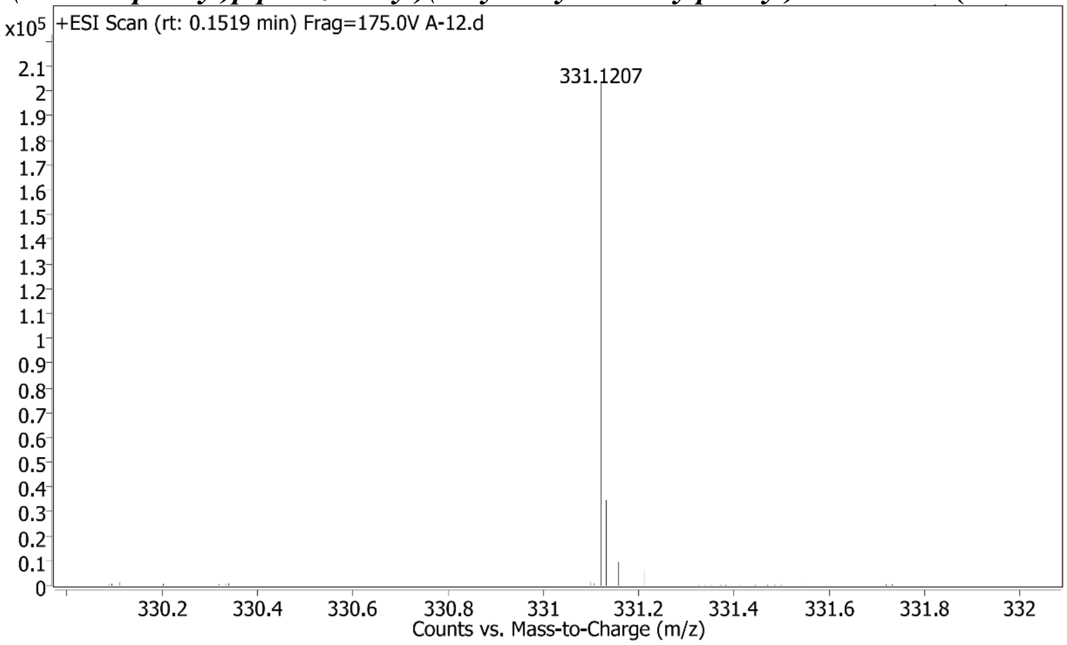

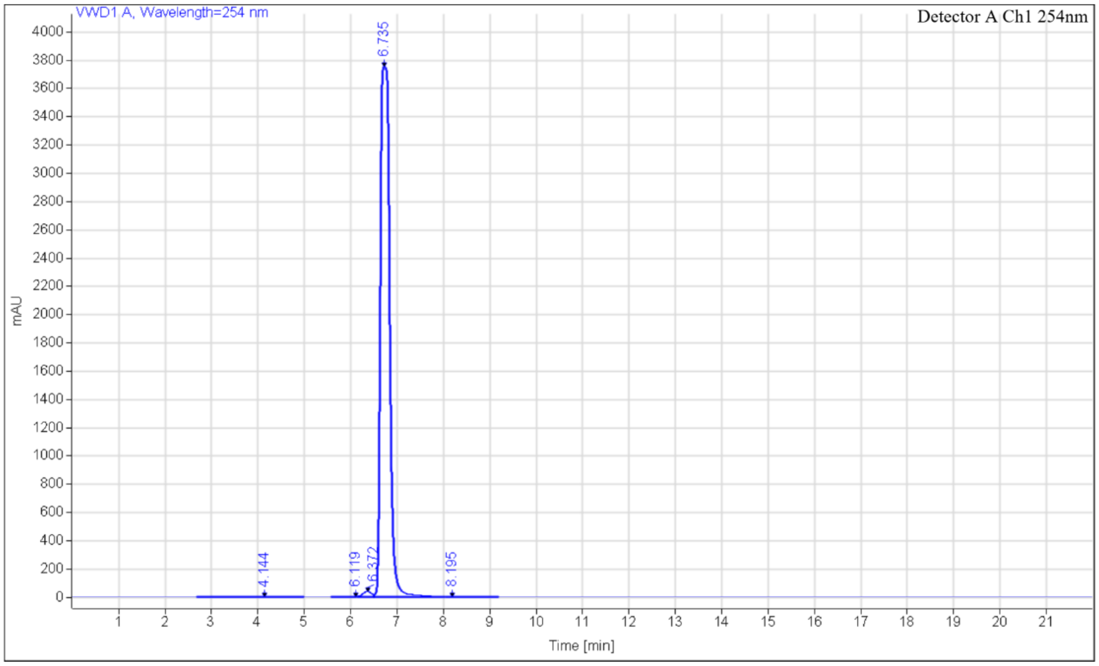

〈Peak table〉

Detector A Ch1 254nm

Number	Retention time	Peak area	Peak area%	Separation efficiency
1	4. 144	117. 52649	0. 22	--
2	6. 119	31. 68930	0. 06	2. 51719
3	6. 372	490. 88986	0. 93	0. 38277
4	6. 735	51957. 20313	98. 67	1. 10679
5	8. 195	60. 96643	0. 12	3. 70880
Total		52658. 27521	100. 00	

(USP)

(4-(4-chlorophenyl)piperazin-1-yl)(5-hydroxy-2-methylphenyl)methanone **(AI10-a27)**

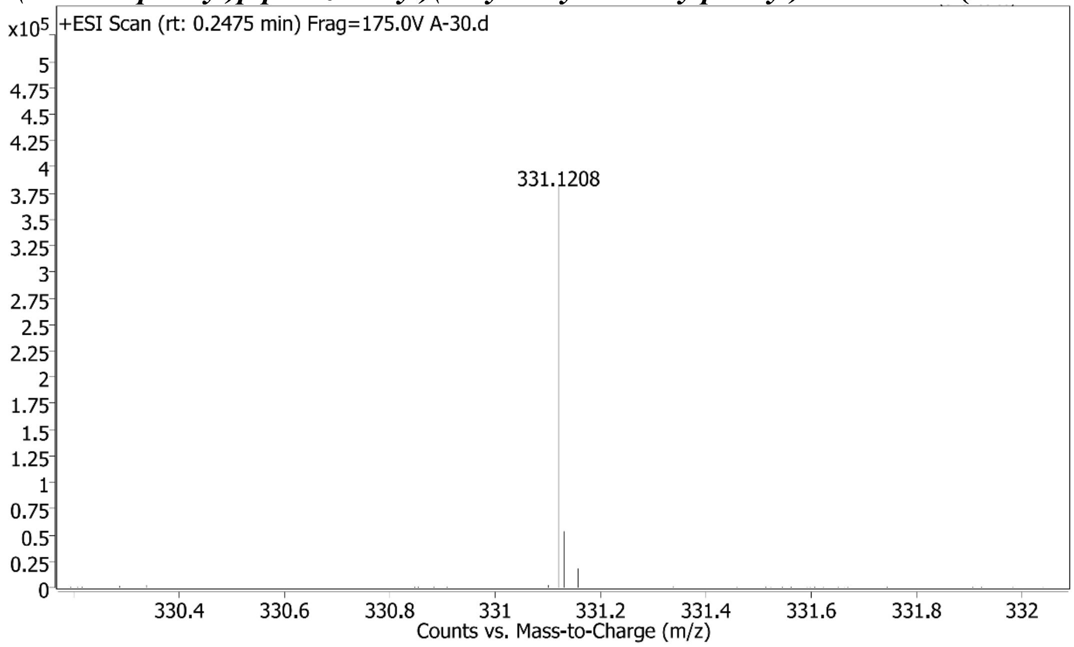

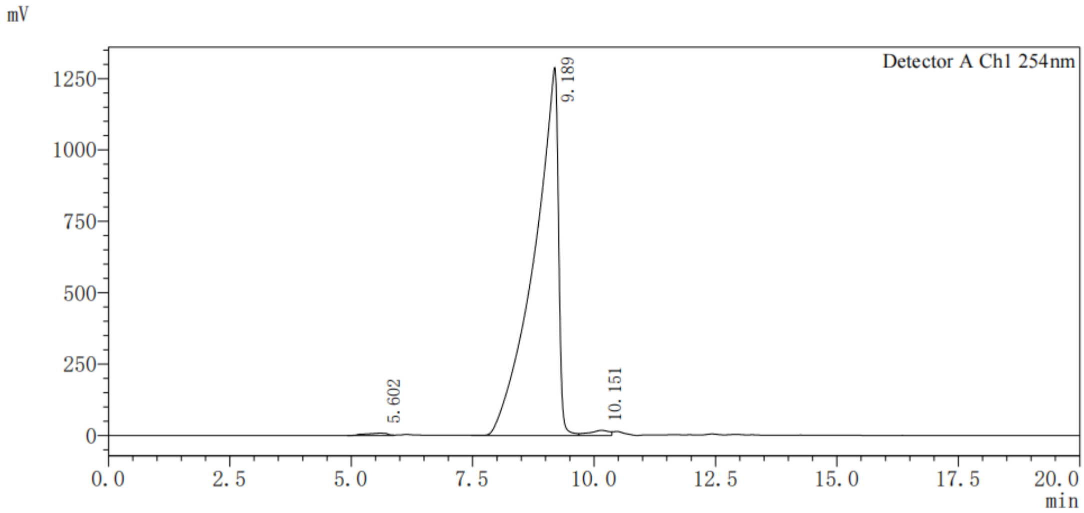

<Peak table>

Detector A Ch1 254nm

Number	Retention time	Peak area	Peak area%	Separation efficiency
1	5.602	250675	0.548	--
2	9.189	45031269	98.359	3.883
3	10.151	500807	1.094	1.102
Total		45782750	100.000	

(USP)

(2,6-dimethylpyridin-4-yl)(6-hydroxy-3,4-dihydroisoquinolin-2(1H) yl)methanone **(AI10-a28)**

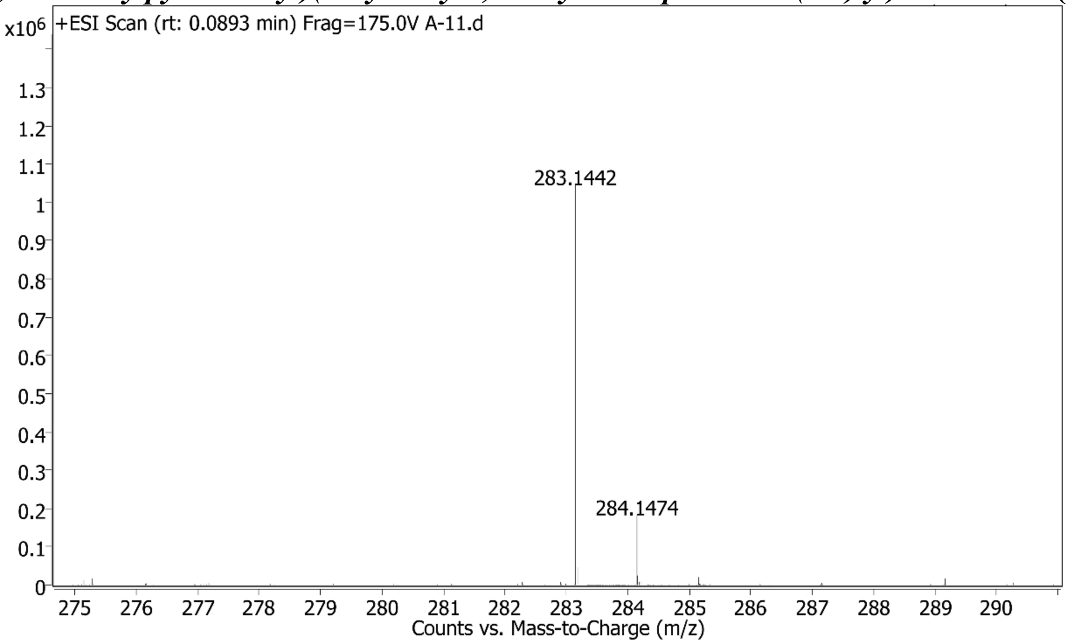

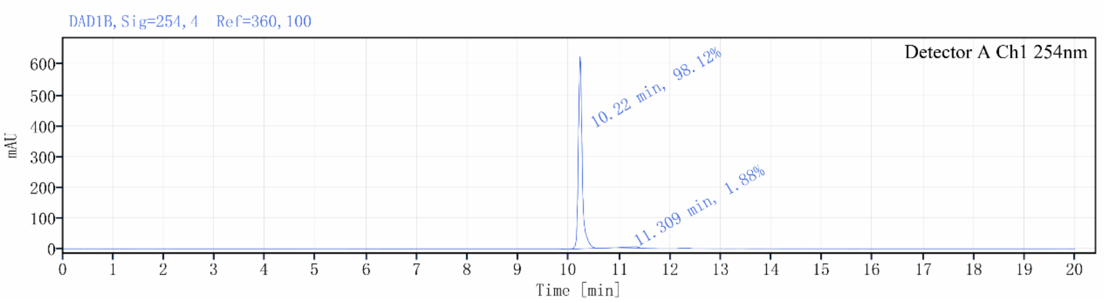

〈Peak table〉

Detector A Ch1 254nm

Number	Retention time	Peak area	Peak area%	Separation efficiency
1	10. 220	3395. 0	98. 12	--
2	11. 309	65. 1	1. 88	--
Total		3460. 1	100. 00	

(USP)

***4-(4-(4-hydroxy-3,5-dimethylbenzoyl)piperazin-1-yl)benzonitrile* (AI10-a29)**

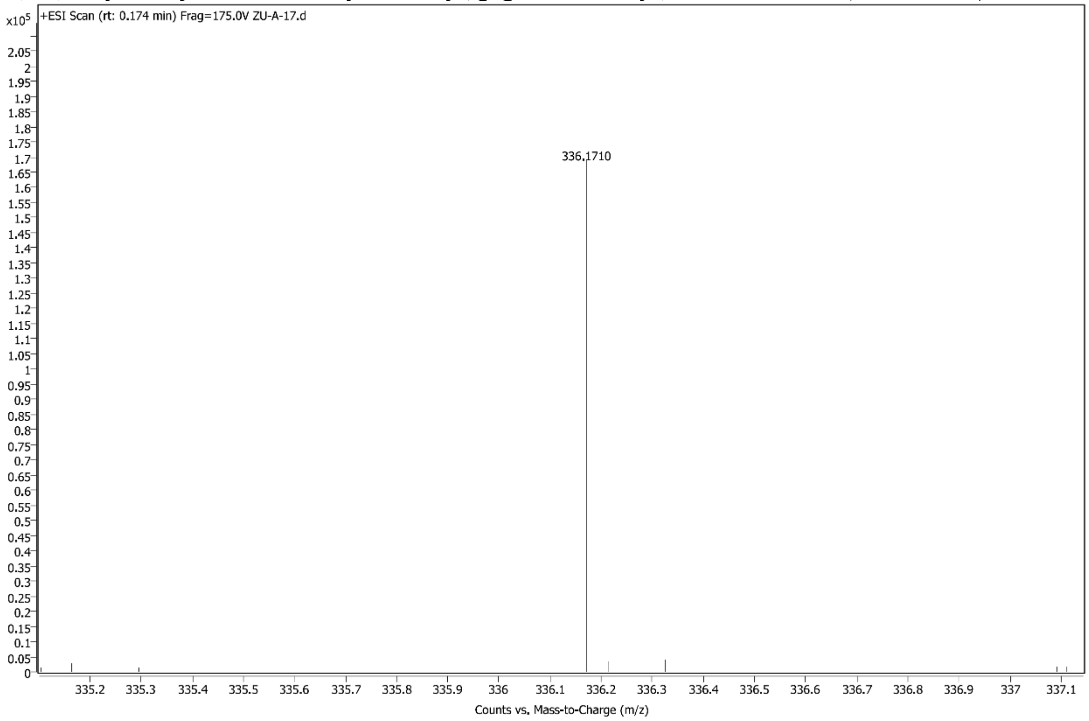

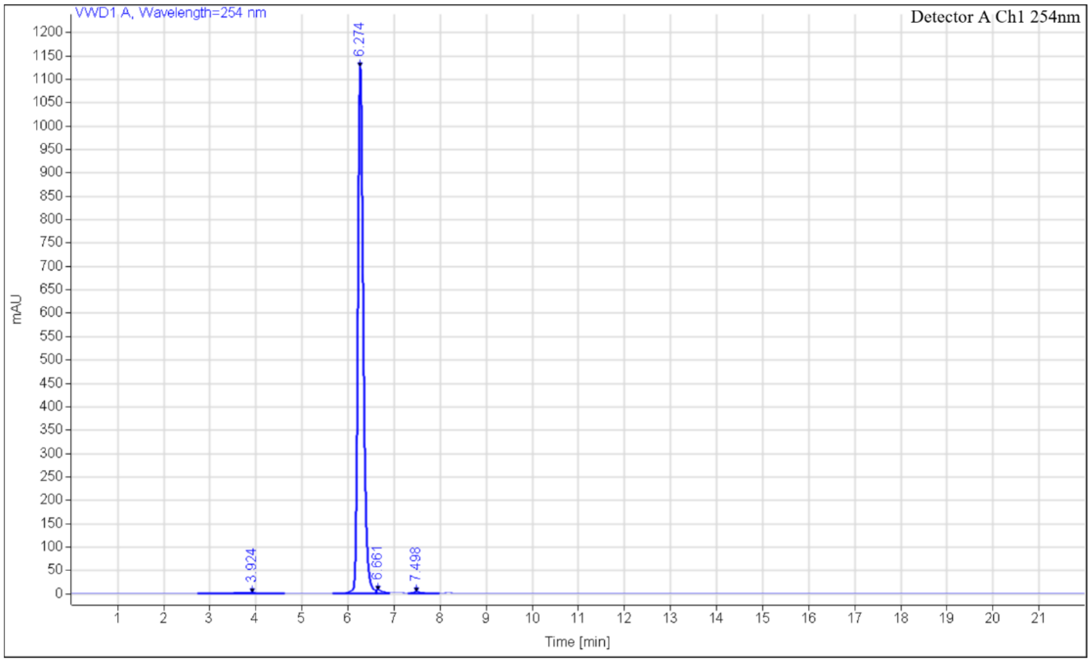

〈Peak table〉

Detector A Ch1 254nm

Number	Retention time	Peak area	Peak area%	Separation efficiency
1	3. 924	76. 80632	0. 81	--
2	6. 274	9289. 99512	98. 15	6. 46062
3	6. 661	65. 35340	0. 69	0. 77686
4	7. 498	32. 55568	0. 34	1. 61107
Total		9464. 71052	100. 00	

(USP)

(4-(4-hydroxybenzoyl)piperazin-1-yl)(2-methylquinolin-4-yl)methanone **(AI10-a30)**

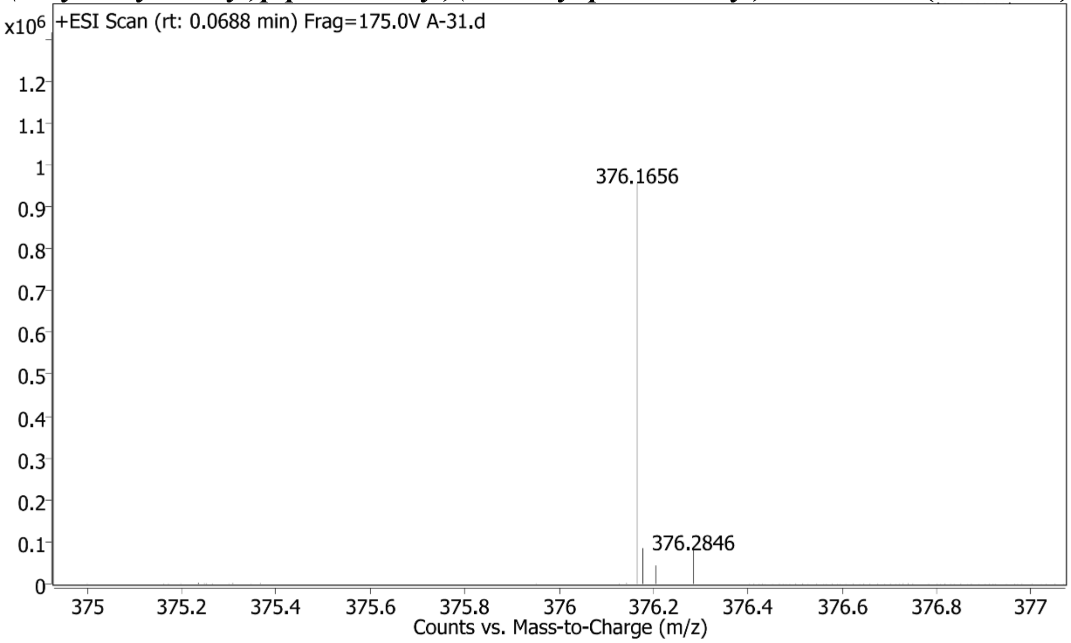

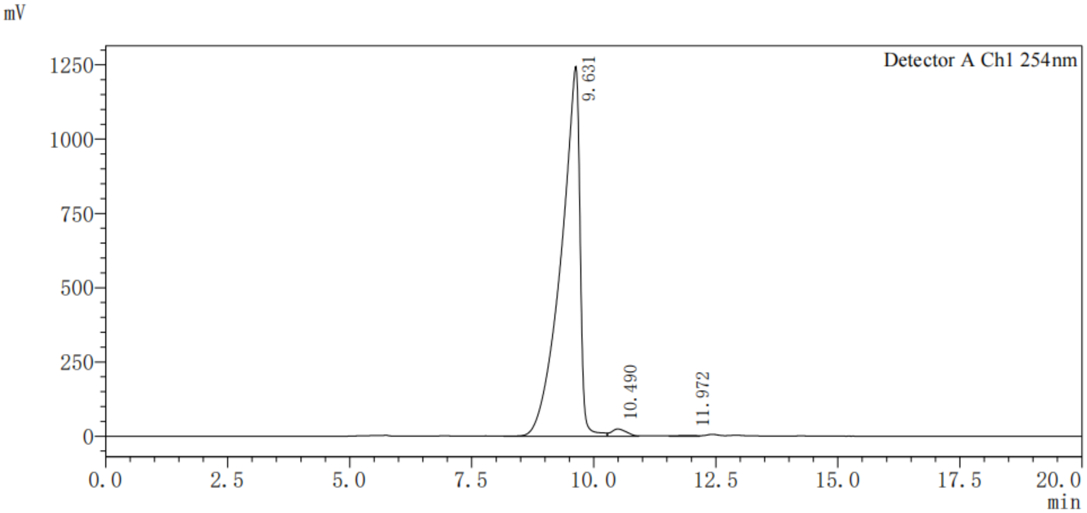

<Peak table>

Detector A Ch1 254nm

Number	Retention time	Peak area	Peak area%	Separation efficiency
1	9.631	33597882	98.242	--
2	10.490	526492	1.539	1.318
3	11.972	74650	0.218	1.795
Total		34199023	100.000	

(USP)

(4-(4-hydroxybenzoyl)piperazin-1-yl)(m-tolyl)methanone **(AI10-a31)**

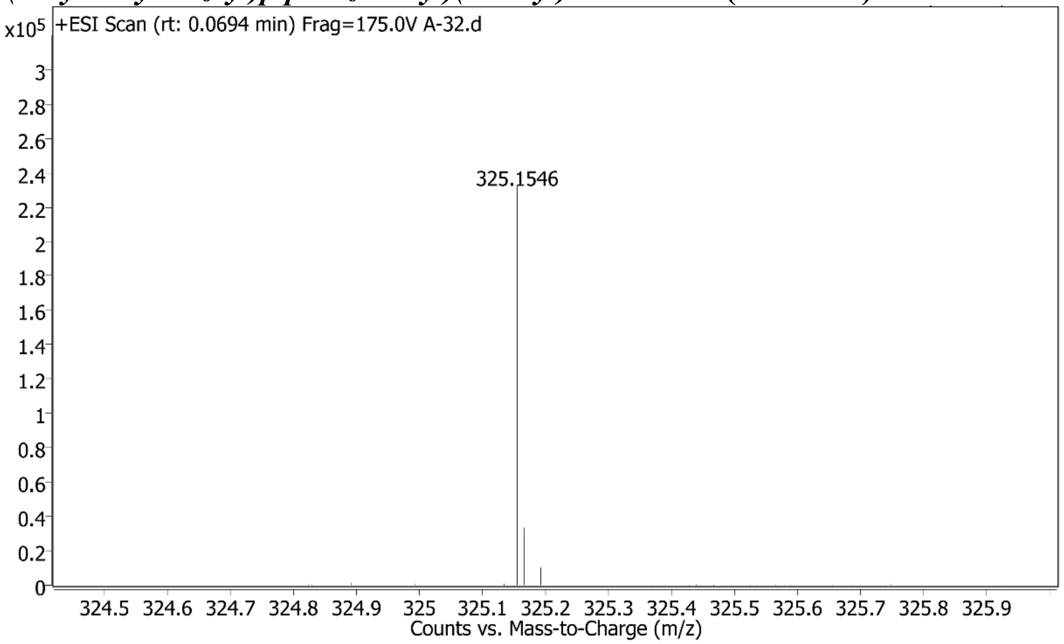

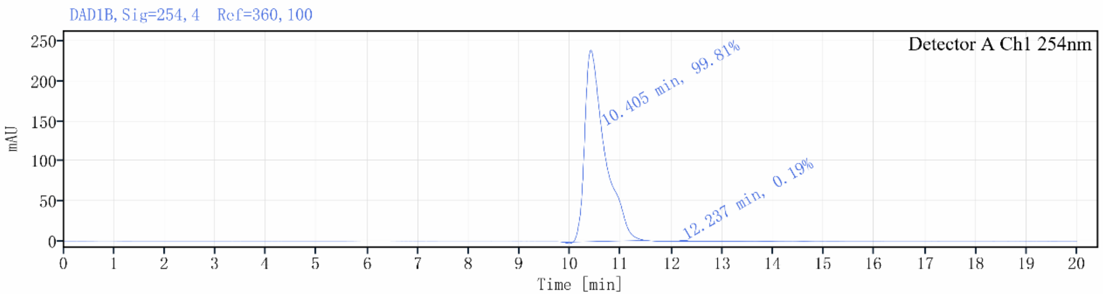

⟨Peak table⟩

Detector A Ch1 254nm

Number	Retention time	Peak area	Peak area%	Separation efficiency
1	10. 405	6633. 4	99. 81	--
2	12. 237	12. 7	0. 19	--
Total		6646. 1	100. 00	

(USP)

1-(4-(4-hydroxybenzoyl)piperazin-1-yl)-3-(m-tolyl)propan-1-one **(AI10-a32)**

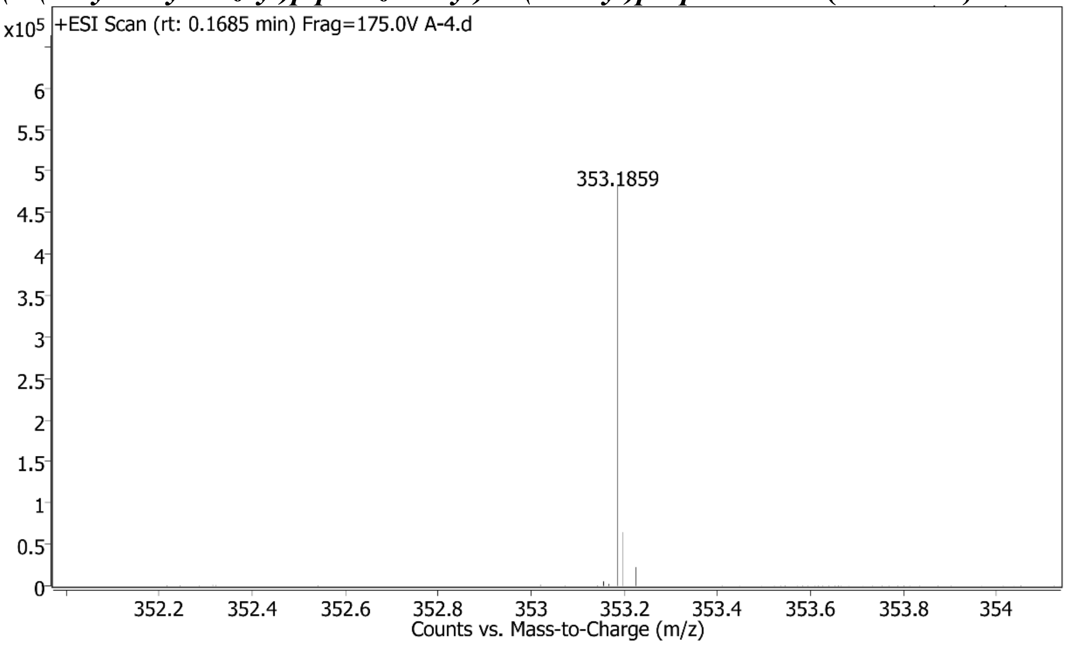

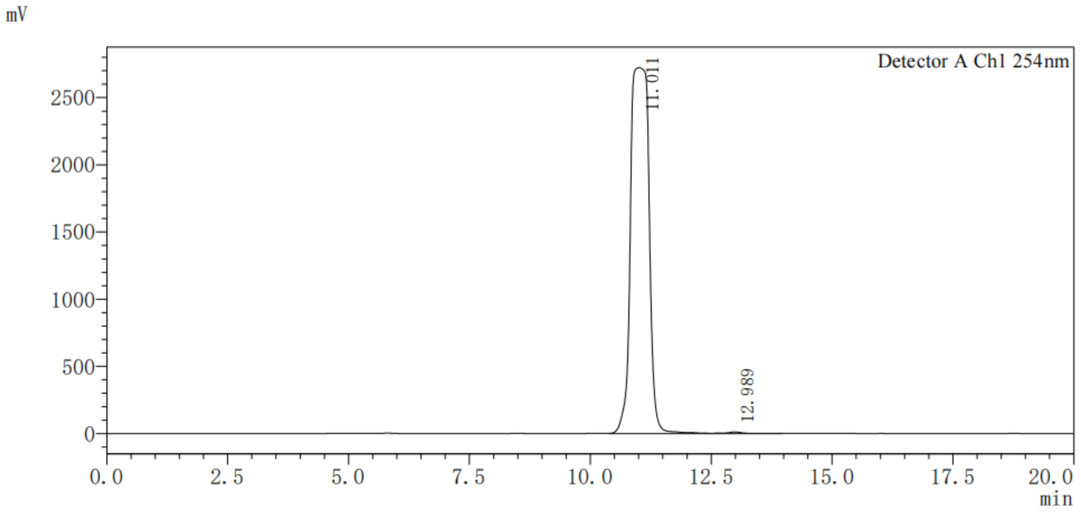

<Peak table>

Detector A Ch1 254nm

Number	Retention time	Peak area	Peak area%	Separation efficiency
1	11.011	72843009	99.610	--
2	12.989	285024	0.390	3.915
Total		73128034	100.000	

(USP)

Structures of compounds generated from seventeen AI driven de novo molecular generation tasks

Task1:

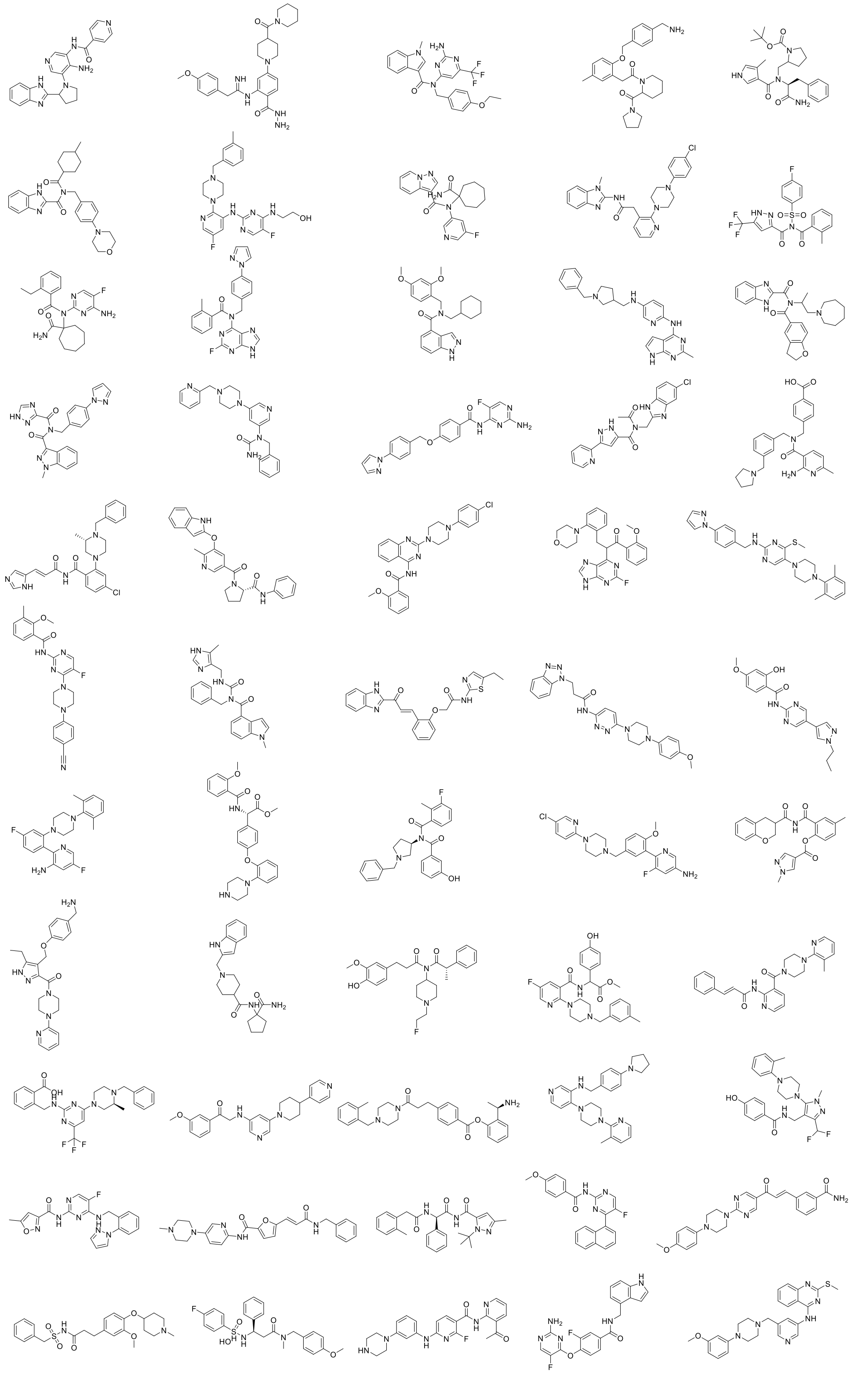

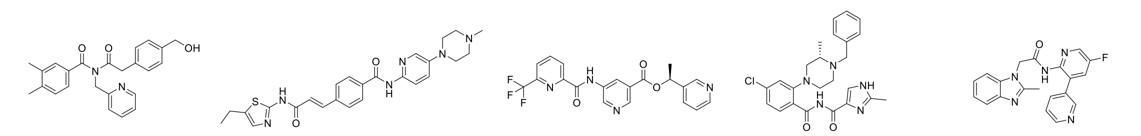
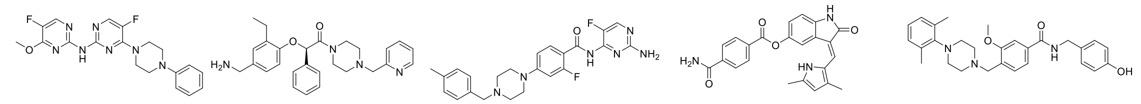
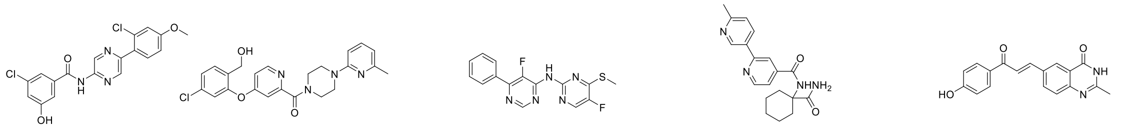
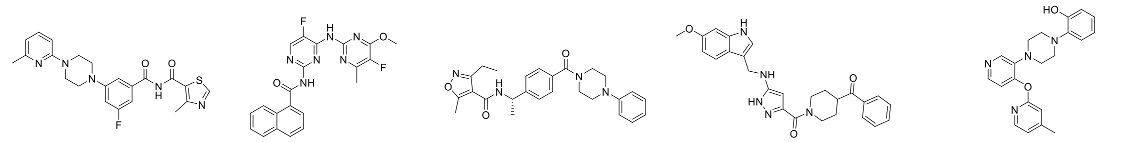
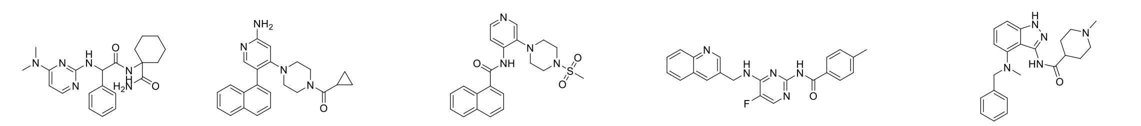
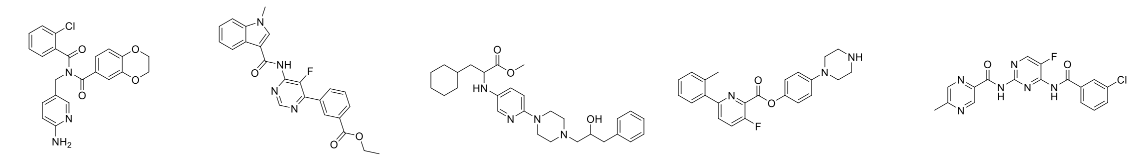
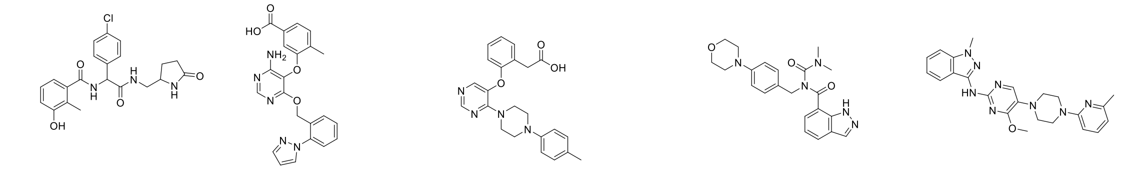
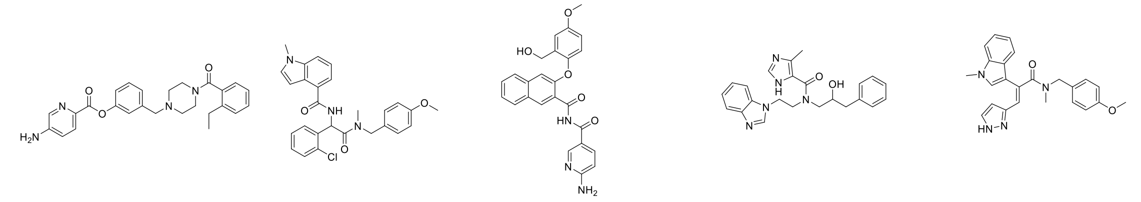
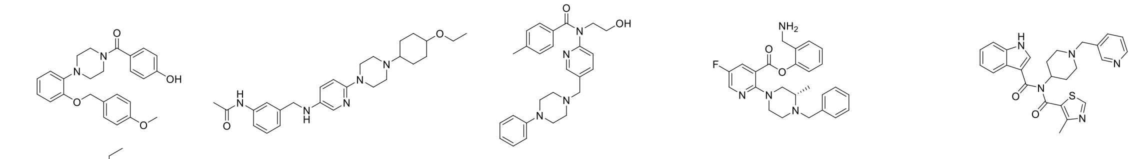
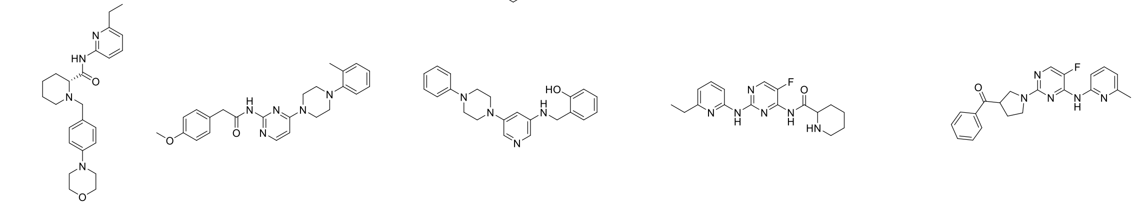
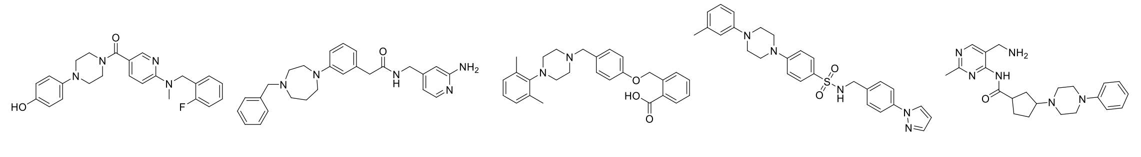

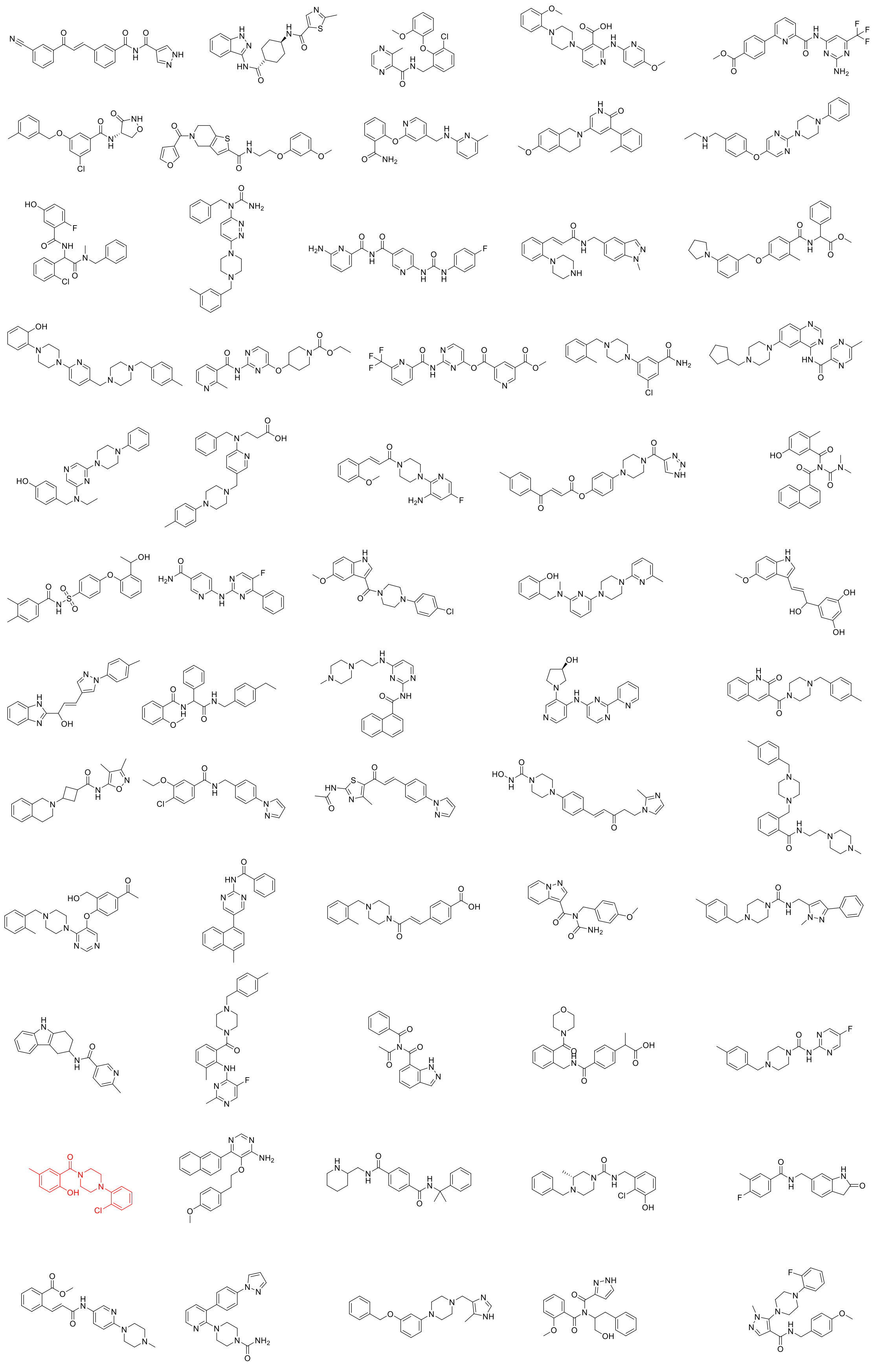

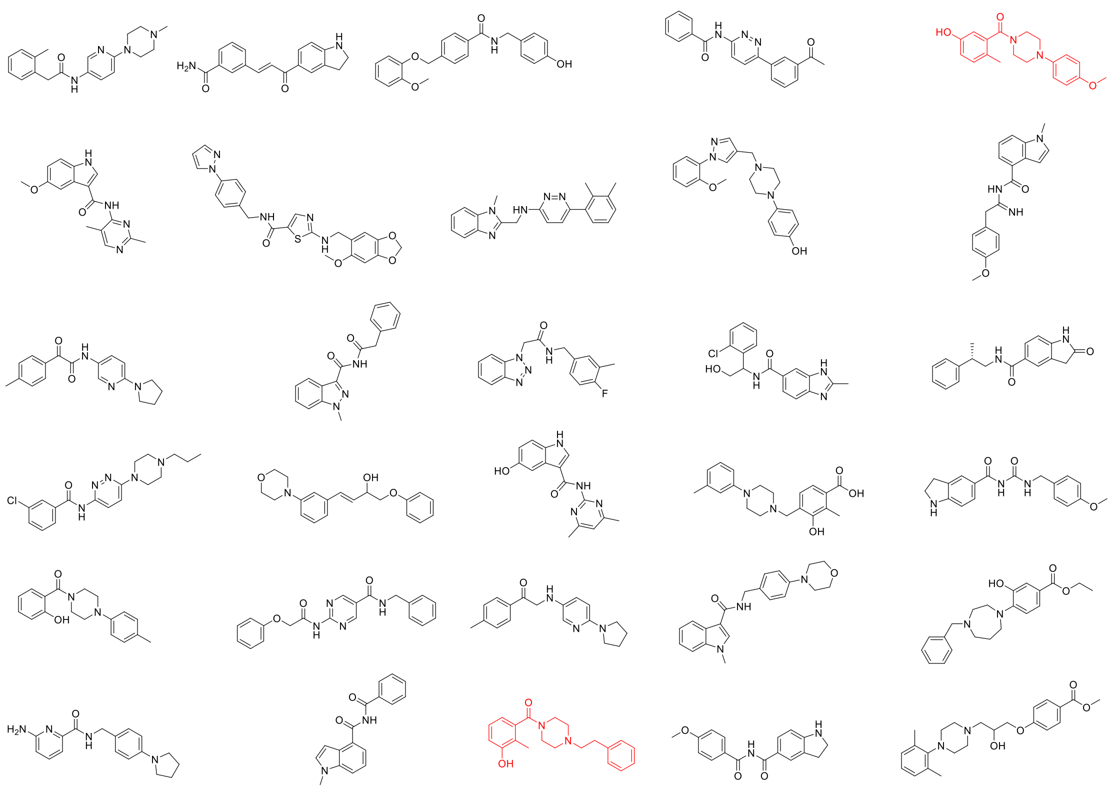

Task 2:

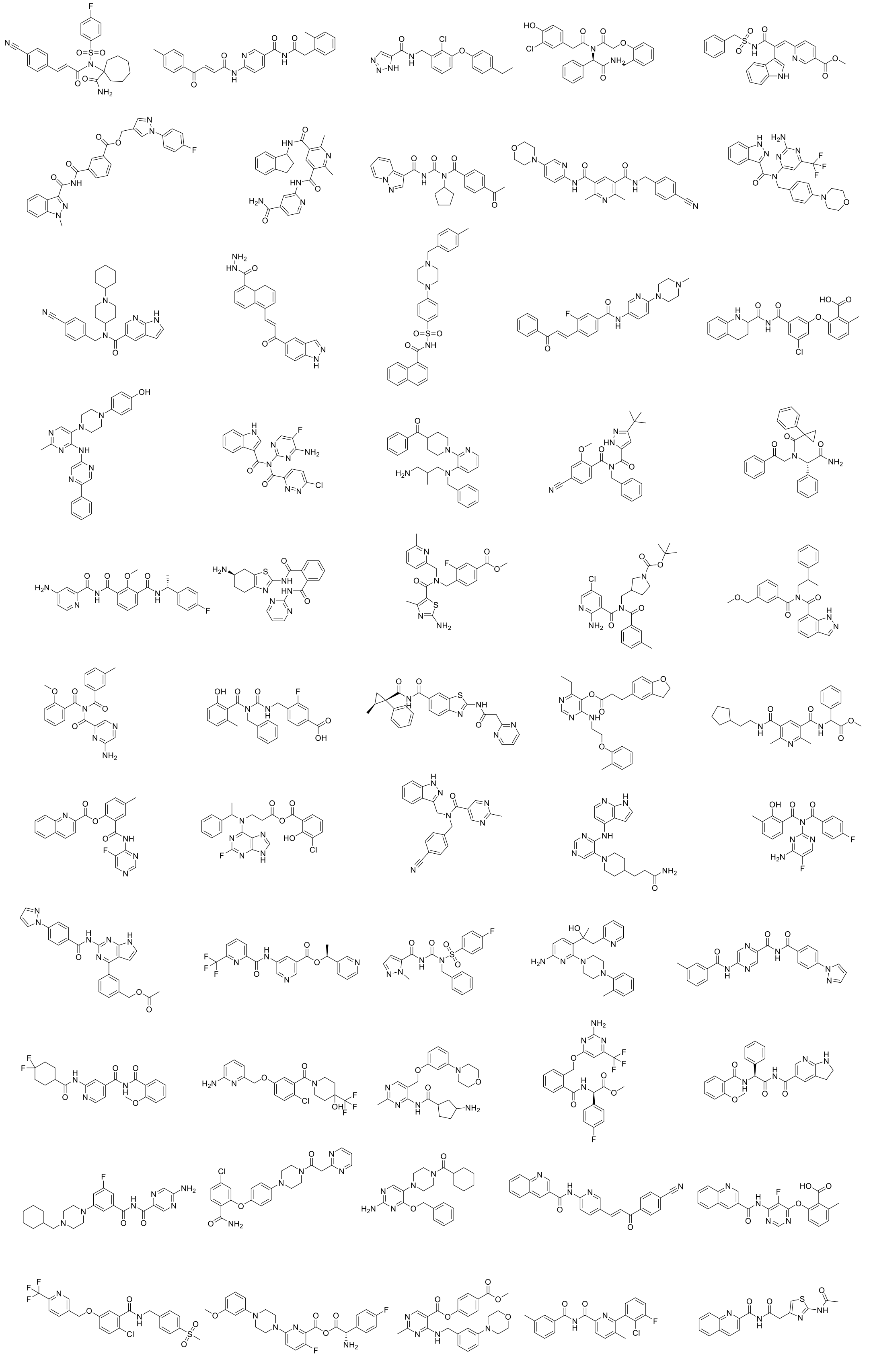

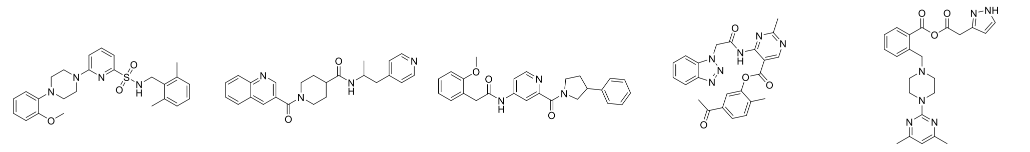
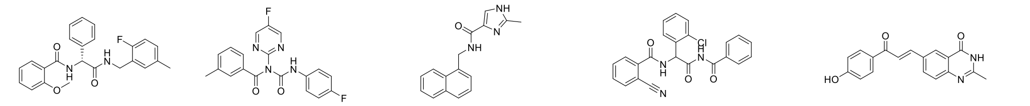
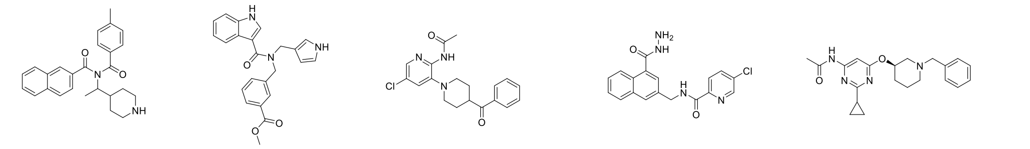
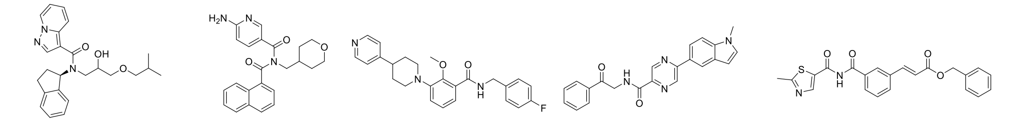
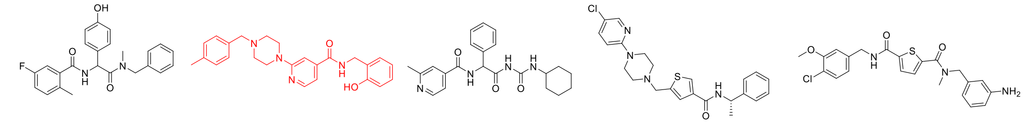
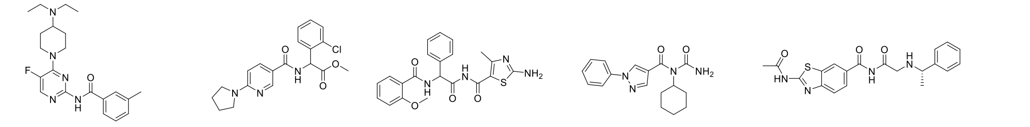
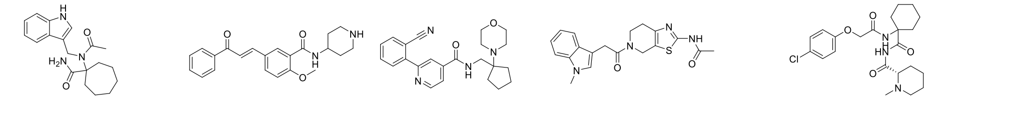
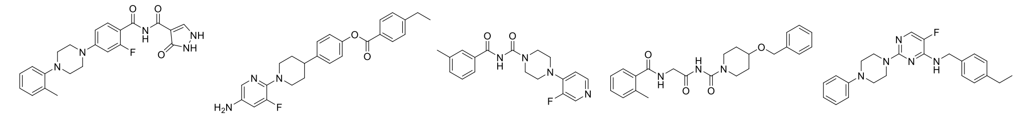
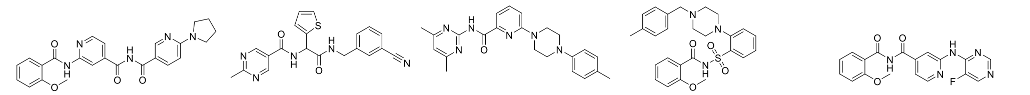
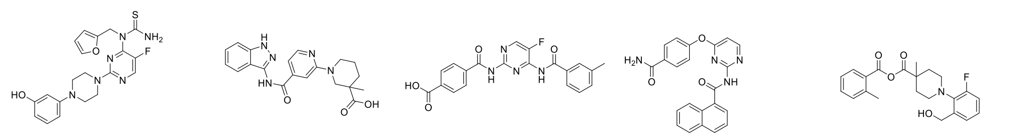
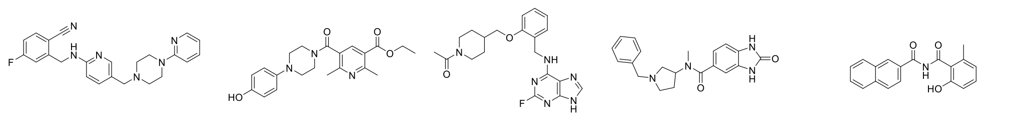
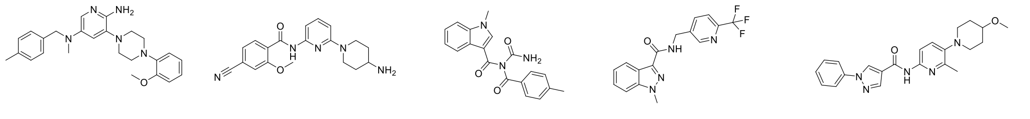

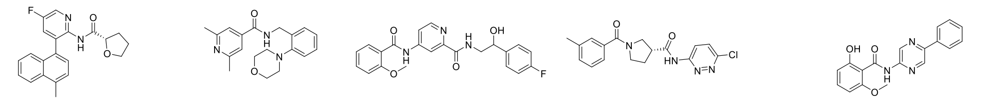

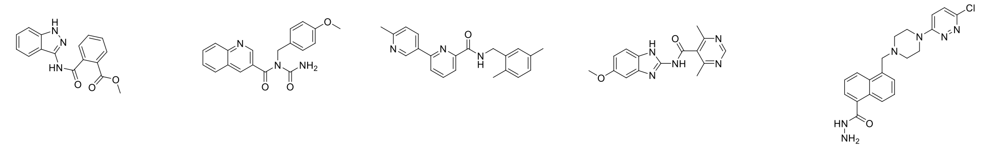

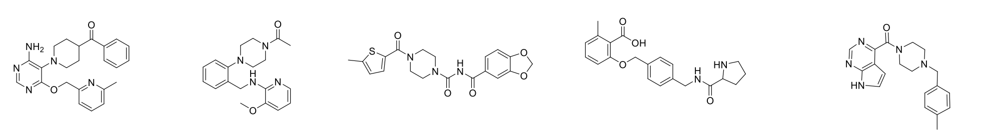

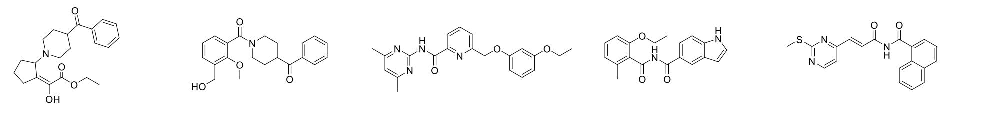

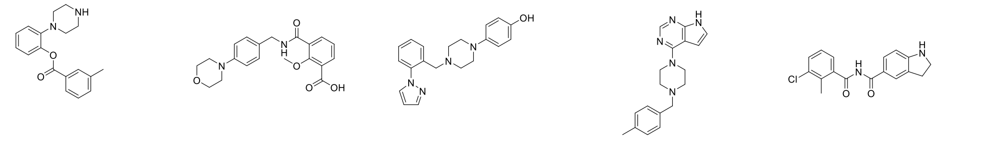

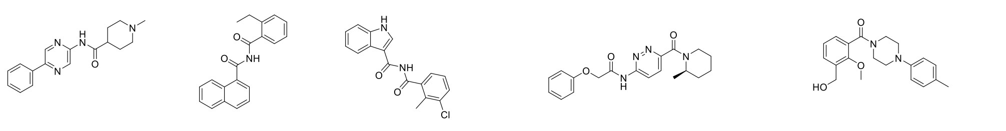

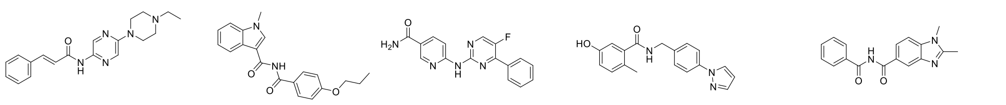

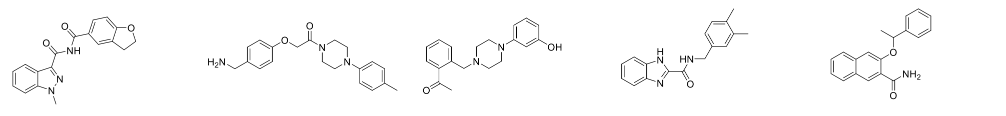

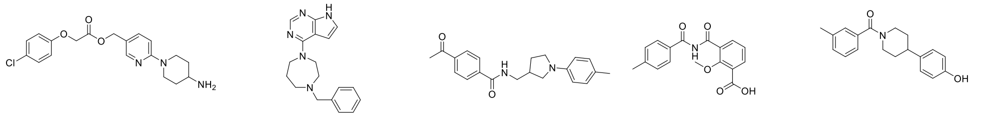

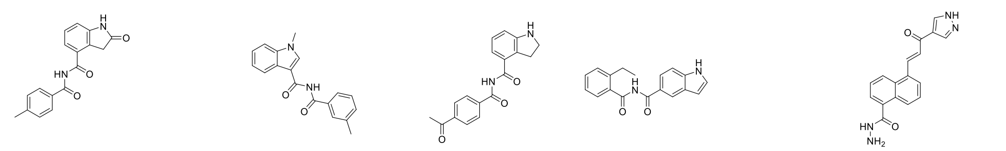

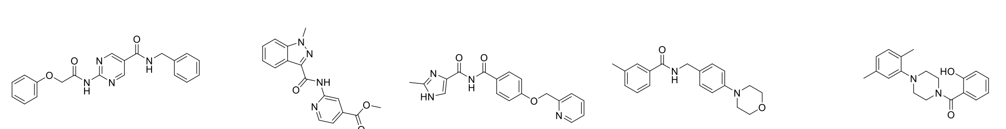

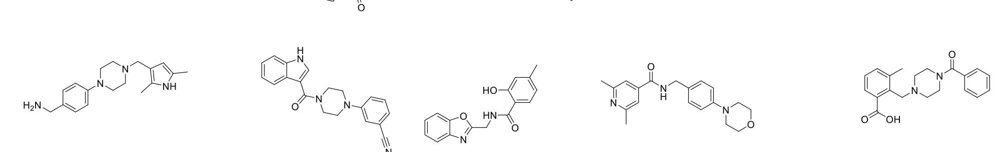

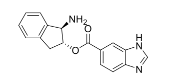

Task 3:

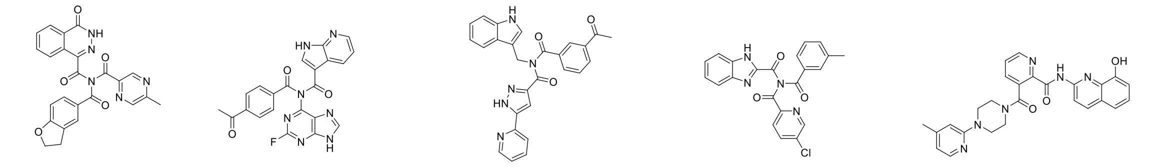

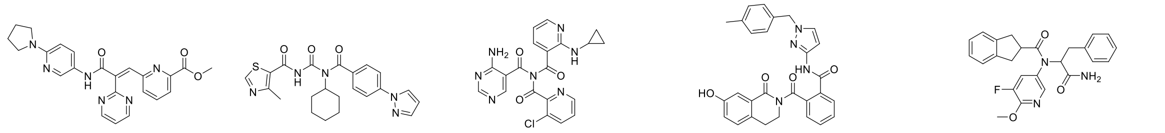

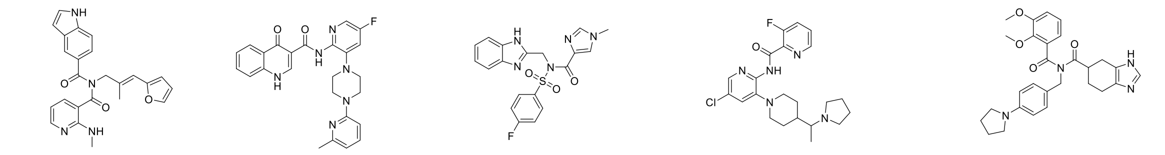

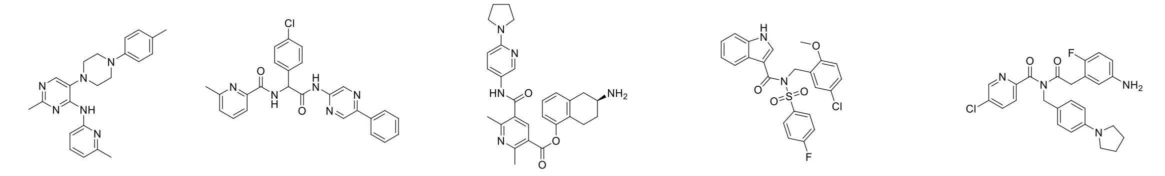

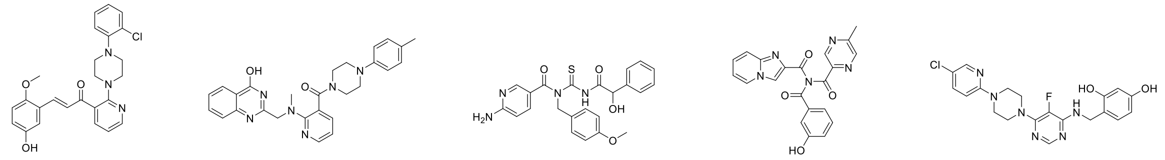

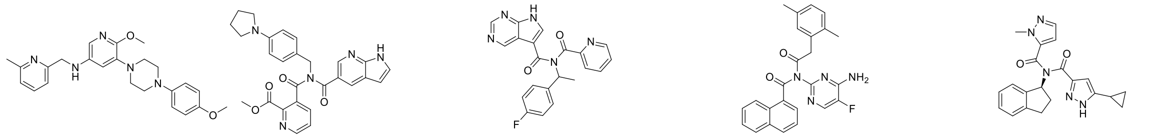

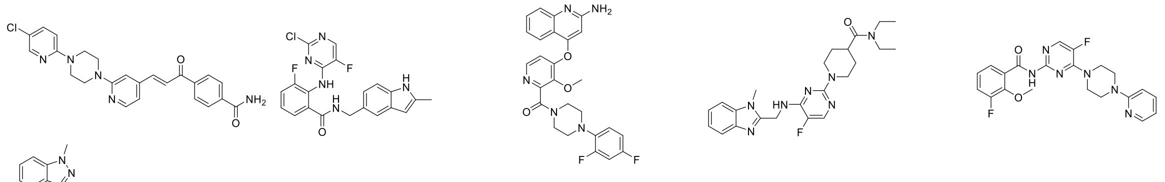

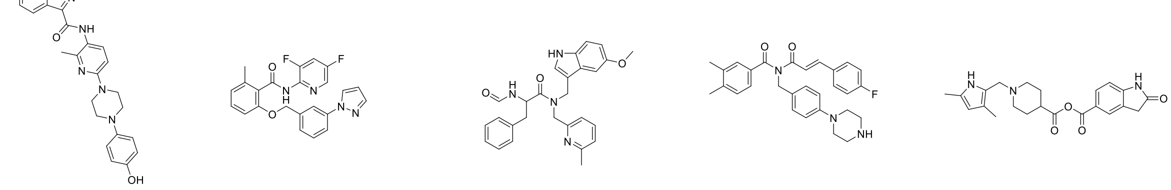

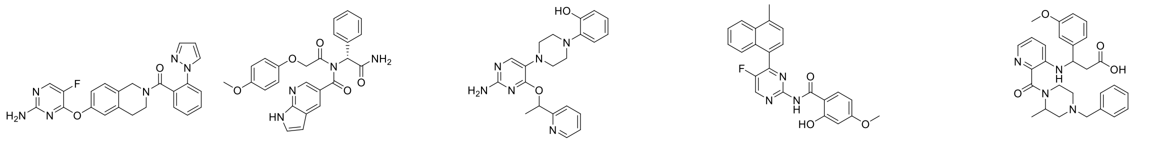

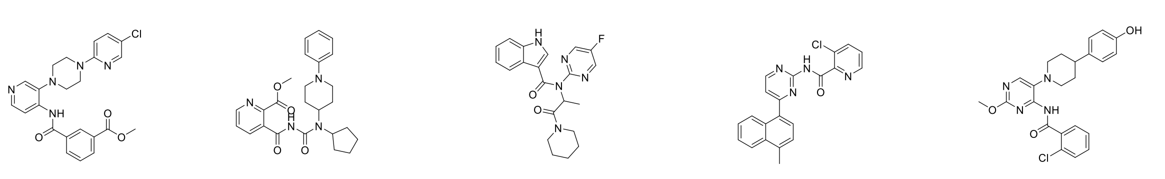

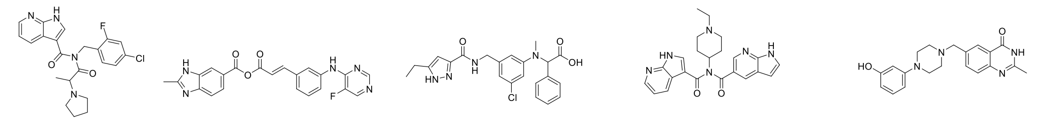

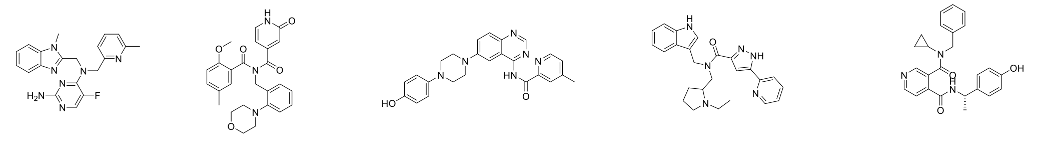

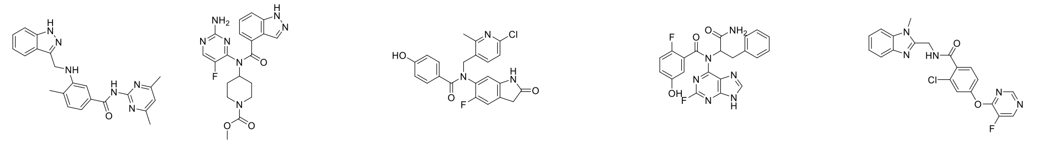

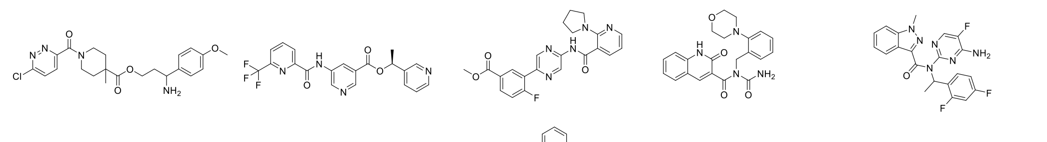

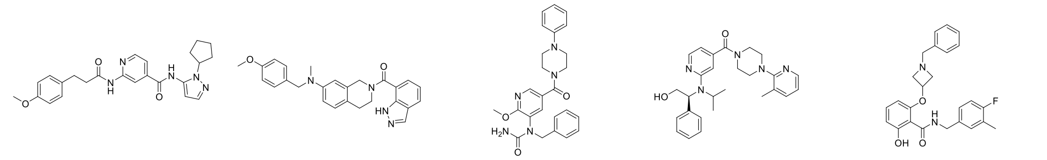

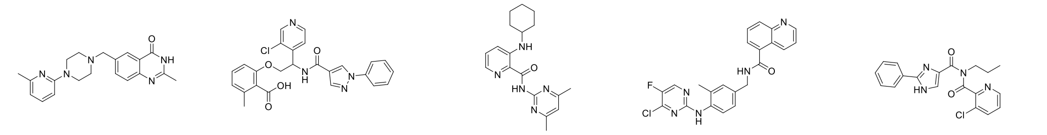

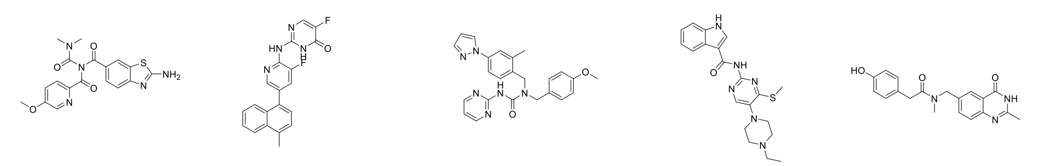

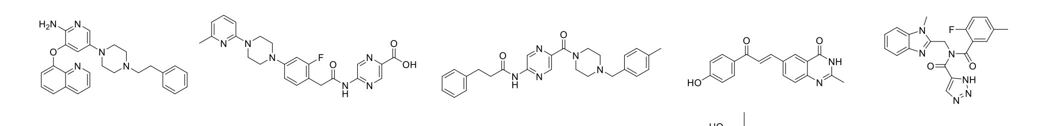

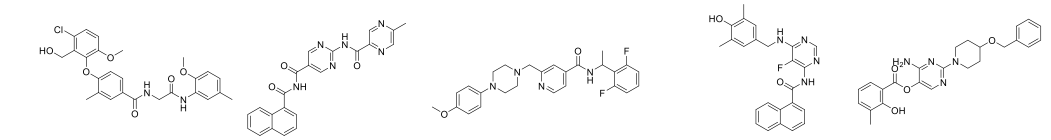

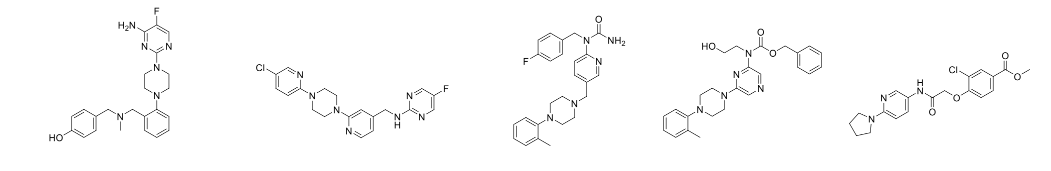

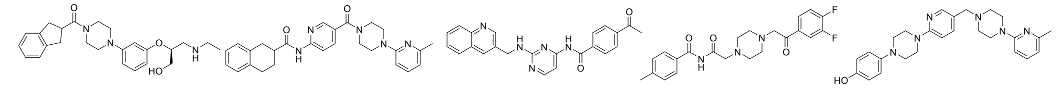

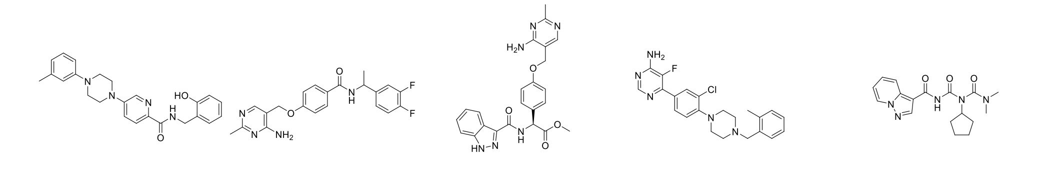

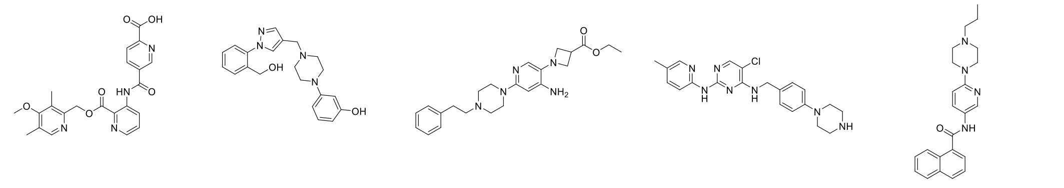
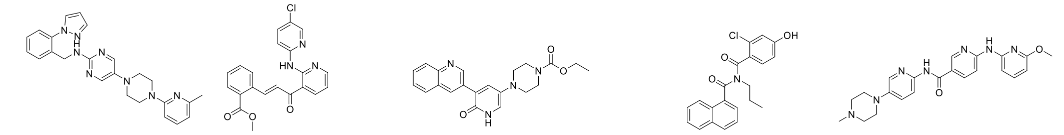
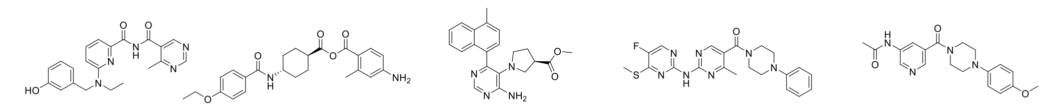
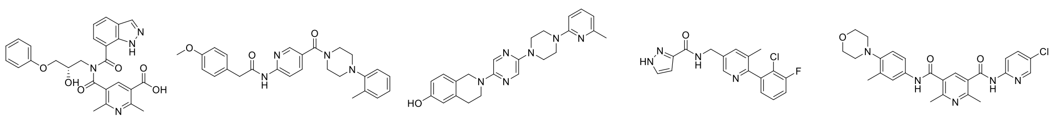
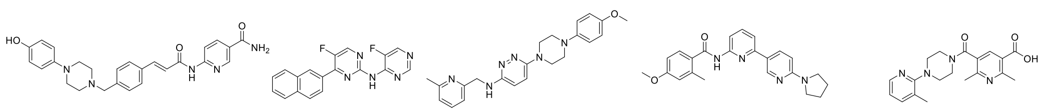
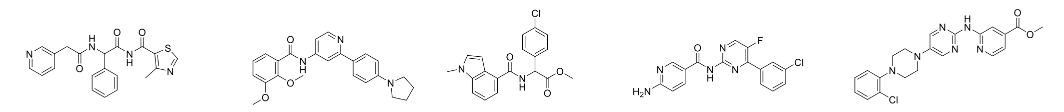
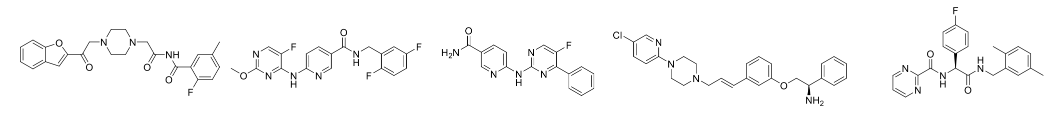
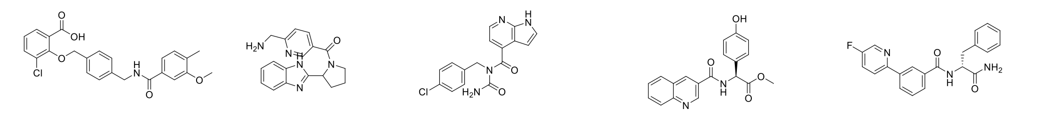
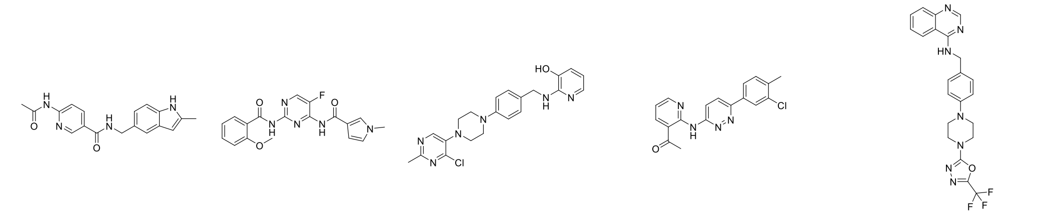
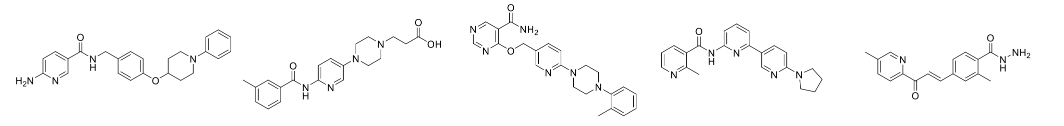
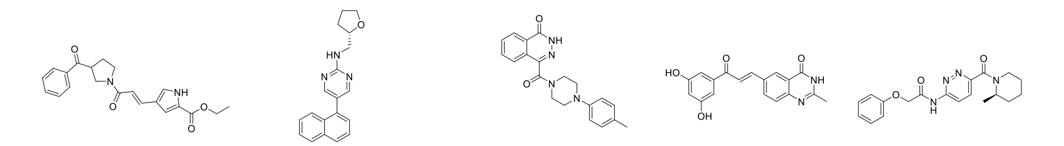
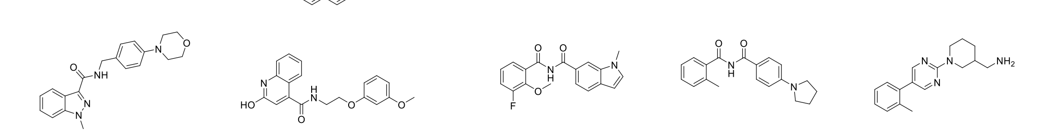

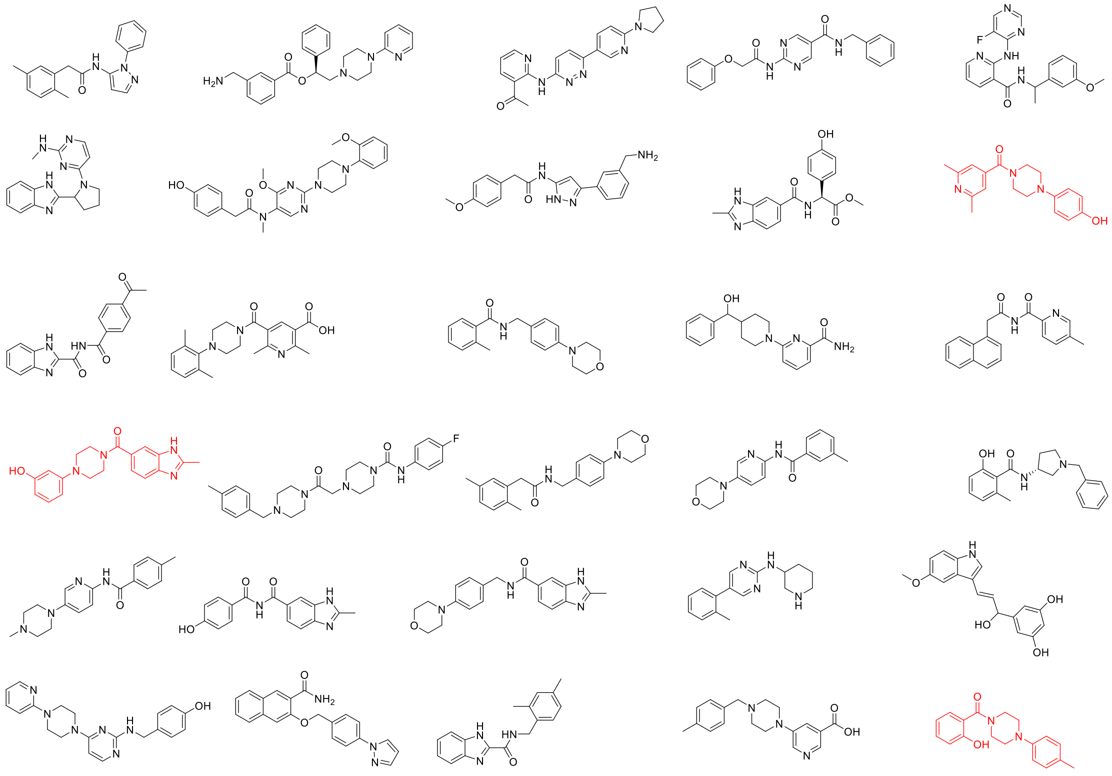
N
H
O
NH2
OH
F
HN
N-N
NH
HO

Task 4:

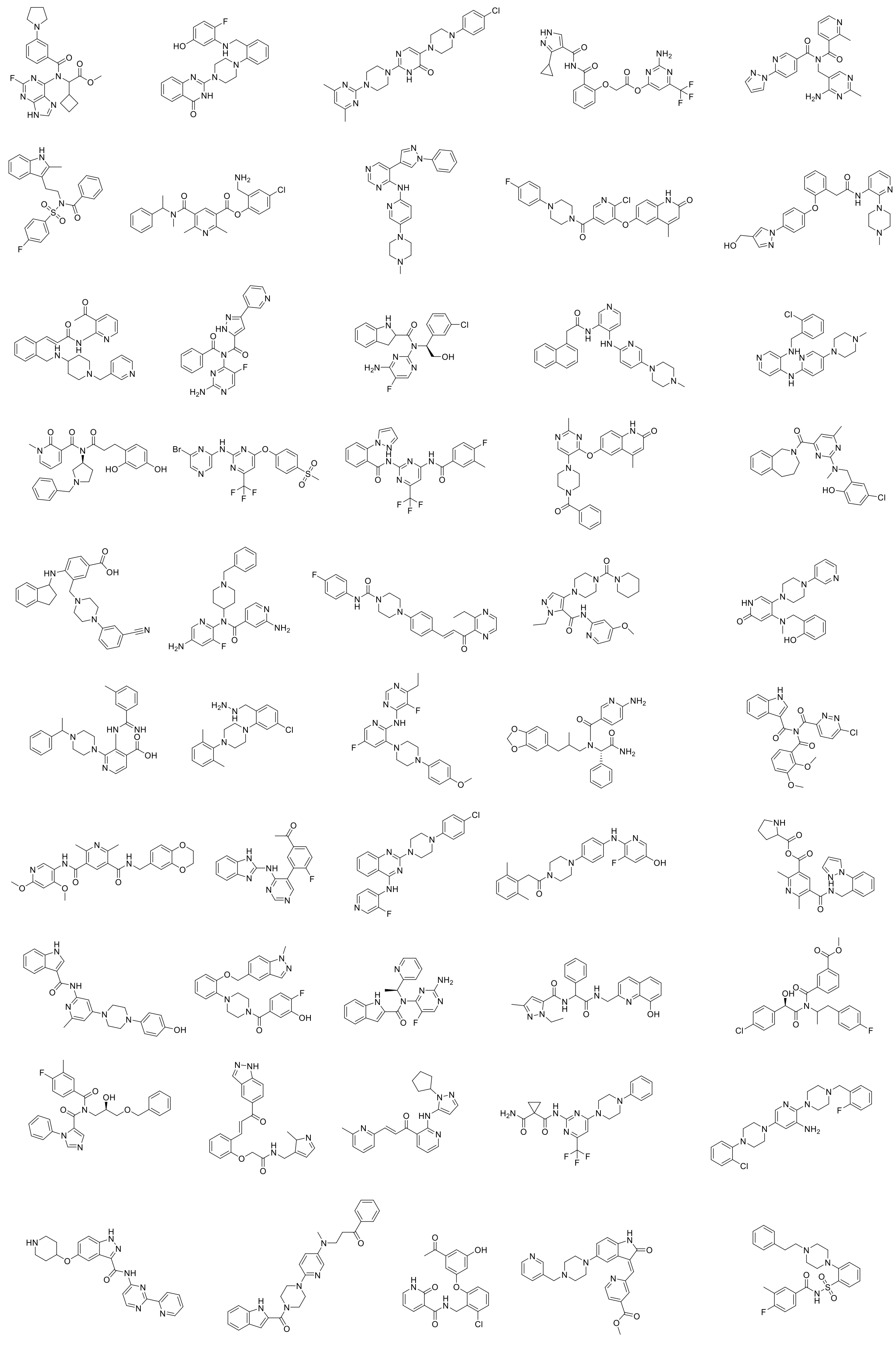

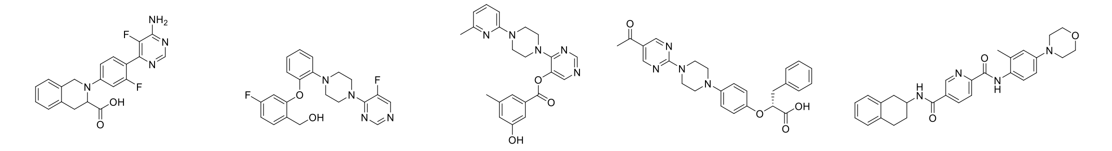
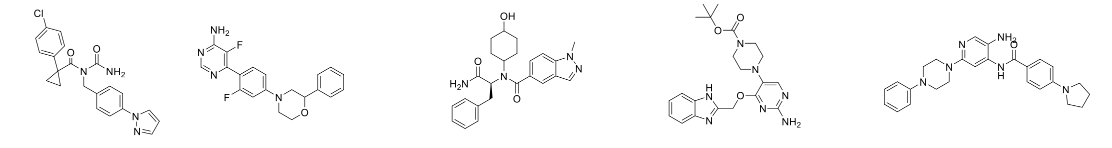
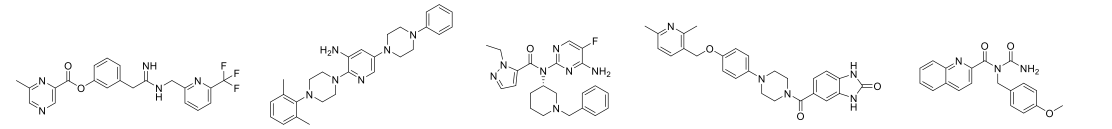
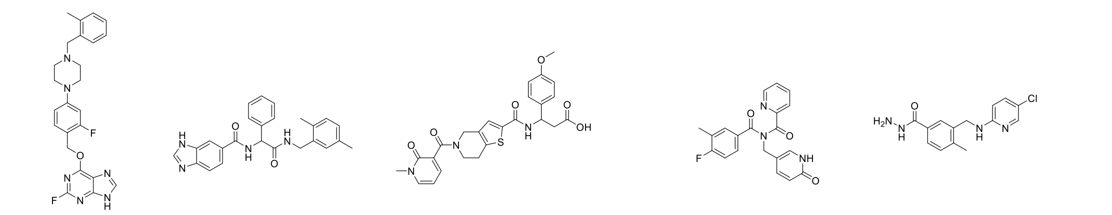
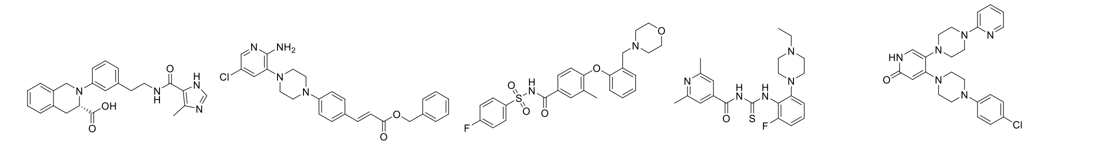
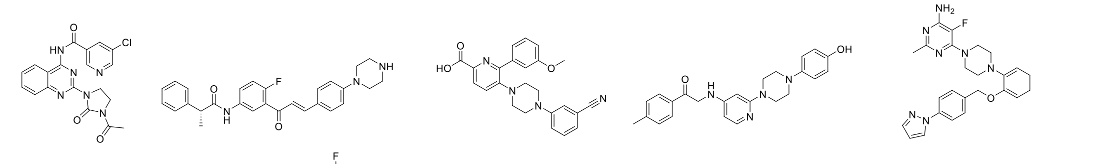
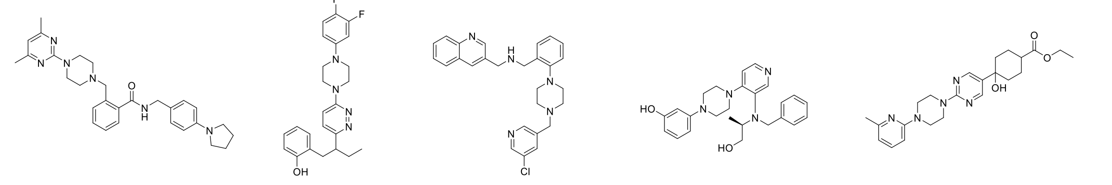
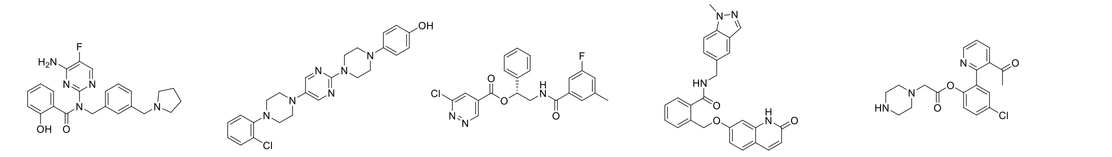
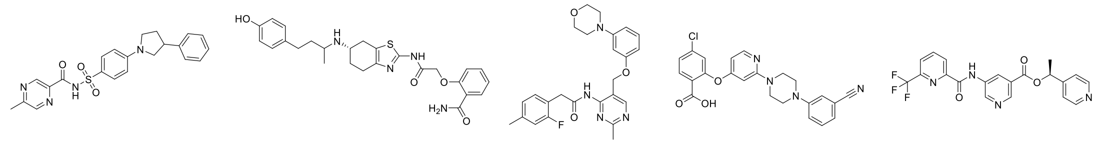
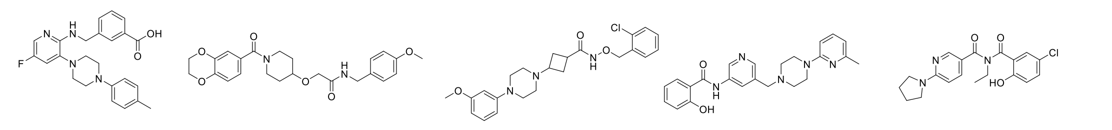
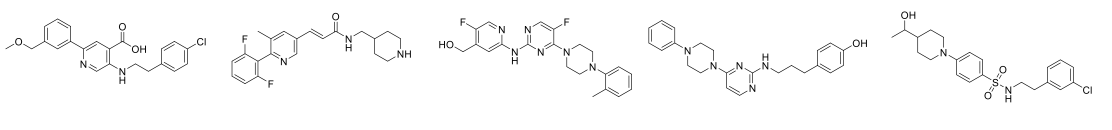

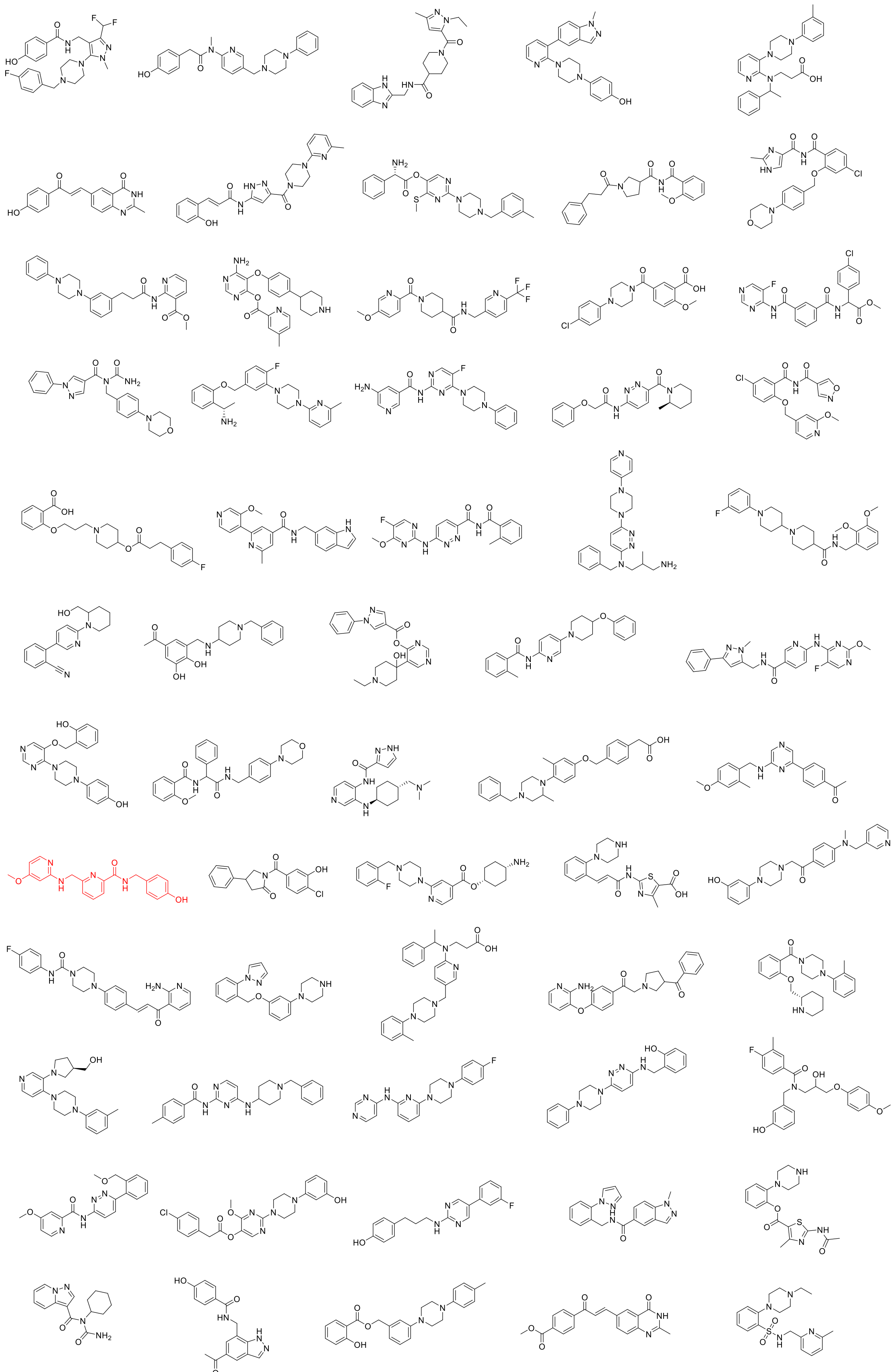

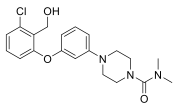
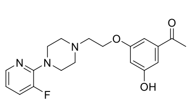
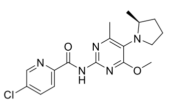
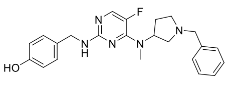
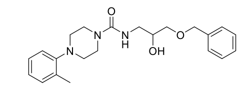
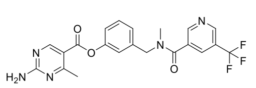
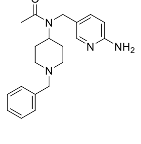
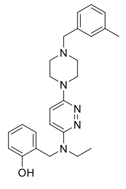
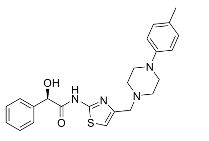
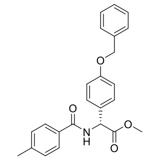
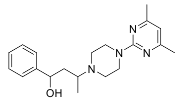
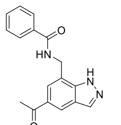
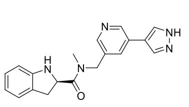
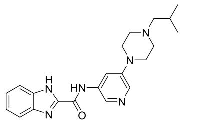
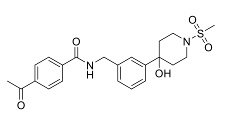
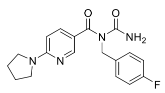
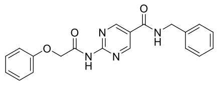
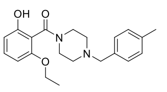
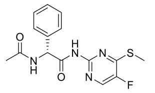
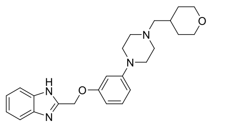
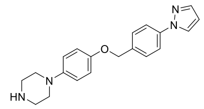
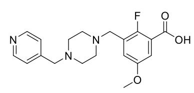
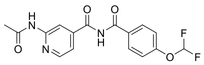
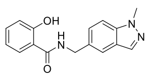
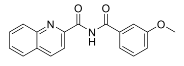
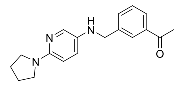
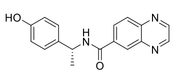
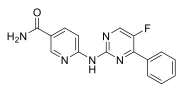
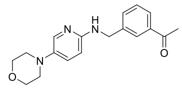
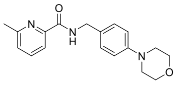
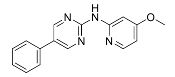
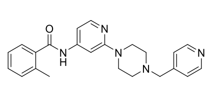
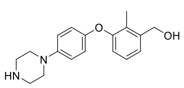
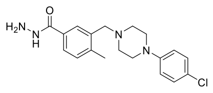
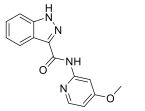

Task 5:

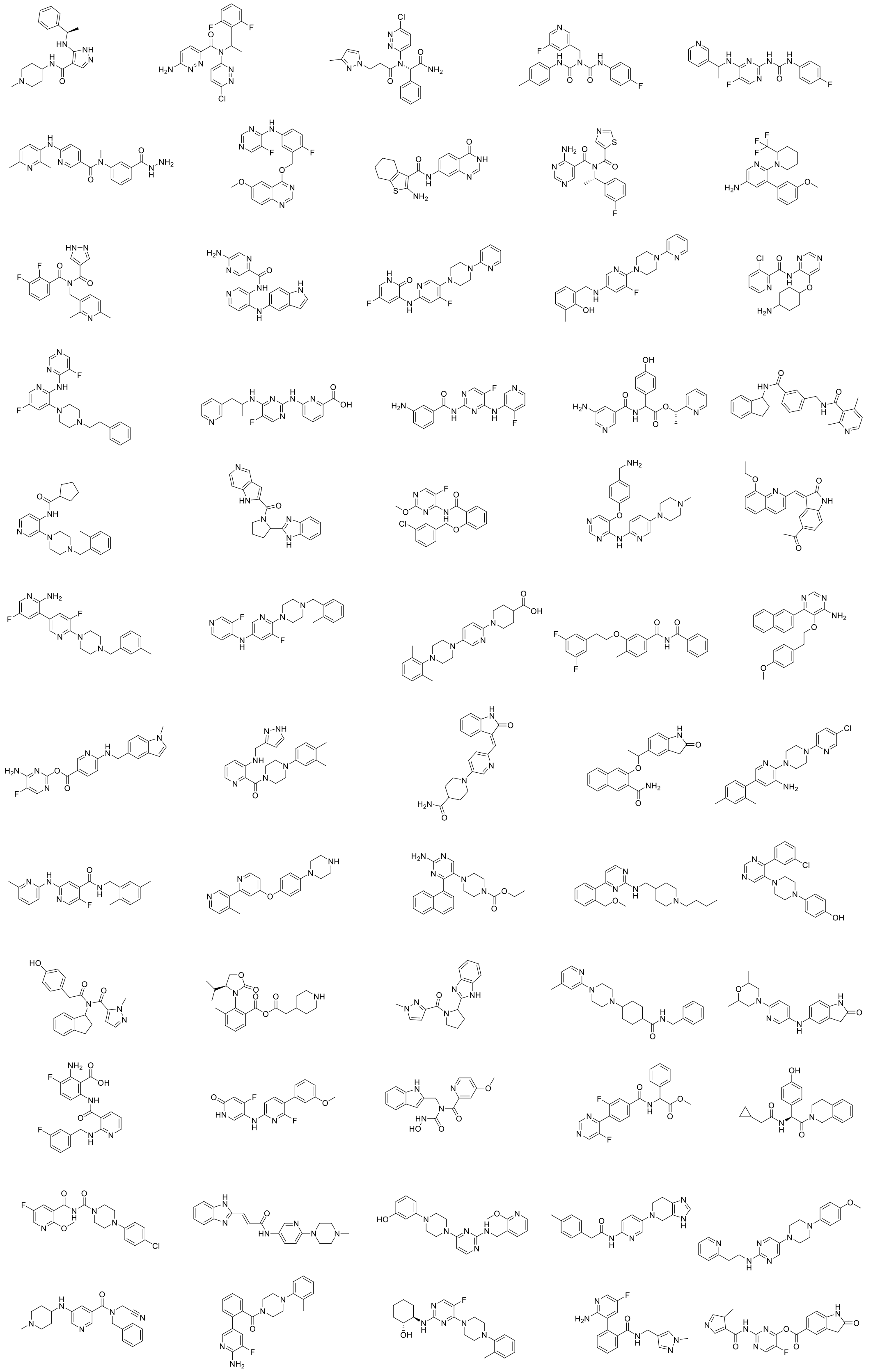

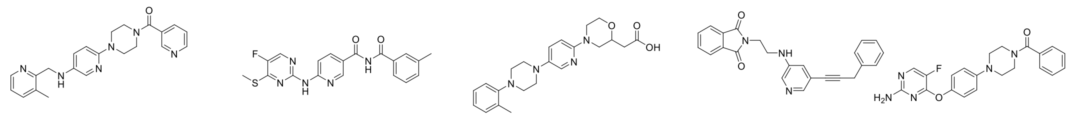
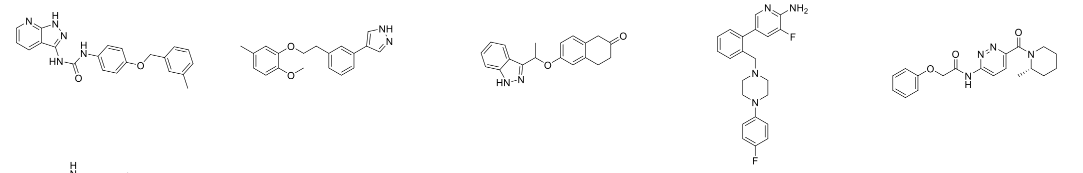
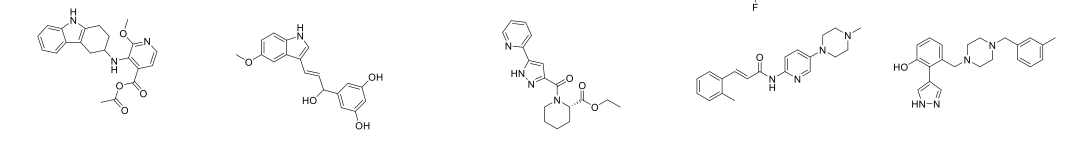
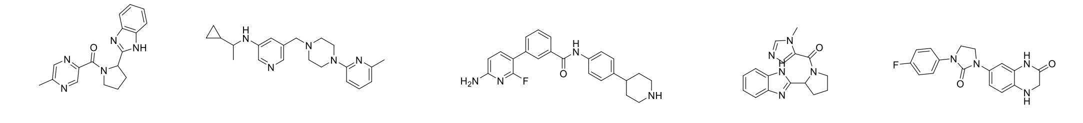
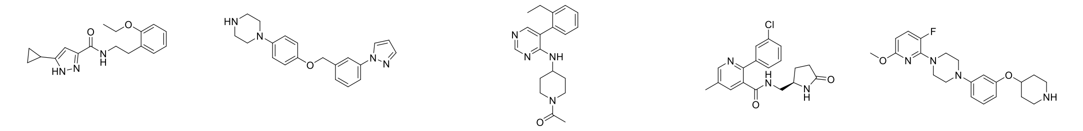
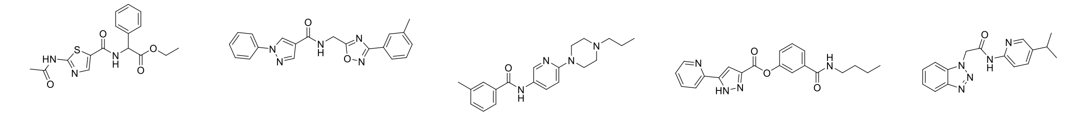
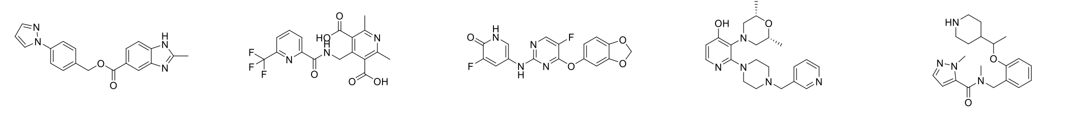
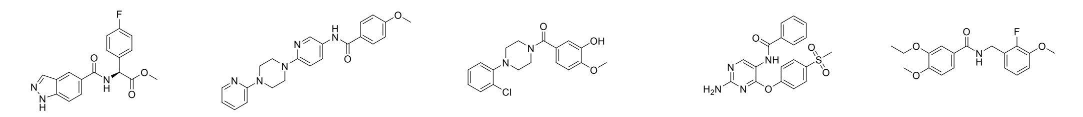
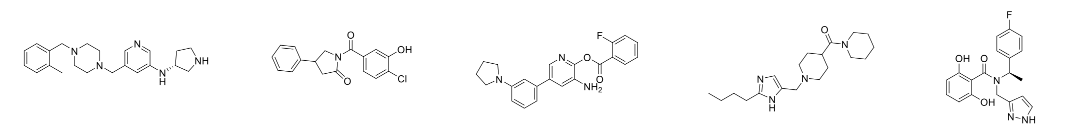
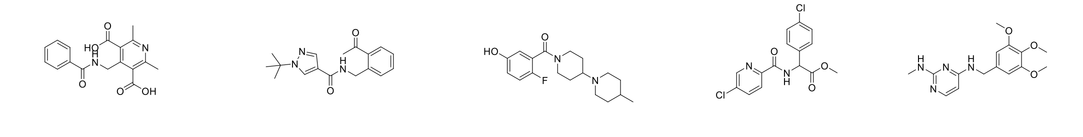
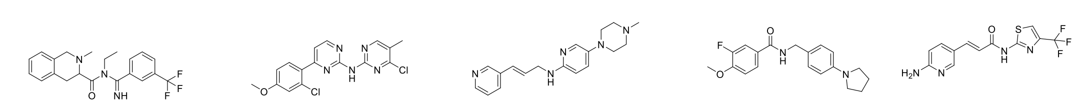
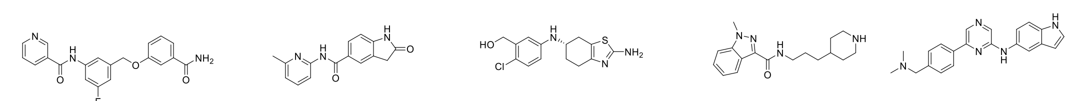

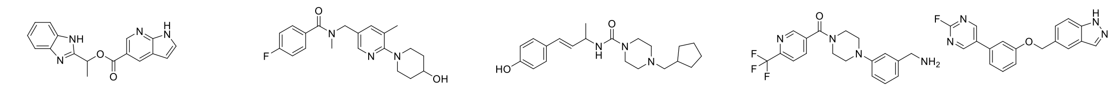
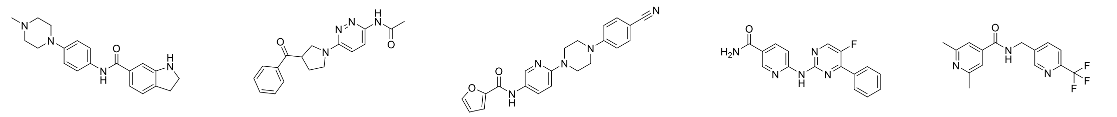
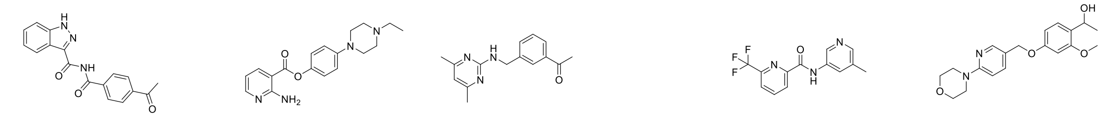
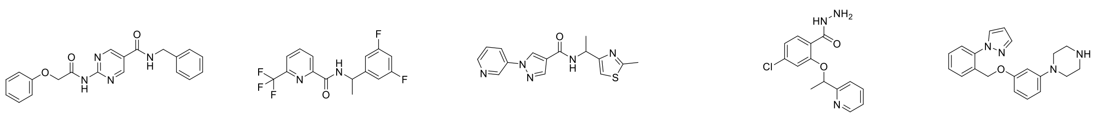
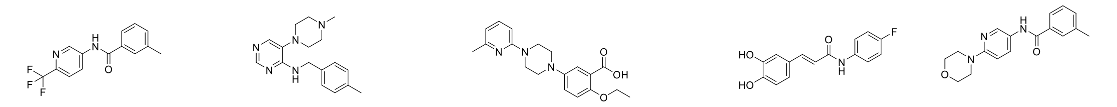
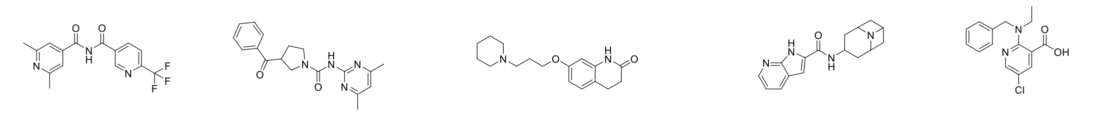
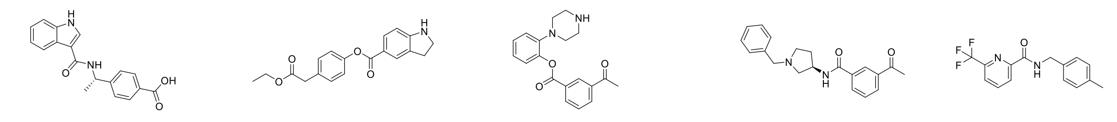
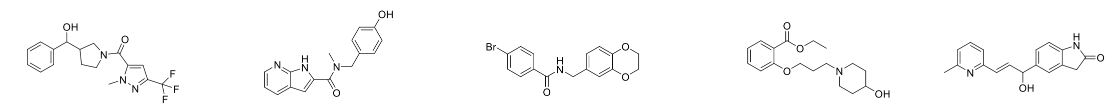
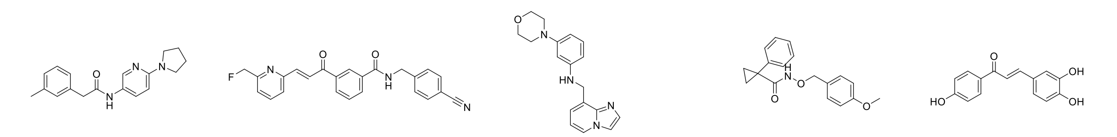
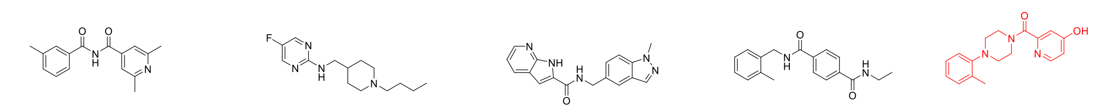
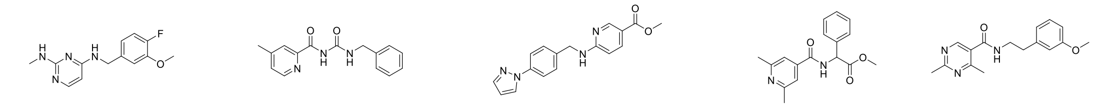
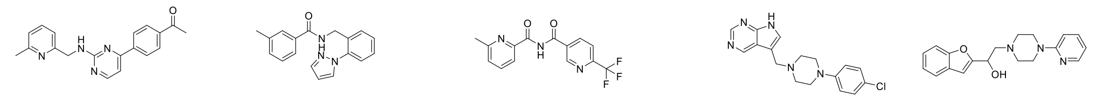

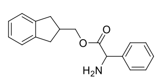
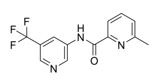
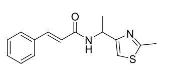
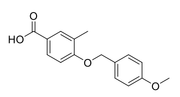
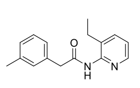
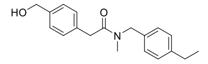
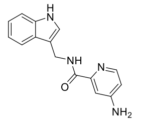
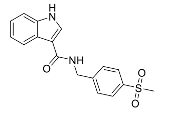
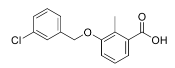
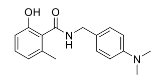
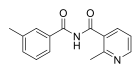
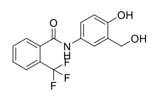
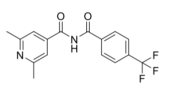
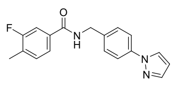
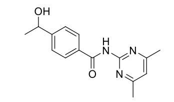
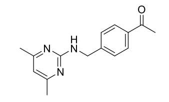
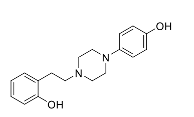
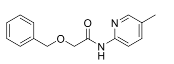
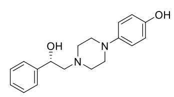
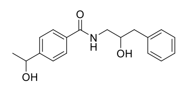

Task 6:

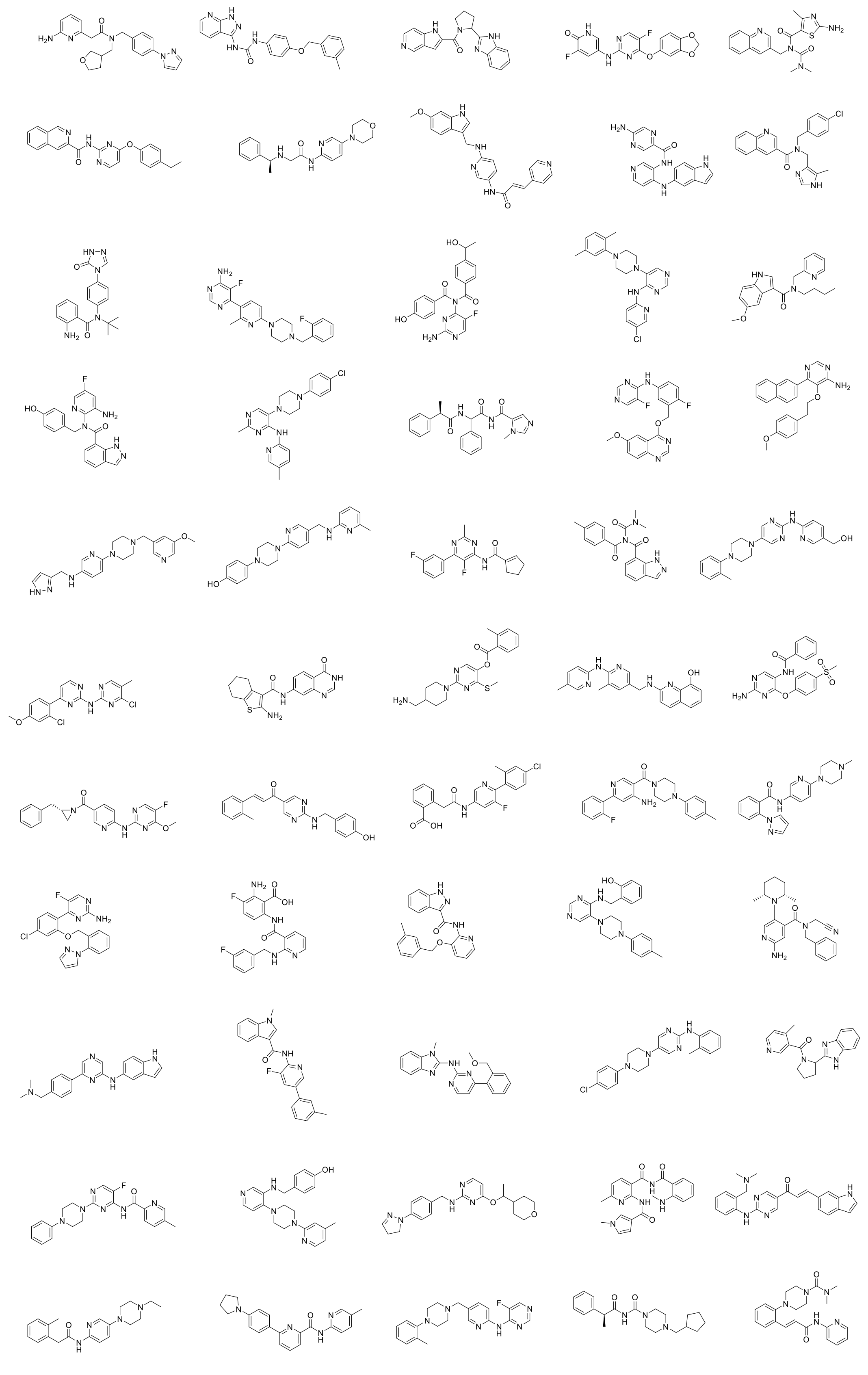

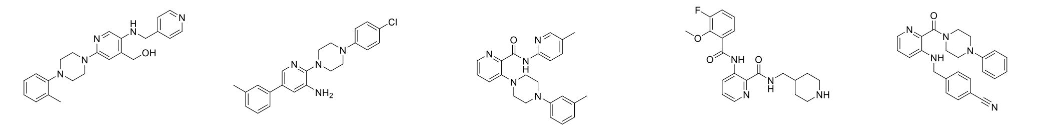
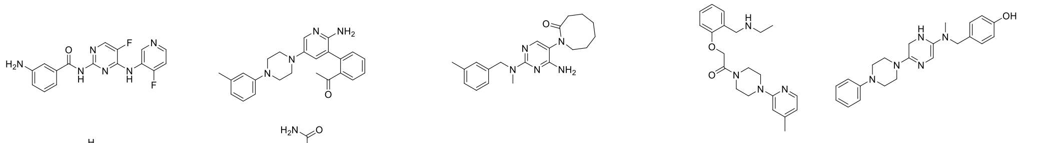
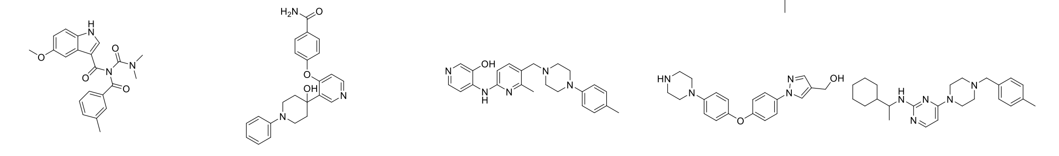
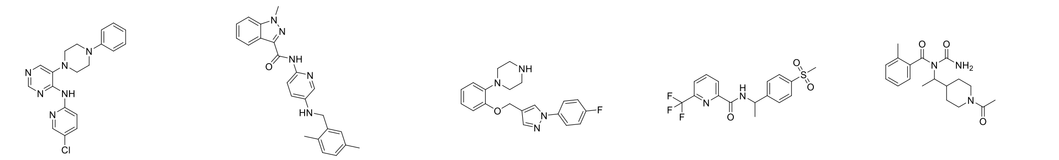
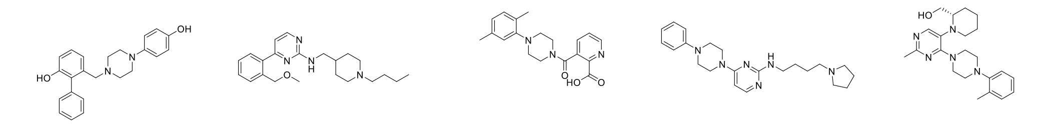
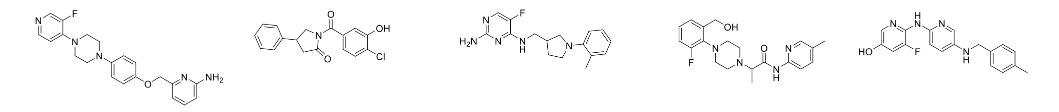
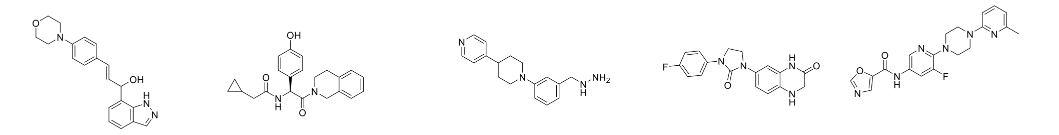
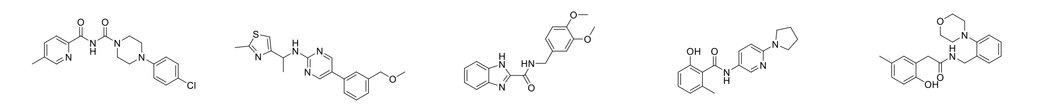
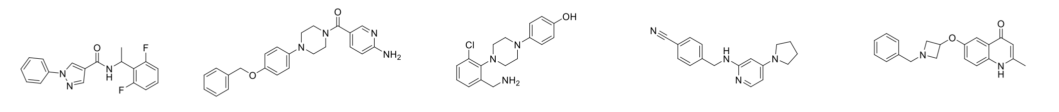
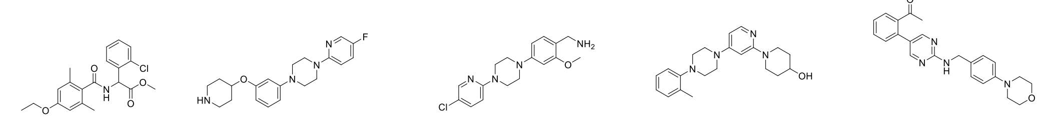
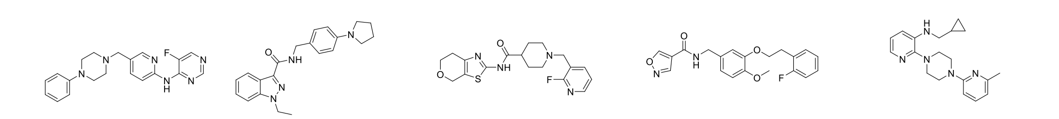
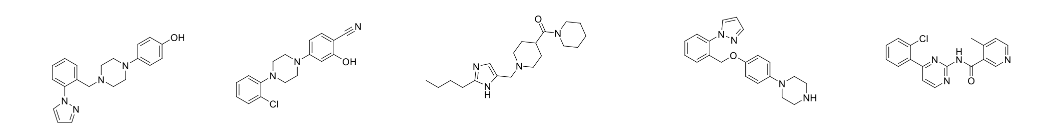

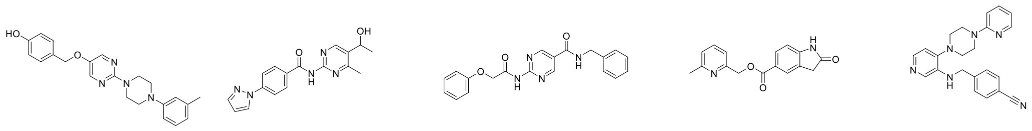
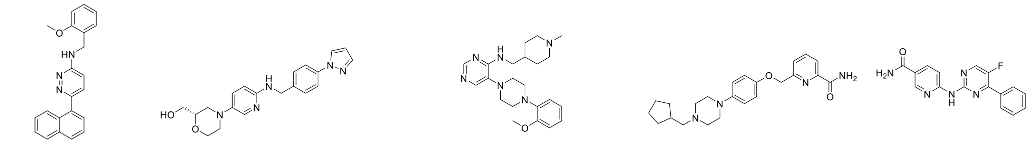
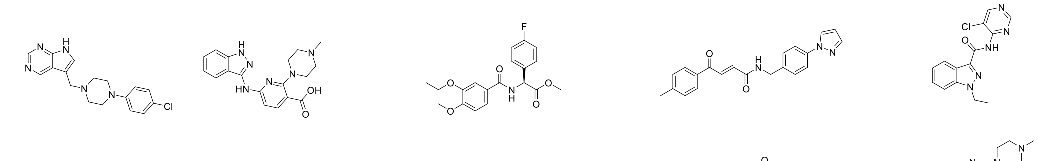
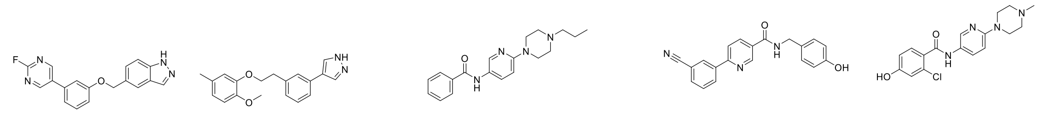
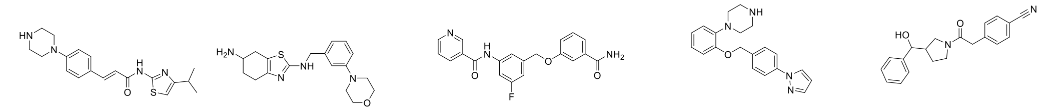
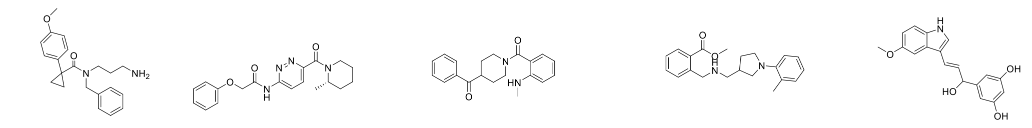
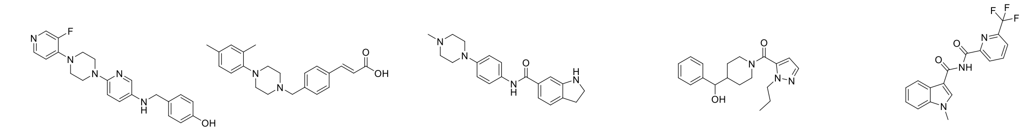
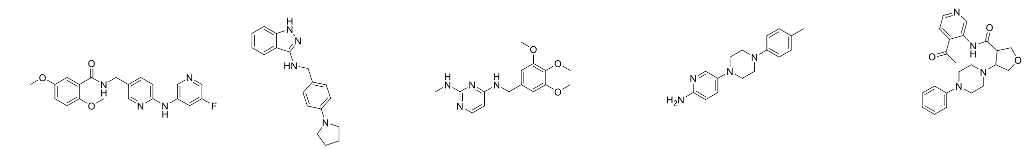
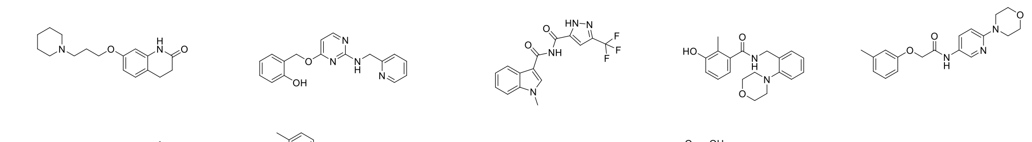
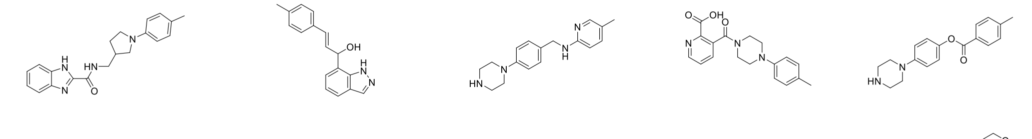
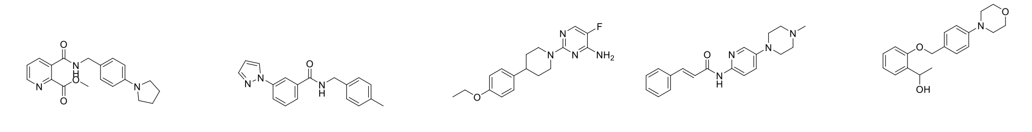
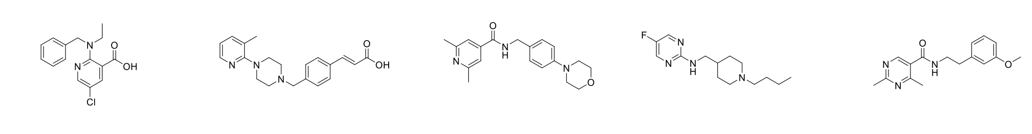

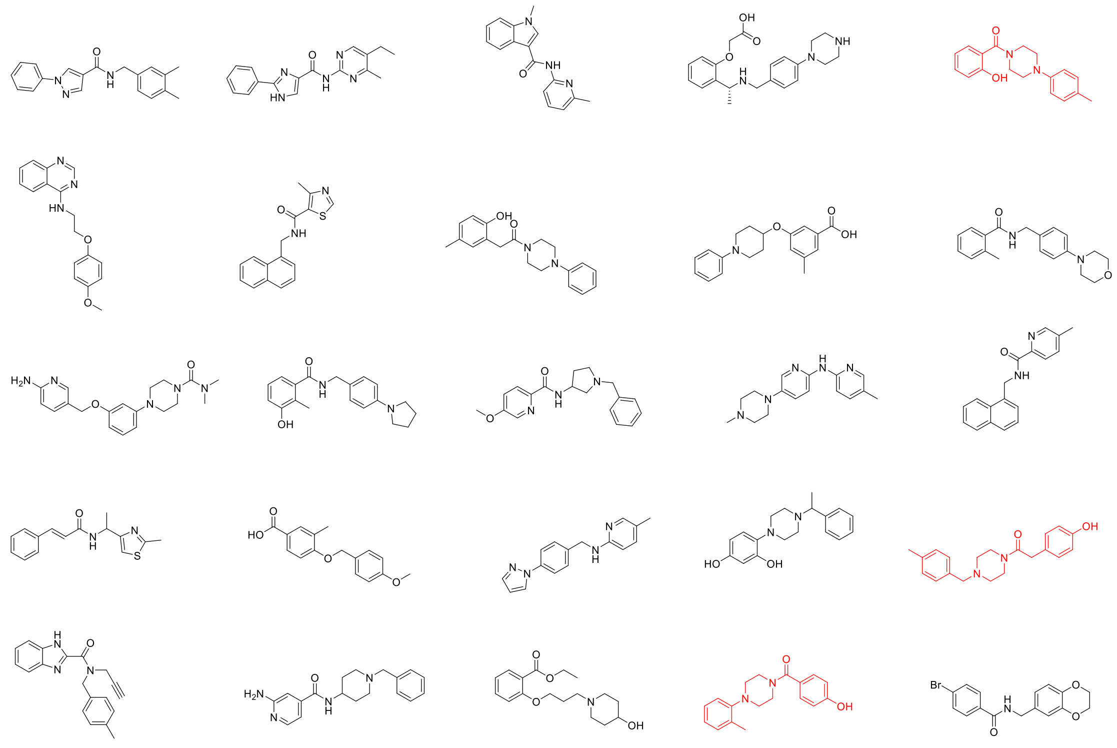

Task 7:

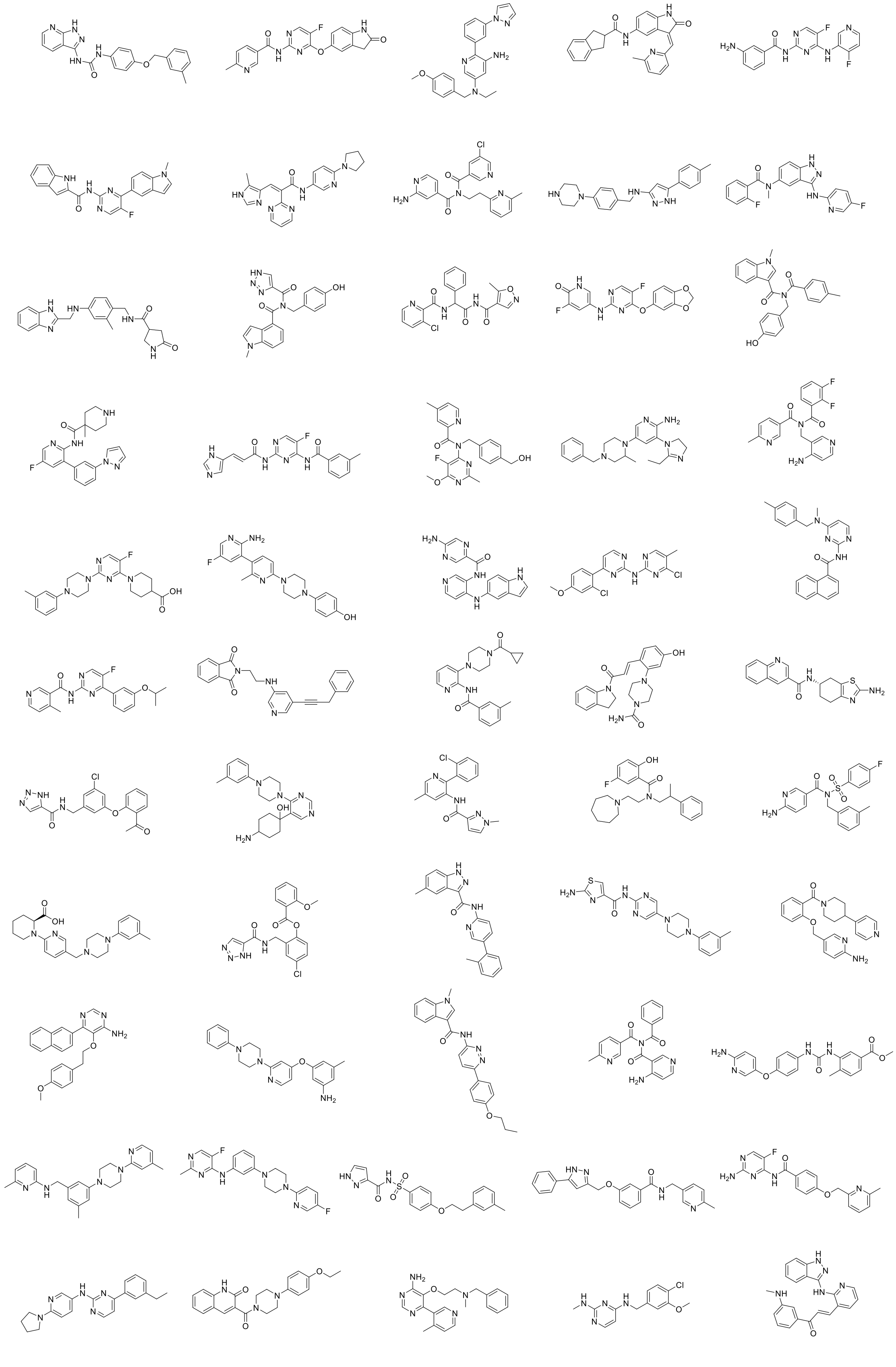

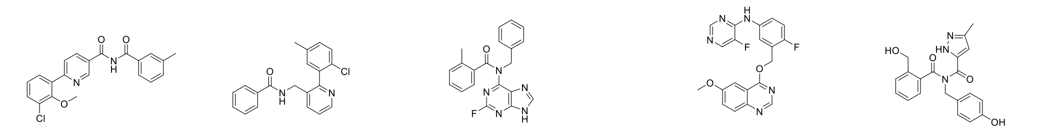
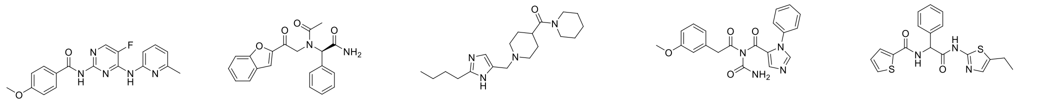
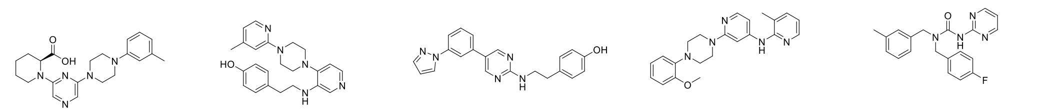
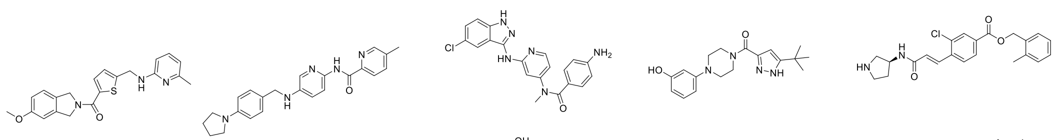
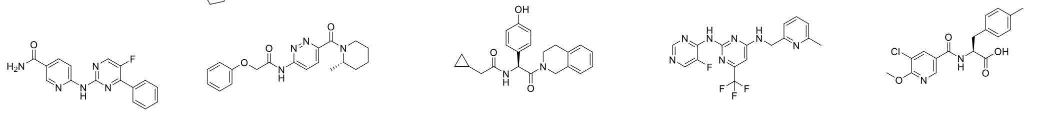
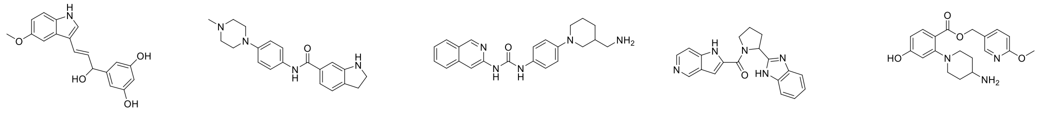
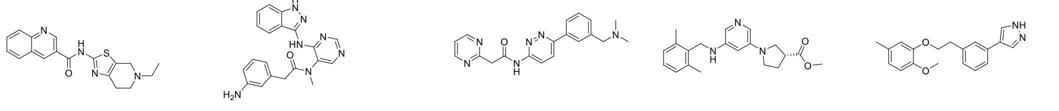
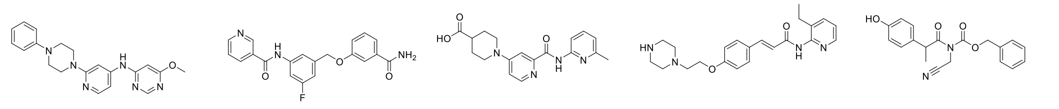
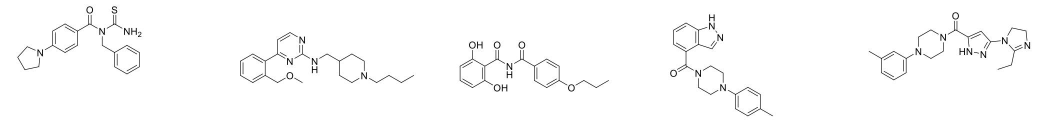
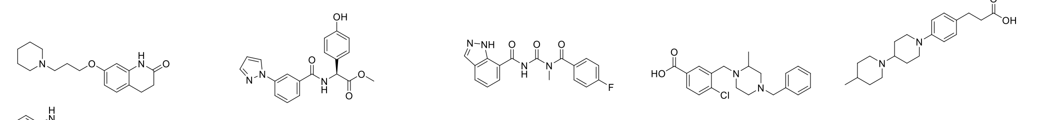
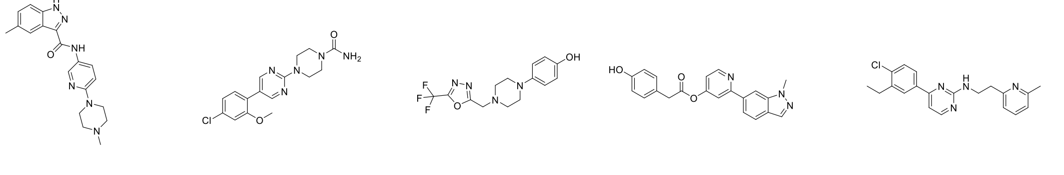
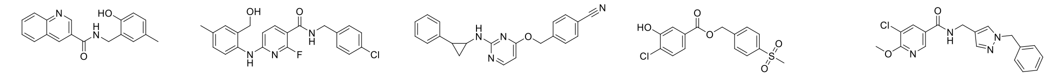
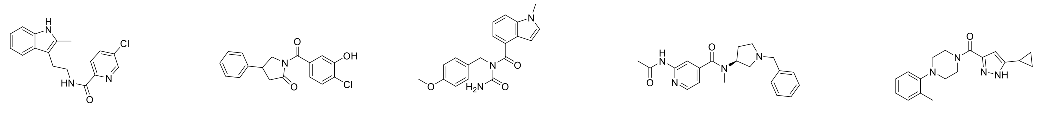

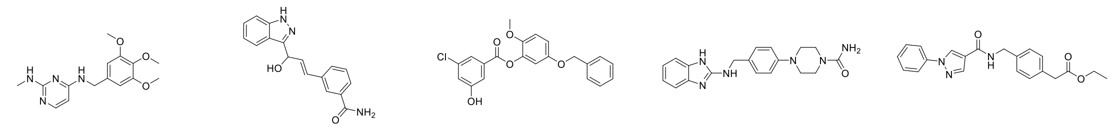
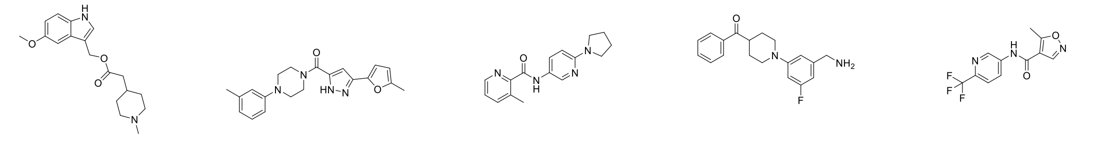
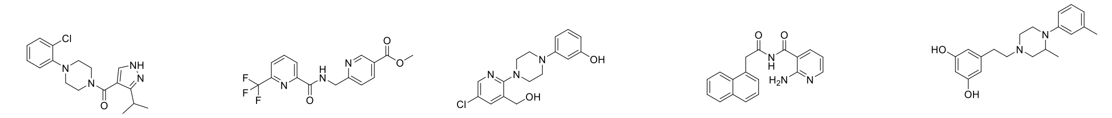
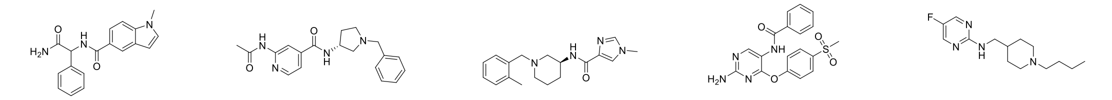
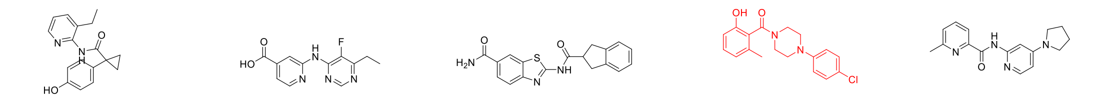
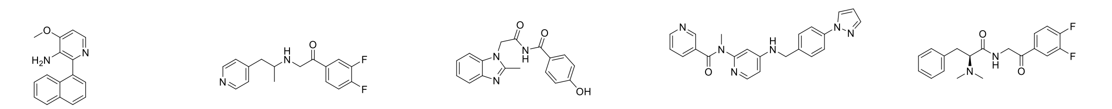
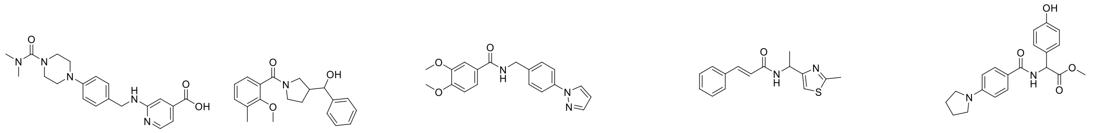
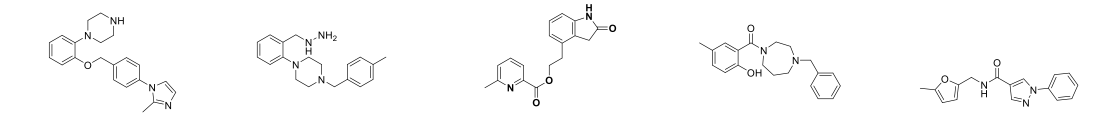
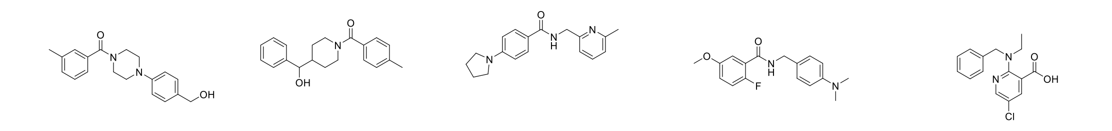
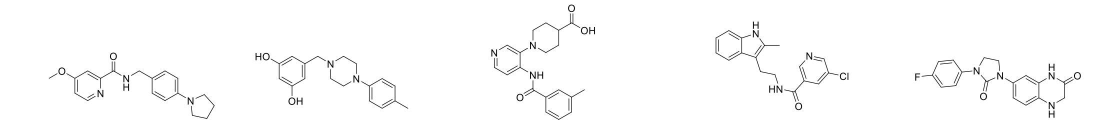
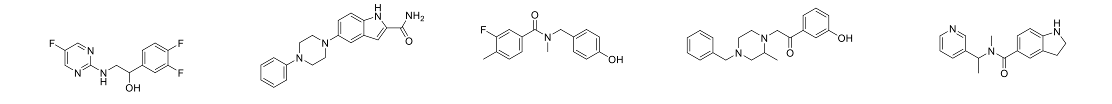
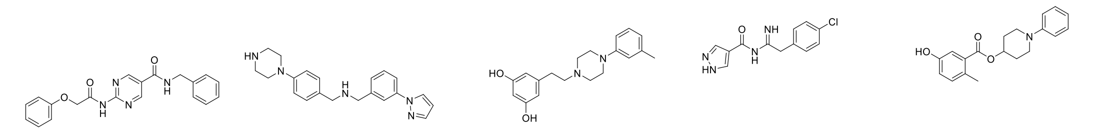

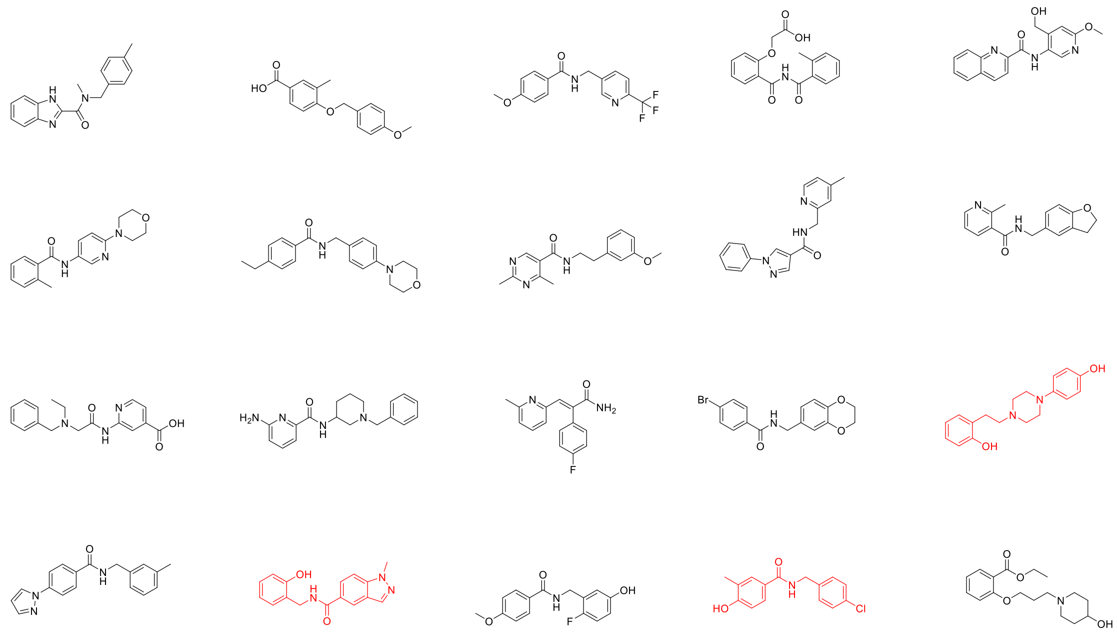

Task 8:

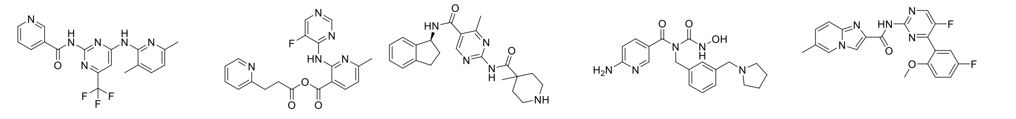

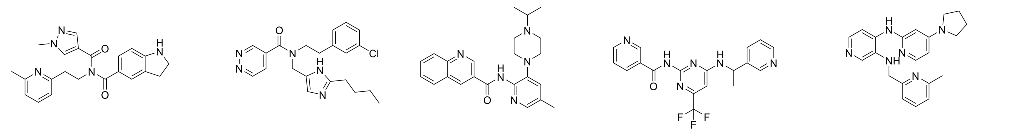

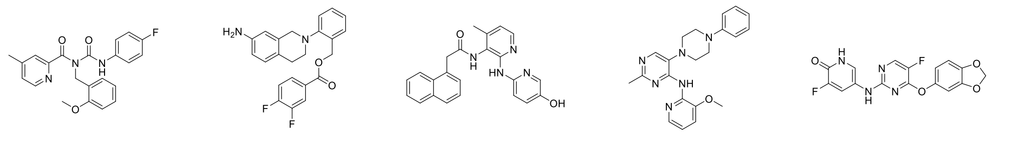

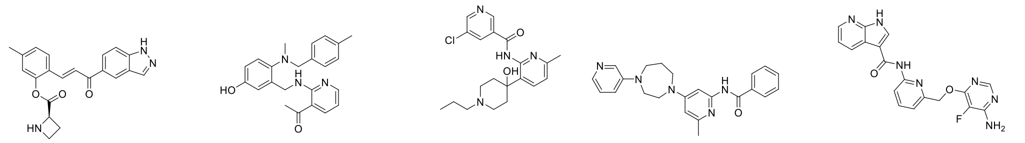

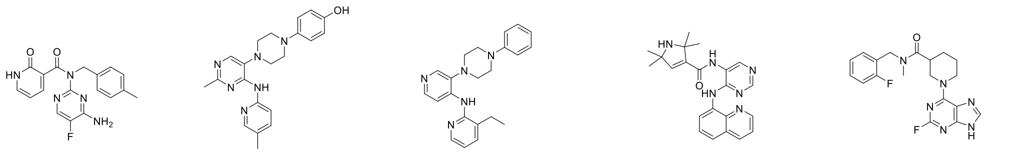

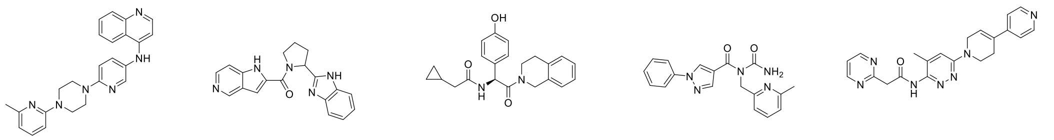

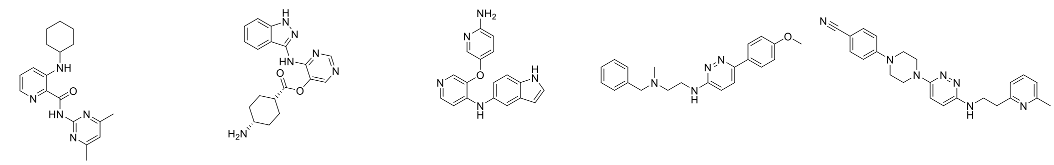

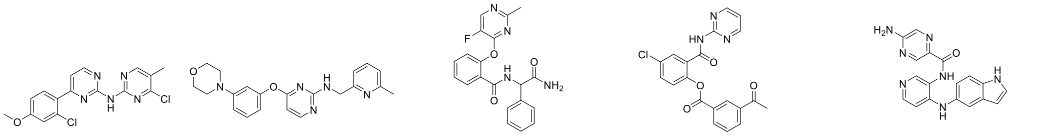

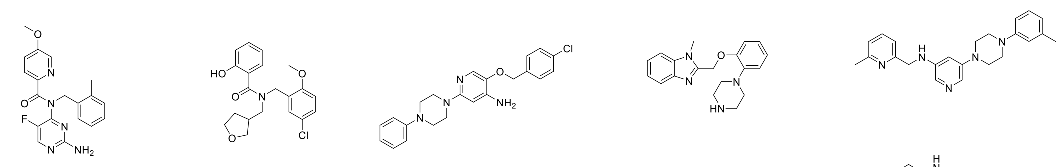

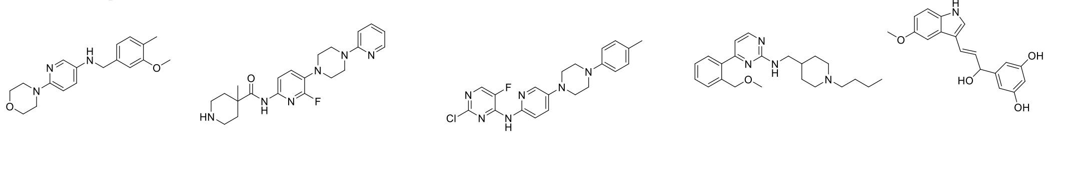

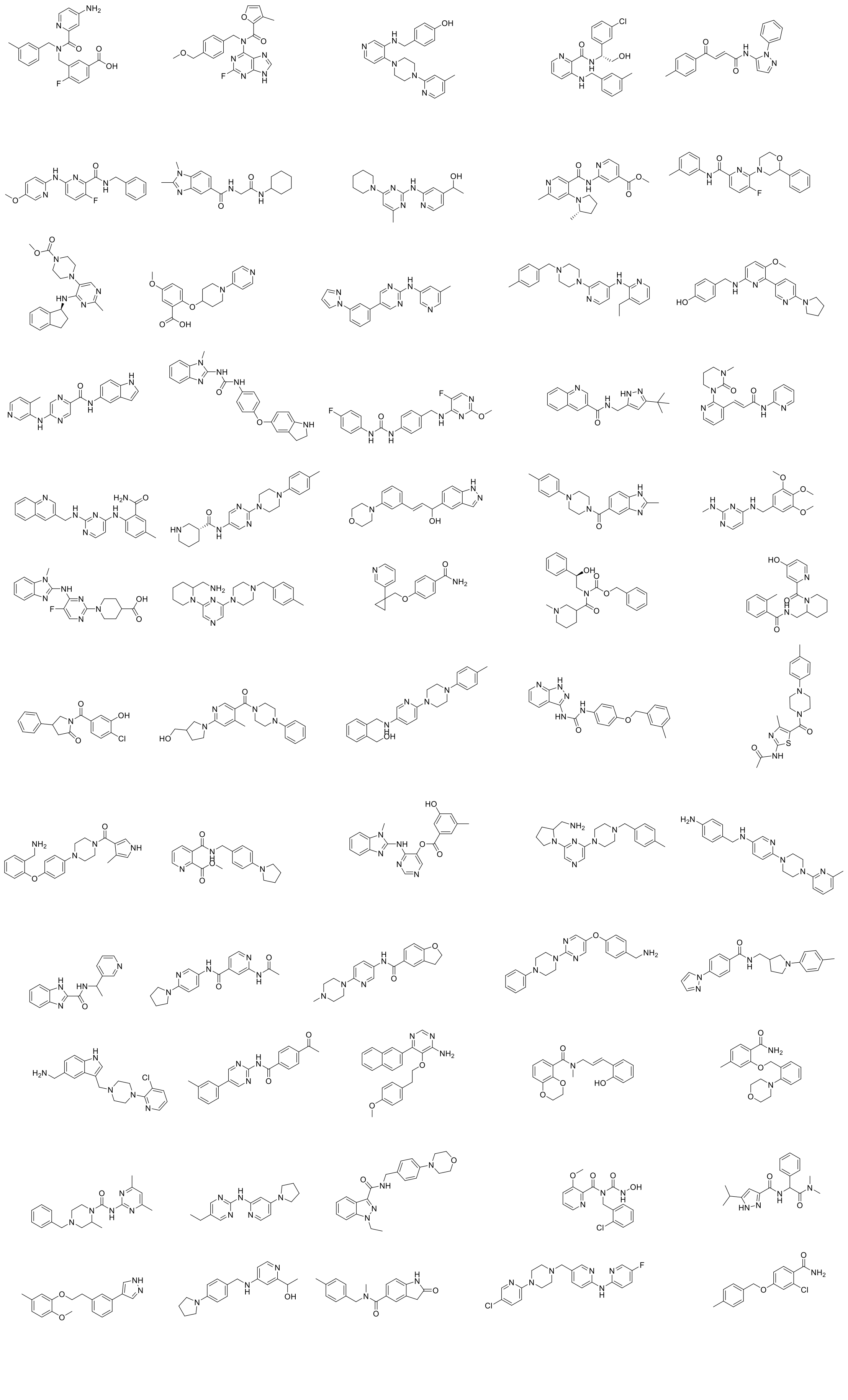

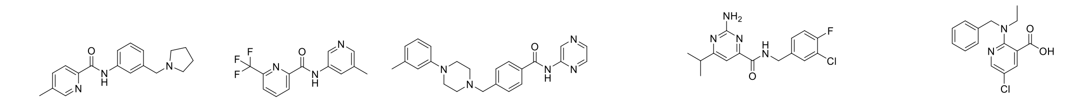
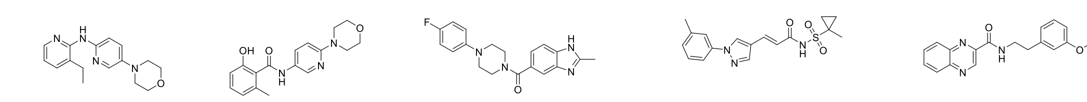
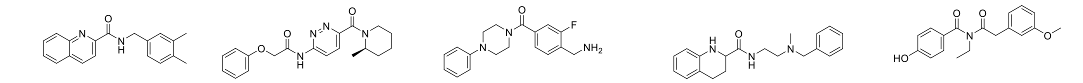
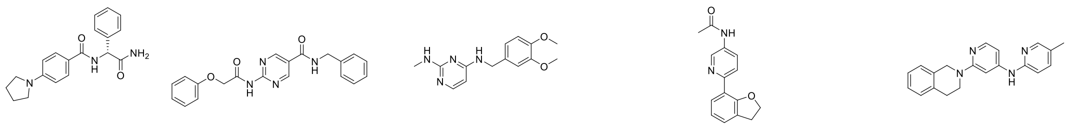
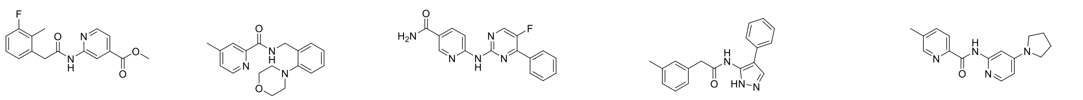
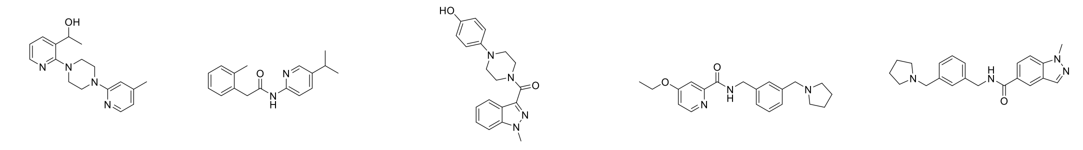
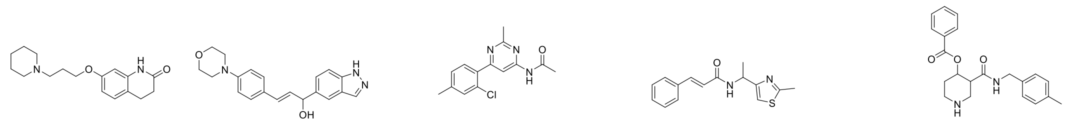
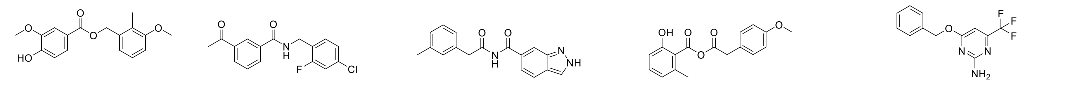
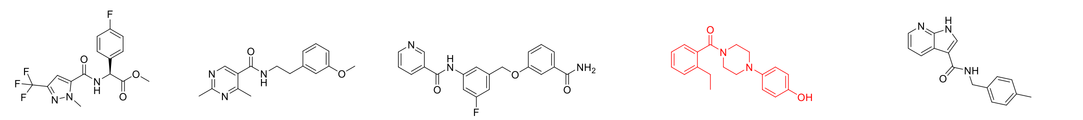
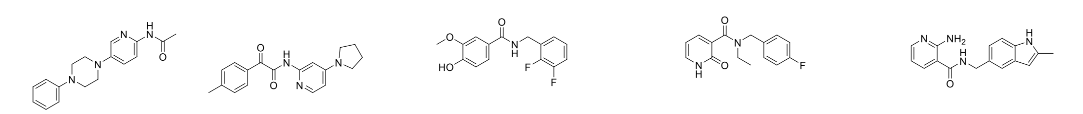
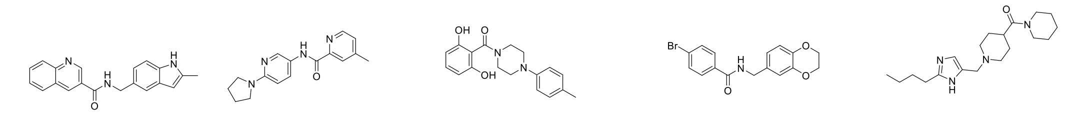
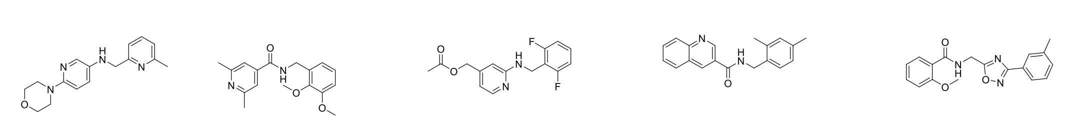
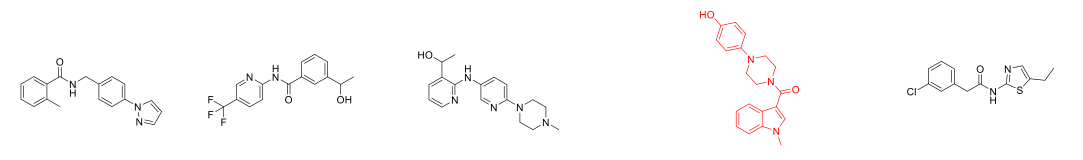

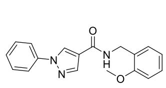
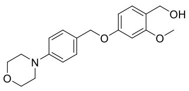
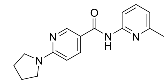
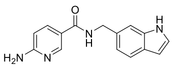
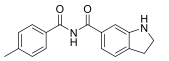
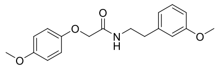
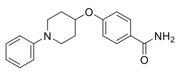
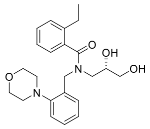
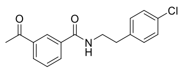
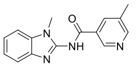
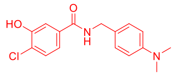
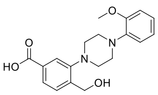
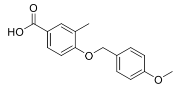
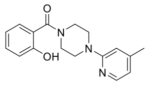
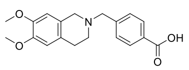
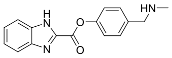
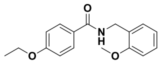
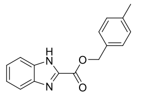
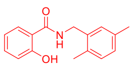
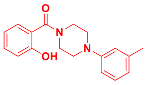
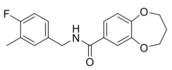
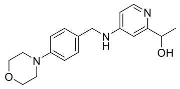
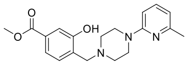
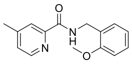
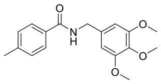

Task 9:

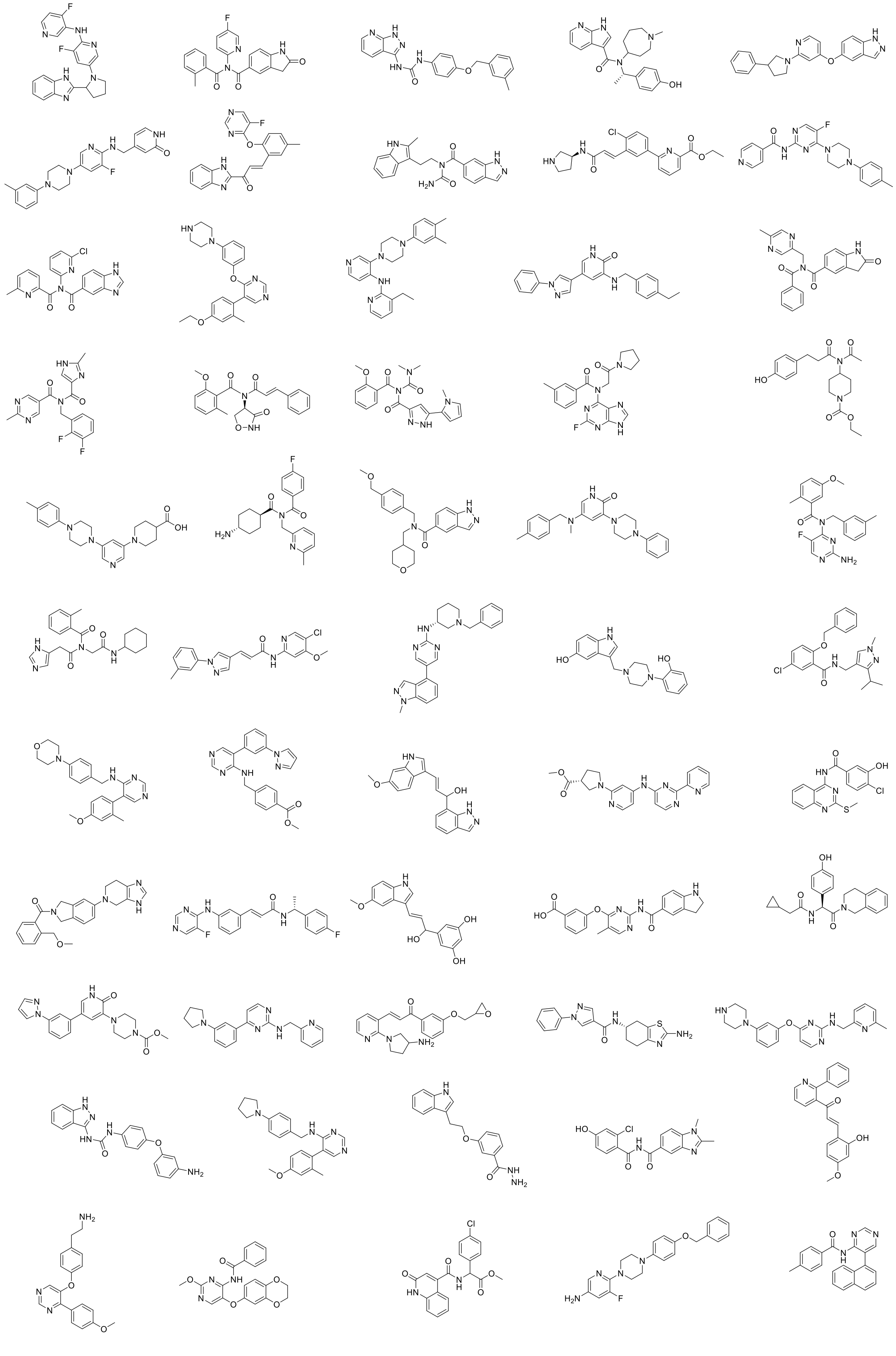

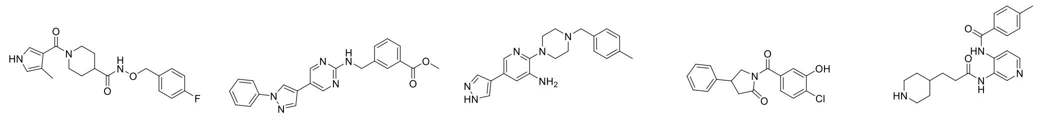
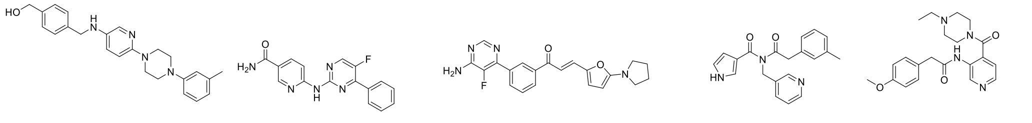
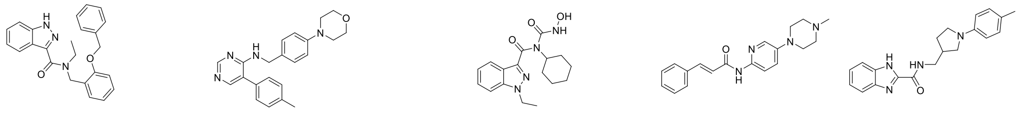
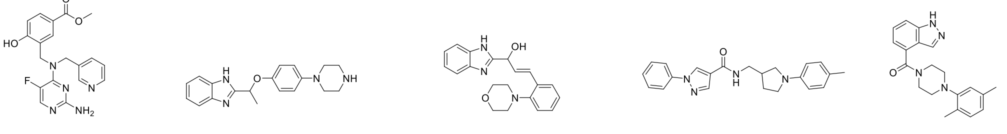
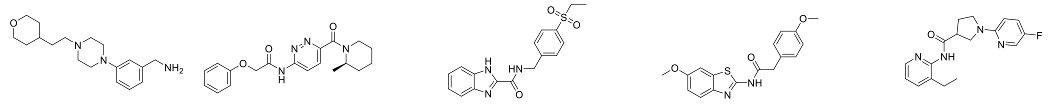
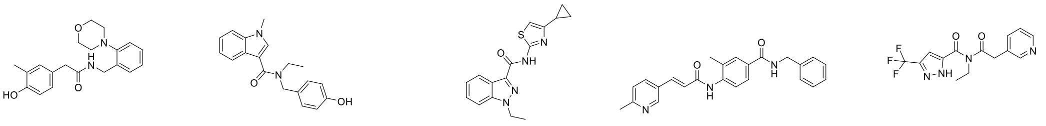
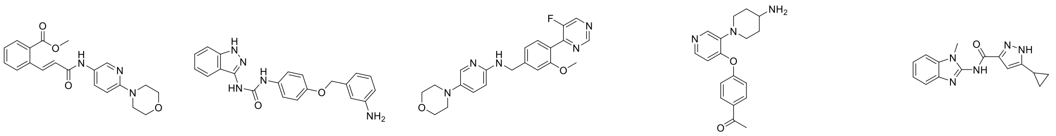
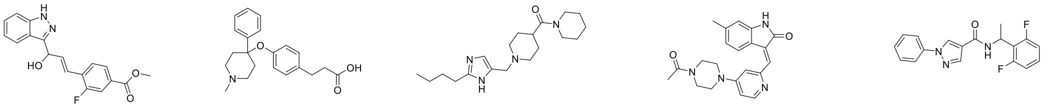
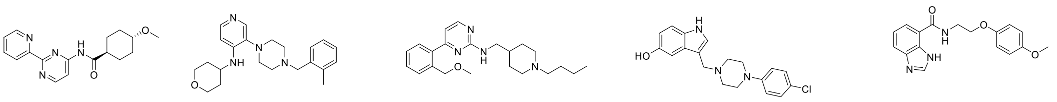
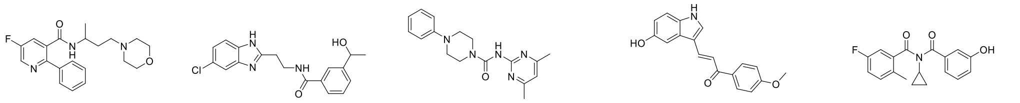
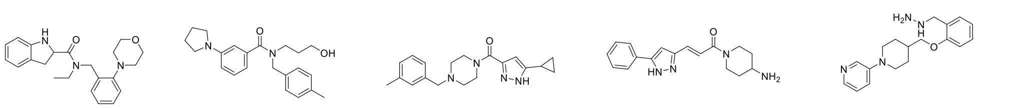
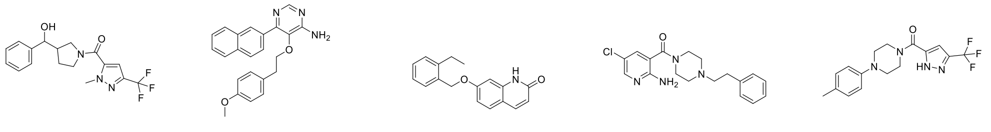

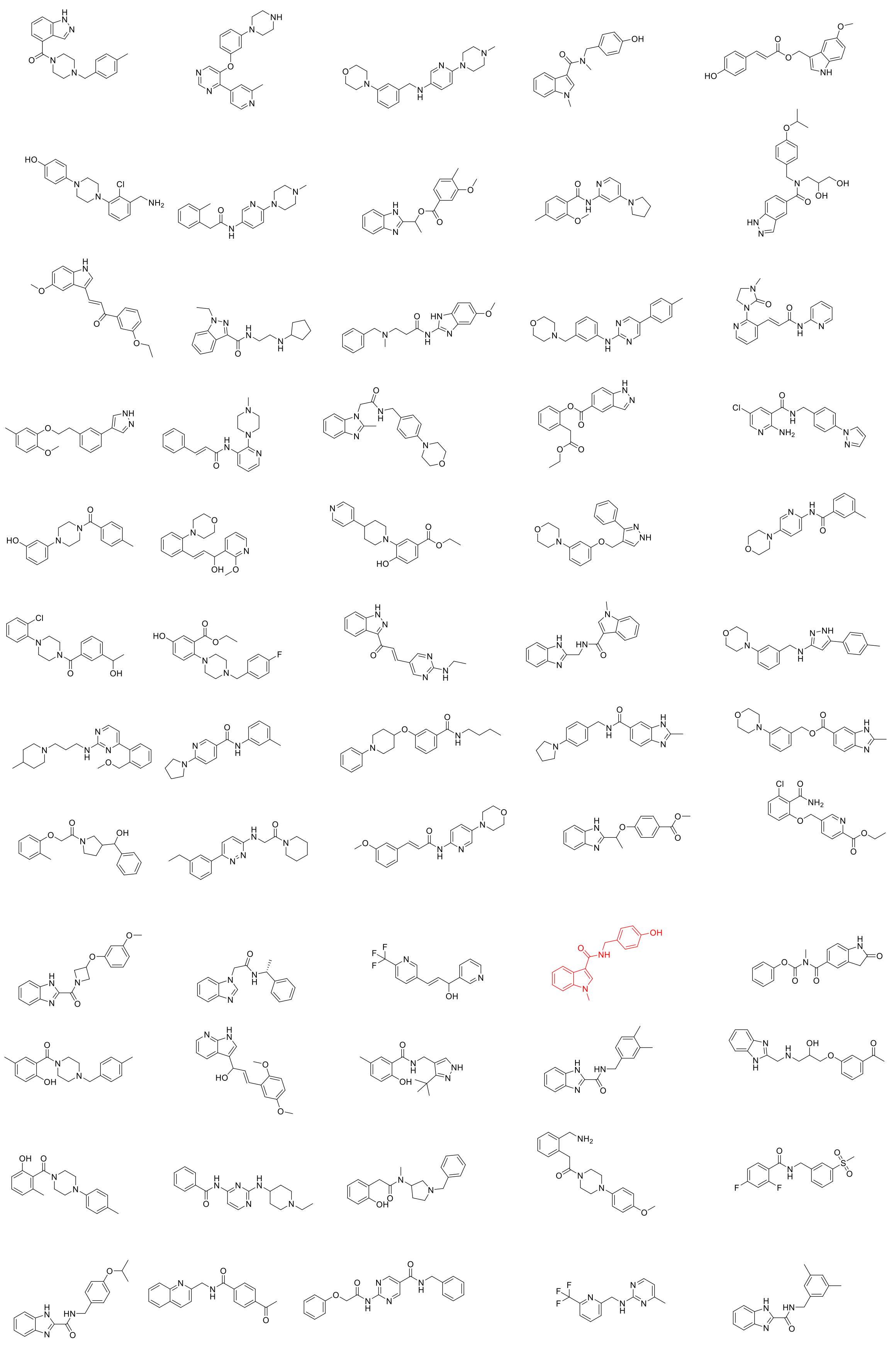

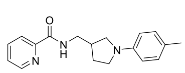
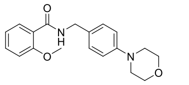
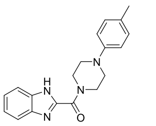
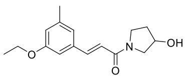
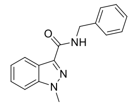
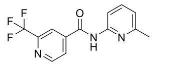
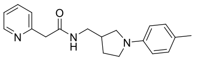
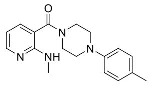
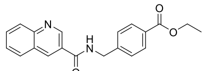
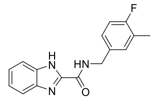
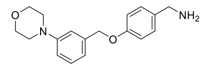
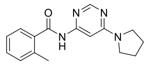
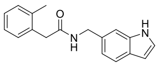
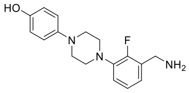
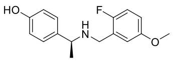
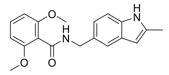
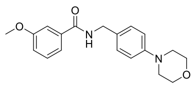
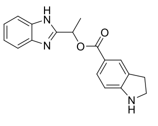
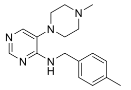
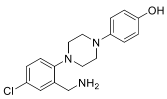
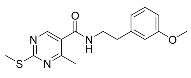
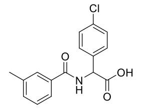
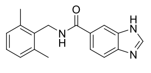
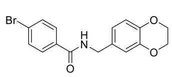
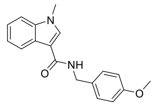

Task10:

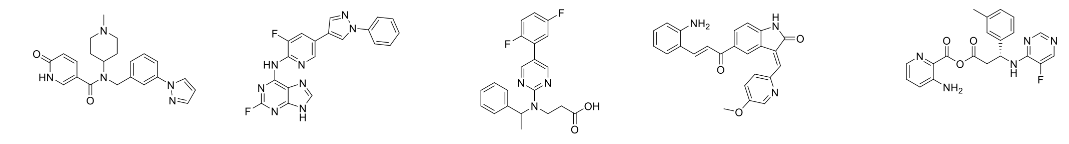

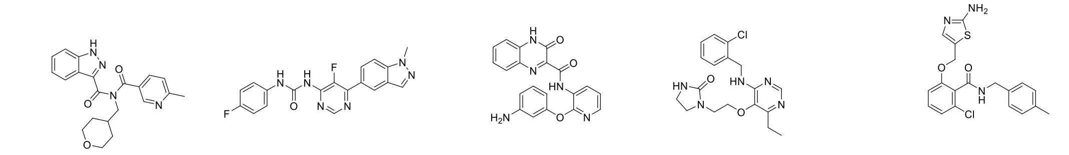

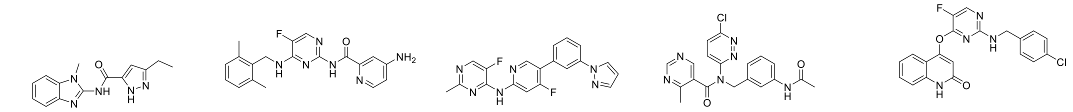

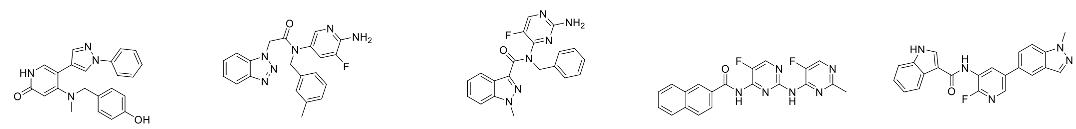

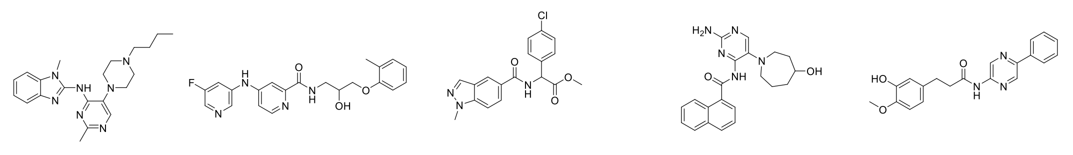

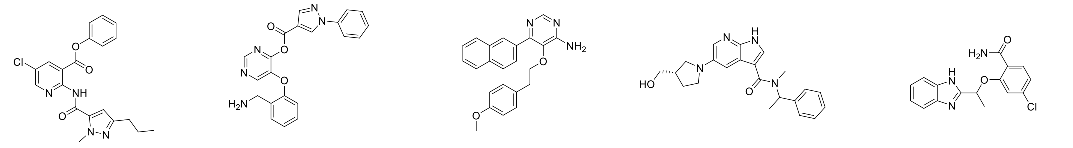

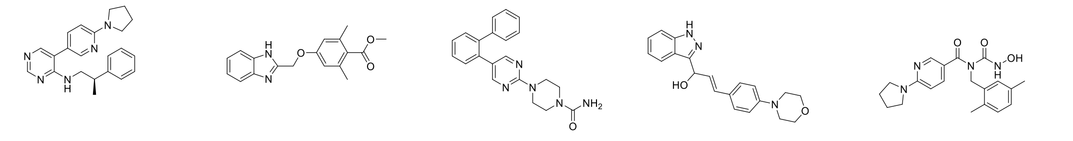

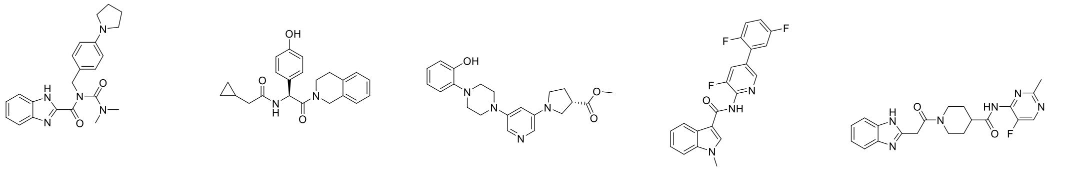

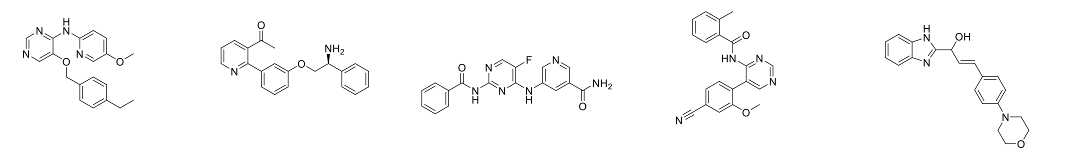

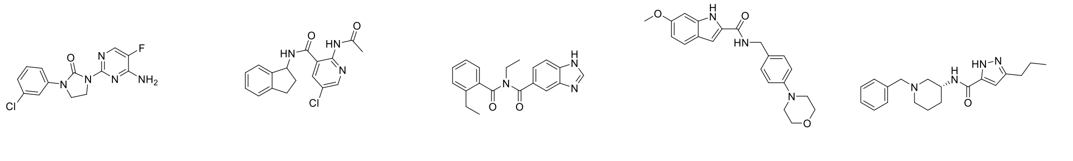

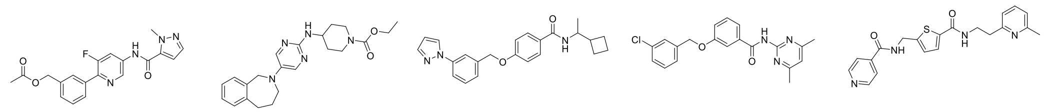
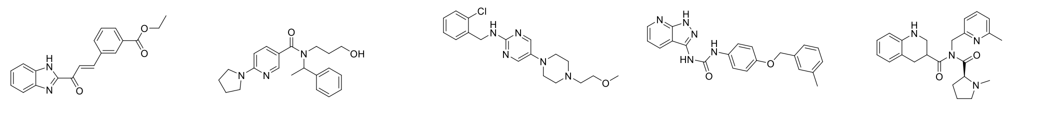
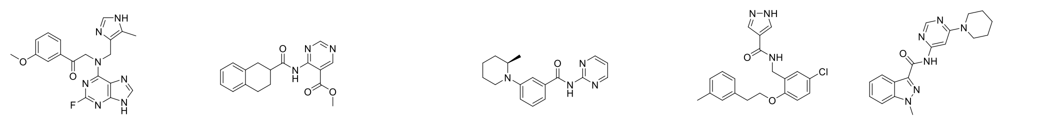
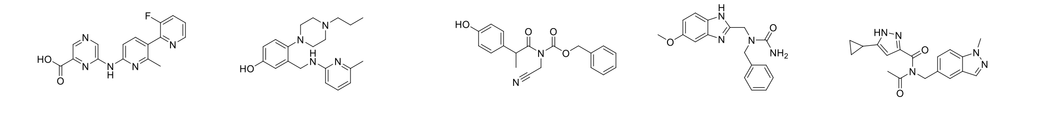
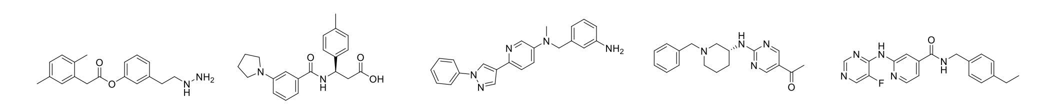
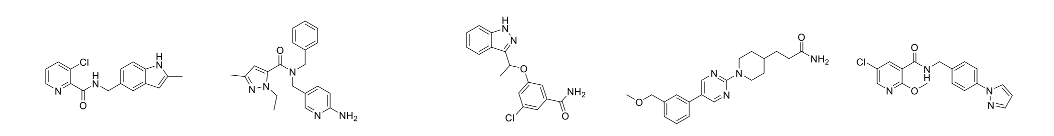
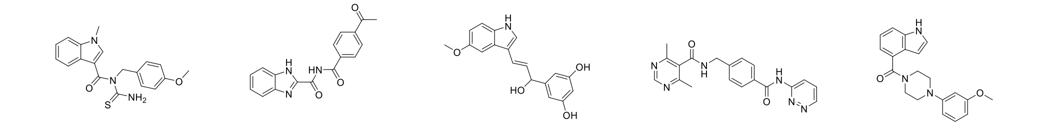
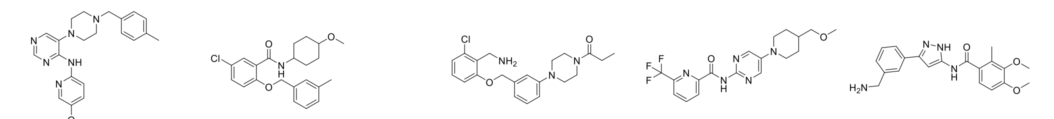
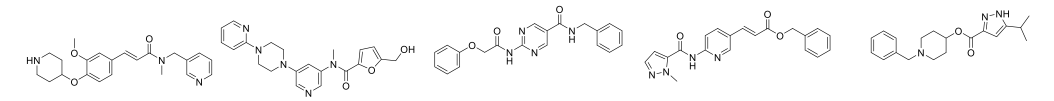
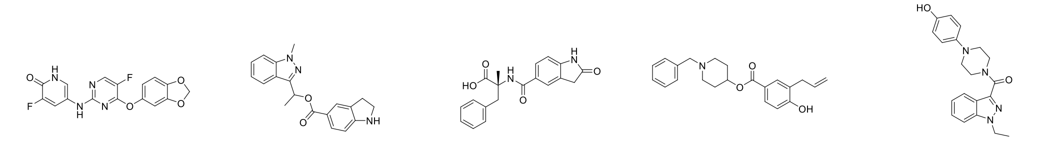
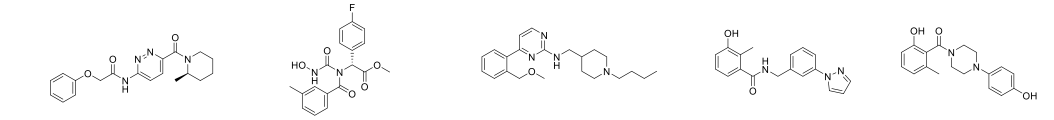
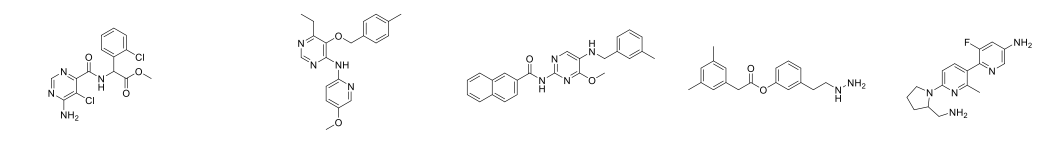

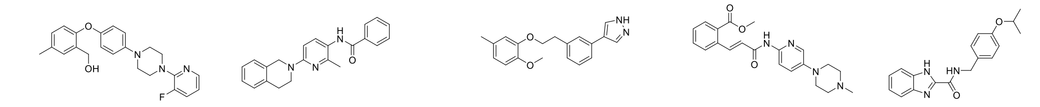
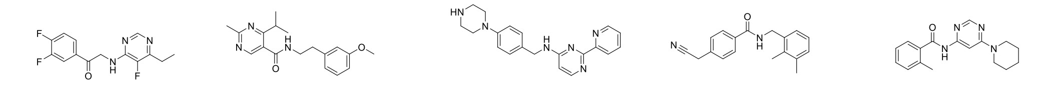
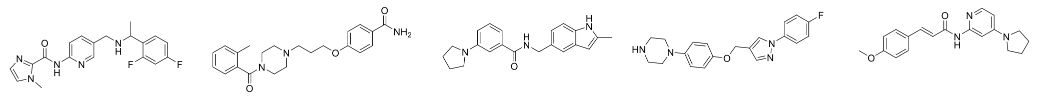
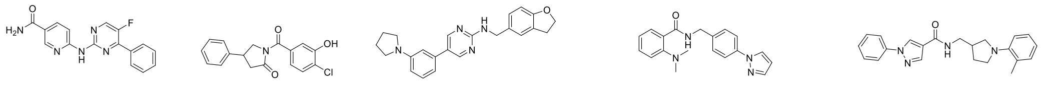
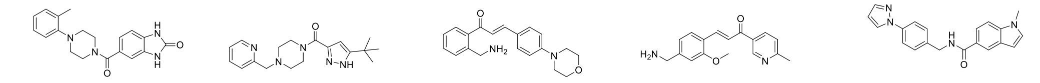
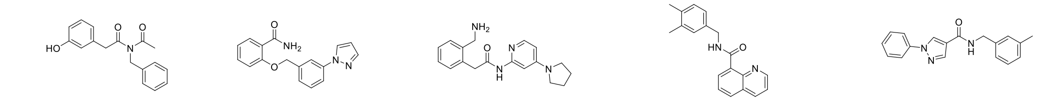
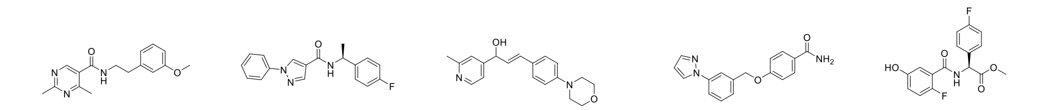
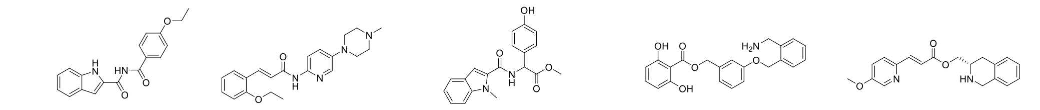
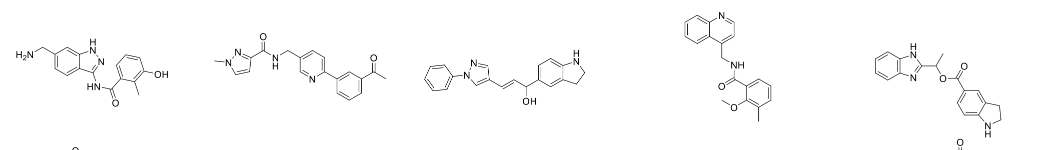
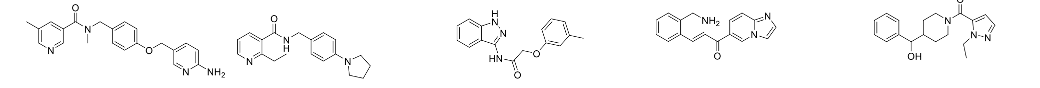
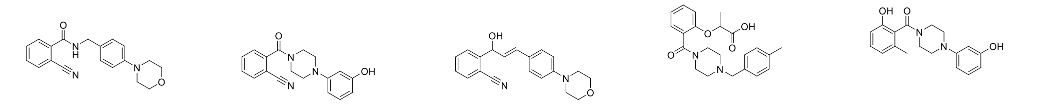
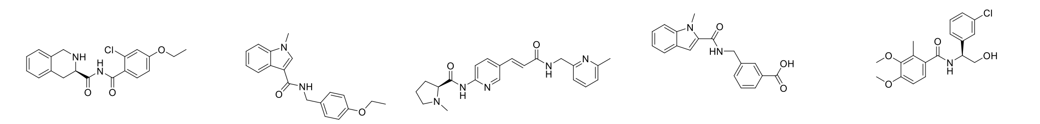
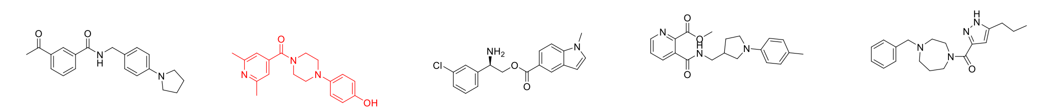
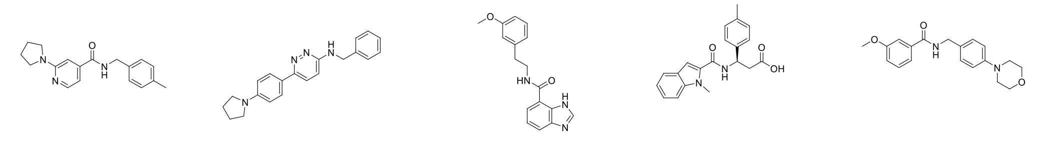

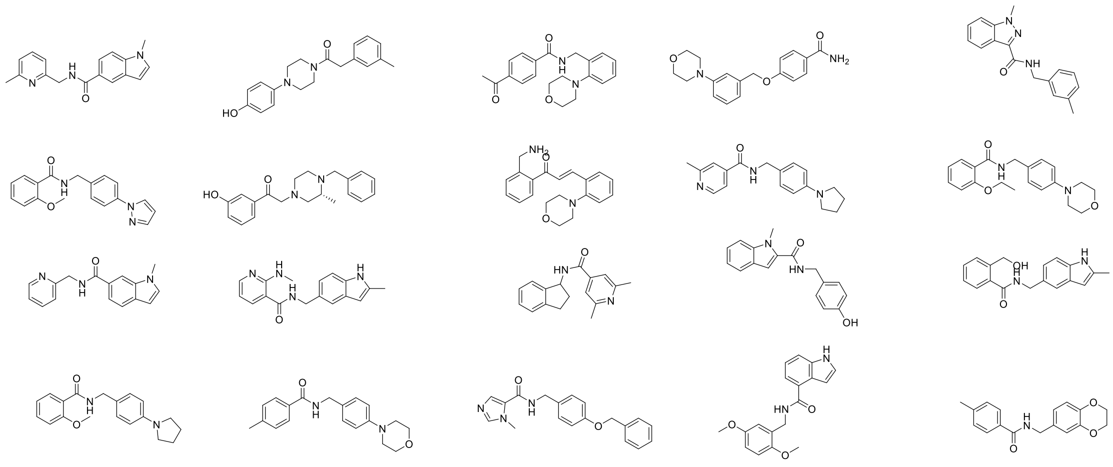

Task 11:

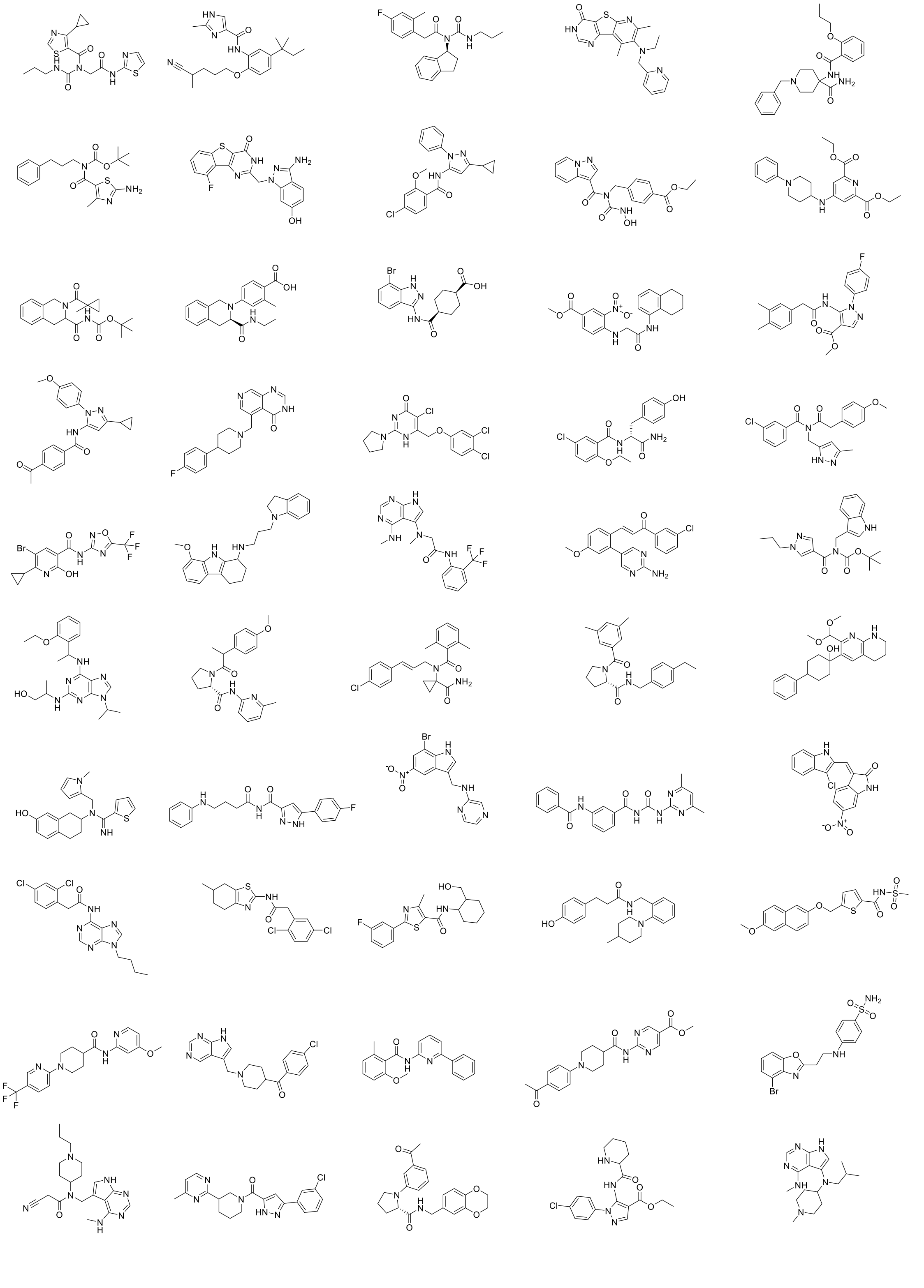

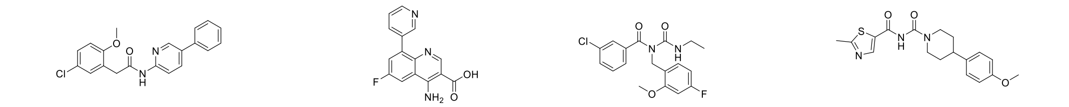
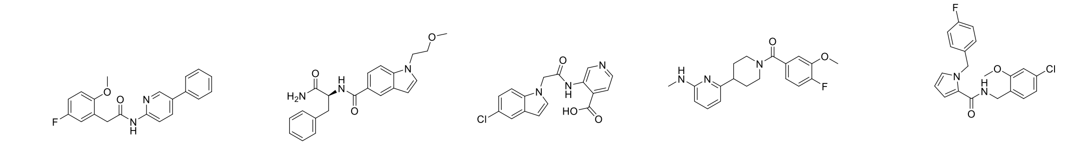
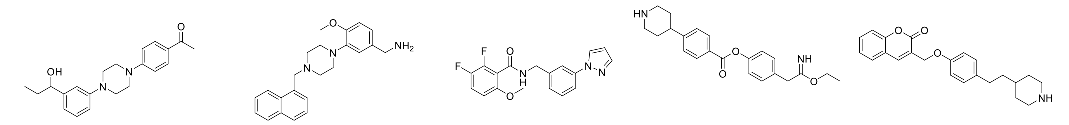
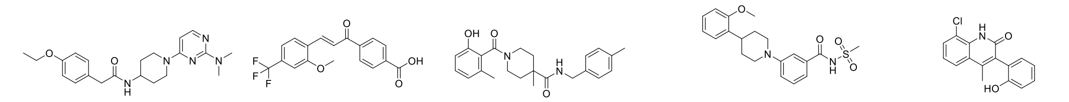
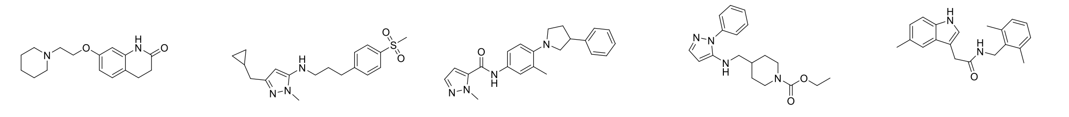
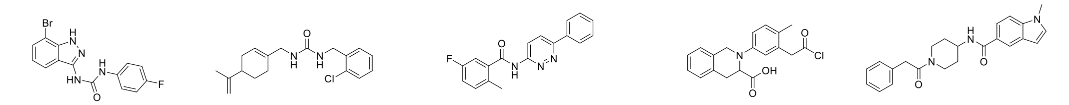
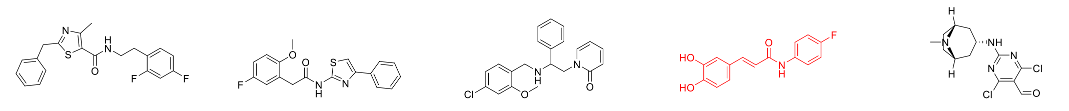
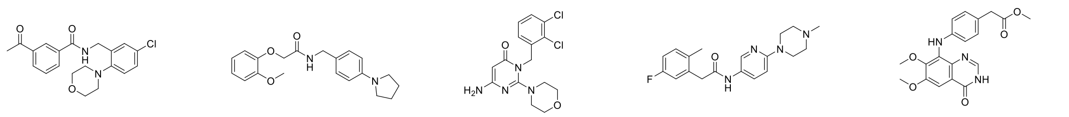
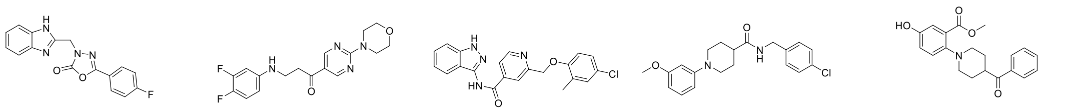
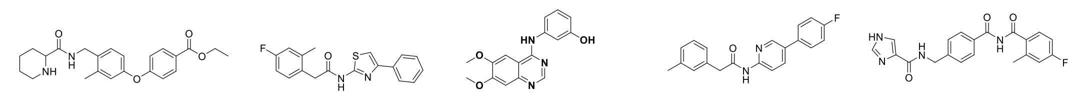
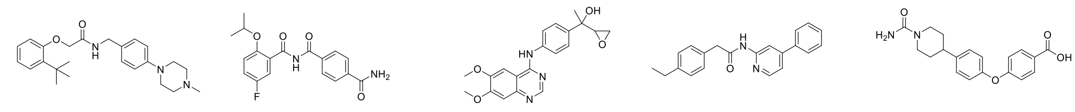
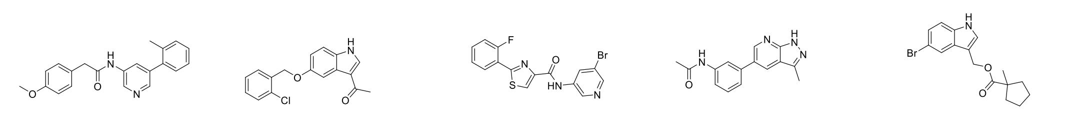
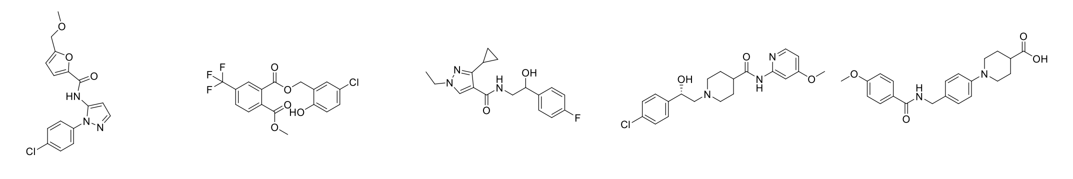

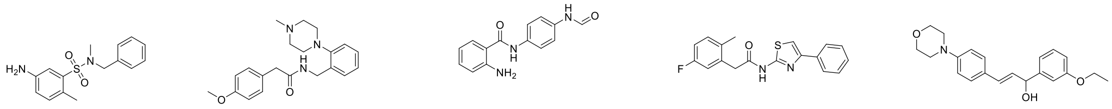
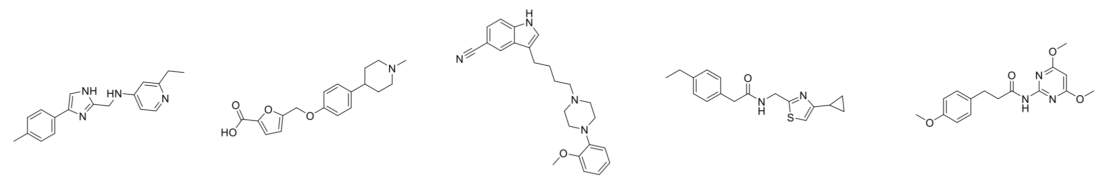
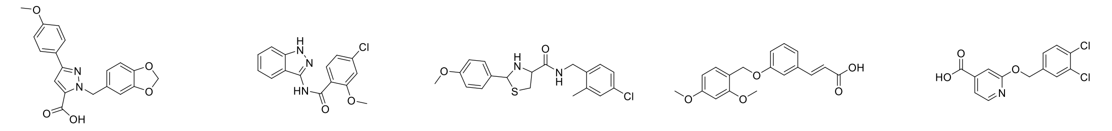
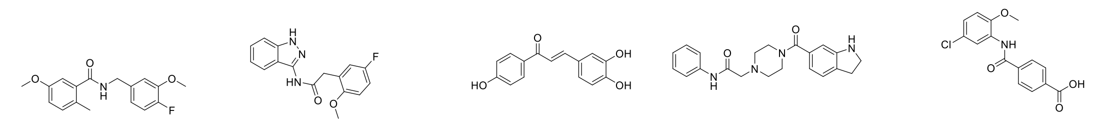
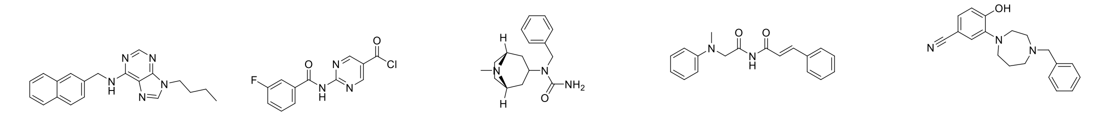
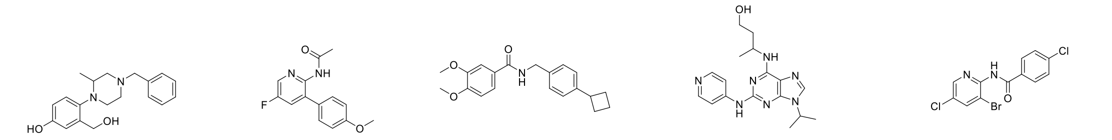
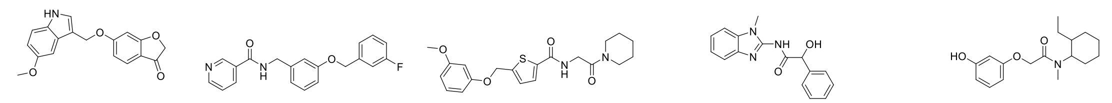
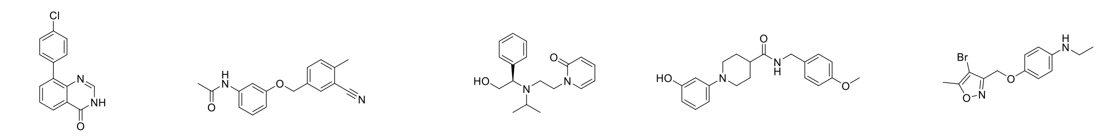
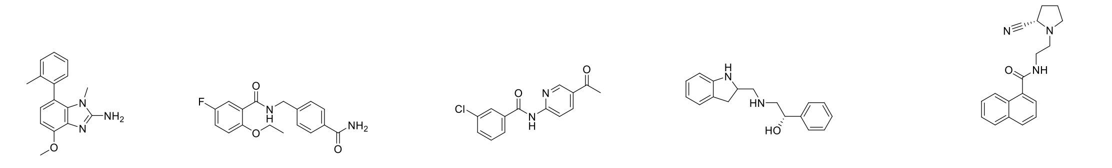

Task 12:

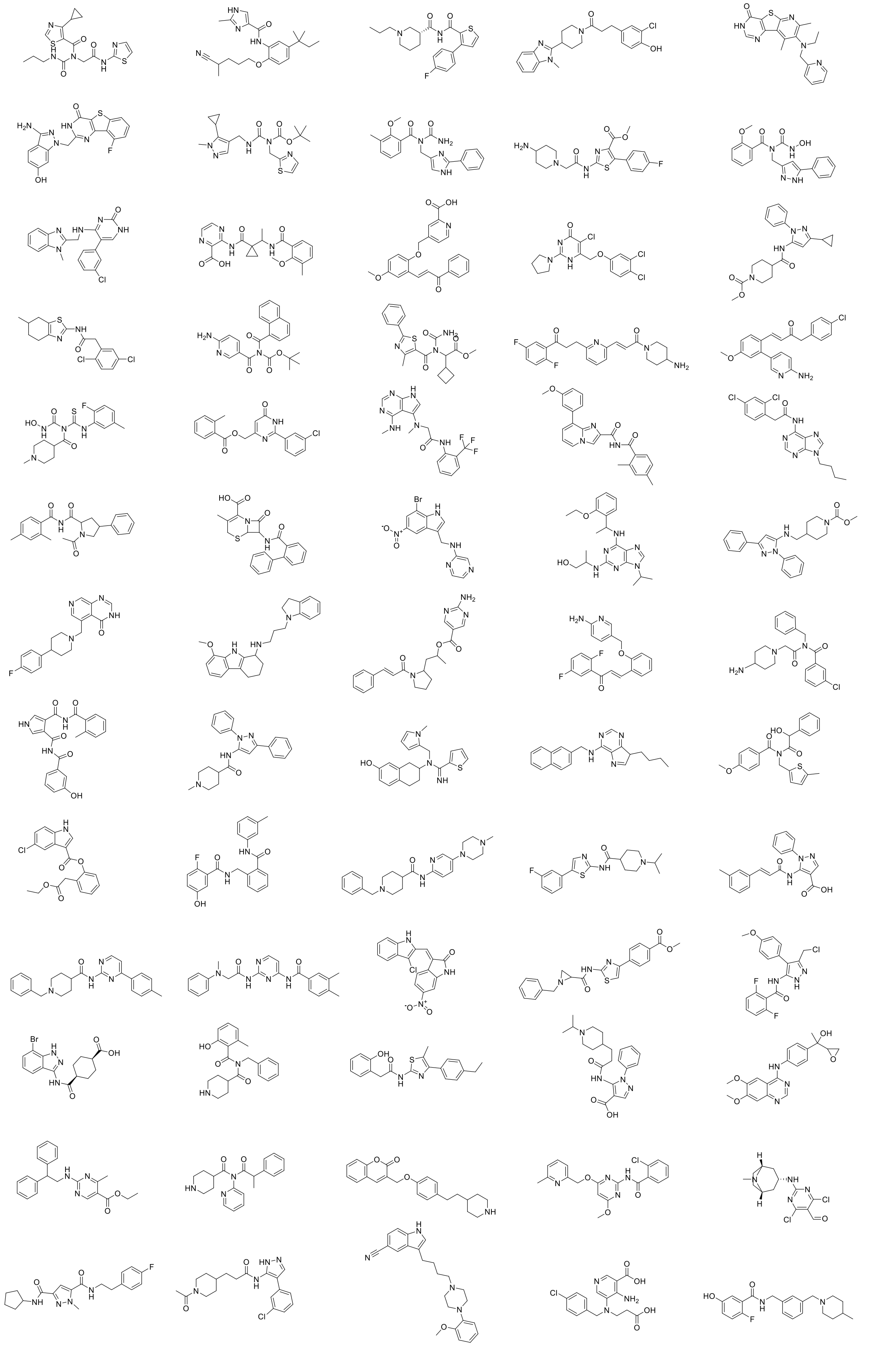

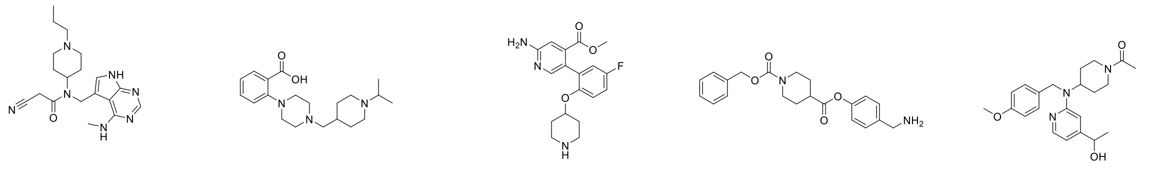
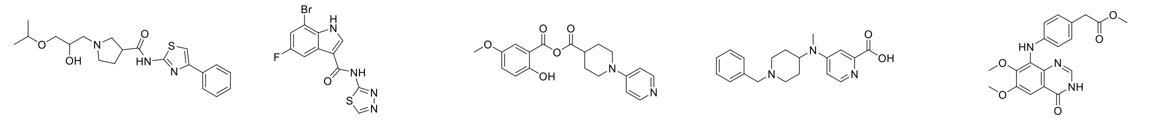
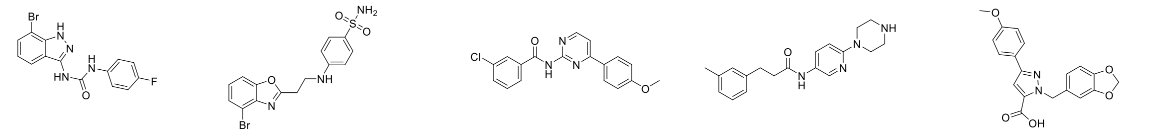
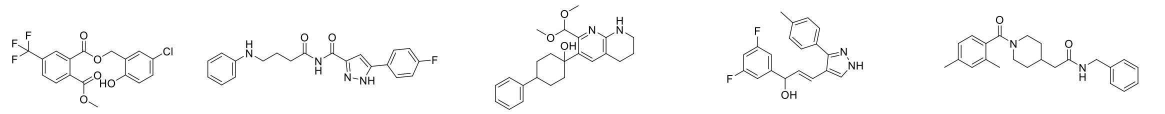
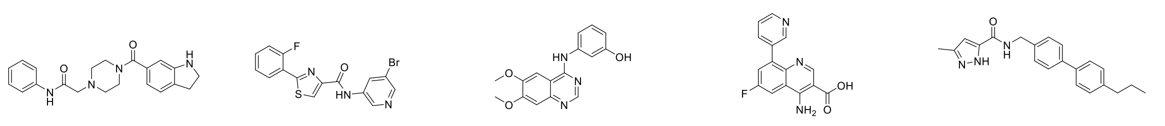
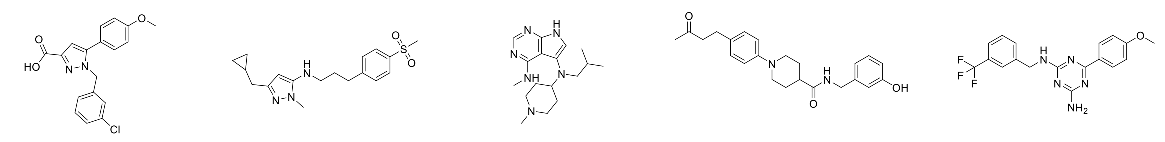
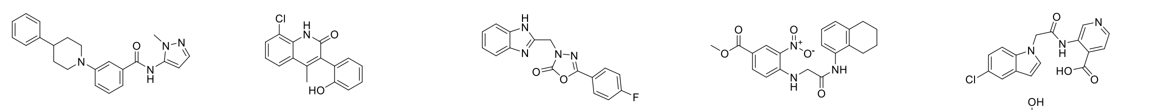
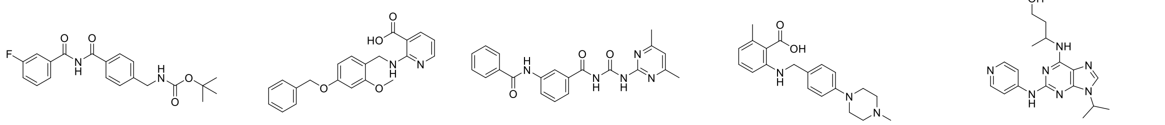
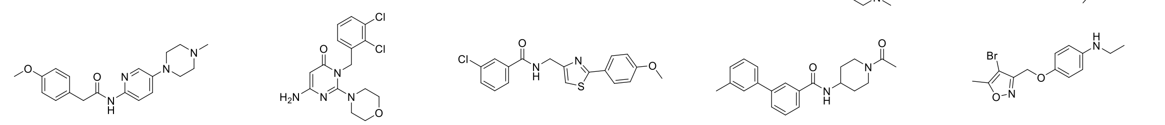
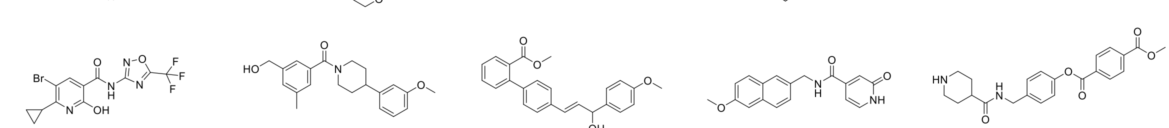
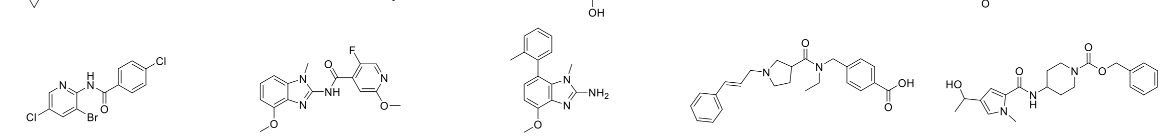
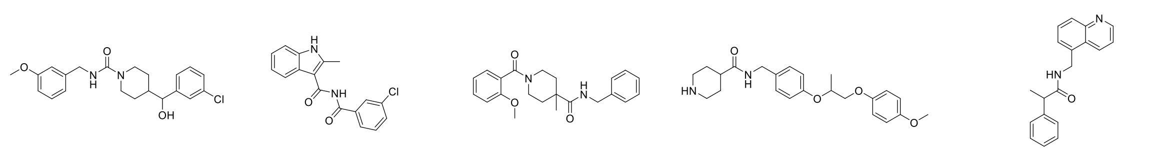
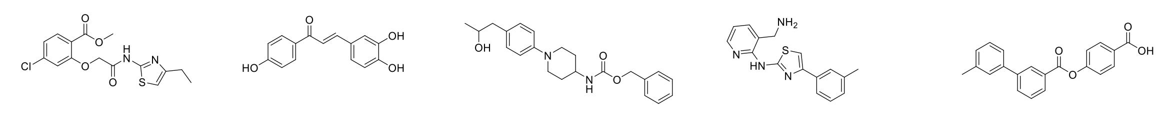

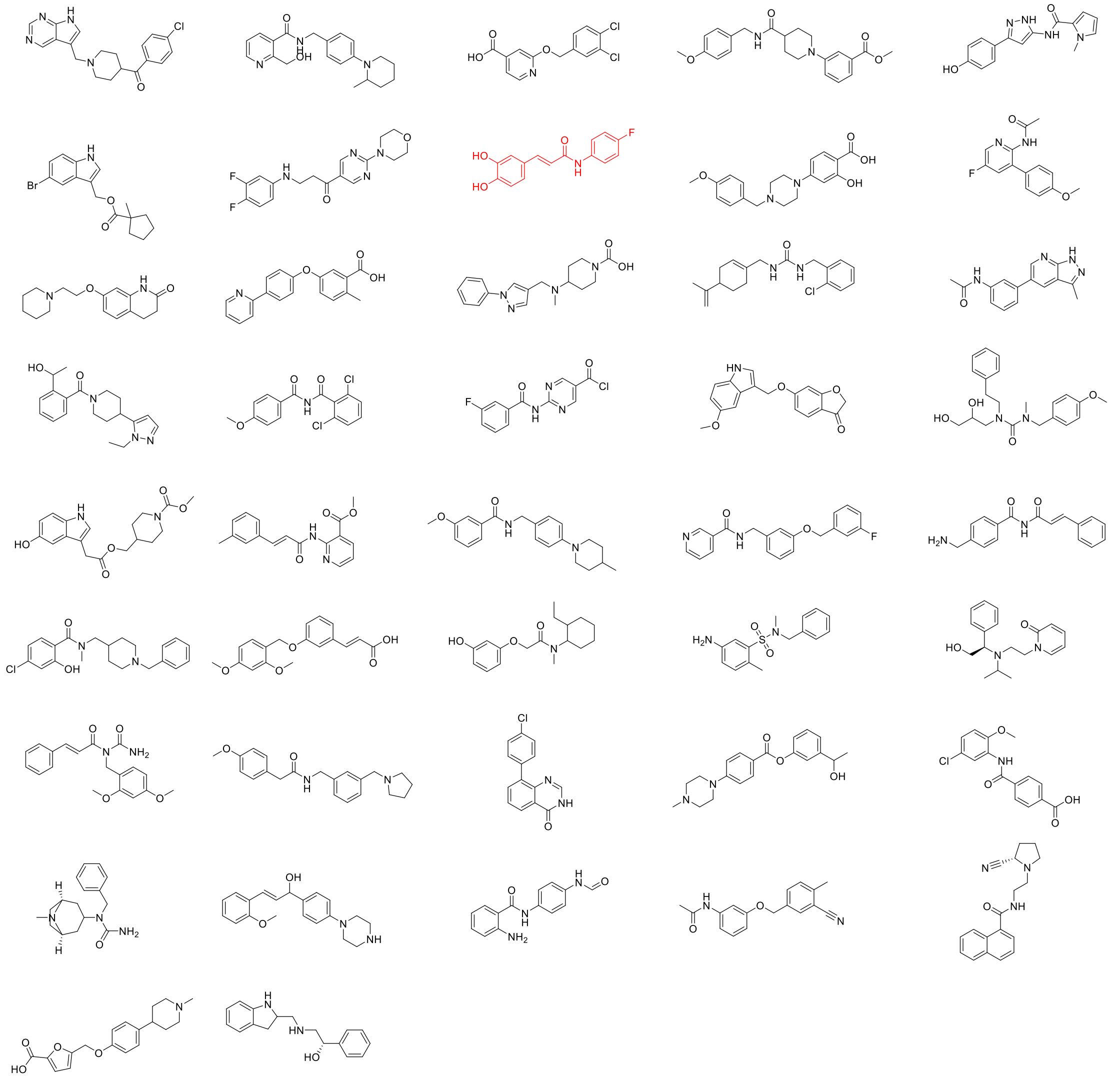

Task 13:

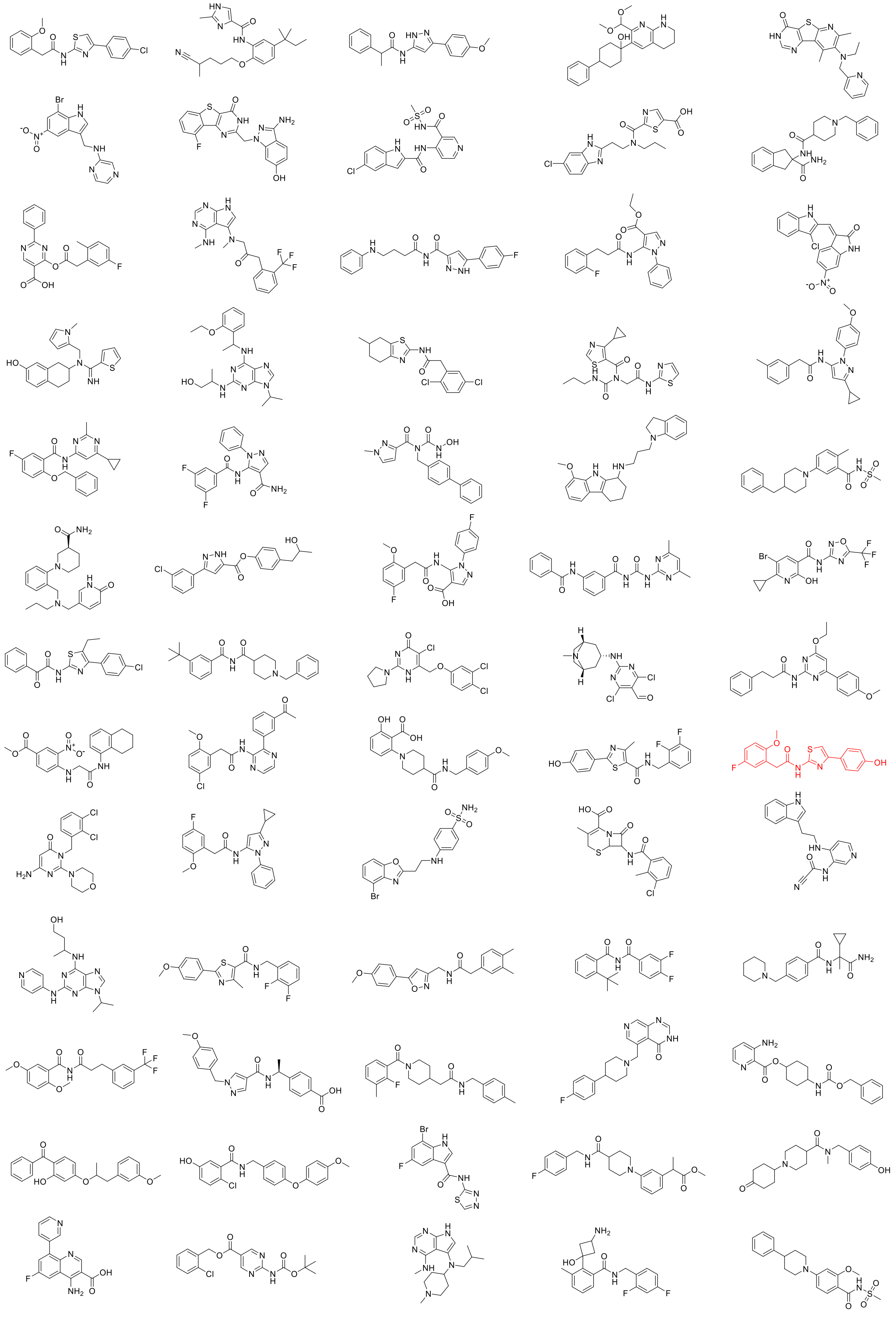

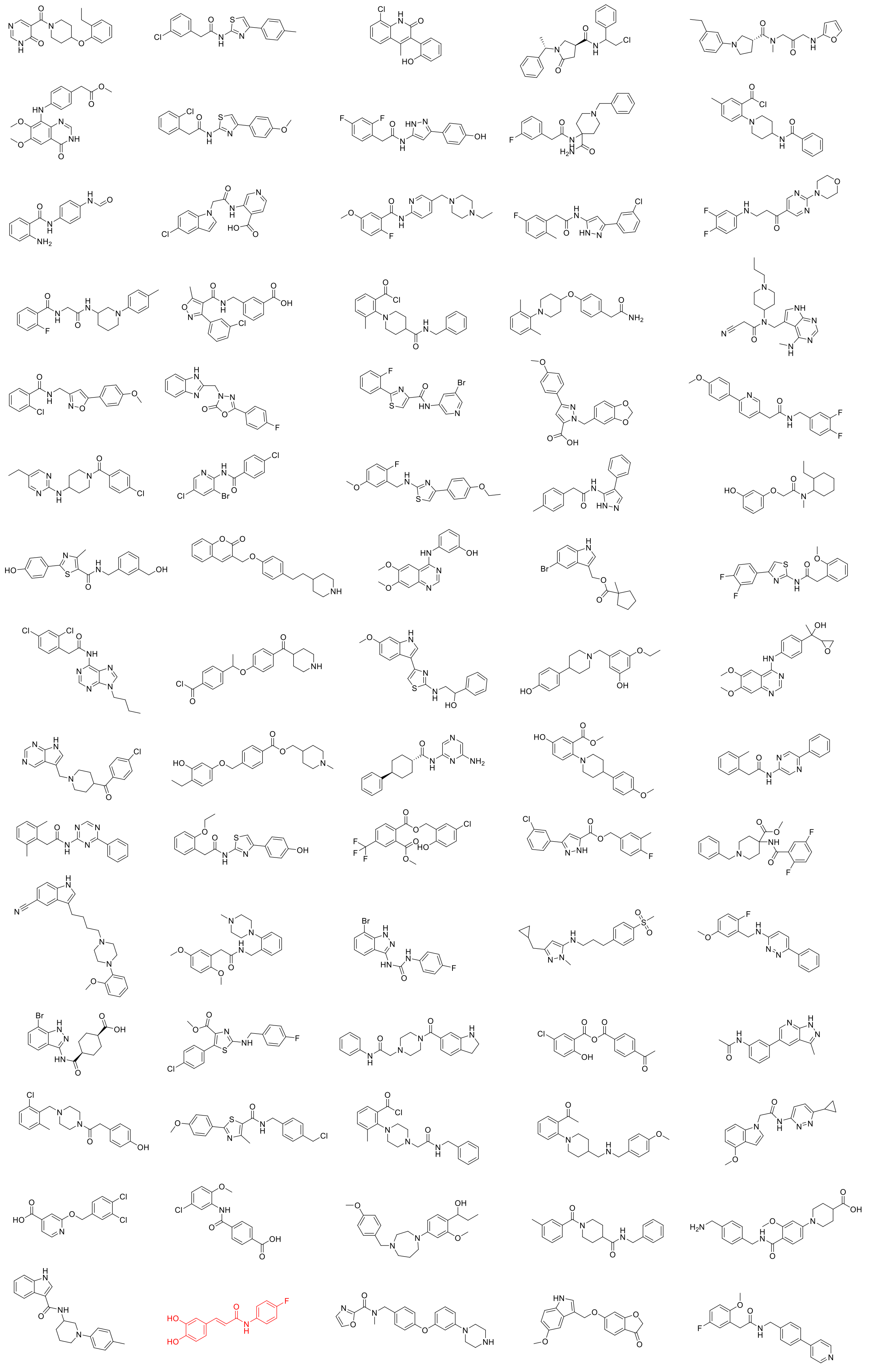

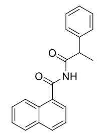
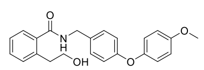
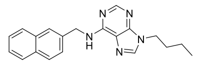
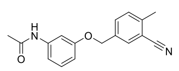
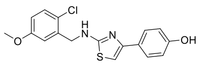
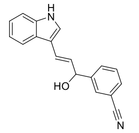
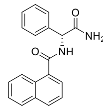
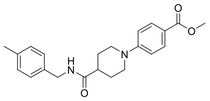
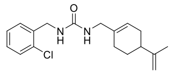
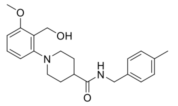
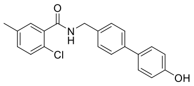
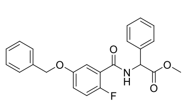
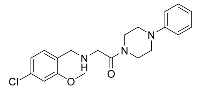
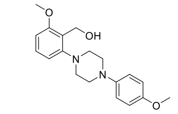
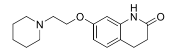
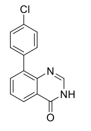
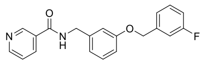
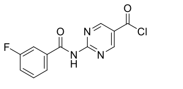
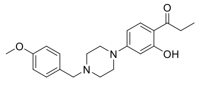
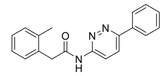
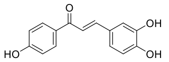
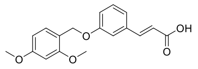
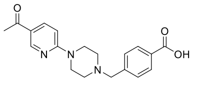
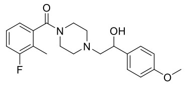
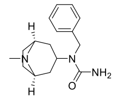
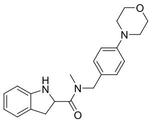
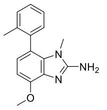
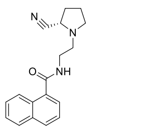
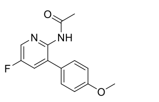
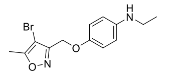
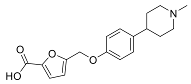
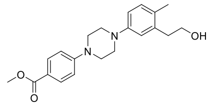
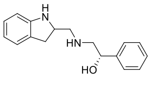
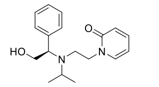
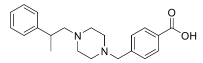
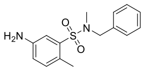

Task 14:

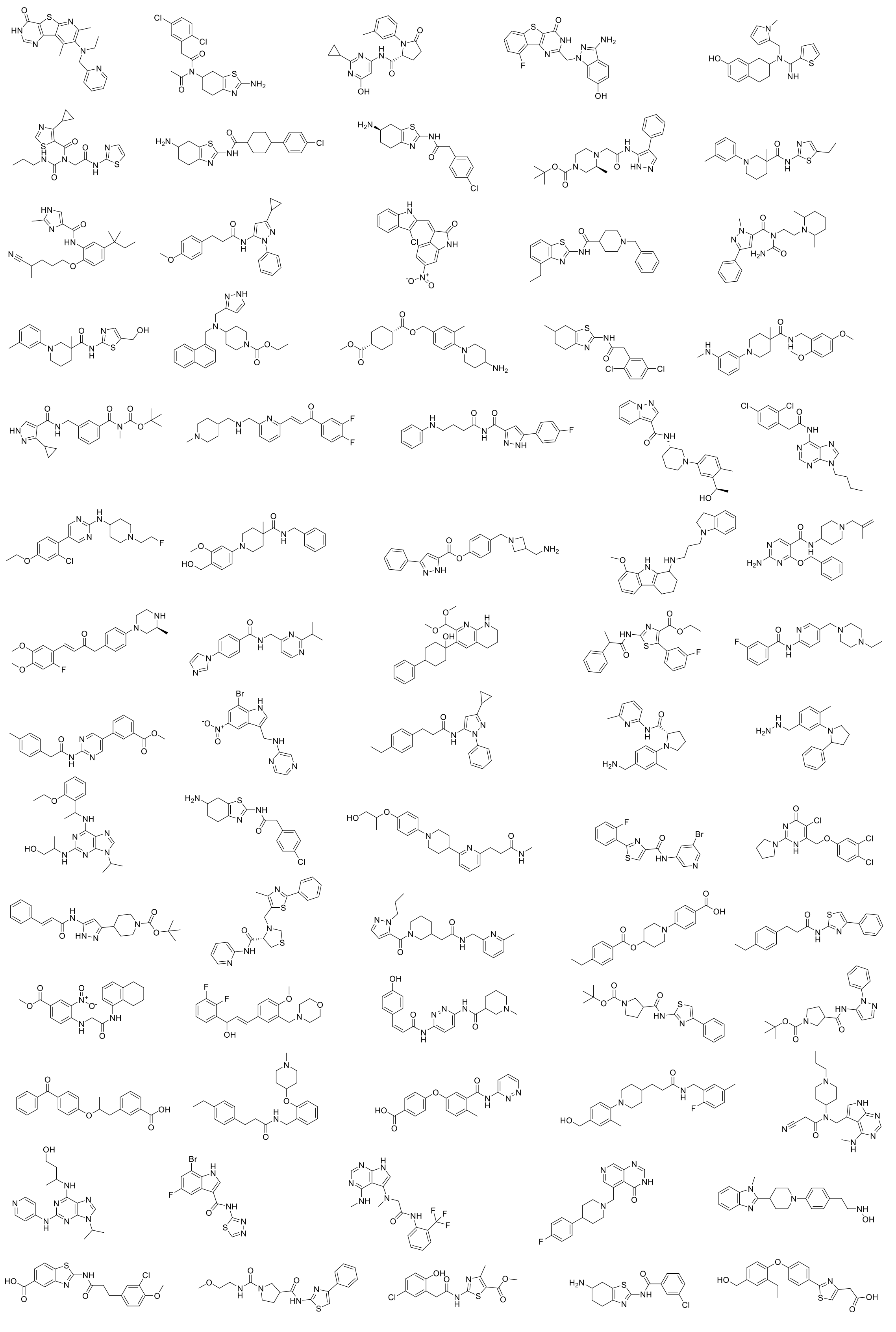

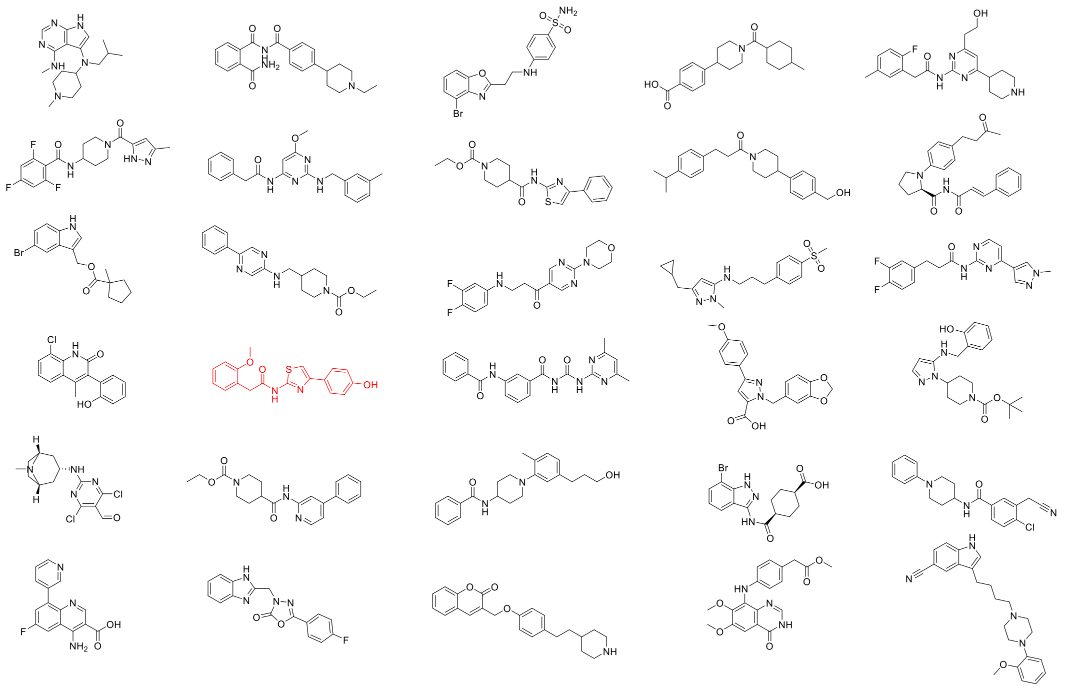

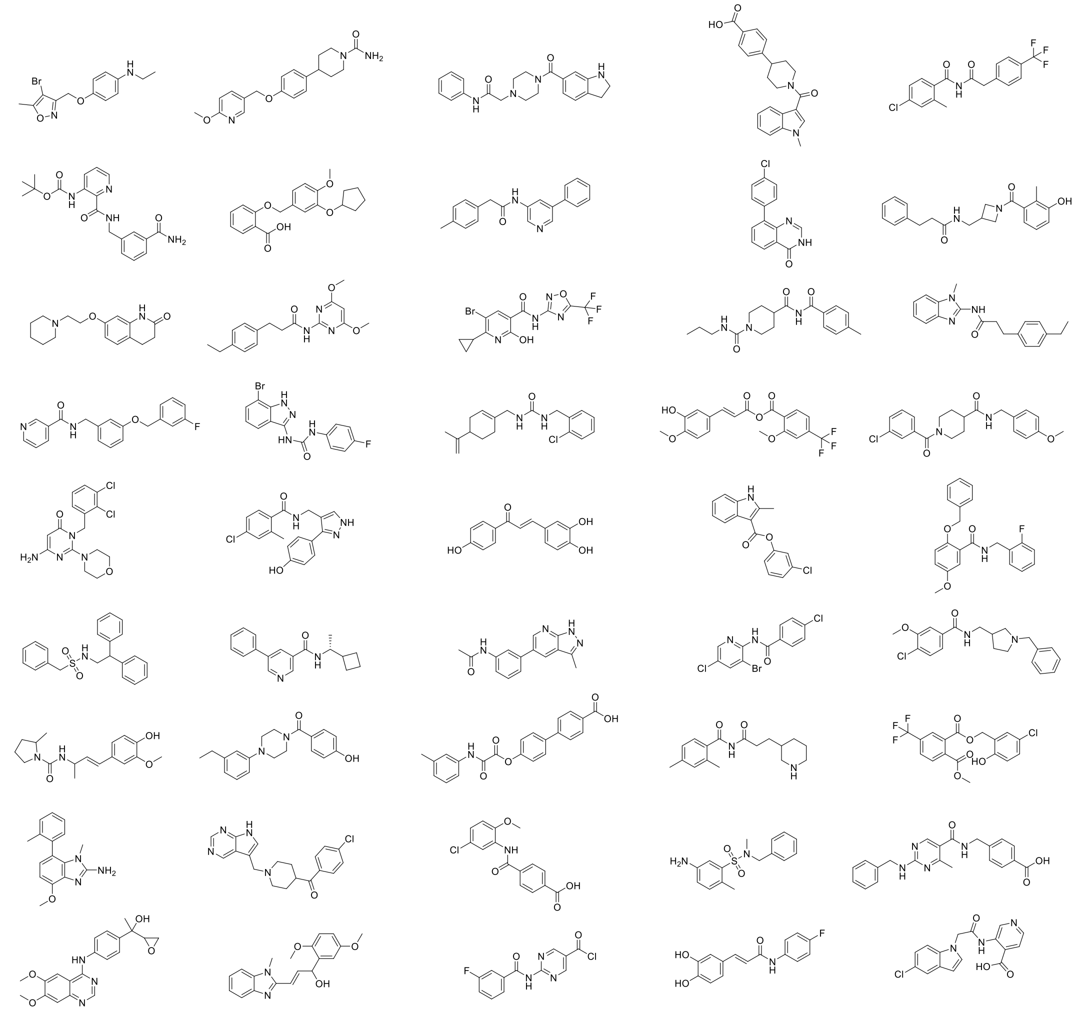

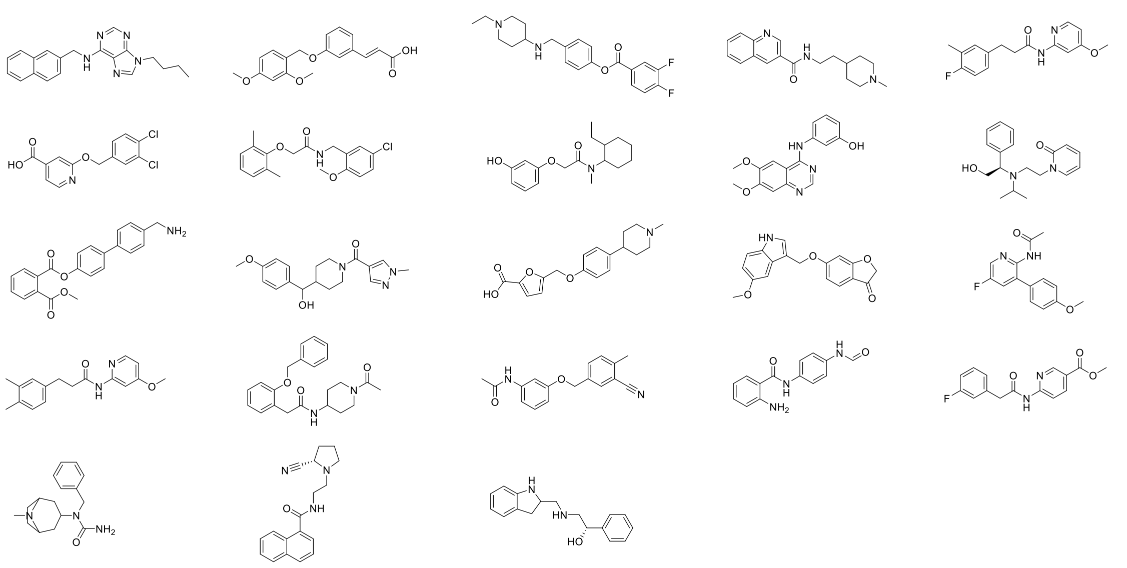

Task15:

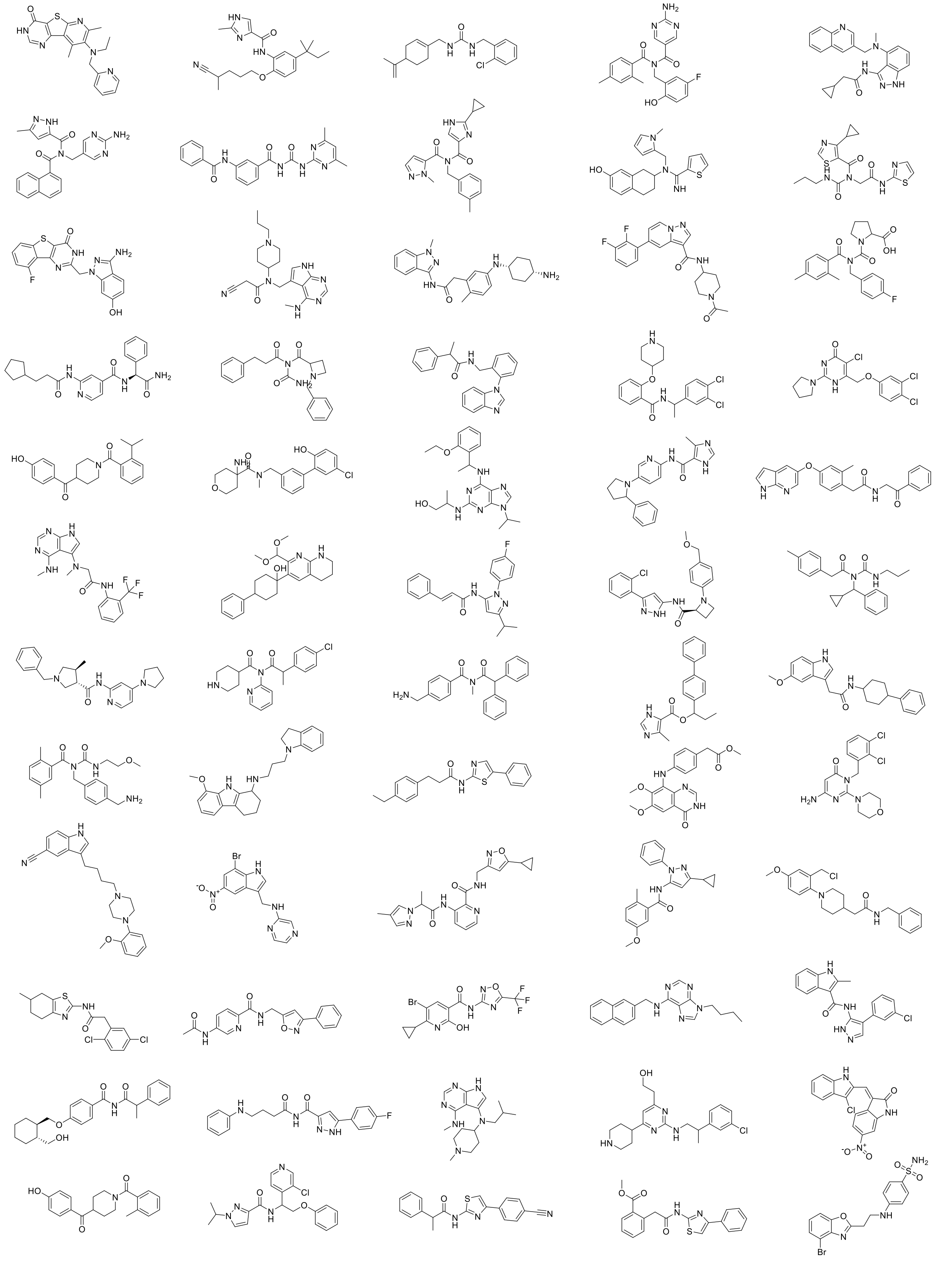

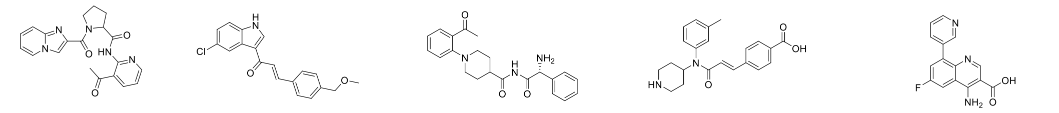
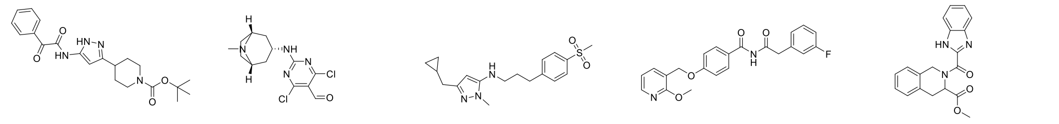
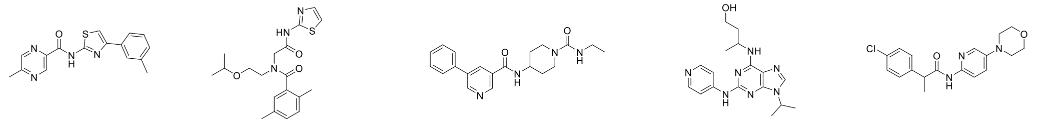
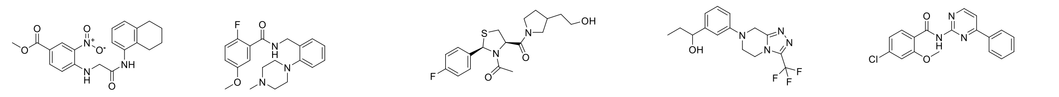
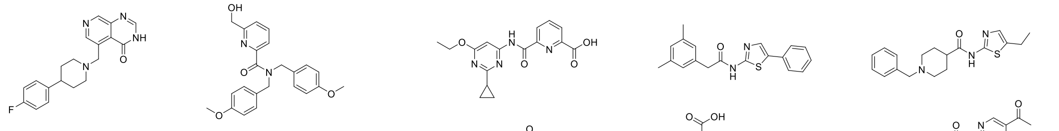
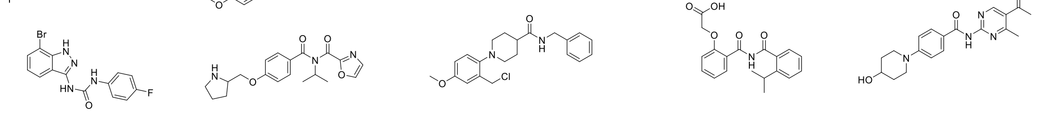
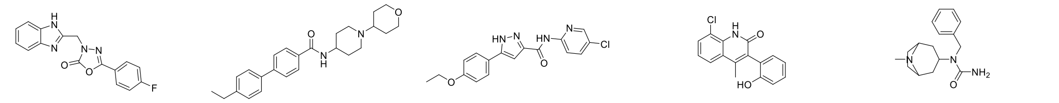
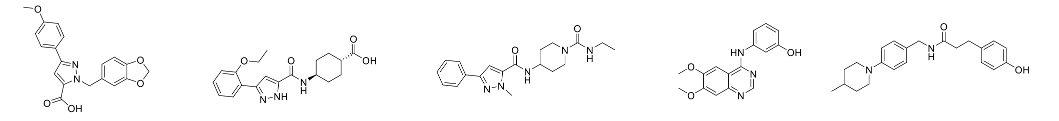
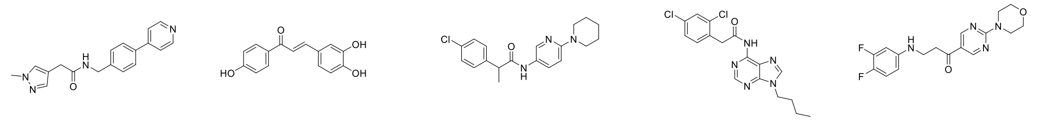
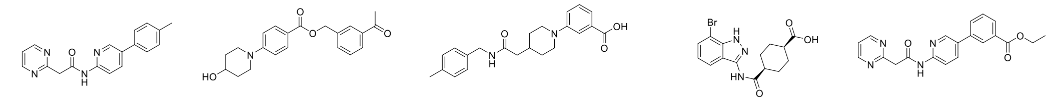
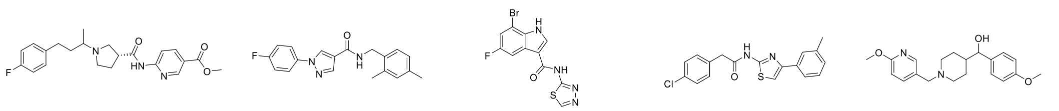
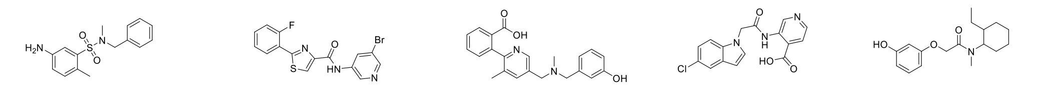
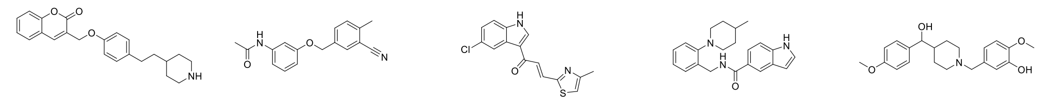
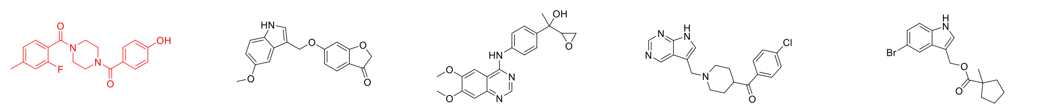
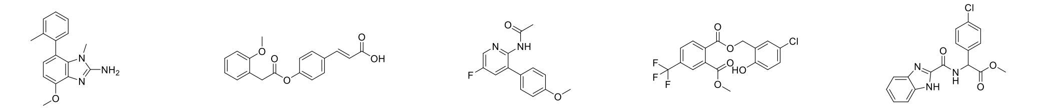

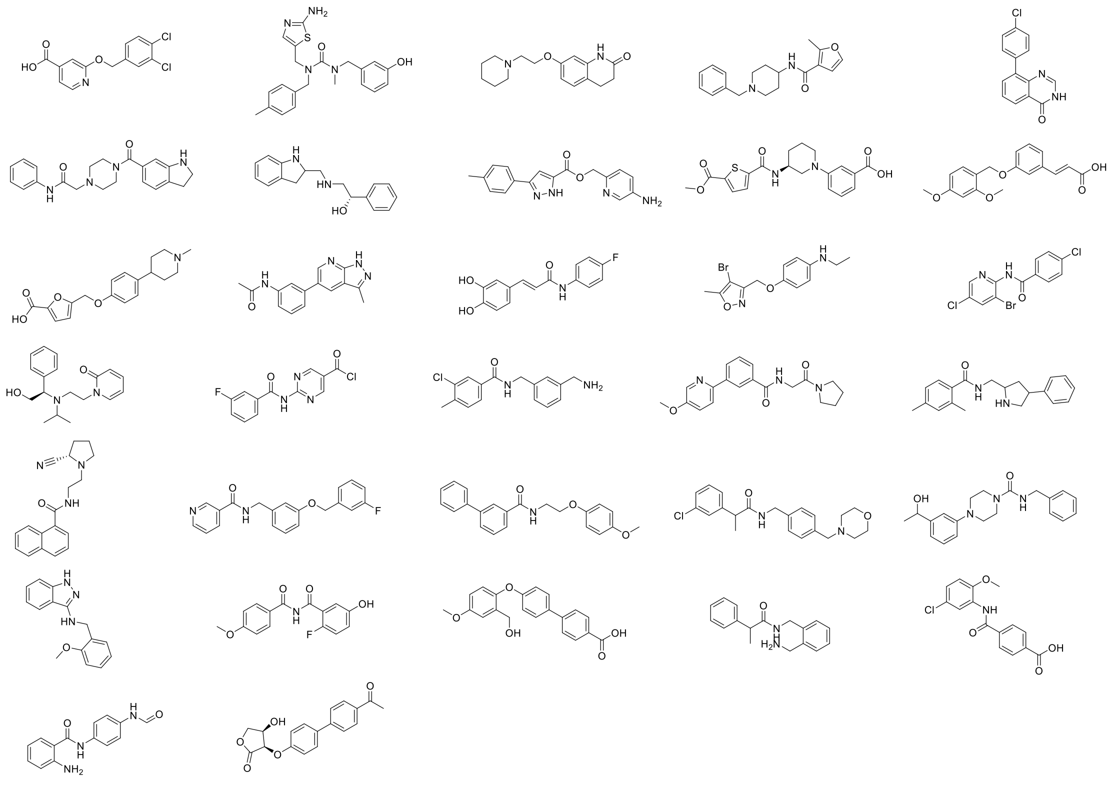

Task16:

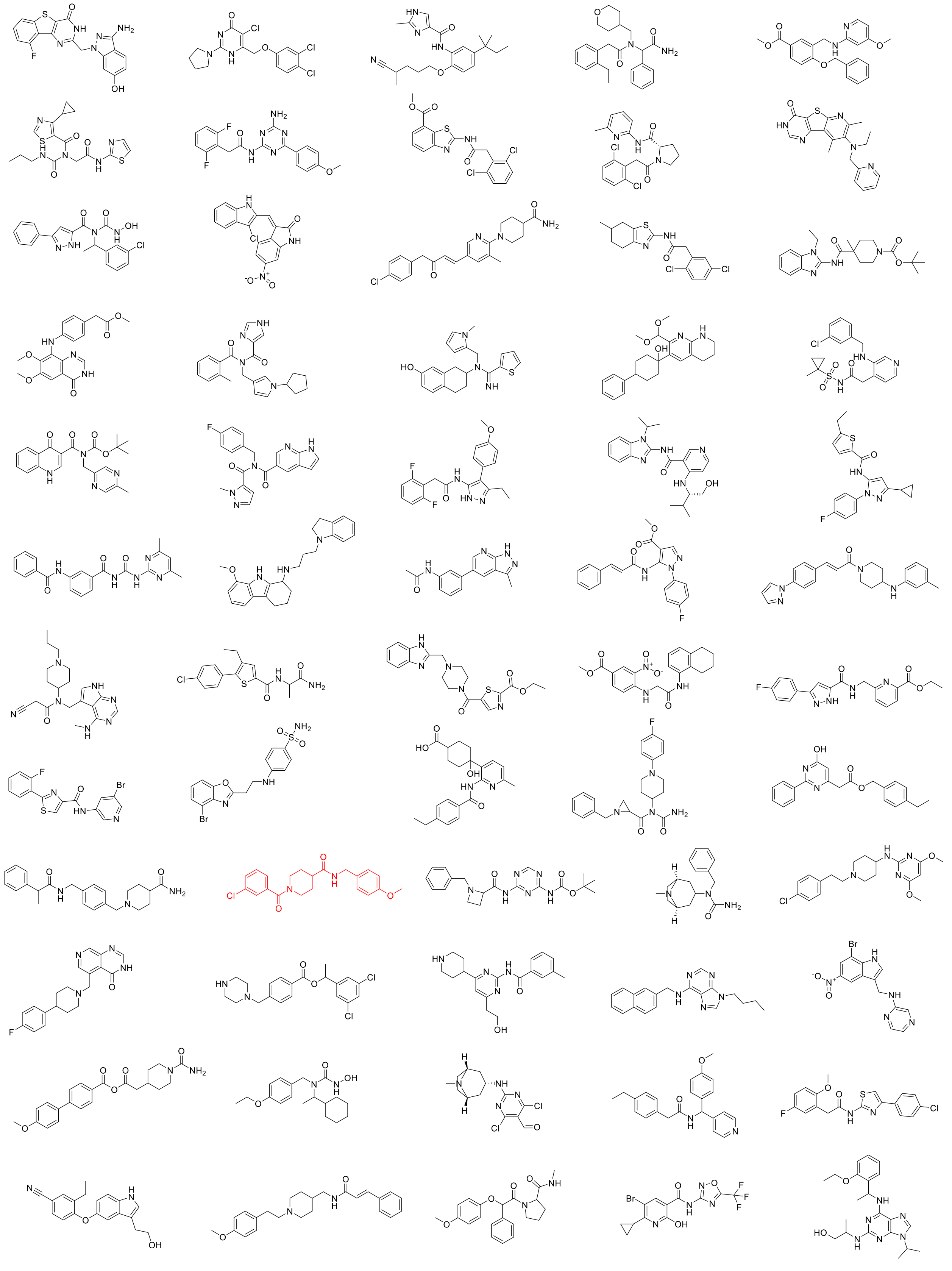

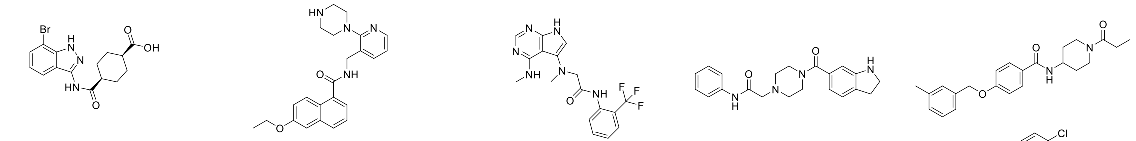
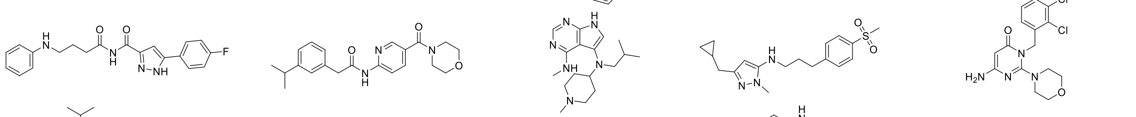
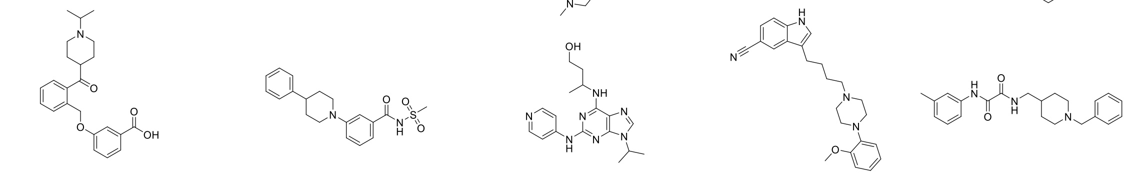
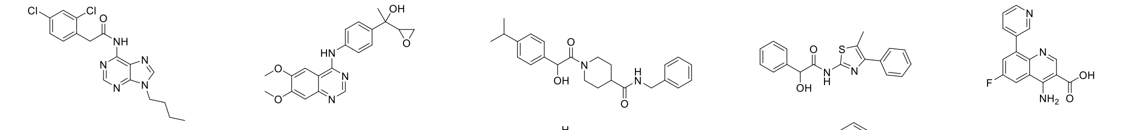
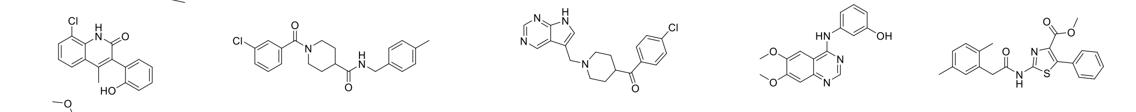
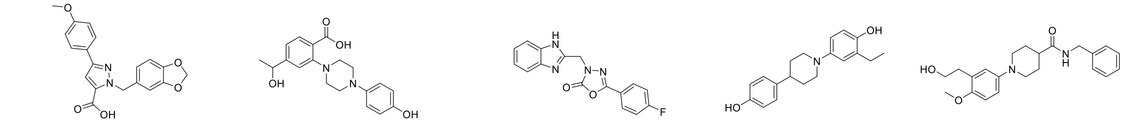
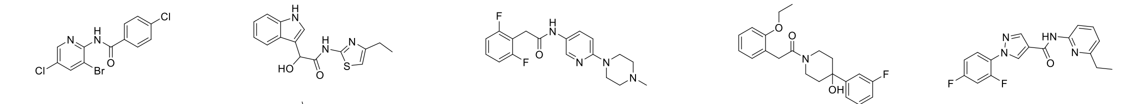
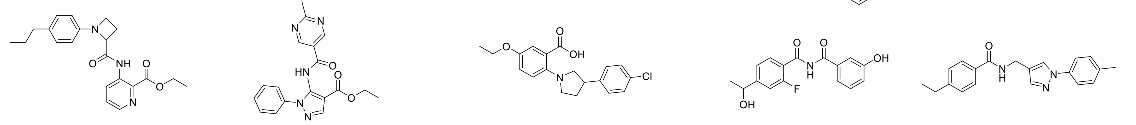
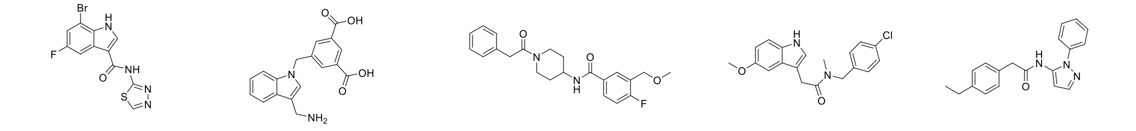
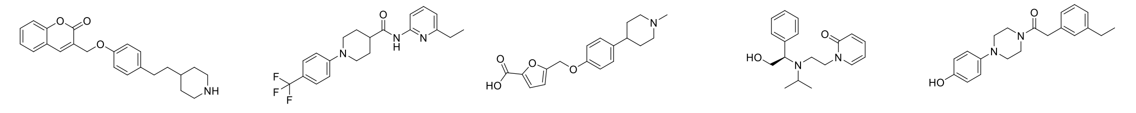
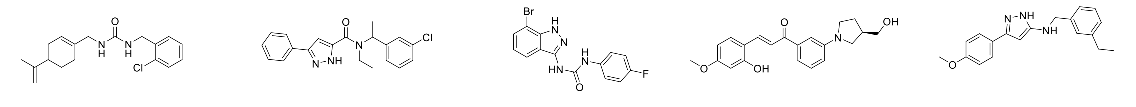
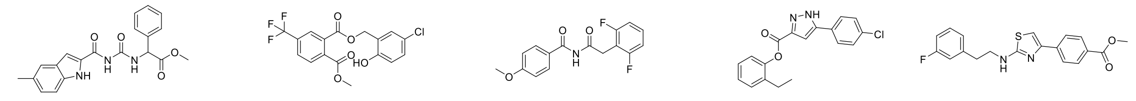
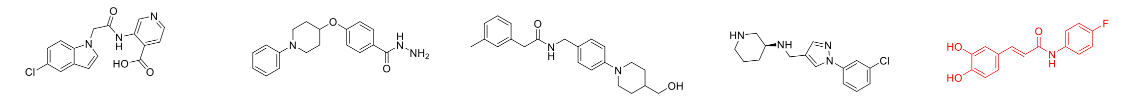
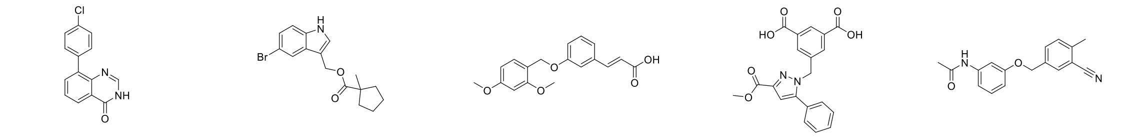

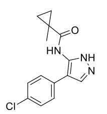
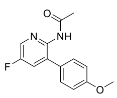
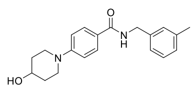
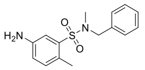
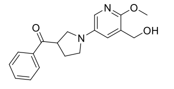
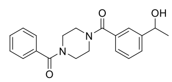
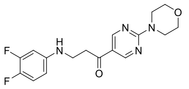
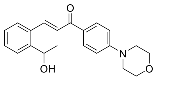
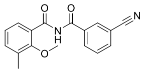
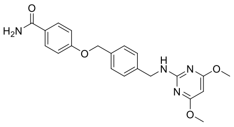
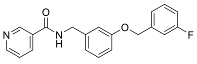
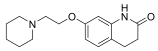
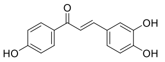
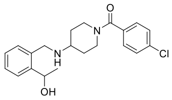
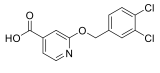
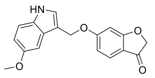
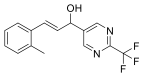
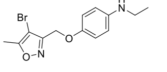
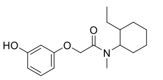
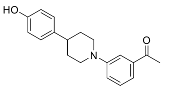
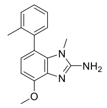
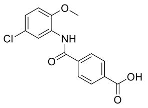
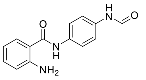
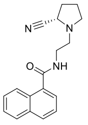
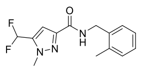
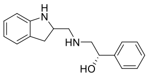
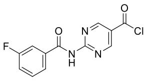

Task17:

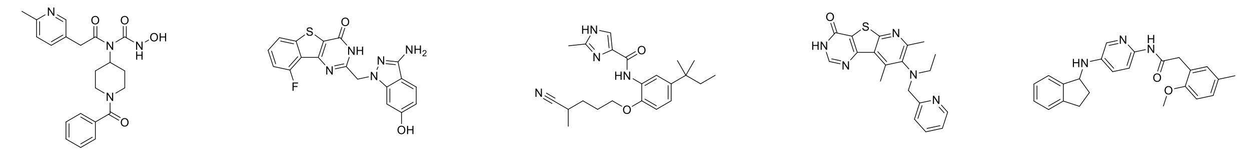

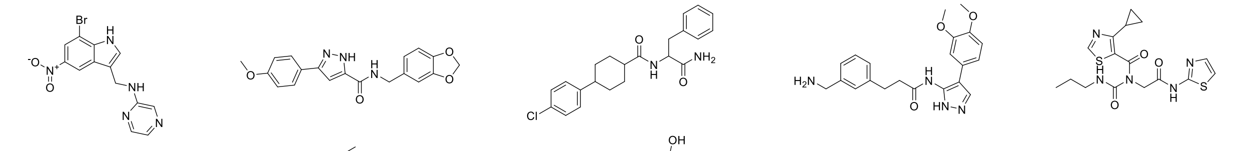

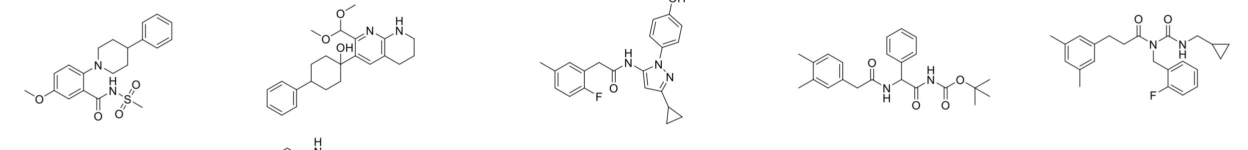

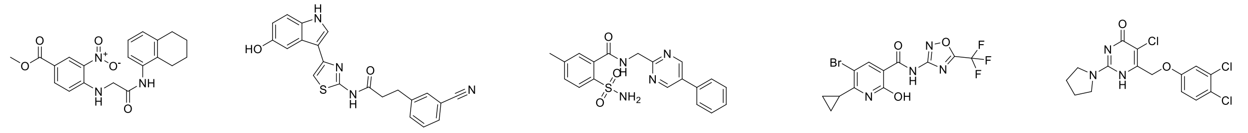

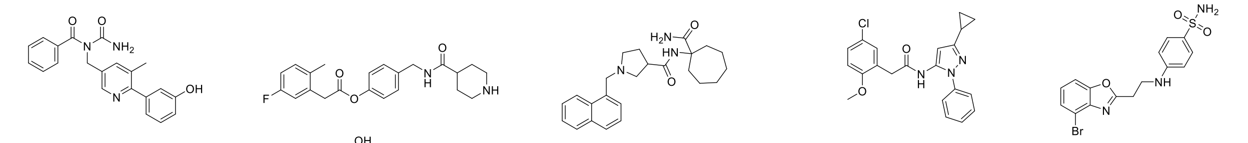

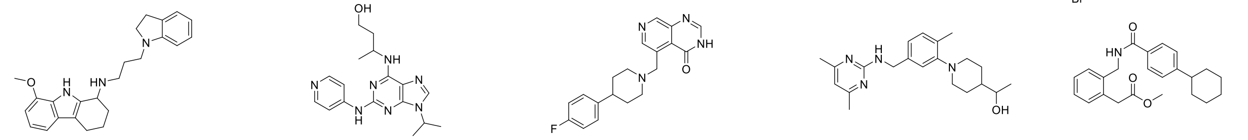

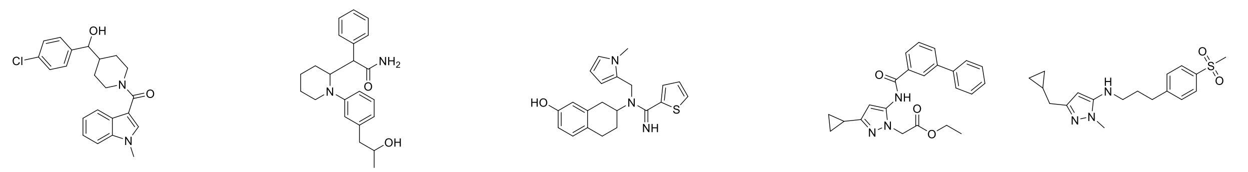

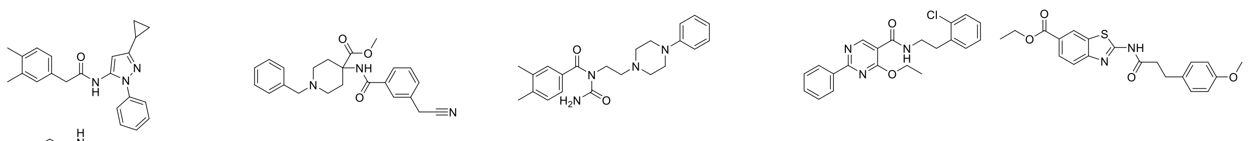

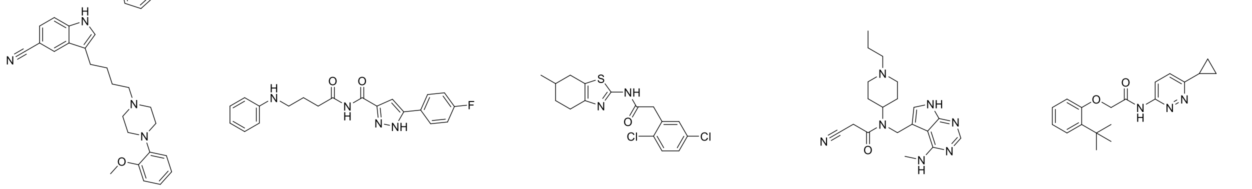

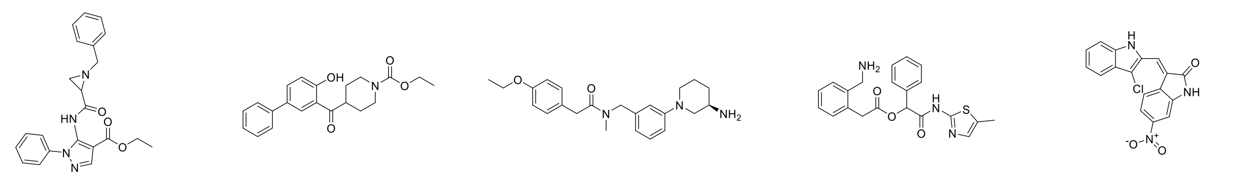

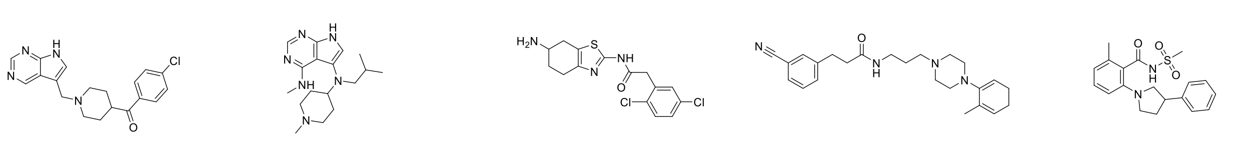

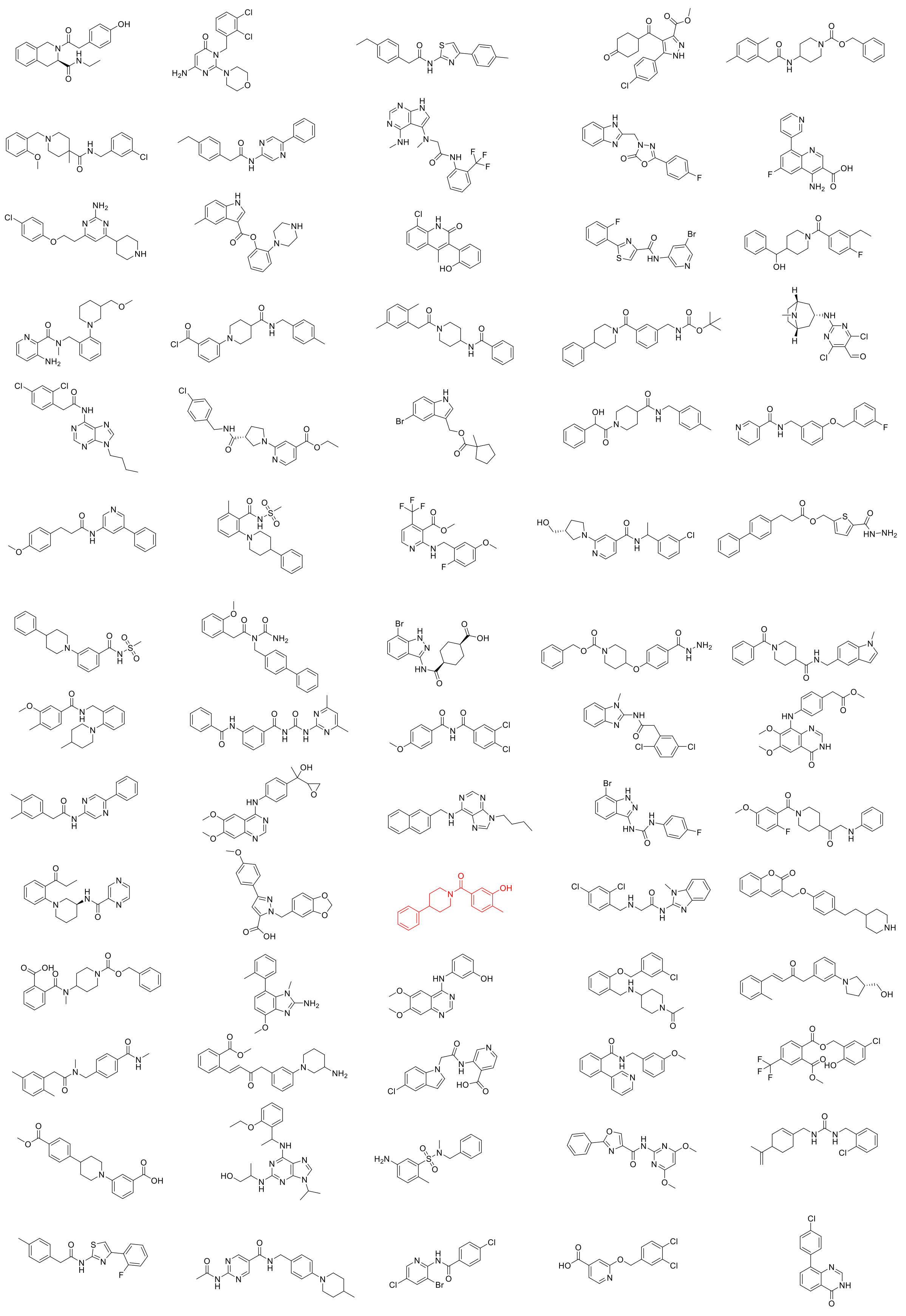

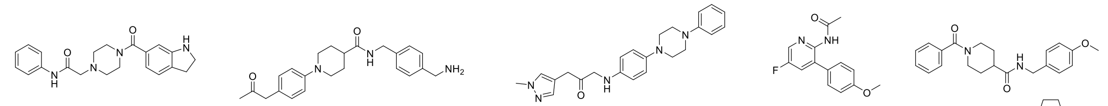
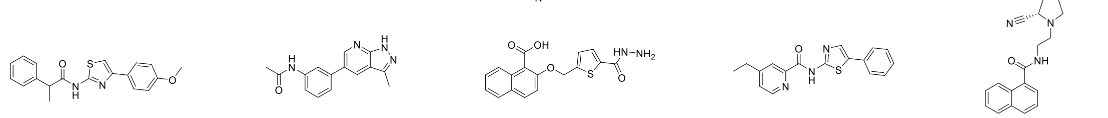
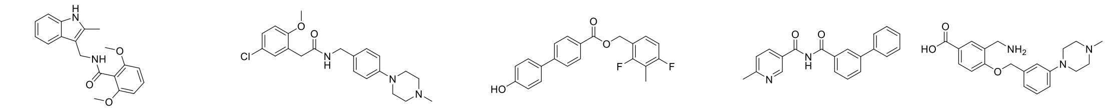
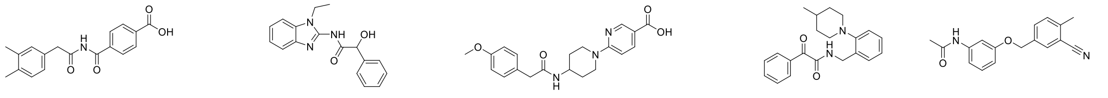
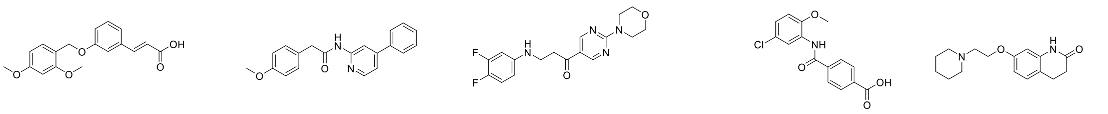
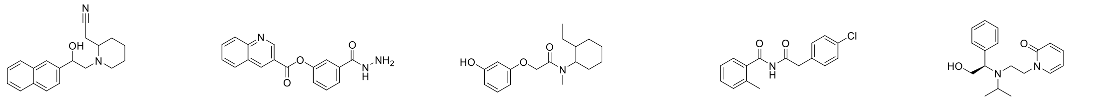
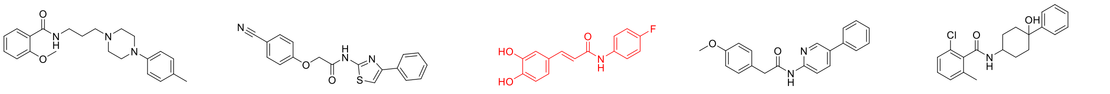
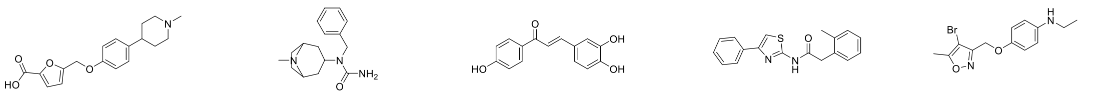
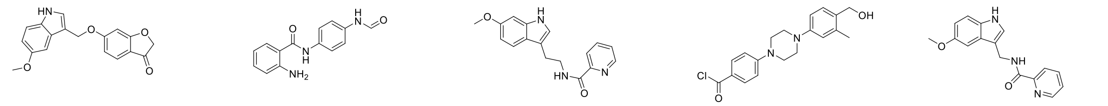
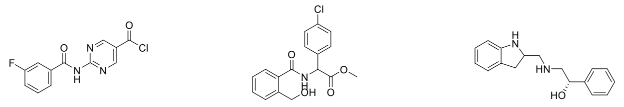

Structures of compounds generated from four AI-driven structural modification tasks on the lead compound AI10

Task1:

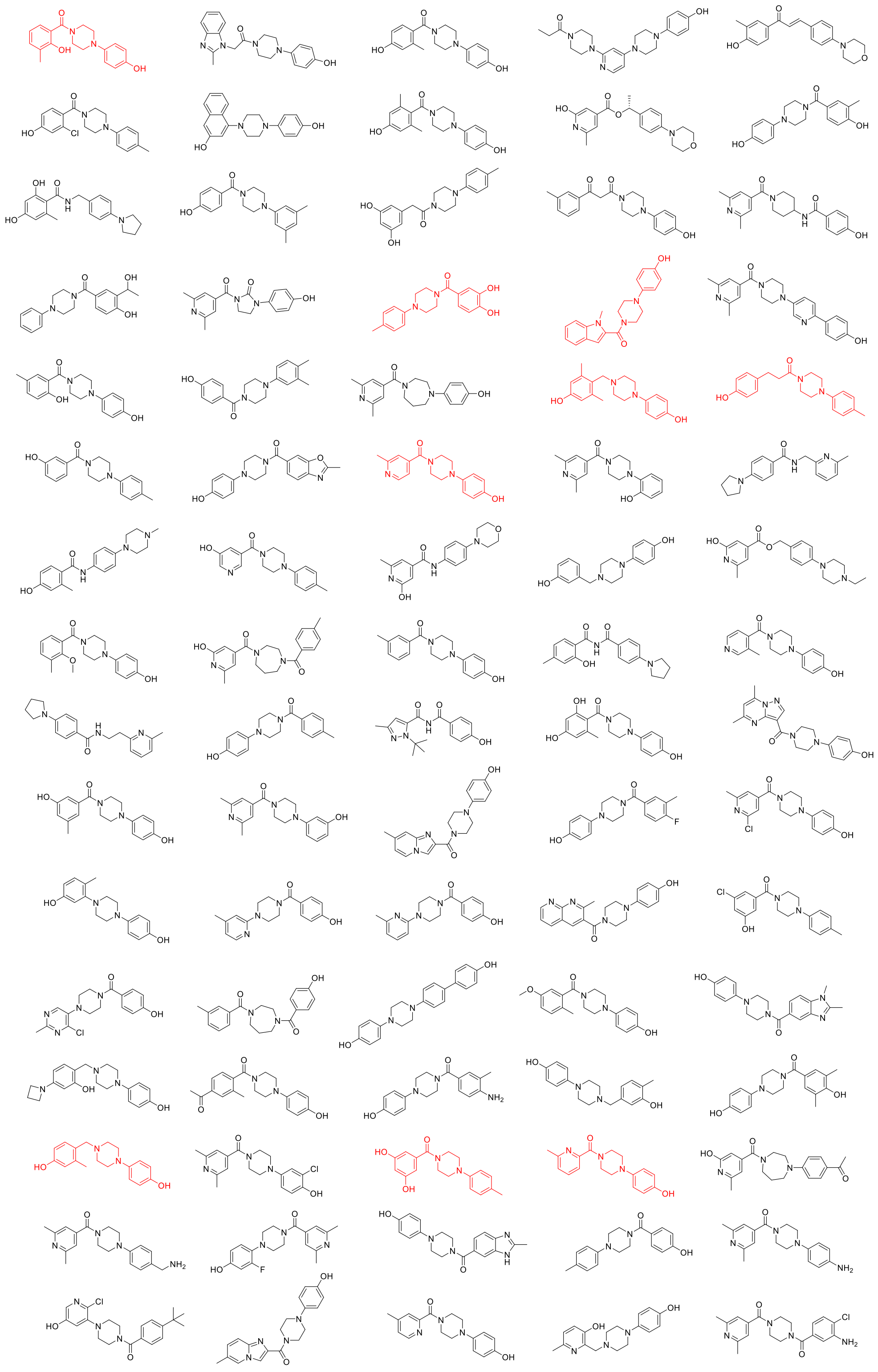

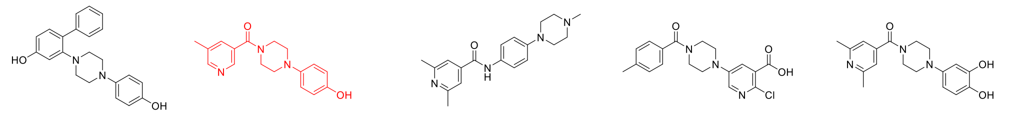
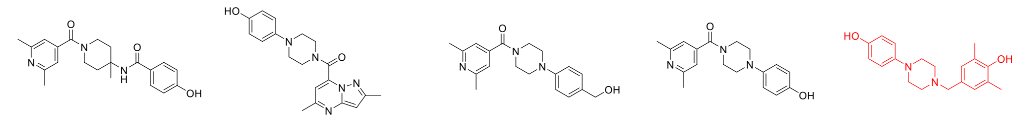
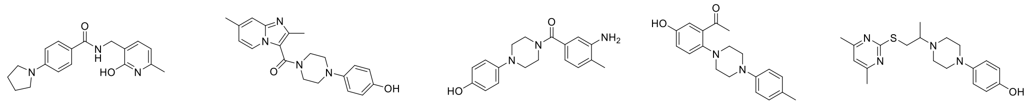
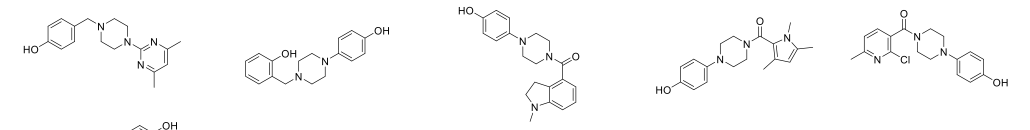
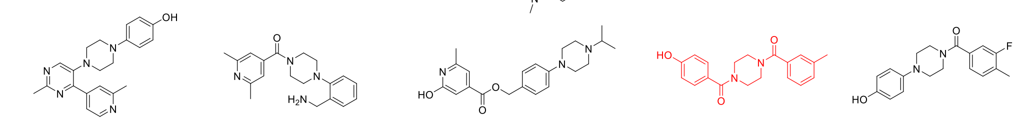
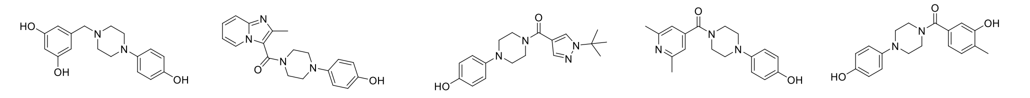
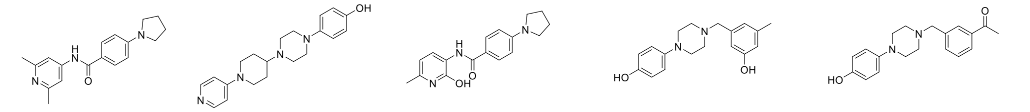
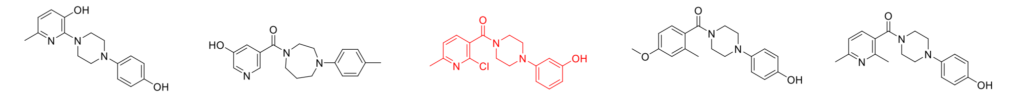
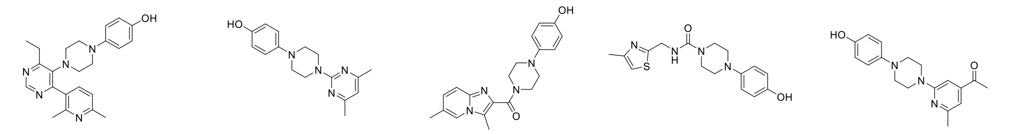
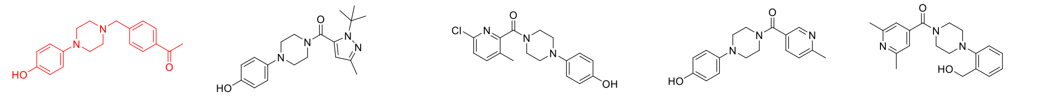
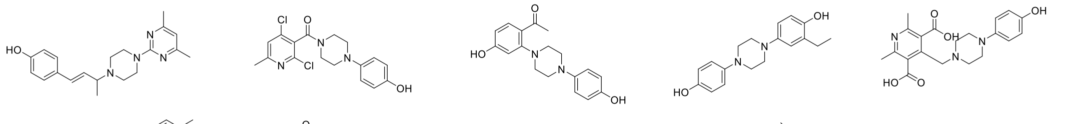
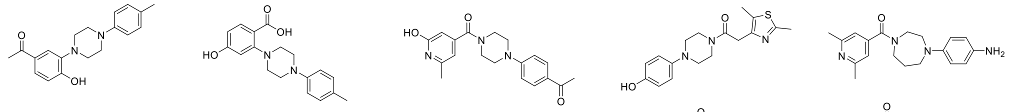
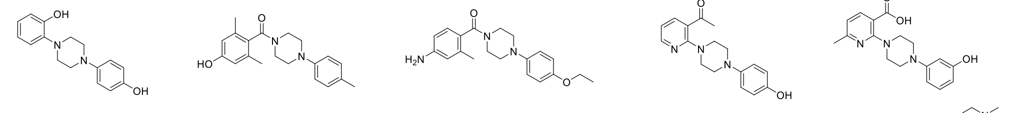
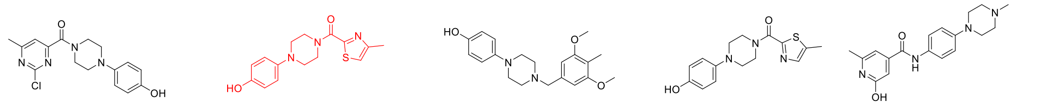
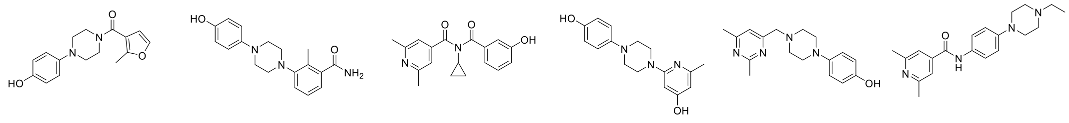

Task 2:

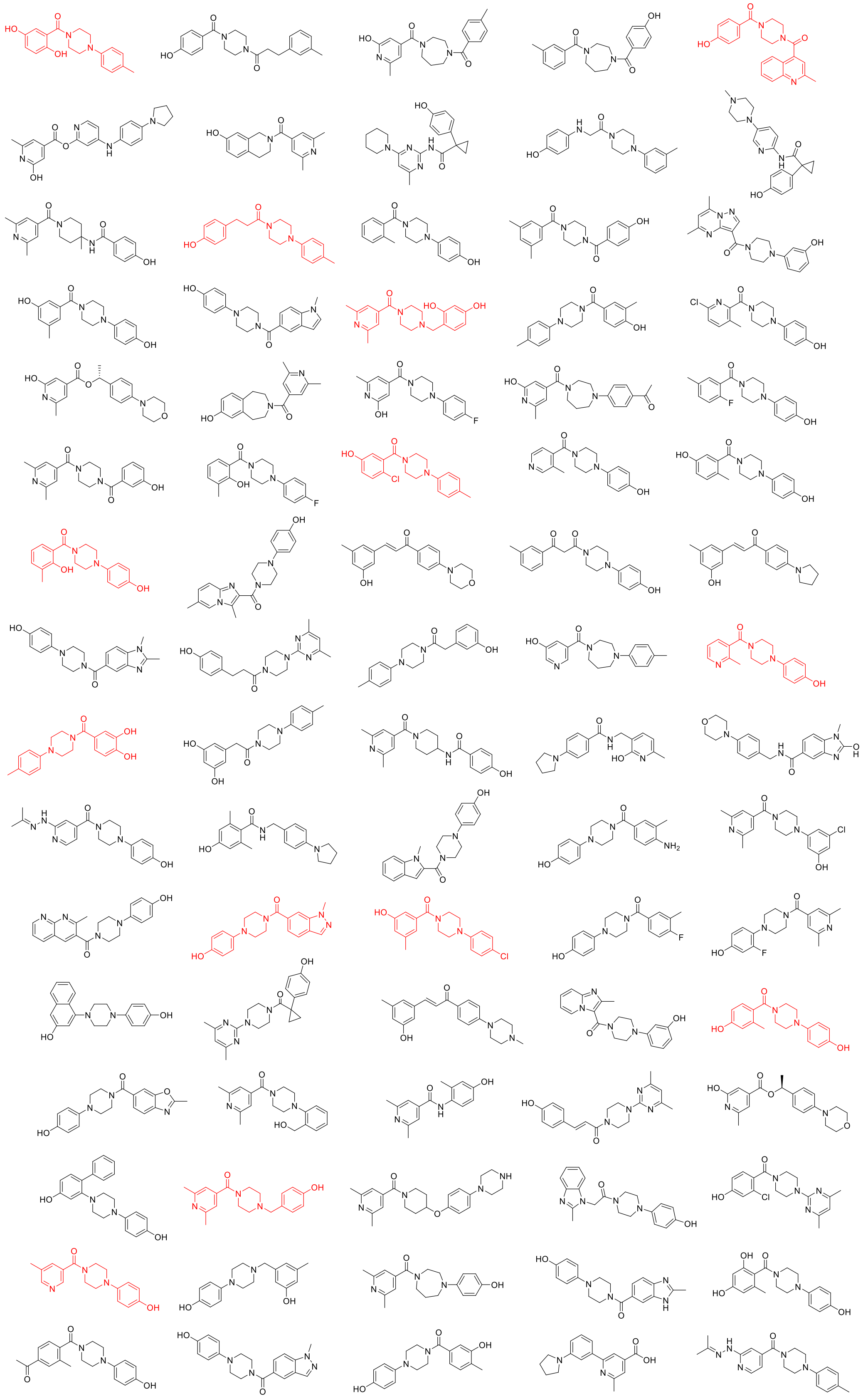

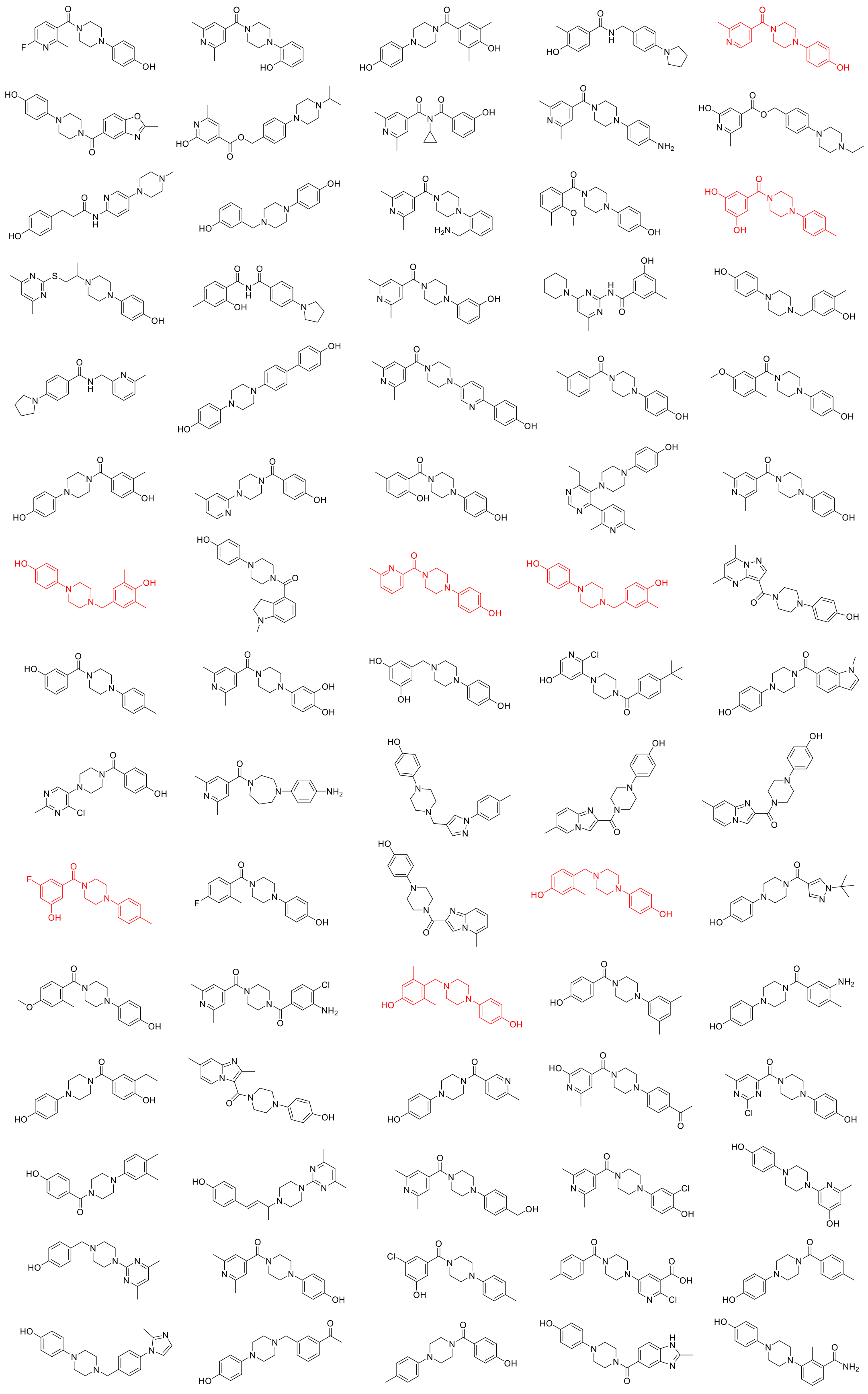

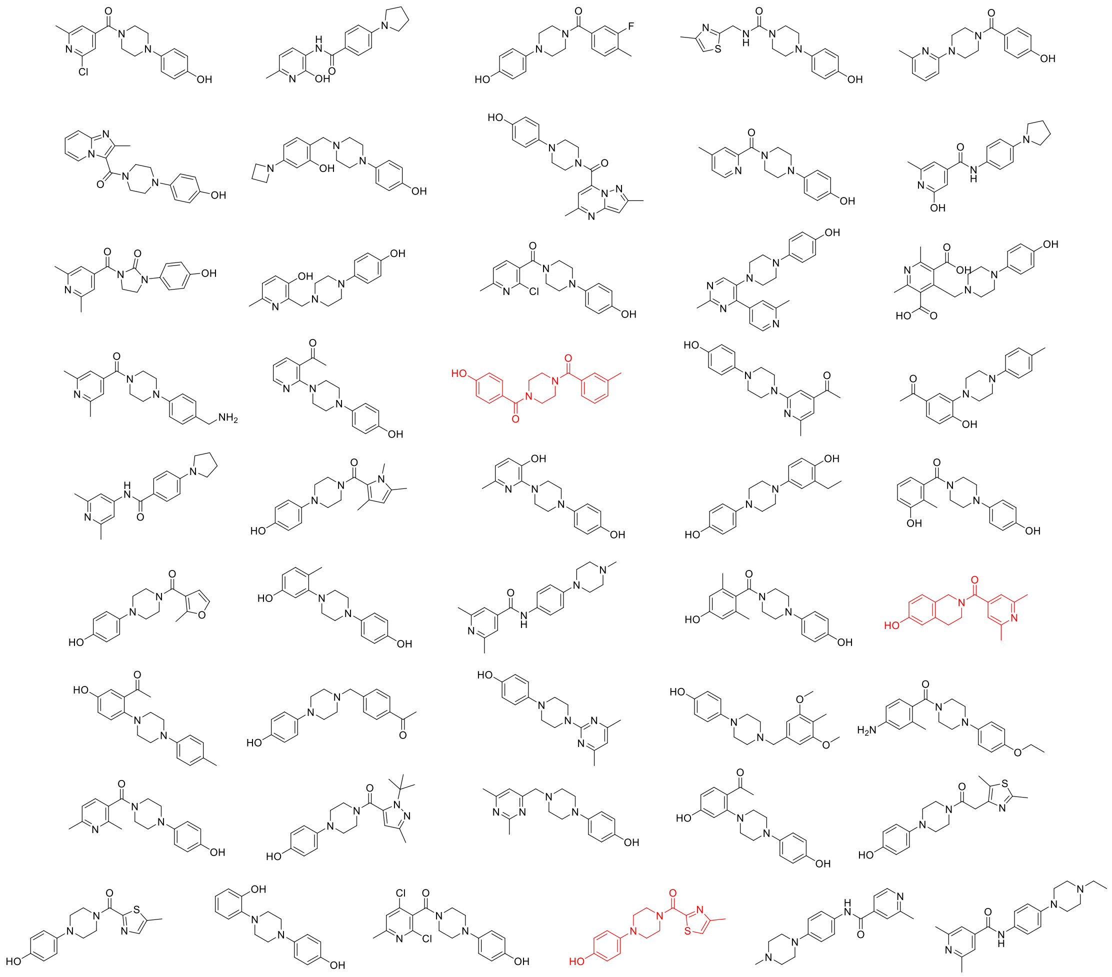

Task 3:

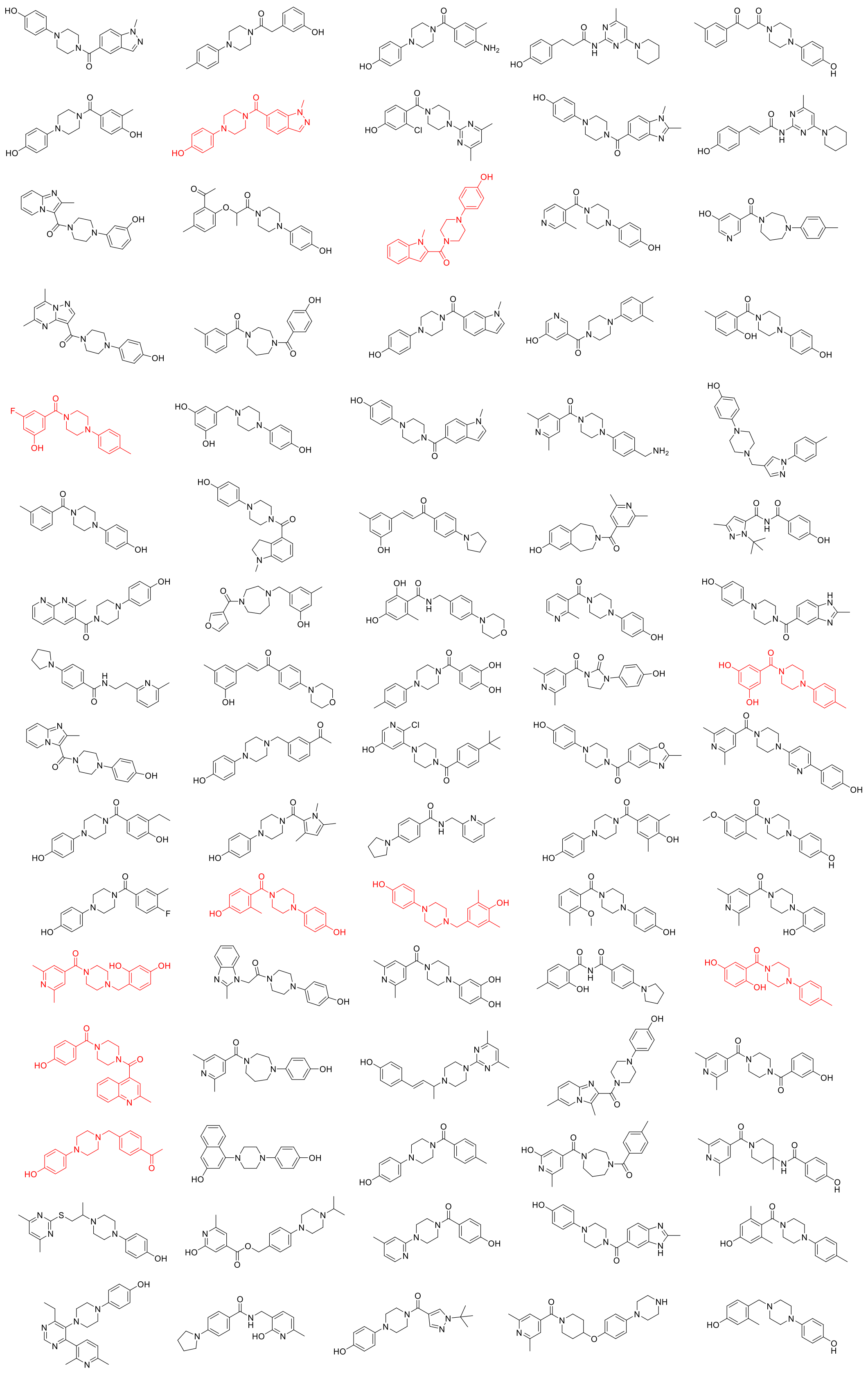

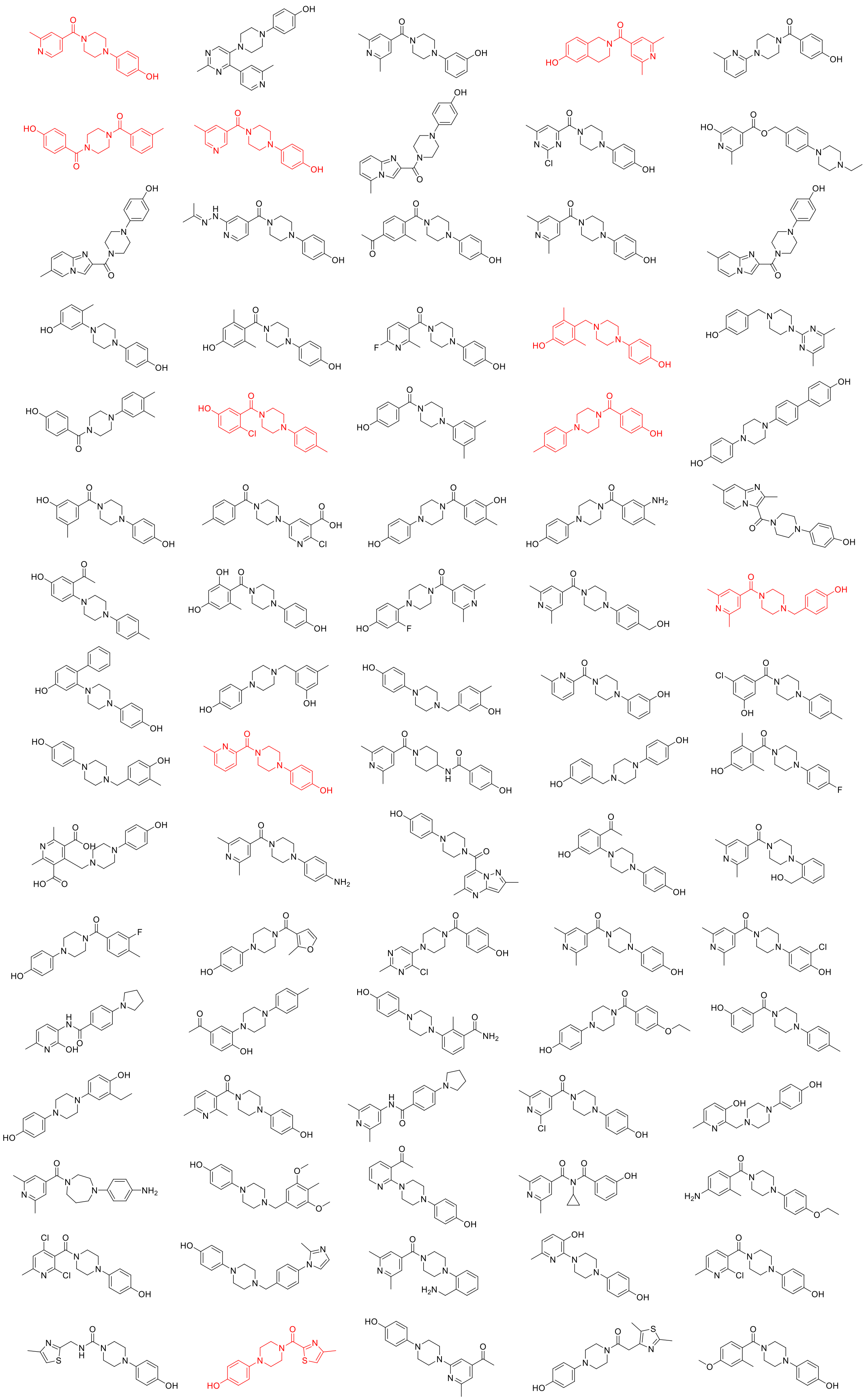

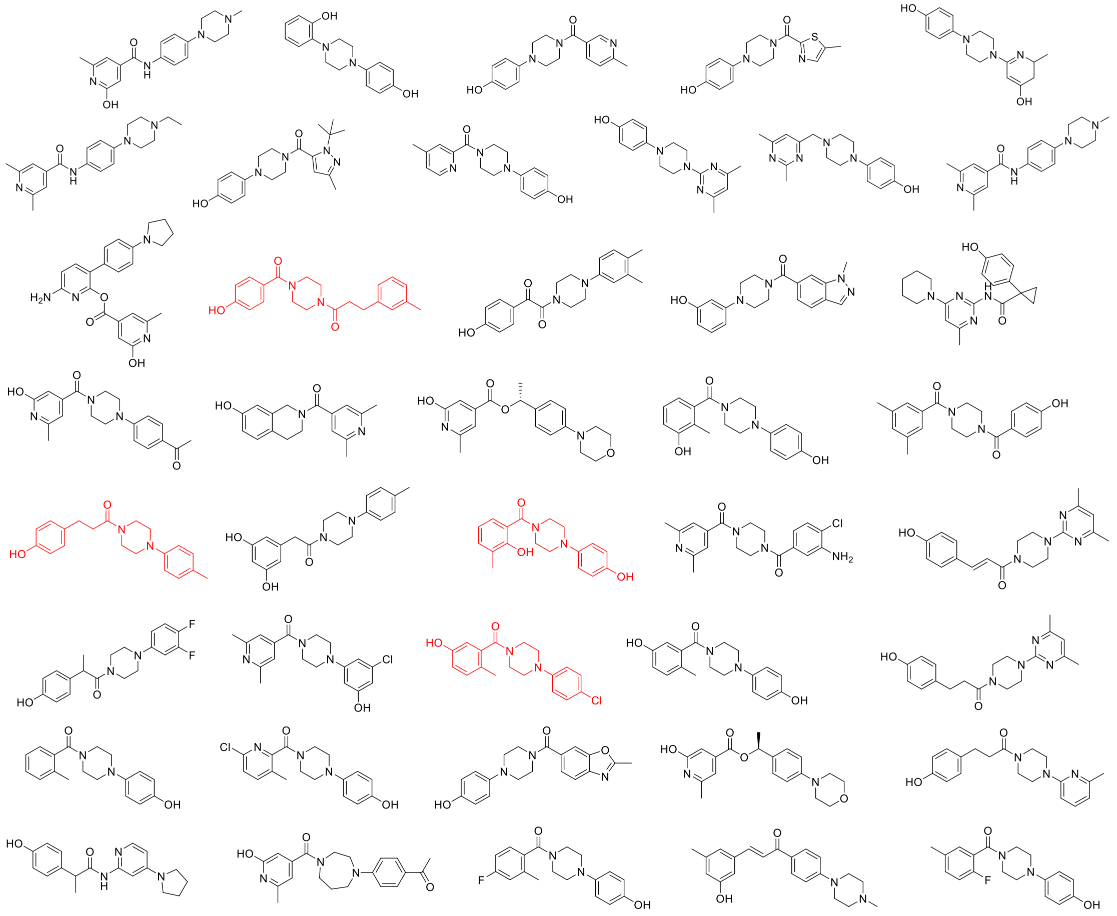

Task 4:

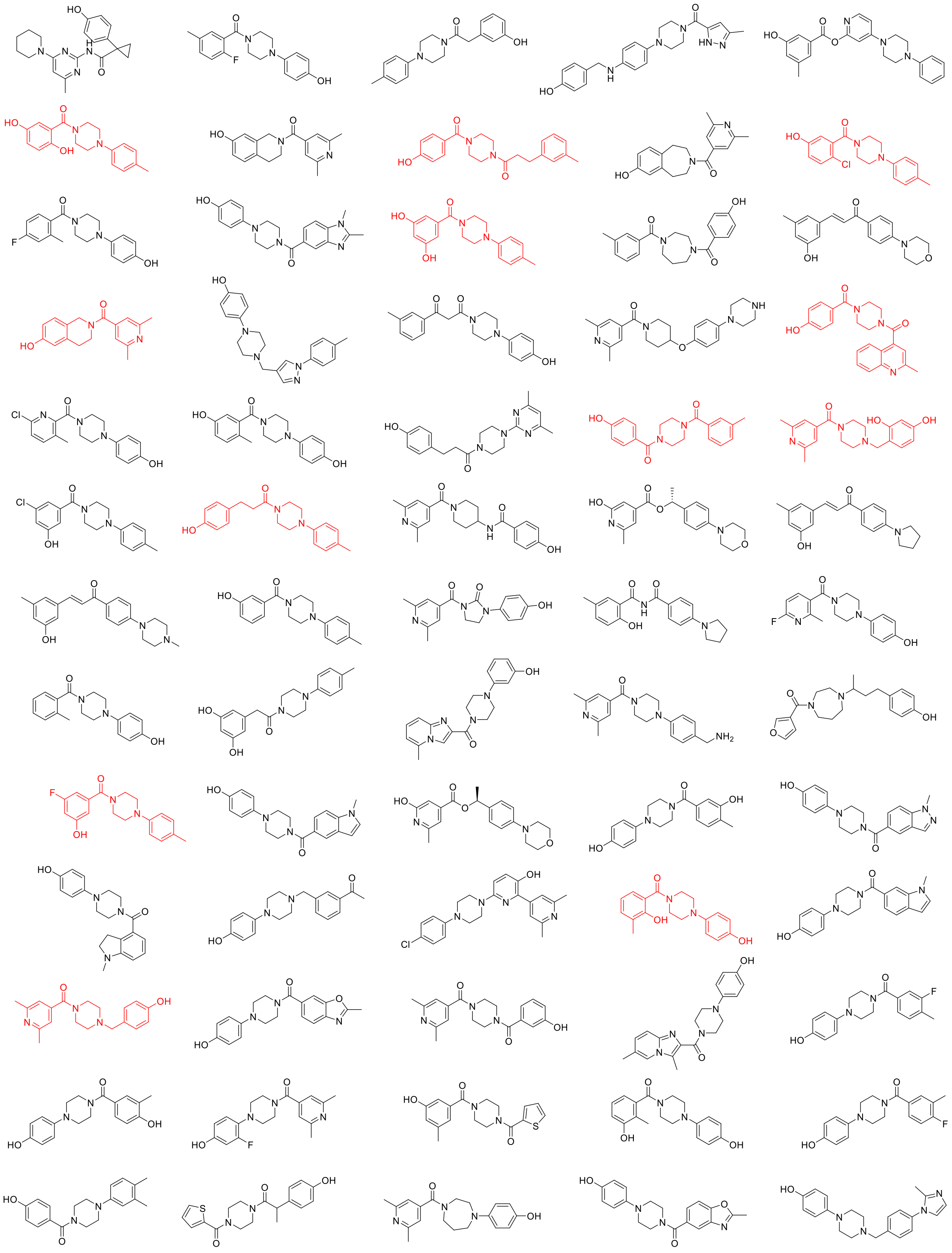

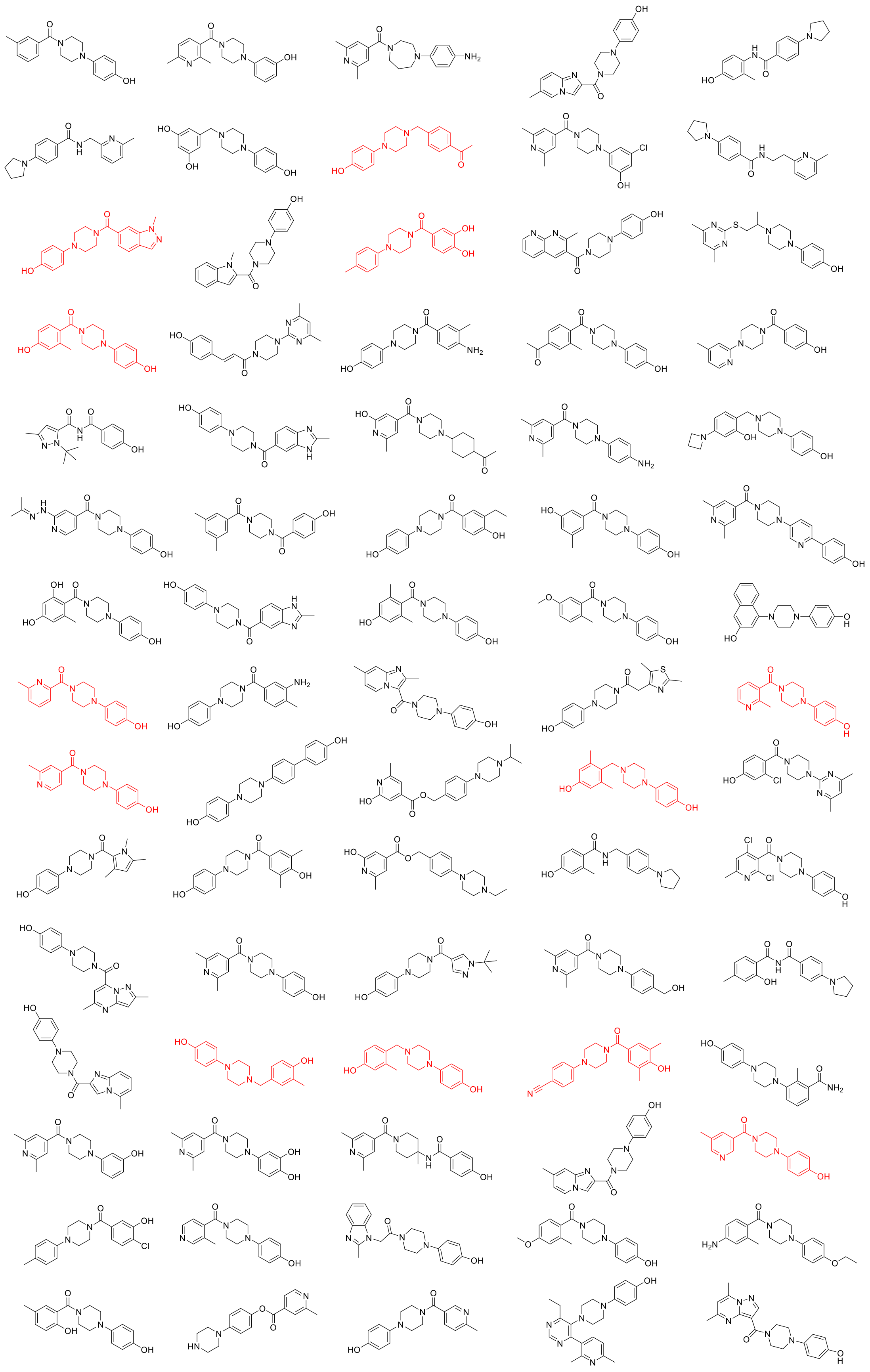

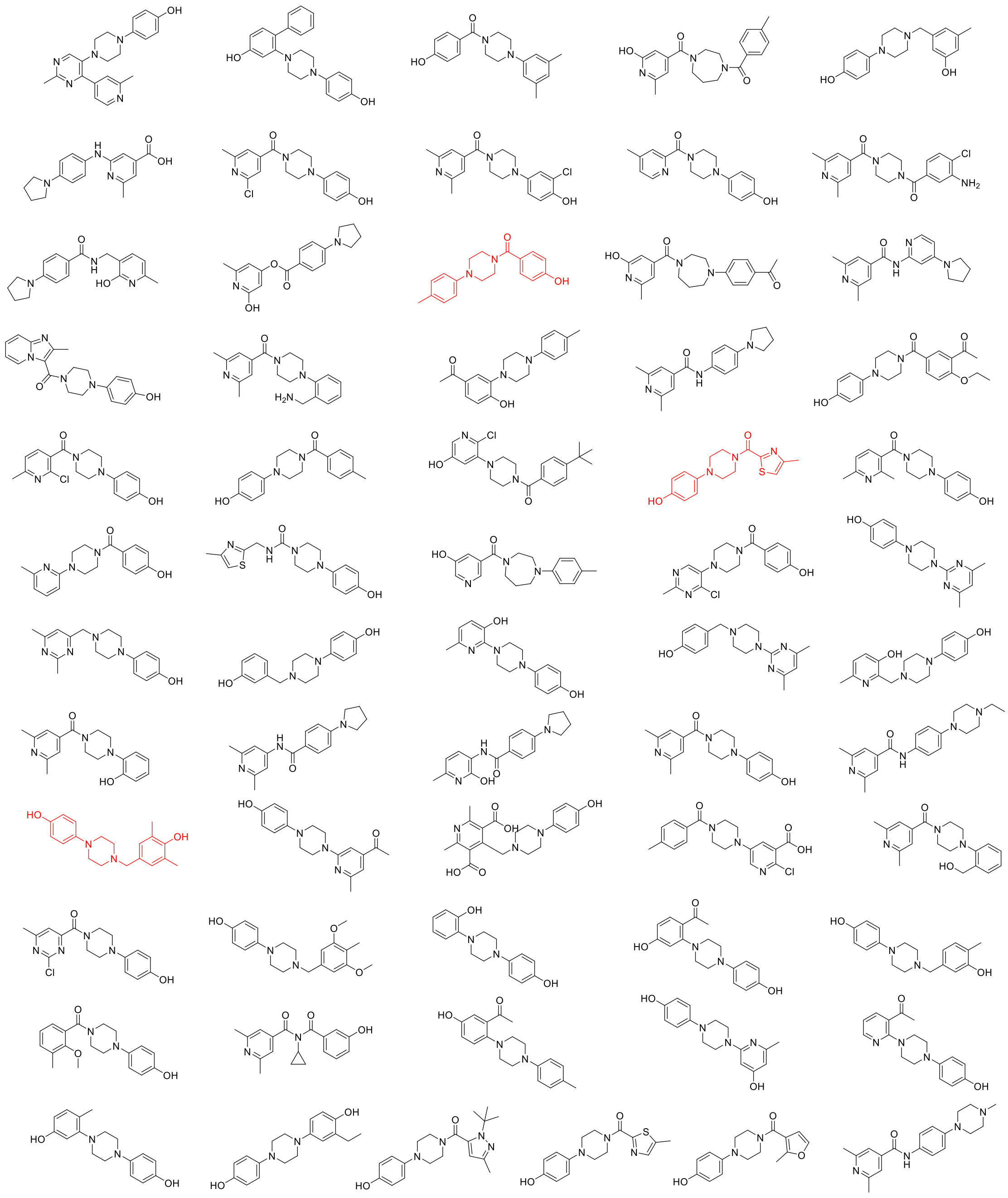

Drug-like properties, bingding sites, synthetic pathway and structural alerts of compounds **AI1-28** and **AI10-a1** to **AI10-a32**

Compounds AI1：

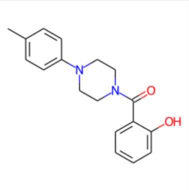

SMILES: Cc1ccc(N2CCN(C(=O)c3ccccc3O)CC2)cc1

San ID	fa6d94ca64427f3fe25e088ef78bfdc3
CAS	No data
InChI	InChI=1S/C18H20N2O2/c1-14-6-8-15(9-7-14)19-10-12-20(13-11-19)18(22)16-4-2-3-5-17(16)21/h2-9,21H,10-13H2,1H3
InChIKey	HAGDKOYHGJXWOV-UHFFFAOYSA-N

View in third-party databases: PubChem

MW	296.15	HBA	3	HBD	1
PAINS Alert	true	QED	0.92	NRot	2
TPSA	43.78	logD	3.13	logP	3.84
logS	-3.83				

Bad Average Good Scoring Notice

Absorption

Caco2	-4.75	HIA	100.00%	P-gp Inhibitor	84.11%
Bioavailability	99.96%	HFE	-8.38		

Distribution

BBB Penetration	86.96%	PPBR	1.00%	VDSS	2.25

Metabolism

CYP2D6 Inhibitor	8.02%	CYP3A4 Inhibitor	74.10%	CYP2C9 Inhibitor	78.16%
CYP2D6 Substrate	99.53%	CYP3A4 Substrate	47.03%	CYP2C9 Substrate	9.90%
CYP2C19 Inhibitor	87.13%	CYP1A2 Inhibitor	7.44%		

Excretion

Half Life (hour)	3.46	Clearance (mL/min/g)	49.57

Tox

hERG	96.04%	DILI	7.88%	NR-AhR	17.85%
Carcinogencity	27.51%	LD_{50}	543.98	Respiratory Toxicity	16.78%
Eye Corrosion	0.11%				

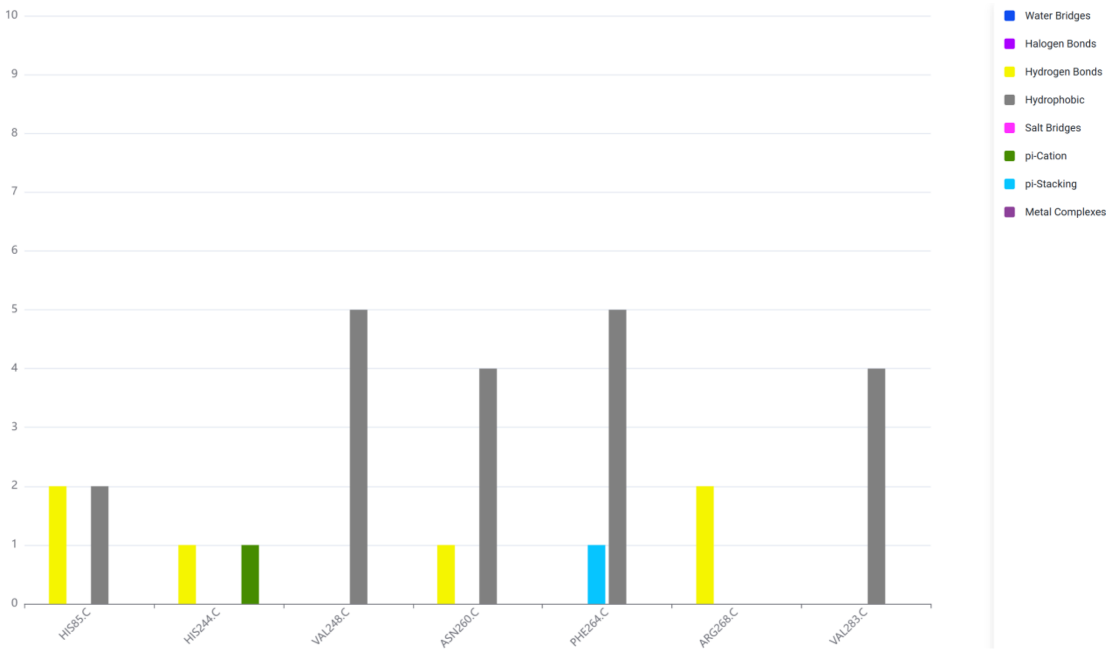

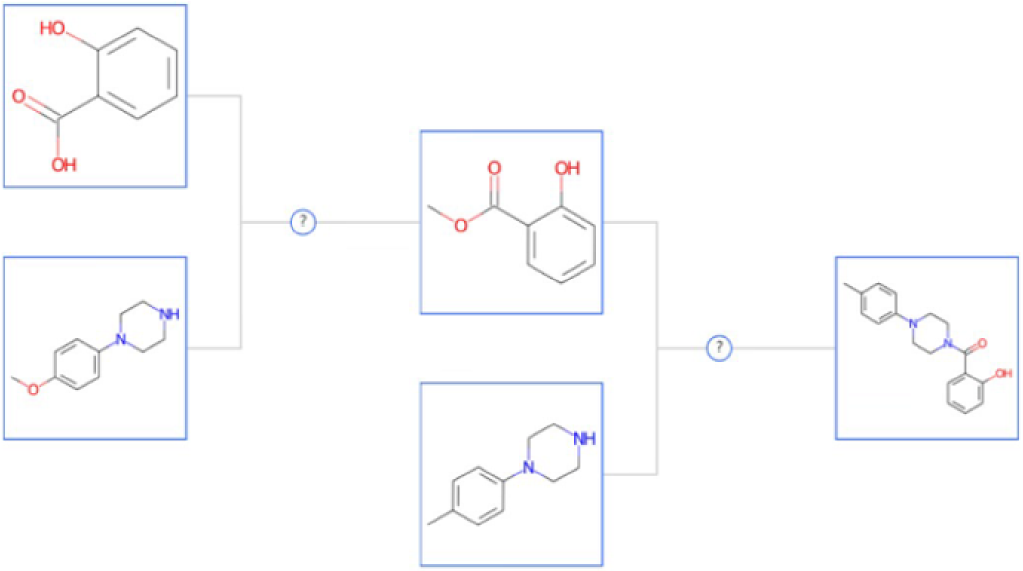

Alert Rule	Alert Structure	Reference
BMS Rule	✓	1. [1] Huth J R, Mendoza R, Olejniczak E T, et al. ALARM NMR: a rapid and robust experimental method to detect reactive false positives in biochemical screens[J]. Journal of the American Chemical Society, 2005, 127(1): 217-224.
Chelator Rule	✓	1. [1] Agrawal A, Johnson S L, Jacobsen J A, et al. Chelator fragment libraries for targeting metalloproteinases[J]. ChemMedChem: Chemistry Enabling Drug Discovery, 2010, 5(2): 195-199.
PAINS	✓	1. [1] Baell J B, Holloway G A. New substructure filters for removal of pan assay interference compounds (PAINS) from screening libraries and for their exclusion in bioassays[J]. Journal of medicinal chemistry, 2010, 53(7): 2719-2740.
Genotoxic Carcinogenicity Rule	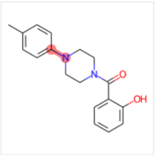	1. [1] Benigni R, Bossa C. Structure alerts for carcinogenicity, and the Salmonella assay system: a novel insight through the chemical relational databases technology[J]. Mutation Research/Reviews in Mutation Research, 2008, 659(3): 248-261. 2. [2] Ashby J, Tennant R W. Chemical structure, Salmonella mutagenicity and extent of carcinogenicity as indicators of genotoxic carcinogenesis among 222 chemicals tested in rodents by the US NCI/NTP[J]. Mutation Research/Genetic Toxicology, 1988, 204(1): 17-115. 3. [3] Kazius J, McGuire R, Bursi R. Derivation and validation of toxicophores for mutagenicity prediction[J]. Journal of medicinal chemistry, 2005, 48(1): 312-320. 4. [4] Bailey A B, Chanderbhan R, Collazo-Braier N, et al. The use of structure-activity relationship analysis in the food contact notification program[J]. Regulatory Toxicology and Pharmacology, 2005, 42(2): 225-235.
NTD	✓	1. [1] Brenk R, Schipani A, James D, et al. Lessons learnt from assembling screening libraries for drug discovery for neglected diseases[J]. ChemMedChem: Chemistry Enabling Drug Discovery, 2008, 3(3): 435-444.
SureChEMBL Rule	✓	1. [1] Sushko I, Salmina E, Potemkin V A, et al. ToxAlerts: a web server of structural alerts for toxic chemicals and compounds with potential adverse reactions[J]. 2012.

Compounds AI2:

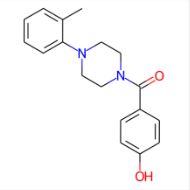

SMILES: Cc1ccccc1N1CCN(C(=O)c2ccc(O)cc2)CC1

San ID	No data
CAS	No data
InChI	InChI=1S/C18H20N2O2/c1-14-4-2-3-5-17(14)19-10-12-20(13-11-19)18(22)15-6-8-16(21)9-7-15/h2-9,21H,10-13H2,1H3
InChIKey	SNFUICMEZCRKEZ-UHFFFAOYSA-N

View in third-party databases: PubChem

MW	296.15	HBA	3	HBD	1
PAINS Alert	false	QED	0.92	NRot	2
TPSA	43.78	logD	2.59	logP	2.68
logS	-3.20				

Bad Average Good Scoring Notice

Absorption

Caco2	-4.61	HIA	99.39%	P-gp Inhibitor	54.70%
Bioavailability	99.92%	HFE	-9.56		

Distribution

BBB Penetration	87.37%	PPBR	1.00%	VDSS	2.18

Metabolism

CYP2D6 Inhibitor	14.19%	CYP3A4 Inhibitor	73.85%	CYP2C9 Inhibitor	81.46%
CYP2D6 Substrate	96.60%	CYP3A4 Substrate	48.68%	CYP2C9 Substrate	0.59%
CYP2C19 Inhibitor	86.49%	CYP1A2 Inhibitor	9.15%		

Excretion

Half Life (hour)	3.24	Clearance (mL/min/g)	50.74

Tox

hERG	96.80%	DILI	13.70%	NR-AhR	14.22%
Carcinogencity	37.51%	LD_{50}	1019.14	Respiratory Toxicity	50.63%
Eye Corrosion	0.07%				

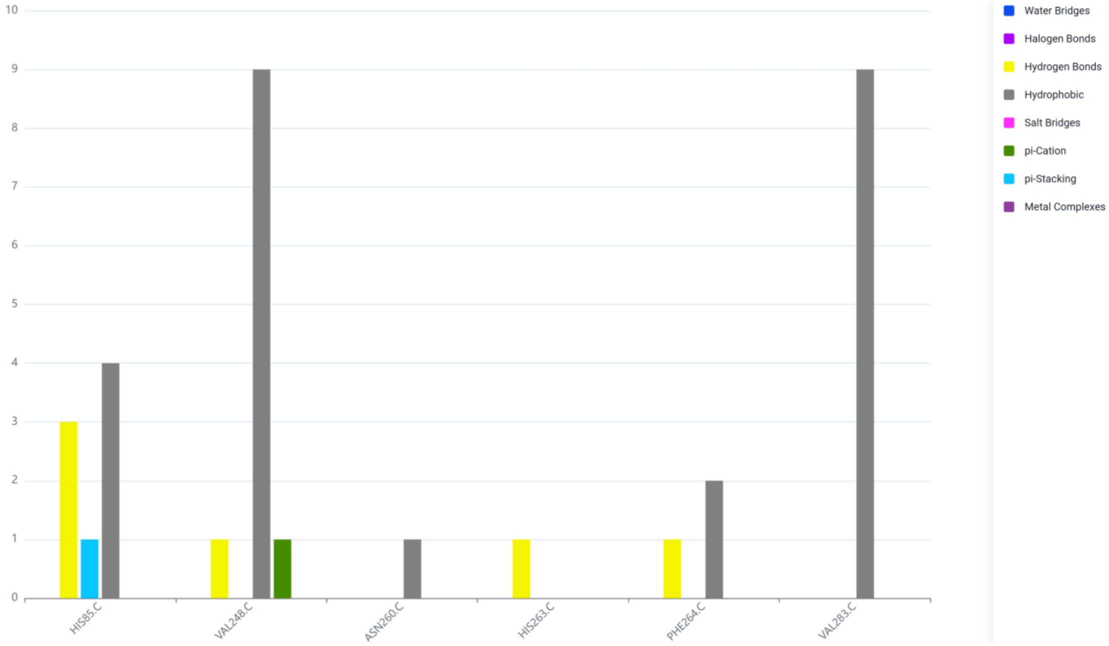

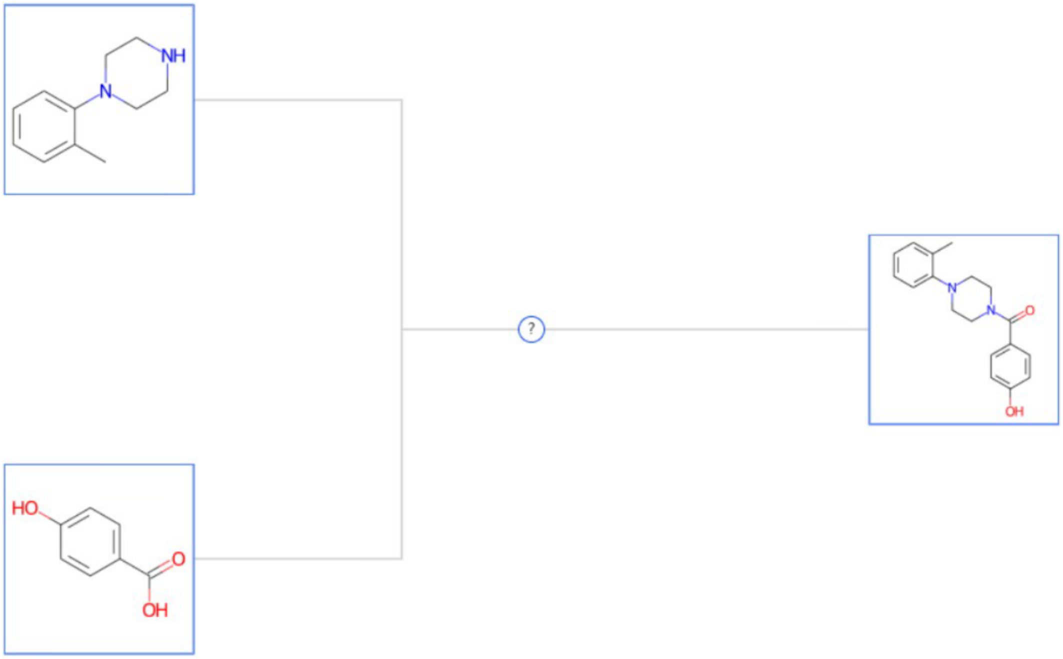

Alert Rule	Alert Structure	Reference
BMS Rule	✔	1. [1] Huth J R, Mendoza R, Olejniczak E T, et al. ALARM NMR: a rapid and robust experimental method to detect reactive false positives in biochemical screens[J]. Journal of the American Chemical Society, 2005, 127(1): 217-224.
Chelator Rule	✔	1. [1] Agrawal A, Johnson S L, Jacobsen J A, et al. Chelator fragment libraries for targeting metalloproteinases[J]. ChemMedChem: Chemistry Enabling Drug Discovery, 2010, 5(2): 195-199.
PAINS	✔	1. [1] Baell J B, Holloway G A. New substructure filters for removal of pan assay interference compounds (PAINS) from screening libraries and for their exclusion in bioassays[J]. Journal of medicinal chemistry, 2010, 53(7): 2719-2740.
Genotoxic Carcinogenicity Rule	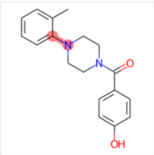	1. [1] Benigni R, Bossa C. Structure alerts for carcinogenicity, and the Salmonella assay system: a novel insight through the chemical relational databases technology[J]. Mutation Research/Reviews in Mutation Research, 2008, 659(3): 248-261.<br>2. [2] Ashby J, Tennant R W. Chemical structure, Salmonella mutagenicity and extent of carcinogenicity as indicators of genotoxic carcinogenesis among 222 chemicals tested in rodents by the US NCI/NTP[J]. Mutation Research/Genetic Toxicology, 1988, 204(1): 17-115.<br>3. [3] Kazius J, McGuire R, Bursi R. Derivation and validation of toxicophores for mutagenicity prediction[J]. Journal of medicinal chemistry, 2005, 48(1): 312-320.<br>4. [4] Bailey A B, Chanderbhan R, Collazo-Braier N, et al. The use of structure-activity relationship analysis in the food contact notification program[J]. Regulatory Toxicology and Pharmacology, 2005, 42(2): 225-235.
NTD	✔	1. [1] Brenk R, Schipani A, James D, et al. Lessons learnt from assembling screening libraries for drug discovery for neglected diseases[J]. ChemMedChem: Chemistry Enabling Drug Discovery, 2008, 3(3): 435-444.
SureChEMBL Rule	✔	1. [1] Sushko I, Salmina E, Potemkin V A, et al. ToxAlerts: a web server of structural alerts for toxic chemicals and compounds with potential adverse reactions[J]. 2012.

Compounds AI3:

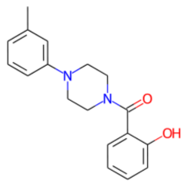

SMILES: Cc1cccc(N2CCN(C(=O)c3ccccc3O)CC2)c1

San ID	8fe5b7b420787b2832ef89c7d5787481
CAS	No data
InChI	InChI=1S/C18H20N2O2/c1-14-5-4-6-15(13-14)19-9-11-20(12-10-19)18(22)16-7-2-3-8-17(16)21/h2-8,13,21H,9-12H2,1H3
InChIKey	GJUCVVOKBJAJRT-UHFFFAOYSA-N

View in third-party databases: PubChem

MW	296.15	HBA	3	HBD	1
PAINS Alert	false	QED	0.92	NRot	2
TPSA	43.78	logD	3.18	logP	3.96
logS	-3.87				

Bad Average Good Scoring Notice

Absorption

Caco2	-4.77	HIA	99.96%	P-gp Inhibitor	83.65%
Bioavailability	99.97%	HFE	-8.36		

Distribution

BBB Penetration	84.42%	PPBR	1.00%	VDSS	1.84

Metabolism

CYP2D6 Inhibitor	12.44%	CYP3A4 Inhibitor	70.16%	CYP2C9 Inhibitor	82.77%
CYP2D6 Substrate	99.38%	CYP3A4 Substrate	48.88%	CYP2C9 Substrate	4.22%
CYP2C19 Inhibitor	90.39%	CYP1A2 Inhibitor	9.21%		

Excretion

Half Life (hour)	2.85	Clearance (mL/min/g)	50.53

Tox

hERG	95.94%	DILI	33.74%	NR-AhR	15.59%
Carcinogencity	15.61%	LD_{50}	469.18	Respiratory Toxicity	39.38%
Eye Corrosion	0.08%				

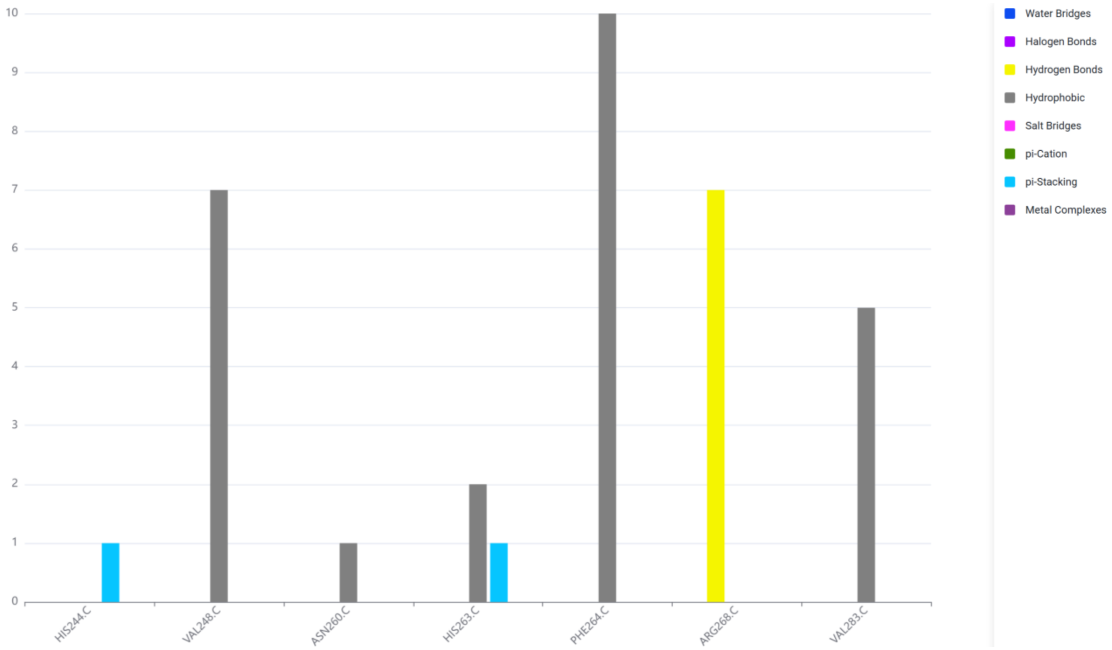

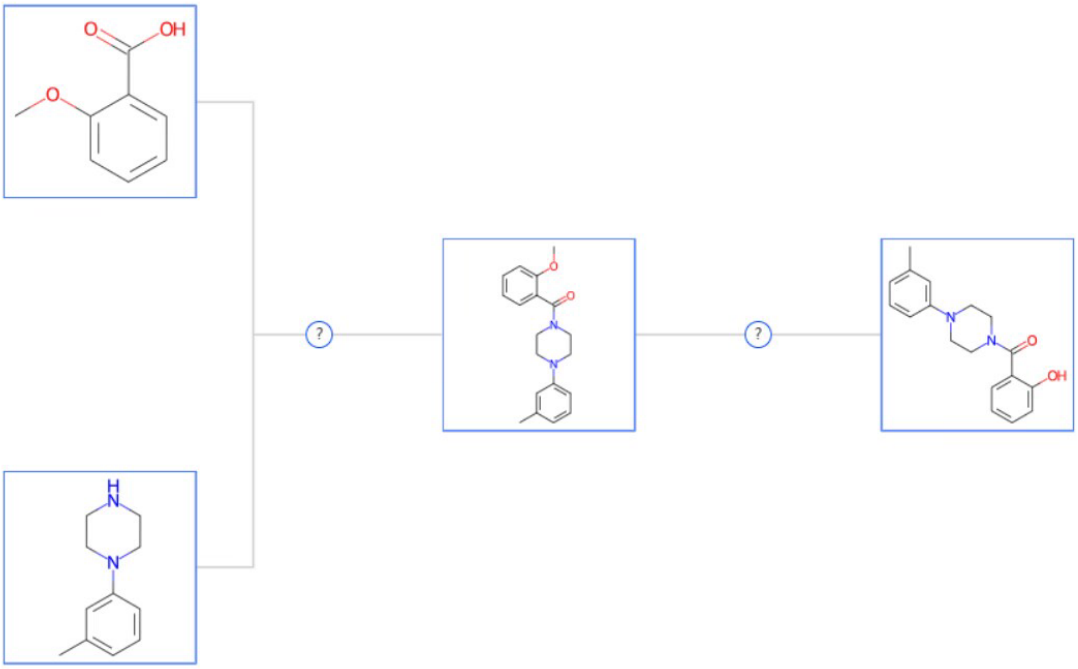

Alert Rule	Alert Structure	Reference
BMS Rule	✔	1. [1] Huth J R, Mendoza R, Olejniczak E T, et al. ALARM NMR: a rapid and robust experimental method to detect reactive false positives in biochemical screens[J]. Journal of the American Chemical Society, 2005, 127(1): 217-224.
Chelator Rule	✔	1. [1] Agrawal A, Johnson S L, Jacobsen J A, et al. Chelator fragment libraries for targeting metalloproteinases[J]. ChemMedChem: Chemistry Enabling Drug Discovery, 2010, 5(2): 195-199.
PAINS	✔	1. [1] Baell J B, Holloway G A. New substructure filters for removal of pan assay interference compounds (PAINS) from screening libraries and for their exclusion in bioassays[J]. Journal of medicinal chemistry, 2010, 53(7): 2719-2740.
Genotoxic Carcinogenicity Rule	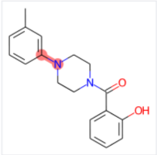	1. [1] Benigni R, Bossa C. Structure alerts for carcinogenicity, and the Salmonella assay system: a novel insight through the chemical relational databases technology[J]. Mutation Research/Reviews in Mutation Research, 2008, 659(3): 248-261. 2. [2] Ashby J, Tennant R W. Chemical structure, Salmonella mutagenicity and extent of carcinogenicity as indicators of genotoxic carcinogenesis among 222 chemicals tested in rodents by the US NCI/NTP[J]. Mutation Research/Genetic Toxicology, 1988, 204(1): 17-115. 3. [3] Kazius J, McGuire R, Bursi R. Derivation and validation of toxicophores for mutagenicity prediction[J]. Journal of medicinal chemistry, 2005, 48(1): 312-320. 4. [4] Bailey A B, Chanderbhan R, Collazo-Braier N, et al. The use of structure-activity relationship analysis in the food contact notification program[J]. Regulatory Toxicology and Pharmacology, 2005, 42(2): 225-235.
NTD	✔	1. [1] Brenk R, Schipani A, James D, et al. Lessons learnt from assembling screening libraries for drug discovery for neglected diseases[J]. ChemMedChem: Chemistry Enabling Drug Discovery, 2008, 3(3): 435-444.
SureChEMBL Rule	✔	1. [1] Sushko I, Salmina E, Potemkin V A, et al. ToxAlerts: a web server of structural alerts for toxic chemicals and compounds with potential adverse reactions[J]. 2012.

Compounds AI4:

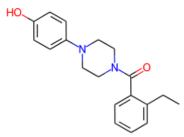

SMILES: CCc1ccccc1C(=O)N1CCN(c2ccc(O)cc2)CC1

San ID	No data
CAS	No data
InChI	InChI=1S/C19H22N2O2/c1-2-15-5-3-4-6-18(15)19(23)21-13-11-20(12-14-21)16-7-9-17(22)10-8-16/h3-10,22H,2,11-14H2,1H3
InChIKey	GBIROSULOGYORV-UHFFFAOYSA-N

View in third-party databases: Loading...

MW	310.17	HBA	3	HBD	1
PAINS Alert	false	QED	0.94	NRot	3
TPSA	43.78	logD	2.63	logP	2.87
logS	-3.55				

Bad Average Good Scoring Notice

Absorption

Caco2	-4.80	HIA	100.00%	P-gp Inhibitor	43.11%
Bioavailability	99.57%	HFE	-9.88		

Distribution

BBB Penetration	88.19%	PPBR	1.00%	VDSS	2.93

Metabolism

CYP2D6 Inhibitor	4.82%	CYP3A4 Inhibitor	79.75%	CYP2C9 Inhibitor	80.86%
CYP2D6 Substrate	84.78%	CYP3A4 Substrate	49.61%	CYP2C9 Substrate	3.27%
CYP2C19 Inhibitor	84.52%	CYP1A2 Inhibitor	10.69%		

Excretion

Half Life (hour)	4.37	Clearance (mL/min/g)	51.91

Tox

hERG	95.67%	DILI	35.75%	NR-AhR	24.13%
Carcinogencity	43.49%	LD_{50}	533.60	Respiratory Toxicity	33.49%
Eye Corrosion	0.29%				

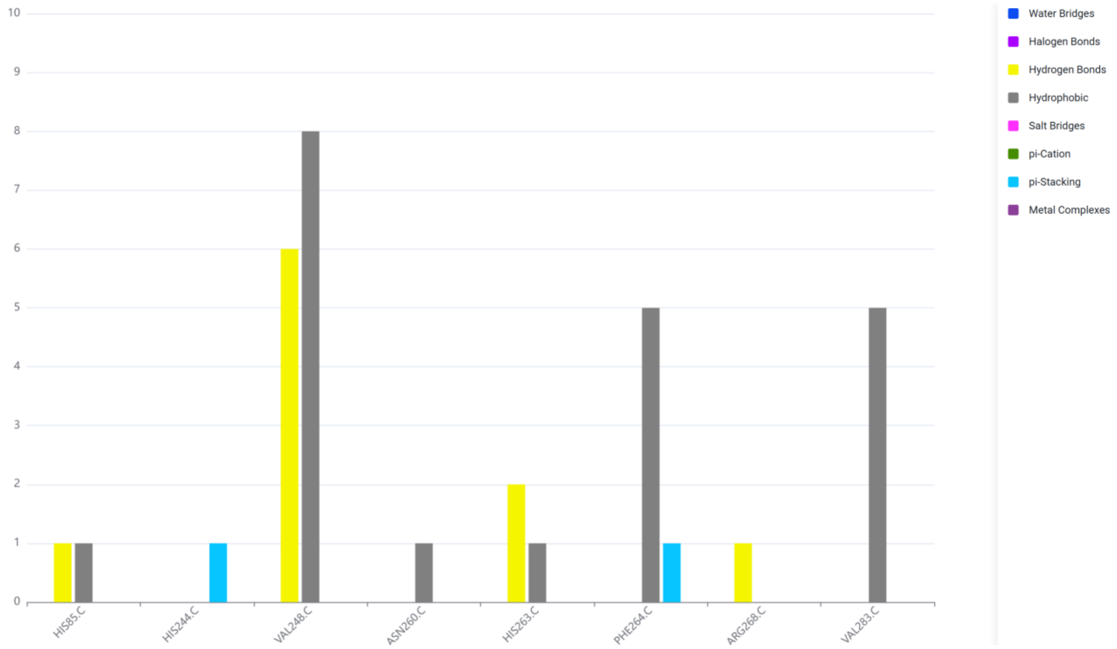

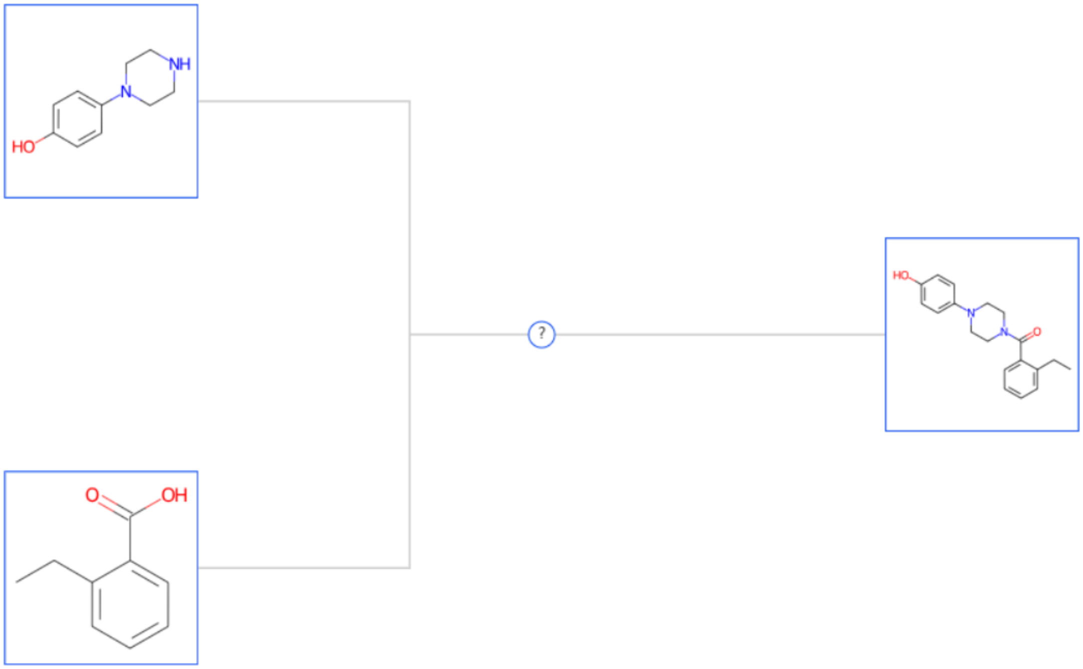

Alert Rule	Alert Structure	Reference
BMS Rule	✓	1. [1] Huth J R, Mendoza R, Olejniczak E T, et al. ALARM NMR: a rapid and robust experimental method to detect reactive false positives in biochemical screens[J]. Journal of the American Chemical Society, 2005, 127(1): 217-224.
Chelator Rule	✓	1. [1] Agrawal A, Johnson S L, Jacobsen J A, et al. Chelator fragment libraries for targeting metalloproteinases[J]. ChemMedChem: Chemistry Enabling Drug Discovery, 2010, 5(2): 195-199.
PAINS	✓	1. [1] Baell J B, Holloway G A. New substructure filters for removal of pan assay interference compounds (PAINS) from screening libraries and for their exclusion in bioassays[J]. Journal of medicinal chemistry, 2010, 53(7): 2719-2740.
Genotoxic Carcinogenicity Rule	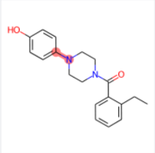	1. [1] Benigni R, Bossa C. Structure alerts for carcinogenicity, and the Salmonella assay system: a novel insight through the chemical relational databases technology[J]. Mutation Research/Reviews in Mutation Research, 2008, 659(3): 248-261.<br>2. [2] Ashby J, Tennant R W. Chemical structure, Salmonella mutagenicity and extent of carcinogenicity as indicators of genotoxic carcinogenesis among 222 chemicals tested in rodents by the US NCI/NTP[J]. Mutation Research/Genetic Toxicology, 1988, 204(1): 17-115.<br>3. [3] Kazius J, McGuire R, Bursi R. Derivation and validation of toxicophores for mutagenicity prediction[J]. Journal of medicinal chemistry, 2005, 48(1): 312-320.<br>4. [4] Bailey A B, Chanderbhan R, Collazo-Braier N, et al. The use of structure-activity relationship analysis in the food contact notification program[J]. Regulatory Toxicology and Pharmacology, 2005, 42(2): 225-235.
NTD	✓	1. [1] Brenk R, Schipani A, James D, et al. Lessons learnt from assembling screening libraries for drug discovery for neglected diseases[J]. ChemMedChem: Chemistry Enabling Drug Discovery, 2008, 3(3): 435-444.
SureChEMBL Rule	✓	1. [1] Sushko I, Salmina E, Potemkin V A, et al. ToxAlerts: a web server of structural alerts for toxic chemicals and compounds with potential adverse reactions[J]. 2012.

Compounds AI5:

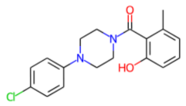

SMILES: Cc1cccc(O)c1C(=O)N1CCN(c2ccc(Cl)cc2)CC1

San ID	No data
CAS	No data
InChI	InChI=1S/C18H19ClN2O2/c1-13-3-2-4-16(22)17(13)18(23)21-11-9-20(10-12-21)15-7-5-14(19)6-8-15/h2-8,22H,9-12H2,1H3
InChIKey	HKQOOUOZIOWURN-UHFFFAOYSA-N

View in third-party databases: No data

MW	330.11	HBA	3	HBD	1
PAINS Alert	false	QED	0.91	NRot	2
TPSA	43.78	logD	3.16	logP	4.15
logS	-3.92				

Bad Average Good Scoring Notice

Absorption

Caco2	-4.70	HIA	99.79%	P-gp Inhibitor	88.13%
Bioavailability	99.98%	HFE	-8.45		

Distribution

BBB Penetration	74.96%	PPBR	1.00%	VDSS	2.46

Metabolism

CYP2D6 Inhibitor	16.68%	CYP3A4 Inhibitor	64.52%	CYP2C9 Inhibitor	85.32%
CYP2D6 Substrate	97.27%	CYP3A4 Substrate	49.27%	CYP2C9 Substrate	74.37%
CYP2C19 Inhibitor	94.10%	CYP1A2 Inhibitor	19.31%		

Excretion

Half Life (hour)	3.51	Clearance (mL/min/g)	51.25

Tox

hERG	95.73%	DILI	51.49%	NR-AhR	16.59%
Carcinogencity	18.80%	LD_{50}	549.23	Respiratory Toxicity	12.84%
Eye Corrosion	0.04%				

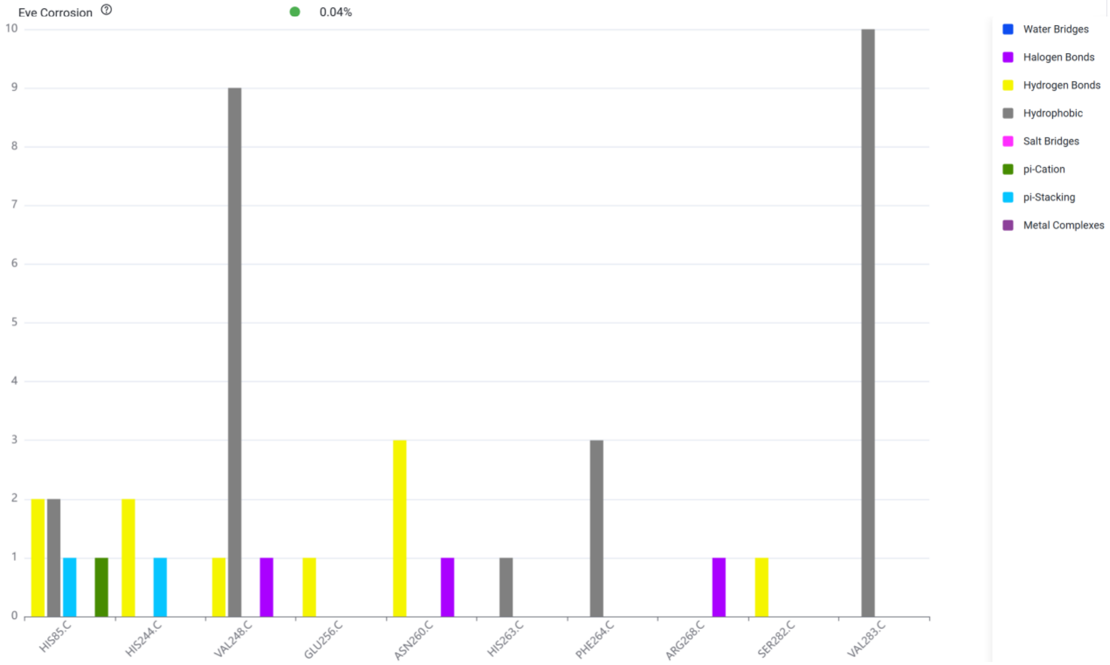

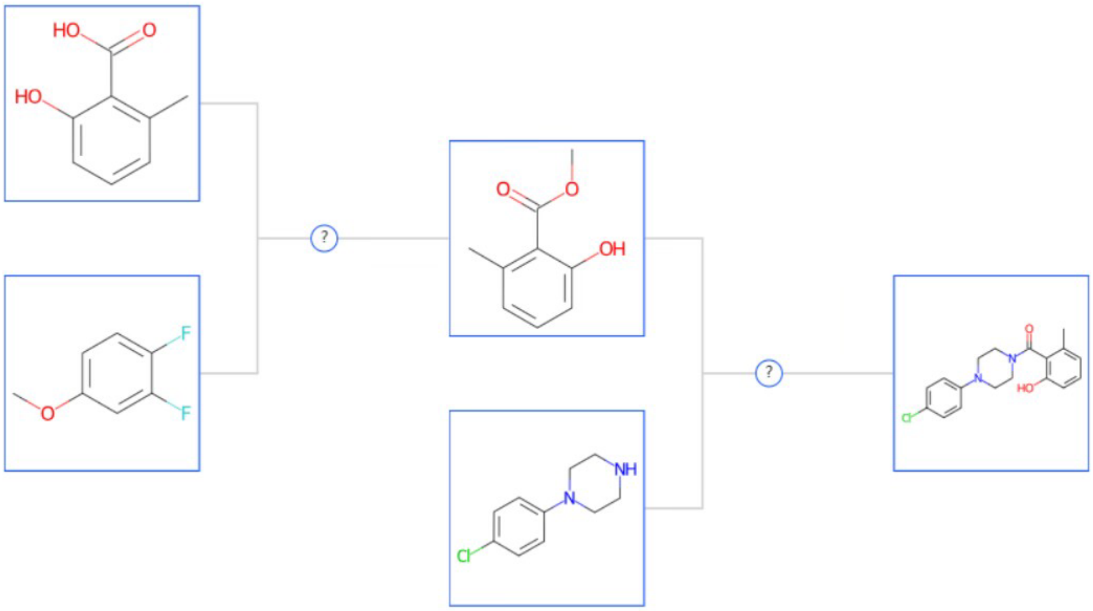

Alert Rule	Alert Structure	Reference
BMS Rule	✔	1. [1] Huth J R, Mendoza R, Olejniczak E T, et al. ALARM NMR: a rapid and robust experimental method to detect reactive false positives in biochemical screens[J]. Journal of the American Chemical Society, 2005, 127(1): 217-224.
Chelator Rule	✔	1. [1] Agrawal A, Johnson S L, Jacobsen J A, et al. Chelator fragment libraries for targeting metalloproteinases[J]. ChemMedChem: Chemistry Enabling Drug Discovery, 2010, 5(2): 195-199.
PAINS	✔	1. [1] Baell J B, Holloway G A. New substructure filters for removal of pan assay interference compounds (PAINS) from screening libraries and for their exclusion in bioassays[J]. Journal of medicinal chemistry, 2010, 53(7): 2719-2740.
Genotoxic Carcinogenicity Rule	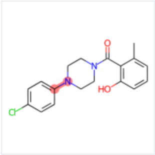	1. [1] Benigni R, Bossa C. Structure alerts for carcinogenicity, and the Salmonella assay system: a novel insight through the chemical relational databases technology[J]. Mutation Research/Reviews in Mutation Research, 2008, 659(3): 248-261.<br>2. [2] Ashby J, Tennant R W. Chemical structure, Salmonella mutagenicity and extent of carcinogenicity as indicators of genotoxic carcinogenesis among 222 chemicals tested in rodents by the US NCI/NTP[J]. Mutation Research/Genetic Toxicology, 1988, 204(1): 17-115.<br>3. [3] Kazius J, McGuire R, Bursi R. Derivation and validation of toxicophores for mutagenicity prediction[J]. Journal of medicinal chemistry, 2005, 48(1): 312-320.<br>4. [4] Bailey A B, Chanderbhan R, Collazo-Braier N, et al. The use of structure-activity relationship analysis in the food contact notification program[J]. Regulatory Toxicology and Pharmacology, 2005, 42(2): 225-235.
NTD	✔	1. [1] Brenk R, Schipani A, James D, et al. Lessons learnt from assembling screening libraries for drug discovery for neglected diseases[J]. ChemMedChem: Chemistry Enabling Drug Discovery, 2008, 3(3): 435-444.
SureChEMBL Rule	✔	1. [1] Sushko I, Salmina E, Potemkin V A, et al. ToxAlerts: a web server of structural alerts for toxic chemicals and compounds with potential adverse reactions[J]. 2012.

Compounds AI6:

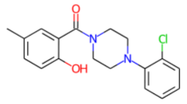

SMILES: Cc1ccc(O)c(C(=O)N2CCN(c3ccccc3Cl)CC2)c1

San ID	No data
CAS	No data
InChI	InChI=1S/C18H19ClN2O2/c1-13-6-7-17(22)14(12-13)18(23)21-10-8-20(9-11-21)16-5-3-2-4-15(16)19/h2-7,12,22H,8-11H2,1H3
InChIKey	LILUKTRUHNLJNC-UHFFFAOYSA-N

View in third-party databases: PubChem

MW	330.11	HBA	3	HBD	1
PAINS Alert	false	QED	0.91	NRot	2
TPSA	43.78	logD	3.85	logP	4.45
logS	-4.05				

Bad Average Good Scoring Notice

Absorption

Caco2	-4.66	HIA	100.00%	P-gp Inhibitor	84.21%
Bioavailability	99.98%	HFE	-8.22		

Distribution

BBB Penetration	89.42%	PPBR	1.00%	VDSS	1.85

Metabolism

CYP2D6 Inhibitor	15.41%	CYP3A4 Inhibitor	65.78%	CYP2C9 Inhibitor	86.36%
CYP2D6 Substrate	98.19%	CYP3A4 Substrate	52.88%	CYP2C9 Substrate	79.92%
CYP2C19 Inhibitor	93.38%	CYP1A2 Inhibitor	10.84%		

Excretion

Half Life (hour)	3.82	Clearance (mL/min/g)	53.56

Tox

hERG	96.48%	DILI	61.07%	NR-AhR	15.78%
Carcinogencity	2.11%	LD_{50}	854.69	Respiratory Toxicity	13.63%
Eye Corrosion	0.21%				

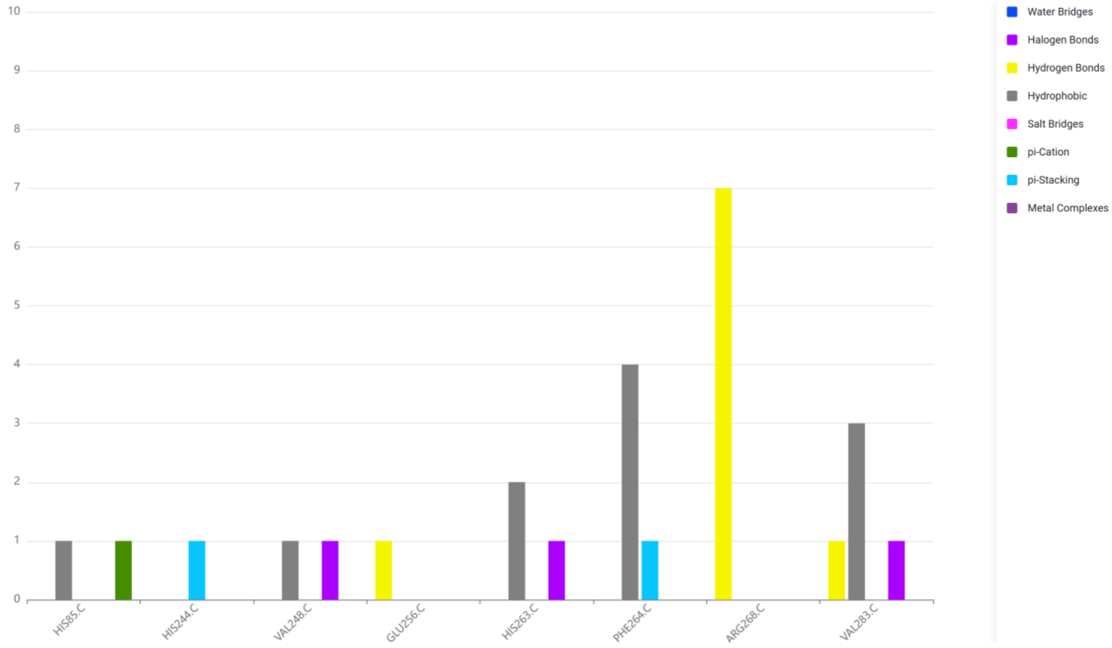

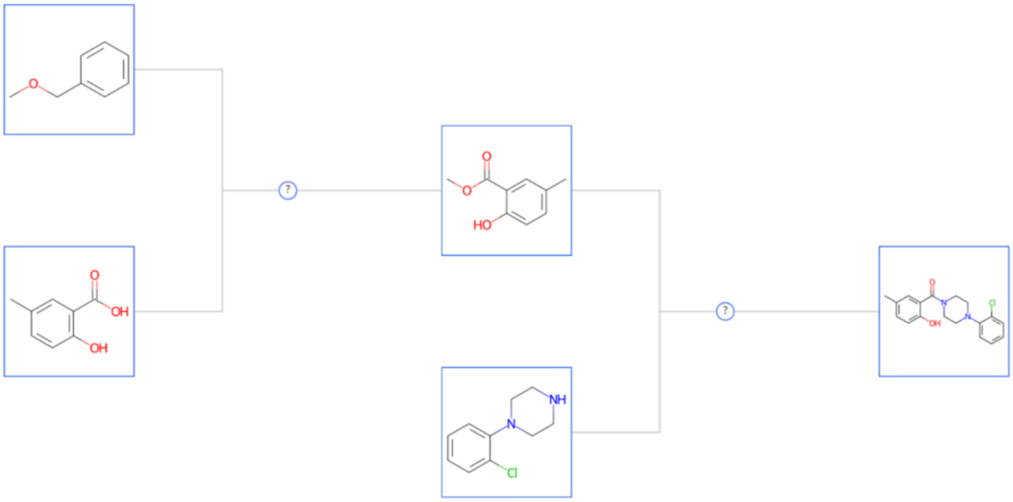

Alert Rule	Alert Structure	Reference
BMS Rule	✔	1. [1] Huth J R, Mendoza R, Olejniczak E T, et al. ALARM NMR: a rapid and robust experimental method to detect reactive false positives in biochemical screens[J]. Journal of the American Chemical Society, 2005, 127(1): 217-224.
Chelator Rule	✔	1. [1] Agrawal A, Johnson S L, Jacobsen J A, et al. Chelator fragment libraries for targeting metalloproteinases[J]. ChemMedChem: Chemistry Enabling Drug Discovery, 2010, 5(2): 195-199.
PAINS	✔	1. [1] Baell J B, Holloway G A. New substructure filters for removal of pan assay interference compounds (PAINS) from screening libraries and for their exclusion in bioassays[J]. Journal of medicinal chemistry, 2010, 53(7): 2719-2740.
Genotoxic Carcinogenicity Rule	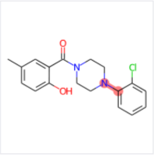	1. [1] Benigni R, Bossa C. Structure alerts for carcinogenicity, and the Salmonella assay system: a novel insight through the chemical relational databases technology[J]. Mutation Research/Reviews in Mutation Research, 2008, 659(3): 248-261. 2. [2] Ashby J, Tennant R W. Chemical structure, Salmonella mutagenicity and extent of carcinogenicity as indicators of genotoxic carcinogenesis among 222 chemicals tested in rodents by the US NCI/NTP[J]. Mutation Research/Genetic Toxicology, 1988, 204(1): 17-115. 3. [3] Kazius J, McGuire R, Bursi R. Derivation and validation of toxicophores for mutagenicity prediction[J]. Journal of medicinal chemistry, 2005, 48(1): 312-320. 4. [4] Bailey A B, Chanderbhan R, Collazo-Braier N, et al. The use of structure-activity relationship analysis in the food contact notification program[J]. Regulatory Toxicology and Pharmacology, 2005, 42(2): 225-235.
NTD	✔	1. [1] Brenk R, Schipani A, James D, et al. Lessons learnt from assembling screening libraries for drug discovery for neglected diseases[J]. ChemMedChem: Chemistry Enabling Drug Discovery, 2008, 3(3): 435-444.
SureChEMBL Rule	✔	1. [1] Sushko I, Salmina E, Potemkin V A, et al. ToxAlerts: a web server of structural alerts for toxic chemicals and compounds with potential adverse reactions[J]. 2012.

Compounds AI7:

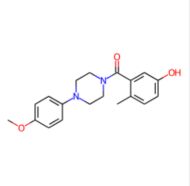

SMILES: COc1ccc(N2CCN(C(=O)c3cc(O)ccc3C)CC2)cc1

San ID	No data
CAS	No data
InChI	InChI=1S/C19H22N2O3/c1-14-3-6-16(22)13-18(14)19(23)21-11-9-20(10-12-21)15-4-7-17(24-2)8-5-15/h3-8,13,22H,9-12H2,1-2H3
InChIKey	YJXCXENINWFZCB-UHFFFAOYSA-N

View in third-party databases: No data

MW	326.16	HBA	4	HBD	1
PAINS Alert	true	QED	0.94	NRot	3
TPSA	53.01	logD	2.09	logP	2.2
logS	-3.71				

Bad Average Good Scoring Notice

Absorption

Caco2	-4.64	HIA	99.27%	P-gp Inhibitor	31.68%
Bioavailability	99.98%	HFE	-10.59		

Distribution

BBB Penetration	82.65%	PPBR	1.00%	VDSS	2.68

Metabolism

CYP2D6 Inhibitor	7.13%	CYP3A4 Inhibitor	84.51%	CYP2C9 Inhibitor	75.81%
CYP2D6 Substrate	95.25%	CYP3A4 Substrate	64.40%	CYP2C9 Substrate	99.85%
CYP2C19 Inhibitor	77.41%	CYP1A2 Inhibitor	7.62%		

Excretion

Half Life (hour)	3.55	Clearance (mL/min/g)	58.57

Tox

hERG	95.76%	DILI	53.72%	NR-AhR	17.02%
Carcinogencity	30.54%	LD_{50}	656.10	Respiratory Toxicity	73.13%
Eye Corrosion	0.18%				

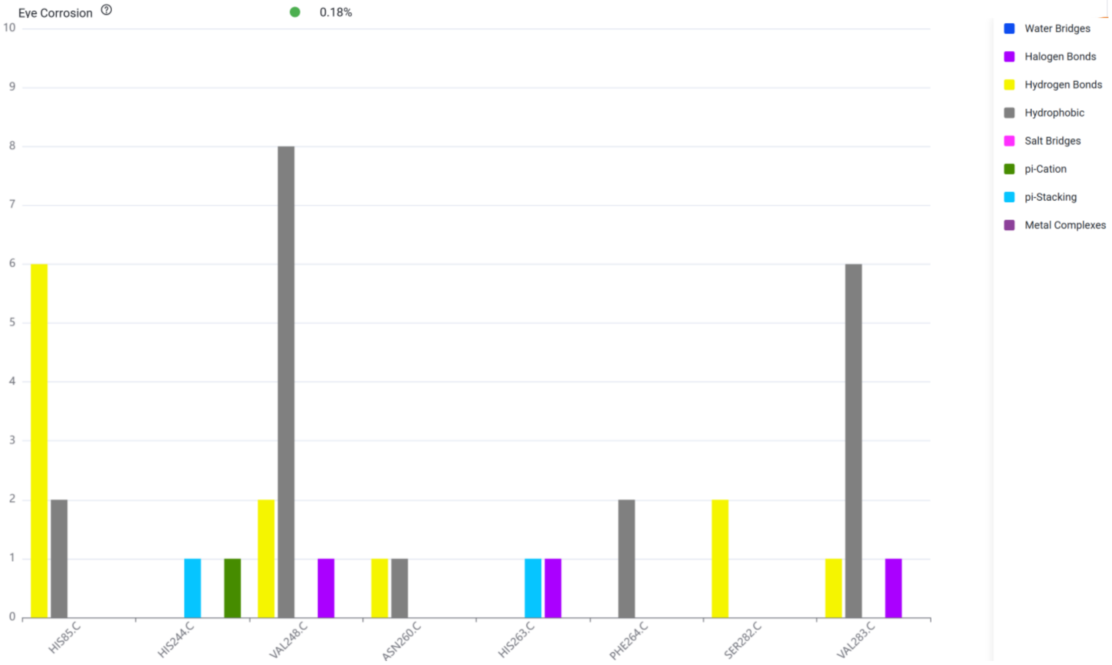

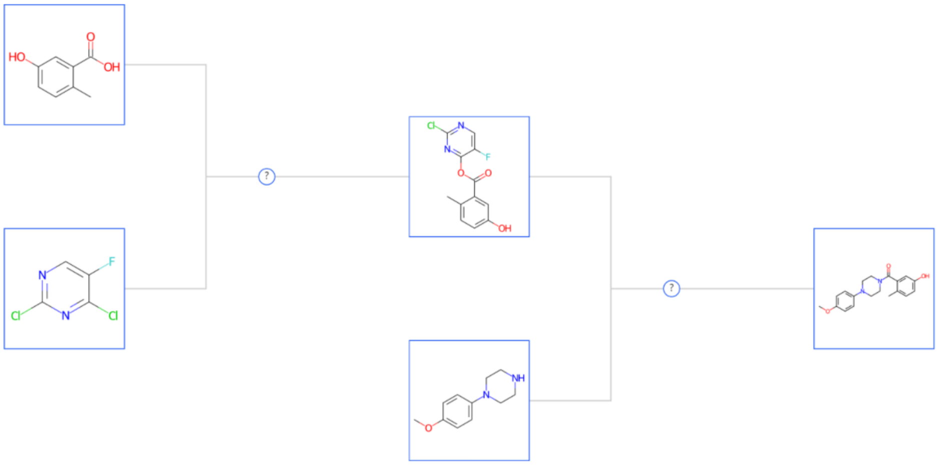

Alert Rule	Alert Structure	Reference
BMS Rule	✓	1. [1] Huth J R, Mendoza R, Olejniczak E T, et al. ALARM NMR: a rapid and robust experimental method to detect reactive false positives in biochemical screens[J]. Journal of the American Chemical Society, 2005, 127(1): 217-224.
Chelator Rule	✓	1. [1] Agrawal A, Johnson S L, Jacobsen J A, et al. Chelator fragment libraries for targeting metalloproteinases[J]. ChemMedChem: Chemistry Enabling Drug Discovery, 2010, 5(2): 195-199.
PAINS	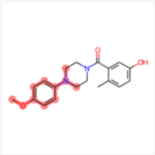	1. [1] Baell J B, Holloway G A. New substructure filters for removal of pan assay interference compounds (PAINS) from screening libraries and for their exclusion in bioassays[J]. Journal of medicinal chemistry, 2010, 53(7): 2719-2740.
Genotoxic Carcinogenicity Rule	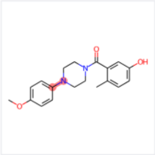	1. [1] Benigni R, Bossa C. Structure alerts for carcinogenicity, and the Salmonella assay system: a novel insight through the chemical relational databases technology[J]. Mutation Research/Reviews in Mutation Research, 2008, 659(3): 248-261.<br>2. [2] Ashby J, Tennant R W. Chemical structure, Salmonella mutagenicity and extent of carcinogenicity as indicators of genotoxic carcinogenesis among 222 chemicals tested in rodents by the US NCI/NTP[J]. Mutation Research/Genetic Toxicology, 1988, 204(1): 17-115.<br>3. [3] Kazius J, McGuire R, Bursi R. Derivation and validation of toxicophores for mutagenicity prediction[J]. Journal of medicinal chemistry, 2005, 48(1): 312-320.<br>4. [4] Bailey A B, Chanderbhan R, Collazo-Braier N, et al. The use of structure-activity relationship analysis in the food contact notification program[J]. Regulatory Toxicology and Pharmacology, 2005, 42(2): 225-235.
NTD	✓	1. [1] Brenk R, Schipani A, James D, et al. Lessons learnt from assembling screening libraries for drug discovery for neglected diseases[J]. ChemMedChem: Chemistry Enabling Drug Discovery, 2008, 3(3): 435-444.
SureChEMBL Rule	✓	1. [1] Sushko I, Salmina E, Potemkin V A, et al. ToxAlerts: a web server of structural alerts for toxic chemicals and compounds with potential adverse reactions[J]. 2012.

Compounds AI8:

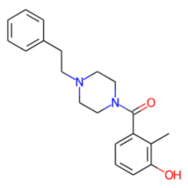

SMILES: Cc1c(O)cccc1C(=O)N1CCN(CCc2ccccc2)CC1

San ID	No data
CAS	No data
InChI	InChI=1S/C20H24N2O2/c1-16-18(8-5-9-19(16)23)20(24)22-14-12-21(13-15-22)11-10-17-6-3-2-4-7-17/h2-9,23H,10-15H2,1H3
InChIKey	FNEZUBFBTVGHGW-UHFFFAOYSA-N

View in third-party databases: No data

MW	324.18	HBA	3	HBD	1
PAINS Alert	false	QED	0.94	NRot	4
TPSA	43.78	logD	2.29	logP	2.23
logS	-3.03				

Bad Average Good Scoring Notice

Absorption

Caco2	-4.74	HIA	98.86%	P-gp Inhibitor	86.65%
Bioavailability	87.12%	HFE	-9.55		

Distribution

BBB Penetration	81.43%	PPBR	1.00%	VDSS	1.94

Metabolism

CYP2D6 Inhibitor	62.07%	CYP3A4 Inhibitor	43.57%	CYP2C9 Inhibitor	58.16%
CYP2D6 Substrate	98.98%	CYP3A4 Substrate	31.09%	CYP2C9 Substrate	4.84%
CYP2C19 Inhibitor	70.83%	CYP1A2 Inhibitor	8.54%		

Excretion

Half Life (hour)	3.88	Clearance (mL/min/g)	49.84

Tox

hERG	94.50%	DILI	22.60%	NR-AhR	10.15%
Carcinogencity	0.03%	LD_{50}	333.07	Respiratory Toxicity	94.54%
Eye Corrosion	0.30%				

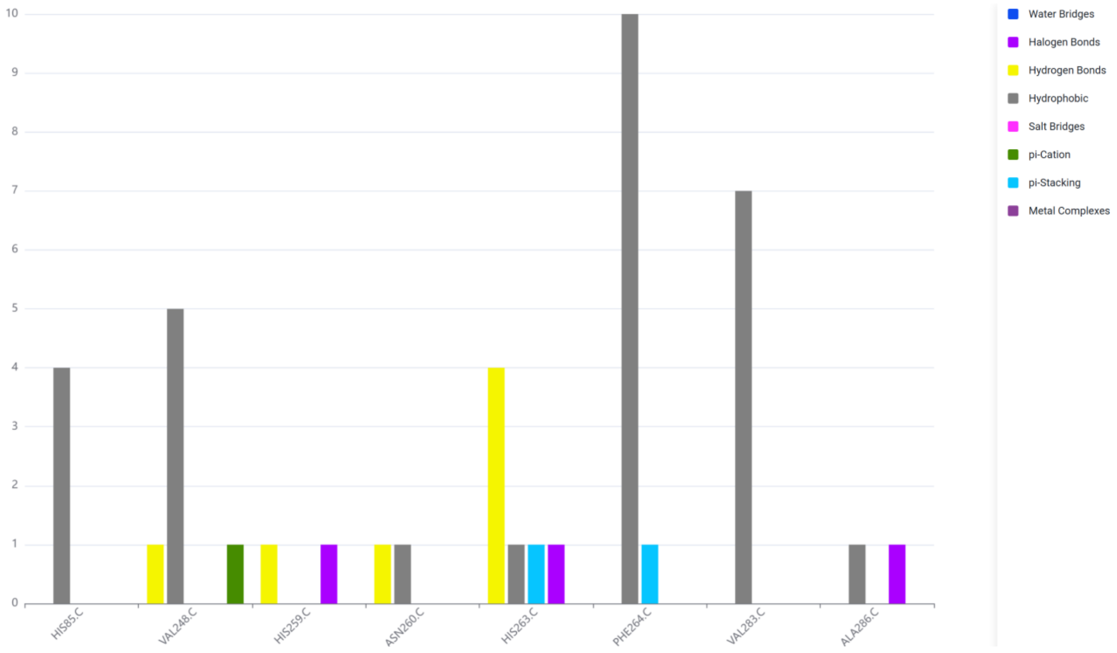

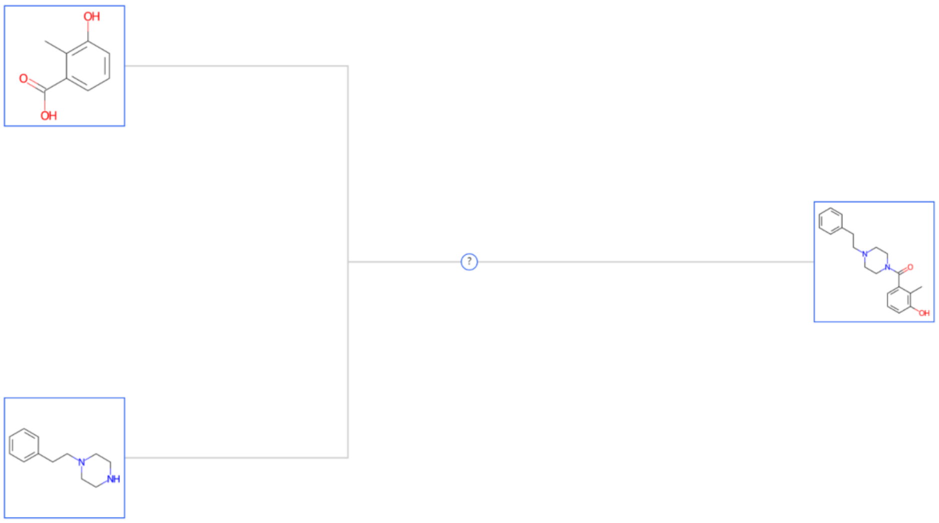

Alert Rule	Alert Structure	Reference
BMS Rule	✔	1. [1] Huth J R, Mendoza R, Olejniczak E T, et al. ALARM NMR: a rapid and robust experimental method to detect reactive false positives in biochemical screens[J]. Journal of the American Chemical Society, 2005, 127(1): 217-224.
Chelator Rule	✔	1. [1] Agrawal A, Johnson S L, Jacobsen J A, et al. Chelator fragment libraries for targeting metalloproteinases[J]. ChemMedChem: Chemistry Enabling Drug Discovery, 2010, 5(2): 195-199.
PAINS	✔	1. [1] Baell J B, Holloway G A. New substructure filters for removal of pan assay interference compounds (PAINS) from screening libraries and for their exclusion in bioassays[J]. Journal of medicinal chemistry, 2010, 53(7): 2719-2740.
Genotoxic Carcinogenicity Rule	✔	1. [1] Benigni R, Bossa C. Structure alerts for carcinogenicity, and the Salmonella assay system: a novel insight through the chemical relational databases technology[J]. Mutation Research/Reviews in Mutation Research, 2008, 659(3): 248-261.<br>2. [2] Ashby J, Tennant R W. Chemical structure, Salmonella mutagenicity and extent of carcinogenicity as indicators of genotoxic carcinogenesis among 222 chemicals tested in rodents by the US NCI/NTP[J]. Mutation Research/Genetic Toxicology, 1988, 204(1): 17-115.<br>3. [3] Kazius J, McGuire R, Bursi R. Derivation and validation of toxicophores for mutagenicity prediction[J]. Journal of medicinal chemistry, 2005, 48(1): 312-320.<br>4. [4] Bailey A B, Chanderbhan R, Collazo-Braier N, et al. The use of structure-activity relationship analysis in the food contact notification program[J]. Regulatory Toxicology and Pharmacology, 2005, 42(2): 225-235.
NTD	✔	1. [1] Brenk R, Schipani A, James D, et al. Lessons learnt from assembling screening libraries for drug discovery for neglected diseases[J]. ChemMedChem: Chemistry Enabling Drug Discovery, 2008, 3(3): 435-444.
SureChEMBL Rule	✔	1. [1] Sushko I, Salmina E, Potemkin V A, et al. ToxAlerts: a web server of structural alerts for toxic chemicals and compounds with potential adverse reactions[J]. 2012.

Compounds AI9:

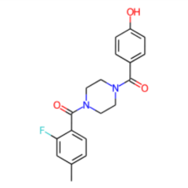

SMILES: Cc1ccc(C(=O)N2CCN(C(=O)c3ccc(O)cc3)CC2)c(F)c1

**San ID**	No data
**CAS**	No data
**InChI**	InChI=1S/C19H19FN2O3/c1-13-2-7-16(17(20)12-13)19(25)22-10-8-21(9-11-22)18(24)14-3-5-15(23)6-4-14/h2-7,12,23H,8-11H2,1H3
**InChIKey**	XEMAMRONPXTRLT-UHFFFAOYSA-N

View in third-party databases: No data

MW	342.14	HBA	3	HBD	1
PAINS Alert	false	QED	0.91	NRot	2
TPSA	60.85	logD	1.39	logP	2.68
logS	-4.04				

Bad Average Good Scoring Notice

Absorption

Caco2	-4.80	HIA	100.00%	P-gp Inhibitor	17.33%
Bioavailability	99.63%	HFE	-10.45		

Distribution

BBB Penetration	72.19%	PPBR	1.00%	VDSS	2.52

Metabolism

CYP2D6 Inhibitor	4.28%	CYP3A4 Inhibitor	55.31%	CYP2C9 Inhibitor	55.91%
CYP2D6 Substrate	96.73%	CYP3A4 Substrate	63.65%	CYP2C9 Substrate	0.08%
CYP2C19 Inhibitor	72.86%	CYP1A2 Inhibitor	3.93%		

Excretion

Half Life (hour)	4.14	Clearance (mL/min/g)	46.40

Tox

hERG	91.34%	DILI	82.44%	NR-AhR	5.39%
Carcinogencity	24.92%	LD_{50}	994.36	Respiratory Toxicity	3.11%
Eye Corrosion	0.10%				

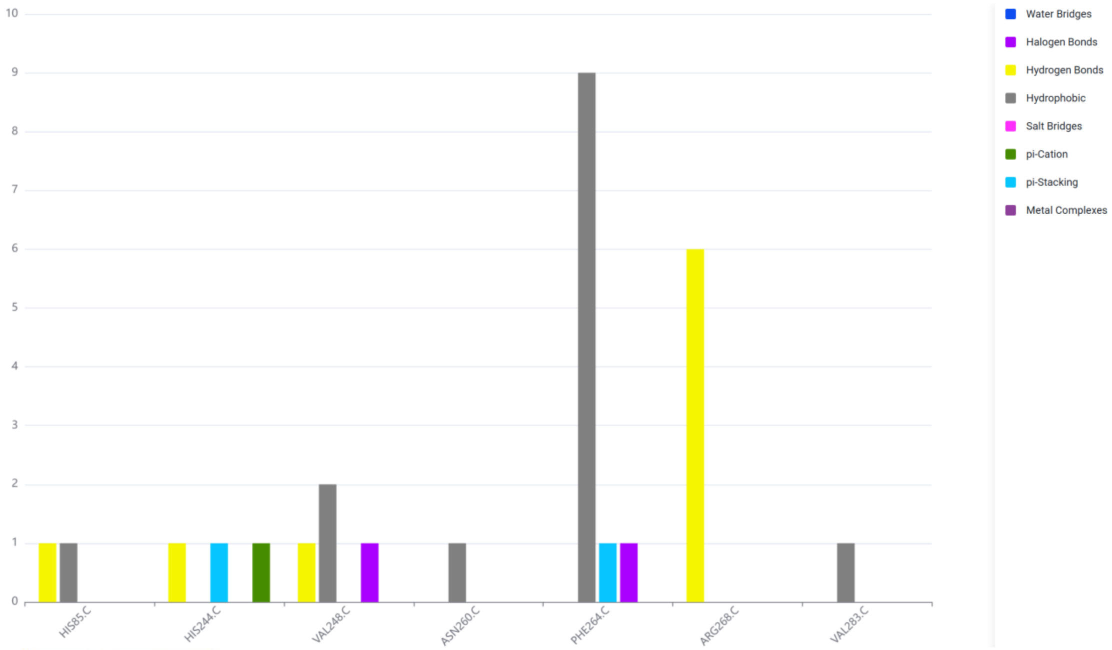

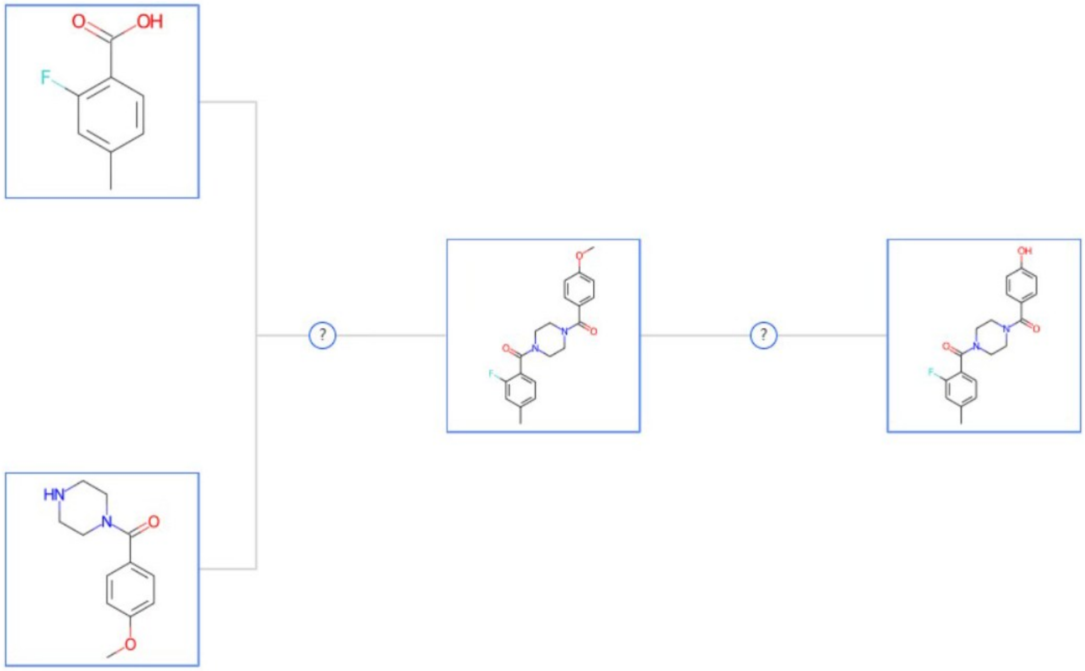

Alert Rule	Alert Structure	Reference
BMS Rule	✔	1. [1] Huth J R, Mendoza R, Olejniczak E T, et al. ALARM NMR: a rapid and robust experimental method to detect reactive false positives in biochemical screens[J]. Journal of the American Chemical Society, 2005, 127(1): 217-224.
Chelator Rule	✔	1. [1] Agrawal A, Johnson S L, Jacobsen J A, et al. Chelator fragment libraries for targeting metalloproteinases[J]. ChemMedChem: Chemistry Enabling Drug Discovery, 2010, 5(2): 195-199.
PAINS	✔	1. [1] Baell J B, Holloway G A. New substructure filters for removal of pan assay interference compounds (PAINS) from screening libraries and for their exclusion in bioassays[J]. Journal of medicinal chemistry, 2010, 53(7): 2719-2740.
Genotoxic Carcinogenicity Rule	✔	1. [1] Benigni R, Bossa C. Structure alerts for carcinogenicity, and the Salmonella assay system: a novel insight through the chemical relational databases technology[J]. Mutation Research/Reviews in Mutation Research, 2008, 659(3): 248-261.<br>2. [2] Ashby J, Tennant R W. Chemical structure, Salmonella mutagenicity and extent of carcinogenicity as indicators of genotoxic carcinogenesis among 222 chemicals tested in rodents by the US NCI/NTP[J]. Mutation Research/Genetic Toxicology, 1988, 204(1): 17-115.<br>3. [3] Kazius J, McGuire R, Bursi R. Derivation and validation of toxicophores for mutagenicity prediction[J]. Journal of medicinal chemistry, 2005, 48(1): 312-320.<br>4. [4] Bailey A B, Chanderbhan R, Collazo-Braier N, et al. The use of structure-activity relationship analysis in the food contact notification program[J]. Regulatory Toxicology and Pharmacology, 2005, 42(2): 225-235.
NTD	✔	1. [1] Brenk R, Schipani A, James D, et al. Lessons learnt from assembling screening libraries for drug discovery for neglected diseases[J]. ChemMedChem: Chemistry Enabling Drug Discovery, 2008, 3(3): 435-444.
SureChEMBL Rule	✔	1. [1] Sushko I, Salmina E, Potemkin V A, et al. ToxAlerts: a web server of structural alerts for toxic chemicals and compounds with potential adverse reactions[J]. 2012.

Compounds AI10:

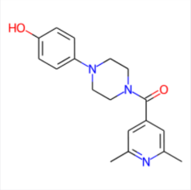

SMILES: Cc1cc(C(=O)N2CCN(c3ccc(O)cc3)CC2)cc(C)n1

San ID	No data
CAS	No data
InChI	InChI=1S/C18H21N3O2/c1-13-11-15(12-14(2)19-13)18(23)21-9-7-20(8-10-21)16-3-5-17(22)6-4-16/h3-6,11-12,22H,7-10H2,1-2H3
InChIKey	JKOLHQINYQYQSN-UHFFFAOYSA-N

View in third-party databases: Loading...

MW	311.16	HBA	4	HBD	1
PAINS Alert	false	QED	0.92	NRot	2
TPSA	56.67	logD	1.34	logP	1.3
logS	-2.71				

Bad Average Good Scoring Notice

Absorption

Caco2	-4.60	HIA	98.81%	P-gp Inhibitor	12.80%
Bioavailability	99.97%	HFE	-9.59		

Distribution

BBB Penetration	78.65%	PPBR	1.00%	VDSS	2.60

Metabolism

CYP2D6 Inhibitor	5.41%	CYP3A4 Inhibitor	58.47%	CYP2C9 Inhibitor	58.25%
CYP2D6 Substrate	96.44%	CYP3A4 Substrate	72.01%	CYP2C9 Substrate	0.36%
CYP2C19 Inhibitor	57.59%	CYP1A2 Inhibitor	12.68%		

Excretion

Half Life (hour)	3.47	Clearance (mL/min/g)	51.08

Tox

hERG	91.18%	DILI	33.06%	NR-AhR	19.99%
Carcinogencity	7.73%	LD_{50}	686.78	Respiratory Toxicity	95.69%
Eye Corrosion	0.37%				

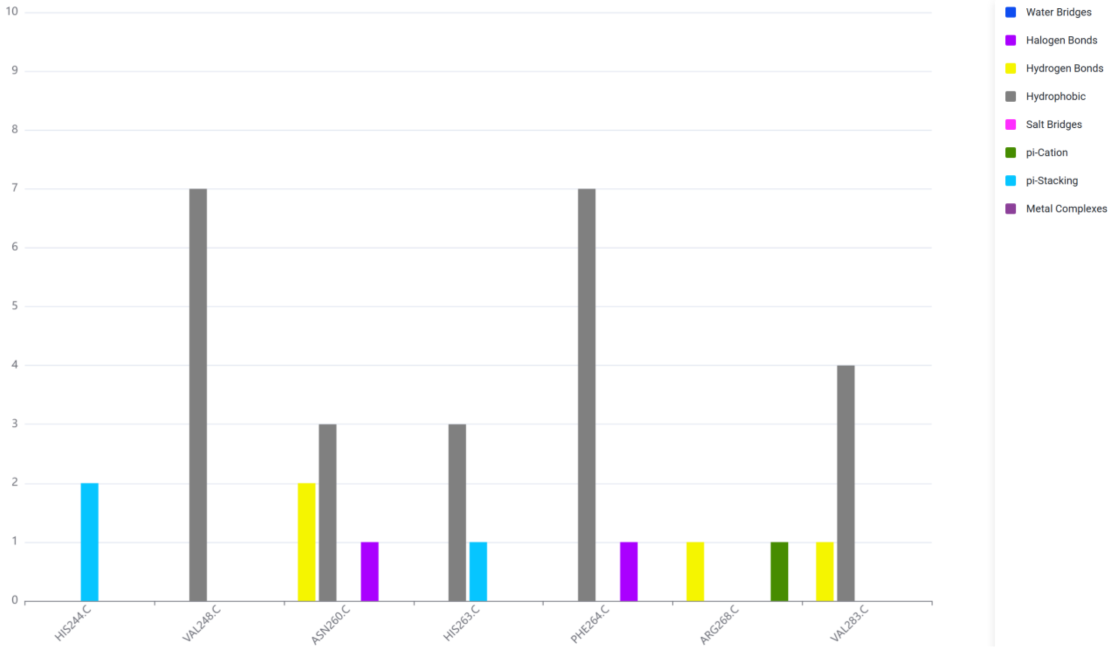

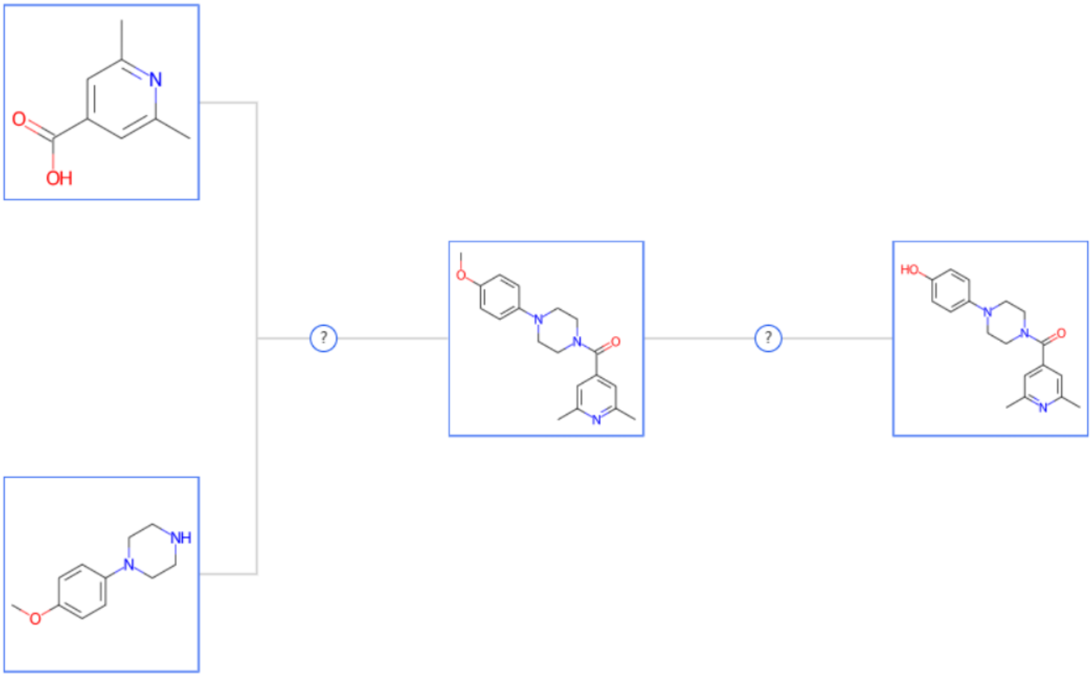

Alert Rule	Alert Structure	Reference
BMS Rule	✔	1. [1] Huth J R, Mendoza R, Olejniczak E T, et al. ALARM NMR: a rapid and robust experimental method to detect reactive false positives in biochemical screens[J]. Journal of the American Chemical Society, 2005, 127(1): 217-224.
Chelator Rule	✔	1. [1] Agrawal A, Johnson S L, Jacobsen J A, et al. Chelator fragment libraries for targeting metalloproteinases[J]. ChemMedChem: Chemistry Enabling Drug Discovery, 2010, 5(2): 195-199.
PAINS	✔	1. [1] Baell J B, Holloway G A. New substructure filters for removal of pan assay interference compounds (PAINS) from screening libraries and for their exclusion in bioassays[J]. Journal of medicinal chemistry, 2010, 53(7): 2719-2740.
Genotoxic Carcinogenicity Rule	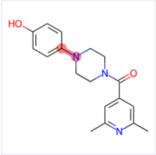	1. [1] Benigni R, Bossa C. Structure alerts for carcinogenicity, and the Salmonella assay system: a novel insight through the chemical relational databases technology[J]. Mutation Research/Reviews in Mutation Research, 2008, 659(3): 248-261.<br>2. [2] Ashby J, Tennant R W. Chemical structure, Salmonella mutagenicity and extent of carcinogenicity as indicators of genotoxic carcinogenesis among 222 chemicals tested in rodents by the US NCI/NTP[J]. Mutation Research/Genetic Toxicology, 1988, 204(1): 17-115.<br>3. [3] Kazius J, McGuire R, Bursi R. Derivation and validation of toxicophores for mutagenicity prediction[J]. Journal of medicinal chemistry, 2005, 48(1): 312-320.<br>4. [4] Bailey A B, Chanderbhan R, Collazo-Braier N, et al. The use of structure-activity relationship analysis in the food contact notification program[J]. Regulatory Toxicology and Pharmacology, 2005, 42(2): 225-235.
NTD	✔	1. [1] Brenk R, Schipani A, James D, et al. Lessons learnt from assembling screening libraries for drug discovery for neglected diseases[J]. ChemMedChem: Chemistry Enabling Drug Discovery, 2008, 3(3): 435-444.
SureChEMBL Rule	✔	1. [1] Sushko I, Salmina E, Potemkin V A, et al. ToxAlerts: a web server of structural alerts for toxic chemicals and compounds with potential adverse reactions[J]. 2012.

Compounds AI11:

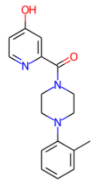

SMILES: Cc1ccccc1N1CCN(C(=O)c2cc(O)ccn2)CC1

**San ID**	No data
**CAS**	No data
**InChI**	InChI=1S/C17H19N3O2/c1-13-4-2-3-5-16(13)19-8-10-20(11-9-19)17(22)15-12-14(21)6-7-18-15/h2-7,12H,8-11H2,1H3,(H,18,21)
**InChIKey**	VTGIEMZSGRNDOI-UHFFFAOYSA-N

View in third-party databases: No data

MW	297.15	HBA	4	HBD	1
PAINS Alert	false	QED	0.92	NRot	2
TPSA	56.67	logD	2.16	logP	2.15
logS	-2.53				

Bad Average Good Scoring Notice

Absorption

Caco2	-4.69	HIA	100.00%	P-gp Inhibitor	27.15%
Bioavailability	100.00%	HFE	-9.68		

Distribution

BBB Penetration	78.50%	PPBR	1.00%	VDSS	1.30

Metabolism

CYP2D6 Inhibitor	9.89%	CYP3A4 Inhibitor	58.96%	CYP2C9 Inhibitor	70.52%
CYP2D6 Substrate	99.91%	CYP3A4 Substrate	47.24%	CYP2C9 Substrate	99.95%
CYP2C19 Inhibitor	74.35%	CYP1A2 Inhibitor	7.06%		

Excretion

Half Life (hour)	2.89	Clearance (mL/min/g)	53.91

Tox

hERG	85.44%	DILI	56.37%	NR-AhR	11.53%
Carcinogencity	12.92%	LD_{50}	509.00	Respiratory Toxicity	77.59%
Eye Corrosion	0.03%				

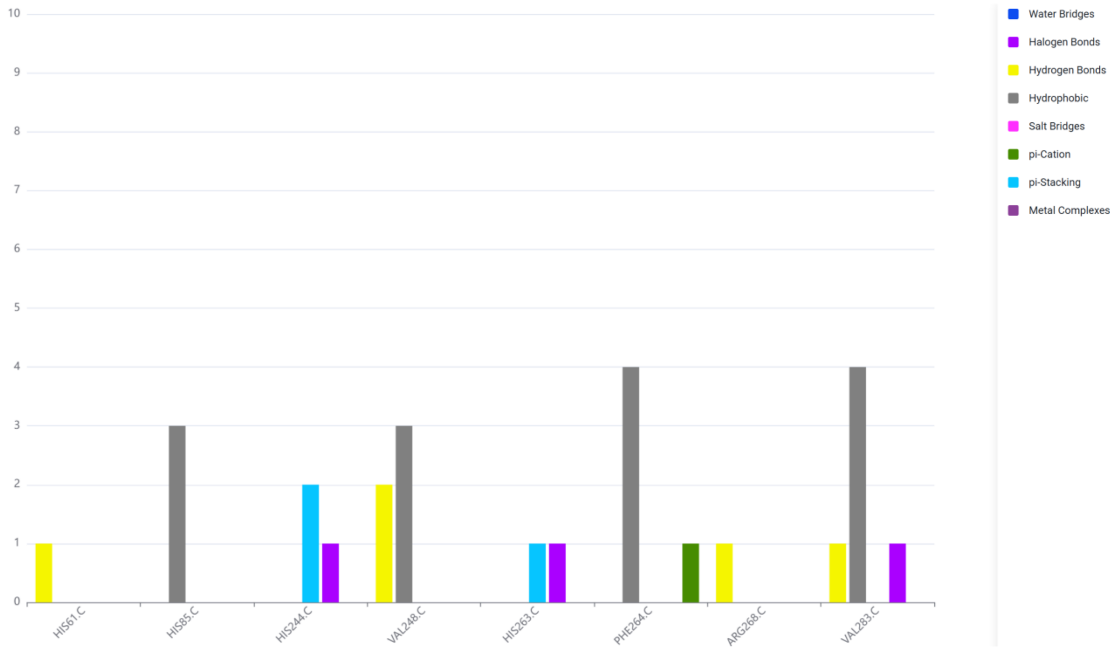

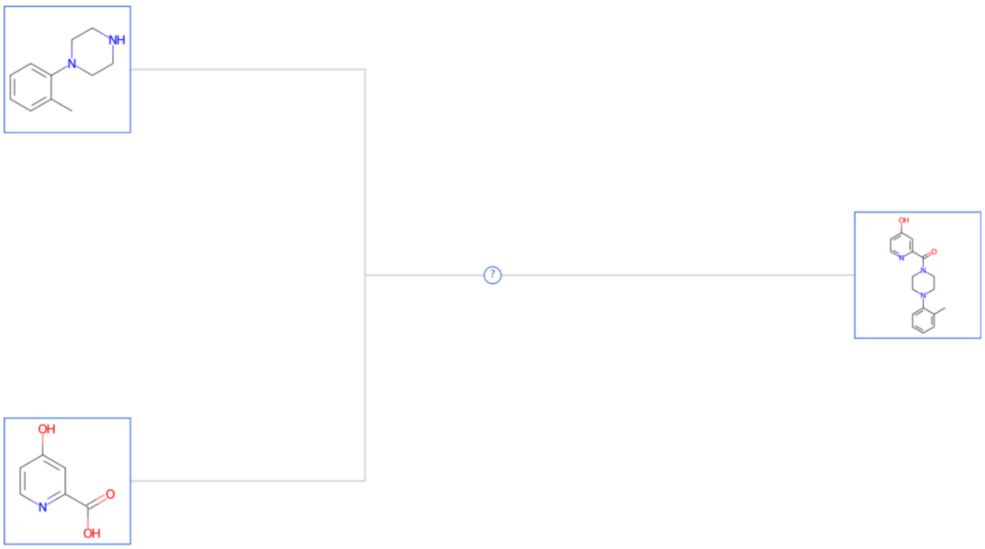

Alert Rule	Alert Structure	Reference
BMS Rule	✔	1. [1] Huth J R, Mendoza R, Olejniczak E T, et al. ALARM NMR: a rapid and robust experimental method to detect reactive false positives in biochemical screens[J]. Journal of the American Chemical Society, 2005, 127(1): 217-224.
Chelator Rule	✔	1. [1] Agrawal A, Johnson S L, Jacobsen J A, et al. Chelator fragment libraries for targeting metalloproteinases[J]. ChemMedChem: Chemistry Enabling Drug Discovery, 2010, 5(2): 195-199.
PAINS	✔	1. [1] Baell J B, Holloway G A. New substructure filters for removal of pan assay interference compounds (PAINS) from screening libraries and for their exclusion in bioassays[J]. Journal of medicinal chemistry, 2010, 53(7): 2719-2740.
Genotoxic Carcinogenicity Rule	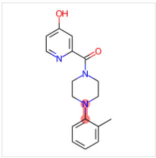	1. [1] Benigni R, Bossa C. Structure alerts for carcinogenicity, and the Salmonella assay system: a novel insight through the chemical relational databases technology[J]. Mutation Research/Reviews in Mutation Research, 2008, 659(3): 248-261.<br>2. [2] Ashby J, Tennant R W. Chemical structure, Salmonella mutagenicity and extent of carcinogenicity as indicators of genotoxic carcinogenesis among 222 chemicals tested in rodents by the US NCI/NTP[J]. Mutation Research/Genetic Toxicology, 1988, 204(1): 17-115.<br>3. [3] Kazius J, McGuire R, Bursi R. Derivation and validation of toxicophores for mutagenicity prediction[J]. Journal of medicinal chemistry, 2005, 48(1): 312-320.<br>4. [4] Bailey A B, Chanderbhan R, Collazo-Braier N, et al. The use of structure-activity relationship analysis in the food contact notification program[J]. Regulatory Toxicology and Pharmacology, 2005, 42(2): 225-235.
NTD	✔	1. [1] Brenk R, Schipani A, James D, et al. Lessons learnt from assembling screening libraries for drug discovery for neglected diseases[J]. ChemMedChem: Chemistry Enabling Drug Discovery, 2008, 3(3): 435-444.
SureChEMBL Rule	✔	1. [1] Sushko I, Salmina E, Potemkin V A, et al. ToxAlerts: a web server of structural alerts for toxic chemicals and compounds with potential adverse reactions[J]. 2012.

Compounds AI12:

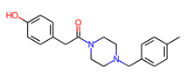

SMILES: Cc1ccc(CN2CCN(C(=O)Cc3ccc(O)cc3)CC2)cc1

**San ID**	No data
**CAS**	No data
**InChI**	InChI=1S/C20H24N2O2/c1-16-2-4-18(5-3-16)15-21-10-12-22(13-11-21)20(24)14-17-6-8-19(23)9-7-17/h2-9,23H,10-15H2,1H3
**InChIKey**	DRSOBYPZTVNHPQ-UHFFFAOYSA-N

View in third-party databases: Loading...

MW	324.18	HBA	3	HBD	1
PAINS Alert	false	QED	0.94	NRot	4
TPSA	43.78	logD	2.55	logP	2.7
logS	-2.49				

Bad Average Good Scoring Notice

Absorption

Caco2	-4.81	HIA	100.00%	P-gp Inhibitor	66.33%
Bioavailability	79.03%	HFE	-9.34		

Distribution

BBB Penetration	85.83%	PPBR	1.00%	VDSS	1.70

Metabolism

CYP2D6 Inhibitor	65.26%	CYP3A4 Inhibitor	50.81%	CYP2C9 Inhibitor	49.86%
CYP2D6 Substrate	36.59%	CYP3A4 Substrate	38.59%	CYP2C9 Substrate	12.10%
CYP2C19 Inhibitor	72.03%	CYP1A2 Inhibitor	14.36%		

Excretion

Half Life (hour)	3.61	Clearance (mL/min/g)	45.18

Tox

hERG	93.73%	DILI	19.71%	NR-AhR	7.62%
Carcinogencity	1.33%	LD_{50}	347.76	Respiratory Toxicity	86.70%
Eye Corrosion	0.04%				

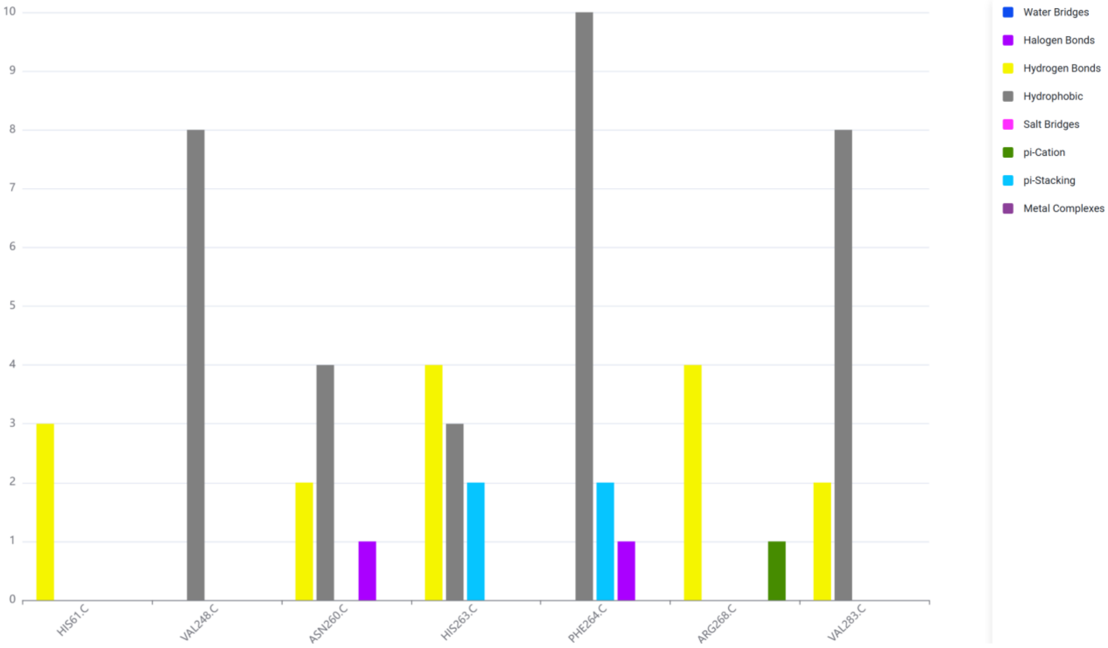

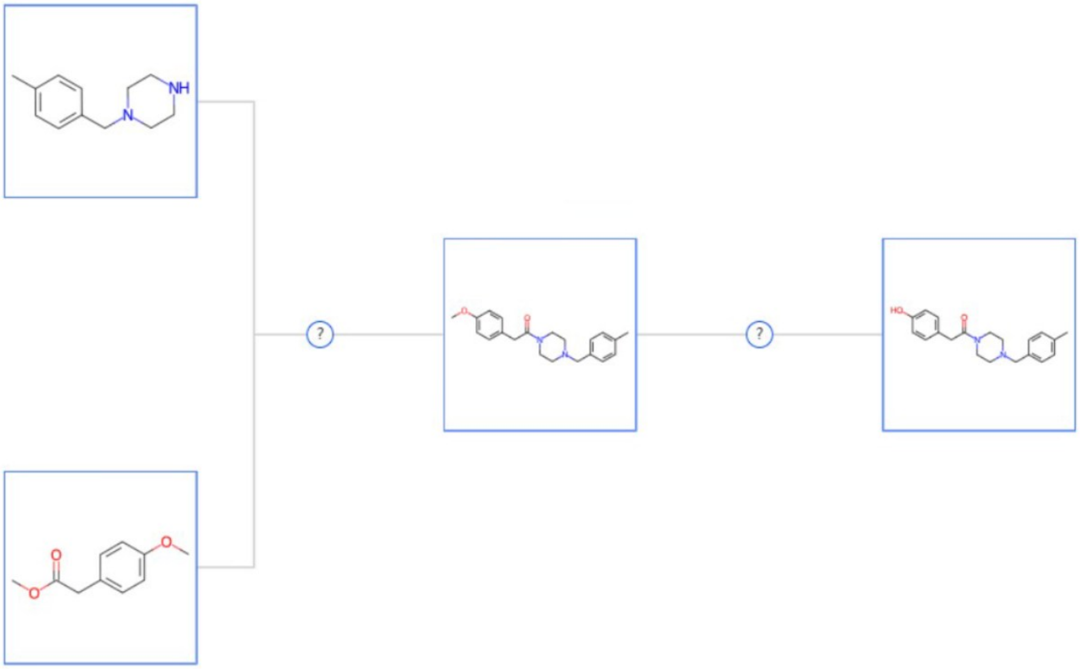

Alert Rule	Alert Structure	Reference
BMS Rule	✅	1. [1] Huth J R, Mendoza R, Olejniczak E T, et al. ALARM NMR: a rapid and robust experimental method to detect reactive false positives in biochemical screens[J]. Journal of the American Chemical Society, 2005, 127(1): 217-224.
Chelator Rule	✅	1. [1] Agrawal A, Johnson S L, Jacobsen J A, et al. Chelator fragment libraries for targeting metalloproteinases[J]. ChemMedChem: Chemistry Enabling Drug Discovery, 2010, 5(2): 195-199.
PAINS	✅	1. [1] Baell J B, Holloway G A. New substructure filters for removal of pan assay interference compounds (PAINS) from screening libraries and for their exclusion in bioassays[J]. Journal of medicinal chemistry, 2010, 53(7): 2719-2740.
Genotoxic Carcinogenicity Rule	✅	1. [1] Benigni R, Bossa C. Structure alerts for carcinogenicity, and the Salmonella assay system: a novel insight through the chemical relational databases technology[J]. Mutation Research/Reviews in Mutation Research, 2008, 659(3): 248-261.<br>2. [2] Ashby J, Tennant R W. Chemical structure, Salmonella mutagenicity and extent of carcinogenicity as indicators of genotoxic carcinogenesis among 222 chemicals tested in rodents by the US NCI/NTP[J]. Mutation Research/Genetic Toxicology, 1988, 204(1): 17-115.<br>3. [3] Kazius J, McGuire R, Bursi R. Derivation and validation of toxicophores for mutagenicity prediction[J]. Journal of medicinal chemistry, 2005, 48(1): 312-320.<br>4. [4] Bailey A B, Chanderbhan R, Collazo-Braier N, et al. The use of structure-activity relationship analysis in the food contact notification program[J]. Regulatory Toxicology and Pharmacology, 2005, 42(2): 225-235.
NTD	✅	1. [1] Brenk R, Schipani A, James D, et al. Lessons learnt from assembling screening libraries for drug discovery for neglected diseases[J]. ChemMedChem: Chemistry Enabling Drug Discovery, 2008, 3(3): 435-444.
SureChEMBL Rule	✅	1. [1] Sushko I, Salmina E, Potemkin V A, et al. ToxAlerts: a web server of structural alerts for toxic chemicals and compounds with potential adverse reactions[J]. 2012.

Compounds AI13:

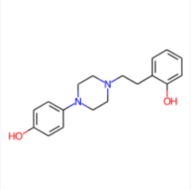

SMILES: Oc1ccc(N2CCN(CCc3ccccc3O)CC2)cc1

San ID	No data
CAS	No data
InChI	InChI=1S/C18H22N2O2/c21-17-7-5-16(6-8-17)20-13-11-19(12-14-20)10-9-15-3-1-2-4-18(15)22/h1-8,21-22H,9-14H2
InChIKey	SDCTWJBYGCCHJJ-UHFFFAOYSA-N

View in third-party databases: No data

MW	298.17	HBA	4	HBD	2
PAINS Alert	false	QED	0.91	NRot	4
TPSA	46.94	logD	1.92	logP	2.18
logS	-2.32				

Bad Average Good Scoring Notice

Absorption

Caco2	-4.95	HIA	100.00%	P-gp Inhibitor	76.25%
Bioavailability	15.99%	HFE	-10.41		

Distribution

BBB Penetration	82.74%	PPBR	1.00%	VDSS	2.33

Metabolism

CYP2D6 Inhibitor	39.38%	CYP3A4 Inhibitor	26.27%	CYP2C9 Inhibitor	27.28%
CYP2D6 Substrate	95.88%	CYP3A4 Substrate	0.00%	CYP2C9 Substrate	15.70%
CYP2C19 Inhibitor	39.00%	CYP1A2 Inhibitor	15.30%		

Excretion

Half Life (hour)	3.77	Clearance (mL/min/g)	45.78

Tox

hERG	95.73%	DILI	7.91%	NR-AhR	38.10%
Carcinogencity	0.08%	LD_{50}	719.96	Respiratory Toxicity	99.80%
Eye Corrosion	11.23%				

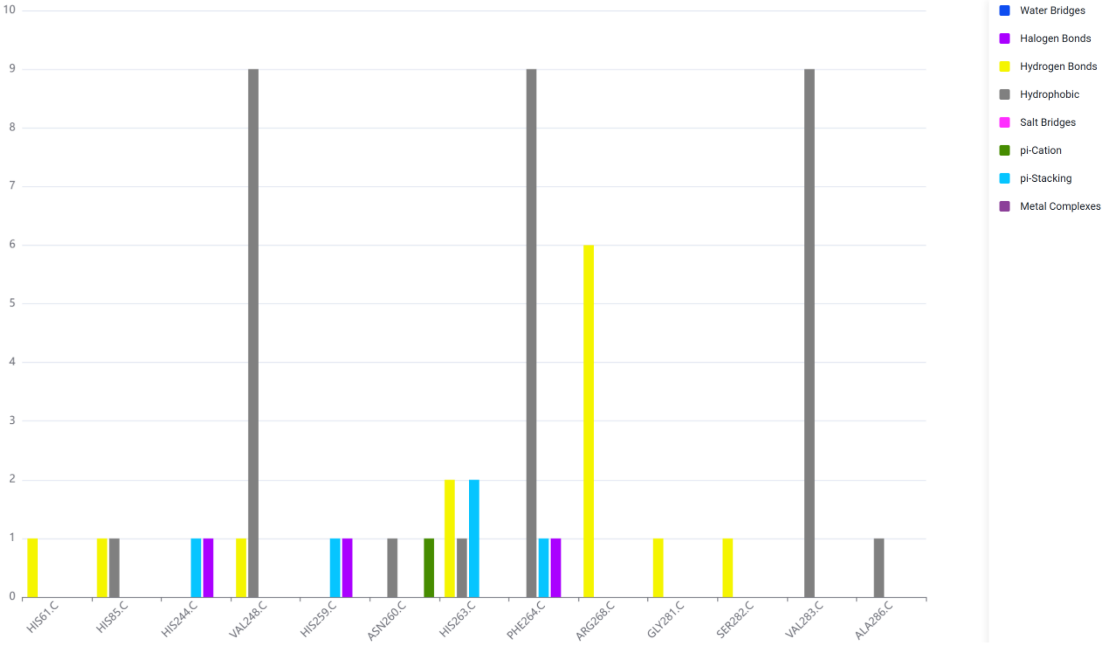

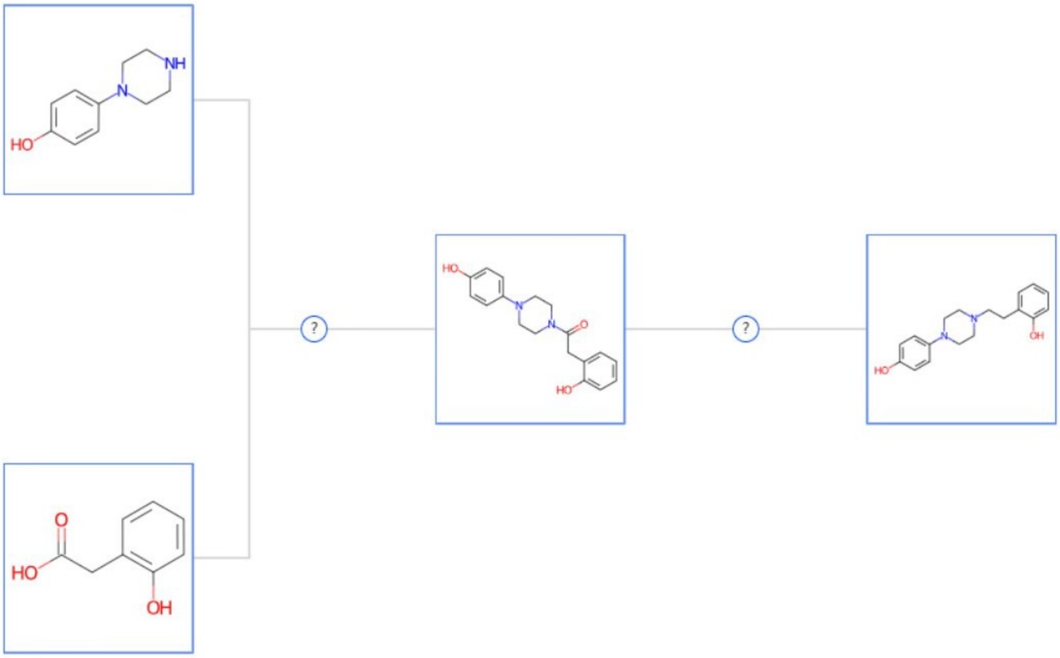

Alert Rule	Alert Structure	Reference
BMS Rule	✓	1. [1] Huth J R, Mendoza R, Olejniczak E T, et al. ALARM NMR: a rapid and robust experimental method to detect reactive false positives in biochemical screens[J]. Journal of the American Chemical Society, 2005, 127(1): 217-224.
Chelator Rule	✓	1. [1] Agrawal A, Johnson S L, Jacobsen J A, et al. Chelator fragment libraries for targeting metalloproteinases[J]. ChemMedChem: Chemistry Enabling Drug Discovery, 2010, 5(2): 195-199.
PAINS	✓	1. [1] Baell J B, Holloway G A. New substructure filters for removal of pan assay interference compounds (PAINS) from screening libraries and for their exclusion in bioassays[J]. Journal of medicinal chemistry, 2010, 53(7): 2719-2740.
Genotoxic Carcinogenicity Rule	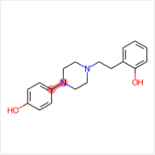	1. [1] Benigni R, Bossa C. Structure alerts for carcinogenicity, and the Salmonella assay system: a novel insight through the chemical relational databases technology[J]. Mutation Research/Reviews in Mutation Research, 2008, 659(3): 248-261.<br>2. [2] Ashby J, Tennant R W. Chemical structure, Salmonella mutagenicity and extent of carcinogenicity as indicators of genotoxic carcinogenesis among 222 chemicals tested in rodents by the US NCI/NTP[J]. Mutation Research/Genetic Toxicology, 1988, 204(1): 17-115.<br>3. [3] Kazius J, McGuire R, Bursi R. Derivation and validation of toxicophores for mutagenicity prediction[J]. Journal of medicinal chemistry, 2005, 48(1): 312-320.<br>4. [4] Bailey A B, Chanderbhan R, Collazo-Braier N, et al. The use of structure-activity relationship analysis in the food contact notification program[J]. Regulatory Toxicology and Pharmacology, 2005, 42(2): 225-235.
NTD	✓	1. [1] Brenk R, Schipani A, James D, et al. Lessons learnt from assembling screening libraries for drug discovery for neglected diseases[J]. ChemMedChem: Chemistry Enabling Drug Discovery, 2008, 3(3): 435-444.
SureChEMBL Rule	✓	1. [1] Sushko I, Salmina E, Potemkin V A, et al. ToxAlerts: a web server of structural alerts for toxic chemicals and compounds with potential adverse reactions[J]. 2012.

Compounds AI14:

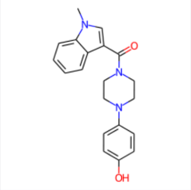

SMILES: Cn1cc(C(=O)N2CCN(c3ccc(O)cc3)CC2)c2ccccc21

San ID	No data
CAS	No data
InChI	InChI=1S/C20H21N3O2/c1-21-14-18(17-4-2-3-5-19(17)21)20(25)23-12-10-22(11-13-23)15-6-8-16(24)9-7-15/h2-9,14,24H,10-13H2,1H3
InChIKey	FLXDKFUGTIBEBW-UHFFFAOYSA-N

View in third-party databases: PubChem

MW	335.16	HBA	4	HBD	1
PAINS Alert	false	QED	0.78	NRot	2
TPSA	48.71	logD	2.17	logP	2.17
logS	-3.24				

Bad Average Good Scoring Notice

Absorption

Caco2	-4.82	HIA	100.00%	P-gp Inhibitor	54.55%
Bioavailability	99.95%	HFE	-10.90		

Distribution

BBB Penetration	89.01%	PPBR	1.00%	VDSS	1.15

Metabolism

CYP2D6 Inhibitor	8.08%	CYP3A4 Inhibitor	80.42%	CYP2C9 Inhibitor	76.93%
CYP2D6 Substrate	94.50%	CYP3A4 Substrate	69.35%	CYP2C9 Substrate	99.99%
CYP2C19 Inhibitor	82.45%	CYP1A2 Inhibitor	45.78%		

Excretion

Half Life (hour)	4.47	Clearance (mL/min/g)	52.93

Tox

hERG	96.21%	DILI	74.41%	NR-AhR	46.11%
Carcinogencity	6.29%	LD_{50}	1316.62	Respiratory Toxicity	98.00%
Eye Corrosion	0.02%				

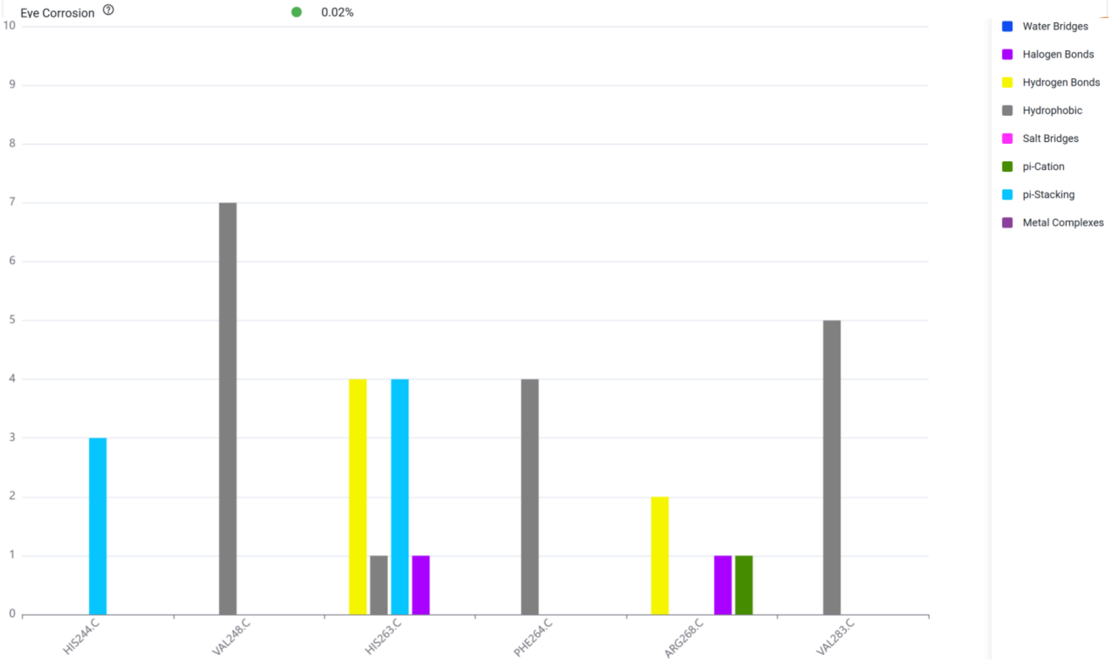

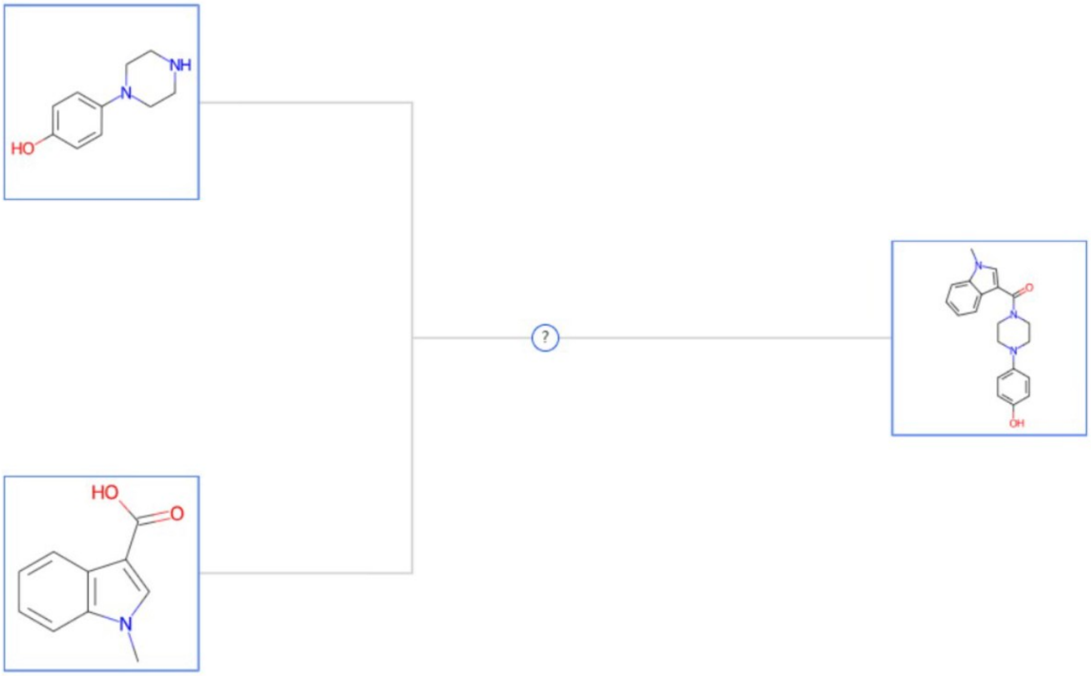

Alert Rule	Alert Structure	Reference
BMS Rule	✔	1. [1] Huth J R, Mendoza R, Olejniczak E T, et al. ALARM NMR: a rapid and robust experimental method to detect reactive false positives in biochemical screens[J]. Journal of the American Chemical Society, 2005, 127(1): 217-224.
Chelator Rule	✔	1. [1] Agrawal A, Johnson S L, Jacobsen J A, et al. Chelator fragment libraries for targeting metalloproteinases[J]. ChemMedChem: Chemistry Enabling Drug Discovery, 2010, 5(2): 195-199.
PAINS	✔	1. [1] Baell J B, Holloway G A. New substructure filters for removal of pan assay interference compounds (PAINS) from screening libraries and for their exclusion in bioassays[J]. Journal of medicinal chemistry, 2010, 53(7): 2719-2740.
Genotoxic Carcinogenicity Rule	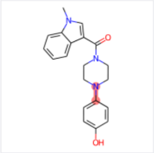	1. [1] Benigni R, Bossa C. Structure alerts for carcinogenicity, and the Salmonella assay system: a novel insight through the chemical relational databases technology[J]. Mutation Research/Reviews in Mutation Research, 2008, 659(3): 248-261.<br>2. [2] Ashby J, Tennant R W. Chemical structure, Salmonella mutagenicity and extent of carcinogenicity as indicators of genotoxic carcinogenesis among 222 chemicals tested in rodents by the US NCI/NTP[J]. Mutation Research/Genetic Toxicology, 1988, 204(1): 17-115.<br>3. [3] Kazius J, McGuire R, Bursi R. Derivation and validation of toxicophores for mutagenicity prediction[J]. Journal of medicinal chemistry, 2005, 48(1): 312-320.<br>4. [4] Bailey A B, Chanderbhan R, Collazo-Braier N, et al. The use of structure-activity relationship analysis in the food contact notification program[J]. Regulatory Toxicology and Pharmacology, 2005, 42(2): 225-235.
NTD	✔	1. [1] Brenk R, Schipani A, James D, et al. Lessons learnt from assembling screening libraries for drug discovery for neglected diseases[J]. ChemMedChem: Chemistry Enabling Drug Discovery, 2008, 3(3): 435-444.
SureChEMBL Rule	✔	1. [1] Sushko I, Salmina E, Potemkin V A, et al. ToxAlerts: a web server of structural alerts for toxic chemicals and compounds with potential adverse reactions[J]. 2012.

Compounds AI15:

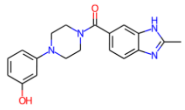

SMILES: Cc1nc2ccc(C(=O)N3CCN(c4cccc(O)c4)CC3)cc2[nH]1

San ID	No data
CAS	No data
InChI	InChI=1S/C19H20N4O2/c1-13-20-17-6-5-14(11-18(17)21-13)19(25)23-9-7-22(8-10-23)15-3-2-4-16(24)12-15/h2-6,11-12,24H,7-10H2,1H3,(H,20,21)
InChIKey	VBNBEYZIKBYSPO-UHFFFAOYSA-N

View in third-party databases: Loading...

MW	336.16	HBA	4	HBD	2
PAINS Alert	false	QED	0.75	NRot	2
TPSA	72.46	logD	1.51	logP	2.01
logS	-4.10				

Bad Average Good Scoring Notice

Absorption

Caco2	-4.88	HIA	100.00%	P-gp Inhibitor	17.46%
Bioavailability	100.00%	HFE	-11.83		

Distribution

BBB Penetration	60.75%	PPBR	1.00%	VDSS	0.66

Metabolism

CYP2D6 Inhibitor	5.46%	CYP3A4 Inhibitor	63.02%	CYP2C9 Inhibitor	60.76%
CYP2D6 Substrate	89.92%	CYP3A4 Substrate	76.80%	CYP2C9 Substrate	0.97%
CYP2C19 Inhibitor	61.56%	CYP1A2 Inhibitor	31.61%		

Excretion

Half Life (hour)	2.77	Clearance (mL/min/g)	58.25

Tox

hERG	97.66%	DILI	62.33%	NR-AhR	41.04%
Carcinogencity	1.87%	LD_{50}	667.17	Respiratory Toxicity	93.09%
Eye Corrosion	0.02%				

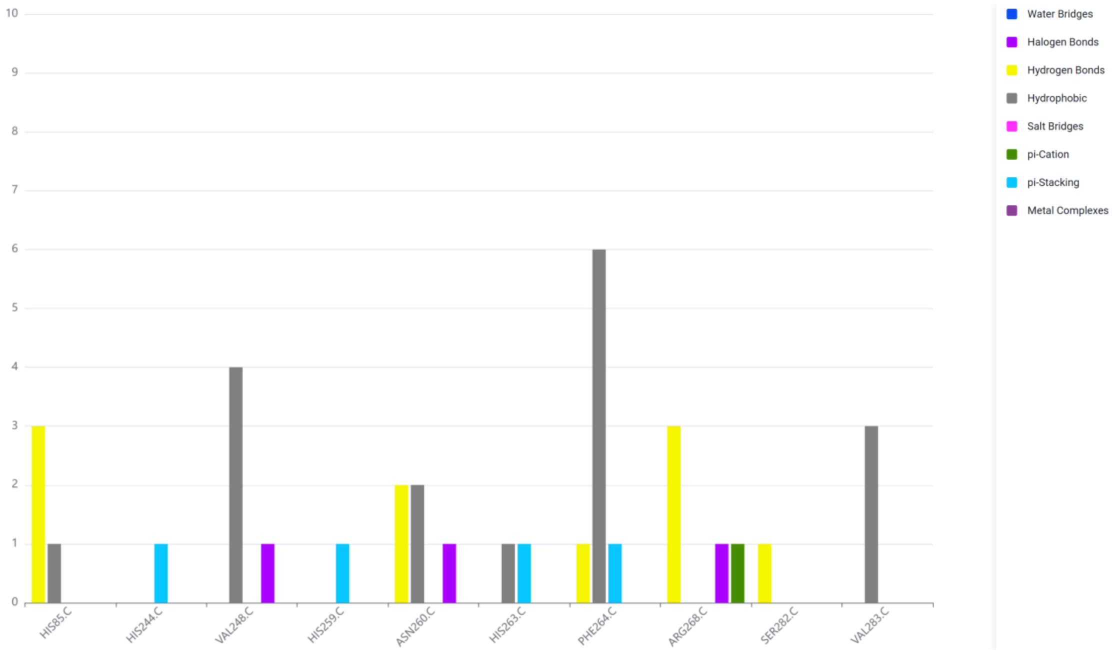

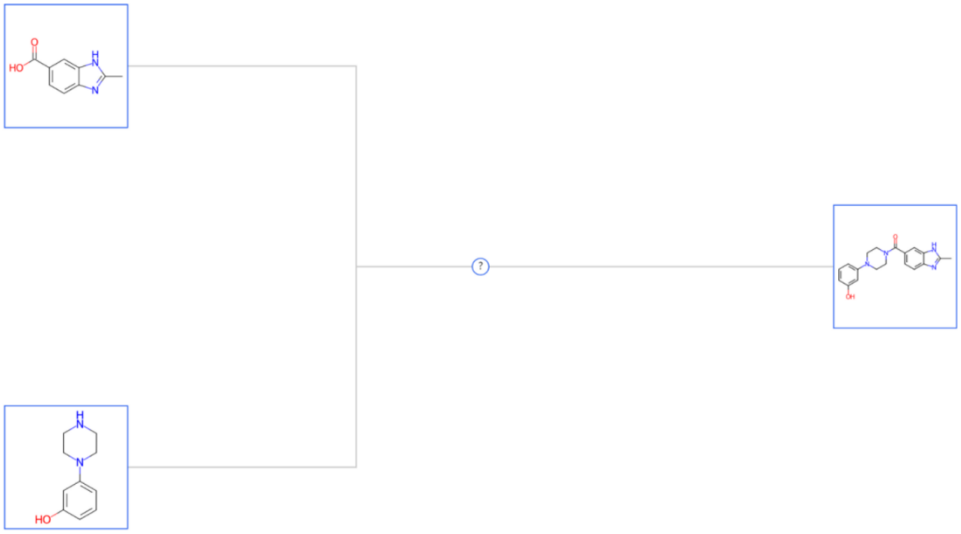

Alert Rule	Alert Structure	Reference
BMS Rule	✓	1. [1] Huth J R, Mendoza R, Olejniczak E T, et al. ALARM NMR: a rapid and robust experimental method to detect reactive false positives in biochemical screens[J]. Journal of the American Chemical Society, 2005, 127(1): 217-224.
Chelator Rule	✓	1. [1] Agrawal A, Johnson S L, Jacobsen J A, et al. Chelator fragment libraries for targeting metalloproteinases[J]. ChemMedChem: Chemistry Enabling Drug Discovery, 2010, 5(2): 195-199.
PAINS	✓	1. [1] Baell J B, Holloway G A. New substructure filters for removal of pan assay interference compounds (PAINS) from screening libraries and for their exclusion in bioassays[J]. Journal of medicinal chemistry, 2010, 53(7): 2719-2740.
Genotoxic Carcinogenicity Rule	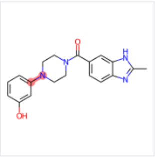	1. [1] Benigni R, Bossa C. Structure alerts for carcinogenicity, and the Salmonella assay system: a novel insight through the chemical relational databases technology[J]. Mutation Research/Reviews in Mutation Research, 2008, 659(3): 248-261. 2. [2] Ashby J, Tennant R W. Chemical structure, Salmonella mutagenicity and extent of carcinogenicity as indicators of genotoxic carcinogenesis among 222 chemicals tested in rodents by the US NCI/NTP[J]. Mutation Research/Genetic Toxicology, 1988, 204(1): 17-115. 3. [3] Kazius J, McGuire R, Bursi R. Derivation and validation of toxicophores for mutagenicity prediction[J]. Journal of medicinal chemistry, 2005, 48(1): 312-320. 4. [4] Bailey A B, Chanderbhan R, Collazo-Braier N, et al. The use of structure-activity relationship analysis in the food contact notification program[J]. Regulatory Toxicology and Pharmacology, 2005, 42(2): 225-235.
NTD	✓	1. [1] Brenk R, Schipani A, James D, et al. Lessons learnt from assembling screening libraries for drug discovery for neglected diseases[J]. ChemMedChem: Chemistry Enabling Drug Discovery, 2008, 3(3): 435-444.
SureChEMBL Rule	✓	1. [1] Sushko I, Salmina E, Potemkin V A, et al. ToxAlerts: a web server of structural alerts for toxic chemicals and compounds with potential adverse reactions[J]. 2012.

Compounds AI16:

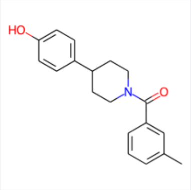

SMILES: Cc1cccc(C(=O)N2CCC(c3ccc(O)cc3)CC2)c1

San ID	No data
CAS	No data
InChI	InChI=1S/C19H21NO2/c1-14-3-2-4-17(13-14)19(22)20-11-9-16(10-12-20)15-5-7-18(21)8-6-15/h2-8,13,16,21H,9-12H2,1H3
InChIKey	MDTVXEHNINWTMP-UHFFFAOYSA-N

View in third-party databases: Loading...

MW	295.16	HBA	2	HBD	1
PAINS Alert	false	QED	0.91	NRot	2
TPSA	40.54	logD	3.42	logP	3.59
logS	-4.10				

Bad Average Good Scoring Notice

Absorption

Caco2	-4.60	HIA	99.60%	P-gp Inhibitor	51.28%
Bioavailability	97.75%	HFE	-9.41		

Distribution

BBB Penetration	80.76%	PPBR	1.00%	VDSS	1.75

Metabolism

CYP2D6 Inhibitor	12.46%	CYP3A4 Inhibitor	83.09%	CYP2C9 Inhibitor	74.69%
CYP2D6 Substrate	98.98%	CYP3A4 Substrate	40.91%	CYP2C9 Substrate	0.38%
CYP2C19 Inhibitor	84.47%	CYP1A2 Inhibitor	29.52%		

Excretion

Half Life (hour)	3.60	Clearance (mL/min/g)	44.54

Tox

hERG	92.61%	DILI	33.89%	NR-AhR	13.02%
Carcinogencity	16.37%	LD_{50}	503.56	Respiratory Toxicity	1.50%
Eye Corrosion	0.01%				

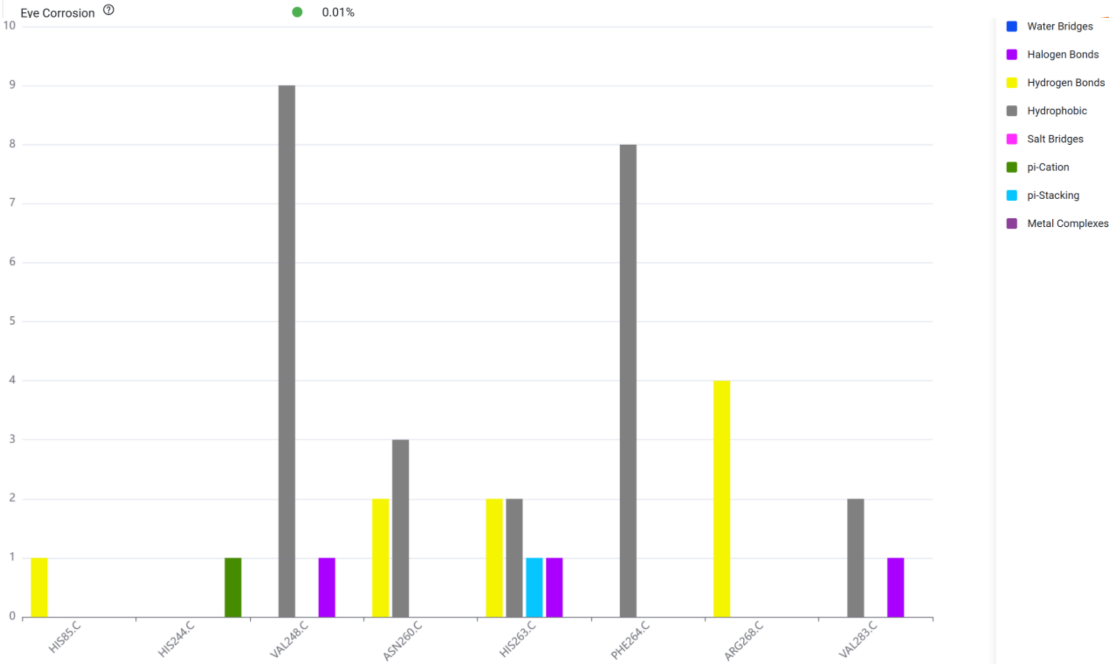

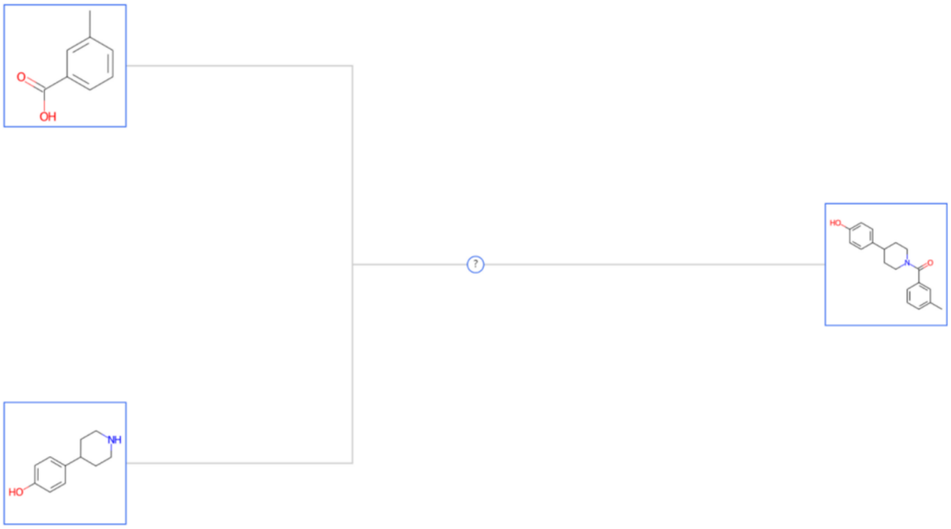

Alert Rule	Alert Structure	Reference
BMS Rule	✔	1. [1] Huth J R, Mendoza R, Olejniczak E T, et al. ALARM NMR: a rapid and robust experimental method to detect reactive false positives in biochemical screens[J]. Journal of the American Chemical Society, 2005, 127(1): 217-224.
Chelator Rule	✔	1. [1] Agrawal A, Johnson S L, Jacobsen J A, et al. Chelator fragment libraries for targeting metalloproteinases[J]. ChemMedChem: Chemistry Enabling Drug Discovery, 2010, 5(2): 195-199.
PAINS	✔	1. [1] Baell J B, Holloway G A. New substructure filters for removal of pan assay interference compounds (PAINS) from screening libraries and for their exclusion in bioassays[J]. Journal of medicinal chemistry, 2010, 53(7): 2719-2740.
Genotoxic Carcinogenicity Rule	✔	1. [1] Benigni R, Bossa C. Structure alerts for carcinogenicity, and the Salmonella assay system: a novel insight through the chemical relational databases technology[J]. Mutation Research/Reviews in Mutation Research, 2008, 659(3): 248-261.<br>2. [2] Ashby J, Tennant R W. Chemical structure, Salmonella mutagenicity and extent of carcinogenicity as indicators of genotoxic carcinogenesis among 222 chemicals tested in rodents by the US NCI/NTP[J]. Mutation Research/Genetic Toxicology, 1988, 204(1): 17-115.<br>3. [3] Kazius J, McGuire R, Bursi R. Derivation and validation of toxicophores for mutagenicity prediction[J]. Journal of medicinal chemistry, 2005, 48(1): 312-320.<br>4. [4] Bailey A B, Chanderbhan R, Collazo-Braier N, et al. The use of structure-activity relationship analysis in the food contact notification program[J]. Regulatory Toxicology and Pharmacology, 2005, 42(2): 225-235.
NTD	✔	1. [1] Brenk R, Schipani A, James D, et al. Lessons learnt from assembling screening libraries for drug discovery for neglected diseases[J]. ChemMedChem: Chemistry Enabling Drug Discovery, 2008, 3(3): 435-444.
SureChEMBL Rule	✔	1. [1] Sushko I, Salmina E, Potemkin V A, et al. ToxAlerts: a web server of structural alerts for toxic chemicals and compounds with potential adverse reactions[J]. 2012.

Compounds AI17:

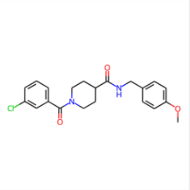

SMILES: COc1ccc(CNC(=O)C2CCN(C(=O)c3cccc(Cl)c3)CC2)cc1

San ID	f3d2e5be8511ec5977c3dfcf750a6f31
CAS	No data
InChI	InChI=1S/C21H23ClN2O3/c1-27-19-7-5-15(6-8-19)14-23-20(25)16-9-11-24(12-10-16)21(26)17-3-2-4-18(22)13-17/h2-8,13,16H,9-12,14H2,1H3,(H,...
InChIKey	FMSJCMWOYABMRX-UHFFFAOYSA-N

View in third-party databases: Loading...

MW	386.14	HBA	3	HBD	1
PAINS Alert	false	QED	0.85	NRot	5
TPSA	58.64	logD	3.05	logP	3.59
logS	-4.00				

Bad Average Good Scoring Notice

Absorption

Caco2	-4.89	HIA	100.00%	P-gp Inhibitor	63.93%
Bioavailability	99.39%	HFE	-11.23		

Distribution

BBB Penetration	81.76%	PPBR	1.00%	VDSS	1.20

Metabolism

CYP2D6 Inhibitor	18.51%	CYP3A4 Inhibitor	89.18%	CYP2C9 Inhibitor	41.46%
CYP2D6 Substrate	31.94%	CYP3A4 Substrate	72.74%	CYP2C9 Substrate	0.00%
CYP2C19 Inhibitor	73.93%	CYP1A2 Inhibitor	7.13%		

Excretion

Half Life (hour)	4.80	Clearance (mL/min/g)	47.42

Tox

hERG	88.18%	DILI	57.21%	NR-AhR	8.58%
Carcinogencity	0.16%	LD_{50}	729.08	Respiratory Toxicity	2.97%
Eye Corrosion	0.08%				

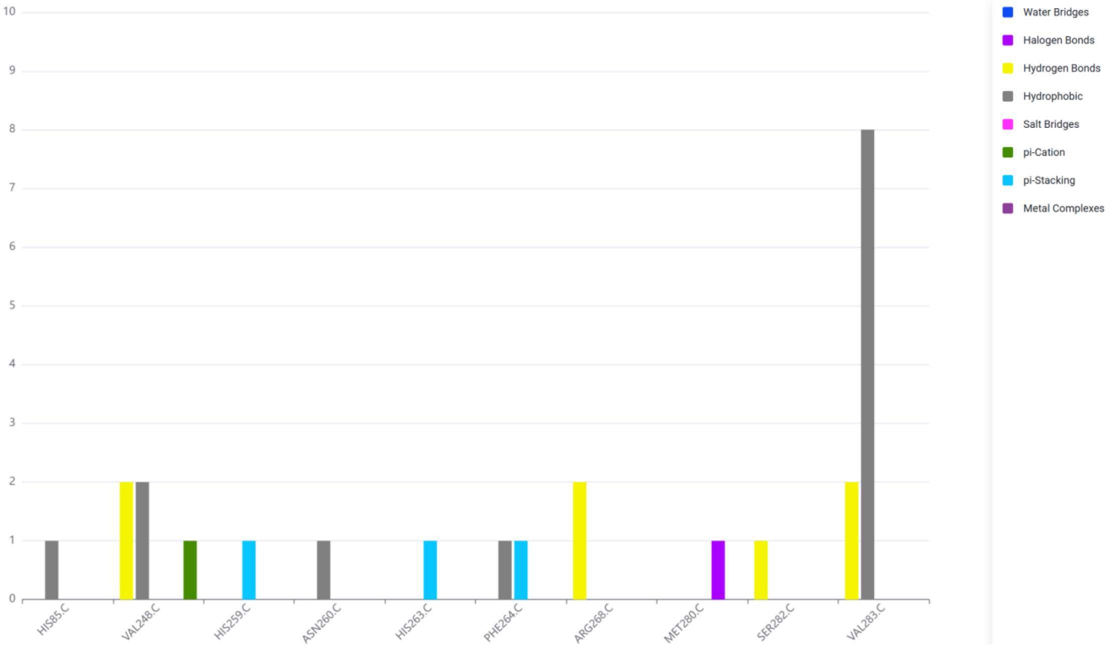

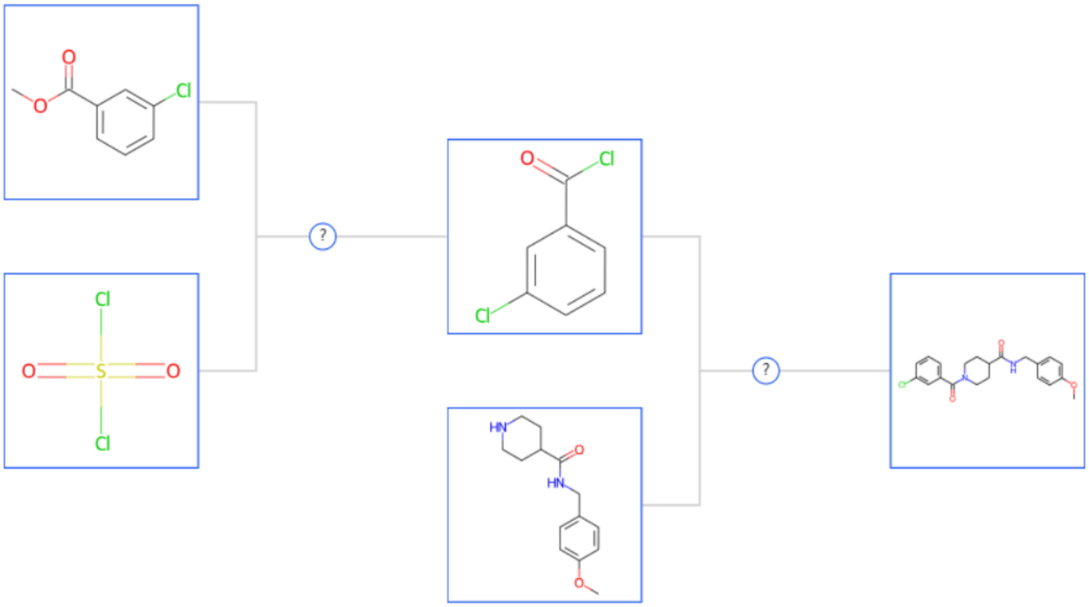

Alert Rule	Alert Structure	Reference
BMS Rule	✓	1. [1] Huth J R, Mendoza R, Olejniczak E T, et al. ALARM NMR: a rapid and robust experimental method to detect reactive false positives in biochemical screens[J]. Journal of the American Chemical Society, 2005, 127(1): 217-224.
Chelator Rule	✓	1. [1] Agrawal A, Johnson S L, Jacobsen J A, et al. Chelator fragment libraries for targeting metalloproteinases[J]. ChemMedChem: Chemistry Enabling Drug Discovery, 2010, 5(2): 195-199.
PAINS	✓	1. [1] Baell J B, Holloway G A. New substructure filters for removal of pan assay interference compounds (PAINS) from screening libraries and for their exclusion in bioassays[J]. Journal of medicinal chemistry, 2010, 53(7): 2719-2740.
Genotoxic Carcinogenicity Rule	✓	1. [1] Benigni R, Bossa C. Structure alerts for carcinogenicity, and the Salmonella assay system: a novel insight through the chemical relational databases technology[J]. Mutation Research/Reviews in Mutation Research, 2008, 659(3): 248-261.<br>2. [2] Ashby J, Tennant R W. Chemical structure, Salmonella mutagenicity and extent of carcinogenicity as indicators of genotoxic carcinogenesis among 222 chemicals tested in rodents by the US NCI/NTP[J]. Mutation Research/Genetic Toxicology, 1988, 204(1): 17-115.<br>3. [3] Kazius J, McGuire R, Bursi R. Derivation and validation of toxicophores for mutagenicity prediction[J]. Journal of medicinal chemistry, 2005, 48(1): 312-320.<br>4. [4] Bailey A B, Chanderbhan R, Collazo-Braier N, et al. The use of structure-activity relationship analysis in the food contact notification program[J]. Regulatory Toxicology and Pharmacology, 2005, 42(2): 225-235.
NTD	✓	1. [1] Brenk R, Schipani A, James D, et al. Lessons learnt from assembling screening libraries for drug discovery for neglected diseases[J]. ChemMedChem: Chemistry Enabling Drug Discovery, 2008, 3(3): 435-444.
SureChEMBL Rule	✓	1. [1] Sushko I, Salmina E, Potemkin V A, et al. ToxAlerts: a web server of structural alerts for toxic chemicals and compounds with potential adverse reactions[J]. 2012.

Compounds AI18:

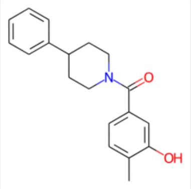

SMILES: Cc1ccc(C(=O)N2CCC(c3ccccc3)CC2)cc1O

San ID	No data
CAS	No data
InChI	InChI=1S/C19H21NO2/c1-14-7-8-17(13-18(14)21)19(22)20-11-9-16(10-12-20)15-5-3-2-4-6-15/h2-8,13,16,21H,9-12H2,1H3
InChIKey	LPDASUAPGIILCJ-UHFFFAOYSA-N

View in third-party databases: Loading...

MW	295.16	HBA	2	HBD	1
PAINS Alert	false	QED	0.91	NRot	2
TPSA	40.54	logD	3.43	logP	3.39
logS	-4.07				

Bad Average Good Scoring Notice

Absorption

Caco2	-4.67	HIA	99.68%	P-gp Inhibitor	65.32%
Bioavailability	97.66%	HFE	-9.29		

Distribution

BBB Penetration	82.69%	PPBR	1.00%	VDSS	2.14

Metabolism

CYP2D6 Inhibitor	19.99%	CYP3A4 Inhibitor	73.66%	CYP2C9 Inhibitor	76.19%
CYP2D6 Substrate	99.51%	CYP3A4 Substrate	41.32%	CYP2C9 Substrate	0.73%
CYP2C19 Inhibitor	86.67%	CYP1A2 Inhibitor	40.19%		

Excretion

Half Life (hour)	3.80	Clearance (mL/min/g)	46.79

Tox

hERG	92.68%	DILI	36.24%	NR-AhR	11.49%
Carcinogencity	6.90%	LD_{50}	490.31	Respiratory Toxicity	1.45%
Eye Corrosion	0.01%				

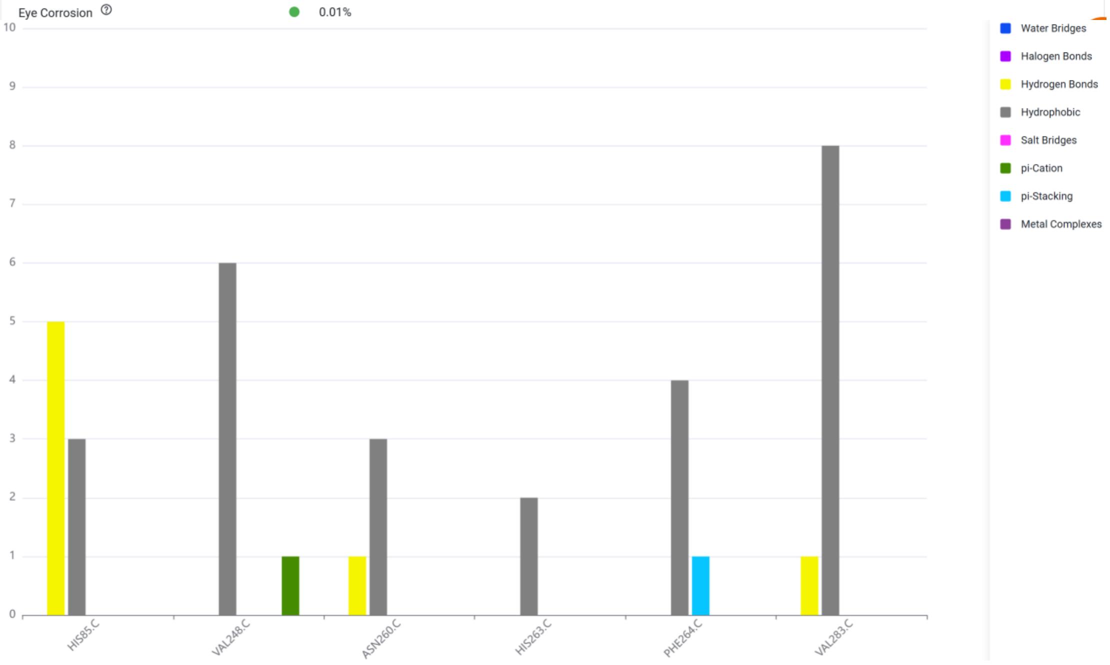

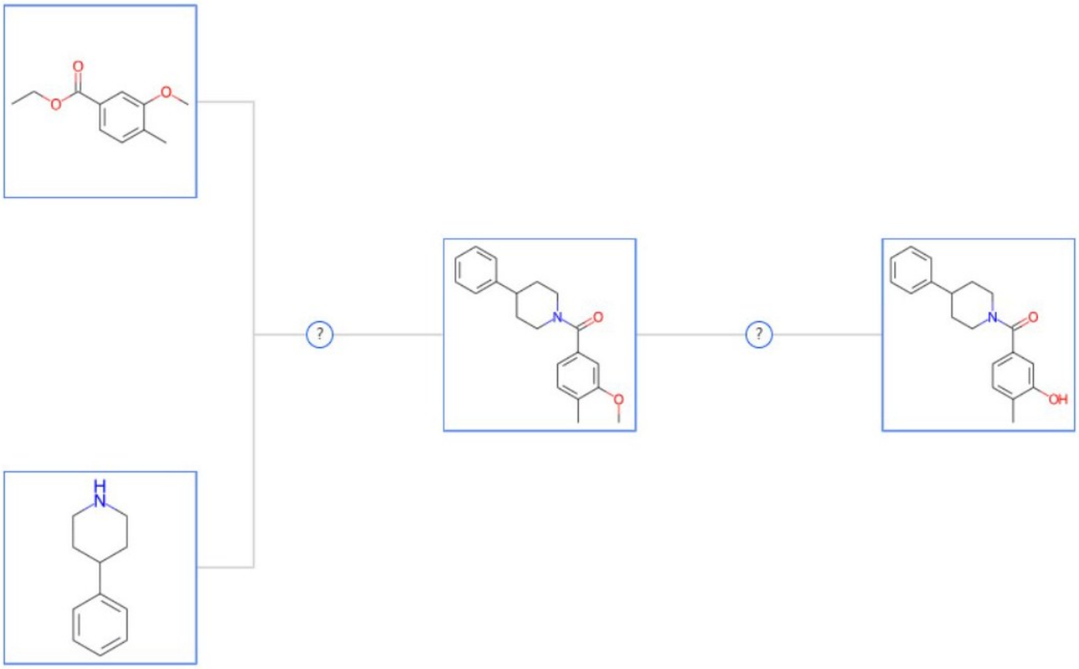

Alert Rule	Alert Structure	Reference
BMS Rule	✓	1. [1] Huth J R, Mendoza R, Olejniczak E T, et al. ALARM NMR: a rapid and robust experimental method to detect reactive false positives in biochemical screens[J]. Journal of the American Chemical Society, 2005, 127(1): 217-224.
Chelator Rule	✓	1. [1] Agrawal A, Johnson S L, Jacobsen J A, et al. Chelator fragment libraries for targeting metalloproteinases[J]. ChemMedChem: Chemistry Enabling Drug Discovery, 2010, 5(2): 195-199.
PAINS	✓	1. [1] Baell J B, Holloway G A. New substructure filters for removal of pan assay interference compounds (PAINS) from screening libraries and for their exclusion in bioassays[J]. Journal of medicinal chemistry, 2010, 53(7): 2719-2740.
Genotoxic Carcinogenicity Rule	✓	1. [1] Benigni R, Bossa C. Structure alerts for carcinogenicity, and the Salmonella assay system: a novel insight through the chemical relational databases technology[J]. Mutation Research/Reviews in Mutation Research, 2008, 659(3): 248-261.<br>2. [2] Ashby J, Tennant R W. Chemical structure, Salmonella mutagenicity and extent of carcinogenicity as indicators of genotoxic carcinogenesis among 222 chemicals tested in rodents by the US NCI/NTP[J]. Mutation Research/Genetic Toxicology, 1988, 204(1): 17-115.<br>3. [3] Kazius J, McGuire R, Bursi R. Derivation and validation of toxicophores for mutagenicity prediction[J]. Journal of medicinal chemistry, 2005, 48(1): 312-320.<br>4. [4] Bailey A B, Chanderbhan R, Collazo-Braier N, et al. The use of structure-activity relationship analysis in the food contact notification program[J]. Regulatory Toxicology and Pharmacology, 2005, 42(2): 225-235.
NTD	✓	1. [1] Brenk R, Schipani A, James D, et al. Lessons learnt from assembling screening libraries for drug discovery for neglected diseases[J]. ChemMedChem: Chemistry Enabling Drug Discovery, 2008, 3(3): 435-444.
SureChEMBL Rule	✓	1. [1] Sushko I, Salmina E, Potemkin V A, et al. ToxAlerts: a web server of structural alerts for toxic chemicals and compounds with potential adverse reactions[J]. 2012.

Compounds AI19:

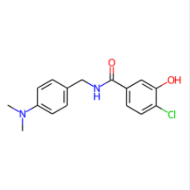

SMILES: CN(C)c1ccc(CNC(=O)c2ccc(Cl)c(O)c2)cc1

San ID	No data
CAS	No data
InChI	InChI=1S/C16H17ClN2O2/c1-19(2)13-6-3-11(4-7-13)10-18-16(21)12-5-8-14(17)15(20)9-12/h3-9,20H,10H2,1-2H3,(H,18,21)
InChIKey	ZFVYMPZRZAXYGP-UHFFFAOYSA-N

View in third-party databases: PubChem

MW	304.1	HBA	3	HBD	2
PAINS Alert	true	QED	0.91	NRot	4
TPSA	52.57	logD	2.4	logP	2.77
logS	-4.08				

Bad Average Good Scoring Notice

Absorption

Caco2	-4.76	HIA	100.00%	P-gp Inhibitor	84.10%
Bioavailability	100.00%	HFE	-9.99		

Distribution

BBB Penetration	81.00%	PPBR	1.00%	VDSS	3.84

Metabolism

CYP2D6 Inhibitor	43.48%	CYP3A4 Inhibitor	76.99%	CYP2C9 Inhibitor	63.35%
CYP2D6 Substrate	0.41%	CYP3A4 Substrate	34.87%	CYP2C9 Substrate	0.01%
CYP2C19 Inhibitor	81.69%	CYP1A2 Inhibitor	98.36%		

Excretion

Half Life (hour)	2.57	Clearance (mL/min/g)	39.25

Tox

hERG	85.48%	DILI	66.50%	NR-AhR	47.99%
Carcinogencity	2.95%	LD_{50}	1313.81	Respiratory Toxicity	26.33%
Eye Corrosion	1.27%				

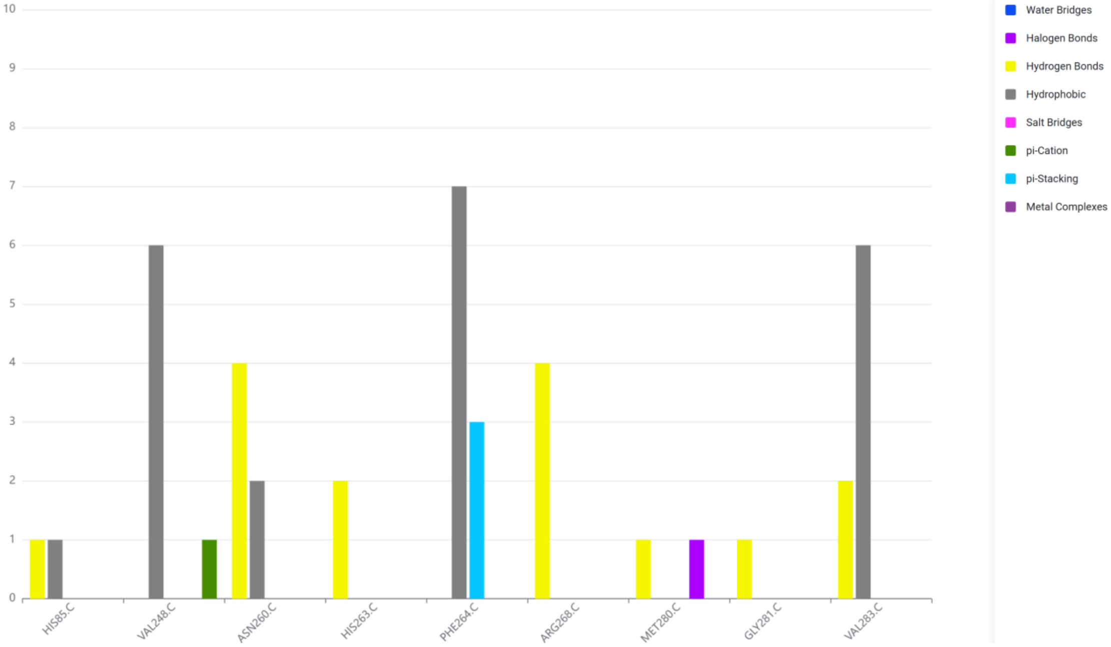

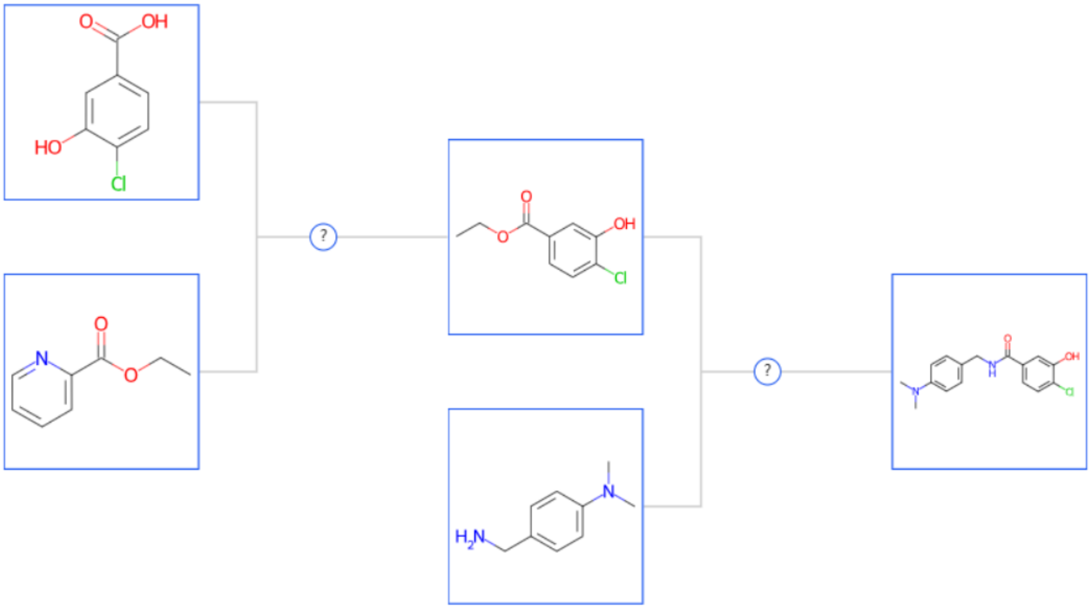

Alert Rule	Alert Structure	Reference
BMS Rule	✔	1. [1] Huth J R, Mendoza R, Olejniczak E T, et al. ALARM NMR: a rapid and robust experimental method to detect reactive false positives in biochemical screens[J]. Journal of the American Chemical Society, 2005, 127(1): 217-224.
Chelator Rule	✔	1. [1] Agrawal A, Johnson S L, Jacobsen J A, et al. Chelator fragment libraries for targeting metalloproteinases[J]. ChemMedChem: Chemistry Enabling Drug Discovery, 2010, 5(2): 195-199.
PAINS	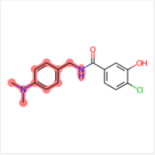	1. [1] Baell J B, Holloway G A. New substructure filters for removal of pan assay interference compounds (PAINS) from screening libraries and for their exclusion in bioassays[J]. Journal of medicinal chemistry, 2010, 53(7): 2719-2740.
Genotoxic Carcinogenicity Rule	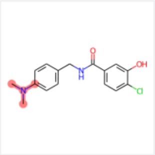	1. [1] Benigni R, Bossa C. Structure alerts for carcinogenicity, and the Salmonella assay system: a novel insight through the chemical relational databases technology[J]. Mutation Research/Reviews in Mutation Research, 2008, 659(3): 248-261.<br>2. [2] Ashby J, Tennant R W. Chemical structure, Salmonella mutagenicity and extent of carcinogenicity as indicators of genotoxic carcinogenesis among 222 chemicals tested in rodents by the US NCI/NTP[J]. Mutation Research/Genetic Toxicology, 1988, 204(1): 17-115.<br>3. [3] Kazius J, McGuire R, Bursi R. Derivation and validation of toxicophores for mutagenicity prediction[J]. Journal of medicinal chemistry, 2005, 48(1): 312-320.<br>4. [4] Bailey A B, Chanderbhan R, Collazo-Braier N, et al. The use of structure-activity relationship analysis in the food contact notification program[J]. Regulatory Toxicology and Pharmacology, 2005, 42(2): 225-235.
NTD	✔	1. [1] Brenk R, Schipani A, James D, et al. Lessons learnt from assembling screening libraries for drug discovery for neglected diseases[J]. ChemMedChem: Chemistry Enabling Drug Discovery, 2008, 3(3): 435-444.
SureChEMBL Rule	✔	1. [1] Sushko I, Salmina E, Potemkin V A, et al. ToxAlerts: a web server of structural alerts for toxic chemicals and compounds with potential adverse reactions[J]. 2012.

Compounds AI20:

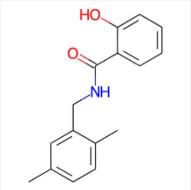

SMILES: Cc1ccc(C)c(CNC(=O)c2ccccc2O)c1

San ID	No data
CAS	No data
InChI	InChI=1S/C16H17NO2/c1-11-7-8-12(2)13(9-11)10-17-16(19)14-5-3-4-6-15(14)18/h3-9,18H,10H2,1-2H3,(H,17,19)
InChIKey	OBRMKMORFJEPFS-UHFFFAOYSA-N

View in third-party databases: Loading...

MW	255.13	HBA	2	HBD	2
PAINS Alert	false	QED	0.88	NRot	3
TPSA	49.33	logD	3.99	logP	3.89
logS	-4.23				

Bad Average Good Scoring Notice

Absorption

Caco2	-4.62	HIA	99.80%	P-gp Inhibitor	79.40%
Bioavailability	99.98%	HFE	-8.24		

Distribution

BBB Penetration	61.98%	PPBR	1.00%	VDSS	2.08

Metabolism

CYP2D6 Inhibitor	36.53%	CYP3A4 Inhibitor	85.45%	CYP2C9 Inhibitor	62.24%
CYP2D6 Substrate	70.39%	CYP3A4 Substrate	68.81%	CYP2C9 Substrate	4.74%
CYP2C19 Inhibitor	89.84%	CYP1A2 Inhibitor	58.98%		

Excretion

Half Life (hour)	2.26	Clearance (mL/min/g)	42.88

Tox

hERG	85.84%	DILI	35.08%	NR-AhR	12.52%
Carcinogencity	77.76%	LD_{50}	1073.67	Respiratory Toxicity	0.95%
Eye Corrosion	6.76%				

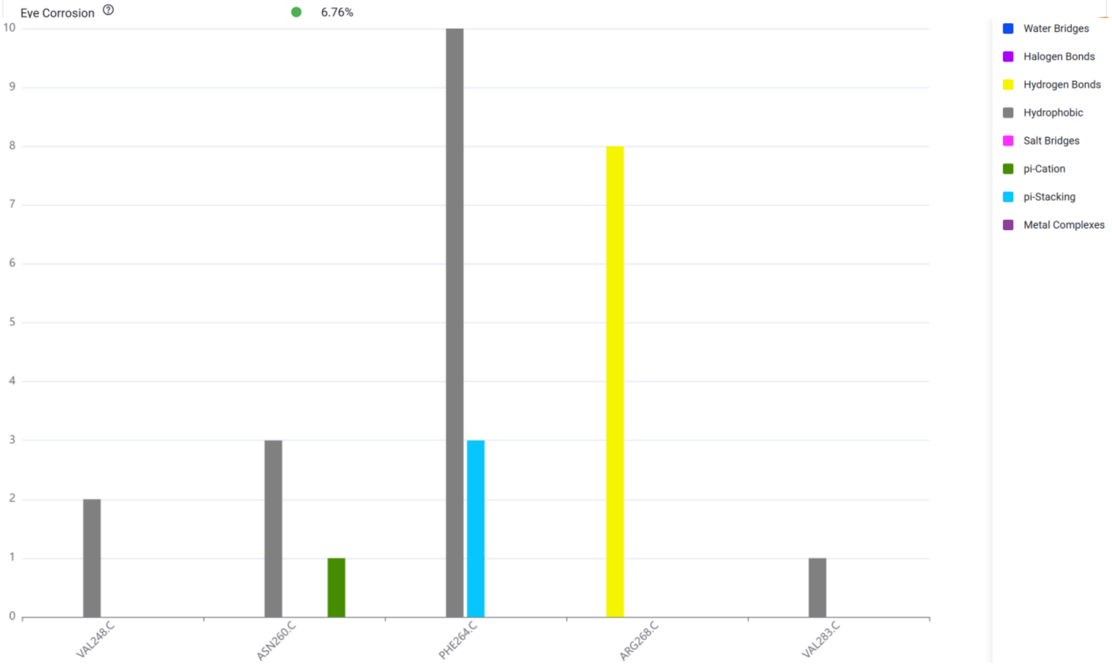

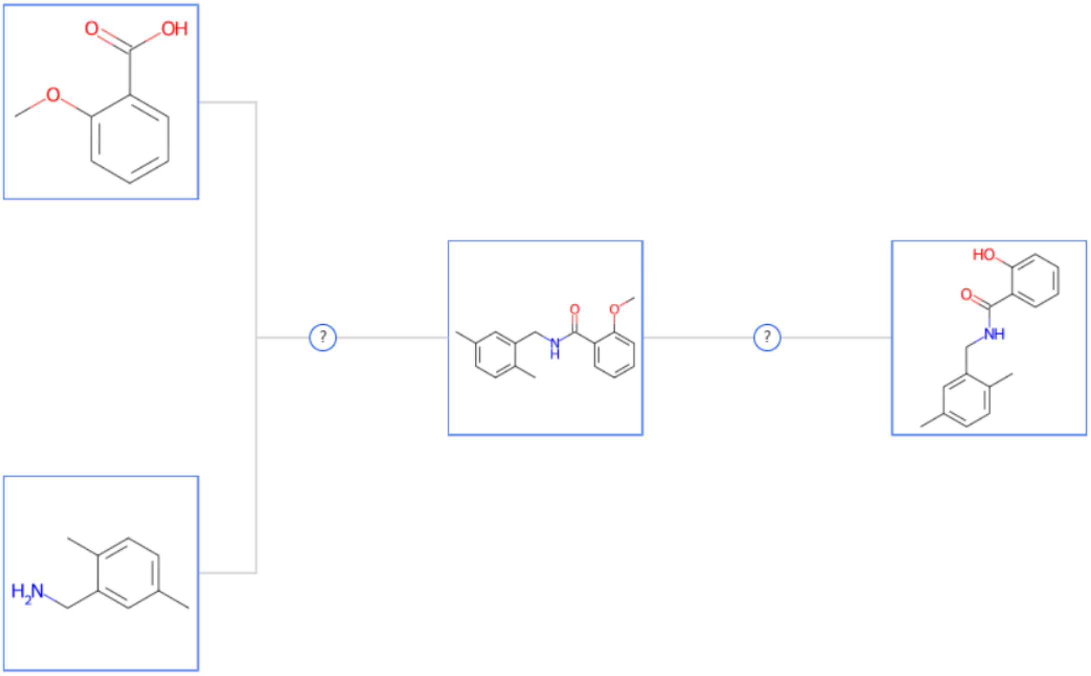

Alert Rule	Alert Structure	Reference
BMS Rule	✓	1. [1] Huth J R, Mendoza R, Olejniczak E T, et al. ALARM NMR: a rapid and robust experimental method to detect reactive false positives in biochemical screens[J]. Journal of the American Chemical Society, 2005, 127(1): 217-224.
Chelator Rule	✓	1. [1] Agrawal A, Johnson S L, Jacobsen J A, et al. Chelator fragment libraries for targeting metalloproteinases[J]. ChemMedChem: Chemistry Enabling Drug Discovery, 2010, 5(2): 195-199.
PAINS	✓	1. [1] Baell J B, Holloway G A. New substructure filters for removal of pan assay interference compounds (PAINS) from screening libraries and for their exclusion in bioassays[J]. Journal of medicinal chemistry, 2010, 53(7): 2719-2740.
Genotoxic Carcinogenicity Rule	✓	1. [1] Benigni R, Bossa C. Structure alerts for carcinogenicity, and the Salmonella assay system: a novel insight through the chemical relational databases technology[J]. Mutation Research/Reviews in Mutation Research, 2008, 659(3): 248-261.<br>2. [2] Ashby J, Tennant R W. Chemical structure, Salmonella mutagenicity and extent of carcinogenicity as indicators of genotoxic carcinogenesis among 222 chemicals tested in rodents by the US NCI/NTP[J]. Mutation Research/Genetic Toxicology, 1988, 204(1): 17-115.<br>3. [3] Kazius J, McGuire R, Bursi R. Derivation and validation of toxicophores for mutagenicity prediction[J]. Journal of medicinal chemistry, 2005, 48(1): 312-320.<br>4. [4] Bailey A B, Chanderbhan R, Collazo-Braier N, et al. The use of structure-activity relationship analysis in the food contact notification program[J]. Regulatory Toxicology and Pharmacology, 2005, 42(2): 225-235.
NTD	✓	1. [1] Brenk R, Schipani A, James D, et al. Lessons learnt from assembling screening libraries for drug discovery for neglected diseases[J]. ChemMedChem: Chemistry Enabling Drug Discovery, 2008, 3(3): 435-444.
SureChEMBL Rule	✓	1. [1] Sushko I, Salmina E, Potemkin V A, et al. ToxAlerts: a web server of structural alerts for toxic chemicals and compounds with potential adverse reactions[J]. 2012.

Compounds AI21:

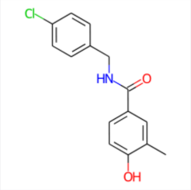

SMILES: Cc1cc(C(=O)NCc2ccc(Cl)cc2)ccc1O

San ID	8ed0eea73ed9546c964e878821cb68c2
CAS	No data
InChI	InChI=1S/C15H14ClNO2/c1-10-8-12(4-7-14(10)18)15(19)17-9-11-2-5-13(16)6-3-11/h2-8,18H,9H2,1H3,(H,17,19)
InChIKey	OOCPSZARDRXWLY-UHFFFAOYSA-N

View in third-party databases: Loading...

MW	275.07	HBA	2	HBD	2
PAINS Alert	false	QED	0.90	NRot	3
TPSA	49.33	logD	3.53	logP	3.29
logS	-3.79				

Bad Average Good Scoring Notice

Absorption

Caco2	-4.80	HIA	100.00%	P-gp Inhibitor	39.55%
Bioavailability	99.96%	HFE	-9.38		

Distribution

BBB Penetration	78.25%	PPBR	1.00%	VDSS	3.44

Metabolism

CYP2D6 Inhibitor	41.22%	CYP3A4 Inhibitor	65.96%	CYP2C9 Inhibitor	71.29%
CYP2D6 Substrate	3.68%	CYP3A4 Substrate	29.75%	CYP2C9 Substrate	0.29%
CYP2C19 Inhibitor	92.32%	CYP1A2 Inhibitor	97.00%		

Excretion

Half Life (hour)	2.34	Clearance (mL/min/g)	35.75

Tox

hERG	84.25%	DILI	73.93%	NR-AhR	15.62%
Carcinogencity	56.10%	LD_{50}	944.20	Respiratory Toxicity	0.99%
Eye Corrosion	0.27%				

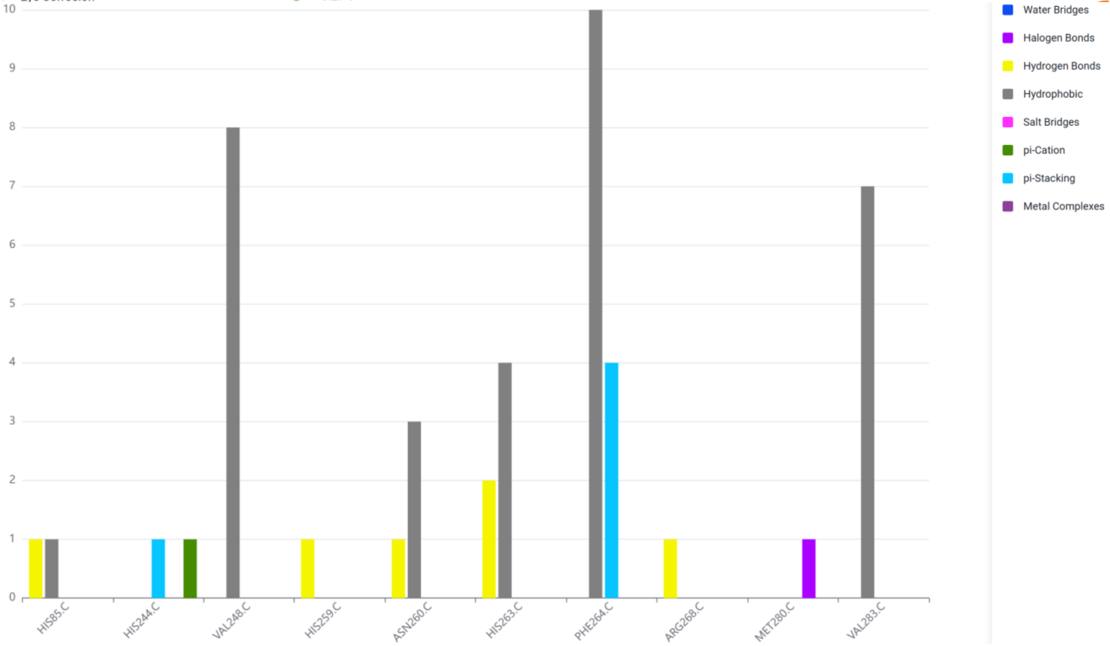

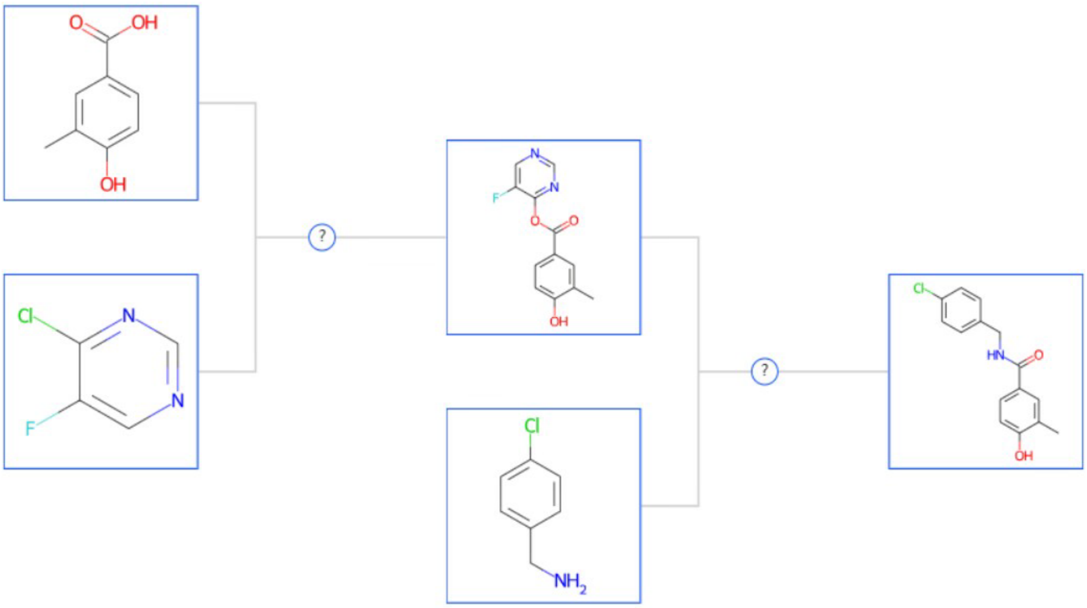

Alert Rule	Alert Structure	Reference
BMS Rule	✔	1. [1] Huth J R, Mendoza R, Olejniczak E T, et al. ALARM NMR: a rapid and robust experimental method to detect reactive false positives in biochemical screens[J]. Journal of the American Chemical Society, 2005, 127(1): 217-224.
Chelator Rule	✔	1. [1] Agrawal A, Johnson S L, Jacobsen J A, et al. Chelator fragment libraries for targeting metalloproteinases[J]. ChemMedChem: Chemistry Enabling Drug Discovery, 2010, 5(2): 195-199.
PAINS	✔	1. [1] Baell J B, Holloway G A. New substructure filters for removal of pan assay interference compounds (PAINS) from screening libraries and for their exclusion in bioassays[J]. Journal of medicinal chemistry, 2010, 53(7): 2719-2740.
Genotoxic Carcinogenicity Rule	✔	1. [1] Benigni R, Bossa C. Structure alerts for carcinogenicity, and the Salmonella assay system: a novel insight through the chemical relational databases technology[J]. Mutation Research/Reviews in Mutation Research, 2008, 659(3): 248-261.<br>2. [2] Ashby J, Tennant R W. Chemical structure, Salmonella mutagenicity and extent of carcinogenicity as indicators of genotoxic carcinogenesis among 222 chemicals tested in rodents by the US NCI/NTP[J]. Mutation Research/Genetic Toxicology, 1988, 204(1): 17-115.<br>3. [3] Kazius J, McGuire R, Bursi R. Derivation and validation of toxicophores for mutagenicity prediction[J]. Journal of medicinal chemistry, 2005, 48(1): 312-320.<br>4. [4] Bailey A B, Chanderbhan R, Collazo-Braier N, et al. The use of structure-activity relationship analysis in the food contact notification program[J]. Regulatory Toxicology and Pharmacology, 2005, 42(2): 225-235.
NTD	✔	1. [1] Brenk R, Schipani A, James D, et al. Lessons learnt from assembling screening libraries for drug discovery for neglected diseases[J]. ChemMedChem: Chemistry Enabling Drug Discovery, 2008, 3(3): 435-444.
SureChEMBL Rule	✔	1. [1] Sushko I, Salmina E, Potemkin V A, et al. ToxAlerts: a web server of structural alerts for toxic chemicals and compounds with potential adverse reactions[J]. 2012.

Compounds AI22:

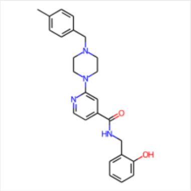

SMILES: Cc1ccc(CN2CCN(c3cc(C(=O)NCc4ccccc4O)ccn3)CC2)cc1

**San ID**	No data
**CAS**	No data
**InChI**	InChI=1S/C25H28N4O2/c1-19-6-8-20(9-7-19)18-28-12-14-29(15-13-28)24-16-21(10-11-26-24)25(31)27-17-22-4-2-3-5-23(22)30/h2-11,16,30H,12-...
**InChIKey**	GAXSUQUETVPVPM-UHFFFAOYSA-N

View in third-party databases: Loading...

MW	416.22	HBA	5	HBD	2
PAINS Alert	true	QED	0.64	NRot	6
TPSA	68.7	logD	4.05	logP	3.58
logS	-3.26				

Bad Average Good Scoring Notice

Absorption

Caco2	-5.28	HIA	100.00%	P-gp Inhibitor	91.61%
Bioavailability	100.00%	HFE	-10.93		

Distribution

BBB Penetration	49.13%	PPBR	1.00%	VDSS	1.35

Metabolism

CYP2D6 Inhibitor	48.87%	CYP3A4 Inhibitor	62.52%	CYP2C9 Inhibitor	60.58%
CYP2D6 Substrate	99.33%	CYP3A4 Substrate	79.85%	CYP2C9 Substrate	35.25%
CYP2C19 Inhibitor	72.22%	CYP1A2 Inhibitor	22.18%		

Excretion

Half Life (hour)	3.14	Clearance (mL/min/g)	62.30

Tox

hERG	96.29%	DILI	30.14%	NR-AhR	7.11%
Carcinogencity	0.79%	LD_{50}	934.75	Respiratory Toxicity	97.24%
Eye Corrosion	0.04%				

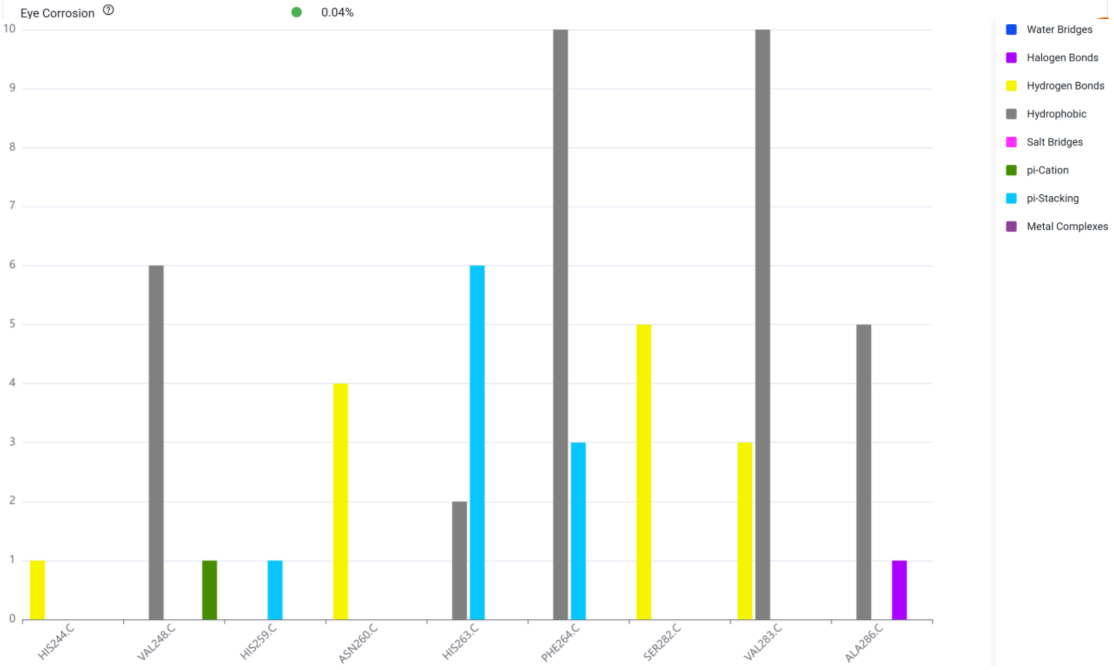

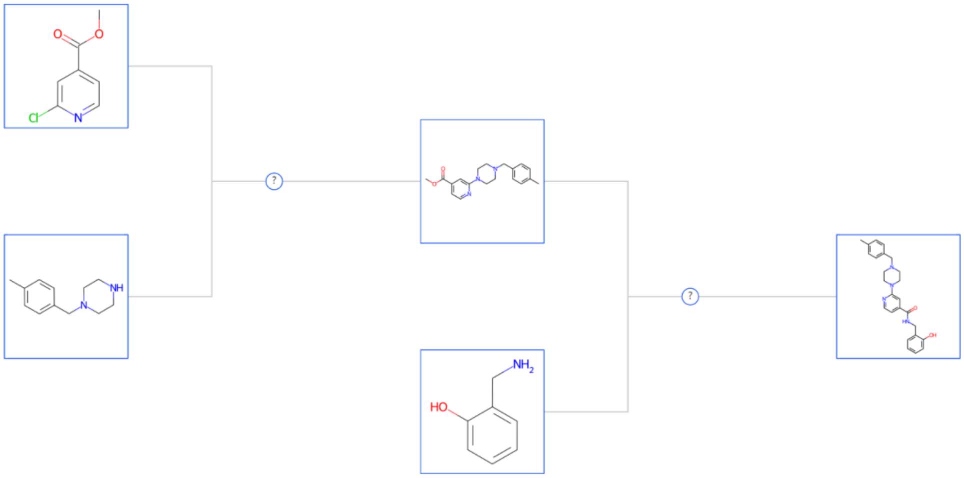

Alert Rule	Alert Structure	Reference
BMS Rule	✓	1. [1] Huth J R, Mendoza R, Olejniczak E T, et al. ALARM NMR: a rapid and robust experimental method to detect reactive false positives in biochemical screens[J]. Journal of the American Chemical Society, 2005, 127(1): 217-224.
Chelator Rule	✓	1. [1] Agrawal A, Johnson S L, Jacobsen J A, et al. Chelator fragment libraries for targeting metalloproteinases[J]. ChemMedChem: Chemistry Enabling Drug Discovery, 2010, 5(2): 195-199.
PAINS	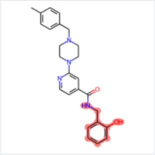	1. [1] Baell J B, Holloway G A. New substructure filters for removal of pan assay interference compounds (PAINS) from screening libraries and for their exclusion in bioassays[J]. Journal of medicinal chemistry, 2010, 53(7): 2719-2740.
Genotoxic Carcinogenicity Rule	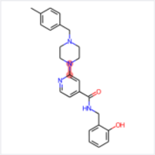	1. [1] Benigni R, Bossa C. Structure alerts for carcinogenicity, and the Salmonella assay system: a novel insight through the chemical relational databases technology[J]. Mutation Research/Reviews in Mutation Research, 2008, 659(3): 248-261.<br>2. [2] Ashby J, Tennant R W. Chemical structure, Salmonella mutagenicity and extent of carcinogenicity as indicators of genotoxic carcinogenesis among 222 chemicals tested in rodents by the US NCI/NTP[J]. Mutation Research/Genetic Toxicology, 1988, 204(1): 17-115.<br>3. [3] Kazius J, McGuire R, Bursi R. Derivation and validation of toxicophores for mutagenicity prediction[J]. Journal of medicinal chemistry, 2005, 48(1): 312-320.<br>4. [4] Bailey A B, Chanderbhan R, Collazo-Braier N, et al. The use of structure-activity relationship analysis in the food contact notification program[J]. Regulatory Toxicology and Pharmacology, 2005, 42(2): 225-235.
NTD	✓	1. [1] Brenk R, Schipani A, James D, et al. Lessons learnt from assembling screening libraries for drug discovery for neglected diseases[J]. ChemMedChem: Chemistry Enabling Drug Discovery, 2008, 3(3): 435-444.
SureChEMBL Rule	✓	1. [1] Sushko I, Salmina E, Potemkin V A, et al. ToxAlerts: a web server of structural alerts for toxic chemicals and compounds with potential adverse reactions[J]. 2012.

Compounds AI23:

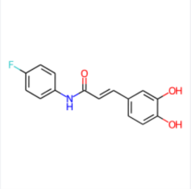

SMILES: O=C(/C=C/c1ccc(O)c(O)c1)Nc1ccc(F)cc1

San ID	b95e9e8a5660c77bf0ee2d85ec9b6f14
CAS	No data
InChI	InChI=1S/C15H12FNO3/c16-11-3-5-12(6-4-11)17-15(20)8-2-10-1-7-13(18)14(19)9-10/h1-9,18-19H,(H,17,20)/b8-2+
InChIKey	MLPHDUQOZKAGHK-KRXBUXKQSA-N

View in third-party databases: Loading...

MW	273.08	HBA	3	HBD	3
PAINS Alert	true	QED	0.59	NRot	3
TPSA	69.56	logD	2.37	logP	2.97
logS	-3.87				

Bad Average Good Scoring Notice

Absorption

Caco2	-5.01	HIA	99.40%	P-gp Inhibitor	13.03%
Bioavailability	99.84%	HFE	-10.63		

Distribution

BBB Penetration	81.44%	PPBR	1.00%	VDSS	2.54

Metabolism

CYP2D6 Inhibitor	17.54%	CYP3A4 Inhibitor	34.62%	CYP2C9 Inhibitor	51.42%
CYP2D6 Substrate	0.00%	CYP3A4 Substrate	41.53%	CYP2C9 Substrate	96.80%
CYP2C19 Inhibitor	61.86%	CYP1A2 Inhibitor	93.30%		

Excretion

Half Life (hour)	1.75	Clearance (mL/min/g)	42.48

Tox

hERG	62.85%	DILI	91.44%	NR-AhR	59.16%
Carcinogencity	30.31%	LD_{50}	539.35	Respiratory Toxicity	3.96%
Eye Corrosion	0.59%				

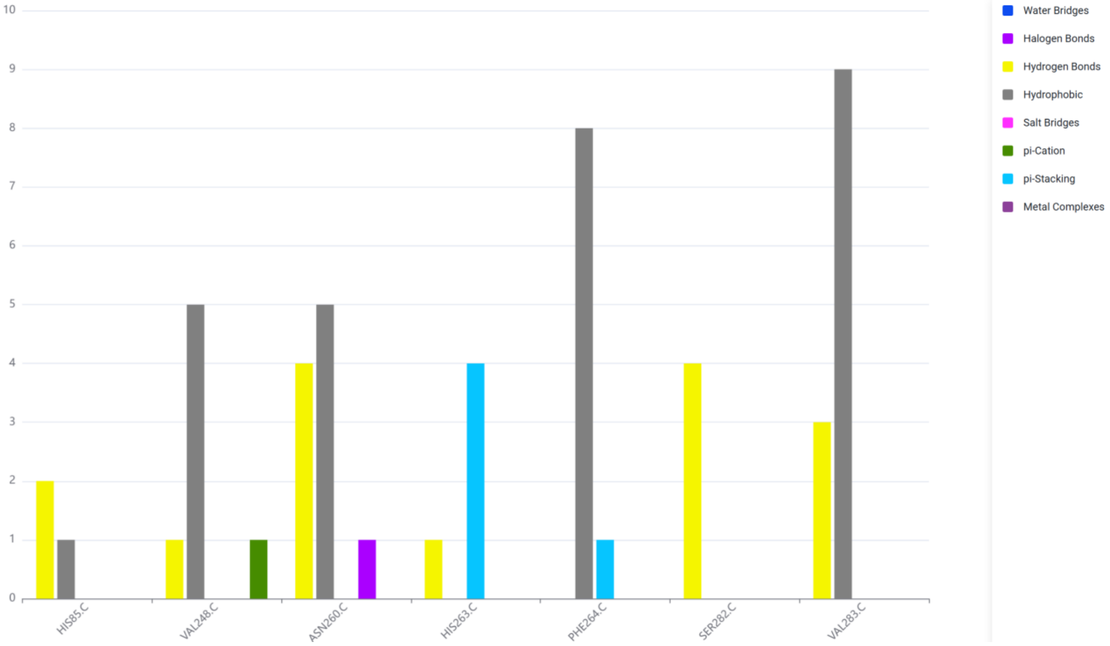

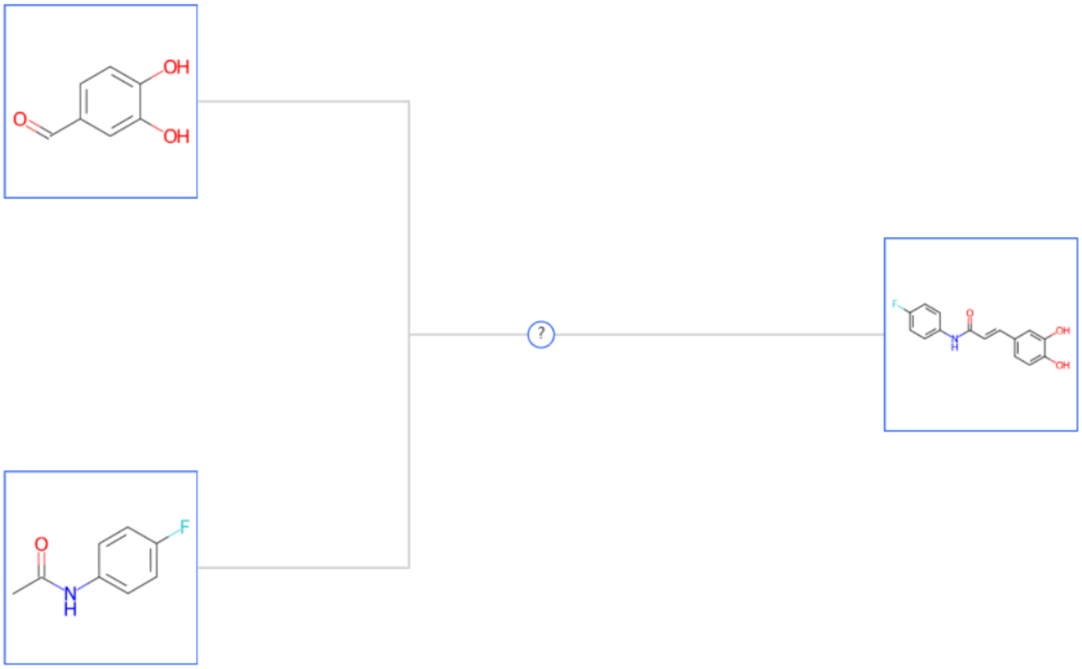

Alert Rule	Alert Structure	Reference
BMS Rule	✔	1. [1] Huth J R, Mendoza R, Olejniczak E T, et al. ALARM NMR: a rapid and robust experimental method to detect reactive false positives in biochemical screens[J]. Journal of the American Chemical Society, 2005, 127(1): 217-224.
Chelator Rule	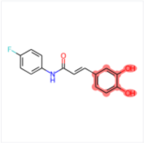	1. [1] Agrawal A, Johnson S L, Jacobsen J A, et al. Chelator fragment libraries for targeting metalloproteinases[J]. ChemMedChem: Chemistry Enabling Drug Discovery, 2010, 5(2): 195-199.
PAINS	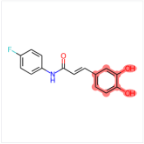	1. [1] Baell J B, Holloway G A. New substructure filters for removal of pan assay interference compounds (PAINS) from screening libraries and for their exclusion in bioassays[J]. Journal of medicinal chemistry, 2010, 53(7): 2719-2740.
Genotoxic Carcinogenicity Rule	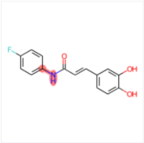	1. [1] Benigni R, Bossa C. Structure alerts for carcinogenicity, and the Salmonella assay system: a novel insight through the chemical relational databases technology[J]. Mutation Research/Reviews in Mutation Research, 2008, 659(3): 248-261.<br>2. [2] Ashby J, Tennant R W. Chemical structure, Salmonella mutagenicity and extent of carcinogenicity as indicators of genotoxic carcinogenesis among 222 chemicals tested in rodents by the US NCI/NTP[J]. Mutation Research/Genetic Toxicology, 1988, 204(1): 17-115.<br>3. [3] Kazius J, McGuire R, Bursi R. Derivation and validation of toxicophores for mutagenicity prediction[J]. Journal of medicinal chemistry, 2005, 48(1): 312-320.<br>4. [4] Bailey A B, Chanderbhan R, Collazo-Braier N, et al. The use of structure-activity relationship analysis in the food contact notification program[J]. Regulatory Toxicology and Pharmacology, 2005, 42(2): 225-235.
NTD	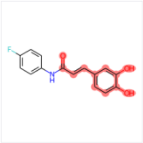	1. [1] Brenk R, Schipani A, James D, et al. Lessons learnt from assembling screening libraries for drug discovery for neglected diseases[J]. ChemMedChem: Chemistry Enabling Drug Discovery, 2008, 3(3): 435-444.
SureChEMBL Rule	✔	1. [1] Sushko I, Salmina E, Potemkin V A, et al. ToxAlerts: a web server of structural alerts for toxic chemicals and compounds with potential adverse reactions[J]. 2012.

Compounds AI24:

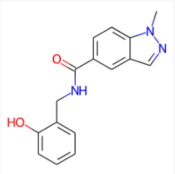

SMILES: Cn1ncc2cc(C(=O)NCc3ccccc3O)ccc21

San ID	No data
CAS	No data
InChI	InChI=1S/C16H15N3O2/c1-19-14-7-6-11(8-13(14)10-18-19)16(21)17-9-12-4-2-3-5-15(12)20/h2-8,10,20H,9H2,1H3,(H,17,21)
InChIKey	SGAJCVHWNLYHKD-UHFFFAOYSA-N

View in third-party databases: Loading...

MW	281.12	HBA	4	HBD	2
PAINS Alert	true	QED	0.77	NRot	3
TPSA	67.15	logD	2.52	logP	2.05
logS	-3.04				

Bad Average Good Scoring Notice

Absorption

Caco2	-4.88	HIA	99.93%	P-gp Inhibitor	27.34%
Bioavailability	100.00%	HFE	-11.78		

Distribution

BBB Penetration	73.26%	PPBR	1.00%	VDSS	1.84

Metabolism

CYP2D6 Inhibitor	15.06%	CYP3A4 Inhibitor	44.04%	CYP2C9 Inhibitor	31.24%
CYP2D6 Substrate	9.70%	CYP3A4 Substrate	74.82%	CYP2C9 Substrate	0.08%
CYP2C19 Inhibitor	35.89%	CYP1A2 Inhibitor	30.67%		

Excretion

Half Life (hour)	3.07	Clearance (mL/min/g)	46.95

Tox

hERG	70.70%	DILI	80.55%	NR-AhR	30.85%
Carcinogencity	1.35%	LD_{50}	869.52	Respiratory Toxicity	3.54%
Eye Corrosion	0.11%				

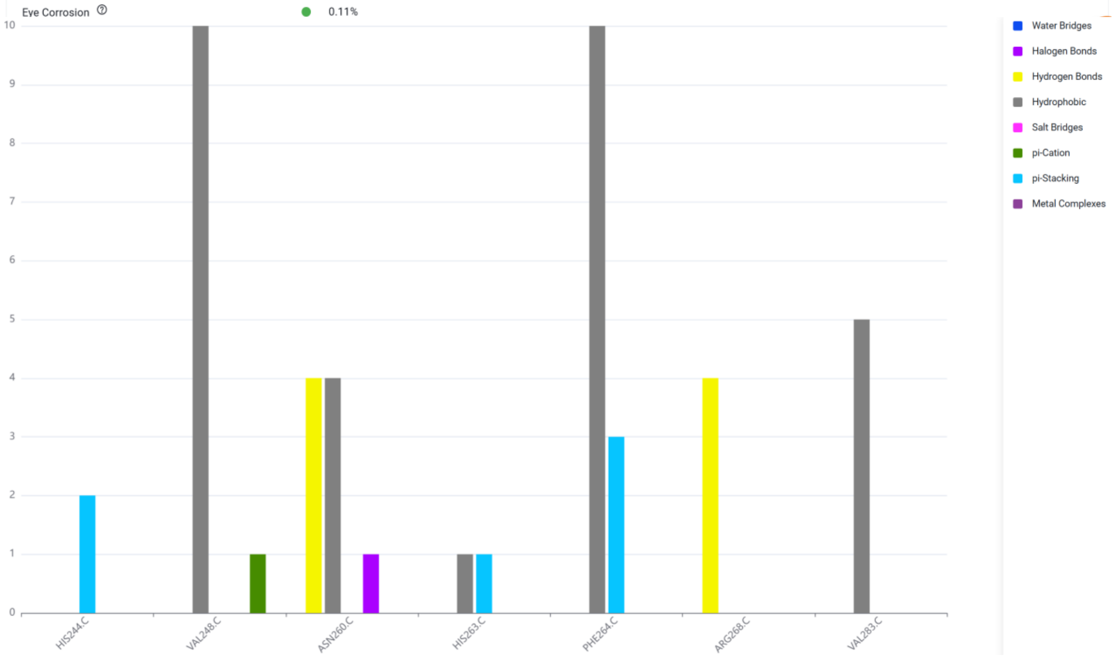

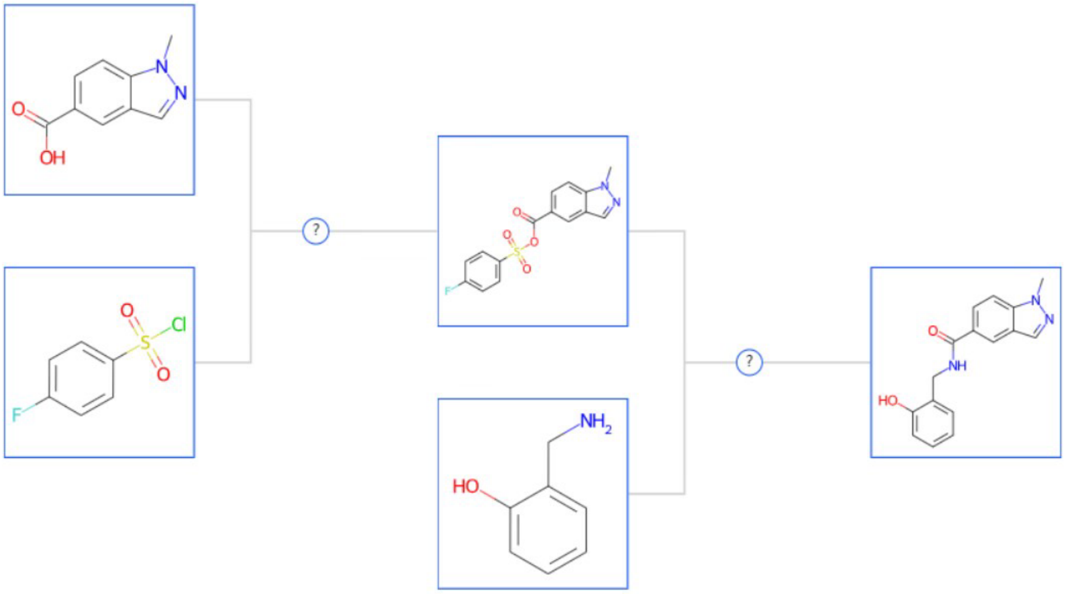

Alert Rule	Alert Structure	Reference
BMS Rule	✔	1. [1] Huth J R, Mendoza R, Olejniczak E T, et al. ALARM NMR: a rapid and robust experimental method to detect reactive false positives in biochemical screens[J]. Journal of the American Chemical Society, 2005, 127(1): 217-224.
Chelator Rule	✔	1. [1] Agrawal A, Johnson S L, Jacobsen J A, et al. Chelator fragment libraries for targeting metalloproteinases[J]. ChemMedChem: Chemistry Enabling Drug Discovery, 2010, 5(2): 195-199.
PAINS	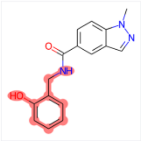	1. [1] Baell J B, Holloway G A. New substructure filters for removal of pan assay interference compounds (PAINS) from screening libraries and for their exclusion in bioassays[J]. Journal of medicinal chemistry, 2010, 53(7): 2719-2740.
Genotoxic Carcinogenicity Rule	✔	1. [1] Benigni R, Bossa C. Structure alerts for carcinogenicity, and the Salmonella assay system: a novel insight through the chemical relational databases technology[J]. Mutation Research/Reviews in Mutation Research, 2008, 659(3): 248-261.<br>2. [2] Ashby J, Tennant R W. Chemical structure, Salmonella mutagenicity and extent of carcinogenicity as indicators of genotoxic carcinogenesis among 222 chemicals tested in rodents by the US NCI/NTP[J]. Mutation Research/Genetic Toxicology, 1988, 204(1): 17-115.<br>3. [3] Kazius J, McGuire R, Bursi R. Derivation and validation of toxicophores for mutagenicity prediction[J]. Journal of medicinal chemistry, 2005, 48(1): 312-320.<br>4. [4] Bailey A B, Chanderbhan R, Collazo-Braier N, et al. The use of structure-activity relationship analysis in the food contact notification program[J]. Regulatory Toxicology and Pharmacology, 2005, 42(2): 225-235.
NTD	✔	1. [1] Brenk R, Schipani A, James D, et al. Lessons learnt from assembling screening libraries for drug discovery for neglected diseases[J]. ChemMedChem: Chemistry Enabling Drug Discovery, 2008, 3(3): 435-444.
SureChEMBL Rule	✔	1. [1] Sushko I, Salmina E, Potemkin V A, et al. ToxAlerts: a web server of structural alerts for toxic chemicals and compounds with potential adverse reactions[J]. 2012.

Compounds AI25:

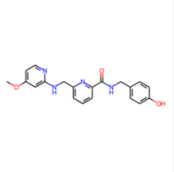

SMILES: COc1ccnc(NCc2cccc(C(=O)NCc3ccc(O)cc3)n2)c1

San ID	No data
CAS	No data
InChI	InChI=1S/C20H20N4O3/c1-27-17-9-10-21-19(11-17)22-13-15-3-2-4-18(24-15)20(26)23-12-14-5-7-16(25)8-6-14/h2-11,25H,12-13H2,1H3,(H,21,22)(H,23,26)
InChIKey	PSMSFAAGCWSKKO-UHFFFAOYSA-N

View in third-party databases: Loading...

MW	364.15	HBA	6	HBD	3
PAINS Alert	false	QED	0.59	NRot	7
TPSA	96.37	logD	2.7	logP	2.62
logS	-4.59				

Bad Average Good Scoring Notice

Absorption

Caco2	-5.47	HIA	100.00%	P-gp Inhibitor	38.81%
Bioavailability	100.00%	HFE	-11.75		

Distribution

BBB Penetration	51.29%	PPBR	1.00%	VDSS	0.73

Metabolism

CYP2D6 Inhibitor	33.87%	CYP3A4 Inhibitor	80.90%	CYP2C9 Inhibitor	15.03%
CYP2D6 Substrate	99.89%	CYP3A4 Substrate	42.85%	CYP2C9 Substrate	99.81%
CYP2C19 Inhibitor	61.62%	CYP1A2 Inhibitor	96.80%		

Excretion

Half Life (hour)	4.40	Clearance (mL/min/g)	57.78

Tox

hERG	77.39%	DILI	81.66%	NR-AhR	33.21%
Carcinogencity	14.21%	LD_{50}	1783.39	Respiratory Toxicity	9.29%
Eye Corrosion	0.52%				

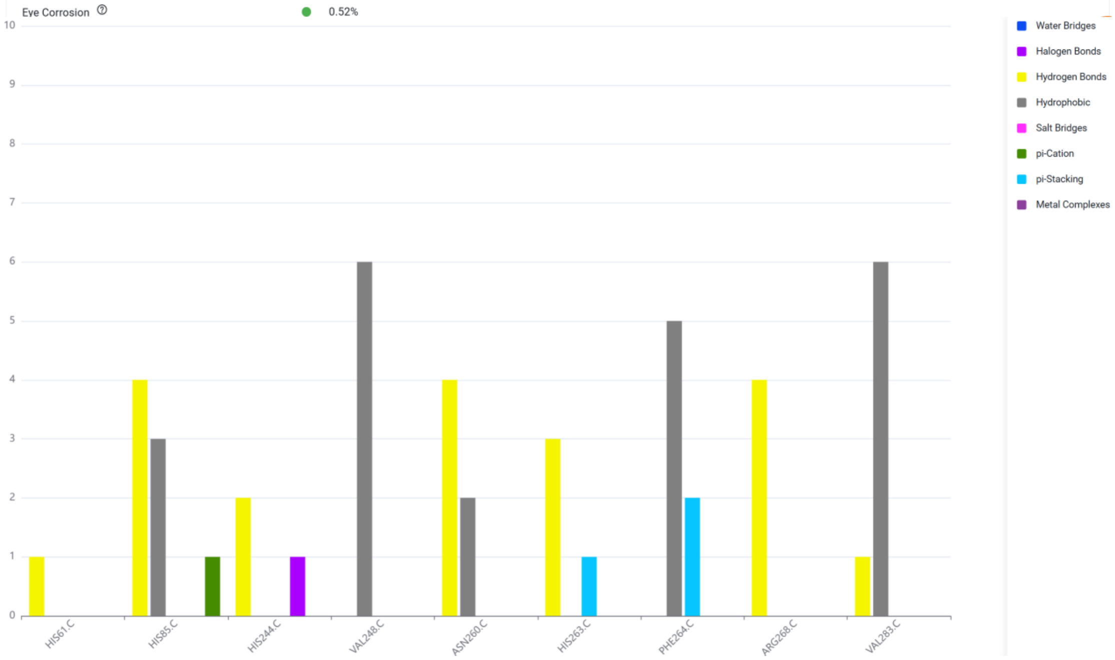

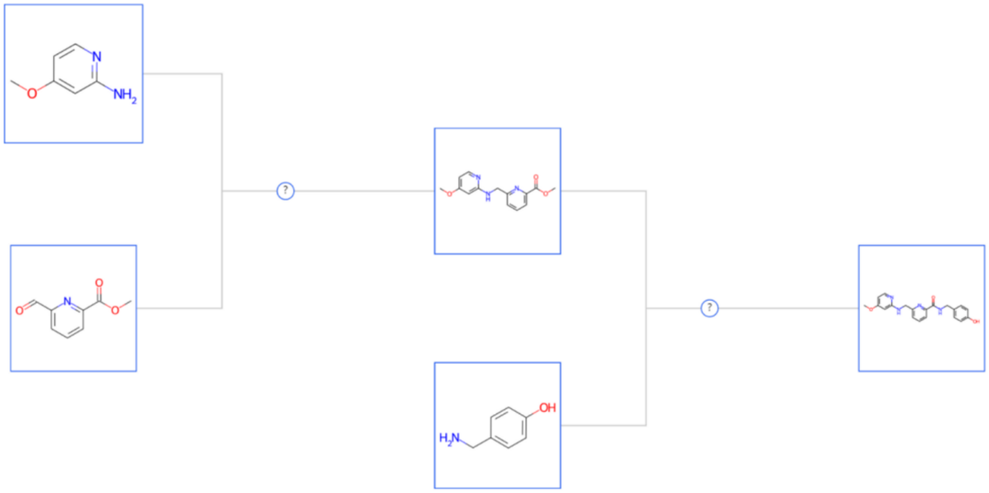

Alert Rule	Alert Structure	Reference
BMS Rule	✅	1. [1] Huth J R, Mendoza R, Olejniczak E T, et al. ALARM NMR: a rapid and robust experimental method to detect reactive false positives in biochemical screens[J]. Journal of the American Chemical Society, 2005, 127(1): 217-224.
Chelator Rule	✅	1. [1] Agrawal A, Johnson S L, Jacobsen J A, et al. Chelator fragment libraries for targeting metalloproteinases[J]. ChemMedChem: Chemistry Enabling Drug Discovery, 2010, 5(2): 195-199.
PAINS	✅	1. [1] Baell J B, Holloway G A. New substructure filters for removal of pan assay interference compounds (PAINS) from screening libraries and for their exclusion in bioassays[J]. Journal of medicinal chemistry, 2010, 53(7): 2719-2740.
Genotoxic Carcinogenicity Rule	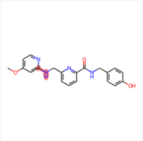	1. [1] Benigni R, Bossa C. Structure alerts for carcinogenicity, and the Salmonella assay system: a novel insight through the chemical relational databases technology[J]. Mutation Research/Reviews in Mutation Research, 2008, 659(3): 248-261.<br>2. [2] Ashby J, Tennant R W. Chemical structure, Salmonella mutagenicity and extent of carcinogenicity as indicators of genotoxic carcinogenesis among 222 chemicals tested in rodents by the US NCI/NTP[J]. Mutation Research/Genetic Toxicology, 1988, 204(1): 17-115.<br>3. [3] Kazius J, McGuire R, Bursi R. Derivation and validation of toxicophores for mutagenicity prediction[J]. Journal of medicinal chemistry, 2005, 48(1): 312-320.<br>4. [4] Bailey A B, Chanderbhan R, Collazo-Braier N, et al. The use of structure-activity relationship analysis in the food contact notification program[J]. Regulatory Toxicology and Pharmacology, 2005, 42(2): 225-235.
NTD	✅	1. [1] Brenk R, Schipani A, James D, et al. Lessons learnt from assembling screening libraries for drug discovery for neglected diseases[J]. ChemMedChem: Chemistry Enabling Drug Discovery, 2008, 3(3): 435-444.
SureChEMBL Rule	✅	1. [1] Sushko I, Salmina E, Potemkin V A, et al. ToxAlerts: a web server of structural alerts for toxic chemicals and compounds with potential adverse reactions[J]. 2012.

Compounds AI26:

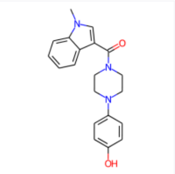

SMILES: Cn1cc(C(=O)N2CCN(c3ccc(O)cc3)CC2)c2ccccc21

San ID	No data
CAS	No data
InChI	InChI=1S/C20H21N3O2/c1-21-14-18(17-4-2-3-5-19(17)21)20(25)23-12-10-22(11-13-23)15-6-8-16(24)9-7-15/h2-9,14,24H,10-13H2,1H3
InChIKey	FLXDKFUGTIBEBW-UHFFFAOYSA-N

View in third-party databases: PubChem

MW	335.16	HBA	4	HBD	1
PAINS Alert	false	QED	0.78	NRot	2
TPSA	48.71	logD	2.17	logP	2.17
logS	-3.24				

Bad Average Good Scoring Notice

Absorption

Caco2	-4.82	HIA	100.00%	P-gp Inhibitor	54.55%
Bioavailability	99.95%	HFE	-10.90		

Distribution

BBB Penetration	89.01%	PPBR	1.00%	VDSS	1.15

Metabolism

CYP2D6 Inhibitor	8.08%	CYP3A4 Inhibitor	80.42%	CYP2C9 Inhibitor	76.93%
CYP2D6 Substrate	94.50%	CYP3A4 Substrate	69.35%	CYP2C9 Substrate	99.99%
CYP2C19 Inhibitor	82.45%	CYP1A2 Inhibitor	45.78%		

Excretion

Half Life (hour)	4.47	Clearance (mL/min/g)	52.93

Tox

hERG	96.21%	DILI	74.41%	NR-AhR	46.11%
Carcinogencity	6.29%	LD_{50}	1316.62	Respiratory Toxicity	98.00%
Eye Corrosion	0.02%				

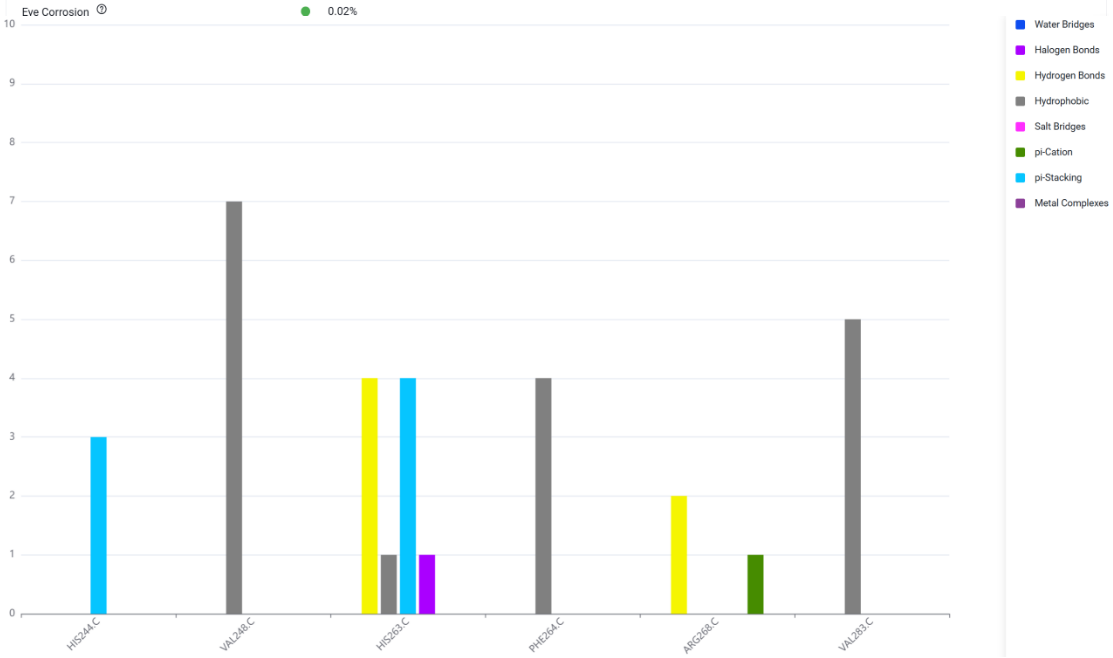

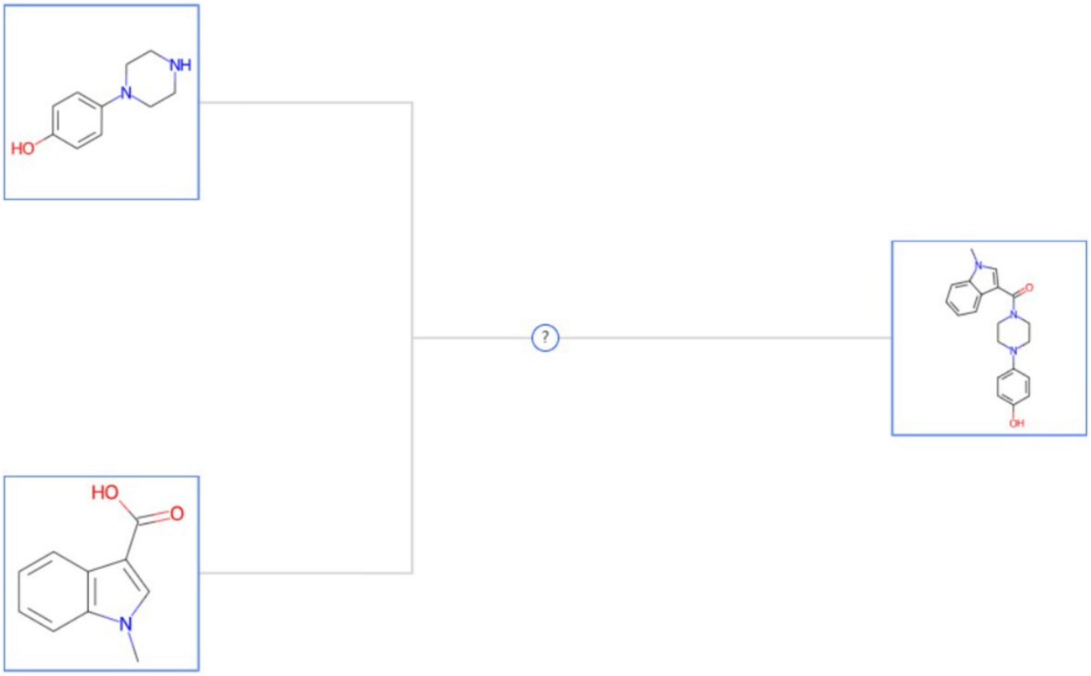

Alert Rule	Alert Structure	Reference
BMS Rule	✔	1. [1] Huth J R, Mendoza R, Olejniczak E T, et al. ALARM NMR: a rapid and robust experimental method to detect reactive false positives in biochemical screens[J]. Journal of the American Chemical Society, 2005, 127(1): 217-224.
Chelator Rule	✔	1. [1] Agrawal A, Johnson S L, Jacobsen J A, et al. Chelator fragment libraries for targeting metalloproteinases[J]. ChemMedChem: Chemistry Enabling Drug Discovery, 2010, 5(2): 195-199.
PAINS	✔	1. [1] Baell J B, Holloway G A. New substructure filters for removal of pan assay interference compounds (PAINS) from screening libraries and for their exclusion in bioassays[J]. Journal of medicinal chemistry, 2010, 53(7): 2719-2740.
Genotoxic Carcinogenicity Rule	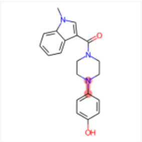	1. [1] Benigni R, Bossa C. Structure alerts for carcinogenicity, and the Salmonella assay system: a novel insight through the chemical relational databases technology[J]. Mutation Research/Reviews in Mutation Research, 2008, 659(3): 248-261.<br>2. [2] Ashby J, Tennant R W. Chemical structure, Salmonella mutagenicity and extent of carcinogenicity as indicators of genotoxic carcinogenesis among 222 chemicals tested in rodents by the US NCI/NTP[J]. Mutation Research/Genetic Toxicology, 1988, 204(1): 17-115.<br>3. [3] Kazius J, McGuire R, Bursi R. Derivation and validation of toxicophores for mutagenicity prediction[J]. Journal of medicinal chemistry, 2005, 48(1): 312-320.<br>4. [4] Bailey A B, Chanderbhan R, Collazo-Braier N, et al. The use of structure-activity relationship analysis in the food contact notification program[J]. Regulatory Toxicology and Pharmacology, 2005, 42(2): 225-235.
NTD	✔	1. [1] Brenk R, Schipani A, James D, et al. Lessons learnt from assembling screening libraries for drug discovery for neglected diseases[J]. ChemMedChem: Chemistry Enabling Drug Discovery, 2008, 3(3): 435-444.
SureChEMBL Rule	✔	1. [1] Sushko I, Salmina E, Potemkin V A, et al. ToxAlerts: a web server of structural alerts for toxic chemicals and compounds with potential adverse reactions[J]. 2012.

Compounds AI27:

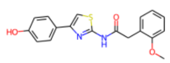

SMILES: COc1ccccc1CC(=O)Nc1nc(-c2ccc(O)cc2)cs1

San ID	No data
CAS	No data
InChI	InChI=1S/C18H16N2O3S/c1-23-16-5-3-2-4-13(16)10-17(22)20-18-19-15(11-24-18)12-6-8-14(21)9-7-12/h2-9,11,21H,10H2,1H3,(H,19,20,22)
InChIKey	JXGYIUDKJWWQBL-UHFFFAOYSA-N

View in third-party databases: Loading...

MW	340.09	HBA	5	HBD	2
PAINS Alert	false	QED	0.74	NRot	5
TPSA	71.45	logD	4.36	logP	4.58
logS	-4.59				

Bad Average Good Scoring Notice

Absorption

Caco2	-4.95	HIA	96.39%	P-gp Inhibitor	47.43%
Bioavailability	99.96%	HFE	-10.11		

Distribution

BBB Penetration	61.33%	PPBR	1.00%	VDSS	2.65

Metabolism

CYP2D6 Inhibitor	13.65%	CYP3A4 Inhibitor	76.55%	CYP2C9 Inhibitor	83.19%
CYP2D6 Substrate	3.54%	CYP3A4 Substrate	41.95%	CYP2C9 Substrate	99.74%
CYP2C19 Inhibitor	89.82%	CYP1A2 Inhibitor	90.49%		

Excretion

Half Life (hour)	3.89	Clearance (mL/min/g)	57.58

Tox

hERG	71.01%	DILI	88.19%	NR-AhR	46.53%
Carcinogencity	0.41%	LD_{50}	1837.61	Respiratory Toxicity	38.88%
Eye Corrosion	0.01%				

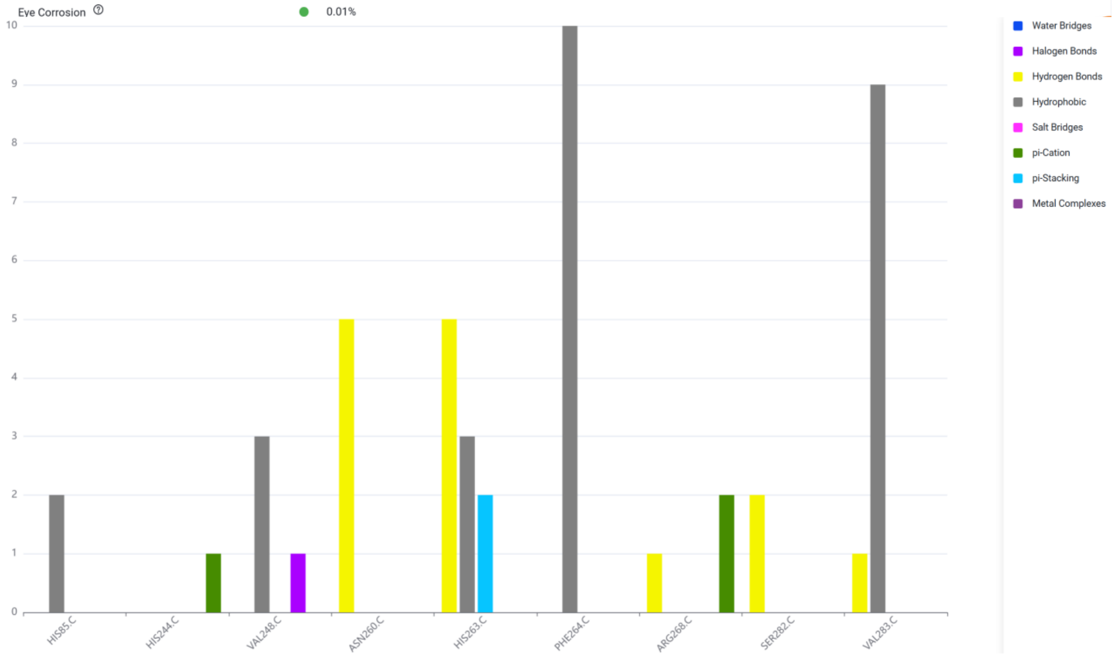

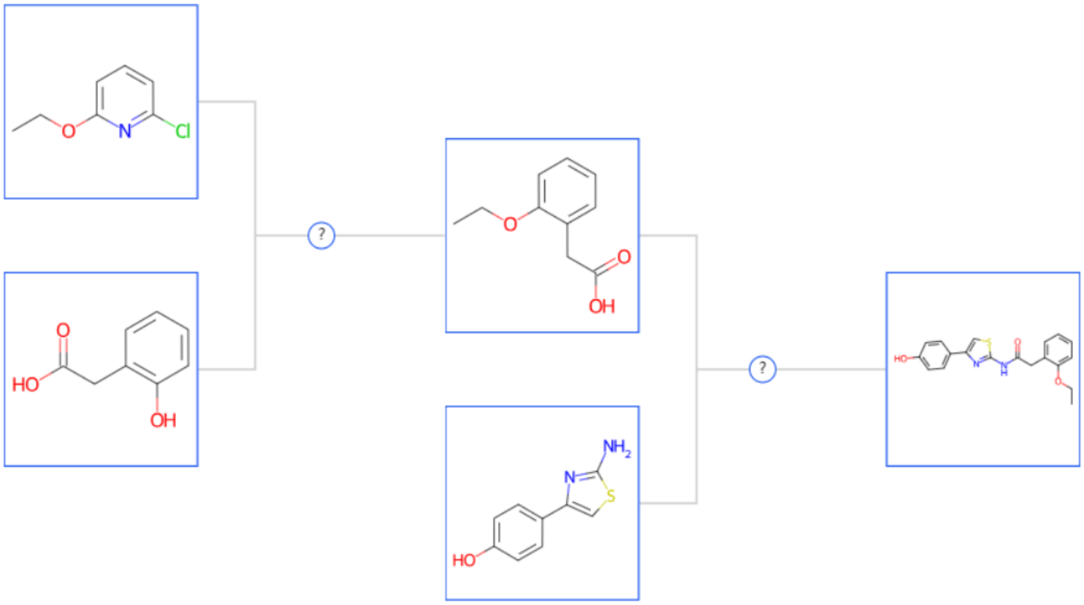

Alert Rule	Alert Structure	Reference
BMS Rule	✓	1. [1] Huth J R, Mendoza R, Olejniczak E T, et al. ALARM NMR: a rapid and robust experimental method to detect reactive false positives in biochemical screens[J]. Journal of the American Chemical Society, 2005, 127(1): 217-224.
Chelator Rule	✓	1. [1] Agrawal A, Johnson S L, Jacobsen J A, et al. Chelator fragment libraries for targeting metalloproteinases[J]. ChemMedChem: Chemistry Enabling Drug Discovery, 2010, 5(2): 195-199.
PAINS	✓	1. [1] Baell J B, Holloway G A. New substructure filters for removal of pan assay interference compounds (PAINS) from screening libraries and for their exclusion in bioassays[J]. Journal of medicinal chemistry, 2010, 53(7): 2719-2740.
Genotoxic Carcinogenicity Rule	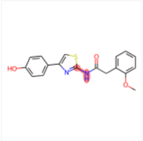	1. [1] Benigni R, Bossa C. Structure alerts for carcinogenicity, and the Salmonella assay system: a novel insight through the chemical relational databases technology[J]. Mutation Research/Reviews in Mutation Research, 2008, 659(3): 248-261.<br>2. [2] Ashby J, Tennant R W. Chemical structure, Salmonella mutagenicity and extent of carcinogenicity as indicators of genotoxic carcinogenesis among 222 chemicals tested in rodents by the US NCI/NTP[J]. Mutation Research/Genetic Toxicology, 1988, 204(1): 17-115.<br>3. [3] Kazius J, McGuire R, Bursi R. Derivation and validation of toxicophores for mutagenicity prediction[J]. Journal of medicinal chemistry, 2005, 48(1): 312-320.<br>4. [4] Bailey A B, Chanderbhan R, Collazo-Braier N, et al. The use of structure-activity relationship analysis in the food contact notification program[J]. Regulatory Toxicology and Pharmacology, 2005, 42(2): 225-235.
NTD	✓	1. [1] Brenk R, Schipani A, James D, et al. Lessons learnt from assembling screening libraries for drug discovery for neglected diseases[J]. ChemMedChem: Chemistry Enabling Drug Discovery, 2008, 3(3): 435-444.
SureChEMBL Rule	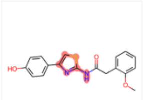	1. [1] Sushko I, Salmina E, Potemkin V A, et al. ToxAlerts: a web server of structural alerts for toxic chemicals and compounds with potential adverse reactions[J]. 2012.

Compounds AI28:

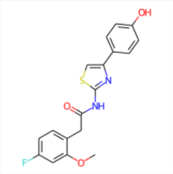

SMILES: COc1cc(F)ccc1CC(=O)Nc1nc(-c2ccc(O)cc2)cs1

San ID	No data
CAS	No data
InChI	InChI=1S/C18H15FN2O3S/c1-24-16-9-13(19)5-2-12(16)8-17(23)21-18-20-15(10-25-18)11-3-6-14(22)7-4-11/h2-7,9-10,22H,8H2,1H3,(H,20,21,23)
InChIKey	NVVZLKACHYMXCV-UHFFFAOYSA-N

View in third-party databases: No data

MW	358.08	HBA	5	HBD	2
PAINS Alert	false	QED	0.72	NRot	5
TPSA	71.45	logD	4.34	logP	4.82
logS	-4.80				

Bad Average Good Scoring Notice

Absorption

Caco2	-4.91	HIA	100.00%	P-gp Inhibitor	35.77%
Bioavailability	99.99%	HFE	-10.24		

Distribution

BBB Penetration	73.66%	PPBR	1.00%	VDSS	2.89

Metabolism

CYP2D6 Inhibitor	14.63%	CYP3A4 Inhibitor	74.86%	CYP2C9 Inhibitor	82.44%
CYP2D6 Substrate	3.23%	CYP3A4 Substrate	43.56%	CYP2C9 Substrate	96.93%
CYP2C19 Inhibitor	90.91%	CYP1A2 Inhibitor	92.33%		

Excretion

Half Life (hour)	3.50	Clearance (mL/min/g)	57.22

Tox

hERG	65.72%	DILI	96.01%	NR-AhR	46.15%
Carcinogencity	0.35%	LD_{50}	998.27	Respiratory Toxicity	30.16%
Eye Corrosion	0.01%				

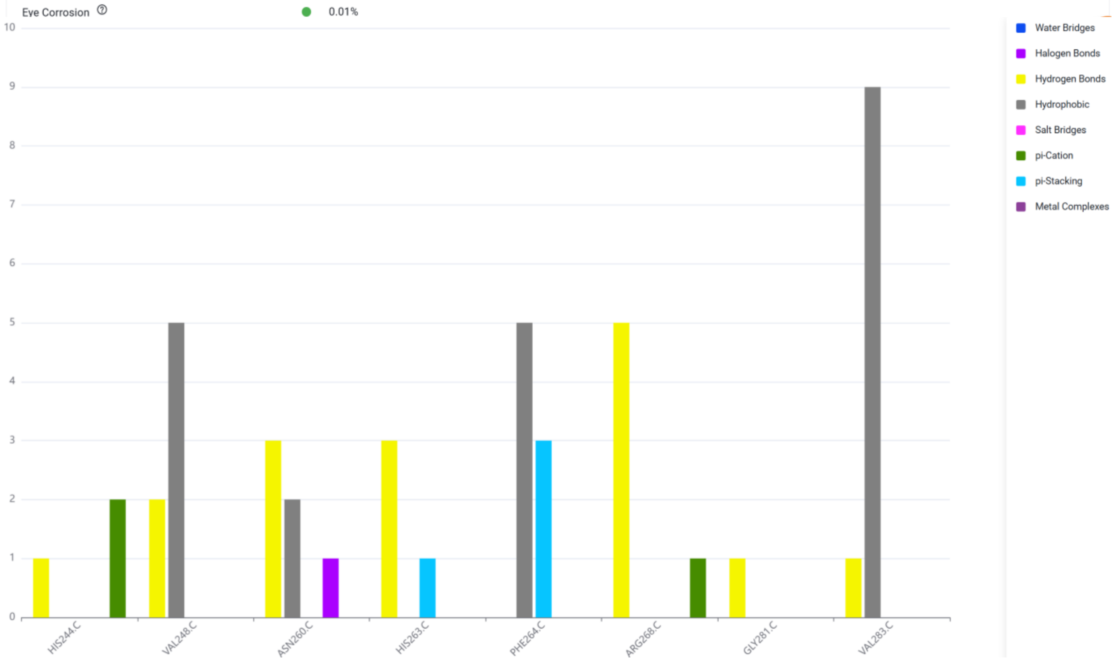

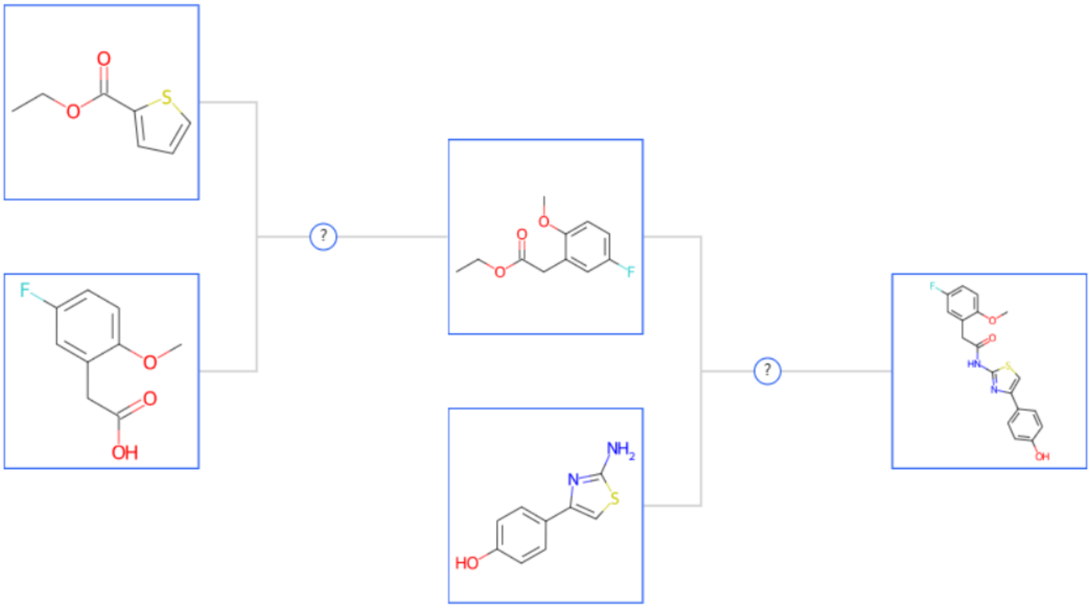

Alert Rule	Alert Structure	Reference
BMS Rule	✓	1. [1] Huth J R, Mendoza R, Olejniczak E T, et al. ALARM NMR: a rapid and robust experimental method to detect reactive false positives in biochemical screens[J]. Journal of the American Chemical Society, 2005, 127(1): 217-224.
Chelator Rule	✓	1. [1] Agrawal A, Johnson S L, Jacobsen J A, et al. Chelator fragment libraries for targeting metalloproteinases[J]. ChemMedChem: Chemistry Enabling Drug Discovery, 2010, 5(2): 195-199.
PAINS	✓	1. [1] Baell J B, Holloway G A. New substructure filters for removal of pan assay interference compounds (PAINS) from screening libraries and for their exclusion in bioassays[J]. Journal of medicinal chemistry, 2010, 53(7): 2719-2740.
Genotoxic Carcinogenicity Rule	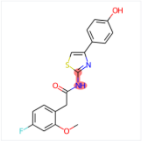	1. [1] Benigni R, Bossa C. Structure alerts for carcinogenicity, and the Salmonella assay system: a novel insight through the chemical relational databases technology[J]. Mutation Research/Reviews in Mutation Research, 2008, 659(3): 248-261. 2. [2] Ashby J, Tennant R W. Chemical structure, Salmonella mutagenicity and extent of carcinogenicity as indicators of genotoxic carcinogenesis among 222 chemicals tested in rodents by the US NCI/NTP[J]. Mutation Research/Genetic Toxicology, 1988, 204(1): 17-115. 3. [3] Kazius J, McGuire R, Bursi R. Derivation and validation of toxicophores for mutagenicity prediction[J]. Journal of medicinal chemistry, 2005, 48(1): 312-320. 4. [4] Bailey A B, Chanderbhan R, Collazo-Braier N, et al. The use of structure-activity relationship analysis in the food contact notification program[J]. Regulatory Toxicology and Pharmacology, 2005, 42(2): 225-235.
NTD	✓	1. [1] Brenk R, Schipani A, James D, et al. Lessons learnt from assembling screening libraries for drug discovery for neglected diseases[J]. ChemMedChem: Chemistry Enabling Drug Discovery, 2008, 3(3): 435-444.
SureChEMBL Rule	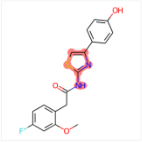	1. [1] Sushko I, Salmina E, Potemkin V A, et al. ToxAlerts: a web server of structural alerts for toxic chemicals and compounds with potential adverse reactions[J]. 2012.

Compounds AI10-a1:

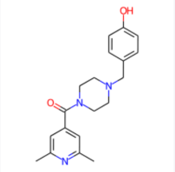

SMILES: Cc1cc(C(=O)N2CCN(Cc3ccc(O)cc3)CC2)cc(C)n1

San ID	No data
CAS	No data
InChI	InChI=1S/C19H23N3O2/c1-14-11-17(12-15(2)20-14)19(24)22-9-7-21(8-10-22)13-16-3-5-18(23)6-4-16/h3-6,11-12,23H,7-10,13H2,1-2H3
InChIKey	HVJANXACIGBWLF-UHFFFAOYSA-N

View in third-party databases: No data

MW	325.18	HBA	4	HBD	1
PAINS Alert	false	QED	0.941	NRot	3
TPSA	56.67	logD	1.58	logP	1.33
logS	-2.15				

Bad Average Good Scoring Notice

Absorption

Caco2	-4.76	HIA	99.79%	P-gp Inhibitor	21.14%
Bioavailability	99.37%	HFE	-10.72		

Distribution

BBB Penetration	80.88%	PPBR	1.00%	VDSS	2.23

Metabolism

CYP2D6 Inhibitor	34.60%	CYP3A4 Inhibitor	19.28%	CYP2C9 Inhibitor	27.15%
CYP2D6 Substrate	98.62%	CYP3A4 Substrate	66.53%	CYP2C9 Substrate	0.01%
CYP2C19 Inhibitor	34.58%	CYP1A2 Inhibitor	8.92%		

Excretion

Half Life (hour)	3.21	Clearance (mL/min/g)	47.16

Tox

hERG	89.07%	DILI	34.81%	NR-AhR	4.55%
Carcinogencity	0.21%	LD_{50}	698.51	Respiratory Toxicity	99.88%
Eye Corrosion	0.25%				

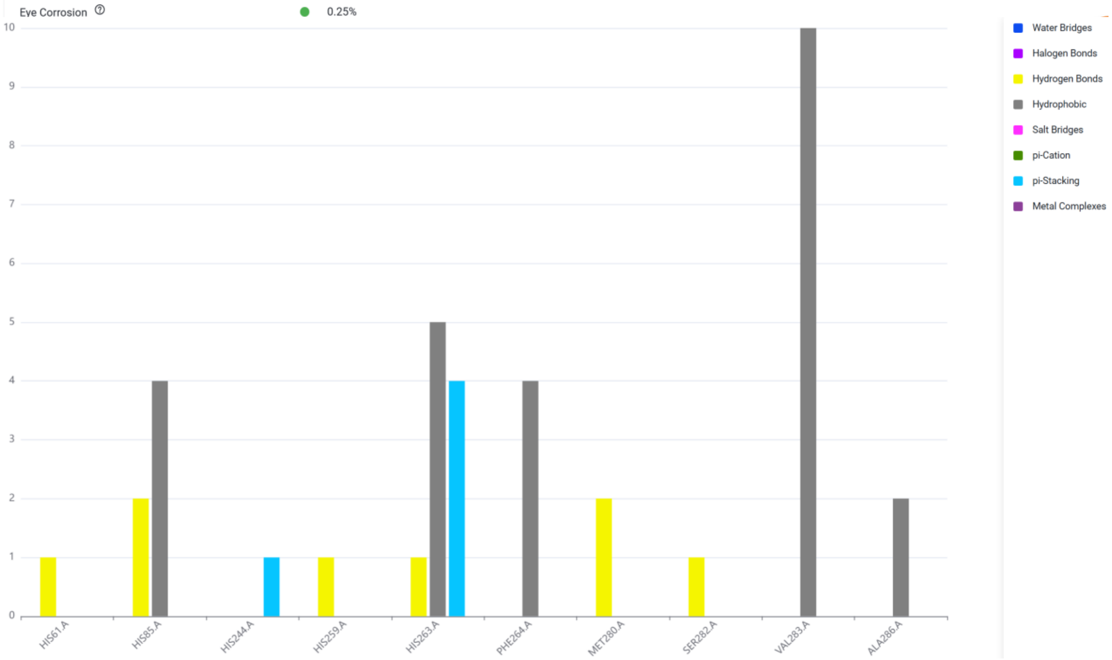

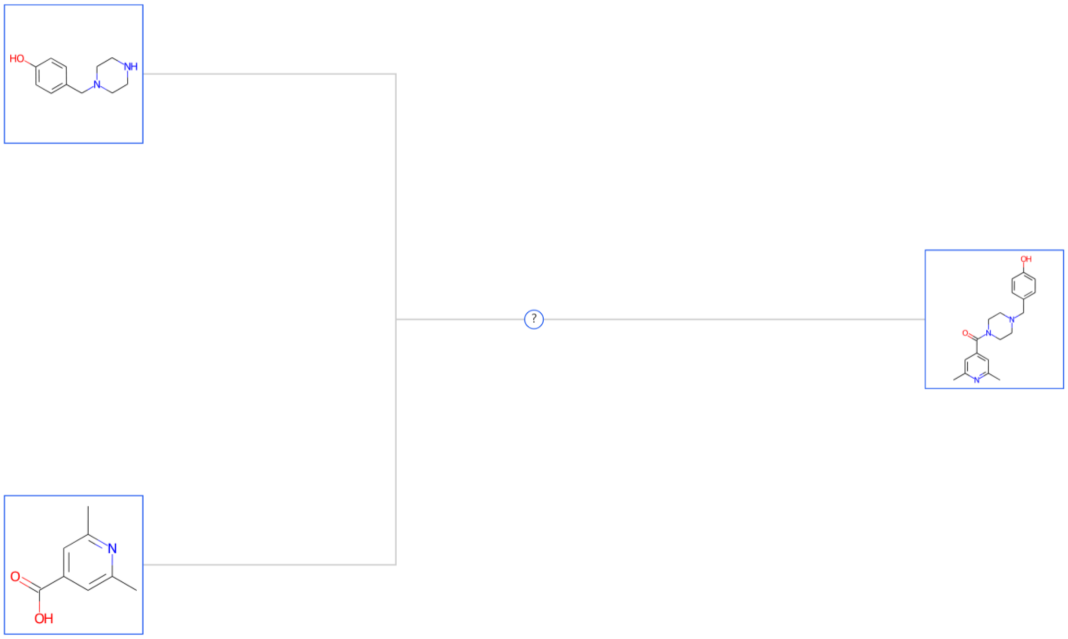

Alert Rule	Alert Structure	Reference
BMS Rule	✓	1. [1] Huth J R, Mendoza R, Olejniczak E T, et al. ALARM NMR: a rapid and robust experimental method to detect reactive false positives in biochemical screens[J]. Journal of the American Chemical Society, 2005, 127(1): 217-224.
Chelator Rule	✓	1. [1] Agrawal A, Johnson S L, Jacobsen J A, et al. Chelator fragment libraries for targeting metalloproteinases[J]. ChemMedChem: Chemistry Enabling Drug Discovery, 2010, 5(2): 195-199.
PAINS	✓	1. [1] Baell J B, Holloway G A. New substructure filters for removal of pan assay interference compounds (PAINS) from screening libraries and for their exclusion in bioassays[J]. Journal of medicinal chemistry, 2010, 53(7): 2719-2740.
Genotoxic Carcinogenicity Rule	✓	1. [1] Benigni R, Bossa C. Structure alerts for carcinogenicity, and the Salmonella assay system: a novel insight through the chemical relational databases technology[J]. Mutation Research/Reviews in Mutation Research, 2008, 659(3): 248-261.<br>2. [2] Ashby J, Tennant R W. Chemical structure, Salmonella mutagenicity and extent of carcinogenicity as indicators of genotoxic carcinogenesis among 222 chemicals tested in rodents by the US NCI/NTP[J]. Mutation Research/Genetic Toxicology, 1988, 204(1): 17-115.<br>3. [3] Kazius J, McGuire R, Bursi R. Derivation and validation of toxicophores for mutagenicity prediction[J]. Journal of medicinal chemistry, 2005, 48(1): 312-320.<br>4. [4] Bailey A B, Chanderbhan R, Collazo-Braier N, et al. The use of structure-activity relationship analysis in the food contact notification program[J]. Regulatory Toxicology and Pharmacology, 2005, 42(2): 225-235.
NTD	✓	1. [1] Brenk R, Schipani A, James D, et al. Lessons learnt from assembling screening libraries for drug discovery for neglected diseases[J]. ChemMedChem: Chemistry Enabling Drug Discovery, 2008, 3(3): 435-444.
SureChEMBL Rule	✓	1. [1] Sushko I, Salmina E, Potemkin V A, et al. ToxAlerts: a web server of structural alerts for toxic chemicals and compounds with potential adverse reactions[J]. 2012.

Compounds AI10-a2:

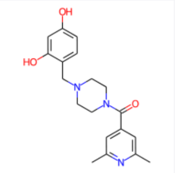

SMILES: Cc1cc(C(=O)N2CCN(Cc3ccc(O)cc3O)CC2)cc(C)n1

San ID	No data
CAS	No data
InChI	InChI=1S/C19H23N3O3/c1-13-9-16(10-14(2)20-13)19(25)22-7-5-21(6-8-22)12-15-3-4-17(23)11-18(15)24/h3-4,9-11,23-24H,5-8,12H2,1-2H3
InChIKey	DGDKCAXJOJNEDI-UHFFFAOYSA-N

View in third-party databases: Loading...

MW	341.17	HBA	5	HBD	2
PAINS Alert	true	QED	0.894	NRot	3
TPSA	76.9	logD	1.27	logP	0.7
logS	-2.75				

Bad Average Good Scoring Notice

Absorption

Caco2	-4.85	HIA	100.00%	P-gp Inhibitor	37.74%
Bioavailability	97.18%	HFE	-10.96		

Distribution

BBB Penetration	67.43%	PPBR	1.00%	VDSS	1.27

Metabolism

CYP2D6 Inhibitor	27.62%	CYP3A4 Inhibitor	35.53%	CYP2C9 Inhibitor	46.66%
CYP2D6 Substrate	92.36%	CYP3A4 Substrate	77.52%	CYP2C9 Substrate	0.01%
CYP2C19 Inhibitor	61.69%	CYP1A2 Inhibitor	9.79%		

Excretion

Half Life (hour)	3.25	Clearance (mL/min/g)	52.32

Tox

hERG	85.24%	DILI	27.88%	NR-AhR	9.93%
Carcinogencity	0.03%	LD_{50}	512.38	Respiratory Toxicity	99.84%
Eye Corrosion	0.37%				

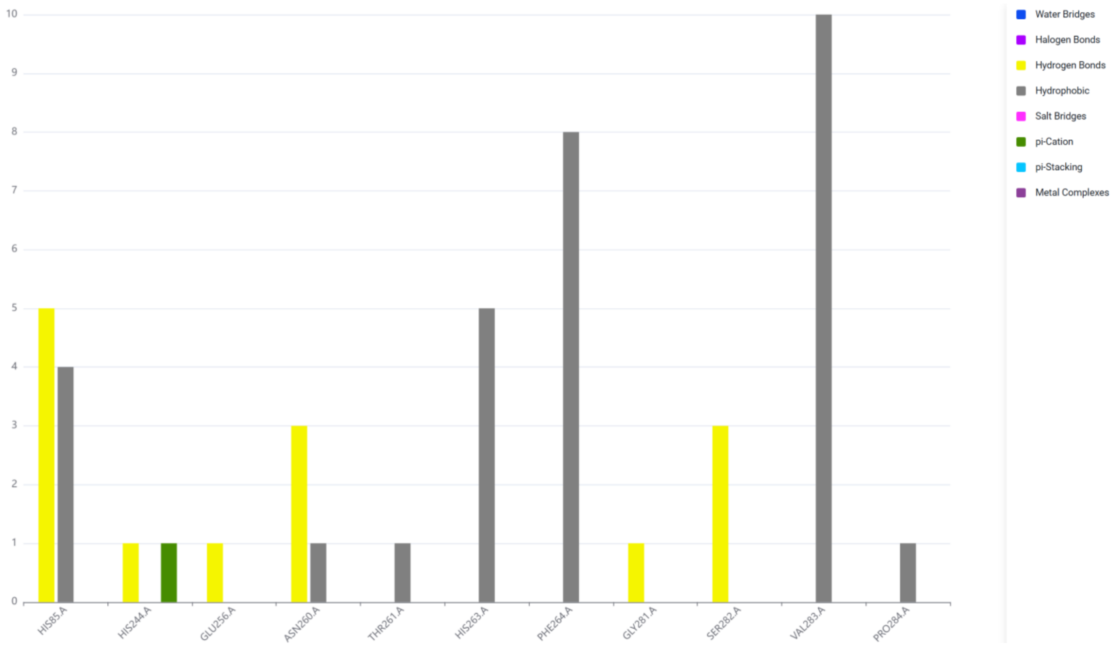

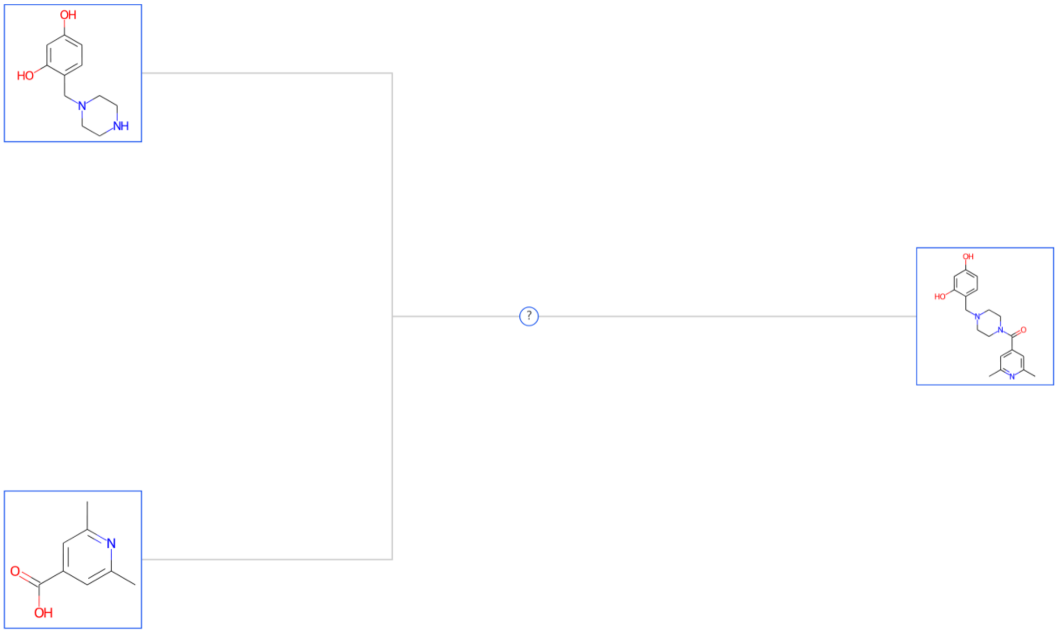

Alert Rule	Alert Structure	Reference
BMS Rule	✔	1. [1] Huth J R, Mendoza R, Olejniczak E T, et al. ALARM NMR: a rapid and robust experimental method to detect reactive false positives in biochemical screens[J]. Journal of the American Chemical Society, 2005, 127(1): 217-224.
Chelator Rule	✔	1. [1] Agrawal A, Johnson S L, Jacobsen J A, et al. Chelator fragment libraries for targeting metalloproteinases[J]. ChemMedChem: Chemistry Enabling Drug Discovery, 2010, 5(2): 195-199.
PAINS	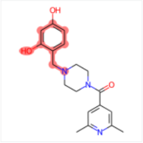	1. [1] Baell J B, Holloway G A. New substructure filters for removal of pan assay interference compounds (PAINS) from screening libraries and for their exclusion in bioassays[J]. Journal of medicinal chemistry, 2010, 53(7): 2719-2740.
Genotoxic Carcinogenicity Rule	✔	1. [1] Benigni R, Bossa C. Structure alerts for carcinogenicity, and the Salmonella assay system: a novel insight through the chemical relational databases technology[J]. Mutation Research/Reviews in Mutation Research, 2008, 659(3): 248-261.<br>2. [2] Ashby J, Tennant R W. Chemical structure, Salmonella mutagenicity and extent of carcinogenicity as indicators of genotoxic carcinogenesis among 222 chemicals tested in rodents by the US NCI/NTP[J]. Mutation Research/Genetic Toxicology, 1988, 204(1): 17-115.<br>3. [3] Kazius J, McGuire R, Bursi R. Derivation and validation of toxicophores for mutagenicity prediction[J]. Journal of medicinal chemistry, 2005, 48(1): 312-320.<br>4. [4] Bailey A B, Chanderbhan R, Collazo-Braier N, et al. The use of structure-activity relationship analysis in the food contact notification program[J]. Regulatory Toxicology and Pharmacology, 2005, 42(2): 225-235.
NTD	✔	1. [1] Brenk R, Schipani A, James D, et al. Lessons learnt from assembling screening libraries for drug discovery for neglected diseases[J]. ChemMedChem: Chemistry Enabling Drug Discovery, 2008, 3(3): 435-444.
SureChEMBL Rule	✔	1. [1] Sushko I, Salmina E, Potemkin V A, et al. ToxAlerts: a web server of structural alerts for toxic chemicals and compounds with potential adverse reactions[J]. 2012.

Compounds AI10-a3:

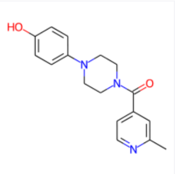

SMILES: Cc1cc(C(=O)N2CCN(c3ccc(O)cc3)CC2)ccn1

**San ID**	b43010683d3c9c3acb8957dff3d45fbb
**CAS**	No data
**InChI**	InChI=1S/C17H19N3O2/c1-13-12-14(6-7-18-13)17(22)20-10-8-19(9-11-20)15-2-4-16(21)5-3-15/h2-7,12,21H,8-11H2,1H3
**InChIKey**	SXFUBPYXGKQDGV-UHFFFAOYSA-N

View in third-party databases: No data

MW	297.15	HBA	4	HBD	1
PAINS Alert	false	QED	0.922	NRot	2
TPSA	56.67	logD	1.01	logP	1.16
logS	-2.43				

Bad Average Good Scoring Notice

Absorption

Caco2	-4.60	HIA	99.51%	P-gp Inhibitor	13.26%
Bioavailability	99.99%	HFE	-10.06		

Distribution

BBB Penetration	82.60%	PPBR	1.00%	VDSS	2.40

Metabolism

CYP2D6 Inhibitor	4.71%	CYP3A4 Inhibitor	47.39%	CYP2C9 Inhibitor	63.50%
CYP2D6 Substrate	98.06%	CYP3A4 Substrate	44.77%	CYP2C9 Substrate	0.32%
CYP2C19 Inhibitor	51.84%	CYP1A2 Inhibitor	6.89%		

Excretion

Half Life (hour)	3.57	Clearance (mL/min/g)	50.67

Tox

hERG	88.16%	DILI	33.82%	NR-AhR	21.75%
Carcinogencity	25.82%	LD_{50}	838.18	Respiratory Toxicity	44.90%
Eye Corrosion	0.42%				

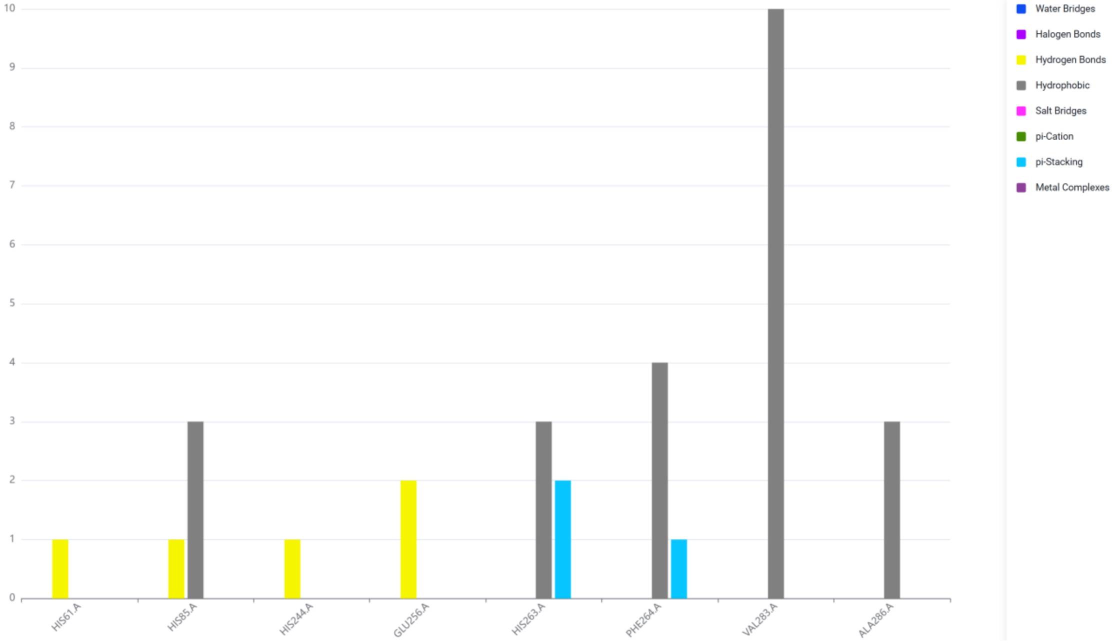

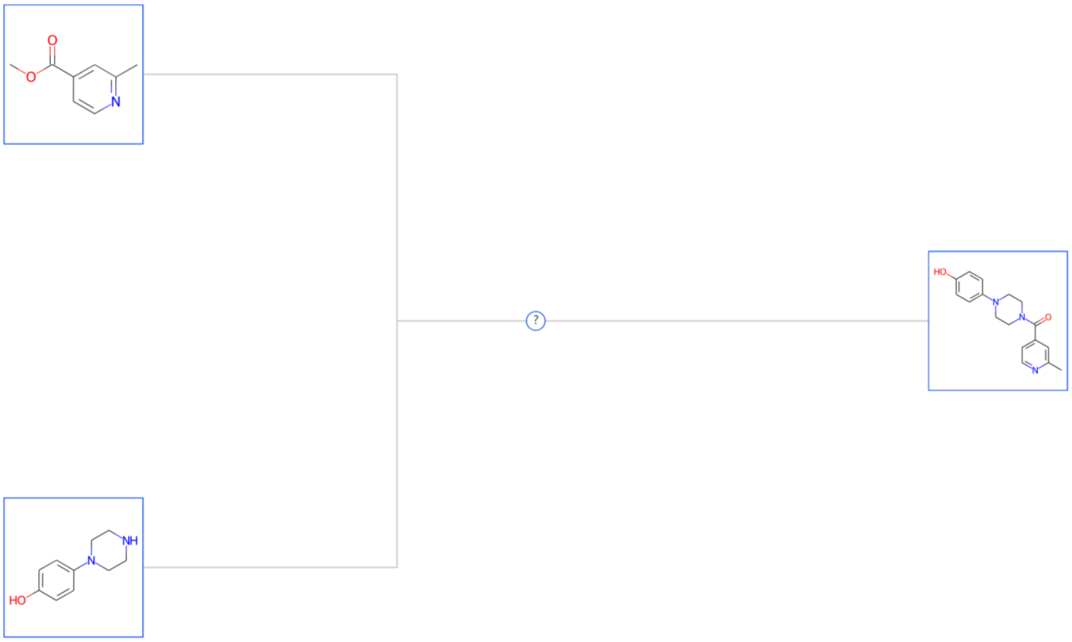

Alert Rule	Alert Structure	Reference
BMS Rule	✓	1. [1] Huth J R, Mendoza R, Olejniczak E T, et al. ALARM NMR: a rapid and robust experimental method to detect reactive false positives in biochemical screens[J]. Journal of the American Chemical Society, 2005, 127(1): 217-224.
Chelator Rule	✓	1. [1] Agrawal A, Johnson S L, Jacobsen J A, et al. Chelator fragment libraries for targeting metalloproteinases[J]. ChemMedChem: Chemistry Enabling Drug Discovery, 2010, 5(2): 195-199.
PAINS	✓	1. [1] Baell J B, Holloway G A. New substructure filters for removal of pan assay interference compounds (PAINS) from screening libraries and for their exclusion in bioassays[J]. Journal of medicinal chemistry, 2010, 53(7): 2719-2740.
Genotoxic Carcinogenicity Rule	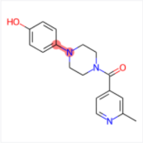	1. [1] Benigni R, Bossa C. Structure alerts for carcinogenicity, and the Salmonella assay system: a novel insight through the chemical relational databases technology[J]. Mutation Research/Reviews in Mutation Research, 2008, 659(3): 248-261.<br>2. [2] Ashby J, Tennant R W. Chemical structure, Salmonella mutagenicity and extent of carcinogenicity as indicators of genotoxic carcinogenesis among 222 chemicals tested in rodents by the US NCI/NTP[J]. Mutation Research/Genetic Toxicology, 1988, 204(1): 17-115.<br>3. [3] Kazius J, McGuire R, Bursi R. Derivation and validation of toxicophores for mutagenicity prediction[J]. Journal of medicinal chemistry, 2005, 48(1): 312-320.<br>4. [4] Bailey A B, Chanderbhan R, Collazo-Braier N, et al. The use of structure-activity relationship analysis in the food contact notification program[J]. Regulatory Toxicology and Pharmacology, 2005, 42(2): 225-235.
NTD	✓	1. [1] Brenk R, Schipani A, James D, et al. Lessons learnt from assembling screening libraries for drug discovery for neglected diseases[J]. ChemMedChem: Chemistry Enabling Drug Discovery, 2008, 3(3): 435-444.
SureChEMBL Rule	✓	1. [1] Sushko I, Salmina E, Potemkin V A, et al. ToxAlerts: a web server of structural alerts for toxic chemicals and compounds with potential adverse reactions[J]. 2012.

Compounds AI10-a4:

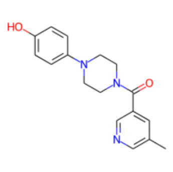

SMILES: Cc1cncc(C(=O)N2CCN(c3ccc(O)cc3)CC2)c1

**San ID**	7b9f1afbf65c3410b25f69e4f7c30af4
**CAS**	No data
**InChI**	InChI=1S/C17H19N3O2/c1-13-10-14(12-18-11-13)17(22)20-8-6-19(7-9-20)15-2-4-16(21)5-3-15/h2-5,10-12,21H,6-9H2,1H3
**InChIKey**	LUHTVHJOYIGDBP-UHFFFAOYSA-N

View in third-party databases: No data

MW	297.15	HBA	4	HBD	1
PAINS Alert	false	QED	0.922	NRot	2
TPSA	56.67	logD	1.53	logP	1.35
logS	-2.38				

Bad Average Good Scoring Notice

Absorption

Caco2	-4.66	HIA	98.42%	P-gp Inhibitor	12.58%
Bioavailability	99.96%	HFE	-9.93		

Distribution

BBB Penetration	90.62%	PPBR	1.00%	VDSS	1.49

Metabolism

CYP2D6 Inhibitor	4.76%	CYP3A4 Inhibitor	73.73%	CYP2C9 Inhibitor	72.24%
CYP2D6 Substrate	94.27%	CYP3A4 Substrate	49.27%	CYP2C9 Substrate	0.21%
CYP2C19 Inhibitor	61.30%	CYP1A2 Inhibitor	8.57%		

Excretion

Half Life (hour)	3.24	Clearance (mL/min/g)	49.22

Tox

hERG	87.76%	DILI	27.15%	NR-AhR	23.86%
Carcinogencity	48.48%	LD_{50}	1126.16	Respiratory Toxicity	30.42%
Eye Corrosion	0.21%				

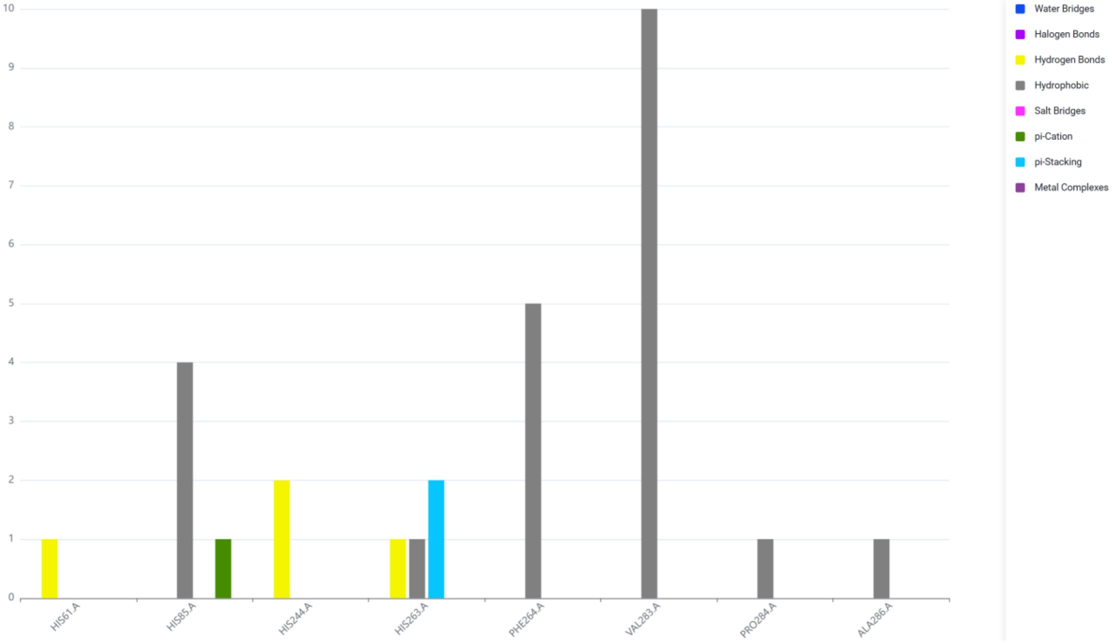

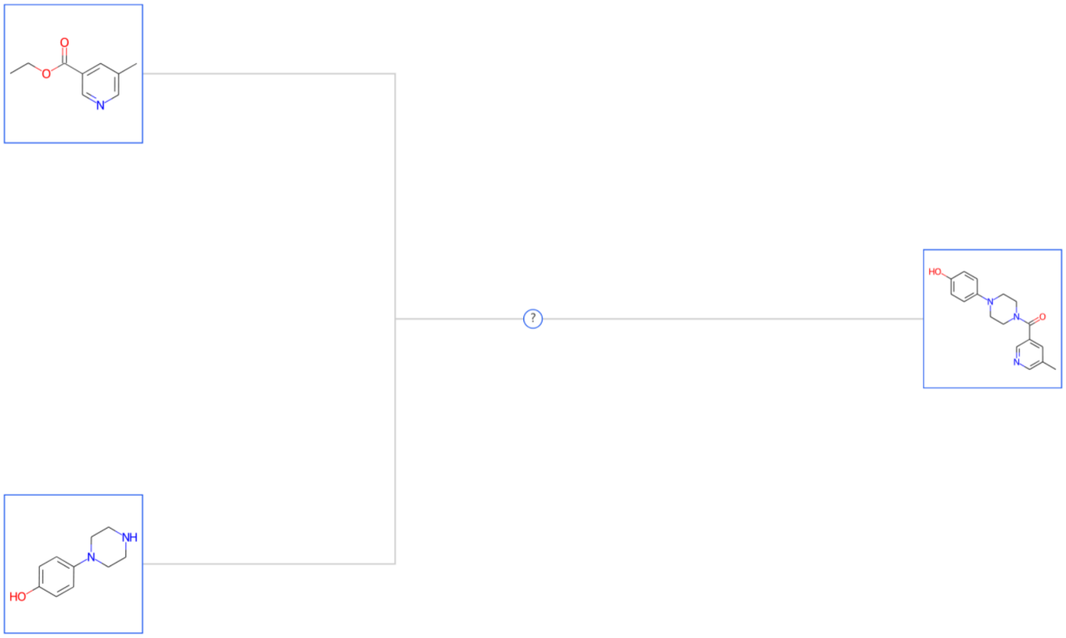

Alert Rule	Alert Structure	Reference
BMS Rule	✔	1. [1] Huth J R, Mendoza R, Olejniczak E T, et al. ALARM NMR: a rapid and robust experimental method to detect reactive false positives in biochemical screens[J]. Journal of the American Chemical Society, 2005, 127(1): 217-224.
Chelator Rule	✔	1. [1] Agrawal A, Johnson S L, Jacobsen J A, et al. Chelator fragment libraries for targeting metalloproteinases[J]. ChemMedChem: Chemistry Enabling Drug Discovery, 2010, 5(2): 195-199.
PAINS	✔	1. [1] Baell J B, Holloway G A. New substructure filters for removal of pan assay interference compounds (PAINS) from screening libraries and for their exclusion in bioassays[J]. Journal of medicinal chemistry, 2010, 53(7): 2719-2740.
Genotoxic Carcinogenicity Rule	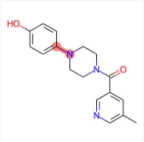	1. [1] Benigni R, Bossa C. Structure alerts for carcinogenicity, and the Salmonella assay system: a novel insight through the chemical relational databases technology[J]. Mutation Research/Reviews in Mutation Research, 2008, 659(3): 248-261. 2. [2] Ashby J, Tennant R W. Chemical structure, Salmonella mutagenicity and extent of carcinogenicity as indicators of genotoxic carcinogenesis among 222 chemicals tested in rodents by the US NCI/NTP[J]. Mutation Research/Genetic Toxicology, 1988, 204(1): 17-115. 3. [3] Kazius J, McGuire R, Bursi R. Derivation and validation of toxicophores for mutagenicity prediction[J]. Journal of medicinal chemistry, 2005, 48(1): 312-320. 4. [4] Bailey A B, Chanderbhan R, Collazo-Braier N, et al. The use of structure-activity relationship analysis in the food contact notification program[J]. Regulatory Toxicology and Pharmacology, 2005, 42(2): 225-235.
NTD	✔	1. [1] Brenk R, Schipani A, James D, et al. Lessons learnt from assembling screening libraries for drug discovery for neglected diseases[J]. ChemMedChem: Chemistry Enabling Drug Discovery, 2008, 3(3): 435-444.
SureChEMBL Rule	✔	1. [1] Sushko I, Salmina E, Potemkin V A, et al. ToxAlerts: a web server of structural alerts for toxic chemicals and compounds with potential adverse reactions[J]. 2012.

Compounds AI10-a5:

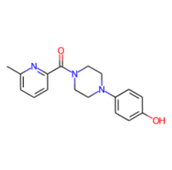

SMILES: Cc1cccc(C(=O)N2CCN(c3ccc(O)cc3)CC2)n1

San ID	7291583040974a70c77e655b6451f434
CAS	No data
InChI	InChI=1S/C17H19N3O2/c1-13-3-2-4-16(18-13)17(22)20-11-9-19(10-12-20)14-5-7-15(21)8-6-14/h2-8,21H,9-12H2,1H3
InChIKey	OSZSBOBXTOXRCK-UHFFFAOYSA-N

View in third-party databases: No data

MW	297.15	HBA	4	HBD	1
PAINS Alert	false	QED	0.922	NRot	2
TPSA	56.67	logD	1.45	logP	2.12
logS	-2.88				

Bad Average Good Scoring Notice

Absorption

Caco2	-4.70	HIA	100.00%	P-gp Inhibitor	15.10%
Bioavailability	99.97%	HFE	-9.62		

Distribution

BBB Penetration	74.38%	PPBR	1.00%	VDSS	1.82

Metabolism

CYP2D6 Inhibitor	3.46%	CYP3A4 Inhibitor	51.02%	CYP2C9 Inhibitor	62.18%
CYP2D6 Substrate	98.73%	CYP3A4 Substrate	44.64%	CYP2C9 Substrate	83.33%
CYP2C19 Inhibitor	56.66%	CYP1A2 Inhibitor	8.11%		

Excretion

Half Life (hour)	3.40	Clearance (mL/min/g)	53.04

Tox

hERG	90.46%	DILI	38.44%	NR-AhR	24.29%
Carcinogencity	20.70%	LD_{50}	1168.66	Respiratory Toxicity	53.66%
Eye Corrosion	0.41%				

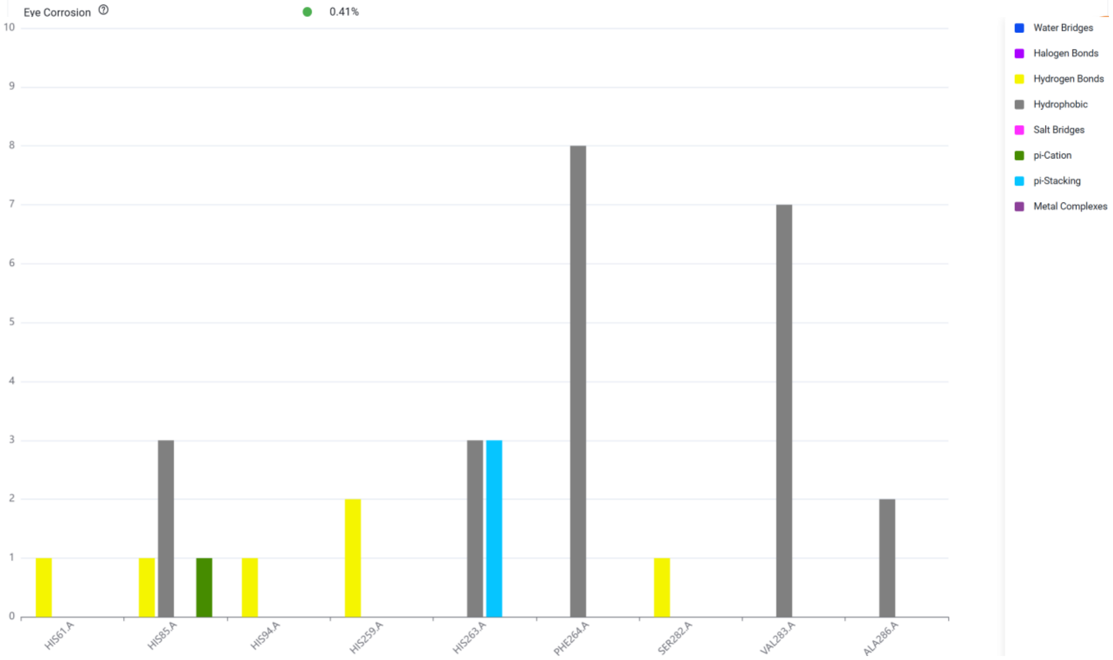

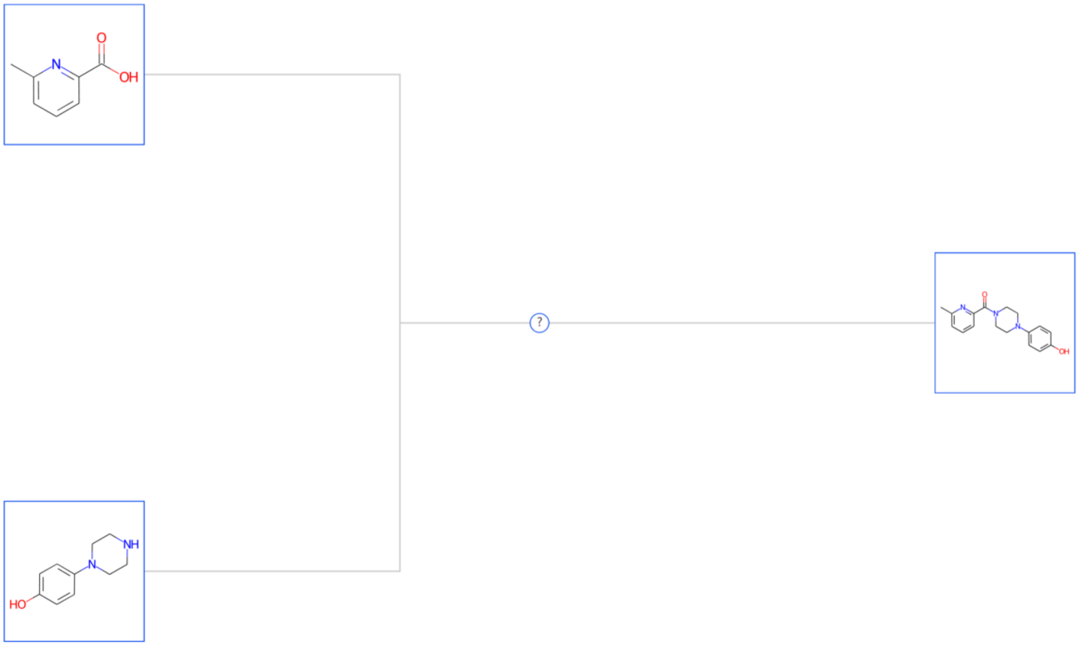

Alert Rule	Alert Structure	Reference
BMS Rule	✓	1. [1] Huth J R, Mendoza R, Olejniczak E T, et al. ALARM NMR: a rapid and robust experimental method to detect reactive false positives in biochemical screens[J]. Journal of the American Chemical Society, 2005, 127(1): 217-224.
Chelator Rule	✓	1. [1] Agrawal A, Johnson S L, Jacobsen J A, et al. Chelator fragment libraries for targeting metalloproteinases[J]. ChemMedChem: Chemistry Enabling Drug Discovery, 2010, 5(2): 195-199.
PAINS	✓	1. [1] Baell J B, Holloway G A. New substructure filters for removal of pan assay interference compounds (PAINS) from screening libraries and for their exclusion in bioassays[J]. Journal of medicinal chemistry, 2010, 53(7): 2719-2740.
Genotoxic Carcinogenicity Rule	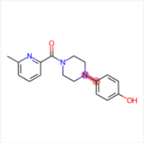	1. [1] Benigni R, Bossa C. Structure alerts for carcinogenicity, and the Salmonella assay system: a novel insight through the chemical relational databases technology[J]. Mutation Research/Reviews in Mutation Research, 2008, 659(3): 248-261.<br>2. [2] Ashby J, Tennant R W. Chemical structure, Salmonella mutagenicity and extent of carcinogenicity as indicators of genotoxic carcinogenesis among 222 chemicals tested in rodents by the US NCI/NTP[J]. Mutation Research/Genetic Toxicology, 1988, 204(1): 17-115.<br>3. [3] Kazius J, McGuire R, Bursi R. Derivation and validation of toxicophores for mutagenicity prediction[J]. Journal of medicinal chemistry, 2005, 48(1): 312-320.<br>4. [4] Bailey A B, Chanderbhan R, Collazo-Braier N, et al. The use of structure-activity relationship analysis in the food contact notification program[J]. Regulatory Toxicology and Pharmacology, 2005, 42(2): 225-235.
NTD	✓	1. [1] Brenk R, Schipani A, James D, et al. Lessons learnt from assembling screening libraries for drug discovery for neglected diseases[J]. ChemMedChem: Chemistry Enabling Drug Discovery, 2008, 3(3): 435-444.
SureChEMBL Rule	✓	1. [1] Sushko I, Salmina E, Potemkin V A, et al. ToxAlerts: a web server of structural alerts for toxic chemicals and compounds with potential adverse reactions[J]. 2012.

Compounds AI10-a6:

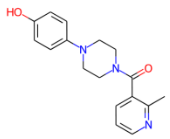

SMILES: Cc1ncccc1C(=O)N1CCN(c2ccc(O)cc2)CC1

San ID	9cd8241ae8ed073bf7f8d1f68ecb8dbf
CAS	No data
InChI	InChI=1S/C17H19N3O2/c1-13-16(3-2-8-18-13)17(22)20-11-9-19(10-12-20)14-4-6-15(21)7-5-14/h2-8,21H,9-12H2,1H3
InChIKey	SREVRRXYVUHGRF-UHFFFAOYSA-N

View in third-party databases: No data

MW	297.15	HBA	4	HBD	1
PAINS Alert	false	QED	0.922	NRot	2
TPSA	56.67	logD	1.09	logP	1.23
logS	-2.77				

Bad Average Good Scoring Notice

Absorption

Caco2	-4.69	HIA	100.00%	P-gp Inhibitor	9.56%
Bioavailability	100.00%	HFE	-10.06		

Distribution

BBB Penetration	85.43%	PPBR	1.00%	VDSS	0.85

Metabolism

CYP2D6 Inhibitor	3.15%	CYP3A4 Inhibitor	41.93%	CYP2C9 Inhibitor	56.63%
CYP2D6 Substrate	97.47%	CYP3A4 Substrate	36.71%	CYP2C9 Substrate	99.56%
CYP2C19 Inhibitor	45.44%	CYP1A2 Inhibitor	5.11%		

Excretion

Half Life (hour)	3.62	Clearance (mL/min/g)	49.18

Tox

hERG	87.98%	DILI	42.01%	NR-AhR	18.54%
Carcinogencity	4.35%	LD_{50}	466.51	Respiratory Toxicity	34.81%
Eye Corrosion	0.60%				

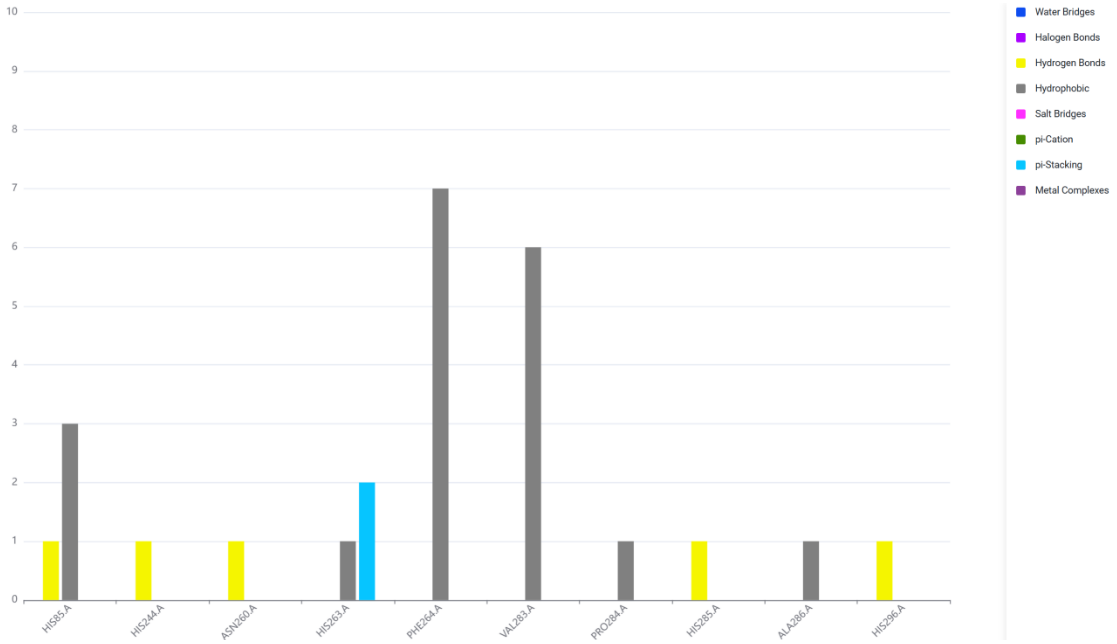

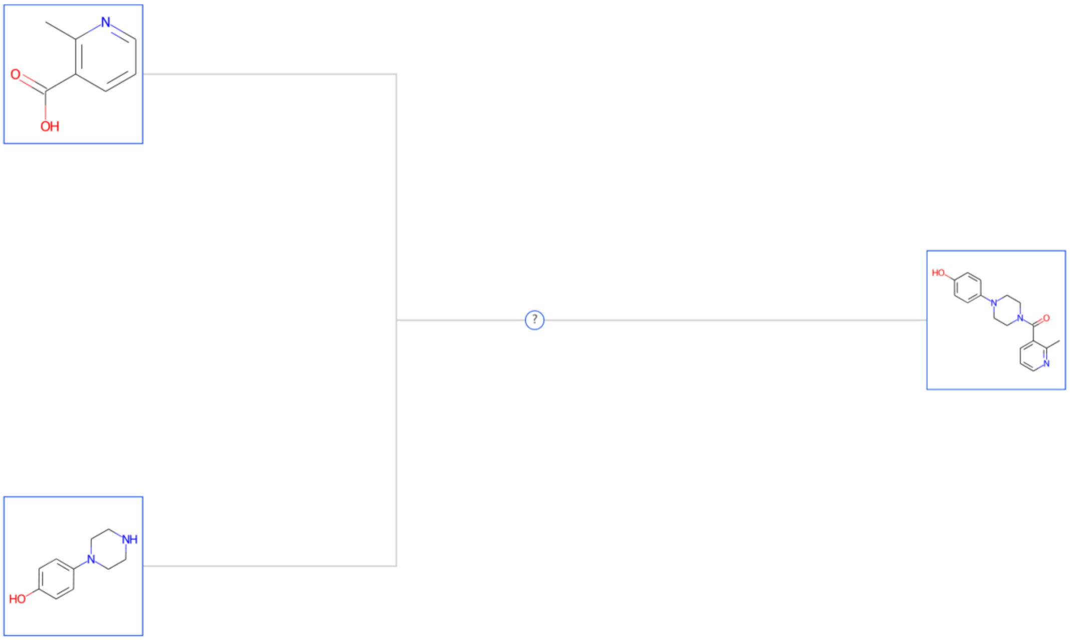

Alert Rule	Alert Structure	Reference
BMS Rule	✔	1. [1] Huth J R, Mendoza R, Olejniczak E T, et al. ALARM NMR: a rapid and robust experimental method to detect reactive false positives in biochemical screens[J]. Journal of the American Chemical Society, 2005, 127(1): 217-224.
Chelator Rule	✔	1. [1] Agrawal A, Johnson S L, Jacobsen J A, et al. Chelator fragment libraries for targeting metalloproteinases[J]. ChemMedChem: Chemistry Enabling Drug Discovery, 2010, 5(2): 195-199.
PAINS	✔	1. [1] Baell J B, Holloway G A. New substructure filters for removal of pan assay interference compounds (PAINS) from screening libraries and for their exclusion in bioassays[J]. Journal of medicinal chemistry, 2010, 53(7): 2719-2740.
Genotoxic Carcinogenicity Rule	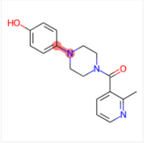	1. [1] Benigni R, Bossa C. Structure alerts for carcinogenicity, and the Salmonella assay system: a novel insight through the chemical relational databases technology[J]. Mutation Research/Reviews in Mutation Research, 2008, 659(3): 248-261.<br>2. [2] Ashby J, Tennant R W. Chemical structure, Salmonella mutagenicity and extent of carcinogenicity as indicators of genotoxic carcinogenesis among 222 chemicals tested in rodents by the US NCI/NTP[J]. Mutation Research/Genetic Toxicology, 1988, 204(1): 17-115.<br>3. [3] Kazius J, McGuire R, Bursi R. Derivation and validation of toxicophores for mutagenicity prediction[J]. Journal of medicinal chemistry, 2005, 48(1): 312-320.<br>4. [4] Bailey A B, Chanderbhan R, Collazo-Braier N, et al. The use of structure-activity relationship analysis in the food contact notification program[J]. Regulatory Toxicology and Pharmacology, 2005, 42(2): 225-235.
NTD	✔	1. [1] Brenk R, Schipani A, James D, et al. Lessons learnt from assembling screening libraries for drug discovery for neglected diseases[J]. ChemMedChem: Chemistry Enabling Drug Discovery, 2008, 3(3): 435-444.
SureChEMBL Rule	✔	1. [1] Sushko I, Salmina E, Potemkin V A, et al. ToxAlerts: a web server of structural alerts for toxic chemicals and compounds with potential adverse reactions[J]. 2012.

Compounds AI10-a7:

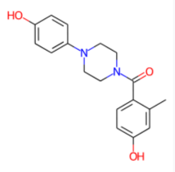

SMILES: Cc1cc(O)ccc1C(=O)N1CCN(c2ccc(O)cc2)CC1

San ID	No data
CAS	No data
InChI	InChI=1S/C18H20N2O3/c1-13-12-16(22)6-7-17(13)18(23)20-10-8-19(9-11-20)14-2-4-15(21)5-3-14/h2-7,12,21-22H,8-11H2,1H3
InChIKey	JRTMNFDUQHMMFQ-UHFFFAOYSA-N

View in third-party databases: Loading...

MW	312.15	HBA	4	HBD	2
PAINS Alert	false	QED	0.893	NRot	2
TPSA	64.01	logD	1.71	logP	1.39
logS	-2.97				

Bad Average Good Scoring Notice

Absorption

Caco2	-4.87	HIA	100.00%	P-gp Inhibitor	16.74%
Bioavailability	99.76%	HFE	-11.38		

Distribution

BBB Penetration	63.10%	PPBR	1.00%	VDSS	1.41

Metabolism

CYP2D6 Inhibitor	5.43%	CYP3A4 Inhibitor	76.95%	CYP2C9 Inhibitor	80.33%
CYP2D6 Substrate	65.56%	CYP3A4 Substrate	49.18%	CYP2C9 Substrate	0.46%
CYP2C19 Inhibitor	76.26%	CYP1A2 Inhibitor	13.22%		

Excretion

Half Life (hour)	3.41	Clearance (mL/min/g)	52.77

Tox

hERG	94.63%	DILI	27.87%	NR-AhR	32.08%
Carcinogencity	41.53%	LD₅₀	954.67	Respiratory Toxicity	37.42%
Eye Corrosion	0.23%				

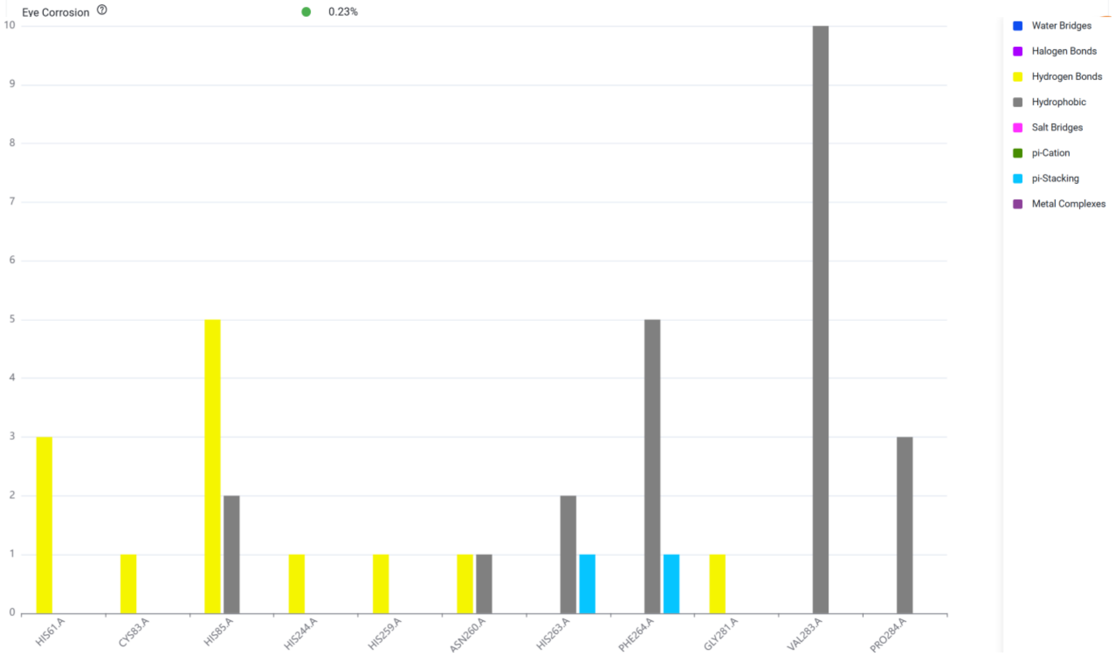

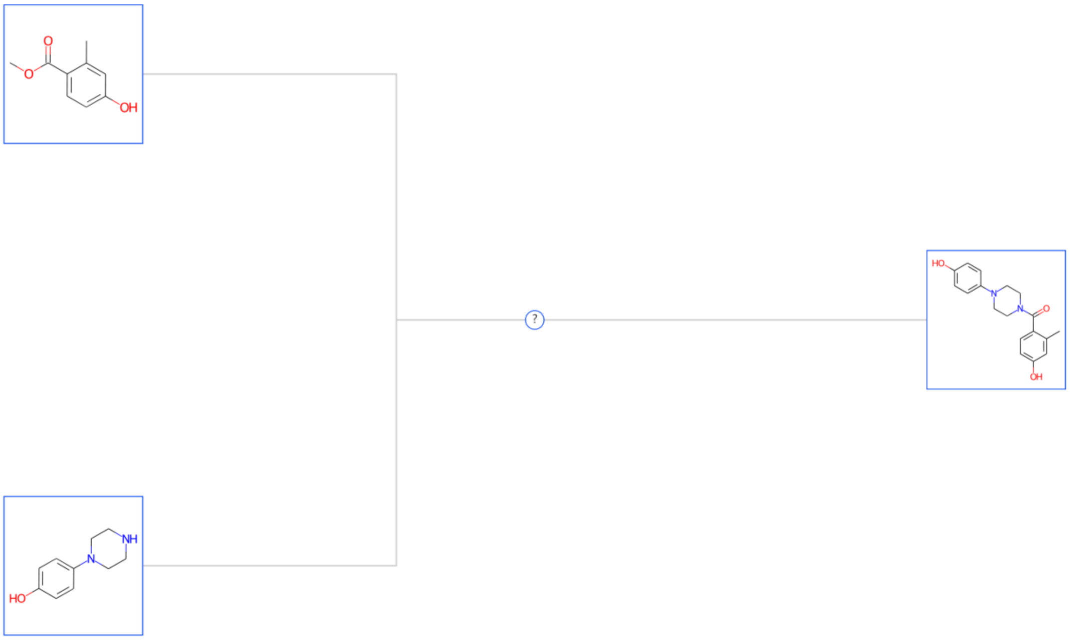

Alert Rule	Alert Structure	Reference
BMS Rule	✓	1. [1] Huth J R, Mendoza R, Olejniczak E T, et al. ALARM NMR: a rapid and robust experimental method to detect reactive false positives in biochemical screens[J]. Journal of the American Chemical Society, 2005, 127(1): 217-224.
Chelator Rule	✓	1. [1] Agrawal A, Johnson S L, Jacobsen J A, et al. Chelator fragment libraries for targeting metalloproteinases[J]. ChemMedChem: Chemistry Enabling Drug Discovery, 2010, 5(2): 195-199.
PAINS	✓	1. [1] Baell J B, Holloway G A. New substructure filters for removal of pan assay interference compounds (PAINS) from screening libraries and for their exclusion in bioassays[J]. Journal of medicinal chemistry, 2010, 53(7): 2719-2740.
Genotoxic Carcinogenicity Rule	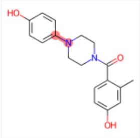	1. [1] Benigni R, Bossa C. Structure alerts for carcinogenicity, and the Salmonella assay system: a novel insight through the chemical relational databases technology[J]. Mutation Research/Reviews in Mutation Research, 2008, 659(3): 248-261.<br>2. [2] Ashby J, Tennant R W. Chemical structure, Salmonella mutagenicity and extent of carcinogenicity as indicators of genotoxic carcinogenesis among 222 chemicals tested in rodents by the US NCI/NTP[J]. Mutation Research/Genetic Toxicology, 1988, 204(1): 17-115.<br>3. [3] Kazius J, McGuire R, Bursi R. Derivation and validation of toxicophores for mutagenicity prediction[J]. Journal of medicinal chemistry, 2005, 48(1): 312-320.<br>4. [4] Bailey A B, Chanderbhan R, Collazo-Braier N, et al. The use of structure-activity relationship analysis in the food contact notification program[J]. Regulatory Toxicology and Pharmacology, 2005, 42(2): 225-235.
NTD	✓	1. [1] Brenk R, Schipani A, James D, et al. Lessons learnt from assembling screening libraries for drug discovery for neglected diseases[J]. ChemMedChem: Chemistry Enabling Drug Discovery, 2008, 3(3): 435-444.
SureChEMBL Rule	✓	1. [1] Sushko I, Salmina E, Potemkin V A, et al. ToxAlerts: a web server of structural alerts for toxic chemicals and compounds with potential adverse reactions[J]. 2012.

Compounds AI10-a8:

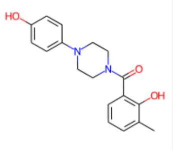

SMILES: Cc1cccc(C(=O)N2CCN(c3ccc(O)cc3)CC2)c1O

San ID	No data
CAS	No data
InChI	InChI=1S/C18H20N2O3/c1-13-3-2-4-16(17(13)22)18(23)20-11-9-19(10-12-20)14-5-7-15(21)8-6-14/h2-8,21-22H,9-12H2,1H3
InChIKey	PFZHGDHAYXAOSX-UHFFFAOYSA-N

View in third-party databases: PubChem

MW	312.15	HBA	4	HBD	2
PAINS Alert	false	QED	0.893	NRot	2
TPSA	64.01	logD	2.25	logP	2.3
logS	-3.39				

Bad Average Good Scoring Notice

Absorption

Caco2	-4.85	HIA	100.00%	P-gp Inhibitor	20.36%
Bioavailability	99.88%	HFE	-10.83		

Distribution

BBB Penetration	76.82%	PPBR	1.00%	VDSS	1.93

Metabolism

CYP2D6 Inhibitor	5.19%	CYP3A4 Inhibitor	61.98%	CYP2C9 Inhibitor	77.01%
CYP2D6 Substrate	91.43%	CYP3A4 Substrate	41.26%	CYP2C9 Substrate	0.57%
CYP2C19 Inhibitor	78.30%	CYP1A2 Inhibitor	7.69%		

Excretion

Half Life (hour)	3.15	Clearance (mL/min/g)	52.66

Tox

hERG	95.61%	DILI	40.53%	NR-AhR	34.13%
Carcinogencity	21.40%	LD_{50}	819.57	Respiratory Toxicity	25.46%
Eye Corrosion	0.38%				

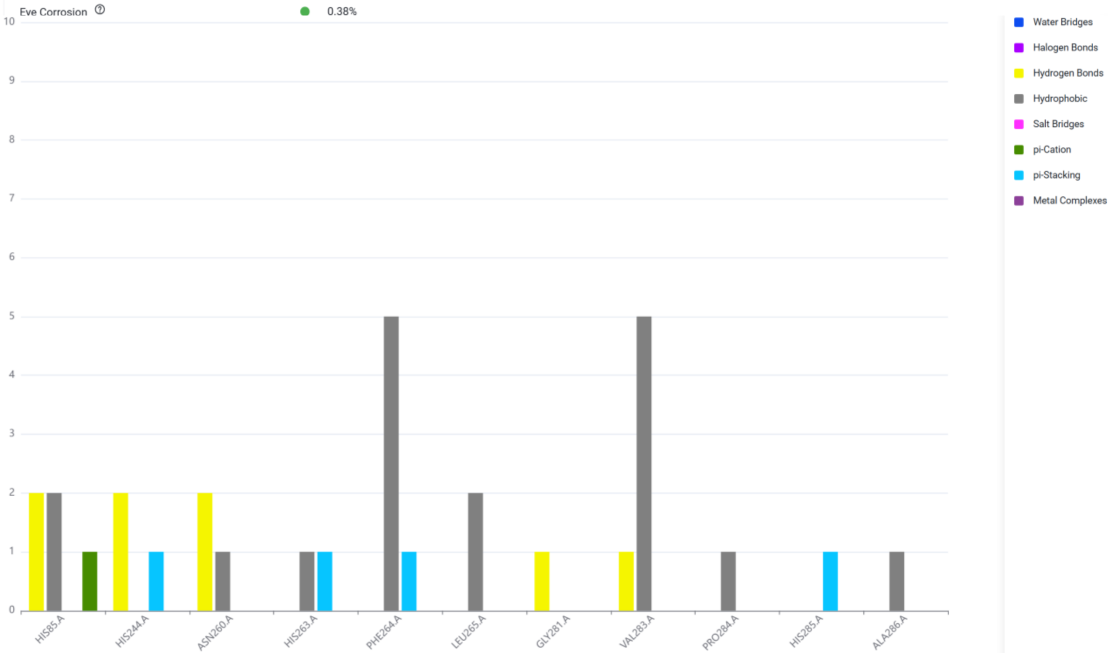

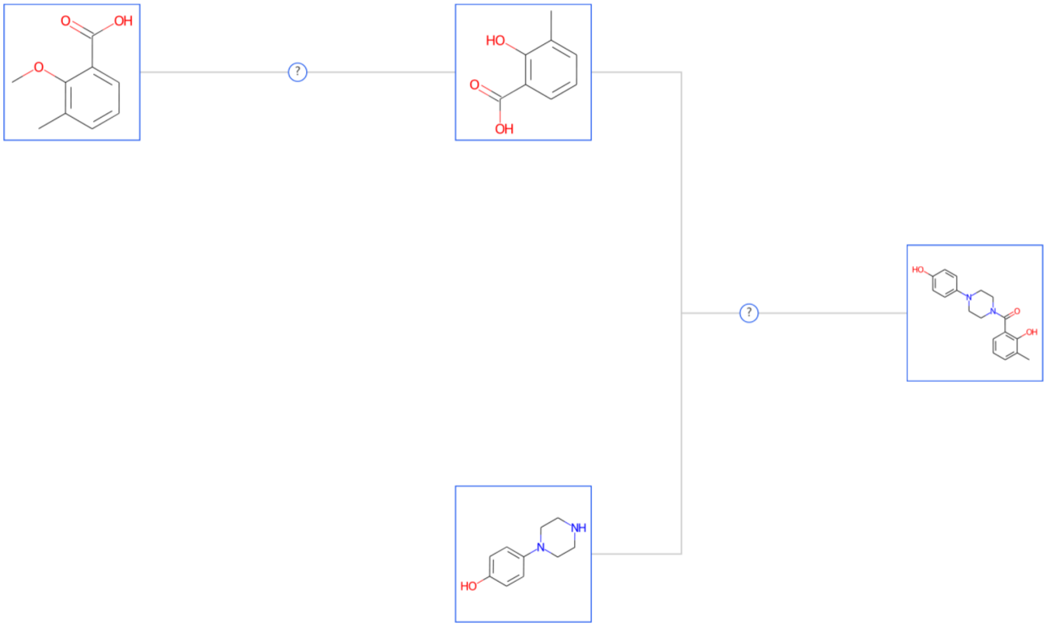

Alert Rule	Alert Structure	Reference
BMS Rule	✓	1. [1] Huth J R, Mendoza R, Olejniczak E T, et al. ALARM NMR: a rapid and robust experimental method to detect reactive false positives in biochemical screens[J]. Journal of the American Chemical Society, 2005, 127(1): 217-224.
Chelator Rule	✓	1. [1] Agrawal A, Johnson S L, Jacobsen J A, et al. Chelator fragment libraries for targeting metalloproteinases[J]. ChemMedChem: Chemistry Enabling Drug Discovery, 2010, 5(2): 195-199.
PAINS	✓	1. [1] Baell J B, Holloway G A. New substructure filters for removal of pan assay interference compounds (PAINS) from screening libraries and for their exclusion in bioassays[J]. Journal of medicinal chemistry, 2010, 53(7): 2719-2740.
Genotoxic Carcinogenicity Rule	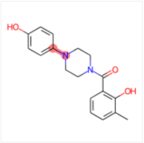	1. [1] Benigni R, Bossa C. Structure alerts for carcinogenicity, and the Salmonella assay system: a novel insight through the chemical relational databases technology[J]. Mutation Research/Reviews in Mutation Research, 2008, 659(3): 248-261.<br>2. [2] Ashby J, Tennant R W. Chemical structure, Salmonella mutagenicity and extent of carcinogenicity as indicators of genotoxic carcinogenesis among 222 chemicals tested in rodents by the US NCI/NTP[J]. Mutation Research/Genetic Toxicology, 1988, 204(1): 17-115.<br>3. [3] Kazius J, McGuire R, Bursi R. Derivation and validation of toxicophores for mutagenicity prediction[J]. Journal of medicinal chemistry, 2005, 48(1): 312-320.<br>4. [4] Bailey A B, Chanderbhan R, Collazo-Braier N, et al. The use of structure-activity relationship analysis in the food contact notification program[J]. Regulatory Toxicology and Pharmacology, 2005, 42(2): 225-235.
NTD	✓	1. [1] Brenk R, Schipani A, James D, et al. Lessons learnt from assembling screening libraries for drug discovery for neglected diseases[J]. ChemMedChem: Chemistry Enabling Drug Discovery, 2008, 3(3): 435-444.
SureChEMBL Rule	✓	1. [1] Sushko I, Salmina E, Potemkin V A, et al. ToxAlerts: a web server of structural alerts for toxic chemicals and compounds with potential adverse reactions[J]. 2012.

Compounds AI10-a9:

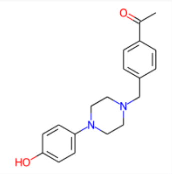

SMILES: CC(=O)c1ccc(CN2CCN(c3ccc(O)cc3)CC2)cc1

San ID	No data
CAS	No data
InChI	InChI=1S/C19H22N2O2/c1-15(22)17-4-2-16(3-5-17)14-20-10-12-21(13-11-20)18-6-8-19(23)9-7-18/h2-9,23H,10-14H2,1H3
InChIKey	TWUQYYOHFWCNRU-UHFFFAOYSA-N

View in third-party databases: No data

MW	310.17	HBA	4	HBD	1
PAINS Alert	false	QED	0.882	NRot	4
TPSA	43.78	logD	2.51	logP	2.56
logS	-2.48				

Bad Average Good Scoring Notice

Absorption

Caco2	-4.76	HIA	99.67%	P-gp Inhibitor	36.30%
Bioavailability	99.77%	HFE	-10.06		

Distribution

BBB Penetration	87.74%	PPBR	1.00%	VDSS	5.01

Metabolism

CYP2D6 Inhibitor	33.63%	CYP3A4 Inhibitor	24.24%	CYP2C9 Inhibitor	33.33%
CYP2D6 Substrate	28.59%	CYP3A4 Substrate	34.58%	CYP2C9 Substrate	0.10%
CYP2C19 Inhibitor	42.24%	CYP1A2 Inhibitor	16.62%		

Excretion

Half Life (hour)	4.12	Clearance (mL/min/g)	48.88

Tox

hERG	88.69%	DILI	20.03%	NR-AhR	15.41%
Carcinogencity	0.82%	LD_{50}	678.26	Respiratory Toxicity	96.64%
Eye Corrosion	1.25%				

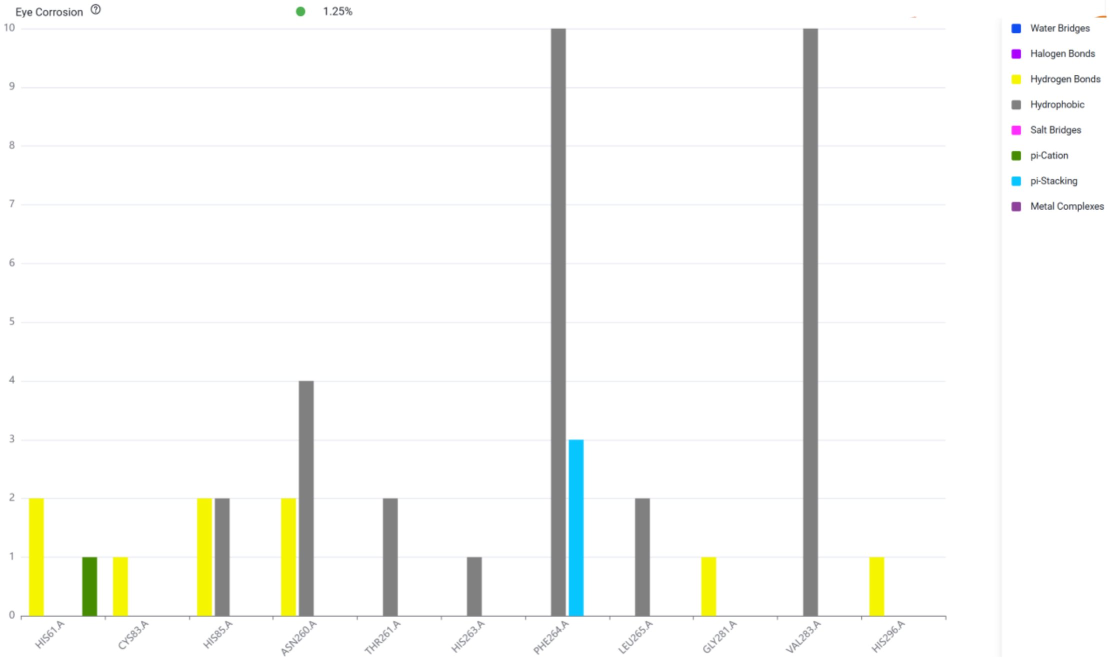

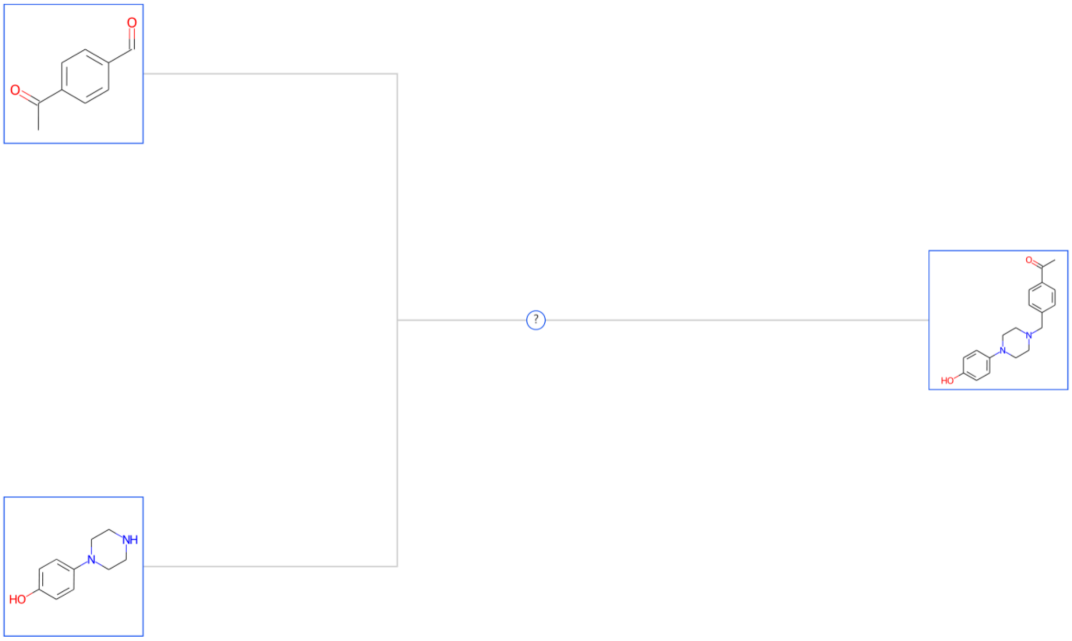

Alert Rule	Alert Structure	Reference
BMS Rule	✓	1. [1] Huth J R, Mendoza R, Olejniczak E T, et al. ALARM NMR: a rapid and robust experimental method to detect reactive false positives in biochemical screens[J]. Journal of the American Chemical Society, 2005, 127(1): 217-224.
Chelator Rule	✓	1. [1] Agrawal A, Johnson S L, Jacobsen J A, et al. Chelator fragment libraries for targeting metalloproteinases[J]. ChemMedChem: Chemistry Enabling Drug Discovery, 2010, 5(2): 195-199.
PAINS	✓	1. [1] Baell J B, Holloway G A. New substructure filters for removal of pan assay interference compounds (PAINS) from screening libraries and for their exclusion in bioassays[J]. Journal of medicinal chemistry, 2010, 53(7): 2719-2740.
Genotoxic Carcinogenicity Rule	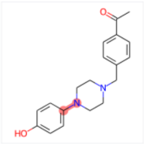	1. [1] Benigni R, Bossa C. Structure alerts for carcinogenicity, and the Salmonella assay system: a novel insight through the chemical relational databases technology[J]. Mutation Research/Reviews in Mutation Research, 2008, 659(3): 248-261.<br>2. [2] Ashby J, Tennant R W. Chemical structure, Salmonella mutagenicity and extent of carcinogenicity as indicators of genotoxic carcinogenesis among 222 chemicals tested in rodents by the US NCI/NTP[J]. Mutation Research/Genetic Toxicology, 1988, 204(1): 17-115.<br>3. [3] Kazius J, McGuire R, Bursi R. Derivation and validation of toxicophores for mutagenicity prediction[J]. Journal of medicinal chemistry, 2005, 48(1): 312-320.<br>4. [4] Bailey A B, Chanderbhan R, Collazo-Braier N, et al. The use of structure-activity relationship analysis in the food contact notification program[J]. Regulatory Toxicology and Pharmacology, 2005, 42(2): 225-235.
NTD	✓	1. [1] Brenk R, Schipani A, James D, et al. Lessons learnt from assembling screening libraries for drug discovery for neglected diseases[J]. ChemMedChem: Chemistry Enabling Drug Discovery, 2008, 3(3): 435-444.
SureChEMBL Rule	✓	1. [1] Sushko I, Salmina E, Potemkin V A, et al. ToxAlerts: a web server of structural alerts for toxic chemicals and compounds with potential adverse reactions[J]. 2012.

Compounds AI10-a10:

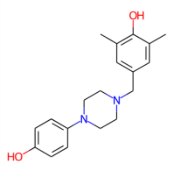

SMILES: Cc1cc(CN2CCN(c3ccc(O)cc3)CC2)cc(C)c1O

San ID	No data
CAS	No data
InChI	InChI=1S/C19H24N2O2/c1-14-11-16(12-15(2)19(14)23)13-20-7-9-21(10-8-20)17-3-5-18(22)6-4-17/h3-6,11-12,22-23H,7-10,13H2,1-2H3
InChIKey	GNEDXRYOZHZGSC-UHFFFAOYSA-N

View in third-party databases: No data

MW	312.18	HBA	4	HBD	2
PAINS Alert	false	QED	0.914	NRot	3
TPSA	46.94	logD	2.5	logP	2.32
logS	-2.64				

Bad Average Good Scoring Notice

Absorption

Caco2	-4.75	HIA	100.00%	P-gp Inhibitor	46.49%
Bioavailability	71.31%	HFE	-9.83		

Distribution

BBB Penetration	76.74%	PPBR	1.00%	VDSS	4.67

Metabolism

CYP2D6 Inhibitor	31.78%	CYP3A4 Inhibitor	42.29%	CYP2C9 Inhibitor	33.74%
CYP2D6 Substrate	32.58%	CYP3A4 Substrate	58.16%	CYP2C9 Substrate	2.36%
CYP2C19 Inhibitor	37.28%	CYP1A2 Inhibitor	39.21%		

Excretion

Half Life (hour)	3.63	Clearance (mL/min/g)	49.24

Tox

hERG	97.23%	DILI	12.56%	NR-AhR	33.88%
Carcinogencity	1.11%	LD_{50}	462.10	Respiratory Toxicity	99.97%
Eye Corrosion	33.40%				

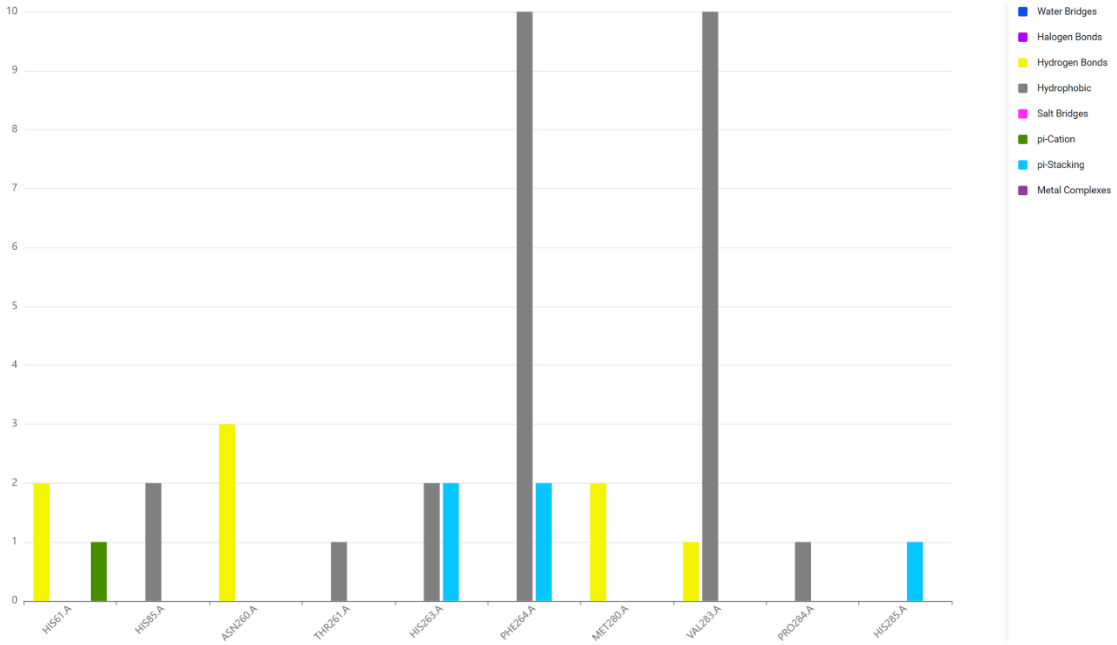

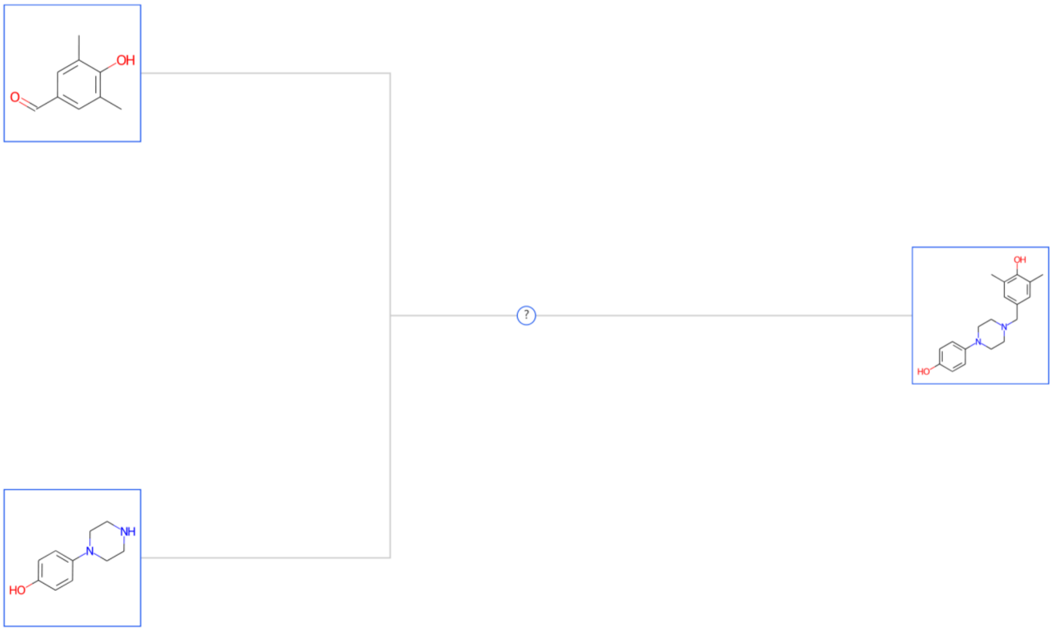

Alert Rule	Alert Structure	Reference
BMS Rule	✔	1. [1] Huth J R, Mendoza R, Olejniczak E T, et al. ALARM NMR: a rapid and robust experimental method to detect reactive false positives in biochemical screens[J]. Journal of the American Chemical Society, 2005, 127(1): 217-224.
Chelator Rule	✔	1. [1] Agrawal A, Johnson S L, Jacobsen J A, et al. Chelator fragment libraries for targeting metalloproteinases[J]. ChemMedChem: Chemistry Enabling Drug Discovery, 2010, 5(2): 195-199.
PAINS	✔	1. [1] Baell J B, Holloway G A. New substructure filters for removal of pan assay interference compounds (PAINS) from screening libraries and for their exclusion in bioassays[J]. Journal of medicinal chemistry, 2010, 53(7): 2719-2740.
Genotoxic Carcinogenicity Rule	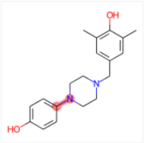	1. [1] Benigni R, Bossa C. Structure alerts for carcinogenicity, and the Salmonella assay system: a novel insight through the chemical relational databases technology[J]. Mutation Research/Reviews in Mutation Research, 2008, 659(3): 248-261.<br>2. [2] Ashby J, Tennant R W. Chemical structure, Salmonella mutagenicity and extent of carcinogenicity as indicators of genotoxic carcinogenesis among 222 chemicals tested in rodents by the US NCI/NTP[J]. Mutation Research/Genetic Toxicology, 1988, 204(1): 17-115.<br>3. [3] Kazius J, McGuire R, Bursi R. Derivation and validation of toxicophores for mutagenicity prediction[J]. Journal of medicinal chemistry, 2005, 48(1): 312-320.<br>4. [4] Bailey A B, Chanderbhan R, Collazo-Braier N, et al. The use of structure-activity relationship analysis in the food contact notification program[J]. Regulatory Toxicology and Pharmacology, 2005, 42(2): 225-235.
NTD	✔	1. [1] Brenk R, Schipani A, James D, et al. Lessons learnt from assembling screening libraries for drug discovery for neglected diseases[J]. ChemMedChem: Chemistry Enabling Drug Discovery, 2008, 3(3): 435-444.
SureChEMBL Rule	✔	1. [1] Sushko I, Salmina E, Potemkin V A, et al. ToxAlerts: a web server of structural alerts for toxic chemicals and compounds with potential adverse reactions[J]. 2012.

Compounds AI10-a11:

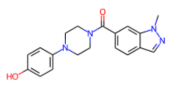

SMILES: Cn1ncc2ccc(C(=O)N3CCN(c4ccc(O)cc4)CC3)cc21

San ID	No data
CAS	No data
InChI	InChI=1S/C19H20N4O2/c1-21-18-12-14(2-3-15(18)13-20-21)19(25)23-10-8-22(9-11-23)16-4-6-17(24)7-5-16/h2-7,12-13,24H,8-11H2,1H3
InChIKey	DLDVGVPNGCXAQX-UHFFFAOYSA-N

View in third-party databases: No data

MW	336.16	HBA	5	HBD	1
PAINS Alert	false	QED	0.78	NRot	2
TPSA	61.6	logD	2.1	logP	1.85
logS	-3.73				

Bad Average Good Scoring Notice

Absorption

Caco2	-4.93	HIA	99.54%	P-gp Inhibitor	12.34%
Bioavailability	100.00%	HFE	-11.78		

Distribution

BBB Penetration	83.08%	PPBR	1.00%	VDSS	1.66

Metabolism

CYP2D6 Inhibitor	4.51%	CYP3A4 Inhibitor	69.97%	CYP2C9 Inhibitor	67.98%
CYP2D6 Substrate	43.25%	CYP3A4 Substrate	60.91%	CYP2C9 Substrate	99.73%
CYP2C19 Inhibitor	51.72%	CYP1A2 Inhibitor	9.91%		

Excretion

Half Life (hour)	4.18	Clearance (mL/min/g)	55.30

Tox

hERG	91.71%	DILI	57.79%	NR-AhR	49.16%
Carcinogencity	5.11%	LD_{50}	697.10	Respiratory Toxicity	46.47%
Eye Corrosion	0.01%				

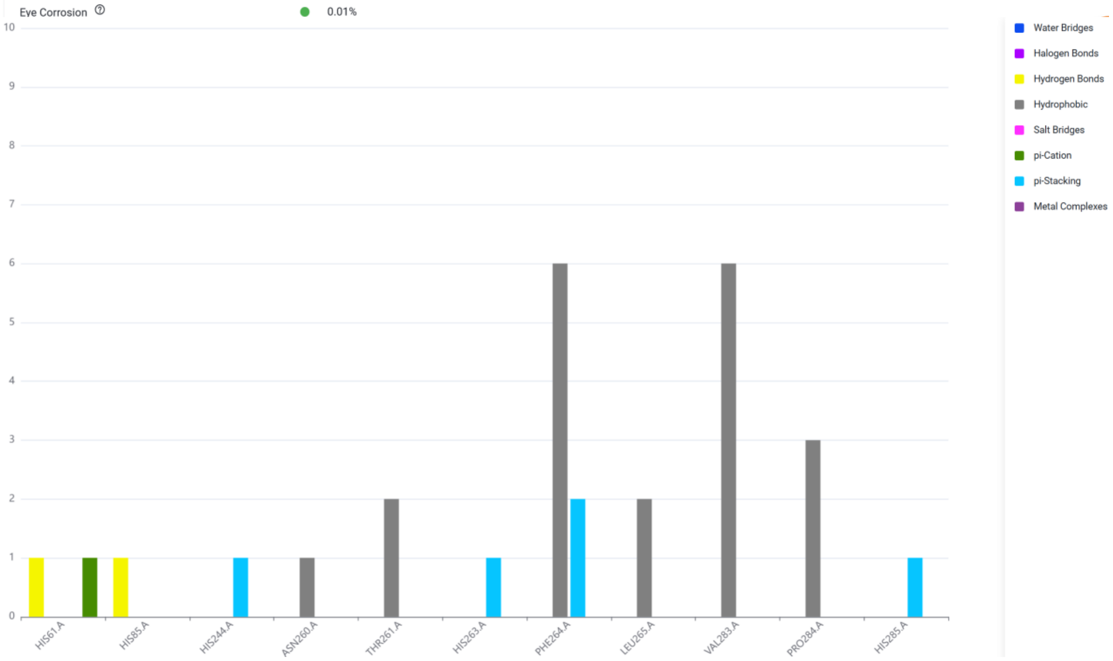

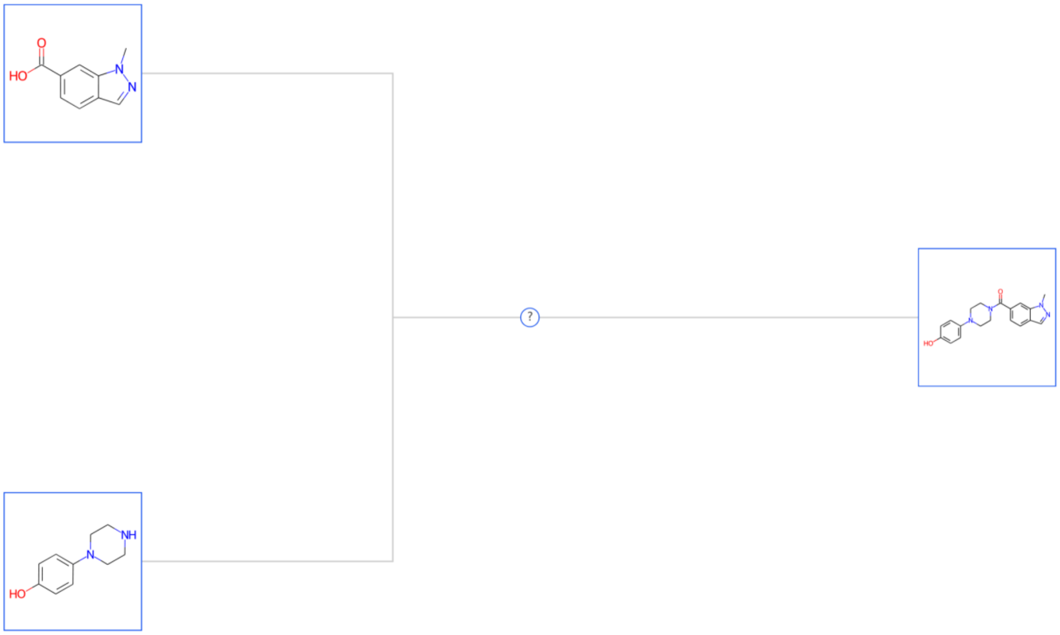

Alert Rule	Alert Structure	Reference
BMS Rule	✓	1. [1] Huth J R, Mendoza R, Olejniczak E T, et al. ALARM NMR: a rapid and robust experimental method to detect reactive false positives in biochemical screens[J]. Journal of the American Chemical Society, 2005, 127(1): 217-224.
Chelator Rule	✓	1. [1] Agrawal A, Johnson S L, Jacobsen J A, et al. Chelator fragment libraries for targeting metalloproteinases[J]. ChemMedChem: Chemistry Enabling Drug Discovery, 2010, 5(2): 195-199.
PAINS	✓	1. [1] Baell J B, Holloway G A. New substructure filters for removal of pan assay interference compounds (PAINS) from screening libraries and for their exclusion in bioassays[J]. Journal of medicinal chemistry, 2010, 53(7): 2719-2740.
Genotoxic Carcinogenicity Rule	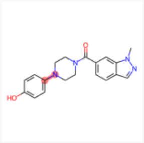	1. [1] Benigni R, Bossa C. Structure alerts for carcinogenicity, and the Salmonella assay system: a novel insight through the chemical relational databases technology[J]. Mutation Research/Reviews in Mutation Research, 2008, 659(3): 248-261.<br>2. [2] Ashby J, Tennant R W. Chemical structure, Salmonella mutagenicity and extent of carcinogenicity as indicators of genotoxic carcinogenesis among 222 chemicals tested in rodents by the US NCI/NTP[J]. Mutation Research/Genetic Toxicology, 1988, 204(1): 17-115.<br>3. [3] Kazius J, McGuire R, Bursi R. Derivation and validation of toxicophores for mutagenicity prediction[J]. Journal of medicinal chemistry, 2005, 48(1): 312-320.<br>4. [4] Bailey A B, Chanderbhan R, Collazo-Braier N, et al. The use of structure-activity relationship analysis in the food contact notification program[J]. Regulatory Toxicology and Pharmacology, 2005, 42(2): 225-235.
NTD	✓	1. [1] Brenk R, Schipani A, James D, et al. Lessons learnt from assembling screening libraries for drug discovery for neglected diseases[J]. ChemMedChem: Chemistry Enabling Drug Discovery, 2008, 3(3): 435-444.
SureChEMBL Rule	✓	1. [1] Sushko I, Salmina E, Potemkin V A, et al. ToxAlerts: a web server of structural alerts for toxic chemicals and compounds with potential adverse reactions[J]. 2012.

Compounds AI10-a12:

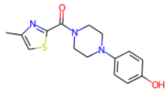

SMILES: Cc1csc(C(=O)N2CCN(c3ccc(O)cc3)CC2)n1

San ID	No data
CAS	No data
InChI	InChI=1S/C15H17N3O2S/c1-11-10-21-14(16-11)15(20)18-8-6-17(7-9-18)12-2-4-13(19)5-3-12/h2-5,10,19H,6-9H2,1H3
InChIKey	VUIJGYQDGJEQBB-UHFFFAOYSA-N

View in third-party databases: No data

MW	303.1	HBA	5	HBD	1
PAINS Alert	false	QED	0.923	NRot	2
TPSA	56.67	logD	1.35	logP	1.84
logS	-2.82				

Bad Average Good Scoring Notice

Absorption

Caco2	-4.74	HIA	100.00%	P-gp Inhibitor	18.92%
Bioavailability	99.13%	HFE	-9.63		

Distribution

BBB Penetration	80.38%	PPBR	1.00%	VDSS	2.13

Metabolism

CYP2D6 Inhibitor	4.16%	CYP3A4 Inhibitor	56.27%	CYP2C9 Inhibitor	72.77%
CYP2D6 Substrate	95.91%	CYP3A4 Substrate	33.05%	CYP2C9 Substrate	99.94%
CYP2C19 Inhibitor	76.83%	CYP1A2 Inhibitor	19.08%		

Excretion

Half Life (hour)	2.98	Clearance (mL/min/g)	48.54

Tox

hERG	86.28%	DILI	68.78%	NR-AhR	33.56%
Carcinogencity	14.57%	LD_{50}	1064.32	Respiratory Toxicity	60.47%
Eye Corrosion	0.17%				

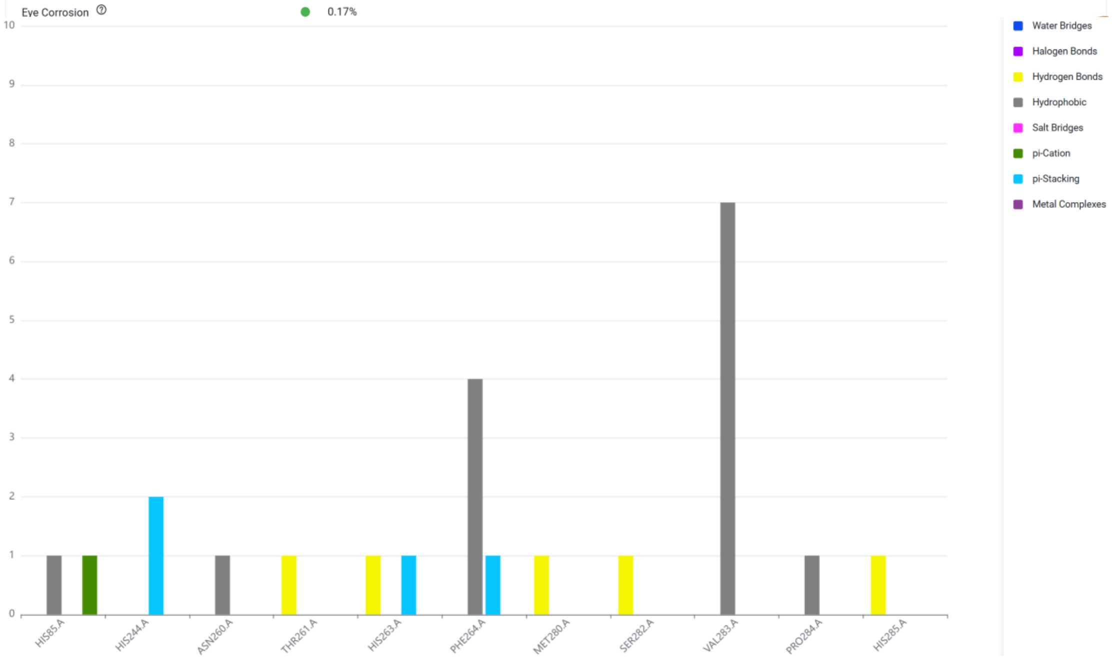

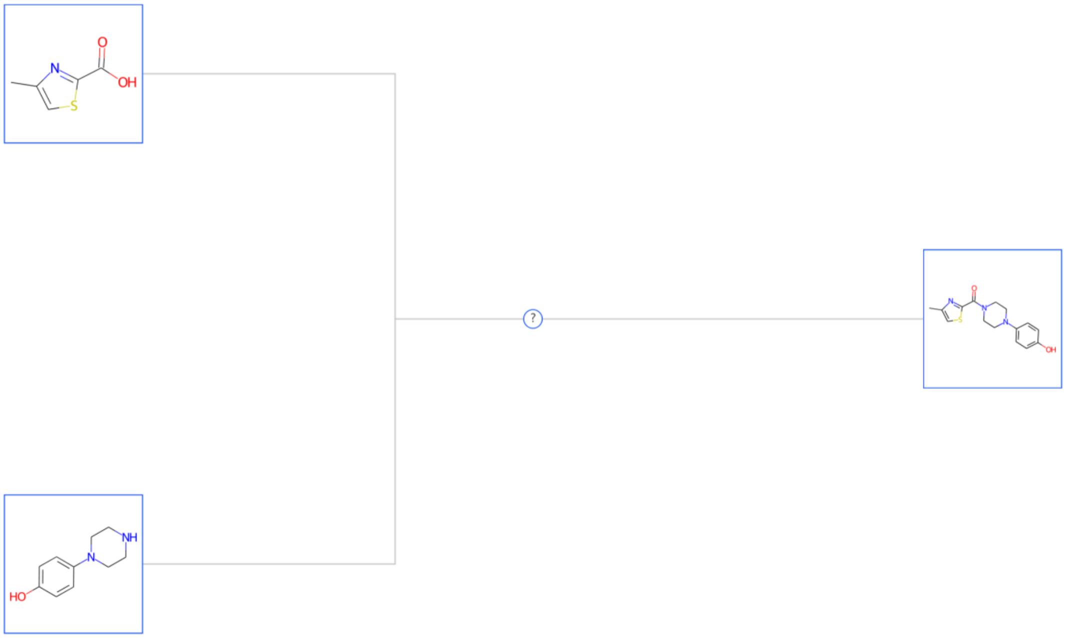

Alert Rule	Alert Structure	Reference
BMS Rule	✔	1. [1] Huth J R, Mendoza R, Olejniczak E T, et al. ALARM NMR: a rapid and robust experimental method to detect reactive false positives in biochemical screens[J]. Journal of the American Chemical Society, 2005, 127(1): 217-224.
Chelator Rule	✔	1. [1] Agrawal A, Johnson S L, Jacobsen J A, et al. Chelator fragment libraries for targeting metalloproteinases[J]. ChemMedChem: Chemistry Enabling Drug Discovery, 2010, 5(2): 195-199.
PAINS	✔	1. [1] Baell J B, Holloway G A. New substructure filters for removal of pan assay interference compounds (PAINS) from screening libraries and for their exclusion in bioassays[J]. Journal of medicinal chemistry, 2010, 53(7): 2719-2740.
Genotoxic Carcinogenicity Rule	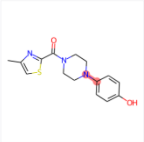	1. [1] Benigni R, Bossa C. Structure alerts for carcinogenicity, and the Salmonella assay system: a novel insight through the chemical relational databases technology[J]. Mutation Research/Reviews in Mutation Research, 2008, 659(3): 248-261.<br>2. [2] Ashby J, Tennant R W. Chemical structure, Salmonella mutagenicity and extent of carcinogenicity as indicators of genotoxic carcinogenesis among 222 chemicals tested in rodents by the US NCI/NTP[J]. Mutation Research/Genetic Toxicology, 1988, 204(1): 17-115.<br>3. [3] Kazius J, McGuire R, Bursi R. Derivation and validation of toxicophores for mutagenicity prediction[J]. Journal of medicinal chemistry, 2005, 48(1): 312-320.<br>4. [4] Bailey A B, Chanderbhan R, Collazo-Braier N, et al. The use of structure-activity relationship analysis in the food contact notification program[J]. Regulatory Toxicology and Pharmacology, 2005, 42(2): 225-235.
NTD	✔	1. [1] Brenk R, Schipani A, James D, et al. Lessons learnt from assembling screening libraries for drug discovery for neglected diseases[J]. ChemMedChem: Chemistry Enabling Drug Discovery, 2008, 3(3): 435-444.
SureChEMBL Rule	✔	1. [1] Sushko I, Salmina E, Potemkin V A, et al. ToxAlerts: a web server of structural alerts for toxic chemicals and compounds with potential adverse reactions[J]. 2012.

Compounds AI10-a13:

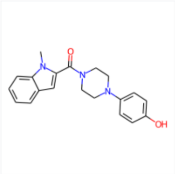

SMILES: Cn1c(C(=O)N2CCN(c3ccc(O)cc3)CC2)cc2ccccc21

San ID	No data
CAS	No data
InChI	InChI=1S/C20H21N3O2/c1-21-18-5-3-2-4-15(18)14-19(21)20(25)23-12-10-22(11-13-23)16-6-8-17(24)9-7-16/h2-9,14,24H,10-13H2,1H3
InChIKey	HJBNDUIONMEUCG-UHFFFAOYSA-N

View in third-party databases: No data

MW	335.16	HBA	4	HBD	1
PAINS Alert	false	QED	0.783	NRot	2
TPSA	48.71	logD	3.06	logP	2.98
logS	-3.81				

Bad Average Good Scoring Notice

Absorption

Caco2	-4.85	HIA	100.00%	P-gp Inhibitor	69.89%
Bioavailability	99.99%	HFE	-10.74		

Distribution

BBB Penetration	71.48%	PPBR	1.00%	VDSS	0.79

Metabolism

CYP2D6 Inhibitor	6.39%	CYP3A4 Inhibitor	73.85%	CYP2C9 Inhibitor	74.91%
CYP2D6 Substrate	93.05%	CYP3A4 Substrate	67.76%	CYP2C9 Substrate	99.99%
CYP2C19 Inhibitor	78.51%	CYP1A2 Inhibitor	50.27%		

Excretion

Half Life (hour)	4.61	Clearance (mL/min/g)	53.00

Tox

hERG	96.30%	DILI	68.32%	NR-AhR	48.28%
Carcinogencity	7.15%	LD_{50}	901.60	Respiratory Toxicity	97.80%
Eye Corrosion	0.04%				

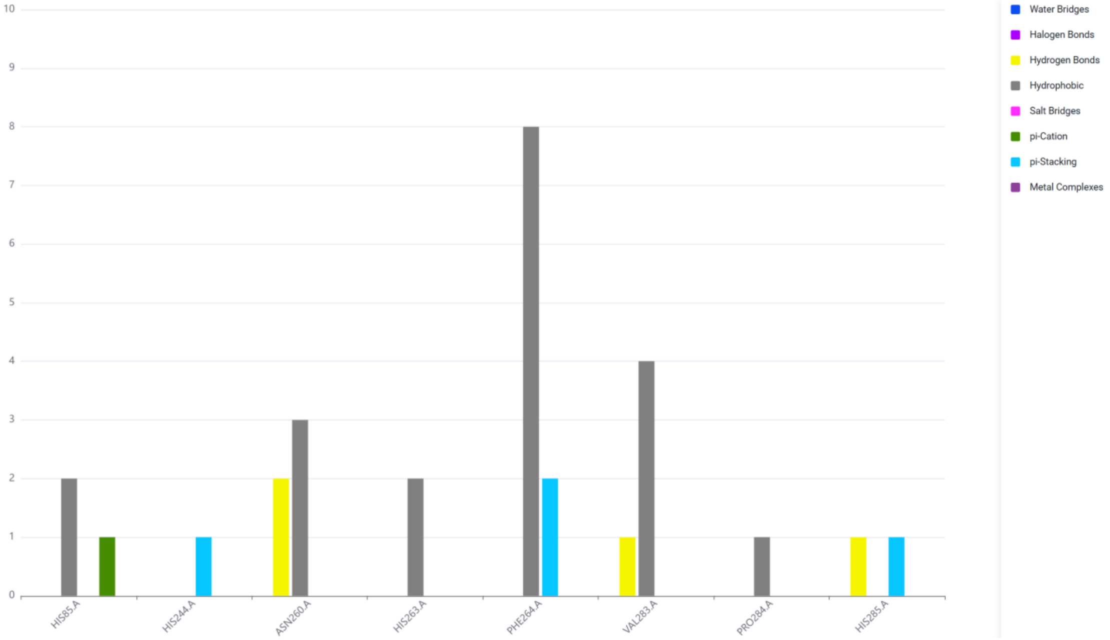

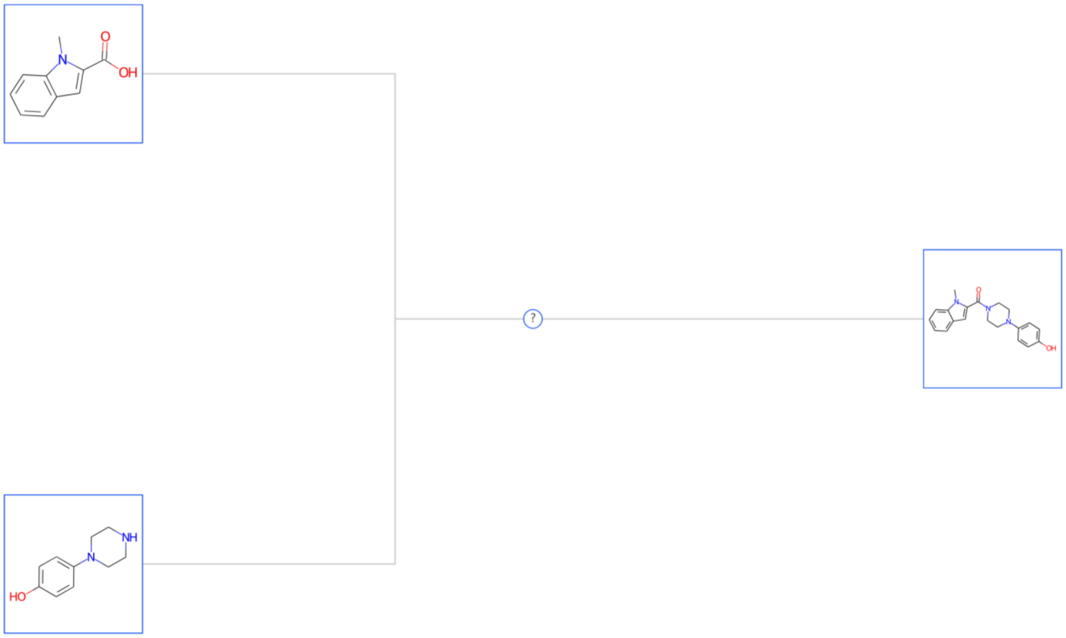

Alert Rule	Alert Structure	Reference
BMS Rule	✓	1. [1] Huth J R, Mendoza R, Olejniczak E T, et al. ALARM NMR: a rapid and robust experimental method to detect reactive false positives in biochemical screens[J]. Journal of the American Chemical Society, 2005, 127(1): 217-224.
Chelator Rule	✓	1. [1] Agrawal A, Johnson S L, Jacobsen J A, et al. Chelator fragment libraries for targeting metalloproteinases[J]. ChemMedChem: Chemistry Enabling Drug Discovery, 2010, 5(2): 195-199.
PAINS	✓	1. [1] Baell J B, Holloway G A. New substructure filters for removal of pan assay interference compounds (PAINS) from screening libraries and for their exclusion in bioassays[J]. Journal of medicinal chemistry, 2010, 53(7): 2719-2740.
Genotoxic Carcinogenicity Rule	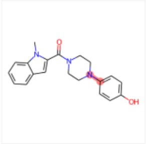	1. [1] Benigni R, Bossa C. Structure alerts for carcinogenicity, and the Salmonella assay system: a novel insight through the chemical relational databases technology[J]. Mutation Research/Reviews in Mutation Research, 2008, 659(3): 248-261.<br>2. [2] Ashby J, Tennant R W. Chemical structure, Salmonella mutagenicity and extent of carcinogenicity as indicators of genotoxic carcinogenesis among 222 chemicals tested in rodents by the US NCI/NTP[J]. Mutation Research/Genetic Toxicology, 1988, 204(1): 17-115.<br>3. [3] Kazius J, McGuire R, Bursi R. Derivation and validation of toxicophores for mutagenicity prediction[J]. Journal of medicinal chemistry, 2005, 48(1): 312-320.<br>4. [4] Bailey A B, Chanderbhan R, Collazo-Braier N, et al. The use of structure-activity relationship analysis in the food contact notification program[J]. Regulatory Toxicology and Pharmacology, 2005, 42(2): 225-235.
NTD	✓	1. [1] Brenk R, Schipani A, James D, et al. Lessons learnt from assembling screening libraries for drug discovery for neglected diseases[J]. ChemMedChem: Chemistry Enabling Drug Discovery, 2008, 3(3): 435-444.
SureChEMBL Rule	✓	1. [1] Sushko I, Salmina E, Potemkin V A, et al. ToxAlerts: a web server of structural alerts for toxic chemicals and compounds with potential adverse reactions[J]. 2012.

Compounds AI10-a14:

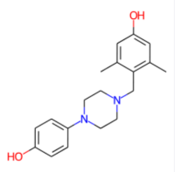

SMILES: Cc1cc(O)cc(C)c1CN1CCN(c2ccc(O)cc2)CC1

San ID	No data
CAS	No data
InChI	InChI=1S/C19H24N2O2/c1-14-11-18(23)12-15(2)19(14)13-20-7-9-21(10-8-20)16-3-5-17(22)6-4-16/h3-6,11-12,22-23H,7-10,13H2,1-2H3
InChIKey	ONSCDACKBGNJQW-UHFFFAOYSA-N

View in third-party databases: No data

MW	312.18	HBA	4	HBD	2
PAINS Alert	false	QED	0.914	NRot	3
TPSA	46.94	logD	2.34	logP	2.54
logS	-2.54				

Bad Average Good Scoring Notice

Absorption

Caco2	-4.71	HIA	100.00%	P-gp Inhibitor	44.58%
Bioavailability	68.61%	HFE	-10.80		

Distribution

BBB Penetration	70.38%	PPBR	1.00%	VDSS	4.48

Metabolism

CYP2D6 Inhibitor	33.60%	CYP3A4 Inhibitor	42.70%	CYP2C9 Inhibitor	26.75%
CYP2D6 Substrate	48.66%	CYP3A4 Substrate	65.30%	CYP2C9 Substrate	0.65%
CYP2C19 Inhibitor	31.07%	CYP1A2 Inhibitor	24.03%		

Excretion

Half Life (hour)	3.44	Clearance (mL/min/g)	46.98

Tox

hERG	97.21%	DILI	15.91%	NR-AhR	35.25%
Carcinogencity	2.15%	LD_{50}	745.98	Respiratory Toxicity	99.97%
Eye Corrosion	21.23%				

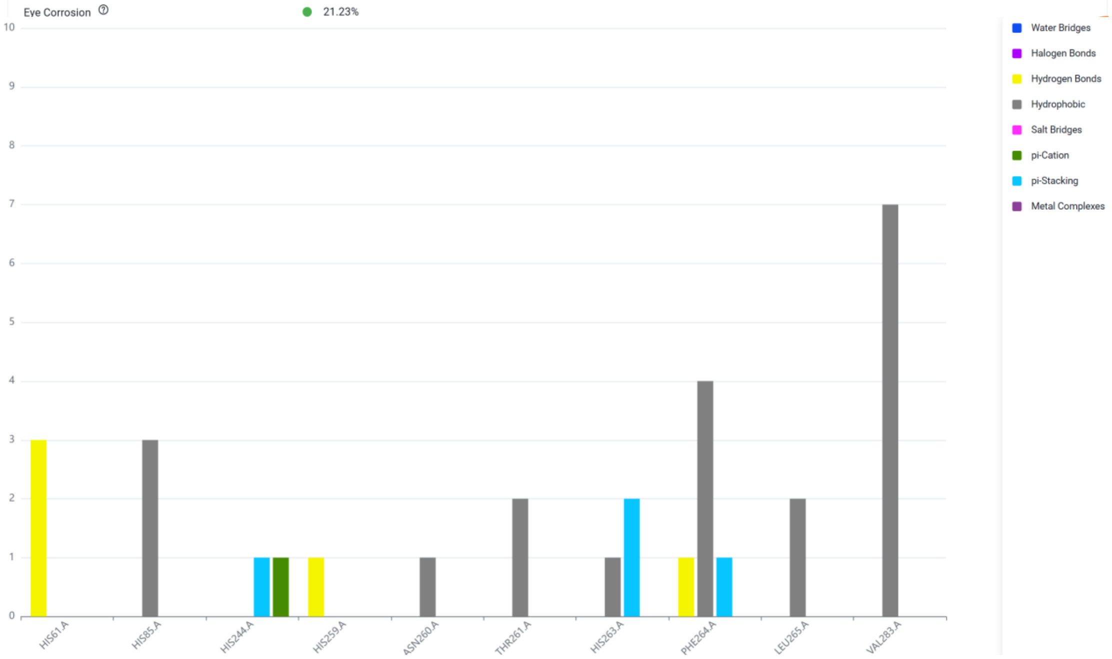

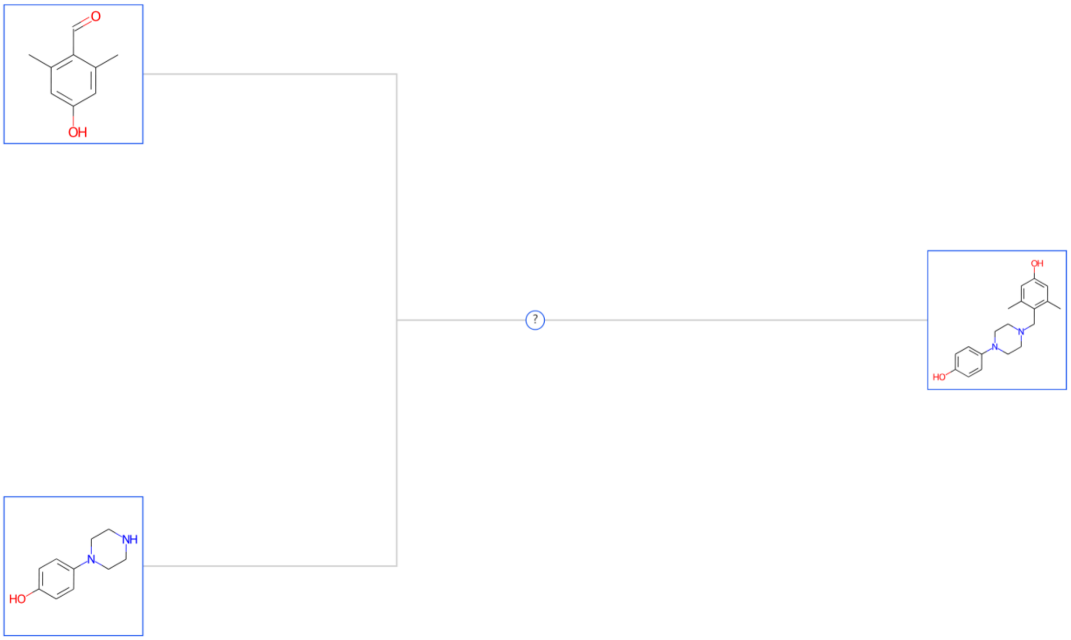

Alert Rule	Alert Structure	Reference
BMS Rule	✓	1. [1] Huth J R, Mendoza R, Olejniczak E T, et al. ALARM NMR: a rapid and robust experimental method to detect reactive false positives in biochemical screens[J]. Journal of the American Chemical Society, 2005, 127(1): 217-224.
Chelator Rule	✓	1. [1] Agrawal A, Johnson S L, Jacobsen J A, et al. Chelator fragment libraries for targeting metalloproteinases[J]. ChemMedChem: Chemistry Enabling Drug Discovery, 2010, 5(2): 195-199.
PAINS	✓	1. [1] Baell J B, Holloway G A. New substructure filters for removal of pan assay interference compounds (PAINS) from screening libraries and for their exclusion in bioassays[J]. Journal of medicinal chemistry, 2010, 53(7): 2719-2740.
Genotoxic Carcinogenicity Rule	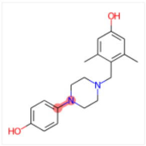	1. [1] Benigni R, Bossa C. Structure alerts for carcinogenicity, and the Salmonella assay system: a novel insight through the chemical relational databases technology[J]. Mutation Research/Reviews in Mutation Research, 2008, 659(3): 248-261.<br>2. [2] Ashby J, Tennant R W. Chemical structure, Salmonella mutagenicity and extent of carcinogenicity as indicators of genotoxic carcinogenesis among 222 chemicals tested in rodents by the US NCI/NTP[J]. Mutation Research/Genetic Toxicology, 1988, 204(1): 17-115.<br>3. [3] Kazius J, McGuire R, Bursi R. Derivation and validation of toxicophores for mutagenicity prediction[J]. Journal of medicinal chemistry, 2005, 48(1): 312-320.<br>4. [4] Bailey A B, Chanderbhan R, Collazo-Braier N, et al. The use of structure-activity relationship analysis in the food contact notification program[J]. Regulatory Toxicology and Pharmacology, 2005, 42(2): 225-235.
NTD	✓	1. [1] Brenk R, Schipani A, James D, et al. Lessons learnt from assembling screening libraries for drug discovery for neglected diseases[J]. ChemMedChem: Chemistry Enabling Drug Discovery, 2008, 3(3): 435-444.
SureChEMBL Rule	✓	1. [1] Sushko I, Salmina E, Potemkin V A, et al. ToxAlerts: a web server of structural alerts for toxic chemicals and compounds with potential adverse reactions[J]. 2012.

Compounds AI10-a15:

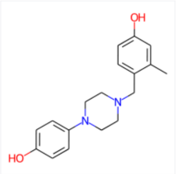

SMILES: Cc1cc(O)ccc1CN1CCN(c2ccc(O)cc2)CC1

San ID	No data
CAS	No data
InChI	InChI=1S/C18H22N2O2/c1-14-12-18(22)5-2-15(14)13-19-8-10-20(11-9-19)16-3-6-17(21)7-4-16/h2-7,12,21-22H,8-11,13H2,1H3
InChIKey	YKHFATYRDLGFTM-UHFFFAOYSA-N

View in third-party databases: Loading...

MW	298.17	HBA	4	HBD	2
PAINS Alert	false	QED	0.914	NRot	3
TPSA	46.94	logD	2.27	logP	2.37
logS	-2.18				

Bad Average Good Scoring Notice

Absorption

Caco2	-4.79	HIA	100.00%	P-gp Inhibitor	43.55%
Bioavailability	79.62%	HFE	-10.58		

Distribution

BBB Penetration	77.45%	PPBR	1.00%	VDSS	3.93

Metabolism

CYP2D6 Inhibitor	35.85%	CYP3A4 Inhibitor	37.05%	CYP2C9 Inhibitor	27.69%
CYP2D6 Substrate	47.98%	CYP3A4 Substrate	43.61%	CYP2C9 Substrate	1.03%
CYP2C19 Inhibitor	32.37%	CYP1A2 Inhibitor	21.06%		

Excretion

Half Life (hour)	3.89	Clearance (mL/min/g)	46.24

Tox

hERG	96.50%	DILI	12.00%	NR-AhR	33.53%
Carcinogencity	4.29%	LD_{50}	448.20	Respiratory Toxicity	99.91%
Eye Corrosion	14.68%				

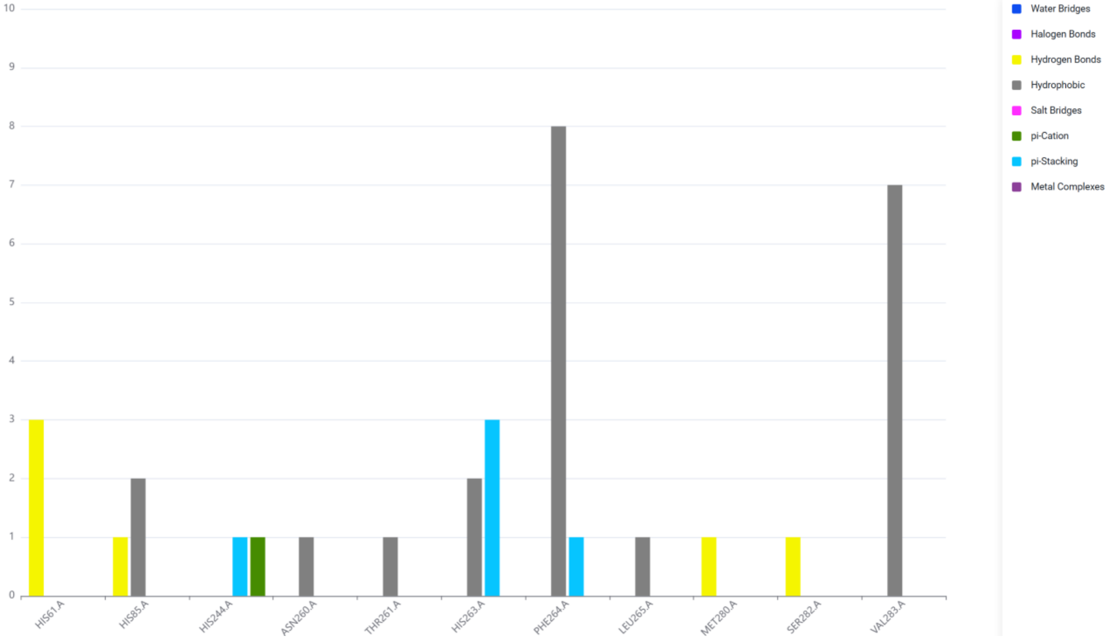

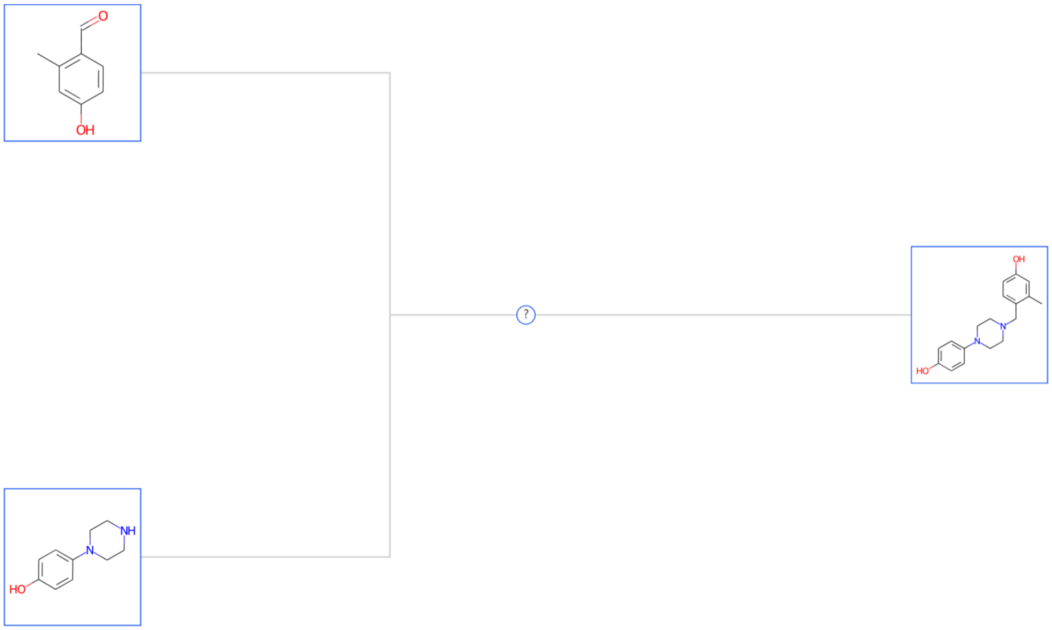

Alert Rule	Alert Structure	Reference
BMS Rule	✔	1. [1] Huth J R, Mendoza R, Olejniczak E T, et al. ALARM NMR: a rapid and robust experimental method to detect reactive false positives in biochemical screens[J]. Journal of the American Chemical Society, 2005, 127(1): 217-224.
Chelator Rule	✔	1. [1] Agrawal A, Johnson S L, Jacobsen J A, et al. Chelator fragment libraries for targeting metalloproteinases[J]. ChemMedChem: Chemistry Enabling Drug Discovery, 2010, 5(2): 195-199.
PAINS	✔	1. [1] Baell J B, Holloway G A. New substructure filters for removal of pan assay interference compounds (PAINS) from screening libraries and for their exclusion in bioassays[J]. Journal of medicinal chemistry, 2010, 53(7): 2719-2740.
Genotoxic Carcinogenicity Rule	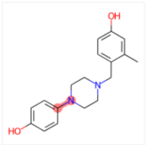	1. [1] Benigni R, Bossa C. Structure alerts for carcinogenicity, and the Salmonella assay system: a novel insight through the chemical relational databases technology[J]. Mutation Research/Reviews in Mutation Research, 2008, 659(3): 248-261.<br>2. [2] Ashby J, Tennant R W. Chemical structure, Salmonella mutagenicity and extent of carcinogenicity as indicators of genotoxic carcinogenesis among 222 chemicals tested in rodents by the US NCI/NTP[J]. Mutation Research/Genetic Toxicology, 1988, 204(1): 17-115.<br>3. [3] Kazius J, McGuire R, Bursi R. Derivation and validation of toxicophores for mutagenicity prediction[J]. Journal of medicinal chemistry, 2005, 48(1): 312-320.<br>4. [4] Bailey A B, Chanderbhan R, Collazo-Braier N, et al. The use of structure-activity relationship analysis in the food contact notification program[J]. Regulatory Toxicology and Pharmacology, 2005, 42(2): 225-235.
NTD	✔	1. [1] Brenk R, Schipani A, James D, et al. Lessons learnt from assembling screening libraries for drug discovery for neglected diseases[J]. ChemMedChem: Chemistry Enabling Drug Discovery, 2008, 3(3): 435-444.
SureChEMBL Rule	✔	1. [1] Sushko I, Salmina E, Potemkin V A, et al. ToxAlerts: a web server of structural alerts for toxic chemicals and compounds with potential adverse reactions[J]. 2012.

Compounds AI10-a16:

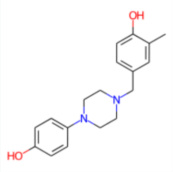

SMILES: Cc1cc(CN2CCN(c3ccc(O)cc3)CC2)ccc1O

San ID	No data
CAS	No data
InChI	InChI=1S/C18H22N2O2/c1-14-12-15(2-7-18(14)22)13-19-8-10-20(11-9-19)16-3-5-17(21)6-4-16/h2-7,12,21-22H,8-11,13H2,1H3
InChIKey	RLVOAHSWFINCBR-UHFFFAOYSA-N

View in third-party databases: No data

MW	298.17	HBA	4	HBD	2
PAINS Alert	false	QED	0.914	NRot	3
TPSA	46.94	logD	2.39	logP	2.28
logS	-2.35				

Bad Average Good Scoring Notice

Absorption

Caco2	-4.89	HIA	100.00%	P-gp Inhibitor	43.58%
Bioavailability	85.39%	HFE	-10.24		

Distribution

BBB Penetration	82.14%	PPBR	1.00%	VDSS	3.61

Metabolism

CYP2D6 Inhibitor	34.64%	CYP3A4 Inhibitor	30.96%	CYP2C9 Inhibitor	35.52%
CYP2D6 Substrate	12.74%	CYP3A4 Substrate	26.11%	CYP2C9 Substrate	3.52%
CYP2C19 Inhibitor	36.66%	CYP1A2 Inhibitor	26.27%		

Excretion

Half Life (hour)	3.45	Clearance (mL/min/g)	48.52

Tox

hERG	96.60%	DILI	9.64%	NR-AhR	27.83%
Carcinogencity	3.81%	LD₅₀	672.73	Respiratory Toxicity	99.91%
Eye Corrosion	14.75%				

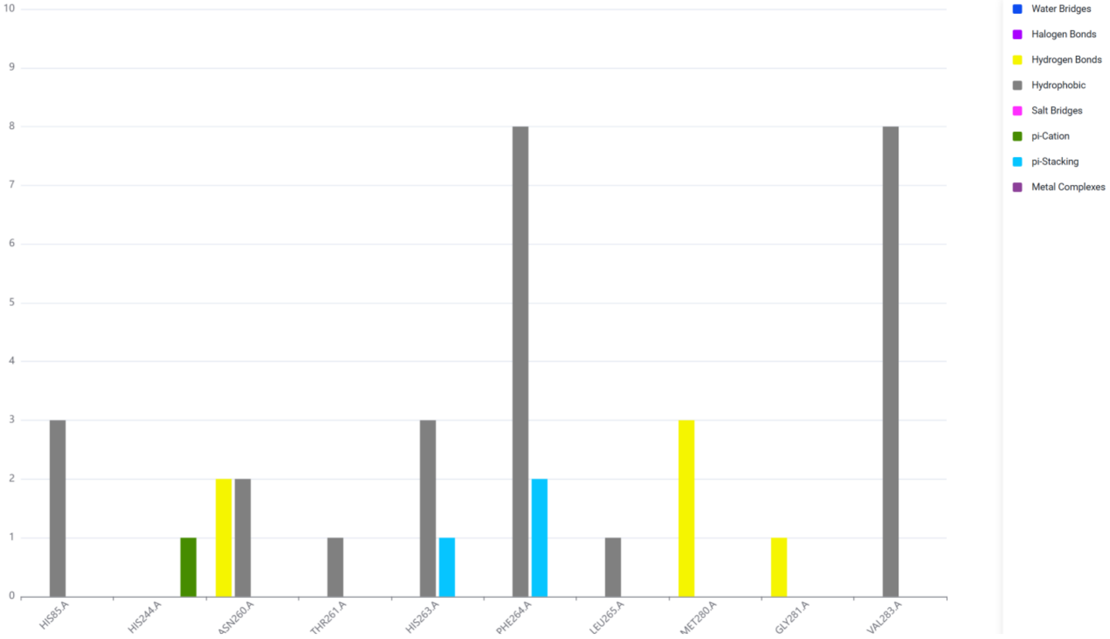

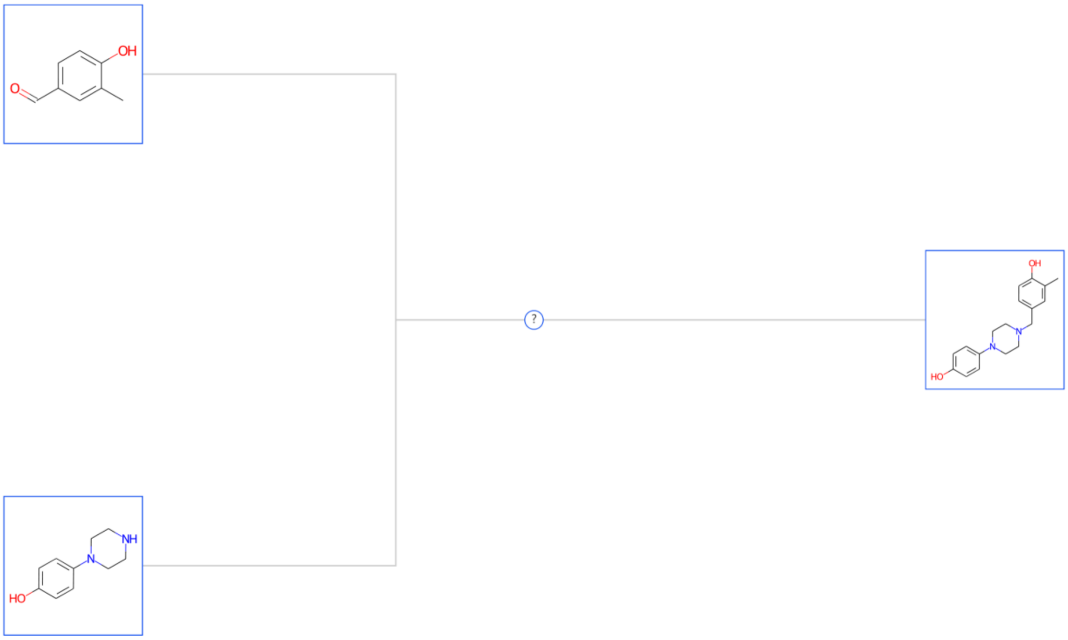

Alert Rule	Alert Structure	Reference
BMS Rule	✔	1. [1] Huth J R, Mendoza R, Olejniczak E T, et al. ALARM NMR: a rapid and robust experimental method to detect reactive false positives in biochemical screens[J]. Journal of the American Chemical Society, 2005, 127(1): 217-224.
Chelator Rule	✔	1. [1] Agrawal A, Johnson S L, Jacobsen J A, et al. Chelator fragment libraries for targeting metalloproteinases[J]. ChemMedChem: Chemistry Enabling Drug Discovery, 2010, 5(2): 195-199.
PAINS	✔	1. [1] Baell J B, Holloway G A. New substructure filters for removal of pan assay interference compounds (PAINS) from screening libraries and for their exclusion in bioassays[J]. Journal of medicinal chemistry, 2010, 53(7): 2719-2740.
Genotoxic Carcinogenicity Rule	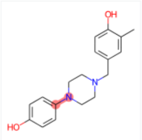	1. [1] Benigni R, Bossa C. Structure alerts for carcinogenicity, and the Salmonella assay system: a novel insight through the chemical relational databases technology[J]. Mutation Research/Reviews in Mutation Research, 2008, 659(3): 248-261. 2. [2] Ashby J, Tennant R W. Chemical structure, Salmonella mutagenicity and extent of carcinogenicity as indicators of genotoxic carcinogenesis among 222 chemicals tested in rodents by the US NCI/NTP[J]. Mutation Research/Genetic Toxicology, 1988, 204(1): 17-115. 3. [3] Kazius J, McGuire R, Bursi R. Derivation and validation of toxicophores for mutagenicity prediction[J]. Journal of medicinal chemistry, 2005, 48(1): 312-320. 4. [4] Bailey A B, Chanderbhan R, Collazo-Braier N, et al. The use of structure-activity relationship analysis in the food contact notification program[J]. Regulatory Toxicology and Pharmacology, 2005, 42(2): 225-235.
NTD	✔	1. [1] Brenk R, Schipani A, James D, et al. Lessons learnt from assembling screening libraries for drug discovery for neglected diseases[J]. ChemMedChem: Chemistry Enabling Drug Discovery, 2008, 3(3): 435-444.
SureChEMBL Rule	✔	1. [1] Sushko I, Salmina E, Potemkin V A, et al. ToxAlerts: a web server of structural alerts for toxic chemicals and compounds with potential adverse reactions[J]. 2012.

Compounds AI10-a17:

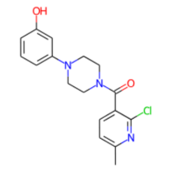

SMILES: Cc1ccc(C(=O)N2CCN(c3cccc(O)c3)CC2)c(Cl)n1

San ID	No data
CAS	No data
InChI	InChI=1S/C17H18ClN3O2/c1-12-5-6-15(16(18)19-12)17(23)21-9-7-20(8-10-21)13-3-2-4-14(22)11-13/h2-6,11,22H,7-10H2,1H3
InChIKey	JHKLZZXCEQLYJV-UHFFFAOYSA-N

View in third-party databases: No data

MW	331.11	HBA	4	HBD	1
PAINS Alert	false	QED	0.86	NRot	2
TPSA	56.67	logD	1.98	logP	2.13
logS	-3.42				

Bad Average Good Scoring Notice

Absorption

Caco2	-4.58	HIA	100.00%	P-gp Inhibitor	14.75%
Bioavailability	100.00%	HFE	-9.70		

Distribution

BBB Penetration	83.55%	PPBR	1.00%	VDSS	1.05

Metabolism

CYP2D6 Inhibitor	4.97%	CYP3A4 Inhibitor	46.98%	CYP2C9 Inhibitor	65.32%
CYP2D6 Substrate	98.95%	CYP3A4 Substrate	48.49%	CYP2C9 Substrate	97.53%
CYP2C19 Inhibitor	78.34%	CYP1A2 Inhibitor	6.95%		

Excretion

Half Life (hour)	3.01	Clearance (mL/min/g)	52.85

Tox

hERG	92.06%	DILI	75.91%	NR-AhR	12.03%
Carcinogencity	23.99%	LD_{50}	364.08	Respiratory Toxicity	23.69%
Eye Corrosion	0.43%				

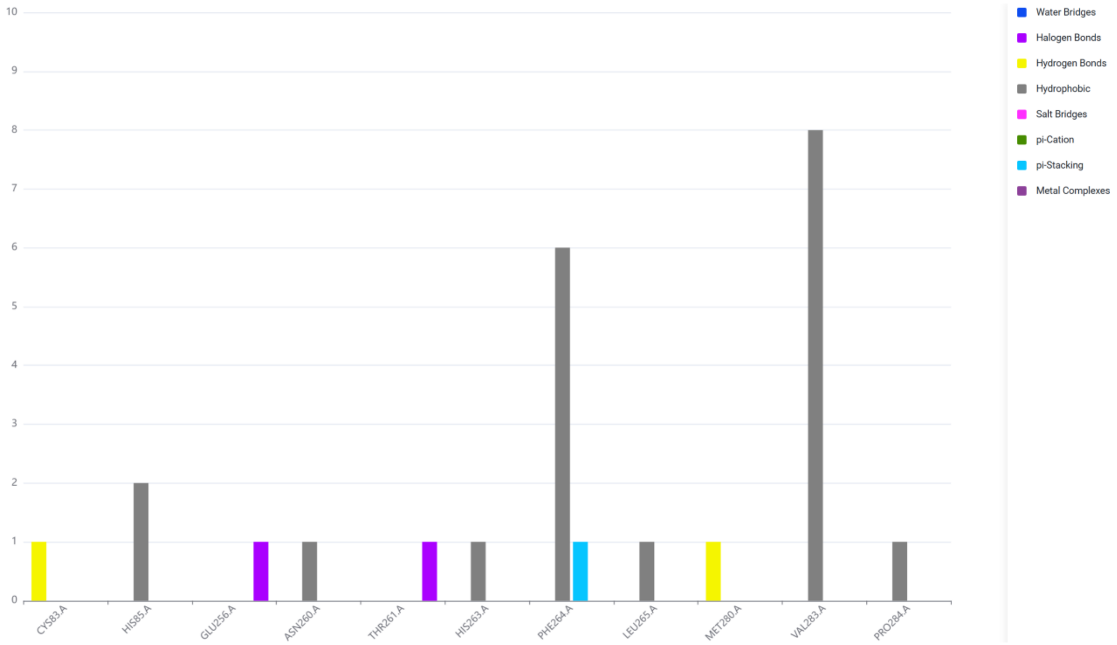
10
9
8
7
6
5
4
3
2
1
0
CYS83.A
HIS85.A
GLU256.A
ASN260.A
THR261.A
HIS263.A
PHE264.A
LEU265.A
MET280.A
VAL283.A
PRO284.A
Water Bridges
Halogen Bonds
Hydrogen Bonds
Hydrophobic
Salt Bridges
pi-Cation
pi-Stacking
Metal Complexes

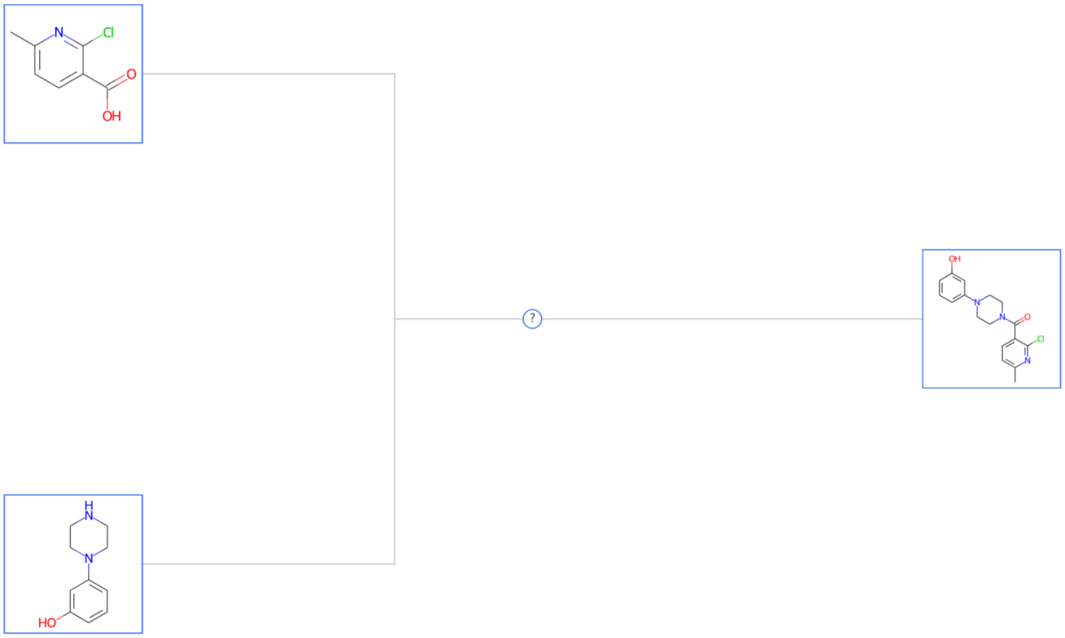
N
Cl
O
OH
?
H
N
N
HO

Alert Rule	Alert Structure	Reference
BMS Rule	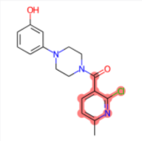	1. [1] Huth J R, Mendoza R, Olejniczak E T, et al. ALARM NMR: a rapid and robust experimental method to detect reactive false positives in biochemical screens[J]. Journal of the American Chemical Society, 2005, 127(1): 217-224.
Chelator Rule	✓	1. [1] Agrawal A, Johnson S L, Jacobsen J A, et al. Chelator fragment libraries for targeting metalloproteinases[J]. ChemMedChem: Chemistry Enabling Drug Discovery, 2010, 5(2): 195-199.
PAINS	✓	1. [1] Baell J B, Holloway G A. New substructure filters for removal of pan assay interference compounds (PAINS) from screening libraries and for their exclusion in bioassays[J]. Journal of medicinal chemistry, 2010, 53(7): 2719-2740.
Genotoxic Carcinogenicity Rule	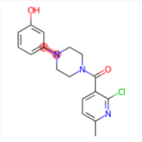	1. [1] Benigni R, Bossa C. Structure alerts for carcinogenicity, and the Salmonella assay system: a novel insight through the chemical relational databases technology[J]. Mutation Research/Reviews in Mutation Research, 2008, 659(3): 248-261.<br>2. [2] Ashby J, Tennant R W. Chemical structure, Salmonella mutagenicity and extent of carcinogenicity as indicators of genotoxic carcinogenesis among 222 chemicals tested in rodents by the US NCI/NTP[J]. Mutation Research/Genetic Toxicology, 1988, 204(1): 17-115.<br>3. [3] Kazius J, McGuire R, Bursi R. Derivation and validation of toxicophores for mutagenicity prediction[J]. Journal of medicinal chemistry, 2005, 48(1): 312-320.<br>4. [4] Bailey A B, Chanderbhan R, Collazo-Braier N, et al. The use of structure-activity relationship analysis in the food contact notification program[J]. Regulatory Toxicology and Pharmacology, 2005, 42(2): 225-235.
NTD	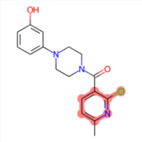	1. [1] Brenk R, Schipani A, James D, et al. Lessons learnt from assembling screening libraries for drug discovery for neglected diseases[J]. ChemMedChem: Chemistry Enabling Drug Discovery, 2008, 3(3): 435-444.
SureChEMBL Rule	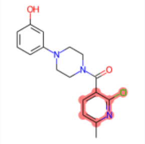	1. [1] Sushko I, Salmina E, Potemkin V A, et al. ToxAlerts: a web server of structural alerts for toxic chemicals and compounds with potential adverse reactions[J]. 2012.

Compounds AI10-a18:

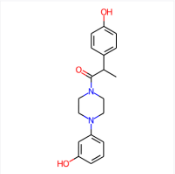

SMILES: CC(C(=O)N1CCN(c2cccc(O)c2)CC1)c1ccc(O)cc1

San ID	No data
CAS	No data
InChI	InChI=1S/C19H22N2O3/c1-14(15-5-7-17(22)8-6-15)19(24)21-11-9-20(10-12-21)16-3-2-4-18(23)13-16/h2-8,13-14,22-23H,9-12H2,1H3
InChIKey	YGQJZEATOSYLIT-UHFFFAOYSA-N

View in third-party databases: No data

MW	326.16	HBA	4	HBD	2
PAINS Alert	false	QED	0.91	NRot	3
TPSA	64.01	logD	1.76	logP	1.78
logS	-2.78				

Bad Average Good Scoring Notice

Absorption

Caco2	-4.85	HIA	100.00%	P-gp Inhibitor	41.24%
Bioavailability	99.01%	HFE	-11.64		

Distribution

BBB Penetration	74.05%	PPBR	1.00%	VDSS	0.66

Metabolism

CYP2D6 Inhibitor	8.19%	CYP3A4 Inhibitor	83.20%	CYP2C9 Inhibitor	76.88%
CYP2D6 Substrate	26.97%	CYP3A4 Substrate	68.60%	CYP2C9 Substrate	98.22%
CYP2C19 Inhibitor	84.39%	CYP1A2 Inhibitor	12.18%		

Excretion

Half Life (hour)	3.04	Clearance (mL/min/g)	56.37

Tox

hERG	88.34%	DILI	16.46%	NR-AhR	19.94%
Carcinogencity	20.50%	LD_{50}	636.97	Respiratory Toxicity	36.74%
Eye Corrosion	0.01%				

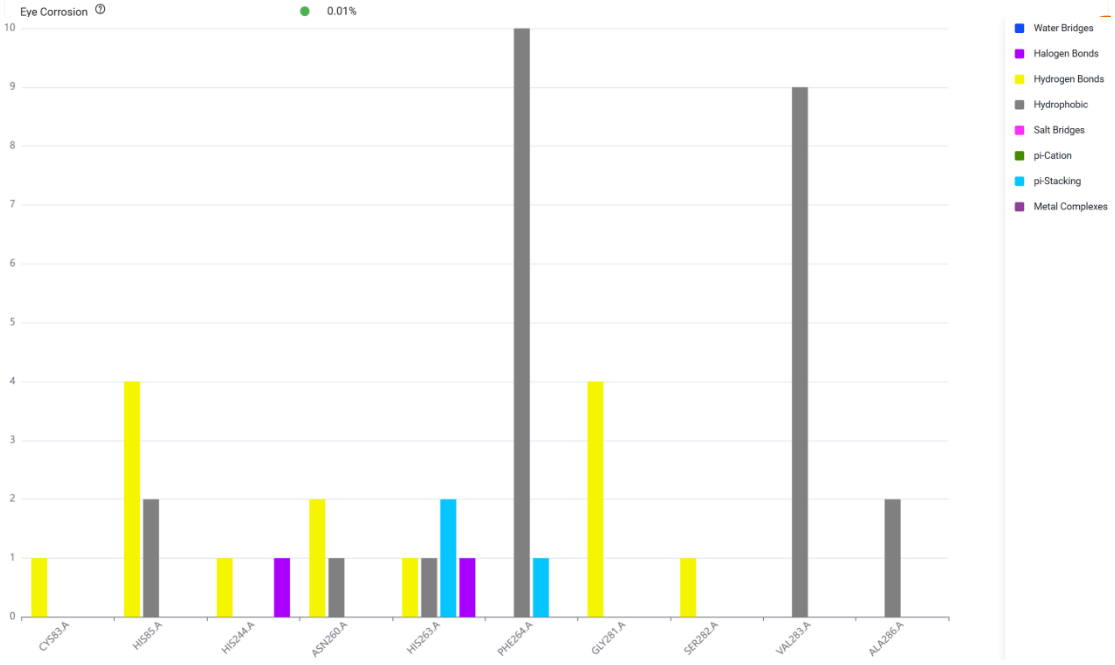

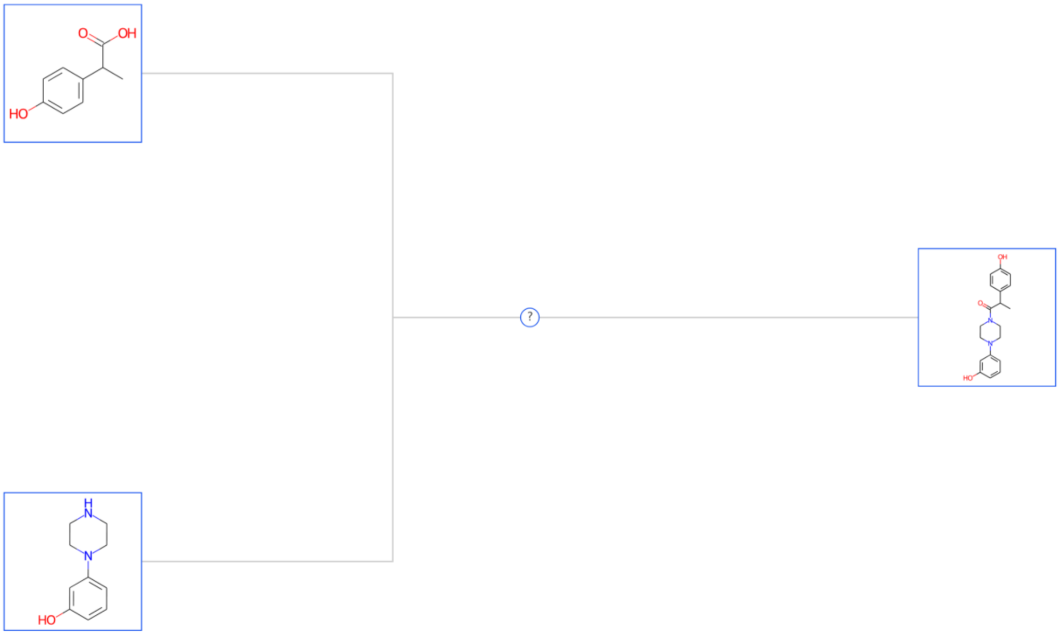

Alert Rule	Alert Structure	Reference
BMS Rule	✓	1. [1] Huth J R, Mendoza R, Olejniczak E T, et al. ALARM NMR: a rapid and robust experimental method to detect reactive false positives in biochemical screens[J]. Journal of the American Chemical Society, 2005, 127(1): 217-224.
Chelator Rule	✓	1. [1] Agrawal A, Johnson S L, Jacobsen J A, et al. Chelator fragment libraries for targeting metalloproteinases[J]. ChemMedChem: Chemistry Enabling Drug Discovery, 2010, 5(2): 195-199.
PAINS	✓	1. [1] Baell J B, Holloway G A. New substructure filters for removal of pan assay interference compounds (PAINS) from screening libraries and for their exclusion in bioassays[J]. Journal of medicinal chemistry, 2010, 53(7): 2719-2740.
Genotoxic Carcinogenicity Rule	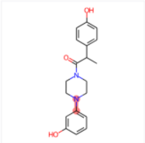	1. [1] Benigni R, Bossa C. Structure alerts for carcinogenicity, and the Salmonella assay system: a novel insight through the chemical relational databases technology[J]. Mutation Research/Reviews in Mutation Research, 2008, 659(3): 248-261.<br>2. [2] Ashby J, Tennant R W. Chemical structure, Salmonella mutagenicity and extent of carcinogenicity as indicators of genotoxic carcinogenesis among 222 chemicals tested in rodents by the US NCI/NTP[J]. Mutation Research/Genetic Toxicology, 1988, 204(1): 17-115.<br>3. [3] Kazius J, McGuire R, Bursi R. Derivation and validation of toxicophores for mutagenicity prediction[J]. Journal of medicinal chemistry, 2005, 48(1): 312-320.<br>4. [4] Bailey A B, Chanderbhan R, Collazo-Braier N, et al. The use of structure-activity relationship analysis in the food contact notification program[J]. Regulatory Toxicology and Pharmacology, 2005, 42(2): 225-235.
NTD	✓	1. [1] Brenk R, Schipani A, James D, et al. Lessons learnt from assembling screening libraries for drug discovery for neglected diseases[J]. ChemMedChem: Chemistry Enabling Drug Discovery, 2008, 3(3): 435-444.
SureChEMBL Rule	✓	1. [1] Sushko I, Salmina E, Potemkin V A, et al. ToxAlerts: a web server of structural alerts for toxic chemicals and compounds with potential adverse reactions[J]. 2012.

Compounds AI10-a19:

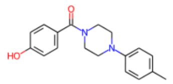

SMILES: Cc1ccc(N2CCN(C(=O)c3ccc(O)cc3)CC2)cc1

San ID	No data
CAS	No data
InChI	InChI=1S/C18H20N2O2/c1-14-2-6-16(7-3-14)19-10-12-20(13-11-19)18(22)15-4-8-17(21)9-5-15/h2-9,21H,10-13H2,1H3
InChIKey	CWKFZYVQUJIVCS-UHFFFAOYSA-N

View in third-party databases: Loading...

MW	296.15	HBA	3	HBD	1
PAINS Alert	true	QED	0.926	NRot	2
TPSA	43.78	logD	2.47	logP	2.92
logS	-3.02				

Bad Average Good Scoring Notice

Absorption

Caco2	-4.79	HIA	100.00%	P-gp Inhibitor	40.27%
Bioavailability	99.78%	HFE	-8.36		

Distribution

BBB Penetration	83.31%	PPBR	1.00%	VDSS	1.83

Metabolism

CYP2D6 Inhibitor	6.82%	CYP3A4 Inhibitor	79.54%	CYP2C9 Inhibitor	74.94%
CYP2D6 Substrate	96.50%	CYP3A4 Substrate	52.92%	CYP2C9 Substrate	0.86%
CYP2C19 Inhibitor	83.60%	CYP1A2 Inhibitor	14.01%		

Excretion

Half Life (hour)	3.45	Clearance (mL/min/g)	47.52

Tox

hERG	95.70%	DILI	15.65%	NR-AhR	19.06%
Carcinogencity	80.76%	LD_{50}	831.59	Respiratory Toxicity	9.54%
Eye Corrosion	0.09%				

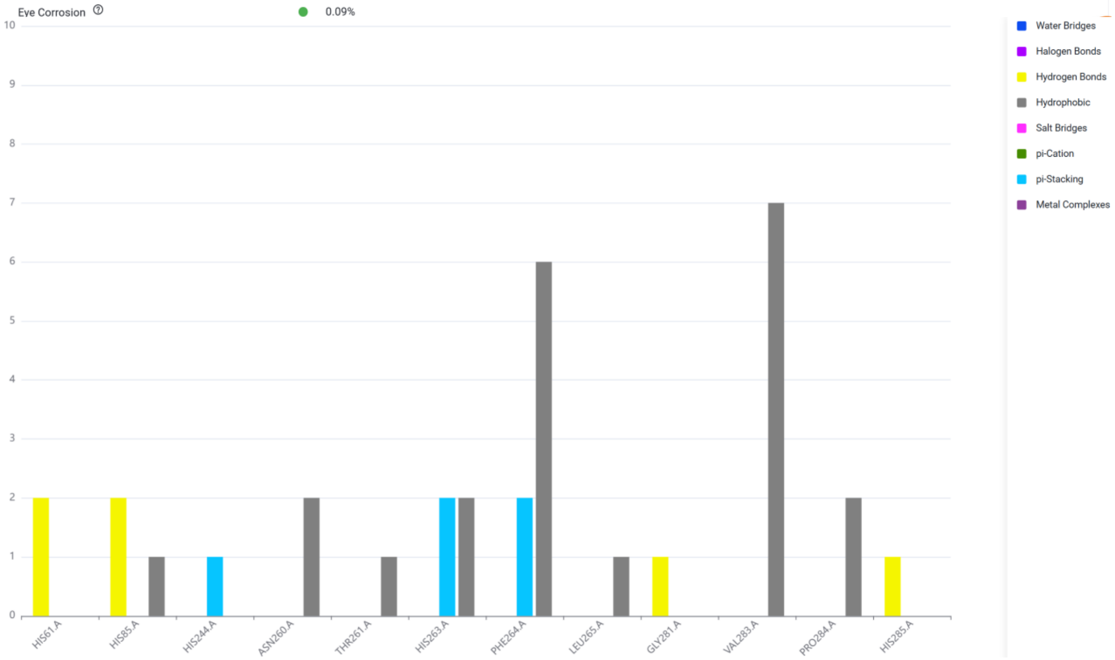

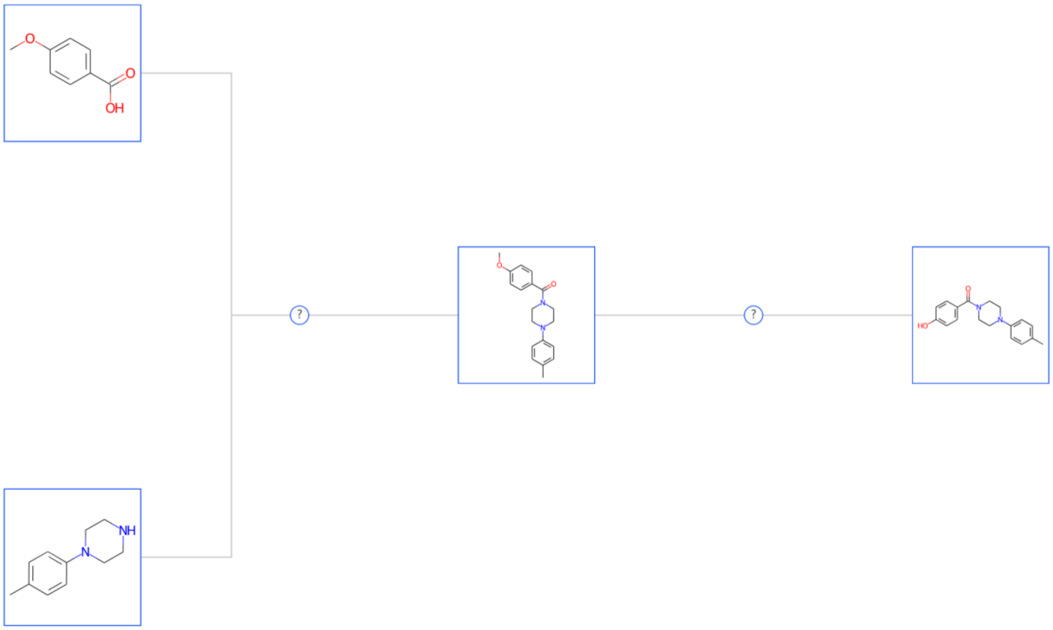

Alert Rule	Alert Structure	Reference
BMS Rule	✓	1. [1] Huth J R, Mendoza R, Olejniczak E T, et al. ALARM NMR: a rapid and robust experimental method to detect reactive false positives in biochemical screens[J]. Journal of the American Chemical Society, 2005, 127(1): 217-224.
Chelator Rule	✓	1. [1] Agrawal A, Johnson S L, Jacobsen J A, et al. Chelator fragment libraries for targeting metalloproteinases[J]. ChemMedChem: Chemistry Enabling Drug Discovery, 2010, 5(2): 195-199.
PAINS	✓	1. [1] Baell J B, Holloway G A. New substructure filters for removal of pan assay interference compounds (PAINS) from screening libraries and for their exclusion in bioassays[J]. Journal of medicinal chemistry, 2010, 53(7): 2719-2740.
Genotoxic Carcinogenicity Rule	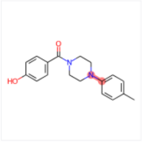	1. [1] Benigni R, Bossa C. Structure alerts for carcinogenicity, and the Salmonella assay system: a novel insight through the chemical relational databases technology[J]. Mutation Research/Reviews in Mutation Research, 2008, 659(3): 248-261.<br>2. [2] Ashby J, Tennant R W. Chemical structure, Salmonella mutagenicity and extent of carcinogenicity as indicators of genotoxic carcinogenesis among 222 chemicals tested in rodents by the US NCI/NTP[J]. Mutation Research/Genetic Toxicology, 1988, 204(1): 17-115.<br>3. [3] Kazius J, McGuire R, Bursi R. Derivation and validation of toxicophores for mutagenicity prediction[J]. Journal of medicinal chemistry, 2005, 48(1): 312-320.<br>4. [4] Bailey A B, Chanderbhan R, Collazo-Braier N, et al. The use of structure-activity relationship analysis in the food contact notification program[J]. Regulatory Toxicology and Pharmacology, 2005, 42(2): 225-235.
NTD	✓	1. [1] Brenk R, Schipani A, James D, et al. Lessons learnt from assembling screening libraries for drug discovery for neglected diseases[J]. ChemMedChem: Chemistry Enabling Drug Discovery, 2008, 3(3): 435-444.
SureChEMBL Rule	✓	1. [1] Sushko I, Salmina E, Potemkin V A, et al. ToxAlerts: a web server of structural alerts for toxic chemicals and compounds with potential adverse reactions[J]. 2012.

Compounds AI10-a20:

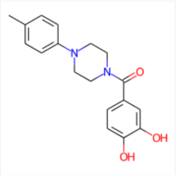

SMILES: Cc1ccc(N2CCN(C(=O)c3ccc(O)c(O)c3)CC2)cc1

San ID	No data
CAS	No data
InChI	InChI=1S/C18H20N2O3/c1-13-2-5-15(6-3-13)19-8-10-20(11-9-19)18(23)14-4-7-16(21)17(22)12-14/h2-7,12,21-22H,8-11H2,1H3
InChIKey	WCTIJOVIWLPYHU-UHFFFAOYSA-N

View in third-party databases: No data

MW	312.15	HBA	4	HBD	2
PAINS Alert	true	QED	0.836	NRot	2
TPSA	64.01	logD	1.48	logP	2.36
logS	-3.08				

Bad Average Good Scoring Notice

Absorption

Caco2	-4.97	HIA	100.00%	P-gp Inhibitor	21.47%
Bioavailability	99.76%	HFE	-10.74		

Distribution

BBB Penetration	71.53%	PPBR	1.00%	VDSS	0.75

Metabolism

CYP2D6 Inhibitor	7.45%	CYP3A4 Inhibitor	84.86%	CYP2C9 Inhibitor	78.97%
CYP2D6 Substrate	0.36%	CYP3A4 Substrate	32.24%	CYP2C9 Substrate	1.16%
CYP2C19 Inhibitor	77.19%	CYP1A2 Inhibitor	20.84%		

Excretion

Half Life (hour)	2.81	Clearance (mL/min/g)	51.50

Tox

hERG	92.86%	DILI	19.15%	NR-AhR	31.53%
Carcinogencity	25.35%	LD_{50}	950.87	Respiratory Toxicity	29.19%
Eye Corrosion	0.08%				

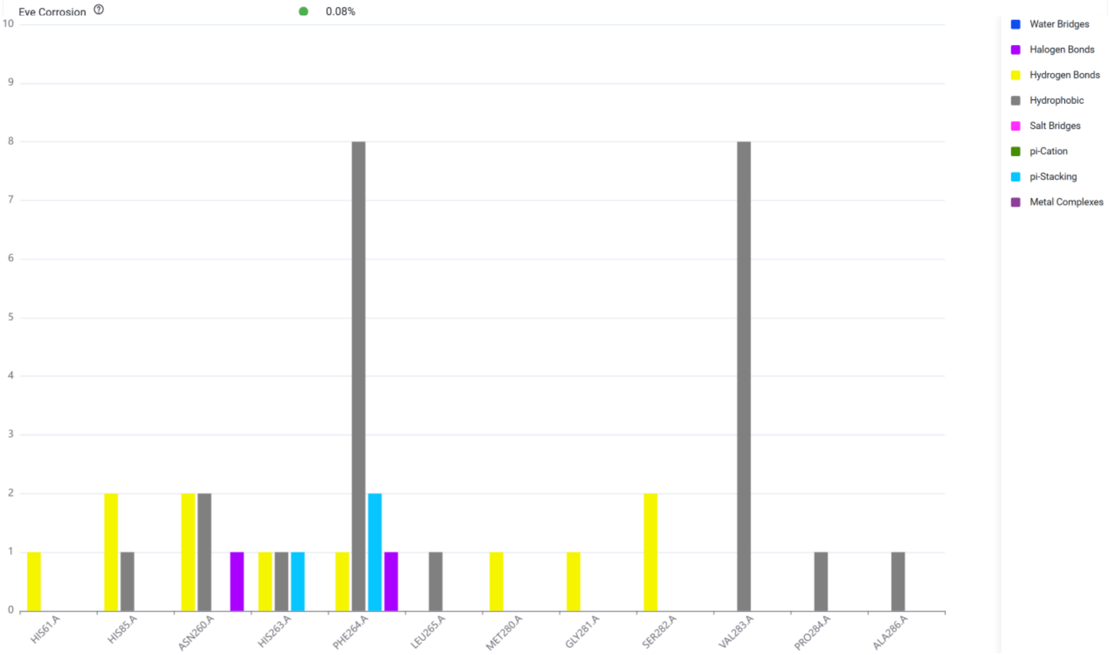

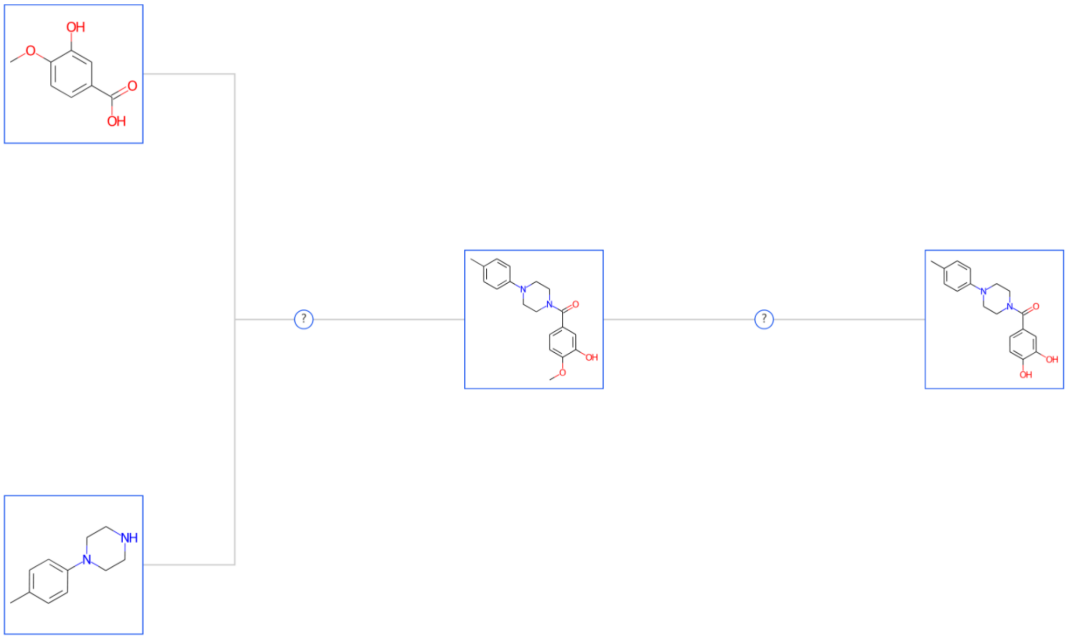

Alert Rule	Alert Structure	Reference
BMS Rule	✔	1. [1] Huth J R, Mendoza R, Olejniczak E T, et al. ALARM NMR: a rapid and robust experimental method to detect reactive false positives in biochemical screens[J]. Journal of the American Chemical Society, 2005, 127(1): 217-224.
Chelator Rule	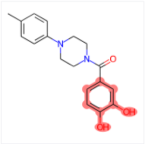	1. [1] Agrawal A, Johnson S L, Jacobsen J A, et al. Chelator fragment libraries for targeting metalloproteinases[J]. ChemMedChem: Chemistry Enabling Drug Discovery, 2010, 5(2): 195-199.
PAINS	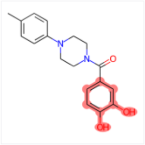	1. [1] Baell J B, Holloway G A. New substructure filters for removal of pan assay interference compounds (PAINS) from screening libraries and for their exclusion in bioassays[J]. Journal of medicinal chemistry, 2010, 53(7): 2719-2740.
Genotoxic Carcinogenicity Rule	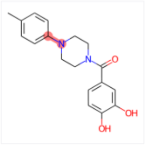	1. [1] Benigni R, Bossa C. Structure alerts for carcinogenicity, and the Salmonella assay system: a novel insight through the chemical relational databases technology[J]. Mutation Research/Reviews in Mutation Research, 2008, 659(3): 248-261.<br>2. [2] Ashby J, Tennant R W. Chemical structure, Salmonella mutagenicity and extent of carcinogenicity as indicators of genotoxic carcinogenesis among 222 chemicals tested in rodents by the US NCI/NTP[J]. Mutation Research/Genetic Toxicology, 1988, 204(1): 17-115.<br>3. [3] Kazius J, McGuire R, Bursi R. Derivation and validation of toxicophores for mutagenicity prediction[J]. Journal of medicinal chemistry, 2005, 48(1): 312-320.<br>4. [4] Bailey A B, Chanderbhan R, Collazo-Braier N, et al. The use of structure-activity relationship analysis in the food contact notification program[J]. Regulatory Toxicology and Pharmacology, 2005, 42(2): 225-235.
NTD	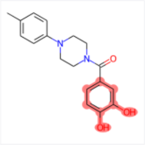	1. [1] Brenk R, Schipani A, James D, et al. Lessons learnt from assembling screening libraries for drug discovery for neglected diseases[J]. ChemMedChem: Chemistry Enabling Drug Discovery, 2008, 3(3): 435-444.
SureChEMBL Rule	✔	1. [1] Sushko I, Salmina E, Potemkin V A, et al. ToxAlerts: a web server of structural alerts for toxic chemicals and compounds with potential adverse reactions[J]. 2012.

Compounds AI10-a21:

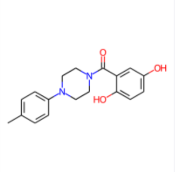

SMILES: Cc1ccc(N2CCN(C(=O)c3cc(O)ccc3O)CC2)cc1

San ID	No data
CAS	No data
InChI	InChI=1S/C18H20N2O3/c1-13-2-4-14(5-3-13)19-8-10-20(11-9-19)18(23)16-12-15(21)6-7-17(16)22/h2-7,12,21-22H,8-11H2,1H3
InChIKey	QPBUABHBHLVRTO-UHFFFAOYSA-N

View in third-party databases: Loading...

MW	312.15	HBA	4	HBD	2
PAINS Alert	true	QED	0.836	NRot	2
TPSA	64.01	logD	2.24	logP	2.76
logS	-4.08				

Bad Average Good Scoring Notice

Absorption

Caco2	-4.90	HIA	99.19%	P-gp Inhibitor	74.70%
Bioavailability	99.97%	HFE	-10.05		

Distribution

BBB Penetration	74.88%	PPBR	1.00%	VDSS	2.08

Metabolism

CYP2D6 Inhibitor	11.64%	CYP3A4 Inhibitor	80.86%	CYP2C9 Inhibitor	79.95%
CYP2D6 Substrate	47.24%	CYP3A4 Substrate	33.57%	CYP2C9 Substrate	24.49%
CYP2C19 Inhibitor	86.28%	CYP1A2 Inhibitor	26.35%		

Excretion

Half Life (hour)	3.44	Clearance (mL/min/g)	51.40

Tox

hERG	93.14%	DILI	51.85%	NR-AhR	26.63%
Carcinogencity	29.71%	LD_{50}	517.70	Respiratory Toxicity	32.28%
Eye Corrosion	0.09%				

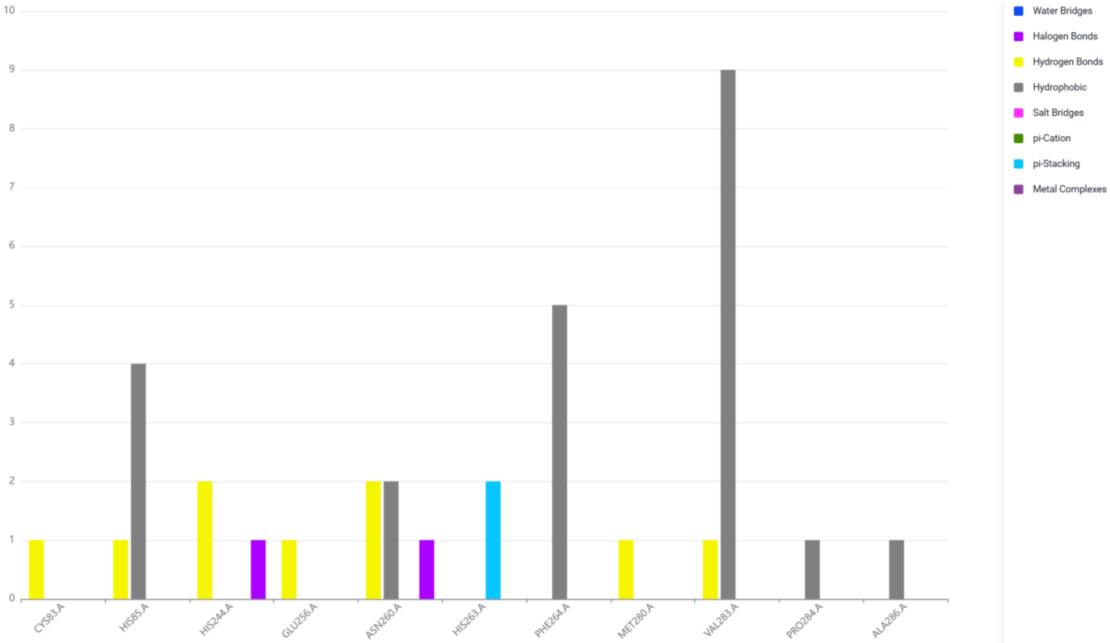

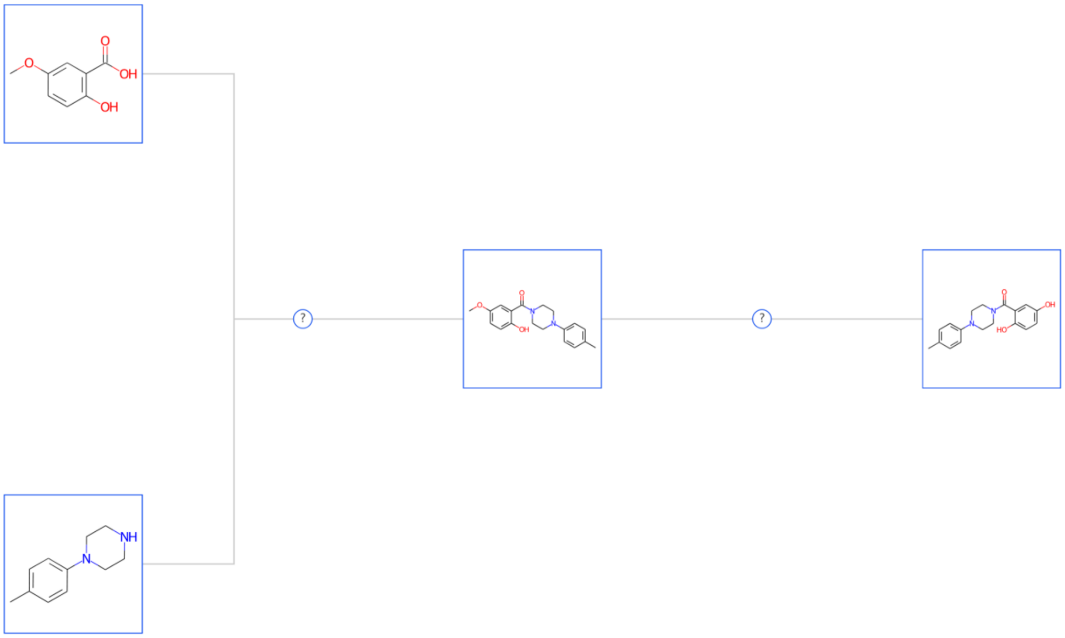

Alert Rule	Alert Structure	Reference
BMS Rule	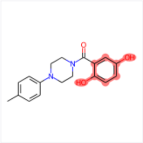	1. [1] Huth J R, Mendoza R, Olejniczak E T, et al. ALARM NMR: a rapid and robust experimental method to detect reactive false positives in biochemical screens[J]. Journal of the American Chemical Society, 2005, 127(1): 217-224.
Chelator Rule	✓	1. [1] Agrawal A, Johnson S L, Jacobsen J A, et al. Chelator fragment libraries for targeting metalloproteinases[J]. ChemMedChem: Chemistry Enabling Drug Discovery, 2010, 5(2): 195-199.
PAINS	✓	1. [1] Baell J B, Holloway G A. New substructure filters for removal of pan assay interference compounds (PAINS) from screening libraries and for their exclusion in bioassays[J]. Journal of medicinal chemistry, 2010, 53(7): 2719-2740.
Genotoxic Carcinogenicity Rule	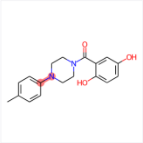	1. [1] Benigni R, Bossa C. Structure alerts for carcinogenicity, and the Salmonella assay system: a novel insight through the chemical relational databases technology[J]. Mutation Research/Reviews in Mutation Research, 2008, 659(3): 248-261.<br>2. [2] Ashby J, Tennant R W. Chemical structure, Salmonella mutagenicity and extent of carcinogenicity as indicators of genotoxic carcinogenesis among 222 chemicals tested in rodents by the US NCI/NTP[J]. Mutation Research/Genetic Toxicology, 1988, 204(1): 17-115.<br>3. [3] Kazius J, McGuire R, Bursi R. Derivation and validation of toxicophores for mutagenicity prediction[J]. Journal of medicinal chemistry, 2005, 48(1): 312-320.<br>4. [4] Bailey A B, Chanderbhan R, Collazo-Braier N, et al. The use of structure-activity relationship analysis in the food contact notification program[J]. Regulatory Toxicology and Pharmacology, 2005, 42(2): 225-235.
NTD	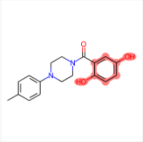	1. [1] Brenk R, Schipani A, James D, et al. Lessons learnt from assembling screening libraries for drug discovery for neglected diseases[J]. ChemMedChem: Chemistry Enabling Drug Discovery, 2008, 3(3): 435-444.
SureChEMBL Rule	✓	1. [1] Sushko I, Salmina E, Potemkin V A, et al. ToxAlerts: a web server of structural alerts for toxic chemicals and compounds with potential adverse reactions[J]. 2012.

Compounds AI10-a22:

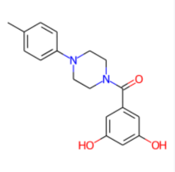

SMILES: Cc1ccc(N2CCN(C(=O)c3cc(O)cc(O)c3)CC2)cc1

San ID	No data
CAS	No data
InChI	InChI=1S/C18H20N2O3/c1-13-2-4-15(5-3-13)19-6-8-20(9-7-19)18(23)14-10-16(21)12-17(22)11-14/h2-5,10-12,21-22H,6-9H2,1H3
InChIKey	GLNLCYWGEBKWID-UHFFFAOYSA-N

View in third-party databases: Loading...

MW	312.15	HBA	4	HBD	2
PAINS Alert	true	QED	0.893	NRot	2
TPSA	64.01	logD	2.15	logP	2.08
logS	-3.15				

Bad Average Good Scoring Notice

Absorption

Caco2	-4.85	HIA	99.69%	P-gp Inhibitor	89.35%
Bioavailability	99.69%	HFE	-10.71		

Distribution

BBB Penetration	77.82%	PPBR	1.00%	VDSS	1.29

Metabolism

CYP2D6 Inhibitor	9.18%	CYP3A4 Inhibitor	94.04%	CYP2C9 Inhibitor	82.47%
CYP2D6 Substrate	9.98%	CYP3A4 Substrate	29.83%	CYP2C9 Substrate	1.60%
CYP2C19 Inhibitor	88.82%	CYP1A2 Inhibitor	53.12%		

Excretion

Half Life (hour)	3.61	Clearance (mL/min/g)	49.59

Tox

hERG	93.91%	DILI	23.86%	NR-AhR	30.77%
Carcinogencity	25.92%	LD_{50}	603.74	Respiratory Toxicity	20.75%
Eye Corrosion	0.06%				

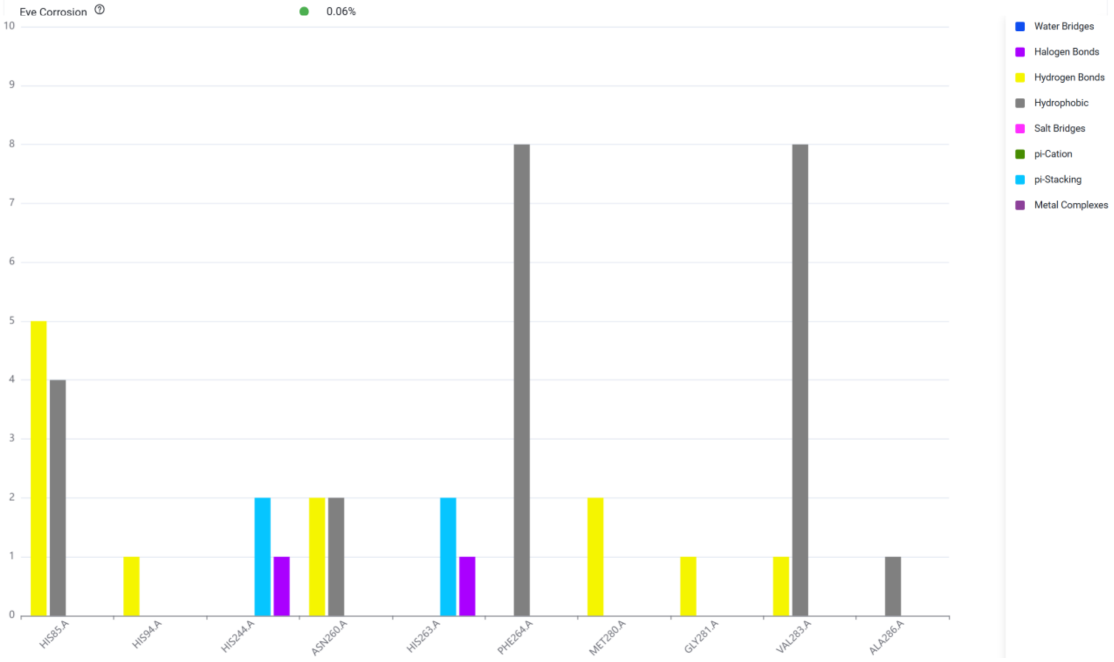

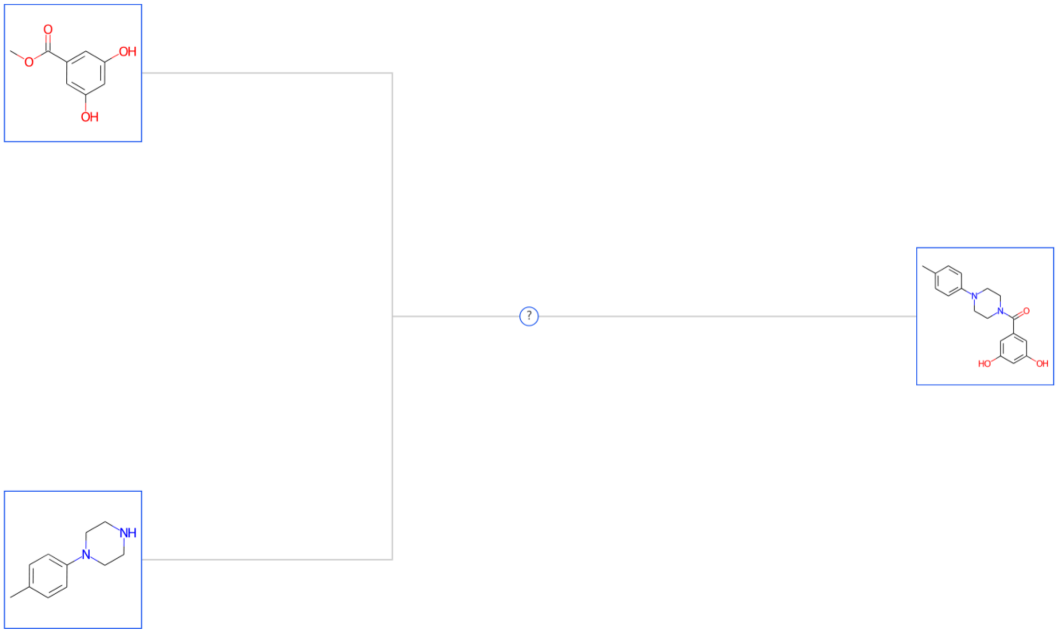

Alert Rule	Alert Structure	Reference
BMS Rule	✓	1. [1] Huth J R, Mendoza R, Olejniczak E T, et al. ALARM NMR: a rapid and robust experimental method to detect reactive false positives in biochemical screens[J]. Journal of the American Chemical Society, 2005, 127(1): 217-224.
Chelator Rule	✓	1. [1] Agrawal A, Johnson S L, Jacobsen J A, et al. Chelator fragment libraries for targeting metalloproteinases[J]. ChemMedChem: Chemistry Enabling Drug Discovery, 2010, 5(2): 195-199.
PAINS	✓	1. [1] Baell J B, Holloway G A. New substructure filters for removal of pan assay interference compounds (PAINS) from screening libraries and for their exclusion in bioassays[J]. Journal of medicinal chemistry, 2010, 53(7): 2719-2740.
Genotoxic Carcinogenicity Rule	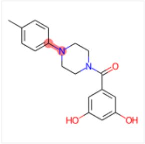	1. [1] Benigni R, Bossa C. Structure alerts for carcinogenicity, and the Salmonella assay system: a novel insight through the chemical relational databases technology[J]. Mutation Research/Reviews in Mutation Research, 2008, 659(3): 248-261.<br>2. [2] Ashby J, Tennant R W. Chemical structure, Salmonella mutagenicity and extent of carcinogenicity as indicators of genotoxic carcinogenesis among 222 chemicals tested in rodents by the US NCI/NTP[J]. Mutation Research/Genetic Toxicology, 1988, 204(1): 17-115.<br>3. [3] Kazius J, McGuire R, Bursi R. Derivation and validation of toxicophores for mutagenicity prediction[J]. Journal of medicinal chemistry, 2005, 48(1): 312-320.<br>4. [4] Bailey A B, Chanderbhan R, Collazo-Braier N, et al. The use of structure-activity relationship analysis in the food contact notification program[J]. Regulatory Toxicology and Pharmacology, 2005, 42(2): 225-235.
NTD	✓	1. [1] Brenk R, Schipani A, James D, et al. Lessons learnt from assembling screening libraries for drug discovery for neglected diseases[J]. ChemMedChem: Chemistry Enabling Drug Discovery, 2008, 3(3): 435-444.
SureChEMBL Rule	✓	1. [1] Sushko I, Salmina E, Potemkin V A, et al. ToxAlerts: a web server of structural alerts for toxic chemicals and compounds with potential adverse reactions[J]. 2012.

Compounds AI10-a23:

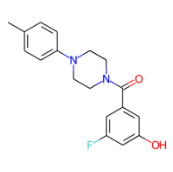

SMILES: Cc1ccc(N2CCN(C(=O)c3cc(O)cc(F)c3)CC2)cc1

San ID	No data
CAS	No data
InChI	InChI=1S/C18H19FN2O2/c1-13-2-4-16(5-3-13)20-6-8-21(9-7-20)18(23)14-10-15(19)12-17(22)11-14/h2-5,10-12,22H,6-9H2,1H3
InChIKey	UKXGKSFCAGUJPV-UHFFFAOYSA-N

View in third-party databases: Loading...

MW	314.14	HBA	3	HBD	1
PAINS Alert	true	QED	0.927	NRot	2
TPSA	43.78	logD	2.9	logP	3.27
logS	-4.32				

Bad Average Good Scoring Notice

Absorption

Caco2	-4.62	HIA	99.72%	P-gp Inhibitor	68.98%
Bioavailability	99.98%	HFE	-8.74		

Distribution

BBB Penetration	84.41%	PPBR	1.00%	VDSS	2.86

Metabolism

CYP2D6 Inhibitor	8.56%	CYP3A4 Inhibitor	83.56%	CYP2C9 Inhibitor	77.50%
CYP2D6 Substrate	92.34%	CYP3A4 Substrate	42.56%	CYP2C9 Substrate	1.79%
CYP2C19 Inhibitor	87.09%	CYP1A2 Inhibitor	25.50%		

Excretion

Half Life (hour)	4.75	Clearance (mL/min/g)	48.81

Tox

hERG	95.99%	DILI	79.99%	NR-AhR	24.99%
Carcinogencity	16.86%	LD_{50}	579.82	Respiratory Toxicity	10.71%
Eye Corrosion	0.18%				

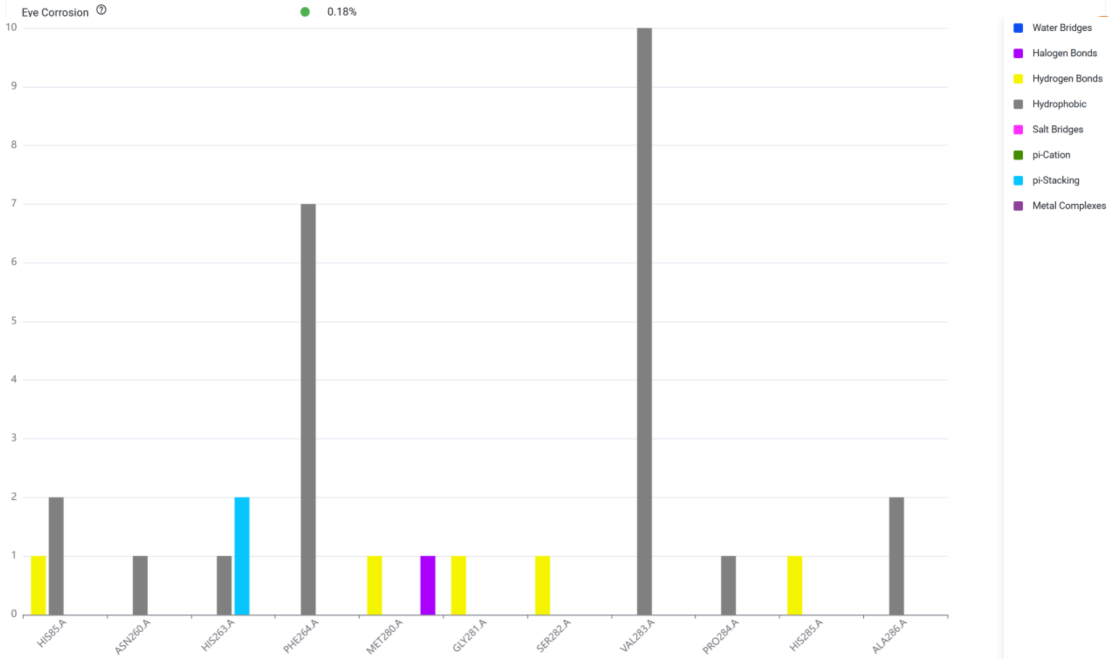

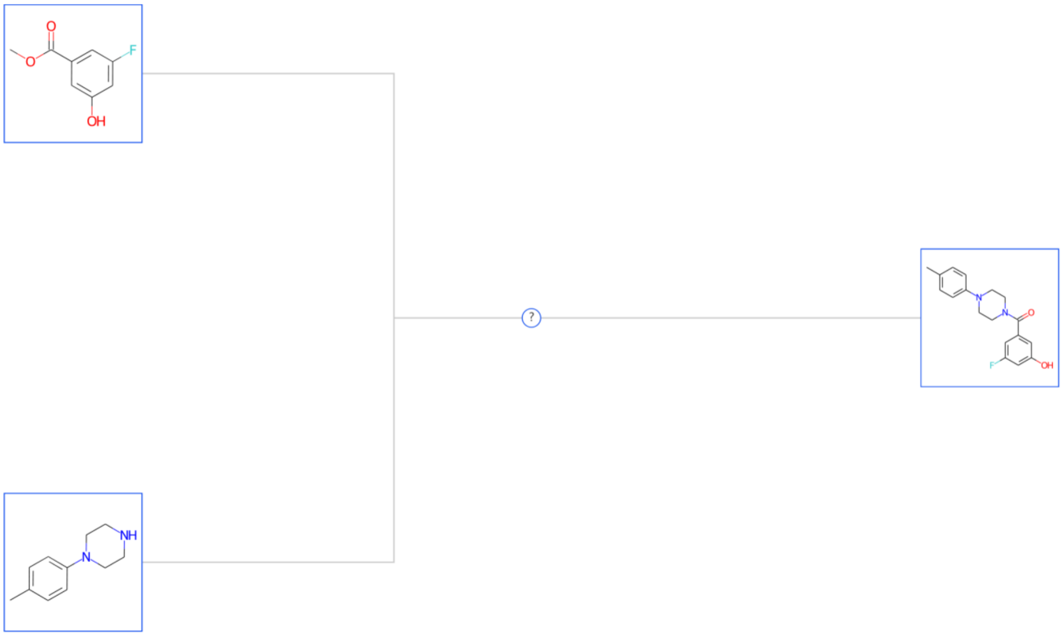

Alert Rule	Alert Structure	Reference
BMS Rule	✓	1. [1] Huth J R, Mendoza R, Olejniczak E T, et al. ALARM NMR: a rapid and robust experimental method to detect reactive false positives in biochemical screens[J]. Journal of the American Chemical Society, 2005, 127(1): 217-224.
Chelator Rule	✓	1. [1] Agrawal A, Johnson S L, Jacobsen J A, et al. Chelator fragment libraries for targeting metalloproteinases[J]. ChemMedChem: Chemistry Enabling Drug Discovery, 2010, 5(2): 195-199.
PAINS	✓	1. [1] Baell J B, Holloway G A. New substructure filters for removal of pan assay interference compounds (PAINS) from screening libraries and for their exclusion in bioassays[J]. Journal of medicinal chemistry, 2010, 53(7): 2719-2740.
Genotoxic Carcinogenicity Rule	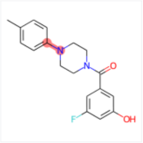	1. [1] Benigni R, Bossa C. Structure alerts for carcinogenicity, and the Salmonella assay system: a novel insight through the chemical relational databases technology[J]. Mutation Research/Reviews in Mutation Research, 2008, 659(3): 248-261.<br>2. [2] Ashby J, Tennant R W. Chemical structure, Salmonella mutagenicity and extent of carcinogenicity as indicators of genotoxic carcinogenesis among 222 chemicals tested in rodents by the US NCI/NTP[J]. Mutation Research/Genetic Toxicology, 1988, 204(1): 17-115.<br>3. [3] Kazius J, McGuire R, Bursi R. Derivation and validation of toxicophores for mutagenicity prediction[J]. Journal of medicinal chemistry, 2005, 48(1): 312-320.<br>4. [4] Bailey A B, Chanderbhan R, Collazo-Braier N, et al. The use of structure-activity relationship analysis in the food contact notification program[J]. Regulatory Toxicology and Pharmacology, 2005, 42(2): 225-235.
NTD	✓	1. [1] Brenk R, Schipani A, James D, et al. Lessons learnt from assembling screening libraries for drug discovery for neglected diseases[J]. ChemMedChem: Chemistry Enabling Drug Discovery, 2008, 3(3): 435-444.
SureChEMBL Rule	✓	1. [1] Sushko I, Salmina E, Potemkin V A, et al. ToxAlerts: a web server of structural alerts for toxic chemicals and compounds with potential adverse reactions[J]. 2012.

Compounds AI10-a24:

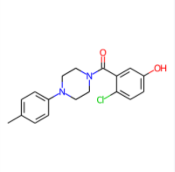

SMILES: Cc1ccc(N2CCN(C(=O)c3cc(O)ccc3Cl)CC2)cc1

San ID	No data
CAS	No data
InChI	InChI=1S/C18H19ClN2O2/c1-13-2-4-14(5-3-13)20-8-10-21(11-9-20)18(23)16-12-15(22)6-7-17(16)19/h2-7,12,22H,8-11H2,1H3
InChIKey	BZNKFMPCTWCIEJ-UHFFFAOYSA-N

View in third-party databases: No data

MW	330.11	HBA	3	HBD	1
PAINS Alert	true	QED	0.918	NRot	2
TPSA	43.78	logD	2.96	logP	3.46
logS	-4.06				

Bad Average Good Scoring Notice

Absorption

Caco2	-4.63	HIA	99.63%	P-gp Inhibitor	65.39%
Bioavailability	99.86%	HFE	-8.78		

Distribution

BBB Penetration	87.62%	PPBR	1.00%	VDSS	2.56

Metabolism

CYP2D6 Inhibitor	9.77%	CYP3A4 Inhibitor	83.65%	CYP2C9 Inhibitor	80.86%
CYP2D6 Substrate	96.95%	CYP3A4 Substrate	35.77%	CYP2C9 Substrate	99.69%
CYP2C19 Inhibitor	91.02%	CYP1A2 Inhibitor	22.88%		

Excretion

Half Life (hour)	3.99	Clearance (mL/min/g)	47.62

Tox

hERG	95.85%	DILI	75.15%	NR-AhR	23.15%
Carcinogencity	25.30%	LD_{50}	525.81	Respiratory Toxicity	12.63%
Eye Corrosion	0.20%				

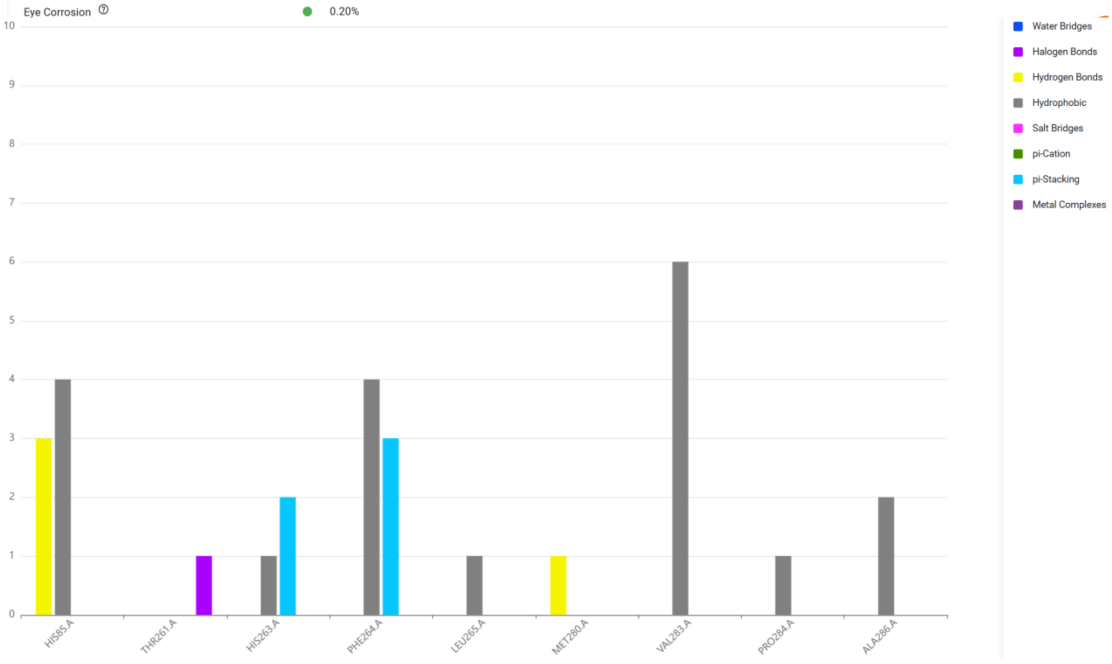

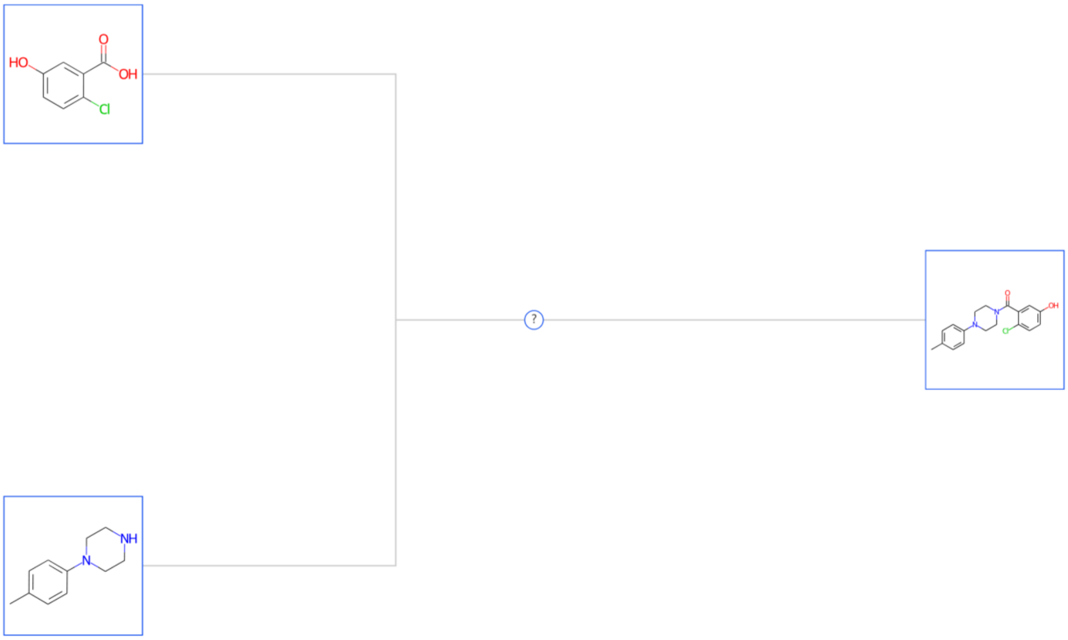

Alert Rule	Alert Structure	Reference
BMS Rule	✔	1. [1] Huth J R, Mendoza R, Olejniczak E T, et al. ALARM NMR: a rapid and robust experimental method to detect reactive false positives in biochemical screens[J]. Journal of the American Chemical Society, 2005, 127(1): 217-224.
Chelator Rule	✔	1. [1] Agrawal A, Johnson S L, Jacobsen J A, et al. Chelator fragment libraries for targeting metalloproteinases[J]. ChemMedChem: Chemistry Enabling Drug Discovery, 2010, 5(2): 195-199.
PAINS	✔	1. [1] Baell J B, Holloway G A. New substructure filters for removal of pan assay interference compounds (PAINS) from screening libraries and for their exclusion in bioassays[J]. Journal of medicinal chemistry, 2010, 53(7): 2719-2740.
Genotoxic Carcinogenicity Rule	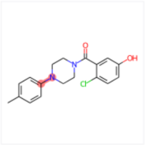	1. [1] Benigni R, Bossa C. Structure alerts for carcinogenicity, and the Salmonella assay system: a novel insight through the chemical relational databases technology[J]. Mutation Research/Reviews in Mutation Research, 2008, 659(3): 248-261.<br>2. [2] Ashby J, Tennant R W. Chemical structure, Salmonella mutagenicity and extent of carcinogenicity as indicators of genotoxic carcinogenesis among 222 chemicals tested in rodents by the US NCI/NTP[J]. Mutation Research/Genetic Toxicology, 1988, 204(1): 17-115.<br>3. [3] Kazius J, McGuire R, Bursi R. Derivation and validation of toxicophores for mutagenicity prediction[J]. Journal of medicinal chemistry, 2005, 48(1): 312-320.<br>4. [4] Bailey A B, Chanderbhan R, Collazo-Braier N, et al. The use of structure-activity relationship analysis in the food contact notification program[J]. Regulatory Toxicology and Pharmacology, 2005, 42(2): 225-235.
NTD	✔	1. [1] Brenk R, Schipani A, James D, et al. Lessons learnt from assembling screening libraries for drug discovery for neglected diseases[J]. ChemMedChem: Chemistry Enabling Drug Discovery, 2008, 3(3): 435-444.
SureChEMBL Rule	✔	1. [1] Sushko I, Salmina E, Potemkin V A, et al. ToxAlerts: a web server of structural alerts for toxic chemicals and compounds with potential adverse reactions[J]. 2012.

Compounds AI10-a25:

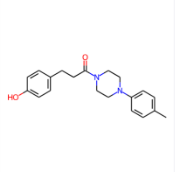

SMILES: Cc1ccc(N2CCN(C(=O)CCc3ccc(O)cc3)CC2)cc1

San ID	No data
CAS	No data
InChI	InChI=1S/C20H24N2O2/c1-16-2-7-18(8-3-16)21-12-14-22(15-13-21)20(24)11-6-17-4-9-19(23)10-5-17/h2-5,7-10,23H,6,11-15H2,1H3
InChIKey	XWNCRCXXKRSALC-UHFFFAOYSA-N

View in third-party databases: Loading...

MW	324.18	HBA	3	HBD	1
PAINS Alert	true	QED	0.94	NRot	4
TPSA	43.78	logD	2.72	logP	3.29
logS	-3.45				

Bad Average Good Scoring Notice

Absorption

Caco2	-4.84	HIA	100.00%	P-gp Inhibitor	69.30%
Bioavailability	99.46%	HFE	-9.52		

Distribution

BBB Penetration	83.51%	PPBR	1.00%	VDSS	1.07

Metabolism

CYP2D6 Inhibitor	11.83%	CYP3A4 Inhibitor	92.02%	CYP2C9 Inhibitor	77.79%
CYP2D6 Substrate	83.72%	CYP3A4 Substrate	36.30%	CYP2C9 Substrate	24.29%
CYP2C19 Inhibitor	91.87%	CYP1A2 Inhibitor	28.18%		

Excretion

Half Life (hour)	3.95	Clearance (mL/min/g)	51.00

Tox

hERG	95.83%	DILI	19.03%	NR-AhR	22.43%
Carcinogencity	13.38%	LD_{50}	856.52	Respiratory Toxicity	6.80%
Eye Corrosion	0.01%				

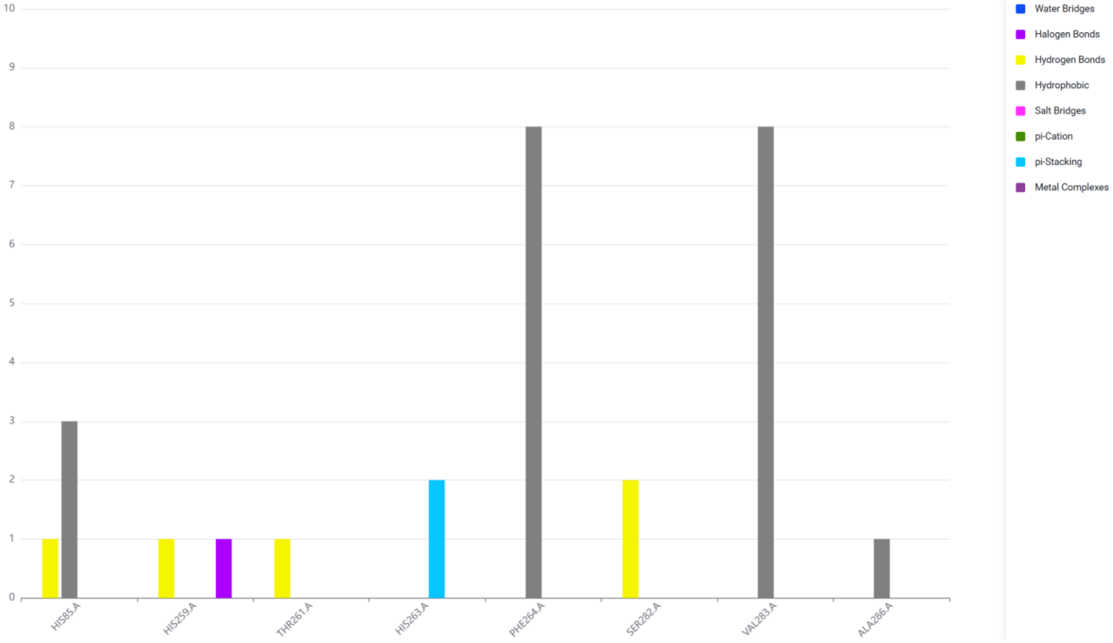

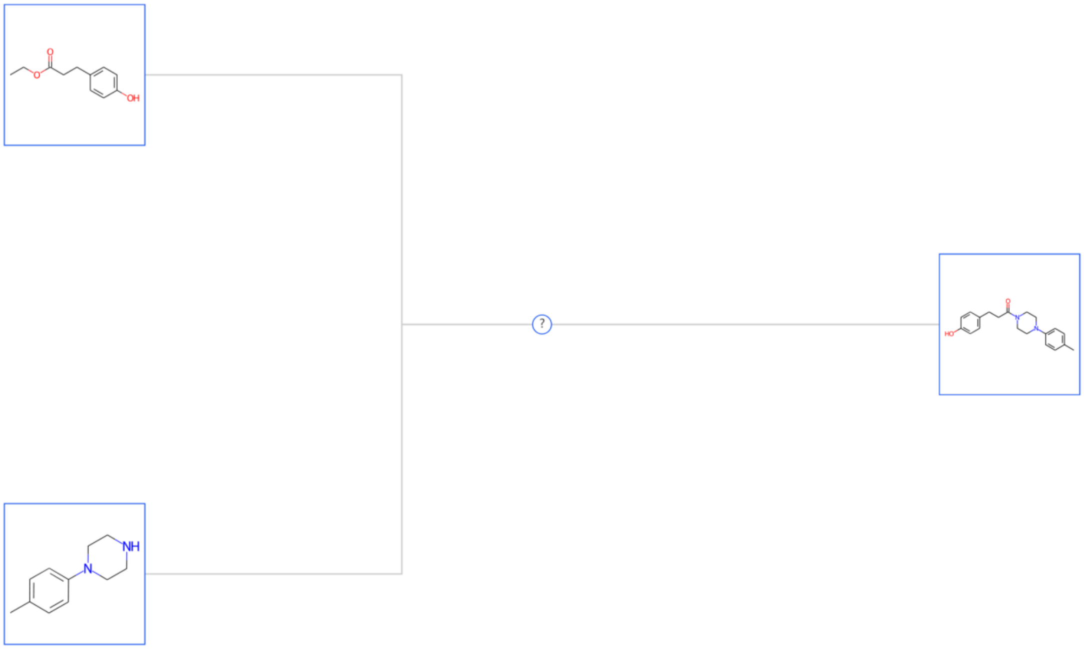

Alert Rule	Alert Structure	Reference
BMS Rule	✓	1. [1] Huth J R, Mendoza R, Olejniczak E T, et al. ALARM NMR: a rapid and robust experimental method to detect reactive false positives in biochemical screens[J]. Journal of the American Chemical Society, 2005, 127(1): 217-224.
Chelator Rule	✓	1. [1] Agrawal A, Johnson S L, Jacobsen J A, et al. Chelator fragment libraries for targeting metalloproteinases[J]. ChemMedChem: Chemistry Enabling Drug Discovery, 2010, 5(2): 195-199.
PAINS	✓	1. [1] Baell J B, Holloway G A. New substructure filters for removal of pan assay interference compounds (PAINS) from screening libraries and for their exclusion in bioassays[J]. Journal of medicinal chemistry, 2010, 53(7): 2719-2740.
Genotoxic Carcinogenicity Rule	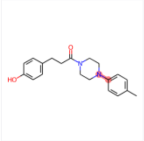	1. [1] Benigni R, Bossa C. Structure alerts for carcinogenicity, and the Salmonella assay system: a novel insight through the chemical relational databases technology[J]. Mutation Research/Reviews in Mutation Research, 2008, 659(3): 248-261.<br>2. [2] Ashby J, Tennant R W. Chemical structure, Salmonella mutagenicity and extent of carcinogenicity as indicators of genotoxic carcinogenesis among 222 chemicals tested in rodents by the US NCI/NTP[J]. Mutation Research/Genetic Toxicology, 1988, 204(1): 17-115.<br>3. [3] Kazius J, McGuire R, Bursi R. Derivation and validation of toxicophores for mutagenicity prediction[J]. Journal of medicinal chemistry, 2005, 48(1): 312-320.<br>4. [4] Bailey A B, Chanderbhan R, Collazo-Braier N, et al. The use of structure-activity relationship analysis in the food contact notification program[J]. Regulatory Toxicology and Pharmacology, 2005, 42(2): 225-235.
NTD	✓	1. [1] Brenk R, Schipani A, James D, et al. Lessons learnt from assembling screening libraries for drug discovery for neglected diseases[J]. ChemMedChem: Chemistry Enabling Drug Discovery, 2008, 3(3): 435-444.
SureChEMBL Rule	✓	1. [1] Sushko I, Salmina E, Potemkin V A, et al. ToxAlerts: a web server of structural alerts for toxic chemicals and compounds with potential adverse reactions[J]. 2012.

Compounds AI10-a26:

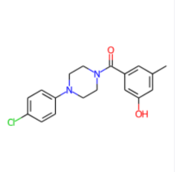

SMILES: Cc1cc(O)cc(C(=O)N2CCN(c3ccc(Cl)cc3)CC2)c1

San ID	No data
CAS	No data
InChI	InChI=1S/C18H19ClN2O2/c1-13-10-14(12-17(22)11-13)18(23)21-8-6-20(7-9-21)16-4-2-15(19)3-5-16/h2-5,10-12,22H,6-9H2,1H3
InChIKey	UPGKYBJPPJBCJU-UHFFFAOYSA-N

View in third-party databases: Loading...

MW	330.11	HBA	3	HBD	1
PAINS Alert	false	QED	0.918	NRot	2
TPSA	43.78	logD	3.54	logP	3.74
logS	-3.86				

Bad Average Good Scoring Notice

Absorption

Caco2	-4.62	HIA	99.59%	P-gp Inhibitor	82.14%
Bioavailability	99.86%	HFE	-8.86		

Distribution

BBB Penetration	84.53%	PPBR	1.00%	VDSS	2.95

Metabolism

CYP2D6 Inhibitor	16.33%	CYP3A4 Inhibitor	72.66%	CYP2C9 Inhibitor	82.29%
CYP2D6 Substrate	87.09%	CYP3A4 Substrate	56.17%	CYP2C9 Substrate	0.32%
CYP2C19 Inhibitor	92.28%	CYP1A2 Inhibitor	40.66%		

Excretion

Half Life (hour)	4.02	Clearance (mL/min/g)	48.12

Tox

hERG	96.30%	DILI	57.83%	NR-AhR	18.57%
Carcinogencity	28.02%	LD_{50}	1048.80	Respiratory Toxicity	7.77%
Eye Corrosion	0.05%				

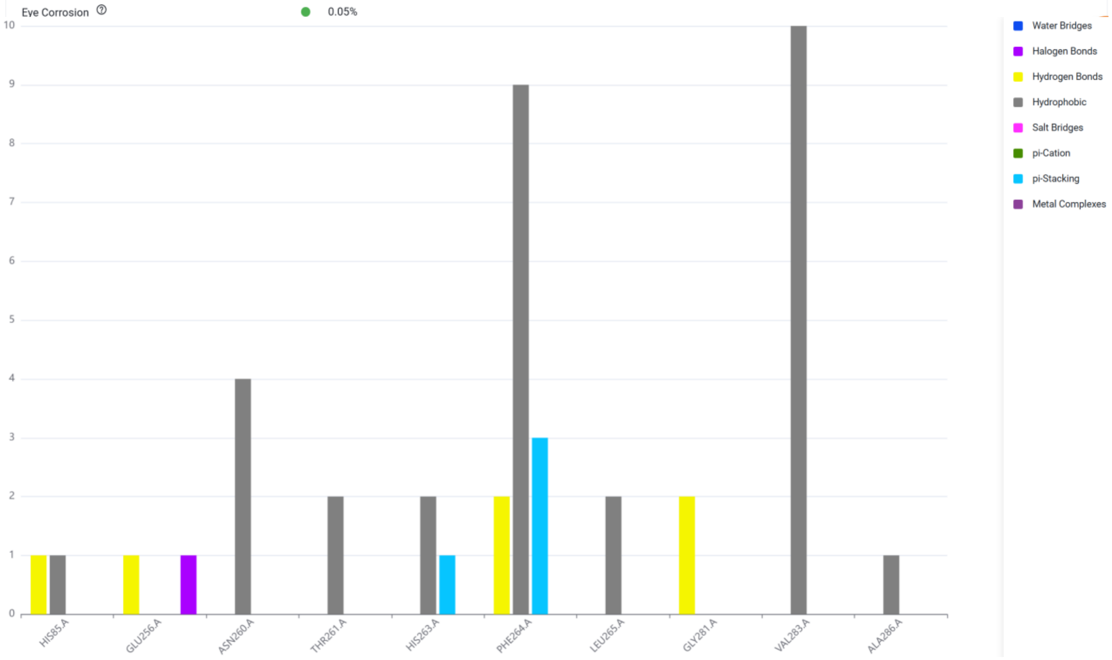

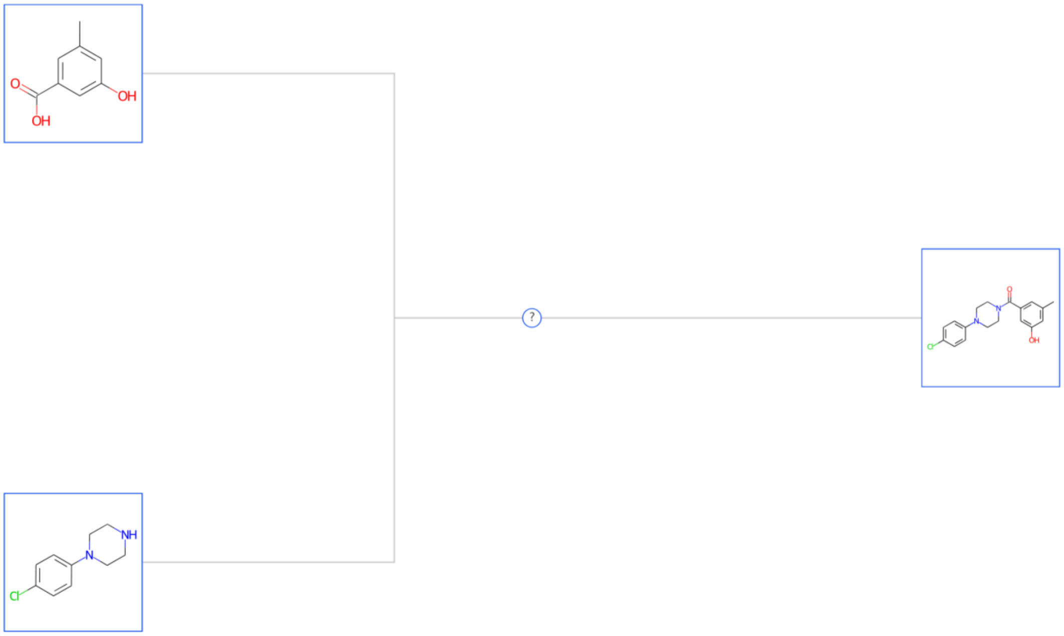

Alert Rule	Alert Structure	Reference
BMS Rule	✅	1. [1] Huth J R, Mendoza R, Olejniczak E T, et al. ALARM NMR: a rapid and robust experimental method to detect reactive false positives in biochemical screens[J]. Journal of the American Chemical Society, 2005, 127(1): 217-224.
Chelator Rule	✅	1. [1] Agrawal A, Johnson S L, Jacobsen J A, et al. Chelator fragment libraries for targeting metalloproteinases[J]. ChemMedChem: Chemistry Enabling Drug Discovery, 2010, 5(2): 195-199.
PAINS	✅	1. [1] Baell J B, Holloway G A. New substructure filters for removal of pan assay interference compounds (PAINS) from screening libraries and for their exclusion in bioassays[J]. Journal of medicinal chemistry, 2010, 53(7): 2719-2740.
Genotoxic Carcinogenicity Rule	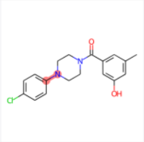	1. [1] Benigni R, Bossa C. Structure alerts for carcinogenicity, and the Salmonella assay system: a novel insight through the chemical relational databases technology[J]. Mutation Research/Reviews in Mutation Research, 2008, 659(3): 248-261.<br>2. [2] Ashby J, Tennant R W. Chemical structure, Salmonella mutagenicity and extent of carcinogenicity as indicators of genotoxic carcinogenesis among 222 chemicals tested in rodents by the US NCI/NTP[J]. Mutation Research/Genetic Toxicology, 1988, 204(1): 17-115.<br>3. [3] Kazius J, McGuire R, Bursi R. Derivation and validation of toxicophores for mutagenicity prediction[J]. Journal of medicinal chemistry, 2005, 48(1): 312-320.<br>4. [4] Bailey A B, Chanderbhan R, Collazo-Braier N, et al. The use of structure-activity relationship analysis in the food contact notification program[J]. Regulatory Toxicology and Pharmacology, 2005, 42(2): 225-235.
NTD	✅	1. [1] Brenk R, Schipani A, James D, et al. Lessons learnt from assembling screening libraries for drug discovery for neglected diseases[J]. ChemMedChem: Chemistry Enabling Drug Discovery, 2008, 3(3): 435-444.
SureChEMBL Rule	✅	1. [1] Sushko I, Salmina E, Potemkin V A, et al. ToxAlerts: a web server of structural alerts for toxic chemicals and compounds with potential adverse reactions[J]. 2012.

Compounds AI10-a27:

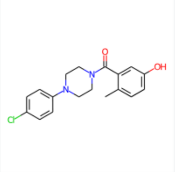

SMILES: Cc1ccc(O)cc1C(=O)N1CCN(c2ccc(Cl)cc2)CC1

San ID	No data
CAS	No data
InChI	InChI=1S/C18H19ClN2O2/c1-13-2-7-16(22)12-17(13)18(23)21-10-8-20(9-11-21)15-5-3-14(19)4-6-15/h2-7,12,22H,8-11H2,1H3
InChIKey	JTQVSZOUWVEKIH-UHFFFAOYSA-N

View in third-party databases: Loading...

MW	330.11	HBA	3	HBD	1
PAINS Alert	false	QED	0.918	NRot	2
TPSA	43.78	logD	3.28	logP	3.61
logS	-3.83				

Bad Average Good Scoring Notice

Absorption

Caco2	-4.69	HIA	99.38%	P-gp Inhibitor	76.39%
Bioavailability	99.98%	HFE	-9.08		

Distribution

BBB Penetration	80.21%	PPBR	1.00%	VDSS	2.81

Metabolism

CYP2D6 Inhibitor	16.65%	CYP3A4 Inhibitor	76.70%	CYP2C9 Inhibitor	86.37%
CYP2D6 Substrate	94.03%	CYP3A4 Substrate	49.69%	CYP2C9 Substrate	99.73%
CYP2C19 Inhibitor	93.94%	CYP1A2 Inhibitor	36.80%		

Excretion

Half Life (hour)	3.94	Clearance (mL/min/g)	49.10

Tox

hERG	96.28%	DILI	64.18%	NR-AhR	19.15%
Carcinogencity	31.75%	LD_{50}	487.74	Respiratory Toxicity	9.67%
Eye Corrosion	0.03%				

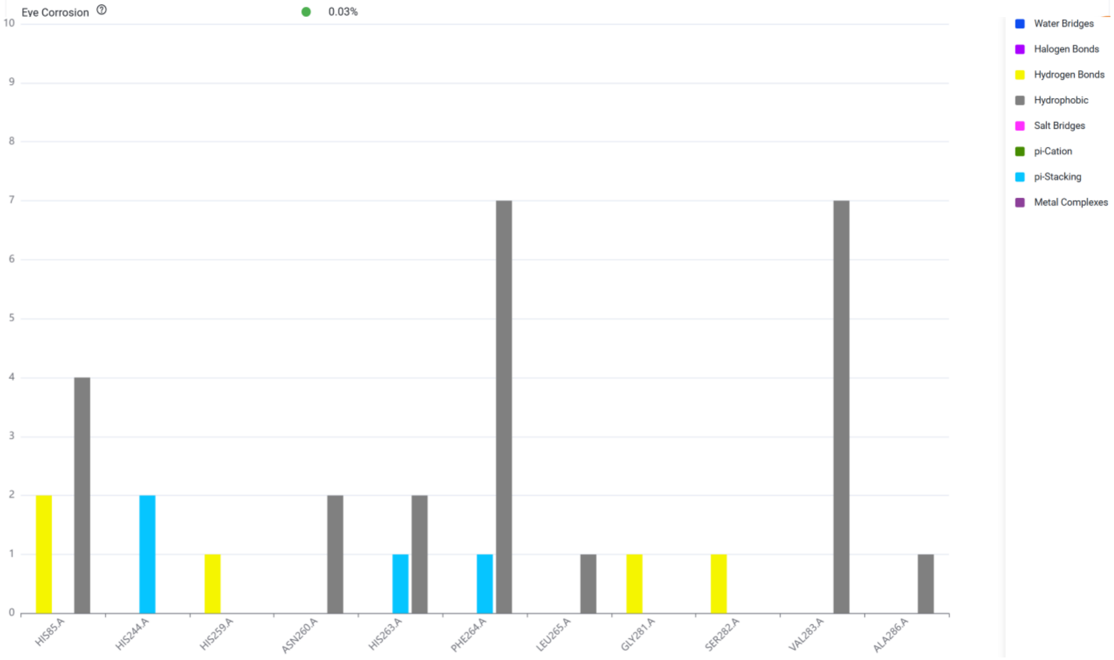

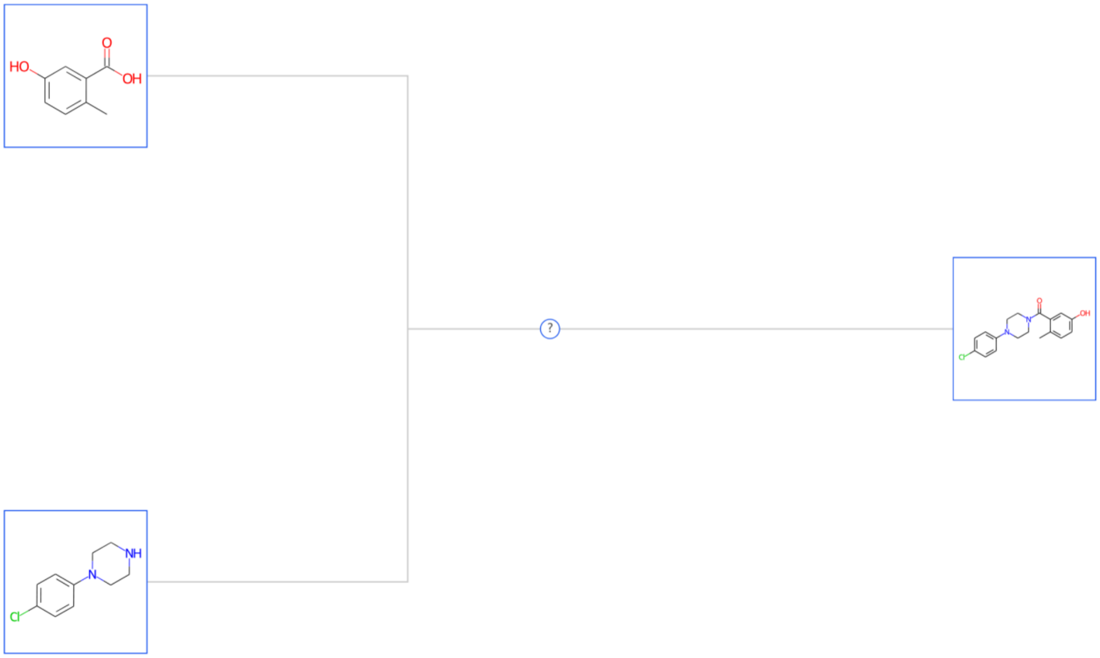

Alert Rule	Alert Structure	Reference
BMS Rule	✔	1. [1] Huth J R, Mendoza R, Olejniczak E T, et al. ALARM NMR: a rapid and robust experimental method to detect reactive false positives in biochemical screens[J]. Journal of the American Chemical Society, 2005, 127(1): 217-224.
Chelator Rule	✔	1. [1] Agrawal A, Johnson S L, Jacobsen J A, et al. Chelator fragment libraries for targeting metalloproteinases[J]. ChemMedChem: Chemistry Enabling Drug Discovery, 2010, 5(2): 195-199.
PAINS	✔	1. [1] Baell J B, Holloway G A. New substructure filters for removal of pan assay interference compounds (PAINS) from screening libraries and for their exclusion in bioassays[J]. Journal of medicinal chemistry, 2010, 53(7): 2719-2740.
Genotoxic Carcinogenicity Rule	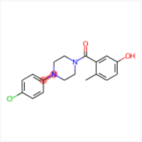	1. [1] Benigni R, Bossa C. Structure alerts for carcinogenicity, and the Salmonella assay system: a novel insight through the chemical relational databases technology[J]. Mutation Research/Reviews in Mutation Research, 2008, 659(3): 248-261.<br>2. [2] Ashby J, Tennant R W. Chemical structure, Salmonella mutagenicity and extent of carcinogenicity as indicators of genotoxic carcinogenesis among 222 chemicals tested in rodents by the US NCI/NTP[J]. Mutation Research/Genetic Toxicology, 1988, 204(1): 17-115.<br>3. [3] Kazius J, McGuire R, Bursi R. Derivation and validation of toxicophores for mutagenicity prediction[J]. Journal of medicinal chemistry, 2005, 48(1): 312-320.<br>4. [4] Bailey A B, Chanderbhan R, Collazo-Braier N, et al. The use of structure-activity relationship analysis in the food contact notification program[J]. Regulatory Toxicology and Pharmacology, 2005, 42(2): 225-235.
NTD	✔	1. [1] Brenk R, Schipani A, James D, et al. Lessons learnt from assembling screening libraries for drug discovery for neglected diseases[J]. ChemMedChem: Chemistry Enabling Drug Discovery, 2008, 3(3): 435-444.
SureChEMBL Rule	✔	1. [1] Sushko I, Salmina E, Potemkin V A, et al. ToxAlerts: a web server of structural alerts for toxic chemicals and compounds with potential adverse reactions[J]. 2012.

Compounds AI10-a28:

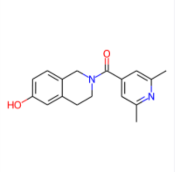

SMILES: Cc1cc(C(=O)N2CCc3cc(O)ccc3C2)cc(C)n1

San ID	No data
CAS	No data
InChI	InChI=1S/C17H18N2O2/c1-11-7-15(8-12(2)18-11)17(21)19-6-5-13-9-16(20)4-3-14(13)10-19/h3-4,7-9,20H,5-6,10H2,1-2H3
InChIKey	WQLDUAUFGZIWPA-UHFFFAOYSA-N

View in third-party databases: No data

MW	282.14	HBA	3	HBD	1
PAINS Alert	false	QED	0.874	NRot	1
TPSA	53.43	logD	1.61	logP	1.22
logS	-2.59				

Bad Average Good Scoring Notice

Absorption

Caco2	-4.60	HIA	100.00%	P-gp Inhibitor	26.93%
Bioavailability	99.21%	HFE	-9.62		

Distribution

BBB Penetration	79.00%	PPBR	1.00%	VDSS	1.30

Metabolism

CYP2D6 Inhibitor	19.62%	CYP3A4 Inhibitor	63.73%	CYP2C9 Inhibitor	61.46%
CYP2D6 Substrate	89.27%	CYP3A4 Substrate	62.87%	CYP2C9 Substrate	6.82%
CYP2C19 Inhibitor	80.84%	CYP1A2 Inhibitor	82.14%		

Excretion

Half Life (hour)	3.63	Clearance (mL/min/g)	48.94

Tox

hERG	86.59%	DILI	75.00%	NR-AhR	12.05%
Carcinogencity	1.27%	LD_{50}	632.37	Respiratory Toxicity	98.87%
Eye Corrosion	0.06%				

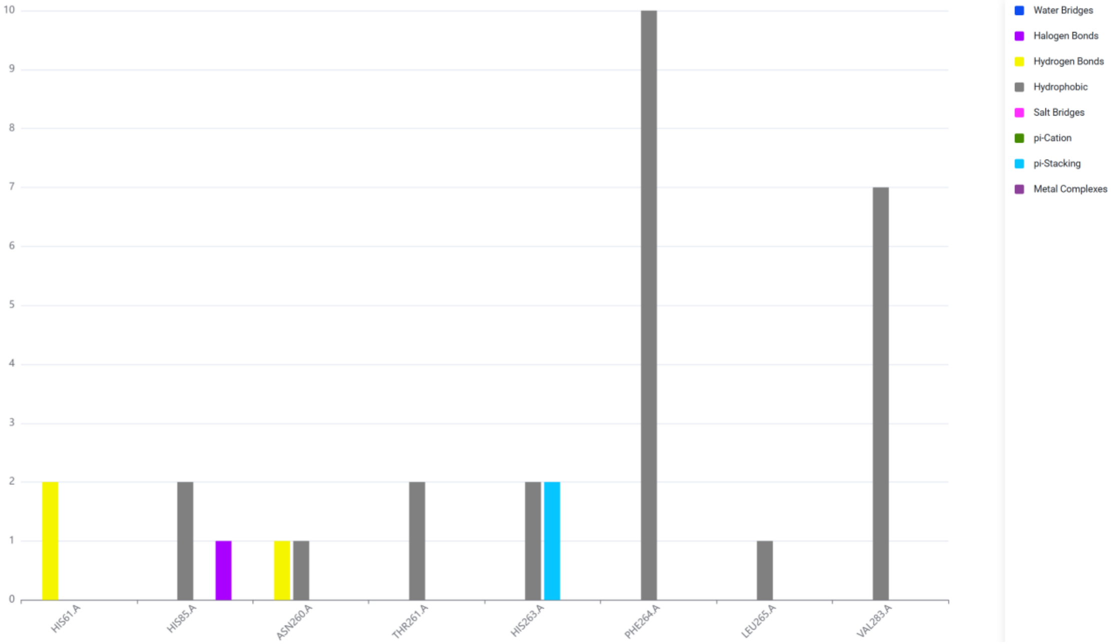

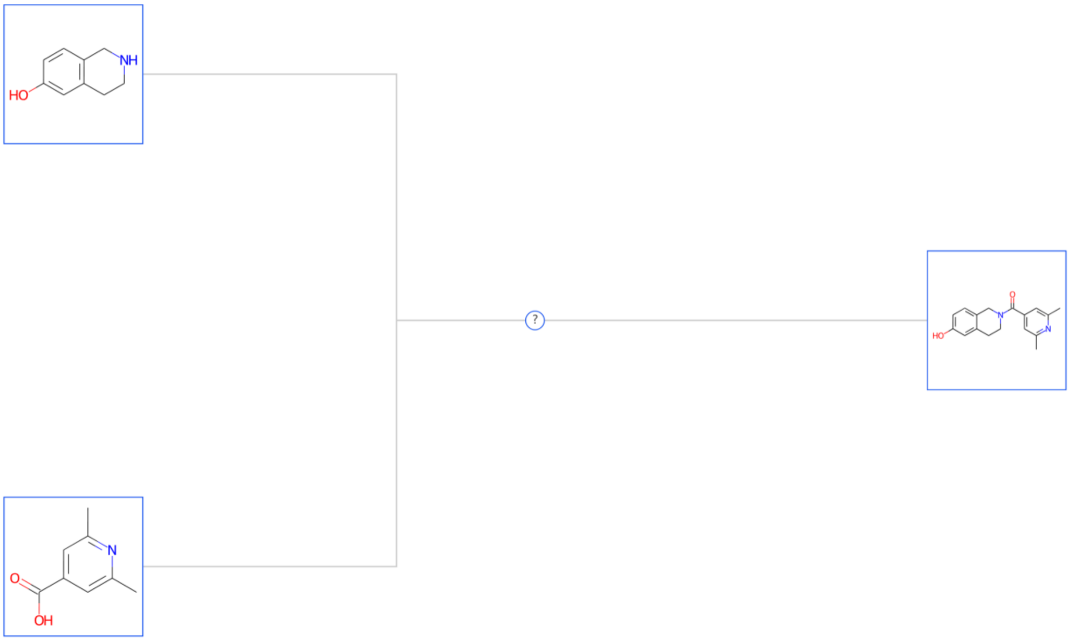

Alert Rule	Alert Structure	Reference
BMS Rule	✔	1. [1] Huth J R, Mendoza R, Olejniczak E T, et al. ALARM NMR: a rapid and robust experimental method to detect reactive false positives in biochemical screens[J]. Journal of the American Chemical Society, 2005, 127(1): 217-224.
Chelator Rule	✔	1. [1] Agrawal A, Johnson S L, Jacobsen J A, et al. Chelator fragment libraries for targeting metalloproteinases[J]. ChemMedChem: Chemistry Enabling Drug Discovery, 2010, 5(2): 195-199.
PAINS	✔	1. [1] Baell J B, Holloway G A. New substructure filters for removal of pan assay interference compounds (PAINS) from screening libraries and for their exclusion in bioassays[J]. Journal of medicinal chemistry, 2010, 53(7): 2719-2740.
Genotoxic Carcinogenicity Rule	✔	1. [1] Benigni R, Bossa C. Structure alerts for carcinogenicity, and the Salmonella assay system: a novel insight through the chemical relational databases technology[J]. Mutation Research/Reviews in Mutation Research, 2008, 659(3): 248-261.<br>2. [2] Ashby J, Tennant R W. Chemical structure, Salmonella mutagenicity and extent of carcinogenicity as indicators of genotoxic carcinogenesis among 222 chemicals tested in rodents by the US NCI/NTP[J]. Mutation Research/Genetic Toxicology, 1988, 204(1): 17-115.<br>3. [3] Kazius J, McGuire R, Bursi R. Derivation and validation of toxicophores for mutagenicity prediction[J]. Journal of medicinal chemistry, 2005, 48(1): 312-320.<br>4. [4] Bailey A B, Chanderbhan R, Collazo-Braier N, et al. The use of structure-activity relationship analysis in the food contact notification program[J]. Regulatory Toxicology and Pharmacology, 2005, 42(2): 225-235.
NTD	✔	1. [1] Brenk R, Schipani A, James D, et al. Lessons learnt from assembling screening libraries for drug discovery for neglected diseases[J]. ChemMedChem: Chemistry Enabling Drug Discovery, 2008, 3(3): 435-444.
SureChEMBL Rule	✔	1. [1] Sushko I, Salmina E, Potemkin V A, et al. ToxAlerts: a web server of structural alerts for toxic chemicals and compounds with potential adverse reactions[J]. 2012.

Compounds AI10-a29:

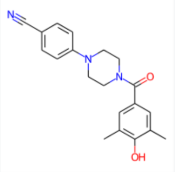

SMILES: Cc1cc(C(=O)N2CCN(c3ccc(C#N)cc3)CC2)cc(C)c1O

San ID	No data
CAS	No data
InChI	InChI=1S/C20H21N3O2/c1-14-11-17(12-15(2)19(14)24)20(25)23-9-7-22(8-10-23)18-5-3-16(13-21)4-6-18/h3-6,11-12,24H,7-10H2,1-2H3
InChIKey	WDQSHNLILDMIBZ-UHFFFAOYSA-N

View in third-party databases: Loading...

MW	335.16	HBA	4	HBD	1
PAINS Alert	false	QED	0.916	NRot	2
TPSA	67.57	logD	3.06	logP	2.83
logS	-4.03				

Bad Average Good Scoring Notice

Absorption

Caco2	-4.72	HIA	100.00%	P-gp Inhibitor	33.18%
Bioavailability	99.96%	HFE	-10.28		

Distribution

BBB Penetration	73.26%	PPBR	1.00%	VDSS	1.62

Metabolism

CYP2D6 Inhibitor	1.91%	CYP3A4 Inhibitor	75.70%	CYP2C9 Inhibitor	80.16%
CYP2D6 Substrate	83.12%	CYP3A4 Substrate	68.13%	CYP2C9 Substrate	0.05%
CYP2C19 Inhibitor	82.90%	CYP1A2 Inhibitor	14.64%		

Excretion

Half Life (hour)	3.40	Clearance (mL/min/g)	52.46

Tox

hERG	97.42%	DILI	35.01%	NR-AhR	18.02%
Carcinogencity	30.32%	LD_{50}	497.78	Respiratory Toxicity	46.62%
Eye Corrosion	0.08%				

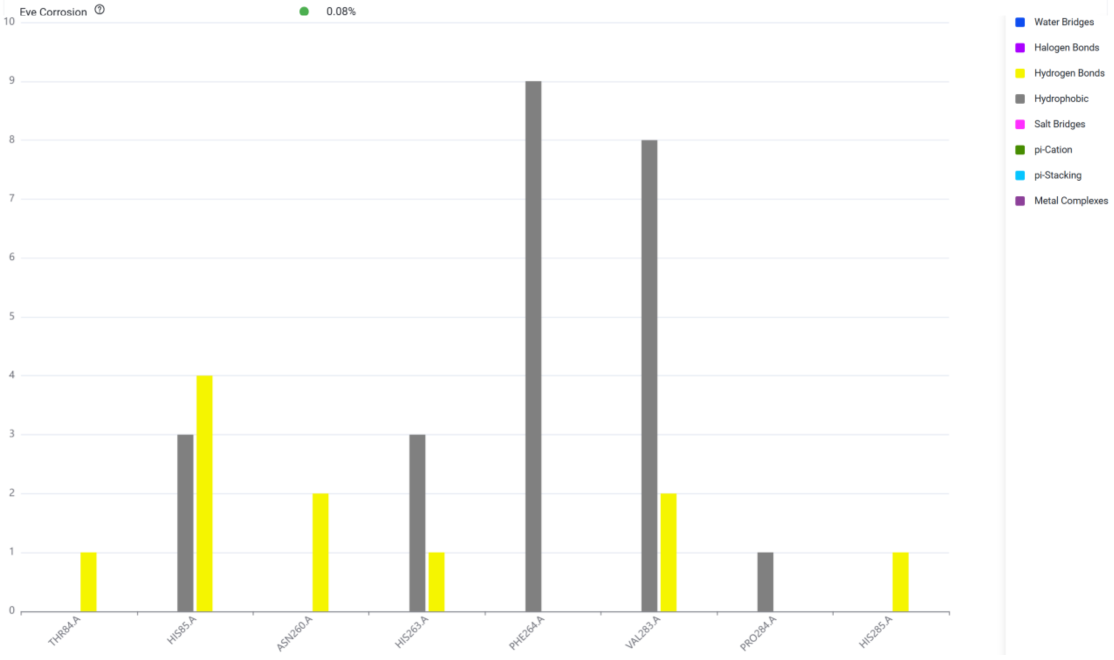

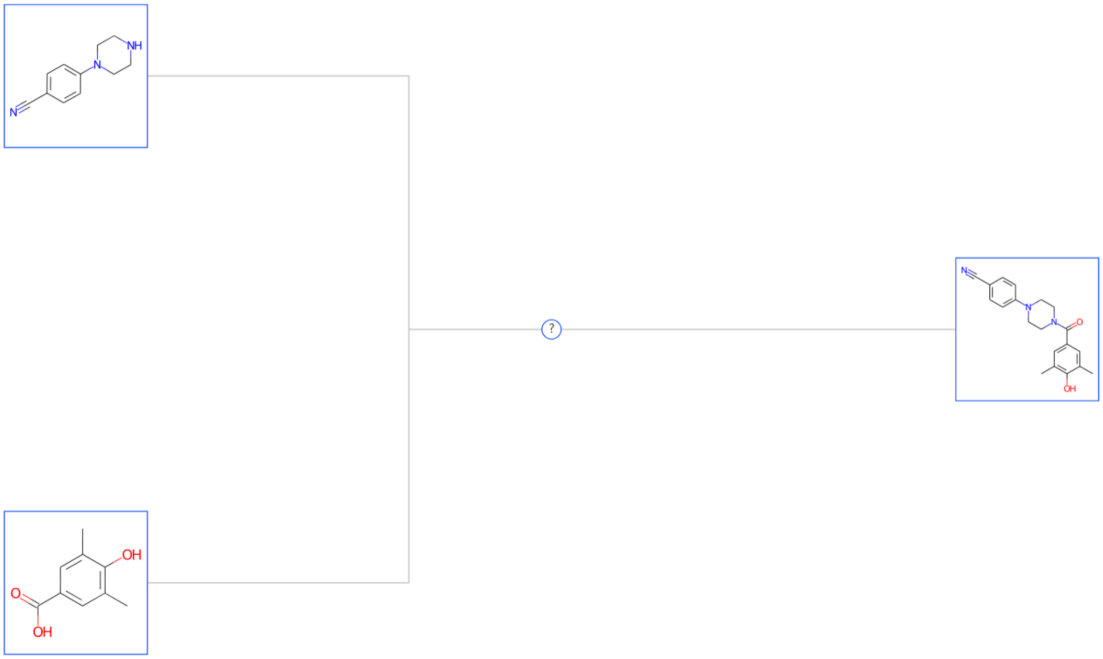

Alert Rule	Alert Structure	Reference
BMS Rule	✔	1. [1] Huth J R, Mendoza R, Olejniczak E T, et al. ALARM NMR: a rapid and robust experimental method to detect reactive false positives in biochemical screens[J]. Journal of the American Chemical Society, 2005, 127(1): 217-224.
Chelator Rule	✔	1. [1] Agrawal A, Johnson S L, Jacobsen J A, et al. Chelator fragment libraries for targeting metalloproteinases[J]. ChemMedChem: Chemistry Enabling Drug Discovery, 2010, 5(2): 195-199.
PAINS	✔	1. [1] Baell J B, Holloway G A. New substructure filters for removal of pan assay interference compounds (PAINS) from screening libraries and for their exclusion in bioassays[J]. Journal of medicinal chemistry, 2010, 53(7): 2719-2740.
Genotoxic Carcinogenicity Rule	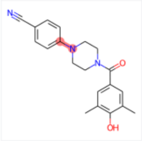	1. [1] Benigni R, Bossa C. Structure alerts for carcinogenicity, and the Salmonella assay system: a novel insight through the chemical relational databases technology[J]. Mutation Research/Reviews in Mutation Research, 2008, 659(3): 248-261.<br>2. [2] Ashby J, Tennant R W. Chemical structure, Salmonella mutagenicity and extent of carcinogenicity as indicators of genotoxic carcinogenesis among 222 chemicals tested in rodents by the US NCI/NTP[J]. Mutation Research/Genetic Toxicology, 1988, 204(1): 17-115.<br>3. [3] Kazius J, McGuire R, Bursi R. Derivation and validation of toxicophores for mutagenicity prediction[J]. Journal of medicinal chemistry, 2005, 48(1): 312-320.<br>4. [4] Bailey A B, Chanderbhan R, Collazo-Braier N, et al. The use of structure-activity relationship analysis in the food contact notification program[J]. Regulatory Toxicology and Pharmacology, 2005, 42(2): 225-235.
NTD	✔	1. [1] Brenk R, Schipani A, James D, et al. Lessons learnt from assembling screening libraries for drug discovery for neglected diseases[J]. ChemMedChem: Chemistry Enabling Drug Discovery, 2008, 3(3): 435-444.
SureChEMBL Rule	✔	1. [1] Sushko I, Salmina E, Potemkin V A, et al. ToxAlerts: a web server of structural alerts for toxic chemicals and compounds with potential adverse reactions[J]. 2012.

Compounds AI10-a30:

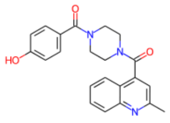

SMILES: Cc1cc(C(=O)N2CCN(C(=O)c3ccc(O)cc3)CC2)c2ccccc2n1

San ID	No data
CAS	No data
InChI	InChI=1S/C22H21N3O3/c1-15-14-19(18-4-2-3-5-20(18)23-15)22(28)25-12-10-24(11-13-25)21(27)16-6-8-17(26)9-7-16/h2-9,14,26H,10-13H2,1H3
InChIKey	MBNKHQYDSWCDCM-UHFFFAOYSA-N

View in third-party databases: Loading..

MW	375.16	HBA	4	HBD	1
PAINS Alert	false	QED	0.747	NRot	2
TPSA	73.74	logD	1.25	logP	2.39
logS	-3.85				

Bad Average Good Scoring Notice

Absorption

Caco2	-4.93	HIA	100.00%	P-gp Inhibitor	20.17%
Bioavailability	99.95%	HFE	-11.38		

Distribution

BBB Penetration	66.36%	PPBR	1.00%	VDSS	1.61

Metabolism

CYP2D6 Inhibitor	5.48%	CYP3A4 Inhibitor	61.65%	CYP2C9 Inhibitor	50.78%
CYP2D6 Substrate	91.08%	CYP3A4 Substrate	68.51%	CYP2C9 Substrate	4.00%
CYP2C19 Inhibitor	57.77%	CYP1A2 Inhibitor	14.59%		

Excretion

Half Life (hour)	4.16	Clearance (mL/min/g)	48.49

Tox

hERG	92.15%	DILI	80.58%	NR-AhR	12.94%
Carcinogencity	4.83%	LD_{50}	645.16	Respiratory Toxicity	99.36%
Eye Corrosion	0.01%				

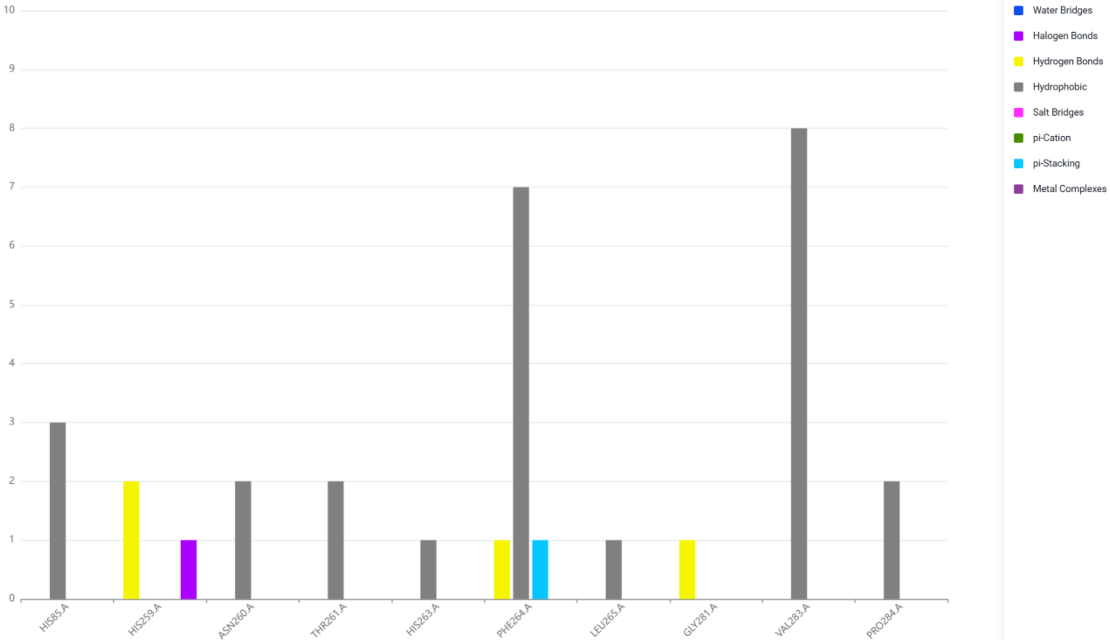

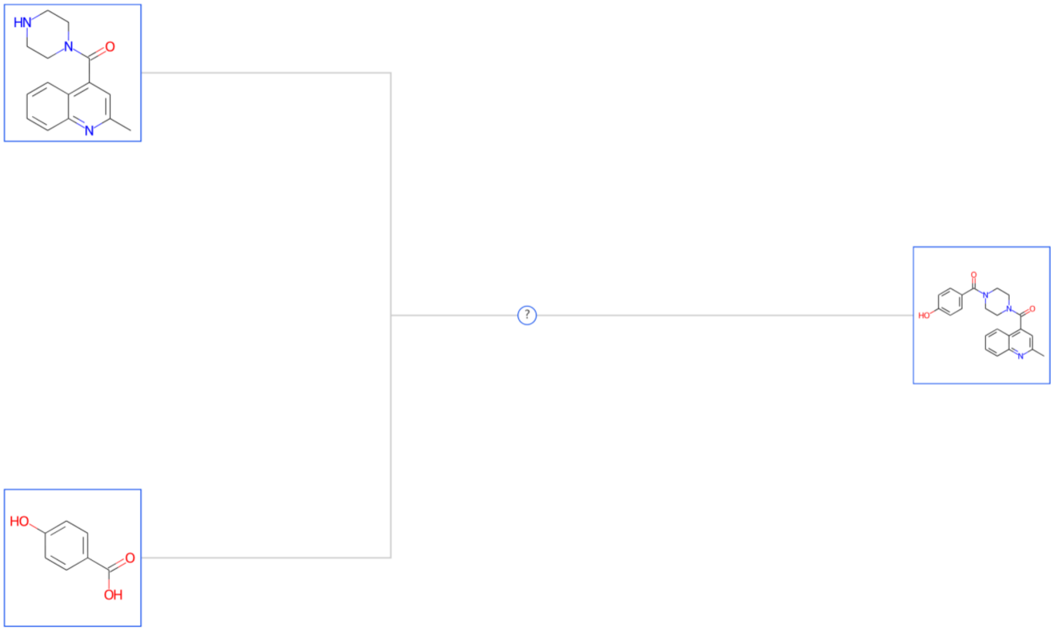

Alert Rule	Alert Structure	Reference
BMS Rule	✔	1. [1] Huth J R, Mendoza R, Olejniczak E T, et al. ALARM NMR: a rapid and robust experimental method to detect reactive false positives in biochemical screens[J]. Journal of the American Chemical Society, 2005, 127(1): 217-224.
Chelator Rule	✔	1. [1] Agrawal A, Johnson S L, Jacobsen J A, et al. Chelator fragment libraries for targeting metalloproteinases[J]. ChemMedChem: Chemistry Enabling Drug Discovery, 2010, 5(2): 195-199.
PAINS	✔	1. [1] Baell J B, Holloway G A. New substructure filters for removal of pan assay interference compounds (PAINS) from screening libraries and for their exclusion in bioassays[J]. Journal of medicinal chemistry, 2010, 53(7): 2719-2740.
Genotoxic Carcinogenicity Rule	✔	1. [1] Benigni R, Bossa C. Structure alerts for carcinogenicity, and the Salmonella assay system: a novel insight through the chemical relational databases technology[J]. Mutation Research/Reviews in Mutation Research, 2008, 659(3): 248-261.<br>2. [2] Ashby J, Tennant R W. Chemical structure, Salmonella mutagenicity and extent of carcinogenicity as indicators of genotoxic carcinogenesis among 222 chemicals tested in rodents by the US NCI/NTP[J]. Mutation Research/Genetic Toxicology, 1988, 204(1): 17-115.<br>3. [3] Kazius J, McGuire R, Bursi R. Derivation and validation of toxicophores for mutagenicity prediction[J]. Journal of medicinal chemistry, 2005, 48(1): 312-320.<br>4. [4] Bailey A B, Chanderbhan R, Collazo-Braier N, et al. The use of structure-activity relationship analysis in the food contact notification program[J]. Regulatory Toxicology and Pharmacology, 2005, 42(2): 225-235.
NTD	✔	1. [1] Brenk R, Schipani A, James D, et al. Lessons learnt from assembling screening libraries for drug discovery for neglected diseases[J]. ChemMedChem: Chemistry Enabling Drug Discovery, 2008, 3(3): 435-444.
SureChEMBL Rule	✔	1. [1] Sushko I, Salmina E, Potemkin V A, et al. ToxAlerts: a web server of structural alerts for toxic chemicals and compounds with potential adverse reactions[J]. 2012.

Compounds AI10-a31:

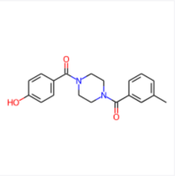

SMILES: Cc1cccc(C(=O)N2CCN(C(=O)c3ccc(O)cc3)CC2)c1

San ID	No data
CAS	No data
InChI	InChI=1S/C19H20N2O3/c1-14-3-2-4-16(13-14)19(24)21-11-9-20(10-12-21)18(23)15-5-7-17(22)8-6-15/h2-8,13,22H,9-12H2,1H3
InChIKey	AQFYPWQLXZBOMS-UHFFFAOYSA-N

View in third-party databases: Loading...

MW	324.15	HBA	3	HBD	1
PAINS Alert	false	QED	0.922	NRot	2
TPSA	60.85	logD	1.1	logP	2.58
logS	-3.53				

Bad Average Good Scoring Notice

Absorption

Caco2	-4.78	HIA	100.00%	P-gp Inhibitor	21.14%
Bioavailability	98.93%	HFE	-10.31		

Distribution

BBB Penetration	77.13%	PPBR	1.00%	VDSS	0.92

Metabolism

CYP2D6 Inhibitor	3.70%	CYP3A4 Inhibitor	58.91%	CYP2C9 Inhibitor	57.23%
CYP2D6 Substrate	89.54%	CYP3A4 Substrate	59.04%	CYP2C9 Substrate	0.03%
CYP2C19 Inhibitor	60.58%	CYP1A2 Inhibitor	3.81%		

Excretion

Half Life (hour)	3.93	Clearance (mL/min/g)	42.15

Tox

hERG	88.74%	DILI	55.22%	NR-AhR	5.55%
Carcinogencity	58.05%	LD_{50}	906.03	Respiratory Toxicity	7.28%
Eye Corrosion	0.03%				

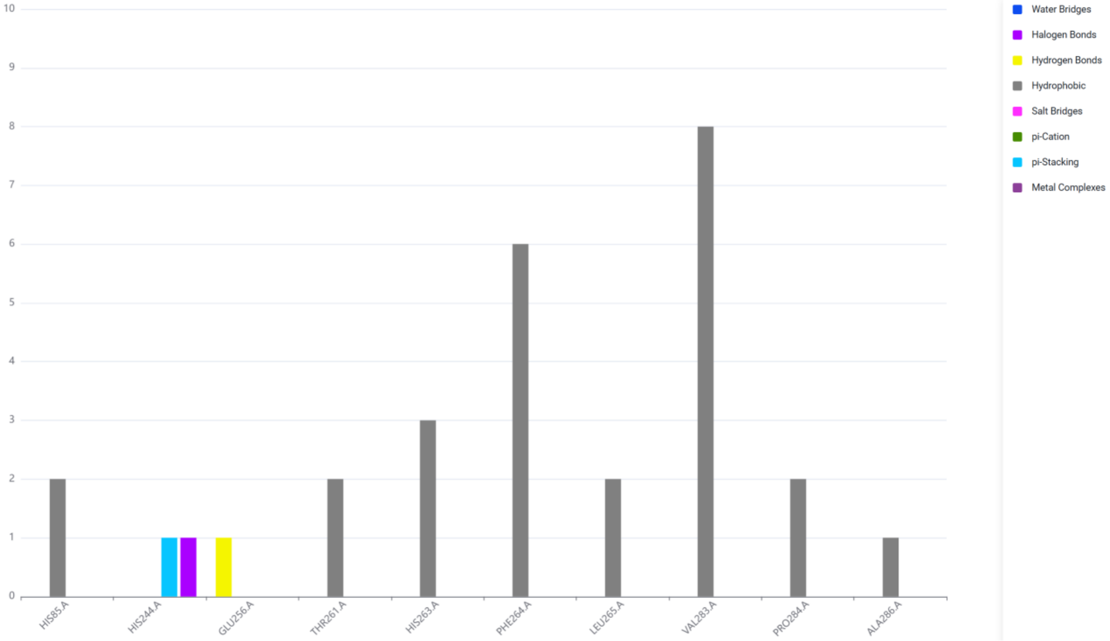

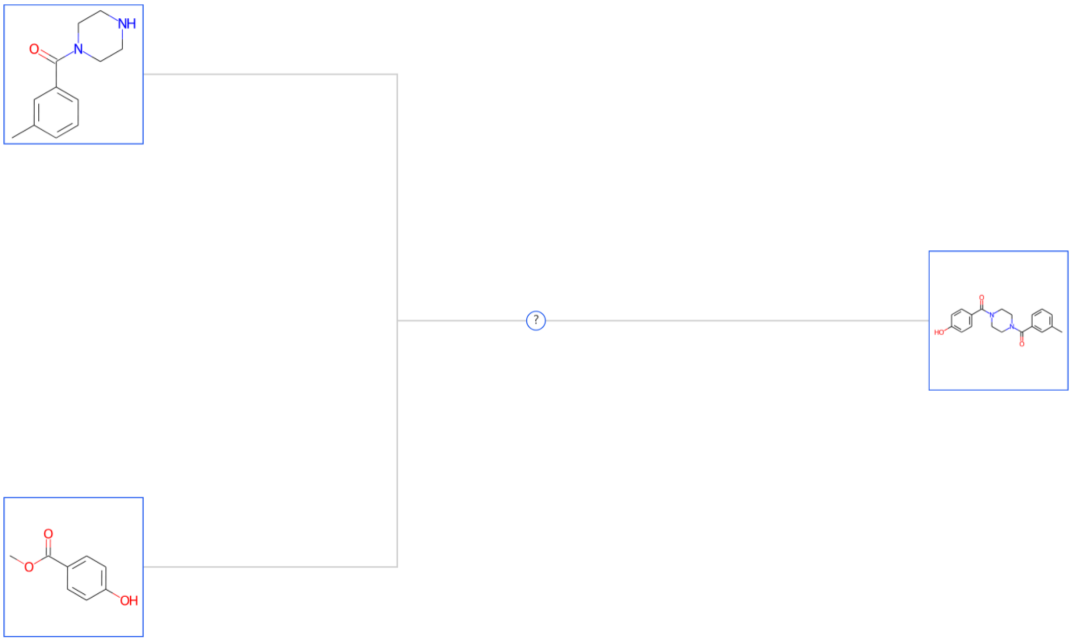

Alert Rule	Alert Structure	Reference
BMS Rule	✓	1. [1] Huth J R, Mendoza R, Olejniczak E T, et al. ALARM NMR: a rapid and robust experimental method to detect reactive false positives in biochemical screens[J]. Journal of the American Chemical Society, 2005, 127(1): 217-224.
Chelator Rule	✓	1. [1] Agrawal A, Johnson S L, Jacobsen J A, et al. Chelator fragment libraries for targeting metalloproteinases[J]. ChemMedChem: Chemistry Enabling Drug Discovery, 2010, 5(2): 195-199.
PAINS	✓	1. [1] Baell J B, Holloway G A. New substructure filters for removal of pan assay interference compounds (PAINS) from screening libraries and for their exclusion in bioassays[J]. Journal of medicinal chemistry, 2010, 53(7): 2719-2740.
Genotoxic Carcinogenicity Rule	✓	1. [1] Benigni R, Bossa C. Structure alerts for carcinogenicity, and the Salmonella assay system: a novel insight through the chemical relational databases technology[J]. Mutation Research/Reviews in Mutation Research, 2008, 659(3): 248-261.<br>2. [2] Ashby J, Tennant R W. Chemical structure, Salmonella mutagenicity and extent of carcinogenicity as indicators of genotoxic carcinogenesis among 222 chemicals tested in rodents by the US NCI/NTP[J]. Mutation Research/Genetic Toxicology, 1988, 204(1): 17-115.<br>3. [3] Kazius J, McGuire R, Bursi R. Derivation and validation of toxicophores for mutagenicity prediction[J]. Journal of medicinal chemistry, 2005, 48(1): 312-320.<br>4. [4] Bailey A B, Chanderbhan R, Collazo-Braier N, et al. The use of structure-activity relationship analysis in the food contact notification program[J]. Regulatory Toxicology and Pharmacology, 2005, 42(2): 225-235.
NTD	✓	1. [1] Brenk R, Schipani A, James D, et al. Lessons learnt from assembling screening libraries for drug discovery for neglected diseases[J]. ChemMedChem: Chemistry Enabling Drug Discovery, 2008, 3(3): 435-444.
SureChEMBL Rule	✓	1. [1] Sushko I, Salmina E, Potemkin V A, et al. ToxAlerts: a web server of structural alerts for toxic chemicals and compounds with potential adverse reactions[J]. 2012.

Compounds AI10-a32:

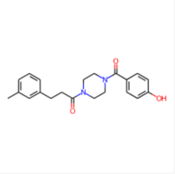

SMILES: Cc1cccc(CCC(=O)N2CCN(C(=O)c3ccc(O)cc3)CC2)c1

San ID	No data
CAS	No data
InChI	InChI=1S/C21H24N2O3/c1-16-3-2-4-17(15-16)5-10-20(25)22-11-13-23(14-12-22)21(26)18-6-8-19(24)9-7-18/h2-4,6-9,15,24H,5,10-14H2,1H3
InChIKey	OOOHAZUUNYQSJT-UHFFFAOYSA-N

View in third-party databases: PubChem

MW	352.18	HBA	3	HBD	1
PAINS Alert	false	QED	0.92	NRot	4
TPSA	60.85	logD	1.69	logP	3.12
logS	-3.44				

Bad Average Good Scoring Notice

Absorption

Caco2	-4.78	HIA	100.00%	P-gp Inhibitor	31.27%
Bioavailability	99.25%	HFE	-10.84		

Distribution

BBB Penetration	79.76%	PPBR	1.00%	VDSS	0.71

Metabolism

CYP2D6 Inhibitor	8.88%	CYP3A4 Inhibitor	81.42%	CYP2C9 Inhibitor	67.71%
CYP2D6 Substrate	67.43%	CYP3A4 Substrate	69.95%	CYP2C9 Substrate	0.08%
CYP2C19 Inhibitor	78.88%	CYP1A2 Inhibitor	7.17%		

Excretion

Half Life (hour)	3.67	Clearance (mL/min/g)	51.18

Tox

hERG	92.00%	DILI	16.57%	NR-AhR	7.19%
Carcinogencity	8.44%	LD_{50}	837.54	Respiratory Toxicity	2.89%
Eye Corrosion	0.00%				

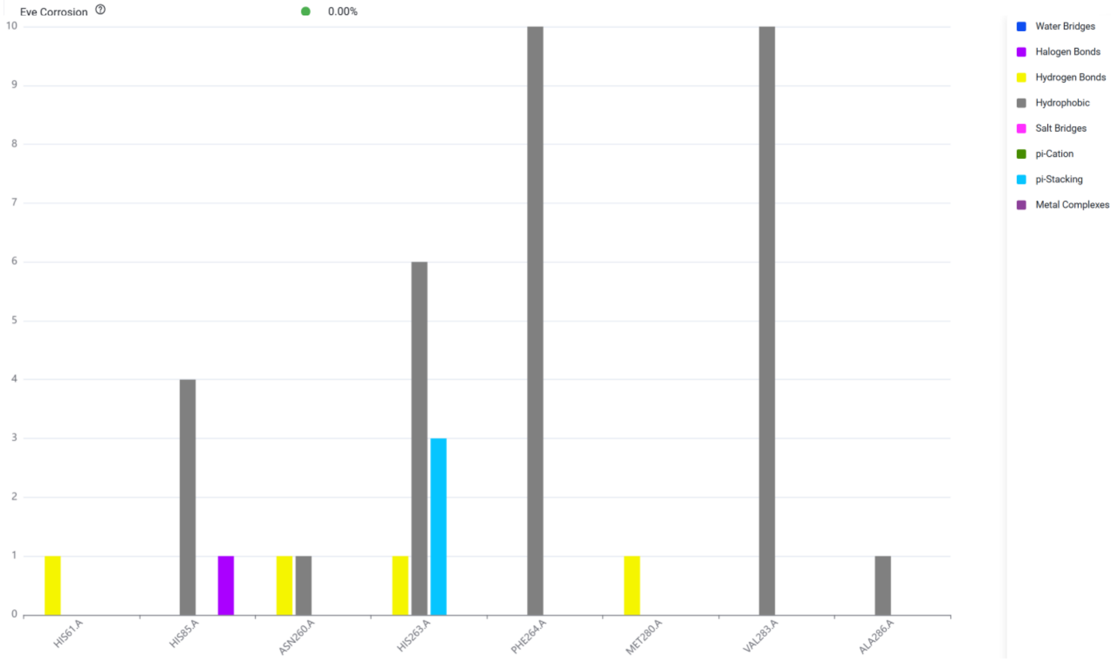

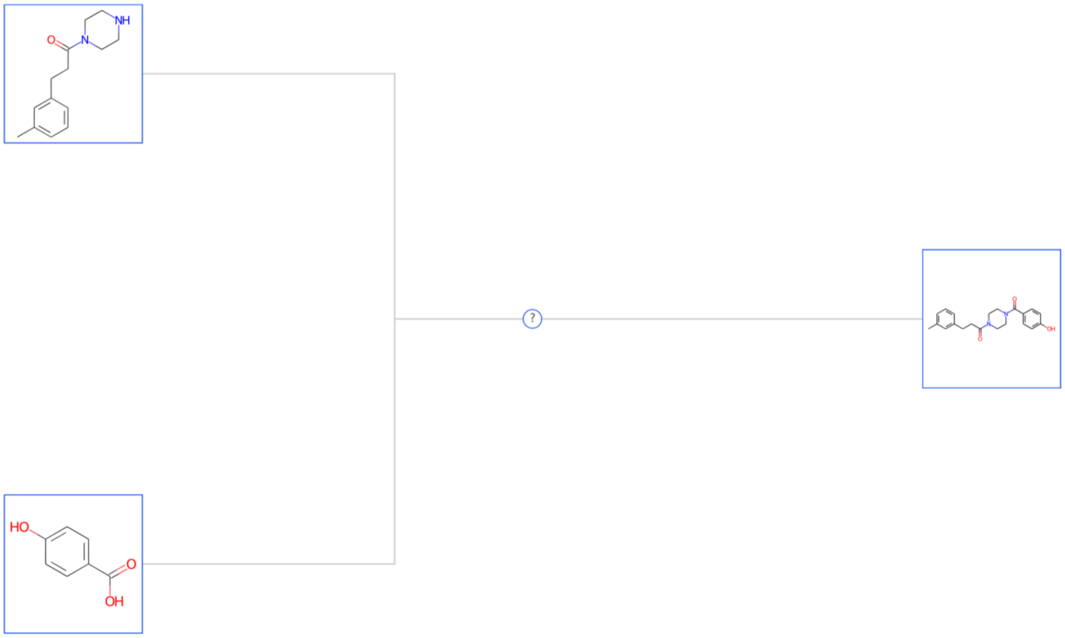

Alert Rule	Alert Structure	Reference
BMS Rule	✓	1. [1] Huth J R, Mendoza R, Olejniczak E T, et al. ALARM NMR: a rapid and robust experimental method to detect reactive false positives in biochemical screens[J]. Journal of the American Chemical Society, 2005, 127(1): 217-224.
Chelator Rule	✓	1. [1] Agrawal A, Johnson S L, Jacobsen J A, et al. Chelator fragment libraries for targeting metalloproteinases[J]. ChemMedChem: Chemistry Enabling Drug Discovery, 2010, 5(2): 195-199.
PAINS	✓	1. [1] Baell J B, Holloway G A. New substructure filters for removal of pan assay interference compounds (PAINS) from screening libraries and for their exclusion in bioassays[J]. Journal of medicinal chemistry, 2010, 53(7): 2719-2740.
Genotoxic Carcinogenicity Rule	✓	1. [1] Benigni R, Bossa C. Structure alerts for carcinogenicity, and the Salmonella assay system: a novel insight through the chemical relational databases technology[J]. Mutation Research/Reviews in Mutation Research, 2008, 659(3): 248-261.<br>2. [2] Ashby J, Tennant R W. Chemical structure, Salmonella mutagenicity and extent of carcinogenicity as indicators of genotoxic carcinogenesis among 222 chemicals tested in rodents by the US NCI/NTP[J]. Mutation Research/Genetic Toxicology, 1988, 204(1): 17-115.<br>3. [3] Kazius J, McGuire R, Bursi R. Derivation and validation of toxicophores for mutagenicity prediction[J]. Journal of medicinal chemistry, 2005, 48(1): 312-320.<br>4. [4] Bailey A B, Chanderbhan R, Collazo-Braier N, et al. The use of structure-activity relationship analysis in the food contact notification program[J]. Regulatory Toxicology and Pharmacology, 2005, 42(2): 225-235.
NTD	✓	1. [1] Brenk R, Schipani A, James D, et al. Lessons learnt from assembling screening libraries for drug discovery for neglected diseases[J]. ChemMedChem: Chemistry Enabling Drug Discovery, 2008, 3(3): 435-444.
SureChEMBL Rule	✓	1. [1] Sushko I, Salmina E, Potemkin V A, et al. ToxAlerts: a web server of structural alerts for toxic chemicals and compounds with potential adverse reactions[J]. 2012.
